# Molecular and Descriptor Spaces for Predicting Initial Rate of Catalytic Homogeneous Quinoline Hydrogenation with Ru, Rh, Os, and Ir Catalysts

**DOI:** 10.1021/acsomega.4c09503

**Published:** 2025-04-30

**Authors:** Rodolfo Izquierdo, Rafael Zadorosny, Merlín Rosales, Yovani Marrero-Ponce, Néstor Cubillan

**Affiliations:** † Departamento de Física e Química, Universidade Estadual Paulista (UNESP), Faculdade de Engenharia, Caixa Postal 31, 15385-000 Ilha Solteira, SP, Brazil; ‡ Grupo de Química Bioorgánica, Facultad de Ciencias Exactas y Naturales, Universidad de Cartagena, Cartagena de Indias 130015, Colombia; § Facultad de Ingeniería, Universidad Panamericana, Augusto Rodin No. 498, Insurgentes Mixcoac, Benito Juárez, Ciudad de México 03920, México; ∥ Grupo de Medicina Molecular y Traslacional (MeM&T), Colegio de Ciencias de la Salud (COCSA), Escuela de Medicina, Universidad San Francisco de Quito (USFQ), Quito 170157, Ecuador; ⊥ Programa de Química, Facultad de Ciencias Básicas, Universidad del Atlántico, Barranquilla 080003, Colombia

## Abstract

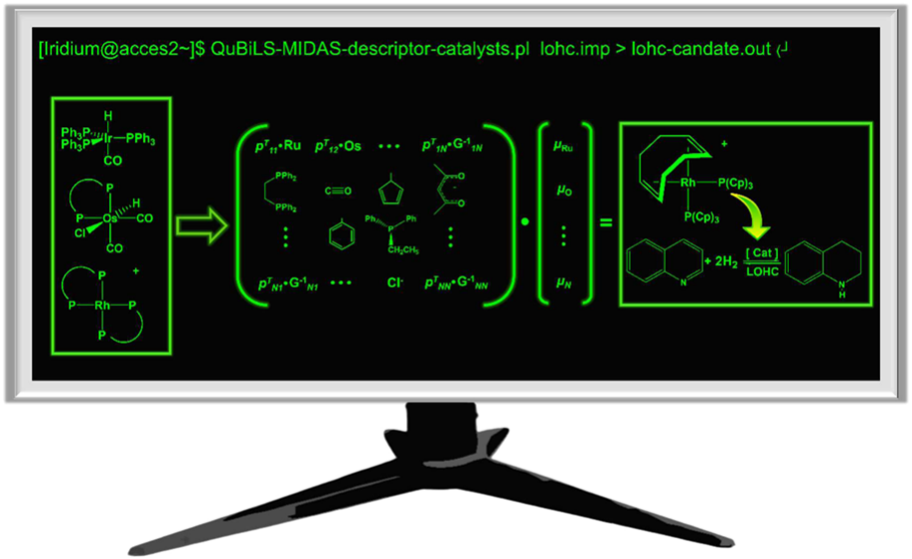

Developing highly active catalysts for quinoline hydrogenation is crucial for efficient hydrogen carrier technologies and clean fossil fuel hydrodenitrogenation. In this work, we employed Tensor Algebra-based 3D-Geometrical Molecular Descriptors (QuBiLS-MIDAS) to develop Quantitative Structure–Property Relationship (QSPR) models predicting the initial rate of homogeneous quinoline hydrogenation catalyzed by transition metal complexes of Ru, Rh, Os, and Ir. A data set of 32 catalytic precursors was used: 25 for model training (training set) and 7 for external validation (testing set). Multiple linear regression analysis yielded a model with good predictive ability for the training set (*R*^2^ = 0.90) and satisfactory external validation for the testing set (Q_EXT_^2^ = 0.86). The model’s descriptors highlighted the importance of hardness, softness, electrophilicity, and mass in predicting catalytic activity. The virtual screening revealed that Rh and Ir complexes with π-acidic ligands (*e.g.*, olefins, diolefins, and η^5^-Cp) and nitrile ligands exhibited the highest predicted catalytic activity, suggesting potential for further improvement through ligand structural modification. Notably, iridium complexes, particularly those with tri(cyclopropyl)phosphine ligands, demonstrated significant potential for hydrogen storage, transport, and production, underscoring their relevance in sustainable energy systems. These findings demonstrate the potential of the QuBiLS-MIDAS approach for *in silico* design of efficient catalysts for quinoline hydrogenation processes.

## 1 Introduction

In the face of growing global challenges, addressing climate change and ensuring energy security have become critical imperatives for humanity’s future. One plausible strategy involves converting unused energy into storable forms and utilizing cleaner biofuels more efficiently during the energetic transition.^1,2^ Certain nitrogen-containing heteroaromatic compounds, including pyridine,^3^ quinoline,^4,5^ acridine,^6,7^ pyrrole,^8−10^ índole,^11,12^ and carbazole,^7,13,14^ have been utilized as model molecules in the hydrodenitrogenation (HDN) reaction related to new biofuel production, and their potential as liquid organic hydrogen carrier (LOHC) materials.^15−17^ Quinoline (**Q**) has gained significant attention due to its dual role in HDN and as an ideal LOHC model. Its simplicity, cost-effectiveness, safety, and capacity to store up to five H_2_ molecules per **Q** molecule (*i.e.*, gravimetric hydrogen carrying capacity up to 7.2%wt H).^3,18−21^

**Scheme 1:**
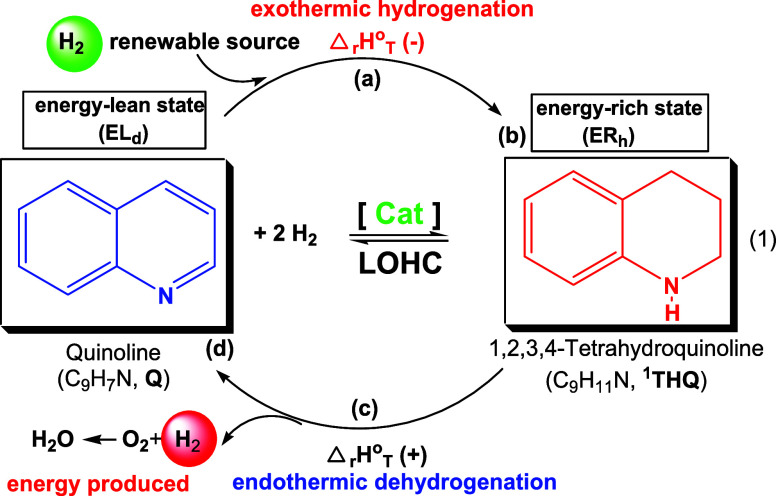
Illustration of LOHC Technology (a) LOHC is hydrogenated to an energy-rich state (ER_h_), (b) hydrogenated LOHC is transported to the end-user, (c) H_2_ is released and used as useful energy, (d) LOHC is dehydrogenated to an energy-lean state (EL_d_), and the dehydrogenated form is transported to the hydrogenation site.

The reversible hydrogenation–dehydrogenation of **Q** to 1,2,3,4-tetrahydroquinoline (^**1**^**THQ**) exemplifies a critical reaction in LOHC technology. It allows the storage of four hydrogen atoms (e.g., H_2_ from renewable sources) in a hydrogen-rich (energy-rich) form and their subsequent release *via* dehydrogenation.^4,22^ This process, illustrated in Scheme 1, provides a prototype for efficient energy storage and release, contributing to the hydrogen economy.^23^

**Q** is a nontoxic liquid at room temperature (melting and boiling points of −15 and 237 °C, respectively) and can be readily obtained through green routes from biomass.^24^ These characteristics significantly enhance its potential as an efficient EL_d_ nucleus in LOHC technologies, offering near-unlimited applicability. In contrast, one of the main disadvantages of using **Q** as an EL_d_ is that, when working with homogeneous catalysts, ^**1**^**THQ** is almost exclusively obtained as the product of quinoline hydrogenation, reducing its overall hydrogen storage capacity. However, the scope of the LOHC based on the **Q**/^**1**^**THQ** pair could be expanded to include other LOHC applications like stationary hydrogen storage technologies, industrial hydrogen logistics, and hydrogen delivery to refilling stations.^25^

Furthermore, the complete catalytic hydrogenation of **Q** is challenging; Chaudret *et al.*(26) reported that RuH_2_ (η^2^-H_2_)_2_(PCy_3_)_2_ hydrogenates the nonheterocyclic ring of **Q**, although its homogeneity has been put in doubt.^27^ In contrast, homogeneous hydrogenation of both hetero- (1,2,3,4-**THQ**) and nonheteroaromatic rings (5,6,7,8-**THQ**), as well as the fully hydrogenated product (decahydroquinoline, **DHQ**), were achieved by a ruthenium dihydride precatalyst, namely RuH_2_(PPh_3_)_4_; for the latter catalyst it was found that **DHQ** was formed by hydrogenation of 5,6,7,8-**THQ**,^28^ which significantly increases the energy cost (*i.e.*, *E*_a_ is higher) during hydrogen charging in the EL_d_. More recently, Chatterjee *et al.*(29) reported the use of the organometallic complex [{RuCl(η^6^-*p*-cymene)}_2_(μ-H-μ-Cl)] as a one-pot dual precatalyst (homogeneous/nanoparticles) for the partial or total hydrogenation of quinoline and other N-heteroaromatic compounds.

Hence, the main problem in the **Q**/^**1**^**THQ** couple is the development of highly active bidirectional hydrogenation catalytic systems combining relatively low cost with high activity, stability, selectivity, and long operation life,^30,31^ whereas highly efficient unidirectional hydrogenation catalysis will be in high demand in the growing biofuel sector.^17,32,33^ Consequently, developing catalysts based on transition metal complexes is a current issue, mainly due to the high selectivity obtained via homogeneous catalysis under moderate reaction conditions.^34^ Although, in recent years, considerable efforts have been dedicated to the development of first-row transition metal-based catalysts (*e.g.*, Mn^35^ and Co^36^) mainly due to their lower cost and improved sustainability, the most studied precatalysts are those based on noble metals, such as Ru, Rh, Os, and Ir.^37^ Additionally, the kinetics and mechanisms of quinoline hydrogenation have been reviewed by Sánchez-Delgado and Rosales.^38^ Understanding these aspects is crucial, as knowledge of reaction kinetics provides insights into the mechanism, aiding in designing more effective catalysts.

Traditional catalyst design methods relied on chemical intuition and a ’trial and error’ approach. These methods involve catalyst synthesis and testing, which are time-consuming, resource-intensive, and often not economically viable. Quantitative structure–property relationships (QSPR) offer a valuable alternative by predicting catalytic activities through molecular descriptor (MD) modeling. QSPR has demonstrated success in various catalytic applications, including predicting enantiomeric excess in asymmetric hydrogenation using constitutional, topological, and geometric descriptors,^39^ enantioselectivity in asymmetric hydroformylation using Rh-diphosphane complexes and GRIND descriptors,^40^ and performance in polymerization reactions by modifying the ligand structure in nickel complexes.^41^ These examples illustrate the potential of QSPR for optimizing both substrates and catalysts, enabling a more rational and efficient design.

Despite the importance of catalytic hydrogenation, its application in QSPR studies is relatively limited. A significant portion of the literature has been dedicated to the prediction of enantiomeric excess, often employing congeneric data sets.^40−42^ In these studies, molecular descriptors (MDs) have frequently focused on ligand codification due to the difficulty in encoding transition metal information (*e.g.*, valence, charge, and spin multiplicity) and the specific geometrical properties of transition metal complexes. Thus, when considering metal characteristics, descriptors are often derived from experimental characterization results,^43,44^ modeling the catalytic activity based on their macroscopic properties. Models encompassing diversity in metal nature and ligands are hence scarce in the literature. Therefore, developing descriptors capable of encoding both electronic and steric information on metal complexes is necessary.

An approach offering a versatile solution is the QuBiLS-MIDAS molecular descriptors [acronym for Quadratic, Bilinear, and *N*-Linear Maps based on *N*-tuple Spatial Metric [(Dis)-Similarity] Matrices and Atomic Weightings].^45^ These descriptors characterize molecules through tensor algebra within the molecular vector space, where vectors store chemical information and matrices represent structural properties. Cabrera *et al*. demonstrated their efficacy in encoding metal-containing compounds for predicting antileishmanial activity in selenium- and tellurium-containing compounds.^46^ This work highlights the potential of these descriptors as a complementary tool for rationalizing experimental catalytic activity in homogeneous catalysts, particularly in uni- and bidirectional quinoline hydrogenation.

This work describes the development of a new QSPR model to predict the initial rate for the hydrogenation of **Q** to ^**1**^**THQ** using a series of Rh, Ir, Ru, and Os-based precatalysts. Molecular structure and chemical information were encoded using the QuBiLS-MIDAS descriptor set. Subsequent analysis of the applicability domain and the descriptor space enabled the identification of promising catalyst candidates. Finally, the predicted catalytic activities were interpreted in the context of established reaction mechanisms.

## 2 Theoretical Background

### 2.1 The QuBiLS-MIDAS Approach

Marrero-Ponce enhanced the concept of topological descriptors by employing tensor algebra to account for higher-order atomic interactions, thus generalizing traditional MDs to describe more intricate molecular details.^47^ In this approach, atoms in a molecule are treated as vector components within an n-dimensional space. Here, a vector represents atomic properties, and a matrix (**G**) encodes the interactions between atoms (*e.g.*, bond connectivity or atomic distances). This matrix can be normalized, yielding variations such as the single (_**ss**_**G**) and double (_**ds**_**G**) stochastic matrices and mutual probability matrix (_**mp**_**G**), which encode atom–atom interaction probabilities.^48,49^

The innovation of QuBiLS-MIDAS lies in expanding these matrices through tensor algebra to capture three- and four-body interactions, encoded in second-, third-, and fourth-rank tensors (**G**, **GT**, **GQ**), also known as *N*-tuple spatial (dis)similarity matrices.^45^ These matrices employ various distance metrics (Minkowski, Chebyshev, and Bhattacharyya) to describe spatial relations between atoms.^49^

Mathematically, QuBiLS-MIDAS generates local vertex invariants (LOVIs) through linear, quadratic, and bilinear mappings between the molecular vector and the structural information matrix.^47^ For example, a bilinear index is calculated from the product of molecular vector components and the matrix **G**. In contrast, higher-order interactions (trilinear and quadrilinear indices) consider tensors **GT** and **GQ**. These LOVIs are then aggregated using operators such as norms, means, and other statistical measures to produce global molecular descriptors.^50^

A further refinement in the QuBiLS-MIDAS approach is the use of cutoff schemes to limit the inclusion of distant atomic interactions based on path length (lag *P* for two-body, lag *3P* for three-body interactions) or angular metrics such as bond angles and dihedral angles.^51^ This comprehensive framework is particularly valuable for modeling the properties of both organic and metal-containing compounds, offering insights into their structure–function relationships. A detailed description of the equations and explanations is provided in the Supporting Information 1 (SI1).

### 2.2 Hydrogenation Mechanisms

The hydrogenation mechanism of **Q** to ^**1**^**THQ** involves several products, intermediates, and isomers, such as 1,2-dihydroquinoline (^**1,2**^**DHQ**), 1,4-dihydroquinoline (^**1,4**^**DHQ**), and 3,4-dihydroquinoline (^**3,4**^**DHQ**).^52^ Based on the most prominent theoretical and experimental conclusions of key research groups (*e.g.*, Sánchez-Delgado *et al.*,^53^ Rosales *et al.*,^54−59^ and Fish *et al*.^60−62^), it can be generally stated that in some species of the catalytic cycle, at least one N-heterocyclic molecule is within the inner coordination sphere of the metal. This observation supports inner-sphere mechanisms (**ISMs**), as illustrated in Scheme S1 in SI1.

In theory, transition state theory (TST) should predict both microscopic (*E*_a_) and macroscopic (**r**_**0**_) parameters. In practice, however, TST’s predictive power is limited, particularly for **r**_**0**_ (*i.e.*, the initial rate as a measure of catalytic activity), although it generally provides reasonable estimations of *E*_a_. Therefore, we will extract information on the reaction mechanism from the optimal **r**_**0**_ obtained in this study from molecular descriptors, without further discussion of TST. A more detailed examination of inner-sphere mechanisms for the homogeneous catalytic hydrogenation of quinoline with Ru, Rh, Os, and Ir catalysts can be found in SI1.

## 3 Methodology

### 3.1 Catalytic Data Set

The homogeneous catalytic hydrogenation of **Q** has been considered a model reaction for elucidating the mechanism of N-heterocycle homogeneous catalytic hydrogenation and the molecular properties influencing hydrogenation efficiency. Data for this end point are limited; therefore, this study compiled information from an extensive literature review on the catalytic hydrogenation of **Q** to ^**1**^**THQ**, supplemented with unpublished data contributed by one of the authors. Thirty-two catalytic precursors were obtained, and relevant data, including initial rates (**r**_**0**_) and catalytic constants (**k**_**cat**_) where available, were used (see Table 1).

**Table 1:** Catalysts Utilized as Training and Test Sets for the Homogeneous Hydrogenation of Quinoline (**Q**) to 1,2,3,4-Tetrahydroquinoline (^1^**THQ)**

entry	catalytic precursors	r_0_ (Ms^-1^)^a^	κ_cat_^b^	experimental conditions^c^					refs
				P (atm)	T (°C)	[M] (mM)	[Q] (mM)	solvent	
training set									
(1)	[Rh(COD)(PPh_3_)_2_]^+^	2.35 × 10^–7^	51.2 M^–2^ s^–1^	1	100	0.3	85	toluene	(53)
(2)	[Rh(COD)(NCPh)(PPh_3_)]^+^	2.78 × 10^–6^	NA	5	50	20	600	CH_2_Cl_2_	(63)
(3)	[Rh(COD)(NCPh)_2_]^+^	2.88 × 10^–5^	NA	5	50	20	600	CH_2_Cl_2_	(63)
(4)	[Ir(COD)(NCPh)(PPh_3_)]^+^	6.11 × 10^–6^	NA	5	50	20	600	CH_2_Cl_2_	(63)
(5)	[Ir(COD)(NCPh)_2_]^+^	1.83 × 10^–5^	NA	5	50	20	600	CH_2_Cl_2_	(63)
(6)	RuHCl(CO)(piperidine)(PPh_3_)_2_	7.80 × 10^–7^	2.0 M^–2^ s^–1^	4	130	1.2	120	xylene	d
(7)	RuHCl(PPh_3_)_3_	1.32 × 10^–6^	NA	5	130	1.0	105	xylene	(59,61)
(8)	RhCl(PPh_3_)_3_	6.58 × 10^–7^	NA	20	85	5.0	50	benzene	(61)
(9)	[Cp*Rh(NCMe)_3_]^2+^	3.56 × 10^–6^	NA	30	40	3.3	66.7	CH_2_Cl_2_	(62)
(10)	[Rh(dppe)_2_]^+^	6.12 × 10^–6^	13.5 M^–2^ s^–1^	4	130	1.0	96	xylene	(57)
(11)	[Rh(Q)_2_(*k*^2^-triphos)]^+^	1.05 × 10^–5^	22.04 M^–2^ s^–1^	4	130	1.0	96	xylene	(56)
(12)	[Ir(Q)_2_(*k*^2^-triphos)]^+^	7.24 × 10^–7^	3.26 M^–1^ s^–1^	4	130	1.0	96	xylene	(56)
(13)	[RuH(CO)(NCMe)_2_(PPh_3_)_2_]^+^	6.74 × 10^–7^	28.5 M^–2^ s^–1^	1	125	0.8	85	xylene	(54)
(14)	[OsH(CO)(NCMe)_2_(PPh_3_)_2_]^+^	1.32 × 10^–6^	47.0 M^–2^ s^–1^	1	125	1.0	85	xylene	(64)
(15)	RuH(CO)*(κ*^*3*^*–OCOCH*_*2*_*Cl)*(PPh_3_)_2_	6.13 × 10^–7^	NA	4	130	1.0	170	xylene	(65)
(16)	RuH(CO)*(κ*^*3*^*–OCOC*_*6*_*H*_*5*_*)*(PPh_3_)_2_	6.94 × 10^–7^	NA	4	130	1.0	170	xylene	(65)
(17)	RuH(CO)*(κ*^*3*^*–OCOCH*_*3*_*)*(PPh_3_)_2_	9.24 × 10^–7^	NA	4	130	1.0	170	xylene	(65)
(18)	RuH(CO)*(κ*^*3*^*–OCOCH(CH*_*3*_*)*_*2*_*)*(PPh_3_)_2_	8.00 × 10^–7^	NA	4	130	1.0	170	xylene	(65)
(19)	OsH(CO)*(κ*^*3*^*–OCOCH*_*2*_*Cl)*(PPh_3_)_2_	2.05 × 10^–7^	NA	4	130	1.0	170	xylene	(65)
(20)	OsH(CO)*(κ*^*3*^*–OCOC*_*6*_*H*_*5*_*)*(PPh_3_)_2_	3.51 × 10^–7^	NA	4	130	1.0	170	xylene	(65)
(21)	OsH(CO)*(κ*^*3*^*–OCOCH*_*3*_*)*(PPh_3_)_2_	4.23 × 10^–7^	NA	4	130	1.0	170	xylene	(65)
(22)	OsH(CO)*(κ*^*3*^*–OCOCH(CH*_*3*_*)*_*2*_*)*(PPh_3_)_2_	2.65 × 10^–7^	NA	4	130	1.0	170	xylene	(65)
(23)	OsHCl(CO)(PPh_3_)_3_	7.85 × 10^–7^	0.04 M^–1^ s^–1^	4	130	1.0	100	xylene	(66)
(24)	Rh(acac)(CO)(P^t^Bu(CH_2_CH = CH_2_)_2_)	1.41 × 10^–5^	6.31 M^–1^ s^–1^	4	130	6.5	80	xylene	(58)
(25)	[Ru(NCMe)_3_(triphos)]^2+^	1.30 × 10^–6^	6.9 × 10^–4^ M^–1^ s^–1^	4	130	1.0	96	xylene	d
test set									
(37)	IrH(CO)(PPh_3_)_3_	7.20 × 10^–7^	NA	4	150	1.0	96	xylene	d
(55)	Rh(acac)(CO)_2_	1.58 × 10^–5^	NA	4	130	1.0	100	xylene	d
(59)	Rh(acac)(CO)(PPh_3_)	1.75 × 10^–5^	NA	4	130	1.0	100	xylene	d
(177)	Rh(acac)(CO)(P^t^Bu(CH_2_CN)_2_)	1.32 × 10^–6^	NA	4	130	1.0	100	xylene	d
(178)	RhH(CO)(PPh_3_)_3_	1.09 × 10^–5^	NA	4	130	1.0	100	xylene	d
(179)	Rh(COE)_2_(Q)_2_^+^	2.00 × 10^–7^	NA	4	130	1.0	100	xylene	d
(180)	Ir(COE)_2_(Q)_2_^+^	5.98 × 10^–7^	NA	4	130	1.0	100	xylene	d

a Initial rate (**r**_**0**_) values for the quinoline hydrogenation, measured as in the reference (67).

b Rate constant of the catalytic reaction.

c T: temperature; P: pressure; [**Q**]: Quinoline molar concentration; [**M**]: molar concentration of the metal

d Rosales, M. Recently obtained results that have not been reported in literature.

Variations in experimental parameters such as temperature, pressure, and solvent can affect **r**_**0**_ by altering the thermodynamic and kinetic environment of the reaction. Currently, there is no consensus on standardized conditions for these experiments to ensure comparability across studies. From an industrial perspective, organometallic catalysts are typically used at temperatures not exceeding 200 °C and pressures around 50 atm. However, laboratory-scale experiments are commonly conducted under milder conditions (*P* < 10 atm and *T* = 130 ± 30 °C).

Table 1 summarizes the experimental conditions reported in the studies from which the data were collected. Most experimental pressures were at or below 4 atm; however, two experiments were conducted at notably higher pressures of 20 and 30 atm. Reported temperatures ranged from 40 to 150 °C, with over 70% of the temperatures falling within the 130 ± 10 °C range. The laboratory-scale conditions in these studies were notably milder, often influenced by the specific catalytic systems and solvents used (*e.g.*, toluene, CH_2_Cl_2_, and xylene). Some of the fastest catalysts require milder conditions for accurate measurement of reaction rates. Conversely, many other catalysts achieve satisfactory conversion rates under more stringent experimental conditions. Consequently, despite some variability, the molecular descriptors are expected to reliably capture the essential chemical and structural information relevant to reactivity.

The next step on data set building involved the systematic division into training and test sets to ensure a rigorous assessment of the model’s reliability. A significant challenge in establishing a rational data-splitting strategy arises from the difficulty in effectively encoding metal-related information with many descriptors. Consequently, a random division based on the response values was implemented. This approach allocated 25 data points to the training set, which is essential for constructing and calibrating the model, and seven precursors to the test set, designated for external validation.

After obtaining the model, the influence of the splitting procedure was analyzed through 75,000 random divisions, followed by a comprehensive evaluation of internal validation, bootstrapping, and external validation, as recommended by Toropov and Toropova.^68^ The molecular data set was constructed to ensure robustness, supporting the development of a predictive model for catalytic activity.

### 3.2 Electronic Structure Calculations

The geometries of the catalytic precursors were optimized using MOPAC2016^69^ with the PM6-D3H4X Hamiltonian. Grimme’s empirical dispersion corrections^70^ were included in all calculations. These corrections accounted for both specific and nonspecific intramolecular interactions affecting the structure of the organometallic compounds and, potentially, their catalytic activity. A vibrational analysis was performed to confirm the absence of imaginary frequencies (negative eigenvalues in the Hessian matrix).

The computational methodology was validated by comparing the calculated geometries with the X-ray crystallographic structures of four representative organometallic compounds. The complexes containing Ru, Rh, Os, and Ir were: RuCl(κ^3^–OCOCH_3_)(CO)(PPh_3_)_2_, OsBr(κ^3^–OCOCH_3_)(CO)(PPh_3_)_2_, RhCl(COD)(PPh_3_), and [IrS_2_(dppe)_2_]^+^.^71−73^ The accuracy of the calculated geometries was assessed in terms of mean unsigned error (MUE), mean signed error (MSE), and relative error (% E_r_) for the angles (a, °), and bond distances (d, Å). The MUE, MSE, and %E_r_ are defined by eqs 1–3, where M is the number of geometrical parameters P_i_ (angles and bond distances).
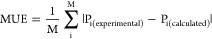
1
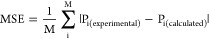
2
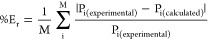
3

### 3.3 Calculation of Molecular Descriptors

The molecular descriptors were calculated with the QuBiLS-MIDAS module of the TOMOCOMD software.^74^ The atomic properties used as weighting of the molecular vectors were: (i) atomic mass (m), (ii) the van der Waals volume (v), (iii) the atomic polarizability (p), (iv) atomic electronegativity in Pauling scale (e), (v) atomic Ghose-Crippen LogP (a),^75^ (vi) Gasteiger-Marsili atomic charge (c),^76^ (vii) atomic polar surface area (psa),^77^ (viii) atomic refractivity (r), (ix) atomic hardness (h), and (x) atomic softness (s). In addition to the primary descriptor set, PaDEL descriptors were calculated to provide a basis for comparison.^78^

### 3.4 Data Modeling

Different multivariable linear models were constructed by varying the atom–atom relations according to the N-tuple indices, i.e., atom pair (*N* = 2), triplet (*N* = 3), and quartet (*N* = 4) relations. Moreover, the different QuBiLS-MIDAS approaches were evaluated: total indices, local-based indices, and cutoff-based indices.

Genetic algorithms (GAs) were employed for variable selection, using the leave-one-out cross-validation coefficient (Q_LOO_^2^) as the fitness function. This variable selection process was applied exclusively to the training set to prevent bias in model performance evaluation. GA evolved over 1000 generations with a population size of 10,000 models. Mutation and crossover rates were set at 1 and 50%, respectively.

The final GA population was stored in a data frame containing the indices of the descriptors included in each model and their corresponding *R*^2^, Q_LOO_^2^, and Q_ext_^2^ values. Better models were extracted based on the maxima values of these metrics. Additional selection criteria included the presence of statistically significant variables (*p* < 0.05) and adherence to statistical assumptions. The statistical robustness of the selected models was further verified using bootstrapping (500 resamplings) and Y-randomization (5000 resamplings) to evaluate and rule out overfitting and chance correlations.

The applicability domain of the model was determined using the Williams plot (leverage vs standardized residuals) to identify outliers and influential observations. Subsequently, the predictive accuracy of the model was assessed by predicting the activity of the compounds in the test set and calculating the external validation coefficient (Q_ext_^2^). Prior to these predictions, the membership of all test set compounds within the model’s applicability domain was verified. All calculations were performed using the statistical software R.^79^

### 3.5 Virtual Screening of the Molecular and Descriptor Space

The descriptor space was evaluated by mapping the catalytic activity onto a dimension-reduced descriptor space. Neighborhood components analysis (NCA) was employed to visualize the multidimensional descriptors in a two-dimensional (2D) space. NCA is a supervised learning method based on a nearest-neighbor procedure to identify the most probable cluster based on a given distance metric. The objective is to find a linear transformation that maximizes the probability of correctly classifying a point using nearest neighbors in the transformed space.^80^

The initial reaction rate (**r**_**0**_) was mapped onto the NCA descriptor space to identify catalysts corresponding to local maxima. Subsequently, various structural modifications of the ancillary ligands of these catalysts were modeled and optimized, and their initial reaction rates (**r**_**0**_) were predicted using the QSPR model. The novelty of these new candidates within the test and prediction sets was then evaluated using the Local Outlier Factor (LOF) method.^81^ Analysis of the molecular descriptors and predicted initial reaction rates (**r**_**0**_) provided insights into the chemical properties, enabling the proposal of new potential catalysts with promising catalytic activities.

## 4 Results and Discussion

### 4.1 Geometry Optimization of Molecular Complexes of Ru, Rh, Os, and Ir Toward Quinoline Hydrogenation Process

**Figure 1:**
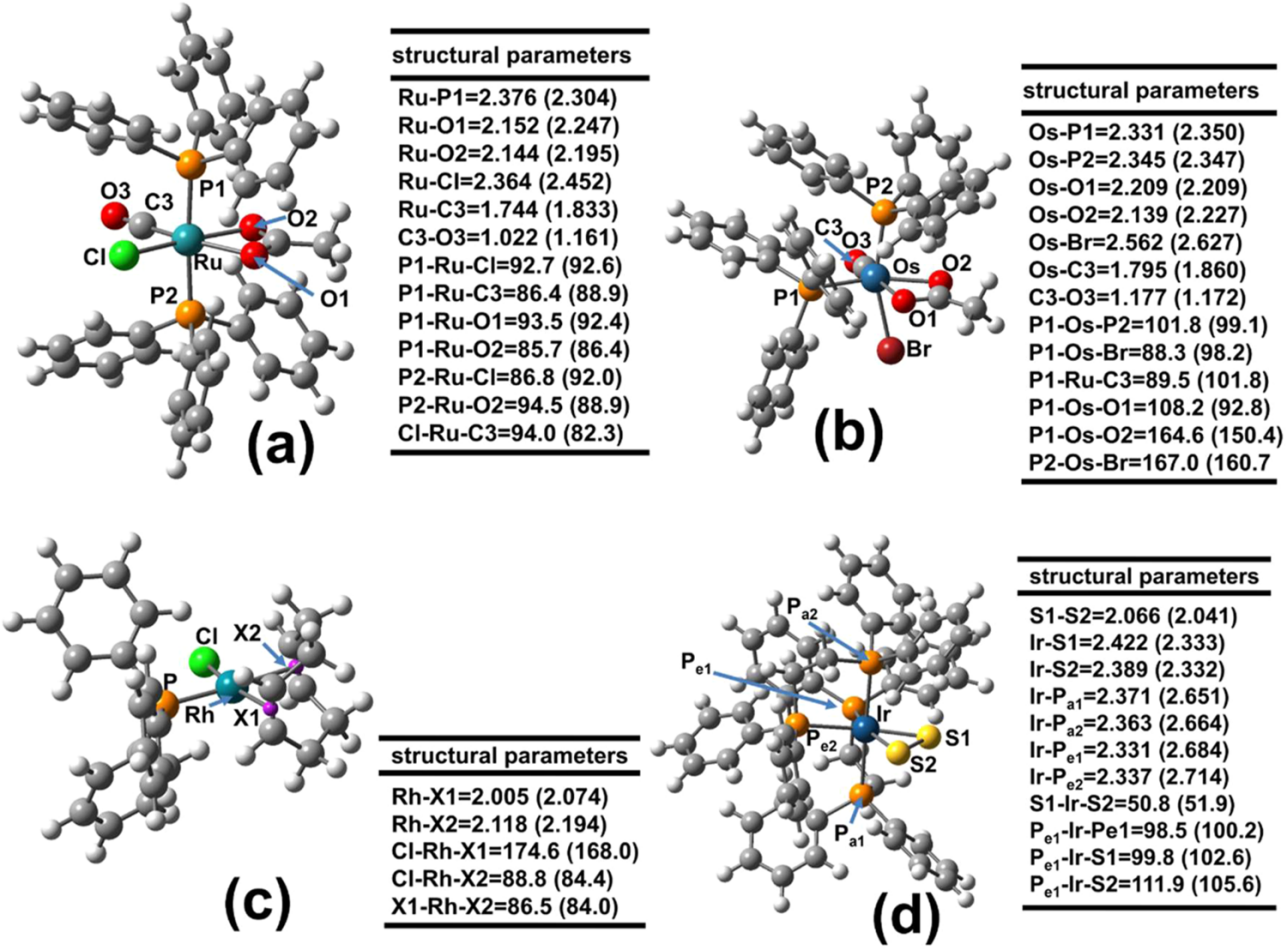
Optimized structures of: (a) RuCl(κ^3^–OCOCH_3_)(CO)(PPh_3_)_2_, (b) OsBr(κ^3^–OCOCH_3_)(CO)(PPh_3_)_2_, (c) RhCl(COD)(PPh_3_), and (d) [IrS_2_(dppe)_2_]^+^. Comparison between experimental and calculated structural parameters: bond angles (α, °) and bond distances (d, Å). Calculated values obtained with PM6-D3H4X are shown in parentheses.

Figure 1 shows the optimized geometries of four representative organometallic compounds whose structures were determined by X-ray diffraction and used to validate the computational methodology. See SI2 for details of the calculated structural parameters. Table 2 summarizes the overall performance of the PM6-D3H4X method for predicting the structural parameters. Based on the MSE values, the PM6-D3H4X method tends to slightly overestimate the experimental bond distances by approximately 0.056 Å for the Ru, Rh, Ir, and Os complexes (see also all bond distances in S2, Figure S2). Conversely, it underestimates the experimental bond angles by only 0.6° (see Figures 1 and S2). While the MUE and MSE values for bond distances show no substantial differences, the MSE for bond angles (ca. 4.0°) is significantly lower than the corresponding MUE. This result is likely due to a fortuitous cancellation of errors, consistent with the performance observed for calculations of structural parameters of europium (Eu), gadolinium (Gd), and ytterbium (Yb) complexes using less sophisticated semiempirical methods such as PM3.^82^

**Table 2:** Mean Unsigned Error (MUE), Mean Signed Error (MSE), and Average Relative Error (% E_r_)^a^ for Bond Distances (d, Å) and Bond Angles (α, °) Calculated with PM6-D3H4X for the Four Complexes Shown in Figure 1

distances (Å)			
method	MUE	MSE	% E_r_
PM6-D3H4X	0.084	–0.056	4.1

angles (°)			
method	MUE	MSE	% E_r_
PM6-D3H4X	4.6	0.6	4.4

a MUE, MSE and % Er with respect to X-ray structure experimental studies^71−72,73^.

The MUE and MSE values in Table 2 are comparable to those reported in previous studies validating PM*n* methods (*n* = 3, 5, and 6) for the structural modeling of cobalt derivatives of vitamin B_12_ complexes.^83^ It is well-known that molecular geometries, especially those involving transition metal centers, can be influenced by crystal packing effects, leading to deviations from their gas-phase geometries. The extent of these deviations depends on the molecular flexibility of the structure. Typically, MUE and MSE values are in the range of 0.01–0.02 Å for metal–ligand bond distances and 1–2° for ligand–metal–ligand bond angles.^84^ The MUE and MSE values presented in Table 2 for bond distances are within the range of errors reported for metal–ligand bond distances calculated with Density Functional Theory (DFT) methods (0.010–0.080 Å).^85,86^ Finally, the %E_r_ values for both angles and bond distances were less than 5%. These results demonstrate reasonable agreement between the PM6-D3H4X-calculated and experimental structures of the Ru, Rh, Os, and Ir organometallic compounds.

In summary, the semiempirical method provides geometrical parameters comparable to crystallographic data. Therefore, the obtained geometries represent a suitable starting point for molecular descriptor calculations. Key geometrical parameters for catalytic precursors **1** – **25** optimized using the PM6-D3H4X method are shown in Figures S3 to S27 in the SI3. These optimized structures served for descriptor calculations.

### 4.2 QuBiLS-MIDAS Models for Predicting Initial Rates

Table 3 presents the best models developed using QuBiLS-MIDAS and PaDEL descriptors^78^ obtained through genetic algorithms. The goodness-of-fit statistics, particularly *R*^2^, indicate that the QuBiLS-MIDAS descriptors yielded a superior model (*R*^2^ > 0.9). Q_LOO_^2^ and Q_BOO_^2^ demonstrated the robustness of these models, as evidenced by *R*^2^ – Q_LOO_^2^ < 0.1 and Q_BOO_^2^ values close to *R*^2^.^87^ While the PaDEL-based model falls within acceptable ranges, its Q_LOO_^2^ value is near the acceptability threshold, and its overall performance is inferior to that of the QuBiLS-MIDAS descriptors. Consequently, further analysis will focus on the latter model.

**Table 3:** Statistical Figures of Merit for the Best Models Obtained with Genetic Algorithms for Predicting the Initial Rate of Homogeneous **Q** Hydrogenation

	coefficient	VIF	importance	abbr.
QuBiLS-MIDAS (TOMOCOMD)				
intercept	–5.72(35)			D0
I50_F_AB_nCi_2_M12_SS1_T_LGP[5]_s_MID	7.90(97)	1.2	8.1	D1
GM_B_AB_nCi_2_M15_SS3_T_LGL[1–2]_e-s_MID	1.72(30)	1.4	5.7	D2
S_B_AB_nCi_2_M3_SS4_T_LGL[2–3]_m-h_MID	–0.443(71)	1.4	6.2	D3
VC_Q_AB_nCi_2_M15_NS4_T_KA_m_MID	0.161(61)	1.3	2.6	D4
goodness-of-fit figures of merit	*R*^2^ = 0.902; s = 0.210; F(5,20) = 46.1			
internal validation	Q_LOO_^2^ = 0.847 and Q_BOO_^2^ = 0.859			
Y-randomization	*R*^2^r = 0.17 and Q^2^r = −0.32			
external validation	Q_ext_^2^ = 0.860			
statistical assumptions tests	Shapiro-Wilk = 0.978 (0.848)			
	Durbin-Watson = 2.444 (0.234)			
PaDEL descriptors (ChemDes)				
intercept	–5.36(15)			P0
ATSC5c	–7.98(1.36)×10^–2^	4.2	5.8	P1
RDF60p	1.33(19)×10^–2^	3.7	4.2	P2
ATSC8p	–2.28(75)×10^–2^	2.0	4.6	P3
MATS 3p	–5.58(49)	1.6	7.7	P4
goodness-of-fit figures of merit and validation				
goodness-of-fit figures of merit	*R*^2^ = 0.828; s = 0.278; F(5,20) = 24.1			
internal validation	Q_LOO_^2^ = 0.730 and Q_BOO_^2^ = 0.692			
Y-randomization	*R*^2^r = 0.17 and Q^2^r = −0.32			
external validation	Q_ext_^2^ = 0.634			
statistical assumptions tests	Shapiro-Wilk = 0.980 (0.892)			
	Durbin-Watson = 0.336 (0.005)			

**Figure 2:**
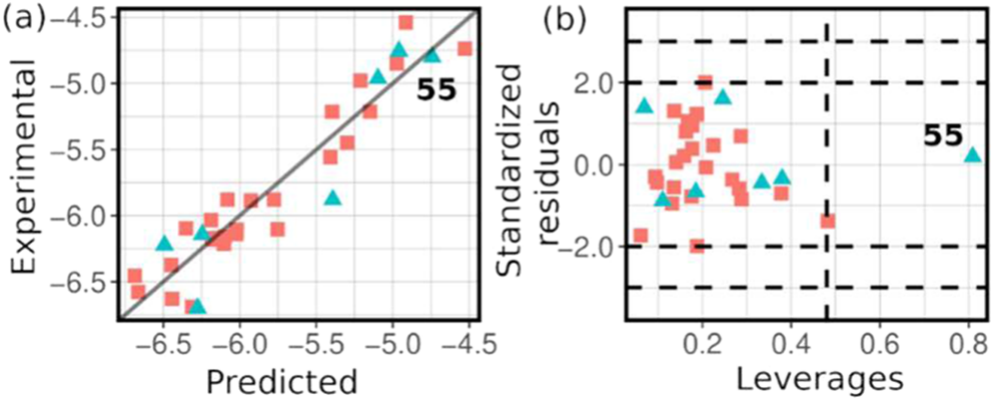
(a) Experimental vs calculated reciprocal logarithm of the initial rate [log(**r**_**0**_)] of quinoline catalytic hydrogenation; (b) Williams plot.

Chance correlation was excluded based on the low mean *R*^2^ and Q_LOO_^2^ values obtained from the Y-randomization test. Statistical assumptions further support the model’s reliability for accurately predicting the response. Figure 2a illustrates the predicted versus experimental **r**_**0**_ values, demonstrating the linearity of the model. The residuals are normally distributed, as confirmed by the Shapiro-Wilk test (*p* > 0.05, Table 3), and this is corroborated by the Q-Q plots shown in Figure S35a in the SI4. The Durbin-Watson test results (Table 3) suggest that the model’s residuals are independent (*p* > 0.05). Additionally, these residuals are homoscedastic, as shown by the plot of residuals vs fitted values (see Figure S35b,c). Regarding multicollinearity, the variance inflation factor (VIF) was close to one in all cases, indicating that each descriptor contained unique information about the catalytic precursor related to the initial rate in the catalytic hydrogenation of **Q** to ^**1**^**THQ**.

After 75,000 random train/test splits, the model demonstrated consistent *R*^2^ and Q_LOO_^2^ values, with median values of 0.89 and 0.85, respectively. The external predictive power was robust, with a Q_EXT_^2^ median of 0.86 and an interquartile range (IQR) of 0.80–0.90. Although occasional low Q_EXT_^2^ values were observed, the majority of results fell within a reliable range. Full details and statistical analyses are provided in SI4.

The applicability domain, analyzed using the Williams plot, revealed one influential observation in the training set (Figure 2b), but its leverage value (0.48) was at the defined limit. In the test set, the influential observation (labeled **55**, [Rh(acac)(CO)_2_]) was well within the applicability domain (leverage <0.81). However, the predicted value is close to the experimental value, which positively contributes to the external validation coefficient. This observation’s location in the high-activity region, where few training data points are available, results in increased uncertainty in the predicted value and a higher leverage value.

The molecular descriptors included in the final model are listed in Table 3. The logarithm of the initial rate is used as the response variable in this model to smooth fluctuations in **r**_**0**_. Each coefficient value indicates the expected change in the response associated with a one-unit increase in the corresponding descriptor. A positive coefficient indicates a direct relationship, meaning that an increase in the descriptor leads to an increase in log(**r**_**0**_), *i.e.*, a decrease in activity. Conversely, a negative coefficient indicates an inverse relationship. The descriptor importance reflects the effect size, suggesting a more substantial impact on the response with a one-unit change in the descriptor.

The most influential variable is I50_F_AB_nCi_2_M12_SS1_T_LGP[5]_s_MID (D1), which accounts for 36% of the determination coefficient. This descriptor encoded the effect of neighboring atoms up to the fifth bond on the atom’s softness (**s**). The atom pair interactions beyond this distance are negligible; thus, long-range interactions do not contribute substantially. In the model, an increase in this descriptor leads to a decrease in catalytic activity.

Another descriptor related to atomic softness is GM_B_AB_nCi_2_M15_SS3_T_LGL[1–2]_e-s_MID (D2), representing 25% of the model’s importance. In this descriptor, softness interacts with electronegativity (**e**) at short-range distances (1.0–2.0 Å), showing a similar effect to D1. These descriptors highlight that the relationship between atomic softness, electronegativity, and catalytic activity is not linear. The model captures a more complex interaction in which these factors, while individually beneficial in some contexts, do not necessarily lead to increased catalytic performance.

In contrast, the S_B_AB_nCi_2_M3_SS4_T_LGL[2–3]_m-h_MID (D3) descriptor, which combines atomic mass (**m**) and hardness (**h**), promotes increased catalytic activity as this descriptor increases. It contributes 27% to the model, indicating that a balance between steric and electronic factors is essential for determining catalytic behavior.

The interplay of increasing and decreasing local quantities, such as hardness, softness, and electronegativity, plays a crucial role in modulating catalyst performance, as captured by the model employed in this study. Literature studies provide further insights into these roles through specific examples. Weissinger *et al.*(88) designed heterobimetallic complexes to combine a harder metal center for hydride generation with a softer metal for substrate binding, illustrating how these complementary properties enhance catalytic efficiency. Similarly, Chen *et al*.^89^ proposed that the metal’s electronegativity influences whether the hydrogen bound to its surface exhibits a hydride or proton-like character, altering its attack mode on CO_2_. High electronegativity promotes hydrogen’s proton character, enabling it to attack oxygen, while low electronegativity enhances the hydride character, facilitating an attack on carbon. These studies highlight the interplay of electronic properties in modifying catalytic activity. Nevertheless, the QuBiLS-MIDAS descriptors effectively capture the overall balance of electronic properties, including fluctuations in hardness, softness, and electronegativity, and generalize them within the total descriptor. This ability to encapsulate such variations makes these descriptors valuable tools for catalyst design, as their integration could help enhance catalytic activity.

Evaluating the overall performance of this model in direct comparison with existing literature is challenging due to the scarcity of comparable models. While significant effort in QSPR modeling in catalysis has focused on predicting outcomes such as enantioselectivity^39,40,90^ and product distributions,^91−93^ to our knowledge, no QSPR models specifically designed for predicting **r**_**0**_ in general catalytic hydrogenation have been reported. Therefore, although a direct performance comparison is not straightforward, the obtained model demonstrates high accuracy and robustness within its specific context (Table 4). Furthermore, despite this limitation in direct comparison, the model exhibits excellent goodness-of-fit metrics, comparable to those reported for other QSPR applications.

**Table 4:** Comparison of the Model Obtained in this Work with Published QSPR Models

descriptors (models)	n^a^	*R*^2^^b^	q^2^^c^	method^d^	refs
QuBiLS-MIDAS	25	0.90	0.85	RLM	this work
GRIND	14	0.94	0.80	PLSR	(90)
WHIM	13	0.9	0.7	PLSR	(39)
GRIND	21	0.9	0.7	PLSR	(40)
constitutional, topological, geometric and electronic	14	0.9		PLSR	(91)
topological: bite angle; electrostatic: relations n: iso	39	0.8		PLSR	(92)
topological: bite angle and flexibility	80	0.7–0.9		PLSR and ANN	(93)

a Number of molecules present in the model.

b Correlation coefficient,

c Cross validation.

d Method for obtaining the model, RLM: multiple linear regression, PLSR: Regression for Partial Least Squares, and ANN: Artificial Neural Network.

Additionally, this model’s robustness and predictive power mitigate potential inconsistencies arising from the nonstandard experimental parameters reported in the literature. The experimental conditions were generally mild, with pressures below 5 atm and temperatures around 130 °C. The model demonstrated excellent predictive performance on the test set despite the broader range of experimental parameters used in the training set (complexes **1**–**25**, Table 1). These results highlight the model’s reliability in capturing the essential molecular determinants of catalytic activity despite the variability in reaction conditions. Moreover, this model provides a valuable starting point for acquiring more extensive and higher-quality data sets for further refinement and standardization of experimental conditions.

### 4.3 Virtual Screening of Molecular and Descriptor Spaces

**Figure 3:**
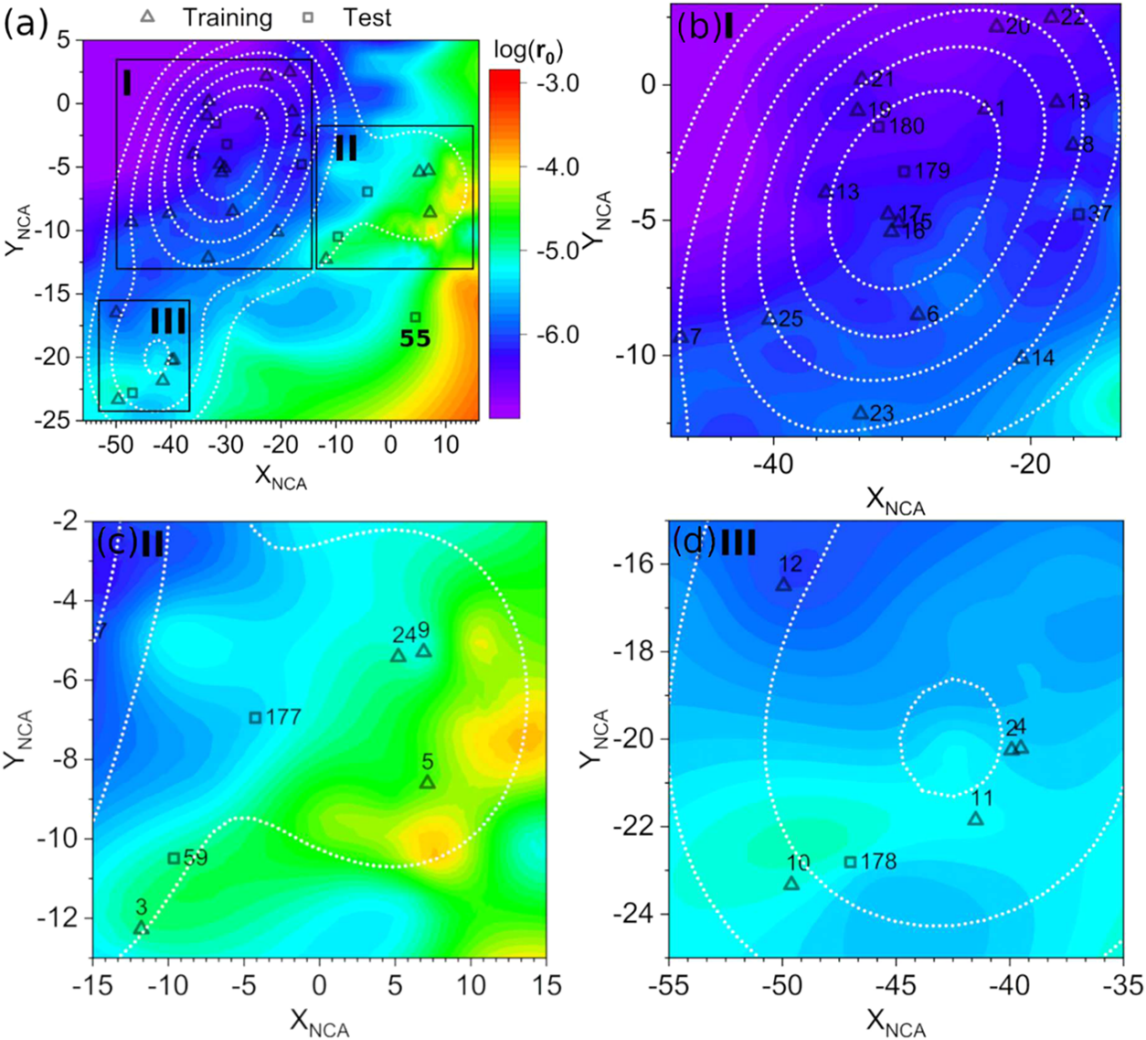
(a) Applicability domain of the obtained model supplied by neighborhood component analysis, mapped with the predicted logarithm of the initial rate for catalytic homogeneous quinoline hydrogenation, log(**r**_**0**_). The dotted white line represents the density of points projected in the NCA space. Panels (b), (c), and (d) provide a zoomed-in view of three identified zones in (a).

Figure 3 shows the neighborhood component analysis (NCA) projection of the descriptor space, mapped with the predicted initial rate. The new dimensions were evaluated using a k-nearest neighbor clustering analysis, which resulted in a classification accuracy of 1.00 for the training set and 0.86 for the test set. The local outlier factor analysis indicated no outliers in the training set, and all test set points were within the applicability domain, except for test complex **55**. This exclusion was observed in the Williams plot, where the density of points in the training set excluded this complex from the applicability domain, see the white dotted line in Figure 3a. Despite this limitation, the results validate the model’s robustness for predictions and assessing the novelty of potential candidates.

Analysis of the activity contour map (Figure 3a) identifies three distinct regions. Region I (Figure 3b) comprises 16 compounds characterized by the lowest activity values [log(**r**_**0**_) > −5], among which precursors **7** and **25** exhibit the highest activity within the ruthenium (Ru) complexes. Region II (Figure 3c) encompasses compounds exhibiting the highest activity levels, with catalysts **3**, **5**, **9**, and **24** representing the activity maxima for rhodium (Rh) and iridium (Ir)-based catalysts. Region III (Figure 3d) contains compounds with moderate activities, exemplified by complexes **10** and **11**, which share similar reaction mechanisms, as reported by Rosales *et al*.^56,57^

**Scheme 2:**
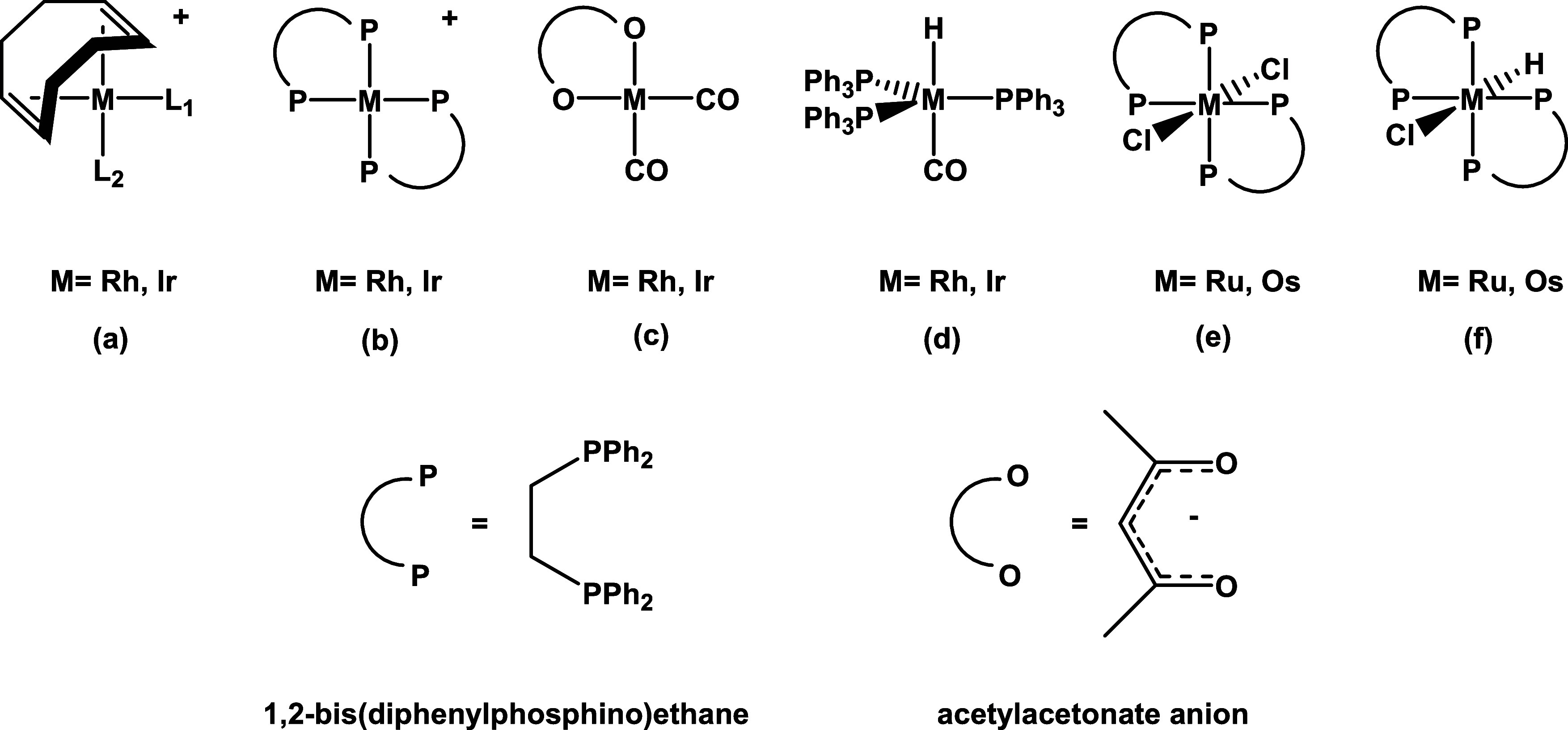
Complexes Used as Starting Points (a–c) and Some Structural Nuclei for New Candidates (d–f) to Explore the Chemical Space and Improve Catalytic Activity

**Scheme 3:**
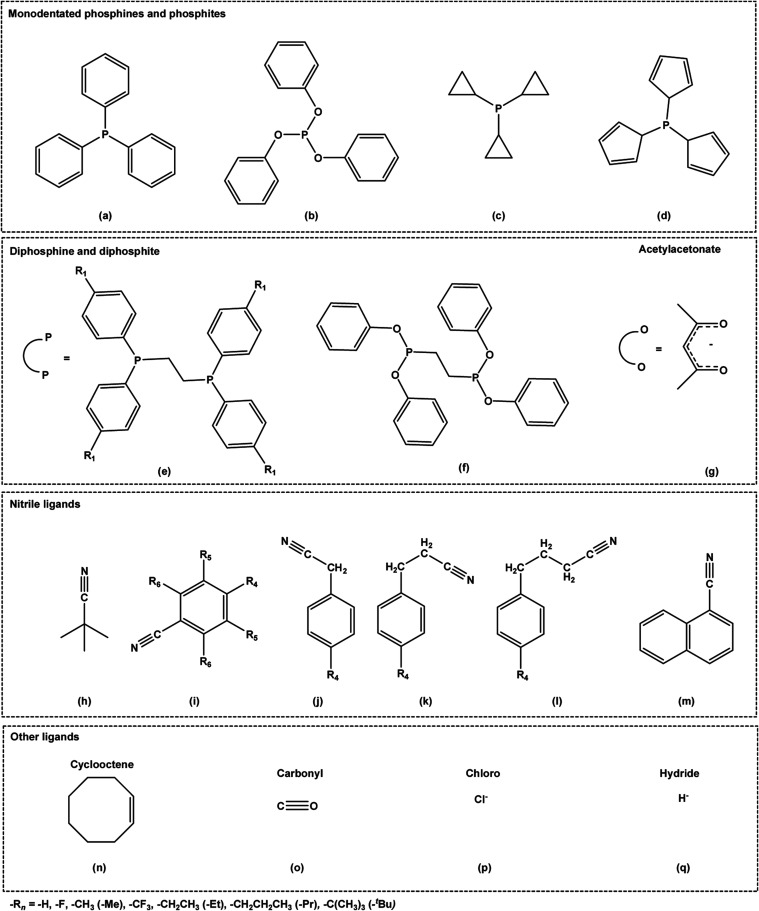
Ligand Modification Proposals to Explore the Chemical Space and Enhance Catalytic Activity: Monodentate Phosphines and Phosphites (a–d), Diphosphines and Diphosphites (e,f), Acetylacetonates (g), Nitriles (h–m), and Other Ligands (n–q)

The precursors identified in Regions I and III demonstrate potential efficacy and are promising candidates for further study. The model predicts only slight changes in activity upon ligand modification for Ru and Os precatalysts, whereas significant variations are predicted for Rh and Ir complexes. Consequently, a more in-depth analysis of the factors governing the catalytic performance of these Rh and Ir complexes is required. The structures with Rh and Ir were subsequently used as starting points for metal and ligand modifications to explore the chemical space and enhance activity (Schemes 2 and 3). Detailed structural information and predicted initial reaction rates (**r**_**0**_) are available in SI3 and SI5.

#### 4.3.1 New Catalysts for Quinoline Hydrogenation: Influence of Phosphine- and Phosphite-Type Ligands

Catalyst **1** was selected for initial investigation due to its superior electronic and steric properties compared to structural analogs 2–5 (Table 1). Its catalytic activity was measured under mild reaction conditions (*T* = 370 K and P = 1.0 atm), further supporting its potential as a promising candidate for this reaction, as previously reported by Sánchez-Delgado *et al.*(53) Mechanistically, among complexes containing the 1,5-cyclooctadiene (COD) ligand, only complex **1** has been experimentally shown to retain the COD ligand within the metal coordination sphere throughout the catalytic cycle *via* an inner-sphere mechanism (**ISM**) without undergoing hydrogenation. This intrinsic thermodynamic resistance to COD hydrogenation could positively influence the reversible hydrogenation-dehydrogenation of **Q** to ^**1**^**THQ**. Indeed, in our previous work,^94^ DFT calculations (M06L/LanL2DZ//6–31+G(d,p)) showed that all elementary steps of the catalytic cycle *via* the **ISM** were reversible, except the reductive elimination of ^**1**^**THQ**. Furthermore, the π-acidic nature of the 1,5-cyclooctadiene (COD) ligand plays a crucial role in the hydrogenation of **Q** to ^**1**^**THQ** through electronic effects when the [Rh(COD)(PPh_3_)_2_]^+^ precursor is employed.

**Figure 4:**
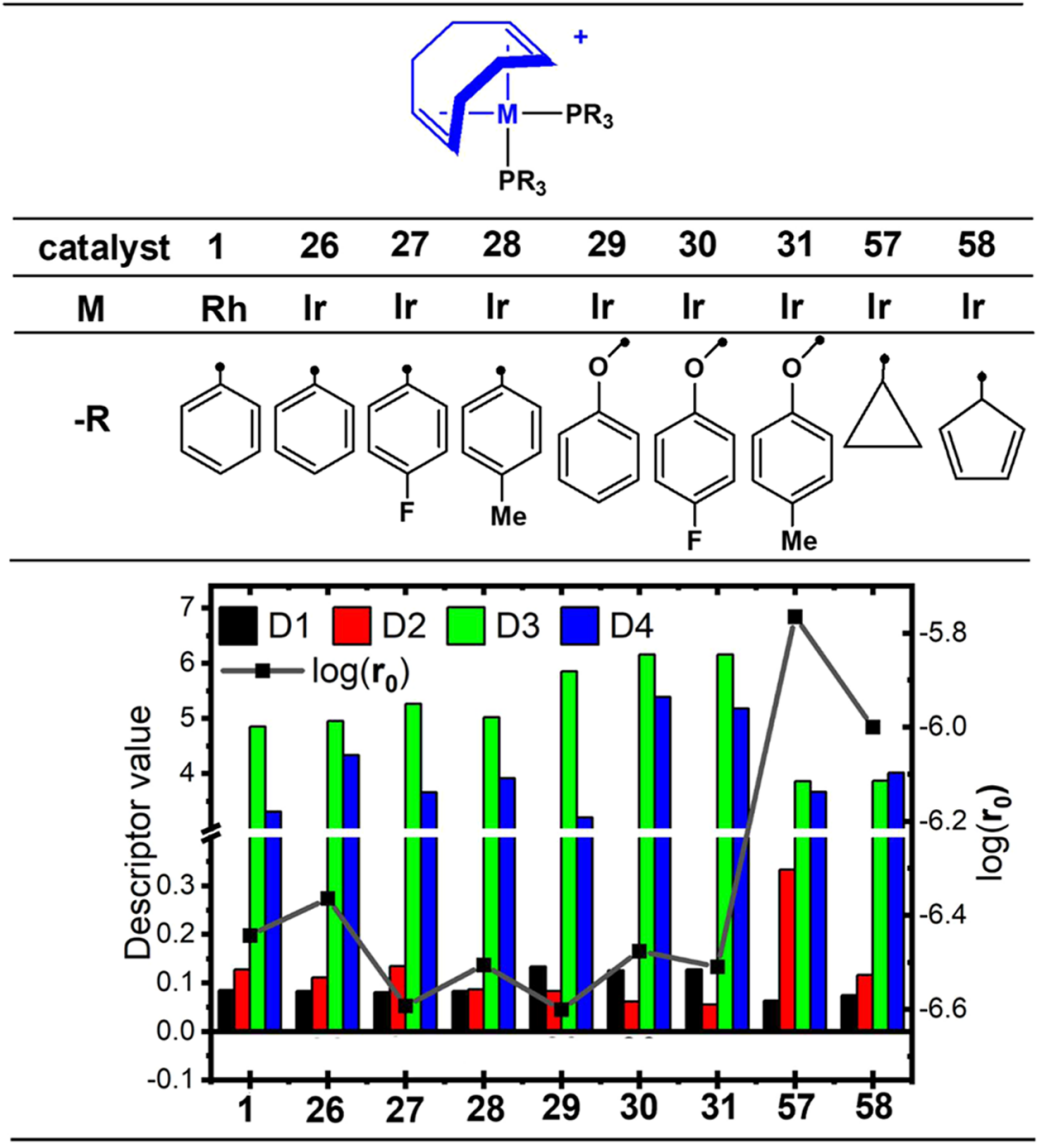
Relation of predicted initial rate with descriptor values for Ir complexes [Ir(COD)(PR_3_)_2_]^+^ by modification of metal and phosphine in complex **1** as a function of molecular descriptors. The ● symbols denote the connection points.

The experimental **r**_**0**_ of catalyst [Rh(COD)(PPh_3_)_2_]^+^ (**1**) was 2.35 × 10^–7^ M·s^–1^, significantly lower than the maximum initial rate (∼10^–5^ M·s^–1^). To address this limitation, a virtual screening study was performed, involving metal substitution and ligand modification to develop novel catalysts (Schemes 2a and 3a–d). Upon replacing the central rhodium atom in complex **1** with iridium to form complex **26**, [Ir(COD)(PPh_3_)_2_]^+^, descriptors D1 and D2 exhibited slight decreases, while D3 and D4 increased, with D4 showing a more pronounced increase than D3. The increase in D3 correlated with a decrease in activity, whereas increases in D1, D2, and D4 correlated with enhanced activity (see regression coefficients in Table 3). Consequently, catalyst **26** demonstrated a modest increase in activity (Figure 4, **r**_**0**_ = 4.32 × 10^–7^ M s^–1^ in SI5). The descriptors associated with electronic properties showed only marginal changes, suggesting that the observed activity enhancement can be primarily attributed to the difference in molar mass.

Similarly, complexes **27**–**31** followed this trend (Figure 4), with **r**_0_ values ranging from 2.56 × 10^–7^ M s^–1^ to 3.3 × 10^–7^ M s^–1^ (SI5). The *para* substitution of the phenyl groups on the triphenylphosphine ligands had no substantial effect on the predicted **r**_**0**_ for **Q** hydrogenation (*e.g.*, see the structures for the ligands P(Ph)_3_ and P(Ph-*p*-F)_3_ in Scheme 3a and Figure 4, respectively). The unfavorable influence on the activity is primarily attributed to descriptor D3, related to the mass and hardness of neighboring atoms separated by 2–3 Å (*i.e.*, noncovalent interactions). For example, in complex **27** ([Ir(COD)(P(Ph-*p*-F)_3_)_2_)]^+^, Figure 4, the increased hardness of the P(Ph-*p*-F)_3_ ligand compared to P(Ph)_3_ affected the noncovalently contacted neighboring atoms, leading to an increase in D3. Likewise, the opposite effect in complex **28**,[Ir(COD)(P(Ph-*p*-Me)_3_)_2_)]^+^, Figure 4, is consistent with the reduced hardness attributed to the methyl group. This general trend was also observed with P(OPh-*p*-X)_3_, but with an overall increase in D3 compared to the first three catalysts. In these groups, descriptors D1 and D2 canceled the electronic contribution, resulting in a slightly altered activity trend addressed by D3.

Interestingly, substituting the phenyl groups in PPh_3_ of catalyst **26** with cyclopropyl (complex **57**, [Ir(COD)(P(C_3_H_5_)_3_)_2_]^+^) and cyclopentadienyl (complex **58**, [Ir(COD)(P(Cp)_3_)_2_]^+^) resulted in a decrease in D3 and an increase in **r**_**0**_ to 1.72 × 10^–6^ M·s^–1^ and 1.01 × 10^–6^ M·s^–1^, respectively (see SI5). This trend was also observed for catalysts **187** ([Rh(COD)(PEtPh_2_)_2_]^+^, **r**_**0**_ = 6.81 × 10^–7^ M s^–1^) and **188** ([Ir(COD)(PEtPh_2_)_2_]^+^, **r**_**0**_ = 5.99 × 10^–7^ M s^–1^), suggesting that the electronic nature of the ligands plays a crucial role in directing catalytic activity (structures of **187** and **188** are shown in SI5). In complex **57**, the elevated **r**_**0**_ value is, in addition to the aforementioned factors, attributed to an increase in D2.

**Figure 5:**
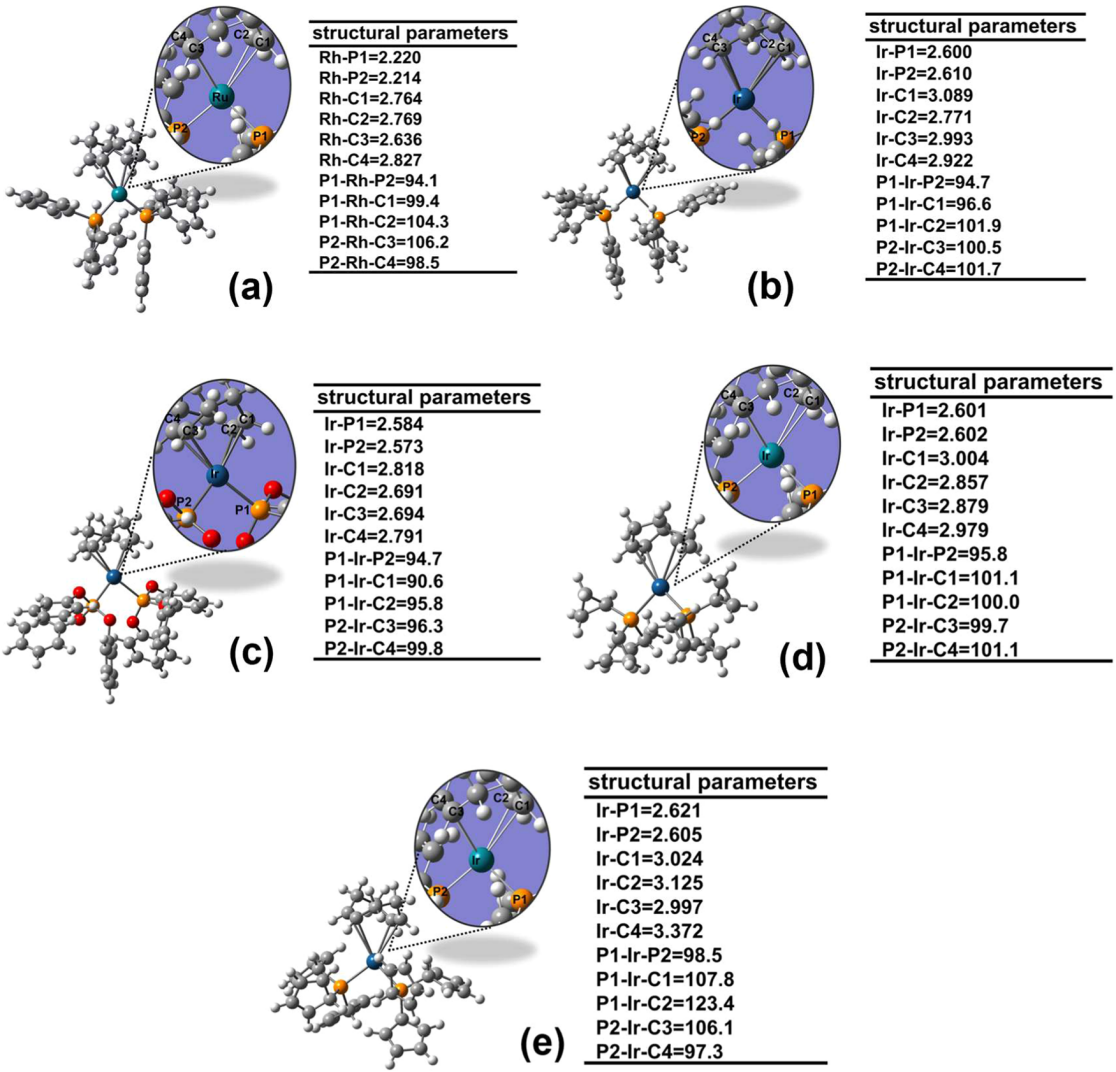
Optimized structures of: (a) [Rh(COD)(P(Ph)_3_)_2_]^+^ (**1**), (b) [Ir(COD)(P(Ph)_3_)_2_]^+^ (**26**), (c) [Ir(COD)(P(OPh)_3_)_2_]^+^ (**29**), (d) [Ir(COD)(P(C_3_H_5_)_3_)_2_]^+^ (**57**), and (e) [Ir(COD)(P(Cp)_3_)_2_]^+^ (**58**). Angles (α, °) and bond distances (d, Å).

To our knowledge, no reports of Ir-based catalysts achieving reversible hydrogenation–dehydrogenation of **Q** to ^**1**^**THQ***via***ISM**. Conversely, Ir-based catalysts are known to be best for reversible **Q** hydrogenation-dehydrogenation.^95^ Thus, we propose [Ir(COD)(PC_3_H_5_)_2_]^+^ (**57**) as the best starting point for the rational design of novel disruptive **ISM** catalysts for LOHC technologies (see optimized structures of the best candidates in Figure 5). Currently, it can be a hot topic in the experimental catalytic field.

These differences between rhodium and iridium catalysts of the [M(COD)(PPh_3_)_2_]^+^ type are attributed to variations in the reaction mechanism. A kinetic and mechanistic study by Sánchez-Delgado *et al*.^53^ on the hydrogenation of quinoline with the rhodium complex revealed the formation of [Rh(COD)(Q)_2_]^+^ (resulting from dissociation of the two PPh_3_ ligands) under the reaction conditions; this species was identified as the active catalyst. However, in the case of the iridium analog, evidence suggests that the two triphenylphosphine ligands remain coordinated to the metal center, potentially forming a dihydride species, [Ir(H)_2_(Q)_2_(PPh_3_)_2_]^+^, as reported by Rosales *et al*.^96^ This latter aspect is of particular interest for [M(COD)(P(C_3_H_5_)_3_)_2_]^+^ (M = Rh, Ir), as this highly basic and relatively unbulky phosphine is likely to remain coordinated to the metal center, forming species such as [M(H)_2_(Q)_2_(P(C_3_H_5_)_3_)_2_]^+^ (M = Rh, Ir). These species exhibit higher catalytic activity than those containing less basic phosphines or those lacking phosphine ligands.

**Figure 6:**
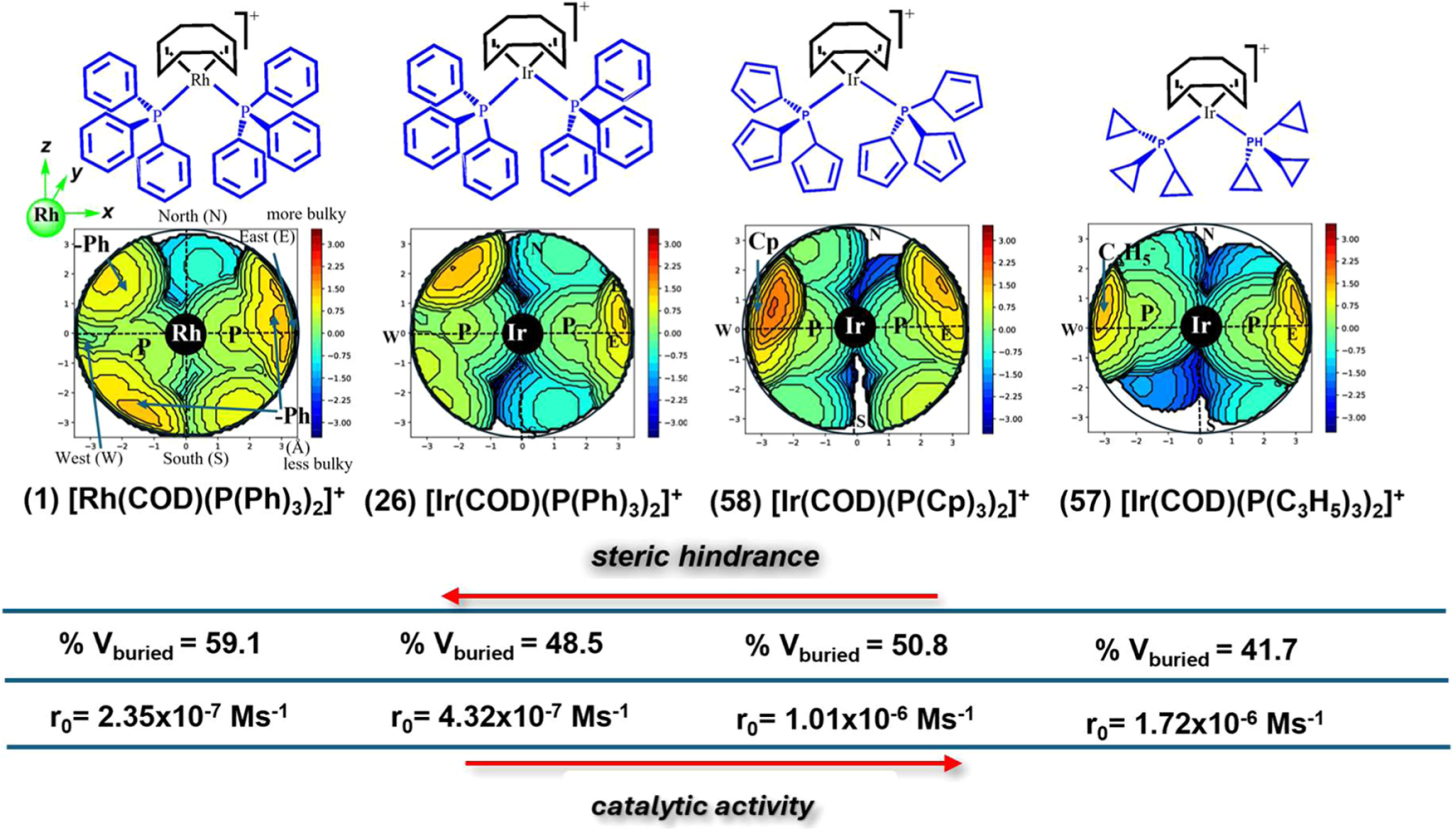
Steric maps of complexes **1**, **26**, **57**, and **58**. The buried volume (%V_buried_) represents the fraction of a sphere’s volume centered on the metal atom occupied by a given ligand. Note: The %V_buried_ values for all complexes describe the steric hindrance of two PR_3_ ligands.

To further elucidate catalytic performance and complement the electronic aspects represented by our descriptors, steric hindrance was visualized using SambVca 2.1^97^ (Figure 6). The steric maps of complexes **1** and **26**, both containing PPh_3_, illustrate how the same ligand can create distinct catalytic pockets, even in structurally analogous complexes.^98^ This observation highlights the importance of designing descriptors—such as those developed in this work—that accurately capture the steric and electronic effects of both the active metal and the catalyst’s ligands.

The steric map of complex **57** ([Ir(COD)(P(C_3_H_5_)_3_)_2_]^+^, Figure 6) reveals a catalytic pocket with reduced steric hindrance in both the southern and northern hemispheres. This feature significantly enhances H_2_ activation, particularly via oxidative addition, a property that is especially advantageous for hydrogen loading in liquid organic hydrogen carrier (LOHC) systems. The %V_buried_ values further indicate that the progressive reduction of steric hindrance in these regions correlates with increased catalytic activity. Consequently, our descriptors effectively capture both electronic and steric effects, demonstrating performance comparable to that of more advanced molecular descriptors.

#### 4.3.2 New Catalysts for Quinoline Hydrogenation: Influence of Nitrile Ligands

**Figure 7:**
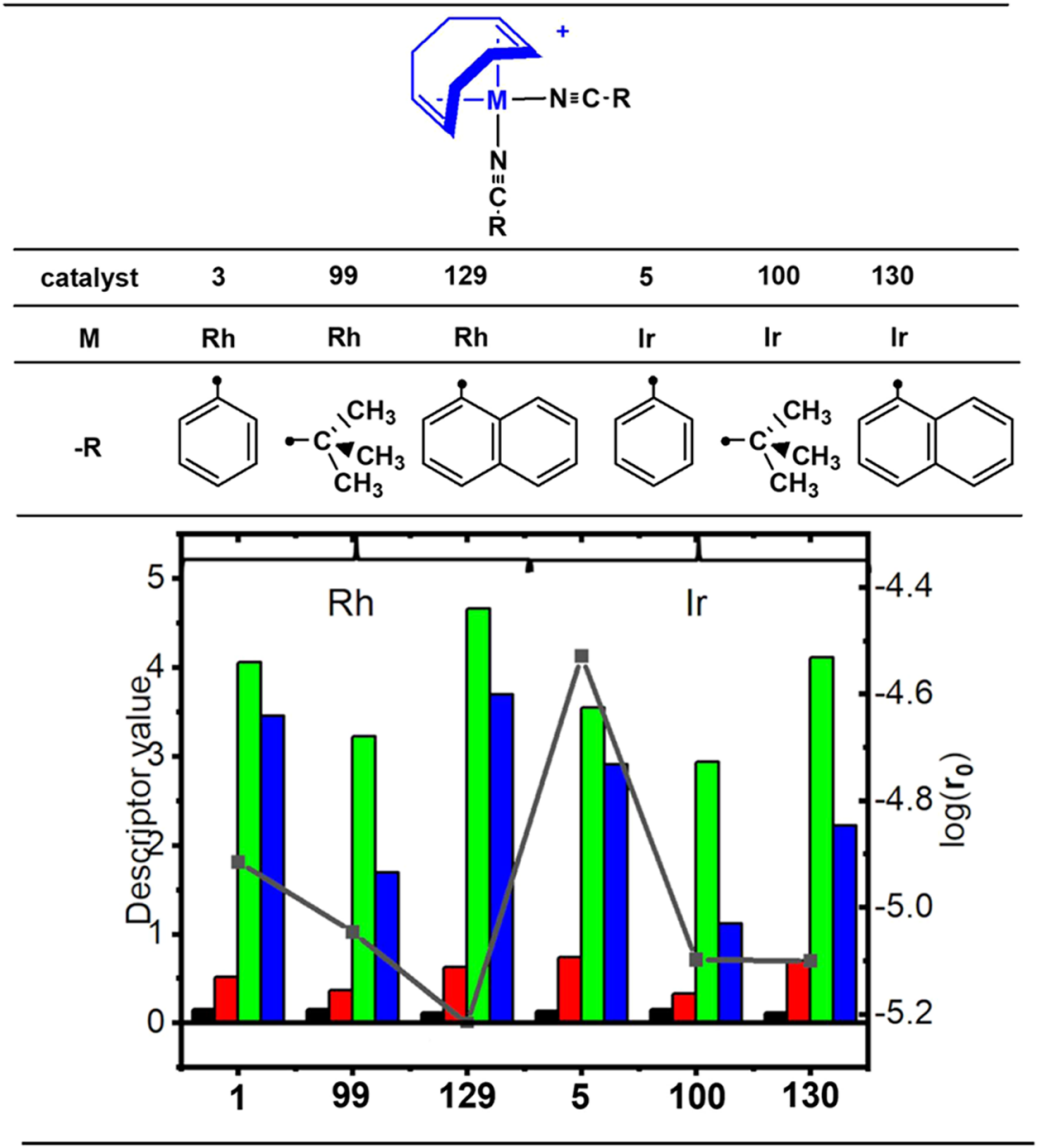
Effect of nitrile ligand modification on the initial rate activity of [M(COD)(NCR)_2_]^+^ complexes (aromatic and bulky groups). The ● symbols denote the connection points. Color scheme and plot attributes are consistent with Figure 4.

**Figure 8:**
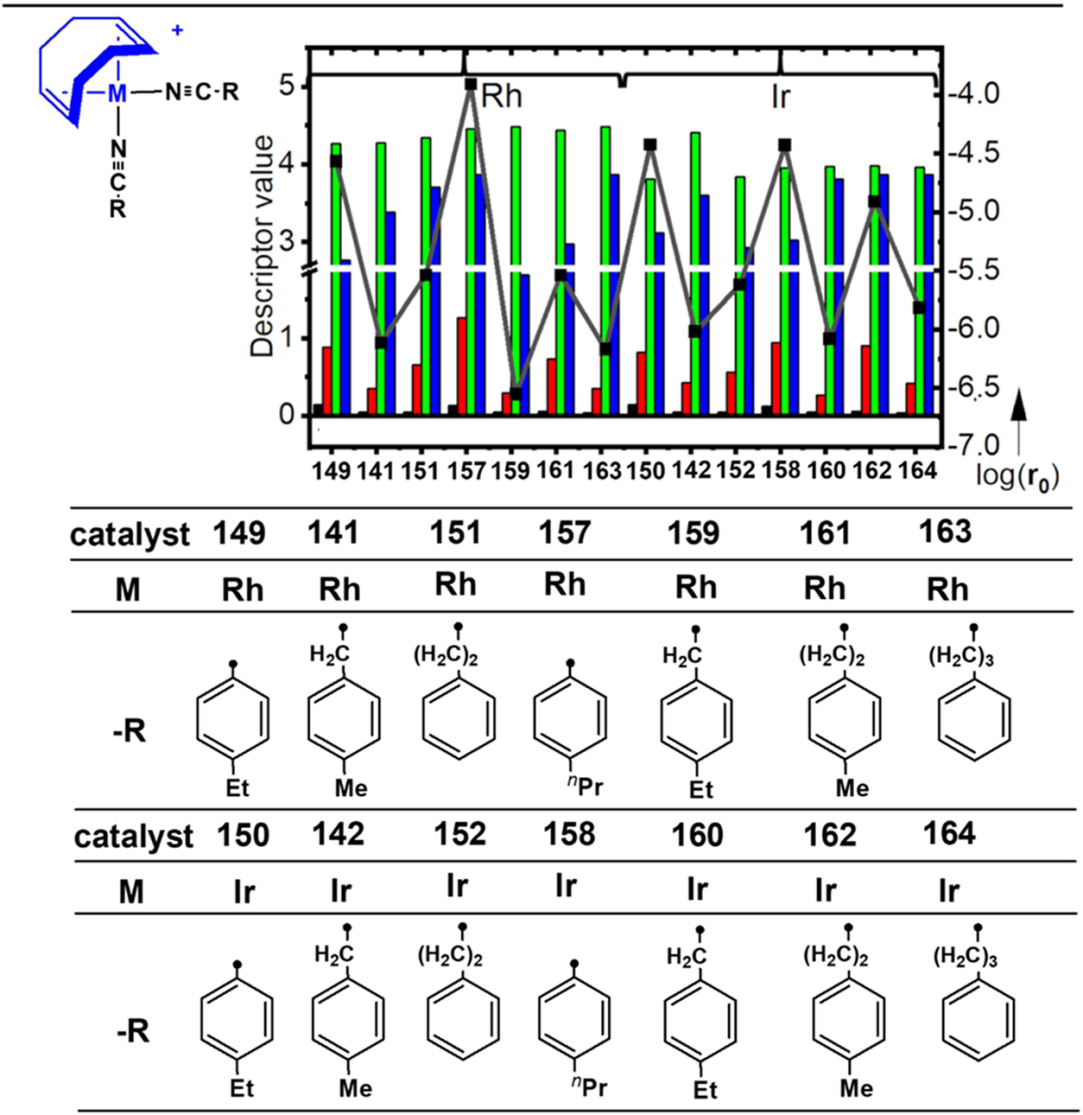
Effect of nitrile ligand modification on the initial rate activity of [M(COD)(NCR)_2_]^+^ complexes (alkyl groups). The ● symbols denote the connection points. Color scheme and plot attributes are consistent with Figure 4.

The most promising results were obtained with the [M(COD)(NCPh)_2_]^+^ complexes and their derivatives, some of which exhibited higher predicted reaction rates. Modifications to the benzonitrile ligands were investigated through three approaches: (i) Change of the nitrile ligand’s phenyl group, (ii) substitution at the *para* position of the benzonitrile phenyl group, and (iii) introduction of a (CH_2_)_*n*_ carbon chain (*n* = 1, 2, and 3) as a bridge between the phenyl and cyano groups (Figures 7 and 8).

Initially, virtual screening of analogs of complexes **3** and **5** was conducted, involving the replacement of the phenyl group in the benzonitrile ligand with other aryl or alkyl groups (Schemes 2a and 3h–4l). Replacing the phenyl group with alkyl groups in both Rh- and Ir-based catalysts resulted in decreased activity (Figure 7). Regardless of the active metal, further substitution with bulky alkyl (^t^BuCN) and naphthyl (NapCN) fragments also led to decreased activity, attributed to variations in the proportions of descriptors D2 and D3 in each catalyst (Figure 7).

Descriptor D2 is associated with the product of Mulliken electronegativity (**χ**) and softness (**s**) of atoms interacting at short distances (1–2 Å). **s** is the inverse of hardness (**h**), and **χ** is equal to the negative of the chemical potential (**μ**). These quantities define electrophilicity (**μ**^2^/2**η**), a key reactivity descriptor. Thus, D2 is related to electrophilicity, and its influence on activity is evident.

While no consensus exists regarding whether activities correlate more strongly with global or local reactivity indices, previous studies have highlighted the complexities of this relationship. For example, Altan and Yilmaz found no correlation based on the Parr-Pearson principle between global hardness, softness, and electrophilicity of phosphine-amino-alcohol tridentate ligands in a ruthenium-based catalyst reacting with benzophenones under asymmetric hydrogenation.^99^ Furthermore, Kumar-Nayak and Roy demonstrated that ligand modification can lead to changes in local descriptors, sometimes resulting in opposing changes in global descriptors.^100^ For instance, their substitution of −Cl with −SnCl_3_ in PdCl_2_X (X = COD, PPh_3_, and various N-heterocyclic carbenes) increased the electron density of palladium while decreasing the total electron density. The QuBILS-MIDAS descriptors successfully captured the local information relevant to catalytic activity and generalized it.

Optimal activities were predicted for candidates **157** ([Rh(COD)(NCPh-*p*-^n^Pr)_2_]^+^, **r**_**0**_ = 1.12 × 10^–4^ M s^–1^) and **158** ([Ir(COD)(NC-Ph-*p*-^n^Pr)_2_]^+^, **r**_**0**_ = 3.74 × 10^–5^ M s^–1^), representing approximately 4- and 2-fold increases in activity compared to complexes **3** and **5**, respectively (Figure 8).

The *para*-propyl groups on the benzonitrile ligands provided the most significant positive inductive effect (*+I*) on the aromatic phenyl ring. This effect is transmitted through the ring, slightly increasing the electron density at the nitrile group (−CN). This *+I* effect can reduce the local electronegativity of the benzonitrile ligands. Subsequently, back-donation mechanisms in complexes **157** and **158** can increase the local electronegativity of the active metal. Conversely, substitution with electron-withdrawing groups on the aromatic phenyl ring of catalysts **3** and **5** (e.g., -F and -NO_2_) substantially improves the catalytic behavior of the candidates. The Ir-based candidates **78** ([Ir(COD)(NC-Ph-*p*-F)_2_]^+^, **r**_**0**_ > 10^–3^ M s^–1^) and **118** ([Ir(COD)(NC-Ph-*p*-F)_2_]^+^, **r**_**0**_ > 10^–4^ M s^–1^) proposed in SI5 exhibited promising **r**_**0**_ values. Complex **78**, containing a fluorine atom at the para position of benzonitrile, had the highest predicted initial rate of all complexes in the training and test sets. However, most of these predicted values were outside the applicability domain of our model (Figure 3 and Section SI5; complexes **78**, **82**, and **117**–**120**).

**Figure 9:**
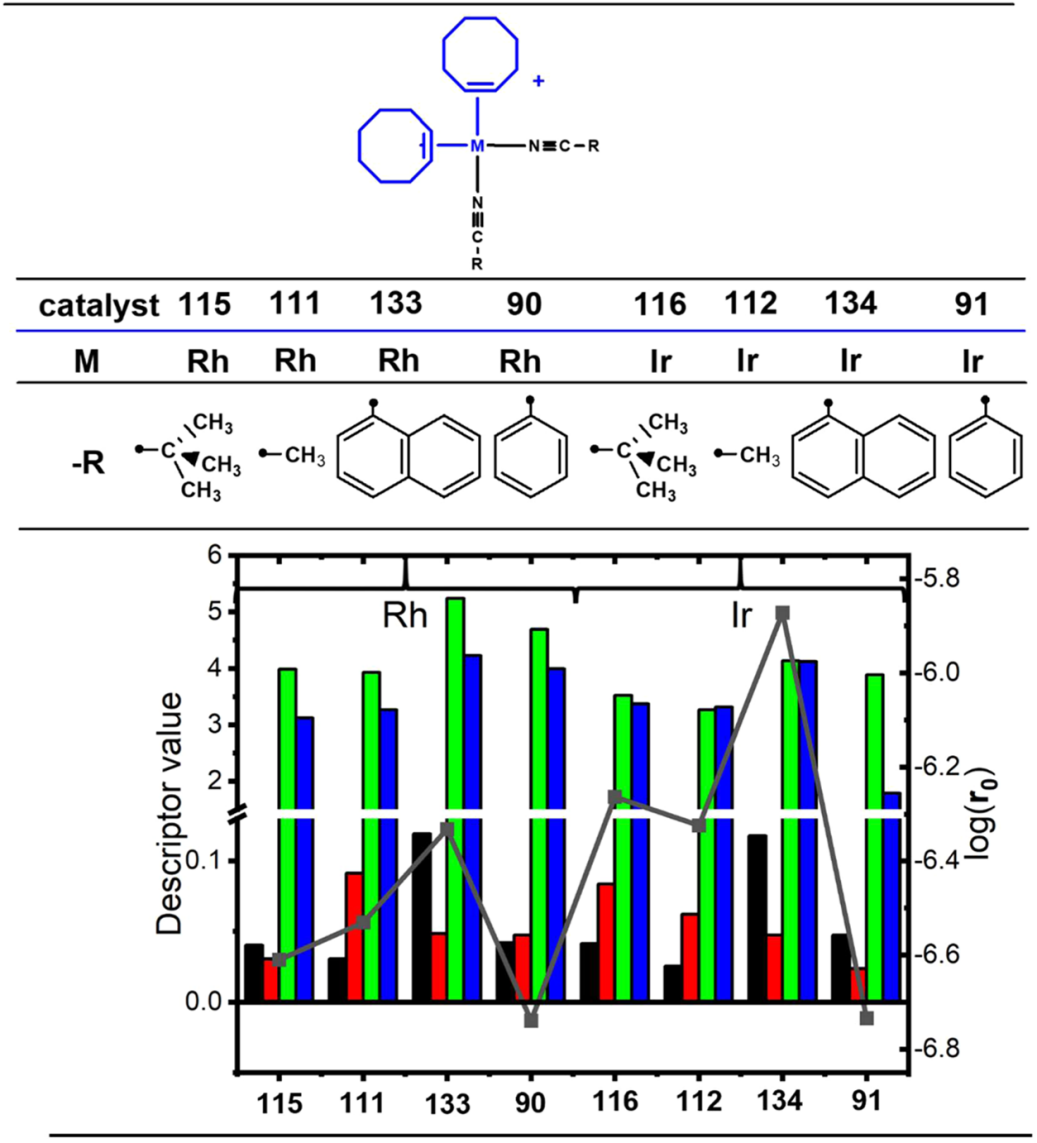
Effect of nitrile ligand modification on the initial rate activity of [M(COE)_2_(NCR)_2_]^+^ complexes. The ● symbols denote the connection points. Color scheme and plot attributes are consistent with Figure 4.

Furthermore, replacing COD with two COE ligands (as described in Schemes 2 and 3) resulted in decreased calculated **r**_**0**_ values (Figure 9; candidates **90**, **91**, **111**, **112**, **115**, **116**, **133**, and **134**). These findings underscore the challenge of optimizing the catalysts in Table 1 to achieve activity levels approaching the maximum catalytic potential for this inner-sphere mechanism. However, virtual screening using our descriptor-based model (D1–D4) suggests that strategic modifications to the nitrile ligands represent the most effective approach for enhancing the activity of these catalysts in reversible **Q** hydrogenation. Specifically, this improvement could be achieved by substituting alkyl chains and charge-attracting groups rather than making abrupt changes to the structure of COD-type ligands.

These results are also correlated with the reaction mechanism. This is evidenced by the significant effect observed when the R group of the nitrile ligand (NCR) is replaced by an electron-donating or electron-withdrawing group, or when substitution occurs at the *para* position of the phenyl group of the two benzonitrile ligands. These results suggest that at least one of these ligands remains coordinated to the metal center, favoring the catalytic process in the case of rhodium and decreasing the reaction rate in the case of iridium. In contrast, it has been reported that the initial step in the mechanism of unsaturated substrate (*e.g.*, cyclohexene) hydrogenation with coordinatively unsaturated precatalysts (such as RhCl(PPh_3_)_3_) is the ligand dissociation followed by oxidative addition of hydrogen.^38^ This scenario could also occur in the **Q** hydrogenation with complexes of the types [M(COD)(PPh_3_)_2_]^+^, [M(COD)(NCR)_2_]^+^, and [M(COE)_2_(NCR)_2_]^+^ (with M = Rh, Ir).

As previously mentioned, the hydrogenation of **Q** with [Rh(COD)(PPh_3_)_2_]^+^ proceeds through the substitution of two PPh_3_ ligands by **Q**, whereas this substitution does not occur with the iridium analog. In both cases, a dissociation of one **Q** ligand is required, a process that would be disfavored by Le Chatelier’s principle in an excess of the **Q** substrate. For the [M(COD)(NCPh)_2_]^+^ and [M(COE)_2_(NCPh)_2_]^+^ complexes, it is likely that one nitrile ligand remains coordinated to the metal center, coordinating only one **Q** molecule. Consequently, the steric and electronic effects of the nitrile ligand influence the catalytic activity of these precursors, resulting in either an increase or a decrease.

To illustrate this, consider the case of IrH(CO)(PPh_3_)_3_ (**37**, **r**_**0**_ = 5.68 × 10^–7^ M s^–1^). Replacing iridium with rhodium (**178**, r_0_ = 7.99 × 10^–6^ M s^–1^) increases the activity by nearly an order of magnitude. Notably, maintaining iridium and introducing a fluorine (**38**, **r**_**0**_ = 5.15 × 10^–6^ M s^–1^) or a methyl group (**39**, **r**_**0**_ = 2.19 × 10^–6^ M s^–1^) at the *para* position of the phenyl ring in PPh_3_ achieves a similar enhancement in activity. In terms of descriptors, both metal exchange and methyl substitution lead to increases in D1 and D4, accompanied by a decrease in D3, which relates to mass and hardness at long-range interactions. However, fluorine substitution also significantly increases D2 (see the comma-separated values file in the Supporting Information). As observed with [M(COD)(PR_3_)_2_]^+^ complexes, the local hardness of rhodium promotes the dissociation of one or even two phosphine ligands. In contrast, only one phosphine dissociates with iridium.

Analysis of the chemical space guided the *in silico* design of candidates. The descriptor space in Figure 3 indicates that rhodium and iridium candidates of the types MH(CO)(PPh_3_)_3_, Rh(acac)(CO)(L) and [M(CO)_2_(NCC_6_H_4_-*p*-X)_2_]^+^ exhibited higher **r**_**0**_, and their activity could be further enhanced by slight variations in the steric and electronic properties of their ligands. Specifically, for the Rh(acac)(CO)(L) complexes (L = CO (**55**), PPh_3_ (**59**), P^t^Bu(CH_2_–CH = CH_2_)_2_ (**24**) and P^t^Bu(CH_2_–CH = CH_2_)_2_ (**177**)), the complex without a phosphine ligand (**55**, **r**_**0**_ = 1.81 × 10^–5^ M s^–1^) showed higher predicted activity than the complex with triphenylphosphine (**59**, **r**_**0**_ = 1.09 × 10^–6^ M s^–1^) and those with functionalized phosphines. Among the latter, the complex with diallylphosphine (**24**, **r**_**0**_ = 1.41 × 10^–5^ M s^–1^) exhibited higher activity than the complex with dicyanomethylphosphine (**177**, **r**_**0**_ = 4.75 × 10^–6^ M s^–1^). This difference is likely attributable to the coordination of the allyl group to the metal center (a *π-*acid and therefore soft fragment) during the catalytic process, as proposed by Rosales *et al*.^58^

**Figure 10:**
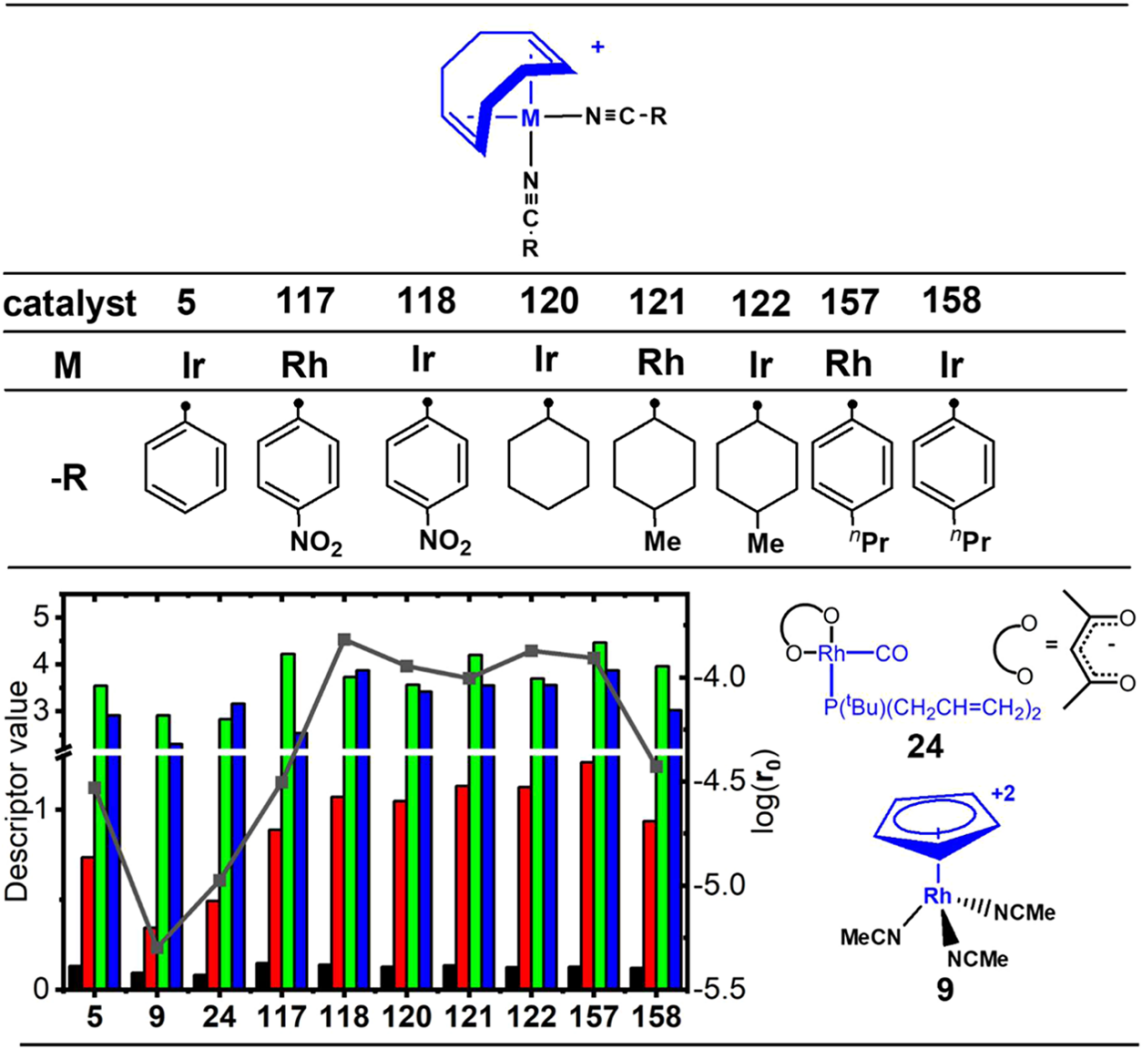
Relationships between catalytic activities and D_n_ values for the most prominent catalytic systems in this work. The ● symbols denote the connection points. Color scheme and plot attributes are consistent with Figure 4.

**Figure 11:**
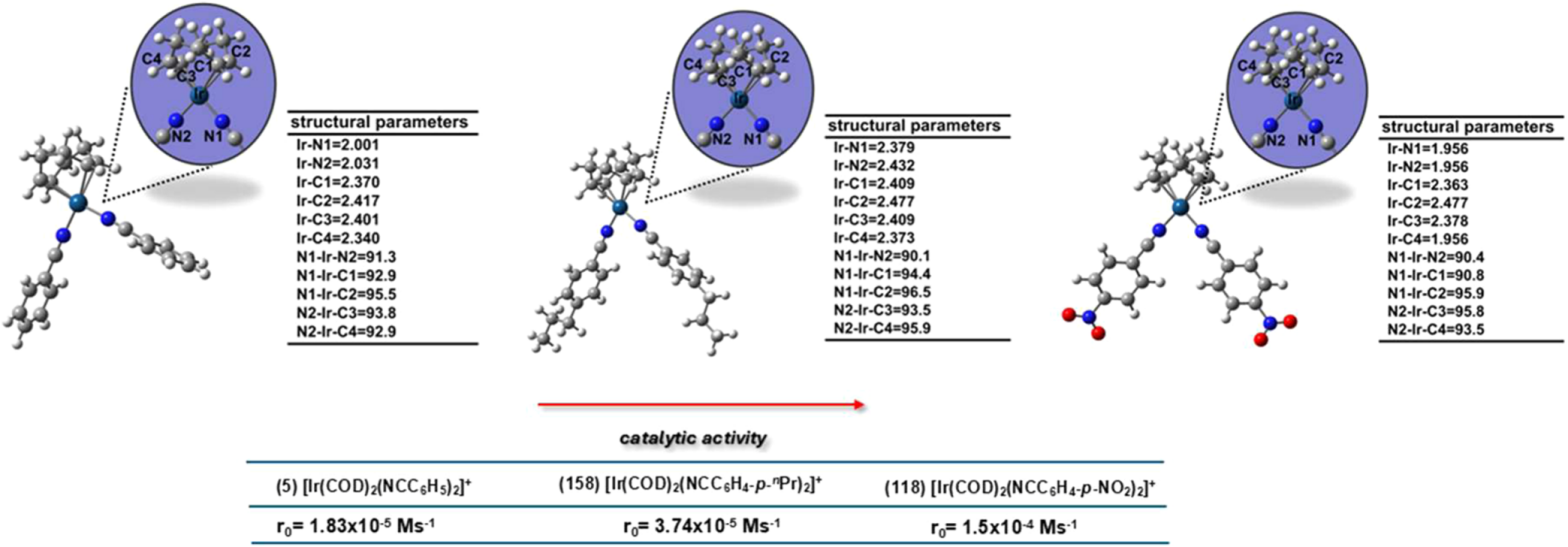
Optimized structures of selected catalysts with prominent predicted activities: from left to right, complexes 5, 158, and 118. Angles (α, °) and bond distances (d, Å).

Finally, the activity of the [M(COD)_2_(NCC_6_H_4_-p-X)_2_]^+^ complexes, such as **117** (M = Rh, X = NO_2_), **118** (M = Ir, X = NO_2_), **157** (M = Rh, X = propyl), and **158** (M = Ir, X = propyl), as well as [M(COD)_2_(NCC_6_H_11_-p-X)_2_]^+^ complexes, such as **120** (M = Ir, X = H), **121** (M = Rh, X = CH_3_), **122** (M = Ir, X = CH_3_), which are located in the zone of precatalysts **5**, **9**, and **24** (Zone II in Figure 3), was analyzed. Iridium complexes were the most active catalysts, particularly complex **118** (Figures 10 and 11). As explained above, the presence of π-acid ligands such as COD (a soft ligand) and nitrile ligands play an important role in the catalytic activity of these complexes.

The activity within this group is mainly governed by descriptor D2 (Figure 9), highlighting the importance of changes in electrophilicity for enhancing the activity of these complexes. For a deeper understanding of the structural details of the top candidates, see Figure 11. The results of this work, particularly those involving the iridium complexes, are important for their potential use in the storage, transportation, and release of green hydrogen (LOHCs), as these metal complexes have demonstrated activity for the reversible hydrogenation/dehydrogenation of quinoline and tetrahydroquinoline.^95^

A final consideration in the *in silico* screening of new catalysts is their synthetic feasibility. The use of earth-abundant metals such as manganese (Mn)^35^ and cobalt (Co),^36^ with abundances in Earth’s crust of approximately 950 and 25 ppm, respectively, would be economically advantageous. However, the active metal iridium in our top-performing catalyst candidates is significantly more abundant (approximately 0.001 ppm) than rhodium, one of the rarest metals with an abundance in Earth’s crust of approximately 0.0002 ppm. Moreover, iridium is available at a comparatively lower market price, further enhancing its feasibility as a catalytic material. It is important to note, however, that only 21 elements on Earth have crustal abundances exceeding 100 ppm (even manganese is notably absent from this list). Given the finite nature of these resources, catalyst recycling and reuse must be integral to the design of sustainable catalytic systems.^95,96^ In this context, our homogeneous catalysts could provide a conceptual basis for developing biphasic catalysis technologies, enabling improved catalyst recovery and reuse. All ligands employed in the top catalytic candidates exhibit stability under standard conditions: CAS 111-78-4 (COD), CAS 100-47-0 (benzonitrile, C_6_H_5_CN), CAS 619-72-7(4-nitrobenzonitrile, NO_2_C_5_H_4_CN), CAS 101-02-0 (P(OPh)_3_), CAS 603-35-0 (P(Ph)_3_), and CAS 13118–24–6 (P(C_3_H_5_)_3_), *etc*. Finally, among the key ligands, only P(Cp)_3_ presents synthetic challenges within established phosphorus chemistry methods. Furthermore, new biphasic catalysts could be designed by modifying the proposed phosphines into ionophilic^101^ or other water-soluble sulfonated derivatives.^102^

## 5 Conclusions

Geometric modeling of the metallic complexes used in this work yielded satisfactory results, as verified by comparison of experimental bond distances and angles with literature values. Most calculated values fell within the reported ranges, with only minimal differences observed. The QuBiLS-MIDAS descriptors effectively encoded molecular structural information by considering interactions among two, three, and four atoms. The best model exhibited good predictive ability for the training set (*R*^2^ = 0.90) and satisfactory external validation for the testing set (Q_EXT_^2^ = 0.86). These descriptors revealed the importance of electronic properties, expressed by the reactivity descriptors hardness, softness, electrophilicity, and mass. The model indicates that the coupling of Mulliken electronegativity with the hardness of atoms interacting at distances between 1–2 Å significantly increased activity, highlighting the effect of electrophilicity.

The descriptor space defined three distinct regions in the activity contour map. The first region comprises the least active catalysts, among which the ruthenium complexes RuHCl(PPh_3_)_3_ and [Ru(NCMe)_3_(triphos)]_2_^+^ exhibit notable activity. While ruthenium and osmium catalysts did not show substantial differences in predicted activities, we are currently investigating the use of such compounds for the partial or total hydrogenation of Quinoline (**Q**) using these complexes as homogeneous and dual (homogeneous/nanoparticle) precatalysts.

The second region corresponds to complexes with the highest initial rates, including rhodium and iridium complexes with π-acidic ligands (primarily olefins, diolefins, and η^5^-Cp) acting as soft bases, and nitrile ligands. Substituent groups on the ligands of these complexes generate appreciable changes in the predicted reaction rates, making them promising candidates for developing improved precatalysts for the hydrogenation of **Q** to **THQ**. This relationship is linked to the reaction mechanism and the formation of the active species, which depends on the specific ligand that dissociates in the reaction medium. Finally, the third region comprises rhodium complexes with bi- and tridentate phosphine ligands, such as [Rh(dppe)_2_] and [Rh(Q)_2_(triphos)], which exhibit moderate catalytic activities.

The QuBiLS-MIDAS approach effectively predicted catalytic activity for quinoline hydrogenation, but its generalized nature limits interpretability. A promising avenue for future research is to explore the local contributions of molecular fragments to the overall descriptor values. Analysis of local invariants will enable interpretation of the effects of structural changes on atoms, ligands, or functional groups by exploiting the mathematical definition of QuBiLS-MIDAS. These results suggest that the QuBiLS-MIDAS approach is a valuable tool for the *in silico* design of novel and efficient catalysts for quinoline hydrogenation and potentially in other catalytic transformations.

## Supporting Information Available

Supporting Information SI1: theoretical background, QuBiLS-MIDAS approach, and internal sphere mechanisms of catalytic homogeneous quinoline hydrogenation with Ru, Rh, Os, and Ir catalysts. Supporting Information SI2: optimized structures for the validation of the computational methodology. Supporting Information SI3: optimized structures for catalytic precursors. Supporting Information SI4: multiple linear regression results. Supporting Information SI5: proposed catalysts from the multiple linear regression model. Information of additional files: comma-separated values of descriptors and predicted values of initial rate and molecular data set in structured data format file (PDF)

## References

[ref1] Elkhatat A.; Al-Muhtaseb S. Climate Change and Energy Security: A Comparative Analysis of the Role of Energy Policies in Advancing Environmental Sustainability. Energies 2024, 17, 3179 10.3390/en17133179.

[ref2] Hassan Q.; Viktor P.; J Al-Musawi T.; Mahmood Ali B.; Algburi S.; Alzoubi H. M.; et al. The renewable energy role in the global energy Transformations. Renewable Energy Focus 2024, 48, 100545 10.1016/J.REF.2024.100545.

[ref3] Verevkin S. P.; Safronov S. P.; Samarov A. A.; Vostrikov S. V. Hydrogen storage: Thermodynamic analysis of alkyl-quinolines and alkyl-pyridines as potential liquid organic hydrogen carriers (LOHC). Appl. Sci. 2021, 11, 11758 10.3390/app112411758.

[ref4] Wei Z.; Shao F.; Wang J. Recent advances in heterogeneous catalytic hydrogenation and dehydrogenation of N-heterocycles. Chin. J. Catal. 2019, 40, 980–1002. 10.1016/S1872-2067(19)63336-X.

[ref5] Xie D.; Liu X.; Lv H.; Guo Y. Products, pathways, and kinetics for catalytic hydrodenitrogenation of quinoline in hydrothermal condition. J. Supercrit. Fluids 2022, 182, 105509 10.1016/j.supflu.2021.105509.

[ref6] Forberg D.; Schwob T.; Zaheer M.; Friedrich M.; Miyajima N.; Kempe R. Single-catalyst high-weight% hydrogen storage in an N-heterocycle synthesized from lignin hydrogenolysis products and ammonia. Nat. Commun. 2016, 7, 13201 10.1038/ncomms13201. 27762267 PMC5080437

[ref7] Venuti Björkman J.; Hruby S. L.; Pettersson L. J.; Kantarelis E. Investigating the Effects of Organonitrogen Types on Hydrodearomatization Reactions over Commercial NiMoS Catalyst. Catalysts 2022, 12, 736 10.3390/catal12070736.

[ref8] Guo Y.; He H.; Liu X.; Chen Z.; Rioux R. M.; Janik M. J.; Savage P. E. Ring-opening and hydrodenitrogenation of indole under hydrothermal conditions over Ni, Pt, Ru, and Ni-Ru bimetallic catalysts. Chem. Eng. J. 2021, 406, 126853 10.1016/j.cej.2020.126853.

[ref9] Izquierdo R.; Cubillan N.; Guerra M.; Rosales M. Substituted heterocycles as new candidates for liquid organic hydrogen carriers: In silico design from DFT calculations. Int. J. Hydrogen Energy 2021, 46, 17853–17870. 10.1016/j.ijhydene.2021.02.201.

[ref10] Bermudez Aponte N. A.; Meille V. Use of Biosourced Molecules as Liquid Organic Hydrogen Carriers (LOHC) and for Circular Storage. Reactions 2024, 5, 195–212. 10.3390/reactions5010008.

[ref11] Vostrikov S. V.; Konnova M. E.; Turovtsev V. V.; Müller K.; Bara J. E.; Verevkin S. P. Thermodynamics of Hydrogen Storage: Equilibrium Study of Liquid Organic Hydrogen Carrier System 1-Methylindole/octahydro-1-methylindole. AppliedChem 2023, 3, 45–62. 10.3390/appliedchem3010004.

[ref12] Huang W.; Huang M.; Ma W.; Yang H.; Li R.; Wang J.; et al. Rational design of titanium-doped Y zeolite for hydrodenitrogenation of aromatic N-heterocyclic compounds. Chem. Eng. J. 2024, 498, 155221 10.1016/j.cej.2024.155221.

[ref13] Zhang J.; Yang F.; Wang B.; Li D.; Wei M.; Fang T.; Zhang Z. Heterogeneous Catalysts in N-Heterocycles and Aromatics as Liquid Organic Hydrogen Carriers (LOHCs): History, Present Status and Future. Materials 2023, 16, 3735 10.3390/ma16103735. 37241361 PMC10220885

[ref14] Uijthof E. M. T.; Chavan B. S.; Sluijer M. J.; Komath V. C.; van der Ham A. G. J.; van den Berg H.; et al. Liquid organic hydrogen carriers: Process design and economic analysis for manufacturing N-ethylcarbazole. J. Adv. Manuf. Process 2024, 6, e10173 10.1002/amp2.10173.

[ref15] Zheng P.; Xiao C.; Song S.; Duan A.; Xu C. DFT insights into the hydrodenitrogenation mechanism of quinoline catalyzed by different Ni-promoted MoS2 edge sites: Effect of the active phase morphology. J. Hazard Mater. 2021, 411, 125127 10.1016/j.jhazmat.2021.125127. 33485219

[ref16] Naveen K.; Mahvelati-Shamsabadi T.; Sharma P.; Lee S.; Hur S. H.; Choi W. M.; et al. MOF-derived Co/Zn single-atom catalysts for reversible hydrogenation and dehydrogenation of quinoline hydrogen carrier. Appl. Catal., B 2023, 328, 122482 10.1016/J.APCATB.2023.122482.

[ref17] Gil A.; Sancho-Sanz I.; Korili S. A. Progress and Perspectives in the Catalytic Hydrotreatment of Bio-Oils: Effect of the Nature of the Metal Catalyst. Ind. Eng. Chem. Res. 2024, 63, 11759–11175. 10.1021/acs.iecr.4c00747.

[ref18] Li Y.; Guo X.; Zhang S.; He Y. A Perspective Review on N-Heterocycles as Liquid Organic Hydrogen Carriers and Their Hydrogenation/Dehydrogenation Catalysts. Energy Fuels 2024, 38, 12447–12471. 10.1021/acs.energyfuels.4c01633.

[ref19] Zhou M. J.; Miao Y.; Gu Y.; Xie Y. Recent Advances in Reversible Liquid Organic Hydrogen Carrier Systems: From Hydrogen Carriers to Catalysts. Adv. Mater. 2024, 36, 2311355 10.1002/adma.202311355. 38374727

[ref20] Cho J. Y.; Kim H.; Oh J. E.; Park B. Y. Recent Advances in Homogeneous/Heterogeneous Catalytic Hydrogenation and Dehydrogenation for Potential Liquid Organic Hydrogen Carrier (LOHC) Systems. Catalysts 2021, 11, 1497 10.3390/catal11121497.

[ref21] Zhang Y. Q.; Markiewicz M.; Filser J.; Stolte S. Toxicity of a Quinaldine-Based Liquid Organic Hydrogen Carrier (LOHC) System toward Soil Organisms Arthrobacter globiformis and Folsomia candida. Environ. Sci. Technol. 2018, 52, 258–265. 10.1021/acs.est.7b04434. 29206024

[ref22] Maji B.; Bhandari A.; Bhattacharya D.; Choudhury J. Reusable Single Homogeneous Ir(III)-NHC Catalysts for Bidirectional Hydrogenation-Dehydrogenation of N-Heteroarenes in Water. Organometallics 2022, 41, 1609–1620. 10.1021/acs.organomet.2c00107.

[ref23] Chu C.; Wu K.; Luo B.; Cao Q.; Zhang H. Hydrogen storage by liquid organic hydrogen carriers: Catalyst, renewable carrier, and technology - A review. Carbon Resour. Convers. 2023, 6, 334 10.1016/j.crcon.2023.03.007.

[ref24] Mao T.; Liu Z.; Zhang X.; Feng H.; Huang Y.; Xu Y.; et al. Interaction evolution and N product distribution during biomass co-pyrolysis for endogenous N-doping bio-carbon. J. Energy Inst. 2025, 118, 101902 10.1016/j.joei.2024.101902.

[ref25] Tan K. C.; Chua Y. S.; He T.; Chen P. Strategies of thermodynamic alternation on organic hydrogen carriers for hydrogen storage application: A review. Green Energy Resour. 2023, 1, 100020 10.1016/j.gerr.2023.100020.

[ref26] Borowski A. F.; Sabo-Etienne S.; Donnadieu B.; Chaudret B. Reactivity of the bis(dihydrogen) complex [RuH2(η2-H2)2 (PCy3)2] toward N-heteroaromatic compounds. Regioselective hydrogenation of acridine to 1,2,3,4,5,6,7,8-octahydroacridine. Organometallics 2003, 22, 1630–1637. 10.1021/om020995p.

[ref27] Dyson P. J. Arene hydrogenation by homogeneous catalysts: fact or fiction?. Dalton Trans. 2003, 2964–2674. 10.1039/b303250g.

[ref28] Frediani P.; Pistolesi V.; Frediani M.; Rosi L. Catalytic activity of dihydride ruthenium complexes in the hydrogenation of nitrogen containing heterocycles. Inorg. Chim. Acta 2006, 359, 917–925. 10.1016/j.ica.2005.06.025.

[ref29] Chatterjee B.; Kalsi D.; Kaithal A.; Bordet A.; Leitner W.; Gunanathan C. One-pot dual catalysis for the hydrogenation of heteroarenes and arenes. Catal. Sci. Technol. 2020, 10, 5163–5170. 10.1039/D0CY00928H.

[ref30] Gao B.; Han Z.; Meng W.; Feng X.; Du H. Asymmetric Reduction of Quinolines: A Competition between Enantioselective Transfer Hydrogenation and Racemic Borane Catalysis. J. Org. Chem. 2023, 88, 3335–3339. 10.1021/acs.joc.2c02905. 36799068

[ref31] Makaryan I. A.; Sedov I. V. Hydrogenation/Dehydrogenation Catalysts for Hydrogen Storage Systems Based on Liquid Organic Carriers (A Review). Pet Chem. 2021, 61, 977–988. 10.1134/S0965544121090085.

[ref32] Zuorro A.; García-Martínez J. B.; Barajas-Solano A. F. The Application of Catalytic Processes on the Production of Algae-Based Biofuels: A Review. Catalysts 2021, 11, 22 10.3390/catal11010022.

[ref33] Zhou Y.; Hu C. Catalytic Thermochemical Conversion of Algae and Upgrading of Algal Oil for the Production of High-Grade Liquid Fuel: A Review. Catalysts 2020, 10, 145 10.3390/catal10020145.

[ref34] Jayaraj A.; Raveedran A. V.; Latha A. T.; Priyadarshini D.; Swamy P. C. A. Coordination Versatility of NHC-metal Topologies in Asymmetric Catalysis: Synthetic Insights and Recent Trends. Coord. Chem. Rev. 2023, 478, 214922 10.1016/j.ccr.2022.214922.

[ref35] Papa V.; Cao Y.; Spannenberg A.; Junge K.; Beller M. Development of a practical non-noble metal catalyst for hydrogenation of N-heteroarenes. Nat. Catal. 2020, 3, 135–142. 10.1038/s41929-019-0404-6.

[ref36] Xu R.; Chakraborty S.; Yuan H.; Jones W. D. Acceptorless, Reversible Dehydrogenation and Hydrogenation of N-Heterocycles with a Cobalt Pincer Catalyst. ACS Catal. 2015, 5, 6350–6354. 10.1021/acscatal.5b02002.

[ref37] El-Shahat M. Advances in the reduction of quinolines to 1,2,3,4-tetrahydroquinolines. J. Heterocycl. Chem. 2022, 59, 399–421. 10.1002/jhet.4394.

[ref38] Sánchez-Delgado R. A.; Rosales M. Kinetic studies as a tool for the elucidation of the mechanisms of metal complex-catalyzed homogeneous hydrogenation reactions. Coord. Chem. Rev. 2000, 196, 249–280. 10.1016/S0010-8545(99)00168-X.

[ref39] Van Der Linden J. B.; Ras E. J.; Hooijschuur S. M.; Klaus G. M.; Luchters N. T.; Dani P.; et al. Asymmetric Catalytic Ketone Hydrogenation: Relating Substrate Structure and Product Enantiomeric Excess Using QSPR. QSAR Comb. Sci. 2005, 24, 94–98. 10.1002/qsar.200420060.

[ref40] Aguado-Ullate S.; Guasch L.; Urbano-Cuadrado M.; Bo C.; Carbó J. J. 3D-QSPR models for predicting the enantioselectivity and the activity for asymmetric hydroformylation of styrene catalyzed by Rh–diphosphane. Catal. Sci. Technol. 2012, 2, 1694–1704. 10.1039/c2cy20089a.

[ref41] Meraz M. M.; Malik A. A.; Yang W.; Sun W. H. Catalytic performance of cycloalkyl-fused aryliminopyridyl nickel complexes toward ethylene polymerization by qspr modeling. Catalysts 2021, 11, 920 10.3390/catal11080920.

[ref42] Sciabola S.; Alex A.; Higginson P. D.; Mitchell J. C.; Snowden M. J.; Morao I. Theoretical prediction of the enantiomeric excess in asymmetric catalysis. An alignment-independent molecular interaction field based approach. J. Org. Chem. 2005, 70, 9025–9027. 10.1021/jo051496b. 16238344

[ref43] Corma A.; Serra J. M.; Serna P.; Moliner M. Integrating high-throughput characterization into combinatorial heterogeneous catalysis: unsupervised construction of quantitative structure/property relationship models. J. Catal. 2005, 232, 335–341. 10.1016/j.jcat.2005.03.019.

[ref44] Nkulikiyinka P.; Wagland S. T.; Manovic V.; Clough P. T. Prediction of Combined Sorbent and Catalyst Materials for SE-SMR, Using QSPR and Multitask Learning. Ind. Eng. Chem. Res. 2022, 61, 9218–9233. 10.1021/acs.iecr.2c00971. 35818477 PMC9264356

[ref45] Garcia-Jacas C.; Marrero-Ponce Y.; Barigye S.; Valdes-Martini J.; Rivera-Borroto O.; Olivero-Verbel J. N-linear algebraic maps for chemical structure codification: a suitable generalization for atom-pair approaches?. Curr. Drug Metab. 2014, 15, 441–469. 10.2174/1389200215666140605124506. 24909423

[ref46] Cabrera N.; Mora J. R.; Márquez E.; Flores-Morales V.; Calle L.; Cortés E. QSAR and molecular docking modelling of anti-leishmanial activities of organic selenium and tellurium compounds. 2020; Vol. 32, pp 29–50 10.1080/1062936X.2020.1848914. 33241943

[ref47] Ponce Y. M. Total and Local Quadratic Indices of the Molecular Pseudograph’s Atom Adjacency Matrix: Applications to the Prediction of Physical Properties of Organic Compounds. Molecules 2003, 8, 687–726. 10.3390/80900687.

[ref48] Cubillán N.; Marrero-Ponce Y.; Ariza-Rico H.; Barigye S. J.; García-Jacas C. R.; Valdes-Martini J. R.; Alvarado Y. J. Novel global and local 3D atom-based linear descriptors of the Minkowski distance matrix: theory, diversity–variability analysis and QSPR applications. J. Math. Chem. 2015, 53, 2028–2064. 10.1007/s10910-015-0533-3.

[ref49] Marrero-Ponce Y.; García-Jacas C.; Barigye S.; Valdés-Martiní J.; Rivera-Borroto O.; Pino-Urias R.; et al. Optimum Search Strategies or Novel 3D Molecular Descriptors: is there a Stalemate?. Curr. Bioinf. 2015, 10, 533–564. 10.2174/1574893610666151008011457.

[ref50] Marrero-Ponce Y.; Santiago O. M.; López Y. M.; Barigye S. J.; Torrens F. Derivatives in discrete mathematics: a novel graph-theoretical invariant for generating new 2/3D molecular descriptors. I. Theory and QSPR application. J. Comput. Aided Mol. Des. 2012, 26, 1229–1246. 10.1007/s10822-012-9591-9. 23124489

[ref51] García-Jacas C. R.; Marrero-Ponce Y.; Barigye S. J.; Hernández-Ortega T.; Cabrera-Leyva L.; Fernández-Castillo A. N-tuple topological/geometric cutoffs for 3D N-linear algebraic molecular codifications: variability, linear independence and QSAR analysis. 2016; Vol. 27, pp 949–975 10.1080/1062936X.2016.1231714. 27707004

[ref52] Voutchkova A. M.; Gnanamgari D.; Jakobsche C. E.; Butler C.; Miller S. J.; Parr J.; Crabtree R. H. Selective partial reduction of quinolines: Hydrosilylation vs. transfer hydrogenation. J. Organomet. Chem. 2008, 693, 1815–1821. 10.1016/j.jorganchem.2008.02.004.

[ref53] Sánchez-Delgado R. A.; Rondón D.; Andriollo A.; Herrera V.; Martín G.; Chaudret B. Kinetics and Mechanism of the Regioselective Homogeneous Hydrogenation of Quinoline Using [Rh(COD)(PPh3)2]PF6 as the Catalyst Precursor. Organometallics 1993, 12, 4291–4296. 10.1021/om00035a013.

[ref54] Rosales M.; Alvarado Y.; Boves M.; Rubio R.; Soscún H.; Sánchez-Delgado R. A. Kinetics and mechanisms of homogeneous catalytic reactions. Part 3. Regioselective, catalysed [RuH(CO)(NCMe)2(PPh3)2]BF4 reduction of quinoline. Transition Met. Chem. 1995, 20, 246–251. 10.1007/BF00143486.

[ref55] Rosales M.; González A.; Navarro J.; Soscún H.; Zárraga J. Synthesis and catalytic properties of the complex [OsH(CO)(NCMe)2(PPh3)2]BF4. Inorg. Chim. Acta 1997, 257, 131–135. 10.1016/S0020-1693(96)05434-5.

[ref56] Rosales M.; Vallejo R.; Soto J. J.; Bastidas L. J.; Molina K.; Baricelli P. J. Kinetics and mechanisms of homogeneous catalytic reactions. Part 10. regioselective hydrogenation of quinoline catalyzed by the systems M 2Cl2(COE)4/2 Triphos [M = Rh, Ir; COE = cyclooctene; Triphos = 1,1,1-tris(diphenylphosphinomethyl)ethane]. Catal. Lett. 2010, 134, 56–62. 10.1007/s10562-009-0225-3.

[ref57] Rosales M.; Bastidas L. J.; González B.; Vallejo R.; Baricelli P. J.; Molina K.; et al. Kinetics and mechanisms of homogeneous catalytic reactions. Part 11. Regioselective hydrogenation of quinoline catalyzed by rhodium systems containing 1,2-bis(diphenylphosphino)ethane. Catal. Lett. 2011, 141, 1305–1310. 10.1007/s10562-011-0641-z.

[ref58] Rosales M.; Molina K.; Vallejo R.; Ocando-Mavárez E. Kinetics and mechanisms of homogeneous catalytic reactions: Part 13. Regioselective reduction of quinoline catalysed by Rh(acac)(CO)[P(tBu)(CH2CH = CH2)2]. Transition Met. Chem. 2016, 41, 467–473. 10.1007/s11243-016-0042-7.

[ref59] Rosales M.; Molina K.; Arrieta F.; Fernández D.; Baricelli P. J. Kinetics and mechanisms of homogeneous catalytic reactions. Part 16. Regioselective hydrogenation of quinoline catalyzed by dichlorotris(triphenylphosphine)ruthenium(II). Mol. Catal. 2020, 490, 110970 10.1016/j.mcat.2020.110970.

[ref60] Fish R. H.; Thormodsen A. D.; Cremer G. A.; et al. Homogeneous Catalytic Hydrogenation. 1. Regiospecific Reductions of Polynuclear Aromatic and Polynuclear Heteroaromatic Nitrogen Compounds Catalyzed by Transition-Metal Carbonyl Hydrides. J. Am. Chem. Soc. 1982, 104, 5234–5237. 10.1021/ja00383a044.

[ref61] Fish R. H.; Tan J. L.; Thormodsen A. D. Homogeneous Catalytic Hydrogenation. 2. Selective Reductions of Polynuclear Heteroaromatic Compounds Catalyzed by Chlorotris(triphenylphosphine)rhodium(I). J. Org. Chem. 1984, 49, 4500–4505. 10.1021/jo00197a035.

[ref62] Baralt E.; Smith S. J.; Hurwitz J.; Horváth I. T.; Fish R. H. Homogeneous Catalytic Hydrogenation. 6. Synthetic and Mechanistic Aspects of the Regioselective Reductions of Model Coal Nitrogen, Sulfur, and Oxygen Heteroaromatic Compounds Using the (η.5-Pentamethylcyclopentadienyl)rhodium Tris(acetonitrile) Dication Complex as the Catalyst Precursor. J. Am. Chem. Soc. 1992, 114, 5187–5196. 10.1021/ja00039a033.

[ref63] Chin C. S.; Park Y.; Lee B. Regioselective catalytic hydrogenation of nitrogen rings of fused heteroaromatic compounds with an iridium-triphenylphosphine complex. Catal. Lett. 1995, 31, 239–243. 10.1007/BF00808836.

[ref64] Rosales M.; González Á.; Mora M.; Nader N.; Navarro J.; Sánchez L.; Soscún H. Kinetics and mechanisms of homogeneous catalytic reactions. Part 4. Hydrogenation of cyclohexanone and 2-cyclohexen-1-one catalysed by the complexes [MH(CO)(NCMe) 2 (PPh 3) 2 ]BF 4 (M = Ru, Os). Transition Met. Chem. 2004, 29, 205–211. 10.1023/B:TMCH.0000019423.68981.fa.

[ref65] Rosales M.; Alvarado B.; Arrieta F.; De La Cruz C.; González Á.; Molina K.; et al. A general route for the synthesis of hydrido-carboxylate complexes of the type MH(CO)(κ3-OCOR)(PPh3)2 [M = Ru, Os; R = CH3, CH2Cl, C6H5, CH(CH3)2] and their use as precatalysts for hydrogenation and hydroformylation reactions. Polyhedron 2008, 27, 530–536. 10.1016/j.poly.2007.10.007.

[ref66] Rosales M.; Arteaga M.A.; González A.; González B.; Molina K.; Perez H.; Vallejo R. A detailed kinetic and mechanistic study of the regioselective hydrogenation of quinoline to 1, 2, 3, 4 tetrahydroquinoline catalyzed by [Os(H) (Cl) (CO) (PPh3)3]. Ciencia 2006, 14, 70–82.

[ref67] Casado J.; López-Quintela M. A.; Lorenzo-Barral F. M. The initial rate method in chemical kinetics: Evaluation and experimental illustration. J. Chem. Educ. 1986, 63, 450–452. 10.1021/ed063p450.

[ref68] Toropov A. A.; Toropova A. P. QSPR/QSAR: State-of-Art, Weirdness, the Future. Molecules 2020, 25, 1292 10.3390/molecules25061292. 32178379 PMC7143984

[ref69] MOPAC Home Page. Stewart Computational Chemistry. http://openmopac.net/ (accessed Jan 11, 2023).

[ref70] Brahmkshatriya P.S.; Dobes P.; Fanfrlik J.; Rezac J.; Paruch K.; Bronowska A.; et al. Quantum Mechanical Scoring: Structural and Energetic Insights into Cyclin-Dependent Kinase 2 Inhibition by Pyrazolo[1,5-a]pyrimidines. Curr. Comput.-Aided Drug Des. 2013, 9, 118–129. 10.2174/1573409911309010011. 23157414

[ref71] Horn Q. L.; Jones D. S.; Evans R. N.; Ogle C. A.; Masterman T. C. Chloro(1,5-cyclooctadiene)(triphenylphosphine)rhodium(I). Acta Crystallogr., Sect. E:Struct. Rep. Online 2002, 58, m51–m52. 10.1107/S1600536802000077.

[ref72] Bonds W. D.; Ibers J. A. P-Bonded Disulfur. Structure of Disulfurbis (bis (diphenylphosphino) ethane) iridium (I) Chloride. Acetonitrile. J. Am. Chem. Soc. 1972, 94, 3413–3419. 10.1021/ja00765a026.

[ref73] Sanchez-Delgado R. A.; Thewalt U.; Valencia N.; Andriollo A.; Marquez-Silva R. L.; Puga J.; et al. Chemistry and Catalytic Properties of Ruthenium and Osmium Complexes. 2. Synthesis and Characterization of New Mononuclear and Dinuclear Complexes with Hydride, Carboxylate, and Phosphine Ligands. X-ray Crystal and Molecular Structures of OsBr(OCOMe) CO)(PPh3)2, RuCl(OCOMe)(CO)(PPh3)2, and [PPh3Me]+[Ru2Cl2(.mu.-Cl)3(CO)2(PPh3)2]-. Inorg. Chem. 1986, 25, 1097–1106. 10.1021/ic00228a009.

[ref74] García-Jacas C. R.; Marrero-Ponce Y.; Acevedo-Martínez L.; Barigye S. J.; Valdés-Martiní J. R.; Contreras-Torres E. QuBiLS-MIDAS: A parallel free-software for molecular descriptors computation based on multilinear algebraic maps. J. Comput. Chem. 2014, 35, 1395–1409. 10.1002/jcc.23640. 24889018

[ref75] Ghose A. K.; Viswanadhan V. N.; Wendoloski J. J. Prediction of hydrophobic (lipophilic) properties of small organic molecules using fragmental methods: An analysis of ALOGP and CLOGP methods. J. Phys. Chem. A 1998, 102, 3762–3772. 10.1021/jp980230o.

[ref76] Gasteiger J.; Marsili M. Iterative partial equalization of orbital electronegativity—a rapid access to atomic charges. Tetrahedron 1980, 36, 3219–3228. 10.1016/0040-4020(80)80168-2.

[ref77] Ertl P.; Rohde B.; Selzer P. Fast calculation of molecular polar surface area as a sum of fragment-based contributions and its application to the prediction of drug transport properties. J. Med. Chem. 2000, 43, 3714–3717. 10.1021/jm000942e. 11020286

[ref78] Yap C. W. PaDEL-descriptor: An open source software to calculate molecular descriptors and fingerprints. J. Comput. Chem. 2011, 32, 1466–1474. 10.1002/jcc.21707. 21425294

[ref79] R Core Team. R: A Language and Environment for Statistical Computing. 2023.

[ref80] Zhou Z.-H. Dimensionality Reduction and Metric Learning. Mach. Learn. 2021, 241–264. 10.1007/978-981-15-1967-3_10.

[ref81] Alghushairy O.; Alsini R.; Soule T.; Ma X. A Review of Local Outlier Factor Algorithms for Outlier Detection in Big Data Streams. Big Data Cogn. Comput. 2021, 5, 1 10.3390/bdcc5010001.

[ref82] McNamara J. P.; Berrigan S. D.; Hillier I. H. Semiempirical molecular orbital scheme to study lanthanide(III) complexes: PM3 parameters for europium, gadolinium, and ytterbium. J. Chem. Theory Comput 2007, 3, 1014–1027. 10.1021/ct600304g. 26627420

[ref83] Kwiecień R. A.; Rostkowski M.; Dybała-Defratyka A.; Paneth P. Validation of semiempirical methods for modeling of corrinoid systems. J. Inorg. Biochem. 2004, 98, 1078–1086. 10.1016/j.jinorgbio.2004.02.030. 15149818

[ref84] Martín A.; Orpen A. G. Structural systematics. 6. 1Apparent flexibility of metal complexes in crystals. J. Am. Chem. Soc. 1996, 118, 1464–1470. 10.1021/ja953301v.

[ref85] Minenkov Y.; Singstad Å.; Occhipinti G.; Jensen V. R. The accuracy of DFT-optimized geometries of functional transition metal compounds: A validation study of catalysts for olefin metathesis and other reactions in the homogeneous phase. Dalton Trans. 2012, 41, 5526–5541. 10.1039/c2dt12232d. 22430848

[ref86] Bühl M.; Reimann C.; Pantazis D. A.; Bredow T.; Neese F. Geometries of third-row transition-metal complexes from density-functional theory. J. Chem. Theory Comput. 2008, 4, 1449–1459. 10.1021/ct800172j. 26621431

[ref87] Király P.; Kiss R.; Kovács D.; Ballaj A.; Tóth G. The Relevance of Goodness-of-fit, Robustness and Prediction Validation Categories of OECD-QSAR Principles with Respect to Sample Size and Model Type. Mol. Inf. 2022, 41, 2200072 10.1002/minf.202200072. PMC9787734 35773201

[ref88] Wiesinger M.; Knüpfer C.; Elsen H.; Mai J.; Langer J.; Harder S. Heterometallic Mg–Ba Hydride Clusters in Hydrogenation Catalysis. ChemCatChem 2021, 13, 4567–4577. 10.1002/cctc.202101071.

[ref89] Chen H.; Yang M.; Liu J.; Lu G.; Feng X. Insight into the effects of electronegativity on the H2 catalytic activation for CO2 hydrogenation: four transition metal cases from a DFT study. Catal. Sci. Technol. 2020, 10, 5641–5647. 10.1039/D0CY01009J.

[ref90] Sciabola S.; Alex A.; Higginson P. D.; Mitchell J. C.; Snowden M. J. Theoretical Prediction of the Enantiomeric Excess in Asymmetric Catalysis. An Alignment-Independent Molecular Interaction Field Based Approach. 2005, pp 9025–9027. 10.1021/jo051496b 16238344

[ref91] Occhipinti G.; Bjørsvik H. R.; Jensen V. R. Quantitative structure-activity relationships of ruthenium catalysts for olefin metathesis. J. Am. Chem. Soc. 2006, 128, 6952–6964. 10.1021/ja060832i. 16719476

[ref92] Hageman J. A.; Westerhuis J. A.; Frühauf H. W.; Rothenberg G. Design and Assembly of Virtual Homogeneous Catalyst Libraries – Towards in silico Catalyst Optimisation. Adv. Synth. Catal. 2006, 348, 361–369. 10.1002/adsc.200505299.

[ref93] Burello E.; Rothenberg G. Topological Mapping of Bidentate Ligands: A Fast Approach for Screening Homogeneous Catalysts. Adv. Synth. Catal. 2005, 347, 1969–1977. 10.1002/adsc.200505220.

[ref94] Rosales M.; Arrieta F.; Baricelli P.; Colina A.; Izquierdo R. A detailed DFT theoretical investigation of the mechanism of quinoline hydrogenation catalyzed by a (1,5-cyclooctadiene)rhodium(I) complex. Catal. Today 2025, 447, 115160 10.1016/j.cattod.2024.115160.

[ref95] Li Y.; Guo X.; Zhang S.; He Y. A Perspective Review on N-Heterocycles as Liquid Organic Hydrogen Carriers and Their Hydrogenation/Dehydrogenation Catalysts. Energy Fuels 2024, 38, 12447 10.1021/acs.energyfuels.4c01633.

[ref96] Rosales M.; González T.; Atencio R.; Sánchez-Delgado R. A. Synthesis and reactivity of iridium complexes with pyridine and piperidine ligands: Models for hydrodenitrogenation. Dalton Trans. 2004, 2952–2956. 10.1039/b406949h. 15349172

[ref97] Falivene L.; Cao Z.; Petta A.; Serra L.; Poater A.; Oliva R.; et al. Towards the online computer-aided design of catalytic pockets. Nat. Chem. 2019, 11, 872–879. 10.1038/s41557-019-0319-5. 31477851

[ref98] Falivene L.; Credendino R.; Poater A.; Petta A.; Serra L.; Oliva R.; et al. SambVca 2. A Web Tool for Analyzing Catalytic Pockets with Topographic Steric Maps. Organometallics 2016, 35, 2286–2293. 10.1021/acs.organomet.6b00371.

[ref99] Altan O.; Yılmaz M. K. New phosphine-amino-alcohol tridentate ligands for ruthenium catalyzed asymmetric transfer hydrogenation of ketones. J. Organomet. Chem. 2018, 861, 252–262. 10.1016/j.jorganchem.2018.02.046.

[ref100] Nayak M. K.; Roy S. Electronic contribution of ligands and metal in palladium and palladium-tin bimetallic complexes: A comparative study of reactivity descriptors using different ligands. J. Mol. Struct. 2024, 1312, 138626 10.1016/j.molstruc.2024.138626.

[ref101] Consorti C. S.; Aydos G. L. P.; Ebeling G.; Dupont J. Ionophilic phosphines: Versatile ligands for ionic liquid biphasic catalysis. Org. Lett. 2008, 10, 237–240. 10.1021/ol702664a. 18154296

[ref102] Baricelli P. J.; Pereira J. C.; Rosales M. Aqueous-biphasic catalysis: A technological alternative for the use of organometallic complexes in hydrogenation and hydroformylation reactions with possible industrial application. Catal. Today 2025, 443, 114969 10.1016/j.cattod.2024.114969.

