# Supplementary material for: Molecular and Descriptor Spaces for Predicting Initial Rate of Catalytic Homogeneous Quinoline Hydrogenation with Ru, Rh, Os, and Ir Catalysts
Source: ACS Omega. 2025 Apr 30;10(18):18312–31. doi: 10.1021/acsomega.4c09503 (PMC12079282; doi:10.1021/acsomega.4c09503)
Supplement: Supplementary file 1 — ao4c09503_si_001.pdf [file ao4c09503_si_001.pdf]

Supporting Information (SI):

Molecular and Descriptor Spaces for Predicting Initial Rate of Catalytic Homogeneous Quinoline Hydrogenation with Ru, Rh, Os, and Ir Catalysts

Rodolfo Izquierdo,[1*] Rafael Zadorosny,[1] Merlín Rosales,[2] Yovani Marrero-Ponce,[3,4] and Néstor Cubillan[5*]

[1] Departamento de Física e Química, Universidade Estadual Paulista (UNESP), Faculdade de Engenharia, Caixa Postal 31, 15385-000, Ilha Solteira, SP, Brazil.

[2] Grupo de Química Bioorgánica, Facultad de Ciencias Exactas y Naturales, Universidad de Cartagena, Cartagena de Indias 130015, Colombia.

[3] Facultad de Ingeniería, Universidad Panamericana, Augusto Rodin No. 498, Insurgentes Mixcoac, Benito Juárez, Ciudad de México 03920, México.

[4]Universidad San Francisco de Quito (USFQ), Grupo de Medicina Molecular y Traslacional (MeM&T), Colegio de Ciencias de la Salud (COCSA), Escuela de Medicina, Quito, Ecuador.

[5] Programa de Química, Facultad de Ciencias Básicas, Universidad del Atlántico, Barranquilla 080003, Colombia

* Authors to whom correspondence should be addressed: rodolfo.izquierdo@unesp.br, nestorcubillan@mail.uniatlantico.edu.co

Summary

		Page
SI1	Theoretical Background	2
SI1.1	QuBiLS-MIDAS Approach	2
SI1.2	Internal sphere mechanisms of catalytic homogeneous quinoline hydrogenation with Ru, Rh, Os, and Ir catalysts	7
SI2	Optimized structures for the validation of the computational methodology	9
SI3	Optimized structures for catalytic precursors	10
SI3.1	Training set	10
SI3.2	Test set	23
SI4	Multiple Linear Regression Results.	27
SI5	Proposed Catalysts from the Multiple Linear Regression Model.	29
SI6	Information of Additional Files	48
SI7	Values of descriptors and predicted values of initial rate (descriptors and NCA projection)	49
SI8	Optimized structures in cvs format	56
	References	576

SI1. Theoretical Background

SI1.1. QuBiLS-MIDAS Approach

Molecular Descriptors (MD) are numerical values characterizing the properties of a molecule, and these can be a physicochemical property or a number obtained from a logical or mathematical procedure [1]. The molecular representation defines the MD's dimension, *i.e.*, one-dimensional as a countable (atom, group, protein) or physicochemical property; bidimensional or topological MD's such as those calculated from graph theory. Representations taken from 3D structures (R^3 coordinates, Z-matrix or distance matrix), or 4D (surface) generate the well-known 3D MD's.

Marrero-Ponce [2] defined several families of descriptors introducing the molecular vector space concept. The *n* atoms in a molecule form the molecular vector and the atomic properties are the vector components, *i.e.*, a canonical basis vectors in R^n. Each component is weighted with different "standard" atom properties (weights) for the atoms in a molecule. A matrix (**G**) encoding relations between atoms (bonds or interactions) can be a canonical basis set for the molecular vector, and hereinafter named structural information matrix. The adjacency matrix of vertices [2] and edges [3] of the molecular pseudograph, and the atom-atom Minkowski distance matrix [4] have been used as canonical basis sets. Moreover, $\mathbf{G}$ can be normalized by obtaining a matrix encoding the atom-atom interaction probability as a Markov chain process. The sum of the row elements is one, this matrix is called single stochastic matrix (${}_{ss}\boldsymbol{G}$) [50]. Two additional normalization schemes were proposed carried out to sum one in the row- and column-elements, and it is called double stochastic matrix (${}_{ds}\boldsymbol{G}$) [4], and the mutual probability matrix (${}_{mp}\boldsymbol{G}$) [5].

García-Jacas *et al*. [5] redefined the structural information matrix by expanding the atom-pair interactions to consider three and four interacting atoms through the tensor algebra. The well-known atom-pair interactions are codified in a second-rank tensor, whereas the interactions between three and four atoms involve third and fourth-rank tensors. The generalized matrices obtained with this approach give the N-Tuple Spatial-(Dis) Similarity Matrices. In this sense, the second, third- and fourth-rank tensors are the two-, three-, and four-tuple spatial-(dis)similarity matrices: $\boldsymbol{G}$, $\boldsymbol{GT}$, and $\boldsymbol{GQ}$, respectively.

The metric for interatom relations evolved from 2D topological and 3D Minkowski distances(**M1**-**M7**) to other distance measurements. Among these Chebyshev/Lagrange (**M8**), Canberra (**M10**), Lance-Williams/Bray-Curtis (**M11**), Clark/Coefficient of Divergence (**M12**), Soergel (**M13**), Bhattacharyya (**M14**), Wave-Edges (**M15**), and Angular Separation/[1-Cosine (Ochiai)] (**M16**) distances measurements are codified [6]. For three and four atoms, including lone pairs, the metrics are in Table S1. $\boldsymbol{GT}$, and $\boldsymbol{GQ}$ components are their distance values if the tensor indices pointed different atoms, but it is the distance to the molecule center or the number of lone pairs if the subindices are equals (tensor diagonal), and zero otherwise.

Table S1. Measures of three and four atoms' relations in a molecule.

Atoms	Measure	Symbol
3, 4	Perimeter	**M19-M20**
3	Triangle Area	**M21-M21**
3, 4	Sides Summation	**M25-M26**
3	Bond Angle	**M27-M28**
4	Volume	**M23-M24**
4	Dihedral Angle	**M29-M30**

The main advantages of the matrix representation are the possibility of encoding a molecular fragment. It is possible partitioning the molecule by creating a matrix with structural information of the fragment (F) from the total matrix, as follows:

$$g_{ij}(F) = g_{ij} \text{ if } i \ \wedge j \ \in F$$

$$g_{ij}(F) = \frac{k}{m} g_{ij} \text{ if } i \vee j \ \in F \tag{1}$$

$$g_{ij}(F) = 0 \quad \text{otherwise}$$

m takes values of 2, 3, and 4 for the $\boldsymbol{G}$, $\boldsymbol{GT}$, and $\boldsymbol{GQ}$ matrices, respectively and k is the number of atoms in the fragment F. With this approach, for N molecular fragments forming any molecule, it must be possible to divide the total matrix into N local matrices. Consequently, the total matrix is the sum of the local matrices.

Moreover, the structural information matrix and molecular vectors comprises the information to one real number through an $R^n \rightarrow R^1$ application. These algebraic mapping can be linear, quadratic and bilinear as formulated by Marrero-Ponce [2]. In the linear form, an atom "a" in a molecule is a linear combination of the molecular

vector component *j* multiplied by the g_{aj} component of structural information matrix giving an atomic invariant (L_a) equivalent to a local vertex invariant (LOVI):

$$L_a = \sum_{j=1}^{n} g_{aj} x_j = \boldsymbol{G}[\vec{X}] \quad (2)$$

Then, the molecule is a vector with LOVI's as components.

The QuBILS-MIDAS approach broadens the linear descriptor concept by including tensor algebra operations on the *N*-tuple Spatial Metric [(Dis)-Similarity] Matrices. The LOVI's can be calculated according to N-linear algebraic forms as follow:

(a) Two-linear indices [3]:

$$_{m}L_a = \sum_{i=1}^{n}\sum_{j=1}^{n} g_{ij}^{a,k} x^i y^j = \bar{x}^T \, \boldsymbol{G}^{a,k} \, \bar{y} \quad (3)$$

(b) Three-Linear indices [2]:

$$_{tr}L_a = \sum_{i=1}^{n}\sum_{j=1}^{n}\sum_{l=1}^{n} gt_{ijl}^{a,k} x^i y^j z^l = \boldsymbol{GT}^{a,k} \cdot \bar{x} \cdot \bar{y} \cdot \bar{z} \quad (4)$$

(c) Four-Linear indices [7]:

$$_{qu}L_a = \sum_{i=1}^{n}\sum_{j=1}^{n}\sum_{l=1}^{n}\sum_{h=1}^{n} gq_{ijl}^{a,k} x^i y^j z^l w^h = \boldsymbol{GQ}^{a,k} \cdot \bar{x} \cdot \bar{y} \cdot \bar{z} \cdot \bar{w} \quad (5)$$

where x, y, z and w are the components of molecular vectors $\bar{x}$, $\bar{y}$, $\bar{z}$, and $\bar{w}$ and k is the power of the elements from the N-Tuple Spatial-(Dis) Similarity Matrix, related to the chemical interaction nature.

Accordingly, the kth two-linear indices (Eqs. 3-5) are calculated with the linear ($\mathbf{F}$), quadratic ($\mathbf{Q}$), and bilinear ($\mathbf{B}$) forms as formulated in the in Ref. [2]. For the kth three- and four-linear LOVI's, the algebraic forms take the name and nature of the aforementioned algebraic forms depending on the molecular weighting vectors $\bar{x}$, $\bar{y}$, $\bar{z}$, and $\bar{w}$ (see Table S2).

Table S2. Algebraic mapping forms for indices definition in the QuBILS-MIDAS approach.

N-linear Map	Algebraic Form	Symbol	Condition
Threelinear		$\boldsymbol{Tr}$	$\bar{x} \neq \bar{y} \neq \bar{z}$
	Quadratic – Bilinear	$\boldsymbol{TrQB}$	$(\bar{x} = \bar{y}) \neq \bar{z}$
	Bilinear	$\boldsymbol{TrB}$	$\bar{x} = \bar{y}, \bar{z} = \bar{u}^{*}$
	Cubic	$\boldsymbol{TrC}$	$\bar{x} = \bar{y} = \bar{z}$
	Linear	$\boldsymbol{TrF}$	$\bar{x} = \bar{y} = \bar{u}$
Fourlinear		$\boldsymbol{Qu}$	$\bar{x} \neq \bar{y} \neq \bar{z} \neq \bar{w}$
	Quadratic – Threelinear	$\boldsymbol{QuQTr}$	$(\bar{x} = \bar{y}) \neq \bar{z} \neq \bar{w}$
	Threelinear	$\boldsymbol{QuTr}$	$(\bar{x} = \bar{u}) \neq \bar{y} \neq \bar{z} \neq \bar{w}$
	Cubic – Bilinear	$\boldsymbol{QuCB}$	$(\bar{x} = \bar{y} = \bar{z}) \neq \bar{w}$
	Bilinear	$\boldsymbol{QuB}$	$(\bar{x} = \bar{y} = \bar{u}) \neq \bar{z} \neq \bar{w}$
	Quadruple	$\boldsymbol{QuQd}$	$\bar{x} = \bar{y} = \bar{z} = \bar{w}$
	Linear	$\boldsymbol{QuF}$	$\bar{x} \neq (\bar{y} = \bar{z} = \bar{w} = \bar{u})$

* $\bar{u}$ is a vector with all entries being 1.

Once the LOVIs have been obtained, the next step is to obtain the global (whole molecule) descriptor through an aggregation operation. In the traditional scheme, the linear descriptor for a whole molecule is the sum of the atom-level descriptors [2]. In the new scheme, the hypothesis is based on the principle that the total index is not necessarily the sum of its parts [8]. In this sense, several aggregation operators have been suggested based on the norms, means, and other statistical invariants of the LOVI's vectors. Table SI-I-1-3 shows the aggregation operators included in the QuBILS-MIDAS scheme. These operators produce molecular descriptors with more reliable results [9]. Additionally, the N-tuple Spatial-(Dis) Similarity Matrices are suitable to be simplified by neglecting interactions between farthest chemical entities. García-Jacas *et al.* proposed a cutoff scheme to accomplish this. If the involved atoms are into the imposed limits named *lag*, the interaction is taken into account, else is neglected [10]. Thereby, the authors defined the path cutoff (*lag* P or **LGP**), based on the topological distance for each N-tuple Spatial-(Dis) Similarity Matrices: *lag* P for N = 2, *lag* 3P for N = 3 and *lag* 4P for N = 4. Likewise, the length cutoff takes account for the Euclidean geometric distance, denoted as *lag* L (**LGL**) for N = 2, *lag* 3L for N = 3 and *lag* 4L for N = 4. For three-atoms relations the cutoff considers bond angles (*lag* BA or **LGBA**), atom-triangle area (*lag* A or **LGA**), and triangle perimeter (*lag* TP ot **LGTP**). Finally, the four-atoms relations include volume (lag V or **LGV**), dihedral angle (*lag* DA or **LGDA**), and quaternary perimeter (*lag* QP or **LGQP**).

Table S3. Aggregation operators

Group	Aggregation operation
Norms	**N1**: Manhattan distance
	**N2**: Euclidean distance
	**N3**: Minkowski distance
Means	**GM**: Geometric Mean
	**AM**: Arithmetic mean (α=1)
	**P2**: Quadratic mean (α=2)
	**P3**: Potential mean (α=3)
	**HM**: Harmonic mean (α=-1)
Statistics	**V**: Variance
	**S**: Skewness
	**K**: Kurtosis
	**SD**: Standard deviation
	**VC**: Variation coefficient
	**RA**: Range
	**Q1**: Percentile 25
	**Q2**: Percentile 50
	**Q3**: Percentile 75
	**I50**: Interquartile range (Q3-Q1)
	**MX**: Maximum
	**MN**: Minimum
Classics	**AC**: Autocorrelation
	**GV**: Gravitational
	**TIC**: Total information content
	**MIC**: Mean Information content
	**SIC**: Standardized information content
	**TS**: Total sum
	**IB**: Ivanciuc-Balaban
	**ES**: Electrotopological state
	**KH**: Kier-Hall connectivity

The QuBiLS-MIDAS calculates descriptors with geometrical parameters of chemical compounds and the different approaches and constraints summarized as projects. The lexical structure of the descriptors is displayed in Figure S1.

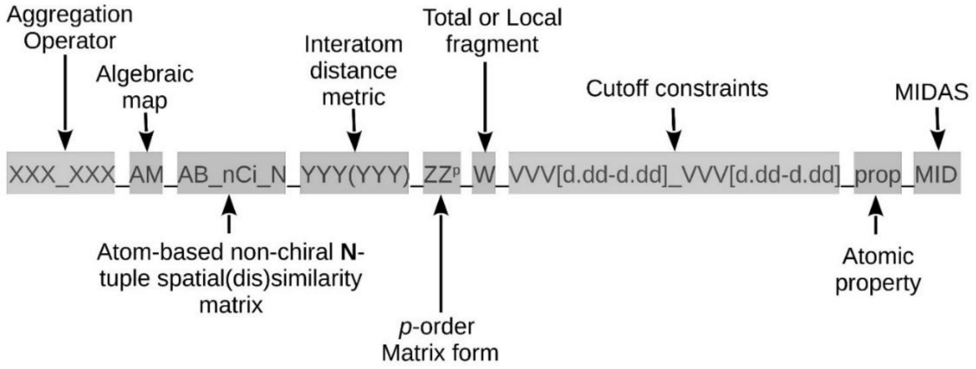

Figure S1. Lexical structure of descriptor name codification in QuBiLS-MIDAS software. The interatomic distance metric is described in the text and Table S1. The algebraic maps and aggregation operators are depicted in Table S2 and S3, respectively. The cutoff lag is abbreviated as mentioned in the text and the limits written between square brackets.

SI1.2. Internal sphere mechanisms of catalytic homogeneous quinoline hydrogenation with Ru, Rh, Os, and Ir catalysts

According to the kinetic, coordination chemistry, and theoretical studies performed by Sánchez-Delgado *et al.* and Rosales *et al.,* conventional catalysts based on Rh and Ir tend to form dihydride-type active species, $M(H)_2$, and present consecutive elementary steps of oxidative addition of dihydrogen followed by the concomitant transference of hydride ligands to the heteroaromatic moiety [11–14].

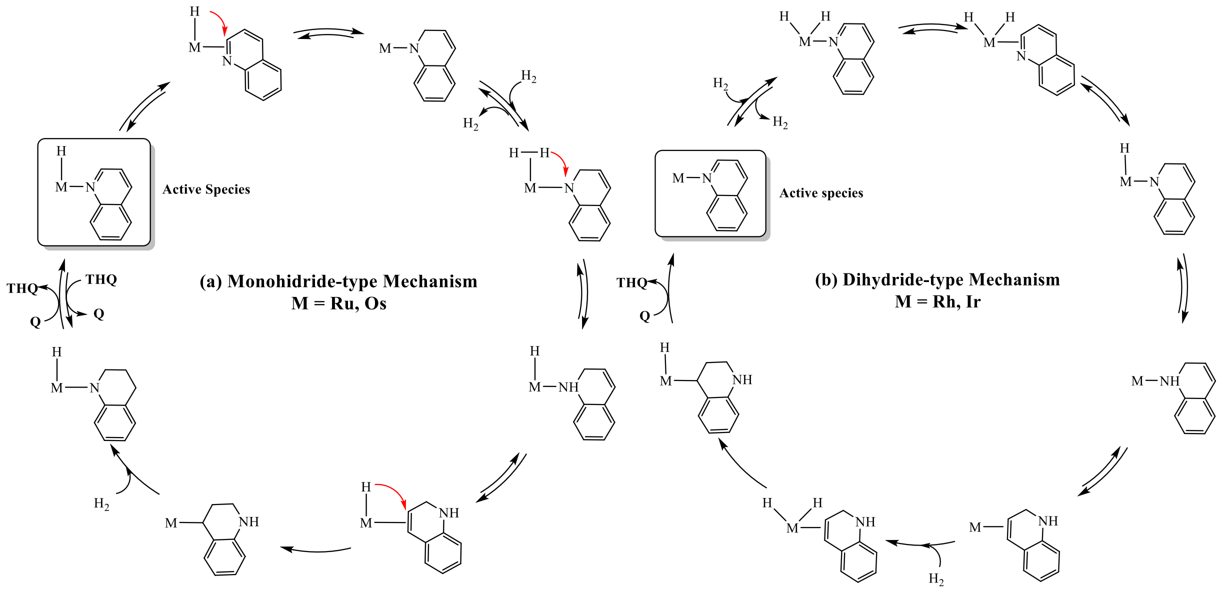

Scheme S1. Catalytic cycle for the hydrogenation of Q to ^{1}**THQ** *via* **ISM** for catalysts based on Ru and Os (monohydride catalyst, a) and Rh and Ir (dihydride catalyst, b).

In contrast, catalysts based on Ru and Os generate catalytically active species (CASs) of the monohydride-type (M-H). In the latter type of mechanism, it has been proposed that the first hydrogenation occurs by heterolytic addition of hydrogen to form a heterocycle-metal species (M-^{1}THQ), which has been isolated or detected spectroscopically [14–16]. On the whole, the mechanisms of hydrogenation of **Q** to ^{1}**THQ** (*via* **ISM**) consider the existence of one or more equilibria between metallic species containing N-heterocycles coordinated through nitrogen (*κ***N**), and another where the coordination could be by the bond C=N (*κ*^{2}**C,N**). Yet, as far as we know, this has not been proven experimentally or theoretically. Scheme S1 shows the proposed catalytic cycles for the hydrogenation of **Q** to ^{1}**THQ** through **ISM** for the Ru and Os

monohydride precursors (Scheme S1a) and Rh and Ir dihydride precursors (Scheme S1b).

In addition, it is known that the hydrogenation mechanism of **Q** to **^{1}THQ** is complex and has high activation barriers (E_a), which makes the process slow (*i.e.*, low initial rate as a measure of low catalytic activity, $\mathbf{r_0}$). Thus, the transition state theory (TST) should predict both the microscopic (E_a) and macroscopic ($\mathbf{r_0}$) parameters. Unfortunately, TST, in many cases, fails to predict $\mathbf{r_o}$, whereas, in general, it correctly predicts E_a. Therefore, we will extract information on the reaction mechanism from the best $\mathbf{r_0}$-DMs obtained in this paper without going into depth about the TST.

S2. Optimized structures for the validation of the computational methodology

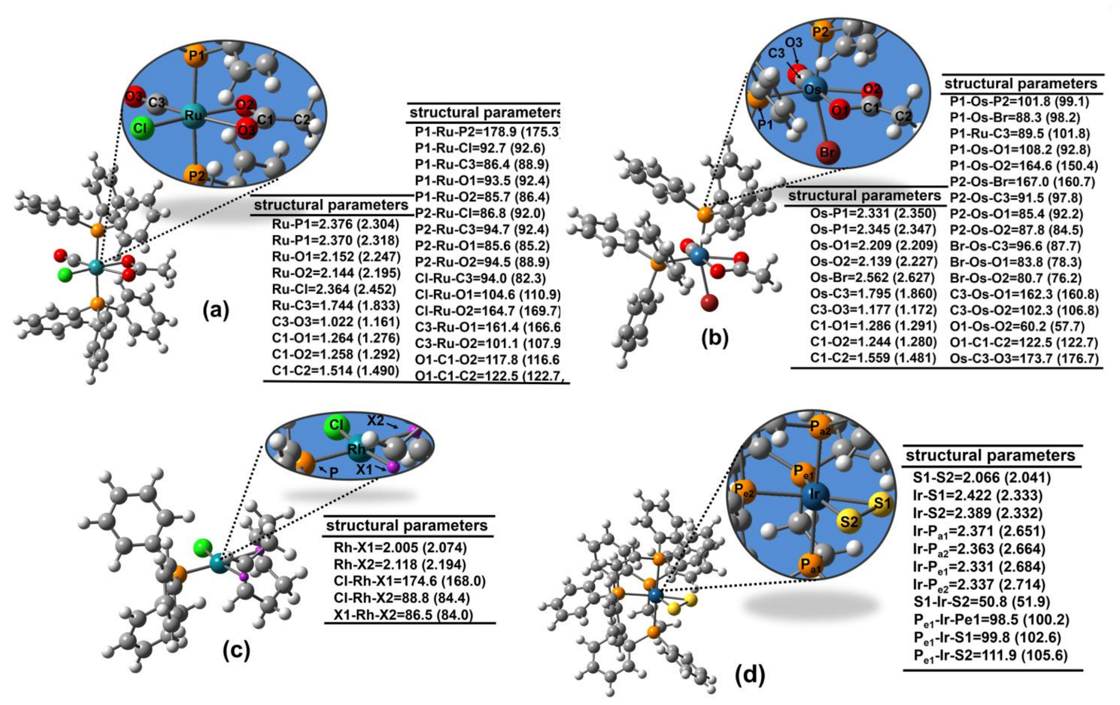

Figure S2. Optimized structures of: (a) $RuCl(\kappa^3\text{-}OCOCH_3)(CO)(PPh_3)_2$, (b) $OsBr(\kappa^3\text{-}OCOCH_3)(CO)(PPh_3)_2$, (c) $RhCl(COD)(PPh_3)$ and (d) $[IrS_2(dppe)_2]^+$. Comparison between experimental and calculated structural parameters: bond angles (α, °) and bond distances (d, Å). Calculated values obtained with PM6-D3H4X are shown in parentheses.

SI3. Optimized structures for catalytic precursors

SI3.1. Training set

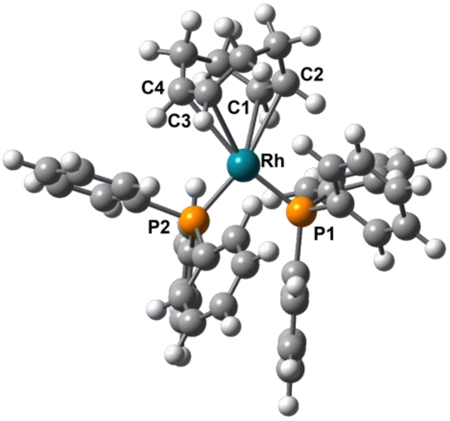

(1) $[Rh(COD)(PPh_3)_2]^+$

structural parameters
Rh-P1=2.220
Rh-P2=2.2139
Rh-C1=2.764
Rh-C2=2.769
Rh-C3=2.636
Rh-C4=2.827
P1-Rh-P2=94.1
P1-Rh-C1=99.4
P1-Rh-C2=104.3
P2-Rh-C3=106.2
P2-Rh-C4=98.5

Figure S3. Optimized structure for precursor (1)

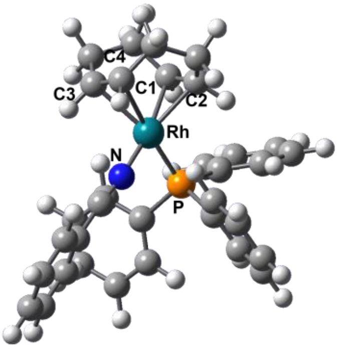

(2) $[Rh(COD)(NCPh)(PPh_3)]^+$

structural parameters
Rh-P=2.139
Rh-N=1.733
Rh-C1=2.215
Rh-C2=2.269
Rh-C3=2.401
Rh-C4=2.354
P1-Rh-N=78.9
P1-Rh-C1=99.6
P1-Rh-C2=101.1
N-Rh-C3=98.2
N-Rh-C4=94.6

Figure S4. Optimized structure for precursor (2)

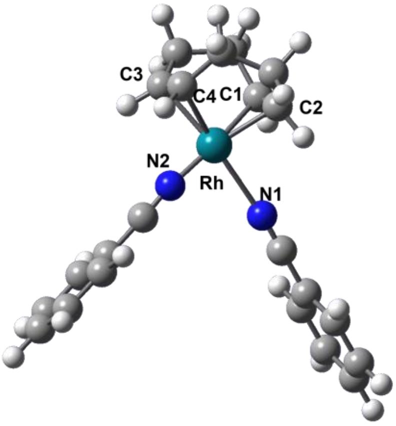

(3) $[Rh(COD)(NCPh)_2]^+$

structural parameters
Rh-N1=2.467
Rh-N2=1.675
Rh-C1=2.045
Rh-C2=2.075
Rh-C3=2.188
Rh-C4=2.175
N1-Rh-N2=86.6
N1-Rh-C1=89.5
N1-Rh-C2=90.8
N2-Rh-C3=94.7
N2-Rh-C4=91.6

Figure S5. Optimized structure for precursor (3)

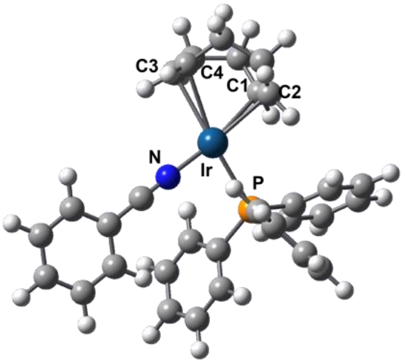

(4) $[Ir(COD)(NCPh)(PPh_3)]^+$

structural parameters
Ir-P=2.602
Ir-N=1.933
Ir-C1=2.486
Ir-C2=2.645
Ir-C3=2.814
Ir-C4=2.742
P-Ir-N=85.2
P-Ir-C1=100.6
P-Ir-C2=99.7
N-Ir-C3=104.8
N-Ir-C4=101.2

Figure S6. Optimized structure for precursor (4)

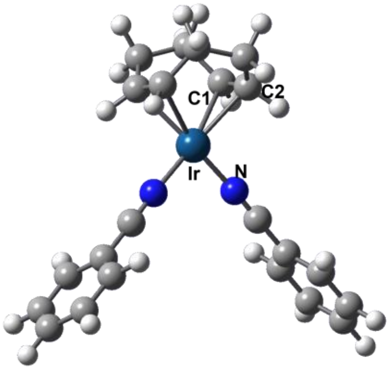

(5) $[Ir(COD)(NCPh)_2]^+$

structural parameters
Ir-N=1.965
Ir-N=1.965
Ir-C1=2.371
Ir-C2=2.418
N-Ir-N=91.3
N-Ir-C1=93.0
N-Ir-C2=95.8

Figure S7. Optimized structure for precursor (5)

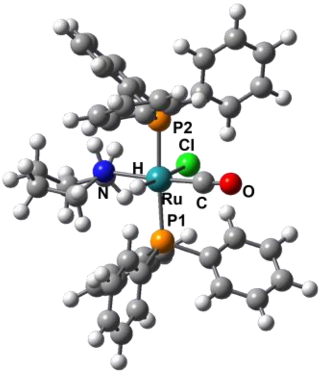

(6) $RuHCl(CO)(piperidine)(PPh_3)_2$

structural parameters
Ru-N=2.246
Ru-P1=2.348
Ru-P2=2.288
Ru-C=2.857
Ru-Cl=2.834
Ru-H=2.006
N-Ru-H=90.4
N-Ru-Cl=88.6
C-Ru-H=86.0
P1-Ru-C=87.8
P2-Ru-N=88.0
P1-Ru-P1=174.6

Figure S8. Optimized structure for precursor (6)

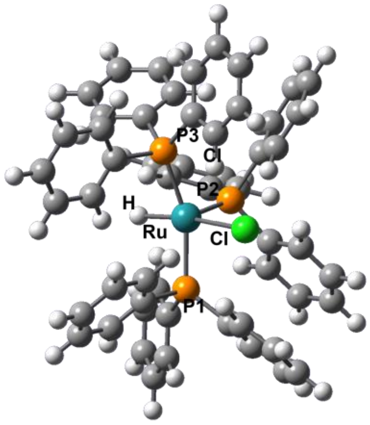

(7) $RuHCl(PPh_3)_3$

structural parameters
Ru-H=2.009
Ru-P1=2.338
Ru-P2=2.211
Ru-P3=2.357
Ru-Cl=2.847
H-Ru-P1=86.6
H-Ru-P2=170.7
H-Ru-P3=84.9
Cl-Ru-P1=88.2
Cl-Ru-P2=97.3

Figure S9. Optimized structure for precursor (7)

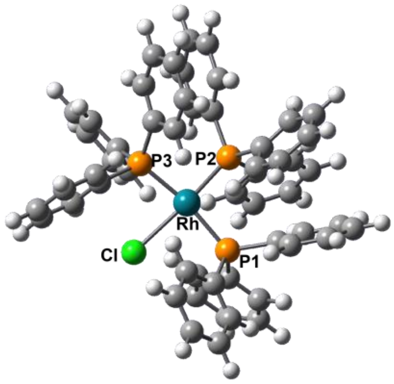

(8) $RhCl(PPh_3)_3$

structural parameters
Rh-P1=2.249
Rh-P2=2.275
Rh-P3=2.225
Rh-Cl=2.608
Cl-Ru-P1=87.4
Cl-Ru-P3=83.2
P1-Ru-P2=96.1

Figure S10. Optimized structure for precursor (8)

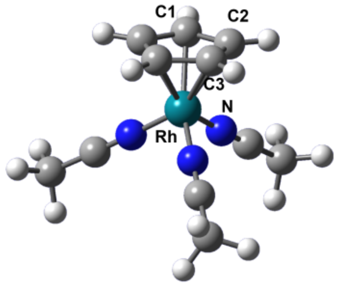

(9) $[Cp^{*}Rh(NCMe)_3]^{2+}$

structural parameters
Rh-C1=2.149
Rh-C2=2.152
Rh-C3=2.117
Rh-N=1.700
N-Rh-C1=118.9
N-Rh-C2=90.7
N-Rh-C3=97.7

Figure S11. Optimized structure for precursor (9)

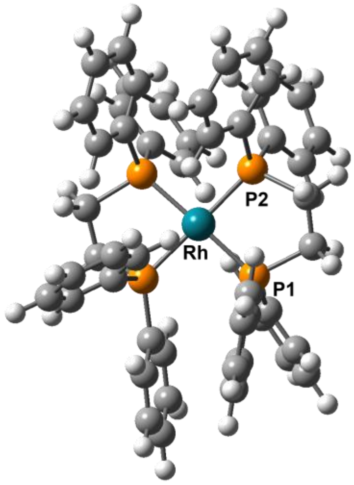

(10) $[Rh(dppe)_2]^{+}$

structural parameters
Rh-P1=2.252
Rh-P2=2.24
P1-Rh-P2=87.4

Figure S12. Optimized structure for precursor (10)

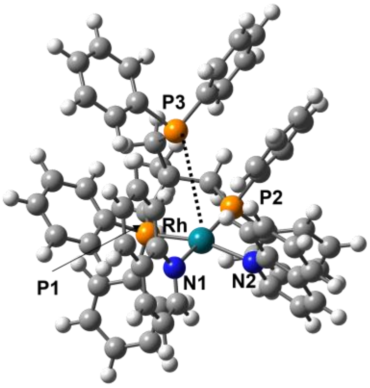

(11) $[Rh(Q)_2(\kappa^2\text{-triphos})]^+$

**structural parameters**
**Rh-P1=2.286**
**Rh-P2=2.295**
**Rh-P3=4.179**
**Rh-N1=2.203**
**Rh-N2=2.226**
**P1-Rh-P2=91.8**
**P2-Rh-P3=63.7**
**N1-Rh-P1=92.1**

Figure S13. Optimized structure for precursor (11)

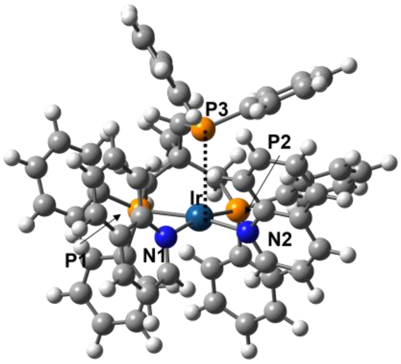

(12) $[Ir(Q)_2(\kappa^2\text{-triphos})]^+$

**structural parameters**
**Ir-P1=2.603**
**Ir-P2=2.643**
**Ir-P3=3.253**
**Ir-N1=2.098**
**Ir-N2=2.085**
**P1-Ir-P2=87.8**
**P2-Ir-P3=74.2**
**N1-Ir-P1=87.8**

Figure S14. Optimized structure for precursor (12)

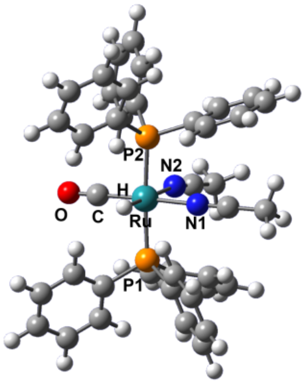

(13) $[RuH(CO)(NCMe)_2(PPh_3)_2]^+$

structural parameters
Ru-P1=2.277
Ru-P2=2.295
Ru-N1=2.206
Ru-N2=2.417
Ru-H=2.002
Ru-C=1.863
C-O=1.153
P1-Ru-P2=174.9
P1-Ru-N1=85.0
P2-Ru-N2=88.2
P1-Ru-C=92.8
P2-Ru-H=89.5

Figure S15. Optimized structure for precursor (13)

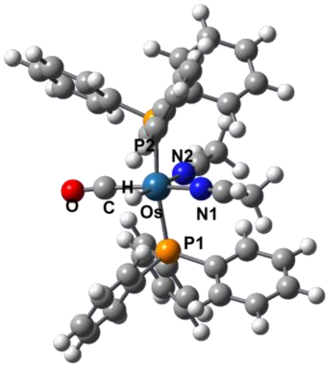

(14) $[OsH(CO)(NCMe)_2(PPh_3)_2]^+$

structural parameters
Os-P1=2.375
Os-P2=2.375
Os-N1=1.945
Os-N2=1.969
Os-H=1.660
Os-C=1.965
C-O=1.162
P1-Os-P2=171.0
P1-Os-N1=86.9
P2-Os-N2=86.9
P1-Os-C=93.8
P2-Os-H=92.9

Figure S16. Optimized structure for precursor (14)

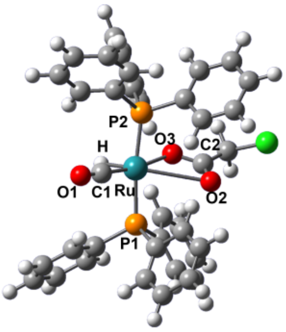

(15) RuH(CO)*(κ^3-OCOCH$_2$Cl)*(PPh$_3$)$_2$

structural parameters
Ru-P1=2.277
Ru-P2=2.242
Ru-C1=1.855
C1-O1=1.593
Ru-H=1.995
Ru-O2=3.065
Ru-O3=1.310
O2-C2=1.224
O3-C2=1.310
P1-Ru-P2=169.7
P1-Ru-C1=89.4
P1-Ru-H=90.5
P1-Ru-O2=78.7
P1-Ru-O3=100.0
O2-C2-O3=123.4

Figure S17. Optimized structure for precursor (15).

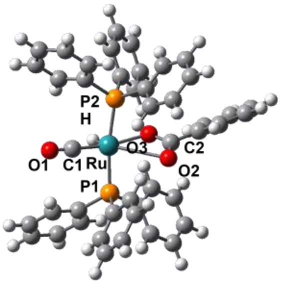

(16) RuH(CO)*(κ^3-OCOC$_6$H$_5$)*(PPh$_3$)$_2$

structural parameters
Ru-P1=2.275
Ru-P2=2.242
Ru-C1=1.852
C1-O1=1.1593
Ru-H=1.995
Ru-O2=3.026
Ru-O3=2.122
O2-C2=1.232
O3-C2=1.311
P1-Ru-P2=170.2
P1-Ru-C1=89.1
P1-Ru-H=90.7
P1-Ru-O2=79.7
P1-Ru-O3=100.5
O2-C2-O3=121.9

Figure S18. Optimized structure for precursor (16).

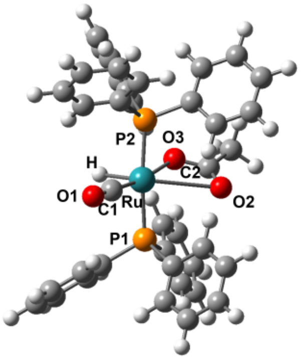

(17) RuH(CO)*(κ³-OCOCH₃)*(PPh₃)₂

structural parameters
Ru-P1=2.277
Ru-P2=2.239
Ru-C1=1.855
C1-O1=1.159
Ru-H=1.996
Ru-O2=3.019
Ru-O3=2.115
O2-C2=1.232
O3-C2=1.313
P1-Ru-P2=170.1
P1-Ru-C1=89.0
P1-Ru-H=90.2
P1-Ru-O2=79.6
P1-Ru-O3=100.2
O2-C2-O3=121.8

Figure S19. Optimized structure for precursor (17)

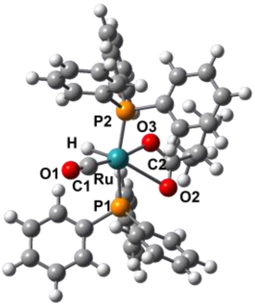

(18) RuH(CO)*(κ³-OCOCH(CH₃)₂)*(PPh₃)₂

structural parameters
Ru-P1=2.281
Ru-P2=2.233
Ru-C1=1.859
C1-O1=1.158
Ru-H=1.995
Ru-O2=2.838
Ru-O3=2.126
O2-C2=1.248
O3-C2=1.300
P1-Ru-P2=166.6
P1-Ru-C1=92.0
P1-Ru-H=94.4
P1-Ru-O2=54.4
P1-Ru-O3=97.7
O2-C2-O3=119.6

Figure S20. Optimized structure for precursor (18)

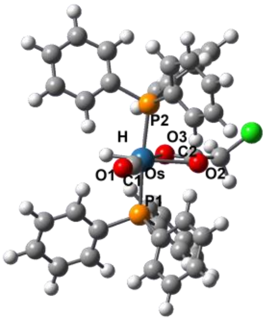

(19) OsH(CO)(*κ*[3]*-OCOCH$_2$Cl*)(PPh$_3$)$_2$

structural parameters
Os-P1=2.368
Os-P2=2.369
Os-C1=1.857
C1-O1=1.177
Os-H=1.601
Os-O2=2.386
Os-O3=2.243
O2-C2=1.265
O3-C2=1.284
P1-Os-P2=171.8
P1-Os-C1=94.0
P1-Os-H=90.7
P1-Os-O2=87.4
P1-Os-O3=86.1
O2-C2-O3=170.0

Figure S21. Optimized structure for precursor (19)

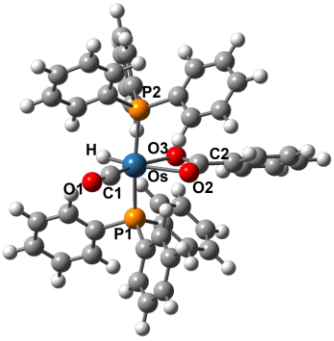

(20) OsH(CO)(*κ*[3]*-OCOC$_6$H$_5$*)(PPh$_3$)$_2$

structural parameters
Os-P1=2.364
Os-P2=2.363
Os-C1=1.863
C1-O1=1.176
Os-H=1.604
Os-O2=2.348
Os-O3=2.247
O2-C2=1.272
O3-C2=1.286
P1-Os-P2=169.0
P1-Os-C1=95.4
P1-Os-H=89.6
P1-Os-O2=88.3
P1-Os-O3=85.4
O2-C2-O3=115.3

Figure S22. Optimized structure for precursor (20)

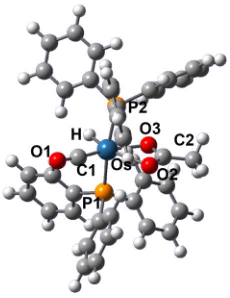

(21) OsH(CO)*(κ³-OCOCH₃)*(PPh₃)₂

structural parameters
Os-P1=2.367
Os-P2=2.362
Os-C1=1.855
C1-O1=1.177
Os-H=1.603
Os-O2=2.369
Os-O3=2.239
O2-C2=1.271
O3-C2=1.286
P1-Os-P2=171.8
P1-Os-C1=94.0
P1-Os-H=90.2
P1-Os-O2=87.9
P1-Os-O3=85.9
O2-C2-O3=115.7

Figure S23. Optimized structure for precursor (21)

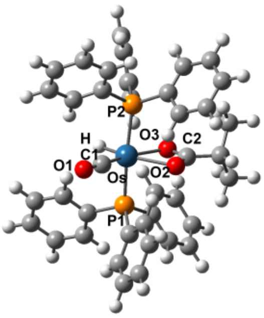

(22) OsH(CO)*(κ³-OCOCH(CH₃)₂)*(PPh₃)₂

structural parameters
Os-P1=2.364
Os-P2=2.362
Os-C1=1.868
C1-O1=1.177
Os-H=1.603
Os-O2=2.339
Os-O3=2.248
O2-C2=1.271
O3-C2=1.283
P1-Os-P2=170.3
P1-Os-C1=94.6
P1-Os-H=89.4
P1-Os-O2=88.3
P1-Os-O3=87.0
O2-C2-O3=115.4

Figure S24. Optimized structure for precursor (22)

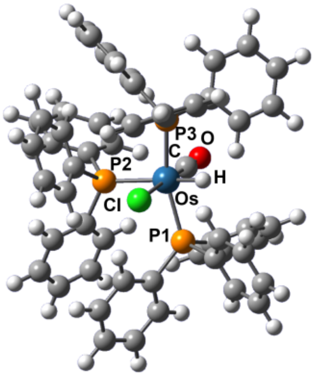

(23) $OsHCl(CO)(PPh_3)_3$

structural parameters
Os-P1=2.375
Os-P2=2.391
Os-P3=2.377
Os-C=1.870
C-O=1.179
Os-H=1.652
P1-Os-P2=101.5
P2-Os-P3=97.3
P1-Os-Cl=85.4
P1-Os-H=80.2
P1-Os-C=92.8

Figure S25. Optimized structure for precursor (23)

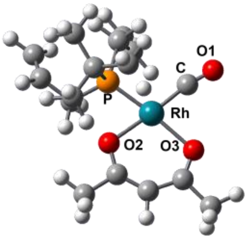

(24) $[Rh(acac)(CO)(P^tBu(CH_2CH{=}CH_2)_2]^+$

structural parameters
Rh-P=2.107
Rh-C=1.807
Rh-O2=2.039
Rh-O3=2.193
C-O=1.144
P-Rh-C=104.3
P-Rh-O2=71.9
C-Rh-O3=81.1

Figure S26. Optimized structure for precursor (24)

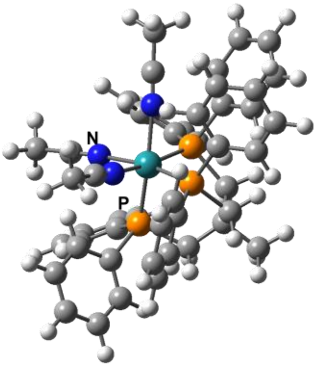

(25) $[Ru(NCMe)_3(triphos)]^{2+}$

structural parameters
Ru-P=2.268
Ru-N=2.141
P-Ru-P=94.8
P-Rh-N=78.8

Figure S27. Optimized structure for precursor (25)

SI3.2. Test set

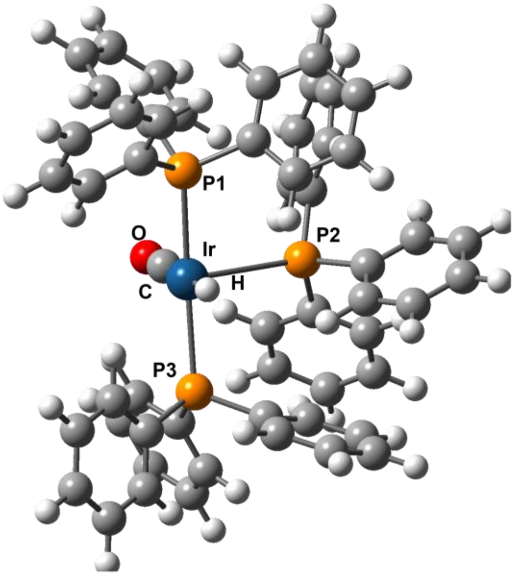

(37) $IrH(CO)(PPh_3)_3$

structural parameters
Ir-P1=2.723
Ir-P2=3.210
Ir-P3=2.703
Ir-H=1.330
Ir-C=1.552
C-O=1.165
P1-Ir-P2=90.1
P1-Ir-P3=171.4
H-Ir-P2=100.3
C-Ir-P2=79.8

Figure S28. Optimized structure for precursor (37)

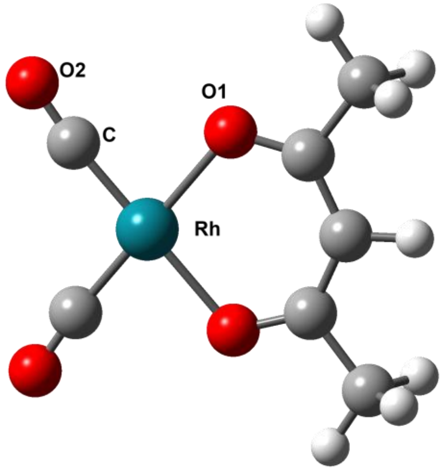

(55) $Rh(acac)(CO)_2$

structural parameters
Rh-O1=2.081
Rh-C=1.777
C-O=1.145
O1-Rh-C=79.8

Figure S29. Optimized structure for precursor (55)

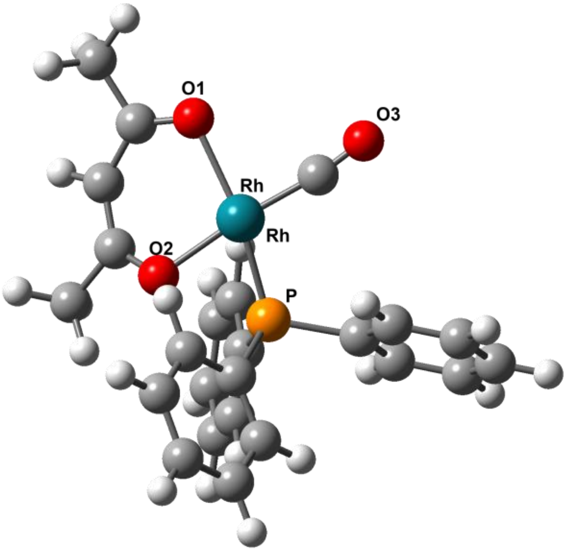

structural parameters
Rh-O1=2.255
Rh-O2=2.082
Rh-P=2.129
Rh-C=1.799
C-O3=1.144
O1-Rh-C=85.6
P-Rh-C=104.3

(59) Rh(acac)(CO)(PPh_3)

Figure S30. Optimized structure for precursor (59)

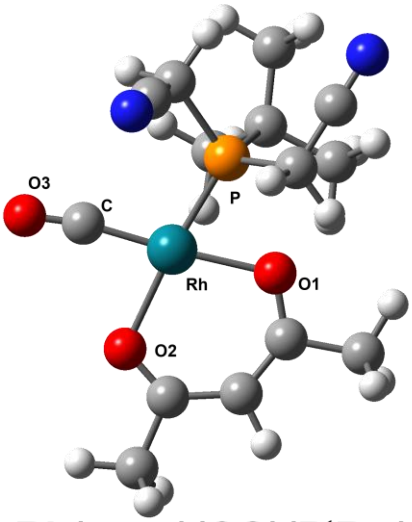

structural parameters
Rh-O1=2.048
Rh-O2=2.163
Rh-P=2.088
Rh-C=1.797
C-O3=1.143
O1-Rh-C=175.5
P-Rh-C=104.7

(177) Rh(acac)(CO)($P^tBu(CH_2CN)_2$)

Figure S31. Optimized structure for precursor (177)

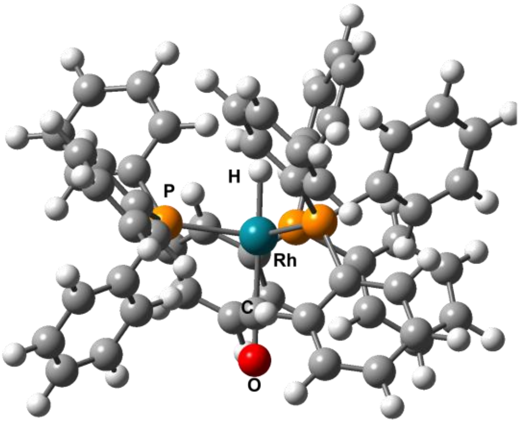

(178) $RuH(CO)(PPh_3)_3$

structural parameters
Rh-H=1.626
Rh-P=2.373
Rh-C=1.939
C-O=1.172
H-Rh-P=83.8
P-Rh-C=95.4

Figure S32. Optimized structure for precursor (178)

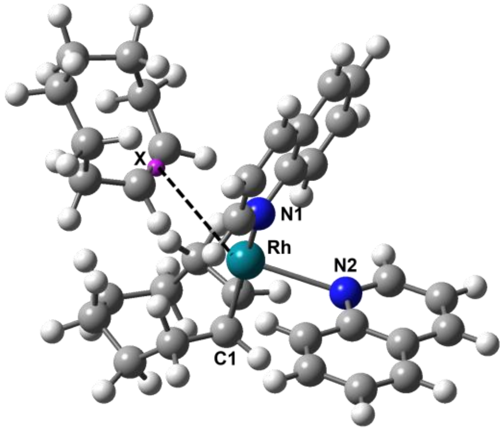

(179) $Rh(COE)_2(Q)_2^+$

structural parameters
Rh-X=3.070
Rh-N1=1.701
Rh-N2=2.654
Rh-C1=2.041
X-Rh-N1=72.5
X-Rh-C1=115.1

Figure S33. Optimized structure for precursor (179)

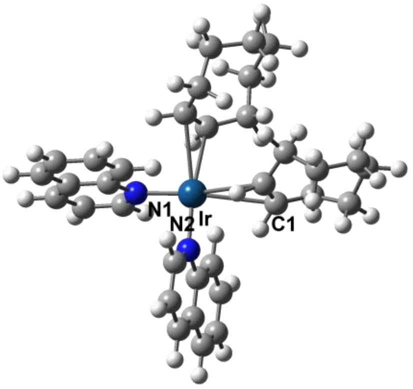

(180) $Ir(COE)_2(Q)_2^+$

structural parameters
Ir-N1=2.016
Ir-N2=2.013
Ir-C1=2.915
N1-Ir-N2=88.9
N2-Ir-C1=94.1

Figure S34. Optimized structure for precursor (180)

SI4. Multiple Linear Regression Results

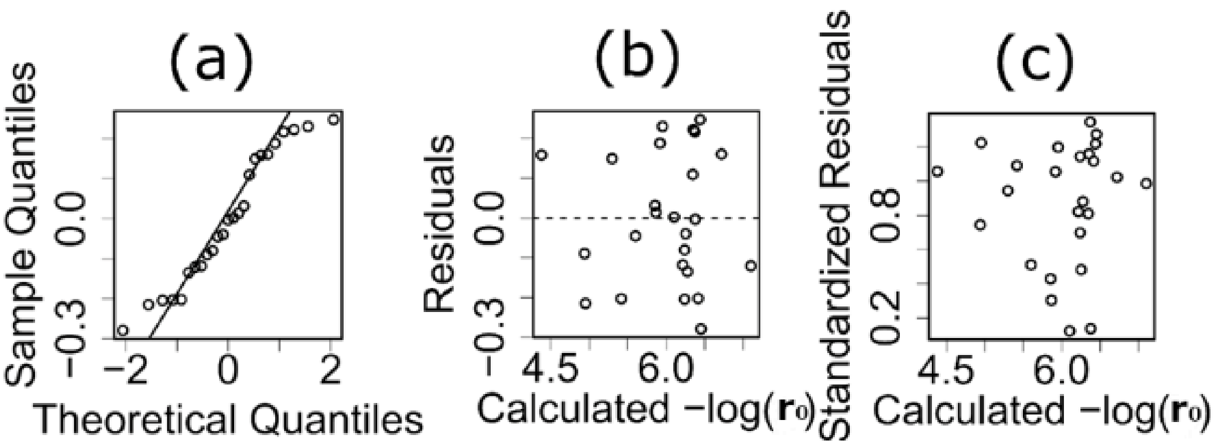

Figure S35. Diagnostic plot for assessing statistical assumptions in multiple linear regression: (a) QQ plot, (b) residual and (c) standardized residual vs. predicted values.

SI4.1. Evaluation of randomly dataset splitting

The conducted experiment consisted in randomly varying the training and testing sets over 75,000 iterations. The results were analyzed by comparing the R^2, Q^2_{LOO}, and Q^2_{ext} values across iterations. The key statistics from this analysis are summarized in the table below:

Table S4. Statistical results of R^2, Q^2_{LOO}, and Q^2_{EXT} for randomly training/test splitted models.

	R^2	Q^2_{LOO}	Q^2_{ext}
Min.	0.87	0.81	0.22
1st Qu.	0.89	0.84	0.80
Median	0.89	0.84	0.86
Mean	0.89	0.85	0.84
3rd Qu.	0.90	0.85	0.90
Max.	0.93	0.90	0.99

The mean and median values for R^2 (0.89 and 0.89, respectively) and Q^2LOO (0.85 and 0.84) are closely aligned, indicating a symmetric distribution and high consistency in internal validation results. Both R^2 and Q^2LOO exhibit a consistent spread, as reflected in their interquartile ranges (IQR), with values tightly clustered between the first (Q1) and third quartiles (Q3). Their symmetric distributions further demonstrate this behavior. As pointed by Toropov and Toropova [17]:

‘An examination of several splits decreases the probability of “chance correlations”: solely one good correlation easily can become chance correlation; but three (five, six, seven, ...) good correlations hardly can be “chance correlations”.’

In contrast, while the mean (0.84) and median (0.86) of Q^2_{ext} remain relatively close, Q^2_{ext} exhibits a variability at the extremes, with a minimum value of 0.22 and a maximum of 0.99. However, the interquartile range (Q1 = 0.80, Q3 = 0.90) indicates that most Q^2_{ext} values are distributed within a stable and reliable range. The density plot for Q^2_{ext} reveals a tail toward lower values, corresponding to the observed minimum (see Figure S36). Importantly, these extremely low values occur infrequently, as evidenced by the low density in these regions. Most Q^2_{ext} values remain tightly clustered within the interquartile range, reinforcing the consistency of the model’s external predictive performance. Overall, this analysis confirms that while Q²ext contains a slight variability at the extremes, the core results remain stable, with acceptable predictive performance.

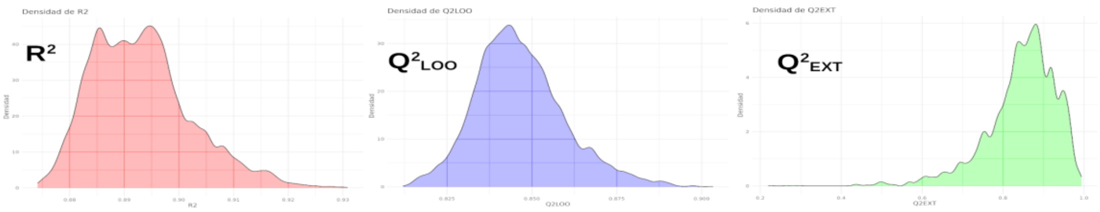

Figure S36. Density distribution of R^2, Q^2_{LOO}, and Q^2_{EXT} for randomly training/test splitted models.

SI5. Proposed Catalysts from the Multiple Linear Regression Model

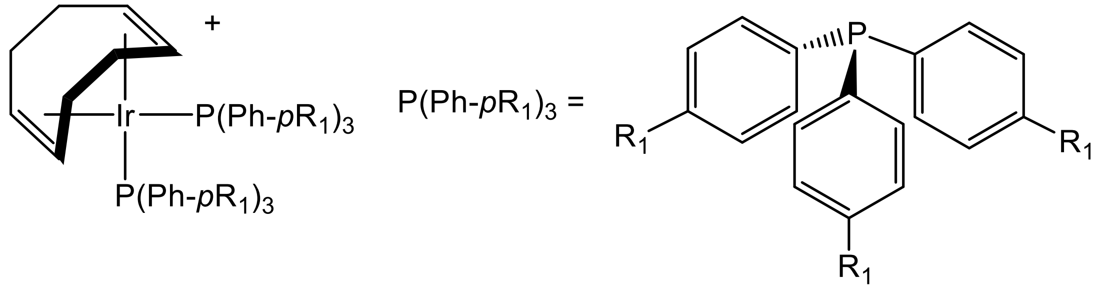

Label	26	27	28
R_1	-H	-F	$-CH_3$
**r_0** (Ms^{-1})	4.32×10^{-7}	2.56×10^{-7}	3.12×10^{-7}

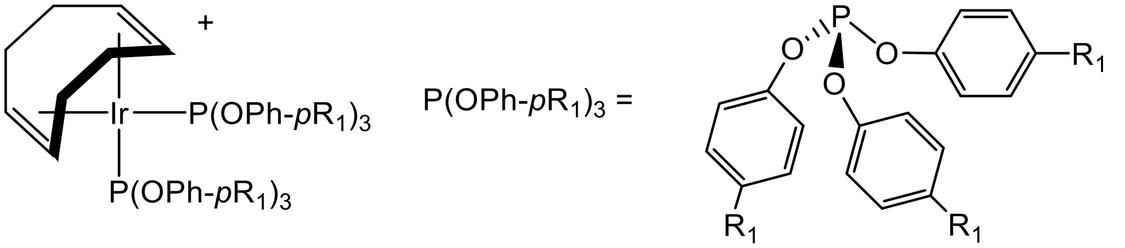

Label	29	30	31
R_1	-H	-F	$-CH_3$
**r_0** (Ms^{-1})	2.50×10^{-7}	3.35×10^{-7}	3.10×10^{-7}

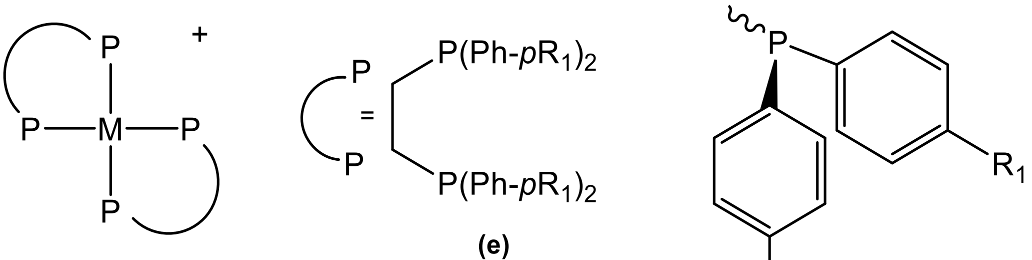

M= Rh, Ir

(e)

Label	10	32	33	34	35	36
M	Rh	Rh	Rh	Ir	Ir	Ir
R_1	-H	-F	$-CH_3$	-H	-F	$-CH_3$
**r_0** (Ms^{-1})	6.12×10^{-6}	6.44×10^{-6}	2.58×10^{-6}	5.55×10^{-6}	9.01×10^{-7}	2.36×10^{-6}

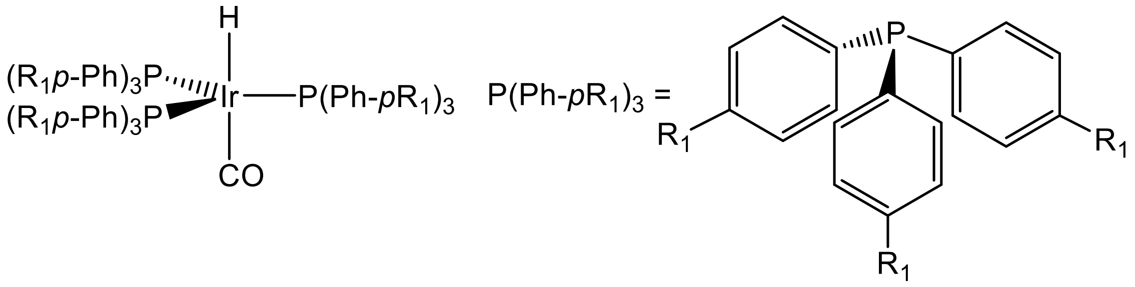

**Label**	**37**	**38**	**39**
R_1	-H	-F	$-CH_3$
**r_0** (Ms^{-1})	5.68×10^{-7}	5.14×10^{-6}	2.19×10^{-6}

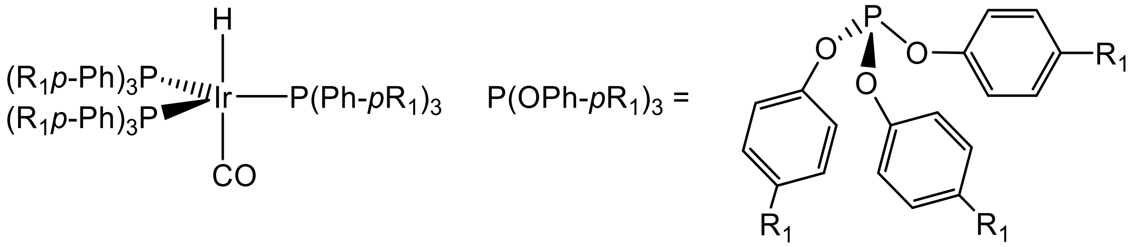

**Label**	**40**	**41**	**42**
R_1	-H	-F	$-CH_3$
**r_0** (Ms^{-1})	2.01×10^{-6}	8.17×10^{-7}	1.14×10^{-6}

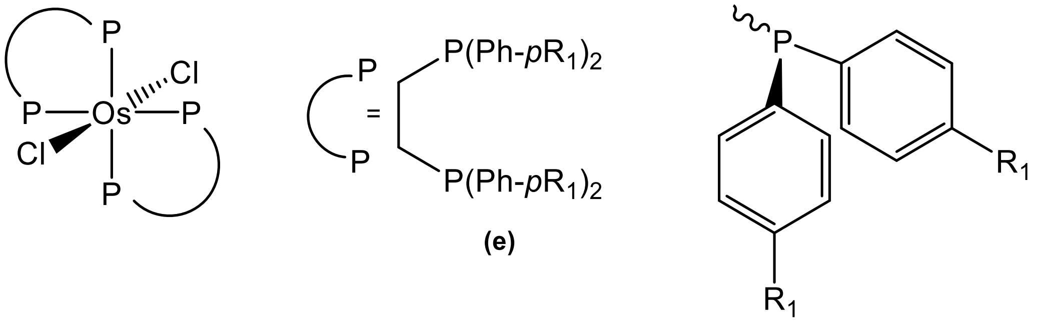

(e)

**Label**	**43**	**44**	**45**
R_1	-H	-F	$-CH_3$
**r_0** (Ms^{-1})	1.32×10^{-6}	9.83×10^{-7}	5.82×10^{-7}

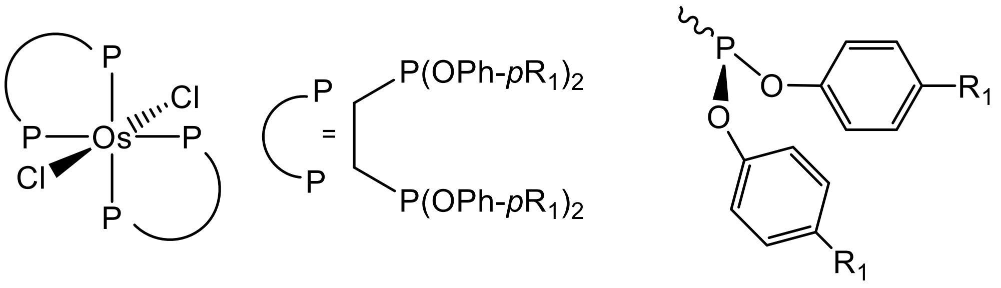

**Label**	**46**	**47**	**48**
R_1	-H	-F	$-CH_3$
**r_0** (Ms^{-1})	4.63×10^{-7}	1.66×10^{-7}	1.63×10^{-7}

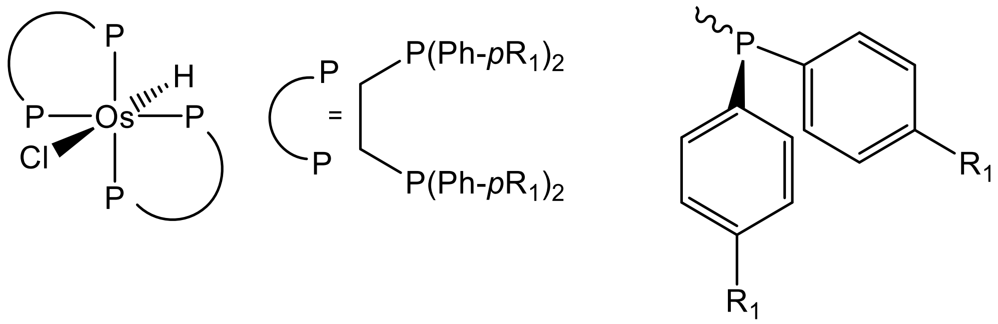

**Label**	**49**	**50**	**51**
R_1	-H	-F	$-CH_3$
$\mathbf{r_0}$ (Ms^{-1})	1.10×10^{-6}	1.20×10^{-6}	1.46×10^{-6}

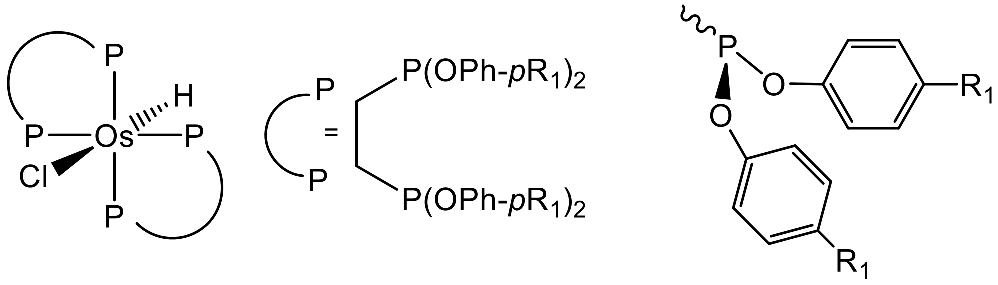

**Label**	**52**	**53**	**54**
R_1	-H	-F	$-CH_3$
$\mathbf{r_0}$ (Ms^{-1})	1.01×10^{-6}	4.30×10^{-7}	1.37×10^{-7}

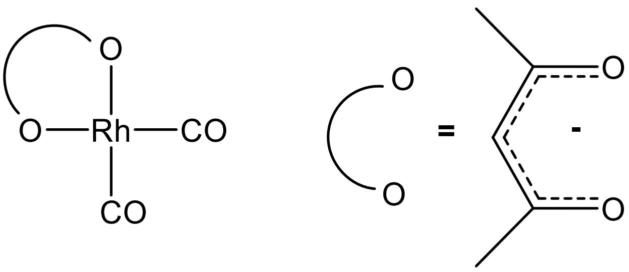

55 $\mathbf{r_0}$ (Ms^{-1})= 1.81×10^{-5}

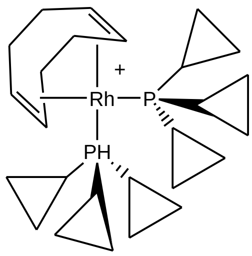

56

$\mathbf{r_0}$ (Ms^{-1}) = 1.47×10^{-6}

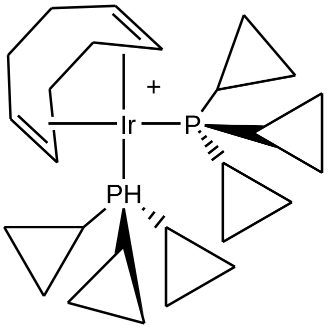

57

$\mathbf{r_0}$ (Ms^{-1}) = 1.72 x 10^{-6}

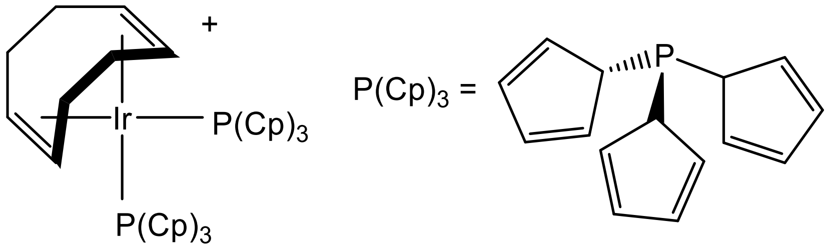

58

$\mathbf{r_0}$ (Ms^{-1}) = 1.01 x 10^{-6}

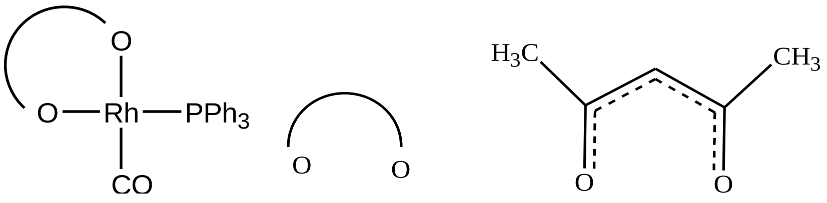

59

$\mathbf{r_0}$ (Ms^{-1}) = 1.10 x 10^{-5}

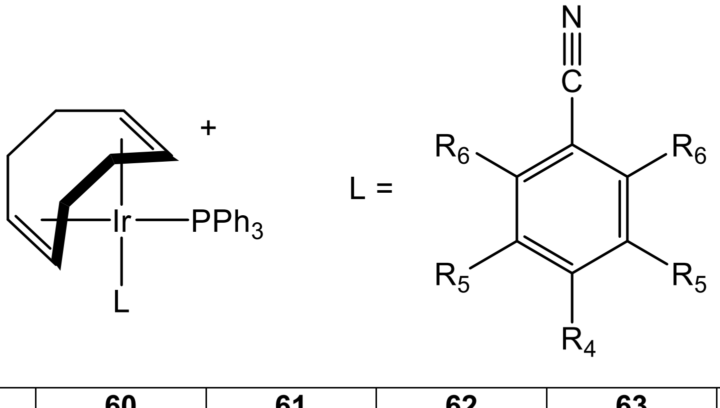

**Label**	**4**	**60**	**61**	**62**	**63**	**64**	**65**
R_4	-H	-F	-H	-H	-CH_3	-H	-H
R_5	-H	-H	-F	-H	-H	-CH_3	-H
R_6	-H	-H	-H	-F	-H	-H	CH_3
$\mathbf{r_0}$ (Ms^{-1})	6.11 x 10^{-6}	3.31 x 10^{-6}	2.61 x 10^{-6}	2.43 x 10^{-6}	3.23 x 10^{-6}	2.95 x 10^{-6}	2.79 x 10^{-6}

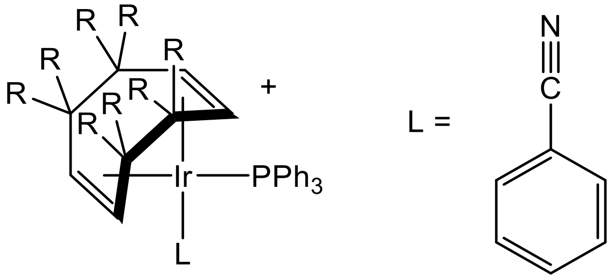

**Label**	**66**	**67**
R	-F	$-CH_3$
$\mathbf{r_0}$ (Ms^{-1})	4.51×10^{-6}	8.94×10^{-7}

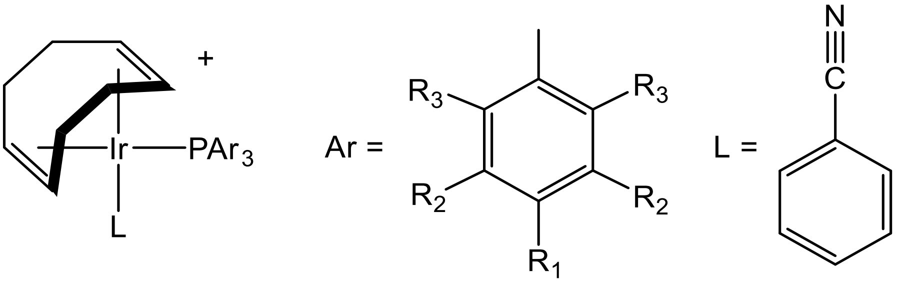

**Label**	**68**	**69**	**70**	**71**	**72**	**73**
R_1	-F	-H	-H	$-CH_3$	-H	-H
R_2	-H	-F	-H	-H	$-CH_3$	-H
R_3	-H	-H	-F	-H	-H	CH_3
$\mathbf{r_0}$ (Ms^{-1})	3.56×10^{-6}	1.94×10^{-6}	3.02×10^{-6}	2.02×10^{-6}	3.8×10^{-6}	1.05×10^{-5}

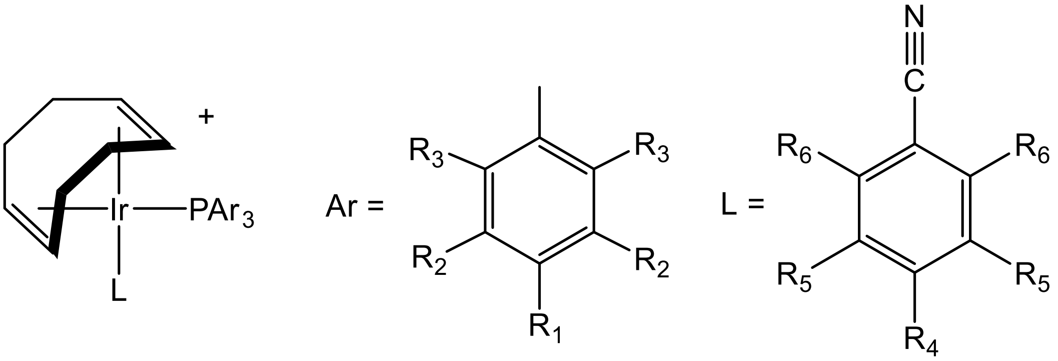

**Label**	**74**	**75**	**76**	**77**
R_1	-F	$-CH_3$	$-CH_3$	-F
R_2	-H	-H	-H	-H
R_3	-H	-H	-H	-H
R_4	-F	$-CH_3$	-F	$-CH_3$
R_5	-H	-H	-H	-H
R_6	-H	-H	-H	-H
$\mathbf{r_0}$ (Ms^{-1})	2.52×10^{-6}	2.20×10^{-6}	2.95×10^{-6}	3.03×10^{-6}

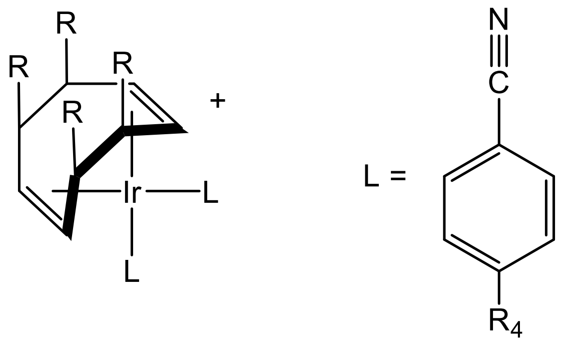

Label	5	78	79	80	81
M	Ir	Ir	Ir	Ir	Ir
R	-H	-H	-H	-F	$-CH_3$
R_4	-H	-F	$-CH_3$	-H	-H
**r_0** (Ms^{-1})	1.83×10^{-5}	1.19×10^{-3}	4.46×10^{-5}	3.26×10^{-6}	1.02×10^{-6}
**Label**	**3**	**82**	**83**	**84**	**85**
M	Rh	Rh	Rh	Rh	Rh
R	-H	-H	-H	-F	$-CH_3$
R_4	-H	-F	$-CH_3$	-H	-H
**r_0** (Ms^{-1})	**2.88×10^{-5}**	1.34×10^{-5}	**5.08×10^{-5}**	3.35×10^{-6}	12.19×10^{-6}

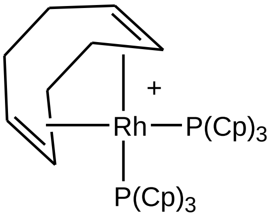

86

r_0 (Ms^{-1}) = 9.9×10^{-7}

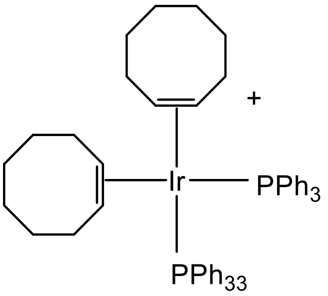

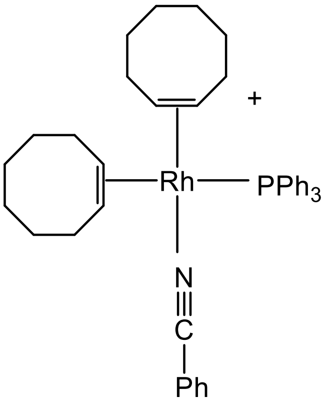

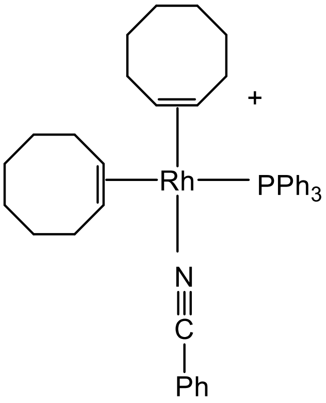

87 **88** **89**

r_0 (Ms^{-1}) = 4.52×10^{-7} **r_0** (Ms^{-1}) = 4.13×10^{-7} **r_0** (Ms^{-1}) = 1.29×10^{-7}

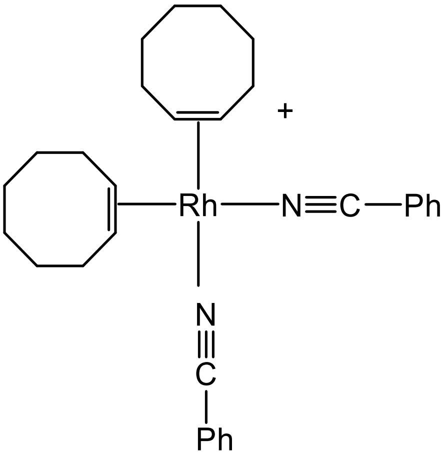

90

$\mathbf{r_0}$ (Ms^{-1}) = 1.82 x 10^{-7}

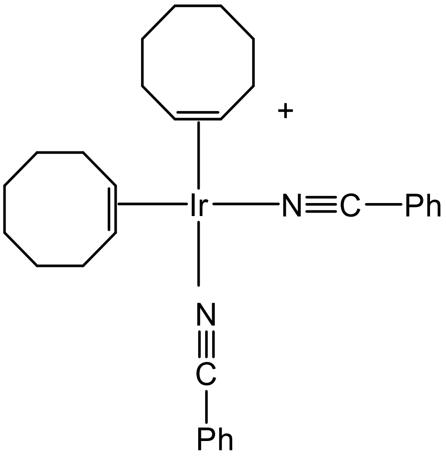

91

$\mathbf{r_0}$ (Ms^{-1}) = 1.84 x 10^{-7}

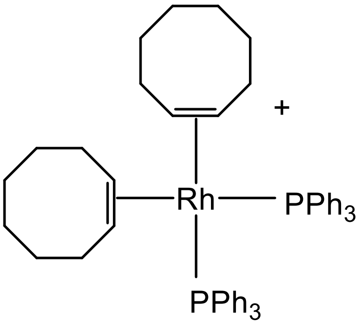

92

$\mathbf{r_0}$ (Ms^{-1}) = 6.6 x 10^{-7}

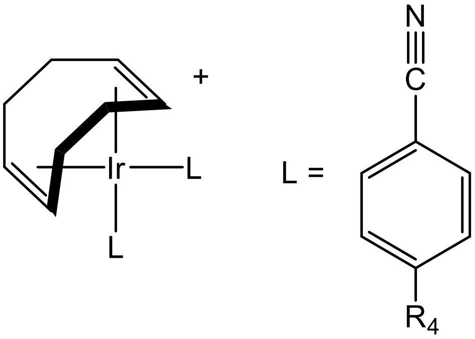

**Label**	**93**	**94**	**95**	**96**
M	Rh	Ir	Rh	Ir
R_4	-^{t}Bu	-^{t}Bu	-CF_3	-CF_3
$\mathbf{r_0}$ (Ms^{-1})	1.87 x 10^{-5}	4.34 x 10^{-5}	1.22 x 10^{-4}	3.20 x 10^{-4}

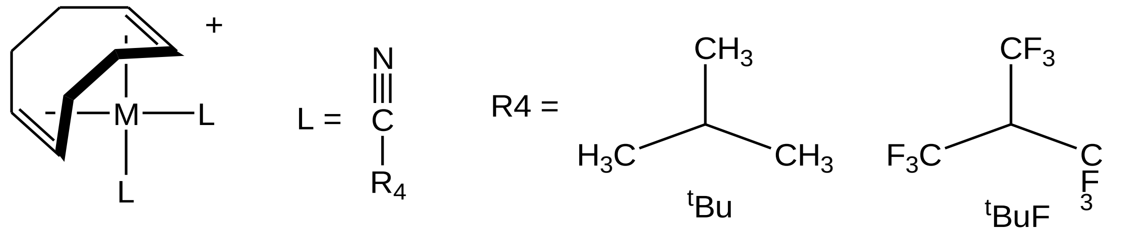

Label	97	98	99	100	101	102
M	Rh	Ir	Rh	Ir	Rh	Ir
R_4	$-CH_3$	$-CH_3$	-^{t}Bu	-^{t}Bu	-^{t}Bu-F	-^{t}Bu-F
**r_0** (Ms^{-1})	3.67×10^{-5}	8.91×10^{-6}	8.98×10^{-6}	7.97×10^{-6}	8.36×10^{-6}	6.54×10^{-6}

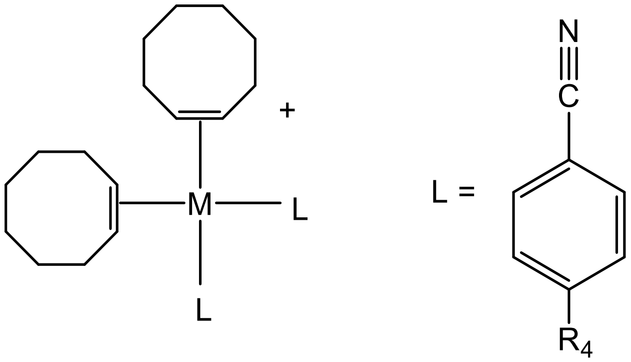

Label	103	104	105	106
M	Rh	Ir	Rh	Ir
R_4	-F	-F	$-CH_3$	$-CH_3$
**r_0** (Ms^{-1})	2.7×10^{-7}	4.1×10^{-7}	2.37×10^{-7}	6.28×10^{-7}
**Label**	**107**	**108**	**109**	**110**
M	Rh	Ir	Rh	Ir
R_4	$-CF_3$	$-CF_3$	-^{t}Bu	-^{t}Bu
**r_0** (Ms^{-1})	6.28×10^{-7}	1.47×10^{-5}	2.39×10^{-6}	4.06×10^{-6}

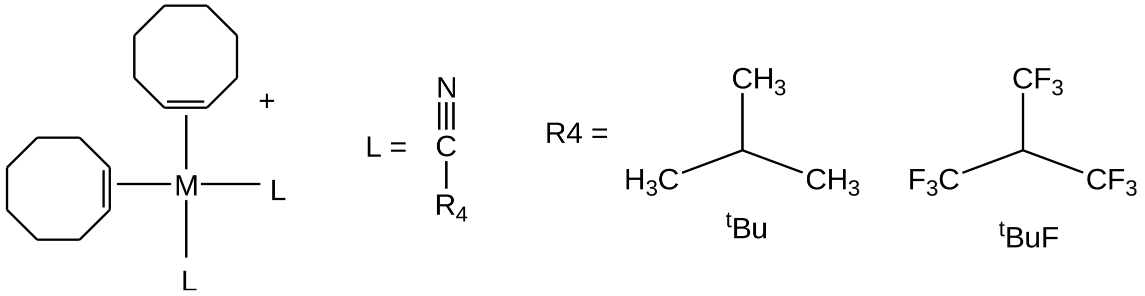

Label	111	112	113	114	115	116
M	Rh	Ir	Rh	Ir	Rh	Ir
R_4	$-CH_3$	$-CH_3$	$-CF_3$	$-CF_3$	-^{t}Bu	-^{t}Bu
**r_0** (Ms^{-1})	2.94×10^{-7}	4.74×10^{-7}	6.41×10^{-7}	3.51×10^{-6}	2.45×10^{-7}	5.44×10^{-7}

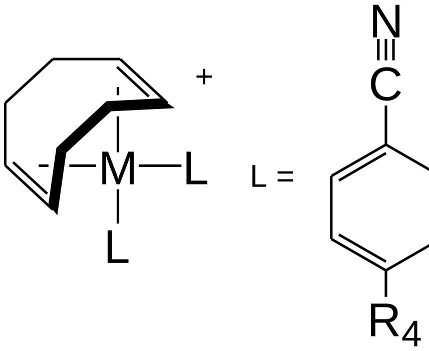

Label	117	118
M	Rh	Ir
R_4	$-NO_2$	$-NO_2$
**r_0** (Ms^{-1})	3.13×10^{-5}	1.5×10^{-4}

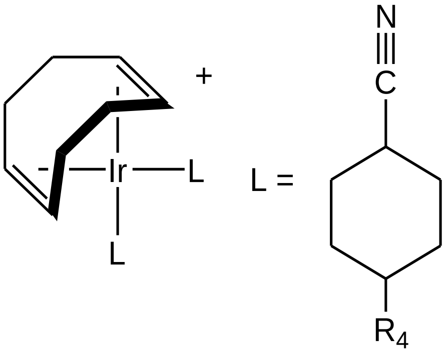

Label	119	120	121	122
M	Rh	Ir	Rh	Ir
R_4	-H	-H	$-CH_3$	$-CH_3$
**r_0** (Ms^{-1})	5.31×10^{-5}	1.12×10^{-4}	9.88×10^{-5}	1.34×10^{-4}

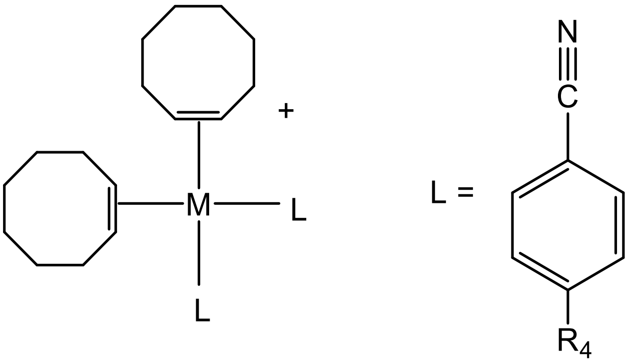

Label	123	124
M	Rh	Ir
R_4	$-NO_2$	$-NO_2$
**r_0** (Ms^{-1})	5.72×10^{-7}	6.65×10^{-6}

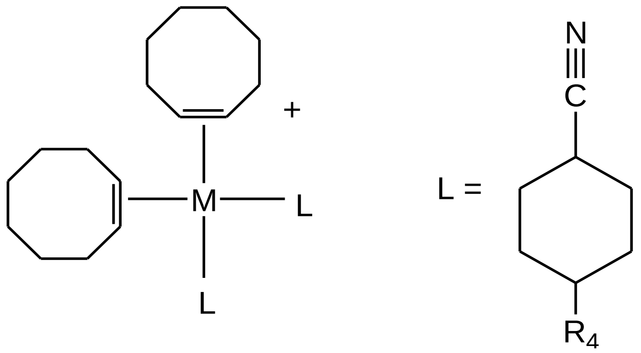

Label	125	126	127	128
M	Rh	Ir	Rh	Ir
R_4	-H	-H	$-CH_3$	$-CH_3$
**r_0** (Ms^{-1})	1.4×10^{-7}	1.75×10^{-7}	1.36×10^{-7}	1.74×10^{-7}

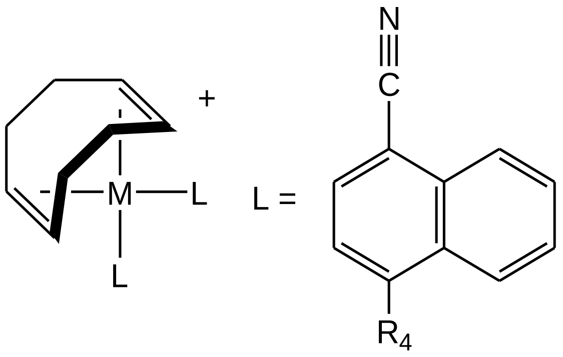

Label	129	130	131	132
M	Rh	Ir	Rh	Ir
R_4	-H	-H	$-CH_3$	$-CH_3$
**r_0** (Ms^{-1})	6.04×10^{-6}	7.93×10^{-6}	4.87×10^{-6}	6.20×10^{-6}

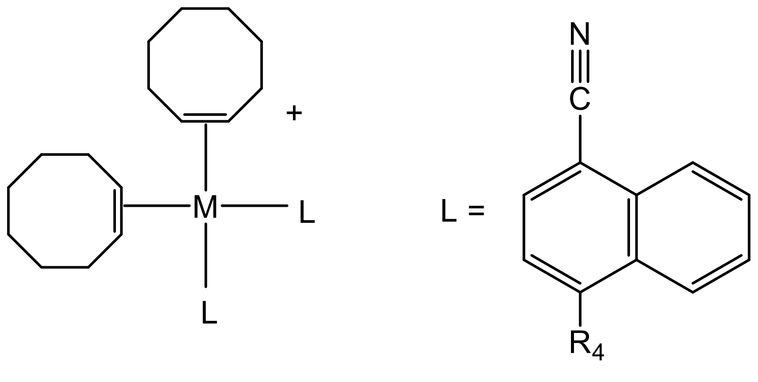

Label	133	134	135	136
M	Rh	Ir	Rh	Ir
R_4	-H	-H	$-CH_3$	$-CH_3$
**r_0** (Ms^{-1})	4.65×10^{-7}	1.34×10^{-6}	4.15×10^{-7}	7.76×10^{-7}

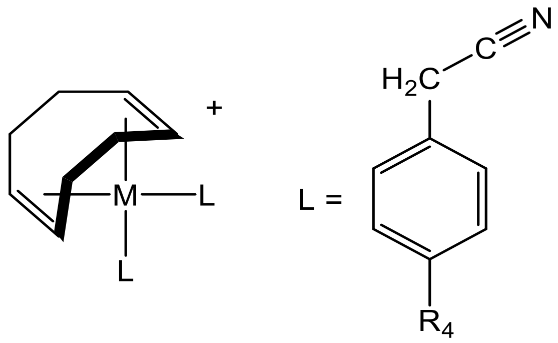

Label	137	138	139	140	141	142
M	Rh	Ir	Rh	Ir	Rh	Ir
R_4	-H	-H	-F	-F	$-CH_3$	$-CH_3$
**r_0** (Ms^{-1})	6.67×10^{-7}	6.93×10^{-6}	3.62×10^{-7}	4.30×10^{-6}	7.6×10^{-7}	9.57×10^{-7}

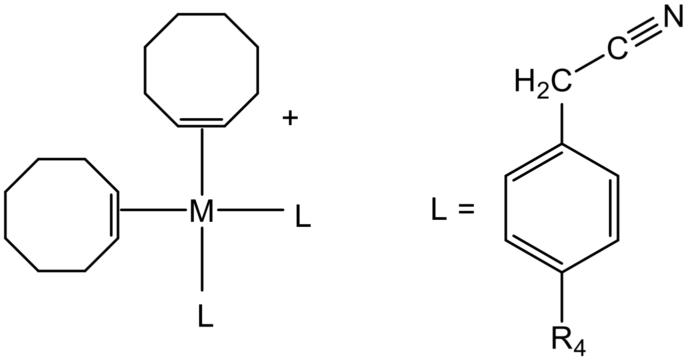

Label	143	144	145	146	147	148
M	Rh	Ir	Rh	Ir	Rh	Ir
R_4	-H	-H	-F	-F	$-CH_3$	$-CH_3$
**r_0** (Ms^{-1})	7.66×10^{-7}	8.18×10^{-7}	5.76×10^{-7}	7.61×10^{-7}	6.28×10^{-7}	5.54×10^{-7}

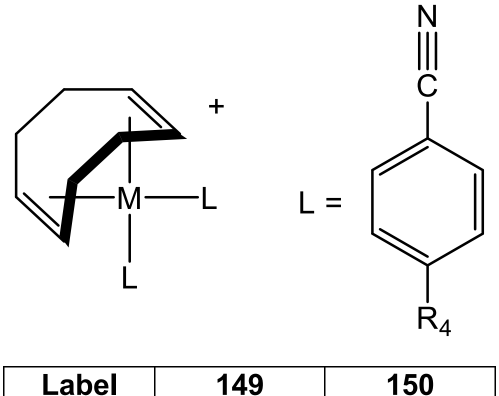

Label	149	150
M	Rh	Ir
R_4	-Et	-Et
**r_0** (Ms^{-1})	2.71×10^{-5}	3.77×10^{-5}

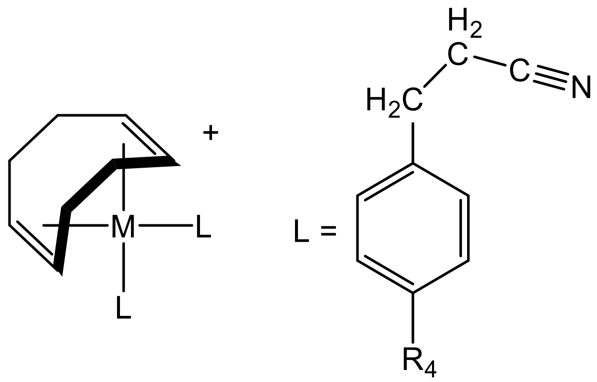

Label	151	152
M	Rh	Ir
R_4	-H	-H
**r_0** (Ms^{-1})	2.89×10^{-6}	2.40×10^{-6}

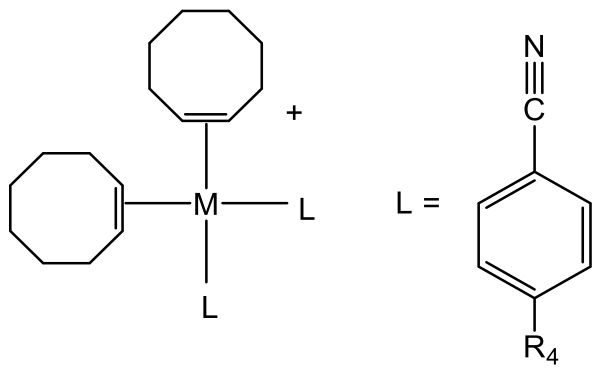

Label	153	154
M	Rh	Ir
R_4	-Et	-Et
**r_0** (Ms^{-1})	7.67×10^{-7}	1.60×10^{-6}

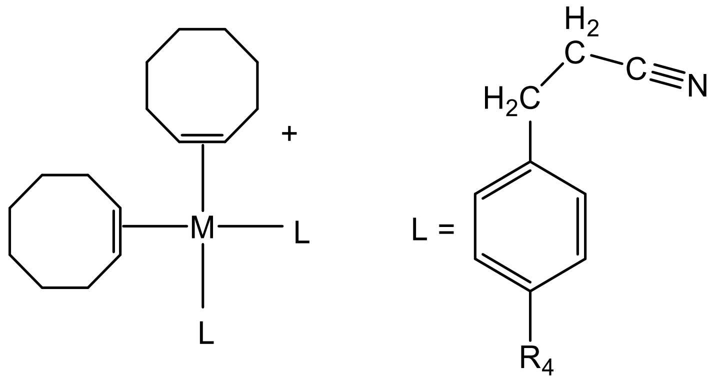

Label	155	156
M	Rh	Ir
R_4	-H	-H
**r_0** (Ms^{-1})	4.93×10^{-7}	9.71×10^{-7}

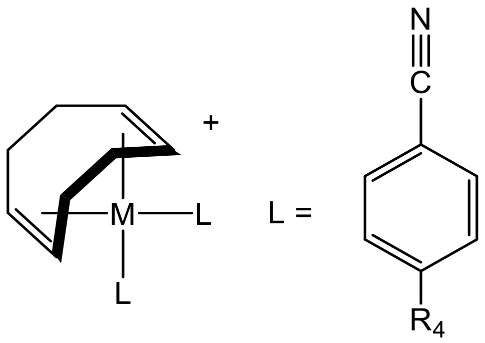

Label	157	158
M	Rh	Ir
R_4	-Pr	-Pr
**r_0** (Ms^{-1})	1.24 x 10^{-4}	3.74x 10^{-5}

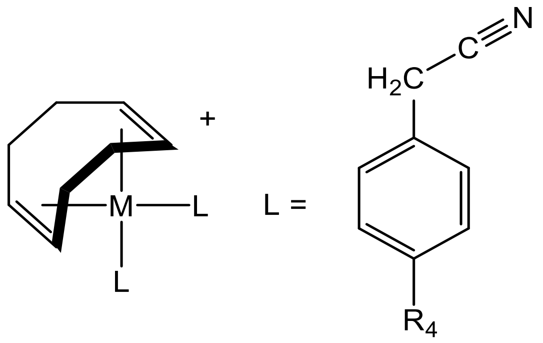

Label	159	160
M	Rh	Ir
R_4	-Et	-Et
**r_0** (Ms^{-1})	2.78 x 10^{-7}	8.31 x 10^{-7}

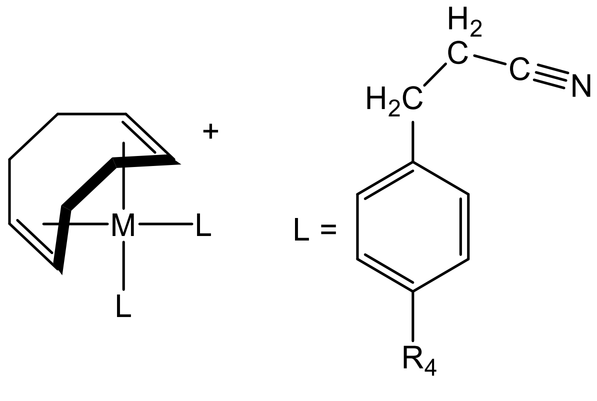

Label	161	162
M	Rh	Ir
R_4	$-CH_3$	$-CH_3$
**r_0** (Ms^{-1})	2.89 x 10^{-6}	1.23 x 10^{-5}

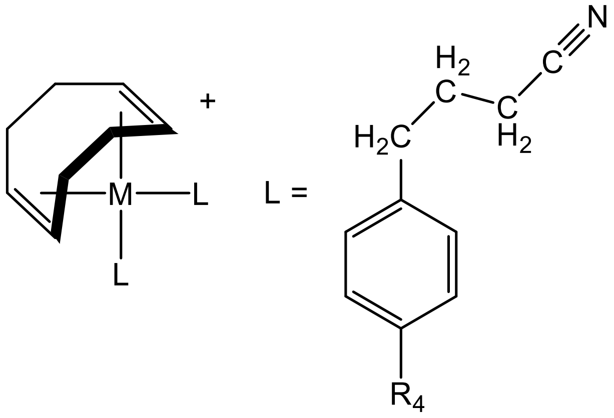

Label	163	164
M	Rh	Ir
R_4	-H	-H
**r_0** (Ms^{-1})	6.75 x 10^{-7}	1.52 x 10^{-6}

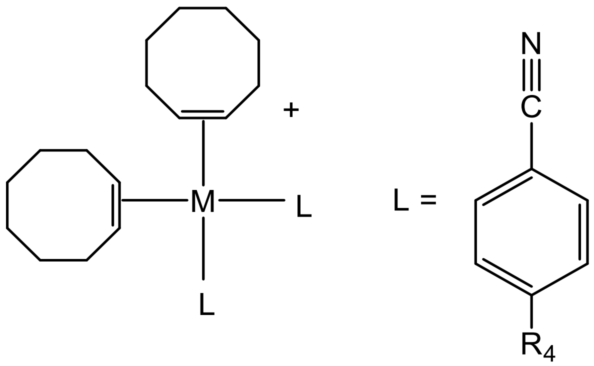

Label	165	166
M	Rh	Ir
R_4	-Pr	-Pr
**r_0** (Ms^{-1})	1.28 x 10^{-6}	1.98x 10^{-6}

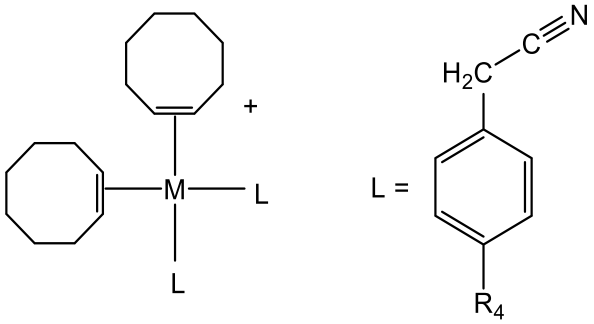

Label	167	168
M	Rh	Ir
R_4	-Et	-Et
**r_0** (Ms^{-1})	8.39 x 10^{-7}	5.02 x 10^{-7}

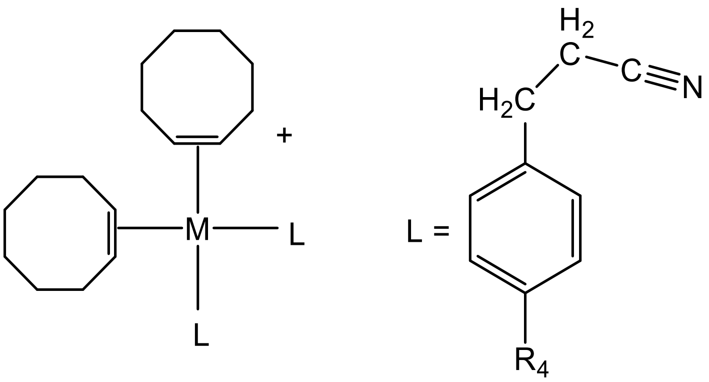

Label	169	170
M	Rh	Ir
R_4	$-CH_3$	$-CH_3$
**r_0** (Ms^{-1})	3.54×10^{-7}	1.27×10^{-6}

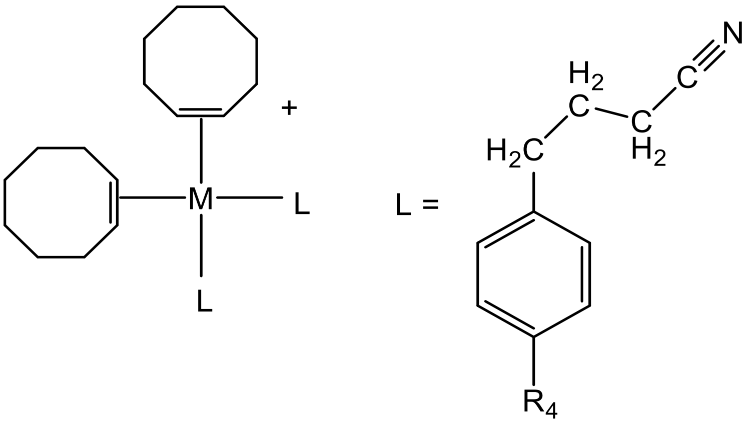

Label	171	172
M	Rh	Ir
R_4	-H	-H
**r_0** (Ms^{-1})	5.23×10^{-7}	8.32×10^{-7}

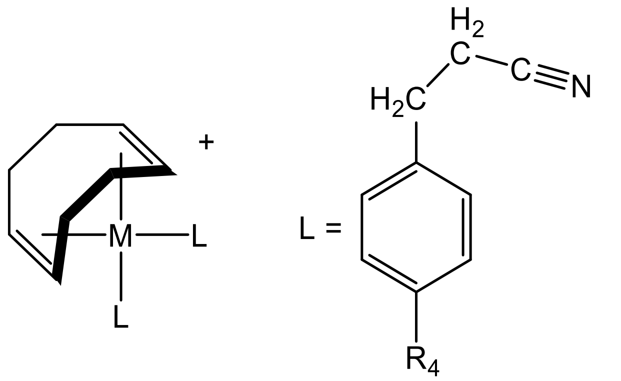

Label	173	174	175	176
M	Rh	Ir	Rh	Ir
R_4	-Et	-Et	-Pr	-Pr
**r_0** (Ms^{-1})	1.73×10^{-6}	5.04×10^{-6}	9.78×10^{-7}	3.59×10^{-6}

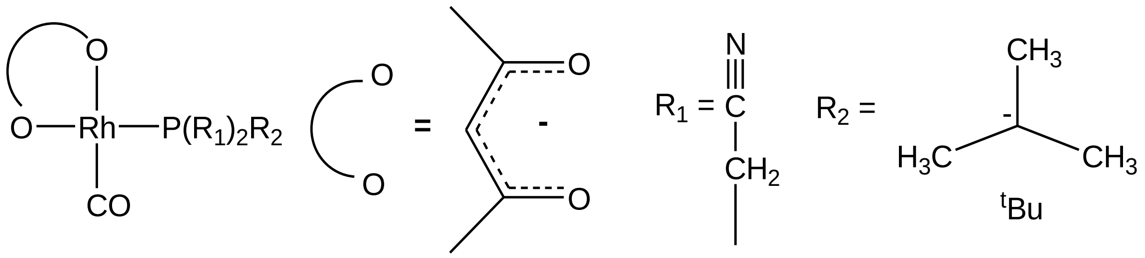

177

$\mathbf{r_0}$ (Ms^{-1}) = 4.75 x 10^{-6}

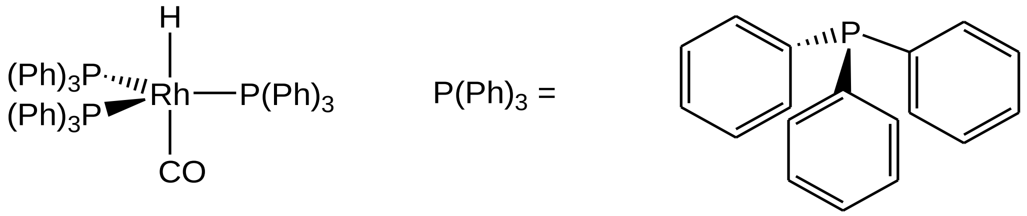

178

$\mathbf{r_0}$ (Ms^{-1}) = 7.99 x 10^{-6}

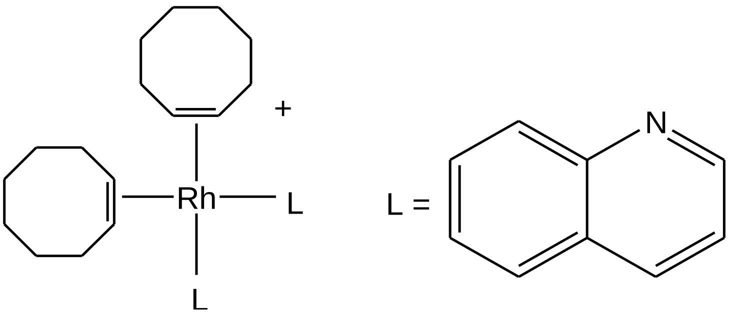

179

$\mathbf{r_0}$ (Ms^{-1}) = 5.29 x 10^{-7}

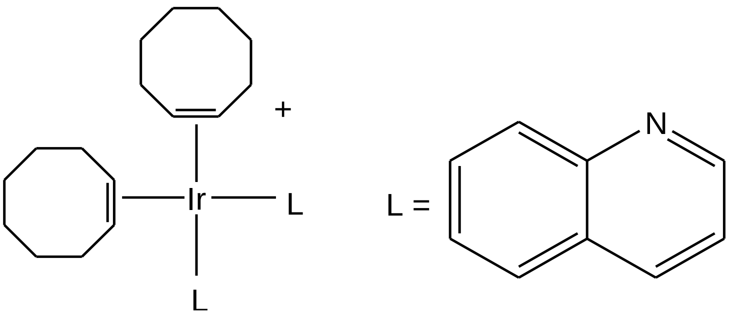

180

$\mathbf{r_0}$ (Ms^{-1}) = 3.21 x 10^{-7}

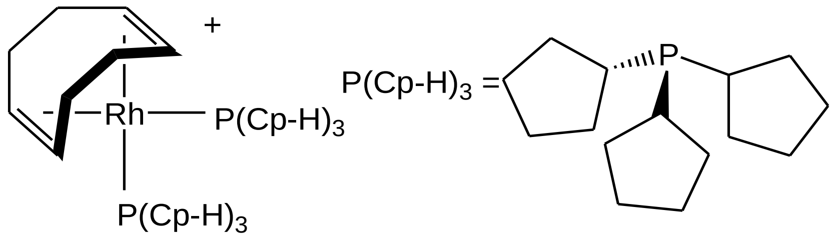

181

$\mathbf{r_0}$ (Ms^{-1}) = 8.80 x 10^{-7}

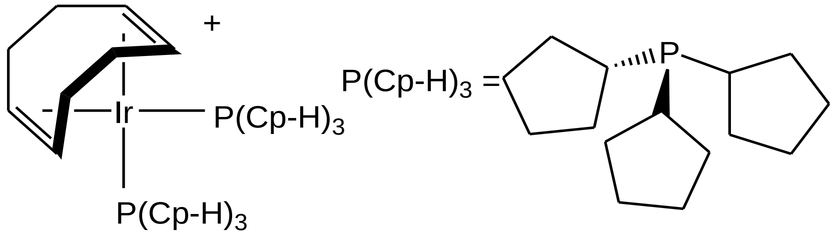

182

$\mathbf{r_0}$ (Ms^{-1}) = 1.34 x 10^{-6}

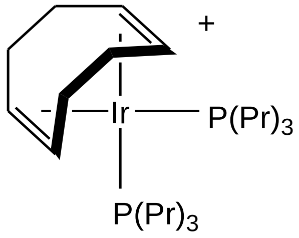

183

Failed to optimize

$\mathbf{r_0}$ (Ms^{-1}) = -

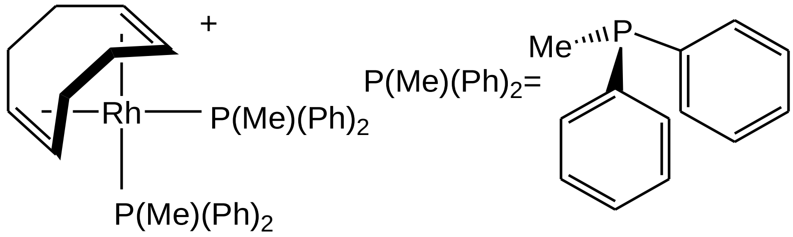

184

Failed to optimize

$\mathbf{r_0}$ (Ms^{-1}) = -

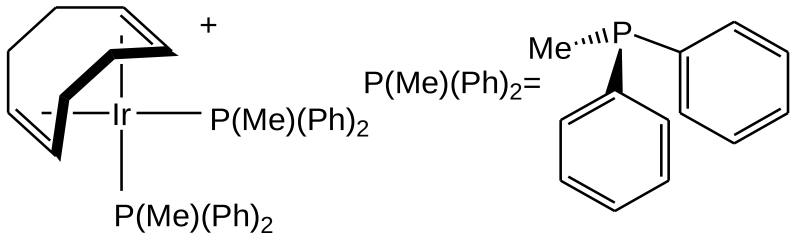

185

$\mathbf{r_0}$ (Ms^{-1}) = 1.86 x 10^{-7}

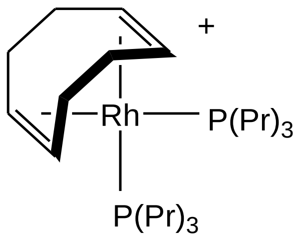

186

Failed to optimize

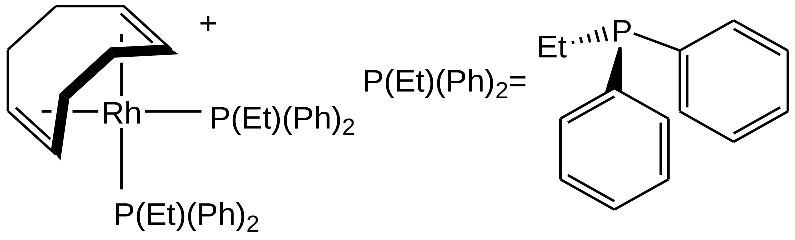

187

$\mathbf{r_0}$ (Ms^{-1}) = 6.81 x 10^{-7}

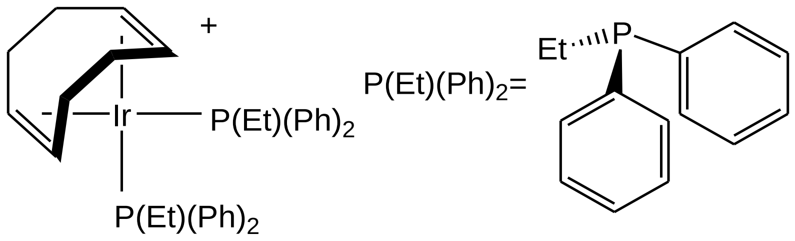

188

$\mathbf{r_0}$ (Ms^{-1}) = 5.99 x 10^{-7}

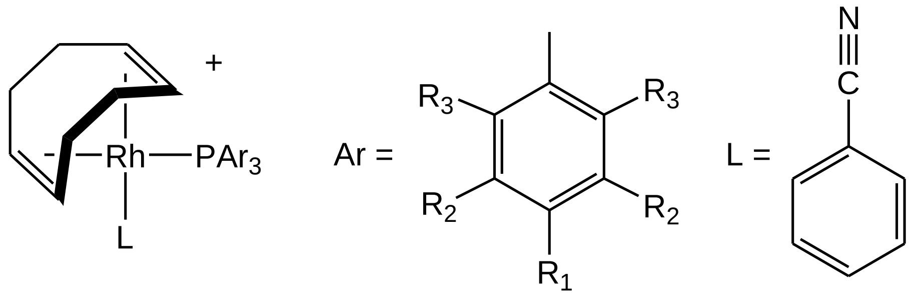

Label	189	190	191
R_1	$-CH_3$	-H	-H
R_2	-H	$-CH_3$	-H
R_3	-H	-H	$-CH_3$
**r_0** (Ms^{-1})	1.57×10^{-6}	5.54×10^{-6}	1.0×10^{-5}

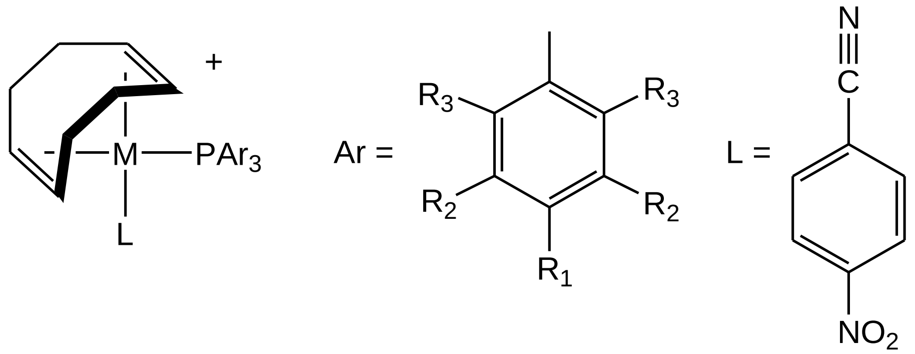

Label	192	194	196	193	195	197
	Rh	Rh	Rh	Ir	Ir	Ir
R_1	$-CH_3$	-H	-H	$-CH_3$	-H	-H
R_2	-H	$-CH_3$	-H	-H	$-CH_3$	-H
R_3	-H	-H	$-CH_3$	-H	-H	$-CH_3$
**r_0** (Ms^{-1})	3.56×10^{-6}	3.45×10^{-5}	4.9×10^{-6}	2.65×10^{-5}	1.63×10^{-2}	4.7×10^{-6}

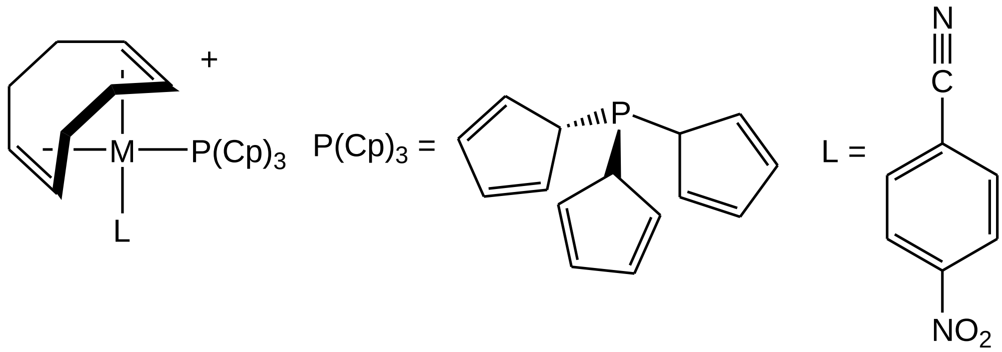

Label	198	199
	Rh	Ir
**r_0** (Ms^{-1})	2.28×10^{-5}	1.28×10^{-5}

SI6. Information of Additional Files

1. Comma-separated values of descriptors and predicted values of initial rate

The file contains several columns with the values of:

Molecules: Catalyst codification

log_r0: logarithm of initial rate

r_0: initial rate

I50_F_AB_nCi_2_M12_SS1_T_LGP[5]_s_MID: First descriptor in the model, called D1 across the manuscript.

GM_B_AB_nCi_2_M15_SS3_T_LGL[1-2]_e-s_MID: Second descriptor in the model, called D2 across the manuscript.

S_B_AB_nCi_2_M3_SS4_T_LGL[2-3]_m-h_MID: Third descriptor in the model, called D3 across the manuscript.

VC_Q_AB_nCi_2_M15_NS4_T_KA_m_MID: Fifth descriptor the model, called D4 across the manuscript.

X(NCA): Neighborhood Components Analysis (NCA) dimension projected on the X axis

Y(NCA): Neighborhood Components Analysis (NCA) dimension projected on the Y axis

Set: Train, test, or prediction set of molecules used for model obtaining and testing, and prediction on new catalyst

2. Molecular dataset in Structured Data Format file.

The file contains the atomic coordinates of optimized structures of catalysts.

SI7. Values of descriptors and predicted values of initial rate (descriptors and NCA projection)

molecules;log_r0;r_0;I50_F_AB_nCi_2_M12_SS1_T_LGP[5]_s_MID;GM_B_AB_nCi_2_M15_SS3_T_LGL[1-2]_e-s_MID;S_B_AB_nCi_2_M3_SS4_T_LGL[2-3]_m-h_MID;VC_Q_AB_nCi_2_M15_NS4_T_KA_m_MID;X(NCA);Y(NCA);Set
mol001;-6.443392327;0.0000003603;0.085022018;0.127483283;4.853080581;3.307375548;-3.85992569;-0.61124448;train
mol002;-5.407885443;0.000003909;0.184536343;0.112702059;4.533217136;4.134701483;1.57590544;1.58584542;train
mol003;-4.91488393;0.00001217;0.14811011;0.508915217;4.057745947;3.45195265;-3.68099105;-9.21992106;train
mol004;-5.395984265;0.000004018;0.180280622;0.103291233;4.411075782;4.181589919;1.98685047;0.50403373;train
mol005;-4.529028771;0.00002958;0.130463343;0.733923181;3.545524565;2.907016656;-3.68055431;-8.12286672;train
mol006;-6.020786293;0.0000009533;0.104087735;0.048762069;4.126337122;3.836417495;1.93957346;-1.71402498;train
mol007;-6.081544075;0.0000008288;0.108093079;0.123920314;5.125753146;5.206618509;0.43629988;7.78725456;train
mol008;-6.169703503;0.0000006765;0.108028671;0.242422907;4.907176252;2.803495153;-4.29258257;-2.36126262;train
mol009;-5.298203809;0.000005033;0.09449303;0.343618647;2.90695336;2.300679395;-1.34623746;-8.19724991;train
mol010;-5.148452873;0.000007105;0.182119263;0.154687386;4.484142191;5.278965016;2.50667579;7.55652384;train
mol011;-5.211889757;0.000006139;0.197900279;0.197051857;4.729085363;4.335469251;0.49833488;3.60173529;train
mol012;-6.023801603;0.0000009467;0.198618341;0.09768826;6.045449602;3.938715744;-3.56894943;3.25544549;train
mol013;-6.183192265;0.0000006559;0.075599259;0.165493671;4.782134465;4.78133702;1.537928;6.15266529;train
mol014;-5.776340289;0.000001674;0.100522329;0.022842475;3.300084744;3.534050954;2.37605147;-3.866874;train
mol015;-6.104471639;0.0000007862;0.067132392;0.073100999;3.992449879;4.499362601;4.87591732;-0.02449237;train
mol016;-6.100302584;0.0000007938;0.076145252;0.092965917;4.188490995;4.410900445;3.60229239;0.38628043;train
mol017;-6.188624473;0.0000006477;0.06279349;0.031343521;3.944095541;4.501729377;4.49304519;-1.20202435;train
mol018;-6.351607738;0.000000445;0.062606579;0.070537442;4.027413954;3.312859704;0.86550218;-2.82460156;train
mol019;-6.311956683;0.0000004876;0.058099666;0.18101368;4.806708741;4.741913208;0.55692039;5.52501021;train
mol020;-6.686361755;0.0000002059;0.06806951;0.091043172;4.921208579;3.205821453;-2.77077398;-0.56211822;train
mol021;-6.447930335;0.0000003565;0.049280706;0.131992884;4.738107593;4.664078166;1.96196129;4.91261392;train
mol022;-6.664151207;0.0000002167;0.069497848;0.095576494;4.794378687;2.87737677;-2.85083894;-1.90165551;train
mol023;-5.753241744;0.000001765;0.140485203;0.162369318;4.664881645;3.982760135;0.10620323;1.72462782;train
mol024;-4.973974531;0.00001062;0.081539184;0.492583404;2.833973008;3.158304703;-2.33233648;-8.63312074;train
mol025;-5.927771878;0.000001181;0.116685086;0.230027567;5.190700727;4.788606654;-0.93627147;6.24942051;train
mol037;-6.245574476;0.0000005681;0.129246463;0.124796569;4.789551963;2.224037404;-3.62161108;-1.09636072;test
mol055;-4.742681577;0.00001808;0.147447029;0.217535906;2.054936802;2.155652944;-1.5696076;-7.39449473;test
mol059;-4.959884841;0.00001097;0.114900783;0.446646907;3.43060179;3.739244517;-3.33001954;-8.38898988;test
mol026;-6.364503354;0.000000432;0.083168567;0.111057438;4.949855719;4.326985473;1.40706333;5.04932968;prediction
mol027;-6.592148369;0.0000002558;0.080510105;0.133870915;5.261916077;3.660200983;3.65885446;0.385306354;prediction
mol028;-6.505663839;0.0000003121;0.083002547;0.086680498;5.019342721;3.910504864;0.037069816;2.31429774;prediction

mol029;-6.600450289;0.0000002509;0.13309214;0.083105606;5.853491384;3.199786981;3.96837896;2.89756994;prediction
mol030;-6.4756657;0.0000003345;0.125728454;0.06179289;6.153476787;5.382745506;0.181136436;8.26431682;prediction
mol031;-6.509493126;0.0000003094;0.127333928;0.055600433;6.158726892;5.174917023;0.465472147;8.1396265;prediction
mol032;-5.191017381;0.000006441;0.166972842;0.207130248;4.402320096;4.97384354;1.89351023;7.56763741;prediction
mol033;-5.588762382;0.000002578;0.166679076;0.140061594;4.745987978;4.180317724;0.474150166;2.76779136;prediction
mol034;-5.255759895;0.000005549;0.175233314;0.206640148;4.809359507;5.290643058;1.49806755;8.01128062;prediction
mol035;-6.045099424;0.0000009014;0.150802612;0.195624168;5.110298181;2.538283684;4.04674824;-1.15790064;prediction
mol036;-5.626294506;0.000002364;0.149292376;0.170456096;5.169790639;5.637663627;0.311521016;8.27869282;prediction
mol038;-5.288328305;0.000005148;0.169969658;0.174897804;4.845645059;5.783507931;1.26775804;8.34373659;prediction
mol039;-5.65909185;0.000002192;0.171273873;0.131146681;5.037461257;4.414353519;1.1938473;5.60325438;prediction
mol040;-5.694878012;0.000002019;0.160595606;0.181417326;5.637433997;5.826305708;0.038951797;8.22073118;prediction
mol041;-6.087515725;0.0000008175;0.161103505;0.080397777;5.457221458;3.949038593;3.74428744;3.79802988;prediction
mol042;-5.940119402;0.000001148;0.161415676;0.0525825;5.831691734;6.170148038;0.175120875;8.3829448;prediction
mol043;-5.879168109;0.000001321;0.141296651;0.207402648;5.621931186;5.309634184;0.43203258;7.7921874;prediction
mol044;-6.007403193;0.0000009831;0.142527338;0.230916319;5.992876622;5.222593213;0.846088547;7.47707786;prediction
mol045;-6.234632916;0.0000005826;0.141093753;0.159303544;6.031650538;4.753047461;3.10389833;5.26734805;prediction
mol046;-6.33427329;0.0000004632;0.173500648;0.146307907;6.614246102;4.287229972;3.74548933;4.10728932;prediction
mol047;-6.777404477;0.000000167;0.156408409;0.097075051;6.860105714;3.576142045;3.8345564;3.90836038;prediction
mol048;-6.786167867;0.0000001636;0.158647987;0.096816146;6.964515178;3.701510985;3.823022;3.942648;prediction
mol049;-5.956407739;0.000001106;0.154599168;0.189748697;5.407754241;3.780622502;3.43032365;2.22824492;prediction
mol050;-5.917697961;0.000001209;0.152266903;0.24280593;5.678849651;4.313941682;1.47192287;6.6576022;prediction
mol051;-5.836208237;0.000001458;0.15409136;0.19508732;5.766941005;5.478782256;0.271030767;7.99732696;prediction
mol052;-5.99341848;0.000001015;0.169664678;0.116875009;6.177068895;5.700181795;0.113828008;8.2518466;prediction
mol053;-6.366313145;0.0000004302;0.134137622;0.105242733;6.462418091;6.034336403;0.094531872;8.29022592;prediction
mol054;-6.863157242;0.000000137;0.135800955;0.084564774;6.547831789;3.329185437;3.86442458;3.79274011;prediction
mol056;-5.831565961;0.000001474;0.073960645;0.115807387;3.500518296;4.052217037;3.27201187;-1.63601575;prediction
mol057;-5.765232199;0.000001717;0.063288628;0.333045751;3.861168308;3.664006978;4.1099935;-8.74472284;prediction
mol058;-5.999478347;0.000001001;0.074044038;0.115913324;3.865928156;4.009268799;2.40271965;-0.38405454;prediction
mol060;-5.479859883;0.000003312;0.168824095;0.11711104;4.471455252;4.240994899;1.52435459;1.81352253;prediction
mol061;-5.570243437;0.00000269;0.168786474;0.062881878;4.485994852;4.299576931;1.81673621;1.59226641;prediction
mol062;-5.61316773;0.000002437;0.180856065;0.132434794;4.515382386;2.784129081;3.37355041;-0.926392689;prediction
mol063;-5.49055958;0.000003232;0.168859316;0.110693986;4.471367614;4.240998462;1.55811957;1.78298691;prediction
mol064;-5.529536726;0.000002954;0.168890199;0.093392591;4.514209169;4.299575949;1.61692039;1.

78125916;prediction
mol065;-5.554273841;0.000002791;0.172004026;0.090033587;4.585811892;4.226189065;1.43136537;1.94167024;prediction
mol066;-5.345424235;0.000004514;0.105462886;0.036960913;2.716208973;4.209968353;2.40237731;-2.86440455;prediction
mol067;-6.048427271;0.0000008945;0.110783012;0.130154722;4.364573108;3.125578424;2.19572406;-1.04793618;prediction
mol068;-5.448155143;0.000003563;0.167796664;0.240657673;4.592501228;3.505678892;1.20023661;1.86281606;prediction
mol069;-5.711696913;0.000001942;0.169300663;0.130421239;4.622988014;3.055563441;3.47232932;-0.491211693;prediction
mol070;-5.519311061;0.000003025;0.184538166;0.109827801;4.761773877;4.101929092;0.779000236;2.37450903;prediction
mol071;-5.692662875;0.000002029;0.180543642;0.073610704;4.738881253;3.545768515;3.5546861;0.273088489;prediction
mol072;-5.409987518;0.000003891;0.202375704;0.068034697;4.747206271;4.311285159;1.39945303;2.27375823;prediction
mol073;-4.978239087;0.00001051;0.227995491;0.047472864;4.227665757;4.526253064;3.87506863;0.641462865;prediction
mol074;-5.597495776;0.000002526;0.164262179;0.155686254;4.640355154;3.788433807;0.046864294;2.34352876;prediction
mol075;-5.656514317;0.000002205;0.1657011;0.137276328;4.65676204;3.593245092;2.19916988;1.26069721;prediction
mol076;-5.530184649;0.00000295;0.164389337;0.170521959;4.648420963;4.063553679;0.262656187;2.69395915;prediction
mol077;-5.517873634;0.000003035;0.166536339;0.166891853;4.681845544;4.165119455;0.284631231;3.03032797;prediction
mol078;-2.924174347;0.001191;0.127305614;1.685824291;3.66184861;3.198324283;3.91908732;-7.43884294;prediction
mol079;-4.349926584;0.00004468;0.128846684;0.819034041;3.658265894;3.49971465;3.84615288;-7.64165821;prediction
mol080;-5.486681128;0.000003261;0.086578904;0.389938944;3.643710214;3.048760761;3.27973847;-8.44093984;prediction
mol081;-5.987805657;0.000001028;0.077699131;0.152366786;3.614197113;2.824438877;0.725705718;-2.36288433;prediction
mol082;-4.870575701;0.00001347;0.145527173;0.595023614;4.203793763;3.337601401;4.00810659;-8.25959269;prediction
mol083;-4.293477438;0.00005088;0.142385222;0.912460019;4.170452971;3.598712627;3.97738421;-7.61912844;prediction
mol084;-5.474013378;0.000003357;0.092955202;0.468792202;4.025353565;3.023698566;3.93526301;-8.52033005;prediction
mol085;-5.659205195;0.000002192;0.091879799;0.359811148;4.262652202;3.739188107;0.944642308;1.05998547;prediction
mol086;-6.003860332;0.0000009912;0.059570124;0.073333778;3.61638318;4.458338985;4.25166905;0.170358906;prediction
mol087;-6.344817542;0.000000452;0.087950027;0.045420965;4.684374784;4.184843573;1.43708818;1.9388608;prediction
mol088;-6.3832736;0.0000004137;0.081958406;0.068609034;4.519912216;3.541822977;1.78088334;-0.025223653;prediction
mol089;-5.888668042;0.000001292;0.095228089;0.099352246;4.15663136;4.633292192;3.90909287;0.849938565;prediction
mol090;-6.73959101;0.0000001821;0.041771048;0.047345959;4.688495379;3.988711735;1.28399316;1.93584644;prediction
mol091;-6.7346233;0.0000001842;0.047289685;0.023627371;3.882247656;1.789866316;2.001365;-1.71010289;prediction
mol092;-6.180376637;0.0000006601;0.095897923;0.018514622;3.950546238;3.087739948;0.676527666;-2.06949529;prediction
mol093;-4.727677946;0.00001872;0.131576572;0.616609193;3.487173253;2.709588672;3.50811202;-7.71959606;prediction
mol094;-4.361861247;0.00004346;0.129484811;0.810211357;3.209283675;2.256607349;3.61819287;-7.5068464;prediction
mol095;-3.911404943;0.0001226;0.131086329;0.772278118;2.458345218;3.312200344;3.30066466;-7.63740516;prediction
mol096;-3.493628181;0.0003209;0.129453045;1.186173449;2.340341194;1.254238957;3.61526393;-7.42556202;prediction

mol097;-4.434848159;0.00003674;0.110048387;0.821623116;3.210850723;2.638050017;3.6525075;-7.5
35072;prediction
mol099;-5.046292091;0.000008989;0.152787235;0.360401822;3.220773824;1.692452911;1.66485192;-7
.43126534;prediction
mol100;-5.098275095;0.000007975;0.147957993;0.332111506;2.931633772;1.114258536;1.6153108;-7.
40198298;prediction
mol101;-5.077598245;0.000008364;0.143384937;0.120869625;3.166465409;4.357144774;4.15780127;-0
.150467876;prediction
mol102;-5.183903409;0.000006548;0.13987245;0.127831934;3.370969453;4.357375879;4.31586454;0.1
98804546;prediction
mol103;-6.568420376;0.0000002701;0.061051851;0.078836311;4.7825685;4.029223095;0.979261213;2.
20284056;prediction
mol104;-6.387095282;0.0000004101;0.054440343;0.05969566;3.80744897;3.004276661;0.742639087;-2
.18783179;prediction
mol105;-6.624889082;0.0000002372;0.060925797;0.060806913;4.802871446;3.933020318;0.958662888;
2.1398394;prediction
mol106;-6.201854072;0.0000006283;0.054562077;0.073620012;3.814048111;4.015935829;2.51836761;-
0.638648482;prediction
mol107;-6.201854072;0.0000006283;0.054562077;0.073620012;3.814048111;4.015935829;2.51836761;-
0.638648482;prediction
mol108;-4.832683043;0.0000147;0.12191772;0.069310079;2.039300891;4.380468683;2.31403521;-3.02
572901;prediction
mol109;-5.627751131;0.000002356;0.129190341;0.052824455;3.583123826;3.509986175;1.67565292;-2
.57492005;prediction
mol110;-5.390581894;0.000004068;0.121769223;0.073108285;2.827069381;3.052288265;1.93127131;-3
.12909099;prediction
mol111;-6.530955719;0.0000002945;0.03046542;0.09126317;3.92267002;3.266063167;0.69997959;-1.9
998172;prediction
mol112;-6.32419271;0.000000474;0.02517219;0.062070101;3.265097047;3.312349952;1.87756941;-2.8
8206564;prediction
mol113;-6.192476969;0.000000642;0.045856361;0.047447859;2.985242337;2.504371419;1.65167658;-3
.21442596;prediction
mol114;-5.454687994;0.00000351;0.050441993;0.019504422;1.736592694;3.722353622;2.12762225;-3.
19215984;prediction
mol115;-6.61028283;0.0000002453;0.040091535;0.030318948;3.98499363;3.122980767;0.640991371;-2
.03222397;prediction
mol116;-6.263612862;0.000000545;0.041248681;0.083478972;3.520052702;3.373074486;1.59480732;-2
.63652558;prediction
mol117;-4.503186958;0.00003139;0.145892876;0.886862163;4.218867881;2.533038417;3.94150401;-7.
52770239;prediction
mol118;-3.818722641;0.0001518;0.138140927;1.069714136;3.731563226;3.870815681;3.91946107;-7.5
3754106;prediction
mol119;-4.274853526;0.00005311;0.140300861;1.038917794;4.072204865;2.201451043;3.90930831;-7.
46339406;prediction
mol120;-3.946986638;0.000113;0.127034585;1.046816995;3.568006062;3.414251673;3.86910888;-7.52
034698;prediction
mol121;-4.004993901;0.00009886;0.133760662;1.131035247;4.194782014;3.549698081;3.97087161;-7.
51636249;prediction
mol122;-3.87252481;0.0001341;0.123979885;1.124107745;3.697584696;3.558388007;3.90589451;-7.50
979997;prediction
mol123;-6.242165226;0.0000005726;0.123682178;0.04056907;4.865855339;3.622536582;3.35172397;0.
306608359;prediction
mol124;-5.575767018;0.000002656;0.131716978;0.040755751;3.692930003;4.138078102;4.04897669;-0
.168978903;prediction
mol125;-6.917802816;0.0000001208;0.042014127;0.041508594;4.662391925;2.863230726;3.16415452;-
0.674596408;prediction
mol126;-6.47451617;0.0000003353;0.042739565;0.061788007;3.91884382;3.318364427;0.797415657;-1
.97260763;prediction
mol127;-6.738664744;0.0000001825;0.061263717;0.035456141;4.756852806;3.355010342;3.30898117;-
0.004533705;prediction
mol128;-6.342772655;0.0000004542;0.053086587;0.078375684;4.013683628;3.712206665;1.41483808;-
1.14326783;prediction
mol129;-5.218849163;0.000006042;0.11275771;0.627508388;4.662066289;3.694585436;4.12135986;-8.
44207064;prediction
mol130;-5.100420102;0.000007936;0.111465895;0.699081359;4.110350502;2.216543526;3.88713057;-7

.59203824;prediction
mol131;-5.312354936;0.000004871;0.110040153;0.691849539;4.802138992;2.948159579;4.12341769;-7.99313381;prediction
mol132;-5.207121958;0.000006207;0.109935046;0.561452396;4.244302804;3.461748477;4.03343667;-8.42845276;prediction
mol133;-6.332018948;0.0000004656;0.118982425;0.048239082;5.23491605;4.226700194;1.6518145;4.90165102;prediction
mol134;-5.871337854;0.000001345;0.117786537;0.047330919;4.13029998;4.118330556;2.41511506;-0.213825706;prediction
mol135;-6.381257968;0.0000004157;0.117252995;0.063820554;5.35644581;4.173935802;3.47442968;4.04013556;prediction
mol136;-6.109975147;0.0000007763;0.119205035;0.054756522;4.258057128;2.842058546;1.36953104;-1.59473923;prediction
mol137;-6.175673819;0.0000006673;0.049606553;0.354085943;4.118233418;2.271932658;4.6028612;-2.43358395;prediction
mol138;-5.158668965;0.00000694;0.051232243;0.679089262;3.595036905;3.600748872;3.75807589;-7.84885347;prediction
mol139;-6.440416232;0.0000003627;0.043154298;0.106767062;4.176914649;3.738949518;1.48034878;-0.770319871;prediction
mol140;-5.360537521;0.00000436;0.040966221;0.664501903;3.727477927;3.370780077;3.79981894;-7.85501515;prediction
mol141;-6.117495537;0.000000763;0.044415838;0.347479968;4.271599868;3.377283319;4.31071843;-8.74577742;prediction
mol142;-6.018853245;0.0000009575;0.043360325;0.424942725;4.411975509;3.601229527;4.1919458;-8.73989995;prediction
mol143;-6.115308358;0.0000007668;0.106155251;0.132945016;4.806852492;4.121321668;0.392588711;2.67136836;prediction
mol144;-6.086855888;0.0000008187;0.098787607;0.053875236;4.016712386;3.331201634;0.742105767;-1.8934418;prediction
mol145;-6.238982598;0.0000005768;0.10355127;0.066673929;4.801059987;4.17148855;1.21307198;2.22271074;prediction
mol146;-6.118070117;0.000000762;0.096143551;0.06073988;4.065882501;3.329036069;0.692060506;-1.87390527;prediction
mol147;-6.201583496;0.0000006287;0.103669264;0.106193846;4.889432896;4.219520431;0.777483548;2.73753737;prediction
mol148;-6.255772789;0.0000005549;0.097786138;0.040099792;4.040126072;2.544463159;0.264491439;-2.00437163;prediction
mol149;-4.565982147;0.00002717;0.13620634;0.884465568;4.262731646;2.763637635;3.95907497;-7.55359006;prediction
mol150;-4.422666789;0.00003779;0.137142878;0.81384859;3.809865724;3.114353709;3.86805934;-7.60931903;prediction
mol151;-5.538161148;0.000002896;0.049191464;0.649476524;4.340321496;3.709210438;4.05785733;-8.25444537;prediction
mol152;-5.619573842;0.000002401;0.045829519;0.561025621;3.835616404;2.925400402;3.75870021;-8.06899635;prediction
mol153;-6.114656519;0.000000768;0.123371073;0.067847441;4.883975908;4.187272511;1.13228057;2.38037266;prediction
mol154;-5.794381856;0.000001606;0.127709311;0.037603192;4.140432082;4.24100347;3.72533329;0.424655144;prediction
mol155;-6.306297852;0.000000494;0.091411804;0.107489115;4.91986285;4.240048228;0.751949516;2.9845984;prediction
mol156;-6.012519651;0.0000009716;0.093178488;0.122600285;4.145972501;3.689737923;1.31614259;-0.975965505;prediction
mol157;-3.907542605;0.0001237;0.125461032;1.263975734;4.459198175;3.870827023;4.00330978;-7.50017984;prediction
mol158;-4.427066637;0.00003741;0.120142947;0.935494371;3.954422063;3.021343617;3.91201849;-7.5398947;prediction
mol159;-6.554531834;0.0000002789;0.044790165;0.293652943;4.48760577;1.816573179;4.35255607;-1.61437213;prediction
mol160;-6.080227319;0.0000008313;0.040850102;0.267177389;3.96892518;3.806306114;0.087263288;2.24340317;prediction
mol161;-5.538270686;0.000002896;0.052682136;0.72757796;4.438927652;2.977609286;4.02998623;-7.78826417;prediction
mol162;-4.909094777;0.00001233;0.051253262;0.897745041;3.977566688;3.869679497;3.95672706;-7.6423696;prediction

```
mol163;-6.170662772;0.0000006751;0.039384341;0.347601941;4.482271803;3.870693531;0.075469966;
3.42532339;prediction
mol164;-5.817876106;0.000001521;0.039724745;0.416718603;3.959220971;3.869484399;4.06793815;-8
.72260467;prediction
mol165;-5.891153117;0.000001285;0.134892714;0.139075134;4.92283823;4.357389319;0.788895182;4.
85638564;prediction
mol166;-5.70308201;0.000001981;0.137677059;0.062839848;4.226917241;4.287924475;3.18605253;0.9
95332416;prediction
mol167;-6.075819931;0.0000008398;0.100646289;0.102793332;4.588584138;4.357298314;1.49953132;2
.04330961;prediction
mol168;-6.298849801;0.0000005025;0.092973665;0.060871327;4.226602053;2.803653597;1.19311537;-
1.67749724;prediction
mol169;-6.450335491;0.0000003545;0.092704924;0.065491757;4.64430747;2.975153447;3.21548827;-0
.583439323;prediction
mol170;-5.895771103;0.000001271;0.089902387;0.166279638;4.236621571;4.357377088;2.6441156;1.3
6310689;prediction
mol171;-6.280892454;0.0000005237;0.092275015;0.043021372;4.469081256;3.804184307;1.46626709;0
.018560956;prediction
mol172;-6.079471849;0.0000008328;0.085076214;0.105019942;4.21099548;4.036642841;1.89812282;1.
04193161;prediction
mol173;-5.930595083;0.000001173;0.050252546;0.544484331;4.611133121;3.085801944;4.13110074;-8
.48978866;prediction
mol174;-5.297516946;0.000005041;0.048852611;0.719294354;4.102408533;3.821304317;3.99488308;-7
.96120562;prediction
mol175;-6.009296204;0.0000009788;0.047658619;0.445242462;4.734948417;4.120367352;0.934763607;
4.14937934;prediction
mol176;-5.444284082;0.000003595;0.045072279;0.655603631;4.222325787;4.103387418;4.06024088;-8
.3327075;prediction
mol177;-5.392301633;0.000004052;0.083900826;0.241042558;2.845124188;3.15722037;-1.37546734;-7
.16192569;test
mol178;-5.097231775;0.000007994;0.165330507;0.132992368;4.030048542;5.402414369;2.36422932;8.
19221664;test
mol179;-6.276334466;0.0000005293;0.078225413;0.117640161;4.608674695;4.108939362;1.05066885;2
.15469389;test
mol180;-6.492620589;0.0000003216;0.060499084;0.0362704;4.513343412;4.239975066;1.75947493;1.5
5767794;test
mol181;-6.055296491;0.0000008804;0.074639829;0.063009017;3.503693568;3.203225518;1.53883102;-
2.6698703;prediction
mol182;-5.870257813;0.000001348;0.067188967;0.170491192;3.823787847;4.449000755;4.05892863;0.
788983745;prediction
mol185;-5.729015019;0.000001866;0.111594909;0.267245596;4.489216587;3.948519529;0.040315887;3
.10268206;prediction
mol186;-6.171588878;0.0000006736;0.09333854;0.222355339;4.682883069;3.108442569;4.25720745;-0
.97530749;prediction
mol187;-6.166442884;0.0000006816;0.085973034;0.082036021;4.437796961;4.320672729;1.93129423;1
.54618165;prediction
mol188;-6.222075842;0.0000005997;0.091397082;0.104519925;4.558127337;3.801691302;0.777608881;
2.03529548;prediction
mol189;-5.588730887;0.000002578;0.178447296;0.086422285;4.621703537;3.834440883;0.915961209;2
.02875743;prediction
mol190;-5.255910516;0.000005547;0.187612655;0.198556944;4.719049618;4.522406789;0.531384635;5
.35795404;prediction
mol191;-4.998251628;0.00001004;0.242944773;0.090806117;4.224934271;3.202655984;0.302476188;-1
.9311594;prediction
mol192;-5.447427818;0.000003569;0.178942172;0.113291591;4.664269777;4.516631669;1.63394884;5.
4986216;prediction
mol193;-5.575821293;0.000002656;0.162979109;0.135416432;4.735769756;4.462840684;1.40900262;5.
46200228;prediction
mol194;-5.461037479;0.000003459;0.185761017;0.183828567;4.722503632;3.508298574;3.90514175;0.
375679595;prediction
mol195;-5.787620731;0.000001631;0.177711514;0.092145149;4.884468513;3.298295258;3.47210378;-0
.032502241;prediction
mol196;-5.309298831;0.000004906;0.184722625;0.092133098;3.730677959;2.753342491;0.642651986;-
2.25783343;prediction
mol197;-5.325859782;0.000004722;0.182949042;0.078606492;4.175708597;4.10232448;2.29785627;0.6
```

55981167;prediction
mol198;-4.640503288;0.00002288;0.2330635;0.27344397;4.148159566;3.749082918;0.018842597;2.449
25704;prediction
mol199;-4.890079997;0.00001288;0.222236919;0.244175507;4.098833784;2.908439416;4.07218605;-1.
68118647;prediction

SI8. Optimized structures in cvs format

```
mol001
Optimized Catalyst 001
Optimized Catalyst 001
 89 98  0  0  0  0  0  0  0  0999 V2000
    0.7913   -0.7044    0.1073 C   0  0  0  0  0  0  0  0  0  0  0  0
   -0.3292    0.0471    0.8609 C   0  0  0  0  0  0  0  0  0  0  0  0
    0.2639   -1.8989   -0.6382 C   0  0  0  0  0  0  0  0  0  0  0  0
   -0.3025   -0.6569   -2.7872 C   0  0  0  0  0  0  0  0  0  0  0  0
   -0.2135   -1.8714   -1.8987 C   0  0  0  0  0  0  0  0  0  0  0  0
   -0.9703    0.5794   -2.1399 C   0  0  0  0  0  0  0  0  0  0  0  0
    0.3186   -2.8423   -0.0811 H   0  0  0  0  0  0  0  0  0  0  0  0
   -0.5388   -2.8067   -2.3878 H   0  0  0  0  0  0  0  0  0  0  0  0
    1.5604   -1.0384    0.8479 H   0  0  0  0  0  0  0  0  0  0  0  0
    1.2971   -0.0109   -0.6098 H   0  0  0  0  0  0  0  0  0  0  0  0
   -0.4039   -0.3801    1.8919 H   0  0  0  0  0  0  0  0  0  0  0  0
   -1.4775    1.1714   -2.9421 H   0  0  0  0  0  0  0  0  0  0  0  0
   -0.8782   -0.9263   -3.7073 H   0  0  0  0  0  0  0  0  0  0  0  0
    0.7384   -0.3805   -3.1007 H   0  0  0  0  0  0  0  0  0  0  0  0
   -1.9748    0.2090   -1.0834 C   0  0  0  0  0  0  0  0  0  0  0  0
   -1.6859   -0.0233    0.2120 C   0  0  0  0  0  0  0  0  0  0  0  0
   -3.0166    0.1773   -1.4292 H   0  0  0  0  0  0  0  0  0  0  0  0
   -2.5009   -0.2555    0.9144 H   0  0  0  0  0  0  0  0  0  0  0  0
   -0.0427    1.1275    0.9513 H   0  0  0  0  0  0  0  0  0  0  0  0
   -0.1868    1.2361   -1.6851 H   0  0  0  0  0  0  0  0  0  0  0  0
   -2.4996   -2.4874   -0.7390 Rh  0  0  0  0  0  0  0  0  0  0  0  0
   -4.7165   -2.4183   -0.6521 P   0  0  0  0  0  0  0  0  0  0  0  0
   -2.3915   -4.6369   -0.2685 P   0  0  0  0  0  0  0  0  0  0  0  0
   -0.6373   -5.2180   -0.1597 C   0  0  0  0  0  0  0  0  0  0  0  0
    0.0221   -5.5636   -1.3463 C   0  0  0  0  0  0  0  0  0  0  0  0
   -0.0216   -5.3958    1.0833 C   0  0  0  0  0  0  0  0  0  0  0  0
    1.3162   -6.0885   -1.2830 C   0  0  0  0  0  0  0  0  0  0  0  0
   -0.4473   -5.4374   -2.3264 H   0  0  0  0  0  0  0  0  0  0  0  0
    1.2738   -5.9226    1.1356 C   0  0  0  0  0  0  0  0  0  0  0  0
   -0.5195   -5.1417    2.0229 H   0  0  0  0  0  0  0  0  0  0  0  0
    1.9421   -6.2681   -0.0437 C   0  0  0  0  0  0  0  0  0  0  0  0
    1.8398   -6.3603   -2.2021 H   0  0  0  0  0  0  0  0  0  0  0  0
    1.7633   -6.0666    2.1012 H   0  0  0  0  0  0  0  0  0  0  0  0
    2.9540   -6.6796    0.0020 H   0  0  0  0  0  0  0  0  0  0  0  0
   -3.1211   -5.7236   -1.5726 C   0  0  0  0  0  0  0  0  0  0  0  0
   -3.3303   -7.0861   -1.3521 C   0  0  0  0  0  0  0  0  0  0  0  0
   -3.3596   -5.1500   -2.8264 C   0  0  0  0  0  0  0  0  0  0  0  0
   -3.8682   -7.8673   -2.3827 C   0  0  0  0  0  0  0  0  0  0  0  0
   -3.0638   -7.5740   -0.4117 H   0  0  0  0  0  0  0  0  0  0  0  0
   -3.8887   -5.9381   -3.8527 C   0  0  0  0  0  0  0  0  0  0  0  0
   -3.1275   -4.0931   -3.0271 H   0  0  0  0  0  0  0  0  0  0  0  0
   -4.1560   -7.2937   -3.6251 C   0  0  0  0  0  0  0  0  0  0  0  0
   -4.0506   -8.9317   -2.2186 H   0  0  0  0  0  0  0  0  0  0  0  0
   -4.0872   -5.4986   -4.8323 H   0  0  0  0  0  0  0  0  0  0  0  0
   -4.5766   -7.9081   -4.4258 H   0  0  0  0  0  0  0  0  0  0  0  0
   -3.1211   -4.8659    1.4141 C   0  0  0  0  0  0  0  0  0  0  0  0
   -3.9330   -5.9307    1.7935 C   0  0  0  0  0  0  0  0  0  0  0  0
   -2.7600   -3.8647    2.3300 C   0  0  0  0  0  0  0  0  0  0  0  0
   -4.3642   -6.0168    3.1266 C   0  0  0  0  0  0  0  0  0  0  0  0
   -4.2754   -6.6888    1.0872 H   0  0  0  0  0  0  0  0  0  0  0  0
   -3.1934   -3.9584    3.6528 C   0  0  0  0  0  0  0  0  0  0  0  0
   -2.1450   -3.0009    2.0167 H   0  0  0  0  0  0  0  0  0  0  0  0
   -3.9902   -5.0417    4.0526 C   0  0  0  0  0  0  0  0  0  0  0  0
   -4.9967   -6.8513    3.4362 H   0  0  0  0  0  0  0  0  0  0  0  0
   -2.9098   -3.1951    4.3798 H   0  0  0  0  0  0  0  0  0  0  0  0
   -4.3201   -5.1184    5.0921 H   0  0  0  0  0  0  0  0  0  0  0  0
   -5.2131   -0.9756    0.3973 C   0  0  0  0  0  0  0  0  0  0  0  0
   -6.0127    0.0304   -0.1507 C   0  0  0  0  0  0  0  0  0  0  0  0
   -4.8282   -0.9369    1.7430 C   0  0  0  0  0  0  0  0  0  0  0  0
   -6.4554    1.0757    0.6705 C   0  0  0  0  0  0  0  0  0  0  0  0
   -6.3025    0.0371   -1.2060 H   0  0  0  0  0  0  0  0  0  0  0  0
```

```
   -5.2772    0.1077    2.5553 C   0  0  0  0  0  0  0  0  0  0  0  0
   -4.1726   -1.6987    2.1750 H   0  0  0  0  0  0  0  0  0  0  0  0
   -6.0950    1.1116    2.0203 C   0  0  0  0  0  0  0  0  0  0  0  0
   -7.0832    1.8656    0.2519 H   0  0  0  0  0  0  0  0  0  0  0  0
   -4.9877    0.1459    3.6068 H   0  0  0  0  0  0  0  0  0  0  0  0
   -6.4463    1.9265    2.6588 H   0  0  0  0  0  0  0  0  0  0  0  0
   -5.2535   -2.0688   -2.3900 C   0  0  0  0  0  0  0  0  0  0  0  0
   -6.6086   -2.1531   -2.7280 C   0  0  0  0  0  0  0  0  0  0  0  0
   -4.2894   -1.7173   -3.3380 C   0  0  0  0  0  0  0  0  0  0  0  0
   -6.9990   -1.8790   -4.0425 C   0  0  0  0  0  0  0  0  0  0  0  0
   -7.3734   -2.4287   -1.9955 H   0  0  0  0  0  0  0  0  0  0  0  0
   -4.6901   -1.4440   -4.6510 C   0  0  0  0  0  0  0  0  0  0  0  0
   -3.2164   -1.6511   -3.0738 H   0  0  0  0  0  0  0  0  0  0  0  0
   -6.0423   -1.5244   -5.0014 C   0  0  0  0  0  0  0  0  0  0  0  0
   -8.0531   -1.9395   -4.3218 H   0  0  0  0  0  0  0  0  0  0  0  0
   -3.9478   -1.1634   -5.4007 H   0  0  0  0  0  0  0  0  0  0  0  0
   -6.3535   -1.3082   -6.0269 H   0  0  0  0  0  0  0  0  0  0  0  0
   -5.7712   -3.8424   -0.1025 C   0  0  0  0  0  0  0  0  0  0  0  0
   -6.2895   -3.8902    1.1937 C   0  0  0  0  0  0  0  0  0  0  0  0
   -6.0963   -4.8351   -1.0349 C   0  0  0  0  0  0  0  0  0  0  0  0
   -7.1270   -4.9503    1.5634 C   0  0  0  0  0  0  0  0  0  0  0  0
   -6.0647   -3.1266    1.9424 H   0  0  0  0  0  0  0  0  0  0  0  0
   -6.9214   -5.8970   -0.6558 C   0  0  0  0  0  0  0  0  0  0  0  0
   -5.7293   -4.8056   -2.0661 H   0  0  0  0  0  0  0  0  0  0  0  0
   -7.4399   -5.9544    0.6431 C   0  0  0  0  0  0  0  0  0  0  0  0
   -7.5379   -4.9880    2.5739 H   0  0  0  0  0  0  0  0  0  0  0  0
   -7.1691   -6.6769   -1.3780 H   0  0  0  0  0  0  0  0  0  0  0  0
   -8.0942   -6.7796    0.9344 H   0  0  0  0  0  0  0  0  0  0  0  0
  1  2  1  0  0  0  0
  1  3  1  0  0  0  0
  1  9  1  0  0  0  0
  1 10  1  0  0  0  0
  2 11  1  0  0  0  0
  2 16  1  0  0  0  0
  2 19  1  0  0  0  0
  3  5  2  0  0  0  0
  3  7  1  0  0  0  0
  3 21  1  0  0  0  0
  4  5  1  0  0  0  0
  4  6  1  0  0  0  0
  4 13  1  0  0  0  0
  4 14  1  0  0  0  0
  5  8  1  0  0  0  0
  5 21  1  0  0  0  0
  6 12  1  0  0  0  0
  6 15  1  0  0  0  0
  6 20  1  0  0  0  0
 15 16  2  0  0  0  0
 15 17  1  0  0  0  0
 15 21  1  0  0  0  0
 16 18  1  0  0  0  0
 16 21  1  0  0  0  0
 21 22  1  0  0  0  0
 21 23  1  0  0  0  0
 22 57  1  0  0  0  0
 22 68  1  0  0  0  0
 22 79  1  0  0  0  0
 23 24  1  0  0  0  0
 23 35  1  0  0  0  0
 23 46  1  0  0  0  0
 24 25  2  0  0  0  0
 24 26  1  0  0  0  0
 25 27  1  0  0  0  0
 25 28  1  0  0  0  0
 26 29  2  0  0  0  0
```

```
 26 30  1  0  0  0  0
 27 31  2  0  0  0  0
 27 32  1  0  0  0  0
 29 31  1  0  0  0  0
 29 33  1  0  0  0  0
 31 34  1  0  0  0  0
 35 36  2  0  0  0  0
 35 37  1  0  0  0  0
 36 38  1  0  0  0  0
 36 39  1  0  0  0  0
 37 40  2  0  0  0  0
 37 41  1  0  0  0  0
 38 42  2  0  0  0  0
 38 43  1  0  0  0  0
 40 42  1  0  0  0  0
 40 44  1  0  0  0  0
 42 45  1  0  0  0  0
 46 47  2  0  0  0  0
 46 48  1  0  0  0  0
 47 49  1  0  0  0  0
 47 50  1  0  0  0  0
 48 51  2  0  0  0  0
 48 52  1  0  0  0  0
 49 53  2  0  0  0  0
 49 54  1  0  0  0  0
 51 53  1  0  0  0  0
 51 55  1  0  0  0  0
 53 56  1  0  0  0  0
 57 58  2  0  0  0  0
 57 59  1  0  0  0  0
 58 60  1  0  0  0  0
 58 61  1  0  0  0  0
 59 62  2  0  0  0  0
 59 63  1  0  0  0  0
 60 64  2  0  0  0  0
 60 65  1  0  0  0  0
 62 64  1  0  0  0  0
 62 66  1  0  0  0  0
 64 67  1  0  0  0  0
 68 69  2  0  0  0  0
 68 70  1  0  0  0  0
 69 71  1  0  0  0  0
 69 72  1  0  0  0  0
 70 73  2  0  0  0  0
 70 74  1  0  0  0  0
 71 75  2  0  0  0  0
 71 76  1  0  0  0  0
 73 75  1  0  0  0  0
 73 77  1  0  0  0  0
 75 78  1  0  0  0  0
 79 80  2  0  0  0  0
 79 81  1  0  0  0  0
 80 82  1  0  0  0  0
 80 83  1  0  0  0  0
 81 84  2  0  0  0  0
 81 85  1  0  0  0  0
 82 86  2  0  0  0  0
 82 87  1  0  0  0  0
 84 86  1  0  0  0  0
 84 88  1  0  0  0  0
 86 89  1  0  0  0  0
M  END
$$$$
mol002
Optimized Catalyst 002
```

```
Optimized Catalyst 002
 68 75  0  0  0  0  0  0  0  0999 V2000
   -2.2450   -2.2528   -0.2385 Rh  0  0  0  0  0  0  0  0  0  0  0  0
   -2.3942   -4.3414   -0.6743 P   0  0  0  0  0  0  0  0  0  0  0  0
   -0.9637    0.2826   -1.4655 C   0  0  0  0  0  0  0  0  0  0  0  0
    0.2793   -0.5172   -1.0275 C   0  0  0  0  0  0  0  0  0  0  0  0
   -2.1989    0.0684   -0.6280 C   0  0  0  0  0  0  0  0  0  0  0  0
   -1.1033    0.0739    1.6700 C   0  0  0  0  0  0  0  0  0  0  0  0
   -2.2671   -0.0521    0.7227 C   0  0  0  0  0  0  0  0  0  0  0  0
   -0.4489   -1.2905    1.9668 C   0  0  0  0  0  0  0  0  0  0  0  0
   -3.1336    0.0688   -1.2183 H   0  0  0  0  0  0  0  0  0  0  0  0
   -3.2441   -0.2045    1.2112 H   0  0  0  0  0  0  0  0  0  0  0  0
   -1.2064   -0.0079   -2.5221 H   0  0  0  0  0  0  0  0  0  0  0  0
   -0.7160    1.3777   -1.4501 H   0  0  0  0  0  0  0  0  0  0  0  0
    0.9648   -0.6044   -1.9124 H   0  0  0  0  0  0  0  0  0  0  0  0
   -1.0836   -1.8147    2.7323 H   0  0  0  0  0  0  0  0  0  0  0  0
   -1.4677    0.5078    2.6385 H   0  0  0  0  0  0  0  0  0  0  0  0
   -0.3510    0.7896    1.2553 H   0  0  0  0  0  0  0  0  0  0  0  0
   -0.2860   -2.2305    0.7950 C   0  0  0  0  0  0  0  0  0  0  0  0
   -0.0196   -1.9052   -0.5150 C   0  0  0  0  0  0  0  0  0  0  0  0
   -0.2042   -3.2870    1.1163 H   0  0  0  0  0  0  0  0  0  0  0  0
    0.2005   -2.7058   -1.2447 H   0  0  0  0  0  0  0  0  0  0  0  0
    0.8393    0.0437   -0.2348 H   0  0  0  0  0  0  0  0  0  0  0  0
    0.5703   -1.1161    2.4063 H   0  0  0  0  0  0  0  0  0  0  0  0
   -3.9452   -2.4099   -0.5355 N   0  0  0  0  0  0  0  0  0  0  0  0
   -5.0907   -2.3950   -0.7404 C   0  0  0  0  0  0  0  0  0  0  0  0
   -6.4885   -2.4749   -1.0195 C   0  0  0  0  0  0  0  0  0  0  0  0
   -6.9230   -3.2520   -2.1110 C   0  0  0  0  0  0  0  0  0  0  0  0
   -7.4133   -1.7915   -0.2094 C   0  0  0  0  0  0  0  0  0  0  0  0
   -8.2882   -3.3407   -2.3842 C   0  0  0  0  0  0  0  0  0  0  0  0
   -6.1968   -3.7768   -2.7345 H   0  0  0  0  0  0  0  0  0  0  0  0
   -8.7760   -1.8916   -0.4970 C   0  0  0  0  0  0  0  0  0  0  0  0
   -7.0692   -1.1903    0.6328 H   0  0  0  0  0  0  0  0  0  0  0  0
   -9.2130   -2.6631   -1.5801 C   0  0  0  0  0  0  0  0  0  0  0  0
   -8.6367   -3.9395   -3.2287 H   0  0  0  0  0  0  0  0  0  0  0  0
   -9.5032   -1.3643    0.1256 H   0  0  0  0  0  0  0  0  0  0  0  0
  -10.2818   -2.7361   -1.8000 H   0  0  0  0  0  0  0  0  0  0  0  0
   -0.7976   -5.2771   -0.8898 C   0  0  0  0  0  0  0  0  0  0  0  0
   -0.2171   -5.9052    0.2166 C   0  0  0  0  0  0  0  0  0  0  0  0
   -0.2194   -5.3794   -2.1578 C   0  0  0  0  0  0  0  0  0  0  0  0
    0.9631   -6.6371    0.0497 C   0  0  0  0  0  0  0  0  0  0  0  0
   -0.6646   -5.8519    1.2134 H   0  0  0  0  0  0  0  0  0  0  0  0
    0.9612   -6.1141   -2.3171 C   0  0  0  0  0  0  0  0  0  0  0  0
   -0.6669   -4.9085   -3.0376 H   0  0  0  0  0  0  0  0  0  0  0  0
    1.5521   -6.7418   -1.2156 C   0  0  0  0  0  0  0  0  0  0  0  0
    1.4235   -7.1324    0.9070 H   0  0  0  0  0  0  0  0  0  0  0  0
    1.4187   -6.2024   -3.3046 H   0  0  0  0  0  0  0  0  0  0  0  0
    2.4725   -7.3173   -1.3434 H   0  0  0  0  0  0  0  0  0  0  0  0
   -3.2696   -5.2411    0.6907 C   0  0  0  0  0  0  0  0  0  0  0  0
   -3.4523   -6.6254    0.6555 C   0  0  0  0  0  0  0  0  0  0  0  0
   -3.6835   -4.4858    1.7931 C   0  0  0  0  0  0  0  0  0  0  0  0
   -4.1091   -7.2540    1.7199 C   0  0  0  0  0  0  0  0  0  0  0  0
   -3.0763   -7.2410   -0.1657 H   0  0  0  0  0  0  0  0  0  0  0  0
   -4.3369   -5.1212    2.8540 C   0  0  0  0  0  0  0  0  0  0  0  0
   -3.4914   -3.3986    1.8480 H   0  0  0  0  0  0  0  0  0  0  0  0
   -4.5572   -6.5031    2.8120 C   0  0  0  0  0  0  0  0  0  0  0  0
   -4.2665   -8.3346    1.7008 H   0  0  0  0  0  0  0  0  0  0  0  0
   -4.6714   -4.5428    3.7170 H   0  0  0  0  0  0  0  0  0  0  0  0
   -5.0730   -6.9982    3.6392 H   0  0  0  0  0  0  0  0  0  0  0  0
   -3.2677   -4.5733   -2.3021 C   0  0  0  0  0  0  0  0  0  0  0  0
   -4.0372   -5.6907   -2.6155 C   0  0  0  0  0  0  0  0  0  0  0  0
   -3.0580   -3.5512   -3.2399 C   0  0  0  0  0  0  0  0  0  0  0  0
   -4.5958   -5.7964   -3.8979 C   0  0  0  0  0  0  0  0  0  0  0  0
   -4.2305   -6.4880   -1.8958 H   0  0  0  0  0  0  0  0  0  0  0  0
   -3.6128   -3.6665   -4.5159 C   0  0  0  0  0  0  0  0  0  0  0  0
```

```
   -2.4742   -2.6509   -2.9774 H   0  0  0  0  0  0  0  0  0  0  0  0
   -4.3780   -4.7940   -4.8459 C   0  0  0  0  0  0  0  0  0  0  0  0
   -5.1967   -6.6711   -4.1565 H   0  0  0  0  0  0  0  0  0  0  0  0
   -3.4515   -2.8826   -5.2584 H   0  0  0  0  0  0  0  0  0  0  0  0
   -4.8048   -4.8867   -5.8482 H   0  0  0  0  0  0  0  0  0  0  0  0
  1  2  1  0  0  0  0
  1 23  1  0  0  0  0
  1  7  1  0  0  0  0
  1  5  1  0  0  0  0
  1 17  1  0  0  0  0
  1 18  1  0  0  0  0
  2 36  1  0  0  0  0
  2 47  1  0  0  0  0
  2 58  1  0  0  0  0
  3  4  1  0  0  0  0
  3  5  1  0  0  0  0
  3 11  1  0  0  0  0
  3 12  1  0  0  0  0
  4 13  1  0  0  0  0
  4 18  1  0  0  0  0
  4 21  1  0  0  0  0
  5  7  2  0  0  0  0
  5  9  1  0  0  0  0
  6  7  1  0  0  0  0
  6  8  1  0  0  0  0
  6 15  1  0  0  0  0
  6 16  1  0  0  0  0
  7 10  1  0  0  0  0
  8 14  1  0  0  0  0
  8 17  1  0  0  0  0
  8 22  1  0  0  0  0
 17 18  2  0  0  0  0
 17 19  1  0  0  0  0
 18 20  1  0  0  0  0
 23 24  3  0  0  0  0
 24 25  1  0  0  0  0
 25 26  2  0  0  0  0
 25 27  1  0  0  0  0
 26 28  1  0  0  0  0
 26 29  1  0  0  0  0
 27 30  2  0  0  0  0
 27 31  1  0  0  0  0
 28 32  2  0  0  0  0
 28 33  1  0  0  0  0
 30 32  1  0  0  0  0
 30 34  1  0  0  0  0
 32 35  1  0  0  0  0
 36 37  2  0  0  0  0
 36 38  1  0  0  0  0
 37 39  1  0  0  0  0
 37 40  1  0  0  0  0
 38 41  2  0  0  0  0
 38 42  1  0  0  0  0
 39 43  2  0  0  0  0
 39 44  1  0  0  0  0
 41 43  1  0  0  0  0
 41 45  1  0  0  0  0
 43 46  1  0  0  0  0
 47 48  2  0  0  0  0
 47 49  1  0  0  0  0
 48 50  1  0  0  0  0
 48 51  1  0  0  0  0
 49 52  2  0  0  0  0
 49 53  1  0  0  0  0
 50 54  2  0  0  0  0
```

```
 50 55  1  0  0  0  0
 52 54  1  0  0  0  0
 52 56  1  0  0  0  0
 54 57  1  0  0  0  0
 58 59  2  0  0  0  0
 58 60  1  0  0  0  0
 59 61  1  0  0  0  0
 59 62  1  0  0  0  0
 60 63  2  0  0  0  0
 60 64  1  0  0  0  0
 61 65  2  0  0  0  0
 61 66  1  0  0  0  0
 63 65  1  0  0  0  0
 63 67  1  0  0  0  0
 65 68  1  0  0  0  0
M  END
$$$$
mol003
Optimized Catalyst 003
Optimized Catalyst 003
 47 52  0  0  0  0  0  0  0  0999 V2000
   -0.9707    0.3464   -1.5686 C   0  0  0  0  0  0  0  0  0  0  0  0
    0.2028   -0.5765   -1.1798 C   0  0  0  0  0  0  0  0  0  0  0  0
   -2.2228    0.1265   -0.7529 C   0  0  0  0  0  0  0  0  0  0  0  0
   -1.0988    0.1616    1.5513 C   0  0  0  0  0  0  0  0  0  0  0  0
   -2.2878    0.0181    0.6300 C   0  0  0  0  0  0  0  0  0  0  0  0
   -0.4204   -1.1939    1.8413 C   0  0  0  0  0  0  0  0  0  0  0  0
   -3.1587    0.2545   -1.3306 H   0  0  0  0  0  0  0  0  0  0  0  0
   -3.2708    0.0128    1.1360 H   0  0  0  0  0  0  0  0  0  0  0  0
   -1.2177    0.1663   -2.6486 H   0  0  0  0  0  0  0  0  0  0  0  0
   -0.6575    1.4189   -1.4569 H   0  0  0  0  0  0  0  0  0  0  0  0
    0.8387   -0.7506   -2.0875 H   0  0  0  0  0  0  0  0  0  0  0  0
   -0.9261   -1.6548    2.7324 H   0  0  0  0  0  0  0  0  0  0  0  0
   -1.4399    0.6061    2.5232 H   0  0  0  0  0  0  0  0  0  0  0  0
   -0.3643    0.8777    1.1014 H   0  0  0  0  0  0  0  0  0  0  0  0
   -0.4962   -2.1842    0.6999 C   0  0  0  0  0  0  0  0  0  0  0  0
   -0.2647   -1.9113   -0.6464 C   0  0  0  0  0  0  0  0  0  0  0  0
   -0.5946   -3.2459    1.0264 H   0  0  0  0  0  0  0  0  0  0  0  0
   -0.2309   -2.7499   -1.3771 H   0  0  0  0  0  0  0  0  0  0  0  0
    0.8513   -0.0798   -0.4133 H   0  0  0  0  0  0  0  0  0  0  0  0
    0.6593   -1.0270    2.0970 H   0  0  0  0  0  0  0  0  0  0  0  0
   -3.9578   -2.0295   -0.4745 N   0  0  0  0  0  0  0  0  0  0  0  0
   -5.0970   -2.2014   -0.6387 C   0  0  0  0  0  0  0  0  0  0  0  0
   -6.5026   -2.3621   -0.8393 C   0  0  0  0  0  0  0  0  0  0  0  0
   -7.0054   -2.5220   -2.1440 C   0  0  0  0  0  0  0  0  0  0  0  0
   -7.3706   -2.3535    0.2685 C   0  0  0  0  0  0  0  0  0  0  0  0
   -8.3807   -2.6704   -2.3327 C   0  0  0  0  0  0  0  0  0  0  0  0
   -6.3277   -2.5296   -2.9978 H   0  0  0  0  0  0  0  0  0  0  0  0
   -8.7431   -2.5041    0.0620 C   0  0  0  0  0  0  0  0  0  0  0  0
   -6.9752   -2.2314    1.2769 H   0  0  0  0  0  0  0  0  0  0  0  0
   -9.2481   -2.6613   -1.2341 C   0  0  0  0  0  0  0  0  0  0  0  0
   -8.7808   -2.7934   -3.3421 H   0  0  0  0  0  0  0  0  0  0  0  0
   -9.4252   -2.4983    0.9157 H   0  0  0  0  0  0  0  0  0  0  0  0
  -10.3243   -2.7772   -1.3889 H   0  0  0  0  0  0  0  0  0  0  0  0
   -2.3265   -4.4199   -0.6576 N   0  0  0  0  0  0  0  0  0  0  0  0
   -2.4329   -5.5545   -0.8727 C   0  0  0  0  0  0  0  0  0  0  0  0
   -2.5564   -6.9561   -1.1364 C   0  0  0  0  0  0  0  0  0  0  0  0
   -2.7969   -7.8469   -0.0743 C   0  0  0  0  0  0  0  0  0  0  0  0
   -2.4337   -7.4317   -2.4548 C   0  0  0  0  0  0  0  0  0  0  0  0
   -2.9138   -9.2127   -0.3382 C   0  0  0  0  0  0  0  0  0  0  0  0
   -2.8904   -7.4728    0.9459 H   0  0  0  0  0  0  0  0  0  0  0  0
   -2.5529   -8.8002   -2.7036 C   0  0  0  0  0  0  0  0  0  0  0  0
   -2.2461   -6.7369   -3.2742 H   0  0  0  0  0  0  0  0  0  0  0  0
   -2.7921   -9.6891   -1.6489 C   0  0  0  0  0  0  0  0  0  0  0  0
   -3.0997   -9.9115    0.4808 H   0  0  0  0  0  0  0  0  0  0  0  0
```

```
   -2.4582   -9.1782   -3.7243 H   0  0  0  0  0  0  0  0  0  0  0  0
   -2.8836  -10.7600   -1.8496 H   0  0  0  0  0  0  0  0  0  0  0  0
   -2.2995   -1.9873   -0.2459 Rh  0  0  0  0  0  0  0  0  0  0  0  0
  1  2  1  0  0  0  0
  1  3  1  0  0  0  0
  1  9  1  0  0  0  0
  1 10  1  0  0  0  0
  2 11  1  0  0  0  0
  2 16  1  0  0  0  0
  2 19  1  0  0  0  0
  3  5  2  0  0  0  0
  3  7  1  0  0  0  0
  3 47  1  0  0  0  0
  4  5  1  0  0  0  0
  4  6  1  0  0  0  0
  4 13  1  0  0  0  0
  4 14  1  0  0  0  0
  5  8  1  0  0  0  0
  5 47  1  0  0  0  0
  6 12  1  0  0  0  0
  6 15  1  0  0  0  0
  6 20  1  0  0  0  0
 15 16  2  0  0  0  0
 15 17  1  0  0  0  0
 15 47  1  0  0  0  0
 16 18  1  0  0  0  0
 16 47  1  0  0  0  0
 21 22  3  0  0  0  0
 21 47  1  0  0  0  0
 22 23  1  0  0  0  0
 23 24  2  0  0  0  0
 23 25  1  0  0  0  0
 24 26  1  0  0  0  0
 24 27  1  0  0  0  0
 25 28  2  0  0  0  0
 25 29  1  0  0  0  0
 26 30  2  0  0  0  0
 26 31  1  0  0  0  0
 28 30  1  0  0  0  0
 28 32  1  0  0  0  0
 30 33  1  0  0  0  0
 34 35  3  0  0  0  0
 34 47  1  0  0  0  0
 35 36  1  0  0  0  0
 36 37  2  0  0  0  0
 36 38  1  0  0  0  0
 37 39  1  0  0  0  0
 37 40  1  0  0  0  0
 38 41  2  0  0  0  0
 38 42  1  0  0  0  0
 39 43  2  0  0  0  0
 39 44  1  0  0  0  0
 41 43  1  0  0  0  0
 41 45  1  0  0  0  0
 43 46  1  0  0  0  0
M  END
$$$$
mol004
Optimized Catalyst 004
Optimized Catalyst 004
 68 75  0  0  0  0  0  0  0  0999 V2000
   -2.4351   -4.6371   -0.7309 P   0  0  0  0  0  0  0  0  0  0  0  0
   -0.4180    0.6029   -0.9929 C   0  0  0  0  0  0  0  0  0  0  0  0
    0.6879   -0.3443   -0.4864 C   0  0  0  0  0  0  0  0  0  0  0  0
   -1.7494    0.5381   -0.2913 C   0  0  0  0  0  0  0  0  0  0  0  0
```

```
   -0.8853    0.3571    2.0845 C   0  0  0  0  0  0  0  0  0  0  0  0
   -1.9638    0.4296    1.0393 C   0  0  0  0  0  0  0  0  0  0  0  0
   -0.3841   -1.0728    2.3675 C   0  0  0  0  0  0  0  0  0  0  0  0
   -2.6076    0.7218   -0.9673 H   0  0  0  0  0  0  0  0  0  0  0  0
   -2.9901    0.4903    1.4430 H   0  0  0  0  0  0  0  0  0  0  0  0
   -0.5727    0.4114   -2.0887 H   0  0  0  0  0  0  0  0  0  0  0  0
   -0.0444    1.6630   -0.8917 H   0  0  0  0  0  0  0  0  0  0  0  0
    1.4375   -0.4691   -1.3163 H   0  0  0  0  0  0  0  0  0  0  0  0
   -1.0625   -1.5391    3.1333 H   0  0  0  0  0  0  0  0  0  0  0  0
   -1.2833    0.7811    3.0474 H   0  0  0  0  0  0  0  0  0  0  0  0
   -0.0311    1.0162    1.7786 H   0  0  0  0  0  0  0  0  0  0  0  0
   -0.2547   -2.0135    1.1911 C   0  0  0  0  0  0  0  0  0  0  0  0
    0.2201   -1.7062   -0.0500 C   0  0  0  0  0  0  0  0  0  0  0  0
   -0.3328   -3.0803    1.4986 H   0  0  0  0  0  0  0  0  0  0  0  0
    0.4406   -2.5152   -0.7765 H   0  0  0  0  0  0  0  0  0  0  0  0
    1.2370    0.1334    0.3702 H   0  0  0  0  0  0  0  0  0  0  0  0
    0.6416   -0.9972    2.8334 H   0  0  0  0  0  0  0  0  0  0  0  0
   -4.2418   -2.1087   -0.6369 N   0  0  0  0  0  0  0  0  0  0  0  0
   -5.3537   -2.1211   -0.9965 C   0  0  0  0  0  0  0  0  0  0  0  0
   -6.7036   -2.1548   -1.4417 C   0  0  0  0  0  0  0  0  0  0  0  0
   -7.2175   -3.3475   -1.9926 C   0  0  0  0  0  0  0  0  0  0  0  0
   -7.5129   -1.0058   -1.3325 C   0  0  0  0  0  0  0  0  0  0  0  0
   -8.5393   -3.3806   -2.4334 C   0  0  0  0  0  0  0  0  0  0  0  0
   -6.5836   -4.2333   -2.0724 H   0  0  0  0  0  0  0  0  0  0  0  0
   -8.8337   -1.0582   -1.7783 C   0  0  0  0  0  0  0  0  0  0  0  0
   -7.1111   -0.0858   -0.9040 H   0  0  0  0  0  0  0  0  0  0  0  0
   -9.3466   -2.2401   -2.3271 C   0  0  0  0  0  0  0  0  0  0  0  0
   -8.9490   -4.2991   -2.8616 H   0  0  0  0  0  0  0  0  0  0  0  0
   -9.4711   -0.1723   -1.6980 H   0  0  0  0  0  0  0  0  0  0  0  0
  -10.3846   -2.2732   -2.6744 H   0  0  0  0  0  0  0  0  0  0  0  0
   -0.7794   -5.3264   -1.1919 C   0  0  0  0  0  0  0  0  0  0  0  0
    0.1114   -5.6783   -0.1693 C   0  0  0  0  0  0  0  0  0  0  0  0
   -0.4514   -5.5317   -2.5340 C   0  0  0  0  0  0  0  0  0  0  0  0
    1.3528   -6.2255   -0.5024 C   0  0  0  0  0  0  0  0  0  0  0  0
   -0.1381   -5.5599    0.8884 H   0  0  0  0  0  0  0  0  0  0  0  0
    0.7962   -6.0804   -2.8575 C   0  0  0  0  0  0  0  0  0  0  0  0
   -1.1406   -5.2908   -3.3485 H   0  0  0  0  0  0  0  0  0  0  0  0
    1.6970   -6.4250   -1.8461 C   0  0  0  0  0  0  0  0  0  0  0  0
    2.0556   -6.5071    0.2858 H   0  0  0  0  0  0  0  0  0  0  0  0
    1.0618   -6.2463   -3.9048 H   0  0  0  0  0  0  0  0  0  0  0  0
    2.6694   -6.8564   -2.1019 H   0  0  0  0  0  0  0  0  0  0  0  0
   -3.0392   -5.6296    0.7116 C   0  0  0  0  0  0  0  0  0  0  0  0
   -2.8070   -7.0068    0.7585 C   0  0  0  0  0  0  0  0  0  0  0  0
   -3.7362   -4.9755    1.7327 C   0  0  0  0  0  0  0  0  0  0  0  0
   -3.2984   -7.7428    1.8435 C   0  0  0  0  0  0  0  0  0  0  0  0
   -2.2454   -7.5294   -0.0226 H   0  0  0  0  0  0  0  0  0  0  0  0
   -4.2256   -5.7191    2.8106 C   0  0  0  0  0  0  0  0  0  0  0  0
   -3.9079   -3.8871    1.7193 H   0  0  0  0  0  0  0  0  0  0  0  0
   -4.0088   -7.1024    2.8637 C   0  0  0  0  0  0  0  0  0  0  0  0
   -3.1239   -8.8209    1.8937 H   0  0  0  0  0  0  0  0  0  0  0  0
   -4.7755   -5.2241    3.6146 H   0  0  0  0  0  0  0  0  0  0  0  0
   -4.3933   -7.6815    3.7089 H   0  0  0  0  0  0  0  0  0  0  0  0
   -3.5827   -4.9622   -2.1540 C   0  0  0  0  0  0  0  0  0  0  0  0
   -4.4338   -6.0678   -2.1260 C   0  0  0  0  0  0  0  0  0  0  0  0
   -3.5431   -4.1097   -3.2652 C   0  0  0  0  0  0  0  0  0  0  0  0
   -5.2510   -6.3311   -3.2344 C   0  0  0  0  0  0  0  0  0  0  0  0
   -4.4904   -6.7418   -1.2655 H   0  0  0  0  0  0  0  0  0  0  0  0
   -4.3596   -4.3813   -4.3656 C   0  0  0  0  0  0  0  0  0  0  0  0
   -2.8885   -3.2320   -3.3051 H   0  0  0  0  0  0  0  0  0  0  0  0
   -5.2099   -5.4956   -4.3530 C   0  0  0  0  0  0  0  0  0  0  0  0
   -5.9131   -7.2008   -3.2253 H   0  0  0  0  0  0  0  0  0  0  0  0
   -4.3325   -3.7289   -5.2421 H   0  0  0  0  0  0  0  0  0  0  0  0
   -5.8388   -5.7105   -5.2219 H   0  0  0  0  0  0  0  0  0  0  0  0
   -2.3923   -2.1192   -0.0736 Ir  0  0  0  0  0  0  0  0  0  0  0  0
  1 35  1  0  0  0  0
```

```
  1 46  1  0  0  0  0
  1 57  1  0  0  0  0
  1 68  1  0  0  0  0
  2  3  1  0  0  0  0
  2  4  1  0  0  0  0
  2 10  1  0  0  0  0
  2 11  1  0  0  0  0
  3 12  1  0  0  0  0
  3 17  1  0  0  0  0
  3 20  1  0  0  0  0
  4  6  2  0  0  0  0
  4  8  1  0  0  0  0
  4 68  1  0  0  0  0
  5  6  1  0  0  0  0
  5  7  1  0  0  0  0
  5 14  1  0  0  0  0
  5 15  1  0  0  0  0
  6  9  1  0  0  0  0
  6 68  1  0  0  0  0
  7 13  1  0  0  0  0
  7 16  1  0  0  0  0
  7 21  1  0  0  0  0
 16 17  2  0  0  0  0
 16 18  1  0  0  0  0
 16 68  1  0  0  0  0
 17 19  1  0  0  0  0
 17 68  1  0  0  0  0
 22 23  3  0  0  0  0
 22 68  1  0  0  0  0
 23 24  1  0  0  0  0
 24 25  2  0  0  0  0
 24 26  1  0  0  0  0
 25 27  1  0  0  0  0
 25 28  1  0  0  0  0
 26 29  2  0  0  0  0
 26 30  1  0  0  0  0
 27 31  2  0  0  0  0
 27 32  1  0  0  0  0
 29 31  1  0  0  0  0
 29 33  1  0  0  0  0
 31 34  1  0  0  0  0
 35 36  2  0  0  0  0
 35 37  1  0  0  0  0
 36 38  1  0  0  0  0
 36 39  1  0  0  0  0
 37 40  2  0  0  0  0
 37 41  1  0  0  0  0
 38 42  2  0  0  0  0
 38 43  1  0  0  0  0
 40 42  1  0  0  0  0
 40 44  1  0  0  0  0
 42 45  1  0  0  0  0
 46 47  2  0  0  0  0
 46 48  1  0  0  0  0
 47 49  1  0  0  0  0
 47 50  1  0  0  0  0
 48 51  2  0  0  0  0
 48 52  1  0  0  0  0
 49 53  2  0  0  0  0
 49 54  1  0  0  0  0
 51 53  1  0  0  0  0
 51 55  1  0  0  0  0
 53 56  1  0  0  0  0
 57 58  2  0  0  0  0
 57 59  1  0  0  0  0
```

```
 58 60  1  0  0  0  0
 58 61  1  0  0  0  0
 59 62  2  0  0  0  0
 59 63  1  0  0  0  0
 60 64  2  0  0  0  0
 60 65  1  0  0  0  0
 62 64  1  0  0  0  0
 62 66  1  0  0  0  0
 64 67  1  0  0  0  0
M  END
$$$$
mol005
Optimized Catalyst 005
Optimized Catalyst 005
 47 52  0  0  0  0  0  0  0  0999 V2000
   -0.8741    0.2727   -1.4927 C   0  0  0  0  0  0  0  0  0  0  0  0
    0.3495   -0.6106   -1.1701 C   0  0  0  0  0  0  0  0  0  0  0  0
   -2.1194    0.0095   -0.6763 C   0  0  0  0  0  0  0  0  0  0  0  0
   -0.9657   -0.0539    1.5985 C   0  0  0  0  0  0  0  0  0  0  0  0
   -2.1669   -0.1561    0.6871 C   0  0  0  0  0  0  0  0  0  0  0  0
   -0.1897   -1.3731    1.7962 C   0  0  0  0  0  0  0  0  0  0  0  0
   -3.0618    0.2053   -1.2375 H   0  0  0  0  0  0  0  0  0  0  0  0
   -3.1452   -0.1451    1.2144 H   0  0  0  0  0  0  0  0  0  0  0  0
   -1.1205    0.1577   -2.5841 H   0  0  0  0  0  0  0  0  0  0  0  0
   -0.5900    1.3534   -1.3318 H   0  0  0  0  0  0  0  0  0  0  0  0
    0.9759   -0.6919   -2.1016 H   0  0  0  0  0  0  0  0  0  0  0  0
   -0.5978   -1.8955    2.7049 H   0  0  0  0  0  0  0  0  0  0  0  0
   -1.3085    0.3037    2.6091 H   0  0  0  0  0  0  0  0  0  0  0  0
   -0.2781    0.7399    1.1976 H   0  0  0  0  0  0  0  0  0  0  0  0
   -0.2015   -2.3342    0.6288 C   0  0  0  0  0  0  0  0  0  0  0  0
    0.0158   -2.0033   -0.6873 C   0  0  0  0  0  0  0  0  0  0  0  0
   -0.1556   -3.4031    0.9397 H   0  0  0  0  0  0  0  0  0  0  0  0
    0.1846   -2.8029   -1.4406 H   0  0  0  0  0  0  0  0  0  0  0  0
    0.9933   -0.1081   -0.3977 H   0  0  0  0  0  0  0  0  0  0  0  0
    0.8897   -1.1260    2.0160 H   0  0  0  0  0  0  0  0  0  0  0  0
   -4.3064   -2.2664   -0.5944 N   0  0  0  0  0  0  0  0  0  0  0  0
   -5.4651   -2.2936   -0.7414 C   0  0  0  0  0  0  0  0  0  0  0  0
   -6.8774   -2.2977   -0.9158 C   0  0  0  0  0  0  0  0  0  0  0  0
   -7.4219   -2.1942   -2.2125 C   0  0  0  0  0  0  0  0  0  0  0  0
   -7.7202   -2.4018    0.2101 C   0  0  0  0  0  0  0  0  0  0  0  0
   -8.8072   -2.1947   -2.3733 C   0  0  0  0  0  0  0  0  0  0  0  0
   -6.7664   -2.1140   -3.0811 H   0  0  0  0  0  0  0  0  0  0  0  0
   -9.1031   -2.4007    0.0301 C   0  0  0  0  0  0  0  0  0  0  0  0
   -7.2950   -2.4820    1.2118 H   0  0  0  0  0  0  0  0  0  0  0  0
   -9.6468   -2.2971   -1.2567 C   0  0  0  0  0  0  0  0  0  0  0  0
   -9.2398   -2.1139   -3.3750 H   0  0  0  0  0  0  0  0  0  0  0  0
   -9.7658   -2.4800    0.8970 H   0  0  0  0  0  0  0  0  0  0  0  0
  -10.7337   -2.2957   -1.3905 H   0  0  0  0  0  0  0  0  0  0  0  0
   -2.3362   -4.2689   -0.6619 N   0  0  0  0  0  0  0  0  0  0  0  0
   -2.3857   -5.4204   -0.8529 C   0  0  0  0  0  0  0  0  0  0  0  0
   -2.4164   -6.8244   -1.0829 C   0  0  0  0  0  0  0  0  0  0  0  0
   -2.5839   -7.7072    0.0040 C   0  0  0  0  0  0  0  0  0  0  0  0
   -2.2753   -7.3209   -2.3953 C   0  0  0  0  0  0  0  0  0  0  0  0
   -2.6078   -9.0818   -0.2304 C   0  0  0  0  0  0  0  0  0  0  0  0
   -2.6932   -7.3191    1.0179 H   0  0  0  0  0  0  0  0  0  0  0  0
   -2.3019   -8.6985   -2.6106 C   0  0  0  0  0  0  0  0  0  0  0  0
   -2.1460   -6.6344   -3.2336 H   0  0  0  0  0  0  0  0  0  0  0  0
   -2.4670   -9.5778   -1.5328 C   0  0  0  0  0  0  0  0  0  0  0  0
   -2.7360   -9.7752    0.6061 H   0  0  0  0  0  0  0  0  0  0  0  0
   -2.1923   -9.0940   -3.6248 H   0  0  0  0  0  0  0  0  0  0  0  0
   -2.4857  -10.6584   -1.7092 H   0  0  0  0  0  0  0  0  0  0  0  0
   -2.3571   -2.3282   -0.3581 Ir  0  0  0  0  0  0  0  0  0  0  0  0
  1  2  1  0  0  0  0
  1  3  1  0  0  0  0
  1  9  1  0  0  0  0
```

```
  1 10  1  0  0  0  0
  2 11  1  0  0  0  0
  2 16  1  0  0  0  0
  2 19  1  0  0  0  0
  3  5  2  0  0  0  0
  3  7  1  0  0  0  0
  3 47  1  0  0  0  0
  4  5  1  0  0  0  0
  4  6  1  0  0  0  0
  4 13  1  0  0  0  0
  4 14  1  0  0  0  0
  5  8  1  0  0  0  0
  5 47  1  0  0  0  0
  6 12  1  0  0  0  0
  6 15  1  0  0  0  0
  6 20  1  0  0  0  0
 15 16  2  0  0  0  0
 15 17  1  0  0  0  0
 15 47  1  0  0  0  0
 16 18  1  0  0  0  0
 16 47  1  0  0  0  0
 21 22  3  0  0  0  0
 21 47  1  0  0  0  0
 22 23  1  0  0  0  0
 23 24  2  0  0  0  0
 23 25  1  0  0  0  0
 24 26  1  0  0  0  0
 24 27  1  0  0  0  0
 25 28  2  0  0  0  0
 25 29  1  0  0  0  0
 26 30  2  0  0  0  0
 26 31  1  0  0  0  0
 28 30  1  0  0  0  0
 28 32  1  0  0  0  0
 30 33  1  0  0  0  0
 34 35  3  0  0  0  0
 34 47  1  0  0  0  0
 35 36  1  0  0  0  0
 36 37  2  0  0  0  0
 36 38  1  0  0  0  0
 37 39  1  0  0  0  0
 37 40  1  0  0  0  0
 38 41  2  0  0  0  0
 38 42  1  0  0  0  0
 39 43  2  0  0  0  0
 39 44  1  0  0  0  0
 41 43  1  0  0  0  0
 41 45  1  0  0  0  0
 43 46  1  0  0  0  0
M  END
$$$$
mol006
Optimized Catalyst 006
Optimized Catalyst 006
 90 96  0  0  0  0  0  0  0  0999 V2000
   -2.5073   -2.8804    0.8569 Ru  0  0  0  0  0  0  0  0  0  0  0  0
   -2.8811   -2.3385    3.0489 P   0  0  0  0  0  0  0  0  0  0  0  0
   -2.1508   -3.2213   -1.4387 P   0  0  0  0  0  0  0  0  0  0  0  0
   -1.8439   -0.8447    3.5470 C   0  0  0  0  0  0  0  0  0  0  0  0
   -0.4596   -0.9988    3.6773 C   0  0  0  0  0  0  0  0  0  0  0  0
   -2.4349    0.4060    3.7225 C   0  0  0  0  0  0  0  0  0  0  0  0
    0.3303    0.0995    4.0313 C   0  0  0  0  0  0  0  0  0  0  0  0
    0.0287   -1.9591    3.4984 H   0  0  0  0  0  0  0  0  0  0  0  0
   -1.6393    1.5061    4.0720 C   0  0  0  0  0  0  0  0  0  0  0  0
   -3.5095    0.5615    3.5876 H   0  0  0  0  0  0  0  0  0  0  0  0
```

```
   -0.2608    1.3521    4.2351 C   0  0  0  0  0  0  0  0  0  0  0  0
    1.4067   -0.0180    4.1454 H   0  0  0  0  0  0  0  0  0  0  0  0
   -2.1006    2.4829    4.2147 H   0  0  0  0  0  0  0  0  0  0  0  0
    0.3555    2.2063    4.5143 H   0  0  0  0  0  0  0  0  0  0  0  0
   -2.5091   -3.6435    4.3494 C   0  0  0  0  0  0  0  0  0  0  0  0
   -3.2080   -4.8457    4.2073 C   0  0  0  0  0  0  0  0  0  0  0  0
   -1.6558   -3.4367    5.4331 C   0  0  0  0  0  0  0  0  0  0  0  0
   -3.0062   -5.8731    5.1365 C   0  0  0  0  0  0  0  0  0  0  0  0
   -3.9224   -5.0056    3.3784 H   0  0  0  0  0  0  0  0  0  0  0  0
   -1.4555   -4.4689    6.3573 C   0  0  0  0  0  0  0  0  0  0  0  0
   -1.1480   -2.4850    5.5969 H   0  0  0  0  0  0  0  0  0  0  0  0
   -2.1250   -5.6880    6.2057 C   0  0  0  0  0  0  0  0  0  0  0  0
   -3.5454   -6.8138    5.0242 H   0  0  0  0  0  0  0  0  0  0  0  0
   -0.7850   -4.3185    7.2014 H   0  0  0  0  0  0  0  0  0  0  0  0
   -1.9670   -6.4893    6.9268 H   0  0  0  0  0  0  0  0  0  0  0  0
   -4.6185   -1.8908    3.5921 C   0  0  0  0  0  0  0  0  0  0  0  0
   -4.8333   -1.3307    4.8563 C   0  0  0  0  0  0  0  0  0  0  0  0
   -5.6901   -2.2165    2.7633 C   0  0  0  0  0  0  0  0  0  0  0  0
   -6.1373   -1.0462    5.2696 C   0  0  0  0  0  0  0  0  0  0  0  0
   -4.0069   -1.1184    5.5378 H   0  0  0  0  0  0  0  0  0  0  0  0
   -6.9975   -1.9336    3.1853 C   0  0  0  0  0  0  0  0  0  0  0  0
   -5.5432   -2.7196    1.7924 H   0  0  0  0  0  0  0  0  0  0  0  0
   -7.2196   -1.3439    4.4307 C   0  0  0  0  0  0  0  0  0  0  0  0
   -6.3145   -0.6031    6.2475 H   0  0  0  0  0  0  0  0  0  0  0  0
   -7.8369   -2.1887    2.5387 H   0  0  0  0  0  0  0  0  0  0  0  0
   -8.2355   -1.1230    4.7568 H   0  0  0  0  0  0  0  0  0  0  0  0
   -0.6783   -2.3991   -2.2882 C   0  0  0  0  0  0  0  0  0  0  0  0
   -0.3356   -2.7564   -3.5976 C   0  0  0  0  0  0  0  0  0  0  0  0
    0.0527   -1.4255   -1.6129 C   0  0  0  0  0  0  0  0  0  0  0  0
    0.7530   -2.1405   -4.2227 C   0  0  0  0  0  0  0  0  0  0  0  0
   -0.9012   -3.5111   -4.1537 H   0  0  0  0  0  0  0  0  0  0  0  0
    1.1421   -0.8068   -2.2404 C   0  0  0  0  0  0  0  0  0  0  0  0
   -0.2111   -1.1266   -0.5864 H   0  0  0  0  0  0  0  0  0  0  0  0
    1.4933   -1.1655   -3.5438 C   0  0  0  0  0  0  0  0  0  0  0  0
    1.0234   -2.4183   -5.2406 H   0  0  0  0  0  0  0  0  0  0  0  0
    1.7112   -0.0442   -1.7108 H   0  0  0  0  0  0  0  0  0  0  0  0
    2.3406   -0.6861   -4.0336 H   0  0  0  0  0  0  0  0  0  0  0  0
   -3.6031   -2.6052   -2.4670 C   0  0  0  0  0  0  0  0  0  0  0  0
   -4.8861   -2.9764   -2.0546 C   0  0  0  0  0  0  0  0  0  0  0  0
   -3.4301   -1.8033   -3.5962 C   0  0  0  0  0  0  0  0  0  0  0  0
   -5.9988   -2.5790   -2.8041 C   0  0  0  0  0  0  0  0  0  0  0  0
   -5.0563   -3.5638   -1.1342 H   0  0  0  0  0  0  0  0  0  0  0  0
   -4.5469   -1.4004   -4.3390 C   0  0  0  0  0  0  0  0  0  0  0  0
   -2.4465   -1.4597   -3.9208 H   0  0  0  0  0  0  0  0  0  0  0  0
   -5.8302   -1.7960   -3.9500 C   0  0  0  0  0  0  0  0  0  0  0  0
   -6.9971   -2.8791   -2.4859 H   0  0  0  0  0  0  0  0  0  0  0  0
   -4.4138   -0.7760   -5.2205 H   0  0  0  0  0  0  0  0  0  0  0  0
   -6.6970   -1.4883   -4.5336 H   0  0  0  0  0  0  0  0  0  0  0  0
   -1.9594   -5.0109   -1.9952 C   0  0  0  0  0  0  0  0  0  0  0  0
   -0.6998   -5.5280   -2.3149 C   0  0  0  0  0  0  0  0  0  0  0  0
   -3.0872   -5.8342   -2.0261 C   0  0  0  0  0  0  0  0  0  0  0  0
   -0.5719   -6.8717   -2.6814 C   0  0  0  0  0  0  0  0  0  0  0  0
    0.2035   -4.9155   -2.2892 H   0  0  0  0  0  0  0  0  0  0  0  0
   -2.9540   -7.1795   -2.3967 C   0  0  0  0  0  0  0  0  0  0  0  0
   -4.0818   -5.4741   -1.7310 H   0  0  0  0  0  0  0  0  0  0  0  0
   -1.7001   -7.6983   -2.7257 C   0  0  0  0  0  0  0  0  0  0  0  0
    0.4071   -7.2748   -2.9323 H   0  0  0  0  0  0  0  0  0  0  0  0
   -3.8358   -7.8201   -2.4134 H   0  0  0  0  0  0  0  0  0  0  0  0
   -1.5990   -8.7443   -3.0116 H   0  0  0  0  0  0  0  0  0  0  0  0
   -4.5265   -4.8696    0.8890 Cl  0  0  0  0  0  0  0  0  0  0  0  0
   -1.0718   -1.4798    0.9175 H   0  0  0  0  0  0  0  0  0  0  0  0
   -3.6589   -1.4965    0.3999 C   0  0  0  0  0  0  0  0  0  0  0  0
   -4.3527   -0.6019    0.1541 O   0  0  0  0  0  0  0  0  0  0  0  0
   -1.5626   -5.8216    1.2779 C   0  0  0  0  0  0  0  0  0  0  0  0
    0.3323   -4.2247    1.0009 C   0  0  0  0  0  0  0  0  0  0  0  0
```

```
   -0.4504   -6.8863    1.2085 C   0  0  0  0  0  0  0  0  0  0  0  0
   -2.1229   -5.7967    0.3030 H   0  0  0  0  0  0  0  0  0  0  0  0
    1.3781   -5.1639    1.6420 C   0  0  0  0  0  0  0  0  0  0  0  0
    0.6086   -3.1526    1.1759 H   0  0  0  0  0  0  0  0  0  0  0  0
    0.7634   -6.5109    2.0707 C   0  0  0  0  0  0  0  0  0  0  0  0
   -0.1242   -7.0025    0.1458 H   0  0  0  0  0  0  0  0  0  0  0  0
    2.1875   -5.3468    0.8964 H   0  0  0  0  0  0  0  0  0  0  0  0
    0.4420   -6.4513    3.1386 H   0  0  0  0  0  0  0  0  0  0  0  0
   -0.9383   -4.3760    2.6060 H   0  0  0  0  0  0  0  0  0  0  0  0
   -2.3114   -6.0789    2.0750 H   0  0  0  0  0  0  0  0  0  0  0  0
   -0.8669   -7.8620    1.5528 H   0  0  0  0  0  0  0  0  0  0  0  0
    1.5359   -7.3104    1.9839 H   0  0  0  0  0  0  0  0  0  0  0  0
    0.2952   -4.4218   -0.0990 H   0  0  0  0  0  0  0  0  0  0  0  0
    1.8295   -4.6720    2.5343 H   0  0  0  0  0  0  0  0  0  0  0  0
   -1.0469   -4.4315    1.5694 N   0  0  0  0  0  0  0  0  0  0  0  0
  1  2  1  0  0  0  0
  1  3  1  0  0  0  0
  1 72  1  0  0  0  0
  1 70  1  0  0  0  0
  1 90  1  0  0  0  0
  1 71  1  0  0  0  0
  2  4  1  0  0  0  0
  2 15  1  0  0  0  0
  2 26  1  0  0  0  0
  3 37  1  0  0  0  0
  3 48  1  0  0  0  0
  3 59  1  0  0  0  0
  4  5  2  0  0  0  0
  4  6  1  0  0  0  0
  5  7  1  0  0  0  0
  5  8  1  0  0  0  0
  6  9  2  0  0  0  0
  6 10  1  0  0  0  0
  7 11  2  0  0  0  0
  7 12  1  0  0  0  0
  9 11  1  0  0  0  0
  9 13  1  0  0  0  0
 11 14  1  0  0  0  0
 15 16  2  0  0  0  0
 15 17  1  0  0  0  0
 16 18  1  0  0  0  0
 16 19  1  0  0  0  0
 17 20  2  0  0  0  0
 17 21  1  0  0  0  0
 18 22  2  0  0  0  0
 18 23  1  0  0  0  0
 20 22  1  0  0  0  0
 20 24  1  0  0  0  0
 22 25  1  0  0  0  0
 26 27  2  0  0  0  0
 26 28  1  0  0  0  0
 27 29  1  0  0  0  0
 27 30  1  0  0  0  0
 28 31  2  0  0  0  0
 28 32  1  0  0  0  0
 29 33  2  0  0  0  0
 29 34  1  0  0  0  0
 31 33  1  0  0  0  0
 31 35  1  0  0  0  0
 33 36  1  0  0  0  0
 37 38  2  0  0  0  0
 37 39  1  0  0  0  0
 38 40  1  0  0  0  0
 38 41  1  0  0  0  0
 39 42  2  0  0  0  0
```

```
 39 43  1  0  0  0  0
 40 44  2  0  0  0  0
 40 45  1  0  0  0  0
 42 44  1  0  0  0  0
 42 46  1  0  0  0  0
 44 47  1  0  0  0  0
 48 49  2  0  0  0  0
 48 50  1  0  0  0  0
 49 51  1  0  0  0  0
 49 52  1  0  0  0  0
 50 53  2  0  0  0  0
 50 54  1  0  0  0  0
 51 55  2  0  0  0  0
 51 56  1  0  0  0  0
 53 55  1  0  0  0  0
 53 57  1  0  0  0  0
 55 58  1  0  0  0  0
 59 60  2  0  0  0  0
 59 61  1  0  0  0  0
 60 62  1  0  0  0  0
 60 63  1  0  0  0  0
 61 64  2  0  0  0  0
 61 65  1  0  0  0  0
 62 66  2  0  0  0  0
 62 67  1  0  0  0  0
 64 66  1  0  0  0  0
 64 68  1  0  0  0  0
 66 69  1  0  0  0  0
 72 73  2  0  0  0  0
 74 76  1  0  0  0  0
 74 77  1  0  0  0  0
 74 85  1  0  0  0  0
 74 90  1  0  0  0  0
 75 78  1  0  0  0  0
 75 79  1  0  0  0  0
 75 88  1  0  0  0  0
 75 90  1  0  0  0  0
 76 80  1  0  0  0  0
 76 81  1  0  0  0  0
 76 86  1  0  0  0  0
 78 80  1  0  0  0  0
 78 82  1  0  0  0  0
 78 89  1  0  0  0  0
 80 83  1  0  0  0  0
 80 87  1  0  0  0  0
 84 90  1  0  0  0  0
M  END
$$$$
mol007
Optimized Catalyst 007
Optimized Catalyst 007
105113  0  0  0  0  0  0  0  0999 V2000
   -0.3527    0.0688    0.3686 Ru  0  0  0  0  0  0  0  0  0  0  0  0
   -1.1422    0.1002   -1.4788 H   0  0  0  0  0  0  0  0  0  0  0  0
    1.6976    0.1114   -0.4570 P   0  0  0  0  0  0  0  0  0  0  0  0
   -0.7265   -2.2387    0.3404 P   0  0  0  0  0  0  0  0  0  0  0  0
   -0.8145    2.3799    0.3771 P   0  0  0  0  0  0  0  0  0  0  0  0
    0.3676   -0.1714    3.1122 Cl  0  0  0  0  0  0  0  0  0  0  0  0
    2.7504    1.5205    0.1871 C   0  0  0  0  0  0  0  0  0  0  0  0
    3.6357    2.2150   -0.6432 C   0  0  0  0  0  0  0  0  0  0  0  0
    2.6241    1.8656    1.5325 C   0  0  0  0  0  0  0  0  0  0  0  0
    4.3948    3.2659   -0.1195 C   0  0  0  0  0  0  0  0  0  0  0  0
    3.7475    1.9793   -1.7027 H   0  0  0  0  0  0  0  0  0  0  0  0
    3.3857    2.9213    2.0526 C   0  0  0  0  0  0  0  0  0  0  0  0
    1.9266    1.3382    2.2142 H   0  0  0  0  0  0  0  0  0  0  0  0
```

```
    4.2691    3.6208    1.2296 C   0  0  0  0  0  0  0  0  0  0  0  0
    5.0849    3.8095   -0.7616 H   0  0  0  0  0  0  0  0  0  0  0  0
    3.2694    3.1964    3.1019 H   0  0  0  0  0  0  0  0  0  0  0  0
    4.8588    4.4432    1.6336 H   0  0  0  0  0  0  0  0  0  0  0  0
    2.6498   -1.4302   -0.0000 C   0  0  0  0  0  0  0  0  0  0  0  0
    2.8963   -2.4371   -0.9384 C   0  0  0  0  0  0  0  0  0  0  0  0
    3.0955   -1.5572    1.3167 C   0  0  0  0  0  0  0  0  0  0  0  0
    3.6833   -3.5349   -0.5787 C   0  0  0  0  0  0  0  0  0  0  0  0
    2.4738   -2.4068   -1.9455 H   0  0  0  0  0  0  0  0  0  0  0  0
    3.8784   -2.6644    1.6734 C   0  0  0  0  0  0  0  0  0  0  0  0
    2.8181   -0.8348    2.0994 H   0  0  0  0  0  0  0  0  0  0  0  0
    4.1896   -3.6391    0.7235 C   0  0  0  0  0  0  0  0  0  0  0  0
    3.8948   -4.3140   -1.3081 H   0  0  0  0  0  0  0  0  0  0  0  0
    4.2237   -2.7679    2.7022 H   0  0  0  0  0  0  0  0  0  0  0  0
    4.8072   -4.4929    1.0000 H   0  0  0  0  0  0  0  0  0  0  0  0
    1.9312    0.2540   -2.3187 C   0  0  0  0  0  0  0  0  0  0  0  0
    3.1612   -0.1039   -2.8827 C   0  0  0  0  0  0  0  0  0  0  0  0
    0.9153    0.7664   -3.1223 C   0  0  0  0  0  0  0  0  0  0  0  0
    3.3589    0.0299   -4.2605 C   0  0  0  0  0  0  0  0  0  0  0  0
    3.9879   -0.4840   -2.2729 H   0  0  0  0  0  0  0  0  0  0  0  0
    1.1181    0.9107   -4.5012 C   0  0  0  0  0  0  0  0  0  0  0  0
   -0.0545    1.0721   -2.7037 H   0  0  0  0  0  0  0  0  0  0  0  0
    2.3370    0.5376   -5.0712 C   0  0  0  0  0  0  0  0  0  0  0  0
    4.3123   -0.2576   -4.7023 H   0  0  0  0  0  0  0  0  0  0  0  0
    0.3230    1.3231   -5.1218 H   0  0  0  0  0  0  0  0  0  0  0  0
    2.4954    0.6451   -6.1443 H   0  0  0  0  0  0  0  0  0  0  0  0
   -0.1715    3.5688    1.6728 C   0  0  0  0  0  0  0  0  0  0  0  0
    0.2125    4.8764    1.3640 C   0  0  0  0  0  0  0  0  0  0  0  0
   -0.1221    3.1027    2.9872 C   0  0  0  0  0  0  0  0  0  0  0  0
    0.6973    5.7092    2.3775 C   0  0  0  0  0  0  0  0  0  0  0  0
    0.1368    5.2790    0.3525 H   0  0  0  0  0  0  0  0  0  0  0  0
    0.3614    3.9430    3.9992 C   0  0  0  0  0  0  0  0  0  0  0  0
   -0.4444    2.0815    3.2568 H   0  0  0  0  0  0  0  0  0  0  0  0
    0.7787    5.2401    3.6942 C   0  0  0  0  0  0  0  0  0  0  0  0
    1.0097    6.7244    2.1424 H   0  0  0  0  0  0  0  0  0  0  0  0
    0.4144    3.5710    5.0225 H   0  0  0  0  0  0  0  0  0  0  0  0
    1.1626    5.8893    4.4799 H   0  0  0  0  0  0  0  0  0  0  0  0
   -2.6757    2.5519    0.5902 C   0  0  0  0  0  0  0  0  0  0  0  0
   -3.5025    1.4799    0.2507 C   0  0  0  0  0  0  0  0  0  0  0  0
   -3.2167    3.7393    1.0892 C   0  0  0  0  0  0  0  0  0  0  0  0
   -4.8895    1.5995    0.4092 C   0  0  0  0  0  0  0  0  0  0  0  0
   -3.1021    0.5321   -0.1350 H   0  0  0  0  0  0  0  0  0  0  0  0
   -4.6030    3.8548    1.2438 C   0  0  0  0  0  0  0  0  0  0  0  0
   -2.5830    4.5845    1.3708 H   0  0  0  0  0  0  0  0  0  0  0  0
   -5.4387    2.7855    0.9041 C   0  0  0  0  0  0  0  0  0  0  0  0
   -5.5350    0.7590    0.1558 H   0  0  0  0  0  0  0  0  0  0  0  0
   -5.0300    4.7771    1.6347 H   0  0  0  0  0  0  0  0  0  0  0  0
   -6.5171    2.8763    1.0295 H   0  0  0  0  0  0  0  0  0  0  0  0
   -0.5038    3.2561   -1.2603 C   0  0  0  0  0  0  0  0  0  0  0  0
   -1.5375    3.3821   -2.1903 C   0  0  0  0  0  0  0  0  0  0  0  0
    0.7764    3.7306   -1.5546 C   0  0  0  0  0  0  0  0  0  0  0  0
   -1.2818    3.9754   -3.4329 C   0  0  0  0  0  0  0  0  0  0  0  0
   -2.5499    3.0296   -1.9744 H   0  0  0  0  0  0  0  0  0  0  0  0
    1.0284    4.3210   -2.7980 C   0  0  0  0  0  0  0  0  0  0  0  0
    1.5971    3.6583   -0.8336 H   0  0  0  0  0  0  0  0  0  0  0  0
    0.0004    4.4412   -3.7386 C   0  0  0  0  0  0  0  0  0  0  0  0
   -2.0863    4.0769   -4.1598 H   0  0  0  0  0  0  0  0  0  0  0  0
    2.0282    4.6845   -3.0311 H   0  0  0  0  0  0  0  0  0  0  0  0
    0.1982    4.8999   -4.7065 H   0  0  0  0  0  0  0  0  0  0  0  0
    0.0751   -3.4037    1.5657 C   0  0  0  0  0  0  0  0  0  0  0  0
    0.9662   -4.3863    1.1247 C   0  0  0  0  0  0  0  0  0  0  0  0
   -0.1905   -3.2476    2.9273 C   0  0  0  0  0  0  0  0  0  0  0  0
    1.5709   -5.2414    2.0510 C   0  0  0  0  0  0  0  0  0  0  0  0
    1.2281   -4.4975    0.0702 H   0  0  0  0  0  0  0  0  0  0  0  0
    0.4288   -4.1004    3.8529 C   0  0  0  0  0  0  0  0  0  0  0  0
```

```
   -0.8569   -2.4646    3.3083 H   0  0  0  0  0  0  0  0  0  0  0  0
    1.3026   -5.0977    3.4171 C   0  0  0  0  0  0  0  0  0  0  0  0
    2.2606   -6.0091    1.7091 H   0  0  0  0  0  0  0  0  0  0  0  0
    0.2341   -3.9656    4.9162 H   0  0  0  0  0  0  0  0  0  0  0  0
    1.7807   -5.7582    4.1384 H   0  0  0  0  0  0  0  0  0  0  0  0
   -0.7290   -3.2498   -1.2467 C   0  0  0  0  0  0  0  0  0  0  0  0
   -0.4114   -2.6372   -2.4568 C   0  0  0  0  0  0  0  0  0  0  0  0
   -1.0367   -4.6141   -1.2043 C   0  0  0  0  0  0  0  0  0  0  0  0
   -0.3773   -3.3952   -3.6352 C   0  0  0  0  0  0  0  0  0  0  0  0
   -0.1957   -1.5668   -2.5203 H   0  0  0  0  0  0  0  0  0  0  0  0
   -1.0086   -5.3678   -2.3821 C   0  0  0  0  0  0  0  0  0  0  0  0
   -1.2961   -5.1152   -0.2662 H   0  0  0  0  0  0  0  0  0  0  0  0
   -0.6736   -4.7596   -3.5974 C   0  0  0  0  0  0  0  0  0  0  0  0
   -0.1205   -2.9157   -4.5788 H   0  0  0  0  0  0  0  0  0  0  0  0
   -1.2461   -6.4304   -2.3524 H   0  0  0  0  0  0  0  0  0  0  0  0
   -0.6467   -5.3505   -4.5126 H   0  0  0  0  0  0  0  0  0  0  0  0
   -2.5334   -2.1638    0.8570 C   0  0  0  0  0  0  0  0  0  0  0  0
   -3.5306   -2.9044    0.2206 C   0  0  0  0  0  0  0  0  0  0  0  0
   -2.8482   -1.2903    1.9037 C   0  0  0  0  0  0  0  0  0  0  0  0
   -4.8577   -2.7881    0.6510 C   0  0  0  0  0  0  0  0  0  0  0  0
   -3.3099   -3.5711   -0.6145 H   0  0  0  0  0  0  0  0  0  0  0  0
   -4.1783   -1.1841    2.3325 C   0  0  0  0  0  0  0  0  0  0  0  0
   -2.0845   -0.6758    2.4219 H   0  0  0  0  0  0  0  0  0  0  0  0
   -5.1805   -1.9335    1.7103 C   0  0  0  0  0  0  0  0  0  0  0  0
   -5.6387   -3.3671    0.1617 H   0  0  0  0  0  0  0  0  0  0  0  0
   -4.4261   -0.5079    3.1508 H   0  0  0  0  0  0  0  0  0  0  0  0
   -6.2123   -1.8511    2.0493 H   0  0  0  0  0  0  0  0  0  0  0  0
  1  3  1  0  0  0  0
  1  4  1  0  0  0  0
  1  5  1  0  0  0  0
  1  6  1  0  0  0  0
  1  2  1  0  0  0  0
  3  7  1  0  0  0  0
  3 18  1  0  0  0  0
  3 29  1  0  0  0  0
  4 73  1  0  0  0  0
  4 84  1  0  0  0  0
  4 95  1  0  0  0  0
  5 40  1  0  0  0  0
  5 51  1  0  0  0  0
  5 62  1  0  0  0  0
  7  8  2  0  0  0  0
  7  9  1  0  0  0  0
  8 10  1  0  0  0  0
  8 11  1  0  0  0  0
  9 12  2  0  0  0  0
  9 13  1  0  0  0  0
 10 14  2  0  0  0  0
 10 15  1  0  0  0  0
 12 14  1  0  0  0  0
 12 16  1  0  0  0  0
 14 17  1  0  0  0  0
 18 19  2  0  0  0  0
 18 20  1  0  0  0  0
 19 21  1  0  0  0  0
 19 22  1  0  0  0  0
 20 23  2  0  0  0  0
 20 24  1  0  0  0  0
 21 25  2  0  0  0  0
 21 26  1  0  0  0  0
 23 25  1  0  0  0  0
 23 27  1  0  0  0  0
 25 28  1  0  0  0  0
 29 30  2  0  0  0  0
 29 31  1  0  0  0  0
```

```
 30 32  1  0  0  0  0
 30 33  1  0  0  0  0
 31 34  2  0  0  0  0
 31 35  1  0  0  0  0
 32 36  2  0  0  0  0
 32 37  1  0  0  0  0
 34 36  1  0  0  0  0
 34 38  1  0  0  0  0
 36 39  1  0  0  0  0
 40 41  2  0  0  0  0
 40 42  1  0  0  0  0
 41 43  1  0  0  0  0
 41 44  1  0  0  0  0
 42 45  2  0  0  0  0
 42 46  1  0  0  0  0
 43 47  2  0  0  0  0
 43 48  1  0  0  0  0
 45 47  1  0  0  0  0
 45 49  1  0  0  0  0
 47 50  1  0  0  0  0
 51 52  2  0  0  0  0
 51 53  1  0  0  0  0
 52 54  1  0  0  0  0
 52 55  1  0  0  0  0
 53 56  2  0  0  0  0
 53 57  1  0  0  0  0
 54 58  2  0  0  0  0
 54 59  1  0  0  0  0
 56 58  1  0  0  0  0
 56 60  1  0  0  0  0
 58 61  1  0  0  0  0
 62 63  2  0  0  0  0
 62 64  1  0  0  0  0
 63 65  1  0  0  0  0
 63 66  1  0  0  0  0
 64 67  2  0  0  0  0
 64 68  1  0  0  0  0
 65 69  2  0  0  0  0
 65 70  1  0  0  0  0
 67 69  1  0  0  0  0
 67 71  1  0  0  0  0
 69 72  1  0  0  0  0
 73 74  2  0  0  0  0
 73 75  1  0  0  0  0
 74 76  1  0  0  0  0
 74 77  1  0  0  0  0
 75 78  2  0  0  0  0
 75 79  1  0  0  0  0
 76 80  2  0  0  0  0
 76 81  1  0  0  0  0
 78 80  1  0  0  0  0
 78 82  1  0  0  0  0
 80 83  1  0  0  0  0
 84 85  2  0  0  0  0
 84 86  1  0  0  0  0
 85 87  1  0  0  0  0
 85 88  1  0  0  0  0
 86 89  2  0  0  0  0
 86 90  1  0  0  0  0
 87 91  2  0  0  0  0
 87 92  1  0  0  0  0
 89 91  1  0  0  0  0
 89 93  1  0  0  0  0
 91 94  1  0  0  0  0
 95 96  2  0  0  0  0
```

```
 95 97  1  0  0  0  0
 96 98  1  0  0  0  0
 96 99  1  0  0  0  0
 97100  2  0  0  0  0
 97101  1  0  0  0  0
 98102  2  0  0  0  0
 98103  1  0  0  0  0
100102  1  0  0  0  0
100104  1  0  0  0  0
102105  1  0  0  0  0
M  END
$$$$
mol008
Optimized Catalyst 008
Optimized Catalyst 008
104112  0  0  0  0  0  0  0  0999 V2000
   -1.3837    0.4109   -0.1331 Rh  0  0  0  0  0  0  0  0  0  0  0  0
    0.8585    0.2634   -0.2206 P   0  0  0  0  0  0  0  0  0  0  0  0
   -1.4689    2.6679    0.1406 P   0  0  0  0  0  0  0  0  0  0  0  0
   -3.5714    0.0948    0.1184 P   0  0  0  0  0  0  0  0  0  0  0  0
   -1.4632   -1.9971   -1.1320 Cl  0  0  0  0  0  0  0  0  0  0  0  0
    1.5911    0.1893   -1.9347 C   0  0  0  0  0  0  0  0  0  0  0  0
    0.7596   -0.2335   -2.9725 C   0  0  0  0  0  0  0  0  0  0  0  0
    2.9283    0.5195   -2.1743 C   0  0  0  0  0  0  0  0  0  0  0  0
    1.2662   -0.2972   -4.2779 C   0  0  0  0  0  0  0  0  0  0  0  0
   -0.2863   -0.5363   -2.7792 H   0  0  0  0  0  0  0  0  0  0  0  0
    3.4254    0.4598   -3.4801 C   0  0  0  0  0  0  0  0  0  0  0  0
    3.6029    0.8220   -1.3706 H   0  0  0  0  0  0  0  0  0  0  0  0
    2.5938    0.0549   -4.5314 C   0  0  0  0  0  0  0  0  0  0  0  0
    0.6231   -0.6365   -5.0888 H   0  0  0  0  0  0  0  0  0  0  0  0
    4.4633    0.7208   -3.6781 H   0  0  0  0  0  0  0  0  0  0  0  0
    2.9870    0.0058   -5.5463 H   0  0  0  0  0  0  0  0  0  0  0  0
    1.9199    1.5264    0.6821 C   0  0  0  0  0  0  0  0  0  0  0  0
    2.2855    1.2824    2.0087 C   0  0  0  0  0  0  0  0  0  0  0  0
    2.3468    2.6871    0.0352 C   0  0  0  0  0  0  0  0  0  0  0  0
    3.0527    2.2266    2.7017 C   0  0  0  0  0  0  0  0  0  0  0  0
    1.9906    0.3649    2.5267 H   0  0  0  0  0  0  0  0  0  0  0  0
    3.1074    3.6327    0.7330 C   0  0  0  0  0  0  0  0  0  0  0  0
    2.1068    2.8810   -1.0149 H   0  0  0  0  0  0  0  0  0  0  0  0
    3.4553    3.4061    2.0679 C   0  0  0  0  0  0  0  0  0  0  0  0
    3.3324    2.0412    3.7375 H   0  0  0  0  0  0  0  0  0  0  0  0
    3.4276    4.5440    0.2308 H   0  0  0  0  0  0  0  0  0  0  0  0
    4.0433    4.1441    2.6119 H   0  0  0  0  0  0  0  0  0  0  0  0
    1.3364   -1.3233    0.6355 C   0  0  0  0  0  0  0  0  0  0  0  0
    2.6441   -1.8112    0.5620 C   0  0  0  0  0  0  0  0  0  0  0  0
    0.3443   -2.0091    1.3393 C   0  0  0  0  0  0  0  0  0  0  0  0
    2.9668   -2.9999    1.2240 C   0  0  0  0  0  0  0  0  0  0  0  0
    3.4204   -1.2938   -0.0022 H   0  0  0  0  0  0  0  0  0  0  0  0
    0.6764   -3.2029    1.9972 C   0  0  0  0  0  0  0  0  0  0  0  0
   -0.6996   -1.6416    1.3747 H   0  0  0  0  0  0  0  0  0  0  0  0
    1.9823   -3.6947    1.9386 C   0  0  0  0  0  0  0  0  0  0  0  0
    3.9800   -3.3926    1.1732 H   0  0  0  0  0  0  0  0  0  0  0  0
   -0.0955   -3.7552    2.5308 H   0  0  0  0  0  0  0  0  0  0  0  0
    2.2356   -4.6270    2.4422 H   0  0  0  0  0  0  0  0  0  0  0  0
   -0.9019    3.1957    1.8338 C   0  0  0  0  0  0  0  0  0  0  0  0
   -0.8575    4.5376    2.2248 C   0  0  0  0  0  0  0  0  0  0  0  0
   -0.5532    2.1821    2.7272 C   0  0  0  0  0  0  0  0  0  0  0  0
   -0.4255    4.8636    3.5141 C   0  0  0  0  0  0  0  0  0  0  0  0
   -1.1706    5.3464    1.5608 H   0  0  0  0  0  0  0  0  0  0  0  0
   -0.1309    2.5132    4.0222 C   0  0  0  0  0  0  0  0  0  0  0  0
   -0.6030    1.1194    2.4361 H   0  0  0  0  0  0  0  0  0  0  0  0
   -0.0588    3.8526    4.4109 C   0  0  0  0  0  0  0  0  0  0  0  0
   -0.3767    5.9071    3.8228 H   0  0  0  0  0  0  0  0  0  0  0  0
    0.1488    1.7233    4.7184 H   0  0  0  0  0  0  0  0  0  0  0  0
    0.2800    4.1125    5.4141 H   0  0  0  0  0  0  0  0  0  0  0  0
```

```
   -0.4224    3.5048   -1.1657 C   0  0  0  0  0  0  0  0  0  0  0  0
   -0.3503    2.8576   -2.4032 C   0  0  0  0  0  0  0  0  0  0  0  0
    0.2142    4.7303   -0.9669 C   0  0  0  0  0  0  0  0  0  0  0  0
    0.3742    3.4382   -3.4499 C   0  0  0  0  0  0  0  0  0  0  0  0
   -0.8371    1.8845   -2.5679 H   0  0  0  0  0  0  0  0  0  0  0  0
    0.9280    5.3140   -2.0219 C   0  0  0  0  0  0  0  0  0  0  0  0
    0.2012    5.2450   -0.0070 H   0  0  0  0  0  0  0  0  0  0  0  0
    1.0091    4.6698   -3.2602 C   0  0  0  0  0  0  0  0  0  0  0  0
    0.4521    2.9199   -4.4064 H   0  0  0  0  0  0  0  0  0  0  0  0
    1.4250    6.2718   -1.8732 H   0  0  0  0  0  0  0  0  0  0  0  0
    1.5709    5.1245   -4.0765 H   0  0  0  0  0  0  0  0  0  0  0  0
   -3.0847    3.5757   -0.1262 C   0  0  0  0  0  0  0  0  0  0  0  0
   -3.7403    4.2016    0.9352 C   0  0  0  0  0  0  0  0  0  0  0  0
   -3.5948    3.6587   -1.4249 C   0  0  0  0  0  0  0  0  0  0  0  0
   -4.9066    4.9376    0.6887 C   0  0  0  0  0  0  0  0  0  0  0  0
   -3.3890    4.1139    1.9676 H   0  0  0  0  0  0  0  0  0  0  0  0
   -4.7741    4.3733   -1.6615 C   0  0  0  0  0  0  0  0  0  0  0  0
   -3.1002    3.1736   -2.2715 H   0  0  0  0  0  0  0  0  0  0  0  0
   -5.4250    5.0211   -0.6065 C   0  0  0  0  0  0  0  0  0  0  0  0
   -5.4146    5.4345    1.5139 H   0  0  0  0  0  0  0  0  0  0  0  0
   -5.1833    4.4214   -2.6704 H   0  0  0  0  0  0  0  0  0  0  0  0
   -6.3368    5.5877   -0.7945 H   0  0  0  0  0  0  0  0  0  0  0  0
   -4.1286   -1.6482    0.5002 C   0  0  0  0  0  0  0  0  0  0  0  0
   -4.4209   -2.0551    1.8046 C   0  0  0  0  0  0  0  0  0  0  0  0
   -4.2709   -2.5351   -0.5734 C   0  0  0  0  0  0  0  0  0  0  0  0
   -4.8650   -3.3613    2.0374 C   0  0  0  0  0  0  0  0  0  0  0  0
   -4.3204   -1.3860    2.6592 H   0  0  0  0  0  0  0  0  0  0  0  0
   -4.7133   -3.8432   -0.3302 C   0  0  0  0  0  0  0  0  0  0  0  0
   -4.0435   -2.2412   -1.6052 H   0  0  0  0  0  0  0  0  0  0  0  0
   -5.0073   -4.2563    0.9710 C   0  0  0  0  0  0  0  0  0  0  0  0
   -5.0947   -3.6826    3.0510 H   0  0  0  0  0  0  0  0  0  0  0  0
   -4.8077   -4.5404   -1.1614 H   0  0  0  0  0  0  0  0  0  0  0  0
   -5.3433   -5.2755    1.1570 H   0  0  0  0  0  0  0  0  0  0  0  0
   -4.4755    0.4731   -1.4693 C   0  0  0  0  0  0  0  0  0  0  0  0
   -5.8668    0.5155   -1.5707 C   0  0  0  0  0  0  0  0  0  0  0  0
   -3.6679    0.6252   -2.5994 C   0  0  0  0  0  0  0  0  0  0  0  0
   -6.4522    0.8181   -2.8058 C   0  0  0  0  0  0  0  0  0  0  0  0
   -6.5150    0.2880   -0.7224 H   0  0  0  0  0  0  0  0  0  0  0  0
   -4.2589    0.9178   -3.8349 C   0  0  0  0  0  0  0  0  0  0  0  0
   -2.5735    0.4770   -2.5317 H   0  0  0  0  0  0  0  0  0  0  0  0
   -5.6490    1.0327   -3.9317 C   0  0  0  0  0  0  0  0  0  0  0  0
   -7.5361    0.8722   -2.8932 H   0  0  0  0  0  0  0  0  0  0  0  0
   -3.6358    1.0411   -4.7192 H   0  0  0  0  0  0  0  0  0  0  0  0
   -6.1099    1.2704   -4.8903 H   0  0  0  0  0  0  0  0  0  0  0  0
   -4.3082    1.0827    1.5321 C   0  0  0  0  0  0  0  0  0  0  0  0
   -5.4860    1.8210    1.4422 C   0  0  0  0  0  0  0  0  0  0  0  0
   -3.6107    1.0054    2.7438 C   0  0  0  0  0  0  0  0  0  0  0  0
   -5.9919    2.4571    2.5860 C   0  0  0  0  0  0  0  0  0  0  0  0
   -6.0177    1.9559    0.4992 H   0  0  0  0  0  0  0  0  0  0  0  0
   -4.1067    1.6572    3.8765 C   0  0  0  0  0  0  0  0  0  0  0  0
   -2.6739    0.4394    2.8215 H   0  0  0  0  0  0  0  0  0  0  0  0
   -5.3066    2.3744    3.7999 C   0  0  0  0  0  0  0  0  0  0  0  0
   -6.9201    3.0229    2.5201 H   0  0  0  0  0  0  0  0  0  0  0  0
   -3.5599    1.6048    4.8170 H   0  0  0  0  0  0  0  0  0  0  0  0
   -5.7022    2.8695    4.6862 H   0  0  0  0  0  0  0  0  0  0  0  0
  1  2  1  0  0  0  0
  1  3  1  0  0  0  0
  1  4  1  0  0  0  0
  1  5  1  0  0  0  0
  2  6  1  0  0  0  0
  2 17  1  0  0  0  0
  2 28  1  0  0  0  0
  3 39  1  0  0  0  0
  3 50  1  0  0  0  0
  3 61  1  0  0  0  0
```

```
  4 72  1  0  0  0  0
  4 83  1  0  0  0  0
  4 94  1  0  0  0  0
  6  7  2  0  0  0  0
  6  8  1  0  0  0  0
  7  9  1  0  0  0  0
  7 10  1  0  0  0  0
  8 11  2  0  0  0  0
  8 12  1  0  0  0  0
  9 13  2  0  0  0  0
  9 14  1  0  0  0  0
 11 13  1  0  0  0  0
 11 15  1  0  0  0  0
 13 16  1  0  0  0  0
 17 18  2  0  0  0  0
 17 19  1  0  0  0  0
 18 20  1  0  0  0  0
 18 21  1  0  0  0  0
 19 22  2  0  0  0  0
 19 23  1  0  0  0  0
 20 24  2  0  0  0  0
 20 25  1  0  0  0  0
 22 24  1  0  0  0  0
 22 26  1  0  0  0  0
 24 27  1  0  0  0  0
 28 29  2  0  0  0  0
 28 30  1  0  0  0  0
 29 31  1  0  0  0  0
 29 32  1  0  0  0  0
 30 33  2  0  0  0  0
 30 34  1  0  0  0  0
 31 35  2  0  0  0  0
 31 36  1  0  0  0  0
 33 35  1  0  0  0  0
 33 37  1  0  0  0  0
 35 38  1  0  0  0  0
 39 40  2  0  0  0  0
 39 41  1  0  0  0  0
 40 42  1  0  0  0  0
 40 43  1  0  0  0  0
 41 44  2  0  0  0  0
 41 45  1  0  0  0  0
 42 46  2  0  0  0  0
 42 47  1  0  0  0  0
 44 46  1  0  0  0  0
 44 48  1  0  0  0  0
 46 49  1  0  0  0  0
 50 51  2  0  0  0  0
 50 52  1  0  0  0  0
 51 53  1  0  0  0  0
 51 54  1  0  0  0  0
 52 55  2  0  0  0  0
 52 56  1  0  0  0  0
 53 57  2  0  0  0  0
 53 58  1  0  0  0  0
 55 57  1  0  0  0  0
 55 59  1  0  0  0  0
 57 60  1  0  0  0  0
 61 62  2  0  0  0  0
 61 63  1  0  0  0  0
 62 64  1  0  0  0  0
 62 65  1  0  0  0  0
 63 66  2  0  0  0  0
 63 67  1  0  0  0  0
 64 68  2  0  0  0  0
```

```
 64 69  1  0  0  0  0
 66 68  1  0  0  0  0
 66 70  1  0  0  0  0
 68 71  1  0  0  0  0
 72 73  2  0  0  0  0
 72 74  1  0  0  0  0
 73 75  1  0  0  0  0
 73 76  1  0  0  0  0
 74 77  2  0  0  0  0
 74 78  1  0  0  0  0
 75 79  2  0  0  0  0
 75 80  1  0  0  0  0
 77 79  1  0  0  0  0
 77 81  1  0  0  0  0
 79 82  1  0  0  0  0
 83 84  2  0  0  0  0
 83 85  1  0  0  0  0
 84 86  1  0  0  0  0
 84 87  1  0  0  0  0
 85 88  2  0  0  0  0
 85 89  1  0  0  0  0
 86 90  2  0  0  0  0
 86 91  1  0  0  0  0
 88 90  1  0  0  0  0
 88 92  1  0  0  0  0
 90 93  1  0  0  0  0
 94 95  2  0  0  0  0
 94 96  1  0  0  0  0
 95 97  1  0  0  0  0
 95 98  1  0  0  0  0
 96 99  2  0  0  0  0
 96100  1  0  0  0  0
 97101  2  0  0  0  0
 97102  1  0  0  0  0
 99101  1  0  0  0  0
 99103  1  0  0  0  0
101104  1  0  0  0  0
M  END
$$$$
mol009
Optimized Catalyst 009
Optimized Catalyst 009
 29 33  0  0  0  0  0  0  0  0999 V2000
    0.4134    1.3044   -0.2501 C   0  0  0  0  0  0  0  0  0  0  0  0
   -0.4812    2.4234   -0.0073 C   0  0  0  0  0  0  0  0  0  0  0  0
   -1.7993    1.9155    0.1271 C   0  0  0  0  0  0  0  0  0  0  0  0
   -1.7436    0.4689   -0.0192 C   0  0  0  0  0  0  0  0  0  0  0  0
   -0.3699    0.1038   -0.1866 C   0  0  0  0  0  0  0  0  0  0  0  0
    1.4831    1.3684   -0.3728 H   0  0  0  0  0  0  0  0  0  0  0  0
   -0.1803    3.4609    0.0464 H   0  0  0  0  0  0  0  0  0  0  0  0
   -2.6982    2.4885    0.3073 H   0  0  0  0  0  0  0  0  0  0  0  0
   -2.5843   -0.2028    0.0594 H   0  0  0  0  0  0  0  0  0  0  0  0
    0.0091   -0.9045   -0.3003 H   0  0  0  0  0  0  0  0  0  0  0  0
   -1.0247    1.3667   -1.8019 Rh  0  0  0  0  0  0  0  0  0  0  0  0
   -0.2769    2.5595   -2.7565 N   0  0  0  0  0  0  0  0  0  0  0  0
   -0.6098    0.1302   -2.8890 N   0  0  0  0  0  0  0  0  0  0  0  0
   -2.5466    1.6003   -2.5259 N   0  0  0  0  0  0  0  0  0  0  0  0
    0.2569    3.3893   -3.3868 C   0  0  0  0  0  0  0  0  0  0  0  0
   -0.3176   -0.7316   -3.6261 C   0  0  0  0  0  0  0  0  0  0  0  0
   -3.6094    1.7561   -2.9916 C   0  0  0  0  0  0  0  0  0  0  0  0
    0.0520   -1.8092   -4.4988 C   0  0  0  0  0  0  0  0  0  0  0  0
    1.1419   -2.0703   -4.3745 H   0  0  0  0  0  0  0  0  0  0  0  0
   -0.5566   -2.7321   -4.2765 H   0  0  0  0  0  0  0  0  0  0  0  0
   -0.1199   -1.5282   -5.5769 H   0  0  0  0  0  0  0  0  0  0  0  0
    0.9229    4.4200   -4.1312 C   0  0  0  0  0  0  0  0  0  0  0  0
```

```
    0.2501    5.3140   -4.2690 H   0  0  0  0  0  0  0  0  0  0  0  0
    1.8547    4.7622   -3.5962 H   0  0  0  0  0  0  0  0  0  0  0  0
    1.2229    4.0468   -5.1518 H   0  0  0  0  0  0  0  0  0  0  0  0
   -4.9244    1.9497   -3.5331 C   0  0  0  0  0  0  0  0  0  0  0  0
   -5.6133    1.1132   -3.2212 H   0  0  0  0  0  0  0  0  0  0  0  0
   -5.3681    2.9212   -3.1719 H   0  0  0  0  0  0  0  0  0  0  0  0
   -4.8937    1.9762   -4.6596 H   0  0  0  0  0  0  0  0  0  0  0  0
  1  2  1  0  0  0  0
  1  5  1  0  0  0  0
  1  6  1  0  0  0  0
  1 11  1  0  0  0  0
  2  3  2  0  0  0  0
  2  7  1  0  0  0  0
  2 11  1  0  0  0  0
  3  4  1  0  0  0  0
  3  8  1  0  0  0  0
  3 11  1  0  0  0  0
  4  5  2  0  0  0  0
  4  9  1  0  0  0  0
  4 11  1  0  0  0  0
  5 10  1  0  0  0  0
  5 11  1  0  0  0  0
 11 12  1  0  0  0  0
 11 13  1  0  0  0  0
 11 14  1  0  0  0  0
 12 15  3  0  0  0  0
 13 16  3  0  0  0  0
 14 17  3  0  0  0  0
 15 22  1  0  0  0  0
 16 18  1  0  0  0  0
 17 26  1  0  0  0  0
 18 19  1  0  0  0  0
 18 20  1  0  0  0  0
 18 21  1  0  0  0  0
 22 23  1  0  0  0  0
 22 24  1  0  0  0  0
 22 25  1  0  0  0  0
 26 27  1  0  0  0  0
 26 28  1  0  0  0  0
 26 29  1  0  0  0  0
M  END
$$$$
mol010
Optimized Catalyst 010
Optimized Catalyst 010
105114  0  0  0  0  0  0  0  0999 V2000
    3.4302    0.7432    0.2138 C   0  0  0  0  0  0  0  0  0  0  0  0
    3.3708   -0.7115   -0.1888 C   0  0  0  0  0  0  0  0  0  0  0  0
    1.6705   -1.5190    0.2001 P   0  0  0  0  0  0  0  0  0  0  0  0
    0.0648    0.0579    0.1116 Rh  0  0  0  0  0  0  0  0  0  0  0  0
   -1.5296    1.6156   -0.2214 P   0  0  0  0  0  0  0  0  0  0  0  0
   -3.0664    0.9639    0.7061 C   0  0  0  0  0  0  0  0  0  0  0  0
   -3.3035   -0.4604    0.2512 C   0  0  0  0  0  0  0  0  0  0  0  0
   -1.6392   -1.4248    0.2781 P   0  0  0  0  0  0  0  0  0  0  0  0
    1.7056    1.5829    0.1238 P   0  0  0  0  0  0  0  0  0  0  0  0
   -3.9526    1.6060    0.4797 H   0  0  0  0  0  0  0  0  0  0  0  0
   -3.7424   -0.4995   -0.7761 H   0  0  0  0  0  0  0  0  0  0  0  0
    3.5363   -0.8266   -1.2924 H   0  0  0  0  0  0  0  0  0  0  0  0
    4.1300    1.3032   -0.4576 H   0  0  0  0  0  0  0  0  0  0  0  0
   -2.8707    1.0177    1.8081 H   0  0  0  0  0  0  0  0  0  0  0  0
   -4.0142   -0.9753    0.9472 H   0  0  0  0  0  0  0  0  0  0  0  0
    4.1673   -1.2960    0.3377 H   0  0  0  0  0  0  0  0  0  0  0  0
    3.8033    0.8579    1.2638 H   0  0  0  0  0  0  0  0  0  0  0  0
    1.8043    2.6999   -1.3554 C   0  0  0  0  0  0  0  0  0  0  0  0
    1.5386    4.0660   -1.2653 C   0  0  0  0  0  0  0  0  0  0  0  0
```

```
    2.1500    2.1166   -2.5807 C   0  0  0  0  0  0  0  0  0  0  0  0
    1.6351    4.8635   -2.4139 C   0  0  0  0  0  0  0  0  0  0  0  0
    1.2367    4.5398   -0.3260 H   0  0  0  0  0  0  0  0  0  0  0  0
    2.2433    2.9177   -3.7224 C   0  0  0  0  0  0  0  0  0  0  0  0
    2.3464    1.0455   -2.6731 H   0  0  0  0  0  0  0  0  0  0  0  0
    1.9894    4.2925   -3.6384 C   0  0  0  0  0  0  0  0  0  0  0  0
    1.4312    5.9330   -2.3477 H   0  0  0  0  0  0  0  0  0  0  0  0
    2.5154    2.4732   -4.6806 H   0  0  0  0  0  0  0  0  0  0  0  0
    2.0678    4.9163   -4.5313 H   0  0  0  0  0  0  0  0  0  0  0  0
    1.7386    2.5869    1.6867 C   0  0  0  0  0  0  0  0  0  0  0  0
    1.0082    2.0830    2.7664 C   0  0  0  0  0  0  0  0  0  0  0  0
    2.5225    3.7347    1.8204 C   0  0  0  0  0  0  0  0  0  0  0  0
    1.0361    2.7579    3.9922 C   0  0  0  0  0  0  0  0  0  0  0  0
    0.4123    1.1637    2.6659 H   0  0  0  0  0  0  0  0  0  0  0  0
    2.5351    4.4127    3.0453 C   0  0  0  0  0  0  0  0  0  0  0  0
    3.1378    4.1169    1.0023 H   0  0  0  0  0  0  0  0  0  0  0  0
    1.7917    3.9277    4.1268 C   0  0  0  0  0  0  0  0  0  0  0  0
    0.4710    2.3732    4.8414 H   0  0  0  0  0  0  0  0  0  0  0  0
    3.1329    5.3188    3.1587 H   0  0  0  0  0  0  0  0  0  0  0  0
    1.8079    4.4597    5.0806 H   0  0  0  0  0  0  0  0  0  0  0  0
    1.8771   -2.3771    1.8381 C   0  0  0  0  0  0  0  0  0  0  0  0
    1.3779   -3.6687    2.0265 C   0  0  0  0  0  0  0  0  0  0  0  0
    2.5226   -1.7047    2.8808 C   0  0  0  0  0  0  0  0  0  0  0  0
    1.5534   -4.3046    3.2616 C   0  0  0  0  0  0  0  0  0  0  0  0
    0.8406   -4.2021    1.2401 H   0  0  0  0  0  0  0  0  0  0  0  0
    2.6954   -2.3461    4.1126 C   0  0  0  0  0  0  0  0  0  0  0  0
    2.8914   -0.6836    2.7710 H   0  0  0  0  0  0  0  0  0  0  0  0
    2.2167   -3.6471    4.3019 C   0  0  0  0  0  0  0  0  0  0  0  0
    1.1666   -5.3133    3.4127 H   0  0  0  0  0  0  0  0  0  0  0  0
    3.2068   -1.8309    4.9269 H   0  0  0  0  0  0  0  0  0  0  0  0
    2.3583   -4.1468    5.2621 H   0  0  0  0  0  0  0  0  0  0  0  0
    1.6558   -2.8214   -1.1280 C   0  0  0  0  0  0  0  0  0  0  0  0
    1.0017   -2.5200   -2.3245 C   0  0  0  0  0  0  0  0  0  0  0  0
    2.3724   -4.0123   -0.9825 C   0  0  0  0  0  0  0  0  0  0  0  0
    1.0389   -3.4371   -3.3818 C   0  0  0  0  0  0  0  0  0  0  0  0
    0.4492   -1.5813   -2.4492 H   0  0  0  0  0  0  0  0  0  0  0  0
    2.3956   -4.9303   -2.0384 C   0  0  0  0  0  0  0  0  0  0  0  0
    2.9278   -4.2490   -0.0713 H   0  0  0  0  0  0  0  0  0  0  0  0
    1.7285   -4.6446   -3.2350 C   0  0  0  0  0  0  0  0  0  0  0  0
    0.5208   -3.2131   -4.3144 H   0  0  0  0  0  0  0  0  0  0  0  0
    2.9427   -5.8682   -1.9313 H   0  0  0  0  0  0  0  0  0  0  0  0
    1.7511   -5.3636   -4.0562 H   0  0  0  0  0  0  0  0  0  0  0  0
   -1.7452   -2.2723    1.9294 C   0  0  0  0  0  0  0  0  0  0  0  0
   -1.0099   -1.7286    2.9839 C   0  0  0  0  0  0  0  0  0  0  0  0
   -2.5988   -3.3604    2.1348 C   0  0  0  0  0  0  0  0  0  0  0  0
   -1.1027   -2.3017    4.2587 C   0  0  0  0  0  0  0  0  0  0  0  0
   -0.3504   -0.8644    2.8295 H   0  0  0  0  0  0  0  0  0  0  0  0
   -2.6813   -3.9333    3.4081 C   0  0  0  0  0  0  0  0  0  0  0  0
   -3.2131   -3.7758    1.3318 H   0  0  0  0  0  0  0  0  0  0  0  0
   -1.9322   -3.4071    4.4675 C   0  0  0  0  0  0  0  0  0  0  0  0
   -0.5197   -1.8909    5.0832 H   0  0  0  0  0  0  0  0  0  0  0  0
   -3.3369   -4.7887    3.5785 H   0  0  0  0  0  0  0  0  0  0  0  0
   -2.0007   -3.8589    5.4592 H   0  0  0  0  0  0  0  0  0  0  0  0
   -1.8421   -2.7178   -1.0438 C   0  0  0  0  0  0  0  0  0  0  0  0
   -2.3261   -2.3259   -2.2968 C   0  0  0  0  0  0  0  0  0  0  0  0
   -1.5172   -4.0529   -0.7931 C   0  0  0  0  0  0  0  0  0  0  0  0
   -2.5198   -3.2886   -3.2932 C   0  0  0  0  0  0  0  0  0  0  0  0
   -2.5462   -1.2814   -2.5356 H   0  0  0  0  0  0  0  0  0  0  0  0
   -1.7086   -5.0110   -1.7965 C   0  0  0  0  0  0  0  0  0  0  0  0
   -1.1074   -4.3797    0.1644 H   0  0  0  0  0  0  0  0  0  0  0  0
   -2.2170   -4.6315   -3.0422 C   0  0  0  0  0  0  0  0  0  0  0  0
   -2.9081   -2.9911   -4.2680 H   0  0  0  0  0  0  0  0  0  0  0  0
   -1.4571   -6.0549   -1.6053 H   0  0  0  0  0  0  0  0  0  0  0  0
   -2.3739   -5.3812   -3.8198 H   0  0  0  0  0  0  0  0  0  0  0  0
   -1.4248    3.4104    0.2473 C   0  0  0  0  0  0  0  0  0  0  0  0
```

```
   -1.4111    4.3890   -0.7500 C   0  0  0  0  0  0  0  0  0  0  0  0
   -1.3945    3.7685    1.5979 C   0  0  0  0  0  0  0  0  0  0  0  0
   -1.3636    5.7412   -0.3883 C   0  0  0  0  0  0  0  0  0  0  0  0
   -1.4254    4.1335   -1.8145 H   0  0  0  0  0  0  0  0  0  0  0  0
   -1.3333    5.1204    1.9524 C   0  0  0  0  0  0  0  0  0  0  0  0
   -1.4114    3.0218    2.3946 H   0  0  0  0  0  0  0  0  0  0  0  0
   -1.3215    6.1068    0.9602 C   0  0  0  0  0  0  0  0  0  0  0  0
   -1.3604    6.5093   -1.1626 H   0  0  0  0  0  0  0  0  0  0  0  0
   -1.2965    5.4045    3.0050 H   0  0  0  0  0  0  0  0  0  0  0  0
   -1.2826    7.1616    1.2399 H   0  0  0  0  0  0  0  0  0  0  0  0
   -2.0038    1.5978   -2.0220 C   0  0  0  0  0  0  0  0  0  0  0  0
   -3.1957    2.1768   -2.4675 C   0  0  0  0  0  0  0  0  0  0  0  0
   -1.1249    0.9846   -2.9164 C   0  0  0  0  0  0  0  0  0  0  0  0
   -3.5070    2.1360   -3.8307 C   0  0  0  0  0  0  0  0  0  0  0  0
   -3.8922    2.6671   -1.7840 H   0  0  0  0  0  0  0  0  0  0  0  0
   -1.4395    0.9511   -4.2808 C   0  0  0  0  0  0  0  0  0  0  0  0
   -0.1876    0.5270   -2.5668 H   0  0  0  0  0  0  0  0  0  0  0  0
   -2.6297    1.5264   -4.7358 C   0  0  0  0  0  0  0  0  0  0  0  0
   -4.4348    2.5838   -4.1911 H   0  0  0  0  0  0  0  0  0  0  0  0
   -0.7549    0.4807   -4.9866 H   0  0  0  0  0  0  0  0  0  0  0  0
   -2.8759    1.5032   -5.7996 H   0  0  0  0  0  0  0  0  0  0  0  0
  1  2  1  0  0  0  0
  1  9  1  0  0  0  0
  1 13  1  0  0  0  0
  1 17  1  0  0  0  0
  2  3  1  0  0  0  0
  2 12  1  0  0  0  0
  2 16  1  0  0  0  0
  3  4  1  0  0  0  0
  3 40  1  0  0  0  0
  3 51  1  0  0  0  0
  4  5  1  0  0  0  0
  4  8  1  0  0  0  0
  4  9  1  0  0  0  0
  5  6  1  0  0  0  0
  5 84  1  0  0  0  0
  5 95  1  0  0  0  0
  6  7  1  0  0  0  0
  6 10  1  0  0  0  0
  6 14  1  0  0  0  0
  7  8  1  0  0  0  0
  7 11  1  0  0  0  0
  7 15  1  0  0  0  0
  8 62  1  0  0  0  0
  8 73  1  0  0  0  0
  9 18  1  0  0  0  0
  9 29  1  0  0  0  0
 18 19  2  0  0  0  0
 18 20  1  0  0  0  0
 19 21  1  0  0  0  0
 19 22  1  0  0  0  0
 20 23  2  0  0  0  0
 20 24  1  0  0  0  0
 21 25  2  0  0  0  0
 21 26  1  0  0  0  0
 23 25  1  0  0  0  0
 23 27  1  0  0  0  0
 25 28  1  0  0  0  0
 29 30  2  0  0  0  0
 29 31  1  0  0  0  0
 30 32  1  0  0  0  0
 30 33  1  0  0  0  0
 31 34  2  0  0  0  0
 31 35  1  0  0  0  0
 32 36  2  0  0  0  0
```

```
 32 37  1  0  0  0  0
 34 36  1  0  0  0  0
 34 38  1  0  0  0  0
 36 39  1  0  0  0  0
 40 41  2  0  0  0  0
 40 42  1  0  0  0  0
 41 43  1  0  0  0  0
 41 44  1  0  0  0  0
 42 45  2  0  0  0  0
 42 46  1  0  0  0  0
 43 47  2  0  0  0  0
 43 48  1  0  0  0  0
 45 47  1  0  0  0  0
 45 49  1  0  0  0  0
 47 50  1  0  0  0  0
 51 52  2  0  0  0  0
 51 53  1  0  0  0  0
 52 54  1  0  0  0  0
 52 55  1  0  0  0  0
 53 56  2  0  0  0  0
 53 57  1  0  0  0  0
 54 58  2  0  0  0  0
 54 59  1  0  0  0  0
 56 58  1  0  0  0  0
 56 60  1  0  0  0  0
 58 61  1  0  0  0  0
 62 63  2  0  0  0  0
 62 64  1  0  0  0  0
 63 65  1  0  0  0  0
 63 66  1  0  0  0  0
 64 67  2  0  0  0  0
 64 68  1  0  0  0  0
 65 69  2  0  0  0  0
 65 70  1  0  0  0  0
 67 69  1  0  0  0  0
 67 71  1  0  0  0  0
 69 72  1  0  0  0  0
 73 74  2  0  0  0  0
 73 75  1  0  0  0  0
 74 76  1  0  0  0  0
 74 77  1  0  0  0  0
 75 78  2  0  0  0  0
 75 79  1  0  0  0  0
 76 80  2  0  0  0  0
 76 81  1  0  0  0  0
 78 80  1  0  0  0  0
 78 82  1  0  0  0  0
 80 83  1  0  0  0  0
 84 85  2  0  0  0  0
 84 86  1  0  0  0  0
 85 87  1  0  0  0  0
 85 88  1  0  0  0  0
 86 89  2  0  0  0  0
 86 90  1  0  0  0  0
 87 91  2  0  0  0  0
 87 92  1  0  0  0  0
 89 91  1  0  0  0  0
 89 93  1  0  0  0  0
 91 94  1  0  0  0  0
 95 96  2  0  0  0  0
 95 97  1  0  0  0  0
 96 98  1  0  0  0  0
 96 99  1  0  0  0  0
 97100  2  0  0  0  0
 97101  1  0  0  0  0
```

```
 98102  2  0  0  0  0
 98103  1  0  0  0  0
100102  1  0  0  0  0
100104  1  0  0  0  0
102105  1  0  0  0  0
M  END
$$$$
mol011
Optimized Catalyst 011
Optimized Catalyst 011
118128  0  0  0  0  0  0  0  0  0    0
    0.1750    1.7690   -1.0350 P   0  0  0  0  0  0  0  0  0  0  0  0
   -2.8910    0.2120    0.0910 P   0  0  0  0  0  0  0  0  0  0  0  0
    0.2580   -1.4710   -1.6120 P   0  0  0  0  0  0  0  0  0  0  0  0
   -1.1810    1.6750   -2.3290 C   0  0  0  0  0  0  0  0  0  0  0  0
   -1.9650    2.3890   -2.0360 H   0  0  0  0  0  0  0  0  0  0  0  0
   -0.7330    2.0810   -3.2450 H   0  0  0  0  0  0  0  0  0  0  0  0
   -1.8640    0.3310   -2.7130 C   0  0  0  0  0  0  0  0  0  0  0  0
   -2.5330    0.5770   -4.1050 C   0  0  0  0  0  0  0  0  0  0  0  0
   -3.0920   -0.3110   -4.4160 H   0  0  0  0  0  0  0  0  0  0  0  0
   -3.2240    1.4240   -4.0470 H   0  0  0  0  0  0  0  0  0  0  0  0
   -1.7730    0.7950   -4.8640 H   0  0  0  0  0  0  0  0  0  0  0  0
   -0.8220   -0.7920   -2.9630 C   0  0  0  0  0  0  0  0  0  0  0  0
   -1.3240   -1.6390   -3.4460 H   0  0  0  0  0  0  0  0  0  0  0  0
   -0.0990   -0.4100   -3.6980 H   0  0  0  0  0  0  0  0  0  0  0  0
   -3.0340   -0.0910   -1.7640 C   0  0  0  0  0  0  0  0  0  0  0  0
   -3.2010   -1.1750   -1.8770 H   0  0  0  0  0  0  0  0  0  0  0  0
   -3.9560    0.3890   -2.1120 H   0  0  0  0  0  0  0  0  0  0  0  0
    1.5530   -2.1510   -2.7240 C   0  0  0  0  0  0  0  0  0  0  0  0
    2.8130   -1.5430   -2.7100 C   0  0  0  0  0  0  0  0  0  0  0  0
    1.2850   -3.2180   -3.5880 C   0  0  0  0  0  0  0  0  0  0  0  0
    3.8130   -2.0000   -3.5770 C   0  0  0  0  0  0  0  0  0  0  0  0
    3.0330   -0.7090   -2.0170 H   0  0  0  0  0  0  0  0  0  0  0  0
    2.2890   -3.6710   -4.4510 C   0  0  0  0  0  0  0  0  0  0  0  0
    0.3140   -3.7210   -3.6050 H   0  0  0  0  0  0  0  0  0  0  0  0
    3.5490   -3.0630   -4.4470 C   0  0  0  0  0  0  0  0  0  0  0  0
    4.7970   -1.5310   -3.5730 H   0  0  0  0  0  0  0  0  0  0  0  0
    2.0890   -4.5030   -5.1270 H   0  0  0  0  0  0  0  0  0  0  0  0
    4.3290   -3.4200   -5.1230 H   0  0  0  0  0  0  0  0  0  0  0  0
   -0.5370   -3.0510   -1.1180 C   0  0  0  0  0  0  0  0  0  0  0  0
   -1.8940   -3.3100   -1.3210 C   0  0  0  0  0  0  0  0  0  0  0  0
    0.2930   -4.0500   -0.5880 C   0  0  0  0  0  0  0  0  0  0  0  0
   -2.4230   -4.5660   -1.0000 C   0  0  0  0  0  0  0  0  0  0  0  0
   -2.5810   -2.5570   -1.7310 H   0  0  0  0  0  0  0  0  0  0  0  0
   -0.2380   -5.3040   -0.2710 C   0  0  0  0  0  0  0  0  0  0  0  0
    1.3620   -3.8810   -0.4180 H   0  0  0  0  0  0  0  0  0  0  0  0
   -1.5970   -5.5640   -0.4760 C   0  0  0  0  0  0  0  0  0  0  0  0
   -3.4850   -4.7610   -1.1560 H   0  0  0  0  0  0  0  0  0  0  0  0
    0.4090   -6.0810    0.1370 H   0  0  0  0  0  0  0  0  0  0  0  0
   -2.0110   -6.5430   -0.2290 H   0  0  0  0  0  0  0  0  0  0  0  0
    1.2400    3.0190   -1.8780 C   0  0  0  0  0  0  0  0  0  0  0  0
    1.7190    2.7390   -3.1660 C   0  0  0  0  0  0  0  0  0  0  0  0
    1.5640    4.2300   -1.2600 C   0  0  0  0  0  0  0  0  0  0  0  0
    2.4710    3.6940   -3.8540 C   0  0  0  0  0  0  0  0  0  0  0  0
    1.5270    1.7810   -3.6570 H   0  0  0  0  0  0  0  0  0  0  0  0
    2.3180    5.1860   -1.9550 C   0  0  0  0  0  0  0  0  0  0  0  0
    1.2550    4.4640   -0.2360 H   0  0  0  0  0  0  0  0  0  0  0  0
    2.7640    4.9250   -3.2520 C   0  0  0  0  0  0  0  0  0  0  0  0
    2.8330    3.4830   -4.8610 H   0  0  0  0  0  0  0  0  0  0  0  0
    2.5570    6.1370   -1.4770 H   0  0  0  0  0  0  0  0  0  0  0  0
    3.3440    5.6740   -3.7950 H   0  0  0  0  0  0  0  0  0  0  0  0
   -0.6160    2.7630    0.2670 C   0  0  0  0  0  0  0  0  0  0  0  0
   -1.2860    3.9610   -0.0190 C   0  0  0  0  0  0  0  0  0  0  0  0
   -0.5510    2.2800    1.5780 C   0  0  0  0  0  0  0  0  0  0  0  0
   -1.8810    4.6790    1.0220 C   0  0  0  0  0  0  0  0  0  0  0  0
```

```
  -1.3550    4.3590   -1.0330 H   0  0  0  0  0  0  0  0  0  0  0  0
  -1.1530    3.0030    2.6160 C   0  0  0  0  0  0  0  0  0  0  0  0
  -0.0300    1.3300    1.8060 H   0  0  0  0  0  0  0  0  0  0  0  0
  -1.8170    4.2000    2.3370 C   0  0  0  0  0  0  0  0  0  0  0  0
  -2.4060    5.6100    0.8090 H   0  0  0  0  0  0  0  0  0  0  0  0
  -1.1040    2.6300    3.6390 H   0  0  0  0  0  0  0  0  0  0  0  0
  -2.2900    4.7640    3.1430 H   0  0  0  0  0  0  0  0  0  0  0  0
  -3.9360    1.7320    0.3230 C   0  0  0  0  0  0  0  0  0  0  0  0
  -4.1670    2.6550   -0.7000 C   0  0  0  0  0  0  0  0  0  0  0  0
  -4.4810    1.9480    1.5950 C   0  0  0  0  0  0  0  0  0  0  0  0
  -4.9700    3.7770   -0.4630 C   0  0  0  0  0  0  0  0  0  0  0  0
  -3.7310    2.5330   -1.6940 H   0  0  0  0  0  0  0  0  0  0  0  0
  -5.2800    3.0720    1.8290 C   0  0  0  0  0  0  0  0  0  0  0  0
  -4.3100    1.2520    2.4200 H   0  0  0  0  0  0  0  0  0  0  0  0
  -5.5300    3.9850    0.8000 C   0  0  0  0  0  0  0  0  0  0  0  0
  -5.1610    4.4880   -1.2650 H   0  0  0  0  0  0  0  0  0  0  0  0
  -5.7130    3.2340    2.8160 H   0  0  0  0  0  0  0  0  0  0  0  0
  -6.1620    4.8550    0.9820 H   0  0  0  0  0  0  0  0  0  0  0  0
  -4.1760   -1.0420    0.5850 C   0  0  0  0  0  0  0  0  0  0  0  0
  -5.5390   -0.8430    0.3450 C   0  0  0  0  0  0  0  0  0  0  0  0
  -3.7370   -2.2170    1.2010 C   0  0  0  0  0  0  0  0  0  0  0  0
  -6.4630   -1.8220    0.7260 C   0  0  0  0  0  0  0  0  0  0  0  0
  -5.9140    0.0680   -0.1290 H   0  0  0  0  0  0  0  0  0  0  0  0
  -4.6640   -3.1950    1.5810 C   0  0  0  0  0  0  0  0  0  0  0  0
  -2.6780   -2.4050    1.3940 H   0  0  0  0  0  0  0  0  0  0  0  0
  -6.0280   -2.9970    1.3460 C   0  0  0  0  0  0  0  0  0  0  0  0
  -7.5260   -1.6650    0.5440 H   0  0  0  0  0  0  0  0  0  0  0  0
  -4.3240   -4.1090    2.0650 H   0  0  0  0  0  0  0  0  0  0  0  0
  -6.7510   -3.7560    1.6460 H   0  0  0  0  0  0  0  0  0  0  0  0
   1.5570   -2.4280    1.9040 C   0  0  0  0  0  0  0  0  0  0  0  0
   3.5330   -1.9520    0.7360 C   0  0  0  0  0  0  0  0  0  0  0  0
   0.1860   -2.1690    2.1530 C   0  0  0  0  0  0  0  0  0  0  0  0
   2.2280   -3.4300    2.6740 C   0  0  0  0  0  0  0  0  0  0  0  0
   4.2520   -2.9300    1.4530 C   0  0  0  0  0  0  0  0  0  0  0  0
   4.0330   -1.3570   -0.0160 H   0  0  0  0  0  0  0  0  0  0  0  0
  -0.4860   -2.8700    3.1280 C   0  0  0  0  0  0  0  0  0  0  0  0
  -0.3070   -1.4150    1.5480 H   0  0  0  0  0  0  0  0  0  0  0  0
   1.5030   -4.1400    3.6680 C   0  0  0  0  0  0  0  0  0  0  0  0
   3.6030   -3.6660    2.4170 C   0  0  0  0  0  0  0  0  0  0  0  0
   5.3000   -3.0830    1.2360 H   0  0  0  0  0  0  0  0  0  0  0  0
   0.1750   -3.8640    3.8930 C   0  0  0  0  0  0  0  0  0  0  0  0
  -1.5340   -2.6660    3.3150 H   0  0  0  0  0  0  0  0  0  0  0  0
   2.0180   -4.8980    4.2470 H   0  0  0  0  0  0  0  0  0  0  0  0
   4.1270   -4.4210    2.9900 H   0  0  0  0  0  0  0  0  0  0  0  0
  -0.3730   -4.4050    4.6540 H   0  0  0  0  0  0  0  0  0  0  0  0
   2.2350   -1.6990    0.9360 N   0  0  0  0  0  0  0  0  0  0  0  0
   2.9560    1.4270    2.2170 C   0  0  0  0  0  0  0  0  0  0  0  0
   3.5940    1.9160    0.0160 C   0  0  0  0  0  0  0  0  0  0  0  0
   2.0830    0.7990    3.1370 C   0  0  0  0  0  0  0  0  0  0  0  0
   4.0620    2.1840    2.7240 C   0  0  0  0  0  0  0  0  0  0  0  0
   4.7020    2.6730    0.4490 C   0  0  0  0  0  0  0  0  0  0  0  0
   3.3880    1.8130   -1.0420 H   0  0  0  0  0  0  0  0  0  0  0  0
   2.2920    0.9140    4.4920 C   0  0  0  0  0  0  0  0  0  0  0  0
   1.2560    0.2240    2.7470 H   0  0  0  0  0  0  0  0  0  0  0  0
   4.2500    2.2860    4.1290 C   0  0  0  0  0  0  0  0  0  0  0  0
   4.9350    2.8070    1.7970 C   0  0  0  0  0  0  0  0  0  0  0  0
   5.3430    3.1380   -0.2880 H   0  0  0  0  0  0  0  0  0  0  0  0
   3.3840    1.6640    4.9960 C   0  0  0  0  0  0  0  0  0  0  0  0
   1.6170    0.4250    5.1830 H   0  0  0  0  0  0  0  0  0  0  0  0
   5.0890    2.8630    4.5000 H   0  0  0  0  0  0  0  0  0  0  0  0
   5.7750    3.3840    2.1650 H   0  0  0  0  0  0  0  0  0  0  0  0
   3.5300    1.7430    6.0650 H   0  0  0  0  0  0  0  0  0  0  0  0
   2.7400    1.3070    0.8490 N   0  0  0  0  0  0  0  0  0  0  0  0
   1.2740   -0.0390   -0.1480 Rh  0  0  0  0  0  0  0  0  0  0  0  0
 1  4  1  0  0  0  0
```

```
  1 40  1  0  0  0  0
  1 51  1  0  0  0  0
  1118  1  0  0  0  0
  2 15  1  0  0  0  0
  2 62  1  0  0  0  0
  2 73  1  0  0  0  0
  3 12  1  0  0  0  0
  3 18  1  0  0  0  0
  3 29  1  0  0  0  0
  3118  1  0  0  0  0
  4  5  1  0  0  0  0
  4  6  1  0  0  0  0
  4  7  1  0  0  0  0
  7  8  1  0  0  0  0
  7 12  1  0  0  0  0
  7 15  1  0  0  0  0
  8  9  1  0  0  0  0
  8 10  1  0  0  0  0
  8 11  1  0  0  0  0
 12 13  1  0  0  0  0
 12 14  1  0  0  0  0
 15 16  1  0  0  0  0
 15 17  1  0  0  0  0
 18 19  4  0  0  0  0
 18 20  4  0  0  0  0
 19 21  4  0  0  0  0
 19 22  1  0  0  0  0
 20 23  4  0  0  0  0
 20 24  1  0  0  0  0
 21 25  4  0  0  0  0
 21 26  1  0  0  0  0
 23 25  4  0  0  0  0
 23 27  1  0  0  0  0
 25 28  1  0  0  0  0
 29 30  4  0  0  0  0
 29 31  4  0  0  0  0
 30 32  4  0  0  0  0
 30 33  1  0  0  0  0
 31 34  4  0  0  0  0
 31 35  1  0  0  0  0
 32 36  4  0  0  0  0
 32 37  1  0  0  0  0
 34 36  4  0  0  0  0
 34 38  1  0  0  0  0
 36 39  1  0  0  0  0
 40 41  4  0  0  0  0
 40 42  4  0  0  0  0
 41 43  4  0  0  0  0
 41 44  1  0  0  0  0
 42 45  4  0  0  0  0
 42 46  1  0  0  0  0
 43 47  4  0  0  0  0
 43 48  1  0  0  0  0
 45 47  4  0  0  0  0
 45 49  1  0  0  0  0
 47 50  1  0  0  0  0
 51 52  4  0  0  0  0
 51 53  4  0  0  0  0
 52 54  4  0  0  0  0
 52 55  1  0  0  0  0
 53 56  4  0  0  0  0
 53 57  1  0  0  0  0
 54 58  4  0  0  0  0
 54 59  1  0  0  0  0
 56 58  4  0  0  0  0
```

```
 56 60  1  0  0  0  0
 58 61  1  0  0  0  0
 62 63  4  0  0  0  0
 62 64  4  0  0  0  0
 63 65  4  0  0  0  0
 63 66  1  0  0  0  0
 64 67  4  0  0  0  0
 64 68  1  0  0  0  0
 65 69  4  0  0  0  0
 65 70  1  0  0  0  0
 67 69  4  0  0  0  0
 67 71  1  0  0  0  0
 69 72  1  0  0  0  0
 73 74  4  0  0  0  0
 73 75  4  0  0  0  0
 74 76  4  0  0  0  0
 74 77  1  0  0  0  0
 75 78  4  0  0  0  0
 75 79  1  0  0  0  0
 76 80  4  0  0  0  0
 76 81  1  0  0  0  0
 78 80  4  0  0  0  0
 78 82  1  0  0  0  0
 80 83  1  0  0  0  0
 84 86  4  0  0  0  0
 84 87  4  0  0  0  0
 84100  1  0  0  0  0
 85 88  4  0  0  0  0
 85 89  1  0  0  0  0
 85100  4  0  0  0  0
 86 90  2  0  0  0  0
 86 91  1  0  0  0  0
 87 92  4  0  0  0  0
 87 93  4  0  0  0  0
 88 93  2  0  0  0  0
 88 94  1  0  0  0  0
 90 95  4  0  0  0  0
 90 96  1  0  0  0  0
 92 95  2  0  0  0  0
 92 97  1  0  0  0  0
 93 98  1  0  0  0  0
 95 99  1  0  0  0  0
100118  1  0  0  0  0
101103  4  0  0  0  0
101104  4  0  0  0  0
101117  1  0  0  0  0
102105  4  0  0  0  0
102106  1  0  0  0  0
102117  4  0  0  0  0
103107  2  0  0  0  0
103108  1  0  0  0  0
104109  4  0  0  0  0
104110  4  0  0  0  0
105110  2  0  0  0  0
105111  1  0  0  0  0
107112  4  0  0  0  0
107113  1  0  0  0  0
109112  2  0  0  0  0
109114  1  0  0  0  0
110115  1  0  0  0  0
112116  1  0  0  0  0
117118  1  0  0  0  0
M  END
$$$$
mol012
```

```
Optimized Catalyst 012
Optimized Catalyst 012
118129  0  0  0  0  0  0  0  0  0    0
   -0.2240   -0.1990    0.4360 Ir  0  0  0  0  0  0  0  0  0  0  0  0
   -0.4270    0.0870   -2.1830 P   0  0  0  0  0  0  0  0  0  0  0  0
   -2.4500    2.1280   -0.0300 P   0  0  0  0  0  0  0  0  0  0  0  0
   -2.3670   -1.6750    0.3410 P   0  0  0  0  0  0  0  0  0  0  0  0
   -2.2190    0.1550   -2.8420 C   0  0  0  0  0  0  0  0  0  0  0  0
   -2.3030    1.1480   -3.3630 H   0  0  0  0  0  0  0  0  0  0  0  0
   -2.2810   -0.6360   -3.6360 H   0  0  0  0  0  0  0  0  0  0  0  0
   -3.4640    0.0150   -1.9330 C   0  0  0  0  0  0  0  0  0  0  0  0
   -4.6570   -0.1080   -2.9470 C   0  0  0  0  0  0  0  0  0  0  0  0
   -5.6300   -0.1250   -2.4080 H   0  0  0  0  0  0  0  0  0  0  0  0
   -4.6660    0.7560   -3.6480 H   0  0  0  0  0  0  0  0  0  0  0  0
   -4.5780   -1.0390   -3.5490 H   0  0  0  0  0  0  0  0  0  0  0  0
   -3.5330   -1.3060   -1.1210 C   0  0  0  0  0  0  0  0  0  0  0  0
   -4.5770   -1.4030   -0.7200 H   0  0  0  0  0  0  0  0  0  0  0  0
   -3.3850   -2.1510   -1.8470 H   0  0  0  0  0  0  0  0  0  0  0  0
   -3.8010    1.2630   -1.0870 C   0  0  0  0  0  0  0  0  0  0  0  0
   -4.6150    0.9740   -0.3780 H   0  0  0  0  0  0  0  0  0  0  0  0
   -4.2250    2.0440   -1.7680 H   0  0  0  0  0  0  0  0  0  0  0  0
   -1.9170   -3.4540    0.0020 C   0  0  0  0  0  0  0  0  0  0  0  0
   -0.5990   -3.7870   -0.3110 C   0  0  0  0  0  0  0  0  0  0  0  0
   -2.9170   -4.4310    0.0350 C   0  0  0  0  0  0  0  0  0  0  0  0
   -0.2750   -5.1170   -0.6050 C   0  0  0  0  0  0  0  0  0  0  0  0
    0.2000   -3.0330   -0.3440 H   0  0  0  0  0  0  0  0  0  0  0  0
   -2.5860   -5.7590   -0.2560 C   0  0  0  0  0  0  0  0  0  0  0  0
   -3.9530   -4.1910    0.2900 H   0  0  0  0  0  0  0  0  0  0  0  0
   -1.2680   -6.1010   -0.5770 C   0  0  0  0  0  0  0  0  0  0  0  0
    0.7520   -5.3840   -0.8590 H   0  0  0  0  0  0  0  0  0  0  0  0
   -3.3580   -6.5310   -0.2300 H   0  0  0  0  0  0  0  0  0  0  0  0
   -1.0140   -7.1390   -0.8050 H   0  0  0  0  0  0  0  0  0  0  0  0
   -3.3910   -1.7430    1.8920 C   0  0  0  0  0  0  0  0  0  0  0  0
   -4.4410   -0.8540    2.1240 C   0  0  0  0  0  0  0  0  0  0  0  0
   -3.0510   -2.7130    2.8440 C   0  0  0  0  0  0  0  0  0  0  0  0
   -5.1640   -0.9410    3.3210 C   0  0  0  0  0  0  0  0  0  0  0  0
   -4.7240   -0.0790    1.4130 H   0  0  0  0  0  0  0  0  0  0  0  0
   -3.7800   -2.7950    4.0350 C   0  0  0  0  0  0  0  0  0  0  0  0
   -2.2270   -3.4160    2.6880 H   0  0  0  0  0  0  0  0  0  0  0  0
   -4.8360   -1.9090    4.2740 C   0  0  0  0  0  0  0  0  0  0  0  0
   -5.9870   -0.2480    3.5090 H   0  0  0  0  0  0  0  0  0  0  0  0
   -3.5230   -3.5510    4.7780 H   0  0  0  0  0  0  0  0  0  0  0  0
   -5.4030   -1.9750    5.2050 H   0  0  0  0  0  0  0  0  0  0  0  0
    0.3460   -1.3890   -3.0320 C   0  0  0  0  0  0  0  0  0  0  0  0
   -0.3680   -2.5760   -3.2100 C   0  0  0  0  0  0  0  0  0  0  0  0
    1.6590   -1.2730   -3.5020 C   0  0  0  0  0  0  0  0  0  0  0  0
    0.2420   -3.6590   -3.8560 C   0  0  0  0  0  0  0  0  0  0  0  0
   -1.3940   -2.7040   -2.8630 H   0  0  0  0  0  0  0  0  0  0  0  0
    2.2610   -2.3570   -4.1490 C   0  0  0  0  0  0  0  0  0  0  0  0
    2.2320   -0.3460   -3.4000 H   0  0  0  0  0  0  0  0  0  0  0  0
    1.5530   -3.5510   -4.3260 C   0  0  0  0  0  0  0  0  0  0  0  0
   -0.3130   -4.5900   -3.9930 H   0  0  0  0  0  0  0  0  0  0  0  0
    3.2810   -2.2680   -4.5270 H   0  0  0  0  0  0  0  0  0  0  0  0
    2.0230   -4.3950   -4.8370 H   0  0  0  0  0  0  0  0  0  0  0  0
    0.3570    1.5350   -3.0590 C   0  0  0  0  0  0  0  0  0  0  0  0
    0.3860    1.5060   -4.4600 C   0  0  0  0  0  0  0  0  0  0  0  0
    0.8810    2.6180   -2.3570 C   0  0  0  0  0  0  0  0  0  0  0  0
    0.9600    2.5720   -5.1570 C   0  0  0  0  0  0  0  0  0  0  0  0
   -0.0210    0.6680   -5.0320 H   0  0  0  0  0  0  0  0  0  0  0  0
    1.4650    3.6800   -3.0640 C   0  0  0  0  0  0  0  0  0  0  0  0
    0.8380    2.6910   -1.2680 H   0  0  0  0  0  0  0  0  0  0  0  0
    1.5040    3.6580   -4.4590 C   0  0  0  0  0  0  0  0  0  0  0  0
    0.9860    2.5580   -6.2490 H   0  0  0  0  0  0  0  0  0  0  0  0
    1.8740    4.5310   -2.5170 H   0  0  0  0  0  0  0  0  0  0  0  0
    1.9540    4.4870   -5.0090 H   0  0  0  0  0  0  0  0  0  0  0  0
```

```
   -1.8990    3.6360   -1.0060 C   0  0  0  0  0  0  0  0  0  0  0  0
   -2.2690    3.8800   -2.3280 C   0  0  0  0  0  0  0  0  0  0  0  0
   -1.0670    4.5360   -0.3300 C   0  0  0  0  0  0  0  0  0  0  0  0
   -1.8010    5.0290   -2.9810 C   0  0  0  0  0  0  0  0  0  0  0  0
   -2.9200    3.2110   -2.8890 H   0  0  0  0  0  0  0  0  0  0  0  0
   -0.6090    5.6860   -0.9820 C   0  0  0  0  0  0  0  0  0  0  0  0
   -0.7620    4.3760    0.7050 H   0  0  0  0  0  0  0  0  0  0  0  0
   -0.9740    5.9330   -2.3100 C   0  0  0  0  0  0  0  0  0  0  0  0
   -2.0870    5.2180   -4.0160 H   0  0  0  0  0  0  0  0  0  0  0  0
    0.0290    6.3940   -0.4540 H   0  0  0  0  0  0  0  0  0  0  0  0
   -0.6180    6.8300   -2.8180 H   0  0  0  0  0  0  0  0  0  0  0  0
   -3.4740    2.9530    1.3210 C   0  0  0  0  0  0  0  0  0  0  0  0
   -4.8370    3.2190    1.1650 C   0  0  0  0  0  0  0  0  0  0  0  0
   -2.8160    3.3330    2.4940 C   0  0  0  0  0  0  0  0  0  0  0  0
   -5.5500    3.8300    2.2040 C   0  0  0  0  0  0  0  0  0  0  0  0
   -5.3770    2.9900    0.2460 H   0  0  0  0  0  0  0  0  0  0  0  0
   -3.5310    3.9520    3.5270 C   0  0  0  0  0  0  0  0  0  0  0  0
   -1.7480    3.1770    2.6360 H   0  0  0  0  0  0  0  0  0  0  0  0
   -4.9000    4.1930    3.3870 C   0  0  0  0  0  0  0  0  0  0  0  0
   -6.6150    4.0350    2.0840 H   0  0  0  0  0  0  0  0  0  0  0  0
   -3.0160    4.2540    4.4390 H   0  0  0  0  0  0  0  0  0  0  0  0
   -5.4570    4.6760    4.1910 H   0  0  0  0  0  0  0  0  0  0  0  0
   -0.5720   -0.6330    3.5670 C   0  0  0  0  0  0  0  0  0  0  0  0
    1.1000   -1.8590    2.4360 C   0  0  0  0  0  0  0  0  0  0  0  0
   -1.6370    0.3240    3.5560 C   0  0  0  0  0  0  0  0  0  0  0  0
   -0.2230   -1.2690    4.7960 C   0  0  0  0  0  0  0  0  0  0  0  0
    1.4870   -2.5190    3.6320 C   0  0  0  0  0  0  0  0  0  0  0  0
    1.6270   -2.0940    1.4900 H   0  0  0  0  0  0  0  0  0  0  0  0
   -2.3080    0.6270    4.7140 C   0  0  0  0  0  0  0  0  0  0  0  0
   -1.9240    0.7900    2.6100 H   0  0  0  0  0  0  0  0  0  0  0  0
   -0.9460   -0.9270    5.9850 C   0  0  0  0  0  0  0  0  0  0  0  0
    0.8320   -2.2280    4.8060 C   0  0  0  0  0  0  0  0  0  0  0  0
    2.3030   -3.2460    3.5970 H   0  0  0  0  0  0  0  0  0  0  0  0
   -1.9580   -0.0030    5.9450 C   0  0  0  0  0  0  0  0  0  0  0  0
   -3.1290    1.3510    4.7040 H   0  0  0  0  0  0  0  0  0  0  0  0
   -0.6700   -1.4190    6.9200 H   0  0  0  0  0  0  0  0  0  0  0  0
    1.1070   -2.7170    5.7460 H   0  0  0  0  0  0  0  0  0  0  0  0
   -2.5120    0.2610    6.8490 H   0  0  0  0  0  0  0  0  0  0  0  0
    0.1040   -0.9450    2.3690 N   0  0  0  0  0  0  0  0  0  0  0  0
    1.9520    1.9540    0.9500 C   0  0  0  0  0  0  0  0  0  0  0  0
    2.6130    0.1230   -0.3870 C   0  0  0  0  0  0  0  0  0  0  0  0
    1.0250    2.5330    1.8770 C   0  0  0  0  0  0  0  0  0  0  0  0
    3.1650    2.6390    0.6480 C   0  0  0  0  0  0  0  0  0  0  0  0
    3.8510    0.7490   -0.6960 C   0  0  0  0  0  0  0  0  0  0  0  0
    2.4050   -0.8900   -0.7840 H   0  0  0  0  0  0  0  0  0  0  0  0
    1.2860    3.7620    2.4290 C   0  0  0  0  0  0  0  0  0  0  0  0
    0.1310    1.9560    2.1520 H   0  0  0  0  0  0  0  0  0  0  0  0
    3.3920    3.9290    1.2280 C   0  0  0  0  0  0  0  0  0  0  0  0
    4.1180    2.0060   -0.2050 C   0  0  0  0  0  0  0  0  0  0  0  0
    4.5700    0.2190   -1.3270 H   0  0  0  0  0  0  0  0  0  0  0  0
    2.4740    4.4760    2.0870 C   0  0  0  0  0  0  0  0  0  0  0  0
    0.5960    4.2110    3.1490 H   0  0  0  0  0  0  0  0  0  0  0  0
    4.3140    4.4600    0.9780 H   0  0  0  0  0  0  0  0  0  0  0  0
    5.0510    2.5230   -0.4490 H   0  0  0  0  0  0  0  0  0  0  0  0
    2.6420    5.4590    2.5350 H   0  0  0  0  0  0  0  0  0  0  0  0
    1.6550    0.7030    0.3700 N   0  0  0  0  0  0  0  0  0  0  0  0
  1  2  1  0  0  0  0
  1  4  1  0  0  0  0
  1118  1  0  0  0  0
  1101  1  0  0  0  0
  1  3  1  0  0  0  0
  2 52  1  0  0  0  0
  2 41  1  0  0  0  0
  2  5  1  0  0  0  0
  3 16  1  0  0  0  0
```

```
  3 63  1  0  0  0  0
  3 74  1  0  0  0  0
  4 13  1  0  0  0  0
  4 19  1  0  0  0  0
  4 30  1  0  0  0  0
  5  7  1  0  0  0  0
  5  6  1  0  0  0  0
  5  8  1  0  0  0  0
  8  9  1  0  0  0  0
  8 13  1  0  0  0  0
  8 16  1  0  0  0  0
  9 11  1  0  0  0  0
  9 12  1  0  0  0  0
  9 10  1  0  0  0  0
 13 15  1  0  0  0  0
 13 14  1  0  0  0  0
 16 18  1  0  0  0  0
 16 17  1  0  0  0  0
 19 20  4  0  0  0  0
 19 21  4  0  0  0  0
 20 22  4  0  0  0  0
 20 23  1  0  0  0  0
 21 24  4  0  0  0  0
 21 25  1  0  0  0  0
 22 27  1  0  0  0  0
 22 26  4  0  0  0  0
 24 26  4  0  0  0  0
 24 28  1  0  0  0  0
 26 29  1  0  0  0  0
 30 31  4  0  0  0  0
 30 32  4  0  0  0  0
 31 34  1  0  0  0  0
 31 33  4  0  0  0  0
 32 36  1  0  0  0  0
 32 35  4  0  0  0  0
 33 38  1  0  0  0  0
 33 37  4  0  0  0  0
 35 37  4  0  0  0  0
 35 39  1  0  0  0  0
 37 40  1  0  0  0  0
 41 43  4  0  0  0  0
 41 42  4  0  0  0  0
 42 44  4  0  0  0  0
 42 45  1  0  0  0  0
 43 46  4  0  0  0  0
 43 47  1  0  0  0  0
 44 48  4  0  0  0  0
 44 49  1  0  0  0  0
 46 50  1  0  0  0  0
 46 48  4  0  0  0  0
 48 51  1  0  0  0  0
 52 53  4  0  0  0  0
 52 54  4  0  0  0  0
 53 55  4  0  0  0  0
 53 56  1  0  0  0  0
 54 57  4  0  0  0  0
 54 58  1  0  0  0  0
 55 60  1  0  0  0  0
 55 59  4  0  0  0  0
 57 59  4  0  0  0  0
 57 61  1  0  0  0  0
 59 62  1  0  0  0  0
 63 64  4  0  0  0  0
 63 65  4  0  0  0  0
 64 66  4  0  0  0  0
```

```
 64 67  1  0  0  0  0
 65 68  4  0  0  0  0
 65 69  1  0  0  0  0
 66 71  1  0  0  0  0
 66 70  4  0  0  0  0
 68 70  4  0  0  0  0
 68 72  1  0  0  0  0
 70 73  1  0  0  0  0
 74 75  4  0  0  0  0
 74 76  4  0  0  0  0
 75 78  1  0  0  0  0
 75 77  4  0  0  0  0
 76 80  1  0  0  0  0
 76 79  4  0  0  0  0
 77 82  1  0  0  0  0
 77 81  4  0  0  0  0
 79 81  4  0  0  0  0
 79 83  1  0  0  0  0
 81 84  1  0  0  0  0
 85101  1  0  0  0  0
 85 87  4  0  0  0  0
 85 88  4  0  0  0  0
 86 90  1  0  0  0  0
 86101  4  0  0  0  0
 86 89  4  0  0  0  0
 87 92  1  0  0  0  0
 87 91  2  0  0  0  0
 88 94  4  0  0  0  0
 88 93  4  0  0  0  0
 89 95  1  0  0  0  0
 89 94  2  0  0  0  0
 91 97  1  0  0  0  0
 91 96  4  0  0  0  0
 93 96  2  0  0  0  0
 93 98  1  0  0  0  0
 94 99  1  0  0  0  0
 96100  1  0  0  0  0
102118  1  0  0  0  0
102105  4  0  0  0  0
102104  4  0  0  0  0
103107  1  0  0  0  0
103106  4  0  0  0  0
103118  4  0  0  0  0
104109  1  0  0  0  0
104108  2  0  0  0  0
105111  4  0  0  0  0
105110  4  0  0  0  0
106112  1  0  0  0  0
106111  2  0  0  0  0
108113  4  0  0  0  0
108114  1  0  0  0  0
110115  1  0  0  0  0
110113  2  0  0  0  0
111116  1  0  0  0  0
113117  1  0  0  0  0
M  END
$$$$
mol013
Optimized Catalyst 013
Optimized Catalyst 013
 84 89  0  0  0  0  0  0  0  0999 V2000
    0.0415    1.6415    0.0249 Ru  0  0  0  0  0  0  0  0  0  0  0  0
    0.4108    1.5999    1.9927 H   0  0  0  0  0  0  0  0  0  0  0  0
   -1.7874    1.6170    0.3800 C   0  0  0  0  0  0  0  0  0  0  0  0
   -2.9271    1.5975    0.5683 O   0  0  0  0  0  0  0  0  0  0  0  0
```

```
   -0.2308    1.7748   -2.3733 N   0  0  0  0  0  0  0  0  0  0  0  0
    2.0570    1.5932   -0.4032 N   0  0  0  0  0  0  0  0  0  0  0  0
    3.1679    1.5747   -0.7339 C   0  0  0  0  0  0  0  0  0  0  0  0
   -0.2053    1.6810   -3.5284 C   0  0  0  0  0  0  0  0  0  0  0  0
    4.5528    1.5621   -1.1187 C   0  0  0  0  0  0  0  0  0  0  0  0
    4.9559    2.6144   -1.1233 H   0  0  0  0  0  0  0  0  0  0  0  0
    5.1530    0.9471   -0.3930 H   0  0  0  0  0  0  0  0  0  0  0  0
    4.6734    1.1262   -2.1465 H   0  0  0  0  0  0  0  0  0  0  0  0
   -0.1819    1.5471   -4.9623 C   0  0  0  0  0  0  0  0  0  0  0  0
    0.7389    2.0250   -5.3891 H   0  0  0  0  0  0  0  0  0  0  0  0
   -0.1877    0.4572   -5.2430 H   0  0  0  0  0  0  0  0  0  0  0  0
   -1.0846    2.0390   -5.4154 H   0  0  0  0  0  0  0  0  0  0  0  0
    0.1098    3.9325    0.0732 P   0  0  0  0  0  0  0  0  0  0  0  0
    0.1756   -0.6307   -0.0283 P   0  0  0  0  0  0  0  0  0  0  0  0
    0.0895   -1.2740   -1.7917 C   0  0  0  0  0  0  0  0  0  0  0  0
   -1.1551   -1.5308   -2.3720 C   0  0  0  0  0  0  0  0  0  0  0  0
    1.2660   -1.4430   -2.5256 C   0  0  0  0  0  0  0  0  0  0  0  0
   -1.2186   -1.9971   -3.6907 C   0  0  0  0  0  0  0  0  0  0  0  0
   -2.0892   -1.3774   -1.8246 H   0  0  0  0  0  0  0  0  0  0  0  0
    1.1966   -1.9085   -3.8441 C   0  0  0  0  0  0  0  0  0  0  0  0
    2.2467   -1.2249   -2.0973 H   0  0  0  0  0  0  0  0  0  0  0  0
   -0.0440   -2.1928   -4.4238 C   0  0  0  0  0  0  0  0  0  0  0  0
   -2.1869   -2.2161   -4.1434 H   0  0  0  0  0  0  0  0  0  0  0  0
    2.1119   -2.0603   -4.4167 H   0  0  0  0  0  0  0  0  0  0  0  0
   -0.0951   -2.5745   -5.4451 H   0  0  0  0  0  0  0  0  0  0  0  0
    1.7333   -1.4232    0.6658 C   0  0  0  0  0  0  0  0  0  0  0  0
    2.4525   -0.7440    1.6506 C   0  0  0  0  0  0  0  0  0  0  0  0
    2.1190   -2.7025    0.2592 C   0  0  0  0  0  0  0  0  0  0  0  0
    3.5871   -1.3382    2.2150 C   0  0  0  0  0  0  0  0  0  0  0  0
    2.1455    0.2480    2.0011 H   0  0  0  0  0  0  0  0  0  0  0  0
    3.2558   -3.2928    0.8237 C   0  0  0  0  0  0  0  0  0  0  0  0
    1.5549   -3.2671   -0.4874 H   0  0  0  0  0  0  0  0  0  0  0  0
    3.9902   -2.6114    1.7998 C   0  0  0  0  0  0  0  0  0  0  0  0
    4.1491   -0.8148    2.9888 H   0  0  0  0  0  0  0  0  0  0  0  0
    3.5630   -4.2915    0.5097 H   0  0  0  0  0  0  0  0  0  0  0  0
    4.8718   -3.0776    2.2440 H   0  0  0  0  0  0  0  0  0  0  0  0
   -1.1130   -1.6288    0.9019 C   0  0  0  0  0  0  0  0  0  0  0  0
   -1.4755   -2.9101    0.4830 C   0  0  0  0  0  0  0  0  0  0  0  0
   -1.6435   -1.0871    2.0751 C   0  0  0  0  0  0  0  0  0  0  0  0
   -2.4045   -3.6435    1.2311 C   0  0  0  0  0  0  0  0  0  0  0  0
   -1.0532   -3.3675   -0.4148 H   0  0  0  0  0  0  0  0  0  0  0  0
   -2.5683   -1.8248    2.8223 C   0  0  0  0  0  0  0  0  0  0  0  0
   -1.3502   -0.0966    2.4341 H   0  0  0  0  0  0  0  0  0  0  0  0
   -2.9520   -3.1016    2.3979 C   0  0  0  0  0  0  0  0  0  0  0  0
   -2.6974   -4.6428    0.9056 H   0  0  0  0  0  0  0  0  0  0  0  0
   -2.9890   -1.4062    3.7375 H   0  0  0  0  0  0  0  0  0  0  0  0
   -3.6753   -3.6759    2.9803 H   0  0  0  0  0  0  0  0  0  0  0  0
    1.7786    4.5835   -0.4929 C   0  0  0  0  0  0  0  0  0  0  0  0
    2.8423    4.6496    0.4118 C   0  0  0  0  0  0  0  0  0  0  0  0
    1.9506    4.9636   -1.8249 C   0  0  0  0  0  0  0  0  0  0  0  0
    4.0864    5.1221   -0.0200 C   0  0  0  0  0  0  0  0  0  0  0  0
    2.7288    4.3415    1.4546 H   0  0  0  0  0  0  0  0  0  0  0  0
    3.1998    5.4313   -2.2527 C   0  0  0  0  0  0  0  0  0  0  0  0
    1.1306    4.9132   -2.5468 H   0  0  0  0  0  0  0  0  0  0  0  0
    4.2643    5.5161   -1.3509 C   0  0  0  0  0  0  0  0  0  0  0  0
    4.9152    5.1959    0.6851 H   0  0  0  0  0  0  0  0  0  0  0  0
    3.3378    5.7420   -3.2891 H   0  0  0  0  0  0  0  0  0  0  0  0
    5.2317    5.8978   -1.6822 H   0  0  0  0  0  0  0  0  0  0  0  0
   -1.1009    4.8923   -0.9984 C   0  0  0  0  0  0  0  0  0  0  0  0
   -0.9706    6.2793   -1.1154 C   0  0  0  0  0  0  0  0  0  0  0  0
   -2.1400    4.2229   -1.6443 C   0  0  0  0  0  0  0  0  0  0  0  0
   -1.8883    6.9980   -1.8892 C   0  0  0  0  0  0  0  0  0  0  0  0
   -0.1684    6.8287   -0.6182 H   0  0  0  0  0  0  0  0  0  0  0  0
   -3.0584    4.9452   -2.4167 C   0  0  0  0  0  0  0  0  0  0  0  0
   -2.2603    3.1388   -1.5709 H   0  0  0  0  0  0  0  0  0  0  0  0
```

```
   -2.9336    6.3320   -2.5381 C   0  0  0  0  0  0  0  0  0  0  0  0
   -1.7927    8.0808   -1.9811 H   0  0  0  0  0  0  0  0  0  0  0  0
   -3.8764    4.4261   -2.9165 H   0  0  0  0  0  0  0  0  0  0  0  0
   -3.6527    6.8960   -3.1351 H   0  0  0  0  0  0  0  0  0  0  0  0
   -0.1708    4.7752    1.7301 C   0  0  0  0  0  0  0  0  0  0  0  0
    0.4510    5.9867    2.0393 C   0  0  0  0  0  0  0  0  0  0  0  0
   -1.0687    4.1910    2.6261 C   0  0  0  0  0  0  0  0  0  0  0  0
    0.1793    6.6117    3.2624 C   0  0  0  0  0  0  0  0  0  0  0  0
    1.1515    6.4714    1.3550 H   0  0  0  0  0  0  0  0  0  0  0  0
   -1.3426    4.8215    3.8455 C   0  0  0  0  0  0  0  0  0  0  0  0
   -1.5713    3.2470    2.4046 H   0  0  0  0  0  0  0  0  0  0  0  0
   -0.7177    6.0314    4.1643 C   0  0  0  0  0  0  0  0  0  0  0  0
    0.6649    7.5568    3.5097 H   0  0  0  0  0  0  0  0  0  0  0  0
   -2.0467    4.3712    4.5462 H   0  0  0  0  0  0  0  0  0  0  0  0
   -0.9324    6.5233    5.1150 H   0  0  0  0  0  0  0  0  0  0  0  0
  1  3  1  0  0  0  0
  1 17  1  0  0  0  0
  1 18  1  0  0  0  0
  1  5  1  0  0  0  0
  1  6  1  0  0  0  0
  1  2  1  0  0  0  0
  3  4  3  0  0  0  0
  5  8  3  0  0  0  0
  6  7  3  0  0  0  0
  7  9  1  0  0  0  0
  8 13  1  0  0  0  0
  9 10  1  0  0  0  0
  9 11  1  0  0  0  0
  9 12  1  0  0  0  0
 13 14  1  0  0  0  0
 13 15  1  0  0  0  0
 13 16  1  0  0  0  0
 17 52  1  0  0  0  0
 17 63  1  0  0  0  0
 17 74  1  0  0  0  0
 18 19  1  0  0  0  0
 18 30  1  0  0  0  0
 18 41  1  0  0  0  0
 19 20  2  0  0  0  0
 19 21  1  0  0  0  0
 20 22  1  0  0  0  0
 20 23  1  0  0  0  0
 21 24  2  0  0  0  0
 21 25  1  0  0  0  0
 22 26  2  0  0  0  0
 22 27  1  0  0  0  0
 24 26  1  0  0  0  0
 24 28  1  0  0  0  0
 26 29  1  0  0  0  0
 30 31  2  0  0  0  0
 30 32  1  0  0  0  0
 31 33  1  0  0  0  0
 31 34  1  0  0  0  0
 32 35  2  0  0  0  0
 32 36  1  0  0  0  0
 33 37  2  0  0  0  0
 33 38  1  0  0  0  0
 35 37  1  0  0  0  0
 35 39  1  0  0  0  0
 37 40  1  0  0  0  0
 41 42  2  0  0  0  0
 41 43  1  0  0  0  0
 42 44  1  0  0  0  0
 42 45  1  0  0  0  0
 43 46  2  0  0  0  0
```

```
 43 47  1  0  0  0  0
 44 48  2  0  0  0  0
 44 49  1  0  0  0  0
 46 48  1  0  0  0  0
 46 50  1  0  0  0  0
 48 51  1  0  0  0  0
 52 53  2  0  0  0  0
 52 54  1  0  0  0  0
 53 55  1  0  0  0  0
 53 56  1  0  0  0  0
 54 57  2  0  0  0  0
 54 58  1  0  0  0  0
 55 59  2  0  0  0  0
 55 60  1  0  0  0  0
 57 59  1  0  0  0  0
 57 61  1  0  0  0  0
 59 62  1  0  0  0  0
 63 64  2  0  0  0  0
 63 65  1  0  0  0  0
 64 66  1  0  0  0  0
 64 67  1  0  0  0  0
 65 68  2  0  0  0  0
 65 69  1  0  0  0  0
 66 70  2  0  0  0  0
 66 71  1  0  0  0  0
 68 70  1  0  0  0  0
 68 72  1  0  0  0  0
 70 73  1  0  0  0  0
 74 75  2  0  0  0  0
 74 76  1  0  0  0  0
 75 77  1  0  0  0  0
 75 78  1  0  0  0  0
 76 79  2  0  0  0  0
 76 80  1  0  0  0  0
 77 81  2  0  0  0  0
 77 82  1  0  0  0  0
 79 81  1  0  0  0  0
 79 83  1  0  0  0  0
 81 84  1  0  0  0  0
M  END
$$$$
mol014
Optimized Catalyst 014
Optimized Catalyst 014
 84 89  0  0  0  0  0  0  0  0999 V2000
   -2.5766   -1.1824    0.1386 H   0  0  0  0  0  0  0  0  0  0  0  0
   -2.2348   -2.3206    2.8571 P   0  0  0  0  0  0  0  0  0  0  0  0
   -3.4728   -3.3915   -1.5861 P   0  0  0  0  0  0  0  0  0  0  0  0
   -0.8125   -2.7100    0.0193 C   0  0  0  0  0  0  0  0  0  0  0  0
    0.2457   -2.4562   -0.3872 O   0  0  0  0  0  0  0  0  0  0  0  0
   -2.8058   -4.6784    1.0876 N   0  0  0  0  0  0  0  0  0  0  0  0
   -2.8102   -5.7816    1.4276 C   0  0  0  0  0  0  0  0  0  0  0  0
   -2.8105   -7.1505    1.8745 C   0  0  0  0  0  0  0  0  0  0  0  0
   -3.6716   -7.7057    1.4190 H   0  0  0  0  0  0  0  0  0  0  0  0
   -1.8548   -7.6543    1.5734 H   0  0  0  0  0  0  0  0  0  0  0  0
   -2.9042   -7.1793    2.9938 H   0  0  0  0  0  0  0  0  0  0  0  0
   -0.4230   -2.0924    3.1447 C   0  0  0  0  0  0  0  0  0  0  0  0
    0.0862   -0.8915    3.6314 C   0  0  0  0  0  0  0  0  0  0  0  0
    0.4169   -3.1648    2.8273 C   0  0  0  0  0  0  0  0  0  0  0  0
    1.4711   -0.7614    3.8087 C   0  0  0  0  0  0  0  0  0  0  0  0
   -0.5606   -0.0453    3.8801 H   0  0  0  0  0  0  0  0  0  0  0  0
    1.7954   -3.0285    3.0096 C   0  0  0  0  0  0  0  0  0  0  0  0
    0.0201   -4.1077    2.4418 H   0  0  0  0  0  0  0  0  0  0  0  0
    2.3214   -1.8258    3.5002 C   0  0  0  0  0  0  0  0  0  0  0  0
    1.8840    0.1746    4.1884 H   0  0  0  0  0  0  0  0  0  0  0  0
```

```
    2.4650   -3.8546    2.7689 H   0  0  0  0  0  0  0  0  0  0  0  0
    3.3994   -1.7214    3.6398 H   0  0  0  0  0  0  0  0  0  0  0  0
   -2.7543   -3.6318    4.0689 C   0  0  0  0  0  0  0  0  0  0  0  0
   -4.0570   -4.1270    3.9750 C   0  0  0  0  0  0  0  0  0  0  0  0
   -1.8709   -4.1158    5.0323 C   0  0  0  0  0  0  0  0  0  0  0  0
   -4.4769   -5.1308    4.8540 C   0  0  0  0  0  0  0  0  0  0  0  0
   -4.7608   -3.7529    3.2285 H   0  0  0  0  0  0  0  0  0  0  0  0
   -2.2953   -5.1233    5.9084 C   0  0  0  0  0  0  0  0  0  0  0  0
   -0.8523   -3.7292    5.1325 H   0  0  0  0  0  0  0  0  0  0  0  0
   -3.5947   -5.6302    5.8189 C   0  0  0  0  0  0  0  0  0  0  0  0
   -5.4924   -5.5215    4.7924 H   0  0  0  0  0  0  0  0  0  0  0  0
   -1.6109   -5.5081    6.6659 H   0  0  0  0  0  0  0  0  0  0  0  0
   -3.9241   -6.4116    6.5064 H   0  0  0  0  0  0  0  0  0  0  0  0
   -3.0620   -0.7879    3.4802 C   0  0  0  0  0  0  0  0  0  0  0  0
   -3.5418   -0.7139    4.7887 C   0  0  0  0  0  0  0  0  0  0  0  0
   -3.2003    0.2836    2.5976 C   0  0  0  0  0  0  0  0  0  0  0  0
   -4.1741    0.4585    5.2179 C   0  0  0  0  0  0  0  0  0  0  0  0
   -3.4383   -1.5449    5.4918 H   0  0  0  0  0  0  0  0  0  0  0  0
   -3.8332    1.4535    3.0329 C   0  0  0  0  0  0  0  0  0  0  0  0
   -2.8269    0.2241    1.5686 H   0  0  0  0  0  0  0  0  0  0  0  0
   -4.3188    1.5398    4.3416 C   0  0  0  0  0  0  0  0  0  0  0  0
   -4.5516    0.5310    6.2386 H   0  0  0  0  0  0  0  0  0  0  0  0
   -3.9429    2.2994    2.3545 H   0  0  0  0  0  0  0  0  0  0  0  0
   -4.8091    2.4538    4.6822 H   0  0  0  0  0  0  0  0  0  0  0  0
   -3.0016   -2.1209   -2.8407 C   0  0  0  0  0  0  0  0  0  0  0  0
   -2.3916   -2.4748   -4.0431 C   0  0  0  0  0  0  0  0  0  0  0  0
   -3.2577   -0.7856   -2.5201 C   0  0  0  0  0  0  0  0  0  0  0  0
   -2.0375   -1.4669   -4.9489 C   0  0  0  0  0  0  0  0  0  0  0  0
   -2.1788   -3.5154   -4.3021 H   0  0  0  0  0  0  0  0  0  0  0  0
   -2.9029    0.2151   -3.4307 C   0  0  0  0  0  0  0  0  0  0  0  0
   -3.7279   -0.5055   -1.5717 H   0  0  0  0  0  0  0  0  0  0  0  0
   -2.2935   -0.1265   -4.6436 C   0  0  0  0  0  0  0  0  0  0  0  0
   -1.5597   -1.7286   -5.8938 H   0  0  0  0  0  0  0  0  0  0  0  0
   -3.0967    1.2617   -3.1964 H   0  0  0  0  0  0  0  0  0  0  0  0
   -2.0157    0.6559   -5.3525 H   0  0  0  0  0  0  0  0  0  0  0  0
   -5.3218   -3.5298   -1.6687 C   0  0  0  0  0  0  0  0  0  0  0  0
   -5.9278   -4.4980   -0.8628 C   0  0  0  0  0  0  0  0  0  0  0  0
   -6.0802   -2.6873   -2.4782 C   0  0  0  0  0  0  0  0  0  0  0  0
   -7.3207   -4.6180   -0.8665 C   0  0  0  0  0  0  0  0  0  0  0  0
   -5.3387   -5.1637   -0.2272 H   0  0  0  0  0  0  0  0  0  0  0  0
   -7.4763   -2.8102   -2.4743 C   0  0  0  0  0  0  0  0  0  0  0  0
   -5.6193   -1.9342   -3.1240 H   0  0  0  0  0  0  0  0  0  0  0  0
   -8.0941   -3.7724   -1.6713 C   0  0  0  0  0  0  0  0  0  0  0  0
   -7.8065   -5.3728   -0.2482 H   0  0  0  0  0  0  0  0  0  0  0  0
   -8.0802   -2.1581   -3.1068 H   0  0  0  0  0  0  0  0  0  0  0  0
   -9.1813   -3.8706   -1.6761 H   0  0  0  0  0  0  0  0  0  0  0  0
   -2.8909   -5.0105   -2.2775 C   0  0  0  0  0  0  0  0  0  0  0  0
   -3.6685   -5.6883   -3.2185 C   0  0  0  0  0  0  0  0  0  0  0  0
   -1.6716   -5.5280   -1.8431 C   0  0  0  0  0  0  0  0  0  0  0  0
   -3.2114   -6.9066   -3.7330 C   0  0  0  0  0  0  0  0  0  0  0  0
   -4.6275   -5.2947   -3.5668 H   0  0  0  0  0  0  0  0  0  0  0  0
   -1.2179   -6.7467   -2.3622 C   0  0  0  0  0  0  0  0  0  0  0  0
   -1.0618   -5.0088   -1.1004 H   0  0  0  0  0  0  0  0  0  0  0  0
   -1.9873   -7.4337   -3.3064 C   0  0  0  0  0  0  0  0  0  0  0  0
   -3.8091   -7.4456   -4.4695 H   0  0  0  0  0  0  0  0  0  0  0  0
   -0.2634   -7.1586   -2.0347 H   0  0  0  0  0  0  0  0  0  0  0  0
   -1.6322   -8.3826   -3.7134 H   0  0  0  0  0  0  0  0  0  0  0  0
   -2.6959   -2.7806    0.5735 Os  0  0  0  0  0  0  0  0  0  0  0  0
   -4.5398   -2.3289    0.9954 N   0  0  0  0  0  0  0  0  0  0  0  0
   -5.5869   -1.8735    1.1703 C   0  0  0  0  0  0  0  0  0  0  0  0
   -6.8932   -1.3006    1.3656 C   0  0  0  0  0  0  0  0  0  0  0  0
   -7.5304   -1.5054    0.4631 H   0  0  0  0  0  0  0  0  0  0  0  0
   -7.3822   -1.7489    2.2693 H   0  0  0  0  0  0  0  0  0  0  0  0
   -6.8047   -0.1913    1.5121 H   0  0  0  0  0  0  0  0  0  0  0  0
  1 78  1  0  0  0  0
```

```
  2 12  1  0  0  0  0
  2 23  1  0  0  0  0
  2 34  1  0  0  0  0
  2 78  1  0  0  0  0
  3 45  1  0  0  0  0
  3 56  1  0  0  0  0
  3 67  1  0  0  0  0
  3 78  1  0  0  0  0
  4  5  2  0  0  0  0
  4 78  1  0  0  0  0
  6  7  3  0  0  0  0
  6 78  1  0  0  0  0
  7  8  1  0  0  0  0
  8  9  1  0  0  0  0
  8 10  1  0  0  0  0
  8 11  1  0  0  0  0
 12 13  2  0  0  0  0
 12 14  1  0  0  0  0
 13 15  1  0  0  0  0
 13 16  1  0  0  0  0
 14 17  2  0  0  0  0
 14 18  1  0  0  0  0
 15 19  2  0  0  0  0
 15 20  1  0  0  0  0
 17 19  1  0  0  0  0
 17 21  1  0  0  0  0
 19 22  1  0  0  0  0
 23 24  2  0  0  0  0
 23 25  1  0  0  0  0
 24 26  1  0  0  0  0
 24 27  1  0  0  0  0
 25 28  2  0  0  0  0
 25 29  1  0  0  0  0
 26 30  2  0  0  0  0
 26 31  1  0  0  0  0
 28 30  1  0  0  0  0
 28 32  1  0  0  0  0
 30 33  1  0  0  0  0
 34 35  2  0  0  0  0
 34 36  1  0  0  0  0
 35 37  1  0  0  0  0
 35 38  1  0  0  0  0
 36 39  2  0  0  0  0
 36 40  1  0  0  0  0
 37 41  2  0  0  0  0
 37 42  1  0  0  0  0
 39 41  1  0  0  0  0
 39 43  1  0  0  0  0
 41 44  1  0  0  0  0
 45 46  2  0  0  0  0
 45 47  1  0  0  0  0
 46 48  1  0  0  0  0
 46 49  1  0  0  0  0
 47 50  2  0  0  0  0
 47 51  1  0  0  0  0
 48 52  2  0  0  0  0
 48 53  1  0  0  0  0
 50 52  1  0  0  0  0
 50 54  1  0  0  0  0
 52 55  1  0  0  0  0
 56 57  2  0  0  0  0
 56 58  1  0  0  0  0
 57 59  1  0  0  0  0
 57 60  1  0  0  0  0
 58 61  2  0  0  0  0
```

```
 58 62  1  0  0  0  0
 59 63  2  0  0  0  0
 59 64  1  0  0  0  0
 61 63  1  0  0  0  0
 61 65  1  0  0  0  0
 63 66  1  0  0  0  0
 67 68  2  0  0  0  0
 67 69  1  0  0  0  0
 68 70  1  0  0  0  0
 68 71  1  0  0  0  0
 69 72  2  0  0  0  0
 69 73  1  0  0  0  0
 70 74  2  0  0  0  0
 70 75  1  0  0  0  0
 72 74  1  0  0  0  0
 72 76  1  0  0  0  0
 74 77  1  0  0  0  0
 78 79  1  0  0  0  0
 79 80  3  0  0  0  0
 80 81  1  0  0  0  0
 81 82  1  0  0  0  0
 81 83  1  0  0  0  0
 81 84  1  0  0  0  0
M  END
$$$$
mol015
Optimized Catalyst 015
Optimized Catalyst 015
 79 85  0  0  0  0  0  0  0  0999 V2000
   -1.0841    2.0749   -0.1382 Ru  0  0  0  0  0  0  0  0  0  0  0  0
   -0.1029    1.9784   -1.8736 H   0  0  0  0  0  0  0  0  0  0  0  0
    0.7671    2.4735    0.8193 O   0  0  0  0  0  0  0  0  0  0  0  0
   -0.0545    1.6259    2.7137 O   0  0  0  0  0  0  0  0  0  0  0  0
    0.8491    2.1684    2.0911 C   0  0  0  0  0  0  0  0  0  0  0  0
    2.2032    2.5494    2.6731 C   0  0  0  0  0  0  0  0  0  0  0  0
    2.8740    1.6588    2.6391 H   0  0  0  0  0  0  0  0  0  0  0  0
    2.6343    3.4077    2.1059 H   0  0  0  0  0  0  0  0  0  0  0  0
    2.0307    3.0503    4.3675 Cl  0  0  0  0  0  0  0  0  0  0  0  0
   -2.6938    2.0271   -1.0556 C   0  0  0  0  0  0  0  0  0  0  0  0
   -3.7092    2.0226   -1.6149 O   0  0  0  0  0  0  0  0  0  0  0  0
   -0.6977    4.2826   -0.0766 P   0  0  0  0  0  0  0  0  0  0  0  0
   -1.1175   -0.1984   -0.0068 P   0  0  0  0  0  0  0  0  0  0  0  0
    0.8035    4.9987   -0.9537 C   0  0  0  0  0  0  0  0  0  0  0  0
    0.7561    6.2799   -1.5095 C   0  0  0  0  0  0  0  0  0  0  0  0
    1.9758    4.2444   -1.0063 C   0  0  0  0  0  0  0  0  0  0  0  0
    1.8951    6.8062   -2.1290 C   0  0  0  0  0  0  0  0  0  0  0  0
   -0.1508    6.8895   -1.4763 H   0  0  0  0  0  0  0  0  0  0  0  0
    3.1153    4.7765   -1.6226 C   0  0  0  0  0  0  0  0  0  0  0  0
    2.0233    3.2380   -0.5613 H   0  0  0  0  0  0  0  0  0  0  0  0
    3.0746    6.0557   -2.1843 C   0  0  0  0  0  0  0  0  0  0  0  0
    1.8630    7.8025   -2.5669 H   0  0  0  0  0  0  0  0  0  0  0  0
    4.0329    4.1920   -1.6619 H   0  0  0  0  0  0  0  0  0  0  0  0
    3.9610    6.4694   -2.6643 H   0  0  0  0  0  0  0  0  0  0  0  0
   -0.5657    4.9600    1.6649 C   0  0  0  0  0  0  0  0  0  0  0  0
   -1.3304    4.3318    2.6517 C   0  0  0  0  0  0  0  0  0  0  0  0
    0.2674    6.0349    1.9742 C   0  0  0  0  0  0  0  0  0  0  0  0
   -1.2748    4.8052    3.9677 C   0  0  0  0  0  0  0  0  0  0  0  0
   -1.9469    3.4517    2.4238 H   0  0  0  0  0  0  0  0  0  0  0  0
    0.3215    6.5011    3.2933 C   0  0  0  0  0  0  0  0  0  0  0  0
    0.8866    6.5189    1.2161 H   0  0  0  0  0  0  0  0  0  0  0  0
   -0.4505    5.8894    4.2864 C   0  0  0  0  0  0  0  0  0  0  0  0
   -1.8540    4.3105    4.7461 H   0  0  0  0  0  0  0  0  0  0  0  0
    0.9739    7.3344    3.5478 H   0  0  0  0  0  0  0  0  0  0  0  0
   -0.3986    6.2485    5.3139 H   0  0  0  0  0  0  0  0  0  0  0  0
   -2.0785    5.3435   -0.8131 C   0  0  0  0  0  0  0  0  0  0  0  0
```

```
   -2.8137    6.2285   -0.0268 C   0  0  0  0  0  0  0  0  0  0  0  0
   -2.3467    5.2120   -2.1791 C   0  0  0  0  0  0  0  0  0  0  0  0
   -3.8308    6.9939   -0.6142 C   0  0  0  0  0  0  0  0  0  0  0  0
   -2.6192    6.3458    1.0430 H   0  0  0  0  0  0  0  0  0  0  0  0
   -3.3590    5.9801   -2.7626 C   0  0  0  0  0  0  0  0  0  0  0  0
   -1.7792    4.5221   -2.8075 H   0  0  0  0  0  0  0  0  0  0  0  0
   -4.1016    6.8717   -1.9791 C   0  0  0  0  0  0  0  0  0  0  0  0
   -4.4096    7.6849   -0.0026 H   0  0  0  0  0  0  0  0  0  0  0  0
   -3.5724    5.8825   -3.8261 H   0  0  0  0  0  0  0  0  0  0  0  0
   -4.8918    7.4678   -2.4348 H   0  0  0  0  0  0  0  0  0  0  0  0
    0.5099   -0.9647    0.5267 C   0  0  0  0  0  0  0  0  0  0  0  0
    0.5452   -2.1736    1.2227 C   0  0  0  0  0  0  0  0  0  0  0  0
    1.6862   -0.2775    0.2207 C   0  0  0  0  0  0  0  0  0  0  0  0
    1.7818   -2.7121    1.5995 C   0  0  0  0  0  0  0  0  0  0  0  0
   -0.3690   -2.7079    1.4921 H   0  0  0  0  0  0  0  0  0  0  0  0
    2.9197   -0.8212    0.5972 C   0  0  0  0  0  0  0  0  0  0  0  0
    1.6599    0.6993   -0.2838 H   0  0  0  0  0  0  0  0  0  0  0  0
    2.9664   -2.0390    1.2837 C   0  0  0  0  0  0  0  0  0  0  0  0
    1.8191   -3.6526    2.1470 H   0  0  0  0  0  0  0  0  0  0  0  0
    3.8410   -0.2919    0.3616 H   0  0  0  0  0  0  0  0  0  0  0  0
    3.9266   -2.4604    1.5799 H   0  0  0  0  0  0  0  0  0  0  0  0
   -1.5209   -1.1678   -1.5710 C   0  0  0  0  0  0  0  0  0  0  0  0
   -0.5331   -1.9145   -2.2128 C   0  0  0  0  0  0  0  0  0  0  0  0
   -2.8236   -1.1295   -2.0765 C   0  0  0  0  0  0  0  0  0  0  0  0
   -0.8519   -2.6319   -3.3736 C   0  0  0  0  0  0  0  0  0  0  0  0
    0.4915   -1.9618   -1.8333 H   0  0  0  0  0  0  0  0  0  0  0  0
   -3.1387   -1.8496   -3.2335 C   0  0  0  0  0  0  0  0  0  0  0  0
   -3.6136   -0.5505   -1.5903 H   0  0  0  0  0  0  0  0  0  0  0  0
   -2.1528   -2.6016   -3.8824 C   0  0  0  0  0  0  0  0  0  0  0  0
   -0.0826   -3.2163   -3.8770 H   0  0  0  0  0  0  0  0  0  0  0  0
   -4.1534   -1.8233   -3.6295 H   0  0  0  0  0  0  0  0  0  0  0  0
   -2.4005   -3.1623   -4.7833 H   0  0  0  0  0  0  0  0  0  0  0  0
   -2.3425   -0.8576    1.2442 C   0  0  0  0  0  0  0  0  0  0  0  0
   -2.5138   -0.0921    2.3984 C   0  0  0  0  0  0  0  0  0  0  0  0
   -3.0268   -2.0618    1.0671 C   0  0  0  0  0  0  0  0  0  0  0  0
   -3.3923   -0.5403    3.3936 C   0  0  0  0  0  0  0  0  0  0  0  0
   -1.9536    0.8502    2.5524 H   0  0  0  0  0  0  0  0  0  0  0  0
   -3.9077   -2.4998    2.0614 C   0  0  0  0  0  0  0  0  0  0  0  0
   -2.8928   -2.6731    0.1721 H   0  0  0  0  0  0  0  0  0  0  0  0
   -4.0886   -1.7399    3.2236 C   0  0  0  0  0  0  0  0  0  0  0  0
   -3.5231    0.0469    4.3017 H   0  0  0  0  0  0  0  0  0  0  0  0
   -4.4507   -3.4343    1.9334 H   0  0  0  0  0  0  0  0  0  0  0  0
   -4.7721   -2.0872    3.9979 H   0  0  0  0  0  0  0  0  0  0  0  0
  1 10  1  0  0  0  0
  1 12  1  0  0  0  0
  1 13  1  0  0  0  0
  1  3  1  0  0  0  0
  1  4  1  0  0  0  0
  1  2  1  0  0  0  0
  3  5  1  0  0  0  0
  4  5  2  0  0  0  0
  5  6  1  0  0  0  0
  6  7  1  0  0  0  0
  6  8  1  0  0  0  0
  6  9  1  0  0  0  0
 10 11  2  0  0  0  0
 12 14  1  0  0  0  0
 12 25  1  0  0  0  0
 12 36  1  0  0  0  0
 13 47  1  0  0  0  0
 13 58  1  0  0  0  0
 13 69  1  0  0  0  0
 14 15  2  0  0  0  0
 14 16  1  0  0  0  0
 15 17  1  0  0  0  0
```

```
 15 18  1  0  0  0  0
 16 19  2  0  0  0  0
 16 20  1  0  0  0  0
 17 21  2  0  0  0  0
 17 22  1  0  0  0  0
 19 21  1  0  0  0  0
 19 23  1  0  0  0  0
 21 24  1  0  0  0  0
 25 26  2  0  0  0  0
 25 27  1  0  0  0  0
 26 28  1  0  0  0  0
 26 29  1  0  0  0  0
 27 30  2  0  0  0  0
 27 31  1  0  0  0  0
 28 32  2  0  0  0  0
 28 33  1  0  0  0  0
 30 32  1  0  0  0  0
 30 34  1  0  0  0  0
 32 35  1  0  0  0  0
 36 37  2  0  0  0  0
 36 38  1  0  0  0  0
 37 39  1  0  0  0  0
 37 40  1  0  0  0  0
 38 41  2  0  0  0  0
 38 42  1  0  0  0  0
 39 43  2  0  0  0  0
 39 44  1  0  0  0  0
 41 43  1  0  0  0  0
 41 45  1  0  0  0  0
 43 46  1  0  0  0  0
 47 48  2  0  0  0  0
 47 49  1  0  0  0  0
 48 50  1  0  0  0  0
 48 51  1  0  0  0  0
 49 52  2  0  0  0  0
 49 53  1  0  0  0  0
 50 54  2  0  0  0  0
 50 55  1  0  0  0  0
 52 54  1  0  0  0  0
 52 56  1  0  0  0  0
 54 57  1  0  0  0  0
 58 59  2  0  0  0  0
 58 60  1  0  0  0  0
 59 61  1  0  0  0  0
 59 62  1  0  0  0  0
 60 63  2  0  0  0  0
 60 64  1  0  0  0  0
 61 65  2  0  0  0  0
 61 66  1  0  0  0  0
 63 65  1  0  0  0  0
 63 67  1  0  0  0  0
 65 68  1  0  0  0  0
 69 70  2  0  0  0  0
 69 71  1  0  0  0  0
 70 72  1  0  0  0  0
 70 73  1  0  0  0  0
 71 74  2  0  0  0  0
 71 75  1  0  0  0  0
 72 76  2  0  0  0  0
 72 77  1  0  0  0  0
 74 76  1  0  0  0  0
 74 78  1  0  0  0  0
 76 79  1  0  0  0  0
M  END
$$$$
```

```
mol016
Optimized Catalyst 016
Optimized Catalyst 016
 86 93  0  0  0  0  0  0  0  0999 V2000
   -1.0579    2.0190   -0.2120 Ru  0  0  0  0  0  0  0  0  0  0  0  0
   -0.1213    1.9207   -1.9716 H   0  0  0  0  0  0  0  0  0  0  0  0
    0.8245    2.4132    0.6870 O   0  0  0  0  0  0  0  0  0  0  0  0
    0.0019    1.6138    2.5930 O   0  0  0  0  0  0  0  0  0  0  0  0
    0.9154    2.1578    1.9699 C   0  0  0  0  0  0  0  0  0  0  0  0
   -2.6914    1.9782   -1.0855 C   0  0  0  0  0  0  0  0  0  0  0  0
   -3.7218    1.9792   -1.6177 O   0  0  0  0  0  0  0  0  0  0  0  0
   -0.6740    4.2276   -0.1519 P   0  0  0  0  0  0  0  0  0  0  0  0
   -1.1105   -0.2520   -0.0819 P   0  0  0  0  0  0  0  0  0  0  0  0
    0.7975    4.9668   -1.0560 C   0  0  0  0  0  0  0  0  0  0  0  0
    0.7355    6.2614   -1.5784 C   0  0  0  0  0  0  0  0  0  0  0  0
    1.9666    4.2135   -1.1591 C   0  0  0  0  0  0  0  0  0  0  0  0
    1.8579    6.8022   -2.2151 C   0  0  0  0  0  0  0  0  0  0  0  0
   -0.1698    6.8695   -1.5051 H   0  0  0  0  0  0  0  0  0  0  0  0
    3.0907    4.7608   -1.7903 C   0  0  0  0  0  0  0  0  0  0  0  0
    2.0222    3.1972   -0.7383 H   0  0  0  0  0  0  0  0  0  0  0  0
    3.0356    6.0534   -2.3187 C   0  0  0  0  0  0  0  0  0  0  0  0
    1.8152    7.8085   -2.6280 H   0  0  0  0  0  0  0  0  0  0  0  0
    4.0072    4.1784   -1.8640 H   0  0  0  0  0  0  0  0  0  0  0  0
    3.9095    6.4796   -2.8103 H   0  0  0  0  0  0  0  0  0  0  0  0
   -0.5105    4.8904    1.5946 C   0  0  0  0  0  0  0  0  0  0  0  0
   -1.3085    4.2952    2.5762 C   0  0  0  0  0  0  0  0  0  0  0  0
    0.3797    5.9148    1.9140 C   0  0  0  0  0  0  0  0  0  0  0  0
   -1.2321    4.7563    3.8951 C   0  0  0  0  0  0  0  0  0  0  0  0
   -1.9679    3.4495    2.3394 H   0  0  0  0  0  0  0  0  0  0  0  0
    0.4565    6.3670    3.2377 C   0  0  0  0  0  0  0  0  0  0  0  0
    1.0301    6.3668    1.1614 H   0  0  0  0  0  0  0  0  0  0  0  0
   -0.3516    5.7930    4.2236 C   0  0  0  0  0  0  0  0  0  0  0  0
   -1.8456    4.2942    4.6668 H   0  0  0  0  0  0  0  0  0  0  0  0
    1.1545    7.1604    3.4988 H   0  0  0  0  0  0  0  0  0  0  0  0
   -0.2885    6.1464    5.2521 H   0  0  0  0  0  0  0  0  0  0  0  0
   -2.0788    5.2887   -0.8443 C   0  0  0  0  0  0  0  0  0  0  0  0
   -2.7925    6.1723   -0.0369 C   0  0  0  0  0  0  0  0  0  0  0  0
   -2.3852    5.1587   -2.2023 C   0  0  0  0  0  0  0  0  0  0  0  0
   -3.8273    6.9364   -0.5944 C   0  0  0  0  0  0  0  0  0  0  0  0
   -2.5676    6.2898    1.0266 H   0  0  0  0  0  0  0  0  0  0  0  0
   -3.4152    5.9254   -2.7561 C   0  0  0  0  0  0  0  0  0  0  0  0
   -1.8343    4.4708   -2.8475 H   0  0  0  0  0  0  0  0  0  0  0  0
   -4.1370    6.8147   -1.9510 C   0  0  0  0  0  0  0  0  0  0  0  0
   -4.3895    7.6259    0.0340 H   0  0  0  0  0  0  0  0  0  0  0  0
   -3.6586    5.8281   -3.8131 H   0  0  0  0  0  0  0  0  0  0  0  0
   -4.9412    7.4094   -2.3833 H   0  0  0  0  0  0  0  0  0  0  0  0
    0.5017   -1.0616    0.4286 C   0  0  0  0  0  0  0  0  0  0  0  0
    0.5141   -2.2943    1.0830 C   0  0  0  0  0  0  0  0  0  0  0  0
    1.6896   -0.3852    0.1467 C   0  0  0  0  0  0  0  0  0  0  0  0
    1.7400   -2.8675    1.4416 C   0  0  0  0  0  0  0  0  0  0  0  0
   -0.4097   -2.8208    1.3327 H   0  0  0  0  0  0  0  0  0  0  0  0
    2.9128   -0.9635    0.5058 C   0  0  0  0  0  0  0  0  0  0  0  0
    1.6792    0.6089   -0.3245 H   0  0  0  0  0  0  0  0  0  0  0  0
    2.9368   -2.2042    1.1506 C   0  0  0  0  0  0  0  0  0  0  0  0
    1.7603   -3.8264    1.9565 H   0  0  0  0  0  0  0  0  0  0  0  0
    3.8440   -0.4409    0.2954 H   0  0  0  0  0  0  0  0  0  0  0  0
    3.8888   -2.6508    1.4357 H   0  0  0  0  0  0  0  0  0  0  0  0
   -1.5562   -1.2132   -1.6409 C   0  0  0  0  0  0  0  0  0  0  0  0
   -0.5879   -1.9696   -2.3011 C   0  0  0  0  0  0  0  0  0  0  0  0
   -2.8663   -1.1591   -2.1249 C   0  0  0  0  0  0  0  0  0  0  0  0
   -0.9341   -2.6814   -3.4575 C   0  0  0  0  0  0  0  0  0  0  0  0
    0.4426   -2.0289   -1.9393 H   0  0  0  0  0  0  0  0  0  0  0  0
   -3.2090   -1.8735   -3.2776 C   0  0  0  0  0  0  0  0  0  0  0  0
   -3.6413   -0.5711   -1.6254 H   0  0  0  0  0  0  0  0  0  0  0  0
   -2.2430   -2.6357   -3.9441 C   0  0  0  0  0  0  0  0  0  0  0  0
```

```
   -0.1800   -3.2733   -3.9747 H   0  0  0  0  0  0  0  0  0  0  0  0
   -4.2297   -1.8348   -3.6565 H   0  0  0  0  0  0  0  0  0  0  0  0
   -2.5120   -3.1921   -4.8415 H   0  0  0  0  0  0  0  0  0  0  0  0
   -2.3355   -0.8801    1.1860 C   0  0  0  0  0  0  0  0  0  0  0  0
   -2.4685   -0.1078    2.3409 C   0  0  0  0  0  0  0  0  0  0  0  0
   -3.0558   -2.0646    1.0213 C   0  0  0  0  0  0  0  0  0  0  0  0
   -3.3460   -0.5284    3.3487 C   0  0  0  0  0  0  0  0  0  0  0  0
   -1.8782    0.8188    2.4841 H   0  0  0  0  0  0  0  0  0  0  0  0
   -3.9355   -2.4751    2.0285 C   0  0  0  0  0  0  0  0  0  0  0  0
   -2.9513   -2.6815    0.1262 H   0  0  0  0  0  0  0  0  0  0  0  0
   -4.0791   -1.7077    3.1909 C   0  0  0  0  0  0  0  0  0  0  0  0
   -3.4482    0.0641    4.2568 H   0  0  0  0  0  0  0  0  0  0  0  0
   -4.5067   -3.3938    1.9101 H   0  0  0  0  0  0  0  0  0  0  0  0
   -4.7622   -2.0333    3.9747 H   0  0  0  0  0  0  0  0  0  0  0  0
    2.1853    2.6213    2.6206 C   0  0  0  0  0  0  0  0  0  0  0  0
    2.1259    3.1024    3.9360 C   0  0  0  0  0  0  0  0  0  0  0  0
    3.4060    2.5884    1.9369 C   0  0  0  0  0  0  0  0  0  0  0  0
    3.2885    3.5591    4.5619 C   0  0  0  0  0  0  0  0  0  0  0  0
    1.1657    3.1160    4.4570 H   0  0  0  0  0  0  0  0  0  0  0  0
    4.5688    3.0392    2.5689 C   0  0  0  0  0  0  0  0  0  0  0  0
    3.4411    2.2136    0.9151 H   0  0  0  0  0  0  0  0  0  0  0  0
    4.5092    3.5264    3.8788 C   0  0  0  0  0  0  0  0  0  0  0  0
    3.2433    3.9370    5.5813 H   0  0  0  0  0  0  0  0  0  0  0  0
    5.5192    3.0131    2.0403 H   0  0  0  0  0  0  0  0  0  0  0  0
    5.4151    3.8804    4.3684 H   0  0  0  0  0  0  0  0  0  0  0  0
  1  6  1  0  0  0  0
  1  8  1  0  0  0  0
  1  9  1  0  0  0  0
  1  2  1  0  0  0  0
  1  4  1  0  0  0  0
  1  3  1  0  0  0  0
  3  5  1  0  0  0  0
  4  5  2  0  0  0  0
  5 76  1  0  0  0  0
  6  7  2  0  0  0  0
  8 10  1  0  0  0  0
  8 21  1  0  0  0  0
  8 32  1  0  0  0  0
  9 43  1  0  0  0  0
  9 54  1  0  0  0  0
  9 65  1  0  0  0  0
 10 11  2  0  0  0  0
 10 12  1  0  0  0  0
 11 13  1  0  0  0  0
 11 14  1  0  0  0  0
 12 15  2  0  0  0  0
 12 16  1  0  0  0  0
 13 17  2  0  0  0  0
 13 18  1  0  0  0  0
 15 17  1  0  0  0  0
 15 19  1  0  0  0  0
 17 20  1  0  0  0  0
 21 22  2  0  0  0  0
 21 23  1  0  0  0  0
 22 24  1  0  0  0  0
 22 25  1  0  0  0  0
 23 26  2  0  0  0  0
 23 27  1  0  0  0  0
 24 28  2  0  0  0  0
 24 29  1  0  0  0  0
 26 28  1  0  0  0  0
 26 30  1  0  0  0  0
 28 31  1  0  0  0  0
 32 33  2  0  0  0  0
 32 34  1  0  0  0  0
```

```
 33 35  1  0  0  0  0
 33 36  1  0  0  0  0
 34 37  2  0  0  0  0
 34 38  1  0  0  0  0
 35 39  2  0  0  0  0
 35 40  1  0  0  0  0
 37 39  1  0  0  0  0
 37 41  1  0  0  0  0
 39 42  1  0  0  0  0
 43 44  2  0  0  0  0
 43 45  1  0  0  0  0
 44 46  1  0  0  0  0
 44 47  1  0  0  0  0
 45 48  2  0  0  0  0
 45 49  1  0  0  0  0
 46 50  2  0  0  0  0
 46 51  1  0  0  0  0
 48 50  1  0  0  0  0
 48 52  1  0  0  0  0
 50 53  1  0  0  0  0
 54 55  2  0  0  0  0
 54 56  1  0  0  0  0
 55 57  1  0  0  0  0
 55 58  1  0  0  0  0
 56 59  2  0  0  0  0
 56 60  1  0  0  0  0
 57 61  2  0  0  0  0
 57 62  1  0  0  0  0
 59 61  1  0  0  0  0
 59 63  1  0  0  0  0
 61 64  1  0  0  0  0
 65 66  2  0  0  0  0
 65 67  1  0  0  0  0
 66 68  1  0  0  0  0
 66 69  1  0  0  0  0
 67 70  2  0  0  0  0
 67 71  1  0  0  0  0
 68 72  2  0  0  0  0
 68 73  1  0  0  0  0
 70 72  1  0  0  0  0
 70 74  1  0  0  0  0
 72 75  1  0  0  0  0
 76 77  2  0  0  0  0
 76 78  1  0  0  0  0
 77 79  1  0  0  0  0
 77 80  1  0  0  0  0
 78 81  2  0  0  0  0
 78 82  1  0  0  0  0
 79 83  2  0  0  0  0
 79 84  1  0  0  0  0
 81 83  1  0  0  0  0
 81 85  1  0  0  0  0
 83 86  1  0  0  0  0
M  END
$$$$
mol017
Optimized Catalyst 017
Optimized Catalyst 017
 79 85  0  0  0  0  0  0  0  0999 V2000
   -1.0901    2.0342   -0.1521 Ru  0  0  0  0  0  0  0  0  0  0  0  0
   -0.0842    1.9542   -1.8743 H   0  0  0  0  0  0  0  0  0  0  0  0
    0.7503    2.4227    0.8160 O   0  0  0  0  0  0  0  0  0  0  0  0
   -0.1396    1.5938    2.6788 O   0  0  0  0  0  0  0  0  0  0  0  0
    0.8075    2.1207    2.0929 C   0  0  0  0  0  0  0  0  0  0  0  0
   -2.6954    1.9877   -1.0812 C   0  0  0  0  0  0  0  0  0  0  0  0
```

```
   -3.7084    1.9848   -1.6454 O   0  0  0  0  0  0  0  0  0  0  0  0
   -0.7093    4.2399   -0.0847 P   0  0  0  0  0  0  0  0  0  0  0  0
   -1.1258   -0.2407   -0.0546 P   0  0  0  0  0  0  0  0  0  0  0  0
    0.7903    4.9620   -0.9607 C   0  0  0  0  0  0  0  0  0  0  0  0
    0.7194    6.2067   -1.5916 C   0  0  0  0  0  0  0  0  0  0  0  0
    1.9881    4.2471   -0.9331 C   0  0  0  0  0  0  0  0  0  0  0  0
    1.8593    6.7343   -2.2086 C   0  0  0  0  0  0  0  0  0  0  0  0
   -0.2061    6.7877   -1.6197 H   0  0  0  0  0  0  0  0  0  0  0  0
    3.1280    4.7806   -1.5473 C   0  0  0  0  0  0  0  0  0  0  0  0
    2.0543    3.2704   -0.4251 H   0  0  0  0  0  0  0  0  0  0  0  0
    3.0634    6.0222   -2.1856 C   0  0  0  0  0  0  0  0  0  0  0  0
    1.8085    7.7015   -2.7054 H   0  0  0  0  0  0  0  0  0  0  0  0
    4.0645    4.2262   -1.5244 H   0  0  0  0  0  0  0  0  0  0  0  0
    3.9499    6.4366   -2.6644 H   0  0  0  0  0  0  0  0  0  0  0  0
   -0.5791    4.9424    1.6495 C   0  0  0  0  0  0  0  0  0  0  0  0
   -1.2904    4.2926    2.6610 C   0  0  0  0  0  0  0  0  0  0  0  0
    0.2027    6.0645    1.9253 C   0  0  0  0  0  0  0  0  0  0  0  0
   -1.2301    4.7880    3.9692 C   0  0  0  0  0  0  0  0  0  0  0  0
   -1.8656    3.3769    2.4590 H   0  0  0  0  0  0  0  0  0  0  0  0
    0.2608    6.5533    3.2358 C   0  0  0  0  0  0  0  0  0  0  0  0
    0.7775    6.5692    1.1455 H   0  0  0  0  0  0  0  0  0  0  0  0
   -0.4572    5.9178    4.2544 C   0  0  0  0  0  0  0  0  0  0  0  0
   -1.7729    4.2794    4.7649 H   0  0  0  0  0  0  0  0  0  0  0  0
    0.8688    7.4272    3.4622 H   0  0  0  0  0  0  0  0  0  0  0  0
   -0.4083    6.2996    5.2737 H   0  0  0  0  0  0  0  0  0  0  0  0
   -2.0974    5.2906   -0.8259 C   0  0  0  0  0  0  0  0  0  0  0  0
   -2.8286    6.1862   -0.0481 C   0  0  0  0  0  0  0  0  0  0  0  0
   -2.3764    5.1375   -2.1876 C   0  0  0  0  0  0  0  0  0  0  0  0
   -3.8515    6.9409   -0.6397 C   0  0  0  0  0  0  0  0  0  0  0  0
   -2.6273    6.3201    1.0182 H   0  0  0  0  0  0  0  0  0  0  0  0
   -3.3943    5.8947   -2.7754 C   0  0  0  0  0  0  0  0  0  0  0  0
   -1.8133    4.4379   -2.8094 H   0  0  0  0  0  0  0  0  0  0  0  0
   -4.1325    6.7973   -2.0004 C   0  0  0  0  0  0  0  0  0  0  0  0
   -4.4271    7.6399   -0.0344 H   0  0  0  0  0  0  0  0  0  0  0  0
   -3.6161    5.7794   -3.8354 H   0  0  0  0  0  0  0  0  0  0  0  0
   -4.9272    7.3847   -2.4591 H   0  0  0  0  0  0  0  0  0  0  0  0
    0.4856   -1.0417    0.4753 C   0  0  0  0  0  0  0  0  0  0  0  0
    0.5001   -2.2965    1.0864 C   0  0  0  0  0  0  0  0  0  0  0  0
    1.6704   -0.3395    0.2488 C   0  0  0  0  0  0  0  0  0  0  0  0
    1.7248   -2.8653    1.4560 C   0  0  0  0  0  0  0  0  0  0  0  0
   -0.4216   -2.8447    1.2932 H   0  0  0  0  0  0  0  0  0  0  0  0
    2.8926   -0.9130    0.6197 C   0  0  0  0  0  0  0  0  0  0  0  0
    1.6600    0.6712   -0.1877 H   0  0  0  0  0  0  0  0  0  0  0  0
    2.9188   -2.1759    1.2193 C   0  0  0  0  0  0  0  0  0  0  0  0
    1.7460   -3.8423    1.9356 H   0  0  0  0  0  0  0  0  0  0  0  0
    3.8199   -0.3693    0.4497 H   0  0  0  0  0  0  0  0  0  0  0  0
    3.8699   -2.6208    1.5101 H   0  0  0  0  0  0  0  0  0  0  0  0
   -1.5195   -1.1723   -1.6457 C   0  0  0  0  0  0  0  0  0  0  0  0
   -0.5220   -1.8894   -2.3061 C   0  0  0  0  0  0  0  0  0  0  0  0
   -2.8213   -1.1350   -2.1534 C   0  0  0  0  0  0  0  0  0  0  0  0
   -0.8302   -2.5790   -3.4865 C   0  0  0  0  0  0  0  0  0  0  0  0
    0.5026   -1.9345   -1.9263 H   0  0  0  0  0  0  0  0  0  0  0  0
   -3.1259   -1.8267   -3.3304 C   0  0  0  0  0  0  0  0  0  0  0  0
   -3.6187   -0.5775   -1.6542 H   0  0  0  0  0  0  0  0  0  0  0  0
   -2.1304   -2.5499   -3.9971 C   0  0  0  0  0  0  0  0  0  0  0  0
   -0.0533   -3.1404   -4.0038 H   0  0  0  0  0  0  0  0  0  0  0  0
   -4.1398   -1.8005   -3.7281 H   0  0  0  0  0  0  0  0  0  0  0  0
   -2.3699   -3.0888   -4.9133 H   0  0  0  0  0  0  0  0  0  0  0  0
   -2.3721   -0.9188    1.1659 C   0  0  0  0  0  0  0  0  0  0  0  0
   -2.5598   -0.1706    2.3287 C   0  0  0  0  0  0  0  0  0  0  0  0
   -3.0573   -2.1177    0.9592 C   0  0  0  0  0  0  0  0  0  0  0  0
   -3.4561   -0.6309    3.3024 C   0  0  0  0  0  0  0  0  0  0  0  0
   -1.9988    0.7683    2.5047 H   0  0  0  0  0  0  0  0  0  0  0  0
   -3.9557   -2.5681    1.9321 C   0  0  0  0  0  0  0  0  0  0  0  0
   -2.9115   -2.7153    0.0569 H   0  0  0  0  0  0  0  0  0  0  0  0
```

```
   -4.1534   -1.8253    3.1027 C   0  0  0  0  0  0  0  0  0  0  0  0
   -3.6009   -0.0568    4.2164 H   0  0  0  0  0  0  0  0  0  0  0  0
   -4.4996   -3.4984    1.7808 H   0  0  0  0  0  0  0  0  0  0  0  0
   -4.8510   -2.1818    3.8598 H   0  0  0  0  0  0  0  0  0  0  0  0
    2.0961    2.5073    2.7649 C   0  0  0  0  0  0  0  0  0  0  0  0
    2.2546    1.8641    3.6599 H   0  0  0  0  0  0  0  0  0  0  0  0
    2.9471    2.3986    2.0585 H   0  0  0  0  0  0  0  0  0  0  0  0
    2.0137    3.5737    3.0844 H   0  0  0  0  0  0  0  0  0  0  0  0
  1  6  1  0  0  0  0
  1  8  1  0  0  0  0
  1  9  1  0  0  0  0
  1  2  1  0  0  0  0
  1  4  1  0  0  0  0
  1  3  1  0  0  0  0
  3  5  1  0  0  0  0
  4  5  2  0  0  0  0
  5 76  1  0  0  0  0
  6  7  2  0  0  0  0
  8 10  1  0  0  0  0
  8 21  1  0  0  0  0
  8 32  1  0  0  0  0
  9 43  1  0  0  0  0
  9 54  1  0  0  0  0
  9 65  1  0  0  0  0
 10 11  2  0  0  0  0
 10 12  1  0  0  0  0
 11 13  1  0  0  0  0
 11 14  1  0  0  0  0
 12 15  2  0  0  0  0
 12 16  1  0  0  0  0
 13 17  2  0  0  0  0
 13 18  1  0  0  0  0
 15 17  1  0  0  0  0
 15 19  1  0  0  0  0
 17 20  1  0  0  0  0
 21 22  2  0  0  0  0
 21 23  1  0  0  0  0
 22 24  1  0  0  0  0
 22 25  1  0  0  0  0
 23 26  2  0  0  0  0
 23 27  1  0  0  0  0
 24 28  2  0  0  0  0
 24 29  1  0  0  0  0
 26 28  1  0  0  0  0
 26 30  1  0  0  0  0
 28 31  1  0  0  0  0
 32 33  2  0  0  0  0
 32 34  1  0  0  0  0
 33 35  1  0  0  0  0
 33 36  1  0  0  0  0
 34 37  2  0  0  0  0
 34 38  1  0  0  0  0
 35 39  2  0  0  0  0
 35 40  1  0  0  0  0
 37 39  1  0  0  0  0
 37 41  1  0  0  0  0
 39 42  1  0  0  0  0
 43 44  2  0  0  0  0
 43 45  1  0  0  0  0
 44 46  1  0  0  0  0
 44 47  1  0  0  0  0
 45 48  2  0  0  0  0
 45 49  1  0  0  0  0
 46 50  2  0  0  0  0
 46 51  1  0  0  0  0
```

```
 48 50  1  0  0  0  0
 48 52  1  0  0  0  0
 50 53  1  0  0  0  0
 54 55  2  0  0  0  0
 54 56  1  0  0  0  0
 55 57  1  0  0  0  0
 55 58  1  0  0  0  0
 56 59  2  0  0  0  0
 56 60  1  0  0  0  0
 57 61  2  0  0  0  0
 57 62  1  0  0  0  0
 59 61  1  0  0  0  0
 59 63  1  0  0  0  0
 61 64  1  0  0  0  0
 65 66  2  0  0  0  0
 65 67  1  0  0  0  0
 66 68  1  0  0  0  0
 66 69  1  0  0  0  0
 67 70  2  0  0  0  0
 67 71  1  0  0  0  0
 68 72  2  0  0  0  0
 68 73  1  0  0  0  0
 70 72  1  0  0  0  0
 70 74  1  0  0  0  0
 72 75  1  0  0  0  0
 76 77  1  0  0  0  0
 76 78  1  0  0  0  0
 76 79  1  0  0  0  0
M  END
$$$$
mol018
Optimized Catalyst 018
Optimized Catalyst 018
 85 91  0  0  0  0  0  0  0  0999 V2000
   -1.0114    2.1345   -0.2688 Ru  0  0  0  0  0  0  0  0  0  0  0  0
    0.1454    2.0989   -1.8940 H   0  0  0  0  0  0  0  0  0  0  0  0
    0.7031    2.6813    0.8628 O   0  0  0  0  0  0  0  0  0  0  0  0
    0.1590    0.8648    1.9840 O   0  0  0  0  0  0  0  0  0  0  0  0
    0.8670    1.8872    1.8800 C   0  0  0  0  0  0  0  0  0  0  0  0
   -2.5342    1.9648   -1.3212 C   0  0  0  0  0  0  0  0  0  0  0  0
   -3.4982    1.8928   -1.9590 O   0  0  0  0  0  0  0  0  0  0  0  0
   -0.8119    4.3575   -0.1870 P   0  0  0  0  0  0  0  0  0  0  0  0
   -0.9014   -0.1182    0.0760 P   0  0  0  0  0  0  0  0  0  0  0  0
    0.7112    5.2421   -0.8433 C   0  0  0  0  0  0  0  0  0  0  0  0
    0.6125    6.5233   -1.3925 C   0  0  0  0  0  0  0  0  0  0  0  0
    1.9491    4.6078   -0.7378 C   0  0  0  0  0  0  0  0  0  0  0  0
    1.7679    7.1734   -1.8399 C   0  0  0  0  0  0  0  0  0  0  0  0
   -0.3473    7.0375   -1.4883 H   0  0  0  0  0  0  0  0  0  0  0  0
    3.1043    5.2644   -1.1808 C   0  0  0  0  0  0  0  0  0  0  0  0
    2.0360    3.5990   -0.3017 H   0  0  0  0  0  0  0  0  0  0  0  0
    3.0138    6.5456   -1.7315 C   0  0  0  0  0  0  0  0  0  0  0  0
    1.6969    8.1701   -2.2717 H   0  0  0  0  0  0  0  0  0  0  0  0
    4.0725    4.7745   -1.0928 H   0  0  0  0  0  0  0  0  0  0  0  0
    3.9122    7.0562   -2.0764 H   0  0  0  0  0  0  0  0  0  0  0  0
   -0.9561    4.9712    1.5831 C   0  0  0  0  0  0  0  0  0  0  0  0
   -1.5648    4.1231    2.5116 C   0  0  0  0  0  0  0  0  0  0  0  0
   -0.4628    6.2198    1.9637 C   0  0  0  0  0  0  0  0  0  0  0  0
   -1.7003    4.5461    3.8402 C   0  0  0  0  0  0  0  0  0  0  0  0
   -1.9247    3.1201    2.2346 H   0  0  0  0  0  0  0  0  0  0  0  0
   -0.6008    6.6359    3.2933 C   0  0  0  0  0  0  0  0  0  0  0  0
    0.0365    6.8800    1.2516 H   0  0  0  0  0  0  0  0  0  0  0  0
   -1.2222    5.8014    4.2285 C   0  0  0  0  0  0  0  0  0  0  0  0
   -2.1721    3.8902    4.5709 H   0  0  0  0  0  0  0  0  0  0  0  0
   -0.2194    7.6082    3.5999 H   0  0  0  0  0  0  0  0  0  0  0  0
   -1.3286    6.1279    5.2624 H   0  0  0  0  0  0  0  0  0  0  0  0
```

```
  -2.1901    5.3186   -1.0542 C   0  0  0  0  0  0  0  0  0  0  0  0
  -3.1035    6.0905   -0.3389 C   0  0  0  0  0  0  0  0  0  0  0  0
  -2.2759    5.2204   -2.4463 C   0  0  0  0  0  0  0  0  0  0  0  0
  -4.1142    6.7782   -1.0251 C   0  0  0  0  0  0  0  0  0  0  0  0
  -3.0560    6.1766    0.7500 H   0  0  0  0  0  0  0  0  0  0  0  0
  -3.2829    5.9109   -3.1279 C   0  0  0  0  0  0  0  0  0  0  0  0
  -1.5695    4.6162   -3.0196 H   0  0  0  0  0  0  0  0  0  0  0  0
  -4.2026    6.6906   -2.4164 C   0  0  0  0  0  0  0  0  0  0  0  0
  -4.8316    7.3809   -0.4697 H   0  0  0  0  0  0  0  0  0  0  0  0
  -3.3546    5.8390   -4.2123 H   0  0  0  0  0  0  0  0  0  0  0  0
  -4.9884    7.2258   -2.9487 H   0  0  0  0  0  0  0  0  0  0  0  0
   0.7780   -0.9665    0.1376 C   0  0  0  0  0  0  0  0  0  0  0  0
   0.9008   -2.3017    0.5230 C   0  0  0  0  0  0  0  0  0  0  0  0
   1.8956   -0.2293   -0.2579 C   0  0  0  0  0  0  0  0  0  0  0  0
   2.1653   -2.9040    0.5184 C   0  0  0  0  0  0  0  0  0  0  0  0
   0.0371   -2.8949    0.8272 H   0  0  0  0  0  0  0  0  0  0  0  0
   3.1564   -0.8376   -0.2667 C   0  0  0  0  0  0  0  0  0  0  0  0
   1.8064    0.8283   -0.5375 H   0  0  0  0  0  0  0  0  0  0  0  0
   3.2905   -2.1740    0.1226 C   0  0  0  0  0  0  0  0  0  0  0  0
   2.2708   -3.9440    0.8221 H   0  0  0  0  0  0  0  0  0  0  0  0
   4.0318   -0.2677   -0.5722 H   0  0  0  0  0  0  0  0  0  0  0  0
   4.2716   -2.6466    0.1181 H   0  0  0  0  0  0  0  0  0  0  0  0
  -1.5907   -1.1714   -1.3615 C   0  0  0  0  0  0  0  0  0  0  0  0
  -2.2538   -2.3769   -1.1343 C   0  0  0  0  0  0  0  0  0  0  0  0
  -1.3545   -0.7321   -2.6647 C   0  0  0  0  0  0  0  0  0  0  0  0
  -2.7003   -3.1404   -2.2206 C   0  0  0  0  0  0  0  0  0  0  0  0
  -2.4405   -2.7482   -0.1237 H   0  0  0  0  0  0  0  0  0  0  0  0
  -1.7965   -1.4971   -3.7507 C   0  0  0  0  0  0  0  0  0  0  0  0
  -0.8260    0.2045   -2.8614 H   0  0  0  0  0  0  0  0  0  0  0  0
  -2.4721   -2.7013   -3.5279 C   0  0  0  0  0  0  0  0  0  0  0  0
  -3.2239   -4.0787   -2.0449 H   0  0  0  0  0  0  0  0  0  0  0  0
  -1.6143   -1.1549   -4.7678 H   0  0  0  0  0  0  0  0  0  0  0  0
  -2.8187   -3.2961   -4.3719 H   0  0  0  0  0  0  0  0  0  0  0  0
  -1.9503   -0.7502    1.4970 C   0  0  0  0  0  0  0  0  0  0  0  0
  -1.3580   -1.2065    2.6748 C   0  0  0  0  0  0  0  0  0  0  0  0
  -3.3420   -0.7172    1.3619 C   0  0  0  0  0  0  0  0  0  0  0  0
  -2.1693   -1.6808    3.7146 C   0  0  0  0  0  0  0  0  0  0  0  0
  -0.2708   -1.1666    2.8165 H   0  0  0  0  0  0  0  0  0  0  0  0
  -4.1463   -1.1922    2.4030 C   0  0  0  0  0  0  0  0  0  0  0  0
  -3.8222   -0.3286    0.4613 H   0  0  0  0  0  0  0  0  0  0  0  0
  -3.5594   -1.6813    3.5759 C   0  0  0  0  0  0  0  0  0  0  0  0
  -1.7120   -2.0406    4.6344 H   0  0  0  0  0  0  0  0  0  0  0  0
  -5.2298   -1.1774    2.3021 H   0  0  0  0  0  0  0  0  0  0  0  0
  -4.1881   -2.0540    4.3832 H   0  0  0  0  0  0  0  0  0  0  0  0
   1.9577    2.2138    2.8993 C   0  0  0  0  0  0  0  0  0  0  0  0
   1.6852    1.6819    3.8487 H   0  0  0  0  0  0  0  0  0  0  0  0
   2.0224    3.7193    3.1631 C   0  0  0  0  0  0  0  0  0  0  0  0
   2.1519    4.2686    2.2008 H   0  0  0  0  0  0  0  0  0  0  0  0
   1.0709    4.0617    3.6311 H   0  0  0  0  0  0  0  0  0  0  0  0
   2.8706    3.9547    3.8391 H   0  0  0  0  0  0  0  0  0  0  0  0
   3.2839    1.6497    2.3736 C   0  0  0  0  0  0  0  0  0  0  0  0
   4.0832    1.7989    3.1308 H   0  0  0  0  0  0  0  0  0  0  0  0
   3.1726    0.5583    2.1693 H   0  0  0  0  0  0  0  0  0  0  0  0
   3.5722    2.1671    1.4320 H   0  0  0  0  0  0  0  0  0  0  0  0
  1  6  1  0  0  0  0
  1  8  1  0  0  0  0
  1  9  1  0  0  0  0
  1  4  1  0  0  0  0
  1  3  1  0  0  0  0
  1  2  1  0  0  0  0
  3  5  1  0  0  0  0
  4  5  2  0  0  0  0
  5 76  1  0  0  0  0
  6  7  3  0  0  0  0
  8 10  1  0  0  0  0
```

```
  8 21  1  0  0  0  0
  8 32  1  0  0  0  0
  9 43  1  0  0  0  0
  9 54  1  0  0  0  0
  9 65  1  0  0  0  0
 10 11  2  0  0  0  0
 10 12  1  0  0  0  0
 11 13  1  0  0  0  0
 11 14  1  0  0  0  0
 12 15  2  0  0  0  0
 12 16  1  0  0  0  0
 13 17  2  0  0  0  0
 13 18  1  0  0  0  0
 15 17  1  0  0  0  0
 15 19  1  0  0  0  0
 17 20  1  0  0  0  0
 21 22  2  0  0  0  0
 21 23  1  0  0  0  0
 22 24  1  0  0  0  0
 22 25  1  0  0  0  0
 23 26  2  0  0  0  0
 23 27  1  0  0  0  0
 24 28  2  0  0  0  0
 24 29  1  0  0  0  0
 26 28  1  0  0  0  0
 26 30  1  0  0  0  0
 28 31  1  0  0  0  0
 32 33  2  0  0  0  0
 32 34  1  0  0  0  0
 33 35  1  0  0  0  0
 33 36  1  0  0  0  0
 34 37  2  0  0  0  0
 34 38  1  0  0  0  0
 35 39  2  0  0  0  0
 35 40  1  0  0  0  0
 37 39  1  0  0  0  0
 37 41  1  0  0  0  0
 39 42  1  0  0  0  0
 43 44  2  0  0  0  0
 43 45  1  0  0  0  0
 44 46  1  0  0  0  0
 44 47  1  0  0  0  0
 45 48  2  0  0  0  0
 45 49  1  0  0  0  0
 46 50  2  0  0  0  0
 46 51  1  0  0  0  0
 48 50  1  0  0  0  0
 48 52  1  0  0  0  0
 50 53  1  0  0  0  0
 54 55  2  0  0  0  0
 54 56  1  0  0  0  0
 55 57  1  0  0  0  0
 55 58  1  0  0  0  0
 56 59  2  0  0  0  0
 56 60  1  0  0  0  0
 57 61  2  0  0  0  0
 57 62  1  0  0  0  0
 59 61  1  0  0  0  0
 59 63  1  0  0  0  0
 61 64  1  0  0  0  0
 65 66  2  0  0  0  0
 65 67  1  0  0  0  0
 66 68  1  0  0  0  0
 66 69  1  0  0  0  0
 67 70  2  0  0  0  0
```

```
 67 71  1  0  0  0  0
 68 72  2  0  0  0  0
 68 73  1  0  0  0  0
 70 72  1  0  0  0  0
 70 74  1  0  0  0  0
 72 75  1  0  0  0  0
 76 77  1  0  0  0  0
 76 78  1  0  0  0  0
 76 82  1  0  0  0  0
 78 79  1  0  0  0  0
 78 80  1  0  0  0  0
 78 81  1  0  0  0  0
 82 83  1  0  0  0  0
 82 84  1  0  0  0  0
 82 85  1  0  0  0  0
M  END
$$$$
mol019
Optimized Catalyst 019
Optimized Catalyst 019
 79 85  0  0  0  0  0  0  0  0999 V2000
   -1.4722    2.0173   -1.5390 H   0  0  0  0  0  0  0  0  0  0  0  0
    1.0917    2.0473   -0.1802 O   0  0  0  0  0  0  0  0  0  0  0  0
    0.3735    2.0478    1.8714 O   0  0  0  0  0  0  0  0  0  0  0  0
    1.3571    2.0609    1.0762 C   0  0  0  0  0  0  0  0  0  0  0  0
    2.7969    2.0545    1.5122 C   0  0  0  0  0  0  0  0  0  0  0  0
    3.0556    1.0464    1.9253 H   0  0  0  0  0  0  0  0  0  0  0  0
    3.4559    2.3169    0.6452 H   0  0  0  0  0  0  0  0  0  0  0  0
    3.0444    3.2682    2.7824 Cl  0  0  0  0  0  0  0  0  0  0  0  0
   -2.9044    2.0263    0.6136 C   0  0  0  0  0  0  0  0  0  0  0  0
   -4.0676    2.0218    0.7906 O   0  0  0  0  0  0  0  0  0  0  0  0
   -0.9853    4.3931    0.0065 P   0  0  0  0  0  0  0  0  0  0  0  0
   -0.9597   -0.3315    0.0389 P   0  0  0  0  0  0  0  0  0  0  0  0
    0.7288    5.0834   -0.0850 C   0  0  0  0  0  0  0  0  0  0  0  0
    1.2035    6.0216    0.8283 C   0  0  0  0  0  0  0  0  0  0  0  0
    1.5518    4.5552   -1.0821 C   0  0  0  0  0  0  0  0  0  0  0  0
    2.5323    6.4540    0.7318 C   0  0  0  0  0  0  0  0  0  0  0  0
    0.5706    6.4201    1.6251 H   0  0  0  0  0  0  0  0  0  0  0  0
    2.8785    4.9916   -1.1730 C   0  0  0  0  0  0  0  0  0  0  0  0
    1.1850    3.7804   -1.7699 H   0  0  0  0  0  0  0  0  0  0  0  0
    3.3655    5.9397   -0.2668 C   0  0  0  0  0  0  0  0  0  0  0  0
    2.9186    7.1823    1.4420 H   0  0  0  0  0  0  0  0  0  0  0  0
    3.5307    4.5883   -1.9447 H   0  0  0  0  0  0  0  0  0  0  0  0
    4.3997    6.2753   -0.3356 H   0  0  0  0  0  0  0  0  0  0  0  0
   -1.7321    5.1127    1.5373 C   0  0  0  0  0  0  0  0  0  0  0  0
   -2.3099    6.3784    1.6019 C   0  0  0  0  0  0  0  0  0  0  0  0
   -1.6938    4.2629    2.6430 C   0  0  0  0  0  0  0  0  0  0  0  0
   -2.8480    6.8128    2.8189 C   0  0  0  0  0  0  0  0  0  0  0  0
   -2.3600    7.0324    0.7304 H   0  0  0  0  0  0  0  0  0  0  0  0
   -2.2315    4.7062    3.8577 C   0  0  0  0  0  0  0  0  0  0  0  0
   -1.2482    3.2517    2.5660 H   0  0  0  0  0  0  0  0  0  0  0  0
   -2.8045    5.9788    3.9429 C   0  0  0  0  0  0  0  0  0  0  0  0
   -3.3051    7.7975    2.8908 H   0  0  0  0  0  0  0  0  0  0  0  0
   -2.2044    4.0580    4.7316 H   0  0  0  0  0  0  0  0  0  0  0  0
   -3.2242    6.3229    4.8877 H   0  0  0  0  0  0  0  0  0  0  0  0
   -1.8775    5.1426   -1.4255 C   0  0  0  0  0  0  0  0  0  0  0  0
   -1.5549    6.4065   -1.9196 C   0  0  0  0  0  0  0  0  0  0  0  0
   -2.8822    4.3649   -1.9973 C   0  0  0  0  0  0  0  0  0  0  0  0
   -2.2790    6.9127   -3.0042 C   0  0  0  0  0  0  0  0  0  0  0  0
   -0.7483    7.0032   -1.4897 H   0  0  0  0  0  0  0  0  0  0  0  0
   -3.6032    4.8795   -3.0838 C   0  0  0  0  0  0  0  0  0  0  0  0
   -3.0994    3.3542   -1.6146 H   0  0  0  0  0  0  0  0  0  0  0  0
   -3.3031    6.1505   -3.5810 C   0  0  0  0  0  0  0  0  0  0  0  0
   -2.0448    7.8975   -3.4029 H   0  0  0  0  0  0  0  0  0  0  0  0
   -4.3941    4.2867   -3.5394 H   0  0  0  0  0  0  0  0  0  0  0  0
```

```
   -3.8652    6.5508   -4.4242 H   0  0  0  0  0  0  0  0  0  0  0  0
    0.7690   -0.9931   -0.0326 C   0  0  0  0  0  0  0  0  0  0  0  0
    1.2579   -1.9068    0.8976 C   0  0  0  0  0  0  0  0  0  0  0  0
    1.5852   -0.4656   -1.0353 C   0  0  0  0  0  0  0  0  0  0  0  0
    2.5960   -2.3125    0.8153 C   0  0  0  0  0  0  0  0  0  0  0  0
    0.6270   -2.3097    1.6939 H   0  0  0  0  0  0  0  0  0  0  0  0
    2.9212   -0.8770   -1.1133 C   0  0  0  0  0  0  0  0  0  0  0  0
    1.2058    0.2901   -1.7383 H   0  0  0  0  0  0  0  0  0  0  0  0
    3.4229   -1.7999   -0.1897 C   0  0  0  0  0  0  0  0  0  0  0  0
    2.9918   -3.0269    1.5346 H   0  0  0  0  0  0  0  0  0  0  0  0
    3.5679   -0.4749   -1.8908 H   0  0  0  0  0  0  0  0  0  0  0  0
    4.4625   -2.1202   -0.2524 H   0  0  0  0  0  0  0  0  0  0  0  0
   -1.8312   -1.1183   -1.3844 C   0  0  0  0  0  0  0  0  0  0  0  0
   -1.4877   -2.3841   -1.8591 C   0  0  0  0  0  0  0  0  0  0  0  0
   -2.8437   -0.3627   -1.9721 C   0  0  0  0  0  0  0  0  0  0  0  0
   -2.1979   -2.9149   -2.9411 C   0  0  0  0  0  0  0  0  0  0  0  0
   -0.6756   -2.9634   -1.4164 H   0  0  0  0  0  0  0  0  0  0  0  0
   -3.5506   -0.9021   -3.0558 C   0  0  0  0  0  0  0  0  0  0  0  0
   -3.0776    0.6499   -1.6037 H   0  0  0  0  0  0  0  0  0  0  0  0
   -3.2291   -2.1750   -3.5342 C   0  0  0  0  0  0  0  0  0  0  0  0
   -1.9474   -3.9015   -3.3253 H   0  0  0  0  0  0  0  0  0  0  0  0
   -4.3472   -0.3268   -3.5240 H   0  0  0  0  0  0  0  0  0  0  0  0
   -3.7799   -2.5945   -4.3756 H   0  0  0  0  0  0  0  0  0  0  0  0
   -1.6970   -1.0380    1.5802 C   0  0  0  0  0  0  0  0  0  0  0  0
   -1.7003   -0.1564    2.6614 C   0  0  0  0  0  0  0  0  0  0  0  0
   -2.2317   -2.3207    1.6748 C   0  0  0  0  0  0  0  0  0  0  0  0
   -2.2364   -0.5832    3.8826 C   0  0  0  0  0  0  0  0  0  0  0  0
   -1.2896    0.8681    2.5591 H   0  0  0  0  0  0  0  0  0  0  0  0
   -2.7677   -2.7389    2.8984 C   0  0  0  0  0  0  0  0  0  0  0  0
   -2.2505   -3.0000    0.8217 H   0  0  0  0  0  0  0  0  0  0  0  0
   -2.7661   -1.8722    3.9983 C   0  0  0  0  0  0  0  0  0  0  0  0
   -2.2434    0.0907    4.7374 H   0  0  0  0  0  0  0  0  0  0  0  0
   -3.1918   -3.7364    2.9938 H   0  0  0  0  0  0  0  0  0  0  0  0
   -3.1851   -2.2035    4.9481 H   0  0  0  0  0  0  0  0  0  0  0  0
   -1.1423    2.0290    0.0283 Os  0  0  0  0  0  0  0  0  0  0  0  0
  1 79  1  0  0  0  0
  2  4  2  0  0  0  0
  2 79  1  0  0  0  0
  3  4  2  0  0  0  0
  3 79  1  0  0  0  0
  4  5  1  0  0  0  0
  5  6  1  0  0  0  0
  5  7  1  0  0  0  0
  5  8  1  0  0  0  0
  9 10  2  0  0  0  0
  9 79  1  0  0  0  0
 11 13  1  0  0  0  0
 11 24  1  0  0  0  0
 11 35  1  0  0  0  0
 11 79  1  0  0  0  0
 12 46  1  0  0  0  0
 12 57  1  0  0  0  0
 12 68  1  0  0  0  0
 12 79  1  0  0  0  0
 13 14  2  0  0  0  0
 13 15  1  0  0  0  0
 14 16  1  0  0  0  0
 14 17  1  0  0  0  0
 15 18  2  0  0  0  0
 15 19  1  0  0  0  0
 16 20  2  0  0  0  0
 16 21  1  0  0  0  0
 18 20  1  0  0  0  0
 18 22  1  0  0  0  0
 20 23  1  0  0  0  0
```

```
 24 25  2  0  0  0  0
 24 26  1  0  0  0  0
 25 27  1  0  0  0  0
 25 28  1  0  0  0  0
 26 29  2  0  0  0  0
 26 30  1  0  0  0  0
 27 31  2  0  0  0  0
 27 32  1  0  0  0  0
 29 31  1  0  0  0  0
 29 33  1  0  0  0  0
 31 34  1  0  0  0  0
 35 36  2  0  0  0  0
 35 37  1  0  0  0  0
 36 38  1  0  0  0  0
 36 39  1  0  0  0  0
 37 40  2  0  0  0  0
 37 41  1  0  0  0  0
 38 42  2  0  0  0  0
 38 43  1  0  0  0  0
 40 42  1  0  0  0  0
 40 44  1  0  0  0  0
 42 45  1  0  0  0  0
 46 47  2  0  0  0  0
 46 48  1  0  0  0  0
 47 49  1  0  0  0  0
 47 50  1  0  0  0  0
 48 51  2  0  0  0  0
 48 52  1  0  0  0  0
 49 53  2  0  0  0  0
 49 54  1  0  0  0  0
 51 53  1  0  0  0  0
 51 55  1  0  0  0  0
 53 56  1  0  0  0  0
 57 58  2  0  0  0  0
 57 59  1  0  0  0  0
 58 60  1  0  0  0  0
 58 61  1  0  0  0  0
 59 62  2  0  0  0  0
 59 63  1  0  0  0  0
 60 64  2  0  0  0  0
 60 65  1  0  0  0  0
 62 64  1  0  0  0  0
 62 66  1  0  0  0  0
 64 67  1  0  0  0  0
 68 69  2  0  0  0  0
 68 70  1  0  0  0  0
 69 71  1  0  0  0  0
 69 72  1  0  0  0  0
 70 73  2  0  0  0  0
 70 74  1  0  0  0  0
 71 75  2  0  0  0  0
 71 76  1  0  0  0  0
 73 75  1  0  0  0  0
 73 77  1  0  0  0  0
 75 78  1  0  0  0  0
M  END
$$$$
mol020
Optimized Catalyst 020
Optimized Catalyst 020
 86 93  0  0  0  0  0  0  0  0999 V2000
   -0.9241    2.0968   -1.6598 H   0  0  0  0  0  0  0  0  0  0  0  0
    1.0655    2.1996    0.6423 O   0  0  0  0  0  0  0  0  0  0  0  0
   -0.4341    2.1619    2.1991 O   0  0  0  0  0  0  0  0  0  0  0  0
    0.8017    2.2017    1.9012 C   0  0  0  0  0  0  0  0  0  0  0  0
```

-2.9301	2.0758	-0.0926	C	0	0	0	0	0	0	0	0	0	0	0	0
-4.0925	2.0143	-0.2639	O	0	0	0	0	0	0	0	0	0	0	0	0
-0.8938	4.4737	-0.1398	P	0	0	0	0	0	0	0	0	0	0	0	0
-0.7905	-0.2292	-0.0200	P	0	0	0	0	0	0	0	0	0	0	0	0
0.4469	4.9411	-1.3218	C	0	0	0	0	0	0	0	0	0	0	0	0
0.5969	6.2378	-1.8150	C	0	0	0	0	0	0	0	0	0	0	0	0
1.2768	3.8995	-1.7317	C	0	0	0	0	0	0	0	0	0	0	0	0
1.6345	6.5040	-2.7138	C	0	0	0	0	0	0	0	0	0	0	0	0
-0.0810	7.0433	-1.5288	H	0	0	0	0	0	0	0	0	0	0	0	0
2.3159	4.1756	-2.6325	C	0	0	0	0	0	0	0	0	0	0	0	0
1.1194	2.8642	-1.3642	H	0	0	0	0	0	0	0	0	0	0	0	0
2.4954	5.4737	-3.1155	C	0	0	0	0	0	0	0	0	0	0	0	0
1.7710	7.5091	-3.1063	H	0	0	0	0	0	0	0	0	0	0	0	0
2.9783	3.3749	-2.9545	H	0	0	0	0	0	0	0	0	0	0	0	0
3.3042	5.6877	-3.8131	H	0	0	0	0	0	0	0	0	0	0	0	0
-0.5372	5.3208	1.4689	C	0	0	0	0	0	0	0	0	0	0	0	0
-1.2273	4.7948	2.5635	C	0	0	0	0	0	0	0	0	0	0	0	0
0.3837	6.3533	1.6195	C	0	0	0	0	0	0	0	0	0	0	0	0
-1.0194	5.3483	3.8324	C	0	0	0	0	0	0	0	0	0	0	0	0
-1.8978	3.9281	2.4492	H	0	0	0	0	0	0	0	0	0	0	0	0
0.5951	6.8966	2.8934	C	0	0	0	0	0	0	0	0	0	0	0	0
0.9556	6.7429	0.7754	H	0	0	0	0	0	0	0	0	0	0	0	0
-0.1105	6.3992	3.9939	C	0	0	0	0	0	0	0	0	0	0	0	0
-1.5601	4.9553	4.6911	H	0	0	0	0	0	0	0	0	0	0	0	0
1.3137	7.7026	3.0268	H	0	0	0	0	0	0	0	0	0	0	0	0
0.0539	6.8277	4.9819	H	0	0	0	0	0	0	0	0	0	0	0	0
-2.4043	5.2998	-0.8138	C	0	0	0	0	0	0	0	0	0	0	0	0
-2.9175	6.4787	-0.2778	C	0	0	0	0	0	0	0	0	0	0	0	0
-3.0058	4.6642	-1.9005	C	0	0	0	0	0	0	0	0	0	0	0	0
-4.0635	7.0407	-0.8539	C	0	0	0	0	0	0	0	0	0	0	0	0
-2.4549	6.9702	0.5803	H	0	0	0	0	0	0	0	0	0	0	0	0
-4.1512	5.2324	-2.4706	C	0	0	0	0	0	0	0	0	0	0	0	0
-2.5998	3.7226	-2.2979	H	0	0	0	0	0	0	0	0	0	0	0	0
-4.6760	6.4191	-1.9477	C	0	0	0	0	0	0	0	0	0	0	0	0
-4.4803	7.9602	-0.4474	H	0	0	0	0	0	0	0	0	0	0	0	0
-4.6364	4.7463	-3.3151	H	0	0	0	0	0	0	0	0	0	0	0	0
-5.5684	6.8593	-2.3914	H	0	0	0	0	0	0	0	0	0	0	0	0
0.9337	-0.7973	0.3504	C	0	0	0	0	0	0	0	0	0	0	0	0
1.2329	-1.6199	1.4329	C	0	0	0	0	0	0	0	0	0	0	0	0
1.9366	-0.2935	-0.4813	C	0	0	0	0	0	0	0	0	0	0	0	0
2.5683	-1.9688	1.6749	C	0	0	0	0	0	0	0	0	0	0	0	0
0.4548	-1.9940	2.1030	H	0	0	0	0	0	0	0	0	0	0	0	0
3.2678	-0.6509	-0.2380	C	0	0	0	0	0	0	0	0	0	0	0	0
1.7002	0.3943	-1.3016	H	0	0	0	0	0	0	0	0	0	0	0	0
3.5804	-1.4899	0.8376	C	0	0	0	0	0	0	0	0	0	0	0	0
2.8167	-2.6092	2.5189	H	0	0	0	0	0	0	0	0	0	0	0	0
4.0587	-0.2702	-0.8811	H	0	0	0	0	0	0	0	0	0	0	0	0
4.6170	-1.7662	1.0264	H	0	0	0	0	0	0	0	0	0	0	0	0
-1.1898	-1.0140	-1.6456	C	0	0	0	0	0	0	0	0	0	0	0	0
-0.6613	-2.2510	-2.0155	C	0	0	0	0	0	0	0	0	0	0	0	0
-2.0325	-0.2953	-2.4905	C	0	0	0	0	0	0	0	0	0	0	0	0
-1.0094	-2.7914	-3.2580	C	0	0	0	0	0	0	0	0	0	0	0	0
0.0241	-2.8007	-1.3681	H	0	0	0	0	0	0	0	0	0	0	0	0
-2.3770	-0.8437	-3.7339	C	0	0	0	0	0	0	0	0	0	0	0	0
-2.4119	0.6973	-2.1990	H	0	0	0	0	0	0	0	0	0	0	0	0
-1.8693	-2.0892	-4.1122	C	0	0	0	0	0	0	0	0	0	0	0	0
-0.6100	-3.7563	-3.5630	H	0	0	0	0	0	0	0	0	0	0	0	0
-3.0381	-0.2962	-4.4032	H	0	0	0	0	0	0	0	0	0	0	0	0
-2.1395	-2.5160	-5.0775	H	0	0	0	0	0	0	0	0	0	0	0	0
-1.8764	-1.0459	1.2355	C	0	0	0	0	0	0	0	0	0	0	0	0
-2.3647	-0.1863	2.2188	C	0	0	0	0	0	0	0	0	0	0	0	0
-2.2092	-2.3990	1.2118	C	0	0	0	0	0	0	0	0	0	0	0	0
-3.1953	-0.7040	3.2215	C	0	0	0	0	0	0	0	0	0	0	0	0
-2.1027	0.8920	2.2148	H	0	0	0	0	0	0	0	0	0	0	0	0
-3.0409	-2.9081	2.2152	C	0	0	0	0	0	0	0	0	0	0	0	0

```
   -1.8450   -3.0669    0.4308 H   0  0  0  0  0  0  0  0  0  0  0  0
   -3.5293   -2.0610    3.2184 C   0  0  0  0  0  0  0  0  0  0  0  0
   -3.5806   -0.0466    3.9985 H   0  0  0  0  0  0  0  0  0  0  0  0
   -3.3133   -3.9613    2.2141 H   0  0  0  0  0  0  0  0  0  0  0  0
   -4.1763   -2.4632    3.9973 H   0  0  0  0  0  0  0  0  0  0  0  0
    1.8797    2.2449    2.9150 C   0  0  0  0  0  0  0  0  0  0  0  0
    1.6294    2.8121    4.1726 C   0  0  0  0  0  0  0  0  0  0  0  0
    3.1414    1.7161    2.6074 C   0  0  0  0  0  0  0  0  0  0  0  0
    2.6502    2.8491    5.1253 C   0  0  0  0  0  0  0  0  0  0  0  0
    0.6390    3.2202    4.3900 H   0  0  0  0  0  0  0  0  0  0  0  0
    4.1544    1.7501    3.5682 C   0  0  0  0  0  0  0  0  0  0  0  0
    3.3137    1.2807    1.6185 H   0  0  0  0  0  0  0  0  0  0  0  0
    3.9090    2.3171    4.8239 C   0  0  0  0  0  0  0  0  0  0  0  0
    2.4643    3.2915    6.1026 H   0  0  0  0  0  0  0  0  0  0  0  0
    5.1346    1.3364    3.3373 H   0  0  0  0  0  0  0  0  0  0  0  0
    4.7023    2.3448    5.5706 H   0  0  0  0  0  0  0  0  0  0  0  0
   -1.0672    2.1186   -0.0619 Os  0  0  0  0  0  0  0  0  0  0  0  0
  1 86  1  0  0  0  0
  2  4  2  0  0  0  0
  2 86  1  0  0  0  0
  3  4  2  0  0  0  0
  3 86  1  0  0  0  0
  4 75  1  0  0  0  0
  5  6  2  0  0  0  0
  5 86  1  0  0  0  0
  7  9  1  0  0  0  0
  7 20  1  0  0  0  0
  7 31  1  0  0  0  0
  7 86  1  0  0  0  0
  8 42  1  0  0  0  0
  8 53  1  0  0  0  0
  8 64  1  0  0  0  0
  8 86  1  0  0  0  0
  9 10  2  0  0  0  0
  9 11  1  0  0  0  0
 10 12  1  0  0  0  0
 10 13  1  0  0  0  0
 11 14  2  0  0  0  0
 11 15  1  0  0  0  0
 12 16  2  0  0  0  0
 12 17  1  0  0  0  0
 14 16  1  0  0  0  0
 14 18  1  0  0  0  0
 16 19  1  0  0  0  0
 20 21  2  0  0  0  0
 20 22  1  0  0  0  0
 21 23  1  0  0  0  0
 21 24  1  0  0  0  0
 22 25  2  0  0  0  0
 22 26  1  0  0  0  0
 23 27  2  0  0  0  0
 23 28  1  0  0  0  0
 25 27  1  0  0  0  0
 25 29  1  0  0  0  0
 27 30  1  0  0  0  0
 31 32  2  0  0  0  0
 31 33  1  0  0  0  0
 32 34  1  0  0  0  0
 32 35  1  0  0  0  0
 33 36  2  0  0  0  0
 33 37  1  0  0  0  0
 34 38  2  0  0  0  0
 34 39  1  0  0  0  0
 36 38  1  0  0  0  0
 36 40  1  0  0  0  0
```

```
 38 41  1  0  0  0  0
 42 43  2  0  0  0  0
 42 44  1  0  0  0  0
 43 45  1  0  0  0  0
 43 46  1  0  0  0  0
 44 47  2  0  0  0  0
 44 48  1  0  0  0  0
 45 49  2  0  0  0  0
 45 50  1  0  0  0  0
 47 49  1  0  0  0  0
 47 51  1  0  0  0  0
 49 52  1  0  0  0  0
 53 54  2  0  0  0  0
 53 55  1  0  0  0  0
 54 56  1  0  0  0  0
 54 57  1  0  0  0  0
 55 58  2  0  0  0  0
 55 59  1  0  0  0  0
 56 60  2  0  0  0  0
 56 61  1  0  0  0  0
 58 60  1  0  0  0  0
 58 62  1  0  0  0  0
 60 63  1  0  0  0  0
 64 65  2  0  0  0  0
 64 66  1  0  0  0  0
 65 67  1  0  0  0  0
 65 68  1  0  0  0  0
 66 69  2  0  0  0  0
 66 70  1  0  0  0  0
 67 71  2  0  0  0  0
 67 72  1  0  0  0  0
 69 71  1  0  0  0  0
 69 73  1  0  0  0  0
 71 74  1  0  0  0  0
 75 76  2  0  0  0  0
 75 77  1  0  0  0  0
 76 78  1  0  0  0  0
 76 79  1  0  0  0  0
 77 80  2  0  0  0  0
 77 81  1  0  0  0  0
 78 82  2  0  0  0  0
 78 83  1  0  0  0  0
 80 82  1  0  0  0  0
 80 84  1  0  0  0  0
 82 85  1  0  0  0  0
M  END
$$$$
mol021
Optimized Catalyst 021
Optimized Catalyst 021
 79 85  0  0  0  0  0  0  0  0999 V2000
   -1.4227    2.0164   -1.5179 H   0  0  0  0  0  0  0  0  0  0  0  0
    1.1037    2.0463   -0.0873 O   0  0  0  0  0  0  0  0  0  0  0  0
    0.3203    2.0570    1.9316 O   0  0  0  0  0  0  0  0  0  0  0  0
    1.3433    2.0582    1.1763 C   0  0  0  0  0  0  0  0  0  0  0  0
    2.7411    2.0720    1.6889 C   0  0  0  0  0  0  0  0  0  0  0  0
    3.2745    1.1605    1.3193 H   0  0  0  0  0  0  0  0  0  0  0  0
    3.2634    2.9821    1.3003 H   0  0  0  0  0  0  0  0  0  0  0  0
   -2.9018    2.0231    0.6151 C   0  0  0  0  0  0  0  0  0  0  0  0
   -4.0667    2.0160    0.7839 O   0  0  0  0  0  0  0  0  0  0  0  0
   -0.9809    4.3934    0.0151 P   0  0  0  0  0  0  0  0  0  0  0  0
   -0.9471   -0.3286    0.0538 P   0  0  0  0  0  0  0  0  0  0  0  0
    0.7317    5.0889   -0.1016 C   0  0  0  0  0  0  0  0  0  0  0  0
    1.1982    6.0825    0.7555 C   0  0  0  0  0  0  0  0  0  0  0  0
    1.5568    4.5143   -1.0705 C   0  0  0  0  0  0  0  0  0  0  0  0
```

```
    2.5212    6.5244    0.6292 C   0  0  0  0  0  0  0  0  0  0  0  0
    0.5616    6.5202    1.5280 H   0  0  0  0  0  0  0  0  0  0  0  0
    2.8781    4.9616   -1.1920 C   0  0  0  0  0  0  0  0  0  0  0  0
    1.1965    3.6958   -1.7114 H   0  0  0  0  0  0  0  0  0  0  0  0
    3.3563    5.9661   -0.3444 C   0  0  0  0  0  0  0  0  0  0  0  0
    2.8995    7.3010    1.2907 H   0  0  0  0  0  0  0  0  0  0  0  0
    3.5320    4.5222   -1.9427 H   0  0  0  0  0  0  0  0  0  0  0  0
    4.3841    6.3141   -0.4412 H   0  0  0  0  0  0  0  0  0  0  0  0
   -1.7224    5.1422    1.5359 C   0  0  0  0  0  0  0  0  0  0  0  0
   -2.2567    6.4274    1.5934 C   0  0  0  0  0  0  0  0  0  0  0  0
   -1.7310    4.2913    2.6410 C   0  0  0  0  0  0  0  0  0  0  0  0
   -2.7968    6.8801    2.8027 C   0  0  0  0  0  0  0  0  0  0  0  0
   -2.2728    7.0827    0.7218 H   0  0  0  0  0  0  0  0  0  0  0  0
   -2.2714    4.7524    3.8481 C   0  0  0  0  0  0  0  0  0  0  0  0
   -1.3208    3.2640    2.5688 H   0  0  0  0  0  0  0  0  0  0  0  0
   -2.8002    6.0443    3.9264 C   0  0  0  0  0  0  0  0  0  0  0  0
   -3.2204    7.8801    2.8686 H   0  0  0  0  0  0  0  0  0  0  0  0
   -2.2826    4.1023    4.7208 H   0  0  0  0  0  0  0  0  0  0  0  0
   -3.2225    6.4022    4.8648 H   0  0  0  0  0  0  0  0  0  0  0  0
   -1.8765    5.1275   -1.4246 C   0  0  0  0  0  0  0  0  0  0  0  0
   -1.5618    6.3903   -1.9270 C   0  0  0  0  0  0  0  0  0  0  0  0
   -2.8741    4.3393   -1.9941 C   0  0  0  0  0  0  0  0  0  0  0  0
   -2.2876    6.8846   -3.0160 C   0  0  0  0  0  0  0  0  0  0  0  0
   -0.7592    6.9949   -1.5014 H   0  0  0  0  0  0  0  0  0  0  0  0
   -3.5972    4.8419   -3.0849 C   0  0  0  0  0  0  0  0  0  0  0  0
   -3.0834    3.3286   -1.6070 H   0  0  0  0  0  0  0  0  0  0  0  0
   -3.3056    6.1119   -3.5895 C   0  0  0  0  0  0  0  0  0  0  0  0
   -2.0592    7.8682   -3.4208 H   0  0  0  0  0  0  0  0  0  0  0  0
   -4.3828    4.2403   -3.5380 H   0  0  0  0  0  0  0  0  0  0  0  0
   -3.8693    6.5030   -4.4359 H   0  0  0  0  0  0  0  0  0  0  0  0
    0.7753   -1.0024   -0.0456 C   0  0  0  0  0  0  0  0  0  0  0  0
    1.2522   -1.9745    0.8300 C   0  0  0  0  0  0  0  0  0  0  0  0
    1.5962   -0.4327   -1.0209 C   0  0  0  0  0  0  0  0  0  0  0  0
    2.5820   -2.3992    0.7170 C   0  0  0  0  0  0  0  0  0  0  0  0
    0.6183   -2.4085    1.6070 H   0  0  0  0  0  0  0  0  0  0  0  0
    2.9245   -0.8624   -1.1291 C   0  0  0  0  0  0  0  0  0  0  0  0
    1.2268    0.3691   -1.6776 H   0  0  0  0  0  0  0  0  0  0  0  0
    3.4134   -1.8452   -0.2623 C   0  0  0  0  0  0  0  0  0  0  0  0
    2.9686   -3.1589    1.3931 H   0  0  0  0  0  0  0  0  0  0  0  0
    3.5753   -0.4261   -1.8842 H   0  0  0  0  0  0  0  0  0  0  0  0
    4.4466   -2.1796   -0.3486 H   0  0  0  0  0  0  0  0  0  0  0  0
   -1.8278   -1.0990   -1.3760 C   0  0  0  0  0  0  0  0  0  0  0  0
   -1.4915   -2.3638   -1.8589 C   0  0  0  0  0  0  0  0  0  0  0  0
   -2.8367   -0.3356   -1.9593 C   0  0  0  0  0  0  0  0  0  0  0  0
   -2.2061   -2.8857   -2.9424 C   0  0  0  0  0  0  0  0  0  0  0  0
   -0.6806   -2.9492   -1.4221 H   0  0  0  0  0  0  0  0  0  0  0  0
   -3.5486   -0.8659   -3.0443 C   0  0  0  0  0  0  0  0  0  0  0  0
   -3.0636    0.6770   -1.5875 H   0  0  0  0  0  0  0  0  0  0  0  0
   -3.2350   -2.1381   -3.5297 C   0  0  0  0  0  0  0  0  0  0  0  0
   -1.9607   -3.8713   -3.3321 H   0  0  0  0  0  0  0  0  0  0  0  0
   -4.3428   -0.2840   -3.5081 H   0  0  0  0  0  0  0  0  0  0  0  0
   -3.7898   -2.5506   -4.3717 H   0  0  0  0  0  0  0  0  0  0  0  0
   -1.6833   -1.0614    1.5849 C   0  0  0  0  0  0  0  0  0  0  0  0
   -1.7035   -0.1929    2.6761 C   0  0  0  0  0  0  0  0  0  0  0  0
   -2.2038   -2.3512    1.6621 C   0  0  0  0  0  0  0  0  0  0  0  0
   -2.2411   -0.6403    3.8894 C   0  0  0  0  0  0  0  0  0  0  0  0
   -1.3047    0.8377    2.5879 H   0  0  0  0  0  0  0  0  0  0  0  0
   -2.7414   -2.7902    2.8776 C   0  0  0  0  0  0  0  0  0  0  0  0
   -2.2110   -3.0206    0.8010 H   0  0  0  0  0  0  0  0  0  0  0  0
   -2.7559   -1.9365    3.9876 C   0  0  0  0  0  0  0  0  0  0  0  0
   -2.2612    0.0237    4.7515 H   0  0  0  0  0  0  0  0  0  0  0  0
   -3.1542   -3.7935    2.9588 H   0  0  0  0  0  0  0  0  0  0  0  0
   -3.1762   -2.2838    4.9309 H   0  0  0  0  0  0  0  0  0  0  0  0
   -1.1314    2.0314    0.0590 Os  0  0  0  0  0  0  0  0  0  0  0  0
    2.7422    2.0836    2.8044 H   0  0  0  0  0  0  0  0  0  0  0  0
```

```
  1 78  1  0  0  0  0
  2  4  2  0  0  0  0
  2 78  1  0  0  0  0
  3  4  2  0  0  0  0
  3 78  1  0  0  0  0
  4  5  1  0  0  0  0
  5  6  1  0  0  0  0
  5  7  1  0  0  0  0
  5 79  1  0  0  0  0
  8  9  2  0  0  0  0
  8 78  1  0  0  0  0
 10 12  1  0  0  0  0
 10 23  1  0  0  0  0
 10 34  1  0  0  0  0
 10 78  1  0  0  0  0
 11 45  1  0  0  0  0
 11 56  1  0  0  0  0
 11 67  1  0  0  0  0
 11 78  1  0  0  0  0
 12 13  2  0  0  0  0
 12 14  1  0  0  0  0
 13 15  1  0  0  0  0
 13 16  1  0  0  0  0
 14 17  2  0  0  0  0
 14 18  1  0  0  0  0
 15 19  2  0  0  0  0
 15 20  1  0  0  0  0
 17 19  1  0  0  0  0
 17 21  1  0  0  0  0
 19 22  1  0  0  0  0
 23 24  2  0  0  0  0
 23 25  1  0  0  0  0
 24 26  1  0  0  0  0
 24 27  1  0  0  0  0
 25 28  2  0  0  0  0
 25 29  1  0  0  0  0
 26 30  2  0  0  0  0
 26 31  1  0  0  0  0
 28 30  1  0  0  0  0
 28 32  1  0  0  0  0
 30 33  1  0  0  0  0
 34 35  2  0  0  0  0
 34 36  1  0  0  0  0
 35 37  1  0  0  0  0
 35 38  1  0  0  0  0
 36 39  2  0  0  0  0
 36 40  1  0  0  0  0
 37 41  2  0  0  0  0
 37 42  1  0  0  0  0
 39 41  1  0  0  0  0
 39 43  1  0  0  0  0
 41 44  1  0  0  0  0
 45 46  2  0  0  0  0
 45 47  1  0  0  0  0
 46 48  1  0  0  0  0
 46 49  1  0  0  0  0
 47 50  2  0  0  0  0
 47 51  1  0  0  0  0
 48 52  2  0  0  0  0
 48 53  1  0  0  0  0
 50 52  1  0  0  0  0
 50 54  1  0  0  0  0
 52 55  1  0  0  0  0
 56 57  2  0  0  0  0
 56 58  1  0  0  0  0
```

```
 57 59  1  0  0  0  0
 57 60  1  0  0  0  0
 58 61  2  0  0  0  0
 58 62  1  0  0  0  0
 59 63  2  0  0  0  0
 59 64  1  0  0  0  0
 61 63  1  0  0  0  0
 61 65  1  0  0  0  0
 63 66  1  0  0  0  0
 67 68  2  0  0  0  0
 67 69  1  0  0  0  0
 68 70  1  0  0  0  0
 68 71  1  0  0  0  0
 69 72  2  0  0  0  0
 69 73  1  0  0  0  0
 70 74  2  0  0  0  0
 70 75  1  0  0  0  0
 72 74  1  0  0  0  0
 72 76  1  0  0  0  0
 74 77  1  0  0  0  0
M  END
$$$$
mol022
Optimized Catalyst 022
Optimized Catalyst 022
 85 91  0  0  0  0  0  0  0  0999 V2000
   -0.8353    2.1199   -1.5761 H   0  0  0  0  0  0  0  0  0  0  0  0
    1.0127    2.2114    0.8370 O   0  0  0  0  0  0  0  0  0  0  0  0
   -0.5732    2.0659    2.2954 O   0  0  0  0  0  0  0  0  0  0  0  0
    0.6721    2.1961    2.0746 C   0  0  0  0  0  0  0  0  0  0  0  0
   -2.9379    2.0347   -0.1091 C   0  0  0  0  0  0  0  0  0  0  0  0
   -4.0907    1.9674   -0.3325 O   0  0  0  0  0  0  0  0  0  0  0  0
   -0.9475    4.4540   -0.0241 P   0  0  0  0  0  0  0  0  0  0  0  0
   -0.8053   -0.2529   -0.0105 P   0  0  0  0  0  0  0  0  0  0  0  0
    0.4314    4.9706   -1.1423 C   0  0  0  0  0  0  0  0  0  0  0  0
    0.5488    6.2683   -1.6415 C   0  0  0  0  0  0  0  0  0  0  0  0
    1.3259    3.9642   -1.5004 C   0  0  0  0  0  0  0  0  0  0  0  0
    1.6205    6.5744   -2.4861 C   0  0  0  0  0  0  0  0  0  0  0  0
   -0.1808    7.0439   -1.4032 H   0  0  0  0  0  0  0  0  0  0  0  0
    2.3988    4.2796   -2.3468 C   0  0  0  0  0  0  0  0  0  0  0  0
    1.1903    2.9254   -1.1367 H   0  0  0  0  0  0  0  0  0  0  0  0
    2.5470    5.5812   -2.8311 C   0  0  0  0  0  0  0  0  0  0  0  0
    1.7323    7.5815   -2.8815 H   0  0  0  0  0  0  0  0  0  0  0  0
    3.1106    3.5062   -2.6277 H   0  0  0  0  0  0  0  0  0  0  0  0
    3.3818    5.8262   -3.4867 H   0  0  0  0  0  0  0  0  0  0  0  0
   -0.6799    5.3147    1.5971 C   0  0  0  0  0  0  0  0  0  0  0  0
   -1.3366    4.7279    2.6802 C   0  0  0  0  0  0  0  0  0  0  0  0
    0.1434    6.4253    1.7625 C   0  0  0  0  0  0  0  0  0  0  0  0
   -1.1843    5.2855    3.9556 C   0  0  0  0  0  0  0  0  0  0  0  0
   -1.9395    3.8127    2.5526 H   0  0  0  0  0  0  0  0  0  0  0  0
    0.2930    6.9782    3.0406 C   0  0  0  0  0  0  0  0  0  0  0  0
    0.6851    6.8707    0.9266 H   0  0  0  0  0  0  0  0  0  0  0  0
   -0.3740    6.4113    4.1324 C   0  0  0  0  0  0  0  0  0  0  0  0
   -1.6907    4.8357    4.8076 H   0  0  0  0  0  0  0  0  0  0  0  0
    0.9309    7.8476    3.1848 H   0  0  0  0  0  0  0  0  0  0  0  0
   -0.2566    6.8458    5.1245 H   0  0  0  0  0  0  0  0  0  0  0  0
   -2.4469    5.2486   -0.7602 C   0  0  0  0  0  0  0  0  0  0  0  0
   -3.0332    6.3911   -0.2206 C   0  0  0  0  0  0  0  0  0  0  0  0
   -2.9628    4.6265   -1.8978 C   0  0  0  0  0  0  0  0  0  0  0  0
   -4.1648    6.9304   -0.8452 C   0  0  0  0  0  0  0  0  0  0  0  0
   -2.6389    6.8715    0.6770 H   0  0  0  0  0  0  0  0  0  0  0  0
   -4.0941    5.1718   -2.5163 C   0  0  0  0  0  0  0  0  0  0  0  0
   -2.5017    3.7120   -2.2978 H   0  0  0  0  0  0  0  0  0  0  0  0
   -4.6911    6.3224   -1.9903 C   0  0  0  0  0  0  0  0  0  0  0  0
   -4.6376    7.8212   -0.4364 H   0  0  0  0  0  0  0  0  0  0  0  0
```

```
   -4.5126    4.6954   -3.4011 H   0  0  0  0  0  0  0  0  0  0  0  0
   -5.5726    6.7446   -2.4715 H   0  0  0  0  0  0  0  0  0  0  0  0
    0.8688   -0.8955    0.4573 C   0  0  0  0  0  0  0  0  0  0  0  0
    1.0510   -1.9470    1.3522 C   0  0  0  0  0  0  0  0  0  0  0  0
    1.9534   -0.2408   -0.1286 C   0  0  0  0  0  0  0  0  0  0  0  0
    2.3525   -2.3722    1.6471 C   0  0  0  0  0  0  0  0  0  0  0  0
    0.2061   -2.4438    1.8345 H   0  0  0  0  0  0  0  0  0  0  0  0
    3.2520   -0.6739    0.1663 C   0  0  0  0  0  0  0  0  0  0  0  0
    1.8032    0.6231   -0.7905 H   0  0  0  0  0  0  0  0  0  0  0  0
    3.4484   -1.7410    1.0490 C   0  0  0  0  0  0  0  0  0  0  0  0
    2.5106   -3.1937    2.3427 H   0  0  0  0  0  0  0  0  0  0  0  0
    4.1064   -0.1753   -0.2873 H   0  0  0  0  0  0  0  0  0  0  0  0
    4.4590   -2.0784    1.2764 H   0  0  0  0  0  0  0  0  0  0  0  0
   -1.0943   -0.9597   -1.6963 C   0  0  0  0  0  0  0  0  0  0  0  0
   -0.5340   -2.1744   -2.0923 C   0  0  0  0  0  0  0  0  0  0  0  0
   -1.8889   -0.2055   -2.5569 C   0  0  0  0  0  0  0  0  0  0  0  0
   -0.7997   -2.6554   -3.3789 C   0  0  0  0  0  0  0  0  0  0  0  0
    0.1142   -2.7519   -1.4309 H   0  0  0  0  0  0  0  0  0  0  0  0
   -2.1511   -0.6944   -3.8444 C   0  0  0  0  0  0  0  0  0  0  0  0
   -2.2939    0.7699   -2.2425 H   0  0  0  0  0  0  0  0  0  0  0  0
   -1.6105   -1.9172   -4.2503 C   0  0  0  0  0  0  0  0  0  0  0  0
   -0.3735   -3.6020   -3.7045 H   0  0  0  0  0  0  0  0  0  0  0  0
   -2.7738   -0.1185   -4.5263 H   0  0  0  0  0  0  0  0  0  0  0  0
   -1.8163   -2.2976   -5.2502 H   0  0  0  0  0  0  0  0  0  0  0  0
   -2.0060   -1.1042    1.1122 C   0  0  0  0  0  0  0  0  0  0  0  0
   -2.5133   -0.3004    2.1321 C   0  0  0  0  0  0  0  0  0  0  0  0
   -2.4067   -2.4297    0.9541 C   0  0  0  0  0  0  0  0  0  0  0  0
   -3.4301   -0.8505    3.0376 C   0  0  0  0  0  0  0  0  0  0  0  0
   -2.2036    0.7601    2.2292 H   0  0  0  0  0  0  0  0  0  0  0  0
   -3.3243   -2.9713    1.8611 C   0  0  0  0  0  0  0  0  0  0  0  0
   -2.0325   -3.0501    0.1388 H   0  0  0  0  0  0  0  0  0  0  0  0
   -3.8306   -2.1827    2.9020 C   0  0  0  0  0  0  0  0  0  0  0  0
   -3.8320   -0.2368    3.8416 H   0  0  0  0  0  0  0  0  0  0  0  0
   -3.6501   -4.0038    1.7545 H   0  0  0  0  0  0  0  0  0  0  0  0
   -4.5448   -2.6097    3.6053 H   0  0  0  0  0  0  0  0  0  0  0  0
    1.6772    2.3446    3.2062 C   0  0  0  0  0  0  0  0  0  0  0  0
    1.0961    2.3778    4.1664 H   0  0  0  0  0  0  0  0  0  0  0  0
    2.4300    3.6653    3.0188 C   0  0  0  0  0  0  0  0  0  0  0  0
    2.9149    3.6861    2.0141 H   0  0  0  0  0  0  0  0  0  0  0  0
    1.7174    4.5212    3.0843 H   0  0  0  0  0  0  0  0  0  0  0  0
    3.2077    3.7755    3.8037 H   0  0  0  0  0  0  0  0  0  0  0  0
    2.6035    1.1262    3.2095 C   0  0  0  0  0  0  0  0  0  0  0  0
    3.3149    1.1899    4.0598 H   0  0  0  0  0  0  0  0  0  0  0  0
    2.0043    0.1902    3.2985 H   0  0  0  0  0  0  0  0  0  0  0  0
    3.1770    1.0822    2.2528 H   0  0  0  0  0  0  0  0  0  0  0  0
   -1.0750    2.0957    0.0106 Os  0  0  0  0  0  0  0  0  0  0  0  0
  1 85  1  0  0  0  0
  2  4  2  0  0  0  0
  2 85  1  0  0  0  0
  3  4  2  0  0  0  0
  3 85  1  0  0  0  0
  4 75  1  0  0  0  0
  5  6  2  0  0  0  0
  5 85  1  0  0  0  0
  7  9  1  0  0  0  0
  7 20  1  0  0  0  0
  7 31  1  0  0  0  0
  7 85  1  0  0  0  0
  8 42  1  0  0  0  0
  8 53  1  0  0  0  0
  8 64  1  0  0  0  0
  8 85  1  0  0  0  0
  9 10  2  0  0  0  0
  9 11  1  0  0  0  0
 10 12  1  0  0  0  0
```

```
 10 13  1  0  0  0  0
 11 14  2  0  0  0  0
 11 15  1  0  0  0  0
 12 16  2  0  0  0  0
 12 17  1  0  0  0  0
 14 16  1  0  0  0  0
 14 18  1  0  0  0  0
 16 19  1  0  0  0  0
 20 21  2  0  0  0  0
 20 22  1  0  0  0  0
 21 23  1  0  0  0  0
 21 24  1  0  0  0  0
 22 25  2  0  0  0  0
 22 26  1  0  0  0  0
 23 27  2  0  0  0  0
 23 28  1  0  0  0  0
 25 27  1  0  0  0  0
 25 29  1  0  0  0  0
 27 30  1  0  0  0  0
 31 32  2  0  0  0  0
 31 33  1  0  0  0  0
 32 34  1  0  0  0  0
 32 35  1  0  0  0  0
 33 36  2  0  0  0  0
 33 37  1  0  0  0  0
 34 38  2  0  0  0  0
 34 39  1  0  0  0  0
 36 38  1  0  0  0  0
 36 40  1  0  0  0  0
 38 41  1  0  0  0  0
 42 43  2  0  0  0  0
 42 44  1  0  0  0  0
 43 45  1  0  0  0  0
 43 46  1  0  0  0  0
 44 47  2  0  0  0  0
 44 48  1  0  0  0  0
 45 49  2  0  0  0  0
 45 50  1  0  0  0  0
 47 49  1  0  0  0  0
 47 51  1  0  0  0  0
 49 52  1  0  0  0  0
 53 54  2  0  0  0  0
 53 55  1  0  0  0  0
 54 56  1  0  0  0  0
 54 57  1  0  0  0  0
 55 58  2  0  0  0  0
 55 59  1  0  0  0  0
 56 60  2  0  0  0  0
 56 61  1  0  0  0  0
 58 60  1  0  0  0  0
 58 62  1  0  0  0  0
 60 63  1  0  0  0  0
 64 65  2  0  0  0  0
 64 66  1  0  0  0  0
 65 67  1  0  0  0  0
 65 68  1  0  0  0  0
 66 69  2  0  0  0  0
 66 70  1  0  0  0  0
 67 71  2  0  0  0  0
 67 72  1  0  0  0  0
 69 71  1  0  0  0  0
 69 73  1  0  0  0  0
 71 74  1  0  0  0  0
 75 76  1  0  0  0  0
 75 77  1  0  0  0  0
```

```
 75 81  1  0  0  0  0
 77 78  1  0  0  0  0
 77 79  1  0  0  0  0
 77 80  1  0  0  0  0
 81 82  1  0  0  0  0
 81 83  1  0  0  0  0
 81 84  1  0  0  0  0
M  END
$$$$
mol023
Optimized Catalyst 023
Optimized Catalyst 023
107115  0  0  0  0  0  0  0  0999 V2000
   -2.1189    0.6111   -0.0566 Os  0  0  0  0  0  0  0  0  0  0  0  0
   -1.8053    2.2309    0.0364 H   0  0  0  0  0  0  0  0  0  0  0  0
   -1.8877    1.1103   -2.3672 P   0  0  0  0  0  0  0  0  0  0  0  0
   -2.0571    0.9282    2.2950 P   0  0  0  0  0  0  0  0  0  0  0  0
   -1.8466   -1.7646   -0.0511 P   0  0  0  0  0  0  0  0  0  0  0  0
   -3.9872    0.6354   -0.1429 C   0  0  0  0  0  0  0  0  0  0  0  0
   -5.1654    0.6798   -0.1355 O   0  0  0  0  0  0  0  0  0  0  0  0
    0.6832    0.7009    0.0121 Cl  0  0  0  0  0  0  0  0  0  0  0  0
   -1.0769    0.0571   -3.6712 C   0  0  0  0  0  0  0  0  0  0  0  0
    0.1134   -0.5611   -3.2911 C   0  0  0  0  0  0  0  0  0  0  0  0
   -1.5587   -0.0595   -4.9749 C   0  0  0  0  0  0  0  0  0  0  0  0
    0.8126   -1.3444   -4.2186 C   0  0  0  0  0  0  0  0  0  0  0  0
    0.5215   -0.4371   -2.2800 H   0  0  0  0  0  0  0  0  0  0  0  0
   -0.8603   -0.8465   -5.8980 C   0  0  0  0  0  0  0  0  0  0  0  0
   -2.4606    0.4547   -5.3079 H   0  0  0  0  0  0  0  0  0  0  0  0
    0.3210   -1.4933   -5.5180 C   0  0  0  0  0  0  0  0  0  0  0  0
    1.7362   -1.8374   -3.9221 H   0  0  0  0  0  0  0  0  0  0  0  0
   -1.2344   -0.9522   -6.9138 H   0  0  0  0  0  0  0  0  0  0  0  0
    0.8589   -2.1089   -6.2369 H   0  0  0  0  0  0  0  0  0  0  0  0
   -1.0189    2.7277   -2.7216 C   0  0  0  0  0  0  0  0  0  0  0  0
   -1.1640    3.3026   -3.9885 C   0  0  0  0  0  0  0  0  0  0  0  0
   -0.2365    3.3366   -1.7468 C   0  0  0  0  0  0  0  0  0  0  0  0
   -0.5171    4.5072   -4.2767 C   0  0  0  0  0  0  0  0  0  0  0  0
   -1.7739    2.8379   -4.7643 H   0  0  0  0  0  0  0  0  0  0  0  0
    0.4154    4.5443   -2.0419 C   0  0  0  0  0  0  0  0  0  0  0  0
   -0.1041    2.8955   -0.7459 H   0  0  0  0  0  0  0  0  0  0  0  0
    0.2756    5.1273   -3.3016 C   0  0  0  0  0  0  0  0  0  0  0  0
   -0.6244    4.9619   -5.2588 H   0  0  0  0  0  0  0  0  0  0  0  0
    1.0362    5.0175   -1.2829 H   0  0  0  0  0  0  0  0  0  0  0  0
    0.7834    6.0629   -3.5305 H   0  0  0  0  0  0  0  0  0  0  0  0
   -3.6242    1.4870   -2.9398 C   0  0  0  0  0  0  0  0  0  0  0  0
   -4.1335    2.7350   -2.5698 C   0  0  0  0  0  0  0  0  0  0  0  0
   -4.4195    0.5621   -3.6126 C   0  0  0  0  0  0  0  0  0  0  0  0
   -5.4560    3.0622   -2.8898 C   0  0  0  0  0  0  0  0  0  0  0  0
   -3.5221    3.4625   -2.0264 H   0  0  0  0  0  0  0  0  0  0  0  0
   -5.7425    0.8943   -3.9311 C   0  0  0  0  0  0  0  0  0  0  0  0
   -4.0450   -0.4207   -3.9003 H   0  0  0  0  0  0  0  0  0  0  0  0
   -6.2598    2.1420   -3.5707 C   0  0  0  0  0  0  0  0  0  0  0  0
   -5.8607    4.0312   -2.6024 H   0  0  0  0  0  0  0  0  0  0  0  0
   -6.3703    0.1761   -4.4555 H   0  0  0  0  0  0  0  0  0  0  0  0
   -7.2904    2.3959   -3.8154 H   0  0  0  0  0  0  0  0  0  0  0  0
   -0.4584    1.6246    2.9148 C   0  0  0  0  0  0  0  0  0  0  0  0
    0.6763    0.8120    2.8676 C   0  0  0  0  0  0  0  0  0  0  0  0
   -0.3776    2.9482    3.3477 C   0  0  0  0  0  0  0  0  0  0  0  0
    1.9080    1.3281    3.2939 C   0  0  0  0  0  0  0  0  0  0  0  0
    0.6341   -0.2215    2.5033 H   0  0  0  0  0  0  0  0  0  0  0  0
    0.8553    3.4588    3.7695 C   0  0  0  0  0  0  0  0  0  0  0  0
   -1.2518    3.6029    3.3652 H   0  0  0  0  0  0  0  0  0  0  0  0
    1.9960    2.6483    3.7417 C   0  0  0  0  0  0  0  0  0  0  0  0
    2.7965    0.7019    3.2473 H   0  0  0  0  0  0  0  0  0  0  0  0
    0.9282    4.4885    4.1107 H   0  0  0  0  0  0  0  0  0  0  0  0
    2.9553    3.0510    4.0632 H   0  0  0  0  0  0  0  0  0  0  0  0
```

```
   -2.4922   -0.4008    3.5343 C   0  0  0  0  0  0  0  0  0  0  0  0
   -3.6942   -1.0784    3.3311 C   0  0  0  0  0  0  0  0  0  0  0  0
   -1.6710   -0.7083    4.6178 C   0  0  0  0  0  0  0  0  0  0  0  0
   -4.0615   -2.1099    4.2036 C   0  0  0  0  0  0  0  0  0  0  0  0
   -4.3588   -0.8211    2.5020 H   0  0  0  0  0  0  0  0  0  0  0  0
   -2.0457   -1.7350    5.4941 C   0  0  0  0  0  0  0  0  0  0  0  0
   -0.7374   -0.1721    4.8048 H   0  0  0  0  0  0  0  0  0  0  0  0
   -3.2346   -2.4399    5.2818 C   0  0  0  0  0  0  0  0  0  0  0  0
   -4.9868   -2.6587    4.0336 H   0  0  0  0  0  0  0  0  0  0  0  0
   -1.4080   -1.9843    6.3399 H   0  0  0  0  0  0  0  0  0  0  0  0
   -3.5186   -3.2446    5.9585 H   0  0  0  0  0  0  0  0  0  0  0  0
   -3.3294    2.2248    2.7307 C   0  0  0  0  0  0  0  0  0  0  0  0
   -3.7829    2.3514    4.0451 C   0  0  0  0  0  0  0  0  0  0  0  0
   -3.8037    3.0608    1.7230 C   0  0  0  0  0  0  0  0  0  0  0  0
   -4.7294    3.3353    4.3512 C   0  0  0  0  0  0  0  0  0  0  0  0
   -3.4195    1.7007    4.8437 H   0  0  0  0  0  0  0  0  0  0  0  0
   -4.7526    4.0437    2.0334 C   0  0  0  0  0  0  0  0  0  0  0  0
   -3.4459    2.9587    0.6845 H   0  0  0  0  0  0  0  0  0  0  0  0
   -5.2134    4.1803    3.3454 C   0  0  0  0  0  0  0  0  0  0  0  0
   -5.0909    3.4425    5.3719 H   0  0  0  0  0  0  0  0  0  0  0  0
   -5.1317    4.6970    1.2500 H   0  0  0  0  0  0  0  0  0  0  0  0
   -5.9514    4.9440    3.5873 H   0  0  0  0  0  0  0  0  0  0  0  0
   -1.7169   -2.6923   -1.6748 C   0  0  0  0  0  0  0  0  0  0  0  0
   -0.7071   -3.6042   -1.9697 C   0  0  0  0  0  0  0  0  0  0  0  0
   -2.7701   -2.4784   -2.5659 C   0  0  0  0  0  0  0  0  0  0  0  0
   -0.7234   -4.2728   -3.2015 C   0  0  0  0  0  0  0  0  0  0  0  0
    0.0960   -3.8292   -1.2672 H   0  0  0  0  0  0  0  0  0  0  0  0
   -2.7875   -3.1520   -3.7918 C   0  0  0  0  0  0  0  0  0  0  0  0
   -3.5886   -1.7993   -2.3149 H   0  0  0  0  0  0  0  0  0  0  0  0
   -1.7567   -4.0417   -4.1135 C   0  0  0  0  0  0  0  0  0  0  0  0
    0.0723   -4.9734   -3.4469 H   0  0  0  0  0  0  0  0  0  0  0  0
   -3.5980   -2.9835   -4.4973 H   0  0  0  0  0  0  0  0  0  0  0  0
   -1.7610   -4.5545   -5.0738 H   0  0  0  0  0  0  0  0  0  0  0  0
   -0.3413   -2.2471    0.9388 C   0  0  0  0  0  0  0  0  0  0  0  0
   -0.4887   -2.6121    2.2788 C   0  0  0  0  0  0  0  0  0  0  0  0
    0.9312   -2.1275    0.3767 C   0  0  0  0  0  0  0  0  0  0  0  0
    0.6415   -2.8858    3.0562 C   0  0  0  0  0  0  0  0  0  0  0  0
   -1.4726   -2.6831    2.7531 H   0  0  0  0  0  0  0  0  0  0  0  0
    2.0627   -2.4029    1.1592 C   0  0  0  0  0  0  0  0  0  0  0  0
    1.0827   -1.8183   -0.6611 H   0  0  0  0  0  0  0  0  0  0  0  0
    1.9189   -2.7819    2.4954 C   0  0  0  0  0  0  0  0  0  0  0  0
    0.5231   -3.1702    4.0995 H   0  0  0  0  0  0  0  0  0  0  0  0
    3.0546   -2.2973    0.7237 H   0  0  0  0  0  0  0  0  0  0  0  0
    2.7988   -2.9902    3.1015 H   0  0  0  0  0  0  0  0  0  0  0  0
   -3.1999   -2.8868    0.6288 C   0  0  0  0  0  0  0  0  0  0  0  0
   -2.8532   -4.1561    1.1014 C   0  0  0  0  0  0  0  0  0  0  0  0
   -4.5369   -2.5047    0.5654 C   0  0  0  0  0  0  0  0  0  0  0  0
   -3.8478   -5.0150    1.5807 C   0  0  0  0  0  0  0  0  0  0  0  0
   -1.8189   -4.5104    1.0979 H   0  0  0  0  0  0  0  0  0  0  0  0
   -5.5344   -3.3694    1.0383 C   0  0  0  0  0  0  0  0  0  0  0  0
   -4.8448   -1.5475    0.1445 H   0  0  0  0  0  0  0  0  0  0  0  0
   -5.1894   -4.6187    1.5578 C   0  0  0  0  0  0  0  0  0  0  0  0
   -3.5771   -5.9969    1.9653 H   0  0  0  0  0  0  0  0  0  0  0  0
   -6.5784   -3.0628    0.9966 H   0  0  0  0  0  0  0  0  0  0  0  0
   -5.9620   -5.2874    1.9337 H   0  0  0  0  0  0  0  0  0  0  0  0
  1  2  1  0  0  0  0
  1  3  1  0  0  0  0
  1  4  1  0  0  0  0
  1  5  1  0  0  0  0
  1  6  1  0  0  0  0
  1  8  1  0  0  0  0
  3  9  1  0  0  0  0
  3 20  1  0  0  0  0
  3 31  1  0  0  0  0
  4 42  1  0  0  0  0
```

```
  4 53  1  0  0  0  0
  4 64  1  0  0  0  0
  5 75  1  0  0  0  0
  5 86  1  0  0  0  0
  5 97  1  0  0  0  0
  6  7  2  0  0  0  0
  9 10  2  0  0  0  0
  9 11  1  0  0  0  0
 10 12  1  0  0  0  0
 10 13  1  0  0  0  0
 11 14  2  0  0  0  0
 11 15  1  0  0  0  0
 12 16  2  0  0  0  0
 12 17  1  0  0  0  0
 14 16  1  0  0  0  0
 14 18  1  0  0  0  0
 16 19  1  0  0  0  0
 20 21  2  0  0  0  0
 20 22  1  0  0  0  0
 21 23  1  0  0  0  0
 21 24  1  0  0  0  0
 22 25  2  0  0  0  0
 22 26  1  0  0  0  0
 23 27  2  0  0  0  0
 23 28  1  0  0  0  0
 25 27  1  0  0  0  0
 25 29  1  0  0  0  0
 27 30  1  0  0  0  0
 31 32  2  0  0  0  0
 31 33  1  0  0  0  0
 32 34  1  0  0  0  0
 32 35  1  0  0  0  0
 33 36  2  0  0  0  0
 33 37  1  0  0  0  0
 34 38  2  0  0  0  0
 34 39  1  0  0  0  0
 36 38  1  0  0  0  0
 36 40  1  0  0  0  0
 38 41  1  0  0  0  0
 42 43  2  0  0  0  0
 42 44  1  0  0  0  0
 43 45  1  0  0  0  0
 43 46  1  0  0  0  0
 44 47  2  0  0  0  0
 44 48  1  0  0  0  0
 45 49  2  0  0  0  0
 45 50  1  0  0  0  0
 47 49  1  0  0  0  0
 47 51  1  0  0  0  0
 49 52  1  0  0  0  0
 53 54  2  0  0  0  0
 53 55  1  0  0  0  0
 54 56  1  0  0  0  0
 54 57  1  0  0  0  0
 55 58  2  0  0  0  0
 55 59  1  0  0  0  0
 56 60  2  0  0  0  0
 56 61  1  0  0  0  0
 58 60  1  0  0  0  0
 58 62  1  0  0  0  0
 60 63  1  0  0  0  0
 64 65  2  0  0  0  0
 64 66  1  0  0  0  0
 65 67  1  0  0  0  0
 65 68  1  0  0  0  0
```

```
 66 69  2  0  0  0  0
 66 70  1  0  0  0  0
 67 71  2  0  0  0  0
 67 72  1  0  0  0  0
 69 71  1  0  0  0  0
 69 73  1  0  0  0  0
 71 74  1  0  0  0  0
 75 76  2  0  0  0  0
 75 77  1  0  0  0  0
 76 78  1  0  0  0  0
 76 79  1  0  0  0  0
 77 80  2  0  0  0  0
 77 81  1  0  0  0  0
 78 82  2  0  0  0  0
 78 83  1  0  0  0  0
 80 82  1  0  0  0  0
 80 84  1  0  0  0  0
 82 85  1  0  0  0  0
 86 87  2  0  0  0  0
 86 88  1  0  0  0  0
 87 89  1  0  0  0  0
 87 90  1  0  0  0  0
 88 91  2  0  0  0  0
 88 92  1  0  0  0  0
 89 93  2  0  0  0  0
 89 94  1  0  0  0  0
 91 93  1  0  0  0  0
 91 95  1  0  0  0  0
 93 96  1  0  0  0  0
 97 98  2  0  0  0  0
 97 99  1  0  0  0  0
 98100  1  0  0  0  0
 98101  1  0  0  0  0
 99102  2  0  0  0  0
 99103  1  0  0  0  0
100104  2  0  0  0  0
100105  1  0  0  0  0
102104  1  0  0  0  0
102106  1  0  0  0  0
104107  1  0  0  0  0
M  END
$$$$
mol024
Optimized Catalyst 024
Optimized Catalyst 024
 47 47  0  0  0  0  0  0  0  0999 V2000
   -1.4873   -0.0105    0.4141 Rh  0  0  2  0  0  0  0  0  0  0  0  0
   -2.4631    0.1627    1.9256 C   0  0  0  0  0  0  0  0  0  0  0  0
   -3.0354    0.4567    2.8716 O   0  0  0  0  0  0  0  0  0  0  0  0
   -1.4548   -2.0857    0.0494 P   0  0  0  0  0  0  0  0  0  0  0  0
   -1.3062    2.1577    0.6860 O   0  0  0  0  0  0  0  0  0  0  0  0
   -0.5953    2.6860   -0.1875 C   0  0  0  0  0  0  0  0  0  0  0  0
   -0.3544   -0.3404   -1.2494 O   0  0  0  0  0  0  0  0  0  0  0  0
   -0.4652    4.1951   -0.0837 C   0  0  0  0  0  0  0  0  0  0  0  0
    0.6000    4.4738    0.0746 H   0  0  0  0  0  0  0  0  0  0  0  0
   -0.8407    4.6723   -1.0159 H   0  0  0  0  0  0  0  0  0  0  0  0
   -1.0698    4.5589    0.7811 H   0  0  0  0  0  0  0  0  0  0  0  0
    0.1184    2.0615   -1.2622 C   0  0  0  0  0  0  0  0  0  0  0  0
    0.7005    2.7723   -1.8575 H   0  0  0  0  0  0  0  0  0  0  0  0
    0.1978    0.7515   -1.6881 C   0  0  0  0  0  0  0  0  0  0  0  0
    1.0651    0.4246   -2.8929 C   0  0  0  0  0  0  0  0  0  0  0  0
    2.1106    0.7579   -2.7016 H   0  0  0  0  0  0  0  0  0  0  0  0
    1.0617   -0.6719   -3.0882 H   0  0  0  0  0  0  0  0  0  0  0  0
    0.6653    0.9546   -3.7882 H   0  0  0  0  0  0  0  0  0  0  0  0
   -2.9088   -2.9365    0.9285 C   0  0  0  0  0  0  0  0  0  0  0  0
```

```
   -2.8931   -4.0327    0.7095 H   0  0  0  0  0  0  0  0  0  0  0  0
   -2.8195   -2.7898    2.0330 H   0  0  0  0  0  0  0  0  0  0  0  0
   -4.1618   -2.2961    0.4261 C   0  0  0  0  0  0  0  0  0  0  0  0
   -4.1441   -1.1912    0.3693 H   0  0  0  0  0  0  0  0  0  0  0  0
   -5.2502   -2.9802    0.0742 C   0  0  0  0  0  0  0  0  0  0  0  0
   -6.1525   -2.4824   -0.2817 H   0  0  0  0  0  0  0  0  0  0  0  0
   -5.3138   -4.0651    0.1273 H   0  0  0  0  0  0  0  0  0  0  0  0
   -1.6762   -2.5558   -1.7692 C   0  0  0  0  0  0  0  0  0  0  0  0
   -0.9065   -1.9863   -2.3679 H   0  0  0  0  0  0  0  0  0  0  0  0
   -2.6874   -2.1857   -2.0888 H   0  0  0  0  0  0  0  0  0  0  0  0
   -1.5371   -4.0248   -1.9627 C   0  0  0  0  0  0  0  0  0  0  0  0
   -0.5194   -4.3740   -2.1682 H   0  0  0  0  0  0  0  0  0  0  0  0
   -2.5596   -4.8815   -1.9420 C   0  0  0  0  0  0  0  0  0  0  0  0
   -2.4215   -5.9487   -2.1065 H   0  0  0  0  0  0  0  0  0  0  0  0
   -3.5906   -4.5699   -1.7802 H   0  0  0  0  0  0  0  0  0  0  0  0
    0.1615   -2.8251    0.8007 C   0  0  0  0  0  0  0  0  0  0  0  0
    0.0009   -4.3112    1.1262 C   0  0  0  0  0  0  0  0  0  0  0  0
   -0.3119   -4.9053    0.2383 H   0  0  0  0  0  0  0  0  0  0  0  0
    0.9915   -4.7067    1.4633 H   0  0  0  0  0  0  0  0  0  0  0  0
   -0.7214   -4.4803    1.9560 H   0  0  0  0  0  0  0  0  0  0  0  0
    0.3977   -1.9910    2.0707 C   0  0  0  0  0  0  0  0  0  0  0  0
    1.3829   -2.2663    2.5160 H   0  0  0  0  0  0  0  0  0  0  0  0
    0.4393   -0.8813    1.8249 H   0  0  0  0  0  0  0  0  0  0  0  0
   -0.3838   -2.1656    2.8432 H   0  0  0  0  0  0  0  0  0  0  0  0
    1.2912   -2.5895   -0.2067 C   0  0  0  0  0  0  0  0  0  0  0  0
    2.2718   -2.8046    0.2792 H   0  0  0  0  0  0  0  0  0  0  0  0
    1.2012   -3.2434   -1.0999 H   0  0  0  0  0  0  0  0  0  0  0  0
    1.3041   -1.5132   -0.5382 H   0  0  0  0  0  0  0  0  0  0  0  0
  1  2  1  6  0  0  0
  1  4  1  0  0  0  0
  1  5  1  0  0  0  0
  1  7  1  0  0  0  0
  2  3  3  0  0  0  0
  4 19  1  0  0  0  0
  4 27  1  0  0  0  0
  4 35  1  0  0  0  0
  5  6  2  0  0  0  0
  6  8  1  0  0  0  0
  6 12  1  0  0  0  0
  7 14  1  0  0  0  0
  8  9  1  0  0  0  0
  8 10  1  0  0  0  0
  8 11  1  0  0  0  0
 12 13  1  0  0  0  0
 12 14  2  0  0  0  0
 14 15  1  0  0  0  0
 15 16  1  0  0  0  0
 15 17  1  0  0  0  0
 15 18  1  0  0  0  0
 19 20  1  0  0  0  0
 19 21  1  0  0  0  0
 19 22  1  0  0  0  0
 22 23  1  0  0  0  0
 22 24  2  0  0  0  0
 24 25  1  0  0  0  0
 24 26  1  0  0  0  0
 27 28  1  0  0  0  0
 27 29  1  0  0  0  0
 27 30  1  0  0  0  0
 30 31  1  0  0  0  0
 30 32  2  0  0  0  0
 32 33  1  0  0  0  0
 32 34  1  0  0  0  0
 35 36  1  0  0  0  0
 35 40  1  0  0  0  0
```

```
 35 44  1  0  0  0  0
 36 37  1  0  0  0  0
 36 38  1  0  0  0  0
 36 39  1  0  0  0  0
 40 41  1  0  0  0  0
 40 43  1  0  0  0  0
 40 42  1  0  0  0  0
 44 45  1  0  0  0  0
 44 46  1  0  0  0  0
 44 47  1  0  0  0  0
M  END
$$$$
mol025
Optimized Catalyst 025
Optimized Catalyst 025
102109  0  0  0  0  0  0  0  0999 V2000
    3.1847   -0.6440   -1.6254 P   0  0  0  0  0  0  0  0  0  0  0  0
    1.5835    1.0477    0.7661 P   0  0  0  0  0  0  0  0  0  0  0  0
    1.7878   -2.2817    0.9268 P   0  0  0  0  0  0  0  0  0  0  0  0
    1.3659   -0.6767   -2.2166 C   0  0  0  0  0  0  0  0  0  0  0  0
    1.1634    0.2580   -2.7932 H   0  0  0  0  0  0  0  0  0  0  0  0
    1.2655   -1.5280   -2.9380 H   0  0  0  0  0  0  0  0  0  0  0  0
    0.2498   -0.8013   -1.1488 C   0  0  0  0  0  0  0  0  0  0  0  0
   -1.0800   -0.9217   -1.9538 C   0  0  0  0  0  0  0  0  0  0  0  0
   -1.9567   -0.9836   -1.2702 H   0  0  0  0  0  0  0  0  0  0  0  0
   -1.2262   -0.0400   -2.6179 H   0  0  0  0  0  0  0  0  0  0  0  0
   -1.0794   -1.8339   -2.5922 H   0  0  0  0  0  0  0  0  0  0  0  0
    0.3436   -2.1038   -0.3146 C   0  0  0  0  0  0  0  0  0  0  0  0
   -0.6087   -2.2266    0.2627 H   0  0  0  0  0  0  0  0  0  0  0  0
    0.3917   -2.9527   -1.0387 H   0  0  0  0  0  0  0  0  0  0  0  0
    0.0893    0.4716   -0.2797 C   0  0  0  0  0  0  0  0  0  0  0  0
   -0.7748    0.2927    0.4049 H   0  0  0  0  0  0  0  0  0  0  0  0
   -0.1993    1.3244   -0.9465 H   0  0  0  0  0  0  0  0  0  0  0  0
    2.4196   -4.0281    0.6824 C   0  0  0  0  0  0  0  0  0  0  0  0
    3.6926   -4.3428    1.1674 C   0  0  0  0  0  0  0  0  0  0  0  0
    1.6210   -5.0224    0.1120 C   0  0  0  0  0  0  0  0  0  0  0  0
    4.2021   -5.6351    1.0048 C   0  0  0  0  0  0  0  0  0  0  0  0
    4.2998   -3.6068    1.6988 H   0  0  0  0  0  0  0  0  0  0  0  0
    2.1339   -6.3154   -0.0509 C   0  0  0  0  0  0  0  0  0  0  0  0
    0.5883   -4.8455   -0.1956 H   0  0  0  0  0  0  0  0  0  0  0  0
    3.4277   -6.6200    0.3820 C   0  0  0  0  0  0  0  0  0  0  0  0
    5.1983   -5.8827    1.3744 H   0  0  0  0  0  0  0  0  0  0  0  0
    1.5145   -7.0937   -0.5051 H   0  0  0  0  0  0  0  0  0  0  0  0
    3.8263   -7.6302    0.2502 H   0  0  0  0  0  0  0  0  0  0  0  0
    0.9182   -2.4640    2.5810 C   0  0  0  0  0  0  0  0  0  0  0  0
   -0.1228   -1.6065    2.9430 C   0  0  0  0  0  0  0  0  0  0  0  0
    1.3030   -3.4957    3.4441 C   0  0  0  0  0  0  0  0  0  0  0  0
   -0.7526   -1.7531    4.1845 C   0  0  0  0  0  0  0  0  0  0  0  0
   -0.4826   -0.8152    2.2892 H   0  0  0  0  0  0  0  0  0  0  0  0
    0.6641   -3.6480    4.6799 C   0  0  0  0  0  0  0  0  0  0  0  0
    2.0881   -4.2118    3.1858 H   0  0  0  0  0  0  0  0  0  0  0  0
   -0.3607   -2.7741    5.0553 C   0  0  0  0  0  0  0  0  0  0  0  0
   -1.5584   -1.0726    4.4686 H   0  0  0  0  0  0  0  0  0  0  0  0
    0.9539   -4.4645    5.3458 H   0  0  0  0  0  0  0  0  0  0  0  0
   -0.8620   -2.8968    6.0196 H   0  0  0  0  0  0  0  0  0  0  0  0
    3.9373   -2.1529   -2.4517 C   0  0  0  0  0  0  0  0  0  0  0  0
    3.2842   -3.3868   -2.4244 C   0  0  0  0  0  0  0  0  0  0  0  0
    5.1545   -2.0237   -3.1292 C   0  0  0  0  0  0  0  0  0  0  0  0
    3.8663   -4.5021   -3.0383 C   0  0  0  0  0  0  0  0  0  0  0  0
    2.3183   -3.5267   -1.9443 H   0  0  0  0  0  0  0  0  0  0  0  0
    5.7291   -3.1372   -3.7524 C   0  0  0  0  0  0  0  0  0  0  0  0
    5.6820   -1.0690   -3.2067 H   0  0  0  0  0  0  0  0  0  0  0  0
    5.0897   -4.3797   -3.7037 C   0  0  0  0  0  0  0  0  0  0  0  0
    3.3578   -5.4682   -3.0041 H   0  0  0  0  0  0  0  0  0  0  0  0
    6.6714   -3.0313   -4.2957 H   0  0  0  0  0  0  0  0  0  0  0  0
```

```
    5.5378   -5.2481   -4.1947 H   0  0  0  0  0  0  0  0  0  0  0  0
    3.9896    0.7239   -2.6200 C   0  0  0  0  0  0  0  0  0  0  0  0
    3.4493    1.1641   -3.8308 C   0  0  0  0  0  0  0  0  0  0  0  0
    5.2048    1.2434   -2.1635 C   0  0  0  0  0  0  0  0  0  0  0  0
    4.0852    2.1898   -4.5417 C   0  0  0  0  0  0  0  0  0  0  0  0
    2.5532    0.7230   -4.2721 H   0  0  0  0  0  0  0  0  0  0  0  0
    5.8385    2.2665   -2.8761 C   0  0  0  0  0  0  0  0  0  0  0  0
    5.6934    0.8524   -1.2685 H   0  0  0  0  0  0  0  0  0  0  0  0
    5.2712    2.7510   -4.0597 C   0  0  0  0  0  0  0  0  0  0  0  0
    3.6600    2.5429   -5.4852 H   0  0  0  0  0  0  0  0  0  0  0  0
    6.7827    2.6805   -2.5188 H   0  0  0  0  0  0  0  0  0  0  0  0
    5.7633    3.5542   -4.6159 H   0  0  0  0  0  0  0  0  0  0  0  0
    1.9532    2.7598    0.0894 C   0  0  0  0  0  0  0  0  0  0  0  0
    2.0152    2.9899   -1.2865 C   0  0  0  0  0  0  0  0  0  0  0  0
    2.1369    3.8211    0.9822 C   0  0  0  0  0  0  0  0  0  0  0  0
    2.3017    4.2716   -1.7710 C   0  0  0  0  0  0  0  0  0  0  0  0
    1.8406    2.2051   -2.0191 H   0  0  0  0  0  0  0  0  0  0  0  0
    2.4119    5.1042    0.4956 C   0  0  0  0  0  0  0  0  0  0  0  0
    2.0609    3.6936    2.0655 H   0  0  0  0  0  0  0  0  0  0  0  0
    2.5006    5.3313   -0.8812 C   0  0  0  0  0  0  0  0  0  0  0  0
    2.3612    4.4446   -2.8479 H   0  0  0  0  0  0  0  0  0  0  0  0
    2.5389    5.9356    1.1933 H   0  0  0  0  0  0  0  0  0  0  0  0
    2.7114    6.3353   -1.2603 H   0  0  0  0  0  0  0  0  0  0  0  0
    0.8590    1.4925    2.4352 C   0  0  0  0  0  0  0  0  0  0  0  0
   -0.4945    1.8040    2.5860 C   0  0  0  0  0  0  0  0  0  0  0  0
    1.7277    1.5914    3.5263 C   0  0  0  0  0  0  0  0  0  0  0  0
   -0.9940    2.1309    3.8530 C   0  0  0  0  0  0  0  0  0  0  0  0
   -1.1909    1.8310    1.7454 H   0  0  0  0  0  0  0  0  0  0  0  0
    1.2267    1.9197    4.7902 C   0  0  0  0  0  0  0  0  0  0  0  0
    2.8039    1.4423    3.4155 H   0  0  0  0  0  0  0  0  0  0  0  0
   -0.1385    2.1760    4.9576 C   0  0  0  0  0  0  0  0  0  0  0  0
   -2.0547    2.3663    3.9760 H   0  0  0  0  0  0  0  0  0  0  0  0
    1.8999    1.9900    5.6459 H   0  0  0  0  0  0  0  0  0  0  0  0
   -0.5327    2.4285    5.9464 H   0  0  0  0  0  0  0  0  0  0  0  0
    4.9405   -1.7297    0.2670 N   0  0  0  0  0  0  0  0  0  0  0  0
    5.9435   -2.2688    0.0384 C   0  0  0  0  0  0  0  0  0  0  0  0
    7.1642   -2.9573   -0.2774 C   0  0  0  0  0  0  0  0  0  0  0  0
    7.3483   -3.7935    0.4526 H   0  0  0  0  0  0  0  0  0  0  0  0
    7.0989   -3.4006   -1.3128 H   0  0  0  0  0  0  0  0  0  0  0  0
    8.0435   -2.2573   -0.2435 H   0  0  0  0  0  0  0  0  0  0  0  0
    4.3753    1.2547    0.7530 N   0  0  0  0  0  0  0  0  0  0  0  0
    4.9904    2.2219    0.9407 C   0  0  0  0  0  0  0  0  0  0  0  0
    5.7227    3.4405    1.1469 C   0  0  0  0  0  0  0  0  0  0  0  0
    6.0086    3.5620    2.2276 H   0  0  0  0  0  0  0  0  0  0  0  0
    6.6625    3.4483    0.5284 H   0  0  0  0  0  0  0  0  0  0  0  0
    5.0918    4.3246    0.8417 H   0  0  0  0  0  0  0  0  0  0  0  0
    3.5138   -1.1140    3.8713 C   0  0  0  0  0  0  0  0  0  0  0  0
    3.3538   -0.8486    2.7520 N   0  0  0  0  0  0  0  0  0  0  0  0
    3.6681   -1.4511    5.2593 C   0  0  0  0  0  0  0  0  0  0  0  0
    2.7822   -2.0559    5.6090 H   0  0  0  0  0  0  0  0  0  0  0  0
    4.6016   -2.0551    5.4272 H   0  0  0  0  0  0  0  0  0  0  0  0
    3.7295   -0.5207    5.8889 H   0  0  0  0  0  0  0  0  0  0  0  0
    3.2037   -0.5338    0.6396 Ru  0  0  0  0  0  0  0  0  0  0  0  0
  1  4  1  0  0  0  0
  1 40  1  0  0  0  0
  1 51  1  0  0  0  0
  1102  1  0  0  0  0
  2 15  1  0  0  0  0
  2 62  1  0  0  0  0
  2 73  1  0  0  0  0
  2102  1  0  0  0  0
  3 12  1  0  0  0  0
  3 18  1  0  0  0  0
  3 29  1  0  0  0  0
  3102  1  0  0  0  0
```

```
  4  5  1  0  0  0  0
  4  6  1  0  0  0  0
  4  7  1  0  0  0  0
  7  8  1  0  0  0  0
  7 12  1  0  0  0  0
  7 15  1  0  0  0  0
  8  9  1  0  0  0  0
  8 10  1  0  0  0  0
  8 11  1  0  0  0  0
 12 13  1  0  0  0  0
 12 14  1  0  0  0  0
 15 16  1  0  0  0  0
 15 17  1  0  0  0  0
 18 19  2  0  0  0  0
 18 20  1  0  0  0  0
 19 21  1  0  0  0  0
 19 22  1  0  0  0  0
 20 23  2  0  0  0  0
 20 24  1  0  0  0  0
 21 25  2  0  0  0  0
 21 26  1  0  0  0  0
 23 25  1  0  0  0  0
 23 27  1  0  0  0  0
 25 28  1  0  0  0  0
 29 30  2  0  0  0  0
 29 31  1  0  0  0  0
 30 32  1  0  0  0  0
 30 33  1  0  0  0  0
 31 34  2  0  0  0  0
 31 35  1  0  0  0  0
 32 36  2  0  0  0  0
 32 37  1  0  0  0  0
 34 36  1  0  0  0  0
 34 38  1  0  0  0  0
 36 39  1  0  0  0  0
 40 41  2  0  0  0  0
 40 42  1  0  0  0  0
 41 43  1  0  0  0  0
 41 44  1  0  0  0  0
 42 45  2  0  0  0  0
 42 46  1  0  0  0  0
 43 47  2  0  0  0  0
 43 48  1  0  0  0  0
 45 47  1  0  0  0  0
 45 49  1  0  0  0  0
 47 50  1  0  0  0  0
 51 52  2  0  0  0  0
 51 53  1  0  0  0  0
 52 54  1  0  0  0  0
 52 55  1  0  0  0  0
 53 56  2  0  0  0  0
 53 57  1  0  0  0  0
 54 58  2  0  0  0  0
 54 59  1  0  0  0  0
 56 58  1  0  0  0  0
 56 60  1  0  0  0  0
 58 61  1  0  0  0  0
 62 63  2  0  0  0  0
 62 64  1  0  0  0  0
 63 65  1  0  0  0  0
 63 66  1  0  0  0  0
 64 67  2  0  0  0  0
 64 68  1  0  0  0  0
 65 69  2  0  0  0  0
 65 70  1  0  0  0  0
```

```
 67 69  1  0  0  0  0
 67 71  1  0  0  0  0
 69 72  1  0  0  0  0
 73 74  2  0  0  0  0
 73 75  1  0  0  0  0
 74 76  1  0  0  0  0
 74 77  1  0  0  0  0
 75 78  2  0  0  0  0
 75 79  1  0  0  0  0
 76 80  2  0  0  0  0
 76 81  1  0  0  0  0
 78 80  1  0  0  0  0
 78 82  1  0  0  0  0
 80 83  1  0  0  0  0
 84 85  3  0  0  0  0
 84102  1  0  0  0  0
 85 86  1  0  0  0  0
 86 87  1  0  0  0  0
 86 88  1  0  0  0  0
 86 89  1  0  0  0  0
 90 91  3  0  0  0  0
 90102  1  0  0  0  0
 91 92  1  0  0  0  0
 92 93  1  0  0  0  0
 92 94  1  0  0  0  0
 92 95  1  0  0  0  0
 96 97  3  0  0  0  0
 96 98  1  0  0  0  0
 97102  1  0  0  0  0
 98 99  1  0  0  0  0
 98100  1  0  0  0  0
 98101  1  0  0  0  0
M  END
$$$$
mol037
Optimized Catalyst 037
Optimized Catalyst 037
106114  0  0  0  0  0  0  0  0999 V2000
   -1.8230    0.2062   -0.3139 H   0  0  0  0  0  0  0  0  0  0  0  0
    1.8523    0.0462   -0.5935 P   0  0  0  0  0  0  0  0  0  0  0  0
   -1.3343   -2.5445    0.5731 P   0  0  0  0  0  0  0  0  0  0  0  0
   -1.0632    2.8599    0.6852 P   0  0  0  0  0  0  0  0  0  0  0  0
    2.9490    1.4993   -0.1213 C   0  0  0  0  0  0  0  0  0  0  0  0
    3.7966    2.0999   -1.0544 C   0  0  0  0  0  0  0  0  0  0  0  0
    2.8785    1.9799    1.1891 C   0  0  0  0  0  0  0  0  0  0  0  0
    4.5761    3.1990   -0.6728 C   0  0  0  0  0  0  0  0  0  0  0  0
    3.8644    1.7423   -2.0844 H   0  0  0  0  0  0  0  0  0  0  0  0
    3.6580    3.0810    1.5638 C   0  0  0  0  0  0  0  0  0  0  0  0
    2.2324    1.5243    1.9444 H   0  0  0  0  0  0  0  0  0  0  0  0
    4.5055    3.6911    0.6339 C   0  0  0  0  0  0  0  0  0  0  0  0
    5.2401    3.6691   -1.3970 H   0  0  0  0  0  0  0  0  0  0  0  0
    3.5944    3.4665    2.5818 H   0  0  0  0  0  0  0  0  0  0  0  0
    2.9193   -1.4375   -0.1631 C   0  0  0  0  0  0  0  0  0  0  0  0
    2.7565   -2.6230   -0.8845 C   0  0  0  0  0  0  0  0  0  0  0  0
    3.8237   -1.3725    0.8991 C   0  0  0  0  0  0  0  0  0  0  0  0
    3.4999   -3.7558   -0.5349 C   0  0  0  0  0  0  0  0  0  0  0  0
    2.0562   -2.6964   -1.7216 H   0  0  0  0  0  0  0  0  0  0  0  0
    4.5632   -2.5103    1.2465 C   0  0  0  0  0  0  0  0  0  0  0  0
    3.9793   -0.4546    1.4708 H   0  0  0  0  0  0  0  0  0  0  0  0
    4.4019   -3.7012    0.5322 C   0  0  0  0  0  0  0  0  0  0  0  0
    3.3689   -4.6812   -1.0944 H   0  0  0  0  0  0  0  0  0  0  0  0
    5.2679   -2.4644    2.0763 H   0  0  0  0  0  0  0  0  0  0  0  0
    1.8518    0.0838   -2.4709 C   0  0  0  0  0  0  0  0  0  0  0  0
    2.8980   -0.4675   -3.2162 C   0  0  0  0  0  0  0  0  0  0  0  0
    0.7648    0.6837   -3.1076 C   0  0  0  0  0  0  0  0  0  0  0  0
```

```
    2.8442   -0.4296   -4.6136 C   0  0  0  0  0  0  0  0  0  0  0  0
    3.7644   -0.9301   -2.7359 H   0  0  0  0  0  0  0  0  0  0  0  0
    0.7198    0.7264   -4.5076 C   0  0  0  0  0  0  0  0  0  0  0  0
   -0.0587    1.1375   -2.5479 H   0  0  0  0  0  0  0  0  0  0  0  0
    1.7552    0.1661   -5.2597 C   0  0  0  0  0  0  0  0  0  0  0  0
    3.6539   -0.8622   -5.1998 H   0  0  0  0  0  0  0  0  0  0  0  0
   -0.1248    1.2013   -5.0059 H   0  0  0  0  0  0  0  0  0  0  0  0
   -0.0301    3.8385    1.9012 C   0  0  0  0  0  0  0  0  0  0  0  0
    0.5145    5.0725    1.5350 C   0  0  0  0  0  0  0  0  0  0  0  0
    0.1500    3.3358    3.1913 C   0  0  0  0  0  0  0  0  0  0  0  0
    1.2723    5.7950    2.4638 C   0  0  0  0  0  0  0  0  0  0  0  0
    0.3702    5.5003    0.5394 H   0  0  0  0  0  0  0  0  0  0  0  0
    0.9044    4.0665    4.1180 C   0  0  0  0  0  0  0  0  0  0  0  0
   -0.2818    2.3850    3.5158 H   0  0  0  0  0  0  0  0  0  0  0  0
    1.4691    5.2923    3.7539 C   0  0  0  0  0  0  0  0  0  0  0  0
    1.7077    6.7528    2.1797 H   0  0  0  0  0  0  0  0  0  0  0  0
    1.0483    3.6758    5.1260 H   0  0  0  0  0  0  0  0  0  0  0  0
   -2.7836    3.5488    0.9405 C   0  0  0  0  0  0  0  0  0  0  0  0
   -3.8475    2.6710    1.1525 C   0  0  0  0  0  0  0  0  0  0  0  0
   -2.9850    4.9315    0.9198 C   0  0  0  0  0  0  0  0  0  0  0  0
   -5.1367    3.1857    1.3401 C   0  0  0  0  0  0  0  0  0  0  0  0
   -3.7089    1.5814    1.1798 H   0  0  0  0  0  0  0  0  0  0  0  0
   -4.2747    5.4392    1.1107 C   0  0  0  0  0  0  0  0  0  0  0  0
   -2.1630    5.6319    0.7592 H   0  0  0  0  0  0  0  0  0  0  0  0
   -5.3488    4.5675    1.3203 C   0  0  0  0  0  0  0  0  0  0  0  0
   -5.9740    2.5071    1.5037 H   0  0  0  0  0  0  0  0  0  0  0  0
   -4.4416    6.5160    1.0984 H   0  0  0  0  0  0  0  0  0  0  0  0
   -0.5719    3.4642   -1.0130 C   0  0  0  0  0  0  0  0  0  0  0  0
   -1.5573    3.6357   -1.9892 C   0  0  0  0  0  0  0  0  0  0  0  0
    0.7723    3.7066   -1.3030 C   0  0  0  0  0  0  0  0  0  0  0  0
   -1.1854    4.0495   -3.2737 C   0  0  0  0  0  0  0  0  0  0  0  0
   -2.6160    3.4579   -1.7805 H   0  0  0  0  0  0  0  0  0  0  0  0
    1.1372    4.1158   -2.5908 C   0  0  0  0  0  0  0  0  0  0  0  0
    1.5603    3.5845   -0.5527 H   0  0  0  0  0  0  0  0  0  0  0  0
    0.1598    4.2869   -3.5757 C   0  0  0  0  0  0  0  0  0  0  0  0
   -1.9479    4.1870   -4.0400 H   0  0  0  0  0  0  0  0  0  0  0  0
    2.1877    4.2924   -2.8222 H   0  0  0  0  0  0  0  0  0  0  0  0
   -0.5312   -3.5220    1.9483 C   0  0  0  0  0  0  0  0  0  0  0  0
    0.8589   -3.4621    2.0793 C   0  0  0  0  0  0  0  0  0  0  0  0
   -1.2985   -4.3082    2.8094 C   0  0  0  0  0  0  0  0  0  0  0  0
    1.4893   -4.2045    3.0828 C   0  0  0  0  0  0  0  0  0  0  0  0
    1.4806   -2.8559    1.4140 H   0  0  0  0  0  0  0  0  0  0  0  0
   -0.6612   -5.0432    3.8173 C   0  0  0  0  0  0  0  0  0  0  0  0
   -2.3865   -4.3715    2.7230 H   0  0  0  0  0  0  0  0  0  0  0  0
    0.7292   -4.9937    3.9533 C   0  0  0  0  0  0  0  0  0  0  0  0
    2.5742   -4.1610    3.1836 H   0  0  0  0  0  0  0  0  0  0  0  0
   -1.2544   -5.6545    4.4972 H   0  0  0  0  0  0  0  0  0  0  0  0
   -0.6099   -3.2642   -0.9930 C   0  0  0  0  0  0  0  0  0  0  0  0
   -0.6105   -2.5019   -2.1627 C   0  0  0  0  0  0  0  0  0  0  0  0
   -0.0863   -4.5605   -0.9824 C   0  0  0  0  0  0  0  0  0  0  0  0
   -0.0556   -3.0353   -3.3333 C   0  0  0  0  0  0  0  0  0  0  0  0
   -1.0298   -1.4912   -2.2029 H   0  0  0  0  0  0  0  0  0  0  0  0
    0.4515   -5.0931   -2.1595 C   0  0  0  0  0  0  0  0  0  0  0  0
   -0.0772   -5.1711   -0.0750 H   0  0  0  0  0  0  0  0  0  0  0  0
    0.4722   -4.3298   -3.3321 C   0  0  0  0  0  0  0  0  0  0  0  0
   -0.0288   -2.4310   -4.2415 H   0  0  0  0  0  0  0  0  0  0  0  0
    0.8585   -6.1037   -2.1602 H   0  0  0  0  0  0  0  0  0  0  0  0
   -3.1130   -3.1195    0.5765 C   0  0  0  0  0  0  0  0  0  0  0  0
   -3.5268   -4.1265   -0.2967 C   0  0  0  0  0  0  0  0  0  0  0  0
   -4.0041   -2.5503    1.4915 C   0  0  0  0  0  0  0  0  0  0  0  0
   -4.8528   -4.5756   -0.2480 C   0  0  0  0  0  0  0  0  0  0  0  0
   -2.8472   -4.5778   -1.0248 H   0  0  0  0  0  0  0  0  0  0  0  0
   -5.3254   -3.0082    1.5375 C   0  0  0  0  0  0  0  0  0  0  0  0
   -3.7019   -1.7562    2.1808 H   0  0  0  0  0  0  0  0  0  0  0  0
   -5.7494   -4.0204    0.6689 C   0  0  0  0  0  0  0  0  0  0  0  0
```

```
   -5.1832   -5.3607   -0.9279 H   0  0  0  0  0  0  0  0  0  0  0  0
   -6.0264   -2.5741    2.2505 H   0  0  0  0  0  0  0  0  0  0  0  0
   -0.1601    0.0459    2.0363 C   0  0  0  0  0  0  0  0  0  0  0  0
    0.5303   -0.0038    2.9735 O   0  0  0  0  0  0  0  0  0  0  0  0
    0.4462    4.6021   -4.5792 H   0  0  0  0  0  0  0  0  0  0  0  0
    1.7172    0.1954   -6.3484 H   0  0  0  0  0  0  0  0  0  0  0  0
    0.9010   -4.7438   -4.2452 H   0  0  0  0  0  0  0  0  0  0  0  0
    4.9776   -4.5847    0.8056 H   0  0  0  0  0  0  0  0  0  0  0  0
    1.2220   -5.5675    4.7386 H   0  0  0  0  0  0  0  0  0  0  0  0
    2.0583    5.8579    4.4764 H   0  0  0  0  0  0  0  0  0  0  0  0
   -6.3526    4.9666    1.4713 H   0  0  0  0  0  0  0  0  0  0  0  0
   -6.7806   -4.3737    0.7070 H   0  0  0  0  0  0  0  0  0  0  0  0
    5.1110    4.5478    0.9285 H   0  0  0  0  0  0  0  0  0  0  0  0
   -1.0527    0.1375    0.7692 Ir  0  0  0  0  0  0  0  0  0  0  0  0
  1106  1  0  0  0  0
  2  5  1  0  0  0  0
  2 15  1  0  0  0  0
  2 25  1  0  0  0  0
  2106  1  0  0  0  0
  3 65  1  0  0  0  0
  3 75  1  0  0  0  0
  3 85  1  0  0  0  0
  3106  1  0  0  0  0
  4 35  1  0  0  0  0
  4 45  1  0  0  0  0
  4 55  1  0  0  0  0
  4106  1  0  0  0  0
  5  6  2  0  0  0  0
  5  7  1  0  0  0  0
  6  8  1  0  0  0  0
  6  9  1  0  0  0  0
  7 10  2  0  0  0  0
  7 11  1  0  0  0  0
  8 12  2  0  0  0  0
  8 13  1  0  0  0  0
 10 12  1  0  0  0  0
 10 14  1  0  0  0  0
 12105  1  0  0  0  0
 15 16  2  0  0  0  0
 15 17  1  0  0  0  0
 16 18  1  0  0  0  0
 16 19  1  0  0  0  0
 17 20  2  0  0  0  0
 17 21  1  0  0  0  0
 18 22  2  0  0  0  0
 18 23  1  0  0  0  0
 20 22  1  0  0  0  0
 20 24  1  0  0  0  0
 22100  1  0  0  0  0
 25 26  2  0  0  0  0
 25 27  1  0  0  0  0
 26 28  1  0  0  0  0
 26 29  1  0  0  0  0
 27 30  2  0  0  0  0
 27 31  1  0  0  0  0
 28 32  2  0  0  0  0
 28 33  1  0  0  0  0
 30 32  1  0  0  0  0
 30 34  1  0  0  0  0
 32 98  1  0  0  0  0
 35 36  2  0  0  0  0
 35 37  1  0  0  0  0
 36 38  1  0  0  0  0
 36 39  1  0  0  0  0
 37 40  2  0  0  0  0
```

```
 37 41  1  0  0  0  0
 38 42  2  0  0  0  0
 38 43  1  0  0  0  0
 40 42  1  0  0  0  0
 40 44  1  0  0  0  0
 42102  1  0  0  0  0
 45 46  2  0  0  0  0
 45 47  1  0  0  0  0
 46 48  1  0  0  0  0
 46 49  1  0  0  0  0
 47 50  2  0  0  0  0
 47 51  1  0  0  0  0
 48 52  2  0  0  0  0
 48 53  1  0  0  0  0
 50 52  1  0  0  0  0
 50 54  1  0  0  0  0
 52103  1  0  0  0  0
 55 56  2  0  0  0  0
 55 57  1  0  0  0  0
 56 58  1  0  0  0  0
 56 59  1  0  0  0  0
 57 60  2  0  0  0  0
 57 61  1  0  0  0  0
 58 62  2  0  0  0  0
 58 63  1  0  0  0  0
 60 62  1  0  0  0  0
 60 64  1  0  0  0  0
 62 97  1  0  0  0  0
 65 66  2  0  0  0  0
 65 67  1  0  0  0  0
 66 68  1  0  0  0  0
 66 69  1  0  0  0  0
 67 70  2  0  0  0  0
 67 71  1  0  0  0  0
 68 72  2  0  0  0  0
 68 73  1  0  0  0  0
 70 72  1  0  0  0  0
 70 74  1  0  0  0  0
 72101  1  0  0  0  0
 75 76  2  0  0  0  0
 75 77  1  0  0  0  0
 76 78  1  0  0  0  0
 76 79  1  0  0  0  0
 77 80  2  0  0  0  0
 77 81  1  0  0  0  0
 78 82  2  0  0  0  0
 78 83  1  0  0  0  0
 80 82  1  0  0  0  0
 80 84  1  0  0  0  0
 82 99  1  0  0  0  0
 85 86  2  0  0  0  0
 85 87  1  0  0  0  0
 86 88  1  0  0  0  0
 86 89  1  0  0  0  0
 87 90  2  0  0  0  0
 87 91  1  0  0  0  0
 88 92  2  0  0  0  0
 88 93  1  0  0  0  0
 90 92  1  0  0  0  0
 90 94  1  0  0  0  0
 92104  1  0  0  0  0
 95 96  2  0  0  0  0
 95106  1  0  0  0  0
M  END
$$$$
```

```
mol055
Optimized Catalyst 055
Optimized Catalyst 055
 19 19  0  0  0  0  0  0  0  0999 V2000
   -1.4511   -0.0435    0.4082 Rh  0  0  0  0  0  0  0  0  0  0  0  0
   -2.4415    0.1806    1.8673 C   0  0  0  0  0  0  0  0  0  0  0  0
   -3.0597    0.4457    2.7941 O   0  0  0  0  0  0  0  0  0  0  0  0
   -1.3331    2.0241    0.6177 O   0  0  0  0  0  0  0  0  0  0  0  0
   -0.5848    2.6114   -0.2237 C   0  0  0  0  0  0  0  0  0  0  0  0
   -0.2975   -0.3086   -1.2992 O   0  0  0  0  0  0  0  0  0  0  0  0
   -0.5078    4.1033    0.0221 C   0  0  0  0  0  0  0  0  0  0  0  0
    0.5483    4.3982    0.2205 H   0  0  0  0  0  0  0  0  0  0  0  0
   -0.8853    4.6520   -0.8708 H   0  0  0  0  0  0  0  0  0  0  0  0
   -1.1342    4.3732    0.9072 H   0  0  0  0  0  0  0  0  0  0  0  0
    0.1372    2.0675   -1.2980 C   0  0  0  0  0  0  0  0  0  0  0  0
    0.7114    2.7965   -1.8766 H   0  0  0  0  0  0  0  0  0  0  0  0
    0.2381    0.7469   -1.7614 C   0  0  0  0  0  0  0  0  0  0  0  0
    1.0873    0.4660   -2.9827 C   0  0  0  0  0  0  0  0  0  0  0  0
    2.1375    0.7860   -2.7941 H   0  0  0  0  0  0  0  0  0  0  0  0
    1.0760   -0.6286   -3.2072 H   0  0  0  0  0  0  0  0  0  0  0  0
    0.6809    1.0206   -3.8597 H   0  0  0  0  0  0  0  0  0  0  0  0
   -1.5302   -1.8129    0.2474 C   0  0  0  0  0  0  0  0  0  0  0  0
   -1.5103   -2.9421    0.0575 O   0  0  0  0  0  0  0  0  0  0  0  0
  1  2  1  0  0  0  0
  1 18  1  0  0  0  0
  1  6  1  0  0  0  0
  1  4  1  0  0  0  0
  2  3  3  0  0  0  0
  4  5  2  0  0  0  0
  5  7  1  0  0  0  0
  5 11  1  0  0  0  0
  6 13  2  0  0  0  0
  7  8  1  0  0  0  0
  7  9  1  0  0  0  0
  7 10  1  0  0  0  0
 11 12  1  0  0  0  0
 11 13  1  0  0  0  0
 13 14  1  0  0  0  0
 14 15  1  0  0  0  0
 14 16  1  0  0  0  0
 14 17  1  0  0  0  0
 18 19  3  0  0  0  0
M  END
$$$$
mol059
mol059
Optimized Catalyst 059
 51 54  0  0  0  0  0  0  0  0  0    0
    5.8676    0.2147    0.6862 C   0  0  0  0  0  0  0  0  0  0  0  0
    6.0388    1.6249    0.4263 C   0  0  0  0  0  0  0  0  0  0  0  0
    5.1838    2.6789    0.6355 C   0  0  0  0  0  0  0  0  0  0  0  0
    7.0341    1.8824    0.0565 H   0  0  0  0  0  0  0  0  0  0  0  0
    3.0157    0.9450    0.8095 Rh  0  0  0  0  0  0  0  0  0  0  0  0
    4.8096   -0.4219    0.7581 O   0  0  0  0  0  0  0  0  0  0  0  0
    3.9687    2.6953    1.1283 O   0  0  0  0  0  0  0  0  0  0  0  0
    7.1460   -0.5843    0.8553 C   0  0  0  0  0  0  0  0  0  0  0  0
    6.8904   -1.6376    1.1208 H   0  0  0  0  0  0  0  0  0  0  0  0
    7.7265   -0.5796   -0.0931 H   0  0  0  0  0  0  0  0  0  0  0  0
    7.7620   -0.1409    1.6689 H   0  0  0  0  0  0  0  0  0  0  0  0
    5.6263    4.1013    0.3634 C   0  0  0  0  0  0  0  0  0  0  0  0
    6.5714    4.3205    0.9079 H   0  0  0  0  0  0  0  0  0  0  0  0
    5.7813    4.2332   -0.7322 H   0  0  0  0  0  0  0  0  0  0  0  0
    4.8313    4.8092    0.7046 H   0  0  0  0  0  0  0  0  0  0  0  0
    2.0350   -0.5050    0.4362 C   0  0  0  0  0  0  0  0  0  0  0  0
    1.5391   -1.5131    0.2215 O   0  0  0  0  0  0  0  0  0  0  0  0
```

```
    1.6982    2.5729    0.4236 P   0  0  0  0  0  0  0  0  0  0  0  0
    2.3225    3.5453   -1.0322 C   0  0  0  0  0  0  0  0  0  0  0  0
    1.9730    4.8764   -1.2549 C   0  0  0  0  0  0  0  0  0  0  0  0
    3.1722    2.8583   -1.9060 C   0  0  0  0  0  0  0  0  0  0  0  0
    2.4590    5.5228   -2.3985 C   0  0  0  0  0  0  0  0  0  0  0  0
    1.3396    5.4331   -0.5627 H   0  0  0  0  0  0  0  0  0  0  0  0
    3.6503    3.5108   -3.0475 C   0  0  0  0  0  0  0  0  0  0  0  0
    3.4900    1.8173   -1.6886 H   0  0  0  0  0  0  0  0  0  0  0  0
    3.2883    4.8399   -3.2944 C   0  0  0  0  0  0  0  0  0  0  0  0
    2.1922    6.5619   -2.5879 H   0  0  0  0  0  0  0  0  0  0  0  0
    4.3088    2.9852   -3.7390 H   0  0  0  0  0  0  0  0  0  0  0  0
    3.6598    5.3477   -4.1855 H   0  0  0  0  0  0  0  0  0  0  0  0
    1.4035    3.6957    1.8670 C   0  0  0  0  0  0  0  0  0  0  0  0
    0.3684    4.6352    1.8388 C   0  0  0  0  0  0  0  0  0  0  0  0
    2.2341    3.5588    2.9797 C   0  0  0  0  0  0  0  0  0  0  0  0
    0.1916    5.4806    2.9375 C   0  0  0  0  0  0  0  0  0  0  0  0
   -0.3185    4.7132    0.9936 H   0  0  0  0  0  0  0  0  0  0  0  0
    2.0492    4.4109    4.0774 C   0  0  0  0  0  0  0  0  0  0  0  0
    3.0339    2.7869    3.0000 H   0  0  0  0  0  0  0  0  0  0  0  0
    1.0345    5.3706    4.0520 C   0  0  0  0  0  0  0  0  0  0  0  0
   -0.6042    6.2238    2.9304 H   0  0  0  0  0  0  0  0  0  0  0  0
    2.6977    4.3200    4.9485 H   0  0  0  0  0  0  0  0  0  0  0  0
    0.8917    6.0345    4.9054 H   0  0  0  0  0  0  0  0  0  0  0  0
   -0.0401    2.0872   -0.0660 C   0  0  0  0  0  0  0  0  0  0  0  0
   -0.5454    2.3868   -1.3301 C   0  0  0  0  0  0  0  0  0  0  0  0
   -0.8324    1.4511    0.8949 C   0  0  0  0  0  0  0  0  0  0  0  0
   -1.8691    2.0447   -1.6383 C   0  0  0  0  0  0  0  0  0  0  0  0
    0.0630    2.8800   -2.0935 H   0  0  0  0  0  0  0  0  0  0  0  0
   -2.1532    1.1151    0.5832 C   0  0  0  0  0  0  0  0  0  0  0  0
   -0.4407    1.2104    1.8890 H   0  0  0  0  0  0  0  0  0  0  0  0
   -2.6702    1.4114   -0.6842 C   0  0  0  0  0  0  0  0  0  0  0  0
   -2.2720    2.2732   -2.6249 H   0  0  0  0  0  0  0  0  0  0  0  0
   -2.7798    0.6211    1.3261 H   0  0  0  0  0  0  0  0  0  0  0  0
   -3.6996    1.1445   -0.9270 H   0  0  0  0  0  0  0  0  0  0  0  0
  1  2  4  0  0  0  0
  1  6  2  0  0  0  0
  1  8  1  0  0  0  0
  2  3  2  0  0  0  0
  2  4  1  0  0  0  0
  3  7  4  0  0  0  0
  3 12  1  0  0  0  0
  5  7  1  0  0  0  0
  5 16  1  0  0  0  0
  5 18  1  0  0  0  0
  5  6  1  0  0  0  0
  8  9  1  0  0  0  0
  8 10  1  0  0  0  0
  8 11  1  0  0  0  0
 12 13  1  0  0  0  0
 12 14  1  0  0  0  0
 12 15  1  0  0  0  0
 16 17  3  0  0  0  0
 18 19  1  0  0  0  0
 18 30  1  0  0  0  0
 18 41  1  0  0  0  0
 19 20  4  0  0  0  0
 19 21  4  0  0  0  0
 20 22  4  0  0  0  0
 20 23  1  0  0  0  0
 21 24  4  0  0  0  0
 21 25  1  0  0  0  0
 22 26  4  0  0  0  0
 22 27  1  0  0  0  0
 24 26  4  0  0  0  0
 24 28  1  0  0  0  0
```

```
 26 29  1  0  0  0  0
 30 31  4  0  0  0  0
 30 32  4  0  0  0  0
 31 33  4  0  0  0  0
 31 34  1  0  0  0  0
 32 35  4  0  0  0  0
 32 36  1  0  0  0  0
 33 37  4  0  0  0  0
 33 38  1  0  0  0  0
 35 37  4  0  0  0  0
 35 39  1  0  0  0  0
 37 40  1  0  0  0  0
 41 42  4  0  0  0  0
 41 43  4  0  0  0  0
 42 44  4  0  0  0  0
 42 45  1  0  0  0  0
 43 46  4  0  0  0  0
 43 47  1  0  0  0  0
 44 48  4  0  0  0  0
 44 49  1  0  0  0  0
 46 48  4  0  0  0  0
 46 50  1  0  0  0  0
 48 51  1  0  0  0  0
M  END
$$$$
mol026
Optimized Catalyst 026
Optimized Catalyst 026
 89 98  0  0  0  0  0  0  0  0999 V2000
    0.8803   -0.4107    0.3073 C   0  0  0  0  0  0  0  0  0  0  0  0
   -0.3591    0.3258    0.8511 C   0  0  0  0  0  0  0  0  0  0  0  0
    0.5676   -1.7111   -0.3725 C   0  0  0  0  0  0  0  0  0  0  0  0
    0.1456   -0.7660   -2.7126 C   0  0  0  0  0  0  0  0  0  0  0  0
    0.2353   -1.8545   -1.6735 C   0  0  0  0  0  0  0  0  0  0  0  0
   -0.4970    0.5680   -2.2828 C   0  0  0  0  0  0  0  0  0  0  0  0
    0.7030   -2.6003    0.2633 H   0  0  0  0  0  0  0  0  0  0  0  0
    0.1734   -2.8713   -2.1079 H   0  0  0  0  0  0  0  0  0  0  0  0
    1.5701   -0.6081    1.1716 H   0  0  0  0  0  0  0  0  0  0  0  0
    1.4394    0.2504   -0.4048 H   0  0  0  0  0  0  0  0  0  0  0  0
   -0.6214   -0.1247    1.8436 H   0  0  0  0  0  0  0  0  0  0  0  0
   -0.8318    1.1058   -3.2101 H   0  0  0  0  0  0  0  0  0  0  0  0
   -0.4138   -1.1565   -3.6036 H   0  0  0  0  0  0  0  0  0  0  0  0
    1.1994   -0.5500   -3.0501 H   0  0  0  0  0  0  0  0  0  0  0  0
   -1.6687    0.4283   -1.3519 C   0  0  0  0  0  0  0  0  0  0  0  0
   -1.5927    0.3454   -0.0074 C   0  0  0  0  0  0  0  0  0  0  0  0
   -2.6523    0.5341   -1.8393 H   0  0  0  0  0  0  0  0  0  0  0  0
   -2.5233    0.3951    0.5871 H   0  0  0  0  0  0  0  0  0  0  0  0
   -0.0706    1.4007    1.0318 H   0  0  0  0  0  0  0  0  0  0  0  0
    0.2718    1.2224   -1.7950 H   0  0  0  0  0  0  0  0  0  0  0  0
   -4.9861   -2.2263   -0.7118 P   0  0  0  0  0  0  0  0  0  0  0  0
   -2.2599   -4.8995   -0.3488 P   0  0  0  0  0  0  0  0  0  0  0  0
   -0.4965   -5.4054   -0.1321 C   0  0  0  0  0  0  0  0  0  0  0  0
    0.2498   -5.6966   -1.2825 C   0  0  0  0  0  0  0  0  0  0  0  0
    0.0523   -5.5632    1.1440 C   0  0  0  0  0  0  0  0  0  0  0  0
    1.5675   -6.1398   -1.1467 C   0  0  0  0  0  0  0  0  0  0  0  0
   -0.1690   -5.6038   -2.2894 H   0  0  0  0  0  0  0  0  0  0  0  0
    1.3732   -6.0097    1.2674 C   0  0  0  0  0  0  0  0  0  0  0  0
   -0.5176   -5.3629    2.0559 H   0  0  0  0  0  0  0  0  0  0  0  0
    2.1298   -6.2971    0.1268 C   0  0  0  0  0  0  0  0  0  0  0  0
    2.1600   -6.3714   -2.0358 H   0  0  0  0  0  0  0  0  0  0  0  0
    1.8123   -6.1422    2.2596 H   0  0  0  0  0  0  0  0  0  0  0  0
    3.1609   -6.6498    0.2278 H   0  0  0  0  0  0  0  0  0  0  0  0
   -2.9384   -6.0180   -1.6557 C   0  0  0  0  0  0  0  0  0  0  0  0
   -2.8530   -7.4003   -1.4610 C   0  0  0  0  0  0  0  0  0  0  0  0
   -3.4686   -5.4844   -2.8338 C   0  0  0  0  0  0  0  0  0  0  0  0
```

```
  -3.3576   -8.2588   -2.4448 C   0  0  0  0  0  0  0  0  0  0  0  0
  -2.3869   -7.8385   -0.5727 H   0  0  0  0  0  0  0  0  0  0  0  0
  -3.9661   -6.3510   -3.8126 C   0  0  0  0  0  0  0  0  0  0  0  0
  -3.4922   -4.4054   -3.0333 H   0  0  0  0  0  0  0  0  0  0  0  0
  -3.9189   -7.7365   -3.6144 C   0  0  0  0  0  0  0  0  0  0  0  0
  -3.3024   -9.3414   -2.3022 H   0  0  0  0  0  0  0  0  0  0  0  0
  -4.3832   -5.9482   -4.7387 H   0  0  0  0  0  0  0  0  0  0  0  0
  -4.3113   -8.4114   -4.3814 H   0  0  0  0  0  0  0  0  0  0  0  0
  -3.1364   -5.1461    1.2618 C   0  0  0  0  0  0  0  0  0  0  0  0
  -3.9816   -6.2348    1.4694 C   0  0  0  0  0  0  0  0  0  0  0  0
  -2.8912   -4.2156    2.2835 C   0  0  0  0  0  0  0  0  0  0  0  0
  -4.5676   -6.4145    2.7318 C   0  0  0  0  0  0  0  0  0  0  0  0
  -4.2242   -6.9497    0.6786 H   0  0  0  0  0  0  0  0  0  0  0  0
  -3.4840   -4.3995    3.5331 C   0  0  0  0  0  0  0  0  0  0  0  0
  -2.2388   -3.3454    2.1376 H   0  0  0  0  0  0  0  0  0  0  0  0
  -4.3167   -5.5058    3.7607 C   0  0  0  0  0  0  0  0  0  0  0  0
  -5.2226   -7.2715    2.9058 H   0  0  0  0  0  0  0  0  0  0  0  0
  -3.2946   -3.6890    4.3409 H   0  0  0  0  0  0  0  0  0  0  0  0
  -4.7685   -5.6529    4.7460 H   0  0  0  0  0  0  0  0  0  0  0  0
  -5.3511   -0.7630    0.3629 C   0  0  0  0  0  0  0  0  0  0  0  0
  -6.1891    0.2458   -0.1165 C   0  0  0  0  0  0  0  0  0  0  0  0
  -4.8155   -0.7043    1.6576 C   0  0  0  0  0  0  0  0  0  0  0  0
  -6.5056    1.3268    0.7180 C   0  0  0  0  0  0  0  0  0  0  0  0
  -6.6103    0.2271   -1.1270 H   0  0  0  0  0  0  0  0  0  0  0  0
  -5.1364    0.3776    2.4798 C   0  0  0  0  0  0  0  0  0  0  0  0
  -4.1545   -1.4806    2.0530 H   0  0  0  0  0  0  0  0  0  0  0  0
  -5.9836    1.3927    2.0114 C   0  0  0  0  0  0  0  0  0  0  0  0
  -7.1659    2.1183    0.3528 H   0  0  0  0  0  0  0  0  0  0  0  0
  -4.7306    0.4364    3.4924 H   0  0  0  0  0  0  0  0  0  0  0  0
  -6.2338    2.2367    2.6612 H   0  0  0  0  0  0  0  0  0  0  0  0
  -5.6782   -1.8908   -2.3991 C   0  0  0  0  0  0  0  0  0  0  0  0
  -7.0576   -2.0020   -2.5982 C   0  0  0  0  0  0  0  0  0  0  0  0
  -4.8237   -1.5192   -3.4421 C   0  0  0  0  0  0  0  0  0  0  0  0
  -7.5878   -1.7241   -3.8639 C   0  0  0  0  0  0  0  0  0  0  0  0
  -7.7394   -2.3030   -1.7963 H   0  0  0  0  0  0  0  0  0  0  0  0
  -5.3628   -1.2410   -4.7017 C   0  0  0  0  0  0  0  0  0  0  0  0
  -3.7328   -1.4412   -3.3089 H   0  0  0  0  0  0  0  0  0  0  0  0
  -6.7444   -1.3415   -4.9115 C   0  0  0  0  0  0  0  0  0  0  0  0
  -8.6649   -1.8067   -4.0330 H   0  0  0  0  0  0  0  0  0  0  0  0
  -4.7085   -0.9440   -5.5252 H   0  0  0  0  0  0  0  0  0  0  0  0
  -7.1627   -1.1218   -5.8986 H   0  0  0  0  0  0  0  0  0  0  0  0
  -5.9306   -3.6755   -0.0591 C   0  0  0  0  0  0  0  0  0  0  0  0
  -6.4466   -3.6625    1.2393 C   0  0  0  0  0  0  0  0  0  0  0  0
  -6.1900   -4.7455   -0.9259 C   0  0  0  0  0  0  0  0  0  0  0  0
  -7.2249   -4.7415    1.6774 C   0  0  0  0  0  0  0  0  0  0  0  0
  -6.2746   -2.8333    1.9312 H   0  0  0  0  0  0  0  0  0  0  0  0
  -6.9645   -5.8182   -0.4783 C   0  0  0  0  0  0  0  0  0  0  0  0
  -5.8222   -4.7658   -1.9557 H   0  0  0  0  0  0  0  0  0  0  0  0
  -7.4856   -5.8155    0.8219 C   0  0  0  0  0  0  0  0  0  0  0  0
  -7.6343   -4.7386    2.6901 H   0  0  0  0  0  0  0  0  0  0  0  0
  -7.1747   -6.6549   -1.1481 H   0  0  0  0  0  0  0  0  0  0  0  0
  -8.1022   -6.6509    1.1641 H   0  0  0  0  0  0  0  0  0  0  0  0
  -2.3916   -2.3764   -0.9611 Ir  0  0  0  0  0  0  0  0  0  0  0  0
  1  2  1  0  0  0  0
  1  3  1  0  0  0  0
  1  9  1  0  0  0  0
  1 10  1  0  0  0  0
  2 11  1  0  0  0  0
  2 16  1  0  0  0  0
  2 19  1  0  0  0  0
  3  5  2  0  0  0  0
  3  7  1  0  0  0  0
  3 89  1  0  0  0  0
  4  5  1  0  0  0  0
  4  6  1  0  0  0  0
```

```
  4 13  1  0  0  0  0
  4 14  1  0  0  0  0
  5  8  1  0  0  0  0
  5 89  1  0  0  0  0
  6 12  1  0  0  0  0
  6 15  1  0  0  0  0
  6 20  1  0  0  0  0
 15 16  2  0  0  0  0
 15 17  1  0  0  0  0
 15 89  1  0  0  0  0
 16 18  1  0  0  0  0
 16 89  1  0  0  0  0
 21 56  1  0  0  0  0
 21 67  1  0  0  0  0
 21 78  1  0  0  0  0
 21 89  1  0  0  0  0
 22 23  1  0  0  0  0
 22 34  1  0  0  0  0
 22 45  1  0  0  0  0
 22 89  1  0  0  0  0
 23 24  2  0  0  0  0
 23 25  1  0  0  0  0
 24 26  1  0  0  0  0
 24 27  1  0  0  0  0
 25 28  2  0  0  0  0
 25 29  1  0  0  0  0
 26 30  2  0  0  0  0
 26 31  1  0  0  0  0
 28 30  1  0  0  0  0
 28 32  1  0  0  0  0
 30 33  1  0  0  0  0
 34 35  2  0  0  0  0
 34 36  1  0  0  0  0
 35 37  1  0  0  0  0
 35 38  1  0  0  0  0
 36 39  2  0  0  0  0
 36 40  1  0  0  0  0
 37 41  2  0  0  0  0
 37 42  1  0  0  0  0
 39 41  1  0  0  0  0
 39 43  1  0  0  0  0
 41 44  1  0  0  0  0
 45 46  2  0  0  0  0
 45 47  1  0  0  0  0
 46 48  1  0  0  0  0
 46 49  1  0  0  0  0
 47 50  2  0  0  0  0
 47 51  1  0  0  0  0
 48 52  2  0  0  0  0
 48 53  1  0  0  0  0
 50 52  1  0  0  0  0
 50 54  1  0  0  0  0
 52 55  1  0  0  0  0
 56 57  2  0  0  0  0
 56 58  1  0  0  0  0
 57 59  1  0  0  0  0
 57 60  1  0  0  0  0
 58 61  2  0  0  0  0
 58 62  1  0  0  0  0
 59 63  2  0  0  0  0
 59 64  1  0  0  0  0
 61 63  1  0  0  0  0
 61 65  1  0  0  0  0
 63 66  1  0  0  0  0
 67 68  2  0  0  0  0
```

```
 67 69  1  0  0  0  0
 68 70  1  0  0  0  0
 68 71  1  0  0  0  0
 69 72  2  0  0  0  0
 69 73  1  0  0  0  0
 70 74  2  0  0  0  0
 70 75  1  0  0  0  0
 72 74  1  0  0  0  0
 72 76  1  0  0  0  0
 74 77  1  0  0  0  0
 78 79  2  0  0  0  0
 78 80  1  0  0  0  0
 79 81  1  0  0  0  0
 79 82  1  0  0  0  0
 80 83  2  0  0  0  0
 80 84  1  0  0  0  0
 81 85  2  0  0  0  0
 81 86  1  0  0  0  0
 83 85  1  0  0  0  0
 83 87  1  0  0  0  0
 85 88  1  0  0  0  0
M  END
$$$$
mol027
Optimized Catalyst 027
Optimized Catalyst 027
 89 98  0  0  0  0  0  0  0  0999 V2000
    0.8387   -0.3882    0.3181 C   0  0  0  0  0  0  0  0  0  0  0  0
   -0.4082    0.3616    0.8233 C   0  0  0  0  0  0  0  0  0  0  0  0
    0.5407   -1.7080   -0.3309 C   0  0  0  0  0  0  0  0  0  0  0  0
    0.1589   -0.8257   -2.7016 C   0  0  0  0  0  0  0  0  0  0  0  0
    0.2310   -1.8875   -1.6341 C   0  0  0  0  0  0  0  0  0  0  0  0
   -0.4901    0.5194   -2.3201 C   0  0  0  0  0  0  0  0  0  0  0  0
    0.6737   -2.5795    0.3299 H   0  0  0  0  0  0  0  0  0  0  0  0
    0.1894   -2.9143   -2.0463 H   0  0  0  0  0  0  0  0  0  0  0  0
    1.5183   -0.5575    1.1971 H   0  0  0  0  0  0  0  0  0  0  0  0
    1.4064    0.2546   -0.4050 H   0  0  0  0  0  0  0  0  0  0  0  0
   -0.6850   -0.0561    1.8266 H   0  0  0  0  0  0  0  0  0  0  0  0
   -0.8075    1.0289   -3.2699 H   0  0  0  0  0  0  0  0  0  0  0  0
   -0.3822   -1.2377   -3.5944 H   0  0  0  0  0  0  0  0  0  0  0  0
    1.2206   -0.6198   -3.0233 H   0  0  0  0  0  0  0  0  0  0  0  0
   -1.6779    0.4116   -1.4050 C   0  0  0  0  0  0  0  0  0  0  0  0
   -1.6269    0.3613   -0.0567 C   0  0  0  0  0  0  0  0  0  0  0  0
   -2.6512    0.5213   -1.9114 H   0  0  0  0  0  0  0  0  0  0  0  0
   -2.5666    0.4435    0.5201 H   0  0  0  0  0  0  0  0  0  0  0  0
   -0.1221    1.4428    0.9761 H   0  0  0  0  0  0  0  0  0  0  0  0
    0.2711    1.1899   -1.8409 H   0  0  0  0  0  0  0  0  0  0  0  0
   -4.9672   -2.1872   -0.6695 P   0  0  0  0  0  0  0  0  0  0  0  0
   -2.2620   -4.9168   -0.3827 P   0  0  0  0  0  0  0  0  0  0  0  0
   -0.5047   -5.4208   -0.1371 C   0  0  0  0  0  0  0  0  0  0  0  0
    0.2671   -5.6978   -1.2776 C   0  0  0  0  0  0  0  0  0  0  0  0
    0.0172   -5.5909    1.1517 C   0  0  0  0  0  0  0  0  0  0  0  0
    1.5851   -6.1263   -1.1341 C   0  0  0  0  0  0  0  0  0  0  0  0
   -0.1374   -5.6007   -2.2917 H   0  0  0  0  0  0  0  0  0  0  0  0
    1.3356   -6.0207    1.3124 C   0  0  0  0  0  0  0  0  0  0  0  0
   -0.5799   -5.4075    2.0516 H   0  0  0  0  0  0  0  0  0  0  0  0
    2.0951   -6.2780    0.1641 C   0  0  0  0  0  0  0  0  0  0  0  0
    2.2043   -6.3479   -2.0093 H   0  0  0  0  0  0  0  0  0  0  0  0
    1.7616   -6.1634    2.3106 H   0  0  0  0  0  0  0  0  0  0  0  0
   -2.9044   -6.0187   -1.7163 C   0  0  0  0  0  0  0  0  0  0  0  0
   -2.7362   -7.4029   -1.5633 C   0  0  0  0  0  0  0  0  0  0  0  0
   -3.4920   -5.4816   -2.8670 C   0  0  0  0  0  0  0  0  0  0  0  0
   -3.2134   -8.2701   -2.5467 C   0  0  0  0  0  0  0  0  0  0  0  0
   -2.2217   -7.8351   -0.6967 H   0  0  0  0  0  0  0  0  0  0  0  0
   -3.9728   -6.3339   -3.8621 C   0  0  0  0  0  0  0  0  0  0  0  0
```

```
   -3.5736   -4.3994   -3.0319 H   0  0  0  0  0  0  0  0  0  0  0  0
   -3.8305   -7.7162   -3.6756 C   0  0  0  0  0  0  0  0  0  0  0  0
   -3.0971   -9.3543   -2.4442 H   0  0  0  0  0  0  0  0  0  0  0  0
   -4.4350   -5.9369   -4.7708 H   0  0  0  0  0  0  0  0  0  0  0  0
   -3.1705   -5.2206    1.1981 C   0  0  0  0  0  0  0  0  0  0  0  0
   -3.9705   -6.3532    1.3734 C   0  0  0  0  0  0  0  0  0  0  0  0
   -3.0005   -4.2848    2.2317 C   0  0  0  0  0  0  0  0  0  0  0  0
   -4.6021   -6.5723    2.6024 C   0  0  0  0  0  0  0  0  0  0  0  0
   -4.1414   -7.0795    0.5721 H   0  0  0  0  0  0  0  0  0  0  0  0
   -3.6264   -4.4828    3.4597 C   0  0  0  0  0  0  0  0  0  0  0  0
   -2.3770   -3.3880    2.1062 H   0  0  0  0  0  0  0  0  0  0  0  0
   -4.4148   -5.6338    3.6211 C   0  0  0  0  0  0  0  0  0  0  0  0
   -5.2256   -7.4583    2.7610 H   0  0  0  0  0  0  0  0  0  0  0  0
   -3.5001   -3.7722    4.2812 H   0  0  0  0  0  0  0  0  0  0  0  0
   -5.3239   -0.7055    0.3770 C   0  0  0  0  0  0  0  0  0  0  0  0
   -6.1693    0.2977   -0.1117 C   0  0  0  0  0  0  0  0  0  0  0  0
   -4.7751   -0.6266    1.6672 C   0  0  0  0  0  0  0  0  0  0  0  0
   -6.4852    1.3937    0.6963 C   0  0  0  0  0  0  0  0  0  0  0  0
   -6.5977    0.2608   -1.1204 H   0  0  0  0  0  0  0  0  0  0  0  0
   -5.0783    0.4595    2.4850 C   0  0  0  0  0  0  0  0  0  0  0  0
   -4.1069   -1.3980    2.0647 H   0  0  0  0  0  0  0  0  0  0  0  0
   -5.9360    1.4508    1.9812 C   0  0  0  0  0  0  0  0  0  0  0  0
   -7.1486    2.1852    0.3322 H   0  0  0  0  0  0  0  0  0  0  0  0
   -4.6648    0.5406    3.4943 H   0  0  0  0  0  0  0  0  0  0  0  0
   -5.6747   -1.8861   -2.3513 C   0  0  0  0  0  0  0  0  0  0  0  0
   -7.0607   -1.9982   -2.5284 C   0  0  0  0  0  0  0  0  0  0  0  0
   -4.8315   -1.5354   -3.4131 C   0  0  0  0  0  0  0  0  0  0  0  0
   -7.6194   -1.7442   -3.7821 C   0  0  0  0  0  0  0  0  0  0  0  0
   -7.7296   -2.2845   -1.7081 H   0  0  0  0  0  0  0  0  0  0  0  0
   -5.3724   -1.2762   -4.6721 C   0  0  0  0  0  0  0  0  0  0  0  0
   -3.7381   -1.4605   -3.2903 H   0  0  0  0  0  0  0  0  0  0  0  0
   -6.7625   -1.3832   -4.8293 C   0  0  0  0  0  0  0  0  0  0  0  0
   -8.6997   -1.8240   -3.9422 H   0  0  0  0  0  0  0  0  0  0  0  0
   -4.7345   -0.9941   -5.5151 H   0  0  0  0  0  0  0  0  0  0  0  0
   -5.9146   -3.6166    0.0143 C   0  0  0  0  0  0  0  0  0  0  0  0
   -6.4354   -3.5700    1.3131 C   0  0  0  0  0  0  0  0  0  0  0  0
   -6.1700   -4.7090   -0.8289 C   0  0  0  0  0  0  0  0  0  0  0  0
   -7.2029   -4.6346    1.7917 C   0  0  0  0  0  0  0  0  0  0  0  0
   -6.2695   -2.7172    1.9795 H   0  0  0  0  0  0  0  0  0  0  0  0
   -6.9314   -5.7811   -0.3670 C   0  0  0  0  0  0  0  0  0  0  0  0
   -5.8019   -4.7479   -1.8592 H   0  0  0  0  0  0  0  0  0  0  0  0
   -7.4341   -5.7206    0.9405 C   0  0  0  0  0  0  0  0  0  0  0  0
   -7.6207   -4.6138    2.8028 H   0  0  0  0  0  0  0  0  0  0  0  0
   -7.1457   -6.6375   -1.0119 H   0  0  0  0  0  0  0  0  0  0  0  0
   -2.3738   -2.3690   -0.9044 Ir  0  0  0  0  0  0  0  0  0  0  0  0
   -6.2317    2.4847    2.7573 F   0  0  0  0  0  0  0  0  0  0  0  0
   -5.0204   -5.8255    4.7859 F   0  0  0  0  0  0  0  0  0  0  0  0
    3.3490   -6.6842    0.3090 F   0  0  0  0  0  0  0  0  0  0  0  0
   -4.2938   -8.5330   -4.6120 F   0  0  0  0  0  0  0  0  0  0  0  0
   -7.2851   -1.1372   -6.0230 F   0  0  0  0  0  0  0  0  0  0  0  0
   -8.1516   -6.7420    1.3912 F   0  0  0  0  0  0  0  0  0  0  0  0
  1  2  1  0  0  0  0
  1  3  1  0  0  0  0
  1  9  1  0  0  0  0
  1 10  1  0  0  0  0
  2 11  1  0  0  0  0
  2 16  1  0  0  0  0
  2 19  1  0  0  0  0
  3  5  2  0  0  0  0
  3  7  1  0  0  0  0
  3 83  1  0  0  0  0
  4  5  1  0  0  0  0
  4  6  1  0  0  0  0
  4 13  1  0  0  0  0
  4 14  1  0  0  0  0
```

```
  5  8  1  0  0  0  0
  5 83  1  0  0  0  0
  6 12  1  0  0  0  0
  6 15  1  0  0  0  0
  6 20  1  0  0  0  0
 15 16  2  0  0  0  0
 15 17  1  0  0  0  0
 15 83  1  0  0  0  0
 16 18  1  0  0  0  0
 16 83  1  0  0  0  0
 21 53  1  0  0  0  0
 21 63  1  0  0  0  0
 21 73  1  0  0  0  0
 21 83  1  0  0  0  0
 22 23  1  0  0  0  0
 22 33  1  0  0  0  0
 22 43  1  0  0  0  0
 22 83  1  0  0  0  0
 23 24  2  0  0  0  0
 23 25  1  0  0  0  0
 24 26  1  0  0  0  0
 24 27  1  0  0  0  0
 25 28  2  0  0  0  0
 25 29  1  0  0  0  0
 26 30  2  0  0  0  0
 26 31  1  0  0  0  0
 28 30  1  0  0  0  0
 28 32  1  0  0  0  0
 30 86  1  0  0  0  0
 33 34  2  0  0  0  0
 33 35  1  0  0  0  0
 34 36  1  0  0  0  0
 34 37  1  0  0  0  0
 35 38  2  0  0  0  0
 35 39  1  0  0  0  0
 36 40  2  0  0  0  0
 36 41  1  0  0  0  0
 38 40  1  0  0  0  0
 38 42  1  0  0  0  0
 40 87  1  0  0  0  0
 43 44  2  0  0  0  0
 43 45  1  0  0  0  0
 44 46  1  0  0  0  0
 44 47  1  0  0  0  0
 45 48  2  0  0  0  0
 45 49  1  0  0  0  0
 46 50  2  0  0  0  0
 46 51  1  0  0  0  0
 48 50  1  0  0  0  0
 48 52  1  0  0  0  0
 50 85  1  0  0  0  0
 53 54  2  0  0  0  0
 53 55  1  0  0  0  0
 54 56  1  0  0  0  0
 54 57  1  0  0  0  0
 55 58  2  0  0  0  0
 55 59  1  0  0  0  0
 56 60  2  0  0  0  0
 56 61  1  0  0  0  0
 58 60  1  0  0  0  0
 58 62  1  0  0  0  0
 60 84  1  0  0  0  0
 63 64  2  0  0  0  0
 63 65  1  0  0  0  0
 64 66  1  0  0  0  0
```

```
 64 67  1  0  0  0  0
 65 68  2  0  0  0  0
 65 69  1  0  0  0  0
 66 70  2  0  0  0  0
 66 71  1  0  0  0  0
 68 70  1  0  0  0  0
 68 72  1  0  0  0  0
 70 88  1  0  0  0  0
 73 74  2  0  0  0  0
 73 75  1  0  0  0  0
 74 76  1  0  0  0  0
 74 77  1  0  0  0  0
 75 78  2  0  0  0  0
 75 79  1  0  0  0  0
 76 80  2  0  0  0  0
 76 81  1  0  0  0  0
 78 80  1  0  0  0  0
 78 82  1  0  0  0  0
 80 89  1  0  0  0  0
M  END
$$$$
mol028
Optimized Catalyst 028
Optimized Catalyst 028
107116  0  0  0  0  0  0  0  0999 V2000
    0.8877   -0.3930    0.3693 C   0  0  0  0  0  0  0  0  0  0  0  0
   -0.3643    0.3484    0.8775 C   0  0  0  0  0  0  0  0  0  0  0  0
    0.5879   -1.6930   -0.3179 C   0  0  0  0  0  0  0  0  0  0  0  0
    0.2417   -0.7447   -2.6676 C   0  0  0  0  0  0  0  0  0  0  0  0
    0.2961   -1.8345   -1.6280 C   0  0  0  0  0  0  0  0  0  0  0  0
   -0.4140    0.5898   -2.2579 C   0  0  0  0  0  0  0  0  0  0  0  0
    0.6929   -2.5819    0.3235 H   0  0  0  0  0  0  0  0  0  0  0  0
    0.2323   -2.8508   -2.0632 H   0  0  0  0  0  0  0  0  0  0  0  0
    1.5532   -0.5925    1.2510 H   0  0  0  0  0  0  0  0  0  0  0  0
    1.4662    0.2663   -0.3285 H   0  0  0  0  0  0  0  0  0  0  0  0
   -0.6528   -0.0938    1.8662 H   0  0  0  0  0  0  0  0  0  0  0  0
   -0.7259    1.1223   -3.1957 H   0  0  0  0  0  0  0  0  0  0  0  0
   -0.2909   -1.1338   -3.5753 H   0  0  0  0  0  0  0  0  0  0  0  0
    1.3054   -0.5303   -2.9706 H   0  0  0  0  0  0  0  0  0  0  0  0
   -1.6107    0.4497   -1.3595 C   0  0  0  0  0  0  0  0  0  0  0  0
   -1.5743    0.3627   -0.0143 C   0  0  0  0  0  0  0  0  0  0  0  0
   -2.5795    0.5490   -1.8757 H   0  0  0  0  0  0  0  0  0  0  0  0
   -2.5214    0.4070    0.5532 H   0  0  0  0  0  0  0  0  0  0  0  0
   -0.0809    1.4245    1.0546 H   0  0  0  0  0  0  0  0  0  0  0  0
    0.3399    1.2475   -1.7526 H   0  0  0  0  0  0  0  0  0  0  0  0
   -4.9591   -2.2253   -0.7352 P   0  0  0  0  0  0  0  0  0  0  0  0
   -2.2554   -4.9201   -0.3840 P   0  0  0  0  0  0  0  0  0  0  0  0
   -0.4980   -5.4140   -0.1655 C   0  0  0  0  0  0  0  0  0  0  0  0
    0.2632   -5.7195   -1.3037 C   0  0  0  0  0  0  0  0  0  0  0  0
    0.0525   -5.5462    1.1157 C   0  0  0  0  0  0  0  0  0  0  0  0
    1.5830   -6.1400   -1.1562 C   0  0  0  0  0  0  0  0  0  0  0  0
   -0.1523   -5.6483   -2.3144 H   0  0  0  0  0  0  0  0  0  0  0  0
    1.3733   -5.9678    1.2561 C   0  0  0  0  0  0  0  0  0  0  0  0
   -0.5266   -5.3398    2.0212 H   0  0  0  0  0  0  0  0  0  0  0  0
    2.1558   -6.2697    0.1247 C   0  0  0  0  0  0  0  0  0  0  0  0
    2.1762   -6.3785   -2.0416 H   0  0  0  0  0  0  0  0  0  0  0  0
    1.8039   -6.0752    2.2539 H   0  0  0  0  0  0  0  0  0  0  0  0
   -2.9556   -6.0053   -1.6971 C   0  0  0  0  0  0  0  0  0  0  0  0
   -2.8906   -7.3937   -1.5282 C   0  0  0  0  0  0  0  0  0  0  0  0
   -3.5070   -5.4541   -2.8585 C   0  0  0  0  0  0  0  0  0  0  0  0
   -3.4401   -8.2301   -2.5008 C   0  0  0  0  0  0  0  0  0  0  0  0
   -2.4038   -7.8509   -0.6603 H   0  0  0  0  0  0  0  0  0  0  0  0
   -4.0547   -6.2933   -3.8274 C   0  0  0  0  0  0  0  0  0  0  0  0
   -3.5078   -4.3720   -3.0479 H   0  0  0  0  0  0  0  0  0  0  0  0
   -4.0361   -7.6918   -3.6557 C   0  0  0  0  0  0  0  0  0  0  0  0
```

```
   -3.3940   -9.3131   -2.3689 H   0  0  0  0  0  0  0  0  0  0  0  0
   -4.4898   -5.8666   -4.7332 H   0  0  0  0  0  0  0  0  0  0  0  0
   -3.1313   -5.1575    1.2208 C   0  0  0  0  0  0  0  0  0  0  0  0
   -3.9732   -6.2489    1.4424 C   0  0  0  0  0  0  0  0  0  0  0  0
   -2.8973   -4.2192    2.2381 C   0  0  0  0  0  0  0  0  0  0  0  0
   -4.5557   -6.4240    2.7015 C   0  0  0  0  0  0  0  0  0  0  0  0
   -4.2139   -6.9692    0.6554 H   0  0  0  0  0  0  0  0  0  0  0  0
   -3.4845   -4.3936    3.4873 C   0  0  0  0  0  0  0  0  0  0  0  0
   -2.2558   -3.3410    2.0808 H   0  0  0  0  0  0  0  0  0  0  0  0
   -4.3107   -5.5096    3.7387 C   0  0  0  0  0  0  0  0  0  0  0  0
   -5.2132   -7.2776    2.8745 H   0  0  0  0  0  0  0  0  0  0  0  0
   -3.3009   -3.6680    4.2812 H   0  0  0  0  0  0  0  0  0  0  0  0
   -5.3157   -0.7558    0.3208 C   0  0  0  0  0  0  0  0  0  0  0  0
   -6.1470    0.2583   -0.1679 C   0  0  0  0  0  0  0  0  0  0  0  0
   -4.7997   -0.6892    1.6229 C   0  0  0  0  0  0  0  0  0  0  0  0
   -6.4796    1.3328    0.6585 C   0  0  0  0  0  0  0  0  0  0  0  0
   -6.5493    0.2400   -1.1868 H   0  0  0  0  0  0  0  0  0  0  0  0
   -5.1350    0.3857    2.4427 C   0  0  0  0  0  0  0  0  0  0  0  0
   -4.1356   -1.4608    2.0256 H   0  0  0  0  0  0  0  0  0  0  0  0
   -5.9861    1.4072    1.9733 C   0  0  0  0  0  0  0  0  0  0  0  0
   -7.1334    2.1220    0.2806 H   0  0  0  0  0  0  0  0  0  0  0  0
   -4.7387    0.4373    3.4580 H   0  0  0  0  0  0  0  0  0  0  0  0
   -5.6274   -1.9080   -2.4263 C   0  0  0  0  0  0  0  0  0  0  0  0
   -7.0056   -2.0219   -2.6427 C   0  0  0  0  0  0  0  0  0  0  0  0
   -4.7695   -1.5330   -3.4664 C   0  0  0  0  0  0  0  0  0  0  0  0
   -7.5266   -1.7429   -3.9066 C   0  0  0  0  0  0  0  0  0  0  0  0
   -7.6924   -2.3253   -1.8454 H   0  0  0  0  0  0  0  0  0  0  0  0
   -5.2942   -1.2549   -4.7269 C   0  0  0  0  0  0  0  0  0  0  0  0
   -3.6787   -1.4533   -3.3223 H   0  0  0  0  0  0  0  0  0  0  0  0
   -6.6810   -1.3526   -4.9613 C   0  0  0  0  0  0  0  0  0  0  0  0
   -8.6023   -1.8237   -4.0762 H   0  0  0  0  0  0  0  0  0  0  0  0
   -4.6293   -0.9532   -5.5385 H   0  0  0  0  0  0  0  0  0  0  0  0
   -5.8887   -3.6665   -0.0663 C   0  0  0  0  0  0  0  0  0  0  0  0
   -6.3931   -3.6465    1.2382 C   0  0  0  0  0  0  0  0  0  0  0  0
   -6.1509   -4.7541   -0.9123 C   0  0  0  0  0  0  0  0  0  0  0  0
   -7.1460   -4.7263    1.7040 C   0  0  0  0  0  0  0  0  0  0  0  0
   -6.2238   -2.8049    1.9161 H   0  0  0  0  0  0  0  0  0  0  0  0
   -6.8929   -5.8333   -0.4398 C   0  0  0  0  0  0  0  0  0  0  0  0
   -5.8007   -4.7812   -1.9488 H   0  0  0  0  0  0  0  0  0  0  0  0
   -7.3994   -5.8326    0.8754 C   0  0  0  0  0  0  0  0  0  0  0  0
   -7.5426   -4.7049    2.7196 H   0  0  0  0  0  0  0  0  0  0  0  0
   -7.0943   -6.6808   -1.0968 H   0  0  0  0  0  0  0  0  0  0  0  0
   -2.3628   -2.3856   -0.9669 Ir  0  0  0  0  0  0  0  0  0  0  0  0
   -4.6152   -8.5800   -4.7024 C   0  0  0  0  0  0  0  0  0  0  0  0
   -3.8965   -8.6604   -5.5591 H   0  0  0  0  0  0  0  0  0  0  0  0
   -4.8094   -9.6094   -4.3167 H   0  0  0  0  0  0  0  0  0  0  0  0
   -5.5775   -8.1679   -5.0921 H   0  0  0  0  0  0  0  0  0  0  0  0
   -8.2006   -6.9896    1.3668 C   0  0  0  0  0  0  0  0  0  0  0  0
   -8.3623   -6.9481    2.4696 H   0  0  0  0  0  0  0  0  0  0  0  0
   -9.2046   -6.9826    0.8690 H   0  0  0  0  0  0  0  0  0  0  0  0
   -7.6959   -7.9550    1.1219 H   0  0  0  0  0  0  0  0  0  0  0  0
   -4.9332   -5.6992    5.0796 C   0  0  0  0  0  0  0  0  0  0  0  0
   -5.0280   -6.7840    5.3312 H   0  0  0  0  0  0  0  0  0  0  0  0
   -4.3280   -5.2152    5.8847 H   0  0  0  0  0  0  0  0  0  0  0  0
   -5.9549   -5.2442    5.0866 H   0  0  0  0  0  0  0  0  0  0  0  0
   -6.3706    2.5452    2.8545 C   0  0  0  0  0  0  0  0  0  0  0  0
   -6.2919    3.5162    2.3051 H   0  0  0  0  0  0  0  0  0  0  0  0
   -7.4326    2.4174    3.1874 H   0  0  0  0  0  0  0  0  0  0  0  0
   -5.7262    2.6095    3.7632 H   0  0  0  0  0  0  0  0  0  0  0  0
    3.5603   -6.7361    0.2917 C   0  0  0  0  0  0  0  0  0  0  0  0
    4.0926   -6.1306    1.0664 H   0  0  0  0  0  0  0  0  0  0  0  0
    3.5606   -7.8065    0.6237 H   0  0  0  0  0  0  0  0  0  0  0  0
    4.1408   -6.6674   -0.6593 H   0  0  0  0  0  0  0  0  0  0  0  0
   -7.2419   -1.0266   -6.3024 C   0  0  0  0  0  0  0  0  0  0  0  0
   -8.1860   -1.5886   -6.5020 H   0  0  0  0  0  0  0  0  0  0  0  0
```

```
   -7.4712    0.0698   -6.3485 H   0  0  0  0  0  0  0  0  0  0  0  0
   -6.5189   -1.2651   -7.1196 H   0  0  0  0  0  0  0  0  0  0  0  0
  1  2  1  0  0  0  0
  1  3  1  0  0  0  0
  1  9  1  0  0  0  0
  1 10  1  0  0  0  0
  2 11  1  0  0  0  0
  2 16  1  0  0  0  0
  2 19  1  0  0  0  0
  3  5  2  0  0  0  0
  3  7  1  0  0  0  0
  3 83  1  0  0  0  0
  4  5  1  0  0  0  0
  4  6  1  0  0  0  0
  4 13  1  0  0  0  0
  4 14  1  0  0  0  0
  5  8  1  0  0  0  0
  5 83  1  0  0  0  0
  6 12  1  0  0  0  0
  6 15  1  0  0  0  0
  6 20  1  0  0  0  0
 15 16  2  0  0  0  0
 15 17  1  0  0  0  0
 15 83  1  0  0  0  0
 16 18  1  0  0  0  0
 16 83  1  0  0  0  0
 21 53  1  0  0  0  0
 21 63  1  0  0  0  0
 21 73  1  0  0  0  0
 21 83  1  0  0  0  0
 22 23  1  0  0  0  0
 22 33  1  0  0  0  0
 22 43  1  0  0  0  0
 22 83  1  0  0  0  0
 23 24  2  0  0  0  0
 23 25  1  0  0  0  0
 24 26  1  0  0  0  0
 24 27  1  0  0  0  0
 25 28  2  0  0  0  0
 25 29  1  0  0  0  0
 26 30  2  0  0  0  0
 26 31  1  0  0  0  0
 28 30  1  0  0  0  0
 28 32  1  0  0  0  0
 30100  1  0  0  0  0
 33 34  2  0  0  0  0
 33 35  1  0  0  0  0
 34 36  1  0  0  0  0
 34 37  1  0  0  0  0
 35 38  2  0  0  0  0
 35 39  1  0  0  0  0
 36 40  2  0  0  0  0
 36 41  1  0  0  0  0
 38 40  1  0  0  0  0
 38 42  1  0  0  0  0
 40 84  1  0  0  0  0
 43 44  2  0  0  0  0
 43 45  1  0  0  0  0
 44 46  1  0  0  0  0
 44 47  1  0  0  0  0
 45 48  2  0  0  0  0
 45 49  1  0  0  0  0
 46 50  2  0  0  0  0
 46 51  1  0  0  0  0
 48 50  1  0  0  0  0
```

```
 48 52  1  0  0  0  0
 50 92  1  0  0  0  0
 53 54  2  0  0  0  0
 53 55  1  0  0  0  0
 54 56  1  0  0  0  0
 54 57  1  0  0  0  0
 55 58  2  0  0  0  0
 55 59  1  0  0  0  0
 56 60  2  0  0  0  0
 56 61  1  0  0  0  0
 58 60  1  0  0  0  0
 58 62  1  0  0  0  0
 60 96  1  0  0  0  0
 63 64  2  0  0  0  0
 63 65  1  0  0  0  0
 64 66  1  0  0  0  0
 64 67  1  0  0  0  0
 65 68  2  0  0  0  0
 65 69  1  0  0  0  0
 66 70  2  0  0  0  0
 66 71  1  0  0  0  0
 68 70  1  0  0  0  0
 68 72  1  0  0  0  0
 70104  1  0  0  0  0
 73 74  2  0  0  0  0
 73 75  1  0  0  0  0
 74 76  1  0  0  0  0
 74 77  1  0  0  0  0
 75 78  2  0  0  0  0
 75 79  1  0  0  0  0
 76 80  2  0  0  0  0
 76 81  1  0  0  0  0
 78 80  1  0  0  0  0
 78 82  1  0  0  0  0
 80 88  1  0  0  0  0
 84 85  1  0  0  0  0
 84 86  1  0  0  0  0
 84 87  1  0  0  0  0
 88 89  1  0  0  0  0
 88 90  1  0  0  0  0
 88 91  1  0  0  0  0
 92 93  1  0  0  0  0
 92 94  1  0  0  0  0
 92 95  1  0  0  0  0
 96 97  1  0  0  0  0
 96 98  1  0  0  0  0
 96 99  1  0  0  0  0
100101  1  0  0  0  0
100102  1  0  0  0  0
100103  1  0  0  0  0
104105  1  0  0  0  0
104106  1  0  0  0  0
104107  1  0  0  0  0
M  END
$$$$
mol029
Optimized Catalyst 029
Optimized Catalyst 029
 95104  0  0  0  0  0  0  0  0999 V2000
    0.9495    0.0651    0.8457 C   0  0  0  0  0  0  0  0  0  0  0  0
   -0.3965    0.8020    0.9820 C   0  0  0  0  0  0  0  0  0  0  0  0
    0.8519   -1.3348    0.3069 C   0  0  0  0  0  0  0  0  0  0  0  0
    0.9342   -0.7487   -2.1902 C   0  0  0  0  0  0  0  0  0  0  0  0
    0.8529   -1.6749   -1.0051 C   0  0  0  0  0  0  0  0  0  0  0  0
    0.1902    0.5976   -2.0989 C   0  0  0  0  0  0  0  0  0  0  0  0
```

```
    0.9087   -2.1233    1.0793 H   0  0  0  0  0  0  0  0  0  0  0  0
    0.9831   -2.7440   -1.2875 H   0  0  0  0  0  0  0  0  0  0  0  0
    1.4226    0.0266    1.8658 H   0  0  0  0  0  0  0  0  0  0  0  0
    1.6494    0.6535    0.1949 H   0  0  0  0  0  0  0  0  0  0  0  0
   -0.8654    0.5008    1.9578 H   0  0  0  0  0  0  0  0  0  0  0  0
    0.0693    0.9925   -3.1453 H   0  0  0  0  0  0  0  0  0  0  0  0
    0.5688   -1.2928   -3.1033 H   0  0  0  0  0  0  0  0  0  0  0  0
    2.0301   -0.5297   -2.3527 H   0  0  0  0  0  0  0  0  0  0  0  0
   -1.1649    0.5284   -1.4509 C   0  0  0  0  0  0  0  0  0  0  0  0
   -1.4073    0.6256   -0.1204 C   0  0  0  0  0  0  0  0  0  0  0  0
   -2.0082    0.5255   -2.1650 H   0  0  0  0  0  0  0  0  0  0  0  0
   -2.4538    0.7632    0.2324 H   0  0  0  0  0  0  0  0  0  0  0  0
   -0.1820    1.9090    1.0458 H   0  0  0  0  0  0  0  0  0  0  0  0
    0.8164    1.3480   -1.5473 H   0  0  0  0  0  0  0  0  0  0  0  0
   -4.3328   -1.9467   -0.6149 P   0  0  0  0  0  0  0  0  0  0  0  0
   -1.6412   -4.5868   -0.2053 P   0  0  0  0  0  0  0  0  0  0  0  0
    0.3586   -6.1395   -0.9933 C   0  0  0  0  0  0  0  0  0  0  0  0
    0.5013   -6.5446   -2.3221 C   0  0  0  0  0  0  0  0  0  0  0  0
    0.7383   -6.9104    0.1058 C   0  0  0  0  0  0  0  0  0  0  0  0
    1.0583   -7.8069   -2.5510 C   0  0  0  0  0  0  0  0  0  0  0  0
    0.1968   -5.9003   -3.1448 H   0  0  0  0  0  0  0  0  0  0  0  0
    1.2895   -8.1695   -0.1567 C   0  0  0  0  0  0  0  0  0  0  0  0
    0.6137   -6.5540    1.1294 H   0  0  0  0  0  0  0  0  0  0  0  0
    1.4422   -8.6163   -1.4747 C   0  0  0  0  0  0  0  0  0  0  0  0
    1.1929   -8.1597   -3.5764 H   0  0  0  0  0  0  0  0  0  0  0  0
    1.6051   -8.8040    0.6755 H   0  0  0  0  0  0  0  0  0  0  0  0
    1.8695   -9.6038   -1.6658 H   0  0  0  0  0  0  0  0  0  0  0  0
   -2.7575   -6.5504   -1.6526 C   0  0  0  0  0  0  0  0  0  0  0  0
   -2.4381   -7.6517   -0.8589 C   0  0  0  0  0  0  0  0  0  0  0  0
   -3.1211   -6.6410   -2.9996 C   0  0  0  0  0  0  0  0  0  0  0  0
   -2.4768   -8.9123   -1.4673 C   0  0  0  0  0  0  0  0  0  0  0  0
   -2.1525   -7.5688    0.1909 H   0  0  0  0  0  0  0  0  0  0  0  0
   -3.1615   -7.9144   -3.5740 C   0  0  0  0  0  0  0  0  0  0  0  0
   -3.3682   -5.7467   -3.5699 H   0  0  0  0  0  0  0  0  0  0  0  0
   -2.8355   -9.0438   -2.8129 C   0  0  0  0  0  0  0  0  0  0  0  0
   -2.2204   -9.7979   -0.8800 H   0  0  0  0  0  0  0  0  0  0  0  0
   -3.4444   -8.0246   -4.6239 H   0  0  0  0  0  0  0  0  0  0  0  0
   -2.8621  -10.0339   -3.2733 H   0  0  0  0  0  0  0  0  0  0  0  0
   -2.6717   -5.2780    2.1273 C   0  0  0  0  0  0  0  0  0  0  0  0
   -3.9040   -5.8960    1.9070 C   0  0  0  0  0  0  0  0  0  0  0  0
   -2.3446   -4.5986    3.3052 C   0  0  0  0  0  0  0  0  0  0  0  0
   -4.8588   -5.8105    2.9271 C   0  0  0  0  0  0  0  0  0  0  0  0
   -4.1306   -6.4349    0.9875 H   0  0  0  0  0  0  0  0  0  0  0  0
   -3.3169   -4.5362    4.3081 C   0  0  0  0  0  0  0  0  0  0  0  0
   -1.3548   -4.1677    3.4474 H   0  0  0  0  0  0  0  0  0  0  0  0
   -4.5690   -5.1340    4.1173 C   0  0  0  0  0  0  0  0  0  0  0  0
   -5.8362   -6.2786    2.7866 H   0  0  0  0  0  0  0  0  0  0  0  0
   -3.0926   -4.0229    5.2467 H   0  0  0  0  0  0  0  0  0  0  0  0
   -5.3204   -5.0805    4.9083 H   0  0  0  0  0  0  0  0  0  0  0  0
   -5.8957    0.0753    0.1966 C   0  0  0  0  0  0  0  0  0  0  0  0
   -6.5292    0.8840   -0.7487 C   0  0  0  0  0  0  0  0  0  0  0  0
   -6.3982   -0.1703    1.4752 C   0  0  0  0  0  0  0  0  0  0  0  0
   -7.7550    1.4544   -0.3904 C   0  0  0  0  0  0  0  0  0  0  0  0
   -6.0811    1.0754   -1.7228 H   0  0  0  0  0  0  0  0  0  0  0  0
   -7.6287    0.4102    1.8027 C   0  0  0  0  0  0  0  0  0  0  0  0
   -5.8407   -0.7590    2.2024 H   0  0  0  0  0  0  0  0  0  0  0  0
   -8.3051    1.2101    0.8738 C   0  0  0  0  0  0  0  0  0  0  0  0
   -8.2806    2.0956   -1.1026 H   0  0  0  0  0  0  0  0  0  0  0  0
   -8.0570    0.2449    2.7942 H   0  0  0  0  0  0  0  0  0  0  0  0
   -9.2647    1.6591    1.1425 H   0  0  0  0  0  0  0  0  0  0  0  0
   -6.1533   -1.6935   -2.5530 C   0  0  0  0  0  0  0  0  0  0  0  0
   -7.4054   -1.9724   -2.0052 C   0  0  0  0  0  0  0  0  0  0  0  0
   -5.9584   -0.8882   -3.6791 C   0  0  0  0  0  0  0  0  0  0  0  0
   -8.5205   -1.3991   -2.6279 C   0  0  0  0  0  0  0  0  0  0  0  0
   -7.5382   -2.6227   -1.1427 H   0  0  0  0  0  0  0  0  0  0  0  0
```

```
   -7.0930   -0.3389   -4.2848 C   0  0  0  0  0  0  0  0  0  0  0  0
   -4.9616   -0.7206   -4.0836 H   0  0  0  0  0  0  0  0  0  0  0  0
   -8.3659   -0.5863   -3.7566 C   0  0  0  0  0  0  0  0  0  0  0  0
   -9.5187   -1.5950   -2.2280 H   0  0  0  0  0  0  0  0  0  0  0  0
   -6.9812    0.2835   -5.1768 H   0  0  0  0  0  0  0  0  0  0  0  0
   -9.2460   -0.1497   -4.2357 H   0  0  0  0  0  0  0  0  0  0  0  0
   -5.9727   -3.8294    0.3881 C   0  0  0  0  0  0  0  0  0  0  0  0
   -7.0085   -3.6014    1.2966 C   0  0  0  0  0  0  0  0  0  0  0  0
   -5.9664   -4.8822   -0.5316 C   0  0  0  0  0  0  0  0  0  0  0  0
   -8.0911   -4.4870    1.2795 C   0  0  0  0  0  0  0  0  0  0  0  0
   -6.9622   -2.7793    2.0078 H   0  0  0  0  0  0  0  0  0  0  0  0
   -7.0643   -5.7480   -0.5271 C   0  0  0  0  0  0  0  0  0  0  0  0
   -5.1392   -5.0374   -1.2277 H   0  0  0  0  0  0  0  0  0  0  0  0
   -8.1193   -5.5523    0.3726 C   0  0  0  0  0  0  0  0  0  0  0  0
   -8.9171   -4.3434    1.9807 H   0  0  0  0  0  0  0  0  0  0  0  0
   -7.0947   -6.5831   -1.2320 H   0  0  0  0  0  0  0  0  0  0  0  0
   -8.9703   -6.2379    0.3678 H   0  0  0  0  0  0  0  0  0  0  0  0
   -1.7645   -2.0205   -0.4839 Ir  0  0  0  0  0  0  0  0  0  0  0  0
   -1.6014   -5.4416    1.2078 O   0  0  0  0  0  0  0  0  0  0  0  0
   -0.1271   -4.8087   -0.8029 O   0  0  0  0  0  0  0  0  0  0  0  0
   -2.8260   -5.2188   -1.1584 O   0  0  0  0  0  0  0  0  0  0  0  0
   -4.5964   -0.4008   -0.1314 O   0  0  0  0  0  0  0  0  0  0  0  0
   -4.9742   -2.3261   -2.0716 O   0  0  0  0  0  0  0  0  0  0  0  0
   -4.8233   -3.0160    0.5378 O   0  0  0  0  0  0  0  0  0  0  0  0
  1  2  1  0  0  0  0
  1  3  1  0  0  0  0
  1  9  1  0  0  0  0
  1 10  1  0  0  0  0
  2 11  1  0  0  0  0
  2 16  1  0  0  0  0
  2 19  1  0  0  0  0
  3  5  2  0  0  0  0
  3  7  1  0  0  0  0
  3 89  1  0  0  0  0
  4  5  1  0  0  0  0
  4  6  1  0  0  0  0
  4 13  1  0  0  0  0
  4 14  1  0  0  0  0
  5  8  1  0  0  0  0
  5 89  1  0  0  0  0
  6 12  1  0  0  0  0
  6 15  1  0  0  0  0
  6 20  1  0  0  0  0
 15 16  2  0  0  0  0
 15 17  1  0  0  0  0
 15 89  1  0  0  0  0
 16 18  1  0  0  0  0
 16 89  1  0  0  0  0
 21 93  1  0  0  0  0
 21 94  1  0  0  0  0
 21 95  1  0  0  0  0
 21 89  1  0  0  0  0
 22 90  1  0  0  0  0
 22 91  1  0  0  0  0
 22 92  1  0  0  0  0
 22 89  1  0  0  0  0
 23 24  2  0  0  0  0
 23 25  1  0  0  0  0
 23 91  1  0  0  0  0
 24 26  1  0  0  0  0
 24 27  1  0  0  0  0
 25 28  2  0  0  0  0
 25 29  1  0  0  0  0
 26 30  2  0  0  0  0
 26 31  1  0  0  0  0
```

```
 28 30  1  0  0  0  0
 28 32  1  0  0  0  0
 30 33  1  0  0  0  0
 34 35  2  0  0  0  0
 34 36  1  0  0  0  0
 34 92  1  0  0  0  0
 35 37  1  0  0  0  0
 35 38  1  0  0  0  0
 36 39  2  0  0  0  0
 36 40  1  0  0  0  0
 37 41  2  0  0  0  0
 37 42  1  0  0  0  0
 39 41  1  0  0  0  0
 39 43  1  0  0  0  0
 41 44  1  0  0  0  0
 45 46  2  0  0  0  0
 45 47  1  0  0  0  0
 45 90  1  0  0  0  0
 46 48  1  0  0  0  0
 46 49  1  0  0  0  0
 47 50  2  0  0  0  0
 47 51  1  0  0  0  0
 48 52  2  0  0  0  0
 48 53  1  0  0  0  0
 50 52  1  0  0  0  0
 50 54  1  0  0  0  0
 52 55  1  0  0  0  0
 56 57  2  0  0  0  0
 56 58  1  0  0  0  0
 56 93  1  0  0  0  0
 57 59  1  0  0  0  0
 57 60  1  0  0  0  0
 58 61  2  0  0  0  0
 58 62  1  0  0  0  0
 59 63  2  0  0  0  0
 59 64  1  0  0  0  0
 61 63  1  0  0  0  0
 61 65  1  0  0  0  0
 63 66  1  0  0  0  0
 67 68  2  0  0  0  0
 67 69  1  0  0  0  0
 67 94  1  0  0  0  0
 68 70  1  0  0  0  0
 68 71  1  0  0  0  0
 69 72  2  0  0  0  0
 69 73  1  0  0  0  0
 70 74  2  0  0  0  0
 70 75  1  0  0  0  0
 72 74  1  0  0  0  0
 72 76  1  0  0  0  0
 74 77  1  0  0  0  0
 78 79  2  0  0  0  0
 78 80  1  0  0  0  0
 78 95  1  0  0  0  0
 79 81  1  0  0  0  0
 79 82  1  0  0  0  0
 80 83  2  0  0  0  0
 80 84  1  0  0  0  0
 81 85  2  0  0  0  0
 81 86  1  0  0  0  0
 83 85  1  0  0  0  0
 83 87  1  0  0  0  0
 85 88  1  0  0  0  0
M  END
$$$$
```

```
mol030
Optimized Catalyst 030
Optimized Catalyst 030
 95104  0  0  0  0  0  0  0  0999 V2000
    0.9317    0.0452    0.8491 C   0  0  0  0  0  0  0  0  0  0  0  0
   -0.4117    0.7866    0.9835 C   0  0  0  0  0  0  0  0  0  0  0  0
    0.8342   -1.3543    0.3081 C   0  0  0  0  0  0  0  0  0  0  0  0
    0.9316   -0.7628   -2.1862 C   0  0  0  0  0  0  0  0  0  0  0  0
    0.8406   -1.6934   -1.0053 C   0  0  0  0  0  0  0  0  0  0  0  0
    0.1802    0.5792   -2.0972 C   0  0  0  0  0  0  0  0  0  0  0  0
    0.8945   -2.1436    1.0798 H   0  0  0  0  0  0  0  0  0  0  0  0
    0.9770   -2.7616   -1.2895 H   0  0  0  0  0  0  0  0  0  0  0  0
    1.4031    0.0040    1.8705 H   0  0  0  0  0  0  0  0  0  0  0  0
    1.6347    0.6346    0.2016 H   0  0  0  0  0  0  0  0  0  0  0  0
   -0.8820    0.4940    1.9615 H   0  0  0  0  0  0  0  0  0  0  0  0
    0.0594    0.9730   -3.1445 H   0  0  0  0  0  0  0  0  0  0  0  0
    0.5832   -1.3042   -3.1076 H   0  0  0  0  0  0  0  0  0  0  0  0
    2.0292   -0.5375   -2.3337 H   0  0  0  0  0  0  0  0  0  0  0  0
   -1.1761    0.5088   -1.4507 C   0  0  0  0  0  0  0  0  0  0  0  0
   -1.4214    0.6095   -0.1197 C   0  0  0  0  0  0  0  0  0  0  0  0
   -2.0181    0.5142   -2.1670 H   0  0  0  0  0  0  0  0  0  0  0  0
   -2.4677    0.7589    0.2303 H   0  0  0  0  0  0  0  0  0  0  0  0
   -0.1930    1.8940    1.0428 H   0  0  0  0  0  0  0  0  0  0  0  0
    0.8033    1.3338   -1.5465 H   0  0  0  0  0  0  0  0  0  0  0  0
   -4.3269   -1.9294   -0.6168 P   0  0  0  0  0  0  0  0  0  0  0  0
   -1.6444   -4.5811   -0.2111 P   0  0  0  0  0  0  0  0  0  0  0  0
    0.3494   -6.1354   -0.9976 C   0  0  0  0  0  0  0  0  0  0  0  0
    0.4829   -6.5519   -2.3274 C   0  0  0  0  0  0  0  0  0  0  0  0
    0.7446   -6.8971    0.1066 C   0  0  0  0  0  0  0  0  0  0  0  0
    1.0286   -7.8137   -2.5651 C   0  0  0  0  0  0  0  0  0  0  0  0
    0.1770   -5.9058   -3.1506 H   0  0  0  0  0  0  0  0  0  0  0  0
    1.2865   -8.1607   -0.1352 C   0  0  0  0  0  0  0  0  0  0  0  0
    0.6353   -6.5239    1.1281 H   0  0  0  0  0  0  0  0  0  0  0  0
    1.4114   -8.5915   -1.4632 C   0  0  0  0  0  0  0  0  0  0  0  0
    1.1572   -8.1833   -3.5867 H   0  0  0  0  0  0  0  0  0  0  0  0
    1.6124   -8.7950    0.6946 H   0  0  0  0  0  0  0  0  0  0  0  0
   -2.7467   -6.5591   -1.6487 C   0  0  0  0  0  0  0  0  0  0  0  0
   -2.4442   -7.6683   -0.8537 C   0  0  0  0  0  0  0  0  0  0  0  0
   -3.0848   -6.6438   -3.0060 C   0  0  0  0  0  0  0  0  0  0  0  0
   -2.4795   -8.9301   -1.4520 C   0  0  0  0  0  0  0  0  0  0  0  0
   -2.1692   -7.5843    0.2008 H   0  0  0  0  0  0  0  0  0  0  0  0
   -3.1274   -7.9059   -3.5978 C   0  0  0  0  0  0  0  0  0  0  0  0
   -3.3154   -5.7422   -3.5754 H   0  0  0  0  0  0  0  0  0  0  0  0
   -2.8187   -9.0221   -2.8080 C   0  0  0  0  0  0  0  0  0  0  0  0
   -2.2471   -9.8268   -0.8693 H   0  0  0  0  0  0  0  0  0  0  0  0
   -3.3936   -8.0194   -4.6531 H   0  0  0  0  0  0  0  0  0  0  0  0
   -2.6691   -5.2781    2.1154 C   0  0  0  0  0  0  0  0  0  0  0  0
   -3.8975   -5.9124    1.8966 C   0  0  0  0  0  0  0  0  0  0  0  0
   -2.3516   -4.5912    3.2957 C   0  0  0  0  0  0  0  0  0  0  0  0
   -4.8686   -5.8279    2.8965 C   0  0  0  0  0  0  0  0  0  0  0  0
   -4.1095   -6.4634    0.9787 H   0  0  0  0  0  0  0  0  0  0  0  0
   -3.3219   -4.5112    4.2949 C   0  0  0  0  0  0  0  0  0  0  0  0
   -1.3608   -4.1574    3.4389 H   0  0  0  0  0  0  0  0  0  0  0  0
   -4.5629   -5.1230    4.0677 C   0  0  0  0  0  0  0  0  0  0  0  0
   -5.8422   -6.3086    2.7658 H   0  0  0  0  0  0  0  0  0  0  0  0
   -3.1134   -3.9940    5.2364 H   0  0  0  0  0  0  0  0  0  0  0  0
   -5.8922    0.0838    0.1898 C   0  0  0  0  0  0  0  0  0  0  0  0
   -6.5463    0.8798   -0.7570 C   0  0  0  0  0  0  0  0  0  0  0  0
   -6.3775   -0.1513    1.4804 C   0  0  0  0  0  0  0  0  0  0  0  0
   -7.7812    1.4291   -0.4116 C   0  0  0  0  0  0  0  0  0  0  0  0
   -6.0979    1.0785   -1.7318 H   0  0  0  0  0  0  0  0  0  0  0  0
   -7.6155    0.3954    1.8226 C   0  0  0  0  0  0  0  0  0  0  0  0
   -5.7948   -0.7163    2.2096 H   0  0  0  0  0  0  0  0  0  0  0  0
   -8.2942    1.1634    0.8661 C   0  0  0  0  0  0  0  0  0  0  0  0
   -8.3299    2.0582   -1.1193 H   0  0  0  0  0  0  0  0  0  0  0  0
```

```
   -8.0377    0.2409    2.8200 H   0  0  0  0  0  0  0  0  0  0  0  0
   -6.1680   -1.6998   -2.5350 C   0  0  0  0  0  0  0  0  0  0  0  0
   -7.4303   -1.9936   -2.0098 C   0  0  0  0  0  0  0  0  0  0  0  0
   -5.9627   -0.8811   -3.6537 C   0  0  0  0  0  0  0  0  0  0  0  0
   -8.5449   -1.4202   -2.6250 C   0  0  0  0  0  0  0  0  0  0  0  0
   -7.5651   -2.6608   -1.1586 H   0  0  0  0  0  0  0  0  0  0  0  0
   -7.0788   -0.3161   -4.2710 C   0  0  0  0  0  0  0  0  0  0  0  0
   -4.9586   -0.7127   -4.0443 H   0  0  0  0  0  0  0  0  0  0  0  0
   -8.3456   -0.5913   -3.7374 C   0  0  0  0  0  0  0  0  0  0  0  0
   -9.5530   -1.6228   -2.2499 H   0  0  0  0  0  0  0  0  0  0  0  0
   -6.9665    0.3185   -5.1558 H   0  0  0  0  0  0  0  0  0  0  0  0
   -5.9526   -3.8027    0.4032 C   0  0  0  0  0  0  0  0  0  0  0  0
   -6.9910   -3.5781    1.3140 C   0  0  0  0  0  0  0  0  0  0  0  0
   -5.9449   -4.8578   -0.5192 C   0  0  0  0  0  0  0  0  0  0  0  0
   -8.0813   -4.4497    1.2985 C   0  0  0  0  0  0  0  0  0  0  0  0
   -6.9402   -2.7596    2.0322 H   0  0  0  0  0  0  0  0  0  0  0  0
   -7.0379   -5.7240   -0.5355 C   0  0  0  0  0  0  0  0  0  0  0  0
   -5.1110   -5.0110   -1.2106 H   0  0  0  0  0  0  0  0  0  0  0  0
   -8.0827   -5.5008    0.3722 C   0  0  0  0  0  0  0  0  0  0  0  0
   -8.9122   -4.3152    1.9981 H   0  0  0  0  0  0  0  0  0  0  0  0
   -7.0761   -6.5587   -1.2420 H   0  0  0  0  0  0  0  0  0  0  0  0
   -1.7568   -2.0104   -0.4907 Ir  0  0  0  0  0  0  0  0  0  0  0  0
   -1.5973   -5.4449    1.2035 O   0  0  0  0  0  0  0  0  0  0  0  0
   -0.1294   -4.8058   -0.8139 O   0  0  0  0  0  0  0  0  0  0  0  0
   -2.8377   -5.2353   -1.1455 O   0  0  0  0  0  0  0  0  0  0  0  0
   -4.5927   -0.3767   -0.1448 O   0  0  0  0  0  0  0  0  0  0  0  0
   -5.0018   -2.3472   -2.0529 O   0  0  0  0  0  0  0  0  0  0  0  0
   -4.8036   -2.9940    0.5543 O   0  0  0  0  0  0  0  0  0  0  0  0
    1.9149   -9.8010   -1.6894 F   0  0  0  0  0  0  0  0  0  0  0  0
   -2.8455  -10.2251   -3.3740 F   0  0  0  0  0  0  0  0  0  0  0  0
   -5.4981   -5.0270    5.0086 F   0  0  0  0  0  0  0  0  0  0  0  0
   -9.4805    1.6690    1.1880 F   0  0  0  0  0  0  0  0  0  0  0  0
   -9.4084   -0.0381   -4.3128 F   0  0  0  0  0  0  0  0  0  0  0  0
   -9.1239   -6.3270    0.3533 F   0  0  0  0  0  0  0  0  0  0  0  0
  1  2  1  0  0  0  0
  1  3  1  0  0  0  0
  1  9  1  0  0  0  0
  1 10  1  0  0  0  0
  2 11  1  0  0  0  0
  2 16  1  0  0  0  0
  2 19  1  0  0  0  0
  3  5  2  0  0  0  0
  3  7  1  0  0  0  0
  3 83  1  0  0  0  0
  4  5  1  0  0  0  0
  4  6  1  0  0  0  0
  4 13  1  0  0  0  0
  4 14  1  0  0  0  0
  5  8  1  0  0  0  0
  5 83  1  0  0  0  0
  6 12  1  0  0  0  0
  6 15  1  0  0  0  0
  6 20  1  0  0  0  0
 15 16  2  0  0  0  0
 15 17  1  0  0  0  0
 15 83  1  0  0  0  0
 16 18  1  0  0  0  0
 16 83  1  0  0  0  0
 21 87  1  0  0  0  0
 21 88  1  0  0  0  0
 21 89  1  0  0  0  0
 21 83  1  0  0  0  0
 22 84  1  0  0  0  0
 22 85  1  0  0  0  0
 22 86  1  0  0  0  0
```

```
 22 83  1  0  0  0  0
 23 24  2  0  0  0  0
 23 25  1  0  0  0  0
 23 85  1  0  0  0  0
 24 26  1  0  0  0  0
 24 27  1  0  0  0  0
 25 28  2  0  0  0  0
 25 29  1  0  0  0  0
 26 30  2  0  0  0  0
 26 31  1  0  0  0  0
 28 30  1  0  0  0  0
 28 32  1  0  0  0  0
 30 90  1  0  0  0  0
 33 34  2  0  0  0  0
 33 35  1  0  0  0  0
 33 86  1  0  0  0  0
 34 36  1  0  0  0  0
 34 37  1  0  0  0  0
 35 38  2  0  0  0  0
 35 39  1  0  0  0  0
 36 40  2  0  0  0  0
 36 41  1  0  0  0  0
 38 40  1  0  0  0  0
 38 42  1  0  0  0  0
 40 91  1  0  0  0  0
 43 44  2  0  0  0  0
 43 45  1  0  0  0  0
 43 84  1  0  0  0  0
 44 46  1  0  0  0  0
 44 47  1  0  0  0  0
 45 48  2  0  0  0  0
 45 49  1  0  0  0  0
 46 50  2  0  0  0  0
 46 51  1  0  0  0  0
 48 50  1  0  0  0  0
 48 52  1  0  0  0  0
 50 92  1  0  0  0  0
 53 54  2  0  0  0  0
 53 55  1  0  0  0  0
 53 87  1  0  0  0  0
 54 56  1  0  0  0  0
 54 57  1  0  0  0  0
 55 58  2  0  0  0  0
 55 59  1  0  0  0  0
 56 60  2  0  0  0  0
 56 61  1  0  0  0  0
 58 60  1  0  0  0  0
 58 62  1  0  0  0  0
 60 93  1  0  0  0  0
 63 64  2  0  0  0  0
 63 65  1  0  0  0  0
 63 88  1  0  0  0  0
 64 66  1  0  0  0  0
 64 67  1  0  0  0  0
 65 68  2  0  0  0  0
 65 69  1  0  0  0  0
 66 70  2  0  0  0  0
 66 71  1  0  0  0  0
 68 70  1  0  0  0  0
 68 72  1  0  0  0  0
 70 94  1  0  0  0  0
 73 74  2  0  0  0  0
 73 75  1  0  0  0  0
 73 89  1  0  0  0  0
 74 76  1  0  0  0  0
```

```
 74 77  1  0  0  0  0
 75 78  2  0  0  0  0
 75 79  1  0  0  0  0
 76 80  2  0  0  0  0
 76 81  1  0  0  0  0
 78 80  1  0  0  0  0
 78 82  1  0  0  0  0
 80 95  1  0  0  0  0
M  END
$$$$
mol031
Optimized Catalyst 031
Optimized Catalyst 031
113122  0  0  0  0  0  0  0  0999 V2000
    0.9827    0.0517    0.8629 C   0  0  0  0  0  0  0  0  0  0  0  0
   -0.3632    0.7938    0.9739 C   0  0  0  0  0  0  0  0  0  0  0  0
    0.8873   -1.3472    0.3212 C   0  0  0  0  0  0  0  0  0  0  0  0
    1.0263   -0.7622   -2.1713 C   0  0  0  0  0  0  0  0  0  0  0  0
    0.9098   -1.6879   -0.9892 C   0  0  0  0  0  0  0  0  0  0  0  0
    0.2913    0.5902   -2.0953 C   0  0  0  0  0  0  0  0  0  0  0  0
    0.9175   -2.1357    1.0944 H   0  0  0  0  0  0  0  0  0  0  0  0
    1.0238   -2.7591   -1.2690 H   0  0  0  0  0  0  0  0  0  0  0  0
    1.4358    0.0093    1.8914 H   0  0  0  0  0  0  0  0  0  0  0  0
    1.6956    0.6388    0.2260 H   0  0  0  0  0  0  0  0  0  0  0  0
   -0.8559    0.4874    1.9359 H   0  0  0  0  0  0  0  0  0  0  0  0
    0.1952    0.9860   -3.1435 H   0  0  0  0  0  0  0  0  0  0  0  0
    0.6771   -1.3022   -3.0925 H   0  0  0  0  0  0  0  0  0  0  0  0
    2.1270   -0.5520   -2.3075 H   0  0  0  0  0  0  0  0  0  0  0  0
   -1.0773    0.5309   -1.4762 C   0  0  0  0  0  0  0  0  0  0  0  0
   -1.3489    0.6273   -0.1523 C   0  0  0  0  0  0  0  0  0  0  0  0
   -1.9048    0.5274   -2.2079 H   0  0  0  0  0  0  0  0  0  0  0  0
   -2.4023    0.7649    0.1766 H   0  0  0  0  0  0  0  0  0  0  0  0
   -0.1449    1.8987    1.0495 H   0  0  0  0  0  0  0  0  0  0  0  0
    0.9116    1.3348   -1.5300 H   0  0  0  0  0  0  0  0  0  0  0  0
   -4.3142   -1.9427   -0.6263 P   0  0  0  0  0  0  0  0  0  0  0  0
   -1.6500   -4.6054   -0.2282 P   0  0  0  0  0  0  0  0  0  0  0  0
    0.3534   -6.1424   -1.0013 C   0  0  0  0  0  0  0  0  0  0  0  0
    0.4476   -6.5653   -2.3294 C   0  0  0  0  0  0  0  0  0  0  0  0
    0.7699   -6.9104    0.0879 C   0  0  0  0  0  0  0  0  0  0  0  0
    0.9580   -7.8421   -2.5678 C   0  0  0  0  0  0  0  0  0  0  0  0
    0.1320   -5.9205   -3.1482 H   0  0  0  0  0  0  0  0  0  0  0  0
    1.2757   -8.1831   -0.1791 C   0  0  0  0  0  0  0  0  0  0  0  0
    0.7015   -6.5363    1.1107 H   0  0  0  0  0  0  0  0  0  0  0  0
    1.3605   -8.6650   -1.4997 C   0  0  0  0  0  0  0  0  0  0  0  0
    1.0400   -8.2012   -3.5950 H   0  0  0  0  0  0  0  0  0  0  0  0
    1.6124   -8.8109    0.6486 H   0  0  0  0  0  0  0  0  0  0  0  0
   -2.7049   -6.5897   -1.6747 C   0  0  0  0  0  0  0  0  0  0  0  0
   -2.3837   -7.6968   -0.8896 C   0  0  0  0  0  0  0  0  0  0  0  0
   -3.0125   -6.6767   -3.0371 C   0  0  0  0  0  0  0  0  0  0  0  0
   -2.3548   -8.9473   -1.5140 C   0  0  0  0  0  0  0  0  0  0  0  0
   -2.1346   -7.6206    0.1702 H   0  0  0  0  0  0  0  0  0  0  0  0
   -2.9842   -7.9366   -3.6323 C   0  0  0  0  0  0  0  0  0  0  0  0
   -3.2702   -5.7820   -3.6031 H   0  0  0  0  0  0  0  0  0  0  0  0
   -2.6450   -9.0801   -2.8825 C   0  0  0  0  0  0  0  0  0  0  0  0
   -2.0945   -9.8273   -0.9225 H   0  0  0  0  0  0  0  0  0  0  0  0
   -3.2230   -8.0354   -4.6935 H   0  0  0  0  0  0  0  0  0  0  0  0
   -2.7615   -5.2280    2.0621 C   0  0  0  0  0  0  0  0  0  0  0  0
   -3.9163   -5.9923    1.8712 C   0  0  0  0  0  0  0  0  0  0  0  0
   -2.5839   -4.3704    3.1524 C   0  0  0  0  0  0  0  0  0  0  0  0
   -4.9427   -5.8647    2.8074 C   0  0  0  0  0  0  0  0  0  0  0  0
   -4.0284   -6.6671    1.0231 H   0  0  0  0  0  0  0  0  0  0  0  0
   -3.6260   -4.2613    4.0752 C   0  0  0  0  0  0  0  0  0  0  0  0
   -1.6491   -3.8292    3.2937 H   0  0  0  0  0  0  0  0  0  0  0  0
   -4.8146   -4.9946    3.9068 C   0  0  0  0  0  0  0  0  0  0  0  0
   -5.8583   -6.4457    2.6788 H   0  0  0  0  0  0  0  0  0  0  0  0
```

```
   -3.5058   -3.6064    4.9403 H   0  0  0  0  0  0  0  0  0  0  0  0
   -5.8731    0.0518    0.2084 C   0  0  0  0  0  0  0  0  0  0  0  0
   -6.6211    0.7770   -0.7204 C   0  0  0  0  0  0  0  0  0  0  0  0
   -6.2723   -0.1426    1.5333 C   0  0  0  0  0  0  0  0  0  0  0  0
   -7.8589    1.2765   -0.3132 C   0  0  0  0  0  0  0  0  0  0  0  0
   -6.2477    0.9583   -1.7287 H   0  0  0  0  0  0  0  0  0  0  0  0
   -7.5137    0.3660    1.9159 C   0  0  0  0  0  0  0  0  0  0  0  0
   -5.6265   -0.6504    2.2496 H   0  0  0  0  0  0  0  0  0  0  0  0
   -8.3226    1.0645    0.9979 C   0  0  0  0  0  0  0  0  0  0  0  0
   -8.4662    1.8386   -1.0255 H   0  0  0  0  0  0  0  0  0  0  0  0
   -7.8576    0.2283    2.9428 H   0  0  0  0  0  0  0  0  0  0  0  0
   -6.1285   -1.7361   -2.5554 C   0  0  0  0  0  0  0  0  0  0  0  0
   -7.3931   -2.1131   -2.1011 C   0  0  0  0  0  0  0  0  0  0  0  0
   -5.9255   -0.8268   -3.5994 C   0  0  0  0  0  0  0  0  0  0  0  0
   -8.5030   -1.5215   -2.7080 C   0  0  0  0  0  0  0  0  0  0  0  0
   -7.5273   -2.8558   -1.3153 H   0  0  0  0  0  0  0  0  0  0  0  0
   -7.0508   -0.2561   -4.1928 C   0  0  0  0  0  0  0  0  0  0  0  0
   -4.9214   -0.5905   -3.9489 H   0  0  0  0  0  0  0  0  0  0  0  0
   -8.3457   -0.5849   -3.7460 C   0  0  0  0  0  0  0  0  0  0  0  0
   -9.5039   -1.8007   -2.3735 H   0  0  0  0  0  0  0  0  0  0  0  0
   -6.9246    0.4474   -5.0189 H   0  0  0  0  0  0  0  0  0  0  0  0
   -5.9503   -3.7688    0.4614 C   0  0  0  0  0  0  0  0  0  0  0  0
   -6.9713   -3.4728    1.3660 C   0  0  0  0  0  0  0  0  0  0  0  0
   -5.9839   -4.8722   -0.3996 C   0  0  0  0  0  0  0  0  0  0  0  0
   -8.0775   -4.3244    1.4048 C   0  0  0  0  0  0  0  0  0  0  0  0
   -6.8958   -2.6208    2.0394 H   0  0  0  0  0  0  0  0  0  0  0  0
   -7.1007   -5.7043   -0.3428 C   0  0  0  0  0  0  0  0  0  0  0  0
   -5.1621   -5.0876   -1.0877 H   0  0  0  0  0  0  0  0  0  0  0  0
   -8.1537   -5.4424    0.5565 C   0  0  0  0  0  0  0  0  0  0  0  0
   -8.8863   -4.1151    2.1069 H   0  0  0  0  0  0  0  0  0  0  0  0
   -7.1562   -6.5734   -1.0020 H   0  0  0  0  0  0  0  0  0  0  0  0
   -1.7468   -2.0390   -0.5147 Ir  0  0  0  0  0  0  0  0  0  0  0  0
   -1.6339   -5.4112    1.2164 O   0  0  0  0  0  0  0  0  0  0  0  0
   -0.1219   -4.8109   -0.7986 O   0  0  0  0  0  0  0  0  0  0  0  0
   -2.8333   -5.2691   -1.1621 O   0  0  0  0  0  0  0  0  0  0  0  0
   -4.5775   -0.3818   -0.1867 O   0  0  0  0  0  0  0  0  0  0  0  0
   -4.9615   -2.3818   -2.0657 O   0  0  0  0  0  0  0  0  0  0  0  0
   -4.7705   -2.9921    0.5577 O   0  0  0  0  0  0  0  0  0  0  0  0
   -5.9377   -4.8690    4.8823 C   0  0  0  0  0  0  0  0  0  0  0  0
   -6.0272   -5.8184    5.4692 H   0  0  0  0  0  0  0  0  0  0  0  0
   -5.7726   -4.0325    5.6010 H   0  0  0  0  0  0  0  0  0  0  0  0
   -6.9003   -4.6894    4.3464 H   0  0  0  0  0  0  0  0  0  0  0  0
   -9.3277   -6.3621    0.6120 C   0  0  0  0  0  0  0  0  0  0  0  0
   -9.6921   -6.5961   -0.4180 H   0  0  0  0  0  0  0  0  0  0  0  0
   -9.0305   -7.3200    1.1083 H   0  0  0  0  0  0  0  0  0  0  0  0
  -10.1756   -5.9201    1.1865 H   0  0  0  0  0  0  0  0  0  0  0  0
   -2.5967  -10.4149   -3.5495 C   0  0  0  0  0  0  0  0  0  0  0  0
   -3.6274  -10.7200   -3.8596 H   0  0  0  0  0  0  0  0  0  0  0  0
   -1.9517  -10.3683   -4.4608 H   0  0  0  0  0  0  0  0  0  0  0  0
   -2.1885  -11.2032   -2.8746 H   0  0  0  0  0  0  0  0  0  0  0  0
    1.8825  -10.0400   -1.7547 C   0  0  0  0  0  0  0  0  0  0  0  0
    2.9921  -10.0504   -1.6042 H   0  0  0  0  0  0  0  0  0  0  0  0
    1.4235  -10.7726   -1.0481 H   0  0  0  0  0  0  0  0  0  0  0  0
    1.6693  -10.3780   -2.7957 H   0  0  0  0  0  0  0  0  0  0  0  0
   -9.5357    0.0453   -4.3883 C   0  0  0  0  0  0  0  0  0  0  0  0
   -9.3537    1.1286   -4.5900 H   0  0  0  0  0  0  0  0  0  0  0  0
   -9.7416   -0.4643   -5.3646 H   0  0  0  0  0  0  0  0  0  0  0  0
  -10.4465   -0.0417   -3.7506 H   0  0  0  0  0  0  0  0  0  0  0  0
   -9.6610    1.5682    1.4245 C   0  0  0  0  0  0  0  0  0  0  0  0
  -10.3413    0.7051    1.6289 H   0  0  0  0  0  0  0  0  0  0  0  0
   -9.5675    2.1770    2.3583 H   0  0  0  0  0  0  0  0  0  0  0  0
  -10.1331    2.2097    0.6432 H   0  0  0  0  0  0  0  0  0  0  0  0
  1  2  1  0  0  0  0
  1  3  1  0  0  0  0
  1  9  1  0  0  0  0
```

```
  1 10  1  0  0  0  0
  2 11  1  0  0  0  0
  2 16  1  0  0  0  0
  2 19  1  0  0  0  0
  3  5  2  0  0  0  0
  3  7  1  0  0  0  0
  3 83  1  0  0  0  0
  4  5  1  0  0  0  0
  4  6  1  0  0  0  0
  4 13  1  0  0  0  0
  4 14  1  0  0  0  0
  5  8  1  0  0  0  0
  5 83  1  0  0  0  0
  6 12  1  0  0  0  0
  6 15  1  0  0  0  0
  6 20  1  0  0  0  0
 15 16  2  0  0  0  0
 15 17  1  0  0  0  0
 15 83  1  0  0  0  0
 16 18  1  0  0  0  0
 16 83  1  0  0  0  0
 21 87  1  0  0  0  0
 21 88  1  0  0  0  0
 21 89  1  0  0  0  0
 21 83  1  0  0  0  0
 22 84  1  0  0  0  0
 22 85  1  0  0  0  0
 22 86  1  0  0  0  0
 22 83  1  0  0  0  0
 23 24  2  0  0  0  0
 23 25  1  0  0  0  0
 23 85  1  0  0  0  0
 24 26  1  0  0  0  0
 24 27  1  0  0  0  0
 25 28  2  0  0  0  0
 25 29  1  0  0  0  0
 26 30  2  0  0  0  0
 26 31  1  0  0  0  0
 28 30  1  0  0  0  0
 28 32  1  0  0  0  0
 30102  1  0  0  0  0
 33 34  2  0  0  0  0
 33 35  1  0  0  0  0
 33 86  1  0  0  0  0
 34 36  1  0  0  0  0
 34 37  1  0  0  0  0
 35 38  2  0  0  0  0
 35 39  1  0  0  0  0
 36 40  2  0  0  0  0
 36 41  1  0  0  0  0
 38 40  1  0  0  0  0
 38 42  1  0  0  0  0
 40 98  1  0  0  0  0
 43 44  2  0  0  0  0
 43 45  1  0  0  0  0
 43 84  1  0  0  0  0
 44 46  1  0  0  0  0
 44 47  1  0  0  0  0
 45 48  2  0  0  0  0
 45 49  1  0  0  0  0
 46 50  2  0  0  0  0
 46 51  1  0  0  0  0
 48 50  1  0  0  0  0
 48 52  1  0  0  0  0
 50 90  1  0  0  0  0
```

```
 53 54  2  0  0  0  0
 53 55  1  0  0  0  0
 53 87  1  0  0  0  0
 54 56  1  0  0  0  0
 54 57  1  0  0  0  0
 55 58  2  0  0  0  0
 55 59  1  0  0  0  0
 56 60  2  0  0  0  0
 56 61  1  0  0  0  0
 58 60  1  0  0  0  0
 58 62  1  0  0  0  0
 60110  1  0  0  0  0
 63 64  2  0  0  0  0
 63 65  1  0  0  0  0
 63 88  1  0  0  0  0
 64 66  1  0  0  0  0
 64 67  1  0  0  0  0
 65 68  2  0  0  0  0
 65 69  1  0  0  0  0
 66 70  2  0  0  0  0
 66 71  1  0  0  0  0
 68 70  1  0  0  0  0
 68 72  1  0  0  0  0
 70106  1  0  0  0  0
 73 74  2  0  0  0  0
 73 75  1  0  0  0  0
 73 89  1  0  0  0  0
 74 76  1  0  0  0  0
 74 77  1  0  0  0  0
 75 78  2  0  0  0  0
 75 79  1  0  0  0  0
 76 80  2  0  0  0  0
 76 81  1  0  0  0  0
 78 80  1  0  0  0  0
 78 82  1  0  0  0  0
 80 94  1  0  0  0  0
 90 91  1  0  0  0  0
 90 92  1  0  0  0  0
 90 93  1  0  0  0  0
 94 95  1  0  0  0  0
 94 96  1  0  0  0  0
 94 97  1  0  0  0  0
 98 99  1  0  0  0  0
 98100  1  0  0  0  0
 98101  1  0  0  0  0
102103  1  0  0  0  0
102104  1  0  0  0  0
102105  1  0  0  0  0
106107  1  0  0  0  0
106108  1  0  0  0  0
106109  1  0  0  0  0
110111  1  0  0  0  0
110112  1  0  0  0  0
110113  1  0  0  0  0
M  END
$$$$
mol032
Optimized Catalyst 032
Optimized Catalyst 032
105114  0  0  0  0  0  0  0  0999 V2000
    3.4273    0.7434    0.2136 C   0  0  0  0  0  0  0  0  0  0  0  0
    3.3678   -0.7118   -0.1873 C   0  0  0  0  0  0  0  0  0  0  0  0
    1.6698   -1.5225    0.2006 P   0  0  0  0  0  0  0  0  0  0  0  0
    0.0645    0.0576    0.1111 Rh  0  0  0  0  0  0  0  0  0  0  0  0
   -1.5296    1.6180   -0.2252 P   0  0  0  0  0  0  0  0  0  0  0  0
```

```
   -3.0637    0.9638    0.7027 C   0  0  0  0  0  0  0  0  0  0  0  0
   -3.3007   -0.4614    0.2502 C   0  0  0  0  0  0  0  0  0  0  0  0
   -1.6390   -1.4284    0.2793 P   0  0  0  0  0  0  0  0  0  0  0  0
    1.7052    1.5859    0.1237 P   0  0  0  0  0  0  0  0  0  0  0  0
   -3.9528    1.6032    0.4773 H   0  0  0  0  0  0  0  0  0  0  0  0
   -3.7422   -0.5029   -0.7758 H   0  0  0  0  0  0  0  0  0  0  0  0
    3.5351   -0.8281   -1.2909 H   0  0  0  0  0  0  0  0  0  0  0  0
    4.1296    1.3013   -0.4581 H   0  0  0  0  0  0  0  0  0  0  0  0
   -2.8687    1.0195    1.8049 H   0  0  0  0  0  0  0  0  0  0  0  0
   -4.0135   -0.9727    0.9478 H   0  0  0  0  0  0  0  0  0  0  0  0
    4.1668   -1.2946    0.3386 H   0  0  0  0  0  0  0  0  0  0  0  0
    3.8023    0.8598    1.2631 H   0  0  0  0  0  0  0  0  0  0  0  0
    1.8025    2.7026   -1.3529 C   0  0  0  0  0  0  0  0  0  0  0  0
    1.5380    4.0711   -1.2615 C   0  0  0  0  0  0  0  0  0  0  0  0
    2.1502    2.1154   -2.5780 C   0  0  0  0  0  0  0  0  0  0  0  0
    1.6296    4.8775   -2.4009 C   0  0  0  0  0  0  0  0  0  0  0  0
    1.2399    4.5437   -0.3189 H   0  0  0  0  0  0  0  0  0  0  0  0
    2.2464    2.9014   -3.7269 C   0  0  0  0  0  0  0  0  0  0  0  0
    2.3489    1.0425   -2.6644 H   0  0  0  0  0  0  0  0  0  0  0  0
    1.9862    4.2735   -3.6099 C   0  0  0  0  0  0  0  0  0  0  0  0
    1.4307    5.9498   -2.3430 H   0  0  0  0  0  0  0  0  0  0  0  0
    2.5208    2.4630   -4.6886 H   0  0  0  0  0  0  0  0  0  0  0  0
    1.7375    2.5909    1.6828 C   0  0  0  0  0  0  0  0  0  0  0  0
    1.0054    2.0831    2.7617 C   0  0  0  0  0  0  0  0  0  0  0  0
    2.5254    3.7391    1.8168 C   0  0  0  0  0  0  0  0  0  0  0  0
    1.0236    2.7449    3.9921 C   0  0  0  0  0  0  0  0  0  0  0  0
    0.4107    1.1621    2.6558 H   0  0  0  0  0  0  0  0  0  0  0  0
    2.5464    4.4246    3.0342 C   0  0  0  0  0  0  0  0  0  0  0  0
    3.1431    4.1181    0.9964 H   0  0  0  0  0  0  0  0  0  0  0  0
    1.7899    3.9122    4.0951 C   0  0  0  0  0  0  0  0  0  0  0  0
    0.4635    2.3633    4.8471 H   0  0  0  0  0  0  0  0  0  0  0  0
    3.1443    5.3313    3.1574 H   0  0  0  0  0  0  0  0  0  0  0  0
    1.8745   -2.3802    1.8362 C   0  0  0  0  0  0  0  0  0  0  0  0
    1.3750   -3.6744    2.0239 C   0  0  0  0  0  0  0  0  0  0  0  0
    2.5230   -1.7049    2.8778 C   0  0  0  0  0  0  0  0  0  0  0  0
    1.5439   -4.3204    3.2514 C   0  0  0  0  0  0  0  0  0  0  0  0
    0.8396   -4.2065    1.2335 H   0  0  0  0  0  0  0  0  0  0  0  0
    2.7016   -2.3313    4.1136 C   0  0  0  0  0  0  0  0  0  0  0  0
    2.8938   -0.6834    2.7613 H   0  0  0  0  0  0  0  0  0  0  0  0
    2.2113   -3.6330    4.2711 C   0  0  0  0  0  0  0  0  0  0  0  0
    1.1628   -5.3311    3.4114 H   0  0  0  0  0  0  0  0  0  0  0  0
    3.2140   -1.8216    4.9324 H   0  0  0  0  0  0  0  0  0  0  0  0
    1.6535   -2.8250   -1.1233 C   0  0  0  0  0  0  0  0  0  0  0  0
    0.9978   -2.5197   -2.3206 C   0  0  0  0  0  0  0  0  0  0  0  0
    2.3740   -4.0163   -0.9783 C   0  0  0  0  0  0  0  0  0  0  0  0
    1.0271   -3.4237   -3.3857 C   0  0  0  0  0  0  0  0  0  0  0  0
    0.4492   -1.5767   -2.4405 H   0  0  0  0  0  0  0  0  0  0  0  0
    2.4039   -4.9409   -2.0248 C   0  0  0  0  0  0  0  0  0  0  0  0
    2.9330   -4.2492   -0.0660 H   0  0  0  0  0  0  0  0  0  0  0  0
    1.7250   -4.6235   -3.2074 C   0  0  0  0  0  0  0  0  0  0  0  0
    0.5162   -3.2040   -4.3246 H   0  0  0  0  0  0  0  0  0  0  0  0
    2.9523   -5.8809   -1.9276 H   0  0  0  0  0  0  0  0  0  0  0  0
   -1.7429   -2.2776    1.9263 C   0  0  0  0  0  0  0  0  0  0  0  0
   -1.0062   -1.7296    2.9808 C   0  0  0  0  0  0  0  0  0  0  0  0
   -2.6008   -3.3651    2.1318 C   0  0  0  0  0  0  0  0  0  0  0  0
   -1.0897   -2.2892    4.2591 C   0  0  0  0  0  0  0  0  0  0  0  0
   -0.3500   -0.8624    2.8205 H   0  0  0  0  0  0  0  0  0  0  0  0
   -2.6916   -3.9463    3.3981 C   0  0  0  0  0  0  0  0  0  0  0  0
   -3.2179   -3.7768    1.3265 H   0  0  0  0  0  0  0  0  0  0  0  0
   -1.9282   -3.3948    4.4353 C   0  0  0  0  0  0  0  0  0  0  0  0
   -0.5146   -1.8797    5.0911 H   0  0  0  0  0  0  0  0  0  0  0  0
   -3.3484   -4.8004    3.5782 H   0  0  0  0  0  0  0  0  0  0  0  0
   -1.8400   -2.7202   -1.0407 C   0  0  0  0  0  0  0  0  0  0  0  0
   -2.3265   -2.3249   -2.2940 C   0  0  0  0  0  0  0  0  0  0  0  0
   -1.5152   -4.0576   -0.7890 C   0  0  0  0  0  0  0  0  0  0  0  0
```

```
   -2.5235   -3.2736   -3.2996 C   0  0  0  0  0  0  0  0  0  0  0  0
   -2.5490   -1.2782   -2.5267 H   0  0  0  0  0  0  0  0  0  0  0  0
   -1.7013   -5.0233   -1.7820 C   0  0  0  0  0  0  0  0  0  0  0  0
   -1.1067   -4.3821    0.1716 H   0  0  0  0  0  0  0  0  0  0  0  0
   -2.2105   -4.6088   -3.0172 C   0  0  0  0  0  0  0  0  0  0  0  0
   -2.9135   -2.9826   -4.2772 H   0  0  0  0  0  0  0  0  0  0  0  0
   -1.4555   -6.0714   -1.5990 H   0  0  0  0  0  0  0  0  0  0  0  0
   -1.4237    3.4108    0.2436 C   0  0  0  0  0  0  0  0  0  0  0  0
   -1.4120    4.3903   -0.7558 C   0  0  0  0  0  0  0  0  0  0  0  0
   -1.3953    3.7662    1.5974 C   0  0  0  0  0  0  0  0  0  0  0  0
   -1.3633    5.7434   -0.4076 C   0  0  0  0  0  0  0  0  0  0  0  0
   -1.4293    4.1324   -1.8213 H   0  0  0  0  0  0  0  0  0  0  0  0
   -1.3338    5.1114    1.9671 C   0  0  0  0  0  0  0  0  0  0  0  0
   -1.4168    3.0145    2.3915 H   0  0  0  0  0  0  0  0  0  0  0  0
   -1.3235    6.0741    0.9508 C   0  0  0  0  0  0  0  0  0  0  0  0
   -1.3631    6.5187   -1.1766 H   0  0  0  0  0  0  0  0  0  0  0  0
   -1.3017    5.4031    3.0192 H   0  0  0  0  0  0  0  0  0  0  0  0
   -2.0031    1.6025   -2.0229 C   0  0  0  0  0  0  0  0  0  0  0  0
   -3.1978    2.1825   -2.4671 C   0  0  0  0  0  0  0  0  0  0  0  0
   -1.1209    0.9878   -2.9159 C   0  0  0  0  0  0  0  0  0  0  0  0
   -3.5207    2.1471   -3.8251 C   0  0  0  0  0  0  0  0  0  0  0  0
   -3.8942    2.6713   -1.7794 H   0  0  0  0  0  0  0  0  0  0  0  0
   -1.4228    0.9440   -4.2802 C   0  0  0  0  0  0  0  0  0  0  0  0
   -0.1845    0.5301   -2.5603 H   0  0  0  0  0  0  0  0  0  0  0  0
   -2.6210    1.5294   -4.7036 C   0  0  0  0  0  0  0  0  0  0  0  0
   -4.4483    2.5922   -4.1938 H   0  0  0  0  0  0  0  0  0  0  0  0
   -0.7432    0.4747   -4.9927 H   0  0  0  0  0  0  0  0  0  0  0  0
    2.0757    5.0307   -4.6985 F   0  0  0  0  0  0  0  0  0  0  0  0
    1.8038    4.5596    5.2558 F   0  0  0  0  0  0  0  0  0  0  0  0
   -1.2667    7.3575    1.2918 F   0  0  0  0  0  0  0  0  0  0  0  0
   -2.9218    1.4927   -5.9975 F   0  0  0  0  0  0  0  0  0  0  0  0
   -2.3940   -5.5189   -3.9682 F   0  0  0  0  0  0  0  0  0  0  0  0
   -2.0068   -3.9457    5.6423 F   0  0  0  0  0  0  0  0  0  0  0  0
    2.3777   -4.2373    5.4430 F   0  0  0  0  0  0  0  0  0  0  0  0
    1.7476   -5.4985   -4.2073 F   0  0  0  0  0  0  0  0  0  0  0  0
  1  2  1  0  0  0  0
  1  9  1  0  0  0  0
  1 13  1  0  0  0  0
  1 17  1  0  0  0  0
  2  3  1  0  0  0  0
  2 12  1  0  0  0  0
  2 16  1  0  0  0  0
  3  4  1  0  0  0  0
  3 38  1  0  0  0  0
  3 48  1  0  0  0  0
  4  5  1  0  0  0  0
  4  8  1  0  0  0  0
  4  9  1  0  0  0  0
  5  6  1  0  0  0  0
  5 78  1  0  0  0  0
  5 88  1  0  0  0  0
  6  7  1  0  0  0  0
  6 10  1  0  0  0  0
  6 14  1  0  0  0  0
  7  8  1  0  0  0  0
  7 11  1  0  0  0  0
  7 15  1  0  0  0  0
  8 58  1  0  0  0  0
  8 68  1  0  0  0  0
  9 18  1  0  0  0  0
  9 28  1  0  0  0  0
 18 19  2  0  0  0  0
 18 20  1  0  0  0  0
 19 21  1  0  0  0  0
 19 22  1  0  0  0  0
```

```
 20 23  2  0  0  0  0
 20 24  1  0  0  0  0
 21 25  2  0  0  0  0
 21 26  1  0  0  0  0
 23 25  1  0  0  0  0
 23 27  1  0  0  0  0
 25 98  1  0  0  0  0
 28 29  2  0  0  0  0
 28 30  1  0  0  0  0
 29 31  1  0  0  0  0
 29 32  1  0  0  0  0
 30 33  2  0  0  0  0
 30 34  1  0  0  0  0
 31 35  2  0  0  0  0
 31 36  1  0  0  0  0
 33 35  1  0  0  0  0
 33 37  1  0  0  0  0
 35 99  1  0  0  0  0
 38 39  2  0  0  0  0
 38 40  1  0  0  0  0
 39 41  1  0  0  0  0
 39 42  1  0  0  0  0
 40 43  2  0  0  0  0
 40 44  1  0  0  0  0
 41 45  2  0  0  0  0
 41 46  1  0  0  0  0
 43 45  1  0  0  0  0
 43 47  1  0  0  0  0
 45104  1  0  0  0  0
 48 49  2  0  0  0  0
 48 50  1  0  0  0  0
 49 51  1  0  0  0  0
 49 52  1  0  0  0  0
 50 53  2  0  0  0  0
 50 54  1  0  0  0  0
 51 55  2  0  0  0  0
 51 56  1  0  0  0  0
 53 55  1  0  0  0  0
 53 57  1  0  0  0  0
 55105  1  0  0  0  0
 58 59  2  0  0  0  0
 58 60  1  0  0  0  0
 59 61  1  0  0  0  0
 59 62  1  0  0  0  0
 60 63  2  0  0  0  0
 60 64  1  0  0  0  0
 61 65  2  0  0  0  0
 61 66  1  0  0  0  0
 63 65  1  0  0  0  0
 63 67  1  0  0  0  0
 65103  1  0  0  0  0
 68 69  2  0  0  0  0
 68 70  1  0  0  0  0
 69 71  1  0  0  0  0
 69 72  1  0  0  0  0
 70 73  2  0  0  0  0
 70 74  1  0  0  0  0
 71 75  2  0  0  0  0
 71 76  1  0  0  0  0
 73 75  1  0  0  0  0
 73 77  1  0  0  0  0
 75102  1  0  0  0  0
 78 79  2  0  0  0  0
 78 80  1  0  0  0  0
 79 81  1  0  0  0  0
```

```
 79 82  1  0  0  0  0
 80 83  2  0  0  0  0
 80 84  1  0  0  0  0
 81 85  2  0  0  0  0
 81 86  1  0  0  0  0
 83 85  1  0  0  0  0
 83 87  1  0  0  0  0
 85100  1  0  0  0  0
 88 89  2  0  0  0  0
 88 90  1  0  0  0  0
 89 91  1  0  0  0  0
 89 92  1  0  0  0  0
 90 93  2  0  0  0  0
 90 94  1  0  0  0  0
 91 95  2  0  0  0  0
 91 96  1  0  0  0  0
 93 95  1  0  0  0  0
 93 97  1  0  0  0  0
 95101  1  0  0  0  0
M  END
$$$$
mol033
Optimized Catalyst 033
Optimized Catalyst 033
129138  0  0  0  0  0  0  0  0999 V2000
    3.4365    0.7407    0.2244 C   0  0  0  0  0  0  0  0  0  0  0  0
    3.3801   -0.7129   -0.1827 C   0  0  0  0  0  0  0  0  0  0  0  0
    1.6764   -1.5213    0.1942 P   0  0  0  0  0  0  0  0  0  0  0  0
   -1.5234    1.6192   -0.2189 P   0  0  0  0  0  0  0  0  0  0  0  0
   -3.0644    0.9667    0.7039 C   0  0  0  0  0  0  0  0  0  0  0  0
   -3.3009   -0.4569    0.2462 C   0  0  0  0  0  0  0  0  0  0  0  0
   -1.6364   -1.4217    0.2845 P   0  0  0  0  0  0  0  0  0  0  0  0
    1.7116    1.5820    0.1283 P   0  0  0  0  0  0  0  0  0  0  0  0
   -3.9478    1.6104    0.4742 H   0  0  0  0  0  0  0  0  0  0  0  0
   -3.7306   -0.4946   -0.7848 H   0  0  0  0  0  0  0  0  0  0  0  0
    3.5510   -0.8253   -1.2853 H   0  0  0  0  0  0  0  0  0  0  0  0
    4.1376    1.3038   -0.4419 H   0  0  0  0  0  0  0  0  0  0  0  0
   -2.8713    1.0194    1.8060 H   0  0  0  0  0  0  0  0  0  0  0  0
   -4.0154   -0.9721    0.9367 H   0  0  0  0  0  0  0  0  0  0  0  0
    4.1718   -1.2991    0.3479 H   0  0  0  0  0  0  0  0  0  0  0  0
    3.8017    0.8530    1.2771 H   0  0  0  0  0  0  0  0  0  0  0  0
    1.8099    2.6925   -1.3510 C   0  0  0  0  0  0  0  0  0  0  0  0
    1.5356    4.0586   -1.2663 C   0  0  0  0  0  0  0  0  0  0  0  0
    2.1538    2.1152   -2.5792 C   0  0  0  0  0  0  0  0  0  0  0  0
    1.6201    4.8549   -2.4113 C   0  0  0  0  0  0  0  0  0  0  0  0
    1.2341    4.5315   -0.3256 H   0  0  0  0  0  0  0  0  0  0  0  0
    2.2375    2.9116   -3.7223 C   0  0  0  0  0  0  0  0  0  0  0  0
    2.3570    1.0446   -2.6720 H   0  0  0  0  0  0  0  0  0  0  0  0
    1.9758    4.2918   -3.6486 C   0  0  0  0  0  0  0  0  0  0  0  0
    1.4048    5.9208   -2.3410 H   0  0  0  0  0  0  0  0  0  0  0  0
    2.5075    2.4598   -4.6762 H   0  0  0  0  0  0  0  0  0  0  0  0
    1.7373    2.5902    1.6839 C   0  0  0  0  0  0  0  0  0  0  0  0
    1.0042    2.0941    2.7655 C   0  0  0  0  0  0  0  0  0  0  0  0
    2.5128    3.7444    1.8199 C   0  0  0  0  0  0  0  0  0  0  0  0
    1.0144    2.7770    3.9836 C   0  0  0  0  0  0  0  0  0  0  0  0
    0.4159    1.1686    2.6695 H   0  0  0  0  0  0  0  0  0  0  0  0
    2.5119    4.4342    3.0342 C   0  0  0  0  0  0  0  0  0  0  0  0
    3.1340    4.1222    1.0036 H   0  0  0  0  0  0  0  0  0  0  0  0
    1.7588    3.9619    4.1243 C   0  0  0  0  0  0  0  0  0  0  0  0
    0.4460    2.3896    4.8278 H   0  0  0  0  0  0  0  0  0  0  0  0
    3.1073    5.3412    3.1390 H   0  0  0  0  0  0  0  0  0  0  0  0
    1.8720   -2.3871    1.8253 C   0  0  0  0  0  0  0  0  0  0  0  0
    1.3640   -3.6756    2.0118 C   0  0  0  0  0  0  0  0  0  0  0  0
    2.5032   -1.7209    2.8819 C   0  0  0  0  0  0  0  0  0  0  0  0
    1.5019   -4.3084    3.2504 C   0  0  0  0  0  0  0  0  0  0  0  0
```

```
    0.8431   -4.2102    1.2143 H   0  0  0  0  0  0  0  0  0  0  0  0
    2.6425   -2.3538    4.1179 C   0  0  0  0  0  0  0  0  0  0  0  0
    2.8852   -0.7035    2.7749 H   0  0  0  0  0  0  0  0  0  0  0  0
    2.1420   -3.6542    4.3153 C   0  0  0  0  0  0  0  0  0  0  0  0
    1.0985   -5.3108    3.3886 H   0  0  0  0  0  0  0  0  0  0  0  0
    3.1424   -1.8368    4.9367 H   0  0  0  0  0  0  0  0  0  0  0  0
    1.6495   -2.8131   -1.1382 C   0  0  0  0  0  0  0  0  0  0  0  0
    0.9867   -2.5063   -2.3283 C   0  0  0  0  0  0  0  0  0  0  0  0
    2.3458   -4.0190   -1.0067 C   0  0  0  0  0  0  0  0  0  0  0  0
    0.9793   -3.4265   -3.3803 C   0  0  0  0  0  0  0  0  0  0  0  0
    0.4543   -1.5547   -2.4497 H   0  0  0  0  0  0  0  0  0  0  0  0
    2.3253   -4.9441   -2.0510 C   0  0  0  0  0  0  0  0  0  0  0  0
    2.9171   -4.2606   -0.1062 H   0  0  0  0  0  0  0  0  0  0  0  0
    1.6304   -4.6628   -3.2426 C   0  0  0  0  0  0  0  0  0  0  0  0
    0.4463   -3.1873   -4.2992 H   0  0  0  0  0  0  0  0  0  0  0  0
    2.8557   -5.8904   -1.9457 H   0  0  0  0  0  0  0  0  0  0  0  0
   -1.7328   -2.2627    1.9349 C   0  0  0  0  0  0  0  0  0  0  0  0
   -0.9871   -1.7241    2.9848 C   0  0  0  0  0  0  0  0  0  0  0  0
   -2.5778   -3.3564    2.1531 C   0  0  0  0  0  0  0  0  0  0  0  0
   -1.0491   -2.3057    4.2550 C   0  0  0  0  0  0  0  0  0  0  0  0
   -0.3357   -0.8532    2.8266 H   0  0  0  0  0  0  0  0  0  0  0  0
   -2.6321   -3.9425    3.4175 C   0  0  0  0  0  0  0  0  0  0  0  0
   -3.2058   -3.7671    1.3579 H   0  0  0  0  0  0  0  0  0  0  0  0
   -1.8592   -3.4299    4.4778 C   0  0  0  0  0  0  0  0  0  0  0  0
   -0.4499   -1.8922    5.0649 H   0  0  0  0  0  0  0  0  0  0  0  0
   -3.2811   -4.8013    3.5885 H   0  0  0  0  0  0  0  0  0  0  0  0
   -1.8285   -2.7169   -1.0315 C   0  0  0  0  0  0  0  0  0  0  0  0
   -2.2950   -2.3325   -2.2945 C   0  0  0  0  0  0  0  0  0  0  0  0
   -1.4947   -4.0504   -0.7830 C   0  0  0  0  0  0  0  0  0  0  0  0
   -2.4469   -3.2882   -3.2997 C   0  0  0  0  0  0  0  0  0  0  0  0
   -2.5302   -1.2906   -2.5329 H   0  0  0  0  0  0  0  0  0  0  0  0
   -1.6408   -5.0065   -1.7924 C   0  0  0  0  0  0  0  0  0  0  0  0
   -1.1067   -4.3768    0.1844 H   0  0  0  0  0  0  0  0  0  0  0  0
   -2.1169   -4.6352   -3.0599 C   0  0  0  0  0  0  0  0  0  0  0  0
   -2.8195   -2.9880   -4.2782 H   0  0  0  0  0  0  0  0  0  0  0  0
   -1.3727   -6.0432   -1.5923 H   0  0  0  0  0  0  0  0  0  0  0  0
   -1.4201    3.4098    0.2530 C   0  0  0  0  0  0  0  0  0  0  0  0
   -1.4015    4.3922   -0.7420 C   0  0  0  0  0  0  0  0  0  0  0  0
   -1.3883    3.7744    1.6019 C   0  0  0  0  0  0  0  0  0  0  0  0
   -1.3470    5.7414   -0.3846 C   0  0  0  0  0  0  0  0  0  0  0  0
   -1.4152    4.1358   -1.8068 H   0  0  0  0  0  0  0  0  0  0  0  0
   -1.3213    5.1230    1.9581 C   0  0  0  0  0  0  0  0  0  0  0  0
   -1.4076    3.0285    2.4003 H   0  0  0  0  0  0  0  0  0  0  0  0
   -1.3051    6.1207    0.9683 C   0  0  0  0  0  0  0  0  0  0  0  0
   -1.3347    6.5040   -1.1625 H   0  0  0  0  0  0  0  0  0  0  0  0
   -1.2781    5.3986    3.0114 H   0  0  0  0  0  0  0  0  0  0  0  0
   -1.9919    1.6025   -2.0172 C   0  0  0  0  0  0  0  0  0  0  0  0
   -3.1842    2.1798   -2.4683 C   0  0  0  0  0  0  0  0  0  0  0  0
   -1.1160    0.9921   -2.9158 C   0  0  0  0  0  0  0  0  0  0  0  0
   -3.4980    2.1376   -3.8264 C   0  0  0  0  0  0  0  0  0  0  0  0
   -3.8806    2.6707   -1.7845 H   0  0  0  0  0  0  0  0  0  0  0  0
   -1.4287    0.9538   -4.2786 C   0  0  0  0  0  0  0  0  0  0  0  0
   -0.1768    0.5360   -2.5672 H   0  0  0  0  0  0  0  0  0  0  0  0
   -2.6227    1.5258   -4.7455 C   0  0  0  0  0  0  0  0  0  0  0  0
   -4.4266    2.5849   -4.1817 H   0  0  0  0  0  0  0  0  0  0  0  0
   -0.7378    0.4830   -4.9761 H   0  0  0  0  0  0  0  0  0  0  0  0
    1.5903   -5.6771   -4.3384 C   0  0  0  0  0  0  0  0  0  0  0  0
    2.6272   -5.8890   -4.6989 H   0  0  0  0  0  0  0  0  0  0  0  0
    1.1444   -6.6280   -3.9576 H   0  0  0  0  0  0  0  0  0  0  0  0
    0.9839   -5.3275   -5.2053 H   0  0  0  0  0  0  0  0  0  0  0  0
   -2.2690   -5.6457   -4.1493 C   0  0  0  0  0  0  0  0  0  0  0  0
   -1.7656   -6.6068   -3.8938 H   0  0  0  0  0  0  0  0  0  0  0  0
   -3.3560   -5.8516   -4.3160 H   0  0  0  0  0  0  0  0  0  0  0  0
   -1.8296   -5.2621   -5.1007 H   0  0  0  0  0  0  0  0  0  0  0  0
   -1.9076   -4.0877    5.8180 C   0  0  0  0  0  0  0  0  0  0  0  0
```

```
   -2.9545   -4.0758    6.2105 H   0  0  0  0  0  0  0  0  0  0  0  0
   -1.5726   -5.1499    5.7304 H   0  0  0  0  0  0  0  0  0  0  0  0
   -1.2522   -3.5721    6.5574 H   0  0  0  0  0  0  0  0  0  0  0  0
    2.2915   -4.3160    5.6458 C   0  0  0  0  0  0  0  0  0  0  0  0
    3.3736   -4.5224    5.8412 H   0  0  0  0  0  0  0  0  0  0  0  0
    1.9043   -3.6504    6.4541 H   0  0  0  0  0  0  0  0  0  0  0  0
    1.7377   -5.2822    5.6927 H   0  0  0  0  0  0  0  0  0  0  0  0
   -1.2291    7.5653    1.3392 C   0  0  0  0  0  0  0  0  0  0  0  0
   -1.9464    8.1649    0.7277 H   0  0  0  0  0  0  0  0  0  0  0  0
   -1.4723    7.7278    2.4154 H   0  0  0  0  0  0  0  0  0  0  0  0
   -0.1953    7.9453    1.1493 H   0  0  0  0  0  0  0  0  0  0  0  0
    2.0694    5.1575   -4.8617 C   0  0  0  0  0  0  0  0  0  0  0  0
    1.0860    5.6508   -5.0560 H   0  0  0  0  0  0  0  0  0  0  0  0
    2.8408    5.9510   -4.7009 H   0  0  0  0  0  0  0  0  0  0  0  0
    2.3538    4.5746   -5.7685 H   0  0  0  0  0  0  0  0  0  0  0  0
   -2.9777    1.4907   -6.1956 C   0  0  0  0  0  0  0  0  0  0  0  0
   -3.9203    0.9078   -6.3412 H   0  0  0  0  0  0  0  0  0  0  0  0
   -3.1424    2.5298   -6.5740 H   0  0  0  0  0  0  0  0  0  0  0  0
   -2.1760    1.0194   -6.8098 H   0  0  0  0  0  0  0  0  0  0  0  0
    1.7708    4.6924    5.4265 C   0  0  0  0  0  0  0  0  0  0  0  0
    2.5655    4.2569    6.0837 H   0  0  0  0  0  0  0  0  0  0  0  0
    1.9897    5.7771    5.2841 H   0  0  0  0  0  0  0  0  0  0  0  0
    0.7901    4.5999    5.9501 H   0  0  0  0  0  0  0  0  0  0  0  0
    0.0701    0.0587    0.1153 Rh  0  0  0  0  0  0  0  0  0  0  0  0
  1  2  1  0  0  0  0
  1  8  1  0  0  0  0
  1 12  1  0  0  0  0
  1 16  1  0  0  0  0
  2  3  1  0  0  0  0
  2 11  1  0  0  0  0
  2 15  1  0  0  0  0
  3 37  1  0  0  0  0
  3 47  1  0  0  0  0
  3129  1  0  0  0  0
  4  5  1  0  0  0  0
  4 77  1  0  0  0  0
  4 87  1  0  0  0  0
  4129  1  0  0  0  0
  5  6  1  0  0  0  0
  5  9  1  0  0  0  0
  5 13  1  0  0  0  0
  6  7  1  0  0  0  0
  6 10  1  0  0  0  0
  6 14  1  0  0  0  0
  7 57  1  0  0  0  0
  7 67  1  0  0  0  0
  7129  1  0  0  0  0
  8 17  1  0  0  0  0
  8 27  1  0  0  0  0
  8129  1  0  0  0  0
 17 18  2  0  0  0  0
 17 19  1  0  0  0  0
 18 20  1  0  0  0  0
 18 21  1  0  0  0  0
 19 22  2  0  0  0  0
 19 23  1  0  0  0  0
 20 24  2  0  0  0  0
 20 25  1  0  0  0  0
 22 24  1  0  0  0  0
 22 26  1  0  0  0  0
 24117  1  0  0  0  0
 27 28  2  0  0  0  0
 27 29  1  0  0  0  0
 28 30  1  0  0  0  0
 28 31  1  0  0  0  0
```

```
 29 32  2  0  0  0  0
 29 33  1  0  0  0  0
 30 34  2  0  0  0  0
 30 35  1  0  0  0  0
 32 34  1  0  0  0  0
 32 36  1  0  0  0  0
 34125  1  0  0  0  0
 37 38  2  0  0  0  0
 37 39  1  0  0  0  0
 38 40  1  0  0  0  0
 38 41  1  0  0  0  0
 39 42  2  0  0  0  0
 39 43  1  0  0  0  0
 40 44  2  0  0  0  0
 40 45  1  0  0  0  0
 42 44  1  0  0  0  0
 42 46  1  0  0  0  0
 44109  1  0  0  0  0
 47 48  2  0  0  0  0
 47 49  1  0  0  0  0
 48 50  1  0  0  0  0
 48 51  1  0  0  0  0
 49 52  2  0  0  0  0
 49 53  1  0  0  0  0
 50 54  2  0  0  0  0
 50 55  1  0  0  0  0
 52 54  1  0  0  0  0
 52 56  1  0  0  0  0
 54 97  1  0  0  0  0
 57 58  2  0  0  0  0
 57 59  1  0  0  0  0
 58 60  1  0  0  0  0
 58 61  1  0  0  0  0
 59 62  2  0  0  0  0
 59 63  1  0  0  0  0
 60 64  2  0  0  0  0
 60 65  1  0  0  0  0
 62 64  1  0  0  0  0
 62 66  1  0  0  0  0
 64105  1  0  0  0  0
 67 68  2  0  0  0  0
 67 69  1  0  0  0  0
 68 70  1  0  0  0  0
 68 71  1  0  0  0  0
 69 72  2  0  0  0  0
 69 73  1  0  0  0  0
 70 74  2  0  0  0  0
 70 75  1  0  0  0  0
 72 74  1  0  0  0  0
 72 76  1  0  0  0  0
 74101  1  0  0  0  0
 77 78  2  0  0  0  0
 77 79  1  0  0  0  0
 78 80  1  0  0  0  0
 78 81  1  0  0  0  0
 79 82  2  0  0  0  0
 79 83  1  0  0  0  0
 80 84  2  0  0  0  0
 80 85  1  0  0  0  0
 82 84  1  0  0  0  0
 82 86  1  0  0  0  0
 84113  1  0  0  0  0
 87 88  2  0  0  0  0
 87 89  1  0  0  0  0
 88 90  1  0  0  0  0
```

```
 88 91  1  0  0  0  0
 89 92  2  0  0  0  0
 89 93  1  0  0  0  0
 90 94  2  0  0  0  0
 90 95  1  0  0  0  0
 92 94  1  0  0  0  0
 92 96  1  0  0  0  0
 94121  1  0  0  0  0
 97 98  1  0  0  0  0
 97 99  1  0  0  0  0
 97100  1  0  0  0  0
101102  1  0  0  0  0
101103  1  0  0  0  0
101104  1  0  0  0  0
105106  1  0  0  0  0
105107  1  0  0  0  0
105108  1  0  0  0  0
109110  1  0  0  0  0
109111  1  0  0  0  0
109112  1  0  0  0  0
113114  1  0  0  0  0
113115  1  0  0  0  0
113116  1  0  0  0  0
117118  1  0  0  0  0
117119  1  0  0  0  0
117120  1  0  0  0  0
121122  1  0  0  0  0
121123  1  0  0  0  0
121124  1  0  0  0  0
125126  1  0  0  0  0
125127  1  0  0  0  0
125128  1  0  0  0  0
M  END
$$$$
mol034
Optimized Catalyst 034
Optimized Catalyst 034
105114  0  0  0  0  0  0  0  0999 V2000
    3.6251    0.7316    0.3432 C   0  0  0  0  0  0  0  0  0  0  0  0
    3.5746   -0.6912   -0.1854 C   0  0  0  0  0  0  0  0  0  0  0  0
    1.9996   -1.7030    0.2454 P   0  0  0  0  0  0  0  0  0  0  0  0
   -1.8695    1.7829   -0.2622 P   0  0  0  0  0  0  0  0  0  0  0  0
   -3.3123    0.9298    0.6657 C   0  0  0  0  0  0  0  0  0  0  0  0
   -3.5243   -0.4952    0.1805 C   0  0  0  0  0  0  0  0  0  0  0  0
   -1.9585   -1.6054    0.3154 P   0  0  0  0  0  0  0  0  0  0  0  0
    2.0354    1.7801    0.0957 P   0  0  0  0  0  0  0  0  0  0  0  0
   -4.2524    1.5180    0.4832 H   0  0  0  0  0  0  0  0  0  0  0  0
   -3.8875   -0.5122   -0.8802 H   0  0  0  0  0  0  0  0  0  0  0  0
    3.6607   -0.7059   -1.3066 H   0  0  0  0  0  0  0  0  0  0  0  0
    4.4724    1.2703   -0.1636 H   0  0  0  0  0  0  0  0  0  0  0  0
   -3.1116    0.9661    1.7684 H   0  0  0  0  0  0  0  0  0  0  0  0
   -4.3244   -0.9702    0.8128 H   0  0  0  0  0  0  0  0  0  0  0  0
    4.4607   -1.2544    0.2186 H   0  0  0  0  0  0  0  0  0  0  0  0
    3.8441    0.7466    1.4452 H   0  0  0  0  0  0  0  0  0  0  0  0
    2.2038    2.8318   -1.4150 C   0  0  0  0  0  0  0  0  0  0  0  0
    1.5312    4.0592   -1.4316 C   0  0  0  0  0  0  0  0  0  0  0  0
    2.9628    2.4122   -2.5102 C   0  0  0  0  0  0  0  0  0  0  0  0
    1.6223    4.8757   -2.5631 C   0  0  0  0  0  0  0  0  0  0  0  0
    0.9382    4.4106   -0.5802 H   0  0  0  0  0  0  0  0  0  0  0  0
    3.0534    3.2397   -3.6359 C   0  0  0  0  0  0  0  0  0  0  0  0
    3.4929    1.4590   -2.5227 H   0  0  0  0  0  0  0  0  0  0  0  0
    2.3841    4.4679   -3.6638 C   0  0  0  0  0  0  0  0  0  0  0  0
    1.1012    5.8345   -2.5853 H   0  0  0  0  0  0  0  0  0  0  0  0
    3.6504    2.9259   -4.4948 H   0  0  0  0  0  0  0  0  0  0  0  0
    2.0524    2.9215    1.5577 C   0  0  0  0  0  0  0  0  0  0  0  0
```

```
    1.3048    2.5801    2.6882 C   0  0  0  0  0  0  0  0  0  0  0  0
    2.8296    4.0825    1.5292 C   0  0  0  0  0  0  0  0  0  0  0  0
    1.3243    3.4251    3.8030 C   0  0  0  0  0  0  0  0  0  0  0  0
    0.7030    1.6643    2.7333 H   0  0  0  0  0  0  0  0  0  0  0  0
    2.8417    4.9234    2.6482 C   0  0  0  0  0  0  0  0  0  0  0  0
    3.4317    4.3578    0.6585 H   0  0  0  0  0  0  0  0  0  0  0  0
    2.0897    4.5968    3.7813 C   0  0  0  0  0  0  0  0  0  0  0  0
    0.7451    3.1704    4.6919 H   0  0  0  0  0  0  0  0  0  0  0  0
    3.4434    5.8349    2.6367 H   0  0  0  0  0  0  0  0  0  0  0  0
    2.2114   -2.5456    1.8807 C   0  0  0  0  0  0  0  0  0  0  0  0
    1.4842   -3.7228    2.0983 C   0  0  0  0  0  0  0  0  0  0  0  0
    3.0491   -2.0268    2.8701 C   0  0  0  0  0  0  0  0  0  0  0  0
    1.6013   -4.3852    3.3241 C   0  0  0  0  0  0  0  0  0  0  0  0
    0.8255   -4.1494    1.3370 H   0  0  0  0  0  0  0  0  0  0  0  0
    3.1610   -2.6982    4.0940 C   0  0  0  0  0  0  0  0  0  0  0  0
    3.6282   -1.1142    2.7273 H   0  0  0  0  0  0  0  0  0  0  0  0
    2.4390   -3.8737    4.3218 C   0  0  0  0  0  0  0  0  0  0  0  0
    1.0377   -5.3028    3.5030 H   0  0  0  0  0  0  0  0  0  0  0  0
    3.8184   -2.3034    4.8719 H   0  0  0  0  0  0  0  0  0  0  0  0
    1.9958   -3.0462   -1.0332 C   0  0  0  0  0  0  0  0  0  0  0  0
    1.2119   -2.9094   -2.1811 C   0  0  0  0  0  0  0  0  0  0  0  0
    2.8070   -4.1697   -0.8428 C   0  0  0  0  0  0  0  0  0  0  0  0
    1.2300   -3.9223   -3.1472 C   0  0  0  0  0  0  0  0  0  0  0  0
    0.5758   -2.0352   -2.3562 H   0  0  0  0  0  0  0  0  0  0  0  0
    2.8185   -5.1771   -1.8134 C   0  0  0  0  0  0  0  0  0  0  0  0
    3.4352   -4.2891    0.0446 H   0  0  0  0  0  0  0  0  0  0  0  0
    2.0294   -5.0551   -2.9624 C   0  0  0  0  0  0  0  0  0  0  0  0
    0.6153   -3.8287   -4.0440 H   0  0  0  0  0  0  0  0  0  0  0  0
    3.4477   -6.0591   -1.6752 H   0  0  0  0  0  0  0  0  0  0  0  0
   -2.1507   -2.5103    1.9218 C   0  0  0  0  0  0  0  0  0  0  0  0
   -1.2768   -2.2452    2.9776 C   0  0  0  0  0  0  0  0  0  0  0  0
   -3.1949   -3.4315    2.0635 C   0  0  0  0  0  0  0  0  0  0  0  0
   -1.4386   -2.9280    4.1901 C   0  0  0  0  0  0  0  0  0  0  0  0
   -0.4608   -1.5187    2.8972 H   0  0  0  0  0  0  0  0  0  0  0  0
   -3.3526   -4.1043    3.2790 C   0  0  0  0  0  0  0  0  0  0  0  0
   -3.8955   -3.6467    1.2521 H   0  0  0  0  0  0  0  0  0  0  0  0
   -2.4742   -3.8548    4.3403 C   0  0  0  0  0  0  0  0  0  0  0  0
   -0.7528   -2.7350    5.0173 H   0  0  0  0  0  0  0  0  0  0  0  0
   -4.1652   -4.8239    3.4018 H   0  0  0  0  0  0  0  0  0  0  0  0
   -2.0707   -2.8540   -1.0482 C   0  0  0  0  0  0  0  0  0  0  0  0
   -2.4179   -2.4407   -2.3395 C   0  0  0  0  0  0  0  0  0  0  0  0
   -1.7766   -4.1933   -0.7765 C   0  0  0  0  0  0  0  0  0  0  0  0
   -2.4934   -3.3902   -3.3635 C   0  0  0  0  0  0  0  0  0  0  0  0
   -2.6305   -1.3974   -2.5871 H   0  0  0  0  0  0  0  0  0  0  0  0
   -1.8508   -5.1354   -1.8094 C   0  0  0  0  0  0  0  0  0  0  0  0
   -1.4903   -4.5359    0.2216 H   0  0  0  0  0  0  0  0  0  0  0  0
   -2.2137   -4.7363   -3.0990 C   0  0  0  0  0  0  0  0  0  0  0  0
   -2.7754   -3.0819   -4.3720 H   0  0  0  0  0  0  0  0  0  0  0  0
   -1.6266   -6.1843   -1.6053 H   0  0  0  0  0  0  0  0  0  0  0  0
   -1.7365    3.5264    0.3263 C   0  0  0  0  0  0  0  0  0  0  0  0
   -1.6832    4.5593   -0.6150 C   0  0  0  0  0  0  0  0  0  0  0  0
   -1.6605    3.7946    1.6968 C   0  0  0  0  0  0  0  0  0  0  0  0
   -1.5522    5.8811   -0.1738 C   0  0  0  0  0  0  0  0  0  0  0  0
   -1.7399    4.3703   -1.6920 H   0  0  0  0  0  0  0  0  0  0  0  0
   -1.5266    5.1187    2.1274 C   0  0  0  0  0  0  0  0  0  0  0  0
   -1.7019    3.0056    2.4503 H   0  0  0  0  0  0  0  0  0  0  0  0
   -1.4735    6.1606    1.1941 C   0  0  0  0  0  0  0  0  0  0  0  0
   -1.5172    6.6956   -0.9001 H   0  0  0  0  0  0  0  0  0  0  0  0
   -1.4639    5.3395    3.1948 H   0  0  0  0  0  0  0  0  0  0  0  0
   -2.3890    1.8220   -2.0438 C   0  0  0  0  0  0  0  0  0  0  0  0
   -3.6019    2.4154   -2.4059 C   0  0  0  0  0  0  0  0  0  0  0  0
   -1.5569    1.2371   -3.0019 C   0  0  0  0  0  0  0  0  0  0  0  0
   -3.9890    2.4113   -3.7507 C   0  0  0  0  0  0  0  0  0  0  0  0
   -4.2593    2.8904   -1.6737 H   0  0  0  0  0  0  0  0  0  0  0  0
   -1.9507    1.2393   -4.3450 C   0  0  0  0  0  0  0  0  0  0  0  0
```

```
   -0.5937    0.7787   -2.7381 H   0  0  0  0  0  0  0  0  0  0  0  0
   -3.1659    1.8241   -4.7180 C   0  0  0  0  0  0  0  0  0  0  0  0
   -4.9354    2.8704   -4.0462 H   0  0  0  0  0  0  0  0  0  0  0  0
   -1.3066    0.7901   -5.1033 H   0  0  0  0  0  0  0  0  0  0  0  0
    0.0663    0.0592    0.1142 Ir  0  0  0  0  0  0  0  0  0  0  0  0
   -3.4713    1.8260   -5.7676 H   0  0  0  0  0  0  0  0  0  0  0  0
   -2.2794   -5.4748   -3.9013 H   0  0  0  0  0  0  0  0  0  0  0  0
    2.0405   -5.8450   -3.7174 H   0  0  0  0  0  0  0  0  0  0  0  0
   -1.3742    7.1939    1.5358 H   0  0  0  0  0  0  0  0  0  0  0  0
    2.1040    5.2551    4.6537 H   0  0  0  0  0  0  0  0  0  0  0  0
   -2.6023   -4.3829    5.2885 H   0  0  0  0  0  0  0  0  0  0  0  0
    2.5316   -4.3949    5.2777 H   0  0  0  0  0  0  0  0  0  0  0  0
    2.4577    5.1094   -4.5456 H   0  0  0  0  0  0  0  0  0  0  0  0
  1  2  1  0  0  0  0
  1  8  1  0  0  0  0
  1 12  1  0  0  0  0
  1 16  1  0  0  0  0
  2  3  1  0  0  0  0
  2 11  1  0  0  0  0
  2 15  1  0  0  0  0
  3 37  1  0  0  0  0
  3 47  1  0  0  0  0
  3 97  1  0  0  0  0
  4  5  1  0  0  0  0
  4 77  1  0  0  0  0
  4 87  1  0  0  0  0
  4 97  1  0  0  0  0
  5  6  1  0  0  0  0
  5  9  1  0  0  0  0
  5 13  1  0  0  0  0
  6  7  1  0  0  0  0
  6 10  1  0  0  0  0
  6 14  1  0  0  0  0
  7 57  1  0  0  0  0
  7 67  1  0  0  0  0
  7 97  1  0  0  0  0
  8 17  1  0  0  0  0
  8 27  1  0  0  0  0
  8 97  1  0  0  0  0
 17 18  2  0  0  0  0
 17 19  1  0  0  0  0
 18 20  1  0  0  0  0
 18 21  1  0  0  0  0
 19 22  2  0  0  0  0
 19 23  1  0  0  0  0
 20 24  2  0  0  0  0
 20 25  1  0  0  0  0
 22 24  1  0  0  0  0
 22 26  1  0  0  0  0
 24105  1  0  0  0  0
 27 28  2  0  0  0  0
 27 29  1  0  0  0  0
 28 30  1  0  0  0  0
 28 31  1  0  0  0  0
 29 32  2  0  0  0  0
 29 33  1  0  0  0  0
 30 34  2  0  0  0  0
 30 35  1  0  0  0  0
 32 34  1  0  0  0  0
 32 36  1  0  0  0  0
 34102  1  0  0  0  0
 37 38  2  0  0  0  0
 37 39  1  0  0  0  0
 38 40  1  0  0  0  0
 38 41  1  0  0  0  0
```

```
 39 42  2  0  0  0  0
 39 43  1  0  0  0  0
 40 44  2  0  0  0  0
 40 45  1  0  0  0  0
 42 44  1  0  0  0  0
 42 46  1  0  0  0  0
 44104  1  0  0  0  0
 47 48  2  0  0  0  0
 47 49  1  0  0  0  0
 48 50  1  0  0  0  0
 48 51  1  0  0  0  0
 49 52  2  0  0  0  0
 49 53  1  0  0  0  0
 50 54  2  0  0  0  0
 50 55  1  0  0  0  0
 52 54  1  0  0  0  0
 52 56  1  0  0  0  0
 54100  1  0  0  0  0
 57 58  2  0  0  0  0
 57 59  1  0  0  0  0
 58 60  1  0  0  0  0
 58 61  1  0  0  0  0
 59 62  2  0  0  0  0
 59 63  1  0  0  0  0
 60 64  2  0  0  0  0
 60 65  1  0  0  0  0
 62 64  1  0  0  0  0
 62 66  1  0  0  0  0
 64103  1  0  0  0  0
 67 68  2  0  0  0  0
 67 69  1  0  0  0  0
 68 70  1  0  0  0  0
 68 71  1  0  0  0  0
 69 72  2  0  0  0  0
 69 73  1  0  0  0  0
 70 74  2  0  0  0  0
 70 75  1  0  0  0  0
 72 74  1  0  0  0  0
 72 76  1  0  0  0  0
 74 99  1  0  0  0  0
 77 78  2  0  0  0  0
 77 79  1  0  0  0  0
 78 80  1  0  0  0  0
 78 81  1  0  0  0  0
 79 82  2  0  0  0  0
 79 83  1  0  0  0  0
 80 84  2  0  0  0  0
 80 85  1  0  0  0  0
 82 84  1  0  0  0  0
 82 86  1  0  0  0  0
 84101  1  0  0  0  0
 87 88  2  0  0  0  0
 87 89  1  0  0  0  0
 88 90  1  0  0  0  0
 88 91  1  0  0  0  0
 89 92  2  0  0  0  0
 89 93  1  0  0  0  0
 90 94  2  0  0  0  0
 90 95  1  0  0  0  0
 92 94  1  0  0  0  0
 92 96  1  0  0  0  0
 94 98  1  0  0  0  0
M  END
$$$$
mol035
```

```
Optimized Catalyst 035
Optimized Catalyst 035
105114  0  0  0  0  0  0  0  0999 V2000
    3.6253    0.7242    0.3472 C   0  0  0  0  0  0  0  0  0  0  0  0
    3.5730   -0.6959   -0.1881 C   0  0  0  0  0  0  0  0  0  0  0  0
    1.9966   -1.7092    0.2325 P   0  0  0  0  0  0  0  0  0  0  0  0
   -1.8766    1.7803   -0.2622 P   0  0  0  0  0  0  0  0  0  0  0  0
   -3.3221    0.9220    0.6556 C   0  0  0  0  0  0  0  0  0  0  0  0
   -3.5269   -0.5056    0.1747 C   0  0  0  0  0  0  0  0  0  0  0  0
   -1.9591   -1.6104    0.3250 P   0  0  0  0  0  0  0  0  0  0  0  0
    2.0380    1.7769    0.1053 P   0  0  0  0  0  0  0  0  0  0  0  0
   -4.2639    1.5057    0.4637 H   0  0  0  0  0  0  0  0  0  0  0  0
   -3.8867   -0.5280   -0.8872 H   0  0  0  0  0  0  0  0  0  0  0  0
    3.6638   -0.7049   -1.3089 H   0  0  0  0  0  0  0  0  0  0  0  0
    4.4754    1.2640   -0.1550 H   0  0  0  0  0  0  0  0  0  0  0  0
   -3.1333    0.9623    1.7605 H   0  0  0  0  0  0  0  0  0  0  0  0
   -4.3310   -0.9777    0.8051 H   0  0  0  0  0  0  0  0  0  0  0  0
    4.4596   -1.2623    0.2116 H   0  0  0  0  0  0  0  0  0  0  0  0
    3.8468    0.7324    1.4489 H   0  0  0  0  0  0  0  0  0  0  0  0
    2.2002    2.8256   -1.4057 C   0  0  0  0  0  0  0  0  0  0  0  0
    1.5529    4.0696   -1.4068 C   0  0  0  0  0  0  0  0  0  0  0  0
    2.9306    2.3894   -2.5171 C   0  0  0  0  0  0  0  0  0  0  0  0
    1.6279    4.8932   -2.5305 C   0  0  0  0  0  0  0  0  0  0  0  0
    0.9855    4.4301   -0.5400 H   0  0  0  0  0  0  0  0  0  0  0  0
    3.0199    3.2026   -3.6497 C   0  0  0  0  0  0  0  0  0  0  0  0
    3.4433    1.4245   -2.5362 H   0  0  0  0  0  0  0  0  0  0  0  0
    2.3647    4.4401   -3.6323 C   0  0  0  0  0  0  0  0  0  0  0  0
    1.1342    5.8677   -2.5464 H   0  0  0  0  0  0  0  0  0  0  0  0
    3.5908    2.8839   -4.5259 H   0  0  0  0  0  0  0  0  0  0  0  0
    2.0598    2.9234    1.5588 C   0  0  0  0  0  0  0  0  0  0  0  0
    1.2769    2.6145    2.6770 C   0  0  0  0  0  0  0  0  0  0  0  0
    2.8742    4.0632    1.5341 C   0  0  0  0  0  0  0  0  0  0  0  0
    1.2820    3.4637    3.7854 C   0  0  0  0  0  0  0  0  0  0  0  0
    0.6514    1.7125    2.7164 H   0  0  0  0  0  0  0  0  0  0  0  0
    2.8881    4.9273    2.6308 C   0  0  0  0  0  0  0  0  0  0  0  0
    3.5085    4.3047    0.6737 H   0  0  0  0  0  0  0  0  0  0  0  0
    2.0855    4.6104    3.7332 C   0  0  0  0  0  0  0  0  0  0  0  0
    0.6840    3.2393    4.6711 H   0  0  0  0  0  0  0  0  0  0  0  0
    3.5130    5.8253    2.6302 H   0  0  0  0  0  0  0  0  0  0  0  0
    2.1996   -2.5610    1.8611 C   0  0  0  0  0  0  0  0  0  0  0  0
    1.4825   -3.7501    2.0642 C   0  0  0  0  0  0  0  0  0  0  0  0
    3.0213   -2.0351    2.8637 C   0  0  0  0  0  0  0  0  0  0  0  0
    1.5799   -4.4237    3.2821 C   0  0  0  0  0  0  0  0  0  0  0  0
    0.8402   -4.1788    1.2883 H   0  0  0  0  0  0  0  0  0  0  0  0
    3.1311   -2.6958    4.0902 C   0  0  0  0  0  0  0  0  0  0  0  0
    3.5930   -1.1148    2.7264 H   0  0  0  0  0  0  0  0  0  0  0  0
    2.4064   -3.8789    4.2736 C   0  0  0  0  0  0  0  0  0  0  0  0
    1.0290   -5.3511    3.4585 H   0  0  0  0  0  0  0  0  0  0  0  0
    3.7714   -2.3013    4.8841 H   0  0  0  0  0  0  0  0  0  0  0  0
    1.9882   -3.0399   -1.0537 C   0  0  0  0  0  0  0  0  0  0  0  0
    1.2096   -2.8822   -2.2059 C   0  0  0  0  0  0  0  0  0  0  0  0
    2.7895   -4.1751   -0.8693 C   0  0  0  0  0  0  0  0  0  0  0  0
    1.2058   -3.8787   -3.1842 C   0  0  0  0  0  0  0  0  0  0  0  0
    0.5896   -1.9932   -2.3737 H   0  0  0  0  0  0  0  0  0  0  0  0
    2.7943   -5.1842   -1.8336 C   0  0  0  0  0  0  0  0  0  0  0  0
    3.4210   -4.3016    0.0174 H   0  0  0  0  0  0  0  0  0  0  0  0
    1.9958   -5.0153   -2.9717 C   0  0  0  0  0  0  0  0  0  0  0  0
    0.6042   -3.7755   -4.0903 H   0  0  0  0  0  0  0  0  0  0  0  0
    3.4090   -6.0799   -1.7063 H   0  0  0  0  0  0  0  0  0  0  0  0
   -2.1423   -2.5040    1.9350 C   0  0  0  0  0  0  0  0  0  0  0  0
   -1.2645   -2.2267    2.9881 C   0  0  0  0  0  0  0  0  0  0  0  0
   -3.1821   -3.4334    2.0833 C   0  0  0  0  0  0  0  0  0  0  0  0
   -1.4041   -2.8976    4.2060 C   0  0  0  0  0  0  0  0  0  0  0  0
   -0.4548   -1.4919    2.8967 H   0  0  0  0  0  0  0  0  0  0  0  0
   -3.3383   -4.1118    3.2923 C   0  0  0  0  0  0  0  0  0  0  0  0
```

```
   -3.8860   -3.6523    1.2730 H   0  0  0  0  0  0  0  0  0  0  0  0
   -2.4411   -3.8292    4.3310 C   0  0  0  0  0  0  0  0  0  0  0  0
   -0.7241   -2.6992    5.0382 H   0  0  0  0  0  0  0  0  0  0  0  0
   -4.1424   -4.8407    3.4281 H   0  0  0  0  0  0  0  0  0  0  0  0
   -2.0566   -2.8724   -1.0238 C   0  0  0  0  0  0  0  0  0  0  0  0
   -2.4089   -2.4757   -2.3212 C   0  0  0  0  0  0  0  0  0  0  0  0
   -1.7480   -4.2081   -0.7358 C   0  0  0  0  0  0  0  0  0  0  0  0
   -2.4701   -3.4228   -3.3446 C   0  0  0  0  0  0  0  0  0  0  0  0
   -2.6367   -1.4356   -2.5748 H   0  0  0  0  0  0  0  0  0  0  0  0
   -1.8004   -5.1689   -1.7481 C   0  0  0  0  0  0  0  0  0  0  0  0
   -1.4630   -4.5347    0.2698 H   0  0  0  0  0  0  0  0  0  0  0  0
   -2.1654   -4.7548   -3.0341 C   0  0  0  0  0  0  0  0  0  0  0  0
   -2.7551   -3.1356   -4.3599 H   0  0  0  0  0  0  0  0  0  0  0  0
   -1.5671   -6.2164   -1.5407 H   0  0  0  0  0  0  0  0  0  0  0  0
   -1.7479    3.5200    0.3311 C   0  0  0  0  0  0  0  0  0  0  0  0
   -1.6562    4.5503   -0.6139 C   0  0  0  0  0  0  0  0  0  0  0  0
   -1.7180    3.7921    1.7052 C   0  0  0  0  0  0  0  0  0  0  0  0
   -1.5225    5.8734   -0.1875 C   0  0  0  0  0  0  0  0  0  0  0  0
   -1.6840    4.3548   -1.6928 H   0  0  0  0  0  0  0  0  0  0  0  0
   -1.5839    5.1085    2.1491 C   0  0  0  0  0  0  0  0  0  0  0  0
   -1.7977    3.0029    2.4577 H   0  0  0  0  0  0  0  0  0  0  0  0
   -1.4898    6.1247    1.1891 C   0  0  0  0  0  0  0  0  0  0  0  0
   -1.4576    6.6911   -0.9098 H   0  0  0  0  0  0  0  0  0  0  0  0
   -1.5584    5.3417    3.2169 H   0  0  0  0  0  0  0  0  0  0  0  0
   -2.3852    1.8335   -2.0433 C   0  0  0  0  0  0  0  0  0  0  0  0
   -3.6070    2.4159   -2.4053 C   0  0  0  0  0  0  0  0  0  0  0  0
   -1.5337    1.2775   -3.0048 C   0  0  0  0  0  0  0  0  0  0  0  0
   -3.9950    2.4346   -3.7464 C   0  0  0  0  0  0  0  0  0  0  0  0
   -4.2768    2.8680   -1.6670 H   0  0  0  0  0  0  0  0  0  0  0  0
   -1.9050    1.2857   -4.3510 C   0  0  0  0  0  0  0  0  0  0  0  0
   -0.5649    0.8312   -2.7355 H   0  0  0  0  0  0  0  0  0  0  0  0
   -3.1332    1.8662   -4.6929 C   0  0  0  0  0  0  0  0  0  0  0  0
   -4.9447    2.8845   -4.0510 H   0  0  0  0  0  0  0  0  0  0  0  0
   -1.2538    0.8606   -5.1184 H   0  0  0  0  0  0  0  0  0  0  0  0
    2.4421    5.2167   -4.7065 F   0  0  0  0  0  0  0  0  0  0  0  0
    2.0846    5.4314    4.7767 F   0  0  0  0  0  0  0  0  0  0  0  0
   -1.3582    7.3792    1.6036 F   0  0  0  0  0  0  0  0  0  0  0  0
   -3.4962    1.8755   -5.9697 F   0  0  0  0  0  0  0  0  0  0  0  0
   -2.2129   -5.6594   -4.0050 F   0  0  0  0  0  0  0  0  0  0  0  0
   -2.5794   -4.4750    5.4826 F   0  0  0  0  0  0  0  0  0  0  0  0
    2.5004   -4.5094    5.4384 F   0  0  0  0  0  0  0  0  0  0  0  0
    1.9855   -5.9749   -3.8891 F   0  0  0  0  0  0  0  0  0  0  0  0
    0.0640    0.0582    0.1170 Ir  0  0  0  0  0  0  0  0  0  0  0  0
  1  2  1  0  0  0  0
  1  8  1  0  0  0  0
  1 12  1  0  0  0  0
  1 16  1  0  0  0  0
  2  3  1  0  0  0  0
  2 11  1  0  0  0  0
  2 15  1  0  0  0  0
  3 37  1  0  0  0  0
  3 47  1  0  0  0  0
  3105  1  0  0  0  0
  4  5  1  0  0  0  0
  4 77  1  0  0  0  0
  4 87  1  0  0  0  0
  4105  1  0  0  0  0
  5  6  1  0  0  0  0
  5  9  1  0  0  0  0
  5 13  1  0  0  0  0
  6  7  1  0  0  0  0
  6 10  1  0  0  0  0
  6 14  1  0  0  0  0
  7 57  1  0  0  0  0
  7 67  1  0  0  0  0
```

```
  7105  1  0  0  0  0
  8 17  1  0  0  0  0
  8 27  1  0  0  0  0
  8105  1  0  0  0  0
 17 18  2  0  0  0  0
 17 19  1  0  0  0  0
 18 20  1  0  0  0  0
 18 21  1  0  0  0  0
 19 22  2  0  0  0  0
 19 23  1  0  0  0  0
 20 24  2  0  0  0  0
 20 25  1  0  0  0  0
 22 24  1  0  0  0  0
 22 26  1  0  0  0  0
 24 97  1  0  0  0  0
 27 28  2  0  0  0  0
 27 29  1  0  0  0  0
 28 30  1  0  0  0  0
 28 31  1  0  0  0  0
 29 32  2  0  0  0  0
 29 33  1  0  0  0  0
 30 34  2  0  0  0  0
 30 35  1  0  0  0  0
 32 34  1  0  0  0  0
 32 36  1  0  0  0  0
 34 98  1  0  0  0  0
 37 38  2  0  0  0  0
 37 39  1  0  0  0  0
 38 40  1  0  0  0  0
 38 41  1  0  0  0  0
 39 42  2  0  0  0  0
 39 43  1  0  0  0  0
 40 44  2  0  0  0  0
 40 45  1  0  0  0  0
 42 44  1  0  0  0  0
 42 46  1  0  0  0  0
 44103  1  0  0  0  0
 47 48  2  0  0  0  0
 47 49  1  0  0  0  0
 48 50  1  0  0  0  0
 48 51  1  0  0  0  0
 49 52  2  0  0  0  0
 49 53  1  0  0  0  0
 50 54  2  0  0  0  0
 50 55  1  0  0  0  0
 52 54  1  0  0  0  0
 52 56  1  0  0  0  0
 54104  1  0  0  0  0
 57 58  2  0  0  0  0
 57 59  1  0  0  0  0
 58 60  1  0  0  0  0
 58 61  1  0  0  0  0
 59 62  2  0  0  0  0
 59 63  1  0  0  0  0
 60 64  2  0  0  0  0
 60 65  1  0  0  0  0
 62 64  1  0  0  0  0
 62 66  1  0  0  0  0
 64102  1  0  0  0  0
 67 68  2  0  0  0  0
 67 69  1  0  0  0  0
 68 70  1  0  0  0  0
 68 71  1  0  0  0  0
 69 72  2  0  0  0  0
 69 73  1  0  0  0  0
```

```
 70 74  2  0  0  0  0
 70 75  1  0  0  0  0
 72 74  1  0  0  0  0
 72 76  1  0  0  0  0
 74101  1  0  0  0  0
 77 78  2  0  0  0  0
 77 79  1  0  0  0  0
 78 80  1  0  0  0  0
 78 81  1  0  0  0  0
 79 82  2  0  0  0  0
 79 83  1  0  0  0  0
 80 84  2  0  0  0  0
 80 85  1  0  0  0  0
 82 84  1  0  0  0  0
 82 86  1  0  0  0  0
 84 99  1  0  0  0  0
 87 88  2  0  0  0  0
 87 89  1  0  0  0  0
 88 90  1  0  0  0  0
 88 91  1  0  0  0  0
 89 92  2  0  0  0  0
 89 93  1  0  0  0  0
 90 94  2  0  0  0  0
 90 95  1  0  0  0  0
 92 94  1  0  0  0  0
 92 96  1  0  0  0  0
 94100  1  0  0  0  0
M  END
$$$$
mol036
Optimized Catalyst 036
Optimized Catalyst 036
129138  0  0  0  0  0  0  0  0999 V2000
    3.6056    0.7224    0.4054 C   0  0  0  0  0  0  0  0  0  0  0  0
    3.5725   -0.7009   -0.1243 C   0  0  0  0  0  0  0  0  0  0  0  0
    1.9795   -1.7101    0.2434 P   0  0  0  0  0  0  0  0  0  0  0  0
   -1.8886    1.7718   -0.2682 P   0  0  0  0  0  0  0  0  0  0  0  0
   -3.3182    0.9138    0.6792 C   0  0  0  0  0  0  0  0  0  0  0  0
   -3.5275   -0.5185    0.2122 C   0  0  0  0  0  0  0  0  0  0  0  0
   -1.9511   -1.6156    0.3289 P   0  0  0  0  0  0  0  0  0  0  0  0
    2.0269    1.7778    0.1090 P   0  0  0  0  0  0  0  0  0  0  0  0
   -4.2622    1.4947    0.4985 H   0  0  0  0  0  0  0  0  0  0  0  0
   -3.9067   -0.5464   -0.8422 H   0  0  0  0  0  0  0  0  0  0  0  0
    3.7004   -0.7143   -1.2408 H   0  0  0  0  0  0  0  0  0  0  0  0
    4.4687    1.2618   -0.0718 H   0  0  0  0  0  0  0  0  0  0  0  0
   -3.1059    0.9627    1.7790 H   0  0  0  0  0  0  0  0  0  0  0  0
   -4.3112   -0.9944    0.8630 H   0  0  0  0  0  0  0  0  0  0  0  0
    4.4397   -1.2661    0.3142 H   0  0  0  0  0  0  0  0  0  0  0  0
    3.7877    0.7336    1.5136 H   0  0  0  0  0  0  0  0  0  0  0  0
    2.2167    2.7948   -1.4178 C   0  0  0  0  0  0  0  0  0  0  0  0
    1.4878    3.9893   -1.4969 C   0  0  0  0  0  0  0  0  0  0  0  0
    3.0435    2.3975   -2.4719 C   0  0  0  0  0  0  0  0  0  0  0  0
    1.5994    4.7939   -2.6294 C   0  0  0  0  0  0  0  0  0  0  0  0
    0.8298    4.3216   -0.6854 H   0  0  0  0  0  0  0  0  0  0  0  0
    3.1576    3.2103   -3.6023 C   0  0  0  0  0  0  0  0  0  0  0  0
    3.6104    1.4650   -2.4475 H   0  0  0  0  0  0  0  0  0  0  0  0
    2.4416    4.4170   -3.6930 C   0  0  0  0  0  0  0  0  0  0  0  0
    1.0350    5.7248   -2.6885 H   0  0  0  0  0  0  0  0  0  0  0  0
    3.8131    2.9066   -4.4196 H   0  0  0  0  0  0  0  0  0  0  0  0
    2.0180    2.9419    1.5486 C   0  0  0  0  0  0  0  0  0  0  0  0
    1.3499    2.5645    2.7186 C   0  0  0  0  0  0  0  0  0  0  0  0
    2.6887    4.1665    1.4761 C   0  0  0  0  0  0  0  0  0  0  0  0
    1.3341    3.4274    3.8145 C   0  0  0  0  0  0  0  0  0  0  0  0
    0.8338    1.6001    2.8044 H   0  0  0  0  0  0  0  0  0  0  0  0
    2.6669    5.0303    2.5735 C   0  0  0  0  0  0  0  0  0  0  0  0
```

3.2371	4.4737	0.5798	H	0	0	0	0	0	0	0	0	0	0	0	0
1.9854	4.6743	3.7499	C	0	0	0	0	0	0	0	0	0	0	0	0
0.8165	3.1355	4.7283	H	0	0	0	0	0	0	0	0	0	0	0	0
3.1900	5.9858	2.5162	H	0	0	0	0	0	0	0	0	0	0	0	0
2.1231	-2.5717	1.8705	C	0	0	0	0	0	0	0	0	0	0	0	0
1.4359	-3.7820	2.0327	C	0	0	0	0	0	0	0	0	0	0	0	0
2.8500	-2.0234	2.9312	C	0	0	0	0	0	0	0	0	0	0	0	0
1.4711	-4.4401	3.2622	C	0	0	0	0	0	0	0	0	0	0	0	0
0.8612	-4.2336	1.2188	H	0	0	0	0	0	0	0	0	0	0	0	0
2.8820	-2.6848	4.1605	C	0	0	0	0	0	0	0	0	0	0	0	0
3.3997	-1.0859	2.8357	H	0	0	0	0	0	0	0	0	0	0	0	0
2.1882	-3.8946	4.3423	C	0	0	0	0	0	0	0	0	0	0	0	0
0.9320	-5.3796	3.3865	H	0	0	0	0	0	0	0	0	0	0	0	0
3.4542	-2.2609	4.9869	H	0	0	0	0	0	0	0	0	0	0	0	0
1.9713	-3.0185	-1.0637	C	0	0	0	0	0	0	0	0	0	0	0	0
1.2061	-2.8333	-2.2181	C	0	0	0	0	0	0	0	0	0	0	0	0
2.7402	-4.1774	-0.8988	C	0	0	0	0	0	0	0	0	0	0	0	0
1.1884	-3.8225	-3.2042	C	0	0	0	0	0	0	0	0	0	0	0	0
0.6046	-1.9305	-2.3797	H	0	0	0	0	0	0	0	0	0	0	0	0
2.7159	-5.1651	-1.8827	C	0	0	0	0	0	0	0	0	0	0	0	0
3.3636	-4.3350	-0.0133	H	0	0	0	0	0	0	0	0	0	0	0	0
1.9334	-5.0027	-3.0421	C	0	0	0	0	0	0	0	0	0	0	0	0
0.5827	-3.6776	-4.0988	H	0	0	0	0	0	0	0	0	0	0	0	0
3.3104	-6.0710	-1.7554	H	0	0	0	0	0	0	0	0	0	0	0	0
-2.0734	-2.5063	1.9442	C	0	0	0	0	0	0	0	0	0	0	0	0
-1.2145	-2.1595	2.9900	C	0	0	0	0	0	0	0	0	0	0	0	0
-3.0297	-3.5156	2.1138	C	0	0	0	0	0	0	0	0	0	0	0	0
-1.2879	-2.8472	4.2041	C	0	0	0	0	0	0	0	0	0	0	0	0
-0.4696	-1.3596	2.8936	H	0	0	0	0	0	0	0	0	0	0	0	0
-3.1000	-4.2000	3.3264	C	0	0	0	0	0	0	0	0	0	0	0	0
-3.7286	-3.7886	1.3175	H	0	0	0	0	0	0	0	0	0	0	0	0
-2.2241	-3.8794	4.3818	C	0	0	0	0	0	0	0	0	0	0	0	0
-0.6067	-2.5822	5.0136	H	0	0	0	0	0	0	0	0	0	0	0	0
-3.8424	-4.9880	3.4614	H	0	0	0	0	0	0	0	0	0	0	0	0
-2.0475	-2.8686	-1.0252	C	0	0	0	0	0	0	0	0	0	0	0	0
-2.4316	-2.4792	-2.3142	C	0	0	0	0	0	0	0	0	0	0	0	0
-1.6823	-4.1941	-0.7650	C	0	0	0	0	0	0	0	0	0	0	0	0
-2.4614	-3.4240	-3.3402	C	0	0	0	0	0	0	0	0	0	0	0	0
-2.7041	-1.4480	-2.5570	H	0	0	0	0	0	0	0	0	0	0	0	0
-1.7072	-5.1350	-1.7960	C	0	0	0	0	0	0	0	0	0	0	0	0
-1.3706	-4.5239	0.2303	H	0	0	0	0	0	0	0	0	0	0	0	0
-2.0942	-4.7605	-3.0943	C	0	0	0	0	0	0	0	0	0	0	0	0
-2.7738	-3.1241	-4.3409	H	0	0	0	0	0	0	0	0	0	0	0	0
-1.4208	-6.1669	-1.5910	H	0	0	0	0	0	0	0	0	0	0	0	0
-1.7349	3.5076	0.3264	C	0	0	0	0	0	0	0	0	0	0	0	0
-1.7581	4.5597	-0.5969	C	0	0	0	0	0	0	0	0	0	0	0	0
-1.5434	3.7622	1.6887	C	0	0	0	0	0	0	0	0	0	0	0	0
-1.5766	5.8700	-0.1532	C	0	0	0	0	0	0	0	0	0	0	0	0
-1.9094	4.3873	-1.6680	H	0	0	0	0	0	0	0	0	0	0	0	0
-1.3591	5.0741	2.1274	C	0	0	0	0	0	0	0	0	0	0	0	0
-1.5278	2.9619	2.4324	H	0	0	0	0	0	0	0	0	0	0	0	0
-1.3716	6.1416	1.2121	C	0	0	0	0	0	0	0	0	0	0	0	0
-1.5973	6.6915	-0.8702	H	0	0	0	0	0	0	0	0	0	0	0	0
-1.1966	5.2677	3.1882	H	0	0	0	0	0	0	0	0	0	0	0	0
-2.4187	1.8194	-2.0408	C	0	0	0	0	0	0	0	0	0	0	0	0
-3.6503	2.3841	-2.3939	C	0	0	0	0	0	0	0	0	0	0	0	0
-1.5759	1.2825	-3.0172	C	0	0	0	0	0	0	0	0	0	0	0	0
-4.0407	2.3989	-3.7322	C	0	0	0	0	0	0	0	0	0	0	0	0
-4.3197	2.8226	-1.6491	H	0	0	0	0	0	0	0	0	0	0	0	0
-1.9699	1.2998	-4.3574	C	0	0	0	0	0	0	0	0	0	0	0	0
-0.5986	0.8452	-2.7661	H	0	0	0	0	0	0	0	0	0	0	0	0
-3.2057	1.8569	-4.7289	C	0	0	0	0	0	0	0	0	0	0	0	0
-5.0006	2.8363	-4.0121	H	0	0	0	0	0	0	0	0	0	0	0	0
-1.3086	0.8842	-5.1181	H	0	0	0	0	0	0	0	0	0	0	0	0
1.9055	-6.0760	-4.0788	C	0	0	0	0	0	0	0	0	0	0	0	0

```
    2.9263   -6.1987   -4.5217 H   0  0  0  0  0  0  0  0  0  0  0  0
    1.6030   -7.0482   -3.6187 H   0  0  0  0  0  0  0  0  0  0  0  0
    1.1945   -5.8409   -4.9045 H   0  0  0  0  0  0  0  0  0  0  0  0
   -2.1215   -5.7651   -4.1974 C   0  0  0  0  0  0  0  0  0  0  0  0
   -1.4272   -6.6142   -3.9941 H   0  0  0  0  0  0  0  0  0  0  0  0
   -3.1592   -6.1763   -4.2935 H   0  0  0  0  0  0  0  0  0  0  0  0
   -1.8358   -5.3033   -5.1719 H   0  0  0  0  0  0  0  0  0  0  0  0
   -2.3047   -4.6208    5.6742 C   0  0  0  0  0  0  0  0  0  0  0  0
   -3.1928   -4.2583    6.2528 H   0  0  0  0  0  0  0  0  0  0  0  0
   -2.4264   -5.7167    5.4975 H   0  0  0  0  0  0  0  0  0  0  0  0
   -1.3942   -4.4652    6.2986 H   0  0  0  0  0  0  0  0  0  0  0  0
    2.2210   -4.5922    5.6607 C   0  0  0  0  0  0  0  0  0  0  0  0
    3.1470   -5.2201    5.7206 H   0  0  0  0  0  0  0  0  0  0  0  0
    2.2394   -3.8607    6.5038 H   0  0  0  0  0  0  0  0  0  0  0  0
    1.3373   -5.2592    5.7941 H   0  0  0  0  0  0  0  0  0  0  0  0
   -1.1630    7.5460    1.6701 C   0  0  0  0  0  0  0  0  0  0  0  0
   -2.0074    8.1929    1.3239 H   0  0  0  0  0  0  0  0  0  0  0  0
   -1.1062    7.6187    2.7814 H   0  0  0  0  0  0  0  0  0  0  0  0
   -0.2113    7.9465    1.2416 H   0  0  0  0  0  0  0  0  0  0  0  0
    2.5830    5.3006   -4.8866 C   0  0  0  0  0  0  0  0  0  0  0  0
    1.6036    5.7580   -5.1656 H   0  0  0  0  0  0  0  0  0  0  0  0
    3.3038    6.1248   -4.6507 H   0  0  0  0  0  0  0  0  0  0  0  0
    2.9700    4.7419   -5.7716 H   0  0  0  0  0  0  0  0  0  0  0  0
   -3.6380    1.8840   -6.1562 C   0  0  0  0  0  0  0  0  0  0  0  0
   -4.6271    1.3750   -6.2697 H   0  0  0  0  0  0  0  0  0  0  0  0
   -3.7466    2.9435   -6.4997 H   0  0  0  0  0  0  0  0  0  0  0  0
   -2.9057    1.3757   -6.8263 H   0  0  0  0  0  0  0  0  0  0  0  0
    1.9620    5.5914    4.9267 C   0  0  0  0  0  0  0  0  0  0  0  0
    2.7086    5.2385    5.6838 H   0  0  0  0  0  0  0  0  0  0  0  0
    2.2247    6.6377    4.6422 H   0  0  0  0  0  0  0  0  0  0  0  0
    0.9540    5.6025    5.4058 H   0  0  0  0  0  0  0  0  0  0  0  0
    0.0569    0.0600    0.1047 Ir  0  0  0  0  0  0  0  0  0  0  0  0
  1  2  1  0  0  0  0
  1  8  1  0  0  0  0
  1 12  1  0  0  0  0
  1 16  1  0  0  0  0
  2  3  1  0  0  0  0
  2 11  1  0  0  0  0
  2 15  1  0  0  0  0
  3 37  1  0  0  0  0
  3 47  1  0  0  0  0
  3129  1  0  0  0  0
  4  5  1  0  0  0  0
  4 77  1  0  0  0  0
  4 87  1  0  0  0  0
  4129  1  0  0  0  0
  5  6  1  0  0  0  0
  5  9  1  0  0  0  0
  5 13  1  0  0  0  0
  6  7  1  0  0  0  0
  6 10  1  0  0  0  0
  6 14  1  0  0  0  0
  7 57  1  0  0  0  0
  7 67  1  0  0  0  0
  7129  1  0  0  0  0
  8 17  1  0  0  0  0
  8 27  1  0  0  0  0
  8129  1  0  0  0  0
 17 18  2  0  0  0  0
 17 19  1  0  0  0  0
 18 20  1  0  0  0  0
 18 21  1  0  0  0  0
 19 22  2  0  0  0  0
 19 23  1  0  0  0  0
 20 24  2  0  0  0  0
```

```
 20 25  1  0  0  0  0
 22 24  1  0  0  0  0
 22 26  1  0  0  0  0
 24117  1  0  0  0  0
 27 28  2  0  0  0  0
 27 29  1  0  0  0  0
 28 30  1  0  0  0  0
 28 31  1  0  0  0  0
 29 32  2  0  0  0  0
 29 33  1  0  0  0  0
 30 34  2  0  0  0  0
 30 35  1  0  0  0  0
 32 34  1  0  0  0  0
 32 36  1  0  0  0  0
 34125  1  0  0  0  0
 37 38  2  0  0  0  0
 37 39  1  0  0  0  0
 38 40  1  0  0  0  0
 38 41  1  0  0  0  0
 39 42  2  0  0  0  0
 39 43  1  0  0  0  0
 40 44  2  0  0  0  0
 40 45  1  0  0  0  0
 42 44  1  0  0  0  0
 42 46  1  0  0  0  0
 44109  1  0  0  0  0
 47 48  2  0  0  0  0
 47 49  1  0  0  0  0
 48 50  1  0  0  0  0
 48 51  1  0  0  0  0
 49 52  2  0  0  0  0
 49 53  1  0  0  0  0
 50 54  2  0  0  0  0
 50 55  1  0  0  0  0
 52 54  1  0  0  0  0
 52 56  1  0  0  0  0
 54 97  1  0  0  0  0
 57 58  2  0  0  0  0
 57 59  1  0  0  0  0
 58 60  1  0  0  0  0
 58 61  1  0  0  0  0
 59 62  2  0  0  0  0
 59 63  1  0  0  0  0
 60 64  2  0  0  0  0
 60 65  1  0  0  0  0
 62 64  1  0  0  0  0
 62 66  1  0  0  0  0
 64105  1  0  0  0  0
 67 68  2  0  0  0  0
 67 69  1  0  0  0  0
 68 70  1  0  0  0  0
 68 71  1  0  0  0  0
 69 72  2  0  0  0  0
 69 73  1  0  0  0  0
 70 74  2  0  0  0  0
 70 75  1  0  0  0  0
 72 74  1  0  0  0  0
 72 76  1  0  0  0  0
 74101  1  0  0  0  0
 77 78  2  0  0  0  0
 77 79  1  0  0  0  0
 78 80  1  0  0  0  0
 78 81  1  0  0  0  0
 79 82  2  0  0  0  0
 79 83  1  0  0  0  0
```

```
 80 84  2  0  0  0  0
 80 85  1  0  0  0  0
 82 84  1  0  0  0  0
 82 86  1  0  0  0  0
 84113  1  0  0  0  0
 87 88  2  0  0  0  0
 87 89  1  0  0  0  0
 88 90  1  0  0  0  0
 88 91  1  0  0  0  0
 89 92  2  0  0  0  0
 89 93  1  0  0  0  0
 90 94  2  0  0  0  0
 90 95  1  0  0  0  0
 92 94  1  0  0  0  0
 92 96  1  0  0  0  0
 94121  1  0  0  0  0
 97 98  1  0  0  0  0
 97 99  1  0  0  0  0
 97100  1  0  0  0  0
101102  1  0  0  0  0
101103  1  0  0  0  0
101104  1  0  0  0  0
105106  1  0  0  0  0
105107  1  0  0  0  0
105108  1  0  0  0  0
109110  1  0  0  0  0
109111  1  0  0  0  0
109112  1  0  0  0  0
113114  1  0  0  0  0
113115  1  0  0  0  0
113116  1  0  0  0  0
117118  1  0  0  0  0
117119  1  0  0  0  0
117120  1  0  0  0  0
121122  1  0  0  0  0
121123  1  0  0  0  0
121124  1  0  0  0  0
125126  1  0  0  0  0
125127  1  0  0  0  0
125128  1  0  0  0  0
M  END
$$$$
mol038
Optimized Catalyst 038
Optimized Catalyst 038
106114  0  0  0  0  0  0  0  0999 V2000
   -1.8190    0.2061   -0.3203 H   0  0  0  0  0  0  0  0  0  0  0  0
    1.8587    0.0486   -0.5958 P   0  0  0  0  0  0  0  0  0  0  0  0
   -1.3343   -2.5510    0.5728 P   0  0  0  0  0  0  0  0  0  0  0  0
   -1.0655    2.8655    0.6861 P   0  0  0  0  0  0  0  0  0  0  0  0
    2.9540    1.5001   -0.1249 C   0  0  0  0  0  0  0  0  0  0  0  0
    3.8025    2.0993   -1.0603 C   0  0  0  0  0  0  0  0  0  0  0  0
    2.8811    1.9758    1.1887 C   0  0  0  0  0  0  0  0  0  0  0  0
    4.5830    3.2007   -0.6924 C   0  0  0  0  0  0  0  0  0  0  0  0
    3.8708    1.7381   -2.0910 H   0  0  0  0  0  0  0  0  0  0  0  0
    3.6527    3.0750    1.5787 C   0  0  0  0  0  0  0  0  0  0  0  0
    2.2330    1.5133    1.9407 H   0  0  0  0  0  0  0  0  0  0  0  0
    4.4871    3.6629    0.6233 C   0  0  0  0  0  0  0  0  0  0  0  0
    5.2502    3.6781   -1.4103 H   0  0  0  0  0  0  0  0  0  0  0  0
    3.6003    3.4606    2.5978 H   0  0  0  0  0  0  0  0  0  0  0  0
    2.9222   -1.4352   -0.1638 C   0  0  0  0  0  0  0  0  0  0  0  0
    2.7569   -2.6199   -0.8885 C   0  0  0  0  0  0  0  0  0  0  0  0
    3.8282   -1.3663    0.8989 C   0  0  0  0  0  0  0  0  0  0  0  0
    3.4907   -3.7604   -0.5496 C   0  0  0  0  0  0  0  0  0  0  0  0
    2.0571   -2.6864   -1.7279 H   0  0  0  0  0  0  0  0  0  0  0  0
```

```
    4.5713   -2.4961    1.2586 C   0  0  0  0  0  0  0  0  0  0  0  0
    3.9829   -0.4449    1.4684 H   0  0  0  0  0  0  0  0  0  0  0  0
    4.3839   -3.6691    0.5221 C   0  0  0  0  0  0  0  0  0  0  0  0
    3.3678   -4.6892   -1.1065 H   0  0  0  0  0  0  0  0  0  0  0  0
    5.2793   -2.4572    2.0870 H   0  0  0  0  0  0  0  0  0  0  0  0
    1.8604    0.0833   -2.4699 C   0  0  0  0  0  0  0  0  0  0  0  0
    2.9080   -0.4696   -3.2150 C   0  0  0  0  0  0  0  0  0  0  0  0
    0.7691    0.6849   -3.1013 C   0  0  0  0  0  0  0  0  0  0  0  0
    2.8636   -0.4396   -4.6114 C   0  0  0  0  0  0  0  0  0  0  0  0
    3.7758   -0.9319   -2.7328 H   0  0  0  0  0  0  0  0  0  0  0  0
    0.7074    0.7352   -4.4986 C   0  0  0  0  0  0  0  0  0  0  0  0
   -0.0533    1.1339   -2.5340 H   0  0  0  0  0  0  0  0  0  0  0  0
    1.7590    0.1656   -5.2207 C   0  0  0  0  0  0  0  0  0  0  0  0
    3.6669   -0.8737   -5.2074 H   0  0  0  0  0  0  0  0  0  0  0  0
   -0.1341    1.2097   -5.0041 H   0  0  0  0  0  0  0  0  0  0  0  0
   -0.0297    3.8410    1.8967 C   0  0  0  0  0  0  0  0  0  0  0  0
    0.5133    5.0780    1.5297 C   0  0  0  0  0  0  0  0  0  0  0  0
    0.1475    3.3345    3.1880 C   0  0  0  0  0  0  0  0  0  0  0  0
    1.2730    5.8085    2.4475 C   0  0  0  0  0  0  0  0  0  0  0  0
    0.3643    5.5063    0.5331 H   0  0  0  0  0  0  0  0  0  0  0  0
    0.8993    4.0514    4.1243 C   0  0  0  0  0  0  0  0  0  0  0  0
   -0.2885    2.3817    3.5074 H   0  0  0  0  0  0  0  0  0  0  0  0
    1.4487    5.2746    3.7283 C   0  0  0  0  0  0  0  0  0  0  0  0
    1.7143    6.7677    2.1721 H   0  0  0  0  0  0  0  0  0  0  0  0
    1.0470    3.6661    5.1348 H   0  0  0  0  0  0  0  0  0  0  0  0
   -2.7820    3.5564    0.9394 C   0  0  0  0  0  0  0  0  0  0  0  0
   -3.8442    2.6739    1.1541 C   0  0  0  0  0  0  0  0  0  0  0  0
   -2.9840    4.9414    0.9199 C   0  0  0  0  0  0  0  0  0  0  0  0
   -5.1377    3.1708    1.3432 C   0  0  0  0  0  0  0  0  0  0  0  0
   -3.6978    1.5838    1.1801 H   0  0  0  0  0  0  0  0  0  0  0  0
   -4.2675    5.4599    1.1093 C   0  0  0  0  0  0  0  0  0  0  0  0
   -2.1602    5.6421    0.7575 H   0  0  0  0  0  0  0  0  0  0  0  0
   -5.3169    4.5576    1.3167 C   0  0  0  0  0  0  0  0  0  0  0  0
   -5.9790    2.4964    1.5085 H   0  0  0  0  0  0  0  0  0  0  0  0
   -4.4450    6.5362    1.0990 H   0  0  0  0  0  0  0  0  0  0  0  0
   -0.5727    3.4676   -1.0107 C   0  0  0  0  0  0  0  0  0  0  0  0
   -1.5617    3.6393   -1.9856 C   0  0  0  0  0  0  0  0  0  0  0  0
    0.7742    3.7097   -1.2995 C   0  0  0  0  0  0  0  0  0  0  0  0
   -1.2048    4.0474   -3.2743 C   0  0  0  0  0  0  0  0  0  0  0  0
   -2.6216    3.4635   -1.7710 H   0  0  0  0  0  0  0  0  0  0  0  0
    1.1529    4.1162   -2.5817 C   0  0  0  0  0  0  0  0  0  0  0  0
    1.5591    3.5899   -0.5439 H   0  0  0  0  0  0  0  0  0  0  0  0
    0.1491    4.2755   -3.5423 C   0  0  0  0  0  0  0  0  0  0  0  0
   -1.9629    4.1861   -4.0464 H   0  0  0  0  0  0  0  0  0  0  0  0
    2.2013    4.2977   -2.8218 H   0  0  0  0  0  0  0  0  0  0  0  0
   -0.5314   -3.5273    1.9460 C   0  0  0  0  0  0  0  0  0  0  0  0
    0.8611   -3.4633    2.0780 C   0  0  0  0  0  0  0  0  0  0  0  0
   -1.3016   -4.3145    2.8067 C   0  0  0  0  0  0  0  0  0  0  0  0
    1.5039   -4.1986    3.0755 C   0  0  0  0  0  0  0  0  0  0  0  0
    1.4779   -2.8516    1.4117 H   0  0  0  0  0  0  0  0  0  0  0  0
   -0.6790   -5.0518    3.8195 C   0  0  0  0  0  0  0  0  0  0  0  0
   -2.3911   -4.3779    2.7167 H   0  0  0  0  0  0  0  0  0  0  0  0
    0.7128   -4.9774    3.9281 C   0  0  0  0  0  0  0  0  0  0  0  0
    2.5890   -4.1598    3.1871 H   0  0  0  0  0  0  0  0  0  0  0  0
   -1.2657   -5.6640    4.5058 H   0  0  0  0  0  0  0  0  0  0  0  0
   -0.6093   -3.2699   -0.9903 C   0  0  0  0  0  0  0  0  0  0  0  0
   -0.6142   -2.5037   -2.1602 C   0  0  0  0  0  0  0  0  0  0  0  0
   -0.0856   -4.5683   -0.9788 C   0  0  0  0  0  0  0  0  0  0  0  0
   -0.0651   -3.0225   -3.3373 C   0  0  0  0  0  0  0  0  0  0  0  0
   -1.0378   -1.4927   -2.1937 H   0  0  0  0  0  0  0  0  0  0  0  0
    0.4545   -5.1110   -2.1480 C   0  0  0  0  0  0  0  0  0  0  0  0
   -0.0791   -5.1794   -0.0692 H   0  0  0  0  0  0  0  0  0  0  0  0
    0.4545   -4.3203   -3.3025 C   0  0  0  0  0  0  0  0  0  0  0  0
   -0.0411   -2.4260   -4.2518 H   0  0  0  0  0  0  0  0  0  0  0  0
    0.8646   -6.1219   -2.1578 H   0  0  0  0  0  0  0  0  0  0  0  0
```

```
   -3.1101   -3.1262    0.5744 C   0  0  0  0  0  0  0  0  0  0  0  0
   -3.5251   -4.1336   -0.3018 C   0  0  0  0  0  0  0  0  0  0  0  0
   -3.9996   -2.5518    1.4907 C   0  0  0  0  0  0  0  0  0  0  0  0
   -4.8473   -4.5883   -0.2640 C   0  0  0  0  0  0  0  0  0  0  0  0
   -2.8433   -4.5846   -1.0304 H   0  0  0  0  0  0  0  0  0  0  0  0
   -5.3229   -2.9958    1.5486 C   0  0  0  0  0  0  0  0  0  0  0  0
   -3.6899   -1.7570    2.1787 H   0  0  0  0  0  0  0  0  0  0  0  0
   -5.7154   -4.0088    0.6658 C   0  0  0  0  0  0  0  0  0  0  0  0
   -5.1893   -5.3707   -0.9431 H   0  0  0  0  0  0  0  0  0  0  0  0
   -6.0297   -2.5628    2.2580 H   0  0  0  0  0  0  0  0  0  0  0  0
   -0.1650    0.0457    2.0250 C   0  0  0  0  0  0  0  0  0  0  0  0
    0.5263   -0.0033    2.9634 O   0  0  0  0  0  0  0  0  0  0  0  0
   -1.0498    0.1367    0.7621 Ir  0  0  0  0  0  0  0  0  0  0  0  0
   -6.9741   -4.4392    0.7146 F   0  0  0  0  0  0  0  0  0  0  0  0
    0.9824   -4.8233   -4.4160 F   0  0  0  0  0  0  0  0  0  0  0  0
    1.7077    0.1989   -6.5514 F   0  0  0  0  0  0  0  0  0  0  0  0
    0.4981    4.6569   -4.7691 F   0  0  0  0  0  0  0  0  0  0  0  0
    2.1730    5.9613    4.6092 F   0  0  0  0  0  0  0  0  0  0  0  0
    1.3139   -5.6805    4.8854 F   0  0  0  0  0  0  0  0  0  0  0  0
    5.0825   -4.7521    0.8612 F   0  0  0  0  0  0  0  0  0  0  0  0
    5.2192    4.7167    0.9822 F   0  0  0  0  0  0  0  0  0  0  0  0
   -6.5436    5.0420    1.4961 F   0  0  0  0  0  0  0  0  0  0  0  0
  1 97  1  0  0  0  0
  2  5  1  0  0  0  0
  2 15  1  0  0  0  0
  2 25  1  0  0  0  0
  2 97  1  0  0  0  0
  3 65  1  0  0  0  0
  3 75  1  0  0  0  0
  3 85  1  0  0  0  0
  3 97  1  0  0  0  0
  4 35  1  0  0  0  0
  4 45  1  0  0  0  0
  4 55  1  0  0  0  0
  4 97  1  0  0  0  0
  5  6  2  0  0  0  0
  5  7  1  0  0  0  0
  6  8  1  0  0  0  0
  6  9  1  0  0  0  0
  7 10  2  0  0  0  0
  7 11  1  0  0  0  0
  8 12  2  0  0  0  0
  8 13  1  0  0  0  0
 10 12  1  0  0  0  0
 10 14  1  0  0  0  0
 12105  1  0  0  0  0
 15 16  2  0  0  0  0
 15 17  1  0  0  0  0
 16 18  1  0  0  0  0
 16 19  1  0  0  0  0
 17 20  2  0  0  0  0
 17 21  1  0  0  0  0
 18 22  2  0  0  0  0
 18 23  1  0  0  0  0
 20 22  1  0  0  0  0
 20 24  1  0  0  0  0
 22104  1  0  0  0  0
 25 26  2  0  0  0  0
 25 27  1  0  0  0  0
 26 28  1  0  0  0  0
 26 29  1  0  0  0  0
 27 30  2  0  0  0  0
 27 31  1  0  0  0  0
 28 32  2  0  0  0  0
 28 33  1  0  0  0  0
```

```
 30 32  1  0  0  0  0
 30 34  1  0  0  0  0
 32100  1  0  0  0  0
 35 36  2  0  0  0  0
 35 37  1  0  0  0  0
 36 38  1  0  0  0  0
 36 39  1  0  0  0  0
 37 40  2  0  0  0  0
 37 41  1  0  0  0  0
 38 42  2  0  0  0  0
 38 43  1  0  0  0  0
 40 42  1  0  0  0  0
 40 44  1  0  0  0  0
 42102  1  0  0  0  0
 45 46  2  0  0  0  0
 45 47  1  0  0  0  0
 46 48  1  0  0  0  0
 46 49  1  0  0  0  0
 47 50  2  0  0  0  0
 47 51  1  0  0  0  0
 48 52  2  0  0  0  0
 48 53  1  0  0  0  0
 50 52  1  0  0  0  0
 50 54  1  0  0  0  0
 52106  1  0  0  0  0
 55 56  2  0  0  0  0
 55 57  1  0  0  0  0
 56 58  1  0  0  0  0
 56 59  1  0  0  0  0
 57 60  2  0  0  0  0
 57 61  1  0  0  0  0
 58 62  2  0  0  0  0
 58 63  1  0  0  0  0
 60 62  1  0  0  0  0
 60 64  1  0  0  0  0
 62101  1  0  0  0  0
 65 66  2  0  0  0  0
 65 67  1  0  0  0  0
 66 68  1  0  0  0  0
 66 69  1  0  0  0  0
 67 70  2  0  0  0  0
 67 71  1  0  0  0  0
 68 72  2  0  0  0  0
 68 73  1  0  0  0  0
 70 72  1  0  0  0  0
 70 74  1  0  0  0  0
 72103  1  0  0  0  0
 75 76  2  0  0  0  0
 75 77  1  0  0  0  0
 76 78  1  0  0  0  0
 76 79  1  0  0  0  0
 77 80  2  0  0  0  0
 77 81  1  0  0  0  0
 78 82  2  0  0  0  0
 78 83  1  0  0  0  0
 80 82  1  0  0  0  0
 80 84  1  0  0  0  0
 82 99  1  0  0  0  0
 85 86  2  0  0  0  0
 85 87  1  0  0  0  0
 86 88  1  0  0  0  0
 86 89  1  0  0  0  0
 87 90  2  0  0  0  0
 87 91  1  0  0  0  0
 88 92  2  0  0  0  0
```

```
 88 93  1  0  0  0  0
 90 92  1  0  0  0  0
 90 94  1  0  0  0  0
 92 98  1  0  0  0  0
 95 96  2  0  0  0  0
 95 97  1  0  0  0  0
M  END
$$$$
mol039
Optimized Catalyst 039
Optimized Catalyst 039
133141  0  0  0  0  0  0  0  0999 V2000
   -1.8621    0.2236   -0.2517 H   0  0  0  0  0  0  0  0  0  0  0  0
    1.8425    0.0520   -0.5337 P   0  0  0  0  0  0  0  0  0  0  0  0
   -1.3510   -2.5461    0.5722 P   0  0  0  0  0  0  0  0  0  0  0  0
   -1.0609    2.8704    0.7176 P   0  0  0  0  0  0  0  0  0  0  0  0
    2.9063    1.5261   -0.0664 C   0  0  0  0  0  0  0  0  0  0  0  0
    3.6730    2.2052   -1.0156 C   0  0  0  0  0  0  0  0  0  0  0  0
    2.8809    1.9607    1.2613 C   0  0  0  0  0  0  0  0  0  0  0  0
    4.3917    3.3456   -0.6424 C   0  0  0  0  0  0  0  0  0  0  0  0
    3.7146    1.8790   -2.0581 H   0  0  0  0  0  0  0  0  0  0  0  0
    3.6123    3.0905    1.6369 C   0  0  0  0  0  0  0  0  0  0  0  0
    2.2981    1.4459    2.0312 H   0  0  0  0  0  0  0  0  0  0  0  0
    4.3610    3.8013    0.6855 C   0  0  0  0  0  0  0  0  0  0  0  0
    4.9742    3.8826   -1.3879 H   0  0  0  0  0  0  0  0  0  0  0  0
    3.5796    3.4286    2.6717 H   0  0  0  0  0  0  0  0  0  0  0  0
    2.9148   -1.4166   -0.0766 C   0  0  0  0  0  0  0  0  0  0  0  0
    2.7478   -2.6174   -0.7707 C   0  0  0  0  0  0  0  0  0  0  0  0
    3.8257   -1.3373    0.9794 C   0  0  0  0  0  0  0  0  0  0  0  0
    3.4755   -3.7496   -0.3947 C   0  0  0  0  0  0  0  0  0  0  0  0
    2.0507   -2.7026   -1.6105 H   0  0  0  0  0  0  0  0  0  0  0  0
    4.5509   -2.4708    1.3584 C   0  0  0  0  0  0  0  0  0  0  0  0
    3.9965   -0.4061    1.5252 H   0  0  0  0  0  0  0  0  0  0  0  0
    4.3760   -3.6884    0.6807 C   0  0  0  0  0  0  0  0  0  0  0  0
    3.3342   -4.6808   -0.9396 H   0  0  0  0  0  0  0  0  0  0  0  0
    5.2575   -2.4051    2.1837 H   0  0  0  0  0  0  0  0  0  0  0  0
    1.8613    0.0693   -2.4075 C   0  0  0  0  0  0  0  0  0  0  0  0
    2.9189   -0.4747   -3.1405 C   0  0  0  0  0  0  0  0  0  0  0  0
    0.7746    0.6504   -3.0630 C   0  0  0  0  0  0  0  0  0  0  0  0
    2.8844   -0.4442   -4.5374 C   0  0  0  0  0  0  0  0  0  0  0  0
    3.7837   -0.9275   -2.6472 H   0  0  0  0  0  0  0  0  0  0  0  0
    0.7432    0.6880   -4.4605 C   0  0  0  0  0  0  0  0  0  0  0  0
   -0.0625    1.0945   -2.5140 H   0  0  0  0  0  0  0  0  0  0  0  0
    1.7989    0.1429   -5.2078 C   0  0  0  0  0  0  0  0  0  0  0  0
    3.7060   -0.8760   -5.1045 H   0  0  0  0  0  0  0  0  0  0  0  0
   -0.1056    1.1462   -4.9650 H   0  0  0  0  0  0  0  0  0  0  0  0
    0.0303    3.8512    1.8741 C   0  0  0  0  0  0  0  0  0  0  0  0
    0.5583    5.0826    1.4762 C   0  0  0  0  0  0  0  0  0  0  0  0
    0.2894    3.3554    3.1543 C   0  0  0  0  0  0  0  0  0  0  0  0
    1.3852    5.7994    2.3452 C   0  0  0  0  0  0  0  0  0  0  0  0
    0.3476    5.5128    0.4931 H   0  0  0  0  0  0  0  0  0  0  0  0
    1.1104    4.0760    4.0259 C   0  0  0  0  0  0  0  0  0  0  0  0
   -0.1312    2.4100    3.5094 H   0  0  0  0  0  0  0  0  0  0  0  0
    1.6745    5.2988    3.6250 C   0  0  0  0  0  0  0  0  0  0  0  0
    1.8078    6.7491    2.0232 H   0  0  0  0  0  0  0  0  0  0  0  0
    1.3159    3.6816    5.0204 H   0  0  0  0  0  0  0  0  0  0  0  0
   -2.7587    3.5927    1.0037 C   0  0  0  0  0  0  0  0  0  0  0  0
   -3.8182    2.7450    1.3339 C   0  0  0  0  0  0  0  0  0  0  0  0
   -2.9617    4.9699    0.8838 C   0  0  0  0  0  0  0  0  0  0  0  0
   -5.0943    3.2770    1.5337 C   0  0  0  0  0  0  0  0  0  0  0  0
   -3.6803    1.6599    1.4414 H   0  0  0  0  0  0  0  0  0  0  0  0
   -4.2382    5.5014    1.0849 C   0  0  0  0  0  0  0  0  0  0  0  0
   -2.1465    5.6517    0.6313 H   0  0  0  0  0  0  0  0  0  0  0  0
   -5.3152    4.6598    1.4090 C   0  0  0  0  0  0  0  0  0  0  0  0
   -5.9219    2.6160    1.7872 H   0  0  0  0  0  0  0  0  0  0  0  0
```

```
   -4.3941    6.5744    0.9895 H   0  0  0  0  0  0  0  0  0  0  0  0
   -0.6139    3.4202   -1.0087 C   0  0  0  0  0  0  0  0  0  0  0  0
   -1.6177    3.5226   -1.9754 C   0  0  0  0  0  0  0  0  0  0  0  0
    0.7181    3.6795   -1.3420 C   0  0  0  0  0  0  0  0  0  0  0  0
   -1.2813    3.8571   -3.2907 C   0  0  0  0  0  0  0  0  0  0  0  0
   -2.6704    3.3469   -1.7340 H   0  0  0  0  0  0  0  0  0  0  0  0
    1.0524    4.0126   -2.6557 C   0  0  0  0  0  0  0  0  0  0  0  0
    1.5217    3.6234   -0.5994 H   0  0  0  0  0  0  0  0  0  0  0  0
    0.0573    4.0909   -3.6455 C   0  0  0  0  0  0  0  0  0  0  0  0
   -2.0661    3.9321   -4.0410 H   0  0  0  0  0  0  0  0  0  0  0  0
    2.0939    4.1977   -2.9141 H   0  0  0  0  0  0  0  0  0  0  0  0
   -0.5554   -3.5652    1.9174 C   0  0  0  0  0  0  0  0  0  0  0  0
    0.8232   -3.4510    2.1175 C   0  0  0  0  0  0  0  0  0  0  0  0
   -1.3106   -4.4561    2.6837 C   0  0  0  0  0  0  0  0  0  0  0  0
    1.4538   -4.2402    3.0816 C   0  0  0  0  0  0  0  0  0  0  0  0
    1.4405   -2.7621    1.5323 H   0  0  0  0  0  0  0  0  0  0  0  0
   -0.6799   -5.2380    3.6549 C   0  0  0  0  0  0  0  0  0  0  0  0
   -2.3905   -4.5645    2.5467 H   0  0  0  0  0  0  0  0  0  0  0  0
    0.7069   -5.1391    3.8607 C   0  0  0  0  0  0  0  0  0  0  0  0
    2.5300   -4.1474    3.2244 H   0  0  0  0  0  0  0  0  0  0  0  0
   -1.2692   -5.9272    4.2571 H   0  0  0  0  0  0  0  0  0  0  0  0
   -0.6152   -3.2218   -1.0044 C   0  0  0  0  0  0  0  0  0  0  0  0
   -0.6307   -2.4399   -2.1627 C   0  0  0  0  0  0  0  0  0  0  0  0
   -0.0557   -4.5023   -1.0215 C   0  0  0  0  0  0  0  0  0  0  0  0
   -0.0500   -2.9275   -3.3355 C   0  0  0  0  0  0  0  0  0  0  0  0
   -1.0831   -1.4426   -2.1860 H   0  0  0  0  0  0  0  0  0  0  0  0
    0.5119   -4.9950   -2.2006 C   0  0  0  0  0  0  0  0  0  0  0  0
   -0.0373   -5.1339   -0.1281 H   0  0  0  0  0  0  0  0  0  0  0  0
    0.5306   -4.2074   -3.3631 C   0  0  0  0  0  0  0  0  0  0  0  0
   -0.0325   -2.3009   -4.2277 H   0  0  0  0  0  0  0  0  0  0  0  0
    0.9491   -5.9914   -2.2089 H   0  0  0  0  0  0  0  0  0  0  0  0
   -3.1278   -3.1163    0.5461 C   0  0  0  0  0  0  0  0  0  0  0  0
   -3.5461   -4.0875   -0.3668 C   0  0  0  0  0  0  0  0  0  0  0  0
   -4.0243   -2.5828    1.4761 C   0  0  0  0  0  0  0  0  0  0  0  0
   -4.8700   -4.5337   -0.3429 C   0  0  0  0  0  0  0  0  0  0  0  0
   -2.8639   -4.5125   -1.1091 H   0  0  0  0  0  0  0  0  0  0  0  0
   -5.3464   -3.0340    1.5016 C   0  0  0  0  0  0  0  0  0  0  0  0
   -3.7219   -1.8168    2.1975 H   0  0  0  0  0  0  0  0  0  0  0  0
   -5.7800   -4.0148    0.5936 C   0  0  0  0  0  0  0  0  0  0  0  0
   -5.1980   -5.2871   -1.0572 H   0  0  0  0  0  0  0  0  0  0  0  0
   -6.0424   -2.6172    2.2280 H   0  0  0  0  0  0  0  0  0  0  0  0
   -0.1753    0.0305    2.0818 C   0  0  0  0  0  0  0  0  0  0  0  0
    0.5225   -0.0360    3.0124 O   0  0  0  0  0  0  0  0  0  0  0  0
   -1.0809    0.1402    0.8232 Ir  0  0  0  0  0  0  0  0  0  0  0  0
    2.5880    6.0418    4.5469 C   0  0  0  0  0  0  0  0  0  0  0  0
    2.1953    6.0169    5.5914 H   0  0  0  0  0  0  0  0  0  0  0  0
    3.5956    5.5598    4.5310 H   0  0  0  0  0  0  0  0  0  0  0  0
    2.6969    7.1076    4.2412 H   0  0  0  0  0  0  0  0  0  0  0  0
    5.0909    5.0477    1.0763 C   0  0  0  0  0  0  0  0  0  0  0  0
    6.0540    5.1333    0.5219 H   0  0  0  0  0  0  0  0  0  0  0  0
    4.4522    5.9309    0.8314 H   0  0  0  0  0  0  0  0  0  0  0  0
    5.3118    5.0593    2.1683 H   0  0  0  0  0  0  0  0  0  0  0  0
    0.4390    4.4045   -5.0571 C   0  0  0  0  0  0  0  0  0  0  0  0
   -0.4481    4.4263   -5.7297 H   0  0  0  0  0  0  0  0  0  0  0  0
    0.9414    5.4004   -5.1006 H   0  0  0  0  0  0  0  0  0  0  0  0
    1.1492    3.6263   -5.4306 H   0  0  0  0  0  0  0  0  0  0  0  0
    1.7537    0.1824   -6.7031 C   0  0  0  0  0  0  0  0  0  0  0  0
    1.0438   -0.5982   -7.0689 H   0  0  0  0  0  0  0  0  0  0  0  0
    1.4024    1.1803   -7.0561 H   0  0  0  0  0  0  0  0  0  0  0  0
    2.7571   -0.0126   -7.1458 H   0  0  0  0  0  0  0  0  0  0  0  0
    5.1503   -4.8995    1.0966 C   0  0  0  0  0  0  0  0  0  0  0  0
    6.1705   -4.8544    0.6437 H   0  0  0  0  0  0  0  0  0  0  0  0
    5.2526   -4.9396    2.2058 H   0  0  0  0  0  0  0  0  0  0  0  0
    4.6491   -5.8345    0.7566 H   0  0  0  0  0  0  0  0  0  0  0  0
    1.1790   -4.6987   -4.6173 C   0  0  0  0  0  0  0  0  0  0  0  0
```

```
    0.4831   -4.5808   -5.4820 H   0  0  0  0  0  0  0  0  0  0  0  0
    2.0995   -4.0951   -4.8126 H   0  0  0  0  0  0  0  0  0  0  0  0
    1.4661   -5.7719   -4.5408 H   0  0  0  0  0  0  0  0  0  0  0  0
   -7.1949   -4.4976    0.6071 C   0  0  0  0  0  0  0  0  0  0  0  0
   -7.7370   -4.0715   -0.2726 H   0  0  0  0  0  0  0  0  0  0  0  0
   -7.2261   -5.6110    0.5426 H   0  0  0  0  0  0  0  0  0  0  0  0
   -7.7259   -4.1847    1.5355 H   0  0  0  0  0  0  0  0  0  0  0  0
   -6.6871    5.2151    1.6214 C   0  0  0  0  0  0  0  0  0  0  0  0
   -7.4048    4.7296    0.9169 H   0  0  0  0  0  0  0  0  0  0  0  0
   -7.0169    5.0103    2.6689 H   0  0  0  0  0  0  0  0  0  0  0  0
   -6.7164    6.3157    1.4523 H   0  0  0  0  0  0  0  0  0  0  0  0
    1.3682   -5.9767    4.9081 C   0  0  0  0  0  0  0  0  0  0  0  0
    1.0877   -5.5886    5.9183 H   0  0  0  0  0  0  0  0  0  0  0  0
    1.0333   -7.0375    4.8259 H   0  0  0  0  0  0  0  0  0  0  0  0
    2.4778   -5.9478    4.8142 H   0  0  0  0  0  0  0  0  0  0  0  0
  1 97  1  0  0  0  0
  2  5  1  0  0  0  0
  2 15  1  0  0  0  0
  2 25  1  0  0  0  0
  2 97  1  0  0  0  0
  3 65  1  0  0  0  0
  3 75  1  0  0  0  0
  3 85  1  0  0  0  0
  3 97  1  0  0  0  0
  4 35  1  0  0  0  0
  4 45  1  0  0  0  0
  4 55  1  0  0  0  0
  4 97  1  0  0  0  0
  5  6  2  0  0  0  0
  5  7  1  0  0  0  0
  6  8  1  0  0  0  0
  6  9  1  0  0  0  0
  7 10  2  0  0  0  0
  7 11  1  0  0  0  0
  8 12  2  0  0  0  0
  8 13  1  0  0  0  0
 10 12  1  0  0  0  0
 10 14  1  0  0  0  0
 12102  1  0  0  0  0
 15 16  2  0  0  0  0
 15 17  1  0  0  0  0
 16 18  1  0  0  0  0
 16 19  1  0  0  0  0
 17 20  2  0  0  0  0
 17 21  1  0  0  0  0
 18 22  2  0  0  0  0
 18 23  1  0  0  0  0
 20 22  1  0  0  0  0
 20 24  1  0  0  0  0
 22114  1  0  0  0  0
 25 26  2  0  0  0  0
 25 27  1  0  0  0  0
 26 28  1  0  0  0  0
 26 29  1  0  0  0  0
 27 30  2  0  0  0  0
 27 31  1  0  0  0  0
 28 32  2  0  0  0  0
 28 33  1  0  0  0  0
 30 32  1  0  0  0  0
 30 34  1  0  0  0  0
 32110  1  0  0  0  0
 35 36  2  0  0  0  0
 35 37  1  0  0  0  0
 36 38  1  0  0  0  0
 36 39  1  0  0  0  0
```

```
 37 40  2  0  0  0  0
 37 41  1  0  0  0  0
 38 42  2  0  0  0  0
 38 43  1  0  0  0  0
 40 42  1  0  0  0  0
 40 44  1  0  0  0  0
 42 98  1  0  0  0  0
 45 46  2  0  0  0  0
 45 47  1  0  0  0  0
 46 48  1  0  0  0  0
 46 49  1  0  0  0  0
 47 50  2  0  0  0  0
 47 51  1  0  0  0  0
 48 52  2  0  0  0  0
 48 53  1  0  0  0  0
 50 52  1  0  0  0  0
 50 54  1  0  0  0  0
 52126  1  0  0  0  0
 55 56  2  0  0  0  0
 55 57  1  0  0  0  0
 56 58  1  0  0  0  0
 56 59  1  0  0  0  0
 57 60  2  0  0  0  0
 57 61  1  0  0  0  0
 58 62  2  0  0  0  0
 58 63  1  0  0  0  0
 60 62  1  0  0  0  0
 60 64  1  0  0  0  0
 62106  1  0  0  0  0
 65 66  2  0  0  0  0
 65 67  1  0  0  0  0
 66 68  1  0  0  0  0
 66 69  1  0  0  0  0
 67 70  2  0  0  0  0
 67 71  1  0  0  0  0
 68 72  2  0  0  0  0
 68 73  1  0  0  0  0
 70 72  1  0  0  0  0
 70 74  1  0  0  0  0
 72130  1  0  0  0  0
 75 76  2  0  0  0  0
 75 77  1  0  0  0  0
 76 78  1  0  0  0  0
 76 79  1  0  0  0  0
 77 80  2  0  0  0  0
 77 81  1  0  0  0  0
 78 82  2  0  0  0  0
 78 83  1  0  0  0  0
 80 82  1  0  0  0  0
 80 84  1  0  0  0  0
 82118  1  0  0  0  0
 85 86  2  0  0  0  0
 85 87  1  0  0  0  0
 86 88  1  0  0  0  0
 86 89  1  0  0  0  0
 87 90  2  0  0  0  0
 87 91  1  0  0  0  0
 88 92  2  0  0  0  0
 88 93  1  0  0  0  0
 90 92  1  0  0  0  0
 90 94  1  0  0  0  0
 92122  1  0  0  0  0
 95 96  2  0  0  0  0
 95 97  1  0  0  0  0
 98 99  1  0  0  0  0
```

```
 98100  1  0  0  0  0
 98101  1  0  0  0  0
102103  1  0  0  0  0
102104  1  0  0  0  0
102105  1  0  0  0  0
106107  1  0  0  0  0
106108  1  0  0  0  0
106109  1  0  0  0  0
110111  1  0  0  0  0
110112  1  0  0  0  0
110113  1  0  0  0  0
114115  1  0  0  0  0
114116  1  0  0  0  0
114117  1  0  0  0  0
118119  1  0  0  0  0
118120  1  0  0  0  0
118121  1  0  0  0  0
122123  1  0  0  0  0
122124  1  0  0  0  0
122125  1  0  0  0  0
126127  1  0  0  0  0
126128  1  0  0  0  0
126129  1  0  0  0  0
130131  1  0  0  0  0
130132  1  0  0  0  0
130133  1  0  0  0  0
M  END
$$$$
mol040
Optimized Catalyst 040
Optimized Catalyst 040
115123  0  0  0  0  0  0  0  0999 V2000
   -2.0026   -0.2468    0.3010 H   0  0  0  0  0  0  0  0  0  0  0  0
    1.4676   -0.0115   -0.5611 P   0  0  0  0  0  0  0  0  0  0  0  0
   -1.1029   -2.7335    1.3644 P   0  0  0  0  0  0  0  0  0  0  0  0
   -1.3096    2.5273    0.7775 P   0  0  0  0  0  0  0  0  0  0  0  0
    3.1881    1.9916   -0.3476 C   0  0  0  0  0  0  0  0  0  0  0  0
    3.5901    2.8222   -1.4039 C   0  0  0  0  0  0  0  0  0  0  0  0
    3.0767    2.4485    0.9713 C   0  0  0  0  0  0  0  0  0  0  0  0
    3.8906    4.1552   -1.1181 C   0  0  0  0  0  0  0  0  0  0  0  0
    3.6776    2.4167   -2.4115 H   0  0  0  0  0  0  0  0  0  0  0  0
    3.3734    3.7909    1.2257 C   0  0  0  0  0  0  0  0  0  0  0  0
    2.7837    1.7844    1.7827 H   0  0  0  0  0  0  0  0  0  0  0  0
    3.7727    4.6420    0.1887 C   0  0  0  0  0  0  0  0  0  0  0  0
    4.2220    4.8165   -1.9184 H   0  0  0  0  0  0  0  0  0  0  0  0
    3.2868    4.1757    2.2436 H   0  0  0  0  0  0  0  0  0  0  0  0
    2.9895   -2.0953    0.1832 C   0  0  0  0  0  0  0  0  0  0  0  0
    3.4486   -2.6180   -1.0293 C   0  0  0  0  0  0  0  0  0  0  0  0
    3.6412   -2.3168    1.4049 C   0  0  0  0  0  0  0  0  0  0  0  0
    4.6077   -3.4000   -1.0036 C   0  0  0  0  0  0  0  0  0  0  0  0
    2.9279   -2.4205   -1.9670 H   0  0  0  0  0  0  0  0  0  0  0  0
    4.7945   -3.1023    1.3993 C   0  0  0  0  0  0  0  0  0  0  0  0
    3.2359   -1.8937    2.3226 H   0  0  0  0  0  0  0  0  0  0  0  0
    5.2763   -3.6439    0.2002 C   0  0  0  0  0  0  0  0  0  0  0  0
    4.9897   -3.8215   -1.9336 H   0  0  0  0  0  0  0  0  0  0  0  0
    5.3215   -3.2927    2.3350 H   0  0  0  0  0  0  0  0  0  0  0  0
    1.4841    0.2674   -3.1530 C   0  0  0  0  0  0  0  0  0  0  0  0
    2.6934    0.3344   -3.8582 C   0  0  0  0  0  0  0  0  0  0  0  0
    0.3690    1.0438   -3.4906 C   0  0  0  0  0  0  0  0  0  0  0  0
    2.7803    1.2201   -4.9340 C   0  0  0  0  0  0  0  0  0  0  0  0
    3.5344   -0.2895   -3.5592 H   0  0  0  0  0  0  0  0  0  0  0  0
    0.4874    1.9293   -4.5655 C   0  0  0  0  0  0  0  0  0  0  0  0
   -0.5689    0.9571   -2.9416 H   0  0  0  0  0  0  0  0  0  0  0  0
    1.6862    2.0217   -5.2812 C   0  0  0  0  0  0  0  0  0  0  0  0
    3.7081    1.2881   -5.5028 H   0  0  0  0  0  0  0  0  0  0  0  0
```

```
   -0.3632    2.5556   -4.8391 H   0  0  0  0  0  0  0  0  0  0  0  0
    0.2101    4.5026    1.8135 C   0  0  0  0  0  0  0  0  0  0  0  0
    0.6302    5.5804    1.0278 C   0  0  0  0  0  0  0  0  0  0  0  0
    0.0114    4.5982    3.1959 C   0  0  0  0  0  0  0  0  0  0  0  0
    0.8688    6.8007    1.6660 C   0  0  0  0  0  0  0  0  0  0  0  0
    0.7929    5.4661   -0.0429 H   0  0  0  0  0  0  0  0  0  0  0  0
    0.2514    5.8324    3.8059 C   0  0  0  0  0  0  0  0  0  0  0  0
   -0.3074    3.7372    3.7811 H   0  0  0  0  0  0  0  0  0  0  0  0
    0.6782    6.9281    3.0462 C   0  0  0  0  0  0  0  0  0  0  0  0
    1.2089    7.6555    1.0802 H   0  0  0  0  0  0  0  0  0  0  0  0
    0.1083    5.9371    4.8832 H   0  0  0  0  0  0  0  0  0  0  0  0
   -3.2372    4.3834    0.8011 C   0  0  0  0  0  0  0  0  0  0  0  0
   -4.4241    3.9987    0.1658 C   0  0  0  0  0  0  0  0  0  0  0  0
   -2.8116    5.7113    0.8869 C   0  0  0  0  0  0  0  0  0  0  0  0
   -5.2002    4.9980   -0.4272 C   0  0  0  0  0  0  0  0  0  0  0  0
   -4.7385    2.9560    0.1544 H   0  0  0  0  0  0  0  0  0  0  0  0
   -3.6055    6.6889    0.2791 C   0  0  0  0  0  0  0  0  0  0  0  0
   -1.9076    5.9832    1.4301 H   0  0  0  0  0  0  0  0  0  0  0  0
   -4.7879    6.3353   -0.3803 C   0  0  0  0  0  0  0  0  0  0  0  0
   -6.1331    4.7305   -0.9263 H   0  0  0  0  0  0  0  0  0  0  0  0
   -3.2989    7.7349    0.3267 H   0  0  0  0  0  0  0  0  0  0  0  0
   -1.0357    3.6953   -1.6496 C   0  0  0  0  0  0  0  0  0  0  0  0
   -2.0664    4.4023   -2.2761 C   0  0  0  0  0  0  0  0  0  0  0  0
    0.3176    3.9493   -1.8915 C   0  0  0  0  0  0  0  0  0  0  0  0
   -1.7145    5.4152   -3.1719 C   0  0  0  0  0  0  0  0  0  0  0  0
   -3.1094    4.1558   -2.0808 H   0  0  0  0  0  0  0  0  0  0  0  0
    0.6416    4.9674   -2.7929 C   0  0  0  0  0  0  0  0  0  0  0  0
    1.1006    3.3644   -1.4080 H   0  0  0  0  0  0  0  0  0  0  0  0
   -0.3677    5.6996   -3.4272 C   0  0  0  0  0  0  0  0  0  0  0  0
   -2.4980    5.9822   -3.6767 H   0  0  0  0  0  0  0  0  0  0  0  0
    1.6906    5.1798   -3.0043 H   0  0  0  0  0  0  0  0  0  0  0  0
    0.4993   -4.7029    2.1269 C   0  0  0  0  0  0  0  0  0  0  0  0
    1.5799   -5.1150    1.3414 C   0  0  0  0  0  0  0  0  0  0  0  0
   -0.2239   -5.5671    2.9535 C   0  0  0  0  0  0  0  0  0  0  0  0
    1.9497   -6.4606    1.3962 C   0  0  0  0  0  0  0  0  0  0  0  0
    2.1100   -4.4045    0.7055 H   0  0  0  0  0  0  0  0  0  0  0  0
    0.1654   -6.9096    2.9839 C   0  0  0  0  0  0  0  0  0  0  0  0
   -1.0663   -5.2072    3.5457 H   0  0  0  0  0  0  0  0  0  0  0  0
    1.2436   -7.3546    2.2103 C   0  0  0  0  0  0  0  0  0  0  0  0
    2.7890   -6.8109    0.7944 H   0  0  0  0  0  0  0  0  0  0  0  0
   -0.3777   -7.6114    3.6177 H   0  0  0  0  0  0  0  0  0  0  0  0
   -0.6315   -4.2153   -0.8392 C   0  0  0  0  0  0  0  0  0  0  0  0
    0.3130   -3.9925   -1.8487 C   0  0  0  0  0  0  0  0  0  0  0  0
   -1.0997   -5.4848   -0.4917 C   0  0  0  0  0  0  0  0  0  0  0  0
    0.8347   -5.1058   -2.5090 C   0  0  0  0  0  0  0  0  0  0  0  0
    0.6096   -2.9721   -2.1053 H   0  0  0  0  0  0  0  0  0  0  0  0
   -0.5557   -6.5810   -1.1701 C   0  0  0  0  0  0  0  0  0  0  0  0
   -1.8550   -5.6332    0.2794 H   0  0  0  0  0  0  0  0  0  0  0  0
    0.4104   -6.3958   -2.1646 C   0  0  0  0  0  0  0  0  0  0  0  0
    1.5778   -4.9659   -3.2948 H   0  0  0  0  0  0  0  0  0  0  0  0
   -0.8891   -7.5872   -0.9131 H   0  0  0  0  0  0  0  0  0  0  0  0
   -3.5986   -3.6484    1.7539 C   0  0  0  0  0  0  0  0  0  0  0  0
   -4.1128   -3.5698    0.4570 C   0  0  0  0  0  0  0  0  0  0  0  0
   -4.4105   -3.7432    2.8938 C   0  0  0  0  0  0  0  0  0  0  0  0
   -5.5047   -3.5870    0.3088 C   0  0  0  0  0  0  0  0  0  0  0  0
   -3.4702   -3.5123   -0.4237 H   0  0  0  0  0  0  0  0  0  0  0  0
   -5.7940   -3.7687    2.7125 C   0  0  0  0  0  0  0  0  0  0  0  0
   -3.9580   -3.8067    3.8819 H   0  0  0  0  0  0  0  0  0  0  0  0
   -6.3408   -3.6879    1.4249 C   0  0  0  0  0  0  0  0  0  0  0  0
   -5.9353   -3.5244   -0.6923 H   0  0  0  0  0  0  0  0  0  0  0  0
   -6.4510   -3.8500    3.5799 H   0  0  0  0  0  0  0  0  0  0  0  0
   -0.0663    0.1015    2.4229 C   0  0  0  0  0  0  0  0  0  0  0  0
    0.7086    0.2606    3.2746 O   0  0  0  0  0  0  0  0  0  0  0  0
    1.7665    2.7162   -6.1157 H   0  0  0  0  0  0  0  0  0  0  0  0
    6.1789   -4.2525    0.2066 H   0  0  0  0  0  0  0  0  0  0  0  0
```

```
    3.9969    5.6855    0.4033 H   0  0  0  0  0  0  0  0  0  0  0  0
    1.5368   -8.4033    2.2429 H   0  0  0  0  0  0  0  0  0  0  0  0
   -7.4225   -3.7051    1.2935 H   0  0  0  0  0  0  0  0  0  0  0  0
   -5.3983    7.1061   -0.8510 H   0  0  0  0  0  0  0  0  0  0  0  0
   -0.1046    6.4911   -4.1284 H   0  0  0  0  0  0  0  0  0  0  0  0
    0.8679    7.8839    3.5348 H   0  0  0  0  0  0  0  0  0  0  0  0
    0.8318   -7.2591   -2.6776 H   0  0  0  0  0  0  0  0  0  0  0  0
   -1.1015   -0.0829    1.2659 Ir  0  0  0  0  0  0  0  0  0  0  0  0
   -1.1400   -3.0348   -0.2676 O   0  0  0  0  0  0  0  0  0  0  0  0
    0.2294   -3.3122    2.1431 O   0  0  0  0  0  0  0  0  0  0  0  0
   -2.2326   -3.7710    2.0378 O   0  0  0  0  0  0  0  0  0  0  0  0
   -1.4003    2.5793   -0.8774 O   0  0  0  0  0  0  0  0  0  0  0  0
   -2.5290    3.3870    1.4982 O   0  0  0  0  0  0  0  0  0  0  0  0
    0.1326    3.2384    1.2107 O   0  0  0  0  0  0  0  0  0  0  0  0
    1.3949   -0.6828   -2.1272 O   0  0  0  0  0  0  0  0  0  0  0  0
    3.0322    0.6439   -0.6649 O   0  0  0  0  0  0  0  0  0  0  0  0
    1.7690   -1.4129    0.3013 O   0  0  0  0  0  0  0  0  0  0  0  0
  1106  1  0  0  0  0
  2113  1  0  0  0  0
  2114  1  0  0  0  0
  2115  1  0  0  0  0
  2106  1  0  0  0  0
  3107  1  0  0  0  0
  3108  1  0  0  0  0
  3109  1  0  0  0  0
  3106  1  0  0  0  0
  4110  1  0  0  0  0
  4111  1  0  0  0  0
  4112  1  0  0  0  0
  4106  1  0  0  0  0
  5  6  2  0  0  0  0
  5  7  1  0  0  0  0
  5114  1  0  0  0  0
  6  8  1  0  0  0  0
  6  9  1  0  0  0  0
  7 10  2  0  0  0  0
  7 11  1  0  0  0  0
  8 12  2  0  0  0  0
  8 13  1  0  0  0  0
 10 12  1  0  0  0  0
 10 14  1  0  0  0  0
 12 99  1  0  0  0  0
 15 16  2  0  0  0  0
 15 17  1  0  0  0  0
 15115  1  0  0  0  0
 16 18  1  0  0  0  0
 16 19  1  0  0  0  0
 17 20  2  0  0  0  0
 17 21  1  0  0  0  0
 18 22  2  0  0  0  0
 18 23  1  0  0  0  0
 20 22  1  0  0  0  0
 20 24  1  0  0  0  0
 22 98  1  0  0  0  0
 25 26  2  0  0  0  0
 25 27  1  0  0  0  0
 25113  1  0  0  0  0
 26 28  1  0  0  0  0
 26 29  1  0  0  0  0
 27 30  2  0  0  0  0
 27 31  1  0  0  0  0
 28 32  2  0  0  0  0
 28 33  1  0  0  0  0
 30 32  1  0  0  0  0
 30 34  1  0  0  0  0
```

```
 32 97  1  0  0  0  0
 35 36  2  0  0  0  0
 35 37  1  0  0  0  0
 35112  1  0  0  0  0
 36 38  1  0  0  0  0
 36 39  1  0  0  0  0
 37 40  2  0  0  0  0
 37 41  1  0  0  0  0
 38 42  2  0  0  0  0
 38 43  1  0  0  0  0
 40 42  1  0  0  0  0
 40 44  1  0  0  0  0
 42104  1  0  0  0  0
 45 46  2  0  0  0  0
 45 47  1  0  0  0  0
 45111  1  0  0  0  0
 46 48  1  0  0  0  0
 46 49  1  0  0  0  0
 47 50  2  0  0  0  0
 47 51  1  0  0  0  0
 48 52  2  0  0  0  0
 48 53  1  0  0  0  0
 50 52  1  0  0  0  0
 50 54  1  0  0  0  0
 52102  1  0  0  0  0
 55 56  2  0  0  0  0
 55 57  1  0  0  0  0
 55110  1  0  0  0  0
 56 58  1  0  0  0  0
 56 59  1  0  0  0  0
 57 60  2  0  0  0  0
 57 61  1  0  0  0  0
 58 62  2  0  0  0  0
 58 63  1  0  0  0  0
 60 62  1  0  0  0  0
 60 64  1  0  0  0  0
 62103  1  0  0  0  0
 65 66  2  0  0  0  0
 65 67  1  0  0  0  0
 65108  1  0  0  0  0
 66 68  1  0  0  0  0
 66 69  1  0  0  0  0
 67 70  2  0  0  0  0
 67 71  1  0  0  0  0
 68 72  2  0  0  0  0
 68 73  1  0  0  0  0
 70 72  1  0  0  0  0
 70 74  1  0  0  0  0
 72100  1  0  0  0  0
 75 76  2  0  0  0  0
 75 77  1  0  0  0  0
 75107  1  0  0  0  0
 76 78  1  0  0  0  0
 76 79  1  0  0  0  0
 77 80  2  0  0  0  0
 77 81  1  0  0  0  0
 78 82  2  0  0  0  0
 78 83  1  0  0  0  0
 80 82  1  0  0  0  0
 80 84  1  0  0  0  0
 82105  1  0  0  0  0
 85 86  2  0  0  0  0
 85 87  1  0  0  0  0
 85109  1  0  0  0  0
 86 88  1  0  0  0  0
```

```
 86 89  1  0  0  0  0
 87 90  2  0  0  0  0
 87 91  1  0  0  0  0
 88 92  2  0  0  0  0
 88 93  1  0  0  0  0
 90 92  1  0  0  0  0
 90 94  1  0  0  0  0
 92101  1  0  0  0  0
 95 96  2  0  0  0  0
 95106  1  0  0  0  0
M  END
$$$$
mol041
Optimized Catalyst 041
Optimized Catalyst 041
115123  0  0  0  0  0  0  0  0999 V2000
   -1.9781   -0.1579    0.4019 H   0  0  0  0  0  0  0  0  0  0  0  0
    1.4208    0.1996   -0.4570 P   0  0  0  0  0  0  0  0  0  0  0  0
   -1.1009   -2.6165    1.4832 P   0  0  0  0  0  0  0  0  0  0  0  0
   -1.5593    2.6199    0.8732 P   0  0  0  0  0  0  0  0  0  0  0  0
    3.2244    2.1320   -0.4610 C   0  0  0  0  0  0  0  0  0  0  0  0
    3.3568    2.9400   -1.6016 C   0  0  0  0  0  0  0  0  0  0  0  0
    3.4614    2.6073    0.8370 C   0  0  0  0  0  0  0  0  0  0  0  0
    3.7326    4.2732   -1.4390 C   0  0  0  0  0  0  0  0  0  0  0  0
    3.1860    2.5213   -2.5951 H   0  0  0  0  0  0  0  0  0  0  0  0
    3.8435    3.9400    0.9992 C   0  0  0  0  0  0  0  0  0  0  0  0
    3.3830    1.9427    1.6978 H   0  0  0  0  0  0  0  0  0  0  0  0
    3.9589    4.7442   -0.1398 C   0  0  0  0  0  0  0  0  0  0  0  0
    3.8537    4.9250   -2.3058 H   0  0  0  0  0  0  0  0  0  0  0  0
    4.0650    4.3347    1.9920 H   0  0  0  0  0  0  0  0  0  0  0  0
    2.8757   -1.8870    0.4260 C   0  0  0  0  0  0  0  0  0  0  0  0
    3.3177   -2.4948   -0.7546 C   0  0  0  0  0  0  0  0  0  0  0  0
    3.5176   -2.0613    1.6634 C   0  0  0  0  0  0  0  0  0  0  0  0
    4.4429   -3.3211   -0.6949 C   0  0  0  0  0  0  0  0  0  0  0  0
    2.8043   -2.3310   -1.7040 H   0  0  0  0  0  0  0  0  0  0  0  0
    4.6373   -2.8890    1.7233 C   0  0  0  0  0  0  0  0  0  0  0  0
    3.1247   -1.5672    2.5526 H   0  0  0  0  0  0  0  0  0  0  0  0
    5.0730   -3.5011    0.5404 C   0  0  0  0  0  0  0  0  0  0  0  0
    4.8168   -3.8114   -1.5946 H   0  0  0  0  0  0  0  0  0  0  0  0
    5.1588   -3.0533    2.6679 H   0  0  0  0  0  0  0  0  0  0  0  0
    1.4096    0.2726   -3.0648 C   0  0  0  0  0  0  0  0  0  0  0  0
    2.6052    0.1836   -3.7949 C   0  0  0  0  0  0  0  0  0  0  0  0
    0.3472    1.1041   -3.4483 C   0  0  0  0  0  0  0  0  0  0  0  0
    2.7447    0.9544   -4.9491 C   0  0  0  0  0  0  0  0  0  0  0  0
    3.4065   -0.4728   -3.4528 H   0  0  0  0  0  0  0  0  0  0  0  0
    0.4905    1.8854   -4.5962 C   0  0  0  0  0  0  0  0  0  0  0  0
   -0.5759    1.1362   -2.8683 H   0  0  0  0  0  0  0  0  0  0  0  0
    1.6843    1.7901   -5.3202 C   0  0  0  0  0  0  0  0  0  0  0  0
    3.6577    0.9047   -5.5448 H   0  0  0  0  0  0  0  0  0  0  0  0
   -0.3123    2.5500   -4.9188 H   0  0  0  0  0  0  0  0  0  0  0  0
   -0.1478    4.5158    2.1695 C   0  0  0  0  0  0  0  0  0  0  0  0
    0.2105    5.7810    1.6760 C   0  0  0  0  0  0  0  0  0  0  0  0
    0.5723    3.8548    3.1707 C   0  0  0  0  0  0  0  0  0  0  0  0
    1.3373    6.4096    2.2026 C   0  0  0  0  0  0  0  0  0  0  0  0
   -0.4023    6.2642    0.9129 H   0  0  0  0  0  0  0  0  0  0  0  0
    1.7044    4.4844    3.6963 C   0  0  0  0  0  0  0  0  0  0  0  0
    0.2570    2.8933    3.5767 H   0  0  0  0  0  0  0  0  0  0  0  0
    2.0567    5.7468    3.2060 C   0  0  0  0  0  0  0  0  0  0  0  0
    1.6448    7.3954    1.8465 H   0  0  0  0  0  0  0  0  0  0  0  0
    2.2877    4.0019    4.4847 H   0  0  0  0  0  0  0  0  0  0  0  0
   -3.7704    3.8169   -0.0693 C   0  0  0  0  0  0  0  0  0  0  0  0
   -4.6208    3.4468   -1.1240 C   0  0  0  0  0  0  0  0  0  0  0  0
   -3.6076    5.1399    0.3542 C   0  0  0  0  0  0  0  0  0  0  0  0
   -5.3059    4.4476   -1.8107 C   0  0  0  0  0  0  0  0  0  0  0  0
   -4.7470    2.3930   -1.3763 H   0  0  0  0  0  0  0  0  0  0  0  0
```

```
   -4.2828    6.1439   -0.3446 C   0  0  0  0  0  0  0  0  0  0  0  0
   -2.9848    5.3944    1.2177 H   0  0  0  0  0  0  0  0  0  0  0  0
   -5.1077    5.7767   -1.4133 C   0  0  0  0  0  0  0  0  0  0  0  0
   -5.9808    4.1988   -2.6321 H   0  0  0  0  0  0  0  0  0  0  0  0
   -4.1725    7.1902   -0.0527 H   0  0  0  0  0  0  0  0  0  0  0  0
   -0.6437    3.8492   -1.3603 C   0  0  0  0  0  0  0  0  0  0  0  0
   -1.6031    3.9847   -2.3715 C   0  0  0  0  0  0  0  0  0  0  0  0
    0.4484    4.7134   -1.2196 C   0  0  0  0  0  0  0  0  0  0  0  0
   -1.4883    5.0582   -3.2553 C   0  0  0  0  0  0  0  0  0  0  0  0
   -2.4139    3.2631   -2.4764 H   0  0  0  0  0  0  0  0  0  0  0  0
    0.5684    5.7842   -2.1062 C   0  0  0  0  0  0  0  0  0  0  0  0
    1.1987    4.5377   -0.4458 H   0  0  0  0  0  0  0  0  0  0  0  0
   -0.4084    5.9364   -3.0977 C   0  0  0  0  0  0  0  0  0  0  0  0
   -2.2240    5.2059   -4.0487 H   0  0  0  0  0  0  0  0  0  0  0  0
    1.4067    6.4792   -2.0259 H   0  0  0  0  0  0  0  0  0  0  0  0
    0.4602   -4.6386    2.1714 C   0  0  0  0  0  0  0  0  0  0  0  0
    1.5412   -5.0523    1.3818 C   0  0  0  0  0  0  0  0  0  0  0  0
   -0.2898   -5.5109    2.9695 C   0  0  0  0  0  0  0  0  0  0  0  0
    1.8886   -6.4028    1.3859 C   0  0  0  0  0  0  0  0  0  0  0  0
    2.0938   -4.3294    0.7783 H   0  0  0  0  0  0  0  0  0  0  0  0
    0.0528   -6.8646    2.9665 C   0  0  0  0  0  0  0  0  0  0  0  0
   -1.1255   -5.1461    3.5724 H   0  0  0  0  0  0  0  0  0  0  0  0
    1.1311   -7.2777    2.1750 C   0  0  0  0  0  0  0  0  0  0  0  0
    2.7268   -6.7627    0.7862 H   0  0  0  0  0  0  0  0  0  0  0  0
   -0.5067   -7.5795    3.5722 H   0  0  0  0  0  0  0  0  0  0  0  0
   -0.6799   -4.0420   -0.7668 C   0  0  0  0  0  0  0  0  0  0  0  0
    0.2408   -3.7886   -1.7950 C   0  0  0  0  0  0  0  0  0  0  0  0
   -1.1421   -5.3230   -0.4458 C   0  0  0  0  0  0  0  0  0  0  0  0
    0.7517   -4.8699   -2.5101 C   0  0  0  0  0  0  0  0  0  0  0  0
    0.5246   -2.7562   -2.0271 H   0  0  0  0  0  0  0  0  0  0  0  0
   -0.6273   -6.4066   -1.1624 C   0  0  0  0  0  0  0  0  0  0  0  0
   -1.8781   -5.4903    0.3431 H   0  0  0  0  0  0  0  0  0  0  0  0
    0.3110   -6.1559   -2.1696 C   0  0  0  0  0  0  0  0  0  0  0  0
    1.4720   -4.7140   -3.3145 H   0  0  0  0  0  0  0  0  0  0  0  0
   -0.9526   -7.4237   -0.9356 H   0  0  0  0  0  0  0  0  0  0  0  0
   -3.6045   -3.4207    1.9171 C   0  0  0  0  0  0  0  0  0  0  0  0
   -4.1858   -3.4398    0.6430 C   0  0  0  0  0  0  0  0  0  0  0  0
   -4.3513   -3.3057    3.1004 C   0  0  0  0  0  0  0  0  0  0  0  0
   -5.5757   -3.3332    0.5495 C   0  0  0  0  0  0  0  0  0  0  0  0
   -3.5827   -3.5417   -0.2627 H   0  0  0  0  0  0  0  0  0  0  0  0
   -5.7386   -3.2053    3.0061 C   0  0  0  0  0  0  0  0  0  0  0  0
   -3.8461   -3.3119    4.0670 H   0  0  0  0  0  0  0  0  0  0  0  0
   -6.3185   -3.2191    1.7304 C   0  0  0  0  0  0  0  0  0  0  0  0
   -6.0667   -3.3448   -0.4260 H   0  0  0  0  0  0  0  0  0  0  0  0
   -6.3535   -3.1189    3.9045 H   0  0  0  0  0  0  0  0  0  0  0  0
   -0.1316    0.2482    2.5604 C   0  0  0  0  0  0  0  0  0  0  0  0
    0.6131    0.4507    3.4359 O   0  0  0  0  0  0  0  0  0  0  0  0
    1.8206    2.5368   -6.4169 F   0  0  0  0  0  0  0  0  0  0  0  0
    6.1402   -4.2995    0.5966 F   0  0  0  0  0  0  0  0  0  0  0  0
    4.2969    6.0250    0.0201 F   0  0  0  0  0  0  0  0  0  0  0  0
    1.4516   -8.5715    2.1693 F   0  0  0  0  0  0  0  0  0  0  0  0
   -7.6442   -3.1186    1.6377 F   0  0  0  0  0  0  0  0  0  0  0  0
   -5.7410    6.7377   -2.0858 F   0  0  0  0  0  0  0  0  0  0  0  0
   -0.3019    6.9644   -3.9384 F   0  0  0  0  0  0  0  0  0  0  0  0
    3.1265    6.3512    3.7223 F   0  0  0  0  0  0  0  0  0  0  0  0
    0.8107   -7.1934   -2.8420 F   0  0  0  0  0  0  0  0  0  0  0  0
   -1.1109    0.0343    1.3904 Ir  0  0  0  0  0  0  0  0  0  0  0  0
   -1.1741   -2.8780   -0.1574 O   0  0  0  0  0  0  0  0  0  0  0  0
    0.2328   -3.2434    2.2260 O   0  0  0  0  0  0  0  0  0  0  0  0
   -2.2401   -3.6718    2.1188 O   0  0  0  0  0  0  0  0  0  0  0  0
   -0.6788    2.7144   -0.5377 O   0  0  0  0  0  0  0  0  0  0  0  0
   -3.1836    2.7134    0.5795 O   0  0  0  0  0  0  0  0  0  0  0  0
   -1.3890    4.0521    1.7192 O   0  0  0  0  0  0  0  0  0  0  0  0
    1.2678   -0.5838   -1.9670 O   0  0  0  0  0  0  0  0  0  0  0  0
    3.0083    0.7713   -0.6625 O   0  0  0  0  0  0  0  0  0  0  0  0
```

```
    1.6848   -1.1521    0.5011 O   0  0  0  0  0  0  0  0  0  0  0  0
  1106  1  0  0  0  0
  2113  1  0  0  0  0
  2114  1  0  0  0  0
  2115  1  0  0  0  0
  2106  1  0  0  0  0
  3107  1  0  0  0  0
  3108  1  0  0  0  0
  3109  1  0  0  0  0
  3106  1  0  0  0  0
  4110  1  0  0  0  0
  4111  1  0  0  0  0
  4112  1  0  0  0  0
  4106  1  0  0  0  0
  5  6  2  0  0  0  0
  5  7  1  0  0  0  0
  5114  1  0  0  0  0
  6  8  1  0  0  0  0
  6  9  1  0  0  0  0
  7 10  2  0  0  0  0
  7 11  1  0  0  0  0
  8 12  2  0  0  0  0
  8 13  1  0  0  0  0
 10 12  1  0  0  0  0
 10 14  1  0  0  0  0
 12 99  1  0  0  0  0
 15 16  2  0  0  0  0
 15 17  1  0  0  0  0
 15115  1  0  0  0  0
 16 18  1  0  0  0  0
 16 19  1  0  0  0  0
 17 20  2  0  0  0  0
 17 21  1  0  0  0  0
 18 22  2  0  0  0  0
 18 23  1  0  0  0  0
 20 22  1  0  0  0  0
 20 24  1  0  0  0  0
 22 98  1  0  0  0  0
 25 26  2  0  0  0  0
 25 27  1  0  0  0  0
 25113  1  0  0  0  0
 26 28  1  0  0  0  0
 26 29  1  0  0  0  0
 27 30  2  0  0  0  0
 27 31  1  0  0  0  0
 28 32  2  0  0  0  0
 28 33  1  0  0  0  0
 30 32  1  0  0  0  0
 30 34  1  0  0  0  0
 32 97  1  0  0  0  0
 35 36  2  0  0  0  0
 35 37  1  0  0  0  0
 35112  1  0  0  0  0
 36 38  1  0  0  0  0
 36 39  1  0  0  0  0
 37 40  2  0  0  0  0
 37 41  1  0  0  0  0
 38 42  2  0  0  0  0
 38 43  1  0  0  0  0
 40 42  1  0  0  0  0
 40 44  1  0  0  0  0
 42104  1  0  0  0  0
 45 46  2  0  0  0  0
 45 47  1  0  0  0  0
 45111  1  0  0  0  0
```

```
 46 48  1  0  0  0  0
 46 49  1  0  0  0  0
 47 50  2  0  0  0  0
 47 51  1  0  0  0  0
 48 52  2  0  0  0  0
 48 53  1  0  0  0  0
 50 52  1  0  0  0  0
 50 54  1  0  0  0  0
 52102  1  0  0  0  0
 55 56  2  0  0  0  0
 55 57  1  0  0  0  0
 55110  1  0  0  0  0
 56 58  1  0  0  0  0
 56 59  1  0  0  0  0
 57 60  2  0  0  0  0
 57 61  1  0  0  0  0
 58 62  2  0  0  0  0
 58 63  1  0  0  0  0
 60 62  1  0  0  0  0
 60 64  1  0  0  0  0
 62103  1  0  0  0  0
 65 66  2  0  0  0  0
 65 67  1  0  0  0  0
 65108  1  0  0  0  0
 66 68  1  0  0  0  0
 66 69  1  0  0  0  0
 67 70  2  0  0  0  0
 67 71  1  0  0  0  0
 68 72  2  0  0  0  0
 68 73  1  0  0  0  0
 70 72  1  0  0  0  0
 70 74  1  0  0  0  0
 72100  1  0  0  0  0
 75 76  2  0  0  0  0
 75 77  1  0  0  0  0
 75107  1  0  0  0  0
 76 78  1  0  0  0  0
 76 79  1  0  0  0  0
 77 80  2  0  0  0  0
 77 81  1  0  0  0  0
 78 82  2  0  0  0  0
 78 83  1  0  0  0  0
 80 82  1  0  0  0  0
 80 84  1  0  0  0  0
 82105  1  0  0  0  0
 85 86  2  0  0  0  0
 85 87  1  0  0  0  0
 85109  1  0  0  0  0
 86 88  1  0  0  0  0
 86 89  1  0  0  0  0
 87 90  2  0  0  0  0
 87 91  1  0  0  0  0
 88 92  2  0  0  0  0
 88 93  1  0  0  0  0
 90 92  1  0  0  0  0
 90 94  1  0  0  0  0
 92101  1  0  0  0  0
 95 96  2  0  0  0  0
 95106  1  0  0  0  0
M  END
$$$$
mol042
Optimized Catalyst 042
Optimized Catalyst 042
142150  0  0  0  0  0  0  0  0999 V2000
```

```
   -1.9696   -0.1483    0.4456 H   0  0  0  0  0  0  0  0  0  0  0  0
    1.4111    0.1162   -0.5091 P   0  0  0  0  0  0  0  0  0  0  0  0
   -1.0856   -2.6180    1.4976 P   0  0  0  0  0  0  0  0  0  0  0  0
   -1.4645    2.6353    0.8650 P   0  0  0  0  0  0  0  0  0  0  0  0
    3.1619    2.0851   -0.5234 C   0  0  0  0  0  0  0  0  0  0  0  0
    3.3278    2.8806   -1.6664 C   0  0  0  0  0  0  0  0  0  0  0  0
    3.3150    2.5933    0.7713 C   0  0  0  0  0  0  0  0  0  0  0  0
    3.6599    4.2233   -1.4972 C   0  0  0  0  0  0  0  0  0  0  0  0
    3.2062    2.4459   -2.6593 H   0  0  0  0  0  0  0  0  0  0  0  0
    3.6489    3.9415    0.9149 C   0  0  0  0  0  0  0  0  0  0  0  0
    3.2057    1.9506    1.6432 H   0  0  0  0  0  0  0  0  0  0  0  0
    3.8188    4.7660   -0.2083 C   0  0  0  0  0  0  0  0  0  0  0  0
    3.7903    4.8563   -2.3741 H   0  0  0  0  0  0  0  0  0  0  0  0
    3.7775    4.3500    1.9167 H   0  0  0  0  0  0  0  0  0  0  0  0
    2.8831   -1.9503    0.4006 C   0  0  0  0  0  0  0  0  0  0  0  0
    3.3361   -2.5583   -0.7732 C   0  0  0  0  0  0  0  0  0  0  0  0
    3.5174   -2.1312    1.6384 C   0  0  0  0  0  0  0  0  0  0  0  0
    4.4592   -3.3852   -0.6931 C   0  0  0  0  0  0  0  0  0  0  0  0
    2.8316   -2.3966   -1.7264 H   0  0  0  0  0  0  0  0  0  0  0  0
    4.6328   -2.9644    1.6935 C   0  0  0  0  0  0  0  0  0  0  0  0
    3.1213   -1.6424    2.5273 H   0  0  0  0  0  0  0  0  0  0  0  0
    5.1097   -3.6008    0.5326 C   0  0  0  0  0  0  0  0  0  0  0  0
    4.8298   -3.8644   -1.5981 H   0  0  0  0  0  0  0  0  0  0  0  0
    5.1364   -3.1207    2.6470 H   0  0  0  0  0  0  0  0  0  0  0  0
    1.3892    0.2102   -3.1038 C   0  0  0  0  0  0  0  0  0  0  0  0
    2.5522    0.1050   -3.8775 C   0  0  0  0  0  0  0  0  0  0  0  0
    0.3537    1.0960   -3.4282 C   0  0  0  0  0  0  0  0  0  0  0  0
    2.6832    0.9288   -4.9958 C   0  0  0  0  0  0  0  0  0  0  0  0
    3.3329   -0.5993   -3.5941 H   0  0  0  0  0  0  0  0  0  0  0  0
    0.5129    1.9213   -4.5417 C   0  0  0  0  0  0  0  0  0  0  0  0
   -0.5555    1.1404   -2.8296 H   0  0  0  0  0  0  0  0  0  0  0  0
    1.6756    1.8507   -5.3283 C   0  0  0  0  0  0  0  0  0  0  0  0
    3.5794    0.8554   -5.6107 H   0  0  0  0  0  0  0  0  0  0  0  0
   -0.2761    2.6289   -4.7964 H   0  0  0  0  0  0  0  0  0  0  0  0
   -0.1072    4.5449    2.2082 C   0  0  0  0  0  0  0  0  0  0  0  0
    0.2041    5.8328    1.7521 C   0  0  0  0  0  0  0  0  0  0  0  0
    0.6480    3.8894    3.1854 C   0  0  0  0  0  0  0  0  0  0  0  0
    1.3052    6.4851    2.3064 C   0  0  0  0  0  0  0  0  0  0  0  0
   -0.4190    6.3113    0.9965 H   0  0  0  0  0  0  0  0  0  0  0  0
    1.7490    4.5616    3.7213 C   0  0  0  0  0  0  0  0  0  0  0  0
    0.3844    2.9004    3.5570 H   0  0  0  0  0  0  0  0  0  0  0  0
    2.0838    5.8596    3.2956 C   0  0  0  0  0  0  0  0  0  0  0  0
    1.5538    7.4917    1.9714 H   0  0  0  0  0  0  0  0  0  0  0  0
    2.3480    4.0712    4.4907 H   0  0  0  0  0  0  0  0  0  0  0  0
   -3.6827    3.8179   -0.0917 C   0  0  0  0  0  0  0  0  0  0  0  0
   -4.5305    3.4574   -1.1492 C   0  0  0  0  0  0  0  0  0  0  0  0
   -3.5372    5.1368    0.3417 C   0  0  0  0  0  0  0  0  0  0  0  0
   -5.2254    4.4672   -1.8108 C   0  0  0  0  0  0  0  0  0  0  0  0
   -4.6446    2.4086   -1.4211 H   0  0  0  0  0  0  0  0  0  0  0  0
   -4.2329    6.1333   -0.3482 C   0  0  0  0  0  0  0  0  0  0  0  0
   -2.9139    5.3927    1.2022 H   0  0  0  0  0  0  0  0  0  0  0  0
   -5.0707    5.8130   -1.4282 C   0  0  0  0  0  0  0  0  0  0  0  0
   -5.8965    4.2079   -2.6294 H   0  0  0  0  0  0  0  0  0  0  0  0
   -4.1248    7.1704   -0.0305 H   0  0  0  0  0  0  0  0  0  0  0  0
   -0.5668    3.9150   -1.3463 C   0  0  0  0  0  0  0  0  0  0  0  0
   -1.5061    4.0063   -2.3772 C   0  0  0  0  0  0  0  0  0  0  0  0
    0.4786    4.8304   -1.1939 C   0  0  0  0  0  0  0  0  0  0  0  0
   -1.4016    5.0739   -3.2708 C   0  0  0  0  0  0  0  0  0  0  0  0
   -2.2897    3.2587   -2.4911 H   0  0  0  0  0  0  0  0  0  0  0  0
    0.5695    5.8837   -2.1027 C   0  0  0  0  0  0  0  0  0  0  0  0
    1.2172    4.6998   -0.4003 H   0  0  0  0  0  0  0  0  0  0  0  0
   -0.3679    6.0170   -3.1432 C   0  0  0  0  0  0  0  0  0  0  0  0
   -2.1328    5.1667   -4.0734 H   0  0  0  0  0  0  0  0  0  0  0  0
    1.3814    6.6035   -2.0066 H   0  0  0  0  0  0  0  0  0  0  0  0
    0.4734   -4.6467    2.1723 C   0  0  0  0  0  0  0  0  0  0  0  0
```

```
    1.5485   -5.0596    1.3802 C   0  0  0  0  0  0  0  0  0  0  0  0
   -0.2754   -5.5300    2.9566 C   0  0  0  0  0  0  0  0  0  0  0  0
    1.8798   -6.4148    1.3716 C   0  0  0  0  0  0  0  0  0  0  0  0
    2.1061   -4.3374    0.7816 H   0  0  0  0  0  0  0  0  0  0  0  0
    0.0697   -6.8815    2.9263 C   0  0  0  0  0  0  0  0  0  0  0  0
   -1.1097   -5.1734    3.5633 H   0  0  0  0  0  0  0  0  0  0  0  0
    1.1419   -7.3346    2.1358 C   0  0  0  0  0  0  0  0  0  0  0  0
    2.7134   -6.7514    0.7564 H   0  0  0  0  0  0  0  0  0  0  0  0
   -0.5012   -7.5913    3.5244 H   0  0  0  0  0  0  0  0  0  0  0  0
   -0.6939   -4.0669   -0.7373 C   0  0  0  0  0  0  0  0  0  0  0  0
    0.2245   -3.8356   -1.7701 C   0  0  0  0  0  0  0  0  0  0  0  0
   -1.1525   -5.3416   -0.4000 C   0  0  0  0  0  0  0  0  0  0  0  0
    0.7305   -4.9358   -2.4568 C   0  0  0  0  0  0  0  0  0  0  0  0
    0.5132   -2.8105   -2.0226 H   0  0  0  0  0  0  0  0  0  0  0  0
   -0.6261   -6.4304   -1.1018 C   0  0  0  0  0  0  0  0  0  0  0  0
   -1.8851   -5.5019    0.3913 H   0  0  0  0  0  0  0  0  0  0  0  0
    0.3205   -6.2405   -2.1206 C   0  0  0  0  0  0  0  0  0  0  0  0
    1.4516   -4.7807   -3.2590 H   0  0  0  0  0  0  0  0  0  0  0  0
   -0.9558   -7.4351   -0.8397 H   0  0  0  0  0  0  0  0  0  0  0  0
   -3.5970   -3.4029    1.9420 C   0  0  0  0  0  0  0  0  0  0  0  0
   -4.1670   -3.4281    0.6654 C   0  0  0  0  0  0  0  0  0  0  0  0
   -4.3629   -3.2870    3.1101 C   0  0  0  0  0  0  0  0  0  0  0  0
   -5.5567   -3.3280    0.5676 C   0  0  0  0  0  0  0  0  0  0  0  0
   -3.5584   -3.5283   -0.2355 H   0  0  0  0  0  0  0  0  0  0  0  0
   -5.7485   -3.1952    2.9860 C   0  0  0  0  0  0  0  0  0  0  0  0
   -3.8750   -3.2842    4.0836 H   0  0  0  0  0  0  0  0  0  0  0  0
   -6.3565   -3.2104    1.7174 C   0  0  0  0  0  0  0  0  0  0  0  0
   -6.0220   -3.3468   -0.4185 H   0  0  0  0  0  0  0  0  0  0  0  0
   -6.3617   -3.1105    3.8835 H   0  0  0  0  0  0  0  0  0  0  0  0
   -0.0357    0.2407    2.5439 C   0  0  0  0  0  0  0  0  0  0  0  0
    0.7368    0.4348    3.3949 O   0  0  0  0  0  0  0  0  0  0  0  0
   -1.0656    0.0368    1.4038 Ir  0  0  0  0  0  0  0  0  0  0  0  0
   -1.1829   -2.8925   -0.1380 O   0  0  0  0  0  0  0  0  0  0  0  0
    0.2437   -3.2506    2.2420 O   0  0  0  0  0  0  0  0  0  0  0  0
   -2.2333   -3.6409    2.1669 O   0  0  0  0  0  0  0  0  0  0  0  0
   -0.5628    2.7780   -0.5228 O   0  0  0  0  0  0  0  0  0  0  0  0
   -3.0853    2.7121    0.5436 O   0  0  0  0  0  0  0  0  0  0  0  0
   -1.3331    4.0445    1.7560 O   0  0  0  0  0  0  0  0  0  0  0  0
    1.2619   -0.6686   -2.0189 O   0  0  0  0  0  0  0  0  0  0  0  0
    2.9865    0.7151   -0.7166 O   0  0  0  0  0  0  0  0  0  0  0  0
    1.6928   -1.2135    0.4706 O   0  0  0  0  0  0  0  0  0  0  0  0
   -7.8442   -3.1210    1.5915 C   0  0  0  0  0  0  0  0  0  0  0  0
   -8.2711   -4.1527    1.5534 H   0  0  0  0  0  0  0  0  0  0  0  0
   -8.2896   -2.5808    2.4591 H   0  0  0  0  0  0  0  0  0  0  0  0
   -8.1321   -2.5790    0.6603 H   0  0  0  0  0  0  0  0  0  0  0  0
    0.9006   -7.4082   -2.8549 C   0  0  0  0  0  0  0  0  0  0  0  0
    0.6645   -7.3228   -3.9427 H   0  0  0  0  0  0  0  0  0  0  0  0
    2.0087   -7.4206   -2.7246 H   0  0  0  0  0  0  0  0  0  0  0  0
    0.4898   -8.3731   -2.4802 H   0  0  0  0  0  0  0  0  0  0  0  0
    1.4835   -8.7913    2.1181 C   0  0  0  0  0  0  0  0  0  0  0  0
    1.7987   -9.1135    3.1399 H   0  0  0  0  0  0  0  0  0  0  0  0
    0.5911   -9.3866    1.8112 H   0  0  0  0  0  0  0  0  0  0  0  0
    2.3128   -9.0092    1.4074 H   0  0  0  0  0  0  0  0  0  0  0  0
    6.3133   -4.4877    0.6013 C   0  0  0  0  0  0  0  0  0  0  0  0
    7.2270   -3.8759    0.4034 H   0  0  0  0  0  0  0  0  0  0  0  0
    6.4066   -4.9556    1.6085 H   0  0  0  0  0  0  0  0  0  0  0  0
    6.2535   -5.2998   -0.1586 H   0  0  0  0  0  0  0  0  0  0  0  0
    1.8282    2.7412   -6.5215 C   0  0  0  0  0  0  0  0  0  0  0  0
    1.3566    2.2472   -7.4058 H   0  0  0  0  0  0  0  0  0  0  0  0
    1.3297    3.7230   -6.3492 H   0  0  0  0  0  0  0  0  0  0  0  0
    2.9030    2.9287   -6.7473 H   0  0  0  0  0  0  0  0  0  0  0  0
    4.1411    6.2183   -0.0420 C   0  0  0  0  0  0  0  0  0  0  0  0
    4.9267    6.5274   -0.7704 H   0  0  0  0  0  0  0  0  0  0  0  0
    3.2200    6.8223   -0.2193 H   0  0  0  0  0  0  0  0  0  0  0  0
    4.5128    6.4324    0.9865 H   0  0  0  0  0  0  0  0  0  0  0  0
```

```
    3.2474    6.5684    3.9132 C   0  0  0  0  0  0  0  0  0  0  0  0
    2.9813    6.8683    4.9577 H   0  0  0  0  0  0  0  0  0  0  0  0
    4.1394    5.8994    3.9535 H   0  0  0  0  0  0  0  0  0  0  0  0
    3.5195    7.4860    3.3432 H   0  0  0  0  0  0  0  0  0  0  0  0
   -5.8185    6.8850   -2.1559 C   0  0  0  0  0  0  0  0  0  0  0  0
   -5.8543    6.6691   -3.2490 H   0  0  0  0  0  0  0  0  0  0  0  0
   -6.8646    6.9299   -1.7642 H   0  0  0  0  0  0  0  0  0  0  0  0
   -5.3416    7.8814   -2.0118 H   0  0  0  0  0  0  0  0  0  0  0  0
   -0.2524    7.1604   -4.1008 C   0  0  0  0  0  0  0  0  0  0  0  0
   -0.4925    8.1141   -3.5717 H   0  0  0  0  0  0  0  0  0  0  0  0
    0.7873    7.2219   -4.5013 H   0  0  0  0  0  0  0  0  0  0  0  0
   -0.9524    7.0479   -4.9600 H   0  0  0  0  0  0  0  0  0  0  0  0
  1 97  1  0  0  0  0
  2104  1  0  0  0  0
  2105  1  0  0  0  0
  2106  1  0  0  0  0
  2 97  1  0  0  0  0
  3 98  1  0  0  0  0
  3 99  1  0  0  0  0
  3100  1  0  0  0  0
  3 97  1  0  0  0  0
  4101  1  0  0  0  0
  4102  1  0  0  0  0
  4103  1  0  0  0  0
  4 97  1  0  0  0  0
  5  6  2  0  0  0  0
  5  7  1  0  0  0  0
  5105  1  0  0  0  0
  6  8  1  0  0  0  0
  6  9  1  0  0  0  0
  7 10  2  0  0  0  0
  7 11  1  0  0  0  0
  8 12  2  0  0  0  0
  8 13  1  0  0  0  0
 10 12  1  0  0  0  0
 10 14  1  0  0  0  0
 12127  1  0  0  0  0
 15 16  2  0  0  0  0
 15 17  1  0  0  0  0
 15106  1  0  0  0  0
 16 18  1  0  0  0  0
 16 19  1  0  0  0  0
 17 20  2  0  0  0  0
 17 21  1  0  0  0  0
 18 22  2  0  0  0  0
 18 23  1  0  0  0  0
 20 22  1  0  0  0  0
 20 24  1  0  0  0  0
 22119  1  0  0  0  0
 25 26  2  0  0  0  0
 25 27  1  0  0  0  0
 25104  1  0  0  0  0
 26 28  1  0  0  0  0
 26 29  1  0  0  0  0
 27 30  2  0  0  0  0
 27 31  1  0  0  0  0
 28 32  2  0  0  0  0
 28 33  1  0  0  0  0
 30 32  1  0  0  0  0
 30 34  1  0  0  0  0
 32123  1  0  0  0  0
 35 36  2  0  0  0  0
 35 37  1  0  0  0  0
 35103  1  0  0  0  0
 36 38  1  0  0  0  0
```

```
 36 39  1  0  0  0  0
 37 40  2  0  0  0  0
 37 41  1  0  0  0  0
 38 42  2  0  0  0  0
 38 43  1  0  0  0  0
 40 42  1  0  0  0  0
 40 44  1  0  0  0  0
 42131  1  0  0  0  0
 45 46  2  0  0  0  0
 45 47  1  0  0  0  0
 45102  1  0  0  0  0
 46 48  1  0  0  0  0
 46 49  1  0  0  0  0
 47 50  2  0  0  0  0
 47 51  1  0  0  0  0
 48 52  2  0  0  0  0
 48 53  1  0  0  0  0
 50 52  1  0  0  0  0
 50 54  1  0  0  0  0
 52135  1  0  0  0  0
 55 56  2  0  0  0  0
 55 57  1  0  0  0  0
 55101  1  0  0  0  0
 56 58  1  0  0  0  0
 56 59  1  0  0  0  0
 57 60  2  0  0  0  0
 57 61  1  0  0  0  0
 58 62  2  0  0  0  0
 58 63  1  0  0  0  0
 60 62  1  0  0  0  0
 60 64  1  0  0  0  0
 62139  1  0  0  0  0
 65 66  2  0  0  0  0
 65 67  1  0  0  0  0
 65 99  1  0  0  0  0
 66 68  1  0  0  0  0
 66 69  1  0  0  0  0
 67 70  2  0  0  0  0
 67 71  1  0  0  0  0
 68 72  2  0  0  0  0
 68 73  1  0  0  0  0
 70 72  1  0  0  0  0
 70 74  1  0  0  0  0
 72115  1  0  0  0  0
 75 76  2  0  0  0  0
 75 77  1  0  0  0  0
 75 98  1  0  0  0  0
 76 78  1  0  0  0  0
 76 79  1  0  0  0  0
 77 80  2  0  0  0  0
 77 81  1  0  0  0  0
 78 82  2  0  0  0  0
 78 83  1  0  0  0  0
 80 82  1  0  0  0  0
 80 84  1  0  0  0  0
 82111  1  0  0  0  0
 85 86  2  0  0  0  0
 85 87  1  0  0  0  0
 85100  1  0  0  0  0
 86 88  1  0  0  0  0
 86 89  1  0  0  0  0
 87 90  2  0  0  0  0
 87 91  1  0  0  0  0
 88 92  2  0  0  0  0
 88 93  1  0  0  0  0
```

```
 90 92  1  0  0  0  0
 90 94  1  0  0  0  0
 92107  1  0  0  0  0
 95 96  2  0  0  0  0
 95 97  1  0  0  0  0
107108  1  0  0  0  0
107109  1  0  0  0  0
107110  1  0  0  0  0
111112  1  0  0  0  0
111113  1  0  0  0  0
111114  1  0  0  0  0
115116  1  0  0  0  0
115117  1  0  0  0  0
115118  1  0  0  0  0
119120  1  0  0  0  0
119121  1  0  0  0  0
119122  1  0  0  0  0
123124  1  0  0  0  0
123125  1  0  0  0  0
123126  1  0  0  0  0
127128  1  0  0  0  0
127129  1  0  0  0  0
127130  1  0  0  0  0
131132  1  0  0  0  0
131133  1  0  0  0  0
131134  1  0  0  0  0
135136  1  0  0  0  0
135137  1  0  0  0  0
135138  1  0  0  0  0
139140  1  0  0  0  0
139141  1  0  0  0  0
139142  1  0  0  0  0
M  END
$$$$
mol043
Optimized Catalyst 043
Optimized Catalyst 043
107116  0  0  0  0  0  0  0  0999 V2000
    3.4835    0.6065    0.2088 C   0  0  0  0  0  0  0  0  0  0  0  0
    3.3503   -0.8477   -0.1845 C   0  0  0  0  0  0  0  0  0  0  0  0
    1.7210   -1.6596    0.4112 P   0  0  0  0  0  0  0  0  0  0  0  0
   -1.6418    1.6287   -0.1003 P   0  0  0  0  0  0  0  0  0  0  0  0
   -3.0653    0.9111    0.9300 C   0  0  0  0  0  0  0  0  0  0  0  0
   -3.4043   -0.4721    0.4098 C   0  0  0  0  0  0  0  0  0  0  0  0
   -1.8038   -1.5162    0.2003 P   0  0  0  0  0  0  0  0  0  0  0  0
    1.8137    1.5428    0.1283 P   0  0  0  0  0  0  0  0  0  0  0  0
   -3.9475    1.5832    0.9161 H   0  0  0  0  0  0  0  0  0  0  0  0
   -3.9310   -0.4408   -0.5704 H   0  0  0  0  0  0  0  0  0  0  0  0
    3.3205   -0.9491   -1.3022 H   0  0  0  0  0  0  0  0  0  0  0  0
    4.2047    1.1218   -0.4675 H   0  0  0  0  0  0  0  0  0  0  0  0
   -2.6770    0.8409    1.9882 H   0  0  0  0  0  0  0  0  0  0  0  0
   -4.0542   -0.9919    1.1538 H   0  0  0  0  0  0  0  0  0  0  0  0
    4.2079   -1.4337    0.2147 H   0  0  0  0  0  0  0  0  0  0  0  0
    3.8562    0.6983    1.2579 H   0  0  0  0  0  0  0  0  0  0  0  0
    1.8413    2.5035   -1.4622 C   0  0  0  0  0  0  0  0  0  0  0  0
    1.2794    3.7819   -1.5024 C   0  0  0  0  0  0  0  0  0  0  0  0
    2.3210    1.8977   -2.6233 C   0  0  0  0  0  0  0  0  0  0  0  0
    1.2166    4.4727   -2.7165 C   0  0  0  0  0  0  0  0  0  0  0  0
    0.8775    4.2621   -0.6044 H   0  0  0  0  0  0  0  0  0  0  0  0
    2.2588    2.5968   -3.8389 C   0  0  0  0  0  0  0  0  0  0  0  0
    2.7298    0.8835   -2.6220 H   0  0  0  0  0  0  0  0  0  0  0  0
    1.7084    3.8793   -3.8851 C   0  0  0  0  0  0  0  0  0  0  0  0
    0.7779    5.4672   -2.7519 H   0  0  0  0  0  0  0  0  0  0  0  0
    2.6243    2.1248   -4.7489 H   0  0  0  0  0  0  0  0  0  0  0  0
    1.6562    4.4168   -4.8304 H   0  0  0  0  0  0  0  0  0  0  0  0
```

```
    2.2237    2.8436    1.4068 C   0  0  0  0  0  0  0  0  0  0  0  0
    1.8473    2.6564    2.7320 C   0  0  0  0  0  0  0  0  0  0  0  0
    2.9908    3.9546    1.0408 C   0  0  0  0  0  0  0  0  0  0  0  0
    2.1998    3.6085    3.6996 C   0  0  0  0  0  0  0  0  0  0  0  0
    1.2687    1.7736    3.0452 H   0  0  0  0  0  0  0  0  0  0  0  0
    3.3338    4.9085    2.0037 C   0  0  0  0  0  0  0  0  0  0  0  0
    3.3418    4.1037    0.0167 H   0  0  0  0  0  0  0  0  0  0  0  0
    2.9329    4.7384    3.3346 C   0  0  0  0  0  0  0  0  0  0  0  0
    1.8938    3.4604    4.7337 H   0  0  0  0  0  0  0  0  0  0  0  0
    3.9150    5.7831    1.7187 H   0  0  0  0  0  0  0  0  0  0  0  0
    3.1990    5.4844    4.0817 H   0  0  0  0  0  0  0  0  0  0  0  0
    2.0242   -2.2202    2.1569 C   0  0  0  0  0  0  0  0  0  0  0  0
    1.8228   -3.5479    2.5421 C   0  0  0  0  0  0  0  0  0  0  0  0
    2.3539   -1.2500    3.1033 C   0  0  0  0  0  0  0  0  0  0  0  0
    2.0206   -3.9196    3.8762 C   0  0  0  0  0  0  0  0  0  0  0  0
    1.4849   -4.3105    1.8359 H   0  0  0  0  0  0  0  0  0  0  0  0
    2.5421   -1.6246    4.4422 C   0  0  0  0  0  0  0  0  0  0  0  0
    2.4495   -0.1956    2.8378 H   0  0  0  0  0  0  0  0  0  0  0  0
    2.3830   -2.9574    4.8257 C   0  0  0  0  0  0  0  0  0  0  0  0
    1.8794   -4.9551    4.1770 H   0  0  0  0  0  0  0  0  0  0  0  0
    2.7896   -0.8671    5.1832 H   0  0  0  0  0  0  0  0  0  0  0  0
    2.5282   -3.2472    5.8653 H   0  0  0  0  0  0  0  0  0  0  0  0
    1.8478   -3.2212   -0.5954 C   0  0  0  0  0  0  0  0  0  0  0  0
    1.0255   -3.4021   -1.6993 C   0  0  0  0  0  0  0  0  0  0  0  0
    2.8535   -4.1502   -0.2981 C   0  0  0  0  0  0  0  0  0  0  0  0
    1.1690   -4.5532   -2.4922 C   0  0  0  0  0  0  0  0  0  0  0  0
    0.2710   -2.6547   -1.9891 H   0  0  0  0  0  0  0  0  0  0  0  0
    2.9923   -5.2949   -1.0849 C   0  0  0  0  0  0  0  0  0  0  0  0
    3.5442   -4.0051    0.5353 H   0  0  0  0  0  0  0  0  0  0  0  0
    2.1432   -5.4998   -2.1823 C   0  0  0  0  0  0  0  0  0  0  0  0
    0.5138   -4.6913   -3.3518 H   0  0  0  0  0  0  0  0  0  0  0  0
    3.7625   -6.0266   -0.8521 H   0  0  0  0  0  0  0  0  0  0  0  0
    2.2544   -6.3938   -2.7938 H   0  0  0  0  0  0  0  0  0  0  0  0
   -2.0930   -2.8142    1.4996 C   0  0  0  0  0  0  0  0  0  0  0  0
   -1.2599   -2.8964    2.6064 C   0  0  0  0  0  0  0  0  0  0  0  0
   -3.2107   -3.6522    1.3830 C   0  0  0  0  0  0  0  0  0  0  0  0
   -1.5176   -3.8583    3.5986 C   0  0  0  0  0  0  0  0  0  0  0  0
   -0.4083   -2.2135    2.7433 H   0  0  0  0  0  0  0  0  0  0  0  0
   -3.4628   -4.6078    2.3682 C   0  0  0  0  0  0  0  0  0  0  0  0
   -3.8981   -3.5810    0.5371 H   0  0  0  0  0  0  0  0  0  0  0  0
   -2.6106   -4.7133    3.4778 C   0  0  0  0  0  0  0  0  0  0  0  0
   -0.8574   -3.9199    4.4631 H   0  0  0  0  0  0  0  0  0  0  0  0
   -4.3219   -5.2690    2.2805 H   0  0  0  0  0  0  0  0  0  0  0  0
   -2.8113   -5.4597    4.2448 H   0  0  0  0  0  0  0  0  0  0  0  0
   -2.1086   -2.4155   -1.3974 C   0  0  0  0  0  0  0  0  0  0  0  0
   -2.3284   -1.6441   -2.5396 C   0  0  0  0  0  0  0  0  0  0  0  0
   -2.0492   -3.8086   -1.4787 C   0  0  0  0  0  0  0  0  0  0  0  0
   -2.5404   -2.2811   -3.7714 C   0  0  0  0  0  0  0  0  0  0  0  0
   -2.3227   -0.5478   -2.5150 H   0  0  0  0  0  0  0  0  0  0  0  0
   -2.2733   -4.4395   -2.7069 C   0  0  0  0  0  0  0  0  0  0  0  0
   -1.8066   -4.4278   -0.6116 H   0  0  0  0  0  0  0  0  0  0  0  0
   -2.5214   -3.6745   -3.8528 C   0  0  0  0  0  0  0  0  0  0  0  0
   -2.6991   -1.6786   -4.6642 H   0  0  0  0  0  0  0  0  0  0  0  0
   -2.2419   -5.5244   -2.7726 H   0  0  0  0  0  0  0  0  0  0  0  0
   -2.6899   -4.1672   -4.8089 H   0  0  0  0  0  0  0  0  0  0  0  0
   -1.4365    3.3609    0.5250 C   0  0  0  0  0  0  0  0  0  0  0  0
   -1.6550    4.4586   -0.3096 C   0  0  0  0  0  0  0  0  0  0  0  0
   -1.0342    3.5349    1.8475 C   0  0  0  0  0  0  0  0  0  0  0  0
   -1.4494    5.7508    0.1866 C   0  0  0  0  0  0  0  0  0  0  0  0
   -1.9755    4.3395   -1.3481 H   0  0  0  0  0  0  0  0  0  0  0  0
   -0.8253    4.8305    2.3389 C   0  0  0  0  0  0  0  0  0  0  0  0
   -0.8647    2.6760    2.5116 H   0  0  0  0  0  0  0  0  0  0  0  0
   -1.0299    5.9353    1.5090 C   0  0  0  0  0  0  0  0  0  0  0  0
   -1.6135    6.6113   -0.4582 H   0  0  0  0  0  0  0  0  0  0  0  0
   -0.4900    4.9695    3.3657 H   0  0  0  0  0  0  0  0  0  0  0  0
```

```
   -0.8631    6.9416    1.8907 H   0  0  0  0  0  0  0  0  0  0  0  0
   -2.3324    1.8868   -1.8077 C   0  0  0  0  0  0  0  0  0  0  0  0
   -3.7063    1.8818   -2.0592 C   0  0  0  0  0  0  0  0  0  0  0  0
   -1.4206    2.0574   -2.8484 C   0  0  0  0  0  0  0  0  0  0  0  0
   -4.1686    2.0173   -3.3720 C   0  0  0  0  0  0  0  0  0  0  0  0
   -4.4416    1.7850   -1.2610 H   0  0  0  0  0  0  0  0  0  0  0  0
   -1.8878    2.1924   -4.1647 C   0  0  0  0  0  0  0  0  0  0  0  0
   -0.3383    2.0927   -2.6674 H   0  0  0  0  0  0  0  0  0  0  0  0
   -3.2577    2.1620   -4.4263 C   0  0  0  0  0  0  0  0  0  0  0  0
   -5.2368    2.0097   -3.5755 H   0  0  0  0  0  0  0  0  0  0  0  0
   -1.1699    2.3049   -4.9759 H   0  0  0  0  0  0  0  0  0  0  0  0
   -3.6217    2.2545   -5.4484 H   0  0  0  0  0  0  0  0  0  0  0  0
    0.0354   -0.0174    0.1829 Os  0  0  0  0  0  0  0  0  0  0  0  0
   -0.3017    0.1899    2.9680 Cl  0  0  0  0  0  0  0  0  0  0  0  0
    0.3444   -0.3139   -2.5925 Cl  0  0  0  0  0  0  0  0  0  0  0  0
  1  2  1  0  0  0  0
  1  8  1  0  0  0  0
  1 12  1  0  0  0  0
  1 16  1  0  0  0  0
  2  3  1  0  0  0  0
  2 11  1  0  0  0  0
  2 15  1  0  0  0  0
  3 39  1  0  0  0  0
  3 50  1  0  0  0  0
  3105  1  0  0  0  0
  4  5  1  0  0  0  0
  4 83  1  0  0  0  0
  4 94  1  0  0  0  0
  4105  1  0  0  0  0
  5  6  1  0  0  0  0
  5  9  1  0  0  0  0
  5 13  1  0  0  0  0
  6  7  1  0  0  0  0
  6 10  1  0  0  0  0
  6 14  1  0  0  0  0
  7 61  1  0  0  0  0
  7 72  1  0  0  0  0
  7105  1  0  0  0  0
  8 17  1  0  0  0  0
  8 28  1  0  0  0  0
  8105  1  0  0  0  0
 17 18  2  0  0  0  0
 17 19  1  0  0  0  0
 18 20  1  0  0  0  0
 18 21  1  0  0  0  0
 19 22  2  0  0  0  0
 19 23  1  0  0  0  0
 20 24  2  0  0  0  0
 20 25  1  0  0  0  0
 22 24  1  0  0  0  0
 22 26  1  0  0  0  0
 24 27  1  0  0  0  0
 28 29  2  0  0  0  0
 28 30  1  0  0  0  0
 29 31  1  0  0  0  0
 29 32  1  0  0  0  0
 30 33  2  0  0  0  0
 30 34  1  0  0  0  0
 31 35  2  0  0  0  0
 31 36  1  0  0  0  0
 33 35  1  0  0  0  0
 33 37  1  0  0  0  0
 35 38  1  0  0  0  0
 39 40  2  0  0  0  0
 39 41  1  0  0  0  0
```

```
 40 42  1  0  0  0  0
 40 43  1  0  0  0  0
 41 44  2  0  0  0  0
 41 45  1  0  0  0  0
 42 46  2  0  0  0  0
 42 47  1  0  0  0  0
 44 46  1  0  0  0  0
 44 48  1  0  0  0  0
 46 49  1  0  0  0  0
 50 51  2  0  0  0  0
 50 52  1  0  0  0  0
 51 53  1  0  0  0  0
 51 54  1  0  0  0  0
 52 55  2  0  0  0  0
 52 56  1  0  0  0  0
 53 57  2  0  0  0  0
 53 58  1  0  0  0  0
 55 57  1  0  0  0  0
 55 59  1  0  0  0  0
 57 60  1  0  0  0  0
 61 62  2  0  0  0  0
 61 63  1  0  0  0  0
 62 64  1  0  0  0  0
 62 65  1  0  0  0  0
 63 66  2  0  0  0  0
 63 67  1  0  0  0  0
 64 68  2  0  0  0  0
 64 69  1  0  0  0  0
 66 68  1  0  0  0  0
 66 70  1  0  0  0  0
 68 71  1  0  0  0  0
 72 73  2  0  0  0  0
 72 74  1  0  0  0  0
 73 75  1  0  0  0  0
 73 76  1  0  0  0  0
 74 77  2  0  0  0  0
 74 78  1  0  0  0  0
 75 79  2  0  0  0  0
 75 80  1  0  0  0  0
 77 79  1  0  0  0  0
 77 81  1  0  0  0  0
 79 82  1  0  0  0  0
 83 84  2  0  0  0  0
 83 85  1  0  0  0  0
 84 86  1  0  0  0  0
 84 87  1  0  0  0  0
 85 88  2  0  0  0  0
 85 89  1  0  0  0  0
 86 90  2  0  0  0  0
 86 91  1  0  0  0  0
 88 90  1  0  0  0  0
 88 92  1  0  0  0  0
 90 93  1  0  0  0  0
 94 95  2  0  0  0  0
 94 96  1  0  0  0  0
 95 97  1  0  0  0  0
 95 98  1  0  0  0  0
 96 99  2  0  0  0  0
 96100  1  0  0  0  0
 97101  2  0  0  0  0
 97102  1  0  0  0  0
 99101  1  0  0  0  0
 99103  1  0  0  0  0
101104  1  0  0  0  0
105106  1  0  0  0  0
```

105107 1 0 0 0 0
M END
$$$$
mol044
Optimized Catalyst 044
Optimized Catalyst 044
107116 0 0 0 0 0 0 0 0999 V2000
    3.4846   0.6085   0.2087 C   0 0 0 0 0 0 0 0 0 0 0 0
    3.3545   -0.8463   -0.1828 C   0 0 0 0 0 0 0 0 0 0 0 0
    1.7245   -1.6580   0.4073 P   0 0 0 0 0 0 0 0 0 0 0 0
   -1.6436   1.6279   -0.0977 P   0 0 0 0 0 0 0 0 0 0 0 0
   -3.0656   0.9107   0.9325 C   0 0 0 0 0 0 0 0 0 0 0 0
   -3.4059   -0.4705   0.4080 C   0 0 0 0 0 0 0 0 0 0 0 0
   -1.8060   -1.5143   0.2006 P   0 0 0 0 0 0 0 0 0 0 0 0
    1.8150   1.5418   0.1265 P   0 0 0 0 0 0 0 0 0 0 0 0
   -3.9486   1.5830   0.9244 H   0 0 0 0 0 0 0 0 0 0 0 0
   -3.9347   -0.4366   -0.5711 H   0 0 0 0 0 0 0 0 0 0 0 0
    3.3329   -0.9510   -1.3008 H   0 0 0 0 0 0 0 0 0 0 0 0
    4.2071   1.1241   -0.4671 H   0 0 0 0 0 0 0 0 0 0 0 0
   -2.6777   0.8368   1.9911 H   0 0 0 0 0 0 0 0 0 0 0 0
   -4.0590   -0.9904   1.1501 H   0 0 0 0 0 0 0 0 0 0 0 0
    4.2123   -1.4296   0.2220 H   0 0 0 0 0 0 0 0 0 0 0 0
    3.8592   0.7028   1.2576 H   0 0 0 0 0 0 0 0 0 0 0 0
    1.8401   2.5015   -1.4621 C   0 0 0 0 0 0 0 0 0 0 0 0
    1.2804   3.7813   -1.4982 C   0 0 0 0 0 0 0 0 0 0 0 0
    2.3119   1.8845   -2.6234 C   0 0 0 0 0 0 0 0 0 0 0 0
    1.2107   4.4828   -2.7053 C   0 0 0 0 0 0 0 0 0 0 0 0
    0.8847   4.2628   -0.5964 H   0 0 0 0 0 0 0 0 0 0 0 0
    2.2576   2.5721   -3.8445 C   0 0 0 0 0 0 0 0 0 0 0 0
    2.7224   0.8692   -2.6167 H   0 0 0 0 0 0 0 0 0 0 0 0
    1.7068   3.8541   -3.8522 C   0 0 0 0 0 0 0 0 0 0 0 0
    0.7809   5.4812   -2.7475 H   0 0 0 0 0 0 0 0 0 0 0 0
    2.6164   2.1032   -4.7595 H   0 0 0 0 0 0 0 0 0 0 0 0
    2.2194   2.8433   1.4011 C   0 0 0 0 0 0 0 0 0 0 0 0
    1.8349   2.6538   2.7259 C   0 0 0 0 0 0 0 0 0 0 0 0
    2.9918   3.9507   1.0323 C   0 0 0 0 0 0 0 0 0 0 0 0
    2.1807   3.5959   3.7034 C   0 0 0 0 0 0 0 0 0 0 0 0
    1.2537   1.7689   3.0356 H   0 0 0 0 0 0 0 0 0 0 0 0
    3.3404   4.9122   1.9841 C   0 0 0 0 0 0 0 0 0 0 0 0
    3.3491   4.0933   0.0076 H   0 0 0 0 0 0 0 0 0 0 0 0
    2.9170   4.7105   3.3025 C   0 0 0 0 0 0 0 0 0 0 0 0
    1.8781   3.4547   4.7398 H   0 0 0 0 0 0 0 0 0 0 0 0
    3.9240   5.7886   1.7077 H   0 0 0 0 0 0 0 0 0 0 0 0
    2.0203   -2.2213   2.1516 C   0 0 0 0 0 0 0 0 0 0 0 0
    1.8137   -3.5492   2.5344 C   0 0 0 0 0 0 0 0 0 0 0 0
    2.3452   -1.2445   3.0968 C   0 0 0 0 0 0 0 0 0 0 0 0
    2.0090   -3.9348   3.8644 C   0 0 0 0 0 0 0 0 0 0 0 0
    1.4748   -4.3114   1.8257 H   0 0 0 0 0 0 0 0 0 0 0 0
    2.5408   -1.6046   4.4370 C   0 0 0 0 0 0 0 0 0 0 0 0
    2.4453   -0.1908   2.8254 H   0 0 0 0 0 0 0 0 0 0 0 0
    2.3732   -2.9457   4.7835 C   0 0 0 0 0 0 0 0 0 0 0 0
    1.8683   -4.9687   4.1734 H   0 0 0 0 0 0 0 0 0 0 0 0
    2.7873   -0.8520   5.1839 H   0 0 0 0 0 0 0 0 0 0 0 0
    1.8522   -3.2186   -0.5955 C   0 0 0 0 0 0 0 0 0 0 0 0
    1.0278   -3.3928   -1.7027 C   0 0 0 0 0 0 0 0 0 0 0 0
    2.8586   -4.1455   -0.2944 C   0 0 0 0 0 0 0 0 0 0 0 0
    1.1577   -4.5389   -2.5013 C   0 0 0 0 0 0 0 0 0 0 0 0
    0.2765   -2.6391   -1.9890 H   0 0 0 0 0 0 0 0 0 0 0 0
    3.0060   -5.2962   -1.0704 C   0 0 0 0 0 0 0 0 0 0 0 0
    3.5510   -3.9972   0.5397 H   0 0 0 0 0 0 0 0 0 0 0 0
    2.1393   -5.4655   -2.1582 C   0 0 0 0 0 0 0 0 0 0 0 0
    0.5104   -4.6823   -3.3666 H   0 0 0 0 0 0 0 0 0 0 0 0
    3.7734   -6.0332   -0.8429 H   0 0 0 0 0 0 0 0 0 0 0 0
   -2.0946   -2.8120   1.4968 C   0 0 0 0 0 0 0 0 0 0 0 0
   -1.2580   -2.8869   2.6053 C   0 0 0 0 0 0 0 0 0 0 0 0

```
  -3.2134   -3.6479    1.3784 C   0  0  0  0  0  0  0  0  0  0  0  0
  -1.5010   -3.8435    3.6033 C   0  0  0  0  0  0  0  0  0  0  0  0
  -0.4074   -2.1997    2.7361 H   0  0  0  0  0  0  0  0  0  0  0  0
  -3.4753   -4.6087    2.3555 C   0  0  0  0  0  0  0  0  0  0  0  0
  -3.9023   -3.5743    0.5313 H   0  0  0  0  0  0  0  0  0  0  0  0
  -2.6012   -4.6828    3.4487 C   0  0  0  0  0  0  0  0  0  0  0  0
  -0.8481   -3.9075    4.4738 H   0  0  0  0  0  0  0  0  0  0  0  0
  -4.3327   -5.2736    2.2751 H   0  0  0  0  0  0  0  0  0  0  0  0
  -2.1052   -2.4147   -1.3955 C   0  0  0  0  0  0  0  0  0  0  0  0
  -2.3176   -1.6368   -2.5384 C   0  0  0  0  0  0  0  0  0  0  0  0
  -2.0443   -3.8080   -1.4739 C   0  0  0  0  0  0  0  0  0  0  0  0
  -2.5360   -2.2592   -3.7746 C   0  0  0  0  0  0  0  0  0  0  0  0
  -2.3123   -0.5398   -2.5079 H   0  0  0  0  0  0  0  0  0  0  0  0
  -2.2671   -4.4514   -2.6955 C   0  0  0  0  0  0  0  0  0  0  0  0
  -1.8031   -4.4274   -0.6046 H   0  0  0  0  0  0  0  0  0  0  0  0
  -2.5113   -3.6534   -3.8178 C   0  0  0  0  0  0  0  0  0  0  0  0
  -2.6942   -1.6632   -4.6726 H   0  0  0  0  0  0  0  0  0  0  0  0
  -2.2385   -5.5365   -2.7691 H   0  0  0  0  0  0  0  0  0  0  0  0
  -1.4364    3.3587    0.5245 C   0  0  0  0  0  0  0  0  0  0  0  0
  -1.6547    4.4549   -0.3130 C   0  0  0  0  0  0  0  0  0  0  0  0
  -1.0354    3.5264    1.8507 C   0  0  0  0  0  0  0  0  0  0  0  0
  -1.4508    5.7515    0.1705 C   0  0  0  0  0  0  0  0  0  0  0  0
  -1.9778    4.3340   -1.3523 H   0  0  0  0  0  0  0  0  0  0  0  0
  -0.8223    4.8142    2.3557 C   0  0  0  0  0  0  0  0  0  0  0  0
  -0.8734    2.6625    2.5130 H   0  0  0  0  0  0  0  0  0  0  0  0
  -1.0334    5.8954    1.4976 C   0  0  0  0  0  0  0  0  0  0  0  0
  -1.6151    6.6181   -0.4670 H   0  0  0  0  0  0  0  0  0  0  0  0
  -0.4923    4.9609    3.3838 H   0  0  0  0  0  0  0  0  0  0  0  0
  -2.3318    1.8854   -1.8046 C   0  0  0  0  0  0  0  0  0  0  0  0
  -3.7059    1.8811   -2.0531 C   0  0  0  0  0  0  0  0  0  0  0  0
  -1.4138    2.0513   -2.8453 C   0  0  0  0  0  0  0  0  0  0  0  0
  -4.1815    2.0120   -3.3613 C   0  0  0  0  0  0  0  0  0  0  0  0
  -4.4411    1.7902   -1.2516 H   0  0  0  0  0  0  0  0  0  0  0  0
  -1.8660    2.1891   -4.1645 C   0  0  0  0  0  0  0  0  0  0  0  0
  -0.3325    2.0912   -2.6578 H   0  0  0  0  0  0  0  0  0  0  0  0
  -3.2417    2.1530   -4.3886 C   0  0  0  0  0  0  0  0  0  0  0  0
  -5.2486    2.0056   -3.5726 H   0  0  0  0  0  0  0  0  0  0  0  0
  -1.1543    2.3023   -4.9821 H   0  0  0  0  0  0  0  0  0  0  0  0
   0.0356   -0.0175    0.1825 Os  0  0  0  0  0  0  0  0  0  0  0  0
  -0.2909    0.1843    2.9629 Cl  0  0  0  0  0  0  0  0  0  0  0  0
   0.3365   -0.3089   -2.5889 Cl  0  0  0  0  0  0  0  0  0  0  0  0
   2.2699   -6.5654   -2.9015 F   0  0  0  0  0  0  0  0  0  0  0  0
   2.5632   -3.3039    6.0545 F   0  0  0  0  0  0  0  0  0  0  0  0
  -2.8418   -5.6028    4.3843 F   0  0  0  0  0  0  0  0  0  0  0  0
  -2.7250   -4.2591   -4.9873 F   0  0  0  0  0  0  0  0  0  0  0  0
   3.2438    5.6233    4.2185 F   0  0  0  0  0  0  0  0  0  0  0  0
  -0.8270    7.1275    1.9641 F   0  0  0  0  0  0  0  0  0  0  0  0
   1.6456    4.5135   -5.0104 F   0  0  0  0  0  0  0  0  0  0  0  0
  -3.6893    2.2703   -5.6396 F   0  0  0  0  0  0  0  0  0  0  0  0
 1  2  1  0  0  0  0
 1  8  1  0  0  0  0
 1 12  1  0  0  0  0
 1 16  1  0  0  0  0
 2  3  1  0  0  0  0
 2 11  1  0  0  0  0
 2 15  1  0  0  0  0
 3 37  1  0  0  0  0
 3 47  1  0  0  0  0
 3 97  1  0  0  0  0
 4  5  1  0  0  0  0
 4 77  1  0  0  0  0
 4 87  1  0  0  0  0
 4 97  1  0  0  0  0
 5  6  1  0  0  0  0
 5  9  1  0  0  0  0
```

```
  5 13  1  0  0  0  0
  6  7  1  0  0  0  0
  6 10  1  0  0  0  0
  6 14  1  0  0  0  0
  7 57  1  0  0  0  0
  7 67  1  0  0  0  0
  7 97  1  0  0  0  0
  8 17  1  0  0  0  0
  8 27  1  0  0  0  0
  8 97  1  0  0  0  0
 17 18  2  0  0  0  0
 17 19  1  0  0  0  0
 18 20  1  0  0  0  0
 18 21  1  0  0  0  0
 19 22  2  0  0  0  0
 19 23  1  0  0  0  0
 20 24  2  0  0  0  0
 20 25  1  0  0  0  0
 22 24  1  0  0  0  0
 22 26  1  0  0  0  0
 24106  1  0  0  0  0
 27 28  2  0  0  0  0
 27 29  1  0  0  0  0
 28 30  1  0  0  0  0
 28 31  1  0  0  0  0
 29 32  2  0  0  0  0
 29 33  1  0  0  0  0
 30 34  2  0  0  0  0
 30 35  1  0  0  0  0
 32 34  1  0  0  0  0
 32 36  1  0  0  0  0
 34104  1  0  0  0  0
 37 38  2  0  0  0  0
 37 39  1  0  0  0  0
 38 40  1  0  0  0  0
 38 41  1  0  0  0  0
 39 42  2  0  0  0  0
 39 43  1  0  0  0  0
 40 44  2  0  0  0  0
 40 45  1  0  0  0  0
 42 44  1  0  0  0  0
 42 46  1  0  0  0  0
 44101  1  0  0  0  0
 47 48  2  0  0  0  0
 47 49  1  0  0  0  0
 48 50  1  0  0  0  0
 48 51  1  0  0  0  0
 49 52  2  0  0  0  0
 49 53  1  0  0  0  0
 50 54  2  0  0  0  0
 50 55  1  0  0  0  0
 52 54  1  0  0  0  0
 52 56  1  0  0  0  0
 54100  1  0  0  0  0
 57 58  2  0  0  0  0
 57 59  1  0  0  0  0
 58 60  1  0  0  0  0
 58 61  1  0  0  0  0
 59 62  2  0  0  0  0
 59 63  1  0  0  0  0
 60 64  2  0  0  0  0
 60 65  1  0  0  0  0
 62 64  1  0  0  0  0
 62 66  1  0  0  0  0
 64102  1  0  0  0  0
```

```
 67 68  2  0  0  0  0
 67 69  1  0  0  0  0
 68 70  1  0  0  0  0
 68 71  1  0  0  0  0
 69 72  2  0  0  0  0
 69 73  1  0  0  0  0
 70 74  2  0  0  0  0
 70 75  1  0  0  0  0
 72 74  1  0  0  0  0
 72 76  1  0  0  0  0
 74103  1  0  0  0  0
 77 78  2  0  0  0  0
 77 79  1  0  0  0  0
 78 80  1  0  0  0  0
 78 81  1  0  0  0  0
 79 82  2  0  0  0  0
 79 83  1  0  0  0  0
 80 84  2  0  0  0  0
 80 85  1  0  0  0  0
 82 84  1  0  0  0  0
 82 86  1  0  0  0  0
 84105  1  0  0  0  0
 87 88  2  0  0  0  0
 87 89  1  0  0  0  0
 88 90  1  0  0  0  0
 88 91  1  0  0  0  0
 89 92  2  0  0  0  0
 89 93  1  0  0  0  0
 90 94  2  0  0  0  0
 90 95  1  0  0  0  0
 92 94  1  0  0  0  0
 92 96  1  0  0  0  0
 94107  1  0  0  0  0
 97 98  1  0  0  0  0
 97 99  1  0  0  0  0
M  END
$$$$
mol045
Optimized Catalyst 045
Optimized Catalyst 045
131140  0  0  0  0  0  0  0  0999 V2000
    3.4827    0.6077    0.2107 C   0  0  0  0  0  0  0  0  0  0  0  0
    3.3510   -0.8460   -0.1835 C   0  0  0  0  0  0  0  0  0  0  0  0
    1.7208   -1.6598    0.4106 P   0  0  0  0  0  0  0  0  0  0  0  0
   -1.6404    1.6298   -0.1016 P   0  0  0  0  0  0  0  0  0  0  0  0
   -3.0638    0.9118    0.9299 C   0  0  0  0  0  0  0  0  0  0  0  0
   -3.4047   -0.4695    0.4067 C   0  0  0  0  0  0  0  0  0  0  0  0
   -1.8040   -1.5159    0.1996 P   0  0  0  0  0  0  0  0  0  0  0  0
    1.8122    1.5440    0.1281 P   0  0  0  0  0  0  0  0  0  0  0  0
   -3.9445    1.5852    0.9198 H   0  0  0  0  0  0  0  0  0  0  0  0
   -3.9282   -0.4348   -0.5745 H   0  0  0  0  0  0  0  0  0  0  0  0
    3.3214   -0.9476   -1.3008 H   0  0  0  0  0  0  0  0  0  0  0  0
    4.2035    1.1250   -0.4636 H   0  0  0  0  0  0  0  0  0  0  0  0
   -2.6723    0.8387    1.9863 H   0  0  0  0  0  0  0  0  0  0  0  0
   -4.0567   -0.9906    1.1471 H   0  0  0  0  0  0  0  0  0  0  0  0
    4.2072   -1.4320    0.2175 H   0  0  0  0  0  0  0  0  0  0  0  0
    3.8517    0.6998    1.2608 H   0  0  0  0  0  0  0  0  0  0  0  0
    1.8431    2.5051   -1.4582 C   0  0  0  0  0  0  0  0  0  0  0  0
    1.2829    3.7832   -1.5016 C   0  0  0  0  0  0  0  0  0  0  0  0
    2.3151    1.8980   -2.6226 C   0  0  0  0  0  0  0  0  0  0  0  0
    1.2138    4.4736   -2.7139 C   0  0  0  0  0  0  0  0  0  0  0  0
    0.8846    4.2668   -0.6027 H   0  0  0  0  0  0  0  0  0  0  0  0
    2.2510    2.5927   -3.8378 C   0  0  0  0  0  0  0  0  0  0  0  0
    2.7269    0.8846   -2.6211 H   0  0  0  0  0  0  0  0  0  0  0  0
    1.6953    3.8787   -3.8925 C   0  0  0  0  0  0  0  0  0  0  0  0
```

```
    0.7765    5.4677   -2.7417 H   0  0  0  0  0  0  0  0  0  0  0  0
    2.6141    2.1140   -4.7439 H   0  0  0  0  0  0  0  0  0  0  0  0
    2.2214    2.8426    1.4040 C   0  0  0  0  0  0  0  0  0  0  0  0
    1.8431    2.6604    2.7299 C   0  0  0  0  0  0  0  0  0  0  0  0
    2.9902    3.9528    1.0417 C   0  0  0  0  0  0  0  0  0  0  0  0
    2.1895    3.6125    3.6963 C   0  0  0  0  0  0  0  0  0  0  0  0
    1.2671    1.7761    3.0449 H   0  0  0  0  0  0  0  0  0  0  0  0
    3.3304    4.9101    1.9998 C   0  0  0  0  0  0  0  0  0  0  0  0
    3.3473    4.0988    0.0186 H   0  0  0  0  0  0  0  0  0  0  0  0
    2.9216    4.7511    3.3355 C   0  0  0  0  0  0  0  0  0  0  0  0
    1.8805    3.4599    4.7277 H   0  0  0  0  0  0  0  0  0  0  0  0
    3.9158    5.7786    1.7102 H   0  0  0  0  0  0  0  0  0  0  0  0
    2.0229   -2.2238    2.1519 C   0  0  0  0  0  0  0  0  0  0  0  0
    1.8185   -3.5492    2.5374 C   0  0  0  0  0  0  0  0  0  0  0  0
    2.3491   -1.2567    3.1049 C   0  0  0  0  0  0  0  0  0  0  0  0
    2.0157   -3.9289    3.8688 C   0  0  0  0  0  0  0  0  0  0  0  0
    1.4780   -4.3109    1.8303 H   0  0  0  0  0  0  0  0  0  0  0  0
    2.5419   -1.6331    4.4388 C   0  0  0  0  0  0  0  0  0  0  0  0
    2.4449   -0.2011    2.8423 H   0  0  0  0  0  0  0  0  0  0  0  0
    2.3813   -2.9715    4.8280 C   0  0  0  0  0  0  0  0  0  0  0  0
    1.8677   -4.9656    4.1573 H   0  0  0  0  0  0  0  0  0  0  0  0
    2.7880   -0.8754    5.1787 H   0  0  0  0  0  0  0  0  0  0  0  0
    1.8502   -3.2196   -0.5927 C   0  0  0  0  0  0  0  0  0  0  0  0
    1.0294   -3.4046   -1.6993 C   0  0  0  0  0  0  0  0  0  0  0  0
    2.8551   -4.1477   -0.2979 C   0  0  0  0  0  0  0  0  0  0  0  0
    1.1745   -4.5516   -2.4914 C   0  0  0  0  0  0  0  0  0  0  0  0
    0.2721   -2.6592   -1.9879 H   0  0  0  0  0  0  0  0  0  0  0  0
    3.0006   -5.2922   -1.0828 C   0  0  0  0  0  0  0  0  0  0  0  0
    3.5453   -4.0040    0.5372 H   0  0  0  0  0  0  0  0  0  0  0  0
    2.1549   -5.5034   -2.1872 C   0  0  0  0  0  0  0  0  0  0  0  0
    0.5166   -4.6883   -3.3482 H   0  0  0  0  0  0  0  0  0  0  0  0
    3.7729   -6.0172   -0.8417 H   0  0  0  0  0  0  0  0  0  0  0  0
   -2.0943   -2.8126    1.4956 C   0  0  0  0  0  0  0  0  0  0  0  0
   -1.2627   -2.8985    2.6056 C   0  0  0  0  0  0  0  0  0  0  0  0
   -3.2109   -3.6494    1.3821 C   0  0  0  0  0  0  0  0  0  0  0  0
   -1.5208   -3.8565    3.5963 C   0  0  0  0  0  0  0  0  0  0  0  0
   -0.4078   -2.2190    2.7414 H   0  0  0  0  0  0  0  0  0  0  0  0
   -3.4696   -4.6041    2.3656 C   0  0  0  0  0  0  0  0  0  0  0  0
   -3.8979   -3.5802    0.5347 H   0  0  0  0  0  0  0  0  0  0  0  0
   -2.6210   -4.7144    3.4831 C   0  0  0  0  0  0  0  0  0  0  0  0
   -0.8576   -3.9174    4.4574 H   0  0  0  0  0  0  0  0  0  0  0  0
   -4.3305   -5.2594    2.2687 H   0  0  0  0  0  0  0  0  0  0  0  0
   -2.1080   -2.4159   -1.3942 C   0  0  0  0  0  0  0  0  0  0  0  0
   -2.3231   -1.6489   -2.5425 C   0  0  0  0  0  0  0  0  0  0  0  0
   -2.0477   -3.8071   -1.4757 C   0  0  0  0  0  0  0  0  0  0  0  0
   -2.5397   -2.2866   -3.7689 C   0  0  0  0  0  0  0  0  0  0  0  0
   -2.3153   -0.5519   -2.5214 H   0  0  0  0  0  0  0  0  0  0  0  0
   -2.2712   -4.4453   -2.6998 C   0  0  0  0  0  0  0  0  0  0  0  0
   -1.8043   -4.4263   -0.6078 H   0  0  0  0  0  0  0  0  0  0  0  0
   -2.5198   -3.6868   -3.8546 C   0  0  0  0  0  0  0  0  0  0  0  0
   -2.6980   -1.6841   -4.6606 H   0  0  0  0  0  0  0  0  0  0  0  0
   -2.2356   -5.5295   -2.7524 H   0  0  0  0  0  0  0  0  0  0  0  0
   -1.4347    3.3579    0.5258 C   0  0  0  0  0  0  0  0  0  0  0  0
   -1.6507    4.4601   -0.3030 C   0  0  0  0  0  0  0  0  0  0  0  0
   -1.0319    3.5313    1.8484 C   0  0  0  0  0  0  0  0  0  0  0  0
   -1.4480    5.7497    0.1944 C   0  0  0  0  0  0  0  0  0  0  0  0
   -1.9684    4.3443   -1.3434 H   0  0  0  0  0  0  0  0  0  0  0  0
   -0.8247    4.8224    2.3468 C   0  0  0  0  0  0  0  0  0  0  0  0
   -0.8615    2.6704    2.5105 H   0  0  0  0  0  0  0  0  0  0  0  0
   -1.0284    5.9383    1.5233 C   0  0  0  0  0  0  0  0  0  0  0  0
   -1.6122    6.6090   -0.4500 H   0  0  0  0  0  0  0  0  0  0  0  0
   -0.4865    4.9489    3.3731 H   0  0  0  0  0  0  0  0  0  0  0  0
   -2.3306    1.8889   -1.8057 C   0  0  0  0  0  0  0  0  0  0  0  0
   -3.7023    1.8839   -2.0612 C   0  0  0  0  0  0  0  0  0  0  0  0
   -1.4204    2.0576   -2.8501 C   0  0  0  0  0  0  0  0  0  0  0  0
```

```
   -4.1674    2.0127   -3.3723 C   0  0  0  0  0  0  0  0  0  0  0  0
   -4.4395    1.7916   -1.2633 H   0  0  0  0  0  0  0  0  0  0  0  0
   -1.8847    2.1925   -4.1644 C   0  0  0  0  0  0  0  0  0  0  0  0
   -0.3379    2.0975   -2.6690 H   0  0  0  0  0  0  0  0  0  0  0  0
   -3.2585    2.1531   -4.4358 C   0  0  0  0  0  0  0  0  0  0  0  0
   -5.2356    2.0031   -3.5688 H   0  0  0  0  0  0  0  0  0  0  0  0
   -1.1625    2.3084   -4.9699 H   0  0  0  0  0  0  0  0  0  0  0  0
    0.0353   -0.0174    0.1833 Os  0  0  0  0  0  0  0  0  0  0  0  0
   -0.3003    0.1854    2.9750 Cl  0  0  0  0  0  0  0  0  0  0  0  0
    0.3434   -0.3128   -2.5969 Cl  0  0  0  0  0  0  0  0  0  0  0  0
   -0.7941    7.3251    2.0378 C   0  0  0  0  0  0  0  0  0  0  0  0
   -1.6512    7.9848    1.7678 H   0  0  0  0  0  0  0  0  0  0  0  0
   -0.6769    7.3328    3.1452 H   0  0  0  0  0  0  0  0  0  0  0  0
    0.1382    7.7320    1.5780 H   0  0  0  0  0  0  0  0  0  0  0  0
    3.2892    5.7759    4.3640 C   0  0  0  0  0  0  0  0  0  0  0  0
    4.2836    5.5134    4.7991 H   0  0  0  0  0  0  0  0  0  0  0  0
    3.3518    6.7904    3.9086 H   0  0  0  0  0  0  0  0  0  0  0  0
    2.5352    5.8028    5.1837 H   0  0  0  0  0  0  0  0  0  0  0  0
    1.6235    4.6162   -5.1943 C   0  0  0  0  0  0  0  0  0  0  0  0
    2.5676    5.1940   -5.3381 H   0  0  0  0  0  0  0  0  0  0  0  0
    1.5036    3.9048   -6.0436 H   0  0  0  0  0  0  0  0  0  0  0  0
    0.7625    5.3227   -5.2033 H   0  0  0  0  0  0  0  0  0  0  0  0
   -3.7576    2.2831   -5.8422 C   0  0  0  0  0  0  0  0  0  0  0  0
   -4.7334    1.7615   -5.9684 H   0  0  0  0  0  0  0  0  0  0  0  0
   -3.8920    3.3645   -6.0832 H   0  0  0  0  0  0  0  0  0  0  0  0
   -3.0275    1.8418   -6.5595 H   0  0  0  0  0  0  0  0  0  0  0  0
   -2.7516   -4.3535   -5.1759 C   0  0  0  0  0  0  0  0  0  0  0  0
   -3.7473   -4.0515   -5.5776 H   0  0  0  0  0  0  0  0  0  0  0  0
   -1.9592   -4.0335   -5.8941 H   0  0  0  0  0  0  0  0  0  0  0  0
   -2.7251   -5.4622   -5.0871 H   0  0  0  0  0  0  0  0  0  0  0  0
    2.2953   -6.7334   -3.0303 C   0  0  0  0  0  0  0  0  0  0  0  0
    1.4885   -7.4551   -2.7584 H   0  0  0  0  0  0  0  0  0  0  0  0
    2.1973   -6.4724   -4.1100 H   0  0  0  0  0  0  0  0  0  0  0  0
    3.2828   -7.2228   -2.8740 H   0  0  0  0  0  0  0  0  0  0  0  0
    2.5839   -3.3576    6.2610 C   0  0  0  0  0  0  0  0  0  0  0  0
    3.5914   -3.0239    6.6033 H   0  0  0  0  0  0  0  0  0  0  0  0
    1.8053   -2.8590    6.8876 H   0  0  0  0  0  0  0  0  0  0  0  0
    2.5061   -4.4581    6.4051 H   0  0  0  0  0  0  0  0  0  0  0  0
   -2.8847   -5.7416    4.5409 C   0  0  0  0  0  0  0  0  0  0  0  0
   -2.1605   -6.5824    4.4223 H   0  0  0  0  0  0  0  0  0  0  0  0
   -2.7533   -5.2912    5.5527 H   0  0  0  0  0  0  0  0  0  0  0  0
   -3.9192   -6.1459    4.4670 H   0  0  0  0  0  0  0  0  0  0  0  0
  1  2  1  0  0  0  0
  1  8  1  0  0  0  0
  1 12  1  0  0  0  0
  1 16  1  0  0  0  0
  2  3  1  0  0  0  0
  2 11  1  0  0  0  0
  2 15  1  0  0  0  0
  3 37  1  0  0  0  0
  3 47  1  0  0  0  0
  3 97  1  0  0  0  0
  4  5  1  0  0  0  0
  4 77  1  0  0  0  0
  4 87  1  0  0  0  0
  4 97  1  0  0  0  0
  5  6  1  0  0  0  0
  5  9  1  0  0  0  0
  5 13  1  0  0  0  0
  6  7  1  0  0  0  0
  6 10  1  0  0  0  0
  6 14  1  0  0  0  0
  7 57  1  0  0  0  0
  7 67  1  0  0  0  0
  7 97  1  0  0  0  0
```

```
  8 17  1  0  0  0  0
  8 27  1  0  0  0  0
  8 97  1  0  0  0  0
 17 18  2  0  0  0  0
 17 19  1  0  0  0  0
 18 20  1  0  0  0  0
 18 21  1  0  0  0  0
 19 22  2  0  0  0  0
 19 23  1  0  0  0  0
 20 24  2  0  0  0  0
 20 25  1  0  0  0  0
 22 24  1  0  0  0  0
 22 26  1  0  0  0  0
 24108  1  0  0  0  0
 27 28  2  0  0  0  0
 27 29  1  0  0  0  0
 28 30  1  0  0  0  0
 28 31  1  0  0  0  0
 29 32  2  0  0  0  0
 29 33  1  0  0  0  0
 30 34  2  0  0  0  0
 30 35  1  0  0  0  0
 32 34  1  0  0  0  0
 32 36  1  0  0  0  0
 34104  1  0  0  0  0
 37 38  2  0  0  0  0
 37 39  1  0  0  0  0
 38 40  1  0  0  0  0
 38 41  1  0  0  0  0
 39 42  2  0  0  0  0
 39 43  1  0  0  0  0
 40 44  2  0  0  0  0
 40 45  1  0  0  0  0
 42 44  1  0  0  0  0
 42 46  1  0  0  0  0
 44124  1  0  0  0  0
 47 48  2  0  0  0  0
 47 49  1  0  0  0  0
 48 50  1  0  0  0  0
 48 51  1  0  0  0  0
 49 52  2  0  0  0  0
 49 53  1  0  0  0  0
 50 54  2  0  0  0  0
 50 55  1  0  0  0  0
 52 54  1  0  0  0  0
 52 56  1  0  0  0  0
 54120  1  0  0  0  0
 57 58  2  0  0  0  0
 57 59  1  0  0  0  0
 58 60  1  0  0  0  0
 58 61  1  0  0  0  0
 59 62  2  0  0  0  0
 59 63  1  0  0  0  0
 60 64  2  0  0  0  0
 60 65  1  0  0  0  0
 62 64  1  0  0  0  0
 62 66  1  0  0  0  0
 64128  1  0  0  0  0
 67 68  2  0  0  0  0
 67 69  1  0  0  0  0
 68 70  1  0  0  0  0
 68 71  1  0  0  0  0
 69 72  2  0  0  0  0
 69 73  1  0  0  0  0
 70 74  2  0  0  0  0
```

```
 70 75  1  0  0  0  0
 72 74  1  0  0  0  0
 72 76  1  0  0  0  0
 74116  1  0  0  0  0
 77 78  2  0  0  0  0
 77 79  1  0  0  0  0
 78 80  1  0  0  0  0
 78 81  1  0  0  0  0
 79 82  2  0  0  0  0
 79 83  1  0  0  0  0
 80 84  2  0  0  0  0
 80 85  1  0  0  0  0
 82 84  1  0  0  0  0
 82 86  1  0  0  0  0
 84100  1  0  0  0  0
 87 88  2  0  0  0  0
 87 89  1  0  0  0  0
 88 90  1  0  0  0  0
 88 91  1  0  0  0  0
 89 92  2  0  0  0  0
 89 93  1  0  0  0  0
 90 94  2  0  0  0  0
 90 95  1  0  0  0  0
 92 94  1  0  0  0  0
 92 96  1  0  0  0  0
 94112  1  0  0  0  0
 97 98  1  0  0  0  0
 97 99  1  0  0  0  0
100101  1  0  0  0  0
100102  1  0  0  0  0
100103  1  0  0  0  0
104105  1  0  0  0  0
104106  1  0  0  0  0
104107  1  0  0  0  0
108109  1  0  0  0  0
108110  1  0  0  0  0
108111  1  0  0  0  0
112113  1  0  0  0  0
112114  1  0  0  0  0
112115  1  0  0  0  0
116117  1  0  0  0  0
116118  1  0  0  0  0
116119  1  0  0  0  0
120121  1  0  0  0  0
120122  1  0  0  0  0
120123  1  0  0  0  0
124125  1  0  0  0  0
124126  1  0  0  0  0
124127  1  0  0  0  0
128129  1  0  0  0  0
128130  1  0  0  0  0
128131  1  0  0  0  0
M  END
$$$$
mol046
Optimized Catalyst 046
Optimized Catalyst 046
115124  0  0  0  0  0  0  0  0999 V2000
    3.7873    0.4949   -0.3415 C   0  0  0  0  0  0  0  0  0  0  0  0
    3.5615   -0.9458   -0.7529 C   0  0  0  0  0  0  0  0  0  0  0  0
    2.0129   -1.6006    0.0989 P   0  0  0  0  0  0  0  0  0  0  0  0
   -1.2427    1.6441   -1.0631 P   0  0  0  0  0  0  0  0  0  0  0  0
   -2.7197    1.0780   -0.0242 C   0  0  0  0  0  0  0  0  0  0  0  0
   -3.0174   -0.3818   -0.2873 C   0  0  0  0  0  0  0  0  0  0  0  0
   -1.4507   -1.4347   -0.1601 P   0  0  0  0  0  0  0  0  0  0  0  0
```

2.2297	1.5101	-0.6710	P	0	0	0	0	0	0	0	0	0	0	0	0
-3.6157	1.7023	-0.2469	H	0	0	0	0	0	0	0	0	0	0	0	0
-3.4258	-0.5464	-1.3202	H	0	0	0	0	0	0	0	0	0	0	0	0
3.3819	-1.0285	-1.8639	H	0	0	0	0	0	0	0	0	0	0	0	0
4.6247	0.9357	-0.9424	H	0	0	0	0	0	0	0	0	0	0	0	0
-2.4222	1.2366	1.0545	H	0	0	0	0	0	0	0	0	0	0	0	0
-3.7822	-0.7472	0.4431	H	0	0	0	0	0	0	0	0	0	0	0	0
4.4448	-1.5710	-0.4770	H	0	0	0	0	0	0	0	0	0	0	0	0
4.0511	0.5652	0.7461	H	0	0	0	0	0	0	0	0	0	0	0	0
1.7260	3.0738	-2.6684	C	0	0	0	0	0	0	0	0	0	0	0	0
1.4427	4.3603	-2.1925	C	0	0	0	0	0	0	0	0	0	0	0	0
1.1024	2.5241	-3.7917	C	0	0	0	0	0	0	0	0	0	0	0	0
0.4968	5.1194	-2.8851	C	0	0	0	0	0	0	0	0	0	0	0	0
1.9319	4.7394	-1.2951	H	0	0	0	0	0	0	0	0	0	0	0	0
0.1580	3.3041	-4.4635	C	0	0	0	0	0	0	0	0	0	0	0	0
1.3303	1.5032	-4.1089	H	0	0	0	0	0	0	0	0	0	0	0	0
-0.1386	4.5978	-4.0181	C	0	0	0	0	0	0	0	0	0	0	0	0
0.2465	6.1181	-2.5301	H	0	0	0	0	0	0	0	0	0	0	0	0
-0.3638	2.8889	-5.3256	H	0	0	0	0	0	0	0	0	0	0	0	0
-0.8762	5.1949	-4.5504	H	0	0	0	0	0	0	0	0	0	0	0	0
3.4600	3.0230	1.1301	C	0	0	0	0	0	0	0	0	0	0	0	0
3.3526	2.5425	2.4427	C	0	0	0	0	0	0	0	0	0	0	0	0
4.6044	3.6807	0.6581	C	0	0	0	0	0	0	0	0	0	0	0	0
4.4371	2.7264	3.3013	C	0	0	0	0	0	0	0	0	0	0	0	0
2.4326	2.0491	2.7705	H	0	0	0	0	0	0	0	0	0	0	0	0
5.6742	3.8525	1.5390	C	0	0	0	0	0	0	0	0	0	0	0	0
4.6388	4.0512	-0.3653	H	0	0	0	0	0	0	0	0	0	0	0	0
5.5950	3.3740	2.8519	C	0	0	0	0	0	0	0	0	0	0	0	0
4.3804	2.3615	4.3272	H	0	0	0	0	0	0	0	0	0	0	0	0
6.5756	4.3628	1.1986	H	0	0	0	0	0	0	0	0	0	0	0	0
6.4366	3.5094	3.5289	H	0	0	0	0	0	0	0	0	0	0	0	0
1.7087	-2.3087	2.5841	C	0	0	0	0	0	0	0	0	0	0	0	0
1.0315	-3.5323	2.5223	C	0	0	0	0	0	0	0	0	0	0	0	0
1.5608	-1.4118	3.6495	C	0	0	0	0	0	0	0	0	0	0	0	0
0.1590	-3.8512	3.5662	C	0	0	0	0	0	0	0	0	0	0	0	0
1.1656	-4.2047	1.6734	H	0	0	0	0	0	0	0	0	0	0	0	0
0.6885	-1.7604	4.6828	C	0	0	0	0	0	0	0	0	0	0	0	0
2.0999	-0.4662	3.6458	H	0	0	0	0	0	0	0	0	0	0	0	0
-0.0113	-2.9728	4.6420	C	0	0	0	0	0	0	0	0	0	0	0	0
-0.4003	-4.7853	3.5315	H	0	0	0	0	0	0	0	0	0	0	0	0
0.5423	-1.0746	5.5162	H	0	0	0	0	0	0	0	0	0	0	0	0
-0.7020	-3.2266	5.4434	H	0	0	0	0	0	0	0	0	0	0	0	0
2.8793	-3.8026	-1.0575	C	0	0	0	0	0	0	0	0	0	0	0	0
2.8836	-3.7115	-2.4559	C	0	0	0	0	0	0	0	0	0	0	0	0
3.8309	-4.5423	-0.3442	C	0	0	0	0	0	0	0	0	0	0	0	0
3.8758	-4.3973	-3.1559	C	0	0	0	0	0	0	0	0	0	0	0	0
2.1066	-3.1340	-2.9699	H	0	0	0	0	0	0	0	0	0	0	0	0
4.8141	-5.2191	-1.0704	C	0	0	0	0	0	0	0	0	0	0	0	0
3.7879	-4.5870	0.7429	H	0	0	0	0	0	0	0	0	0	0	0	0
4.8395	-5.1461	-2.4675	C	0	0	0	0	0	0	0	0	0	0	0	0
3.8962	-4.3508	-4.2450	H	0	0	0	0	0	0	0	0	0	0	0	0
5.5635	-5.8084	-0.5420	H	0	0	0	0	0	0	0	0	0	0	0	0
5.6103	-5.6766	-3.0235	H	0	0	0	0	0	0	0	0	0	0	0	0
-2.6673	-2.2974	2.0279	C	0	0	0	0	0	0	0	0	0	0	0	0
-2.6011	-1.4078	3.1087	C	0	0	0	0	0	0	0	0	0	0	0	0
-3.7735	-3.1270	1.7984	C	0	0	0	0	0	0	0	0	0	0	0	0
-3.6858	-1.3553	3.9838	C	0	0	0	0	0	0	0	0	0	0	0	0
-1.7064	-0.7918	3.2543	H	0	0	0	0	0	0	0	0	0	0	0	0
-4.8456	-3.0545	2.6909	C	0	0	0	0	0	0	0	0	0	0	0	0
-3.7787	-3.8111	0.9510	H	0	0	0	0	0	0	0	0	0	0	0	0
-4.8055	-2.1714	3.7758	C	0	0	0	0	0	0	0	0	0	0	0	0
-3.6581	-0.6738	4.8343	H	0	0	0	0	0	0	0	0	0	0	0	0
-5.7164	-3.6921	2.5390	H	0	0	0	0	0	0	0	0	0	0	0	0
-5.6470	-2.1211	4.4644	H	0	0	0	0	0	0	0	0	0	0	0	0
-1.1564	-3.6480	-1.5844	C	0	0	0	0	0	0	0	0	0	0	0	0

```
   -0.7962   -3.6732   -2.9375 C   0  0  0  0  0  0  0  0  0  0  0  0
   -0.7903   -4.6533   -0.6810 C   0  0  0  0  0  0  0  0  0  0  0  0
   -0.0544   -4.7627   -3.3971 C   0  0  0  0  0  0  0  0  0  0  0  0
   -1.0778   -2.8474   -3.5923 H   0  0  0  0  0  0  0  0  0  0  0  0
   -0.0366   -5.7251   -1.1664 C   0  0  0  0  0  0  0  0  0  0  0  0
   -1.0742   -4.5871    0.3699 H   0  0  0  0  0  0  0  0  0  0  0  0
    0.3229   -5.7856   -2.5175 C   0  0  0  0  0  0  0  0  0  0  0  0
    0.2395   -4.8096   -4.4451 H   0  0  0  0  0  0  0  0  0  0  0  0
    0.2782   -6.5132   -0.4838 H   0  0  0  0  0  0  0  0  0  0  0  0
    0.9131   -6.6230   -2.8834 H   0  0  0  0  0  0  0  0  0  0  0  0
   -1.4405    3.7660    0.4900 C   0  0  0  0  0  0  0  0  0  0  0  0
   -2.7049    4.3187    0.7426 C   0  0  0  0  0  0  0  0  0  0  0  0
   -0.4214    3.7522    1.4473 C   0  0  0  0  0  0  0  0  0  0  0  0
   -2.9489    4.8555    2.0085 C   0  0  0  0  0  0  0  0  0  0  0  0
   -3.4644    4.3384   -0.0346 H   0  0  0  0  0  0  0  0  0  0  0  0
   -0.6937    4.2900    2.7073 C   0  0  0  0  0  0  0  0  0  0  0  0
    0.5623    3.3256    1.2183 H   0  0  0  0  0  0  0  0  0  0  0  0
   -1.9520    4.8342    2.9905 C   0  0  0  0  0  0  0  0  0  0  0  0
   -3.9217    5.2924    2.2298 H   0  0  0  0  0  0  0  0  0  0  0  0
    0.0835    4.2759    3.4721 H   0  0  0  0  0  0  0  0  0  0  0  0
   -2.1552    5.2434    3.9779 H   0  0  0  0  0  0  0  0  0  0  0  0
   -3.0354    2.4051   -2.8488 C   0  0  0  0  0  0  0  0  0  0  0  0
   -4.0607    1.5621   -3.3027 C   0  0  0  0  0  0  0  0  0  0  0  0
   -3.2078    3.7901   -2.7256 C   0  0  0  0  0  0  0  0  0  0  0  0
   -5.2964    2.1304   -3.6162 C   0  0  0  0  0  0  0  0  0  0  0  0
   -3.8717    0.4978   -3.4288 H   0  0  0  0  0  0  0  0  0  0  0  0
   -4.4537    4.3336   -3.0427 C   0  0  0  0  0  0  0  0  0  0  0  0
   -2.3730    4.4190   -2.4009 H   0  0  0  0  0  0  0  0  0  0  0  0
   -5.4973    3.5092   -3.4789 C   0  0  0  0  0  0  0  0  0  0  0  0
   -6.1075    1.4945   -3.9713 H   0  0  0  0  0  0  0  0  0  0  0  0
   -4.6101    5.4088   -2.9538 H   0  0  0  0  0  0  0  0  0  0  0  0
   -6.4663    3.9424   -3.7200 H   0  0  0  0  0  0  0  0  0  0  0  0
    0.3897    0.0356   -0.4552 Os  0  0  0  0  0  0  0  0  0  0  0  0
    0.1205    0.7928    2.1661 Cl  0  0  0  0  0  0  0  0  0  0  0  0
    0.6846   -0.7995   -3.0471 Cl  0  0  0  0  0  0  0  0  0  0  0  0
   -1.5525   -2.4407    1.2122 O   0  0  0  0  0  0  0  0  0  0  0  0
   -2.0389   -2.6380   -1.1937 O   0  0  0  0  0  0  0  0  0  0  0  0
   -1.7888    1.8136   -2.6563 O   0  0  0  0  0  0  0  0  0  0  0  0
   -1.1944    3.3260   -0.8134 O   0  0  0  0  0  0  0  0  0  0  0  0
    2.7497    2.3308   -2.0496 O   0  0  0  0  0  0  0  0  0  0  0  0
    2.3589    2.9279    0.2921 O   0  0  0  0  0  0  0  0  0  0  0  0
    1.8246   -3.2295   -0.3515 O   0  0  0  0  0  0  0  0  0  0  0  0
    2.6687   -1.9807    1.6126 O   0  0  0  0  0  0  0  0  0  0  0  0
  1  2  1  0  0  0  0
  1  8  1  0  0  0  0
  1 12  1  0  0  0  0
  1 16  1  0  0  0  0
  2  3  1  0  0  0  0
  2 11  1  0  0  0  0
  2 15  1  0  0  0  0
  3105  1  0  0  0  0
  3114  1  0  0  0  0
  3115  1  0  0  0  0
  4  5  1  0  0  0  0
  4105  1  0  0  0  0
  4110  1  0  0  0  0
  4111  1  0  0  0  0
  5  6  1  0  0  0  0
  5  9  1  0  0  0  0
  5 13  1  0  0  0  0
  6  7  1  0  0  0  0
  6 10  1  0  0  0  0
  6 14  1  0  0  0  0
  7105  1  0  0  0  0
  7108  1  0  0  0  0
```

```
  7109  1  0  0  0  0
  8105  1  0  0  0  0
  8112  1  0  0  0  0
  8113  1  0  0  0  0
 17 18  2  0  0  0  0
 17 19  1  0  0  0  0
 17112  1  0  0  0  0
 18 20  1  0  0  0  0
 18 21  1  0  0  0  0
 19 22  2  0  0  0  0
 19 23  1  0  0  0  0
 20 24  2  0  0  0  0
 20 25  1  0  0  0  0
 22 24  1  0  0  0  0
 22 26  1  0  0  0  0
 24 27  1  0  0  0  0
 28 29  2  0  0  0  0
 28 30  1  0  0  0  0
 28113  1  0  0  0  0
 29 31  1  0  0  0  0
 29 32  1  0  0  0  0
 30 33  2  0  0  0  0
 30 34  1  0  0  0  0
 31 35  2  0  0  0  0
 31 36  1  0  0  0  0
 33 35  1  0  0  0  0
 33 37  1  0  0  0  0
 35 38  1  0  0  0  0
 39 40  2  0  0  0  0
 39 41  1  0  0  0  0
 39115  1  0  0  0  0
 40 42  1  0  0  0  0
 40 43  1  0  0  0  0
 41 44  2  0  0  0  0
 41 45  1  0  0  0  0
 42 46  2  0  0  0  0
 42 47  1  0  0  0  0
 44 46  1  0  0  0  0
 44 48  1  0  0  0  0
 46 49  1  0  0  0  0
 50 51  2  0  0  0  0
 50 52  1  0  0  0  0
 50114  1  0  0  0  0
 51 53  1  0  0  0  0
 51 54  1  0  0  0  0
 52 55  2  0  0  0  0
 52 56  1  0  0  0  0
 53 57  2  0  0  0  0
 53 58  1  0  0  0  0
 55 57  1  0  0  0  0
 55 59  1  0  0  0  0
 57 60  1  0  0  0  0
 61 62  2  0  0  0  0
 61 63  1  0  0  0  0
 61108  1  0  0  0  0
 62 64  1  0  0  0  0
 62 65  1  0  0  0  0
 63 66  2  0  0  0  0
 63 67  1  0  0  0  0
 64 68  2  0  0  0  0
 64 69  1  0  0  0  0
 66 68  1  0  0  0  0
 66 70  1  0  0  0  0
 68 71  1  0  0  0  0
 72 73  2  0  0  0  0
```

```
 72 74  1  0  0  0  0
 72109  1  0  0  0  0
 73 75  1  0  0  0  0
 73 76  1  0  0  0  0
 74 77  2  0  0  0  0
 74 78  1  0  0  0  0
 75 79  2  0  0  0  0
 75 80  1  0  0  0  0
 77 79  1  0  0  0  0
 77 81  1  0  0  0  0
 79 82  1  0  0  0  0
 83 84  2  0  0  0  0
 83 85  1  0  0  0  0
 83111  1  0  0  0  0
 84 86  1  0  0  0  0
 84 87  1  0  0  0  0
 85 88  2  0  0  0  0
 85 89  1  0  0  0  0
 86 90  2  0  0  0  0
 86 91  1  0  0  0  0
 88 90  1  0  0  0  0
 88 92  1  0  0  0  0
 90 93  1  0  0  0  0
 94 95  2  0  0  0  0
 94 96  1  0  0  0  0
 94110  1  0  0  0  0
 95 97  1  0  0  0  0
 95 98  1  0  0  0  0
 96 99  2  0  0  0  0
 96100  1  0  0  0  0
 97101  2  0  0  0  0
 97102  1  0  0  0  0
 99101  1  0  0  0  0
 99103  1  0  0  0  0
101104  1  0  0  0  0
105106  1  0  0  0  0
105107  1  0  0  0  0
M  END
$$$$
mol047
Optimized Catalyst 047
Optimized Catalyst 047
115124  0  0  0  0  0  0  0  0999 V2000
    3.7855    0.5175   -0.3321 C   0  0  0  0  0  0  0  0  0  0  0  0
    3.5712   -0.9182   -0.7693 C   0  0  0  0  0  0  0  0  0  0  0  0
    2.0158   -1.5896    0.0550 P   0  0  0  0  0  0  0  0  0  0  0  0
   -1.2384    1.6744   -1.0552 P   0  0  0  0  0  0  0  0  0  0  0  0
   -2.7253    1.0898   -0.0425 C   0  0  0  0  0  0  0  0  0  0  0  0
   -3.0175   -0.3678   -0.3250 C   0  0  0  0  0  0  0  0  0  0  0  0
   -1.4508   -1.4161   -0.1812 P   0  0  0  0  0  0  0  0  0  0  0  0
    2.2305    1.5305   -0.6728 P   0  0  0  0  0  0  0  0  0  0  0  0
   -3.6213    1.7143   -0.2658 H   0  0  0  0  0  0  0  0  0  0  0  0
   -3.4120   -0.5240   -1.3647 H   0  0  0  0  0  0  0  0  0  0  0  0
    3.4101   -0.9847   -1.8835 H   0  0  0  0  0  0  0  0  0  0  0  0
    4.6349    0.9676   -0.9093 H   0  0  0  0  0  0  0  0  0  0  0  0
   -2.4409    1.2347    1.0418 H   0  0  0  0  0  0  0  0  0  0  0  0
   -3.7941   -0.7417    0.3890 H   0  0  0  0  0  0  0  0  0  0  0  0
    4.4539   -1.5432   -0.4882 H   0  0  0  0  0  0  0  0  0  0  0  0
    4.0286    0.5730    0.7617 H   0  0  0  0  0  0  0  0  0  0  0  0
    1.7144    3.0820   -2.6748 C   0  0  0  0  0  0  0  0  0  0  0  0
    1.3787    4.3523   -2.1845 C   0  0  0  0  0  0  0  0  0  0  0  0
    1.1426    2.5324   -3.8297 C   0  0  0  0  0  0  0  0  0  0  0  0
    0.4384    5.1091   -2.8855 C   0  0  0  0  0  0  0  0  0  0  0  0
    1.8319    4.7283   -1.2643 H   0  0  0  0  0  0  0  0  0  0  0  0
    0.2030    3.2881   -4.5319 C   0  0  0  0  0  0  0  0  0  0  0  0
```

```
    1.4150    1.5227   -4.1531 H   0  0  0  0  0  0  0  0  0  0  0  0
   -0.1212    4.5591   -4.0439 C   0  0  0  0  0  0  0  0  0  0  0  0
    0.1450    6.0956   -2.5293 H   0  0  0  0  0  0  0  0  0  0  0  0
   -0.2788    2.8835   -5.4222 H   0  0  0  0  0  0  0  0  0  0  0  0
    3.4463    3.0405    1.1373 C   0  0  0  0  0  0  0  0  0  0  0  0
    3.3114    2.5921    2.4609 C   0  0  0  0  0  0  0  0  0  0  0  0
    4.6072    3.6704    0.6629 C   0  0  0  0  0  0  0  0  0  0  0  0
    4.3783    2.7729    3.3398 C   0  0  0  0  0  0  0  0  0  0  0  0
    2.3775    2.1188    2.7849 H   0  0  0  0  0  0  0  0  0  0  0  0
    5.6761    3.8509    1.5411 C   0  0  0  0  0  0  0  0  0  0  0  0
    4.6568    4.0162   -0.3707 H   0  0  0  0  0  0  0  0  0  0  0  0
    5.5365    3.3950    2.8570 C   0  0  0  0  0  0  0  0  0  0  0  0
    4.3080    2.4397    4.3761 H   0  0  0  0  0  0  0  0  0  0  0  0
    6.5931    4.3381    1.2078 H   0  0  0  0  0  0  0  0  0  0  0  0
    1.6980   -2.3084    2.5318 C   0  0  0  0  0  0  0  0  0  0  0  0
    1.0001   -3.5224    2.4751 C   0  0  0  0  0  0  0  0  0  0  0  0
    1.5642   -1.3986    3.5918 C   0  0  0  0  0  0  0  0  0  0  0  0
    0.1237   -3.8370    3.5158 C   0  0  0  0  0  0  0  0  0  0  0  0
    1.1265   -4.1994    1.6266 H   0  0  0  0  0  0  0  0  0  0  0  0
    0.6950   -1.7157    4.6361 C   0  0  0  0  0  0  0  0  0  0  0  0
    2.1263   -0.4645    3.5820 H   0  0  0  0  0  0  0  0  0  0  0  0
   -0.0068   -2.9252    4.5683 C   0  0  0  0  0  0  0  0  0  0  0  0
   -0.4511   -4.7619    3.4965 H   0  0  0  0  0  0  0  0  0  0  0  0
    0.5578   -1.0290    5.4707 H   0  0  0  0  0  0  0  0  0  0  0  0
    2.8851   -3.7918   -1.1030 C   0  0  0  0  0  0  0  0  0  0  0  0
    2.9414   -3.6833   -2.5014 C   0  0  0  0  0  0  0  0  0  0  0  0
    3.7837   -4.5722   -0.3623 C   0  0  0  0  0  0  0  0  0  0  0  0
    3.9309   -4.3865   -3.1856 C   0  0  0  0  0  0  0  0  0  0  0  0
    2.2024   -3.0719   -3.0339 H   0  0  0  0  0  0  0  0  0  0  0  0
    4.7757   -5.2769   -1.0465 C   0  0  0  0  0  0  0  0  0  0  0  0
    3.6959   -4.6276    0.7234 H   0  0  0  0  0  0  0  0  0  0  0  0
    4.8245   -5.1665   -2.4403 C   0  0  0  0  0  0  0  0  0  0  0  0
    3.9988   -4.3352   -4.2727 H   0  0  0  0  0  0  0  0  0  0  0  0
    5.4880   -5.9005   -0.5055 H   0  0  0  0  0  0  0  0  0  0  0  0
   -2.6771   -2.2847    1.9997 C   0  0  0  0  0  0  0  0  0  0  0  0
   -2.6212   -1.4037    3.0912 C   0  0  0  0  0  0  0  0  0  0  0  0
   -3.7723   -3.1265    1.7564 C   0  0  0  0  0  0  0  0  0  0  0  0
   -3.7007   -1.3610    3.9711 C   0  0  0  0  0  0  0  0  0  0  0  0
   -1.7328   -0.7782    3.2420 H   0  0  0  0  0  0  0  0  0  0  0  0
   -4.8552   -3.0839    2.6363 C   0  0  0  0  0  0  0  0  0  0  0  0
   -3.7662   -3.8021    0.9002 H   0  0  0  0  0  0  0  0  0  0  0  0
   -4.7937   -2.2003    3.7193 C   0  0  0  0  0  0  0  0  0  0  0  0
   -3.6874   -0.6951    4.8351 H   0  0  0  0  0  0  0  0  0  0  0  0
   -5.7220   -3.7270    2.4829 H   0  0  0  0  0  0  0  0  0  0  0  0
   -1.1389   -3.6430   -1.5703 C   0  0  0  0  0  0  0  0  0  0  0  0
   -0.7276   -3.6675   -2.9115 C   0  0  0  0  0  0  0  0  0  0  0  0
   -0.8151   -4.6534   -0.6536 C   0  0  0  0  0  0  0  0  0  0  0  0
    0.0281   -4.7518   -3.3566 C   0  0  0  0  0  0  0  0  0  0  0  0
   -0.9927   -2.8424   -3.5774 H   0  0  0  0  0  0  0  0  0  0  0  0
   -0.0472   -5.7329   -1.0951 C   0  0  0  0  0  0  0  0  0  0  0  0
   -1.1508   -4.5886    0.3837 H   0  0  0  0  0  0  0  0  0  0  0  0
    0.3515   -5.7561   -2.4357 C   0  0  0  0  0  0  0  0  0  0  0  0
    0.3611   -4.8084   -4.3926 H   0  0  0  0  0  0  0  0  0  0  0  0
    0.2358   -6.5300   -0.4094 H   0  0  0  0  0  0  0  0  0  0  0  0
   -1.4313    3.7751    0.5323 C   0  0  0  0  0  0  0  0  0  0  0  0
   -2.6965    4.3195    0.8021 C   0  0  0  0  0  0  0  0  0  0  0  0
   -0.3974    3.7622    1.4790 C   0  0  0  0  0  0  0  0  0  0  0  0
   -2.9428    4.8544    2.0676 C   0  0  0  0  0  0  0  0  0  0  0  0
   -3.4652    4.3365    0.0312 H   0  0  0  0  0  0  0  0  0  0  0  0
   -0.6408    4.2948    2.7449 C   0  0  0  0  0  0  0  0  0  0  0  0
    0.5848    3.3408    1.2302 H   0  0  0  0  0  0  0  0  0  0  0  0
   -1.9093    4.8238    3.0099 C   0  0  0  0  0  0  0  0  0  0  0  0
   -3.9144    5.2840    2.3096 H   0  0  0  0  0  0  0  0  0  0  0  0
    0.1412    4.2873    3.5049 H   0  0  0  0  0  0  0  0  0  0  0  0
   -3.0223    2.4180   -2.8428 C   0  0  0  0  0  0  0  0  0  0  0  0
```

```
   -3.9918    1.5299   -3.3355 C   0  0  0  0  0  0  0  0  0  0  0  0
   -3.2697    3.7890   -2.6772 C   0  0  0  0  0  0  0  0  0  0  0  0
   -5.2633    2.0198   -3.6331 C   0  0  0  0  0  0  0  0  0  0  0  0
   -3.7322    0.4843   -3.5046 H   0  0  0  0  0  0  0  0  0  0  0  0
   -4.5408    4.2817   -2.9715 C   0  0  0  0  0  0  0  0  0  0  0  0
   -2.4678    4.4535   -2.3370 H   0  0  0  0  0  0  0  0  0  0  0  0
   -5.5093    3.3833   -3.4340 C   0  0  0  0  0  0  0  0  0  0  0  0
   -6.0401    1.3579   -4.0166 H   0  0  0  0  0  0  0  0  0  0  0  0
   -4.7668    5.3423   -2.8550 H   0  0  0  0  0  0  0  0  0  0  0  0
    0.3906    0.0555   -0.4670 Os  0  0  0  0  0  0  0  0  0  0  0  0
    0.1216    0.7896    2.1597 Cl  0  0  0  0  0  0  0  0  0  0  0  0
    0.6813   -0.7555   -3.0666 Cl  0  0  0  0  0  0  0  0  0  0  0  0
   -1.5498   -2.4133    1.1982 O   0  0  0  0  0  0  0  0  0  0  0  0
   -2.0298   -2.6310   -1.2098 O   0  0  0  0  0  0  0  0  0  0  0  0
   -1.7436    1.8946   -2.6551 O   0  0  0  0  0  0  0  0  0  0  0  0
   -1.1931    3.3548   -0.7770 O   0  0  0  0  0  0  0  0  0  0  0  0
    2.7464    2.3603   -2.0485 O   0  0  0  0  0  0  0  0  0  0  0  0
    2.3509    2.9499    0.2922 O   0  0  0  0  0  0  0  0  0  0  0  0
    1.8143   -3.2063   -0.4324 O   0  0  0  0  0  0  0  0  0  0  0  0
    2.6662   -2.0067    1.5626 O   0  0  0  0  0  0  0  0  0  0  0  0
   -0.8507   -3.2211    5.5603 F   0  0  0  0  0  0  0  0  0  0  0  0
   -5.8340   -2.1517    4.5541 F   0  0  0  0  0  0  0  0  0  0  0  0
    5.7747   -5.8378   -3.0944 F   0  0  0  0  0  0  0  0  0  0  0  0
    1.0858   -6.7879   -2.8597 F   0  0  0  0  0  0  0  0  0  0  0  0
   -6.7298    3.8513   -3.7033 F   0  0  0  0  0  0  0  0  0  0  0  0
   -1.0193    5.2848   -4.7161 F   0  0  0  0  0  0  0  0  0  0  0  0
    6.5615    3.5613    3.6954 F   0  0  0  0  0  0  0  0  0  0  0  0
   -2.1455    5.3296    4.2227 F   0  0  0  0  0  0  0  0  0  0  0  0
  1  2  1  0  0  0  0
  1  8  1  0  0  0  0
  1 12  1  0  0  0  0
  1 16  1  0  0  0  0
  2  3  1  0  0  0  0
  2 11  1  0  0  0  0
  2 15  1  0  0  0  0
  3 97  1  0  0  0  0
  3106  1  0  0  0  0
  3107  1  0  0  0  0
  4  5  1  0  0  0  0
  4 97  1  0  0  0  0
  4102  1  0  0  0  0
  4103  1  0  0  0  0
  5  6  1  0  0  0  0
  5  9  1  0  0  0  0
  5 13  1  0  0  0  0
  6  7  1  0  0  0  0
  6 10  1  0  0  0  0
  6 14  1  0  0  0  0
  7 97  1  0  0  0  0
  7100  1  0  0  0  0
  7101  1  0  0  0  0
  8 97  1  0  0  0  0
  8104  1  0  0  0  0
  8105  1  0  0  0  0
 17 18  2  0  0  0  0
 17 19  1  0  0  0  0
 17104  1  0  0  0  0
 18 20  1  0  0  0  0
 18 21  1  0  0  0  0
 19 22  2  0  0  0  0
 19 23  1  0  0  0  0
 20 24  2  0  0  0  0
 20 25  1  0  0  0  0
 22 24  1  0  0  0  0
 22 26  1  0  0  0  0
```

```
24113  1  0  0  0  0
 27 28  2  0  0  0  0
 27 29  1  0  0  0  0
 27105  1  0  0  0  0
 28 30  1  0  0  0  0
 28 31  1  0  0  0  0
 29 32  2  0  0  0  0
 29 33  1  0  0  0  0
 30 34  2  0  0  0  0
 30 35  1  0  0  0  0
 32 34  1  0  0  0  0
 32 36  1  0  0  0  0
 34114  1  0  0  0  0
 37 38  2  0  0  0  0
 37 39  1  0  0  0  0
 37107  1  0  0  0  0
 38 40  1  0  0  0  0
 38 41  1  0  0  0  0
 39 42  2  0  0  0  0
 39 43  1  0  0  0  0
 40 44  2  0  0  0  0
 40 45  1  0  0  0  0
 42 44  1  0  0  0  0
 42 46  1  0  0  0  0
 44108  1  0  0  0  0
 47 48  2  0  0  0  0
 47 49  1  0  0  0  0
 47106  1  0  0  0  0
 48 50  1  0  0  0  0
 48 51  1  0  0  0  0
 49 52  2  0  0  0  0
 49 53  1  0  0  0  0
 50 54  2  0  0  0  0
 50 55  1  0  0  0  0
 52 54  1  0  0  0  0
 52 56  1  0  0  0  0
 54110  1  0  0  0  0
 57 58  2  0  0  0  0
 57 59  1  0  0  0  0
 57100  1  0  0  0  0
 58 60  1  0  0  0  0
 58 61  1  0  0  0  0
 59 62  2  0  0  0  0
 59 63  1  0  0  0  0
 60 64  2  0  0  0  0
 60 65  1  0  0  0  0
 62 64  1  0  0  0  0
 62 66  1  0  0  0  0
 64109  1  0  0  0  0
 67 68  2  0  0  0  0
 67 69  1  0  0  0  0
 67101  1  0  0  0  0
 68 70  1  0  0  0  0
 68 71  1  0  0  0  0
 69 72  2  0  0  0  0
 69 73  1  0  0  0  0
 70 74  2  0  0  0  0
 70 75  1  0  0  0  0
 72 74  1  0  0  0  0
 72 76  1  0  0  0  0
 74111  1  0  0  0  0
 77 78  2  0  0  0  0
 77 79  1  0  0  0  0
 77103  1  0  0  0  0
 78 80  1  0  0  0  0
```

```
 78 81  1  0  0  0  0
 79 82  2  0  0  0  0
 79 83  1  0  0  0  0
 80 84  2  0  0  0  0
 80 85  1  0  0  0  0
 82 84  1  0  0  0  0
 82 86  1  0  0  0  0
 84115  1  0  0  0  0
 87 88  2  0  0  0  0
 87 89  1  0  0  0  0
 87102  1  0  0  0  0
 88 90  1  0  0  0  0
 88 91  1  0  0  0  0
 89 92  2  0  0  0  0
 89 93  1  0  0  0  0
 90 94  2  0  0  0  0
 90 95  1  0  0  0  0
 92 94  1  0  0  0  0
 92 96  1  0  0  0  0
 94112  1  0  0  0  0
 97 98  1  0  0  0  0
 97 99  1  0  0  0  0
M  END
$$$$
mol048
Optimized Catalyst 048
Optimized Catalyst 048
139148  0  0  0  0  0  0  0  0999 V2000
    3.7902    0.6771   -0.4828 C   0  0  0  0  0  0  0  0  0  0  0  0
    3.6156   -0.7611   -0.9267 C   0  0  0  0  0  0  0  0  0  0  0  0
    2.0885   -1.4813   -0.0873 P   0  0  0  0  0  0  0  0  0  0  0  0
   -1.2640    1.6802   -1.2067 P   0  0  0  0  0  0  0  0  0  0  0  0
   -2.7525    1.0318   -0.2332 C   0  0  0  0  0  0  0  0  0  0  0  0
   -2.9774   -0.4298   -0.5514 C   0  0  0  0  0  0  0  0  0  0  0  0
   -1.3835   -1.4230   -0.3194 P   0  0  0  0  0  0  0  0  0  0  0  0
    2.2017    1.6416   -0.8211 P   0  0  0  0  0  0  0  0  0  0  0  0
   -3.6635    1.6262   -0.4746 H   0  0  0  0  0  0  0  0  0  0  0  0
   -3.2935   -0.5825   -1.6179 H   0  0  0  0  0  0  0  0  0  0  0  0
    3.4402   -0.8257   -2.0386 H   0  0  0  0  0  0  0  0  0  0  0  0
    4.6207    1.1586   -1.0608 H   0  0  0  0  0  0  0  0  0  0  0  0
   -2.5023    1.1729    0.8588 H   0  0  0  0  0  0  0  0  0  0  0  0
   -3.7821   -0.8429    0.1060 H   0  0  0  0  0  0  0  0  0  0  0  0
    4.5171   -1.3638   -0.6615 H   0  0  0  0  0  0  0  0  0  0  0  0
    4.0328    0.7369    0.6103 H   0  0  0  0  0  0  0  0  0  0  0  0
    1.6504    3.1570   -2.8421 C   0  0  0  0  0  0  0  0  0  0  0  0
    1.2485    4.4064   -2.3573 C   0  0  0  0  0  0  0  0  0  0  0  0
    1.1198    2.5931   -4.0074 C   0  0  0  0  0  0  0  0  0  0  0  0
    0.2842    5.1146   -3.0773 C   0  0  0  0  0  0  0  0  0  0  0  0
    1.6642    4.8011   -1.4288 H   0  0  0  0  0  0  0  0  0  0  0  0
    0.1596    3.3184   -4.7108 C   0  0  0  0  0  0  0  0  0  0  0  0
    1.4346    1.5979   -4.3304 H   0  0  0  0  0  0  0  0  0  0  0  0
   -0.2614    4.5818   -4.2561 C   0  0  0  0  0  0  0  0  0  0  0  0
   -0.0502    6.0809   -2.7054 H   0  0  0  0  0  0  0  0  0  0  0  0
   -0.2840    2.8865   -5.6066 H   0  0  0  0  0  0  0  0  0  0  0  0
    3.3621    3.1742    1.0027 C   0  0  0  0  0  0  0  0  0  0  0  0
    3.2283    2.6836    2.3098 C   0  0  0  0  0  0  0  0  0  0  0  0
    4.5086    3.8543    0.5735 C   0  0  0  0  0  0  0  0  0  0  0  0
    4.2835    2.8721    3.1983 C   0  0  0  0  0  0  0  0  0  0  0  0
    2.3077    2.1729    2.6104 H   0  0  0  0  0  0  0  0  0  0  0  0
    5.5538    4.0332    1.4816 C   0  0  0  0  0  0  0  0  0  0  0  0
    4.5656    4.2372   -0.4448 H   0  0  0  0  0  0  0  0  0  0  0  0
    5.4549    3.5389    2.7921 C   0  0  0  0  0  0  0  0  0  0  0  0
    4.1983    2.4984    4.2181 H   0  0  0  0  0  0  0  0  0  0  0  0
    6.4501    4.5639    1.1637 H   0  0  0  0  0  0  0  0  0  0  0  0
    1.7943   -2.2447    2.3695 C   0  0  0  0  0  0  0  0  0  0  0  0
```

```
    1.1834   -3.5025    2.2968 C   0  0  0  0  0  0  0  0  0  0  0  0
    1.5601   -1.3535    3.4224 C   0  0  0  0  0  0  0  0  0  0  0  0
    0.2853   -3.8583    3.3025 C   0  0  0  0  0  0  0  0  0  0  0  0
    1.3868   -4.1729    1.4594 H   0  0  0  0  0  0  0  0  0  0  0  0
    0.6608   -1.7328    4.4206 C   0  0  0  0  0  0  0  0  0  0  0  0
    2.0578   -0.3847    3.4392 H   0  0  0  0  0  0  0  0  0  0  0  0
    0.0105   -2.9766    4.3628 C   0  0  0  0  0  0  0  0  0  0  0  0
   -0.2217   -4.8197    3.2511 H   0  0  0  0  0  0  0  0  0  0  0  0
    0.4497   -1.0420    5.2341 H   0  0  0  0  0  0  0  0  0  0  0  0
    3.0079   -3.6488   -1.2882 C   0  0  0  0  0  0  0  0  0  0  0  0
    3.0349   -3.5001   -2.6830 C   0  0  0  0  0  0  0  0  0  0  0  0
    3.9333   -4.4436   -0.6027 C   0  0  0  0  0  0  0  0  0  0  0  0
    4.0115   -4.1832   -3.4014 C   0  0  0  0  0  0  0  0  0  0  0  0
    2.2799   -2.8826   -3.1836 H   0  0  0  0  0  0  0  0  0  0  0  0
    4.9022   -5.1236   -1.3443 C   0  0  0  0  0  0  0  0  0  0  0  0
    3.8809   -4.5302    0.4816 H   0  0  0  0  0  0  0  0  0  0  0  0
    4.9475   -5.0029   -2.7416 C   0  0  0  0  0  0  0  0  0  0  0  0
    4.0443   -4.0864   -4.4860 H   0  0  0  0  0  0  0  0  0  0  0  0
    5.6231   -5.7536   -0.8257 H   0  0  0  0  0  0  0  0  0  0  0  0
   -2.6502   -2.2316    1.8641 C   0  0  0  0  0  0  0  0  0  0  0  0
   -2.6437   -1.3274    2.9340 C   0  0  0  0  0  0  0  0  0  0  0  0
   -3.7297   -3.0919    1.6183 C   0  0  0  0  0  0  0  0  0  0  0  0
   -3.7522   -1.2894    3.7782 C   0  0  0  0  0  0  0  0  0  0  0  0
   -1.7695   -0.6858    3.0968 H   0  0  0  0  0  0  0  0  0  0  0  0
   -4.8285   -3.0366    2.4752 C   0  0  0  0  0  0  0  0  0  0  0  0
   -3.6954   -3.7875    0.7804 H   0  0  0  0  0  0  0  0  0  0  0  0
   -4.8501   -2.1383    3.5568 C   0  0  0  0  0  0  0  0  0  0  0  0
   -3.7577   -0.5934    4.6159 H   0  0  0  0  0  0  0  0  0  0  0  0
   -5.6757   -3.6991    2.3038 H   0  0  0  0  0  0  0  0  0  0  0  0
   -0.9836   -3.7186   -1.5723 C   0  0  0  0  0  0  0  0  0  0  0  0
   -0.5112   -3.8165   -2.8869 C   0  0  0  0  0  0  0  0  0  0  0  0
   -0.6857   -4.6727   -0.5922 C   0  0  0  0  0  0  0  0  0  0  0  0
    0.2805   -4.9135   -3.2232 C   0  0  0  0  0  0  0  0  0  0  0  0
   -0.7465   -3.0321   -3.6085 H   0  0  0  0  0  0  0  0  0  0  0  0
    0.1228   -5.7538   -0.9477 C   0  0  0  0  0  0  0  0  0  0  0  0
   -1.0616   -4.5583    0.4258 H   0  0  0  0  0  0  0  0  0  0  0  0
    0.6107   -5.8828   -2.2586 C   0  0  0  0  0  0  0  0  0  0  0  0
    0.6615   -5.0069   -4.2385 H   0  0  0  0  0  0  0  0  0  0  0  0
    0.3852   -6.4935   -0.1945 H   0  0  0  0  0  0  0  0  0  0  0  0
   -1.5409    3.7393    0.4128 C   0  0  0  0  0  0  0  0  0  0  0  0
   -2.8290    4.2021    0.7091 C   0  0  0  0  0  0  0  0  0  0  0  0
   -0.5034    3.7670    1.3529 C   0  0  0  0  0  0  0  0  0  0  0  0
   -3.0848    4.6822    1.9955 C   0  0  0  0  0  0  0  0  0  0  0  0
   -3.6058    4.1977   -0.0518 H   0  0  0  0  0  0  0  0  0  0  0  0
   -0.7809    4.2460    2.6312 C   0  0  0  0  0  0  0  0  0  0  0  0
    0.5012    3.4170    1.0870 H   0  0  0  0  0  0  0  0  0  0  0  0
   -2.0704    4.6998    2.9652 C   0  0  0  0  0  0  0  0  0  0  0  0
   -4.0818    5.0424    2.2397 H   0  0  0  0  0  0  0  0  0  0  0  0
    0.0127    4.2549    3.3777 H   0  0  0  0  0  0  0  0  0  0  0  0
   -3.0377    2.4021   -3.0172 C   0  0  0  0  0  0  0  0  0  0  0  0
   -3.9737    1.5197   -3.5781 C   0  0  0  0  0  0  0  0  0  0  0  0
   -3.3292    3.7553   -2.8088 C   0  0  0  0  0  0  0  0  0  0  0  0
   -5.2366    2.0069   -3.9063 C   0  0  0  0  0  0  0  0  0  0  0  0
   -3.6920    0.4849   -3.7688 H   0  0  0  0  0  0  0  0  0  0  0  0
   -4.6018    4.2224   -3.1388 C   0  0  0  0  0  0  0  0  0  0  0  0
   -2.5609    4.4257   -2.4082 H   0  0  0  0  0  0  0  0  0  0  0  0
   -5.5649    3.3566   -3.6810 C   0  0  0  0  0  0  0  0  0  0  0  0
   -5.9733    1.3352   -4.3450 H   0  0  0  0  0  0  0  0  0  0  0  0
   -4.8397    5.2727   -2.9777 H   0  0  0  0  0  0  0  0  0  0  0  0
    0.4095    0.1083   -0.6106 Os  0  0  0  0  0  0  0  0  0  0  0  0
    0.0910    0.8481    2.0103 Cl  0  0  0  0  0  0  0  0  0  0  0  0
    0.6983   -0.6894   -3.2170 Cl  0  0  0  0  0  0  0  0  0  0  0  0
   -1.4998   -2.3626    1.0992 O   0  0  0  0  0  0  0  0  0  0  0  0
   -1.8993   -2.6966   -1.3076 O   0  0  0  0  0  0  0  0  0  0  0  0
   -1.7582    1.8908   -2.8114 O   0  0  0  0  0  0  0  0  0  0  0  0
```

```
   -1.2861    3.3554   -0.9064 O   0  0  0  0  0  0  0  0  0  0  0  0
    2.7007    2.4722   -2.2012 O   0  0  0  0  0  0  0  0  0  0  0  0
    2.2871    3.0707    0.1314 O   0  0  0  0  0  0  0  0  0  0  0  0
    1.9498   -3.1067   -0.5673 O   0  0  0  0  0  0  0  0  0  0  0  0
    2.7604   -1.8739    1.4170 O   0  0  0  0  0  0  0  0  0  0  0  0
   -6.9306    3.8555   -4.0372 C   0  0  0  0  0  0  0  0  0  0  0  0
   -7.7082    3.2041   -3.5736 H   0  0  0  0  0  0  0  0  0  0  0  0
   -7.0892    4.8990   -3.6825 H   0  0  0  0  0  0  0  0  0  0  0  0
   -7.0544    3.8344   -5.1467 H   0  0  0  0  0  0  0  0  0  0  0  0
   -1.2940    5.3387   -5.0337 C   0  0  0  0  0  0  0  0  0  0  0  0
   -0.8720    5.6323   -6.0233 H   0  0  0  0  0  0  0  0  0  0  0  0
   -2.1890    4.6900   -5.1971 H   0  0  0  0  0  0  0  0  0  0  0  0
   -1.6183    6.2565   -4.4950 H   0  0  0  0  0  0  0  0  0  0  0  0
   -2.3432    5.1912    4.3532 C   0  0  0  0  0  0  0  0  0  0  0  0
   -1.6160    5.9940    4.6198 H   0  0  0  0  0  0  0  0  0  0  0  0
   -3.3735    5.6015    4.4464 H   0  0  0  0  0  0  0  0  0  0  0  0
   -2.2284    4.3448    5.0719 H   0  0  0  0  0  0  0  0  0  0  0  0
    1.5180   -7.0178   -2.6202 C   0  0  0  0  0  0  0  0  0  0  0  0
    2.5767   -6.6739   -2.5018 H   0  0  0  0  0  0  0  0  0  0  0  0
    1.3484   -7.8961   -1.9584 H   0  0  0  0  0  0  0  0  0  0  0  0
    1.3575   -7.3333   -3.6756 H   0  0  0  0  0  0  0  0  0  0  0  0
    5.9726   -5.7428   -3.5433 C   0  0  0  0  0  0  0  0  0  0  0  0
    5.4623   -6.4578   -4.2317 H   0  0  0  0  0  0  0  0  0  0  0  0
    6.5711   -5.0213   -4.1486 H   0  0  0  0  0  0  0  0  0  0  0  0
    6.6675   -6.3164   -2.8894 H   0  0  0  0  0  0  0  0  0  0  0  0
   -0.9998   -3.3600    5.3996 C   0  0  0  0  0  0  0  0  0  0  0  0
   -2.0191   -3.3072    4.9408 H   0  0  0  0  0  0  0  0  0  0  0  0
   -0.8140   -4.3984    5.7563 H   0  0  0  0  0  0  0  0  0  0  0  0
   -0.9683   -2.6724    6.2735 H   0  0  0  0  0  0  0  0  0  0  0  0
   -6.0406   -2.1029    4.4641 C   0  0  0  0  0  0  0  0  0  0  0  0
   -6.1282   -3.0770    5.0019 H   0  0  0  0  0  0  0  0  0  0  0  0
   -5.9532   -1.2886    5.2186 H   0  0  0  0  0  0  0  0  0  0  0  0
   -6.9680   -1.9331    3.8679 H   0  0  0  0  0  0  0  0  0  0  0  0
    6.5771    3.7176    3.7666 C   0  0  0  0  0  0  0  0  0  0  0  0
    6.2644    4.4337    4.5639 H   0  0  0  0  0  0  0  0  0  0  0  0
    6.8313    2.7389    4.2382 H   0  0  0  0  0  0  0  0  0  0  0  0
    7.4904    4.1163    3.2699 H   0  0  0  0  0  0  0  0  0  0  0  0
  1  2  1  0  0  0  0
  1  8  1  0  0  0  0
  1 12  1  0  0  0  0
  1 16  1  0  0  0  0
  2  3  1  0  0  0  0
  2 11  1  0  0  0  0
  2 15  1  0  0  0  0
  3 97  1  0  0  0  0
  3106  1  0  0  0  0
  3107  1  0  0  0  0
  4  5  1  0  0  0  0
  4 97  1  0  0  0  0
  4102  1  0  0  0  0
  4103  1  0  0  0  0
  5  6  1  0  0  0  0
  5  9  1  0  0  0  0
  5 13  1  0  0  0  0
  6  7  1  0  0  0  0
  6 10  1  0  0  0  0
  6 14  1  0  0  0  0
  7 97  1  0  0  0  0
  7100  1  0  0  0  0
  7101  1  0  0  0  0
  8 97  1  0  0  0  0
  8104  1  0  0  0  0
  8105  1  0  0  0  0
 17 18  2  0  0  0  0
 17 19  1  0  0  0  0
```

```
17104  1  0  0  0  0
18 20  1  0  0  0  0
18 21  1  0  0  0  0
19 22  2  0  0  0  0
19 23  1  0  0  0  0
20 24  2  0  0  0  0
20 25  1  0  0  0  0
22 24  1  0  0  0  0
22 26  1  0  0  0  0
24112  1  0  0  0  0
27 28  2  0  0  0  0
27 29  1  0  0  0  0
27105  1  0  0  0  0
28 30  1  0  0  0  0
28 31  1  0  0  0  0
29 32  2  0  0  0  0
29 33  1  0  0  0  0
30 34  2  0  0  0  0
30 35  1  0  0  0  0
32 34  1  0  0  0  0
32 36  1  0  0  0  0
34136  1  0  0  0  0
37 38  2  0  0  0  0
37 39  1  0  0  0  0
37107  1  0  0  0  0
38 40  1  0  0  0  0
38 41  1  0  0  0  0
39 42  2  0  0  0  0
39 43  1  0  0  0  0
40 44  2  0  0  0  0
40 45  1  0  0  0  0
42 44  1  0  0  0  0
42 46  1  0  0  0  0
44128  1  0  0  0  0
47 48  2  0  0  0  0
47 49  1  0  0  0  0
47106  1  0  0  0  0
48 50  1  0  0  0  0
48 51  1  0  0  0  0
49 52  2  0  0  0  0
49 53  1  0  0  0  0
50 54  2  0  0  0  0
50 55  1  0  0  0  0
52 54  1  0  0  0  0
52 56  1  0  0  0  0
54124  1  0  0  0  0
57 58  2  0  0  0  0
57 59  1  0  0  0  0
57100  1  0  0  0  0
58 60  1  0  0  0  0
58 61  1  0  0  0  0
59 62  2  0  0  0  0
59 63  1  0  0  0  0
60 64  2  0  0  0  0
60 65  1  0  0  0  0
62 64  1  0  0  0  0
62 66  1  0  0  0  0
64132  1  0  0  0  0
67 68  2  0  0  0  0
67 69  1  0  0  0  0
67101  1  0  0  0  0
68 70  1  0  0  0  0
68 71  1  0  0  0  0
69 72  2  0  0  0  0
69 73  1  0  0  0  0
```

```
 70 74  2  0  0  0  0
 70 75  1  0  0  0  0
 72 74  1  0  0  0  0
 72 76  1  0  0  0  0
 74120  1  0  0  0  0
 77 78  2  0  0  0  0
 77 79  1  0  0  0  0
 77103  1  0  0  0  0
 78 80  1  0  0  0  0
 78 81  1  0  0  0  0
 79 82  2  0  0  0  0
 79 83  1  0  0  0  0
 80 84  2  0  0  0  0
 80 85  1  0  0  0  0
 82 84  1  0  0  0  0
 82 86  1  0  0  0  0
 84116  1  0  0  0  0
 87 88  2  0  0  0  0
 87 89  1  0  0  0  0
 87102  1  0  0  0  0
 88 90  1  0  0  0  0
 88 91  1  0  0  0  0
 89 92  2  0  0  0  0
 89 93  1  0  0  0  0
 90 94  2  0  0  0  0
 90 95  1  0  0  0  0
 92 94  1  0  0  0  0
 92 96  1  0  0  0  0
 94108  1  0  0  0  0
 97 98  1  0  0  0  0
 97 99  1  0  0  0  0
108109  1  0  0  0  0
108110  1  0  0  0  0
108111  1  0  0  0  0
112113  1  0  0  0  0
112114  1  0  0  0  0
112115  1  0  0  0  0
116117  1  0  0  0  0
116118  1  0  0  0  0
116119  1  0  0  0  0
120121  1  0  0  0  0
120122  1  0  0  0  0
120123  1  0  0  0  0
124125  1  0  0  0  0
124126  1  0  0  0  0
124127  1  0  0  0  0
128129  1  0  0  0  0
128130  1  0  0  0  0
128131  1  0  0  0  0
132133  1  0  0  0  0
132134  1  0  0  0  0
132135  1  0  0  0  0
136137  1  0  0  0  0
136138  1  0  0  0  0
136139  1  0  0  0  0
M  END
$$$$
mol049
Optimized Catalyst 049
Optimized Catalyst 049
107116  0  0  0  0  0  0  0  0999 V2000
    3.4695    0.6367   -0.2691 C   0  0  0  0  0  0  0  0  0  0  0  0
    3.2802   -0.8303   -0.5985 C   0  0  0  0  0  0  0  0  0  0  0  0
    1.7134   -1.6259    0.1714 P   0  0  0  0  0  0  0  0  0  0  0  0
   -1.7000    1.6229   -0.3978 P   0  0  0  0  0  0  0  0  0  0  0  0
```

```
   -3.1873    0.8688    0.5328 C   0  0  0  0  0  0  0  0  0  0  0  0
   -3.4516   -0.5138   -0.0323 C   0  0  0  0  0  0  0  0  0  0  0  0
   -1.8189   -1.5252    0.0380 P   0  0  0  0  0  0  0  0  0  0  0  0
    1.7897    1.5539   -0.0936 P   0  0  0  0  0  0  0  0  0  0  0  0
   -4.0777    1.5242    0.4459 H   0  0  0  0  0  0  0  0  0  0  0  0
   -3.7956   -0.4750   -1.0913 H   0  0  0  0  0  0  0  0  0  0  0  0
    3.1185   -0.9476   -1.7071 H   0  0  0  0  0  0  0  0  0  0  0  0
    4.0542    1.1315   -1.0786 H   0  0  0  0  0  0  0  0  0  0  0  0
   -2.8825    0.8064    1.6100 H   0  0  0  0  0  0  0  0  0  0  0  0
   -4.2199   -1.0378    0.5789 H   0  0  0  0  0  0  0  0  0  0  0  0
    4.1717   -1.4183   -0.2915 H   0  0  0  0  0  0  0  0  0  0  0  0
    4.0222    0.7665    0.6892 H   0  0  0  0  0  0  0  0  0  0  0  0
    1.8092    2.8241   -1.4434 C   0  0  0  0  0  0  0  0  0  0  0  0
    1.3188    4.1107   -1.2233 C   0  0  0  0  0  0  0  0  0  0  0  0
    2.1847    2.4087   -2.7227 C   0  0  0  0  0  0  0  0  0  0  0  0
    1.2391    5.0105   -2.2927 C   0  0  0  0  0  0  0  0  0  0  0  0
    0.9713    4.4347   -0.2378 H   0  0  0  0  0  0  0  0  0  0  0  0
    2.1066    3.3141   -3.7891 C   0  0  0  0  0  0  0  0  0  0  0  0
    2.4971    1.3779   -2.9287 H   0  0  0  0  0  0  0  0  0  0  0  0
    1.6390    4.6138   -3.5728 C   0  0  0  0  0  0  0  0  0  0  0  0
    0.8549    6.0146   -2.1263 H   0  0  0  0  0  0  0  0  0  0  0  0
    2.3931    2.9944   -4.7894 H   0  0  0  0  0  0  0  0  0  0  0  0
    1.5762    5.3144   -4.4040 H   0  0  0  0  0  0  0  0  0  0  0  0
    2.1366    2.4586    1.4963 C   0  0  0  0  0  0  0  0  0  0  0  0
    1.7043    1.8070    2.6511 C   0  0  0  0  0  0  0  0  0  0  0  0
    2.8336    3.6633    1.5657 C   0  0  0  0  0  0  0  0  0  0  0  0
    1.9479    2.3861    3.9020 C   0  0  0  0  0  0  0  0  0  0  0  0
    1.1703    0.8413    2.5804 H   0  0  0  0  0  0  0  0  0  0  0  0
    3.0680    4.2440    2.8186 C   0  0  0  0  0  0  0  0  0  0  0  0
    3.2088    4.1657    0.6723 H   0  0  0  0  0  0  0  0  0  0  0  0
    2.6232    3.6079    3.9827 C   0  0  0  0  0  0  0  0  0  0  0  0
    1.6113    1.8852    4.8070 H   0  0  0  0  0  0  0  0  0  0  0  0
    3.6001    5.1907    2.8864 H   0  0  0  0  0  0  0  0  0  0  0  0
    2.8090    4.0634    4.9541 H   0  0  0  0  0  0  0  0  0  0  0  0
    2.1275   -2.0986    1.9281 C   0  0  0  0  0  0  0  0  0  0  0  0
    1.5252   -3.2457    2.4537 C   0  0  0  0  0  0  0  0  0  0  0  0
    2.9589   -1.3090    2.7177 C   0  0  0  0  0  0  0  0  0  0  0  0
    1.7835   -3.6182    3.7772 C   0  0  0  0  0  0  0  0  0  0  0  0
    0.8425   -3.8592    1.8572 H   0  0  0  0  0  0  0  0  0  0  0  0
    3.2050   -1.6777    4.0478 C   0  0  0  0  0  0  0  0  0  0  0  0
    3.4262   -0.4017    2.3364 H   0  0  0  0  0  0  0  0  0  0  0  0
    2.6244   -2.8341    4.5745 C   0  0  0  0  0  0  0  0  0  0  0  0
    1.3210   -4.5144    4.1870 H   0  0  0  0  0  0  0  0  0  0  0  0
    3.8506   -1.0592    4.6685 H   0  0  0  0  0  0  0  0  0  0  0  0
    2.8248   -3.1251    5.6042 H   0  0  0  0  0  0  0  0  0  0  0  0
    1.7989   -3.2710   -0.7035 C   0  0  0  0  0  0  0  0  0  0  0  0
    1.0524   -3.4444   -1.8631 C   0  0  0  0  0  0  0  0  0  0  0  0
    2.6564   -4.2785   -0.2493 C   0  0  0  0  0  0  0  0  0  0  0  0
    1.1359   -4.6570   -2.5659 C   0  0  0  0  0  0  0  0  0  0  0  0
    0.3976   -2.6490   -2.2553 H   0  0  0  0  0  0  0  0  0  0  0  0
    2.7335   -5.4862   -0.9471 C   0  0  0  0  0  0  0  0  0  0  0  0
    3.2752   -4.1477    0.6406 H   0  0  0  0  0  0  0  0  0  0  0  0
    1.9674   -5.6769   -2.1056 C   0  0  0  0  0  0  0  0  0  0  0  0
    0.5420   -4.7906   -3.4695 H   0  0  0  0  0  0  0  0  0  0  0  0
    3.3913   -6.2777   -0.5960 H   0  0  0  0  0  0  0  0  0  0  0  0
    2.0283   -6.6196   -2.6467 H   0  0  0  0  0  0  0  0  0  0  0  0
   -2.0617   -2.4436    1.6367 C   0  0  0  0  0  0  0  0  0  0  0  0
   -1.2286   -2.0728    2.6871 C   0  0  0  0  0  0  0  0  0  0  0  0
   -3.0747   -3.3876    1.8175 C   0  0  0  0  0  0  0  0  0  0  0  0
   -1.3876   -2.6797    3.9415 C   0  0  0  0  0  0  0  0  0  0  0  0
   -0.4396   -1.3114    2.5343 H   0  0  0  0  0  0  0  0  0  0  0  0
   -3.2246   -3.9975    3.0670 C   0  0  0  0  0  0  0  0  0  0  0  0
   -3.7577   -3.6617    1.0115 H   0  0  0  0  0  0  0  0  0  0  0  0
   -2.3781   -3.6454    4.1268 C   0  0  0  0  0  0  0  0  0  0  0  0
   -0.7306   -2.3993    4.7630 H   0  0  0  0  0  0  0  0  0  0  0  0
```

```
   -4.0017   -4.7433    3.2189 H   0  0  0  0  0  0  0  0  0  0  0  0
   -2.4981   -4.1250    5.0971 H   0  0  0  0  0  0  0  0  0  0  0  0
   -2.0632   -2.7654   -1.3185 C   0  0  0  0  0  0  0  0  0  0  0  0
   -2.2203   -2.2405   -2.6039 C   0  0  0  0  0  0  0  0  0  0  0  0
   -2.0038   -4.1418   -1.1071 C   0  0  0  0  0  0  0  0  0  0  0  0
   -2.3833   -3.1154   -3.6855 C   0  0  0  0  0  0  0  0  0  0  0  0
   -2.1777   -1.1590   -2.7995 H   0  0  0  0  0  0  0  0  0  0  0  0
   -2.1758   -5.0119   -2.1907 C   0  0  0  0  0  0  0  0  0  0  0  0
   -1.7994   -4.5655   -0.1223 H   0  0  0  0  0  0  0  0  0  0  0  0
   -2.3737   -4.4977   -3.4767 C   0  0  0  0  0  0  0  0  0  0  0  0
   -2.4989   -2.7126   -4.6903 H   0  0  0  0  0  0  0  0  0  0  0  0
   -2.1386   -6.0872   -2.0339 H   0  0  0  0  0  0  0  0  0  0  0  0
   -2.5060   -5.1755   -4.3181 H   0  0  0  0  0  0  0  0  0  0  0  0
   -1.5147    3.3025    0.3746 C   0  0  0  0  0  0  0  0  0  0  0  0
   -1.8114    4.4746   -0.3196 C   0  0  0  0  0  0  0  0  0  0  0  0
   -1.0137    3.3427    1.6752 C   0  0  0  0  0  0  0  0  0  0  0  0
   -1.5939    5.7115    0.3008 C   0  0  0  0  0  0  0  0  0  0  0  0
   -2.2002    4.4555   -1.3410 H   0  0  0  0  0  0  0  0  0  0  0  0
   -0.7840    4.5815    2.2867 C   0  0  0  0  0  0  0  0  0  0  0  0
   -0.7783    2.4193    2.2173 H   0  0  0  0  0  0  0  0  0  0  0  0
   -1.0747    5.7635    1.5989 C   0  0  0  0  0  0  0  0  0  0  0  0
   -1.8257    6.6320   -0.2305 H   0  0  0  0  0  0  0  0  0  0  0  0
   -0.3658    4.6191    3.2912 H   0  0  0  0  0  0  0  0  0  0  0  0
   -0.8959    6.7268    2.0741 H   0  0  0  0  0  0  0  0  0  0  0  0
   -2.3281    2.0104   -2.1053 C   0  0  0  0  0  0  0  0  0  0  0  0
   -3.6849    2.0629   -2.4289 C   0  0  0  0  0  0  0  0  0  0  0  0
   -1.3563    2.2080   -3.0851 C   0  0  0  0  0  0  0  0  0  0  0  0
   -4.0708    2.3085   -3.7507 C   0  0  0  0  0  0  0  0  0  0  0  0
   -4.4642    1.9250   -1.6809 H   0  0  0  0  0  0  0  0  0  0  0  0
   -1.7458    2.4554   -4.4102 C   0  0  0  0  0  0  0  0  0  0  0  0
   -0.2853    2.1741   -2.8488 H   0  0  0  0  0  0  0  0  0  0  0  0
   -3.0999    2.4994   -4.7423 C   0  0  0  0  0  0  0  0  0  0  0  0
   -5.1254    2.3483   -4.0110 H   0  0  0  0  0  0  0  0  0  0  0  0
   -0.9823    2.5929   -5.1738 H   0  0  0  0  0  0  0  0  0  0  0  0
   -3.4043    2.6807   -5.7718 H   0  0  0  0  0  0  0  0  0  0  0  0
   -0.0083    0.0089    0.0394 Os  0  0  0  0  0  0  0  0  0  0  0  0
    0.3416   -0.2927   -2.8805 Cl  0  0  0  0  0  0  0  0  0  0  0  0
   -0.2098    0.2303    1.5981 H   0  0  0  0  0  0  0  0  0  0  0  0
  1  2  1  0  0  0  0
  1  8  1  0  0  0  0
  1 12  1  0  0  0  0
  1 16  1  0  0  0  0
  2  3  1  0  0  0  0
  2 11  1  0  0  0  0
  2 15  1  0  0  0  0
  3 39  1  0  0  0  0
  3 50  1  0  0  0  0
  3105  1  0  0  0  0
  4  5  1  0  0  0  0
  4 83  1  0  0  0  0
  4 94  1  0  0  0  0
  4105  1  0  0  0  0
  5  6  1  0  0  0  0
  5  9  1  0  0  0  0
  5 13  1  0  0  0  0
  6  7  1  0  0  0  0
  6 10  1  0  0  0  0
  6 14  1  0  0  0  0
  7 61  1  0  0  0  0
  7 72  1  0  0  0  0
  7105  1  0  0  0  0
  8 17  1  0  0  0  0
  8 28  1  0  0  0  0
  8105  1  0  0  0  0
 17 18  2  0  0  0  0
```

```
 17 19  1  0  0  0  0
 18 20  1  0  0  0  0
 18 21  1  0  0  0  0
 19 22  2  0  0  0  0
 19 23  1  0  0  0  0
 20 24  2  0  0  0  0
 20 25  1  0  0  0  0
 22 24  1  0  0  0  0
 22 26  1  0  0  0  0
 24 27  1  0  0  0  0
 28 29  2  0  0  0  0
 28 30  1  0  0  0  0
 29 31  1  0  0  0  0
 29 32  1  0  0  0  0
 30 33  2  0  0  0  0
 30 34  1  0  0  0  0
 31 35  2  0  0  0  0
 31 36  1  0  0  0  0
 33 35  1  0  0  0  0
 33 37  1  0  0  0  0
 35 38  1  0  0  0  0
 39 40  2  0  0  0  0
 39 41  1  0  0  0  0
 40 42  1  0  0  0  0
 40 43  1  0  0  0  0
 41 44  2  0  0  0  0
 41 45  1  0  0  0  0
 42 46  2  0  0  0  0
 42 47  1  0  0  0  0
 44 46  1  0  0  0  0
 44 48  1  0  0  0  0
 46 49  1  0  0  0  0
 50 51  2  0  0  0  0
 50 52  1  0  0  0  0
 51 53  1  0  0  0  0
 51 54  1  0  0  0  0
 52 55  2  0  0  0  0
 52 56  1  0  0  0  0
 53 57  2  0  0  0  0
 53 58  1  0  0  0  0
 55 57  1  0  0  0  0
 55 59  1  0  0  0  0
 57 60  1  0  0  0  0
 61 62  2  0  0  0  0
 61 63  1  0  0  0  0
 62 64  1  0  0  0  0
 62 65  1  0  0  0  0
 63 66  2  0  0  0  0
 63 67  1  0  0  0  0
 64 68  2  0  0  0  0
 64 69  1  0  0  0  0
 66 68  1  0  0  0  0
 66 70  1  0  0  0  0
 68 71  1  0  0  0  0
 72 73  2  0  0  0  0
 72 74  1  0  0  0  0
 73 75  1  0  0  0  0
 73 76  1  0  0  0  0
 74 77  2  0  0  0  0
 74 78  1  0  0  0  0
 75 79  2  0  0  0  0
 75 80  1  0  0  0  0
 77 79  1  0  0  0  0
 77 81  1  0  0  0  0
 79 82  1  0  0  0  0
```

```
 83 84  2  0  0  0  0
 83 85  1  0  0  0  0
 84 86  1  0  0  0  0
 84 87  1  0  0  0  0
 85 88  2  0  0  0  0
 85 89  1  0  0  0  0
 86 90  2  0  0  0  0
 86 91  1  0  0  0  0
 88 90  1  0  0  0  0
 88 92  1  0  0  0  0
 90 93  1  0  0  0  0
 94 95  2  0  0  0  0
 94 96  1  0  0  0  0
 95 97  1  0  0  0  0
 95 98  1  0  0  0  0
 96 99  2  0  0  0  0
 96100  1  0  0  0  0
 97101  2  0  0  0  0
 97102  1  0  0  0  0
 99101  1  0  0  0  0
 99103  1  0  0  0  0
101104  1  0  0  0  0
105107  1  0  0  0  0
105106  1  0  0  0  0
M  END
$$$$
mol050
Optimized Catalyst 050
Optimized Catalyst 050
107116  0  0  0  0  0  0  0  0999 V2000
    3.4711    0.6321   -0.2678 C   0  0  0  0  0  0  0  0  0  0  0  0
    3.2834   -0.8356   -0.5936 C   0  0  0  0  0  0  0  0  0  0  0  0
    1.7101   -1.6263    0.1644 P   0  0  0  0  0  0  0  0  0  0  0  0
   -1.6978    1.6291   -0.3908 P   0  0  0  0  0  0  0  0  0  0  0  0
   -3.1843    0.8670    0.5299 C   0  0  0  0  0  0  0  0  0  0  0  0
   -3.4472   -0.5096   -0.0507 C   0  0  0  0  0  0  0  0  0  0  0  0
   -1.8179   -1.5213    0.0277 P   0  0  0  0  0  0  0  0  0  0  0  0
    1.7929    1.5504   -0.1088 P   0  0  0  0  0  0  0  0  0  0  0  0
   -4.0761    1.5228    0.4498 H   0  0  0  0  0  0  0  0  0  0  0  0
   -3.7846   -0.4603   -1.1113 H   0  0  0  0  0  0  0  0  0  0  0  0
    3.1340   -0.9579   -1.7032 H   0  0  0  0  0  0  0  0  0  0  0  0
    4.0652    1.1233   -1.0733 H   0  0  0  0  0  0  0  0  0  0  0  0
   -2.8861    0.7930    1.6082 H   0  0  0  0  0  0  0  0  0  0  0  0
   -4.2240   -1.0368    0.5482 H   0  0  0  0  0  0  0  0  0  0  0  0
    4.1738   -1.4220   -0.2780 H   0  0  0  0  0  0  0  0  0  0  0  0
    4.0174    0.7653    0.6944 H   0  0  0  0  0  0  0  0  0  0  0  0
    1.8186    2.8016   -1.4740 C   0  0  0  0  0  0  0  0  0  0  0  0
    1.3312    4.0926   -1.2730 C   0  0  0  0  0  0  0  0  0  0  0  0
    2.1896    2.3548   -2.7466 C   0  0  0  0  0  0  0  0  0  0  0  0
    1.2502    4.9846   -2.3483 C   0  0  0  0  0  0  0  0  0  0  0  0
    0.9869    4.4330   -0.2908 H   0  0  0  0  0  0  0  0  0  0  0  0
    2.1202    3.2302   -3.8368 C   0  0  0  0  0  0  0  0  0  0  0  0
    2.5025    1.3183   -2.9301 H   0  0  0  0  0  0  0  0  0  0  0  0
    1.6553    4.5275   -3.6052 C   0  0  0  0  0  0  0  0  0  0  0  0
    0.8760    5.9966   -2.2065 H   0  0  0  0  0  0  0  0  0  0  0  0
    2.4025    2.8981   -4.8349 H   0  0  0  0  0  0  0  0  0  0  0  0
    2.1250    2.4686    1.4724 C   0  0  0  0  0  0  0  0  0  0  0  0
    1.6927    1.8065    2.6233 C   0  0  0  0  0  0  0  0  0  0  0  0
    2.8047    3.6837    1.5412 C   0  0  0  0  0  0  0  0  0  0  0  0
    1.9106    2.3786    3.8812 C   0  0  0  0  0  0  0  0  0  0  0  0
    1.1757    0.8302    2.5455 H   0  0  0  0  0  0  0  0  0  0  0  0
    3.0243    4.2825    2.7874 C   0  0  0  0  0  0  0  0  0  0  0  0
    3.1848    4.1860    0.6478 H   0  0  0  0  0  0  0  0  0  0  0  0
    2.5633    3.6135    3.9254 C   0  0  0  0  0  0  0  0  0  0  0  0
    1.5827    1.8769    4.7895 H   0  0  0  0  0  0  0  0  0  0  0  0
```

```
    3.5397    5.2385    2.8651 H   0  0  0  0  0  0  0  0  0  0  0  0
    2.1126   -2.1107    1.9194 C   0  0  0  0  0  0  0  0  0  0  0  0
    1.4869   -3.2517    2.4339 C   0  0  0  0  0  0  0  0  0  0  0  0
    2.9519   -1.3332    2.7143 C   0  0  0  0  0  0  0  0  0  0  0  0
    1.7218   -3.6450    3.7549 C   0  0  0  0  0  0  0  0  0  0  0  0
    0.8004   -3.8529    1.8273 H   0  0  0  0  0  0  0  0  0  0  0  0
    3.1939   -1.6992    4.0454 C   0  0  0  0  0  0  0  0  0  0  0  0
    3.4374   -0.4327    2.3349 H   0  0  0  0  0  0  0  0  0  0  0  0
    2.5746   -2.8528    4.5294 C   0  0  0  0  0  0  0  0  0  0  0  0
    1.2453   -4.5339    4.1664 H   0  0  0  0  0  0  0  0  0  0  0  0
    3.8467   -1.1004    4.6787 H   0  0  0  0  0  0  0  0  0  0  0  0
    1.7977   -3.2693   -0.7131 C   0  0  0  0  0  0  0  0  0  0  0  0
    1.0659   -3.4289   -1.8878 C   0  0  0  0  0  0  0  0  0  0  0  0
    2.6440   -4.2810   -0.2483 C   0  0  0  0  0  0  0  0  0  0  0  0
    1.1450   -4.6311   -2.6046 C   0  0  0  0  0  0  0  0  0  0  0  0
    0.4211   -2.6254   -2.2823 H   0  0  0  0  0  0  0  0  0  0  0  0
    2.7320   -5.4929   -0.9375 C   0  0  0  0  0  0  0  0  0  0  0  0
    3.2539   -4.1540    0.6506 H   0  0  0  0  0  0  0  0  0  0  0  0
    1.9702   -5.6360   -2.1035 C   0  0  0  0  0  0  0  0  0  0  0  0
    0.5745   -4.7639   -3.5237 H   0  0  0  0  0  0  0  0  0  0  0  0
    3.3761   -6.2951   -0.5843 H   0  0  0  0  0  0  0  0  0  0  0  0
   -2.0570   -2.4296    1.6290 C   0  0  0  0  0  0  0  0  0  0  0  0
   -1.2248   -2.0327    2.6736 C   0  0  0  0  0  0  0  0  0  0  0  0
   -3.0637   -3.3788    1.8165 C   0  0  0  0  0  0  0  0  0  0  0  0
   -1.3699   -2.6130    3.9406 C   0  0  0  0  0  0  0  0  0  0  0  0
   -0.4422   -1.2650    2.5032 H   0  0  0  0  0  0  0  0  0  0  0  0
   -3.2205   -3.9811    3.0682 C   0  0  0  0  0  0  0  0  0  0  0  0
   -3.7443   -3.6677    1.0113 H   0  0  0  0  0  0  0  0  0  0  0  0
   -2.3626   -3.5801    4.0996 C   0  0  0  0  0  0  0  0  0  0  0  0
   -0.7220   -2.3206    4.7655 H   0  0  0  0  0  0  0  0  0  0  0  0
   -3.9887   -4.7334    3.2355 H   0  0  0  0  0  0  0  0  0  0  0  0
   -2.0527   -2.7728   -1.3179 C   0  0  0  0  0  0  0  0  0  0  0  0
   -2.2239   -2.2552   -2.6070 C   0  0  0  0  0  0  0  0  0  0  0  0
   -1.9647   -4.1459   -1.0928 C   0  0  0  0  0  0  0  0  0  0  0  0
   -2.3832   -3.1289   -3.6884 C   0  0  0  0  0  0  0  0  0  0  0  0
   -2.2065   -1.1732   -2.8071 H   0  0  0  0  0  0  0  0  0  0  0  0
   -2.1267   -5.0389   -2.1586 C   0  0  0  0  0  0  0  0  0  0  0  0
   -1.7478   -4.5558   -0.1032 H   0  0  0  0  0  0  0  0  0  0  0  0
   -2.3430   -4.5014   -3.4305 C   0  0  0  0  0  0  0  0  0  0  0  0
   -2.5135   -2.7436   -4.6990 H   0  0  0  0  0  0  0  0  0  0  0  0
   -2.0668   -6.1142   -2.0024 H   0  0  0  0  0  0  0  0  0  0  0  0
   -1.4974    3.2946    0.4034 C   0  0  0  0  0  0  0  0  0  0  0  0
   -1.7518    4.4778   -0.2899 C   0  0  0  0  0  0  0  0  0  0  0  0
   -1.0290    3.3064    1.7186 C   0  0  0  0  0  0  0  0  0  0  0  0
   -1.5274    5.7103    0.3353 C   0  0  0  0  0  0  0  0  0  0  0  0
   -2.1177    4.4748   -1.3217 H   0  0  0  0  0  0  0  0  0  0  0  0
   -0.7917    4.5260    2.3626 C   0  0  0  0  0  0  0  0  0  0  0  0
   -0.8280    2.3705    2.2552 H   0  0  0  0  0  0  0  0  0  0  0  0
   -1.0484    5.6990    1.6484 C   0  0  0  0  0  0  0  0  0  0  0  0
   -1.7216    6.6447   -0.1877 H   0  0  0  0  0  0  0  0  0  0  0  0
   -0.4095    4.5545    3.3821 H   0  0  0  0  0  0  0  0  0  0  0  0
   -2.3320    2.0451   -2.0887 C   0  0  0  0  0  0  0  0  0  0  0  0
   -3.6901    2.1188   -2.3997 C   0  0  0  0  0  0  0  0  0  0  0  0
   -1.3586    2.2324   -3.0736 C   0  0  0  0  0  0  0  0  0  0  0  0
   -4.0958    2.3788   -3.7130 C   0  0  0  0  0  0  0  0  0  0  0  0
   -4.4664    1.9881   -1.6446 H   0  0  0  0  0  0  0  0  0  0  0  0
   -1.7377    2.4986   -4.3957 C   0  0  0  0  0  0  0  0  0  0  0  0
   -0.2875    2.1853   -2.8384 H   0  0  0  0  0  0  0  0  0  0  0  0
   -3.1015    2.5554   -4.6813 C   0  0  0  0  0  0  0  0  0  0  0  0
   -5.1500    2.4384   -3.9729 H   0  0  0  0  0  0  0  0  0  0  0  0
   -0.9832    2.6337   -5.1697 H   0  0  0  0  0  0  0  0  0  0  0  0
   -0.0068    0.0102    0.0222 Os  0  0  0  0  0  0  0  0  0  0  0  0
    0.2916   -0.2750   -2.8023 Cl  0  0  0  0  0  0  0  0  0  0  0  0
   -0.2057    0.2425    1.5794 H   0  0  0  0  0  0  0  0  0  0  0  0
    2.0441   -6.7922   -2.7660 F   0  0  0  0  0  0  0  0  0  0  0  0
```

```
    2.8040   -3.2160    5.7926 F   0  0  0  0  0  0  0  0  0  0  0  0
   -2.5050   -5.3444   -4.4524 F   0  0  0  0  0  0  0  0  0  0  0  0
   -2.5069   -4.1535    5.2959 F   0  0  0  0  0  0  0  0  0  0  0  0
   -3.4816    2.7996   -5.9370 F   0  0  0  0  0  0  0  0  0  0  0  0
   -0.8209    6.8691    2.2479 F   0  0  0  0  0  0  0  0  0  0  0  0
    2.7642    4.1816    5.1159 F   0  0  0  0  0  0  0  0  0  0  0  0
    1.5837    5.3691   -4.6380 F   0  0  0  0  0  0  0  0  0  0  0  0
  1  2  1  0  0  0  0
  1  8  1  0  0  0  0
  1 12  1  0  0  0  0
  1 16  1  0  0  0  0
  2  3  1  0  0  0  0
  2 11  1  0  0  0  0
  2 15  1  0  0  0  0
  3 37  1  0  0  0  0
  3 47  1  0  0  0  0
  3 97  1  0  0  0  0
  4  5  1  0  0  0  0
  4 77  1  0  0  0  0
  4 87  1  0  0  0  0
  4 97  1  0  0  0  0
  5  6  1  0  0  0  0
  5  9  1  0  0  0  0
  5 13  1  0  0  0  0
  6  7  1  0  0  0  0
  6 10  1  0  0  0  0
  6 14  1  0  0  0  0
  7 57  1  0  0  0  0
  7 67  1  0  0  0  0
  7 97  1  0  0  0  0
  8 17  1  0  0  0  0
  8 27  1  0  0  0  0
  8 97  1  0  0  0  0
 17 18  2  0  0  0  0
 17 19  1  0  0  0  0
 18 20  1  0  0  0  0
 18 21  1  0  0  0  0
 19 22  2  0  0  0  0
 19 23  1  0  0  0  0
 20 24  2  0  0  0  0
 20 25  1  0  0  0  0
 22 24  1  0  0  0  0
 22 26  1  0  0  0  0
 24107  1  0  0  0  0
 27 28  2  0  0  0  0
 27 29  1  0  0  0  0
 28 30  1  0  0  0  0
 28 31  1  0  0  0  0
 29 32  2  0  0  0  0
 29 33  1  0  0  0  0
 30 34  2  0  0  0  0
 30 35  1  0  0  0  0
 32 34  1  0  0  0  0
 32 36  1  0  0  0  0
 34106  1  0  0  0  0
 37 38  2  0  0  0  0
 37 39  1  0  0  0  0
 38 40  1  0  0  0  0
 38 41  1  0  0  0  0
 39 42  2  0  0  0  0
 39 43  1  0  0  0  0
 40 44  2  0  0  0  0
 40 45  1  0  0  0  0
 42 44  1  0  0  0  0
 42 46  1  0  0  0  0
```

```
 44101  1  0  0  0  0
 47 48  2  0  0  0  0
 47 49  1  0  0  0  0
 48 50  1  0  0  0  0
 48 51  1  0  0  0  0
 49 52  2  0  0  0  0
 49 53  1  0  0  0  0
 50 54  2  0  0  0  0
 50 55  1  0  0  0  0
 52 54  1  0  0  0  0
 52 56  1  0  0  0  0
 54100  1  0  0  0  0
 57 58  2  0  0  0  0
 57 59  1  0  0  0  0
 58 60  1  0  0  0  0
 58 61  1  0  0  0  0
 59 62  2  0  0  0  0
 59 63  1  0  0  0  0
 60 64  2  0  0  0  0
 60 65  1  0  0  0  0
 62 64  1  0  0  0  0
 62 66  1  0  0  0  0
 64103  1  0  0  0  0
 67 68  2  0  0  0  0
 67 69  1  0  0  0  0
 68 70  1  0  0  0  0
 68 71  1  0  0  0  0
 69 72  2  0  0  0  0
 69 73  1  0  0  0  0
 70 74  2  0  0  0  0
 70 75  1  0  0  0  0
 72 74  1  0  0  0  0
 72 76  1  0  0  0  0
 74102  1  0  0  0  0
 77 78  2  0  0  0  0
 77 79  1  0  0  0  0
 78 80  1  0  0  0  0
 78 81  1  0  0  0  0
 79 82  2  0  0  0  0
 79 83  1  0  0  0  0
 80 84  2  0  0  0  0
 80 85  1  0  0  0  0
 82 84  1  0  0  0  0
 82 86  1  0  0  0  0
 84105  1  0  0  0  0
 87 88  2  0  0  0  0
 87 89  1  0  0  0  0
 88 90  1  0  0  0  0
 88 91  1  0  0  0  0
 89 92  2  0  0  0  0
 89 93  1  0  0  0  0
 90 94  2  0  0  0  0
 90 95  1  0  0  0  0
 92 94  1  0  0  0  0
 92 96  1  0  0  0  0
 94104  1  0  0  0  0
 97 99  1  0  0  0  0
 97 98  1  0  0  0  0
M  END
$$$$
mol051
Optimized Catalyst 051
Optimized Catalyst 051
131140  0  0  0  0  0  0  0  0999 V2000
    3.4886    0.6247   -0.2862 C   0  0  0  0  0  0  0  0  0  0  0  0
```

```
    3.3088   -0.8439   -0.6137 C   0  0  0  0  0  0  0  0  0  0  0  0
    1.7146   -1.6339    0.1075 P   0  0  0  0  0  0  0  0  0  0  0  0
   -1.6834    1.6371   -0.4426 P   0  0  0  0  0  0  0  0  0  0  0  0
   -3.1744    0.8684    0.4738 C   0  0  0  0  0  0  0  0  0  0  0  0
   -3.4381   -0.5027   -0.1193 C   0  0  0  0  0  0  0  0  0  0  0  0
   -1.8095   -1.5193   -0.0347 P   0  0  0  0  0  0  0  0  0  0  0  0
    1.8072    1.5492   -0.1653 P   0  0  0  0  0  0  0  0  0  0  0  0
   -4.0633    1.5262    0.3963 H   0  0  0  0  0  0  0  0  0  0  0  0
   -3.7616   -0.4419   -1.1834 H   0  0  0  0  0  0  0  0  0  0  0  0
    3.1881   -0.9699   -1.7248 H   0  0  0  0  0  0  0  0  0  0  0  0
    4.0983    1.1151   -1.0789 H   0  0  0  0  0  0  0  0  0  0  0  0
   -2.8729    0.7852    1.5498 H   0  0  0  0  0  0  0  0  0  0  0  0
   -4.2185   -1.0352    0.4676 H   0  0  0  0  0  0  0  0  0  0  0  0
    4.1866   -1.4316   -0.2688 H   0  0  0  0  0  0  0  0  0  0  0  0
    4.0082    0.7591    0.6896 H   0  0  0  0  0  0  0  0  0  0  0  0
    1.8378    2.7662   -1.5578 C   0  0  0  0  0  0  0  0  0  0  0  0
    1.3205    4.0511   -1.4001 C   0  0  0  0  0  0  0  0  0  0  0  0
    2.2103    2.2992   -2.8207 C   0  0  0  0  0  0  0  0  0  0  0  0
    1.1981    4.8919   -2.5101 C   0  0  0  0  0  0  0  0  0  0  0  0
    0.9796    4.4186   -0.4270 H   0  0  0  0  0  0  0  0  0  0  0  0
    2.0940    3.1427   -3.9312 C   0  0  0  0  0  0  0  0  0  0  0  0
    2.5562    1.2708   -2.9792 H   0  0  0  0  0  0  0  0  0  0  0  0
    1.5813    4.4404   -3.7838 C   0  0  0  0  0  0  0  0  0  0  0  0
    0.7847    5.8889   -2.3858 H   0  0  0  0  0  0  0  0  0  0  0  0
    2.3733    2.7714   -4.9144 H   0  0  0  0  0  0  0  0  0  0  0  0
    2.1184    2.5005    1.4004 C   0  0  0  0  0  0  0  0  0  0  0  0
    1.6911    1.8679    2.5675 C   0  0  0  0  0  0  0  0  0  0  0  0
    2.7665    3.7328    1.4512 C   0  0  0  0  0  0  0  0  0  0  0  0
    1.8820    2.4910    3.8054 C   0  0  0  0  0  0  0  0  0  0  0  0
    1.1981    0.8796    2.5188 H   0  0  0  0  0  0  0  0  0  0  0  0
    2.9466    4.3634    2.6860 C   0  0  0  0  0  0  0  0  0  0  0  0
    3.1461    4.2207    0.5513 H   0  0  0  0  0  0  0  0  0  0  0  0
    2.4972    3.7502    3.8685 C   0  0  0  0  0  0  0  0  0  0  0  0
    1.5459    1.9967    4.7127 H   0  0  0  0  0  0  0  0  0  0  0  0
    3.4348    5.3333    2.7307 H   0  0  0  0  0  0  0  0  0  0  0  0
    2.0926   -2.1334    1.8623 C   0  0  0  0  0  0  0  0  0  0  0  0
    1.4805   -3.2871    2.3610 C   0  0  0  0  0  0  0  0  0  0  0  0
    2.9011   -1.3543    2.6847 C   0  0  0  0  0  0  0  0  0  0  0  0
    1.7103   -3.6820    3.6812 C   0  0  0  0  0  0  0  0  0  0  0  0
    0.8070   -3.8903    1.7429 H   0  0  0  0  0  0  0  0  0  0  0  0
    3.1229   -1.7414    4.0122 C   0  0  0  0  0  0  0  0  0  0  0  0
    3.3716   -0.4376    2.3283 H   0  0  0  0  0  0  0  0  0  0  0  0
    2.5375   -2.9121    4.5156 C   0  0  0  0  0  0  0  0  0  0  0  0
    1.2308   -4.5800    4.0640 H   0  0  0  0  0  0  0  0  0  0  0  0
    3.7507   -1.1260    4.6517 H   0  0  0  0  0  0  0  0  0  0  0  0
    1.8010   -3.2666   -0.7862 C   0  0  0  0  0  0  0  0  0  0  0  0
    1.0734   -3.4177   -1.9636 C   0  0  0  0  0  0  0  0  0  0  0  0
    2.6304   -4.2950   -0.3329 C   0  0  0  0  0  0  0  0  0  0  0  0
    1.1473   -4.6193   -2.6781 C   0  0  0  0  0  0  0  0  0  0  0  0
    0.4354   -2.6084   -2.3563 H   0  0  0  0  0  0  0  0  0  0  0  0
    2.6969   -5.5000   -1.0367 C   0  0  0  0  0  0  0  0  0  0  0  0
    3.2390   -4.1857    0.5678 H   0  0  0  0  0  0  0  0  0  0  0  0
    1.9462   -5.6721   -2.2128 C   0  0  0  0  0  0  0  0  0  0  0  0
    0.5694   -4.7277   -3.5942 H   0  0  0  0  0  0  0  0  0  0  0  0
    3.3341   -6.3010   -0.6736 H   0  0  0  0  0  0  0  0  0  0  0  0
   -2.0465   -2.4207    1.5707 C   0  0  0  0  0  0  0  0  0  0  0  0
   -1.2131   -2.0302    2.6146 C   0  0  0  0  0  0  0  0  0  0  0  0
   -3.0432   -3.3768    1.7709 C   0  0  0  0  0  0  0  0  0  0  0  0
   -1.3521   -2.6243    3.8752 C   0  0  0  0  0  0  0  0  0  0  0  0
   -0.4312   -1.2623    2.4485 H   0  0  0  0  0  0  0  0  0  0  0  0
   -3.1748   -3.9808    3.0234 C   0  0  0  0  0  0  0  0  0  0  0  0
   -3.7292   -3.6680    0.9731 H   0  0  0  0  0  0  0  0  0  0  0  0
   -2.3253   -3.6104    4.0824 C   0  0  0  0  0  0  0  0  0  0  0  0
   -0.6870   -2.3260    4.6824 H   0  0  0  0  0  0  0  0  0  0  0  0
   -3.9366   -4.7395    3.1806 H   0  0  0  0  0  0  0  0  0  0  0  0
```

```
   -2.0448   -2.7703   -1.3790 C   0  0  0  0  0  0  0  0  0  0  0  0
   -2.2023   -2.2619   -2.6727 C   0  0  0  0  0  0  0  0  0  0  0  0
   -1.9662   -4.1430   -1.1600 C   0  0  0  0  0  0  0  0  0  0  0  0
   -2.3502   -3.1447   -3.7455 C   0  0  0  0  0  0  0  0  0  0  0  0
   -2.1761   -1.1814   -2.8776 H   0  0  0  0  0  0  0  0  0  0  0  0
   -2.1196   -5.0281   -2.2338 C   0  0  0  0  0  0  0  0  0  0  0  0
   -1.7613   -4.5572   -0.1705 H   0  0  0  0  0  0  0  0  0  0  0  0
   -2.3185   -4.5328   -3.5311 C   0  0  0  0  0  0  0  0  0  0  0  0
   -2.4652   -2.7496   -4.7527 H   0  0  0  0  0  0  0  0  0  0  0  0
   -2.0609   -6.0985   -2.0589 H   0  0  0  0  0  0  0  0  0  0  0  0
   -1.4814    3.2984    0.3608 C   0  0  0  0  0  0  0  0  0  0  0  0
   -1.6861    4.4883   -0.3370 C   0  0  0  0  0  0  0  0  0  0  0  0
   -1.0436    3.3153    1.6842 C   0  0  0  0  0  0  0  0  0  0  0  0
   -1.4412    5.7110    0.2959 C   0  0  0  0  0  0  0  0  0  0  0  0
   -2.0219    4.4924   -1.3780 H   0  0  0  0  0  0  0  0  0  0  0  0
   -0.7951    4.5378    2.3180 C   0  0  0  0  0  0  0  0  0  0  0  0
   -0.8694    2.3825    2.2329 H   0  0  0  0  0  0  0  0  0  0  0  0
   -0.9883    5.7423    1.6260 C   0  0  0  0  0  0  0  0  0  0  0  0
   -1.5939    6.6394   -0.2476 H   0  0  0  0  0  0  0  0  0  0  0  0
   -0.4275    4.5439    3.3416 H   0  0  0  0  0  0  0  0  0  0  0  0
   -2.3222    2.0676   -2.1343 C   0  0  0  0  0  0  0  0  0  0  0  0
   -3.6780    2.1824   -2.4378 C   0  0  0  0  0  0  0  0  0  0  0  0
   -1.3615    2.2410   -3.1316 C   0  0  0  0  0  0  0  0  0  0  0  0
   -4.0788    2.4662   -3.7468 C   0  0  0  0  0  0  0  0  0  0  0  0
   -4.4518    2.0644   -1.6796 H   0  0  0  0  0  0  0  0  0  0  0  0
   -1.7588    2.5304   -4.4425 C   0  0  0  0  0  0  0  0  0  0  0  0
   -0.2881    2.1604   -2.9140 H   0  0  0  0  0  0  0  0  0  0  0  0
   -3.1195    2.6321   -4.7607 C   0  0  0  0  0  0  0  0  0  0  0  0
   -5.1356    2.5553   -3.9793 H   0  0  0  0  0  0  0  0  0  0  0  0
   -0.9982    2.6512   -5.2106 H   0  0  0  0  0  0  0  0  0  0  0  0
    0.0038    0.0117   -0.0280 Os  0  0  0  0  0  0  0  0  0  0  0  0
    0.3328   -0.2826   -2.9544 Cl  0  0  0  0  0  0  0  0  0  0  0  0
   -0.1806    0.2451    1.5302 H   0  0  0  0  0  0  0  0  0  0  0  0
   -2.4743   -5.4653   -4.6933 C   0  0  0  0  0  0  0  0  0  0  0  0
   -2.4885   -6.5285   -4.3662 H   0  0  0  0  0  0  0  0  0  0  0  0
   -3.4278   -5.2436   -5.2280 H   0  0  0  0  0  0  0  0  0  0  0  0
   -1.6231   -5.3186   -5.4002 H   0  0  0  0  0  0  0  0  0  0  0  0
    1.9945   -6.9613   -2.9746 C   0  0  0  0  0  0  0  0  0  0  0  0
    2.5747   -7.7379   -2.4288 H   0  0  0  0  0  0  0  0  0  0  0  0
    0.9577   -7.3391   -3.1380 H   0  0  0  0  0  0  0  0  0  0  0  0
    2.4747   -6.7862   -3.9666 H   0  0  0  0  0  0  0  0  0  0  0  0
   -2.4538   -4.2851    5.4137 C   0  0  0  0  0  0  0  0  0  0  0  0
   -1.9249   -5.2674    5.3734 H   0  0  0  0  0  0  0  0  0  0  0  0
   -2.0013   -3.6674    6.2223 H   0  0  0  0  0  0  0  0  0  0  0  0
   -3.5255   -4.4619    5.6623 H   0  0  0  0  0  0  0  0  0  0  0  0
    2.7720   -3.3286    5.9351 C   0  0  0  0  0  0  0  0  0  0  0  0
    3.7592   -2.9632    6.2997 H   0  0  0  0  0  0  0  0  0  0  0  0
    1.9675   -2.8975    6.5774 H   0  0  0  0  0  0  0  0  0  0  0  0
    2.7527   -4.4387    6.0290 H   0  0  0  0  0  0  0  0  0  0  0  0
    2.6709    4.4548    5.1791 C   0  0  0  0  0  0  0  0  0  0  0  0
    3.7200    4.8173    5.2849 H   0  0  0  0  0  0  0  0  0  0  0  0
    1.9795    5.3303    5.2164 H   0  0  0  0  0  0  0  0  0  0  0  0
    2.4422    3.7819    6.0360 H   0  0  0  0  0  0  0  0  0  0  0  0
   -0.6989    7.0571    2.2830 C   0  0  0  0  0  0  0  0  0  0  0  0
   -1.5512    7.7591    2.1296 H   0  0  0  0  0  0  0  0  0  0  0  0
   -0.5325    6.9361    3.3771 H   0  0  0  0  0  0  0  0  0  0  0  0
    0.2206    7.4976    1.8286 H   0  0  0  0  0  0  0  0  0  0  0  0
   -3.5444    2.9353   -6.1654 C   0  0  0  0  0  0  0  0  0  0  0  0
   -4.5807    2.5764   -6.3575 H   0  0  0  0  0  0  0  0  0  0  0  0
   -3.5068    4.0383   -6.3277 H   0  0  0  0  0  0  0  0  0  0  0  0
   -2.8611    2.4393   -6.8938 H   0  0  0  0  0  0  0  0  0  0  0  0
    1.4007    5.3258   -4.9780 C   0  0  0  0  0  0  0  0  0  0  0  0
    1.6403    6.3834   -4.7242 H   0  0  0  0  0  0  0  0  0  0  0  0
    2.0559    5.0058   -5.8197 H   0  0  0  0  0  0  0  0  0  0  0  0
    0.3350    5.2637   -5.3087 H   0  0  0  0  0  0  0  0  0  0  0  0
```

```
  1  2  1  0  0  0  0
  1  8  1  0  0  0  0
  1 12  1  0  0  0  0
  1 16  1  0  0  0  0
  2  3  1  0  0  0  0
  2 11  1  0  0  0  0
  2 15  1  0  0  0  0
  3 37  1  0  0  0  0
  3 47  1  0  0  0  0
  3 97  1  0  0  0  0
  4  5  1  0  0  0  0
  4 77  1  0  0  0  0
  4 87  1  0  0  0  0
  4 97  1  0  0  0  0
  5  6  1  0  0  0  0
  5  9  1  0  0  0  0
  5 13  1  0  0  0  0
  6  7  1  0  0  0  0
  6 10  1  0  0  0  0
  6 14  1  0  0  0  0
  7 57  1  0  0  0  0
  7 67  1  0  0  0  0
  7 97  1  0  0  0  0
  8 17  1  0  0  0  0
  8 27  1  0  0  0  0
  8 97  1  0  0  0  0
 17 18  2  0  0  0  0
 17 19  1  0  0  0  0
 18 20  1  0  0  0  0
 18 21  1  0  0  0  0
 19 22  2  0  0  0  0
 19 23  1  0  0  0  0
 20 24  2  0  0  0  0
 20 25  1  0  0  0  0
 22 24  1  0  0  0  0
 22 26  1  0  0  0  0
 24128  1  0  0  0  0
 27 28  2  0  0  0  0
 27 29  1  0  0  0  0
 28 30  1  0  0  0  0
 28 31  1  0  0  0  0
 29 32  2  0  0  0  0
 29 33  1  0  0  0  0
 30 34  2  0  0  0  0
 30 35  1  0  0  0  0
 32 34  1  0  0  0  0
 32 36  1  0  0  0  0
 34116  1  0  0  0  0
 37 38  2  0  0  0  0
 37 39  1  0  0  0  0
 38 40  1  0  0  0  0
 38 41  1  0  0  0  0
 39 42  2  0  0  0  0
 39 43  1  0  0  0  0
 40 44  2  0  0  0  0
 40 45  1  0  0  0  0
 42 44  1  0  0  0  0
 42 46  1  0  0  0  0
 44112  1  0  0  0  0
 47 48  2  0  0  0  0
 47 49  1  0  0  0  0
 48 50  1  0  0  0  0
 48 51  1  0  0  0  0
 49 52  2  0  0  0  0
 49 53  1  0  0  0  0
```

```
 50 54  2  0  0  0  0
 50 55  1  0  0  0  0
 52 54  1  0  0  0  0
 52 56  1  0  0  0  0
 54104  1  0  0  0  0
 57 58  2  0  0  0  0
 57 59  1  0  0  0  0
 58 60  1  0  0  0  0
 58 61  1  0  0  0  0
 59 62  2  0  0  0  0
 59 63  1  0  0  0  0
 60 64  2  0  0  0  0
 60 65  1  0  0  0  0
 62 64  1  0  0  0  0
 62 66  1  0  0  0  0
 64108  1  0  0  0  0
 67 68  2  0  0  0  0
 67 69  1  0  0  0  0
 68 70  1  0  0  0  0
 68 71  1  0  0  0  0
 69 72  2  0  0  0  0
 69 73  1  0  0  0  0
 70 74  2  0  0  0  0
 70 75  1  0  0  0  0
 72 74  1  0  0  0  0
 72 76  1  0  0  0  0
 74100  1  0  0  0  0
 77 78  2  0  0  0  0
 77 79  1  0  0  0  0
 78 80  1  0  0  0  0
 78 81  1  0  0  0  0
 79 82  2  0  0  0  0
 79 83  1  0  0  0  0
 80 84  2  0  0  0  0
 80 85  1  0  0  0  0
 82 84  1  0  0  0  0
 82 86  1  0  0  0  0
 84120  1  0  0  0  0
 87 88  2  0  0  0  0
 87 89  1  0  0  0  0
 88 90  1  0  0  0  0
 88 91  1  0  0  0  0
 89 92  2  0  0  0  0
 89 93  1  0  0  0  0
 90 94  2  0  0  0  0
 90 95  1  0  0  0  0
 92 94  1  0  0  0  0
 92 96  1  0  0  0  0
 94124  1  0  0  0  0
 97 99  1  0  0  0  0
 97 98  1  0  0  0  0
100101  1  0  0  0  0
100102  1  0  0  0  0
100103  1  0  0  0  0
104105  1  0  0  0  0
104106  1  0  0  0  0
104107  1  0  0  0  0
108109  1  0  0  0  0
108110  1  0  0  0  0
108111  1  0  0  0  0
112113  1  0  0  0  0
112114  1  0  0  0  0
112115  1  0  0  0  0
116117  1  0  0  0  0
116118  1  0  0  0  0
```

```
116119  1  0  0  0  0
120121  1  0  0  0  0
120122  1  0  0  0  0
120123  1  0  0  0  0
124125  1  0  0  0  0
124126  1  0  0  0  0
124127  1  0  0  0  0
128129  1  0  0  0  0
128130  1  0  0  0  0
128131  1  0  0  0  0
M  END
$$$$
mol052
Optimized Catalyst 052
Optimized Catalyst 052
115124  0  0  0  0  0  0  0  0999 V2000
    3.7724    0.5116   -0.3228 C   0  0  0  0  0  0  0  0  0  0  0  0
    3.5655   -0.9100   -0.8050 C   0  0  0  0  0  0  0  0  0  0  0  0
    1.9840   -1.6107   -0.0373 P   0  0  0  0  0  0  0  0  0  0  0  0
   -1.2805    1.7043   -0.9893 P   0  0  0  0  0  0  0  0  0  0  0  0
   -2.7913    1.0994   -0.0160 C   0  0  0  0  0  0  0  0  0  0  0  0
   -3.0645   -0.3584   -0.3152 C   0  0  0  0  0  0  0  0  0  0  0  0
   -1.4859   -1.3985   -0.1621 P   0  0  0  0  0  0  0  0  0  0  0  0
    2.2176    1.5368   -0.6482 P   0  0  0  0  0  0  0  0  0  0  0  0
   -3.6835    1.7195   -0.2615 H   0  0  0  0  0  0  0  0  0  0  0  0
   -3.4440   -0.5064   -1.3603 H   0  0  0  0  0  0  0  0  0  0  0  0
    3.4493   -0.9517   -1.9191 H   0  0  0  0  0  0  0  0  0  0  0  0
    4.6285    0.9810   -0.8707 H   0  0  0  0  0  0  0  0  0  0  0  0
   -2.5352    1.2415    1.0737 H   0  0  0  0  0  0  0  0  0  0  0  0
   -3.8404   -0.7503    0.3886 H   0  0  0  0  0  0  0  0  0  0  0  0
    4.4324   -1.5483   -0.5014 H   0  0  0  0  0  0  0  0  0  0  0  0
    3.9936    0.5361    0.7770 H   0  0  0  0  0  0  0  0  0  0  0  0
    1.6882    3.0128   -2.6977 C   0  0  0  0  0  0  0  0  0  0  0  0
    1.4692    4.3474   -2.3342 C   0  0  0  0  0  0  0  0  0  0  0  0
    0.9780    2.3774   -3.7219 C   0  0  0  0  0  0  0  0  0  0  0  0
    0.5052    5.0689   -3.0412 C   0  0  0  0  0  0  0  0  0  0  0  0
    2.0262    4.7911   -1.5086 H   0  0  0  0  0  0  0  0  0  0  0  0
    0.0196    3.1236   -4.4130 C   0  0  0  0  0  0  0  0  0  0  0  0
    1.1527    1.3269   -3.9455 H   0  0  0  0  0  0  0  0  0  0  0  0
   -0.2111    4.4637   -4.0804 C   0  0  0  0  0  0  0  0  0  0  0  0
    0.3044    6.1054   -2.7724 H   0  0  0  0  0  0  0  0  0  0  0  0
   -0.5680    2.6487   -5.1982 H   0  0  0  0  0  0  0  0  0  0  0  0
   -0.9643    5.0312   -4.6229 H   0  0  0  0  0  0  0  0  0  0  0  0
    3.4730    3.0514    1.1518 C   0  0  0  0  0  0  0  0  0  0  0  0
    3.2900    2.5826    2.4605 C   0  0  0  0  0  0  0  0  0  0  0  0
    4.6572    3.6753    0.7343 C   0  0  0  0  0  0  0  0  0  0  0  0
    4.3322    2.7505    3.3723 C   0  0  0  0  0  0  0  0  0  0  0  0
    2.3449    2.1113    2.7487 H   0  0  0  0  0  0  0  0  0  0  0  0
    5.6833    3.8327    1.6684 C   0  0  0  0  0  0  0  0  0  0  0  0
    4.7518    4.0342   -0.2887 H   0  0  0  0  0  0  0  0  0  0  0  0
    5.5256    3.3701    2.9799 C   0  0  0  0  0  0  0  0  0  0  0  0
    4.2129    2.3963    4.3966 H   0  0  0  0  0  0  0  0  0  0  0  0
    6.6118    4.3205    1.3717 H   0  0  0  0  0  0  0  0  0  0  0  0
    6.3320    3.4957    3.6999 H   0  0  0  0  0  0  0  0  0  0  0  0
    1.7260   -2.4596    2.4043 C   0  0  0  0  0  0  0  0  0  0  0  0
    1.0287   -3.6733    2.3810 C   0  0  0  0  0  0  0  0  0  0  0  0
    1.5908   -1.5258    3.4390 C   0  0  0  0  0  0  0  0  0  0  0  0
    0.1499   -3.9441    3.4332 C   0  0  0  0  0  0  0  0  0  0  0  0
    1.1548   -4.3709    1.5547 H   0  0  0  0  0  0  0  0  0  0  0  0
    0.7077   -1.8237    4.4789 C   0  0  0  0  0  0  0  0  0  0  0  0
    2.1436   -0.5879    3.4069 H   0  0  0  0  0  0  0  0  0  0  0  0
   -0.0107   -3.0260    4.4768 C   0  0  0  0  0  0  0  0  0  0  0  0
   -0.4224   -4.8706    3.4299 H   0  0  0  0  0  0  0  0  0  0  0  0
    0.5667   -1.1041    5.2844 H   0  0  0  0  0  0  0  0  0  0  0  0
   -0.7104   -3.2396    5.2819 H   0  0  0  0  0  0  0  0  0  0  0  0
```

```
    2.9273   -3.7745   -1.2208 C   0  0  0  0  0  0  0  0  0  0  0  0
    3.0646   -3.7802   -2.6171 C   0  0  0  0  0  0  0  0  0  0  0  0
    3.8269   -4.4375   -0.3755 C   0  0  0  0  0  0  0  0  0  0  0  0
    4.1324   -4.4841   -3.1741 C   0  0  0  0  0  0  0  0  0  0  0  0
    2.3306   -3.2637   -3.2336 H   0  0  0  0  0  0  0  0  0  0  0  0
    4.8885   -5.1321   -0.9589 C   0  0  0  0  0  0  0  0  0  0  0  0
    3.6919   -4.4049    0.7066 H   0  0  0  0  0  0  0  0  0  0  0  0
    5.0434   -5.1559   -2.3497 C   0  0  0  0  0  0  0  0  0  0  0  0
    4.2561   -4.5102   -4.2567 H   0  0  0  0  0  0  0  0  0  0  0  0
    5.5993   -5.6602   -0.3227 H   0  0  0  0  0  0  0  0  0  0  0  0
    5.8746   -5.7009   -2.7927 H   0  0  0  0  0  0  0  0  0  0  0  0
   -2.7355   -2.2740    2.0265 C   0  0  0  0  0  0  0  0  0  0  0  0
   -2.6011   -1.3725    3.0911 C   0  0  0  0  0  0  0  0  0  0  0  0
   -3.8704   -3.0807    1.8610 C   0  0  0  0  0  0  0  0  0  0  0  0
   -3.6422   -1.2850    4.0148 C   0  0  0  0  0  0  0  0  0  0  0  0
   -1.6881   -0.7741    3.1917 H   0  0  0  0  0  0  0  0  0  0  0  0
   -4.8970   -2.9745    2.8019 C   0  0  0  0  0  0  0  0  0  0  0  0
   -3.9274   -3.7749    1.0252 H   0  0  0  0  0  0  0  0  0  0  0  0
   -4.7871   -2.0797    3.8721 C   0  0  0  0  0  0  0  0  0  0  0  0
   -3.5602   -0.5929    4.8534 H   0  0  0  0  0  0  0  0  0  0  0  0
   -5.7875   -3.5941    2.6999 H   0  0  0  0  0  0  0  0  0  0  0  0
   -5.5928   -2.0033    4.5994 H   0  0  0  0  0  0  0  0  0  0  0  0
   -1.1599   -3.6044   -1.6008 C   0  0  0  0  0  0  0  0  0  0  0  0
   -0.7556   -3.6673   -2.9414 C   0  0  0  0  0  0  0  0  0  0  0  0
   -0.8429   -4.6014   -0.6684 C   0  0  0  0  0  0  0  0  0  0  0  0
   -0.0221   -4.7812   -3.3553 C   0  0  0  0  0  0  0  0  0  0  0  0
   -1.0165   -2.8628   -3.6236 H   0  0  0  0  0  0  0  0  0  0  0  0
   -0.0929   -5.6945   -1.1064 C   0  0  0  0  0  0  0  0  0  0  0  0
   -1.1670   -4.5129    0.3711 H   0  0  0  0  0  0  0  0  0  0  0  0
    0.3101   -5.7904   -2.4440 C   0  0  0  0  0  0  0  0  0  0  0  0
    0.2960   -4.8619   -4.3939 H   0  0  0  0  0  0  0  0  0  0  0  0
    0.1824   -6.4754   -0.3984 H   0  0  0  0  0  0  0  0  0  0  0  0
    0.8951   -6.6463   -2.7731 H   0  0  0  0  0  0  0  0  0  0  0  0
   -1.4677    3.8172    0.5872 C   0  0  0  0  0  0  0  0  0  0  0  0
   -2.7179    4.3552    0.9276 C   0  0  0  0  0  0  0  0  0  0  0  0
   -0.3861    3.8126    1.4739 C   0  0  0  0  0  0  0  0  0  0  0  0
   -2.8787    4.8951    2.2054 C   0  0  0  0  0  0  0  0  0  0  0  0
   -3.5288    4.3599    0.2047 H   0  0  0  0  0  0  0  0  0  0  0  0
   -0.5756    4.3529    2.7479 C   0  0  0  0  0  0  0  0  0  0  0  0
    0.5830    3.3937    1.1831 H   0  0  0  0  0  0  0  0  0  0  0  0
   -1.8146    4.8918    3.1145 C   0  0  0  0  0  0  0  0  0  0  0  0
   -3.8392    5.3201    2.4937 H   0  0  0  0  0  0  0  0  0  0  0  0
    0.2524    4.3458    3.4577 H   0  0  0  0  0  0  0  0  0  0  0  0
   -1.9500    5.3104    4.1096 H   0  0  0  0  0  0  0  0  0  0  0  0
   -3.0439    2.4357   -2.8300 C   0  0  0  0  0  0  0  0  0  0  0  0
   -4.0139    1.5767   -3.3681 C   0  0  0  0  0  0  0  0  0  0  0  0
   -3.2727    3.8083   -2.6646 C   0  0  0  0  0  0  0  0  0  0  0  0
   -5.2499    2.1150   -3.7301 C   0  0  0  0  0  0  0  0  0  0  0  0
   -3.7856    0.5232   -3.5135 H   0  0  0  0  0  0  0  0  0  0  0  0
   -4.5189    4.3206   -3.0280 C   0  0  0  0  0  0  0  0  0  0  0  0
   -2.4816    4.4532   -2.2687 H   0  0  0  0  0  0  0  0  0  0  0  0
   -5.5064    3.4798   -3.5549 C   0  0  0  0  0  0  0  0  0  0  0  0
   -6.0173    1.4666   -4.1529 H   0  0  0  0  0  0  0  0  0  0  0  0
   -4.7192    5.3851   -2.9045 H   0  0  0  0  0  0  0  0  0  0  0  0
   -6.4742    3.8899   -3.8366 H   0  0  0  0  0  0  0  0  0  0  0  0
    0.3670    0.0607   -0.4905 Os  0  0  0  0  0  0  0  0  0  0  0  0
    0.1303    0.7922    2.1563 Cl  0  0  0  0  0  0  0  0  0  0  0  0
   -1.6745   -2.4690    1.1588 O   0  0  0  0  0  0  0  0  0  0  0  0
   -2.0298   -2.5684   -1.2704 O   0  0  0  0  0  0  0  0  0  0  0  0
   -1.7895    1.8757   -2.6020 O   0  0  0  0  0  0  0  0  0  0  0  0
   -1.3114    3.3906   -0.7316 O   0  0  0  0  0  0  0  0  0  0  0  0
    2.7274    2.3069   -2.0680 O   0  0  0  0  0  0  0  0  0  0  0  0
    2.4180    2.9871    0.2587 O   0  0  0  0  0  0  0  0  0  0  0  0
    1.7891   -3.1722   -0.6991 O   0  0  0  0  0  0  0  0  0  0  0  0
    2.6756   -2.1916    1.4096 O   0  0  0  0  0  0  0  0  0  0  0  0
```

```
    0.4546   -0.3336   -2.0563 H   0  0  0  0  0  0  0  0  0  0  0  0
  1  2  1  0  0  0  0
  1  8  1  0  0  0  0
  1 12  1  0  0  0  0
  1 16  1  0  0  0  0
  2  3  1  0  0  0  0
  2 11  1  0  0  0  0
  2 15  1  0  0  0  0
  3105  1  0  0  0  0
  3113  1  0  0  0  0
  3114  1  0  0  0  0
  4  5  1  0  0  0  0
  4105  1  0  0  0  0
  4109  1  0  0  0  0
  4110  1  0  0  0  0
  5  6  1  0  0  0  0
  5  9  1  0  0  0  0
  5 13  1  0  0  0  0
  6  7  1  0  0  0  0
  6 10  1  0  0  0  0
  6 14  1  0  0  0  0
  7105  1  0  0  0  0
  7107  1  0  0  0  0
  7108  1  0  0  0  0
  8105  1  0  0  0  0
  8111  1  0  0  0  0
  8112  1  0  0  0  0
 17 18  2  0  0  0  0
 17 19  1  0  0  0  0
 17111  1  0  0  0  0
 18 20  1  0  0  0  0
 18 21  1  0  0  0  0
 19 22  2  0  0  0  0
 19 23  1  0  0  0  0
 20 24  2  0  0  0  0
 20 25  1  0  0  0  0
 22 24  1  0  0  0  0
 22 26  1  0  0  0  0
 24 27  1  0  0  0  0
 28 29  2  0  0  0  0
 28 30  1  0  0  0  0
 28112  1  0  0  0  0
 29 31  1  0  0  0  0
 29 32  1  0  0  0  0
 30 33  2  0  0  0  0
 30 34  1  0  0  0  0
 31 35  2  0  0  0  0
 31 36  1  0  0  0  0
 33 35  1  0  0  0  0
 33 37  1  0  0  0  0
 35 38  1  0  0  0  0
 39 40  2  0  0  0  0
 39 41  1  0  0  0  0
 39114  1  0  0  0  0
 40 42  1  0  0  0  0
 40 43  1  0  0  0  0
 41 44  2  0  0  0  0
 41 45  1  0  0  0  0
 42 46  2  0  0  0  0
 42 47  1  0  0  0  0
 44 46  1  0  0  0  0
 44 48  1  0  0  0  0
 46 49  1  0  0  0  0
 50 51  2  0  0  0  0
 50 52  1  0  0  0  0
```

```
 50113  1  0  0  0  0
 51 53  1  0  0  0  0
 51 54  1  0  0  0  0
 52 55  2  0  0  0  0
 52 56  1  0  0  0  0
 53 57  2  0  0  0  0
 53 58  1  0  0  0  0
 55 57  1  0  0  0  0
 55 59  1  0  0  0  0
 57 60  1  0  0  0  0
 61 62  2  0  0  0  0
 61 63  1  0  0  0  0
 61107  1  0  0  0  0
 62 64  1  0  0  0  0
 62 65  1  0  0  0  0
 63 66  2  0  0  0  0
 63 67  1  0  0  0  0
 64 68  2  0  0  0  0
 64 69  1  0  0  0  0
 66 68  1  0  0  0  0
 66 70  1  0  0  0  0
 68 71  1  0  0  0  0
 72 73  2  0  0  0  0
 72 74  1  0  0  0  0
 72108  1  0  0  0  0
 73 75  1  0  0  0  0
 73 76  1  0  0  0  0
 74 77  2  0  0  0  0
 74 78  1  0  0  0  0
 75 79  2  0  0  0  0
 75 80  1  0  0  0  0
 77 79  1  0  0  0  0
 77 81  1  0  0  0  0
 79 82  1  0  0  0  0
 83 84  2  0  0  0  0
 83 85  1  0  0  0  0
 83110  1  0  0  0  0
 84 86  1  0  0  0  0
 84 87  1  0  0  0  0
 85 88  2  0  0  0  0
 85 89  1  0  0  0  0
 86 90  2  0  0  0  0
 86 91  1  0  0  0  0
 88 90  1  0  0  0  0
 88 92  1  0  0  0  0
 90 93  1  0  0  0  0
 94 95  2  0  0  0  0
 94 96  1  0  0  0  0
 94109  1  0  0  0  0
 95 97  1  0  0  0  0
 95 98  1  0  0  0  0
 96 99  2  0  0  0  0
 96100  1  0  0  0  0
 97101  2  0  0  0  0
 97102  1  0  0  0  0
 99101  1  0  0  0  0
 99103  1  0  0  0  0
101104  1  0  0  0  0
105115  1  0  0  0  0
105106  1  0  0  0  0
M  END
$$$$
mol053
Optimized Catalyst 053
Optimized Catalyst 053
```

```
115124  0  0  0  0  0  0  0  0999 V2000
    3.7837    0.5375   -0.3816 C   0  0  0  0  0  0  0  0  0  0  0  0
    3.5796   -0.8965   -0.8269 C   0  0  0  0  0  0  0  0  0  0  0  0
    1.9918   -1.5947   -0.0704 P   0  0  0  0  0  0  0  0  0  0  0  0
   -1.2752    1.7287   -0.9874 P   0  0  0  0  0  0  0  0  0  0  0  0
   -2.7918    1.1203   -0.0275 C   0  0  0  0  0  0  0  0  0  0  0  0
   -3.0577   -0.3392   -0.3260 C   0  0  0  0  0  0  0  0  0  0  0  0
   -1.4816   -1.3799   -0.1602 P   0  0  0  0  0  0  0  0  0  0  0  0
    2.2188    1.5510   -0.6862 P   0  0  0  0  0  0  0  0  0  0  0  0
   -3.6859    1.7352   -0.2820 H   0  0  0  0  0  0  0  0  0  0  0  0
   -3.4289   -0.4904   -1.3739 H   0  0  0  0  0  0  0  0  0  0  0  0
    3.4806   -0.9662   -1.9414 H   0  0  0  0  0  0  0  0  0  0  0  0
    4.6236    1.0004   -0.9612 H   0  0  0  0  0  0  0  0  0  0  0  0
   -2.5494    1.2659    1.0648 H   0  0  0  0  0  0  0  0  0  0  0  0
   -3.8402   -0.7298    0.3715 H   0  0  0  0  0  0  0  0  0  0  0  0
    4.4462   -1.5228   -0.4964 H   0  0  0  0  0  0  0  0  0  0  0  0
    4.0347    0.5840    0.7107 H   0  0  0  0  0  0  0  0  0  0  0  0
    1.6495    3.0229   -2.7289 C   0  0  0  0  0  0  0  0  0  0  0  0
    1.4091    4.3512   -2.3479 C   0  0  0  0  0  0  0  0  0  0  0  0
    0.9543    2.3889   -3.7678 C   0  0  0  0  0  0  0  0  0  0  0  0
    0.4535    5.0847   -3.0517 C   0  0  0  0  0  0  0  0  0  0  0  0
    1.9532    4.7874   -1.5071 H   0  0  0  0  0  0  0  0  0  0  0  0
    0.0007    3.1227   -4.4756 C   0  0  0  0  0  0  0  0  0  0  0  0
    1.1455    1.3406   -3.9965 H   0  0  0  0  0  0  0  0  0  0  0  0
   -0.2190    4.4543   -4.1045 C   0  0  0  0  0  0  0  0  0  0  0  0
    0.2356    6.1168   -2.7790 H   0  0  0  0  0  0  0  0  0  0  0  0
   -0.5741    2.6588   -5.2766 H   0  0  0  0  0  0  0  0  0  0  0  0
    3.4748    3.0665    1.1132 C   0  0  0  0  0  0  0  0  0  0  0  0
    3.2840    2.6119    2.4280 C   0  0  0  0  0  0  0  0  0  0  0  0
    4.6623    3.6845    0.6908 C   0  0  0  0  0  0  0  0  0  0  0  0
    4.3160    2.7768    3.3503 C   0  0  0  0  0  0  0  0  0  0  0  0
    2.3342    2.1466    2.7169 H   0  0  0  0  0  0  0  0  0  0  0  0
    5.6963    3.8509    1.6127 C   0  0  0  0  0  0  0  0  0  0  0  0
    4.7568    4.0349   -0.3372 H   0  0  0  0  0  0  0  0  0  0  0  0
    5.4986    3.3903    2.9193 C   0  0  0  0  0  0  0  0  0  0  0  0
    4.1993    2.4386    4.3808 H   0  0  0  0  0  0  0  0  0  0  0  0
    6.6304    4.3311    1.3203 H   0  0  0  0  0  0  0  0  0  0  0  0
    1.6990   -2.4748    2.3552 C   0  0  0  0  0  0  0  0  0  0  0  0
    1.0050   -3.6914    2.3018 C   0  0  0  0  0  0  0  0  0  0  0  0
    1.5503   -1.5575    3.4069 C   0  0  0  0  0  0  0  0  0  0  0  0
    0.1204   -4.0028    3.3366 C   0  0  0  0  0  0  0  0  0  0  0  0
    1.1434   -4.3726    1.4613 H   0  0  0  0  0  0  0  0  0  0  0  0
    0.6696   -1.8688    4.4430 C   0  0  0  0  0  0  0  0  0  0  0  0
    2.1079   -0.6199    3.3983 H   0  0  0  0  0  0  0  0  0  0  0  0
   -0.0249   -3.0827    4.3797 C   0  0  0  0  0  0  0  0  0  0  0  0
   -0.4497   -4.9303    3.3200 H   0  0  0  0  0  0  0  0  0  0  0  0
    0.5174   -1.1728    5.2673 H   0  0  0  0  0  0  0  0  0  0  0  0
    2.9556   -3.7735   -1.2122 C   0  0  0  0  0  0  0  0  0  0  0  0
    3.1301   -3.8208   -2.6051 C   0  0  0  0  0  0  0  0  0  0  0  0
    3.8222   -4.4231   -0.3205 C   0  0  0  0  0  0  0  0  0  0  0  0
    4.1965   -4.5519   -3.1261 C   0  0  0  0  0  0  0  0  0  0  0  0
    2.4217   -3.3080   -3.2558 H   0  0  0  0  0  0  0  0  0  0  0  0
    4.8935   -5.1514   -0.8387 C   0  0  0  0  0  0  0  0  0  0  0  0
    3.6572   -4.3543    0.7577 H   0  0  0  0  0  0  0  0  0  0  0  0
    5.0536   -5.1994   -2.2281 C   0  0  0  0  0  0  0  0  0  0  0  0
    4.3523   -4.6212   -4.2029 H   0  0  0  0  0  0  0  0  0  0  0  0
    5.5825   -5.6738   -0.1738 H   0  0  0  0  0  0  0  0  0  0  0  0
   -2.7334   -2.2415    2.0266 C   0  0  0  0  0  0  0  0  0  0  0  0
   -2.6147   -1.3369    3.0935 C   0  0  0  0  0  0  0  0  0  0  0  0
   -3.8586   -3.0594    1.8453 C   0  0  0  0  0  0  0  0  0  0  0  0
   -3.6587   -1.2467    4.0118 C   0  0  0  0  0  0  0  0  0  0  0  0
   -1.7062   -0.7307    3.2000 H   0  0  0  0  0  0  0  0  0  0  0  0
   -4.9048   -2.9715    2.7654 C   0  0  0  0  0  0  0  0  0  0  0  0
   -3.8995   -3.7537    1.0065 H   0  0  0  0  0  0  0  0  0  0  0  0
   -4.7793   -2.0660    3.8243 C   0  0  0  0  0  0  0  0  0  0  0  0
```

```
  -3.5960   -0.5604    4.8572 H   0  0  0  0  0  0  0  0  0  0  0  0
  -5.7907   -3.5976    2.6613 H   0  0  0  0  0  0  0  0  0  0  0  0
  -1.1610   -3.6015   -1.5653 C   0  0  0  0  0  0  0  0  0  0  0  0
  -0.7035   -3.6357   -2.8913 C   0  0  0  0  0  0  0  0  0  0  0  0
  -0.8926   -4.6239   -0.6417 C   0  0  0  0  0  0  0  0  0  0  0  0
   0.0363   -4.7401   -3.3160 C   0  0  0  0  0  0  0  0  0  0  0  0
  -0.9347   -2.8106   -3.5629 H   0  0  0  0  0  0  0  0  0  0  0  0
  -0.1377   -5.7200   -1.0597 C   0  0  0  0  0  0  0  0  0  0  0  0
  -1.2659   -4.5531    0.3839 H   0  0  0  0  0  0  0  0  0  0  0  0
   0.3051   -5.7520   -2.3872 C   0  0  0  0  0  0  0  0  0  0  0  0
   0.3917   -4.8113   -4.3429 H   0  0  0  0  0  0  0  0  0  0  0  0
   0.0995   -6.5277   -0.3681 H   0  0  0  0  0  0  0  0  0  0  0  0
  -1.4481    3.8095    0.6223 C   0  0  0  0  0  0  0  0  0  0  0  0
  -2.7031    4.3282    0.9768 C   0  0  0  0  0  0  0  0  0  0  0  0
  -0.3598    3.7921    1.5066 C   0  0  0  0  0  0  0  0  0  0  0  0
  -2.8850    4.8257    2.2681 C   0  0  0  0  0  0  0  0  0  0  0  0
  -3.5126    4.3521    0.2496 H   0  0  0  0  0  0  0  0  0  0  0  0
  -0.5398    4.2846    2.7992 C   0  0  0  0  0  0  0  0  0  0  0  0
   0.6145    3.4007    1.1908 H   0  0  0  0  0  0  0  0  0  0  0  0
  -1.7998    4.7829    3.1500 C   0  0  0  0  0  0  0  0  0  0  0  0
  -3.8464    5.2355    2.5761 H   0  0  0  0  0  0  0  0  0  0  0  0
   0.2840    4.2685    3.5139 H   0  0  0  0  0  0  0  0  0  0  0  0
  -3.0329    2.4824   -2.8094 C   0  0  0  0  0  0  0  0  0  0  0  0
  -3.9907    1.6102   -3.3501 C   0  0  0  0  0  0  0  0  0  0  0  0
  -3.2805    3.8512   -2.6231 C   0  0  0  0  0  0  0  0  0  0  0  0
  -5.2461    2.1149   -3.6900 C   0  0  0  0  0  0  0  0  0  0  0  0
  -3.7384    0.5636   -3.5167 H   0  0  0  0  0  0  0  0  0  0  0  0
  -4.5355    4.3582   -2.9574 C   0  0  0  0  0  0  0  0  0  0  0  0
  -2.4918    4.5049   -2.2327 H   0  0  0  0  0  0  0  0  0  0  0  0
  -5.4901    3.4764   -3.4779 C   0  0  0  0  0  0  0  0  0  0  0  0
  -6.0115    1.4646   -4.1139 H   0  0  0  0  0  0  0  0  0  0  0  0
  -4.7589    5.4178   -2.8267 H   0  0  0  0  0  0  0  0  0  0  0  0
   0.3699    0.0757   -0.5108 Os  0  0  0  0  0  0  0  0  0  0  0  0
   0.1498    0.7985    2.1396 Cl  0  0  0  0  0  0  0  0  0  0  0  0
  -1.6525   -2.4281    1.1804 O   0  0  0  0  0  0  0  0  0  0  0  0
  -2.0379   -2.5711   -1.2416 O   0  0  0  0  0  0  0  0  0  0  0  0
  -1.7630    1.9464   -2.6002 O   0  0  0  0  0  0  0  0  0  0  0  0
  -1.2876    3.4149   -0.7052 O   0  0  0  0  0  0  0  0  0  0  0  0
   2.7013    2.3286   -2.1121 O   0  0  0  0  0  0  0  0  0  0  0  0
   2.4199    3.0042    0.2203 O   0  0  0  0  0  0  0  0  0  0  0  0
   1.8021   -3.1515   -0.7485 O   0  0  0  0  0  0  0  0  0  0  0  0
   2.6640   -2.1892    1.3822 O   0  0  0  0  0  0  0  0  0  0  0  0
   0.4430   -0.3133   -2.0787 H   0  0  0  0  0  0  0  0  0  0  0  0
   6.0775   -5.8974   -2.7236 F   0  0  0  0  0  0  0  0  0  0  0  0
   1.0249   -6.8025   -2.7892 F   0  0  0  0  0  0  0  0  0  0  0  0
  -5.7820   -1.9771    4.7012 F   0  0  0  0  0  0  0  0  0  0  0  0
  -0.8755   -3.3756    5.3670 F   0  0  0  0  0  0  0  0  0  0  0  0
  -6.6944    3.9590   -3.7910 F   0  0  0  0  0  0  0  0  0  0  0  0
  -1.1271    5.1585   -4.7858 F   0  0  0  0  0  0  0  0  0  0  0  0
   6.4895    3.5435    3.8006 F   0  0  0  0  0  0  0  0  0  0  0  0
  -1.9754    5.2477    4.3894 F   0  0  0  0  0  0  0  0  0  0  0  0
  1  2  1  0  0  0  0
  1  8  1  0  0  0  0
  1 12  1  0  0  0  0
  1 16  1  0  0  0  0
  2  3  1  0  0  0  0
  2 11  1  0  0  0  0
  2 15  1  0  0  0  0
  3 97  1  0  0  0  0
  3105  1  0  0  0  0
  3106  1  0  0  0  0
  4  5  1  0  0  0  0
  4 97  1  0  0  0  0
  4101  1  0  0  0  0
  4102  1  0  0  0  0
```

```
  5  6  1  0  0  0  0
  5  9  1  0  0  0  0
  5 13  1  0  0  0  0
  6  7  1  0  0  0  0
  6 10  1  0  0  0  0
  6 14  1  0  0  0  0
  7 97  1  0  0  0  0
  7 99  1  0  0  0  0
  7100  1  0  0  0  0
  8 97  1  0  0  0  0
  8103  1  0  0  0  0
  8104  1  0  0  0  0
 17 18  2  0  0  0  0
 17 19  1  0  0  0  0
 17103  1  0  0  0  0
 18 20  1  0  0  0  0
 18 21  1  0  0  0  0
 19 22  2  0  0  0  0
 19 23  1  0  0  0  0
 20 24  2  0  0  0  0
 20 25  1  0  0  0  0
 22 24  1  0  0  0  0
 22 26  1  0  0  0  0
 24113  1  0  0  0  0
 27 28  2  0  0  0  0
 27 29  1  0  0  0  0
 27104  1  0  0  0  0
 28 30  1  0  0  0  0
 28 31  1  0  0  0  0
 29 32  2  0  0  0  0
 29 33  1  0  0  0  0
 30 34  2  0  0  0  0
 30 35  1  0  0  0  0
 32 34  1  0  0  0  0
 32 36  1  0  0  0  0
 34114  1  0  0  0  0
 37 38  2  0  0  0  0
 37 39  1  0  0  0  0
 37106  1  0  0  0  0
 38 40  1  0  0  0  0
 38 41  1  0  0  0  0
 39 42  2  0  0  0  0
 39 43  1  0  0  0  0
 40 44  2  0  0  0  0
 40 45  1  0  0  0  0
 42 44  1  0  0  0  0
 42 46  1  0  0  0  0
 44111  1  0  0  0  0
 47 48  2  0  0  0  0
 47 49  1  0  0  0  0
 47105  1  0  0  0  0
 48 50  1  0  0  0  0
 48 51  1  0  0  0  0
 49 52  2  0  0  0  0
 49 53  1  0  0  0  0
 50 54  2  0  0  0  0
 50 55  1  0  0  0  0
 52 54  1  0  0  0  0
 52 56  1  0  0  0  0
 54108  1  0  0  0  0
 57 58  2  0  0  0  0
 57 59  1  0  0  0  0
 57 99  1  0  0  0  0
 58 60  1  0  0  0  0
 58 61  1  0  0  0  0
```

```
 59 62  2  0  0  0  0
 59 63  1  0  0  0  0
 60 64  2  0  0  0  0
 60 65  1  0  0  0  0
 62 64  1  0  0  0  0
 62 66  1  0  0  0  0
 64110  1  0  0  0  0
 67 68  2  0  0  0  0
 67 69  1  0  0  0  0
 67100  1  0  0  0  0
 68 70  1  0  0  0  0
 68 71  1  0  0  0  0
 69 72  2  0  0  0  0
 69 73  1  0  0  0  0
 70 74  2  0  0  0  0
 70 75  1  0  0  0  0
 72 74  1  0  0  0  0
 72 76  1  0  0  0  0
 74109  1  0  0  0  0
 77 78  2  0  0  0  0
 77 79  1  0  0  0  0
 77102  1  0  0  0  0
 78 80  1  0  0  0  0
 78 81  1  0  0  0  0
 79 82  2  0  0  0  0
 79 83  1  0  0  0  0
 80 84  2  0  0  0  0
 80 85  1  0  0  0  0
 82 84  1  0  0  0  0
 82 86  1  0  0  0  0
 84115  1  0  0  0  0
 87 88  2  0  0  0  0
 87 89  1  0  0  0  0
 87101  1  0  0  0  0
 88 90  1  0  0  0  0
 88 91  1  0  0  0  0
 89 92  2  0  0  0  0
 89 93  1  0  0  0  0
 90 94  2  0  0  0  0
 90 95  1  0  0  0  0
 92 94  1  0  0  0  0
 92 96  1  0  0  0  0
 94112  1  0  0  0  0
 97107  1  0  0  0  0
 97 98  1  0  0  0  0
M  END
$$$$
mol054
Optimized Catalyst 054
Optimized Catalyst 054
139148  0  0  0  0  0  0  0  0999 V2000
    3.7827    0.5699   -0.3522 C   0  0  0  0  0  0  0  0  0  0  0  0
    3.5827   -0.8607   -0.8089 C   0  0  0  0  0  0  0  0  0  0  0  0
    1.9929   -1.5607   -0.0563 P   0  0  0  0  0  0  0  0  0  0  0  0
   -1.2748    1.7613   -0.9818 P   0  0  0  0  0  0  0  0  0  0  0  0
   -2.7833    1.1455   -0.0102 C   0  0  0  0  0  0  0  0  0  0  0  0
   -3.0535   -0.3099   -0.3237 C   0  0  0  0  0  0  0  0  0  0  0  0
   -1.4757   -1.3533   -0.1712 P   0  0  0  0  0  0  0  0  0  0  0  0
    2.2214    1.5897   -0.6732 P   0  0  0  0  0  0  0  0  0  0  0  0
   -3.6767    1.7657   -0.2502 H   0  0  0  0  0  0  0  0  0  0  0  0
   -3.4257   -0.4486   -1.3722 H   0  0  0  0  0  0  0  0  0  0  0  0
    3.4831   -0.9221   -1.9232 H   0  0  0  0  0  0  0  0  0  0  0  0
    4.6285    1.0374   -0.9174 H   0  0  0  0  0  0  0  0  0  0  0  0
   -2.5266    1.2800    1.0797 H   0  0  0  0  0  0  0  0  0  0  0  0
   -3.8312   -0.7086    0.3734 H   0  0  0  0  0  0  0  0  0  0  0  0
```

4.4460	-1.4908	-0.4799	H	0	0	0	0	0	0	0	0	0	0	0	0
4.0163	0.6106	0.7440	H	0	0	0	0	0	0	0	0	0	0	0	0
1.6699	3.0188	-2.7488	C	0	0	0	0	0	0	0	0	0	0	0	0
1.4253	4.3604	-2.4361	C	0	0	0	0	0	0	0	0	0	0	0	0
0.9678	2.3379	-3.7506	C	0	0	0	0	0	0	0	0	0	0	0	0
0.4519	5.0431	-3.1678	C	0	0	0	0	0	0	0	0	0	0	0	0
1.9727	4.8439	-1.6267	H	0	0	0	0	0	0	0	0	0	0	0	0
0.0022	3.0405	-4.4706	C	0	0	0	0	0	0	0	0	0	0	0	0
1.1579	1.2817	-3.9340	H	0	0	0	0	0	0	0	0	0	0	0	0
-0.2602	4.3941	-4.1892	C	0	0	0	0	0	0	0	0	0	0	0	0
0.2414	6.0841	-2.9300	H	0	0	0	0	0	0	0	0	0	0	0	0
-0.5733	2.5249	-5.2376	H	0	0	0	0	0	0	0	0	0	0	0	0
3.4784	3.0932	1.1252	C	0	0	0	0	0	0	0	0	0	0	0	0
3.2962	2.6189	2.4311	C	0	0	0	0	0	0	0	0	0	0	0	0
4.6695	3.7093	0.7149	C	0	0	0	0	0	0	0	0	0	0	0	0
4.3414	2.7624	3.3426	C	0	0	0	0	0	0	0	0	0	0	0	0
2.3480	2.1530	2.7205	H	0	0	0	0	0	0	0	0	0	0	0	0
5.7007	3.8443	1.6432	C	0	0	0	0	0	0	0	0	0	0	0	0
4.7662	4.0772	-0.3052	H	0	0	0	0	0	0	0	0	0	0	0	0
5.5484	3.3706	2.9587	C	0	0	0	0	0	0	0	0	0	0	0	0
4.2120	2.3958	4.3603	H	0	0	0	0	0	0	0	0	0	0	0	0
6.6324	4.3224	1.3441	H	0	0	0	0	0	0	0	0	0	0	0	0
1.6820	-2.4953	2.3365	C	0	0	0	0	0	0	0	0	0	0	0	0
1.0156	-3.7231	2.2382	C	0	0	0	0	0	0	0	0	0	0	0	0
1.4734	-1.6098	3.3998	C	0	0	0	0	0	0	0	0	0	0	0	0
0.0995	-4.0588	3.2347	C	0	0	0	0	0	0	0	0	0	0	0	0
1.1907	-4.3835	1.3893	H	0	0	0	0	0	0	0	0	0	0	0	0
0.5542	-1.9675	4.3874	C	0	0	0	0	0	0	0	0	0	0	0	0
2.0038	-0.6581	3.4325	H	0	0	0	0	0	0	0	0	0	0	0	0
-0.1410	-3.1857	4.3103	C	0	0	0	0	0	0	0	0	0	0	0	0
-0.4491	-4.9954	3.1641	H	0	0	0	0	0	0	0	0	0	0	0	0
0.3640	-1.2774	5.2069	H	0	0	0	0	0	0	0	0	0	0	0	0
2.9362	-3.7216	-1.2567	C	0	0	0	0	0	0	0	0	0	0	0	0
3.1271	-3.6577	-2.6443	C	0	0	0	0	0	0	0	0	0	0	0	0
3.7755	-4.4771	-0.4260	C	0	0	0	0	0	0	0	0	0	0	0	0
4.1745	-4.3837	-3.2098	C	0	0	0	0	0	0	0	0	0	0	0	0
2.4432	-3.0716	-3.2571	H	0	0	0	0	0	0	0	0	0	0	0	0
4.8157	-5.1966	-1.0118	C	0	0	0	0	0	0	0	0	0	0	0	0
3.6094	-4.4935	0.6522	H	0	0	0	0	0	0	0	0	0	0	0	0
5.0221	-5.1602	-2.4025	C	0	0	0	0	0	0	0	0	0	0	0	0
4.3282	-4.3457	-4.2870	H	0	0	0	0	0	0	0	0	0	0	0	0
5.4740	-5.7944	-0.3822	H	0	0	0	0	0	0	0	0	0	0	0	0
-2.7242	-2.2160	2.0244	C	0	0	0	0	0	0	0	0	0	0	0	0
-2.6283	-1.2517	3.0363	C	0	0	0	0	0	0	0	0	0	0	0	0
-3.8184	-3.0879	1.9275	C	0	0	0	0	0	0	0	0	0	0	0	0
-3.6595	-1.1618	3.9693	C	0	0	0	0	0	0	0	0	0	0	0	0
-1.7446	-0.6051	3.0980	H	0	0	0	0	0	0	0	0	0	0	0	0
-4.8348	-2.9863	2.8766	C	0	0	0	0	0	0	0	0	0	0	0	0
-3.8533	-3.8277	1.1303	H	0	0	0	0	0	0	0	0	0	0	0	0
-4.7659	-2.0257	3.9006	C	0	0	0	0	0	0	0	0	0	0	0	0
-3.5953	-0.4142	4.7588	H	0	0	0	0	0	0	0	0	0	0	0	0
-5.6889	-3.6592	2.8212	H	0	0	0	0	0	0	0	0	0	0	0	0
-1.1456	-3.5680	-1.5809	C	0	0	0	0	0	0	0	0	0	0	0	0
-0.6471	-3.5972	-2.8889	C	0	0	0	0	0	0	0	0	0	0	0	0
-0.9076	-4.6015	-0.6643	C	0	0	0	0	0	0	0	0	0	0	0	0
0.1079	-4.7020	-3.2865	C	0	0	0	0	0	0	0	0	0	0	0	0
-0.8495	-2.7686	-3.5630	H	0	0	0	0	0	0	0	0	0	0	0	0
-0.1390	-5.6881	-1.0786	C	0	0	0	0	0	0	0	0	0	0	0	0
-1.3035	-4.5403	0.3517	H	0	0	0	0	0	0	0	0	0	0	0	0
0.3753	-5.7470	-2.3864	C	0	0	0	0	0	0	0	0	0	0	0	0
0.4997	-4.7444	-4.3006	H	0	0	0	0	0	0	0	0	0	0	0	0
0.0715	-6.4931	-0.3769	H	0	0	0	0	0	0	0	0	0	0	0	0
-1.4597	3.8374	0.6276	C	0	0	0	0	0	0	0	0	0	0	0	0
-2.7160	4.3418	0.9888	C	0	0	0	0	0	0	0	0	0	0	0	0
-0.3760	3.8304	1.5141	C	0	0	0	0	0	0	0	0	0	0	0	0

```
   -2.8898    4.8322    2.2842 C   0  0  0  0  0  0  0  0  0  0  0  0
   -3.5287    4.3591    0.2673 H   0  0  0  0  0  0  0  0  0  0  0  0
   -0.5729    4.3195    2.8044 C   0  0  0  0  0  0  0  0  0  0  0  0
    0.6030    3.4484    1.2036 H   0  0  0  0  0  0  0  0  0  0  0  0
   -1.8269    4.8171    3.2015 C   0  0  0  0  0  0  0  0  0  0  0  0
   -3.8590    5.2286    2.5791 H   0  0  0  0  0  0  0  0  0  0  0  0
    0.2589    4.3029    3.5082 H   0  0  0  0  0  0  0  0  0  0  0  0
   -3.0562    2.5142   -2.7852 C   0  0  0  0  0  0  0  0  0  0  0  0
   -4.0247    1.6626   -3.3386 C   0  0  0  0  0  0  0  0  0  0  0  0
   -3.3018    3.8780   -2.5826 C   0  0  0  0  0  0  0  0  0  0  0  0
   -5.2679    2.1941   -3.6746 C   0  0  0  0  0  0  0  0  0  0  0  0
   -3.7874    0.6147   -3.5121 H   0  0  0  0  0  0  0  0  0  0  0  0
   -4.5558    4.3891   -2.9174 C   0  0  0  0  0  0  0  0  0  0  0  0
   -2.5130    4.5227   -2.1787 H   0  0  0  0  0  0  0  0  0  0  0  0
   -5.5473    3.5562   -3.4603 C   0  0  0  0  0  0  0  0  0  0  0  0
   -6.0289    1.5470   -4.1085 H   0  0  0  0  0  0  0  0  0  0  0  0
   -4.7570    5.4476   -2.7583 H   0  0  0  0  0  0  0  0  0  0  0  0
    0.3733    0.1103   -0.5066 Os  0  0  0  0  0  0  0  0  0  0  0  0
    0.1517    0.8429    2.1450 Cl  0  0  0  0  0  0  0  0  0  0  0  0
   -1.6681   -2.4189    1.1544 O   0  0  0  0  0  0  0  0  0  0  0  0
   -2.0249   -2.5324   -1.2668 O   0  0  0  0  0  0  0  0  0  0  0  0
   -1.7933    1.9610   -2.5885 O   0  0  0  0  0  0  0  0  0  0  0  0
   -1.2941    3.4456   -0.7017 O   0  0  0  0  0  0  0  0  0  0  0  0
    2.7243    2.3563   -2.0974 O   0  0  0  0  0  0  0  0  0  0  0  0
    2.4236    3.0431    0.2293 O   0  0  0  0  0  0  0  0  0  0  0  0
    1.8012   -3.1170   -0.7349 O   0  0  0  0  0  0  0  0  0  0  0  0
    2.6640   -2.1615    1.3917 O   0  0  0  0  0  0  0  0  0  0  0  0
    0.4505   -0.2805   -2.0722 H   0  0  0  0  0  0  0  0  0  0  0  0
   -6.8958    4.0978   -3.8196 C   0  0  0  0  0  0  0  0  0  0  0  0
   -7.0689    3.9755   -4.9154 H   0  0  0  0  0  0  0  0  0  0  0  0
   -7.6857    3.5395   -3.2634 H   0  0  0  0  0  0  0  0  0  0  0  0
   -6.9836    5.1786   -3.5668 H   0  0  0  0  0  0  0  0  0  0  0  0
   -1.3026    5.1243   -4.9784 C   0  0  0  0  0  0  0  0  0  0  0  0
   -2.2204    4.4941   -5.0705 H   0  0  0  0  0  0  0  0  0  0  0  0
   -1.5870    6.0811   -4.4862 H   0  0  0  0  0  0  0  0  0  0  0  0
   -0.9079    5.3454   -5.9974 H   0  0  0  0  0  0  0  0  0  0  0  0
   -2.0159    5.3485    4.5890 C   0  0  0  0  0  0  0  0  0  0  0  0
   -1.7036    6.4199    4.6133 H   0  0  0  0  0  0  0  0  0  0  0  0
   -3.0811    5.2751    4.9044 H   0  0  0  0  0  0  0  0  0  0  0  0
   -1.3944    4.7749    5.3152 H   0  0  0  0  0  0  0  0  0  0  0  0
   -5.8716   -1.9356    4.9063 C   0  0  0  0  0  0  0  0  0  0  0  0
   -6.8202   -1.6491    4.3938 H   0  0  0  0  0  0  0  0  0  0  0  0
   -6.0111   -2.9245    5.4034 H   0  0  0  0  0  0  0  0  0  0  0  0
   -5.6487   -1.1758    5.6893 H   0  0  0  0  0  0  0  0  0  0  0  0
   -1.1588   -3.5547    5.3447 C   0  0  0  0  0  0  0  0  0  0  0  0
   -0.9648   -4.5834    5.7239 H   0  0  0  0  0  0  0  0  0  0  0  0
   -1.1419   -2.8476    6.2031 H   0  0  0  0  0  0  0  0  0  0  0  0
   -2.1751   -3.5206    4.8783 H   0  0  0  0  0  0  0  0  0  0  0  0
    6.6747    3.5178    3.9341 C   0  0  0  0  0  0  0  0  0  0  0  0
    7.5614    2.9460    3.5713 H   0  0  0  0  0  0  0  0  0  0  0  0
    6.9507    4.5944    4.0320 H   0  0  0  0  0  0  0  0  0  0  0  0
    6.3942    3.1334    4.9411 H   0  0  0  0  0  0  0  0  0  0  0  0
    6.1431   -5.9484   -3.0049 C   0  0  0  0  0  0  0  0  0  0  0  0
    7.1178   -5.5712   -2.6132 H   0  0  0  0  0  0  0  0  0  0  0  0
    6.0357   -7.0253   -2.7337 H   0  0  0  0  0  0  0  0  0  0  0  0
    6.1523   -5.8640   -4.1152 H   0  0  0  0  0  0  0  0  0  0  0  0
    1.2387   -6.9001   -2.7943 C   0  0  0  0  0  0  0  0  0  0  0  0
    0.8764   -7.8468   -2.3338 H   0  0  0  0  0  0  0  0  0  0  0  0
    1.2496   -7.0262   -3.8995 H   0  0  0  0  0  0  0  0  0  0  0  0
    2.2830   -6.7013   -2.4448 H   0  0  0  0  0  0  0  0  0  0  0  0
  1  2  1  0  0  0  0
  1  8  1  0  0  0  0
  1 12  1  0  0  0  0
  1 16  1  0  0  0  0
  2  3  1  0  0  0  0
```

```
  2 11  1  0  0  0  0
  2 15  1  0  0  0  0
  3 97  1  0  0  0  0
  3105  1  0  0  0  0
  3106  1  0  0  0  0
  4  5  1  0  0  0  0
  4 97  1  0  0  0  0
  4101  1  0  0  0  0
  4102  1  0  0  0  0
  5  6  1  0  0  0  0
  5  9  1  0  0  0  0
  5 13  1  0  0  0  0
  6  7  1  0  0  0  0
  6 10  1  0  0  0  0
  6 14  1  0  0  0  0
  7 97  1  0  0  0  0
  7 99  1  0  0  0  0
  7100  1  0  0  0  0
  8 97  1  0  0  0  0
  8103  1  0  0  0  0
  8104  1  0  0  0  0
 17 18  2  0  0  0  0
 17 19  1  0  0  0  0
 17103  1  0  0  0  0
 18 20  1  0  0  0  0
 18 21  1  0  0  0  0
 19 22  2  0  0  0  0
 19 23  1  0  0  0  0
 20 24  2  0  0  0  0
 20 25  1  0  0  0  0
 22 24  1  0  0  0  0
 22 26  1  0  0  0  0
 24112  1  0  0  0  0
 27 28  2  0  0  0  0
 27 29  1  0  0  0  0
 27104  1  0  0  0  0
 28 30  1  0  0  0  0
 28 31  1  0  0  0  0
 29 32  2  0  0  0  0
 29 33  1  0  0  0  0
 30 34  2  0  0  0  0
 30 35  1  0  0  0  0
 32 34  1  0  0  0  0
 32 36  1  0  0  0  0
 34128  1  0  0  0  0
 37 38  2  0  0  0  0
 37 39  1  0  0  0  0
 37106  1  0  0  0  0
 38 40  1  0  0  0  0
 38 41  1  0  0  0  0
 39 42  2  0  0  0  0
 39 43  1  0  0  0  0
 40 44  2  0  0  0  0
 40 45  1  0  0  0  0
 42 44  1  0  0  0  0
 42 46  1  0  0  0  0
 44124  1  0  0  0  0
 47 48  2  0  0  0  0
 47 49  1  0  0  0  0
 47105  1  0  0  0  0
 48 50  1  0  0  0  0
 48 51  1  0  0  0  0
 49 52  2  0  0  0  0
 49 53  1  0  0  0  0
 50 54  2  0  0  0  0
```

```
 50 55  1  0  0  0  0
 52 54  1  0  0  0  0
 52 56  1  0  0  0  0
 54132  1  0  0  0  0
 57 58  2  0  0  0  0
 57 59  1  0  0  0  0
 57 99  1  0  0  0  0
 58 60  1  0  0  0  0
 58 61  1  0  0  0  0
 59 62  2  0  0  0  0
 59 63  1  0  0  0  0
 60 64  2  0  0  0  0
 60 65  1  0  0  0  0
 62 64  1  0  0  0  0
 62 66  1  0  0  0  0
 64120  1  0  0  0  0
 67 68  2  0  0  0  0
 67 69  1  0  0  0  0
 67100  1  0  0  0  0
 68 70  1  0  0  0  0
 68 71  1  0  0  0  0
 69 72  2  0  0  0  0
 69 73  1  0  0  0  0
 70 74  2  0  0  0  0
 70 75  1  0  0  0  0
 72 74  1  0  0  0  0
 72 76  1  0  0  0  0
 74136  1  0  0  0  0
 77 78  2  0  0  0  0
 77 79  1  0  0  0  0
 77102  1  0  0  0  0
 78 80  1  0  0  0  0
 78 81  1  0  0  0  0
 79 82  2  0  0  0  0
 79 83  1  0  0  0  0
 80 84  2  0  0  0  0
 80 85  1  0  0  0  0
 82 84  1  0  0  0  0
 82 86  1  0  0  0  0
 84116  1  0  0  0  0
 87 88  2  0  0  0  0
 87 89  1  0  0  0  0
 87101  1  0  0  0  0
 88 90  1  0  0  0  0
 88 91  1  0  0  0  0
 89 92  2  0  0  0  0
 89 93  1  0  0  0  0
 90 94  2  0  0  0  0
 90 95  1  0  0  0  0
 92 94  1  0  0  0  0
 92 96  1  0  0  0  0
 94108  1  0  0  0  0
 97107  1  0  0  0  0
 97 98  1  0  0  0  0
108109  1  0  0  0  0
108110  1  0  0  0  0
108111  1  0  0  0  0
112113  1  0  0  0  0
112114  1  0  0  0  0
112115  1  0  0  0  0
116117  1  0  0  0  0
116118  1  0  0  0  0
116119  1  0  0  0  0
120121  1  0  0  0  0
120122  1  0  0  0  0
```

```
120123  1  0  0  0  0
124125  1  0  0  0  0
124126  1  0  0  0  0
124127  1  0  0  0  0
128129  1  0  0  0  0
128130  1  0  0  0  0
128131  1  0  0  0  0
132133  1  0  0  0  0
132134  1  0  0  0  0
132135  1  0  0  0  0
136137  1  0  0  0  0
136138  1  0  0  0  0
136139  1  0  0  0  0
M  END
$$$$
mol056
Optimized Catalyst 056
Optimized Catalyst 056
 83 92  0  0  0  0  0  0  0  0999 V2000
    1.0391   -0.5905    0.0888 C   0  0  0  0  0  0  0  0  0  0  0  0
    0.0524   -0.3507    1.2488 C   0  0  0  0  0  0  0  0  0  0  0  0
    0.4161   -1.2345   -1.1163 C   0  0  0  0  0  0  0  0  0  0  0  0
   -0.4171    0.8970   -2.2562 C   0  0  0  0  0  0  0  0  0  0  0  0
   -0.2040   -0.5831   -2.1196 C   0  0  0  0  0  0  0  0  0  0  0  0
   -0.7660    1.6814   -0.9767 C   0  0  0  0  0  0  0  0  0  0  0  0
    0.6055   -2.3153   -1.1968 H   0  0  0  0  0  0  0  0  0  0  0  0
   -0.5294   -1.1473   -3.0110 H   0  0  0  0  0  0  0  0  0  0  0  0
    1.8721   -1.2410    0.4655 H   0  0  0  0  0  0  0  0  0  0  0  0
    1.5081    0.3794   -0.2221 H   0  0  0  0  0  0  0  0  0  0  0  0
    0.0154   -1.2753    1.8797 H   0  0  0  0  0  0  0  0  0  0  0  0
   -1.2351    2.6540   -1.2842 H   0  0  0  0  0  0  0  0  0  0  0  0
   -1.2247    1.0791   -3.0110 H   0  0  0  0  0  0  0  0  0  0  0  0
    0.5383    1.3255   -2.6754 H   0  0  0  0  0  0  0  0  0  0  0  0
   -1.7049    0.9507   -0.0640 C   0  0  0  0  0  0  0  0  0  0  0  0
   -1.3489    0.0543    0.8746 C   0  0  0  0  0  0  0  0  0  0  0  0
   -2.7535    1.2543   -0.1683 H   0  0  0  0  0  0  0  0  0  0  0  0
   -2.1177   -0.3518    1.5546 H   0  0  0  0  0  0  0  0  0  0  0  0
    0.4692    0.4757    1.8925 H   0  0  0  0  0  0  0  0  0  0  0  0
    0.1689    1.9400   -0.4157 H   0  0  0  0  0  0  0  0  0  0  0  0
   -2.3800   -2.2971   -0.6717 Rh  0  0  0  0  0  0  0  0  0  0  0  0
   -4.9783   -2.1106   -0.6261 P   0  0  0  0  0  0  0  0  0  0  0  0
   -2.1715   -4.8846   -0.3071 P   0  0  0  0  0  0  0  0  0  0  0  0
   -5.3801   -0.2701   -0.7635 C   0  0  1  0  0  0  0  0  0  0  0  0
   -5.0259    0.2702    0.1560 H   0  0  0  0  0  0  0  0  0  0  0  0
   -4.7608    0.2925   -2.0303 C   0  0  0  0  0  0  0  0  0  0  0  0
   -6.8609    0.0058   -0.9613 C   0  0  0  0  0  0  0  0  0  0  0  0
   -3.7010    0.2771   -2.2333 H   0  0  0  0  0  0  0  0  0  0  0  0
   -5.7327    0.8465   -2.7925 C   0  0  0  0  0  0  0  0  0  0  0  0
   -7.6002   -0.2539   -0.2220 H   0  0  0  0  0  0  0  0  0  0  0  0
   -7.0391    0.6720   -2.1247 C   0  0  0  0  0  0  0  0  0  0  0  0
   -5.6253    1.3521   -3.7411 H   0  0  0  0  0  0  0  0  0  0  0  0
   -7.9633    1.0443   -2.5416 H   0  0  0  0  0  0  0  0  0  0  0  0
   -5.7256   -3.1125   -2.0412 C   0  0  1  0  0  0  0  0  0  0  0  0
   -5.8067   -4.1950   -1.7669 H   0  0  0  0  0  0  0  0  0  0  0  0
   -7.0855   -2.6086   -2.4916 C   0  0  0  0  0  0  0  0  0  0  0  0
   -4.8683   -2.9158   -3.2796 C   0  0  0  0  0  0  0  0  0  0  0  0
   -7.9506   -2.6153   -1.8513 H   0  0  0  0  0  0  0  0  0  0  0  0
   -7.0181   -2.2398   -3.7913 C   0  0  0  0  0  0  0  0  0  0  0  0
   -3.8247   -3.1890   -3.3301 H   0  0  0  0  0  0  0  0  0  0  0  0
   -5.6370   -2.4259   -4.2810 C   0  0  0  0  0  0  0  0  0  0  0  0
   -7.8163   -1.8668   -4.4163 H   0  0  0  0  0  0  0  0  0  0  0  0
   -5.3414   -2.1947   -5.2946 H   0  0  0  0  0  0  0  0  0  0  0  0
   -5.6952   -2.7567    1.0007 C   0  0  1  0  0  0  0  0  0  0  0  0
   -5.4824   -3.8411    1.1513 H   0  0  0  0  0  0  0  0  0  0  0  0
   -5.1442   -1.9196    2.1411 C   0  0  0  0  0  0  0  0  0  0  0  0
```

```
   -7.1897   -2.5045    1.0896 C   0  0  0  0  0  0  0  0  0  0  0  0
   -4.1010   -1.9079    2.4108 H   0  0  0  0  0  0  0  0  0  0  0  0
   -6.1681   -1.3051    2.7788 C   0  0  0  0  0  0  0  0  0  0  0  0
   -7.8991   -2.9945    0.4447 H   0  0  0  0  0  0  0  0  0  0  0  0
   -7.4419   -1.6718    2.1260 C   0  0  0  0  0  0  0  0  0  0  0  0
   -6.1195   -0.6544    3.6412 H   0  0  0  0  0  0  0  0  0  0  0  0
   -8.4015   -1.3171    2.4753 H   0  0  0  0  0  0  0  0  0  0  0  0
   -2.3781   -5.2414    1.5387 C   0  0  1  0  0  0  0  0  0  0  0  0
   -3.4463   -5.1309    1.8547 H   0  0  0  0  0  0  0  0  0  0  0  0
   -1.8720   -6.6237    1.9102 C   0  0  0  0  0  0  0  0  0  0  0  0
   -1.4598   -4.3219    2.3211 C   0  0  0  0  0  0  0  0  0  0  0  0
   -2.3326   -7.5249    1.5368 H   0  0  0  0  0  0  0  0  0  0  0  0
   -0.8552   -6.5029    2.7945 C   0  0  0  0  0  0  0  0  0  0  0  0
   -1.5674   -3.2477    2.3263 H   0  0  0  0  0  0  0  0  0  0  0  0
   -0.5965   -5.0703    3.0483 C   0  0  0  0  0  0  0  0  0  0  0  0
   -0.2988   -7.2923    3.2796 H   0  0  0  0  0  0  0  0  0  0  0  0
    0.1692   -4.7287    3.7308 H   0  0  0  0  0  0  0  0  0  0  0  0
   -3.3638   -6.0091   -1.2604 C   0  0  1  0  0  0  0  0  0  0  0  0
   -3.6390   -5.5618   -2.2487 H   0  0  0  0  0  0  0  0  0  0  0  0
   -2.7727   -7.3925   -1.4806 C   0  0  0  0  0  0  0  0  0  0  0  0
   -4.5806   -6.3077   -0.4044 C   0  0  0  0  0  0  0  0  0  0  0  0
   -1.9124   -7.5573   -2.1101 H   0  0  0  0  0  0  0  0  0  0  0  0
   -3.5343   -8.3181   -0.8560 C   0  0  0  0  0  0  0  0  0  0  0  0
   -5.2897   -5.5698   -0.0901 H   0  0  0  0  0  0  0  0  0  0  0  0
   -4.6610   -7.6420   -0.1842 C   0  0  0  0  0  0  0  0  0  0  0  0
   -3.4018   -9.3905   -0.8447 H   0  0  0  0  0  0  0  0  0  0  0  0
   -5.4167   -8.1785    0.3721 H   0  0  0  0  0  0  0  0  0  0  0  0
   -0.3696   -5.2149   -0.7869 C   0  0  1  0  0  0  0  0  0  0  0  0
    0.3092   -4.5777   -0.1520 H   0  0  0  0  0  0  0  0  0  0  0  0
    0.0848   -6.6581   -0.6423 C   0  0  0  0  0  0  0  0  0  0  0  0
   -0.1675   -4.9480   -2.2677 C   0  0  0  0  0  0  0  0  0  0  0  0
    0.0755   -7.1746    0.3036 H   0  0  0  0  0  0  0  0  0  0  0  0
    0.5539   -7.1000   -1.8315 C   0  0  0  0  0  0  0  0  0  0  0  0
   -0.3868   -4.0049   -2.7422 H   0  0  0  0  0  0  0  0  0  0  0  0
    0.3882   -6.0391   -2.8456 C   0  0  0  0  0  0  0  0  0  0  0  0
    0.9960   -8.0602   -2.0557 H   0  0  0  0  0  0  0  0  0  0  0  0
    0.6882   -6.1649   -3.8768 H   0  0  0  0  0  0  0  0  0  0  0  0
  1  2  1  0  0  0  0
  1  3  1  0  0  0  0
  1  9  1  0  0  0  0
  1 10  1  0  0  0  0
  2 11  1  0  0  0  0
  2 16  1  0  0  0  0
  2 19  1  0  0  0  0
  3  5  2  0  0  0  0
  3  7  1  0  0  0  0
  3 21  1  0  0  0  0
  4  5  1  0  0  0  0
  4  6  1  0  0  0  0
  4 13  1  0  0  0  0
  4 14  1  0  0  0  0
  5  8  1  0  0  0  0
  5 21  1  0  0  0  0
  6 12  1  0  0  0  0
  6 15  1  0  0  0  0
  6 20  1  0  0  0  0
 15 16  2  0  0  0  0
 15 17  1  0  0  0  0
 15 21  1  0  0  0  0
 16 18  1  0  0  0  0
 16 21  1  0  0  0  0
 21 23  1  0  0  0  0
 21 22  1  0  0  0  0
 22 24  1  0  0  0  0
 22 34  1  0  0  0  0
```

```
 22 44  1  0  0  0  0
 23 54  1  0  0  0  0
 23 64  1  0  0  0  0
 23 74  1  0  0  0  0
 24 25  1  1  0  0  0
 24 26  1  0  0  0  0
 24 27  1  0  0  0  0
 26 28  1  0  0  0  0
 26 29  2  0  0  0  0
 27 30  1  0  0  0  0
 27 31  2  0  0  0  0
 29 31  1  0  0  0  0
 29 32  1  0  0  0  0
 31 33  1  0  0  0  0
 34 35  1  1  0  0  0
 34 36  1  0  0  0  0
 34 37  1  0  0  0  0
 36 38  1  0  0  0  0
 36 39  2  0  0  0  0
 37 40  1  0  0  0  0
 37 41  2  0  0  0  0
 39 41  1  0  0  0  0
 39 42  1  0  0  0  0
 41 43  1  0  0  0  0
 44 45  1  1  0  0  0
 44 46  1  0  0  0  0
 44 47  1  0  0  0  0
 46 48  1  0  0  0  0
 46 49  2  0  0  0  0
 47 50  1  0  0  0  0
 47 51  2  0  0  0  0
 49 51  1  0  0  0  0
 49 52  1  0  0  0  0
 51 53  1  0  0  0  0
 54 55  1  1  0  0  0
 54 56  1  0  0  0  0
 54 57  1  0  0  0  0
 56 58  1  0  0  0  0
 56 59  2  0  0  0  0
 57 60  1  0  0  0  0
 57 61  2  0  0  0  0
 59 61  1  0  0  0  0
 59 62  1  0  0  0  0
 61 63  1  0  0  0  0
 64 65  1  6  0  0  0
 64 66  1  0  0  0  0
 64 67  1  0  0  0  0
 66 68  1  0  0  0  0
 66 69  2  0  0  0  0
 67 70  1  0  0  0  0
 67 71  2  0  0  0  0
 69 71  1  0  0  0  0
 69 72  1  0  0  0  0
 71 73  1  0  0  0  0
 74 75  1  1  0  0  0
 74 76  1  0  0  0  0
 74 77  1  0  0  0  0
 76 78  1  0  0  0  0
 76 79  2  0  0  0  0
 77 80  1  0  0  0  0
 77 81  2  0  0  0  0
 79 81  1  0  0  0  0
 79 82  1  0  0  0  0
 81 83  1  0  0  0  0
M  END
```

```
$$$$
mol057
Optimized Catalyst 057
Optimized Catalyst 057
 71 80  0  0  0  0  0  0  0  0999 V2000
    0.6832   -0.0052    0.5492 C   0  0  0  0  0  0  0  0  0  0  0  0
   -0.6441    0.7589    0.7219 C   0  0  0  0  0  0  0  0  0  0  0  0
    0.5205   -1.3935   -0.0001 C   0  0  0  0  0  0  0  0  0  0  0  0
    0.5872   -0.7626   -2.4765 C   0  0  0  0  0  0  0  0  0  0  0  0
    0.4772   -1.7099   -1.3122 C   0  0  0  0  0  0  0  0  0  0  0  0
   -0.1631    0.5789   -2.3642 C   0  0  0  0  0  0  0  0  0  0  0  0
    0.5345   -2.1852    0.7649 H   0  0  0  0  0  0  0  0  0  0  0  0
    0.4988   -2.7703   -1.6209 H   0  0  0  0  0  0  0  0  0  0  0  0
    1.1831   -0.0652    1.5533 H   0  0  0  0  0  0  0  0  0  0  0  0
    1.3751    0.5722   -0.1184 H   0  0  0  0  0  0  0  0  0  0  0  0
   -1.0790    0.4871    1.7195 H   0  0  0  0  0  0  0  0  0  0  0  0
   -0.3189    0.9763   -3.4036 H   0  0  0  0  0  0  0  0  0  0  0  0
    0.2350   -1.2842   -3.4047 H   0  0  0  0  0  0  0  0  0  0  0  0
    1.6838   -0.5381   -2.6137 H   0  0  0  0  0  0  0  0  0  0  0  0
   -1.4936    0.4811   -1.6754 C   0  0  0  0  0  0  0  0  0  0  0  0
   -1.6907    0.5585   -0.3416 C   0  0  0  0  0  0  0  0  0  0  0  0
   -2.3538    0.4316   -2.3613 H   0  0  0  0  0  0  0  0  0  0  0  0
   -2.7197    0.6309    0.0527 H   0  0  0  0  0  0  0  0  0  0  0  0
   -0.4108    1.8617    0.7489 H   0  0  0  0  0  0  0  0  0  0  0  0
    0.4712    1.3315   -1.8262 H   0  0  0  0  0  0  0  0  0  0  0  0
   -2.2713   -2.2250   -0.6262 Ir  0  0  0  0  0  0  0  0  0  0  0  0
   -4.8601   -2.1286   -0.8638 P   0  0  0  0  0  0  0  0  0  0  0  0
   -2.1428   -4.7857   -0.1783 P   0  0  0  0  0  0  0  0  0  0  0  0
   -0.3375   -5.2622   -0.1692 C   0  0  0  0  0  0  0  0  0  0  0  0
    0.1081   -6.6852   -0.5050 C   0  0  0  0  0  0  0  0  0  0  0  0
    0.4328   -5.5555   -1.4507 C   0  0  0  0  0  0  0  0  0  0  0  0
    0.2087   -4.7616    0.6521 H   0  0  0  0  0  0  0  0  0  0  0  0
   -0.6192   -7.4492   -0.8114 H   0  0  0  0  0  0  0  0  0  0  0  0
    0.9196   -7.1130    0.1091 H   0  0  0  0  0  0  0  0  0  0  0  0
    1.4770   -5.2004   -1.5040 H   0  0  0  0  0  0  0  0  0  0  0  0
   -0.0614   -5.5211   -2.4314 H   0  0  0  0  0  0  0  0  0  0  0  0
   -2.9985   -5.8081   -1.4842 C   0  0  0  0  0  0  0  0  0  0  0  0
   -3.3017   -7.2855   -1.2362 C   0  0  0  0  0  0  0  0  0  0  0  0
   -4.4348   -6.2949   -1.3522 C   0  0  0  0  0  0  0  0  0  0  0  0
   -2.6431   -5.5420   -2.4976 H   0  0  0  0  0  0  0  0  0  0  0  0
   -3.0985   -7.7458   -0.2577 H   0  0  0  0  0  0  0  0  0  0  0  0
   -3.1159   -7.9849   -2.0690 H   0  0  0  0  0  0  0  0  0  0  0  0
   -5.0501   -6.3021   -2.2677 H   0  0  0  0  0  0  0  0  0  0  0  0
   -5.0328   -6.0826   -0.4580 H   0  0  0  0  0  0  0  0  0  0  0  0
   -2.8628   -5.2446    1.4767 C   0  0  0  0  0  0  0  0  0  0  0  0
   -2.2174   -6.3317    2.3329 C   0  0  0  0  0  0  0  0  0  0  0  0
   -2.1630   -4.8913    2.7825 C   0  0  0  0  0  0  0  0  0  0  0  0
   -3.9654   -5.2041    1.4664 H   0  0  0  0  0  0  0  0  0  0  0  0
   -1.2913   -6.8275    2.0067 H   0  0  0  0  0  0  0  0  0  0  0  0
   -2.8939   -7.0288    2.8550 H   0  0  0  0  0  0  0  0  0  0  0  0
   -2.8035   -4.5747    3.6235 H   0  0  0  0  0  0  0  0  0  0  0  0
   -1.1979   -4.3657    2.7882 H   0  0  0  0  0  0  0  0  0  0  0  0
   -5.7788   -3.1219    0.4243 C   0  0  0  0  0  0  0  0  0  0  0  0
   -5.6718   -2.8219    1.9119 C   0  0  0  0  0  0  0  0  0  0  0  0
   -6.9596   -2.5203    1.1836 C   0  0  0  0  0  0  0  0  0  0  0  0
   -5.8884   -4.1721    0.1057 H   0  0  0  0  0  0  0  0  0  0  0  0
   -5.0530   -1.9892    2.2768 H   0  0  0  0  0  0  0  0  0  0  0  0
   -5.6496   -3.6792    2.6048 H   0  0  0  0  0  0  0  0  0  0  0  0
   -7.8319   -3.1748    1.3522 H   0  0  0  0  0  0  0  0  0  0  0  0
   -7.2620   -1.4751    1.0263 H   0  0  0  0  0  0  0  0  0  0  0  0
   -5.3648   -0.3488   -0.6247 C   0  0  0  0  0  0  0  0  0  0  0  0
   -5.2887    0.7090   -1.7164 C   0  0  0  0  0  0  0  0  0  0  0  0
   -6.6378    0.2553   -1.2129 C   0  0  0  0  0  0  0  0  0  0  0  0
   -5.1184   -0.0243    0.4083 H   0  0  0  0  0  0  0  0  0  0  0  0
   -4.9716    0.4505   -2.7357 H   0  0  0  0  0  0  0  0  0  0  0  0
```

```
   -4.9309    1.7119   -1.4223 H   0  0  0  0  0  0  0  0  0  0  0  0
   -7.2123    0.9366   -0.5599 H   0  0  0  0  0  0  0  0  0  0  0  0
   -7.3013   -0.3293   -1.8621 H   0  0  0  0  0  0  0  0  0  0  0  0
   -5.3786   -2.9179   -2.4755 C   0  0  0  0  0  0  0  0  0  0  0  0
   -6.8551   -3.1232   -2.8072 C   0  0  0  0  0  0  0  0  0  0  0  0
   -6.0172   -2.1982   -3.6553 C   0  0  0  0  0  0  0  0  0  0  0  0
   -4.6978   -3.7511   -2.7341 H   0  0  0  0  0  0  0  0  0  0  0  0
   -7.6371   -2.7057   -2.1572 H   0  0  0  0  0  0  0  0  0  0  0  0
   -7.1497   -4.1025   -3.2195 H   0  0  0  0  0  0  0  0  0  0  0  0
   -5.7107   -2.5347   -4.6622 H   0  0  0  0  0  0  0  0  0  0  0  0
   -6.2132   -1.1196   -3.6403 H   0  0  0  0  0  0  0  0  0  0  0  0
  1  2  1  0  0  0  0
  1  9  1  0  0  0  0
  1  3  1  0  0  0  0
  1 10  1  0  0  0  0
  2 19  1  0  0  0  0
  2 11  1  0  0  0  0
  2 16  1  0  0  0  0
  3  7  1  0  0  0  0
  3  5  2  0  0  0  0
  3 21  1  0  0  0  0
  4  6  1  0  0  0  0
  4  5  1  0  0  0  0
  4 13  1  0  0  0  0
  4 14  1  0  0  0  0
  5  8  1  0  0  0  0
  5 21  1  0  0  0  0
  6 20  1  0  0  0  0
  6 15  1  0  0  0  0
  6 12  1  0  0  0  0
 15 16  2  0  0  0  0
 15 17  1  0  0  0  0
 15 21  1  0  0  0  0
 16 18  1  0  0  0  0
 16 21  1  0  0  0  0
 21 23  1  0  0  0  0
 21 22  1  0  0  0  0
 22 56  1  0  0  0  0
 22 48  1  0  0  0  0
 22 64  1  0  0  0  0
 23 24  1  0  0  0  0
 23 40  1  0  0  0  0
 23 32  1  0  0  0  0
 24 27  1  0  0  0  0
 24 25  1  0  0  0  0
 24 26  1  0  0  0  0
 25 29  1  0  0  0  0
 25 26  1  0  0  0  0
 25 28  1  0  0  0  0
 26 30  1  0  0  0  0
 26 31  1  0  0  0  0
 32 34  1  0  0  0  0
 32 33  1  0  0  0  0
 32 35  1  0  0  0  0
 33 36  1  0  0  0  0
 33 34  1  0  0  0  0
 33 37  1  0  0  0  0
 34 39  1  0  0  0  0
 34 38  1  0  0  0  0
 40 41  1  0  0  0  0
 40 42  1  0  0  0  0
 40 43  1  0  0  0  0
 41 42  1  0  0  0  0
 41 45  1  0  0  0  0
 41 44  1  0  0  0  0
```

```
 42 47  1  0  0  0  0
 42 46  1  0  0  0  0
 48 50  1  0  0  0  0
 48 49  1  0  0  0  0
 48 51  1  0  0  0  0
 49 52  1  0  0  0  0
 49 53  1  0  0  0  0
 49 50  1  0  0  0  0
 50 54  1  0  0  0  0
 50 55  1  0  0  0  0
 56 59  1  0  0  0  0
 56 57  1  0  0  0  0
 56 58  1  0  0  0  0
 57 61  1  0  0  0  0
 57 58  1  0  0  0  0
 57 60  1  0  0  0  0
 58 62  1  0  0  0  0
 58 63  1  0  0  0  0
 64 65  1  0  0  0  0
 64 66  1  0  0  0  0
 64 67  1  0  0  0  0
 65 68  1  0  0  0  0
 65 66  1  0  0  0  0
 65 69  1  0  0  0  0
 66 71  1  0  0  0  0
 66 70  1  0  0  0  0
M  END
$$$$
mol058
Optimized Catalyst 058
Optimized Catalyst 058
 83 92  0  0  0  0  0  0  0  0999 V2000
    1.0391   -0.5905    0.0888 C   0  0  0  0  0  0  0  0  0  0  0  0
    0.0524   -0.3507    1.2488 C   0  0  0  0  0  0  0  0  0  0  0  0
    0.4161   -1.2345   -1.1163 C   0  0  0  0  0  0  0  0  0  0  0  0
   -0.4171    0.8970   -2.2562 C   0  0  0  0  0  0  0  0  0  0  0  0
   -0.2040   -0.5831   -2.1196 C   0  0  0  0  0  0  0  0  0  0  0  0
   -0.7660    1.6814   -0.9767 C   0  0  0  0  0  0  0  0  0  0  0  0
    0.6055   -2.3153   -1.1968 H   0  0  0  0  0  0  0  0  0  0  0  0
   -0.5294   -1.1473   -3.0110 H   0  0  0  0  0  0  0  0  0  0  0  0
    1.8721   -1.2410    0.4655 H   0  0  0  0  0  0  0  0  0  0  0  0
    1.5081    0.3794   -0.2221 H   0  0  0  0  0  0  0  0  0  0  0  0
    0.0154   -1.2753    1.8797 H   0  0  0  0  0  0  0  0  0  0  0  0
   -1.2351    2.6540   -1.2842 H   0  0  0  0  0  0  0  0  0  0  0  0
   -1.2247    1.0791   -3.0110 H   0  0  0  0  0  0  0  0  0  0  0  0
    0.5383    1.3255   -2.6754 H   0  0  0  0  0  0  0  0  0  0  0  0
   -1.7049    0.9507   -0.0640 C   0  0  0  0  0  0  0  0  0  0  0  0
   -1.3489    0.0543    0.8746 C   0  0  0  0  0  0  0  0  0  0  0  0
   -2.7535    1.2543   -0.1683 H   0  0  0  0  0  0  0  0  0  0  0  0
   -2.1177   -0.3518    1.5546 H   0  0  0  0  0  0  0  0  0  0  0  0
    0.4692    0.4757    1.8925 H   0  0  0  0  0  0  0  0  0  0  0  0
    0.1689    1.9400   -0.4157 H   0  0  0  0  0  0  0  0  0  0  0  0
   -2.3800   -2.2971   -0.6717 Ir  0  0  0  0  0  0  0  0  0  0  0  0
   -4.9783   -2.1106   -0.6261 P   0  0  0  0  0  0  0  0  0  0  0  0
   -2.1715   -4.8846   -0.3071 P   0  0  0  0  0  0  0  0  0  0  0  0
   -5.3801   -0.2701   -0.7635 C   0  0  1  0  0  0  0  0  0  0  0  0
   -5.0259    0.2702    0.1560 H   0  0  0  0  0  0  0  0  0  0  0  0
   -4.7608    0.2925   -2.0303 C   0  0  0  0  0  0  0  0  0  0  0  0
   -6.8609    0.0058   -0.9613 C   0  0  0  0  0  0  0  0  0  0  0  0
   -3.7010    0.2771   -2.2333 H   0  0  0  0  0  0  0  0  0  0  0  0
   -5.7327    0.8465   -2.7925 C   0  0  0  0  0  0  0  0  0  0  0  0
   -7.6002   -0.2539   -0.2220 H   0  0  0  0  0  0  0  0  0  0  0  0
   -7.0391    0.6720   -2.1247 C   0  0  0  0  0  0  0  0  0  0  0  0
   -5.6253    1.3521   -3.7411 H   0  0  0  0  0  0  0  0  0  0  0  0
   -7.9633    1.0443   -2.5416 H   0  0  0  0  0  0  0  0  0  0  0  0
```

```
  -5.7256   -3.1125   -2.0412 C   0  0  1  0  0  0  0  0  0  0  0  0
  -5.8067   -4.1950   -1.7669 H   0  0  0  0  0  0  0  0  0  0  0  0
  -7.0855   -2.6086   -2.4916 C   0  0  0  0  0  0  0  0  0  0  0  0
  -4.8683   -2.9158   -3.2796 C   0  0  0  0  0  0  0  0  0  0  0  0
  -7.9506   -2.6153   -1.8513 H   0  0  0  0  0  0  0  0  0  0  0  0
  -7.0181   -2.2398   -3.7913 C   0  0  0  0  0  0  0  0  0  0  0  0
  -3.8247   -3.1890   -3.3301 H   0  0  0  0  0  0  0  0  0  0  0  0
  -5.6370   -2.4259   -4.2810 C   0  0  0  0  0  0  0  0  0  0  0  0
  -7.8163   -1.8668   -4.4163 H   0  0  0  0  0  0  0  0  0  0  0  0
  -5.3414   -2.1947   -5.2946 H   0  0  0  0  0  0  0  0  0  0  0  0
  -5.6952   -2.7567    1.0007 C   0  0  1  0  0  0  0  0  0  0  0  0
  -5.4824   -3.8411    1.1513 H   0  0  0  0  0  0  0  0  0  0  0  0
  -5.1442   -1.9196    2.1411 C   0  0  0  0  0  0  0  0  0  0  0  0
  -7.1897   -2.5045    1.0896 C   0  0  0  0  0  0  0  0  0  0  0  0
  -4.1010   -1.9079    2.4108 H   0  0  0  0  0  0  0  0  0  0  0  0
  -6.1681   -1.3051    2.7788 C   0  0  0  0  0  0  0  0  0  0  0  0
  -7.8991   -2.9945    0.4447 H   0  0  0  0  0  0  0  0  0  0  0  0
  -7.4419   -1.6718    2.1260 C   0  0  0  0  0  0  0  0  0  0  0  0
  -6.1195   -0.6544    3.6412 H   0  0  0  0  0  0  0  0  0  0  0  0
  -8.4015   -1.3171    2.4753 H   0  0  0  0  0  0  0  0  0  0  0  0
  -2.3781   -5.2414    1.5387 C   0  0  1  0  0  0  0  0  0  0  0  0
  -3.4463   -5.1309    1.8547 H   0  0  0  0  0  0  0  0  0  0  0  0
  -1.8720   -6.6237    1.9102 C   0  0  0  0  0  0  0  0  0  0  0  0
  -1.4598   -4.3219    2.3211 C   0  0  0  0  0  0  0  0  0  0  0  0
  -2.3326   -7.5249    1.5368 H   0  0  0  0  0  0  0  0  0  0  0  0
  -0.8552   -6.5029    2.7945 C   0  0  0  0  0  0  0  0  0  0  0  0
  -1.5674   -3.2477    2.3263 H   0  0  0  0  0  0  0  0  0  0  0  0
  -0.5965   -5.0703    3.0483 C   0  0  0  0  0  0  0  0  0  0  0  0
  -0.2988   -7.2923    3.2796 H   0  0  0  0  0  0  0  0  0  0  0  0
   0.1692   -4.7287    3.7308 H   0  0  0  0  0  0  0  0  0  0  0  0
  -3.3638   -6.0091   -1.2604 C   0  0  1  0  0  0  0  0  0  0  0  0
  -3.6390   -5.5618   -2.2487 H   0  0  0  0  0  0  0  0  0  0  0  0
  -2.7727   -7.3925   -1.4806 C   0  0  0  0  0  0  0  0  0  0  0  0
  -4.5806   -6.3077   -0.4044 C   0  0  0  0  0  0  0  0  0  0  0  0
  -1.9124   -7.5573   -2.1101 H   0  0  0  0  0  0  0  0  0  0  0  0
  -3.5343   -8.3181   -0.8560 C   0  0  0  0  0  0  0  0  0  0  0  0
  -5.2897   -5.5698   -0.0901 H   0  0  0  0  0  0  0  0  0  0  0  0
  -4.6610   -7.6420   -0.1842 C   0  0  0  0  0  0  0  0  0  0  0  0
  -3.4018   -9.3905   -0.8447 H   0  0  0  0  0  0  0  0  0  0  0  0
  -5.4167   -8.1785    0.3721 H   0  0  0  0  0  0  0  0  0  0  0  0
  -0.3696   -5.2149   -0.7869 C   0  0  1  0  0  0  0  0  0  0  0  0
   0.3092   -4.5777   -0.1520 H   0  0  0  0  0  0  0  0  0  0  0  0
   0.0848   -6.6581   -0.6423 C   0  0  0  0  0  0  0  0  0  0  0  0
  -0.1675   -4.9480   -2.2677 C   0  0  0  0  0  0  0  0  0  0  0  0
   0.0755   -7.1746    0.3036 H   0  0  0  0  0  0  0  0  0  0  0  0
   0.5539   -7.1000   -1.8315 C   0  0  0  0  0  0  0  0  0  0  0  0
  -0.3868   -4.0049   -2.7422 H   0  0  0  0  0  0  0  0  0  0  0  0
   0.3882   -6.0391   -2.8456 C   0  0  0  0  0  0  0  0  0  0  0  0
   0.9960   -8.0602   -2.0557 H   0  0  0  0  0  0  0  0  0  0  0  0
   0.6882   -6.1649   -3.8768 H   0  0  0  0  0  0  0  0  0  0  0  0
  1  2  1  0  0  0  0
  1  3  1  0  0  0  0
  1  9  1  0  0  0  0
  1 10  1  0  0  0  0
  2 11  1  0  0  0  0
  2 16  1  0  0  0  0
  2 19  1  0  0  0  0
  3  5  2  0  0  0  0
  3  7  1  0  0  0  0
  3 21  1  0  0  0  0
  4  5  1  0  0  0  0
  4  6  1  0  0  0  0
  4 13  1  0  0  0  0
  4 14  1  0  0  0  0
  5  8  1  0  0  0  0
```

```
  5 21  1  0  0  0  0
  6 12  1  0  0  0  0
  6 15  1  0  0  0  0
  6 20  1  0  0  0  0
 15 16  2  0  0  0  0
 15 17  1  0  0  0  0
 15 21  1  0  0  0  0
 16 18  1  0  0  0  0
 16 21  1  0  0  0  0
 21 23  1  0  0  0  0
 21 22  1  0  0  0  0
 22 24  1  0  0  0  0
 22 34  1  0  0  0  0
 22 44  1  0  0  0  0
 23 54  1  0  0  0  0
 23 64  1  0  0  0  0
 23 74  1  0  0  0  0
 24 25  1  1  0  0  0
 24 26  1  0  0  0  0
 24 27  1  0  0  0  0
 26 28  1  0  0  0  0
 26 29  2  0  0  0  0
 27 30  1  0  0  0  0
 27 31  2  0  0  0  0
 29 31  1  0  0  0  0
 29 32  1  0  0  0  0
 31 33  1  0  0  0  0
 34 35  1  1  0  0  0
 34 36  1  0  0  0  0
 34 37  1  0  0  0  0
 36 38  1  0  0  0  0
 36 39  2  0  0  0  0
 37 40  1  0  0  0  0
 37 41  2  0  0  0  0
 39 41  1  0  0  0  0
 39 42  1  0  0  0  0
 41 43  1  0  0  0  0
 44 45  1  1  0  0  0
 44 46  1  0  0  0  0
 44 47  1  0  0  0  0
 46 48  1  0  0  0  0
 46 49  2  0  0  0  0
 47 50  1  0  0  0  0
 47 51  2  0  0  0  0
 49 51  1  0  0  0  0
 49 52  1  0  0  0  0
 51 53  1  0  0  0  0
 54 55  1  1  0  0  0
 54 56  1  0  0  0  0
 54 57  1  0  0  0  0
 56 58  1  0  0  0  0
 56 59  2  0  0  0  0
 57 60  1  0  0  0  0
 57 61  2  0  0  0  0
 59 61  1  0  0  0  0
 59 62  1  0  0  0  0
 61 63  1  0  0  0  0
 64 65  1  6  0  0  0
 64 66  1  0  0  0  0
 64 67  1  0  0  0  0
 66 68  1  0  0  0  0
 66 69  2  0  0  0  0
 67 70  1  0  0  0  0
 67 71  2  0  0  0  0
 69 71  1  0  0  0  0
```

```
 69 72  1  0  0  0  0
 71 73  1  0  0  0  0
 74 75  1  1  0  0  0
 74 76  1  0  0  0  0
 74 77  1  0  0  0  0
 76 78  1  0  0  0  0
 76 79  2  0  0  0  0
 77 80  1  0  0  0  0
 77 81  2  0  0  0  0
 79 81  1  0  0  0  0
 79 82  1  0  0  0  0
 81 83  1  0  0  0  0
M  END
$$$$
mol060
Optimized Catalyst 060
Optimized Catalyst 060
 68 75  0  0  0  0  0  0  0  0999 V2000
   -2.4349   -4.6372   -0.7307 P   0  0  0  0  0  0  0  0  0  0  0  0
   -0.4181    0.6028   -0.9928 C   0  0  0  0  0  0  0  0  0  0  0  0
    0.6878   -0.3444   -0.4864 C   0  0  0  0  0  0  0  0  0  0  0  0
   -1.7494    0.5379   -0.2914 C   0  0  0  0  0  0  0  0  0  0  0  0
   -0.8854    0.3570    2.0845 C   0  0  0  0  0  0  0  0  0  0  0  0
   -1.9638    0.4294    1.0393 C   0  0  0  0  0  0  0  0  0  0  0  0
   -0.3844   -1.0729    2.3673 C   0  0  0  0  0  0  0  0  0  0  0  0
   -2.6074    0.7219   -0.9674 H   0  0  0  0  0  0  0  0  0  0  0  0
   -2.9901    0.4904    1.4431 H   0  0  0  0  0  0  0  0  0  0  0  0
   -0.5725    0.4116   -2.0887 H   0  0  0  0  0  0  0  0  0  0  0  0
   -0.0443    1.6630   -0.8916 H   0  0  0  0  0  0  0  0  0  0  0  0
    1.4375   -0.4691   -1.3163 H   0  0  0  0  0  0  0  0  0  0  0  0
   -1.0625   -1.5392    3.1333 H   0  0  0  0  0  0  0  0  0  0  0  0
   -1.2833    0.7812    3.0474 H   0  0  0  0  0  0  0  0  0  0  0  0
   -0.0312    1.0162    1.7786 H   0  0  0  0  0  0  0  0  0  0  0  0
   -0.2551   -2.0135    1.1908 C   0  0  0  0  0  0  0  0  0  0  0  0
    0.2198   -1.7063   -0.0500 C   0  0  0  0  0  0  0  0  0  0  0  0
   -0.3329   -3.0802    1.4984 H   0  0  0  0  0  0  0  0  0  0  0  0
    0.4405   -2.5152   -0.7765 H   0  0  0  0  0  0  0  0  0  0  0  0
    1.2371    0.1333    0.3702 H   0  0  0  0  0  0  0  0  0  0  0  0
    0.6414   -0.9973    2.8333 H   0  0  0  0  0  0  0  0  0  0  0  0
   -4.2492   -2.1087   -0.6396 N   0  0  0  0  0  0  0  0  0  0  0  0
   -5.3614   -2.1214   -0.9992 C   0  0  0  0  0  0  0  0  0  0  0  0
   -6.7084   -2.1551   -1.4435 C   0  0  0  0  0  0  0  0  0  0  0  0
   -7.2231   -3.3511   -1.9957 C   0  0  0  0  0  0  0  0  0  0  0  0
   -7.5198   -1.0024   -1.3336 C   0  0  0  0  0  0  0  0  0  0  0  0
   -8.5398   -3.3974   -2.4391 C   0  0  0  0  0  0  0  0  0  0  0  0
   -6.5851   -4.2368   -2.0742 H   0  0  0  0  0  0  0  0  0  0  0  0
   -8.8385   -1.0414   -1.7743 C   0  0  0  0  0  0  0  0  0  0  0  0
   -7.1135   -0.0826   -0.9036 H   0  0  0  0  0  0  0  0  0  0  0  0
   -9.3239   -2.2395   -2.3192 C   0  0  0  0  0  0  0  0  0  0  0  0
   -8.9566   -4.3138   -2.8686 H   0  0  0  0  0  0  0  0  0  0  0  0
   -9.4826   -0.1581   -1.6970 H   0  0  0  0  0  0  0  0  0  0  0  0
   -0.7793   -5.3261   -1.1919 C   0  0  0  0  0  0  0  0  0  0  0  0
    0.1115   -5.6782   -0.1693 C   0  0  0  0  0  0  0  0  0  0  0  0
   -0.4514   -5.5315   -2.5340 C   0  0  0  0  0  0  0  0  0  0  0  0
    1.3528   -6.2255   -0.5024 C   0  0  0  0  0  0  0  0  0  0  0  0
   -0.1381   -5.5599    0.8885 H   0  0  0  0  0  0  0  0  0  0  0  0
    0.7961   -6.0803   -2.8576 C   0  0  0  0  0  0  0  0  0  0  0  0
   -1.1405   -5.2908   -3.3486 H   0  0  0  0  0  0  0  0  0  0  0  0
    1.6969   -6.4250   -1.8461 C   0  0  0  0  0  0  0  0  0  0  0  0
    2.0557   -6.5073    0.2858 H   0  0  0  0  0  0  0  0  0  0  0  0
    1.0617   -6.2463   -3.9050 H   0  0  0  0  0  0  0  0  0  0  0  0
    2.6693   -6.8566   -2.1020 H   0  0  0  0  0  0  0  0  0  0  0  0
   -3.0392   -5.6295    0.7116 C   0  0  0  0  0  0  0  0  0  0  0  0
   -2.8069   -7.0067    0.7585 C   0  0  0  0  0  0  0  0  0  0  0  0
   -3.7363   -4.9754    1.7327 C   0  0  0  0  0  0  0  0  0  0  0  0
```

```
  -3.2984   -7.7428    1.8435 C   0  0  0  0  0  0  0  0  0  0  0  0
  -2.2454   -7.5294   -0.0226 H   0  0  0  0  0  0  0  0  0  0  0  0
  -4.2257   -5.7191    2.8105 C   0  0  0  0  0  0  0  0  0  0  0  0
  -3.9078   -3.8869    1.7194 H   0  0  0  0  0  0  0  0  0  0  0  0
  -4.0089   -7.1024    2.8636 C   0  0  0  0  0  0  0  0  0  0  0  0
  -3.1239   -8.8209    1.8937 H   0  0  0  0  0  0  0  0  0  0  0  0
  -4.7756   -5.2242    3.6146 H   0  0  0  0  0  0  0  0  0  0  0  0
  -4.3934   -7.6817    3.7088 H   0  0  0  0  0  0  0  0  0  0  0  0
  -3.5833   -4.9616   -2.1535 C   0  0  0  0  0  0  0  0  0  0  0  0
  -4.4342   -6.0673   -2.1257 C   0  0  0  0  0  0  0  0  0  0  0  0
  -3.5435   -4.1096   -3.2650 C   0  0  0  0  0  0  0  0  0  0  0  0
  -5.2515   -6.3306   -3.2340 C   0  0  0  0  0  0  0  0  0  0  0  0
  -4.4900   -6.7422   -1.2656 H   0  0  0  0  0  0  0  0  0  0  0  0
  -4.3601   -4.3810   -4.3654 C   0  0  0  0  0  0  0  0  0  0  0  0
  -2.8882   -3.2322   -3.3053 H   0  0  0  0  0  0  0  0  0  0  0  0
  -5.2103   -5.4953   -4.3528 C   0  0  0  0  0  0  0  0  0  0  0  0
  -5.9128   -7.2010   -3.2254 H   0  0  0  0  0  0  0  0  0  0  0  0
  -4.3321   -3.7293   -5.2425 H   0  0  0  0  0  0  0  0  0  0  0  0
  -5.8386   -5.7109   -5.2222 H   0  0  0  0  0  0  0  0  0  0  0  0
  -2.3987   -2.1190   -0.0751 Ir  0  0  0  0  0  0  0  0  0  0  0  0
 -10.5798   -2.2805   -2.7404 F   0  0  0  0  0  0  0  0  0  0  0  0
 1 34  1  0  0  0  0
 1 45  1  0  0  0  0
 1 56  1  0  0  0  0
 1 67  1  0  0  0  0
 2  3  1  0  0  0  0
 2  4  1  0  0  0  0
 2 10  1  0  0  0  0
 2 11  1  0  0  0  0
 3 12  1  0  0  0  0
 3 17  1  0  0  0  0
 3 20  1  0  0  0  0
 4  6  2  0  0  0  0
 4  8  1  0  0  0  0
 4 67  1  0  0  0  0
 5  6  1  0  0  0  0
 5  7  1  0  0  0  0
 5 14  1  0  0  0  0
 5 15  1  0  0  0  0
 6  9  1  0  0  0  0
 6 67  1  0  0  0  0
 7 13  1  0  0  0  0
 7 16  1  0  0  0  0
 7 21  1  0  0  0  0
16 17  2  0  0  0  0
16 18  1  0  0  0  0
16 67  1  0  0  0  0
17 19  1  0  0  0  0
17 67  1  0  0  0  0
22 23  3  0  0  0  0
22 67  1  0  0  0  0
23 24  1  0  0  0  0
24 25  2  0  0  0  0
24 26  1  0  0  0  0
25 27  1  0  0  0  0
25 28  1  0  0  0  0
26 29  2  0  0  0  0
26 30  1  0  0  0  0
27 31  2  0  0  0  0
27 32  1  0  0  0  0
29 31  1  0  0  0  0
29 33  1  0  0  0  0
31 68  1  0  0  0  0
34 35  2  0  0  0  0
34 36  1  0  0  0  0
```

```
 35 37  1  0  0  0  0
 35 38  1  0  0  0  0
 36 39  2  0  0  0  0
 36 40  1  0  0  0  0
 37 41  2  0  0  0  0
 37 42  1  0  0  0  0
 39 41  1  0  0  0  0
 39 43  1  0  0  0  0
 41 44  1  0  0  0  0
 45 46  2  0  0  0  0
 45 47  1  0  0  0  0
 46 48  1  0  0  0  0
 46 49  1  0  0  0  0
 47 50  2  0  0  0  0
 47 51  1  0  0  0  0
 48 52  2  0  0  0  0
 48 53  1  0  0  0  0
 50 52  1  0  0  0  0
 50 54  1  0  0  0  0
 52 55  1  0  0  0  0
 56 57  2  0  0  0  0
 56 58  1  0  0  0  0
 57 59  1  0  0  0  0
 57 60  1  0  0  0  0
 58 61  2  0  0  0  0
 58 62  1  0  0  0  0
 59 63  2  0  0  0  0
 59 64  1  0  0  0  0
 61 63  1  0  0  0  0
 61 65  1  0  0  0  0
 63 66  1  0  0  0  0
M  END
$$$$
mol061
Optimized Catalyst 061
Optimized Catalyst 061
 68 75  0  0  0  0  0  0  0  0999 V2000
   -2.4350   -4.6382   -0.7312 P   0  0  0  0  0  0  0  0  0  0  0  0
   -0.4183    0.6022   -0.9928 C   0  0  0  0  0  0  0  0  0  0  0  0
    0.6879   -0.3445   -0.4864 C   0  0  0  0  0  0  0  0  0  0  0  0
   -1.7496    0.5373   -0.2915 C   0  0  0  0  0  0  0  0  0  0  0  0
   -0.8856    0.3568    2.0842 C   0  0  0  0  0  0  0  0  0  0  0  0
   -1.9641    0.4291    1.0391 C   0  0  0  0  0  0  0  0  0  0  0  0
   -0.3847   -1.0731    2.3669 C   0  0  0  0  0  0  0  0  0  0  0  0
   -2.6074    0.7230   -0.9674 H   0  0  0  0  0  0  0  0  0  0  0  0
   -2.9902    0.4913    1.4432 H   0  0  0  0  0  0  0  0  0  0  0  0
   -0.5724    0.4117   -2.0889 H   0  0  0  0  0  0  0  0  0  0  0  0
   -0.0447    1.6626   -0.8915 H   0  0  0  0  0  0  0  0  0  0  0  0
    1.4377   -0.4689   -1.3163 H   0  0  0  0  0  0  0  0  0  0  0  0
   -1.0624   -1.5394    3.1334 H   0  0  0  0  0  0  0  0  0  0  0  0
   -1.2833    0.7812    3.0473 H   0  0  0  0  0  0  0  0  0  0  0  0
   -0.0314    1.0162    1.7786 H   0  0  0  0  0  0  0  0  0  0  0  0
   -0.2555   -2.0136    1.1904 C   0  0  0  0  0  0  0  0  0  0  0  0
    0.2207   -1.7063   -0.0500 C   0  0  0  0  0  0  0  0  0  0  0  0
   -0.3333   -3.0804    1.4980 H   0  0  0  0  0  0  0  0  0  0  0  0
    0.4407   -2.5153   -0.7766 H   0  0  0  0  0  0  0  0  0  0  0  0
    1.2372    0.1335    0.3701 H   0  0  0  0  0  0  0  0  0  0  0  0
    0.6412   -0.9975    2.8329 H   0  0  0  0  0  0  0  0  0  0  0  0
   -4.2368   -2.1079   -0.6363 N   0  0  0  0  0  0  0  0  0  0  0  0
   -5.3477   -2.1209   -0.9946 C   0  0  0  0  0  0  0  0  0  0  0  0
   -6.7069   -2.1549   -1.4426 C   0  0  0  0  0  0  0  0  0  0  0  0
   -7.2066   -3.3488   -1.9894 C   0  0  0  0  0  0  0  0  0  0  0  0
   -7.5027   -1.0042   -1.3287 C   0  0  0  0  0  0  0  0  0  0  0  0
   -8.5377   -3.3566   -2.4244 C   0  0  0  0  0  0  0  0  0  0  0  0
   -6.5800   -4.2400   -2.0739 H   0  0  0  0  0  0  0  0  0  0  0  0
```

```
   -8.8269   -1.0814   -1.7832 C   0  0  0  0  0  0  0  0  0  0  0  0
   -7.1100   -0.0790   -0.9013 H   0  0  0  0  0  0  0  0  0  0  0  0
   -9.3774   -2.2415   -2.3373 C   0  0  0  0  0  0  0  0  0  0  0  0
   -0.7789   -5.3252   -1.1919 C   0  0  0  0  0  0  0  0  0  0  0  0
    0.1118   -5.6778   -0.1691 C   0  0  0  0  0  0  0  0  0  0  0  0
   -0.4512   -5.5311   -2.5341 C   0  0  0  0  0  0  0  0  0  0  0  0
    1.3529   -6.2253   -0.5023 C   0  0  0  0  0  0  0  0  0  0  0  0
   -0.1380   -5.5601    0.8885 H   0  0  0  0  0  0  0  0  0  0  0  0
    0.7962   -6.0801   -2.8575 C   0  0  0  0  0  0  0  0  0  0  0  0
   -1.1407   -5.2910   -3.3486 H   0  0  0  0  0  0  0  0  0  0  0  0
    1.6970   -6.4249   -1.8461 C   0  0  0  0  0  0  0  0  0  0  0  0
    2.0557   -6.5076    0.2858 H   0  0  0  0  0  0  0  0  0  0  0  0
    1.0616   -6.2465   -3.9050 H   0  0  0  0  0  0  0  0  0  0  0  0
    2.6693   -6.8567   -2.1020 H   0  0  0  0  0  0  0  0  0  0  0  0
   -3.0387   -5.6295    0.7119 C   0  0  0  0  0  0  0  0  0  0  0  0
   -2.8066   -7.0066    0.7587 C   0  0  0  0  0  0  0  0  0  0  0  0
   -3.7362   -4.9753    1.7330 C   0  0  0  0  0  0  0  0  0  0  0  0
   -3.2983   -7.7427    1.8437 C   0  0  0  0  0  0  0  0  0  0  0  0
   -2.2456   -7.5295   -0.0227 H   0  0  0  0  0  0  0  0  0  0  0  0
   -4.2256   -5.7189    2.8106 C   0  0  0  0  0  0  0  0  0  0  0  0
   -3.9078   -3.8871    1.7198 H   0  0  0  0  0  0  0  0  0  0  0  0
   -4.0088   -7.1024    2.8637 C   0  0  0  0  0  0  0  0  0  0  0  0
   -3.1241   -8.8210    1.8935 H   0  0  0  0  0  0  0  0  0  0  0  0
   -4.7759   -5.2243    3.6147 H   0  0  0  0  0  0  0  0  0  0  0  0
   -4.3937   -7.6817    3.7088 H   0  0  0  0  0  0  0  0  0  0  0  0
   -3.5837   -4.9611   -2.1533 C   0  0  0  0  0  0  0  0  0  0  0  0
   -4.4342   -6.0672   -2.1257 C   0  0  0  0  0  0  0  0  0  0  0  0
   -3.5435   -4.1090   -3.2650 C   0  0  0  0  0  0  0  0  0  0  0  0
   -5.2511   -6.3307   -3.2342 C   0  0  0  0  0  0  0  0  0  0  0  0
   -4.4906   -6.7420   -1.2656 H   0  0  0  0  0  0  0  0  0  0  0  0
   -4.3597   -4.3808   -4.3653 C   0  0  0  0  0  0  0  0  0  0  0  0
   -2.8878   -3.2324   -3.3058 H   0  0  0  0  0  0  0  0  0  0  0  0
   -5.2101   -5.4955   -4.3529 C   0  0  0  0  0  0  0  0  0  0  0  0
   -5.9144   -7.2002   -3.2252 H   0  0  0  0  0  0  0  0  0  0  0  0
   -4.3323   -3.7293   -5.2426 H   0  0  0  0  0  0  0  0  0  0  0  0
   -5.8399   -5.7106   -5.2216 H   0  0  0  0  0  0  0  0  0  0  0  0
   -2.3958   -2.1205   -0.0722 Ir  0  0  0  0  0  0  0  0  0  0  0  0
  -10.4179   -2.2745   -2.6853 H   0  0  0  0  0  0  0  0  0  0  0  0
   -9.5903   -0.0010   -1.6814 F   0  0  0  0  0  0  0  0  0  0  0  0
   -9.0206   -4.4789   -2.9449 F   0  0  0  0  0  0  0  0  0  0  0  0
  1 32  1  0  0  0  0
  1 43  1  0  0  0  0
  1 54  1  0  0  0  0
  1 65  1  0  0  0  0
  2  3  1  0  0  0  0
  2  4  1  0  0  0  0
  2 10  1  0  0  0  0
  2 11  1  0  0  0  0
  3 12  1  0  0  0  0
  3 17  1  0  0  0  0
  3 20  1  0  0  0  0
  4  6  2  0  0  0  0
  4  8  1  0  0  0  0
  4 65  1  0  0  0  0
  5  6  1  0  0  0  0
  5  7  1  0  0  0  0
  5 14  1  0  0  0  0
  5 15  1  0  0  0  0
  6  9  1  0  0  0  0
  6 65  1  0  0  0  0
  7 13  1  0  0  0  0
  7 16  1  0  0  0  0
  7 21  1  0  0  0  0
 16 17  2  0  0  0  0
 16 18  1  0  0  0  0
```

```
 16 65  1  0  0  0  0
 17 19  1  0  0  0  0
 17 65  1  0  0  0  0
 22 23  3  0  0  0  0
 22 65  1  0  0  0  0
 23 24  1  0  0  0  0
 24 25  2  0  0  0  0
 24 26  1  0  0  0  0
 25 27  1  0  0  0  0
 25 28  1  0  0  0  0
 26 29  2  0  0  0  0
 26 30  1  0  0  0  0
 27 31  2  0  0  0  0
 27 68  1  0  0  0  0
 29 31  1  0  0  0  0
 29 67  1  0  0  0  0
 31 66  1  0  0  0  0
 32 33  2  0  0  0  0
 32 34  1  0  0  0  0
 33 35  1  0  0  0  0
 33 36  1  0  0  0  0
 34 37  2  0  0  0  0
 34 38  1  0  0  0  0
 35 39  2  0  0  0  0
 35 40  1  0  0  0  0
 37 39  1  0  0  0  0
 37 41  1  0  0  0  0
 39 42  1  0  0  0  0
 43 44  2  0  0  0  0
 43 45  1  0  0  0  0
 44 46  1  0  0  0  0
 44 47  1  0  0  0  0
 45 48  2  0  0  0  0
 45 49  1  0  0  0  0
 46 50  2  0  0  0  0
 46 51  1  0  0  0  0
 48 50  1  0  0  0  0
 48 52  1  0  0  0  0
 50 53  1  0  0  0  0
 54 55  2  0  0  0  0
 54 56  1  0  0  0  0
 55 57  1  0  0  0  0
 55 58  1  0  0  0  0
 56 59  2  0  0  0  0
 56 60  1  0  0  0  0
 57 61  2  0  0  0  0
 57 62  1  0  0  0  0
 59 61  1  0  0  0  0
 59 63  1  0  0  0  0
 61 64  1  0  0  0  0
M  END
$$$$
mol062
Optimized Catalyst 062
Optimized Catalyst 062
 68 75  0  0  0  0  0  0  0  0999 V2000
   -2.4186   -4.6762   -0.7434 P   0  0  0  0  0  0  0  0  0  0  0  0
   -0.4678    0.5859   -1.0117 C   0  0  0  0  0  0  0  0  0  0  0  0
    0.6437   -0.3581   -0.5109 C   0  0  0  0  0  0  0  0  0  0  0  0
   -1.7979    0.5121   -0.3088 C   0  0  0  0  0  0  0  0  0  0  0  0
   -0.9264    0.3351    2.0640 C   0  0  0  0  0  0  0  0  0  0  0  0
   -2.0084    0.4027    1.0223 C   0  0  0  0  0  0  0  0  0  0  0  0
   -0.4206   -1.0930    2.3468 C   0  0  0  0  0  0  0  0  0  0  0  0
   -2.6588    0.6926   -0.9824 H   0  0  0  0  0  0  0  0  0  0  0  0
   -3.0339    0.4572    1.4283 H   0  0  0  0  0  0  0  0  0  0  0  0
```

```
   -0.6231    0.4002   -2.1084 H   0  0  0  0  0  0  0  0  0  0  0  0
   -0.0987    1.6471   -0.9055 H   0  0  0  0  0  0  0  0  0  0  0  0
    1.3902   -0.4781   -1.3443 H   0  0  0  0  0  0  0  0  0  0  0  0
   -1.1013   -1.5626    3.1086 H   0  0  0  0  0  0  0  0  0  0  0  0
   -1.3229    0.7592    3.0275 H   0  0  0  0  0  0  0  0  0  0  0  0
   -0.0754    0.9968    1.7549 H   0  0  0  0  0  0  0  0  0  0  0  0
   -0.2808   -2.0325    1.1712 C   0  0  0  0  0  0  0  0  0  0  0  0
    0.1860   -1.7232   -0.0725 C   0  0  0  0  0  0  0  0  0  0  0  0
   -0.3494   -3.0994    1.4800 H   0  0  0  0  0  0  0  0  0  0  0  0
    0.4173   -2.5325   -0.7959 H   0  0  0  0  0  0  0  0  0  0  0  0
    1.1946    0.1212    0.3439 H   0  0  0  0  0  0  0  0  0  0  0  0
    0.6027   -1.0139    2.8180 H   0  0  0  0  0  0  0  0  0  0  0  0
   -4.2707   -2.1401   -0.6635 N   0  0  0  0  0  0  0  0  0  0  0  0
   -5.3917   -2.1141   -0.9927 C   0  0  0  0  0  0  0  0  0  0  0  0
   -6.7444   -2.0794   -1.3896 C   0  0  0  0  0  0  0  0  0  0  0  0
   -7.4494   -3.2472   -1.7769 C   0  0  0  0  0  0  0  0  0  0  0  0
   -7.4890   -0.8724   -1.4232 C   0  0  0  0  0  0  0  0  0  0  0  0
   -8.7834   -3.2283   -2.1717 C   0  0  0  0  0  0  0  0  0  0  0  0
   -8.8251   -0.8190   -1.8086 C   0  0  0  0  0  0  0  0  0  0  0  0
   -9.4686   -2.0056   -2.1834 C   0  0  0  0  0  0  0  0  0  0  0  0
   -0.7420   -5.3358   -1.1707 C   0  0  0  0  0  0  0  0  0  0  0  0
    0.1358   -5.6915   -0.1388 C   0  0  0  0  0  0  0  0  0  0  0  0
   -0.3907   -5.5183   -2.5107 C   0  0  0  0  0  0  0  0  0  0  0  0
    1.3879   -6.2215   -0.4602 C   0  0  0  0  0  0  0  0  0  0  0  0
   -0.1319   -5.5882    0.9159 H   0  0  0  0  0  0  0  0  0  0  0  0
    0.8663   -6.0511   -2.8220 C   0  0  0  0  0  0  0  0  0  0  0  0
   -1.0698   -5.2703   -3.3323 H   0  0  0  0  0  0  0  0  0  0  0  0
    1.7543   -6.4007   -1.8007 C   0  0  0  0  0  0  0  0  0  0  0  0
    2.0816   -6.5060    0.3349 H   0  0  0  0  0  0  0  0  0  0  0  0
    1.1494   -6.2013   -3.8671 H   0  0  0  0  0  0  0  0  0  0  0  0
    2.7345   -6.8198   -2.0470 H   0  0  0  0  0  0  0  0  0  0  0  0
   -3.0286   -5.6630    0.7001 C   0  0  0  0  0  0  0  0  0  0  0  0
   -2.7763   -7.0359    0.7656 C   0  0  0  0  0  0  0  0  0  0  0  0
   -3.7476   -5.0088    1.7057 C   0  0  0  0  0  0  0  0  0  0  0  0
   -3.2697   -7.7676    1.8526 C   0  0  0  0  0  0  0  0  0  0  0  0
   -2.1962   -7.5588   -0.0012 H   0  0  0  0  0  0  0  0  0  0  0  0
   -4.2367   -5.7477    2.7872 C   0  0  0  0  0  0  0  0  0  0  0  0
   -3.9374   -3.9242    1.6779 H   0  0  0  0  0  0  0  0  0  0  0  0
   -4.0003   -7.1267    2.8581 C   0  0  0  0  0  0  0  0  0  0  0  0
   -3.0813   -8.8426    1.9155 H   0  0  0  0  0  0  0  0  0  0  0  0
   -4.8023   -5.2524    3.5800 H   0  0  0  0  0  0  0  0  0  0  0  0
   -4.3853   -7.7024    3.7053 H   0  0  0  0  0  0  0  0  0  0  0  0
   -3.5108   -5.0386   -2.1983 C   0  0  0  0  0  0  0  0  0  0  0  0
   -4.2372   -6.2286   -2.2482 C   0  0  0  0  0  0  0  0  0  0  0  0
   -3.5414   -4.1277   -3.2623 C   0  0  0  0  0  0  0  0  0  0  0  0
   -4.9973   -6.5187   -3.3902 C   0  0  0  0  0  0  0  0  0  0  0  0
   -4.2419   -6.9475   -1.4240 H   0  0  0  0  0  0  0  0  0  0  0  0
   -4.3044   -4.4233   -4.3940 C   0  0  0  0  0  0  0  0  0  0  0  0
   -2.9806   -3.1863   -3.2414 H   0  0  0  0  0  0  0  0  0  0  0  0
   -5.0287   -5.6220   -4.4602 C   0  0  0  0  0  0  0  0  0  0  0  0
   -5.5614   -7.4534   -3.4424 H   0  0  0  0  0  0  0  0  0  0  0  0
   -4.3323   -3.7254   -5.2341 H   0  0  0  0  0  0  0  0  0  0  0  0
   -5.6165   -5.8543   -5.3526 H   0  0  0  0  0  0  0  0  0  0  0  0
   -2.4130   -2.1512   -0.1215 Ir  0  0  0  0  0  0  0  0  0  0  0  0
  -10.5231   -1.9781   -2.4882 H   0  0  0  0  0  0  0  0  0  0  0  0
   -9.2945   -4.1524   -2.4654 H   0  0  0  0  0  0  0  0  0  0  0  0
   -9.3667    0.1350   -1.8170 H   0  0  0  0  0  0  0  0  0  0  0  0
   -6.7938   -4.3993   -1.7502 F   0  0  0  0  0  0  0  0  0  0  0  0
   -6.8674    0.2426   -1.0662 F   0  0  0  0  0  0  0  0  0  0  0  0
  1 30  1  0  0  0  0
  1 41  1  0  0  0  0
  1 52  1  0  0  0  0
  1 63  1  0  0  0  0
  2  3  1  0  0  0  0
  2  4  1  0  0  0  0
```

```
  2 10  1  0  0  0  0
  2 11  1  0  0  0  0
  3 12  1  0  0  0  0
  3 17  1  0  0  0  0
  3 20  1  0  0  0  0
  4  6  2  0  0  0  0
  4  8  1  0  0  0  0
  4 63  1  0  0  0  0
  5  6  1  0  0  0  0
  5  7  1  0  0  0  0
  5 14  1  0  0  0  0
  5 15  1  0  0  0  0
  6  9  1  0  0  0  0
  6 63  1  0  0  0  0
  7 13  1  0  0  0  0
  7 16  1  0  0  0  0
  7 21  1  0  0  0  0
 16 17  2  0  0  0  0
 16 18  1  0  0  0  0
 16 63  1  0  0  0  0
 17 19  1  0  0  0  0
 17 63  1  0  0  0  0
 22 23  3  0  0  0  0
 22 63  1  0  0  0  0
 23 24  1  0  0  0  0
 24 25  2  0  0  0  0
 24 26  1  0  0  0  0
 25 27  1  0  0  0  0
 25 67  1  0  0  0  0
 26 28  2  0  0  0  0
 26 68  1  0  0  0  0
 27 29  2  0  0  0  0
 27 65  1  0  0  0  0
 28 29  1  0  0  0  0
 28 66  1  0  0  0  0
 29 64  1  0  0  0  0
 30 31  2  0  0  0  0
 30 32  1  0  0  0  0
 31 33  1  0  0  0  0
 31 34  1  0  0  0  0
 32 35  2  0  0  0  0
 32 36  1  0  0  0  0
 33 37  2  0  0  0  0
 33 38  1  0  0  0  0
 35 37  1  0  0  0  0
 35 39  1  0  0  0  0
 37 40  1  0  0  0  0
 41 42  2  0  0  0  0
 41 43  1  0  0  0  0
 42 44  1  0  0  0  0
 42 45  1  0  0  0  0
 43 46  2  0  0  0  0
 43 47  1  0  0  0  0
 44 48  2  0  0  0  0
 44 49  1  0  0  0  0
 46 48  1  0  0  0  0
 46 50  1  0  0  0  0
 48 51  1  0  0  0  0
 52 53  2  0  0  0  0
 52 54  1  0  0  0  0
 53 55  1  0  0  0  0
 53 56  1  0  0  0  0
 54 57  2  0  0  0  0
 54 58  1  0  0  0  0
 55 59  2  0  0  0  0
```

```
 55 60  1  0  0  0  0
 57 59  1  0  0  0  0
 57 61  1  0  0  0  0
 59 62  1  0  0  0  0
M  END
$$$$
mol063
Optimized Catalyst 063
Optimized Catalyst 063
 71 78  0  0  0  0  0  0  0  0999 V2000
   -2.4352   -4.6365   -0.7307 P   0  0  0  0  0  0  0  0  0  0  0  0
   -0.4179    0.6033   -0.9928 C   0  0  0  0  0  0  0  0  0  0  0  0
    0.6878   -0.3442   -0.4863 C   0  0  0  0  0  0  0  0  0  0  0  0
   -1.7492    0.5386   -0.2912 C   0  0  0  0  0  0  0  0  0  0  0  0
   -0.8851    0.3573    2.0847 C   0  0  0  0  0  0  0  0  0  0  0  0
   -1.9636    0.4298    1.0394 C   0  0  0  0  0  0  0  0  0  0  0  0
   -0.3838   -1.0726    2.3677 C   0  0  0  0  0  0  0  0  0  0  0  0
   -2.6076    0.7212   -0.9673 H   0  0  0  0  0  0  0  0  0  0  0  0
   -2.9901    0.4898    1.4428 H   0  0  0  0  0  0  0  0  0  0  0  0
   -0.5728    0.4114   -2.0885 H   0  0  0  0  0  0  0  0  0  0  0  0
   -0.0440    1.6632   -0.8918 H   0  0  0  0  0  0  0  0  0  0  0  0
    1.4372   -0.4693   -1.3163 H   0  0  0  0  0  0  0  0  0  0  0  0
   -1.0627   -1.5390    3.1330 H   0  0  0  0  0  0  0  0  0  0  0  0
   -1.2834    0.7812    3.0475 H   0  0  0  0  0  0  0  0  0  0  0  0
   -0.0309    1.0163    1.7786 H   0  0  0  0  0  0  0  0  0  0  0  0
   -0.2541   -2.0135    1.1915 C   0  0  0  0  0  0  0  0  0  0  0  0
    0.2197   -1.7062   -0.0500 C   0  0  0  0  0  0  0  0  0  0  0  0
   -0.3325   -3.0802    1.4989 H   0  0  0  0  0  0  0  0  0  0  0  0
    0.4403   -2.5152   -0.7765 H   0  0  0  0  0  0  0  0  0  0  0  0
    1.2370    0.1333    0.3703 H   0  0  0  0  0  0  0  0  0  0  0  0
    0.6417   -0.9970    2.8339 H   0  0  0  0  0  0  0  0  0  0  0  0
   -4.2435   -2.1094   -0.6376 N   0  0  0  0  0  0  0  0  0  0  0  0
   -5.3561   -2.1212   -0.9978 C   0  0  0  0  0  0  0  0  0  0  0  0
   -6.7008   -2.1552   -1.4397 C   0  0  0  0  0  0  0  0  0  0  0  0
   -7.2224   -3.3468   -1.9917 C   0  0  0  0  0  0  0  0  0  0  0  0
   -7.5182   -1.0080   -1.3324 C   0  0  0  0  0  0  0  0  0  0  0  0
   -8.5384   -3.3823   -2.4324 C   0  0  0  0  0  0  0  0  0  0  0  0
   -8.8334   -1.0575   -1.7763 C   0  0  0  0  0  0  0  0  0  0  0  0
   -9.3605   -2.2396   -2.3366 C   0  0  0  0  0  0  0  0  0  0  0  0
   -0.7796   -5.3267   -1.1919 C   0  0  0  0  0  0  0  0  0  0  0  0
    0.1114   -5.6786   -0.1693 C   0  0  0  0  0  0  0  0  0  0  0  0
   -0.4515   -5.5317   -2.5339 C   0  0  0  0  0  0  0  0  0  0  0  0
    1.3528   -6.2257   -0.5025 C   0  0  0  0  0  0  0  0  0  0  0  0
   -0.1382   -5.5598    0.8883 H   0  0  0  0  0  0  0  0  0  0  0  0
    0.7960   -6.0804   -2.8575 C   0  0  0  0  0  0  0  0  0  0  0  0
   -1.1406   -5.2906   -3.3484 H   0  0  0  0  0  0  0  0  0  0  0  0
    1.6969   -6.4251   -1.8461 C   0  0  0  0  0  0  0  0  0  0  0  0
    2.0555   -6.5072    0.2857 H   0  0  0  0  0  0  0  0  0  0  0  0
    1.0616   -6.2461   -3.9048 H   0  0  0  0  0  0  0  0  0  0  0  0
    2.6692   -6.8565   -2.1020 H   0  0  0  0  0  0  0  0  0  0  0  0
   -3.0390   -5.6295    0.7117 C   0  0  0  0  0  0  0  0  0  0  0  0
   -2.8069   -7.0068    0.7585 C   0  0  0  0  0  0  0  0  0  0  0  0
   -3.7362   -4.9755    1.7326 C   0  0  0  0  0  0  0  0  0  0  0  0
   -3.2983   -7.7428    1.8434 C   0  0  0  0  0  0  0  0  0  0  0  0
   -2.2455   -7.5292   -0.0227 H   0  0  0  0  0  0  0  0  0  0  0  0
   -4.2257   -5.7193    2.8105 C   0  0  0  0  0  0  0  0  0  0  0  0
   -3.9082   -3.8870    1.7191 H   0  0  0  0  0  0  0  0  0  0  0  0
   -4.0088   -7.1024    2.8636 C   0  0  0  0  0  0  0  0  0  0  0  0
   -3.1239   -8.8208    1.8937 H   0  0  0  0  0  0  0  0  0  0  0  0
   -4.7757   -5.2241    3.6143 H   0  0  0  0  0  0  0  0  0  0  0  0
   -4.3933   -7.6817    3.7086 H   0  0  0  0  0  0  0  0  0  0  0  0
   -3.5823   -4.9625   -2.1541 C   0  0  0  0  0  0  0  0  0  0  0  0
   -4.4337   -6.0678   -2.1260 C   0  0  0  0  0  0  0  0  0  0  0  0
   -3.5429   -4.1101   -3.2653 C   0  0  0  0  0  0  0  0  0  0  0  0
   -5.2509   -6.3311   -3.2343 C   0  0  0  0  0  0  0  0  0  0  0  0
```

```
   -4.4904   -6.7417   -1.2654 H   0  0  0  0  0  0  0  0  0  0  0  0
   -4.3595   -4.3815   -4.3658 C   0  0  0  0  0  0  0  0  0  0  0  0
   -2.8889   -3.2318   -3.3047 H   0  0  0  0  0  0  0  0  0  0  0  0
   -5.2099   -5.4957   -4.3530 C   0  0  0  0  0  0  0  0  0  0  0  0
   -5.9132   -7.2005   -3.2250 H   0  0  0  0  0  0  0  0  0  0  0  0
   -4.3326   -3.7289   -5.2420 H   0  0  0  0  0  0  0  0  0  0  0  0
   -5.8389   -5.7104   -5.2217 H   0  0  0  0  0  0  0  0  0  0  0  0
   -2.3905   -2.1186   -0.0743 Ir  0  0  0  0  0  0  0  0  0  0  0  0
   -8.9418   -4.3044   -2.8571 H   0  0  0  0  0  0  0  0  0  0  0  0
   -9.4658   -0.1693   -1.6911 H   0  0  0  0  0  0  0  0  0  0  0  0
   -6.5874   -4.2325   -2.0727 H   0  0  0  0  0  0  0  0  0  0  0  0
   -7.1148   -0.0874   -0.9050 H   0  0  0  0  0  0  0  0  0  0  0  0
  -10.7691   -2.2835   -2.8129 C   0  0  0  0  0  0  0  0  0  0  0  0
  -11.4399   -2.5928   -1.9691 H   0  0  0  0  0  0  0  0  0  0  0  0
  -11.1108   -1.2832   -3.1771 H   0  0  0  0  0  0  0  0  0  0  0  0
  -10.9000   -3.0153   -3.6470 H   0  0  0  0  0  0  0  0  0  0  0  0
  1 30  1  0  0  0  0
  1 41  1  0  0  0  0
  1 52  1  0  0  0  0
  1 63  1  0  0  0  0
  2  3  1  0  0  0  0
  2  4  1  0  0  0  0
  2 10  1  0  0  0  0
  2 11  1  0  0  0  0
  3 12  1  0  0  0  0
  3 17  1  0  0  0  0
  3 20  1  0  0  0  0
  4  6  2  0  0  0  0
  4  8  1  0  0  0  0
  4 63  1  0  0  0  0
  5  6  1  0  0  0  0
  5  7  1  0  0  0  0
  5 14  1  0  0  0  0
  5 15  1  0  0  0  0
  6  9  1  0  0  0  0
  6 63  1  0  0  0  0
  7 13  1  0  0  0  0
  7 16  1  0  0  0  0
  7 21  1  0  0  0  0
 16 17  2  0  0  0  0
 16 18  1  0  0  0  0
 16 63  1  0  0  0  0
 17 19  1  0  0  0  0
 17 63  1  0  0  0  0
 22 23  3  0  0  0  0
 22 63  1  0  0  0  0
 23 24  1  0  0  0  0
 24 25  2  0  0  0  0
 24 26  1  0  0  0  0
 25 27  1  0  0  0  0
 25 66  1  0  0  0  0
 26 28  2  0  0  0  0
 26 67  1  0  0  0  0
 27 29  2  0  0  0  0
 27 64  1  0  0  0  0
 28 29  1  0  0  0  0
 28 65  1  0  0  0  0
 29 68  1  0  0  0  0
 30 31  2  0  0  0  0
 30 32  1  0  0  0  0
 31 33  1  0  0  0  0
 31 34  1  0  0  0  0
 32 35  2  0  0  0  0
 32 36  1  0  0  0  0
 33 37  2  0  0  0  0
```

```
 33 38  1  0  0  0  0
 35 37  1  0  0  0  0
 35 39  1  0  0  0  0
 37 40  1  0  0  0  0
 41 42  2  0  0  0  0
 41 43  1  0  0  0  0
 42 44  1  0  0  0  0
 42 45  1  0  0  0  0
 43 46  2  0  0  0  0
 43 47  1  0  0  0  0
 44 48  2  0  0  0  0
 44 49  1  0  0  0  0
 46 48  1  0  0  0  0
 46 50  1  0  0  0  0
 48 51  1  0  0  0  0
 52 53  2  0  0  0  0
 52 54  1  0  0  0  0
 53 55  1  0  0  0  0
 53 56  1  0  0  0  0
 54 57  2  0  0  0  0
 54 58  1  0  0  0  0
 55 59  2  0  0  0  0
 55 60  1  0  0  0  0
 57 59  1  0  0  0  0
 57 61  1  0  0  0  0
 59 62  1  0  0  0  0
 68 69  1  0  0  0  0
 68 70  1  0  0  0  0
 68 71  1  0  0  0  0
M  END
$$$$
mol064
Optimized Catalyst 064
Optimized Catalyst 064
 74 81  0  0  0  0  0  0  0  0999 V2000
   -2.4368   -4.6376   -0.7319 P   0  0  0  0  0  0  0  0  0  0  0  0
   -0.4180    0.6034   -0.9927 C   0  0  0  0  0  0  0  0  0  0  0  0
    0.6877   -0.3442   -0.4864 C   0  0  0  0  0  0  0  0  0  0  0  0
   -1.7494    0.5385   -0.2913 C   0  0  0  0  0  0  0  0  0  0  0  0
   -0.8856    0.3576    2.0846 C   0  0  0  0  0  0  0  0  0  0  0  0
   -1.9640    0.4299    1.0392 C   0  0  0  0  0  0  0  0  0  0  0  0
   -0.3846   -1.0725    2.3676 C   0  0  0  0  0  0  0  0  0  0  0  0
   -2.6078    0.7212   -0.9675 H   0  0  0  0  0  0  0  0  0  0  0  0
   -2.9905    0.4897    1.4427 H   0  0  0  0  0  0  0  0  0  0  0  0
   -0.5728    0.4118   -2.0885 H   0  0  0  0  0  0  0  0  0  0  0  0
   -0.0441    1.6634   -0.8915 H   0  0  0  0  0  0  0  0  0  0  0  0
    1.4370   -0.4695   -1.3164 H   0  0  0  0  0  0  0  0  0  0  0  0
   -1.0635   -1.5388    3.1329 H   0  0  0  0  0  0  0  0  0  0  0  0
   -1.2838    0.7817    3.0474 H   0  0  0  0  0  0  0  0  0  0  0  0
   -0.0313    1.0164    1.7785 H   0  0  0  0  0  0  0  0  0  0  0  0
   -0.2556   -2.0130    1.1910 C   0  0  0  0  0  0  0  0  0  0  0  0
    0.2187   -1.7059   -0.0501 C   0  0  0  0  0  0  0  0  0  0  0  0
   -0.3358   -3.0797    1.4978 H   0  0  0  0  0  0  0  0  0  0  0  0
    0.4376   -2.5147   -0.7772 H   0  0  0  0  0  0  0  0  0  0  0  0
    1.2371    0.1334    0.3701 H   0  0  0  0  0  0  0  0  0  0  0  0
    0.6409   -0.9970    2.8340 H   0  0  0  0  0  0  0  0  0  0  0  0
   -4.2501   -2.1099   -0.6410 N   0  0  0  0  0  0  0  0  0  0  0  0
   -5.3619   -2.1210   -0.9995 C   0  0  0  0  0  0  0  0  0  0  0  0
   -6.7181   -2.1547   -1.4465 C   0  0  0  0  0  0  0  0  0  0  0  0
   -7.2188   -3.3473   -1.9933 C   0  0  0  0  0  0  0  0  0  0  0  0
   -7.5162   -1.0063   -1.3338 C   0  0  0  0  0  0  0  0  0  0  0  0
   -8.5496   -3.3896   -2.4399 C   0  0  0  0  0  0  0  0  0  0  0  0
   -8.8489   -1.0515   -1.7804 C   0  0  0  0  0  0  0  0  0  0  0  0
   -9.3515   -2.2422   -2.3291 C   0  0  0  0  0  0  0  0  0  0  0  0
   -0.7802   -5.3258   -1.1918 C   0  0  0  0  0  0  0  0  0  0  0  0
```

```
    0.1108   -5.6779   -0.1692 C   0  0  0  0  0  0  0  0  0  0  0  0
   -0.4520   -5.5309   -2.5339 C   0  0  0  0  0  0  0  0  0  0  0  0
    1.3519   -6.2254   -0.5025 C   0  0  0  0  0  0  0  0  0  0  0  0
   -0.1387   -5.5591    0.8884 H   0  0  0  0  0  0  0  0  0  0  0  0
    0.7952   -6.0799   -2.8575 C   0  0  0  0  0  0  0  0  0  0  0  0
   -1.1411   -5.2897   -3.3484 H   0  0  0  0  0  0  0  0  0  0  0  0
    1.6961   -6.4250   -1.8461 C   0  0  0  0  0  0  0  0  0  0  0  0
    2.0546   -6.5073    0.2857 H   0  0  0  0  0  0  0  0  0  0  0  0
    1.0608   -6.2457   -3.9048 H   0  0  0  0  0  0  0  0  0  0  0  0
    2.6681   -6.8567   -2.1020 H   0  0  0  0  0  0  0  0  0  0  0  0
   -3.0394   -5.6297    0.7114 C   0  0  0  0  0  0  0  0  0  0  0  0
   -2.8068   -7.0067    0.7583 C   0  0  0  0  0  0  0  0  0  0  0  0
   -3.7368   -4.9756    1.7322 C   0  0  0  0  0  0  0  0  0  0  0  0
   -3.2982   -7.7429    1.8433 C   0  0  0  0  0  0  0  0  0  0  0  0
   -2.2453   -7.5292   -0.0227 H   0  0  0  0  0  0  0  0  0  0  0  0
   -4.2262   -5.7196    2.8102 C   0  0  0  0  0  0  0  0  0  0  0  0
   -3.9093   -3.8873    1.7186 H   0  0  0  0  0  0  0  0  0  0  0  0
   -4.0091   -7.1025    2.8633 C   0  0  0  0  0  0  0  0  0  0  0  0
   -3.1235   -8.8208    1.8936 H   0  0  0  0  0  0  0  0  0  0  0  0
   -4.7765   -5.2245    3.6138 H   0  0  0  0  0  0  0  0  0  0  0  0
   -4.3938   -7.6819    3.7082 H   0  0  0  0  0  0  0  0  0  0  0  0
   -3.5837   -4.9639   -2.1551 C   0  0  0  0  0  0  0  0  0  0  0  0
   -4.4369   -6.0678   -2.1262 C   0  0  0  0  0  0  0  0  0  0  0  0
   -3.5444   -4.1112   -3.2662 C   0  0  0  0  0  0  0  0  0  0  0  0
   -5.2564   -6.3293   -3.2335 C   0  0  0  0  0  0  0  0  0  0  0  0
   -4.4936   -6.7416   -1.2656 H   0  0  0  0  0  0  0  0  0  0  0  0
   -4.3626   -4.3814   -4.3659 C   0  0  0  0  0  0  0  0  0  0  0  0
   -2.8898   -3.2336   -3.3061 H   0  0  0  0  0  0  0  0  0  0  0  0
   -5.2152   -5.4939   -4.3519 C   0  0  0  0  0  0  0  0  0  0  0  0
   -5.9206   -7.1969   -3.2229 H   0  0  0  0  0  0  0  0  0  0  0  0
   -4.3360   -3.7282   -5.2417 H   0  0  0  0  0  0  0  0  0  0  0  0
   -5.8450   -5.7072   -5.2200 H   0  0  0  0  0  0  0  0  0  0  0  0
   -2.3986   -2.1196   -0.0769 Ir  0  0  0  0  0  0  0  0  0  0  0  0
   -6.5858   -4.2309   -2.0727 H   0  0  0  0  0  0  0  0  0  0  0  0
   -7.1156   -0.0878   -0.9062 H   0  0  0  0  0  0  0  0  0  0  0  0
  -10.3877   -2.2758   -2.6761 H   0  0  0  0  0  0  0  0  0  0  0  0
   -9.7051    0.1648   -1.6644 C   0  0  0  0  0  0  0  0  0  0  0  0
   -9.7872    0.4780   -0.5937 H   0  0  0  0  0  0  0  0  0  0  0  0
   -9.2588    1.0075   -2.2491 H   0  0  0  0  0  0  0  0  0  0  0  0
  -10.7378   -0.0129   -2.0505 H   0  0  0  0  0  0  0  0  0  0  0  0
   -9.0882   -4.6538   -3.0241 C   0  0  0  0  0  0  0  0  0  0  0  0
   -8.4942   -4.9438   -3.9245 H   0  0  0  0  0  0  0  0  0  0  0  0
   -9.0322   -5.4780   -2.2711 H   0  0  0  0  0  0  0  0  0  0  0  0
  -10.1560   -4.5444   -3.3340 H   0  0  0  0  0  0  0  0  0  0  0  0
  1 30  1  0  0  0  0
  1 41  1  0  0  0  0
  1 52  1  0  0  0  0
  1 63  1  0  0  0  0
  2  3  1  0  0  0  0
  2  4  1  0  0  0  0
  2 10  1  0  0  0  0
  2 11  1  0  0  0  0
  3 12  1  0  0  0  0
  3 17  1  0  0  0  0
  3 20  1  0  0  0  0
  4  6  2  0  0  0  0
  4  8  1  0  0  0  0
  4 63  1  0  0  0  0
  5  6  1  0  0  0  0
  5  7  1  0  0  0  0
  5 14  1  0  0  0  0
  5 15  1  0  0  0  0
  6  9  1  0  0  0  0
  6 63  1  0  0  0  0
  7 13  1  0  0  0  0
```

```
  7 16  1  0  0  0  0
  7 21  1  0  0  0  0
 16 17  2  0  0  0  0
 16 18  1  0  0  0  0
 16 63  1  0  0  0  0
 17 19  1  0  0  0  0
 17 63  1  0  0  0  0
 22 23  3  0  0  0  0
 22 63  1  0  0  0  0
 23 24  1  0  0  0  0
 24 25  2  0  0  0  0
 24 26  1  0  0  0  0
 25 27  1  0  0  0  0
 25 64  1  0  0  0  0
 26 28  2  0  0  0  0
 26 65  1  0  0  0  0
 27 29  2  0  0  0  0
 27 71  1  0  0  0  0
 28 29  1  0  0  0  0
 28 67  1  0  0  0  0
 29 66  1  0  0  0  0
 30 31  2  0  0  0  0
 30 32  1  0  0  0  0
 31 33  1  0  0  0  0
 31 34  1  0  0  0  0
 32 35  2  0  0  0  0
 32 36  1  0  0  0  0
 33 37  2  0  0  0  0
 33 38  1  0  0  0  0
 35 37  1  0  0  0  0
 35 39  1  0  0  0  0
 37 40  1  0  0  0  0
 41 42  2  0  0  0  0
 41 43  1  0  0  0  0
 42 44  1  0  0  0  0
 42 45  1  0  0  0  0
 43 46  2  0  0  0  0
 43 47  1  0  0  0  0
 44 48  2  0  0  0  0
 44 49  1  0  0  0  0
 46 48  1  0  0  0  0
 46 50  1  0  0  0  0
 48 51  1  0  0  0  0
 52 53  2  0  0  0  0
 52 54  1  0  0  0  0
 53 55  1  0  0  0  0
 53 56  1  0  0  0  0
 54 57  2  0  0  0  0
 54 58  1  0  0  0  0
 55 59  2  0  0  0  0
 55 60  1  0  0  0  0
 57 59  1  0  0  0  0
 57 61  1  0  0  0  0
 59 62  1  0  0  0  0
 67 68  1  0  0  0  0
 67 69  1  0  0  0  0
 67 70  1  0  0  0  0
 71 72  1  0  0  0  0
 71 73  1  0  0  0  0
 71 74  1  0  0  0  0
M  END
$$$$
mol065
Optimized Catalyst 065
Optimized Catalyst 065
```

```
 74 81  0  0  0  0  0  0  0  0999 V2000
   -2.3757   -4.7339   -0.8046 P   0  0  0  0  0  0  0  0  0  0  0  0
   -0.5878    0.5738   -1.0775 C   0  0  0  0  0  0  0  0  0  0  0  0
    0.5553   -0.3344   -0.5819 C   0  0  0  0  0  0  0  0  0  0  0  0
   -1.9095    0.4619   -0.3625 C   0  0  0  0  0  0  0  0  0  0  0  0
   -1.0146    0.3034    2.0043 C   0  0  0  0  0  0  0  0  0  0  0  0
   -2.1055    0.3434    0.9702 C   0  0  0  0  0  0  0  0  0  0  0  0
   -0.4656   -1.1096    2.2826 C   0  0  0  0  0  0  0  0  0  0  0  0
   -2.7808    0.6182   -1.0287 H   0  0  0  0  0  0  0  0  0  0  0  0
   -3.1285    0.3644    1.3845 H   0  0  0  0  0  0  0  0  0  0  0  0
   -0.7467    0.3766   -2.1720 H   0  0  0  0  0  0  0  0  0  0  0  0
   -0.2500    1.6459   -0.9797 H   0  0  0  0  0  0  0  0  0  0  0  0
    1.3023   -0.4300   -1.4178 H   0  0  0  0  0  0  0  0  0  0  0  0
   -1.1313   -1.6024    3.0430 H   0  0  0  0  0  0  0  0  0  0  0  0
   -1.4164    0.7137    2.9715 H   0  0  0  0  0  0  0  0  0  0  0  0
   -0.1845    0.9889    1.6902 H   0  0  0  0  0  0  0  0  0  0  0  0
   -0.2946   -2.0419    1.1064 C   0  0  0  0  0  0  0  0  0  0  0  0
    0.1446   -1.7155   -0.1433 C   0  0  0  0  0  0  0  0  0  0  0  0
   -0.3233   -3.1107    1.4130 H   0  0  0  0  0  0  0  0  0  0  0  0
    0.4043   -2.5160   -0.8671 H   0  0  0  0  0  0  0  0  0  0  0  0
    1.0940    0.1611    0.2711 H   0  0  0  0  0  0  0  0  0  0  0  0
    0.5544   -1.0001    2.7552 H   0  0  0  0  0  0  0  0  0  0  0  0
   -4.2886   -2.1909   -0.7256 N   0  0  0  0  0  0  0  0  0  0  0  0
   -5.4287   -2.1217   -0.9831 C   0  0  0  0  0  0  0  0  0  0  0  0
   -6.8041   -2.0266   -1.2831 C   0  0  0  0  0  0  0  0  0  0  0  0
   -7.5446   -3.1946   -1.6208 C   0  0  0  0  0  0  0  0  0  0  0  0
   -7.4444   -0.7535   -1.2366 C   0  0  0  0  0  0  0  0  0  0  0  0
   -8.9070   -3.0702   -1.9081 C   0  0  0  0  0  0  0  0  0  0  0  0
   -8.8074   -0.6720   -1.5293 C   0  0  0  0  0  0  0  0  0  0  0  0
   -9.5321   -1.8211   -1.8621 C   0  0  0  0  0  0  0  0  0  0  0  0
   -0.6627   -5.3596   -1.1303 C   0  0  0  0  0  0  0  0  0  0  0  0
    0.1502   -5.7288   -0.0516 C   0  0  0  0  0  0  0  0  0  0  0  0
   -0.2134   -5.4848   -2.4478 C   0  0  0  0  0  0  0  0  0  0  0  0
    1.4379   -6.2109   -0.3009 C   0  0  0  0  0  0  0  0  0  0  0  0
   -0.1936   -5.6707    0.9847 H   0  0  0  0  0  0  0  0  0  0  0  0
    1.0791   -5.9674   -2.6866 C   0  0  0  0  0  0  0  0  0  0  0  0
   -0.8422   -5.2289   -3.3063 H   0  0  0  0  0  0  0  0  0  0  0  0
    1.9043   -6.3268   -1.6168 C   0  0  0  0  0  0  0  0  0  0  0  0
    2.0819   -6.5054    0.5316 H   0  0  0  0  0  0  0  0  0  0  0  0
    1.4406   -6.0695   -3.7132 H   0  0  0  0  0  0  0  0  0  0  0  0
    2.9137   -6.7037   -1.8067 H   0  0  0  0  0  0  0  0  0  0  0  0
   -3.0571   -5.7186    0.6093 C   0  0  0  0  0  0  0  0  0  0  0  0
   -2.7591   -7.0784    0.7302 C   0  0  0  0  0  0  0  0  0  0  0  0
   -3.8735   -5.0702    1.5418 C   0  0  0  0  0  0  0  0  0  0  0  0
   -3.3029   -7.8026    1.7983 C   0  0  0  0  0  0  0  0  0  0  0  0
   -2.1041   -7.5971    0.0230 H   0  0  0  0  0  0  0  0  0  0  0  0
   -4.4156   -5.8022    2.6026 C   0  0  0  0  0  0  0  0  0  0  0  0
   -4.0969   -3.9946    1.4752 H   0  0  0  0  0  0  0  0  0  0  0  0
   -4.1322   -7.1684    2.7286 C   0  0  0  0  0  0  0  0  0  0  0  0
   -3.0751   -8.8664    1.9069 H   0  0  0  0  0  0  0  0  0  0  0  0
   -5.0567   -5.3101    3.3378 H   0  0  0  0  0  0  0  0  0  0  0  0
   -4.5569   -7.7383    3.5607 H   0  0  0  0  0  0  0  0  0  0  0  0
   -3.3728   -5.1353   -2.3176 C   0  0  0  0  0  0  0  0  0  0  0  0
   -3.9812   -6.3853   -2.4420 C   0  0  0  0  0  0  0  0  0  0  0  0
   -3.4470   -4.1894   -3.3474 C   0  0  0  0  0  0  0  0  0  0  0  0
   -4.6697   -6.6962   -3.6227 C   0  0  0  0  0  0  0  0  0  0  0  0
   -3.9429   -7.1364   -1.6481 H   0  0  0  0  0  0  0  0  0  0  0  0
   -4.1352   -4.5084   -4.5211 C   0  0  0  0  0  0  0  0  0  0  0  0
   -2.9770   -3.2011   -3.2668 H   0  0  0  0  0  0  0  0  0  0  0  0
   -4.7427   -5.7636   -4.6604 C   0  0  0  0  0  0  0  0  0  0  0  0
   -5.1419   -7.6759   -3.7335 H   0  0  0  0  0  0  0  0  0  0  0  0
   -4.1937   -3.7833   -5.3366 H   0  0  0  0  0  0  0  0  0  0  0  0
   -5.2715   -6.0130   -5.5851 H   0  0  0  0  0  0  0  0  0  0  0  0
   -2.4135   -2.1992   -0.2198 Ir  0  0  0  0  0  0  0  0  0  0  0  0
  -10.6013   -1.7408   -2.0884 H   0  0  0  0  0  0  0  0  0  0  0  0
```

```
   -9.4931   -3.9537   -2.1708 H   0  0  0  0  0  0  0  0  0  0  0  0
   -9.3182    0.2936   -1.4982 H   0  0  0  0  0  0  0  0  0  0  0  0
   -6.8748   -4.5251   -1.6851 C   0  0  0  0  0  0  0  0  0  0  0  0
   -6.2644   -4.5885   -2.6228 H   0  0  0  0  0  0  0  0  0  0  0  0
   -6.2148   -4.6966   -0.8033 H   0  0  0  0  0  0  0  0  0  0  0  0
   -7.6246   -5.3556   -1.7124 H   0  0  0  0  0  0  0  0  0  0  0  0
   -6.6632    0.4665   -0.8777 C   0  0  0  0  0  0  0  0  0  0  0  0
   -6.2018    0.3520    0.1325 H   0  0  0  0  0  0  0  0  0  0  0  0
   -5.8675    0.6560   -1.6385 H   0  0  0  0  0  0  0  0  0  0  0  0
   -7.3196    1.3745   -0.8455 H   0  0  0  0  0  0  0  0  0  0  0  0
  1 30  1  0  0  0  0
  1 41  1  0  0  0  0
  1 52  1  0  0  0  0
  1 63  1  0  0  0  0
  2  3  1  0  0  0  0
  2  4  1  0  0  0  0
  2 10  1  0  0  0  0
  2 11  1  0  0  0  0
  3 12  1  0  0  0  0
  3 17  1  0  0  0  0
  3 20  1  0  0  0  0
  4  6  2  0  0  0  0
  4  8  1  0  0  0  0
  4 63  1  0  0  0  0
  5  6  1  0  0  0  0
  5  7  1  0  0  0  0
  5 14  1  0  0  0  0
  5 15  1  0  0  0  0
  6  9  1  0  0  0  0
  6 63  1  0  0  0  0
  7 13  1  0  0  0  0
  7 16  1  0  0  0  0
  7 21  1  0  0  0  0
 16 17  2  0  0  0  0
 16 18  1  0  0  0  0
 16 63  1  0  0  0  0
 17 19  1  0  0  0  0
 17 63  1  0  0  0  0
 22 23  3  0  0  0  0
 22 63  1  0  0  0  0
 23 24  1  0  0  0  0
 24 25  2  0  0  0  0
 24 26  1  0  0  0  0
 25 27  1  0  0  0  0
 25 67  1  0  0  0  0
 26 28  2  0  0  0  0
 26 71  1  0  0  0  0
 27 29  2  0  0  0  0
 27 65  1  0  0  0  0
 28 29  1  0  0  0  0
 28 66  1  0  0  0  0
 29 64  1  0  0  0  0
 30 31  2  0  0  0  0
 30 32  1  0  0  0  0
 31 33  1  0  0  0  0
 31 34  1  0  0  0  0
 32 35  2  0  0  0  0
 32 36  1  0  0  0  0
 33 37  2  0  0  0  0
 33 38  1  0  0  0  0
 35 37  1  0  0  0  0
 35 39  1  0  0  0  0
 37 40  1  0  0  0  0
 41 42  2  0  0  0  0
 41 43  1  0  0  0  0
```

```
 42 44  1  0  0  0  0
 42 45  1  0  0  0  0
 43 46  2  0  0  0  0
 43 47  1  0  0  0  0
 44 48  2  0  0  0  0
 44 49  1  0  0  0  0
 46 48  1  0  0  0  0
 46 50  1  0  0  0  0
 48 51  1  0  0  0  0
 52 53  2  0  0  0  0
 52 54  1  0  0  0  0
 53 55  1  0  0  0  0
 53 56  1  0  0  0  0
 54 57  2  0  0  0  0
 54 58  1  0  0  0  0
 55 59  2  0  0  0  0
 55 60  1  0  0  0  0
 57 59  1  0  0  0  0
 57 61  1  0  0  0  0
 59 62  1  0  0  0  0
 67 68  1  0  0  0  0
 67 69  1  0  0  0  0
 67 70  1  0  0  0  0
 71 72  1  0  0  0  0
 71 73  1  0  0  0  0
 71 74  1  0  0  0  0
M  END
$$$$
mol066
Optimized Catalyst 066
Optimized Catalyst 066
 68 75  0  0  0  0  0  0  0  0999 V2000
   -2.5651   -4.5932   -0.4872 P   0  0  0  0  0  0  0  0  0  0  0  0
    0.1913   -0.6363   -1.5915 C   0  0  2  0  0  0  0  0  0  0  0  0
    1.0821   -1.1688   -0.4371 C   0  0  1  0  0  0  0  0  0  0  0  0
   -1.1229   -0.0036   -1.2215 C   0  0  0  0  0  0  0  0  0  0  0  0
   -0.5391    0.9432    1.0576 C   0  0  2  0  0  0  0  0  0  0  0  0
   -1.4422    0.6850   -0.1186 C   0  0  0  0  0  0  0  0  0  0  0  0
   -0.5510   -0.2127    2.0945 C   0  0  2  0  0  0  0  0  0  0  0  0
   -1.8638   -0.0648   -2.0417 H   0  0  0  0  0  0  0  0  0  0  0  0
   -2.4304    1.1763   -0.0433 H   0  0  0  0  0  0  0  0  0  0  0  0
    0.7845    0.0717   -2.2386 H   0  0  0  0  0  0  0  0  0  0  0  0
    0.5027    1.2060    0.7443 H   0  0  0  0  0  0  0  0  0  0  0  0
   -0.4287   -1.6252    1.5624 C   0  0  0  0  0  0  0  0  0  0  0  0
    0.3374   -2.0445    0.5354 C   0  0  0  0  0  0  0  0  0  0  0  0
   -0.8347   -2.3897    2.2830 H   0  0  0  0  0  0  0  0  0  0  0  0
    0.5131   -3.1281    0.3727 H   0  0  0  0  0  0  0  0  0  0  0  0
    1.6309   -0.3377    0.0776 H   0  0  0  0  0  0  0  0  0  0  0  0
    0.2343   -0.0421    2.8852 H   0  0  0  0  0  0  0  0  0  0  0  0
   -4.6282   -2.3261    0.0090 N   0  0  0  0  0  0  0  0  0  0  0  0
   -5.7288   -2.3346   -0.3846 C   0  0  0  0  0  0  0  0  0  0  0  0
   -7.0594   -2.3757   -0.8817 C   0  0  0  0  0  0  0  0  0  0  0  0
   -7.4002   -3.3438   -1.8509 C   0  0  0  0  0  0  0  0  0  0  0  0
   -8.0212   -1.4590   -0.4106 C   0  0  0  0  0  0  0  0  0  0  0  0
   -8.7039   -3.3885   -2.3396 C   0  0  0  0  0  0  0  0  0  0  0  0
   -9.3215   -1.5193   -0.9129 C   0  0  0  0  0  0  0  0  0  0  0  0
   -9.6643   -2.4807   -1.8713 C   0  0  0  0  0  0  0  0  0  0  0  0
   -0.8224   -5.0622   -0.8657 C   0  0  0  0  0  0  0  0  0  0  0  0
   -0.0405   -5.6210    0.1564 C   0  0  0  0  0  0  0  0  0  0  0  0
   -0.3146   -4.9000   -2.1568 C   0  0  0  0  0  0  0  0  0  0  0  0
    1.2755   -5.9949   -0.1203 C   0  0  0  0  0  0  0  0  0  0  0  0
   -0.4320   -5.7886    1.1643 H   0  0  0  0  0  0  0  0  0  0  0  0
    1.0093   -5.2739   -2.4214 C   0  0  0  0  0  0  0  0  0  0  0  0
   -0.9159   -4.4949   -2.9766 H   0  0  0  0  0  0  0  0  0  0  0  0
    1.8034   -5.8154   -1.4073 C   0  0  0  0  0  0  0  0  0  0  0  0
```

```
    1.8960   -6.4364    0.6642 H   0  0  0  0  0  0  0  0  0  0  0  0
    1.4189   -5.1442   -3.4272 H   0  0  0  0  0  0  0  0  0  0  0  0
    2.8380   -6.1050   -1.6170 H   0  0  0  0  0  0  0  0  0  0  0  0
   -3.2339   -5.8737    0.6638 C   0  0  0  0  0  0  0  0  0  0  0  0
   -2.9509   -7.2229    0.4299 C   0  0  0  0  0  0  0  0  0  0  0  0
   -4.0219   -5.4739    1.7478 C   0  0  0  0  0  0  0  0  0  0  0  0
   -3.4925   -8.1888    1.2861 C   0  0  0  0  0  0  0  0  0  0  0  0
   -2.3101   -7.5501   -0.3955 H   0  0  0  0  0  0  0  0  0  0  0  0
   -4.5568   -6.4459    2.5979 C   0  0  0  0  0  0  0  0  0  0  0  0
   -4.2272   -4.4108    1.9623 H   0  0  0  0  0  0  0  0  0  0  0  0
   -4.2957   -7.8027    2.3640 C   0  0  0  0  0  0  0  0  0  0  0  0
   -3.2840   -9.2483    1.1129 H   0  0  0  0  0  0  0  0  0  0  0  0
   -5.1761   -6.1506    3.4490 H   0  0  0  0  0  0  0  0  0  0  0  0
   -4.7170   -8.5617    3.0313 H   0  0  0  0  0  0  0  0  0  0  0  0
   -3.5429   -4.6534   -2.0587 C   0  0  0  0  0  0  0  0  0  0  0  0
   -4.3092   -5.7793   -2.3629 C   0  0  0  0  0  0  0  0  0  0  0  0
   -3.4679   -3.5687   -2.9433 C   0  0  0  0  0  0  0  0  0  0  0  0
   -5.0070   -5.8230   -3.5782 C   0  0  0  0  0  0  0  0  0  0  0  0
   -4.3907   -6.6347   -1.6850 H   0  0  0  0  0  0  0  0  0  0  0  0
   -4.1622   -3.6242   -4.1534 C   0  0  0  0  0  0  0  0  0  0  0  0
   -2.8775   -2.6716   -2.7184 H   0  0  0  0  0  0  0  0  0  0  0  0
   -4.9286   -4.7540   -4.4736 C   0  0  0  0  0  0  0  0  0  0  0  0
   -5.6052   -6.7034   -3.8283 H   0  0  0  0  0  0  0  0  0  0  0  0
   -4.1052   -2.7897   -4.8574 H   0  0  0  0  0  0  0  0  0  0  0  0
   -5.4631   -4.7966   -5.4275 H   0  0  0  0  0  0  0  0  0  0  0  0
   -2.7930   -2.2435    0.5970 Ir  0  0  0  0  0  0  0  0  0  0  0  0
  -10.6884   -2.5246   -2.2571 H   0  0  0  0  0  0  0  0  0  0  0  0
   -8.9804   -4.1338   -3.0902 H   0  0  0  0  0  0  0  0  0  0  0  0
  -10.0760   -0.8114   -0.5550 H   0  0  0  0  0  0  0  0  0  0  0  0
   -6.6480   -4.0482   -2.2116 H   0  0  0  0  0  0  0  0  0  0  0  0
   -7.7529   -0.7107    0.3387 H   0  0  0  0  0  0  0  0  0  0  0  0
   -0.9963    2.0609    1.7065 F   0  0  0  0  0  0  0  0  0  0  0  0
   -1.7628   -0.1546    2.7447 F   0  0  0  0  0  0  0  0  0  0  0  0
    2.0653   -1.9357   -1.0100 F   0  0  0  0  0  0  0  0  0  0  0  0
   -0.1105   -1.7182   -2.3872 F   0  0  0  0  0  0  0  0  0  0  0  0
  1 26  1  0  0  0  0
  1 37  1  0  0  0  0
  1 48  1  0  0  0  0
  1 59  1  0  0  0  0
  2  3  1  0  0  0  0
  2  4  1  0  0  0  0
  2 10  1  6  0  0  0
  2 68  1  0  0  0  0
  3 13  1  0  0  0  0
  3 16  1  1  0  0  0
  3 67  1  0  0  0  0
  4  6  2  0  0  0  0
  4  8  1  0  0  0  0
  4 59  1  0  0  0  0
  5  6  1  0  0  0  0
  5  7  1  0  0  0  0
  5 11  1  6  0  0  0
  5 65  1  0  0  0  0
  6  9  1  0  0  0  0
  6 59  1  0  0  0  0
  7 12  1  0  0  0  0
  7 17  1  1  0  0  0
  7 66  1  0  0  0  0
 12 13  2  0  0  0  0
 12 14  1  0  0  0  0
 12 59  1  0  0  0  0
 13 15  1  0  0  0  0
 13 59  1  0  0  0  0
 18 19  3  0  0  0  0
 18 59  1  0  0  0  0
```

```
 19 20  1  0  0  0  0
 20 21  2  0  0  0  0
 20 22  1  0  0  0  0
 21 23  1  0  0  0  0
 21 63  1  0  0  0  0
 22 24  2  0  0  0  0
 22 64  1  0  0  0  0
 23 25  2  0  0  0  0
 23 61  1  0  0  0  0
 24 25  1  0  0  0  0
 24 62  1  0  0  0  0
 25 60  1  0  0  0  0
 26 27  2  0  0  0  0
 26 28  1  0  0  0  0
 27 29  1  0  0  0  0
 27 30  1  0  0  0  0
 28 31  2  0  0  0  0
 28 32  1  0  0  0  0
 29 33  2  0  0  0  0
 29 34  1  0  0  0  0
 31 33  1  0  0  0  0
 31 35  1  0  0  0  0
 33 36  1  0  0  0  0
 37 38  2  0  0  0  0
 37 39  1  0  0  0  0
 38 40  1  0  0  0  0
 38 41  1  0  0  0  0
 39 42  2  0  0  0  0
 39 43  1  0  0  0  0
 40 44  2  0  0  0  0
 40 45  1  0  0  0  0
 42 44  1  0  0  0  0
 42 46  1  0  0  0  0
 44 47  1  0  0  0  0
 48 49  2  0  0  0  0
 48 50  1  0  0  0  0
 49 51  1  0  0  0  0
 49 52  1  0  0  0  0
 50 53  2  0  0  0  0
 50 54  1  0  0  0  0
 51 55  2  0  0  0  0
 51 56  1  0  0  0  0
 53 55  1  0  0  0  0
 53 57  1  0  0  0  0
 55 58  1  0  0  0  0
M  END
$$$$
mol067
Optimized Catalyst 067
Optimized Catalyst 067
 80 87  0  0  0  0  0  0  0  0999 V2000
   -2.4506   -4.6255   -0.7360 P   0  0  0  0  0  0  0  0  0  0  0  0
   -0.4872    0.5259   -1.0990 C   0  0  2  0  0  0  0  0  0  0  0  0
    0.7302   -0.2485   -0.5152 C   0  0  1  0  0  0  0  0  0  0  0  0
   -1.6657    0.6964   -0.1609 C   0  0  0  0  0  0  0  0  0  0  0  0
   -0.6733    0.3584    2.1906 C   0  0  2  0  0  0  0  0  0  0  0  0
   -1.7590    0.6356    1.1797 C   0  0  0  0  0  0  0  0  0  0  0  0
   -0.4586   -1.1679    2.4113 C   0  0  2  0  0  0  0  0  0  0  0  0
   -2.5692    1.0284   -0.7113 H   0  0  0  0  0  0  0  0  0  0  0  0
   -2.7282    0.8866    1.6451 H   0  0  0  0  0  0  0  0  0  0  0  0
   -0.1209    1.5685   -1.3317 H   0  0  0  0  0  0  0  0  0  0  0  0
    0.2938    0.7980    1.8284 H   0  0  0  0  0  0  0  0  0  0  0  0
   -0.1652   -1.9888    1.1634 C   0  0  0  0  0  0  0  0  0  0  0  0
    0.3488   -1.6294   -0.0379 C   0  0  0  0  0  0  0  0  0  0  0  0
   -0.1936   -3.0768    1.3969 H   0  0  0  0  0  0  0  0  0  0  0  0
```

```
    0.6233   -2.4313   -0.7531 H   0  0  0  0  0  0  0  0  0  0  0  0
    1.1309    0.3432    0.3524 H   0  0  0  0  0  0  0  0  0  0  0  0
    0.4569   -1.2689    3.0658 H   0  0  0  0  0  0  0  0  0  0  0  0
   -4.2960   -2.1224   -0.6600 N   0  0  0  0  0  0  0  0  0  0  0  0
   -5.4076   -2.1359   -1.0197 C   0  0  0  0  0  0  0  0  0  0  0  0
   -6.7588   -2.1700   -1.4627 C   0  0  0  0  0  0  0  0  0  0  0  0
   -7.2764   -3.3613   -2.0124 C   0  0  0  0  0  0  0  0  0  0  0  0
   -7.5653   -1.0193   -1.3512 C   0  0  0  0  0  0  0  0  0  0  0  0
   -8.6001   -3.3911   -2.4489 C   0  0  0  0  0  0  0  0  0  0  0  0
   -8.8874   -1.0683   -1.7935 C   0  0  0  0  0  0  0  0  0  0  0  0
   -9.4052   -2.2495   -2.3394 C   0  0  0  0  0  0  0  0  0  0  0  0
   -0.7764   -5.2756   -1.1864 C   0  0  0  0  0  0  0  0  0  0  0  0
    0.1149   -5.6073   -0.1574 C   0  0  0  0  0  0  0  0  0  0  0  0
   -0.4333   -5.4711   -2.5263 C   0  0  0  0  0  0  0  0  0  0  0  0
    1.3712   -6.1249   -0.4814 C   0  0  0  0  0  0  0  0  0  0  0  0
   -0.1454   -5.4957    0.8987 H   0  0  0  0  0  0  0  0  0  0  0  0
    0.8292   -5.9901   -2.8406 C   0  0  0  0  0  0  0  0  0  0  0  0
   -1.1220   -5.2462   -3.3456 H   0  0  0  0  0  0  0  0  0  0  0  0
    1.7302   -6.3147   -1.8226 C   0  0  0  0  0  0  0  0  0  0  0  0
    2.0740   -6.3912    0.3120 H   0  0  0  0  0  0  0  0  0  0  0  0
    1.1064   -6.1493   -3.8859 H   0  0  0  0  0  0  0  0  0  0  0  0
    2.7139   -6.7236   -2.0713 H   0  0  0  0  0  0  0  0  0  0  0  0
   -3.0527   -5.6434    0.6906 C   0  0  0  0  0  0  0  0  0  0  0  0
   -2.7377   -7.0032    0.7594 C   0  0  0  0  0  0  0  0  0  0  0  0
   -3.8401   -5.0305    1.6706 C   0  0  0  0  0  0  0  0  0  0  0  0
   -3.2287   -7.7622    1.8286 C   0  0  0  0  0  0  0  0  0  0  0  0
   -2.1145   -7.4950    0.0056 H   0  0  0  0  0  0  0  0  0  0  0  0
   -4.3294   -5.7974    2.7322 C   0  0  0  0  0  0  0  0  0  0  0  0
   -4.0872   -3.9593    1.6360 H   0  0  0  0  0  0  0  0  0  0  0  0
   -4.0245   -7.1626    2.8098 C   0  0  0  0  0  0  0  0  0  0  0  0
   -2.9881   -8.8264    1.8963 H   0  0  0  0  0  0  0  0  0  0  0  0
   -4.9495   -5.3342    3.5033 H   0  0  0  0  0  0  0  0  0  0  0  0
   -4.4087   -7.7597    3.6422 H   0  0  0  0  0  0  0  0  0  0  0  0
   -3.5851   -4.9685   -2.1665 C   0  0  0  0  0  0  0  0  0  0  0  0
   -4.4283   -6.0801   -2.1304 C   0  0  0  0  0  0  0  0  0  0  0  0
   -3.5536   -4.1234   -3.2833 C   0  0  0  0  0  0  0  0  0  0  0  0
   -5.2490   -6.3538   -3.2334 C   0  0  0  0  0  0  0  0  0  0  0  0
   -4.4747   -6.7518   -1.2670 H   0  0  0  0  0  0  0  0  0  0  0  0
   -4.3732   -4.4056   -4.3790 C   0  0  0  0  0  0  0  0  0  0  0  0
   -2.9040   -3.2434   -3.3319 H   0  0  0  0  0  0  0  0  0  0  0  0
   -5.2186   -5.5233   -4.3561 C   0  0  0  0  0  0  0  0  0  0  0  0
   -5.9059   -7.2273   -3.2169 H   0  0  0  0  0  0  0  0  0  0  0  0
   -4.3526   -3.7583   -5.2593 H   0  0  0  0  0  0  0  0  0  0  0  0
   -5.8518   -5.7448   -5.2199 H   0  0  0  0  0  0  0  0  0  0  0  0
   -2.4591   -2.1122   -0.0743 Ir  0  0  0  0  0  0  0  0  0  0  0  0
  -10.4448   -2.2805   -2.6820 H   0  0  0  0  0  0  0  0  0  0  0  0
   -9.0127   -4.3079   -2.8778 H   0  0  0  0  0  0  0  0  0  0  0  0
   -9.5215   -0.1801   -1.7140 H   0  0  0  0  0  0  0  0  0  0  0  0
   -6.6447   -4.2484   -2.0946 H   0  0  0  0  0  0  0  0  0  0  0  0
   -7.1598   -0.1003   -0.9234 H   0  0  0  0  0  0  0  0  0  0  0  0
   -1.6461   -1.8288    3.1412 C   0  0  0  0  0  0  0  0  0  0  0  0
   -1.4032   -2.8803    3.4239 H   0  0  0  0  0  0  0  0  0  0  0  0
   -1.8707   -1.2831    4.0892 H   0  0  0  0  0  0  0  0  0  0  0  0
   -2.5729   -1.8091    2.4961 H   0  0  0  0  0  0  0  0  0  0  0  0
   -0.9697    1.0999    3.5060 C   0  0  0  0  0  0  0  0  0  0  0  0
   -0.2079    0.8397    4.2766 H   0  0  0  0  0  0  0  0  0  0  0  0
   -0.9194    2.2013    3.3271 H   0  0  0  0  0  0  0  0  0  0  0  0
   -1.9804    0.8645    3.9050 H   0  0  0  0  0  0  0  0  0  0  0  0
   -1.0014   -0.1048   -2.4102 C   0  0  0  0  0  0  0  0  0  0  0  0
   -1.7373    0.5746   -2.9031 H   0  0  0  0  0  0  0  0  0  0  0  0
   -0.1629   -0.2553   -3.1290 H   0  0  0  0  0  0  0  0  0  0  0  0
   -1.4938   -1.1013   -2.2074 H   0  0  0  0  0  0  0  0  0  0  0  0
    1.9016   -0.3367   -1.5077 C   0  0  0  0  0  0  0  0  0  0  0  0
    2.1294    0.6756   -1.9183 H   0  0  0  0  0  0  0  0  0  0  0  0
    2.8118   -0.7061   -0.9771 H   0  0  0  0  0  0  0  0  0  0  0  0
```

```
    1.6902   -1.0238   -2.3553 H   0  0  0  0  0  0  0  0  0  0  0  0
  1 26  1  0  0  0  0
  1 37  1  0  0  0  0
  1 48  1  0  0  0  0
  1 59  1  0  0  0  0
  2  3  1  0  0  0  0
  2  4  1  0  0  0  0
  2 10  1  6  0  0  0
  2 73  1  0  0  0  0
  3 13  1  0  0  0  0
  3 16  1  1  0  0  0
  3 77  1  0  0  0  0
  4  6  2  0  0  0  0
  4  8  1  0  0  0  0
  4 59  1  0  0  0  0
  5  6  1  0  0  0  0
  5  7  1  0  0  0  0
  5 11  1  6  0  0  0
  5 69  1  0  0  0  0
  6  9  1  0  0  0  0
  6 59  1  0  0  0  0
  7 12  1  0  0  0  0
  7 17  1  1  0  0  0
  7 65  1  0  0  0  0
 12 13  2  0  0  0  0
 12 14  1  0  0  0  0
 12 59  1  0  0  0  0
 13 15  1  0  0  0  0
 13 59  1  0  0  0  0
 18 19  3  0  0  0  0
 18 59  1  0  0  0  0
 19 20  1  0  0  0  0
 20 21  2  0  0  0  0
 20 22  1  0  0  0  0
 21 23  1  0  0  0  0
 21 63  1  0  0  0  0
 22 24  2  0  0  0  0
 22 64  1  0  0  0  0
 23 25  2  0  0  0  0
 23 61  1  0  0  0  0
 24 25  1  0  0  0  0
 24 62  1  0  0  0  0
 25 60  1  0  0  0  0
 26 27  2  0  0  0  0
 26 28  1  0  0  0  0
 27 29  1  0  0  0  0
 27 30  1  0  0  0  0
 28 31  2  0  0  0  0
 28 32  1  0  0  0  0
 29 33  2  0  0  0  0
 29 34  1  0  0  0  0
 31 33  1  0  0  0  0
 31 35  1  0  0  0  0
 33 36  1  0  0  0  0
 37 38  2  0  0  0  0
 37 39  1  0  0  0  0
 38 40  1  0  0  0  0
 38 41  1  0  0  0  0
 39 42  2  0  0  0  0
 39 43  1  0  0  0  0
 40 44  2  0  0  0  0
 40 45  1  0  0  0  0
 42 44  1  0  0  0  0
 42 46  1  0  0  0  0
 44 47  1  0  0  0  0
```

```
 48 49  2  0  0  0  0
 48 50  1  0  0  0  0
 49 51  1  0  0  0  0
 49 52  1  0  0  0  0
 50 53  2  0  0  0  0
 50 54  1  0  0  0  0
 51 55  2  0  0  0  0
 51 56  1  0  0  0  0
 53 55  1  0  0  0  0
 53 57  1  0  0  0  0
 55 58  1  0  0  0  0
 65 66  1  0  0  0  0
 65 67  1  0  0  0  0
 65 68  1  0  0  0  0
 69 70  1  0  0  0  0
 69 71  1  0  0  0  0
 69 72  1  0  0  0  0
 73 74  1  0  0  0  0
 73 75  1  0  0  0  0
 73 76  1  0  0  0  0
 77 78  1  0  0  0  0
 77 79  1  0  0  0  0
 77 80  1  0  0  0  0
M  END
$$$$
mol068
Optimized Catalyst 068
Optimized Catalyst 068
 68 75  0  0  0  0  0  0  0  0999 V2000
   -2.4509   -4.6654   -0.7417 P   0  0  0  0  0  0  0  0  0  0  0  0
   -0.4310    0.5784   -0.9911 C   0  0  0  0  0  0  0  0  0  0  0  0
    0.6774   -0.3688   -0.4907 C   0  0  0  0  0  0  0  0  0  0  0  0
   -1.7673    0.4933   -0.3010 C   0  0  0  0  0  0  0  0  0  0  0  0
   -0.9187    0.2825    2.0792 C   0  0  0  0  0  0  0  0  0  0  0  0
   -1.9906    0.3620    1.0270 C   0  0  0  0  0  0  0  0  0  0  0  0
   -0.4049   -1.1463    2.3450 C   0  0  0  0  0  0  0  0  0  0  0  0
   -2.6216    0.6844   -0.9806 H   0  0  0  0  0  0  0  0  0  0  0  0
   -3.0207    0.4142    1.4242 H   0  0  0  0  0  0  0  0  0  0  0  0
   -0.0662    1.6399   -0.8697 H   0  0  0  0  0  0  0  0  0  0  0  0
   -0.0683    0.9542    1.7892 H   0  0  0  0  0  0  0  0  0  0  0  0
   -0.2665   -2.0688    1.1552 C   0  0  0  0  0  0  0  0  0  0  0  0
    0.2148   -1.7394   -0.0779 C   0  0  0  0  0  0  0  0  0  0  0  0
   -0.3337   -3.1402    1.4465 H   0  0  0  0  0  0  0  0  0  0  0  0
    0.4399   -2.5356   -0.8163 H   0  0  0  0  0  0  0  0  0  0  0  0
    1.2182    0.1002    0.3763 H   0  0  0  0  0  0  0  0  0  0  0  0
    0.6197   -1.0676    2.8135 H   0  0  0  0  0  0  0  0  0  0  0  0
   -4.2605   -2.1147   -0.6609 N   0  0  0  0  0  0  0  0  0  0  0  0
   -5.3747   -2.1058   -1.0150 C   0  0  0  0  0  0  0  0  0  0  0  0
   -6.7270   -2.1097   -1.4538 C   0  0  0  0  0  0  0  0  0  0  0  0
   -7.2091   -3.2073   -2.1983 C   0  0  0  0  0  0  0  0  0  0  0  0
   -7.5725   -1.0239   -1.1461 C   0  0  0  0  0  0  0  0  0  0  0  0
   -8.5324   -3.2069   -2.6351 C   0  0  0  0  0  0  0  0  0  0  0  0
   -8.8949   -1.0426   -1.5898 C   0  0  0  0  0  0  0  0  0  0  0  0
   -9.3750   -2.1285   -2.3321 C   0  0  0  0  0  0  0  0  0  0  0  0
   -0.7816   -5.3430   -1.1637 C   0  0  0  0  0  0  0  0  0  0  0  0
    0.0603   -5.7464   -0.1159 C   0  0  0  0  0  0  0  0  0  0  0  0
   -0.3889   -5.4832   -2.5001 C   0  0  0  0  0  0  0  0  0  0  0  0
    1.3213   -6.2678   -0.4000 C   0  0  0  0  0  0  0  0  0  0  0  0
   -0.2451   -5.6817    0.9341 H   0  0  0  0  0  0  0  0  0  0  0  0
    0.8719   -6.0028   -2.8032 C   0  0  0  0  0  0  0  0  0  0  0  0
   -1.0443   -5.2077   -3.3329 H   0  0  0  0  0  0  0  0  0  0  0  0
    1.7044   -6.3831   -1.7442 C   0  0  0  0  0  0  0  0  0  0  0  0
    1.9915   -6.5890    0.4027 H   0  0  0  0  0  0  0  0  0  0  0  0
    1.1964   -6.1203   -3.8417 H   0  0  0  0  0  0  0  0  0  0  0  0
   -3.0666   -5.6517    0.6963 C   0  0  0  0  0  0  0  0  0  0  0  0
```

```
   -2.8720   -7.0386    0.7321 C   0  0  0  0  0  0  0  0  0  0  0  0
   -3.7235   -4.9818    1.7366 C   0  0  0  0  0  0  0  0  0  0  0  0
   -3.3555   -7.7749    1.8153 C   0  0  0  0  0  0  0  0  0  0  0  0
   -2.3404   -7.5711   -0.0656 H   0  0  0  0  0  0  0  0  0  0  0  0
   -4.2141   -5.7013    2.8264 C   0  0  0  0  0  0  0  0  0  0  0  0
   -3.8632   -3.8863    1.7274 H   0  0  0  0  0  0  0  0  0  0  0  0
   -4.0236   -7.0908    2.8386 C   0  0  0  0  0  0  0  0  0  0  0  0
   -3.2126   -8.8588    1.8664 H   0  0  0  0  0  0  0  0  0  0  0  0
   -4.7304   -5.2009    3.6509 H   0  0  0  0  0  0  0  0  0  0  0  0
   -3.5660   -4.9962   -2.1848 C   0  0  0  0  0  0  0  0  0  0  0  0
   -4.4422   -6.0859   -2.1639 C   0  0  0  0  0  0  0  0  0  0  0  0
   -3.4769   -4.1591   -3.3076 C   0  0  0  0  0  0  0  0  0  0  0  0
   -5.2390   -6.3571   -3.2810 C   0  0  0  0  0  0  0  0  0  0  0  0
   -4.5368   -6.7422   -1.2911 H   0  0  0  0  0  0  0  0  0  0  0  0
   -4.2578   -4.4189   -4.4324 C   0  0  0  0  0  0  0  0  0  0  0  0
   -2.8077   -3.2897   -3.3343 H   0  0  0  0  0  0  0  0  0  0  0  0
   -5.1227   -5.5233   -4.3974 C   0  0  0  0  0  0  0  0  0  0  0  0
   -5.9248   -7.2101   -3.2849 H   0  0  0  0  0  0  0  0  0  0  0  0
   -4.1942   -3.7856   -5.3224 H   0  0  0  0  0  0  0  0  0  0  0  0
   -2.4111   -2.1455   -0.0917 Ir  0  0  0  0  0  0  0  0  0  0  0  0
  -10.4146   -2.1359   -2.6768 H   0  0  0  0  0  0  0  0  0  0  0  0
   -8.9161   -4.0501   -3.2167 H   0  0  0  0  0  0  0  0  0  0  0  0
   -9.5598   -0.2048   -1.3570 H   0  0  0  0  0  0  0  0  0  0  0  0
   -6.5518   -4.0475   -2.4269 H   0  0  0  0  0  0  0  0  0  0  0  0
   -7.1979   -0.1783   -0.5662 H   0  0  0  0  0  0  0  0  0  0  0  0
   -1.3274    0.6888    3.0453 H   0  0  0  0  0  0  0  0  0  0  0  0
   -1.0788   -1.6294    3.1045 H   0  0  0  0  0  0  0  0  0  0  0  0
   -0.5748    0.4045   -2.0913 H   0  0  0  0  0  0  0  0  0  0  0  0
    1.4341   -0.4766   -1.3169 H   0  0  0  0  0  0  0  0  0  0  0  0
   -5.8730   -5.7743   -5.4628 F   0  0  0  0  0  0  0  0  0  0  0  0
   -4.4930   -7.7846    3.8673 F   0  0  0  0  0  0  0  0  0  0  0  0
    2.9048   -6.8748   -2.0221 F   0  0  0  0  0  0  0  0  0  0  0  0
  1 26  1  0  0  0  0
  1 36  1  0  0  0  0
  1 46  1  0  0  0  0
  1 56  1  0  0  0  0
  2  3  1  0  0  0  0
  2  4  1  0  0  0  0
  2 10  1  0  0  0  0
  2 64  1  0  0  0  0
  3 13  1  0  0  0  0
  3 16  1  0  0  0  0
  3 65  1  0  0  0  0
  4  6  2  0  0  0  0
  4  8  1  0  0  0  0
  4 56  1  0  0  0  0
  5  6  1  0  0  0  0
  5  7  1  0  0  0  0
  5 11  1  0  0  0  0
  5 62  1  0  0  0  0
  6  9  1  0  0  0  0
  6 56  1  0  0  0  0
  7 12  1  0  0  0  0
  7 17  1  0  0  0  0
  7 63  1  0  0  0  0
 12 13  2  0  0  0  0
 12 14  1  0  0  0  0
 12 56  1  0  0  0  0
 13 15  1  0  0  0  0
 13 56  1  0  0  0  0
 18 19  3  0  0  0  0
 18 56  1  0  0  0  0
 19 20  1  0  0  0  0
 20 21  2  0  0  0  0
 20 22  1  0  0  0  0
```

```
 21 23  1  0  0  0  0
 21 60  1  0  0  0  0
 22 24  2  0  0  0  0
 22 61  1  0  0  0  0
 23 25  2  0  0  0  0
 23 58  1  0  0  0  0
 24 25  1  0  0  0  0
 24 59  1  0  0  0  0
 25 57  1  0  0  0  0
 26 27  2  0  0  0  0
 26 28  1  0  0  0  0
 27 29  1  0  0  0  0
 27 30  1  0  0  0  0
 28 31  2  0  0  0  0
 28 32  1  0  0  0  0
 29 33  2  0  0  0  0
 29 34  1  0  0  0  0
 31 33  1  0  0  0  0
 31 35  1  0  0  0  0
 33 68  1  0  0  0  0
 36 37  2  0  0  0  0
 36 38  1  0  0  0  0
 37 39  1  0  0  0  0
 37 40  1  0  0  0  0
 38 41  2  0  0  0  0
 38 42  1  0  0  0  0
 39 43  2  0  0  0  0
 39 44  1  0  0  0  0
 41 43  1  0  0  0  0
 41 45  1  0  0  0  0
 43 67  1  0  0  0  0
 46 47  2  0  0  0  0
 46 48  1  0  0  0  0
 47 49  1  0  0  0  0
 47 50  1  0  0  0  0
 48 51  2  0  0  0  0
 48 52  1  0  0  0  0
 49 53  2  0  0  0  0
 49 54  1  0  0  0  0
 51 53  1  0  0  0  0
 51 55  1  0  0  0  0
 53 66  1  0  0  0  0
M  END
$$$$
mol069
Optimized Catalyst 069
Optimized Catalyst 069
 77 84  0  0  0  0  0  0  0  0999 V2000
   -2.4362   -4.6473   -0.7323 P   0  0  0  0  0  0  0  0  0  0  0  0
   -0.4245    0.5928   -1.0013 C   0  0  0  0  0  0  0  0  0  0  0  0
    0.6830   -0.3496   -0.4888 C   0  0  0  0  0  0  0  0  0  0  0  0
   -1.7534    0.5369   -0.2945 C   0  0  0  0  0  0  0  0  0  0  0  0
   -0.8873    0.3538    2.0814 C   0  0  0  0  0  0  0  0  0  0  0  0
   -1.9652    0.4313    1.0361 C   0  0  0  0  0  0  0  0  0  0  0  0
   -0.3972   -1.0797    2.3665 C   0  0  0  0  0  0  0  0  0  0  0  0
   -2.6130    0.7186   -0.9691 H   0  0  0  0  0  0  0  0  0  0  0  0
   -2.9906    0.4931    1.4410 H   0  0  0  0  0  0  0  0  0  0  0  0
   -0.0488    1.6530   -0.9131 H   0  0  0  0  0  0  0  0  0  0  0  0
   -0.0281    1.0058    1.7755 H   0  0  0  0  0  0  0  0  0  0  0  0
   -0.2584   -2.0196    1.1904 C   0  0  0  0  0  0  0  0  0  0  0  0
    0.2187   -1.7118   -0.0497 C   0  0  0  0  0  0  0  0  0  0  0  0
   -0.3354   -3.0871    1.4975 H   0  0  0  0  0  0  0  0  0  0  0  0
    0.4419   -2.5215   -0.7749 H   0  0  0  0  0  0  0  0  0  0  0  0
    1.2281    0.1332    0.3672 H   0  0  0  0  0  0  0  0  0  0  0  0
    0.6228   -1.0097    2.8448 H   0  0  0  0  0  0  0  0  0  0  0  0
```

```
   -4.2445   -2.1248   -0.6405 N   0  0  0  0  0  0  0  0  0  0  0  0
   -5.3559   -2.1302   -0.9997 C   0  0  0  0  0  0  0  0  0  0  0  0
   -6.7058   -2.1593   -1.4453 C   0  0  0  0  0  0  0  0  0  0  0  0
   -7.2196   -3.3509   -1.9975 C   0  0  0  0  0  0  0  0  0  0  0  0
   -7.5136   -1.0097   -1.3346 C   0  0  0  0  0  0  0  0  0  0  0  0
   -8.5417   -3.3827   -2.4374 C   0  0  0  0  0  0  0  0  0  0  0  0
   -8.8345   -1.0608   -1.7798 C   0  0  0  0  0  0  0  0  0  0  0  0
   -9.3482   -2.2421   -2.3291 C   0  0  0  0  0  0  0  0  0  0  0  0
   -0.7813   -5.3221   -1.1895 C   0  0  0  0  0  0  0  0  0  0  0  0
    0.1117   -5.6795   -0.1693 C   0  0  0  0  0  0  0  0  0  0  0  0
   -0.4465   -5.5329   -2.5315 C   0  0  0  0  0  0  0  0  0  0  0  0
    1.3523   -6.2215   -0.4966 C   0  0  0  0  0  0  0  0  0  0  0  0
   -0.1420   -5.5636    0.8886 H   0  0  0  0  0  0  0  0  0  0  0  0
    0.7975   -6.0761   -2.8542 C   0  0  0  0  0  0  0  0  0  0  0  0
   -1.1376   -5.2955   -3.3463 H   0  0  0  0  0  0  0  0  0  0  0  0
    1.7138   -6.4221   -1.8441 C   0  0  0  0  0  0  0  0  0  0  0  0
    2.0463   -6.5050    0.2973 H   0  0  0  0  0  0  0  0  0  0  0  0
    1.0588   -6.2455   -3.9011 H   0  0  0  0  0  0  0  0  0  0  0  0
   -3.0371   -5.6288    0.7102 C   0  0  0  0  0  0  0  0  0  0  0  0
   -2.8050   -7.0074    0.7609 C   0  0  0  0  0  0  0  0  0  0  0  0
   -3.7363   -4.9821    1.7363 C   0  0  0  0  0  0  0  0  0  0  0  0
   -3.2965   -7.7468    1.8378 C   0  0  0  0  0  0  0  0  0  0  0  0
   -2.2395   -7.5267   -0.0204 H   0  0  0  0  0  0  0  0  0  0  0  0
   -4.2265   -5.7239    2.8085 C   0  0  0  0  0  0  0  0  0  0  0  0
   -3.9053   -3.8922    1.7256 H   0  0  0  0  0  0  0  0  0  0  0  0
   -4.0183   -7.1178    2.8680 C   0  0  0  0  0  0  0  0  0  0  0  0
   -3.1138   -8.8227    1.8808 H   0  0  0  0  0  0  0  0  0  0  0  0
   -4.7724   -5.2273    3.6131 H   0  0  0  0  0  0  0  0  0  0  0  0
   -3.5819   -4.9583   -2.1514 C   0  0  0  0  0  0  0  0  0  0  0  0
   -4.4379   -6.0629   -2.1301 C   0  0  0  0  0  0  0  0  0  0  0  0
   -3.5430   -4.1091   -3.2657 C   0  0  0  0  0  0  0  0  0  0  0  0
   -5.2507   -6.3256   -3.2353 C   0  0  0  0  0  0  0  0  0  0  0  0
   -4.4985   -6.7350   -1.2675 H   0  0  0  0  0  0  0  0  0  0  0  0
   -4.3539   -4.3764   -4.3665 C   0  0  0  0  0  0  0  0  0  0  0  0
   -2.8895   -3.2293   -3.3013 H   0  0  0  0  0  0  0  0  0  0  0  0
   -5.2089   -5.4970   -4.3702 C   0  0  0  0  0  0  0  0  0  0  0  0
   -5.9152   -7.1923   -3.2225 H   0  0  0  0  0  0  0  0  0  0  0  0
   -4.3226   -3.7169   -5.2363 H   0  0  0  0  0  0  0  0  0  0  0  0
   -2.3955   -2.1342   -0.0743 Ir  0  0  0  0  0  0  0  0  0  0  0  0
  -10.3863   -2.2742   -2.6749 H   0  0  0  0  0  0  0  0  0  0  0  0
   -8.9516   -4.3010   -2.8644 H   0  0  0  0  0  0  0  0  0  0  0  0
   -9.4713   -0.1749   -1.6984 H   0  0  0  0  0  0  0  0  0  0  0  0
   -6.5855   -4.2368   -2.0784 H   0  0  0  0  0  0  0  0  0  0  0  0
   -7.1109   -0.0905   -0.9052 H   0  0  0  0  0  0  0  0  0  0  0  0
   -1.2832    0.7817    3.0434 H   0  0  0  0  0  0  0  0  0  0  0  0
   -1.0881   -1.5446    3.1220 H   0  0  0  0  0  0  0  0  0  0  0  0
   -0.5847    0.3888   -2.0941 H   0  0  0  0  0  0  0  0  0  0  0  0
    1.4344   -0.4750   -1.3169 H   0  0  0  0  0  0  0  0  0  0  0  0
    3.0356   -7.0103   -2.1984 C   0  0  0  0  0  0  0  0  0  0  0  0
    2.9082   -8.1008   -2.4233 H   0  0  0  0  0  0  0  0  0  0  0  0
    3.4665   -6.5162   -3.1036 H   0  0  0  0  0  0  0  0  0  0  0  0
    3.7731   -6.9103   -1.3663 H   0  0  0  0  0  0  0  0  0  0  0  0
   -6.0733   -5.7925   -5.5472 C   0  0  0  0  0  0  0  0  0  0  0  0
   -6.0870   -6.8899   -5.7646 H   0  0  0  0  0  0  0  0  0  0  0  0
   -7.1214   -5.4625   -5.3382 H   0  0  0  0  0  0  0  0  0  0  0  0
   -5.7174   -5.2680   -6.4669 H   0  0  0  0  0  0  0  0  0  0  0  0
   -4.5441   -7.8997    4.0218 C   0  0  0  0  0  0  0  0  0  0  0  0
   -3.8960   -7.7240    4.9187 H   0  0  0  0  0  0  0  0  0  0  0  0
   -5.5860   -7.5842    4.2763 H   0  0  0  0  0  0  0  0  0  0  0  0
   -4.5573   -8.9961    3.8123 H   0  0  0  0  0  0  0  0  0  0  0  0
  1 26  1  0  0  0  0
  1 36  1  0  0  0  0
  1 46  1  0  0  0  0
  1 56  1  0  0  0  0
  2  3  1  0  0  0  0
```

```
  2  4  1  0  0  0  0
  2 10  1  0  0  0  0
  2 64  1  0  0  0  0
  3 13  1  0  0  0  0
  3 16  1  0  0  0  0
  3 65  1  0  0  0  0
  4  6  2  0  0  0  0
  4  8  1  0  0  0  0
  4 56  1  0  0  0  0
  5  6  1  0  0  0  0
  5  7  1  0  0  0  0
  5 11  1  0  0  0  0
  5 62  1  0  0  0  0
  6  9  1  0  0  0  0
  6 56  1  0  0  0  0
  7 12  1  0  0  0  0
  7 17  1  0  0  0  0
  7 63  1  0  0  0  0
 12 13  2  0  0  0  0
 12 14  1  0  0  0  0
 12 56  1  0  0  0  0
 13 15  1  0  0  0  0
 13 56  1  0  0  0  0
 18 19  3  0  0  0  0
 18 56  1  0  0  0  0
 19 20  1  0  0  0  0
 20 21  2  0  0  0  0
 20 22  1  0  0  0  0
 21 23  1  0  0  0  0
 21 60  1  0  0  0  0
 22 24  2  0  0  0  0
 22 61  1  0  0  0  0
 23 25  2  0  0  0  0
 23 58  1  0  0  0  0
 24 25  1  0  0  0  0
 24 59  1  0  0  0  0
 25 57  1  0  0  0  0
 26 27  2  0  0  0  0
 26 28  1  0  0  0  0
 27 29  1  0  0  0  0
 27 30  1  0  0  0  0
 28 31  2  0  0  0  0
 28 32  1  0  0  0  0
 29 33  2  0  0  0  0
 29 34  1  0  0  0  0
 31 33  1  0  0  0  0
 31 35  1  0  0  0  0
 33 66  1  0  0  0  0
 36 37  2  0  0  0  0
 36 38  1  0  0  0  0
 37 39  1  0  0  0  0
 37 40  1  0  0  0  0
 38 41  2  0  0  0  0
 38 42  1  0  0  0  0
 39 43  2  0  0  0  0
 39 44  1  0  0  0  0
 41 43  1  0  0  0  0
 41 45  1  0  0  0  0
 43 74  1  0  0  0  0
 46 47  2  0  0  0  0
 46 48  1  0  0  0  0
 47 49  1  0  0  0  0
 47 50  1  0  0  0  0
 48 51  2  0  0  0  0
 48 52  1  0  0  0  0
```

```
 49 53  2  0  0  0  0
 49 54  1  0  0  0  0
 51 53  1  0  0  0  0
 51 55  1  0  0  0  0
 53 70  1  0  0  0  0
 66 67  1  0  0  0  0
 66 68  1  0  0  0  0
 66 69  1  0  0  0  0
 70 71  1  0  0  0  0
 70 72  1  0  0  0  0
 70 73  1  0  0  0  0
 74 75  1  0  0  0  0
 74 76  1  0  0  0  0
 74 77  1  0  0  0  0
M  END
$$$$
mol070
Optimized Catalyst 070
Optimized Catalyst 070
 68 75  0  0  0  0  0  0  0  0999 V2000
   -2.3892   -4.5549   -0.7286 P   0  0  0  0  0  0  0  0  0  0  0  0
   -0.3688    0.7014   -0.8269 C   0  0  0  0  0  0  0  0  0  0  0  0
    0.7399   -0.2417   -0.3188 C   0  0  0  0  0  0  0  0  0  0  0  0
   -1.7221    0.5752   -0.1760 C   0  0  0  0  0  0  0  0  0  0  0  0
   -0.9251    0.3626    2.2230 C   0  0  0  0  0  0  0  0  0  0  0  0
   -1.9750    0.4209    1.1469 C   0  0  0  0  0  0  0  0  0  0  0  0
   -0.3718   -1.0506    2.4915 C   0  0  0  0  0  0  0  0  0  0  0  0
   -2.5634    0.7701   -0.8724 H   0  0  0  0  0  0  0  0  0  0  0  0
   -3.0159    0.4501    1.5205 H   0  0  0  0  0  0  0  0  0  0  0  0
   -0.0248    1.7655   -0.6685 H   0  0  0  0  0  0  0  0  0  0  0  0
   -0.0897    1.0640    1.9583 H   0  0  0  0  0  0  0  0  0  0  0  0
   -0.1984   -1.9689    1.3031 C   0  0  0  0  0  0  0  0  0  0  0  0
    0.2878   -1.6244    0.0730 C   0  0  0  0  0  0  0  0  0  0  0  0
   -0.2480   -3.0423    1.5920 H   0  0  0  0  0  0  0  0  0  0  0  0
    0.5536   -2.4132   -0.6610 H   0  0  0  0  0  0  0  0  0  0  0  0
    1.2618    0.2228    0.5626 H   0  0  0  0  0  0  0  0  0  0  0  0
    0.6477   -0.9427    2.9673 H   0  0  0  0  0  0  0  0  0  0  0  0
   -4.0818   -1.9976   -0.6316 N   0  0  0  0  0  0  0  0  0  0  0  0
   -5.1718   -2.0789   -1.0480 C   0  0  0  0  0  0  0  0  0  0  0  0
   -6.4863   -2.2280   -1.5659 C   0  0  0  0  0  0  0  0  0  0  0  0
   -6.9828   -1.3144   -2.5172 C   0  0  0  0  0  0  0  0  0  0  0  0
   -7.2734   -3.3179   -1.1344 C   0  0  0  0  0  0  0  0  0  0  0  0
   -8.2633   -1.5029   -3.0383 C   0  0  0  0  0  0  0  0  0  0  0  0
   -8.5484   -3.4909   -1.6682 C   0  0  0  0  0  0  0  0  0  0  0  0
   -9.0434   -2.5871   -2.6187 C   0  0  0  0  0  0  0  0  0  0  0  0
   -0.8299   -5.4121   -1.2857 C   0  0  0  0  0  0  0  0  0  0  0  0
    0.1512   -5.6593   -0.3204 C   0  0  0  0  0  0  0  0  0  0  0  0
   -0.6823   -5.8101   -2.6106 C   0  0  0  0  0  0  0  0  0  0  0  0
    1.3151   -6.3238   -0.7349 C   0  0  0  0  0  0  0  0  0  0  0  0
    0.0410   -5.3951    0.7333 H   0  0  0  0  0  0  0  0  0  0  0  0
    0.5093   -6.4725   -2.9634 C   0  0  0  0  0  0  0  0  0  0  0  0
   -1.4441   -5.6497   -3.3801 H   0  0  0  0  0  0  0  0  0  0  0  0
    1.5305   -6.7434   -2.0523 C   0  0  0  0  0  0  0  0  0  0  0  0
   -3.0216   -5.5667    0.7088 C   0  0  0  0  0  0  0  0  0  0  0  0
   -2.8244   -6.9440    0.7261 C   0  0  0  0  0  0  0  0  0  0  0  0
   -3.7185   -4.8957    1.7167 C   0  0  0  0  0  0  0  0  0  0  0  0
   -3.3654   -7.6631    1.8088 C   0  0  0  0  0  0  0  0  0  0  0  0
   -2.2714   -7.4818   -0.0520 H   0  0  0  0  0  0  0  0  0  0  0  0
   -4.2436   -5.6706    2.7629 C   0  0  0  0  0  0  0  0  0  0  0  0
   -3.8652   -3.8064    1.7273 H   0  0  0  0  0  0  0  0  0  0  0  0
   -4.0868   -7.0586    2.8400 C   0  0  0  0  0  0  0  0  0  0  0  0
   -3.6538   -4.7977   -2.0880 C   0  0  0  0  0  0  0  0  0  0  0  0
   -4.6947   -5.7036   -1.9088 C   0  0  0  0  0  0  0  0  0  0  0  0
   -3.5258   -4.0338   -3.2522 C   0  0  0  0  0  0  0  0  0  0  0  0
   -5.6343   -5.8319   -2.9489 C   0  0  0  0  0  0  0  0  0  0  0  0
```

```
   -4.8107   -6.3189   -1.0104 H   0  0  0  0  0  0  0  0  0  0  0  0
   -4.4897   -4.2130   -4.2554 C   0  0  0  0  0  0  0  0  0  0  0  0
   -2.7133   -3.3196   -3.4185 H   0  0  0  0  0  0  0  0  0  0  0  0
   -5.5637   -5.1022   -4.1355 C   0  0  0  0  0  0  0  0  0  0  0  0
   -2.2463   -2.0239   -0.0363 Ir  0  0  0  0  0  0  0  0  0  0  0  0
  -10.0476   -2.7289   -3.0332 H   0  0  0  0  0  0  0  0  0  0  0  0
   -8.6598   -0.7986   -3.7766 H   0  0  0  0  0  0  0  0  0  0  0  0
   -9.1680   -4.3316   -1.3417 H   0  0  0  0  0  0  0  0  0  0  0  0
   -6.3732   -0.4694   -2.8433 H   0  0  0  0  0  0  0  0  0  0  0  0
   -6.8857   -4.0154   -0.3903 H   0  0  0  0  0  0  0  0  0  0  0  0
   -1.3688    0.7484    3.1830 H   0  0  0  0  0  0  0  0  0  0  0  0
   -1.0353   -1.5533    3.2479 H   0  0  0  0  0  0  0  0  0  0  0  0
   -0.4790    0.5554   -1.9356 H   0  0  0  0  0  0  0  0  0  0  0  0
    1.5124   -0.3321   -1.1329 H   0  0  0  0  0  0  0  0  0  0  0  0
   -6.3032   -5.2235   -4.9355 H   0  0  0  0  0  0  0  0  0  0  0  0
   -4.5062   -7.6410    3.6705 H   0  0  0  0  0  0  0  0  0  0  0  0
    2.4491   -7.2650   -2.3493 H   0  0  0  0  0  0  0  0  0  0  0  0
    0.6617   -6.8570   -4.2245 F   0  0  0  0  0  0  0  0  0  0  0  0
    2.2566   -6.5642    0.1696 F   0  0  0  0  0  0  0  0  0  0  0  0
   -3.1851   -8.9770    1.8421 F   0  0  0  0  0  0  0  0  0  0  0  0
   -4.9245   -5.0541    3.7217 F   0  0  0  0  0  0  0  0  0  0  0  0
   -6.6430   -6.6788   -2.7805 F   0  0  0  0  0  0  0  0  0  0  0  0
   -4.3844   -3.4920   -5.3653 F   0  0  0  0  0  0  0  0  0  0  0  0
  1 26  1  0  0  0  0
  1 34  1  0  0  0  0
  1 42  1  0  0  0  0
  1 50  1  0  0  0  0
  2  3  1  0  0  0  0
  2  4  1  0  0  0  0
  2 10  1  0  0  0  0
  2 58  1  0  0  0  0
  3 13  1  0  0  0  0
  3 16  1  0  0  0  0
  3 59  1  0  0  0  0
  4  6  2  0  0  0  0
  4  8  1  0  0  0  0
  4 50  1  0  0  0  0
  5  6  1  0  0  0  0
  5  7  1  0  0  0  0
  5 11  1  0  0  0  0
  5 56  1  0  0  0  0
  6  9  1  0  0  0  0
  6 50  1  0  0  0  0
  7 12  1  0  0  0  0
  7 17  1  0  0  0  0
  7 57  1  0  0  0  0
 12 13  2  0  0  0  0
 12 14  1  0  0  0  0
 12 50  1  0  0  0  0
 13 15  1  0  0  0  0
 13 50  1  0  0  0  0
 18 19  3  0  0  0  0
 18 50  1  0  0  0  0
 19 20  1  0  0  0  0
 20 21  2  0  0  0  0
 20 22  1  0  0  0  0
 21 23  1  0  0  0  0
 21 54  1  0  0  0  0
 22 24  2  0  0  0  0
 22 55  1  0  0  0  0
 23 25  2  0  0  0  0
 23 52  1  0  0  0  0
 24 25  1  0  0  0  0
 24 53  1  0  0  0  0
 25 51  1  0  0  0  0
```

```
 26 27  2  0  0  0  0
 26 28  1  0  0  0  0
 27 29  1  0  0  0  0
 27 30  1  0  0  0  0
 28 31  2  0  0  0  0
 28 32  1  0  0  0  0
 29 33  2  0  0  0  0
 29 64  1  0  0  0  0
 31 33  1  0  0  0  0
 31 63  1  0  0  0  0
 33 62  1  0  0  0  0
 34 35  2  0  0  0  0
 34 36  1  0  0  0  0
 35 37  1  0  0  0  0
 35 38  1  0  0  0  0
 36 39  2  0  0  0  0
 36 40  1  0  0  0  0
 37 41  2  0  0  0  0
 37 65  1  0  0  0  0
 39 41  1  0  0  0  0
 39 66  1  0  0  0  0
 41 61  1  0  0  0  0
 42 43  2  0  0  0  0
 42 44  1  0  0  0  0
 43 45  1  0  0  0  0
 43 46  1  0  0  0  0
 44 47  2  0  0  0  0
 44 48  1  0  0  0  0
 45 49  2  0  0  0  0
 45 67  1  0  0  0  0
 47 49  1  0  0  0  0
 47 68  1  0  0  0  0
 49 60  1  0  0  0  0
M  END
$$$$
mol071
Optimized Catalyst 071
Optimized Catalyst 071
 86 93  0  0  0  0  0  0  0  0999 V2000
   -2.4335   -4.6409   -0.7344 P   0  0  0  0  0  0  0  0  0  0  0  0
   -0.4220    0.5927   -1.0010 C   0  0  0  0  0  0  0  0  0  0  0  0
    0.6847   -0.3515   -0.4909 C   0  0  0  0  0  0  0  0  0  0  0  0
   -1.7503    0.5364   -0.2933 C   0  0  0  0  0  0  0  0  0  0  0  0
   -0.8872    0.3534    2.0837 C   0  0  0  0  0  0  0  0  0  0  0  0
   -1.9638    0.4316    1.0373 C   0  0  0  0  0  0  0  0  0  0  0  0
   -0.3963   -1.0799    2.3674 C   0  0  0  0  0  0  0  0  0  0  0  0
   -2.6101    0.7183   -0.9673 H   0  0  0  0  0  0  0  0  0  0  0  0
   -2.9901    0.4953    1.4400 H   0  0  0  0  0  0  0  0  0  0  0  0
   -0.0464    1.6527   -0.9124 H   0  0  0  0  0  0  0  0  0  0  0  0
   -0.0286    1.0067    1.7792 H   0  0  0  0  0  0  0  0  0  0  0  0
   -0.2581   -2.0184    1.1905 C   0  0  0  0  0  0  0  0  0  0  0  0
    0.2185   -1.7126   -0.0503 C   0  0  0  0  0  0  0  0  0  0  0  0
   -0.3354   -3.0861    1.4963 H   0  0  0  0  0  0  0  0  0  0  0  0
    0.4430   -2.5246   -0.7730 H   0  0  0  0  0  0  0  0  0  0  0  0
    1.2328    0.1297    0.3638 H   0  0  0  0  0  0  0  0  0  0  0  0
    0.6233   -1.0112    2.8464 H   0  0  0  0  0  0  0  0  0  0  0  0
   -4.2398   -2.1162   -0.6450 N   0  0  0  0  0  0  0  0  0  0  0  0
   -5.3507   -2.1310   -1.0054 C   0  0  0  0  0  0  0  0  0  0  0  0
   -6.7017   -2.1639   -1.4493 C   0  0  0  0  0  0  0  0  0  0  0  0
   -7.2158   -3.3549   -2.0016 C   0  0  0  0  0  0  0  0  0  0  0  0
   -7.5099   -1.0145   -1.3362 C   0  0  0  0  0  0  0  0  0  0  0  0
   -8.5392   -3.3865   -2.4397 C   0  0  0  0  0  0  0  0  0  0  0  0
   -8.8314   -1.0652   -1.7796 C   0  0  0  0  0  0  0  0  0  0  0  0
   -9.3456   -2.2464   -2.3295 C   0  0  0  0  0  0  0  0  0  0  0  0
   -0.7718   -5.3290   -1.1963 C   0  0  0  0  0  0  0  0  0  0  0  0
```

```
    0.1096   -5.6676   -0.1657 C   0  0  0  0  0  0  0  0  0  0  0  0
   -0.4511   -5.5245   -2.5348 C   0  0  0  0  0  0  0  0  0  0  0  0
    1.3620   -6.2161   -0.4914 C   0  0  0  0  0  0  0  0  0  0  0  0
   -0.1512   -5.5559    0.8873 H   0  0  0  0  0  0  0  0  0  0  0  0
    0.8059   -6.0773   -2.8652 C   0  0  0  0  0  0  0  0  0  0  0  0
   -1.1412   -5.2862   -3.3469 H   0  0  0  0  0  0  0  0  0  0  0  0
    1.6992   -6.4150   -1.8419 C   0  0  0  0  0  0  0  0  0  0  0  0
   -3.0404   -5.6334    0.7151 C   0  0  0  0  0  0  0  0  0  0  0  0
   -2.8080   -7.0045    0.7484 C   0  0  0  0  0  0  0  0  0  0  0  0
   -3.7296   -4.9667    1.7291 C   0  0  0  0  0  0  0  0  0  0  0  0
   -3.3038   -7.7526    1.8371 C   0  0  0  0  0  0  0  0  0  0  0  0
   -2.2448   -7.5205   -0.0328 H   0  0  0  0  0  0  0  0  0  0  0  0
   -4.2236   -5.7095    2.8170 C   0  0  0  0  0  0  0  0  0  0  0  0
   -3.8971   -3.8807    1.7091 H   0  0  0  0  0  0  0  0  0  0  0  0
   -4.0081   -7.0978    2.8557 C   0  0  0  0  0  0  0  0  0  0  0  0
   -3.5862   -4.9646   -2.1610 C   0  0  0  0  0  0  0  0  0  0  0  0
   -4.4336   -6.0634   -2.1242 C   0  0  0  0  0  0  0  0  0  0  0  0
   -3.5393   -4.1005   -3.2601 C   0  0  0  0  0  0  0  0  0  0  0  0
   -5.2582   -6.3329   -3.2409 C   0  0  0  0  0  0  0  0  0  0  0  0
   -4.4881   -6.7377   -1.2657 H   0  0  0  0  0  0  0  0  0  0  0  0
   -4.3579   -4.3636   -4.3704 C   0  0  0  0  0  0  0  0  0  0  0  0
   -2.8780   -3.2310   -3.2915 H   0  0  0  0  0  0  0  0  0  0  0  0
   -5.2109   -5.4834   -4.3500 C   0  0  0  0  0  0  0  0  0  0  0  0
   -2.3915   -2.1230   -0.0796 Ir  0  0  0  0  0  0  0  0  0  0  0  0
  -10.3840   -2.2780   -2.6745 H   0  0  0  0  0  0  0  0  0  0  0  0
   -8.9486   -4.3044   -2.8692 H   0  0  0  0  0  0  0  0  0  0  0  0
   -9.4680   -0.1792   -1.6970 H   0  0  0  0  0  0  0  0  0  0  0  0
   -6.5812   -4.2407   -2.0856 H   0  0  0  0  0  0  0  0  0  0  0  0
   -7.1064   -0.0959   -0.9063 H   0  0  0  0  0  0  0  0  0  0  0  0
   -1.2848    0.7790    3.0457 H   0  0  0  0  0  0  0  0  0  0  0  0
   -1.0871   -1.5457    3.1226 H   0  0  0  0  0  0  0  0  0  0  0  0
   -0.5823    0.3902   -2.0941 H   0  0  0  0  0  0  0  0  0  0  0  0
    1.4337   -0.4788   -1.3207 H   0  0  0  0  0  0  0  0  0  0  0  0
   -5.8354   -5.6913   -5.2213 H   0  0  0  0  0  0  0  0  0  0  0  0
   -4.3935   -7.6749    3.6995 H   0  0  0  0  0  0  0  0  0  0  0  0
    2.6695   -6.8493   -2.0918 H   0  0  0  0  0  0  0  0  0  0  0  0
   -4.9793   -5.0466    3.9208 C   0  0  0  0  0  0  0  0  0  0  0  0
   -6.0690   -5.2830    3.8212 H   0  0  0  0  0  0  0  0  0  0  0  0
   -4.6302   -5.4248    4.9143 H   0  0  0  0  0  0  0  0  0  0  0  0
   -4.8560   -3.9400    3.9104 H   0  0  0  0  0  0  0  0  0  0  0  0
   -3.0692   -9.2245    1.9132 C   0  0  0  0  0  0  0  0  0  0  0  0
   -2.2175   -9.4290    2.6109 H   0  0  0  0  0  0  0  0  0  0  0  0
   -3.9748   -9.7502    2.3046 H   0  0  0  0  0  0  0  0  0  0  0  0
   -2.8200   -9.6594    0.9175 H   0  0  0  0  0  0  0  0  0  0  0  0
   -6.1632   -7.5196   -3.2160 C   0  0  0  0  0  0  0  0  0  0  0  0
   -5.5592   -8.4555   -3.1062 H   0  0  0  0  0  0  0  0  0  0  0  0
   -6.8721   -7.4513   -2.3557 H   0  0  0  0  0  0  0  0  0  0  0  0
   -6.7603   -7.6074   -4.1551 H   0  0  0  0  0  0  0  0  0  0  0  0
   -4.3283   -3.4787   -5.5720 C   0  0  0  0  0  0  0  0  0  0  0  0
   -3.8233   -4.0146   -6.4163 H   0  0  0  0  0  0  0  0  0  0  0  0
   -5.3671   -3.2208   -5.8948 H   0  0  0  0  0  0  0  0  0  0  0  0
   -3.7773   -2.5288   -5.3834 H   0  0  0  0  0  0  0  0  0  0  0  0
    2.3189   -6.6054    0.5867 C   0  0  0  0  0  0  0  0  0  0  0  0
    2.1604   -7.6825    0.8493 H   0  0  0  0  0  0  0  0  0  0  0  0
    3.3772   -6.4820    0.2502 H   0  0  0  0  0  0  0  0  0  0  0  0
    2.1758   -5.9933    1.5072 H   0  0  0  0  0  0  0  0  0  0  0  0
    1.1557   -6.3047   -4.2988 C   0  0  0  0  0  0  0  0  0  0  0  0
    0.5170   -7.1228   -4.7165 H   0  0  0  0  0  0  0  0  0  0  0  0
    0.9857   -5.3773   -4.8969 H   0  0  0  0  0  0  0  0  0  0  0  0
    2.2244   -6.6037   -4.4226 H   0  0  0  0  0  0  0  0  0  0  0  0
  1 26  1  0  0  0  0
  1 34  1  0  0  0  0
  1 42  1  0  0  0  0
  1 50  1  0  0  0  0
  2  3  1  0  0  0  0
```

```
  2  4  1  0  0  0  0
  2 10  1  0  0  0  0
  2 58  1  0  0  0  0
  3 13  1  0  0  0  0
  3 16  1  0  0  0  0
  3 59  1  0  0  0  0
  4  6  2  0  0  0  0
  4  8  1  0  0  0  0
  4 50  1  0  0  0  0
  5  6  1  0  0  0  0
  5  7  1  0  0  0  0
  5 11  1  0  0  0  0
  5 56  1  0  0  0  0
  6  9  1  0  0  0  0
  6 50  1  0  0  0  0
  7 12  1  0  0  0  0
  7 17  1  0  0  0  0
  7 57  1  0  0  0  0
 12 13  2  0  0  0  0
 12 14  1  0  0  0  0
 12 50  1  0  0  0  0
 13 15  1  0  0  0  0
 13 50  1  0  0  0  0
 18 19  3  0  0  0  0
 18 50  1  0  0  0  0
 19 20  1  0  0  0  0
 20 21  2  0  0  0  0
 20 22  1  0  0  0  0
 21 23  1  0  0  0  0
 21 54  1  0  0  0  0
 22 24  2  0  0  0  0
 22 55  1  0  0  0  0
 23 25  2  0  0  0  0
 23 52  1  0  0  0  0
 24 25  1  0  0  0  0
 24 53  1  0  0  0  0
 25 51  1  0  0  0  0
 26 27  2  0  0  0  0
 26 28  1  0  0  0  0
 27 29  1  0  0  0  0
 27 30  1  0  0  0  0
 28 31  2  0  0  0  0
 28 32  1  0  0  0  0
 29 33  2  0  0  0  0
 29 79  1  0  0  0  0
 31 33  1  0  0  0  0
 31 83  1  0  0  0  0
 33 62  1  0  0  0  0
 34 35  2  0  0  0  0
 34 36  1  0  0  0  0
 35 37  1  0  0  0  0
 35 38  1  0  0  0  0
 36 39  2  0  0  0  0
 36 40  1  0  0  0  0
 37 41  2  0  0  0  0
 37 67  1  0  0  0  0
 39 41  1  0  0  0  0
 39 63  1  0  0  0  0
 41 61  1  0  0  0  0
 42 43  2  0  0  0  0
 42 44  1  0  0  0  0
 43 45  1  0  0  0  0
 43 46  1  0  0  0  0
 44 47  2  0  0  0  0
 44 48  1  0  0  0  0
```

```
 45 49  2  0  0  0  0
 45 71  1  0  0  0  0
 47 49  1  0  0  0  0
 47 75  1  0  0  0  0
 49 60  1  0  0  0  0
 63 64  1  0  0  0  0
 63 65  1  0  0  0  0
 63 66  1  0  0  0  0
 67 68  1  0  0  0  0
 67 69  1  0  0  0  0
 67 70  1  0  0  0  0
 71 72  1  0  0  0  0
 71 73  1  0  0  0  0
 71 74  1  0  0  0  0
 75 76  1  0  0  0  0
 75 77  1  0  0  0  0
 75 78  1  0  0  0  0
 79 80  1  0  0  0  0
 79 81  1  0  0  0  0
 79 82  1  0  0  0  0
 83 84  1  0  0  0  0
 83 85  1  0  0  0  0
 83 86  1  0  0  0  0
M  END
$$$$
mol072
Optimized Catalyst 072
Optimized Catalyst 072
 68 75  0  0  0  0  0  0  0  0999 V2000
   -2.4238   -4.6664   -0.6614 P   0  0  0  0  0  0  0  0  0  0  0  0
   -0.4041    0.4898   -1.1596 C   0  0  0  0  0  0  0  0  0  0  0  0
    0.6842   -0.3046   -0.4101 C   0  0  0  0  0  0  0  0  0  0  0  0
   -1.7913    0.4602   -0.5731 C   0  0  0  0  0  0  0  0  0  0  0  0
   -1.1388    0.7545    1.8579 C   0  0  0  0  0  0  0  0  0  0  0  0
   -2.1219    0.5602    0.7363 C   0  0  0  0  0  0  0  0  0  0  0  0
   -0.5812   -0.5611    2.4351 C   0  0  0  0  0  0  0  0  0  0  0  0
   -2.5910    0.4706   -1.3407 H   0  0  0  0  0  0  0  0  0  0  0  0
   -3.1831    0.6177    1.0401 H   0  0  0  0  0  0  0  0  0  0  0  0
   -0.0850    1.5710   -1.2022 H   0  0  0  0  0  0  0  0  0  0  0  0
   -0.3005    1.4094    1.5023 H   0  0  0  0  0  0  0  0  0  0  0  0
   -0.3279   -1.6921    1.4661 C   0  0  0  0  0  0  0  0  0  0  0  0
    0.2186   -1.5908    0.2215 C   0  0  0  0  0  0  0  0  0  0  0  0
   -0.4235   -2.6872    1.9557 H   0  0  0  0  0  0  0  0  0  0  0  0
    0.5146   -2.4999   -0.3380 H   0  0  0  0  0  0  0  0  0  0  0  0
    1.1448    0.3375    0.3887 H   0  0  0  0  0  0  0  0  0  0  0  0
    0.4059   -0.3362    2.9349 H   0  0  0  0  0  0  0  0  0  0  0  0
   -4.1850   -2.1286   -0.5179 N   0  0  0  0  0  0  0  0  0  0  0  0
   -5.2952   -2.1743   -0.8785 C   0  0  0  0  0  0  0  0  0  0  0  0
   -6.6412   -2.2464   -1.3332 C   0  0  0  0  0  0  0  0  0  0  0  0
   -7.4047   -3.3982   -1.0516 C   0  0  0  0  0  0  0  0  0  0  0  0
   -7.1972   -1.1755   -2.0614 C   0  0  0  0  0  0  0  0  0  0  0  0
   -8.7193   -3.4731   -1.5095 C   0  0  0  0  0  0  0  0  0  0  0  0
   -8.5157   -1.2670   -2.5079 C   0  0  0  0  0  0  0  0  0  0  0  0
   -9.2756   -2.4109   -2.2341 C   0  0  0  0  0  0  0  0  0  0  0  0
   -0.8402   -5.3782   -1.2810 C   0  0  0  0  0  0  0  0  0  0  0  0
    0.1175   -5.4406   -0.2595 C   0  0  0  0  0  0  0  0  0  0  0  0
   -0.4822   -5.9454   -2.5025 C   0  0  0  0  0  0  0  0  0  0  0  0
    1.3845   -5.9787   -0.3961 C   0  0  0  0  0  0  0  0  0  0  0  0
    0.7938   -6.4981   -2.7070 C   0  0  0  0  0  0  0  0  0  0  0  0
    1.7140   -6.5113   -1.6587 C   0  0  0  0  0  0  0  0  0  0  0  0
    2.0975   -6.0105    0.4307 H   0  0  0  0  0  0  0  0  0  0  0  0
    1.0579   -6.9280   -3.6794 H   0  0  0  0  0  0  0  0  0  0  0  0
   -2.9805   -5.8620    0.6332 C   0  0  0  0  0  0  0  0  0  0  0  0
   -2.8035   -7.2471    0.4986 C   0  0  0  0  0  0  0  0  0  0  0  0
   -3.6120   -5.4400    1.8091 C   0  0  0  0  0  0  0  0  0  0  0  0
```

```
  -3.2064   -8.1625    1.4748 C   0  0  0  0  0  0  0  0  0  0  0  0
  -4.0339   -6.3067    2.8141 C   0  0  0  0  0  0  0  0  0  0  0  0
  -3.8201   -7.6812    2.6355 C   0  0  0  0  0  0  0  0  0  0  0  0
  -3.0482   -9.2367    1.3350 H   0  0  0  0  0  0  0  0  0  0  0  0
  -4.5172   -5.9325    3.7213 H   0  0  0  0  0  0  0  0  0  0  0  0
  -3.6494   -4.8116   -2.0373 C   0  0  0  0  0  0  0  0  0  0  0  0
  -4.7589   -5.6512   -2.1419 C   0  0  0  0  0  0  0  0  0  0  0  0
  -3.3727   -4.0080   -3.1536 C   0  0  0  0  0  0  0  0  0  0  0  0
  -5.5571   -5.6895   -3.2942 C   0  0  0  0  0  0  0  0  0  0  0  0
  -4.1223   -4.0076   -4.3212 C   0  0  0  0  0  0  0  0  0  0  0  0
  -5.2262   -4.8763   -4.3803 C   0  0  0  0  0  0  0  0  0  0  0  0
  -6.4189   -6.3622   -3.3493 H   0  0  0  0  0  0  0  0  0  0  0  0
  -3.8622   -3.3732   -5.1724 H   0  0  0  0  0  0  0  0  0  0  0  0
  -2.3482   -2.1126    0.0495 Ir  0  0  0  0  0  0  0  0  0  0  0  0
 -10.3111   -2.4734   -2.5846 H   0  0  0  0  0  0  0  0  0  0  0  0
  -9.3212   -4.3610   -1.2969 H   0  0  0  0  0  0  0  0  0  0  0  0
  -8.9587   -0.4399   -3.0705 H   0  0  0  0  0  0  0  0  0  0  0  0
  -6.9720   -4.2180   -0.4760 H   0  0  0  0  0  0  0  0  0  0  0  0
  -6.6046   -0.2832   -2.2707 H   0  0  0  0  0  0  0  0  0  0  0  0
  -1.6462    1.3123    2.6925 H   0  0  0  0  0  0  0  0  0  0  0  0
  -1.2829   -0.9198    3.2377 H   0  0  0  0  0  0  0  0  0  0  0  0
  -0.4475    0.1164   -2.2184 H   0  0  0  0  0  0  0  0  0  0  0  0
   1.5037   -0.5398   -1.1438 H   0  0  0  0  0  0  0  0  0  0  0  0
  -4.1398   -8.3844    3.4137 H   0  0  0  0  0  0  0  0  0  0  0  0
   2.7080   -6.9491   -1.8123 H   0  0  0  0  0  0  0  0  0  0  0  0
  -5.8344   -4.9116   -5.2919 H   0  0  0  0  0  0  0  0  0  0  0  0
  -2.2423   -7.7199   -0.6083 F   0  0  0  0  0  0  0  0  0  0  0  0
  -3.8188   -4.1305    1.9648 F   0  0  0  0  0  0  0  0  0  0  0  0
  -0.3291   -4.9185    0.8867 F   0  0  0  0  0  0  0  0  0  0  0  0
  -1.3456   -5.9864   -3.5070 F   0  0  0  0  0  0  0  0  0  0  0  0
  -5.1162   -6.4185   -1.1197 F   0  0  0  0  0  0  0  0  0  0  0  0
  -2.3207   -3.1997   -3.0039 F   0  0  0  0  0  0  0  0  0  0  0  0
 1 26  1  0  0  0  0
 1 34  1  0  0  0  0
 1 42  1  0  0  0  0
 1 50  1  0  0  0  0
 2  3  1  0  0  0  0
 2  4  1  0  0  0  0
 2 10  1  0  0  0  0
 2 58  1  0  0  0  0
 3 13  1  0  0  0  0
 3 16  1  0  0  0  0
 3 59  1  0  0  0  0
 4  6  2  0  0  0  0
 4  8  1  0  0  0  0
 4 50  1  0  0  0  0
 5  6  1  0  0  0  0
 5  7  1  0  0  0  0
 5 11  1  0  0  0  0
 5 56  1  0  0  0  0
 6  9  1  0  0  0  0
 6 50  1  0  0  0  0
 7 12  1  0  0  0  0
 7 17  1  0  0  0  0
 7 57  1  0  0  0  0
12 13  2  0  0  0  0
12 14  1  0  0  0  0
12 50  1  0  0  0  0
13 15  1  0  0  0  0
13 50  1  0  0  0  0
18 19  3  0  0  0  0
18 50  1  0  0  0  0
19 20  1  0  0  0  0
20 21  2  0  0  0  0
20 22  1  0  0  0  0
```

```
 21 23  1  0  0  0  0
 21 54  1  0  0  0  0
 22 24  2  0  0  0  0
 22 55  1  0  0  0  0
 23 25  2  0  0  0  0
 23 52  1  0  0  0  0
 24 25  1  0  0  0  0
 24 53  1  0  0  0  0
 25 51  1  0  0  0  0
 26 27  1  0  0  0  0
 26 28  2  0  0  0  0
 27 29  2  0  0  0  0
 27 65  1  0  0  0  0
 28 30  1  0  0  0  0
 28 66  1  0  0  0  0
 29 31  1  0  0  0  0
 29 32  1  0  0  0  0
 30 31  2  0  0  0  0
 30 33  1  0  0  0  0
 31 61  1  0  0  0  0
 34 35  2  0  0  0  0
 34 36  1  0  0  0  0
 35 37  1  0  0  0  0
 35 63  1  0  0  0  0
 36 38  2  0  0  0  0
 36 64  1  0  0  0  0
 37 39  2  0  0  0  0
 37 40  1  0  0  0  0
 38 39  1  0  0  0  0
 38 41  1  0  0  0  0
 39 60  1  0  0  0  0
 42 43  2  0  0  0  0
 42 44  1  0  0  0  0
 43 45  1  0  0  0  0
 43 67  1  0  0  0  0
 44 46  2  0  0  0  0
 44 68  1  0  0  0  0
 45 47  2  0  0  0  0
 45 48  1  0  0  0  0
 46 47  1  0  0  0  0
 46 49  1  0  0  0  0
 47 62  1  0  0  0  0
M  END
$$$$
mol073
Optimized Catalyst 073
Optimized Catalyst 073
 86 93  0  0  0  0  0  0  0  0999 V2000
   -2.5836   -4.9340   -0.4905 P   0  0  0  0  0  0  0  0  0  0  0  0
   -0.5589    0.4403   -1.6810 C   0  0  0  0  0  0  0  0  0  0  0  0
    0.6730   -0.2622   -1.0755 C   0  0  0  0  0  0  0  0  0  0  0  0
   -1.8438    0.3663   -0.9054 C   0  0  0  0  0  0  0  0  0  0  0  0
   -0.8976    0.6762    1.4200 C   0  0  0  0  0  0  0  0  0  0  0  0
   -2.0039    0.4516    0.4281 C   0  0  0  0  0  0  0  0  0  0  0  0
   -0.2318   -0.6295    1.9001 C   0  0  0  0  0  0  0  0  0  0  0  0
   -2.7335    0.3158   -1.5531 H   0  0  0  0  0  0  0  0  0  0  0  0
   -3.0175    0.4442    0.8654 H   0  0  0  0  0  0  0  0  0  0  0  0
   -0.3104    1.5330   -1.8025 H   0  0  0  0  0  0  0  0  0  0  0  0
   -0.1318    1.3584    0.9693 H   0  0  0  0  0  0  0  0  0  0  0  0
   -0.0564   -1.7209    0.8690 C   0  0  0  0  0  0  0  0  0  0  0  0
    0.3652   -1.5675   -0.4074 C   0  0  0  0  0  0  0  0  0  0  0  0
   -0.0651   -2.7396    1.3121 H   0  0  0  0  0  0  0  0  0  0  0  0
    0.5623   -2.4574   -1.0287 H   0  0  0  0  0  0  0  0  0  0  0  0
    1.1753    0.4167   -0.3365 H   0  0  0  0  0  0  0  0  0  0  0  0
    0.7944   -0.3765    2.2907 H   0  0  0  0  0  0  0  0  0  0  0  0
```

```
   -4.3658   -2.3202   -0.1713 N   0  0  0  0  0  0  0  0  0  0  0  0
   -5.4441   -2.1723   -0.5962 C   0  0  0  0  0  0  0  0  0  0  0  0
   -6.7541   -1.9976   -1.1216 C   0  0  0  0  0  0  0  0  0  0  0  0
   -7.3497   -3.0420   -1.8579 C   0  0  0  0  0  0  0  0  0  0  0  0
   -7.4392   -0.7834   -0.9131 C   0  0  0  0  0  0  0  0  0  0  0  0
   -8.6277   -2.8628   -2.3850 C   0  0  0  0  0  0  0  0  0  0  0  0
   -8.7171   -0.6216   -1.4488 C   0  0  0  0  0  0  0  0  0  0  0  0
   -9.3104   -1.6561   -2.1830 C   0  0  0  0  0  0  0  0  0  0  0  0
   -0.8112   -5.3310   -0.8938 C   0  0  0  0  0  0  0  0  0  0  0  0
    0.0875   -5.2827    0.2068 C   0  0  0  0  0  0  0  0  0  0  0  0
   -0.3234   -5.6714   -2.1707 C   0  0  0  0  0  0  0  0  0  0  0  0
    1.4657   -5.3384   -0.0281 C   0  0  0  0  0  0  0  0  0  0  0  0
    1.0741   -5.7018   -2.3767 C   0  0  0  0  0  0  0  0  0  0  0  0
    1.9609   -5.5107   -1.3255 C   0  0  0  0  0  0  0  0  0  0  0  0
    2.1726   -5.2718    0.8016 H   0  0  0  0  0  0  0  0  0  0  0  0
    1.4671   -5.9044   -3.3763 H   0  0  0  0  0  0  0  0  0  0  0  0
   -3.1222   -6.2236    0.7393 C   0  0  0  0  0  0  0  0  0  0  0  0
   -2.7202   -7.5663    0.5514 C   0  0  0  0  0  0  0  0  0  0  0  0
   -3.8630   -5.8696    1.8900 C   0  0  0  0  0  0  0  0  0  0  0  0
   -3.0073   -8.5154    1.5512 C   0  0  0  0  0  0  0  0  0  0  0  0
   -4.1438   -6.8436    2.8592 C   0  0  0  0  0  0  0  0  0  0  0  0
   -3.7059   -8.1593    2.6997 C   0  0  0  0  0  0  0  0  0  0  0  0
   -2.6809   -9.5509    1.4300 H   0  0  0  0  0  0  0  0  0  0  0  0
   -4.7112   -6.5851    3.7557 H   0  0  0  0  0  0  0  0  0  0  0  0
   -3.6720   -4.8759   -1.9946 C   0  0  0  0  0  0  0  0  0  0  0  0
   -4.7891   -5.7220   -2.1652 C   0  0  0  0  0  0  0  0  0  0  0  0
   -3.3309   -3.9520   -3.0191 C   0  0  0  0  0  0  0  0  0  0  0  0
   -5.4668   -5.7186   -3.4026 C   0  0  0  0  0  0  0  0  0  0  0  0
   -4.0437   -3.9596   -4.2250 C   0  0  0  0  0  0  0  0  0  0  0  0
   -5.0905   -4.8613   -4.4296 C   0  0  0  0  0  0  0  0  0  0  0  0
   -6.2991   -6.4074   -3.5669 H   0  0  0  0  0  0  0  0  0  0  0  0
   -3.7844   -3.2649   -5.0267 H   0  0  0  0  0  0  0  0  0  0  0  0
   -2.5172   -2.4553    0.3860 Ir  0  0  0  0  0  0  0  0  0  0  0  0
  -10.3134   -1.5212   -2.6006 H   0  0  0  0  0  0  0  0  0  0  0  0
   -9.0995   -3.6659   -2.9570 H   0  0  0  0  0  0  0  0  0  0  0  0
   -9.2579    0.3170   -1.2941 H   0  0  0  0  0  0  0  0  0  0  0  0
   -6.8115   -3.9789   -2.0150 H   0  0  0  0  0  0  0  0  0  0  0  0
   -6.9751    0.0209   -0.3393 H   0  0  0  0  0  0  0  0  0  0  0  0
   -1.3108    1.2108    2.3171 H   0  0  0  0  0  0  0  0  0  0  0  0
   -0.8271   -1.0336    2.7646 H   0  0  0  0  0  0  0  0  0  0  0  0
   -0.7314    0.0171   -2.7041 H   0  0  0  0  0  0  0  0  0  0  0  0
    1.4105   -0.4393   -1.9040 H   0  0  0  0  0  0  0  0  0  0  0  0
   -3.9181   -8.9062    3.4709 H   0  0  0  0  0  0  0  0  0  0  0  0
    3.0408   -5.5249   -1.5010 H   0  0  0  0  0  0  0  0  0  0  0  0
   -5.6180   -4.8802   -5.3881 H   0  0  0  0  0  0  0  0  0  0  0  0
   -2.0093   -8.0977   -0.6541 C   0  0  0  0  0  0  0  0  0  0  0  0
   -0.9254   -7.8151   -0.6200 H   0  0  0  0  0  0  0  0  0  0  0  0
   -2.0502   -9.2170   -0.6816 H   0  0  0  0  0  0  0  0  0  0  0  0
   -2.4605   -7.7427   -1.6065 H   0  0  0  0  0  0  0  0  0  0  0  0
   -4.4269   -4.5021    2.1579 C   0  0  0  0  0  0  0  0  0  0  0  0
   -3.6379   -3.6878    2.1315 H   0  0  0  0  0  0  0  0  0  0  0  0
   -5.2656   -4.2903    1.4496 H   0  0  0  0  0  0  0  0  0  0  0  0
   -4.8677   -4.4552    3.1915 H   0  0  0  0  0  0  0  0  0  0  0  0
   -0.3389   -5.2616    1.6475 C   0  0  0  0  0  0  0  0  0  0  0  0
   -1.1706   -4.5584    1.8938 H   0  0  0  0  0  0  0  0  0  0  0  0
   -0.6673   -6.3026    1.9284 H   0  0  0  0  0  0  0  0  0  0  0  0
    0.5215   -5.0092    2.3195 H   0  0  0  0  0  0  0  0  0  0  0  0
   -1.1277   -6.0835   -3.3636 C   0  0  0  0  0  0  0  0  0  0  0  0
   -2.2006   -6.2867   -3.1627 H   0  0  0  0  0  0  0  0  0  0  0  0
   -1.0622   -5.3125   -4.1713 H   0  0  0  0  0  0  0  0  0  0  0  0
   -0.6938   -7.0351   -3.7748 H   0  0  0  0  0  0  0  0  0  0  0  0
   -2.2416   -2.9198   -2.9110 C   0  0  0  0  0  0  0  0  0  0  0  0
   -1.2277   -3.3920   -2.9080 H   0  0  0  0  0  0  0  0  0  0  0  0
   -2.2657   -2.2290   -3.7945 H   0  0  0  0  0  0  0  0  0  0  0  0
   -2.3679   -2.2626   -1.9959 H   0  0  0  0  0  0  0  0  0  0  0  0
```

```
   -5.3719   -6.6249   -1.1257 C   0  0  0  0  0  0  0  0  0  0  0  0
   -4.7375   -7.5357   -0.9846 H   0  0  0  0  0  0  0  0  0  0  0  0
   -5.4994   -6.1118   -0.1451 H   0  0  0  0  0  0  0  0  0  0  0  0
   -6.3868   -6.9900   -1.4287 H   0  0  0  0  0  0  0  0  0  0  0  0
  1 26  1  0  0  0  0
  1 34  1  0  0  0  0
  1 42  1  0  0  0  0
  1 50  1  0  0  0  0
  2  3  1  0  0  0  0
  2  4  1  0  0  0  0
  2 10  1  0  0  0  0
  2 58  1  0  0  0  0
  3 13  1  0  0  0  0
  3 16  1  0  0  0  0
  3 59  1  0  0  0  0
  4  6  2  0  0  0  0
  4  8  1  0  0  0  0
  4 50  1  0  0  0  0
  5  6  1  0  0  0  0
  5  7  1  0  0  0  0
  5 11  1  0  0  0  0
  5 56  1  0  0  0  0
  6  9  1  0  0  0  0
  6 50  1  0  0  0  0
  7 12  1  0  0  0  0
  7 17  1  0  0  0  0
  7 57  1  0  0  0  0
 12 13  2  0  0  0  0
 12 14  1  0  0  0  0
 12 50  1  0  0  0  0
 13 15  1  0  0  0  0
 13 50  1  0  0  0  0
 18 19  3  0  0  0  0
 18 50  1  0  0  0  0
 19 20  1  0  0  0  0
 20 21  2  0  0  0  0
 20 22  1  0  0  0  0
 21 23  1  0  0  0  0
 21 54  1  0  0  0  0
 22 24  2  0  0  0  0
 22 55  1  0  0  0  0
 23 25  2  0  0  0  0
 23 52  1  0  0  0  0
 24 25  1  0  0  0  0
 24 53  1  0  0  0  0
 25 51  1  0  0  0  0
 26 27  2  0  0  0  0
 26 28  1  0  0  0  0
 27 29  1  0  0  0  0
 27 71  1  0  0  0  0
 28 30  2  0  0  0  0
 28 75  1  0  0  0  0
 29 31  2  0  0  0  0
 29 32  1  0  0  0  0
 30 31  1  0  0  0  0
 30 33  1  0  0  0  0
 31 61  1  0  0  0  0
 34 35  2  0  0  0  0
 34 36  1  0  0  0  0
 35 37  1  0  0  0  0
 35 63  1  0  0  0  0
 36 38  2  0  0  0  0
 36 67  1  0  0  0  0
 37 39  2  0  0  0  0
 37 40  1  0  0  0  0
```

```
 38 39  1  0  0  0  0
 38 41  1  0  0  0  0
 39 60  1  0  0  0  0
 42 43  2  0  0  0  0
 42 44  1  0  0  0  0
 43 45  1  0  0  0  0
 43 83  1  0  0  0  0
 44 46  2  0  0  0  0
 44 79  1  0  0  0  0
 45 47  2  0  0  0  0
 45 48  1  0  0  0  0
 46 47  1  0  0  0  0
 46 49  1  0  0  0  0
 47 62  1  0  0  0  0
 63 64  1  0  0  0  0
 63 65  1  0  0  0  0
 63 66  1  0  0  0  0
 67 68  1  0  0  0  0
 67 69  1  0  0  0  0
 67 70  1  0  0  0  0
 71 72  1  0  0  0  0
 71 73  1  0  0  0  0
 71 74  1  0  0  0  0
 75 76  1  0  0  0  0
 75 77  1  0  0  0  0
 75 78  1  0  0  0  0
 79 80  1  0  0  0  0
 79 81  1  0  0  0  0
 79 82  1  0  0  0  0
 83 84  1  0  0  0  0
 83 85  1  0  0  0  0
 83 86  1  0  0  0  0
M  END
$$$$
mol074
Optimized Catalyst 074
Optimized Catalyst 074
 68 75  0  0  0  0  0  0  0  0999 V2000
   -2.4353   -4.6402   -0.7311 P   0  0  0  0  0  0  0  0  0  0  0  0
   -0.4293    0.5906   -1.0018 C   0  0  0  0  0  0  0  0  0  0  0  0
    0.6783   -0.3506   -0.4887 C   0  0  0  0  0  0  0  0  0  0  0  0
   -1.7598    0.5300   -0.2979 C   0  0  0  0  0  0  0  0  0  0  0  0
   -0.8928    0.3508    2.0783 C   0  0  0  0  0  0  0  0  0  0  0  0
   -1.9721    0.4250    1.0339 C   0  0  0  0  0  0  0  0  0  0  0  0
   -0.4008   -1.0811    2.3653 C   0  0  0  0  0  0  0  0  0  0  0  0
   -2.6183    0.7192   -0.9725 H   0  0  0  0  0  0  0  0  0  0  0  0
   -2.9973    0.4928    1.4396 H   0  0  0  0  0  0  0  0  0  0  0  0
   -0.0568    1.6525   -0.9095 H   0  0  0  0  0  0  0  0  0  0  0  0
   -0.0345    1.0041    1.7707 H   0  0  0  0  0  0  0  0  0  0  0  0
   -0.2649   -2.0213    1.1892 C   0  0  0  0  0  0  0  0  0  0  0  0
    0.2130   -1.7129   -0.0509 C   0  0  0  0  0  0  0  0  0  0  0  0
   -0.3380   -3.0877    1.4979 H   0  0  0  0  0  0  0  0  0  0  0  0
    0.4395   -2.5216   -0.7755 H   0  0  0  0  0  0  0  0  0  0  0  0
    1.2239    0.1318    0.3678 H   0  0  0  0  0  0  0  0  0  0  0  0
    0.6213   -1.0103    2.8409 H   0  0  0  0  0  0  0  0  0  0  0  0
   -4.2474   -2.1052   -0.6410 N   0  0  0  0  0  0  0  0  0  0  0  0
   -5.3607   -2.1186   -0.9987 C   0  0  0  0  0  0  0  0  0  0  0  0
   -6.7070   -2.1537   -1.4414 C   0  0  0  0  0  0  0  0  0  0  0  0
   -7.2223   -3.3504   -1.9927 C   0  0  0  0  0  0  0  0  0  0  0  0
   -7.5188   -1.0015   -1.3328 C   0  0  0  0  0  0  0  0  0  0  0  0
   -8.5375   -3.3970   -2.4380 C   0  0  0  0  0  0  0  0  0  0  0  0
   -8.8372   -1.0413   -1.7738 C   0  0  0  0  0  0  0  0  0  0  0  0
   -9.3224   -2.2390   -2.3191 C   0  0  0  0  0  0  0  0  0  0  0  0
   -0.7832   -5.3319   -1.1911 C   0  0  0  0  0  0  0  0  0  0  0  0
    0.1077   -5.6857   -0.1660 C   0  0  0  0  0  0  0  0  0  0  0  0
```

```
   -0.4561   -5.5380   -2.5365 C   0  0  0  0  0  0  0  0  0  0  0  0
    1.3511   -6.2290   -0.4842 C   0  0  0  0  0  0  0  0  0  0  0  0
   -0.1460   -5.5646    0.8923 H   0  0  0  0  0  0  0  0  0  0  0  0
    0.7861   -6.0807   -2.8737 C   0  0  0  0  0  0  0  0  0  0  0  0
   -1.1495   -5.2960   -3.3492 H   0  0  0  0  0  0  0  0  0  0  0  0
    1.6663   -6.4155   -1.8384 C   0  0  0  0  0  0  0  0  0  0  0  0
    2.0601   -6.5120    0.2998 H   0  0  0  0  0  0  0  0  0  0  0  0
    1.0594   -6.2476   -3.9203 H   0  0  0  0  0  0  0  0  0  0  0  0
   -3.0379   -5.6338    0.7069 C   0  0  0  0  0  0  0  0  0  0  0  0
   -2.8043   -7.0143    0.7544 C   0  0  0  0  0  0  0  0  0  0  0  0
   -3.7361   -4.9769    1.7283 C   0  0  0  0  0  0  0  0  0  0  0  0
   -3.2893   -7.7589    1.8313 C   0  0  0  0  0  0  0  0  0  0  0  0
   -2.2420   -7.5348   -0.0300 H   0  0  0  0  0  0  0  0  0  0  0  0
   -4.2301   -5.7047    2.8105 C   0  0  0  0  0  0  0  0  0  0  0  0
   -3.9051   -3.8862    1.7096 H   0  0  0  0  0  0  0  0  0  0  0  0
   -3.9974   -7.0879    2.8363 C   0  0  0  0  0  0  0  0  0  0  0  0
   -3.1178   -8.8385    1.8906 H   0  0  0  0  0  0  0  0  0  0  0  0
   -4.7804   -5.2143    3.6187 H   0  0  0  0  0  0  0  0  0  0  0  0
   -3.5799   -4.9681   -2.1529 C   0  0  0  0  0  0  0  0  0  0  0  0
   -4.4313   -6.0772   -2.1253 C   0  0  0  0  0  0  0  0  0  0  0  0
   -3.5375   -4.1141   -3.2654 C   0  0  0  0  0  0  0  0  0  0  0  0
   -5.2546   -6.3475   -3.2230 C   0  0  0  0  0  0  0  0  0  0  0  0
   -4.4853   -6.7510   -1.2624 H   0  0  0  0  0  0  0  0  0  0  0  0
   -4.3478   -4.3706   -4.3700 C   0  0  0  0  0  0  0  0  0  0  0  0
   -2.8807   -3.2352   -3.2998 H   0  0  0  0  0  0  0  0  0  0  0  0
   -5.1886   -5.4932   -4.3282 C   0  0  0  0  0  0  0  0  0  0  0  0
   -5.9202   -7.2163   -3.2218 H   0  0  0  0  0  0  0  0  0  0  0  0
   -4.3240   -3.7218   -5.2509 H   0  0  0  0  0  0  0  0  0  0  0  0
   -2.3965   -2.1176   -0.0798 Ir  0  0  0  0  0  0  0  0  0  0  0  0
   -8.9538   -4.3127   -2.8700 H   0  0  0  0  0  0  0  0  0  0  0  0
   -9.4817   -0.1585   -1.6970 H   0  0  0  0  0  0  0  0  0  0  0  0
   -6.5857   -4.2360   -2.0671 H   0  0  0  0  0  0  0  0  0  0  0  0
   -7.1125   -0.0816   -0.9033 H   0  0  0  0  0  0  0  0  0  0  0  0
   -1.2872    0.7812    3.0403 H   0  0  0  0  0  0  0  0  0  0  0  0
   -1.0879   -1.5448    3.1252 H   0  0  0  0  0  0  0  0  0  0  0  0
   -0.5845    0.3911   -2.0962 H   0  0  0  0  0  0  0  0  0  0  0  0
    1.4320   -0.4748   -1.3156 H   0  0  0  0  0  0  0  0  0  0  0  0
   -5.9642   -5.7435   -5.3751 F   0  0  0  0  0  0  0  0  0  0  0  0
   -4.4655   -7.7887    3.8606 F   0  0  0  0  0  0  0  0  0  0  0  0
    2.8479   -6.9323   -2.1488 F   0  0  0  0  0  0  0  0  0  0  0  0
  -10.5776   -2.2805   -2.7407 F   0  0  0  0  0  0  0  0  0  0  0  0
  1 26  1  0  0  0  0
  1 36  1  0  0  0  0
  1 46  1  0  0  0  0
  1 56  1  0  0  0  0
  2  3  1  0  0  0  0
  2  4  1  0  0  0  0
  2 10  1  0  0  0  0
  2 63  1  0  0  0  0
  3 13  1  0  0  0  0
  3 16  1  0  0  0  0
  3 64  1  0  0  0  0
  4  6  2  0  0  0  0
  4  8  1  0  0  0  0
  4 56  1  0  0  0  0
  5  6  1  0  0  0  0
  5  7  1  0  0  0  0
  5 11  1  0  0  0  0
  5 61  1  0  0  0  0
  6  9  1  0  0  0  0
  6 56  1  0  0  0  0
  7 12  1  0  0  0  0
  7 17  1  0  0  0  0
  7 62  1  0  0  0  0
 12 13  2  0  0  0  0
```

```
 12 14  1  0  0  0  0
 12 56  1  0  0  0  0
 13 15  1  0  0  0  0
 13 56  1  0  0  0  0
 18 19  3  0  0  0  0
 18 56  1  0  0  0  0
 19 20  1  0  0  0  0
 20 21  2  0  0  0  0
 20 22  1  0  0  0  0
 21 23  1  0  0  0  0
 21 59  1  0  0  0  0
 22 24  2  0  0  0  0
 22 60  1  0  0  0  0
 23 25  2  0  0  0  0
 23 57  1  0  0  0  0
 24 25  1  0  0  0  0
 24 58  1  0  0  0  0
 25 68  1  0  0  0  0
 26 27  2  0  0  0  0
 26 28  1  0  0  0  0
 27 29  1  0  0  0  0
 27 30  1  0  0  0  0
 28 31  2  0  0  0  0
 28 32  1  0  0  0  0
 29 33  2  0  0  0  0
 29 34  1  0  0  0  0
 31 33  1  0  0  0  0
 31 35  1  0  0  0  0
 33 67  1  0  0  0  0
 36 37  2  0  0  0  0
 36 38  1  0  0  0  0
 37 39  1  0  0  0  0
 37 40  1  0  0  0  0
 38 41  2  0  0  0  0
 38 42  1  0  0  0  0
 39 43  2  0  0  0  0
 39 44  1  0  0  0  0
 41 43  1  0  0  0  0
 41 45  1  0  0  0  0
 43 66  1  0  0  0  0
 46 47  2  0  0  0  0
 46 48  1  0  0  0  0
 47 49  1  0  0  0  0
 47 50  1  0  0  0  0
 48 51  2  0  0  0  0
 48 52  1  0  0  0  0
 49 53  2  0  0  0  0
 49 54  1  0  0  0  0
 51 53  1  0  0  0  0
 51 55  1  0  0  0  0
 53 65  1  0  0  0  0
M  END
$$$$
mol075
Optimized Catalyst 075
Optimized Catalyst 075
 80 87  0  0  0  0  0  0  0  0999 V2000
   -2.4366   -4.6468   -0.7320 P   0  0  0  0  0  0  0  0  0  0  0  0
   -0.4235    0.5937   -1.0004 C   0  0  0  0  0  0  0  0  0  0  0  0
    0.6835   -0.3495   -0.4887 C   0  0  0  0  0  0  0  0  0  0  0  0
   -1.7524    0.5375   -0.2937 C   0  0  0  0  0  0  0  0  0  0  0  0
   -0.8865    0.3548    2.0818 C   0  0  0  0  0  0  0  0  0  0  0  0
   -1.9647    0.4320    1.0368 C   0  0  0  0  0  0  0  0  0  0  0  0
   -0.3958   -1.0785    2.3670 C   0  0  0  0  0  0  0  0  0  0  0  0
   -2.6121    0.7188   -0.9681 H   0  0  0  0  0  0  0  0  0  0  0  0
```

-2.9904	0.4932	1.4410	H	0	0	0	0	0	0	0	0	0	0	0	0
-0.0485	1.6540	-0.9121	H	0	0	0	0	0	0	0	0	0	0	0	0
-0.0280	1.0069	1.7744	H	0	0	0	0	0	0	0	0	0	0	0	0
-0.2577	-2.0185	1.1910	C	0	0	0	0	0	0	0	0	0	0	0	0
0.2180	-1.7114	-0.0498	C	0	0	0	0	0	0	0	0	0	0	0	0
-0.3351	-3.0857	1.4978	H	0	0	0	0	0	0	0	0	0	0	0	0
0.4401	-2.5216	-0.7747	H	0	0	0	0	0	0	0	0	0	0	0	0
1.2293	0.1316	0.3676	H	0	0	0	0	0	0	0	0	0	0	0	0
0.6242	-1.0095	2.8449	H	0	0	0	0	0	0	0	0	0	0	0	0
-4.2475	-2.1251	-0.6402	N	0	0	0	0	0	0	0	0	0	0	0	0
-5.3595	-2.1306	-1.0000	C	0	0	0	0	0	0	0	0	0	0	0	0
-6.7032	-2.1610	-1.4460	C	0	0	0	0	0	0	0	0	0	0	0	0
-7.2221	-3.3517	-2.0001	C	0	0	0	0	0	0	0	0	0	0	0	0
-7.5208	-1.0138	-1.3384	C	0	0	0	0	0	0	0	0	0	0	0	0
-8.5388	-3.3883	-2.4417	C	0	0	0	0	0	0	0	0	0	0	0	0
-8.8351	-1.0648	-1.7834	C	0	0	0	0	0	0	0	0	0	0	0	0
-9.3633	-2.2498	-2.3383	C	0	0	0	0	0	0	0	0	0	0	0	0
-0.7821	-5.3227	-1.1889	C	0	0	0	0	0	0	0	0	0	0	0	0
0.1115	-5.6796	-0.1692	C	0	0	0	0	0	0	0	0	0	0	0	0
-0.4472	-5.5326	-2.5309	C	0	0	0	0	0	0	0	0	0	0	0	0
1.3521	-6.2215	-0.4969	C	0	0	0	0	0	0	0	0	0	0	0	0
-0.1408	-5.5625	0.8889	H	0	0	0	0	0	0	0	0	0	0	0	0
0.7969	-6.0757	-2.8542	C	0	0	0	0	0	0	0	0	0	0	0	0
-1.1376	-5.2935	-3.3457	H	0	0	0	0	0	0	0	0	0	0	0	0
1.7132	-6.4223	-1.8443	C	0	0	0	0	0	0	0	0	0	0	0	0
2.0469	-6.5038	0.2967	H	0	0	0	0	0	0	0	0	0	0	0	0
1.0581	-6.2434	-3.9013	H	0	0	0	0	0	0	0	0	0	0	0	0
-3.0370	-5.6292	0.7101	C	0	0	0	0	0	0	0	0	0	0	0	0
-2.8052	-7.0078	0.7609	C	0	0	0	0	0	0	0	0	0	0	0	0
-3.7366	-4.9823	1.7356	C	0	0	0	0	0	0	0	0	0	0	0	0
-3.2967	-7.7468	1.8381	C	0	0	0	0	0	0	0	0	0	0	0	0
-2.2406	-7.5279	-0.0204	H	0	0	0	0	0	0	0	0	0	0	0	0
-4.2267	-5.7237	2.8082	C	0	0	0	0	0	0	0	0	0	0	0	0
-3.9070	-3.8925	1.7239	H	0	0	0	0	0	0	0	0	0	0	0	0
-4.0182	-7.1175	2.8684	C	0	0	0	0	0	0	0	0	0	0	0	0
-3.1150	-8.8227	1.8810	H	0	0	0	0	0	0	0	0	0	0	0	0
-4.7733	-5.2264	3.6117	H	0	0	0	0	0	0	0	0	0	0	0	0
-3.5818	-4.9583	-2.1514	C	0	0	0	0	0	0	0	0	0	0	0	0
-4.4380	-6.0627	-2.1297	C	0	0	0	0	0	0	0	0	0	0	0	0
-3.5432	-4.1093	-3.2658	C	0	0	0	0	0	0	0	0	0	0	0	0
-5.2513	-6.3255	-3.2345	C	0	0	0	0	0	0	0	0	0	0	0	0
-4.4980	-6.7349	-1.2673	H	0	0	0	0	0	0	0	0	0	0	0	0
-4.3544	-4.3769	-4.3664	C	0	0	0	0	0	0	0	0	0	0	0	0
-2.8899	-3.2294	-3.3023	H	0	0	0	0	0	0	0	0	0	0	0	0
-5.2100	-5.4969	-4.3694	C	0	0	0	0	0	0	0	0	0	0	0	0
-5.9154	-7.1922	-3.2211	H	0	0	0	0	0	0	0	0	0	0	0	0
-4.3227	-3.7181	-5.2366	H	0	0	0	0	0	0	0	0	0	0	0	0
-2.3954	-2.1332	-0.0744	Ir	0	0	0	0	0	0	0	0	0	0	0	0
-8.9380	-4.3094	-2.8706	H	0	0	0	0	0	0	0	0	0	0	0	0
-9.4688	-0.1771	-1.7046	H	0	0	0	0	0	0	0	0	0	0	0	0
-6.5875	-4.2383	-2.0774	H	0	0	0	0	0	0	0	0	0	0	0	0
-7.1190	-0.0951	-0.9052	H	0	0	0	0	0	0	0	0	0	0	0	0
-1.2820	0.7826	3.0438	H	0	0	0	0	0	0	0	0	0	0	0	0
-1.0868	-1.5428	3.1228	H	0	0	0	0	0	0	0	0	0	0	0	0
-0.5832	0.3894	-2.0932	H	0	0	0	0	0	0	0	0	0	0	0	0
1.4345	-0.4751	-1.3170	H	0	0	0	0	0	0	0	0	0	0	0	0
3.0351	-7.0107	-2.1983	C	0	0	0	0	0	0	0	0	0	0	0	0
2.9080	-8.1013	-2.4227	H	0	0	0	0	0	0	0	0	0	0	0	0
3.4662	-6.5165	-3.1034	H	0	0	0	0	0	0	0	0	0	0	0	0
3.7724	-6.9105	-1.3660	H	0	0	0	0	0	0	0	0	0	0	0	0
-4.5438	-7.8999	4.0220	C	0	0	0	0	0	0	0	0	0	0	0	0
-3.8956	-7.7246	4.9189	H	0	0	0	0	0	0	0	0	0	0	0	0
-5.5859	-7.5849	4.2761	H	0	0	0	0	0	0	0	0	0	0	0	0
-4.5567	-8.9964	3.8121	H	0	0	0	0	0	0	0	0	0	0	0	0
-10.7755	-2.2857	-2.8060	C	0	0	0	0	0	0	0	0	0	0	0	0

```
 -11.4688   -2.0404   -1.9607 H   0  0  0  0  0  0  0  0  0  0  0  0
 -10.9312   -1.5282   -3.6164 H   0  0  0  0  0  0  0  0  0  0  0  0
 -11.0643   -3.2871   -3.2051 H   0  0  0  0  0  0  0  0  0  0  0  0
  -6.0749   -5.7914   -5.5465 C   0  0  0  0  0  0  0  0  0  0  0  0
  -6.0890   -6.8886   -5.7642 H   0  0  0  0  0  0  0  0  0  0  0  0
  -7.1226   -5.4599   -5.3370 H   0  0  0  0  0  0  0  0  0  0  0  0
  -5.7186   -5.2665   -6.4659 H   0  0  0  0  0  0  0  0  0  0  0  0
 1 26  1  0  0  0  0
 1 36  1  0  0  0  0
 1 46  1  0  0  0  0
 1 56  1  0  0  0  0
 2  3  1  0  0  0  0
 2  4  1  0  0  0  0
 2 10  1  0  0  0  0
 2 63  1  0  0  0  0
 3 13  1  0  0  0  0
 3 16  1  0  0  0  0
 3 64  1  0  0  0  0
 4  6  2  0  0  0  0
 4  8  1  0  0  0  0
 4 56  1  0  0  0  0
 5  6  1  0  0  0  0
 5  7  1  0  0  0  0
 5 11  1  0  0  0  0
 5 61  1  0  0  0  0
 6  9  1  0  0  0  0
 6 56  1  0  0  0  0
 7 12  1  0  0  0  0
 7 17  1  0  0  0  0
 7 62  1  0  0  0  0
12 13  2  0  0  0  0
12 14  1  0  0  0  0
12 56  1  0  0  0  0
13 15  1  0  0  0  0
13 56  1  0  0  0  0
18 19  3  0  0  0  0
18 56  1  0  0  0  0
19 20  1  0  0  0  0
20 21  2  0  0  0  0
20 22  1  0  0  0  0
21 23  1  0  0  0  0
21 59  1  0  0  0  0
22 24  2  0  0  0  0
22 60  1  0  0  0  0
23 25  2  0  0  0  0
23 57  1  0  0  0  0
24 25  1  0  0  0  0
24 58  1  0  0  0  0
25 73  1  0  0  0  0
26 27  2  0  0  0  0
26 28  1  0  0  0  0
27 29  1  0  0  0  0
27 30  1  0  0  0  0
28 31  2  0  0  0  0
28 32  1  0  0  0  0
29 33  2  0  0  0  0
29 34  1  0  0  0  0
31 33  1  0  0  0  0
31 35  1  0  0  0  0
33 65  1  0  0  0  0
36 37  2  0  0  0  0
36 38  1  0  0  0  0
37 39  1  0  0  0  0
37 40  1  0  0  0  0
38 41  2  0  0  0  0
```

```
 38 42  1  0  0  0  0
 39 43  2  0  0  0  0
 39 44  1  0  0  0  0
 41 43  1  0  0  0  0
 41 45  1  0  0  0  0
 43 69  1  0  0  0  0
 46 47  2  0  0  0  0
 46 48  1  0  0  0  0
 47 49  1  0  0  0  0
 47 50  1  0  0  0  0
 48 51  2  0  0  0  0
 48 52  1  0  0  0  0
 49 53  2  0  0  0  0
 49 54  1  0  0  0  0
 51 53  1  0  0  0  0
 51 55  1  0  0  0  0
 53 77  1  0  0  0  0
 65 66  1  0  0  0  0
 65 67  1  0  0  0  0
 65 68  1  0  0  0  0
 69 70  1  0  0  0  0
 69 71  1  0  0  0  0
 69 72  1  0  0  0  0
 73 74  1  0  0  0  0
 73 75  1  0  0  0  0
 73 76  1  0  0  0  0
 77 78  1  0  0  0  0
 77 79  1  0  0  0  0
 77 80  1  0  0  0  0
M  END
$$$$
mol076
Optimized Catalyst 076
Optimized Catalyst 076
 77 84  0  0  0  0  0  0  0  0999 V2000
   -2.4398   -4.5948   -0.7129 P   0  0  0  0  0  0  0  0  0  0  0  0
   -0.3159    0.6033   -0.9706 C   0  0  0  0  0  0  0  0  0  0  0  0
    0.7700   -0.3537   -0.4398 C   0  0  0  0  0  0  0  0  0  0  0  0
   -1.6540    0.5739   -0.2801 C   0  0  0  0  0  0  0  0  0  0  0  0
   -0.8221    0.3901    2.1066 C   0  0  0  0  0  0  0  0  0  0  0  0
   -1.8858    0.4798    1.0480 C   0  0  0  0  0  0  0  0  0  0  0  0
   -0.3585   -1.0499    2.4028 C   0  0  0  0  0  0  0  0  0  0  0  0
   -2.5014    0.7665   -0.9663 H   0  0  0  0  0  0  0  0  0  0  0  0
   -2.9157    0.5628    1.4388 H   0  0  0  0  0  0  0  0  0  0  0  0
    0.0754    1.6581   -0.8850 H   0  0  0  0  0  0  0  0  0  0  0  0
    0.0503    1.0275    1.8075 H   0  0  0  0  0  0  0  0  0  0  0  0
   -0.2292   -1.9968    1.2309 C   0  0  0  0  0  0  0  0  0  0  0  0
    0.2744   -1.7043   -0.0020 C   0  0  0  0  0  0  0  0  0  0  0  0
   -0.3324   -3.0614    1.5403 H   0  0  0  0  0  0  0  0  0  0  0  0
    0.4855   -2.5206   -0.7228 H   0  0  0  0  0  0  0  0  0  0  0  0
    1.3126    0.1229    0.4212 H   0  0  0  0  0  0  0  0  0  0  0  0
    0.6591   -0.9958    2.8882 H   0  0  0  0  0  0  0  0  0  0  0  0
   -4.2052   -2.1008   -0.6348 N   0  0  0  0  0  0  0  0  0  0  0  0
   -5.2916   -2.1919   -1.0552 C   0  0  0  0  0  0  0  0  0  0  0  0
   -6.6036   -2.3457   -1.5732 C   0  0  0  0  0  0  0  0  0  0  0  0
   -7.2532   -3.5926   -1.4241 C   0  0  0  0  0  0  0  0  0  0  0  0
   -7.2383   -1.2749   -2.2407 C   0  0  0  0  0  0  0  0  0  0  0  0
   -8.5286   -3.7754   -1.9469 C   0  0  0  0  0  0  0  0  0  0  0  0
   -8.5160   -1.4488   -2.7633 C   0  0  0  0  0  0  0  0  0  0  0  0
   -9.1364   -2.6965   -2.6060 C   0  0  0  0  0  0  0  0  0  0  0  0
   -0.8072   -5.2973   -1.1983 C   0  0  0  0  0  0  0  0  0  0  0  0
    0.1012   -5.6366   -0.1841 C   0  0  0  0  0  0  0  0  0  0  0  0
   -0.4989   -5.5449   -2.5392 C   0  0  0  0  0  0  0  0  0  0  0  0
    1.3278   -6.2013   -0.5194 C   0  0  0  0  0  0  0  0  0  0  0  0
   -0.1307   -5.4885    0.8746 H   0  0  0  0  0  0  0  0  0  0  0  0
```

```
   0.7339   -6.1117   -2.8702 C   0  0  0  0  0  0  0  0  0  0  0  0
  -1.2003   -5.3186   -3.3480 H   0  0  0  0  0  0  0  0  0  0  0  0
   1.6608   -6.4471   -1.8681 C   0  0  0  0  0  0  0  0  0  0  0  0
   2.0381   -6.4655    0.2665 H   0  0  0  0  0  0  0  0  0  0  0  0
   0.9757   -6.3021   -3.9180 H   0  0  0  0  0  0  0  0  0  0  0  0
  -3.0329   -5.5747    0.7333 C   0  0  0  0  0  0  0  0  0  0  0  0
  -2.8497   -6.9612    0.7480 C   0  0  0  0  0  0  0  0  0  0  0  0
  -3.6825   -4.9248    1.7894 C   0  0  0  0  0  0  0  0  0  0  0  0
  -3.3442   -7.7059    1.8197 C   0  0  0  0  0  0  0  0  0  0  0  0
  -2.3235   -7.4836   -0.0586 H   0  0  0  0  0  0  0  0  0  0  0  0
  -4.1747   -5.6722    2.8566 C   0  0  0  0  0  0  0  0  0  0  0  0
  -3.8105   -3.8286    1.8052 H   0  0  0  0  0  0  0  0  0  0  0  0
  -4.0173   -7.0743    2.8804 C   0  0  0  0  0  0  0  0  0  0  0  0
  -3.2019   -8.7884    1.8338 H   0  0  0  0  0  0  0  0  0  0  0  0
  -4.6816   -5.1743    3.6853 H   0  0  0  0  0  0  0  0  0  0  0  0
  -3.6422   -4.8703   -2.0915 C   0  0  0  0  0  0  0  0  0  0  0  0
  -4.5944   -5.8879   -1.9937 C   0  0  0  0  0  0  0  0  0  0  0  0
  -3.5790   -4.0633   -3.2367 C   0  0  0  0  0  0  0  0  0  0  0  0
  -5.4987   -6.0918   -3.0396 C   0  0  0  0  0  0  0  0  0  0  0  0
  -4.6648   -6.5382   -1.1152 H   0  0  0  0  0  0  0  0  0  0  0  0
  -4.4794   -4.2723   -4.2773 C   0  0  0  0  0  0  0  0  0  0  0  0
  -2.8405   -3.2604   -3.3403 H   0  0  0  0  0  0  0  0  0  0  0  0
  -5.4522   -5.2912   -4.1923 C   0  0  0  0  0  0  0  0  0  0  0  0
  -6.2398   -6.8896   -2.9606 H   0  0  0  0  0  0  0  0  0  0  0  0
  -4.4319   -3.6472   -5.1712 H   0  0  0  0  0  0  0  0  0  0  0  0
  -2.3685   -2.0888   -0.0298 Ir  0  0  0  0  0  0  0  0  0  0  0  0
  -9.0477   -4.7327   -1.8395 H   0  0  0  0  0  0  0  0  0  0  0  0
  -9.0261   -0.6288   -3.2799 H   0  0  0  0  0  0  0  0  0  0  0  0
  -6.7505   -4.4116   -0.9023 H   0  0  0  0  0  0  0  0  0  0  0  0
  -6.7274   -0.3139   -2.3459 H   0  0  0  0  0  0  0  0  0  0  0  0
  -1.2223    0.8284    3.0620 H   0  0  0  0  0  0  0  0  0  0  0  0
  -1.0624   -1.4989    3.1560 H   0  0  0  0  0  0  0  0  0  0  0  0
  -0.4657    0.3936   -2.0636 H   0  0  0  0  0  0  0  0  0  0  0  0
   1.5298   -0.4954   -1.2575 H   0  0  0  0  0  0  0  0  0  0  0  0
   2.9823   -7.0417   -2.2120 C   0  0  0  0  0  0  0  0  0  0  0  0
   2.9753   -7.5163   -3.2227 H   0  0  0  0  0  0  0  0  0  0  0  0
   3.7638   -6.2392   -2.2079 H   0  0  0  0  0  0  0  0  0  0  0  0
   3.2775   -7.8220   -1.4676 H   0  0  0  0  0  0  0  0  0  0  0  0
  -4.5494   -7.8650    4.0250 C   0  0  0  0  0  0  0  0  0  0  0  0
  -3.9284   -7.6727    4.9374 H   0  0  0  0  0  0  0  0  0  0  0  0
  -5.6047   -7.5735    4.2517 H   0  0  0  0  0  0  0  0  0  0  0  0
  -4.5309   -8.9624    3.8196 H   0  0  0  0  0  0  0  0  0  0  0  0
  -6.4206   -5.4909   -5.3065 C   0  0  0  0  0  0  0  0  0  0  0  0
  -6.9949   -6.4423   -5.1986 H   0  0  0  0  0  0  0  0  0  0  0  0
  -7.1495   -4.6424   -5.3313 H   0  0  0  0  0  0  0  0  0  0  0  0
  -5.8867   -5.5246   -6.2897 H   0  0  0  0  0  0  0  0  0  0  0  0
 -10.3529   -2.8658   -3.1057 F   0  0  0  0  0  0  0  0  0  0  0  0
 1 26  1  0  0  0  0
 1 36  1  0  0  0  0
 1 46  1  0  0  0  0
 1 56  1  0  0  0  0
 2  3  1  0  0  0  0
 2  4  1  0  0  0  0
 2 10  1  0  0  0  0
 2 63  1  0  0  0  0
 3 13  1  0  0  0  0
 3 16  1  0  0  0  0
 3 64  1  0  0  0  0
 4  6  2  0  0  0  0
 4  8  1  0  0  0  0
 4 56  1  0  0  0  0
 5  6  1  0  0  0  0
 5  7  1  0  0  0  0
 5 11  1  0  0  0  0
 5 61  1  0  0  0  0
```

```
  6  9  1  0  0  0  0
  6 56  1  0  0  0  0
  7 12  1  0  0  0  0
  7 17  1  0  0  0  0
  7 62  1  0  0  0  0
 12 13  2  0  0  0  0
 12 14  1  0  0  0  0
 12 56  1  0  0  0  0
 13 15  1  0  0  0  0
 13 56  1  0  0  0  0
 18 19  3  0  0  0  0
 18 56  1  0  0  0  0
 19 20  1  0  0  0  0
 20 21  2  0  0  0  0
 20 22  1  0  0  0  0
 21 23  1  0  0  0  0
 21 59  1  0  0  0  0
 22 24  2  0  0  0  0
 22 60  1  0  0  0  0
 23 25  2  0  0  0  0
 23 57  1  0  0  0  0
 24 25  1  0  0  0  0
 24 58  1  0  0  0  0
 25 77  1  0  0  0  0
 26 27  2  0  0  0  0
 26 28  1  0  0  0  0
 27 29  1  0  0  0  0
 27 30  1  0  0  0  0
 28 31  2  0  0  0  0
 28 32  1  0  0  0  0
 29 33  2  0  0  0  0
 29 34  1  0  0  0  0
 31 33  1  0  0  0  0
 31 35  1  0  0  0  0
 33 65  1  0  0  0  0
 36 37  2  0  0  0  0
 36 38  1  0  0  0  0
 37 39  1  0  0  0  0
 37 40  1  0  0  0  0
 38 41  2  0  0  0  0
 38 42  1  0  0  0  0
 39 43  2  0  0  0  0
 39 44  1  0  0  0  0
 41 43  1  0  0  0  0
 41 45  1  0  0  0  0
 43 69  1  0  0  0  0
 46 47  2  0  0  0  0
 46 48  1  0  0  0  0
 47 49  1  0  0  0  0
 47 50  1  0  0  0  0
 48 51  2  0  0  0  0
 48 52  1  0  0  0  0
 49 53  2  0  0  0  0
 49 54  1  0  0  0  0
 51 53  1  0  0  0  0
 51 55  1  0  0  0  0
 53 73  1  0  0  0  0
 65 66  1  0  0  0  0
 65 67  1  0  0  0  0
 65 68  1  0  0  0  0
 69 70  1  0  0  0  0
 69 71  1  0  0  0  0
 69 72  1  0  0  0  0
 73 74  1  0  0  0  0
 73 75  1  0  0  0  0
```

```
 73 76  1  0  0  0  0
M  END
$$$$
mol077
Optimized Catalyst 077
Optimized Catalyst 077
 71 78  0  0  0  0  0  0  0  0999 V2000
   -2.3677   -4.6479   -0.8095 P   0  0  0  0  0  0  0  0  0  0  0  0
   -0.4376    0.5780   -1.2261 C   0  0  0  0  0  0  0  0  0  0  0  0
    0.6965   -0.3276   -0.7059 C   0  0  0  0  0  0  0  0  0  0  0  0
   -1.7550    0.5134   -0.4988 C   0  0  0  0  0  0  0  0  0  0  0  0
   -0.8476    0.4359    1.8680 C   0  0  0  0  0  0  0  0  0  0  0  0
   -1.9445    0.4433    0.8390 C   0  0  0  0  0  0  0  0  0  0  0  0
   -0.2985   -0.9671    2.1938 C   0  0  0  0  0  0  0  0  0  0  0  0
   -2.6286    0.6629   -1.1645 H   0  0  0  0  0  0  0  0  0  0  0  0
   -2.9656    0.5016    1.2575 H   0  0  0  0  0  0  0  0  0  0  0  0
   -0.0838    1.6487   -1.1698 H   0  0  0  0  0  0  0  0  0  0  0  0
   -0.0184    1.1088    1.5253 H   0  0  0  0  0  0  0  0  0  0  0  0
   -0.1759   -1.9521    1.0526 C   0  0  0  0  0  0  0  0  0  0  0  0
    0.2678   -1.6823   -0.2100 C   0  0  0  0  0  0  0  0  0  0  0  0
   -0.2207   -3.0075    1.4036 H   0  0  0  0  0  0  0  0  0  0  0  0
    0.4943   -2.5132   -0.9092 H   0  0  0  0  0  0  0  0  0  0  0  0
    1.2509    0.1937    0.1218 H   0  0  0  0  0  0  0  0  0  0  0  0
    0.7372   -0.8447    2.6271 H   0  0  0  0  0  0  0  0  0  0  0  0
   -4.1802   -2.1474   -0.6710 N   0  0  0  0  0  0  0  0  0  0  0  0
   -5.2966   -2.1706   -1.0201 C   0  0  0  0  0  0  0  0  0  0  0  0
   -6.6433   -2.2098   -1.4518 C   0  0  0  0  0  0  0  0  0  0  0  0
   -6.9355   -2.4272   -2.8185 C   0  0  0  0  0  0  0  0  0  0  0  0
   -7.6941   -2.0307   -0.5250 C   0  0  0  0  0  0  0  0  0  0  0  0
   -8.2561   -2.4675   -3.2407 C   0  0  0  0  0  0  0  0  0  0  0  0
   -9.0123   -2.0713   -0.9625 C   0  0  0  0  0  0  0  0  0  0  0  0
   -9.3138   -2.2854   -2.3226 C   0  0  0  0  0  0  0  0  0  0  0  0
   -0.7394   -5.3980   -1.2688 C   0  0  0  0  0  0  0  0  0  0  0  0
    0.1598   -5.7074   -0.2361 C   0  0  0  0  0  0  0  0  0  0  0  0
   -0.4417   -5.7081   -2.6006 C   0  0  0  0  0  0  0  0  0  0  0  0
    1.3799   -6.3116   -0.5338 C   0  0  0  0  0  0  0  0  0  0  0  0
   -0.0709   -5.5071    0.8150 H   0  0  0  0  0  0  0  0  0  0  0  0
    0.7782   -6.3116   -2.9179 C   0  0  0  0  0  0  0  0  0  0  0  0
   -1.1411   -5.5039   -3.4181 H   0  0  0  0  0  0  0  0  0  0  0  0
    1.6667   -6.6002   -1.8762 C   0  0  0  0  0  0  0  0  0  0  0  0
    2.0913   -6.5652    0.2576 H   0  0  0  0  0  0  0  0  0  0  0  0
    1.0282   -6.5604   -3.9537 H   0  0  0  0  0  0  0  0  0  0  0  0
   -3.0136   -5.6746    0.5874 C   0  0  0  0  0  0  0  0  0  0  0  0
   -2.8186   -7.0621    0.5697 C   0  0  0  0  0  0  0  0  0  0  0  0
   -3.6976   -5.0480    1.6363 C   0  0  0  0  0  0  0  0  0  0  0  0
   -3.3276   -7.8441    1.6081 C   0  0  0  0  0  0  0  0  0  0  0  0
   -2.2698   -7.5598   -0.2385 H   0  0  0  0  0  0  0  0  0  0  0  0
   -4.2166   -5.8140    2.6806 C   0  0  0  0  0  0  0  0  0  0  0  0
   -3.8365   -3.9545    1.6689 H   0  0  0  0  0  0  0  0  0  0  0  0
   -4.0217   -7.2024    2.6414 C   0  0  0  0  0  0  0  0  0  0  0  0
   -3.1847   -8.9291    1.6161 H   0  0  0  0  0  0  0  0  0  0  0  0
   -4.7572   -5.3490    3.5096 H   0  0  0  0  0  0  0  0  0  0  0  0
   -3.5238   -4.8801   -2.2414 C   0  0  0  0  0  0  0  0  0  0  0  0
   -4.5431   -5.8349   -2.1673 C   0  0  0  0  0  0  0  0  0  0  0  0
   -3.3460   -4.0947   -3.3903 C   0  0  0  0  0  0  0  0  0  0  0  0
   -5.4033   -6.0173   -3.2546 C   0  0  0  0  0  0  0  0  0  0  0  0
   -4.7010   -6.4511   -1.2740 H   0  0  0  0  0  0  0  0  0  0  0  0
   -4.1887   -4.2693   -4.4871 C   0  0  0  0  0  0  0  0  0  0  0  0
   -2.5601   -3.3323   -3.4587 H   0  0  0  0  0  0  0  0  0  0  0  0
   -5.2028   -5.2345   -4.3952 C   0  0  0  0  0  0  0  0  0  0  0  0
   -6.2055   -6.7601   -3.2141 H   0  0  0  0  0  0  0  0  0  0  0  0
   -4.0567   -3.6804   -5.3992 H   0  0  0  0  0  0  0  0  0  0  0  0
   -2.3181   -2.1318   -0.1386 Ir  0  0  0  0  0  0  0  0  0  0  0  0
   -8.4849   -2.6362   -4.2965 H   0  0  0  0  0  0  0  0  0  0  0  0
   -9.8233   -1.9316   -0.2426 H   0  0  0  0  0  0  0  0  0  0  0  0
```

```
   -6.1214   -2.5603   -3.5342 H   0  0  0  0  0  0  0  0  0  0  0  0
   -7.4689   -1.8594    0.5300 H   0  0  0  0  0  0  0  0  0  0  0  0
   -1.2432    0.8829    2.8219 H   0  0  0  0  0  0  0  0  0  0  0  0
   -0.9417   -1.4194    2.9974 H   0  0  0  0  0  0  0  0  0  0  0  0
   -0.6076    0.3448   -2.3116 H   0  0  0  0  0  0  0  0  0  0  0  0
    1.4346   -0.4674   -1.5440 H   0  0  0  0  0  0  0  0  0  0  0  0
  -10.7224   -2.3163   -2.7986 C   0  0  0  0  0  0  0  0  0  0  0  0
  -10.9394   -3.2888   -3.3095 H   0  0  0  0  0  0  0  0  0  0  0  0
  -11.4560   -2.1928   -1.9652 H   0  0  0  0  0  0  0  0  0  0  0  0
  -10.8963   -1.4900   -3.5361 H   0  0  0  0  0  0  0  0  0  0  0  0
    2.8272   -7.1739   -2.1670 F   0  0  0  0  0  0  0  0  0  0  0  0
   -4.5153   -7.9389    3.6285 F   0  0  0  0  0  0  0  0  0  0  0  0
   -6.0163   -5.3963   -5.4319 F   0  0  0  0  0  0  0  0  0  0  0  0
  1 26  1  0  0  0  0
  1 36  1  0  0  0  0
  1 46  1  0  0  0  0
  1 56  1  0  0  0  0
  2  3  1  0  0  0  0
  2  4  1  0  0  0  0
  2 10  1  0  0  0  0
  2 63  1  0  0  0  0
  3 13  1  0  0  0  0
  3 16  1  0  0  0  0
  3 64  1  0  0  0  0
  4  6  2  0  0  0  0
  4  8  1  0  0  0  0
  4 56  1  0  0  0  0
  5  6  1  0  0  0  0
  5  7  1  0  0  0  0
  5 11  1  0  0  0  0
  5 61  1  0  0  0  0
  6  9  1  0  0  0  0
  6 56  1  0  0  0  0
  7 12  1  0  0  0  0
  7 17  1  0  0  0  0
  7 62  1  0  0  0  0
 12 13  2  0  0  0  0
 12 14  1  0  0  0  0
 12 56  1  0  0  0  0
 13 15  1  0  0  0  0
 13 56  1  0  0  0  0
 18 19  3  0  0  0  0
 18 56  1  0  0  0  0
 19 20  1  0  0  0  0
 20 21  2  0  0  0  0
 20 22  1  0  0  0  0
 21 23  1  0  0  0  0
 21 59  1  0  0  0  0
 22 24  2  0  0  0  0
 22 60  1  0  0  0  0
 23 25  2  0  0  0  0
 23 57  1  0  0  0  0
 24 25  1  0  0  0  0
 24 58  1  0  0  0  0
 25 65  1  0  0  0  0
 26 27  2  0  0  0  0
 26 28  1  0  0  0  0
 27 29  1  0  0  0  0
 27 30  1  0  0  0  0
 28 31  2  0  0  0  0
 28 32  1  0  0  0  0
 29 33  2  0  0  0  0
 29 34  1  0  0  0  0
 31 33  1  0  0  0  0
 31 35  1  0  0  0  0
```

```
 33 69  1  0  0  0  0
 36 37  2  0  0  0  0
 36 38  1  0  0  0  0
 37 39  1  0  0  0  0
 37 40  1  0  0  0  0
 38 41  2  0  0  0  0
 38 42  1  0  0  0  0
 39 43  2  0  0  0  0
 39 44  1  0  0  0  0
 41 43  1  0  0  0  0
 41 45  1  0  0  0  0
 43 70  1  0  0  0  0
 46 47  2  0  0  0  0
 46 48  1  0  0  0  0
 47 49  1  0  0  0  0
 47 50  1  0  0  0  0
 48 51  2  0  0  0  0
 48 52  1  0  0  0  0
 49 53  2  0  0  0  0
 49 54  1  0  0  0  0
 51 53  1  0  0  0  0
 51 55  1  0  0  0  0
 53 71  1  0  0  0  0
 65 66  1  0  0  0  0
 65 67  1  0  0  0  0
 65 68  1  0  0  0  0
M  END
$$$$
mol078
Optimized Catalyst 078
Optimized Catalyst 078
 47 52  0  0  0  0  0  0  0  0999 V2000
   -0.8745    0.2720   -1.4925 C   0  0  0  0  0  0  0  0  0  0  0  0
    0.3491   -0.6110   -1.1700 C   0  0  0  0  0  0  0  0  0  0  0  0
   -2.1197    0.0086   -0.6762 C   0  0  0  0  0  0  0  0  0  0  0  0
   -0.9660   -0.0542    1.5981 C   0  0  0  0  0  0  0  0  0  0  0  0
   -2.1672   -0.1560    0.6871 C   0  0  0  0  0  0  0  0  0  0  0  0
   -0.1903   -1.3734    1.7957 C   0  0  0  0  0  0  0  0  0  0  0  0
   -3.0618    0.2047   -1.2374 H   0  0  0  0  0  0  0  0  0  0  0  0
   -3.1455   -0.1459    1.2145 H   0  0  0  0  0  0  0  0  0  0  0  0
   -1.1206    0.1576   -2.5841 H   0  0  0  0  0  0  0  0  0  0  0  0
   -0.5902    1.3528   -1.3315 H   0  0  0  0  0  0  0  0  0  0  0  0
    0.9756   -0.6918   -2.1016 H   0  0  0  0  0  0  0  0  0  0  0  0
   -0.5980   -1.8958    2.7046 H   0  0  0  0  0  0  0  0  0  0  0  0
   -1.3086    0.3034    2.6089 H   0  0  0  0  0  0  0  0  0  0  0  0
   -0.2780    0.7397    1.1975 H   0  0  0  0  0  0  0  0  0  0  0  0
   -0.2009   -2.3344    0.6287 C   0  0  0  0  0  0  0  0  0  0  0  0
    0.0158   -2.0036   -0.6871 C   0  0  0  0  0  0  0  0  0  0  0  0
   -0.1569   -3.4033    0.9399 H   0  0  0  0  0  0  0  0  0  0  0  0
    0.1839   -2.8025   -1.4408 H   0  0  0  0  0  0  0  0  0  0  0  0
    0.9929   -0.1082   -0.3976 H   0  0  0  0  0  0  0  0  0  0  0  0
    0.8892   -1.1262    2.0159 H   0  0  0  0  0  0  0  0  0  0  0  0
   -4.3137   -2.2707   -0.5935 N   0  0  0  0  0  0  0  0  0  0  0  0
   -5.4729   -2.2953   -0.7404 C   0  0  0  0  0  0  0  0  0  0  0  0
   -6.8817   -2.2972   -0.9173 C   0  0  0  0  0  0  0  0  0  0  0  0
   -7.4441   -1.6728   -2.0545 C   0  0  0  0  0  0  0  0  0  0  0  0
   -7.7098   -2.9205    0.0443 C   0  0  0  0  0  0  0  0  0  0  0  0
   -8.8233   -1.6693   -2.2326 C   0  0  0  0  0  0  0  0  0  0  0  0
   -6.7961   -1.1946   -2.7932 H   0  0  0  0  0  0  0  0  0  0  0  0
   -9.0899   -2.9219   -0.1263 C   0  0  0  0  0  0  0  0  0  0  0  0
   -7.2669   -3.3994    0.9210 H   0  0  0  0  0  0  0  0  0  0  0  0
   -9.6215   -2.2947   -1.2632 C   0  0  0  0  0  0  0  0  0  0  0  0
   -9.2758   -1.1905   -3.1081 H   0  0  0  0  0  0  0  0  0  0  0  0
   -9.7473   -3.3985    0.6086 H   0  0  0  0  0  0  0  0  0  0  0  0
   -2.3420   -4.2761   -0.6607 N   0  0  0  0  0  0  0  0  0  0  0  0
```

```
   -2.3890   -5.4282   -0.8520 C   0  0  0  0  0  0  0  0  0  0  0  0
   -2.4150   -6.8285   -1.0844 C   0  0  0  0  0  0  0  0  0  0  0  0
   -3.3042   -7.6471   -0.3509 C   0  0  0  0  0  0  0  0  0  0  0  0
   -1.5485   -7.3926   -2.0491 C   0  0  0  0  0  0  0  0  0  0  0  0
   -3.3292   -9.0190   -0.5764 C   0  0  0  0  0  0  0  0  0  0  0  0
   -3.9702   -7.2034    0.3931 H   0  0  0  0  0  0  0  0  0  0  0  0
   -1.5669   -8.7636   -2.2810 C   0  0  0  0  0  0  0  0  0  0  0  0
   -0.8658   -6.7519   -2.6125 H   0  0  0  0  0  0  0  0  0  0  0  0
   -2.4585   -9.5523   -1.5386 C   0  0  0  0  0  0  0  0  0  0  0  0
   -4.0101   -9.6692   -0.0171 H   0  0  0  0  0  0  0  0  0  0  0  0
   -0.9024   -9.2168   -3.0249 H   0  0  0  0  0  0  0  0  0  0  0  0
   -2.3632   -2.3341   -0.3619 Ir  0  0  0  0  0  0  0  0  0  0  0  0
   -2.4788  -10.8592   -1.7564 F   0  0  0  0  0  0  0  0  0  0  0  0
  -10.9362   -2.2934   -1.4291 F   0  0  0  0  0  0  0  0  0  0  0  0
  1  2  1  0  0  0  0
  1  3  1  0  0  0  0
  1  9  1  0  0  0  0
  1 10  1  0  0  0  0
  2 11  1  0  0  0  0
  2 16  1  0  0  0  0
  2 19  1  0  0  0  0
  3  5  2  0  0  0  0
  3  7  1  0  0  0  0
  3 45  1  0  0  0  0
  4  5  1  0  0  0  0
  4  6  1  0  0  0  0
  4 13  1  0  0  0  0
  4 14  1  0  0  0  0
  5  8  1  0  0  0  0
  5 45  1  0  0  0  0
  6 12  1  0  0  0  0
  6 15  1  0  0  0  0
  6 20  1  0  0  0  0
 15 16  2  0  0  0  0
 15 17  1  0  0  0  0
 15 45  1  0  0  0  0
 16 18  1  0  0  0  0
 16 45  1  0  0  0  0
 21 22  3  0  0  0  0
 21 45  1  0  0  0  0
 22 23  1  0  0  0  0
 23 24  2  0  0  0  0
 23 25  1  0  0  0  0
 24 26  1  0  0  0  0
 24 27  1  0  0  0  0
 25 28  2  0  0  0  0
 25 29  1  0  0  0  0
 26 30  2  0  0  0  0
 26 31  1  0  0  0  0
 28 30  1  0  0  0  0
 28 32  1  0  0  0  0
 30 47  1  0  0  0  0
 33 34  3  0  0  0  0
 33 45  1  0  0  0  0
 34 35  1  0  0  0  0
 35 36  2  0  0  0  0
 35 37  1  0  0  0  0
 36 38  1  0  0  0  0
 36 39  1  0  0  0  0
 37 40  2  0  0  0  0
 37 41  1  0  0  0  0
 38 42  2  0  0  0  0
 38 43  1  0  0  0  0
 40 42  1  0  0  0  0
 40 44  1  0  0  0  0
```

```
 42 46  1  0  0  0  0
M  END
$$$$
mol079
Optimized Catalyst 079
Optimized Catalyst 079
 53 58  0  0  0  0  0  0  0  0999 V2000
   -0.7445    0.2006   -1.5182 C   0  0  0  0  0  0  0  0  0  0  0  0
    0.4357   -0.6889   -1.0732 C   0  0  0  0  0  0  0  0  0  0  0  0
   -2.0502   -0.0101   -0.7846 C   0  0  0  0  0  0  0  0  0  0  0  0
   -1.0616    0.0005    1.5686 C   0  0  0  0  0  0  0  0  0  0  0  0
   -2.1973   -0.1229    0.5785 C   0  0  0  0  0  0  0  0  0  0  0  0
   -0.3233   -1.3197    1.8753 C   0  0  0  0  0  0  0  0  0  0  0  0
   -2.9470    0.1792   -1.4186 H   0  0  0  0  0  0  0  0  0  0  0  0
   -3.2095   -0.0704    1.0341 H   0  0  0  0  0  0  0  0  0  0  0  0
   -0.9147    0.0442   -2.6191 H   0  0  0  0  0  0  0  0  0  0  0  0
   -0.4523    1.2817   -1.3797 H   0  0  0  0  0  0  0  0  0  0  0  0
    1.1250   -0.8194   -1.9532 H   0  0  0  0  0  0  0  0  0  0  0  0
   -0.8043   -1.7974    2.7729 H   0  0  0  0  0  0  0  0  0  0  0  0
   -1.4693    0.4047    2.5363 H   0  0  0  0  0  0  0  0  0  0  0  0
   -0.3348    0.7671    1.1851 H   0  0  0  0  0  0  0  0  0  0  0  0
   -0.2719   -2.3278    0.7494 C   0  0  0  0  0  0  0  0  0  0  0  0
    0.0383   -2.0545   -0.5617 C   0  0  0  0  0  0  0  0  0  0  0  0
   -0.2725   -3.3832    1.1043 H   0  0  0  0  0  0  0  0  0  0  0  0
    0.2417   -2.8862   -1.2704 H   0  0  0  0  0  0  0  0  0  0  0  0
    1.0326   -0.1662   -0.2771 H   0  0  0  0  0  0  0  0  0  0  0  0
    0.7419   -1.0798    2.1613 H   0  0  0  0  0  0  0  0  0  0  0  0
   -4.2966   -2.2435   -0.6873 N   0  0  0  0  0  0  0  0  0  0  0  0
   -5.4566   -2.2776   -0.8277 C   0  0  0  0  0  0  0  0  0  0  0  0
   -6.8652   -2.2893   -0.9862 C   0  0  0  0  0  0  0  0  0  0  0  0
   -7.4407   -2.7025   -2.2075 C   0  0  0  0  0  0  0  0  0  0  0  0
   -7.6957   -1.8848    0.0837 C   0  0  0  0  0  0  0  0  0  0  0  0
   -8.8232   -2.7122   -2.3501 C   0  0  0  0  0  0  0  0  0  0  0  0
   -6.8003   -3.0112   -3.0360 H   0  0  0  0  0  0  0  0  0  0  0  0
   -9.0752   -1.8979   -0.0726 C   0  0  0  0  0  0  0  0  0  0  0  0
   -7.2518   -1.5642    1.0283 H   0  0  0  0  0  0  0  0  0  0  0  0
   -9.6596   -2.3118   -1.2887 C   0  0  0  0  0  0  0  0  0  0  0  0
   -9.2660   -3.0310   -3.2971 H   0  0  0  0  0  0  0  0  0  0  0  0
   -9.7178   -1.5826    0.7539 H   0  0  0  0  0  0  0  0  0  0  0  0
   -2.3414   -4.2622   -0.6598 N   0  0  0  0  0  0  0  0  0  0  0  0
   -2.3828   -5.4149   -0.8489 C   0  0  0  0  0  0  0  0  0  0  0  0
   -2.4034   -6.8135   -1.0763 C   0  0  0  0  0  0  0  0  0  0  0  0
   -3.2816   -7.3623   -2.0376 C   0  0  0  0  0  0  0  0  0  0  0  0
   -1.5435   -7.6592   -0.3417 C   0  0  0  0  0  0  0  0  0  0  0  0
   -3.2948   -8.7339   -2.2531 C   0  0  0  0  0  0  0  0  0  0  0  0
   -3.9435   -6.7082   -2.6079 H   0  0  0  0  0  0  0  0  0  0  0  0
   -1.5664   -9.0300   -0.5674 C   0  0  0  0  0  0  0  0  0  0  0  0
   -0.8635   -7.2353    0.3999 H   0  0  0  0  0  0  0  0  0  0  0  0
   -2.4399   -9.5871   -1.5231 C   0  0  0  0  0  0  0  0  0  0  0  0
   -3.9713   -9.1600   -2.9986 H   0  0  0  0  0  0  0  0  0  0  0  0
   -0.8974   -9.6813    0.0013 H   0  0  0  0  0  0  0  0  0  0  0  0
   -2.3527   -2.3154   -0.3871 Ir  0  0  0  0  0  0  0  0  0  0  0  0
  -11.1395   -2.3047   -1.4421 C   0  0  0  0  0  0  0  0  0  0  0  0
  -11.6371   -2.7471   -0.5429 H   0  0  0  0  0  0  0  0  0  0  0  0
  -11.4965   -1.2474   -1.5528 H   0  0  0  0  0  0  0  0  0  0  0  0
  -11.4757   -2.8798   -2.3385 H   0  0  0  0  0  0  0  0  0  0  0  0
   -2.4580  -11.0530   -1.7766 C   0  0  0  0  0  0  0  0  0  0  0  0
   -1.8861  -11.6218   -1.0040 H   0  0  0  0  0  0  0  0  0  0  0  0
   -3.5077  -11.4409   -1.7860 H   0  0  0  0  0  0  0  0  0  0  0  0
   -1.9978  -11.2684   -2.7758 H   0  0  0  0  0  0  0  0  0  0  0  0
  1  2  1  0  0  0  0
  1  3  1  0  0  0  0
  1  9  1  0  0  0  0
  1 10  1  0  0  0  0
  2 11  1  0  0  0  0
```

```
  2 16  1  0  0  0  0
  2 19  1  0  0  0  0
  3  5  2  0  0  0  0
  3  7  1  0  0  0  0
  3 45  1  0  0  0  0
  4  5  1  0  0  0  0
  4  6  1  0  0  0  0
  4 13  1  0  0  0  0
  4 14  1  0  0  0  0
  5  8  1  0  0  0  0
  5 45  1  0  0  0  0
  6 12  1  0  0  0  0
  6 15  1  0  0  0  0
  6 20  1  0  0  0  0
 15 16  2  0  0  0  0
 15 17  1  0  0  0  0
 15 45  1  0  0  0  0
 16 18  1  0  0  0  0
 16 45  1  0  0  0  0
 21 22  3  0  0  0  0
 21 45  1  0  0  0  0
 22 23  1  0  0  0  0
 23 24  2  0  0  0  0
 23 25  1  0  0  0  0
 24 26  1  0  0  0  0
 24 27  1  0  0  0  0
 25 28  2  0  0  0  0
 25 29  1  0  0  0  0
 26 30  2  0  0  0  0
 26 31  1  0  0  0  0
 28 30  1  0  0  0  0
 28 32  1  0  0  0  0
 30 46  1  0  0  0  0
 33 34  3  0  0  0  0
 33 45  1  0  0  0  0
 34 35  1  0  0  0  0
 35 36  2  0  0  0  0
 35 37  1  0  0  0  0
 36 38  1  0  0  0  0
 36 39  1  0  0  0  0
 37 40  2  0  0  0  0
 37 41  1  0  0  0  0
 38 42  2  0  0  0  0
 38 43  1  0  0  0  0
 40 42  1  0  0  0  0
 40 44  1  0  0  0  0
 42 50  1  0  0  0  0
 46 47  1  0  0  0  0
 46 48  1  0  0  0  0
 46 49  1  0  0  0  0
 50 51  1  0  0  0  0
 50 52  1  0  0  0  0
 50 53  1  0  0  0  0
M  END
$$$$
mol080
Optimized Catalyst 080
Optimized Catalyst 080
 47 52  0  0  0  0  0  0  0  0999 V2000
   -0.8573    0.2617   -1.4362 C   0  0  2  0  0  0  0  0  0  0  0  0
    0.3062   -0.7528   -1.2261 C   0  0  1  0  0  0  0  0  0  0  0  0
   -2.1502   -0.1613   -0.7584 C   0  0  0  0  0  0  0  0  0  0  0  0
   -1.1178   -0.1026    1.6009 C   0  0  2  0  0  0  0  0  0  0  0  0
   -2.2712   -0.3389    0.6371 C   0  0  0  0  0  0  0  0  0  0  0  0
   -0.1876   -1.3436    1.7476 C   0  0  2  0  0  0  0  0  0  0  0  0
```

```
   -3.0559    0.1199   -1.3451 H   0  0  0  0  0  0  0  0  0  0  0  0
   -3.2712   -0.2128    1.1130 H   0  0  0  0  0  0  0  0  0  0  0  0
   -0.5657    1.3056   -1.1433 H   0  0  0  0  0  0  0  0  0  0  0  0
   -0.5440    0.8230    1.3350 H   0  0  0  0  0  0  0  0  0  0  0  0
   -0.3885   -2.3758    0.6503 C   0  0  0  0  0  0  0  0  0  0  0  0
   -0.1723   -2.1050   -0.7187 C   0  0  0  0  0  0  0  0  0  0  0  0
   -0.2671   -3.4221    1.0166 H   0  0  0  0  0  0  0  0  0  0  0  0
    0.0940   -2.9461   -1.3999 H   0  0  0  0  0  0  0  0  0  0  0  0
    1.1164   -0.3335   -0.5748 H   0  0  0  0  0  0  0  0  0  0  0  0
    0.8929   -1.0529    1.8412 H   0  0  0  0  0  0  0  0  0  0  0  0
   -4.2381   -2.2047   -0.5588 N   0  0  0  0  0  0  0  0  0  0  0  0
   -5.3979   -2.2239   -0.7031 C   0  0  0  0  0  0  0  0  0  0  0  0
   -6.8092   -2.2465   -0.8832 C   0  0  0  0  0  0  0  0  0  0  0  0
   -7.3457   -2.1543   -2.1844 C   0  0  0  0  0  0  0  0  0  0  0  0
   -7.6587   -2.3612    0.2363 C   0  0  0  0  0  0  0  0  0  0  0  0
   -8.7290   -2.1845   -2.3564 C   0  0  0  0  0  0  0  0  0  0  0  0
   -6.6857   -2.0584   -3.0480 H   0  0  0  0  0  0  0  0  0  0  0  0
   -9.0399   -2.3901    0.0451 C   0  0  0  0  0  0  0  0  0  0  0  0
   -7.2408   -2.4254    1.2424 H   0  0  0  0  0  0  0  0  0  0  0  0
   -9.5752   -2.3034   -1.2464 C   0  0  0  0  0  0  0  0  0  0  0  0
   -9.1552   -2.1140   -3.3619 H   0  0  0  0  0  0  0  0  0  0  0  0
   -9.7081   -2.4799    0.9072 H   0  0  0  0  0  0  0  0  0  0  0  0
   -2.2684   -4.1984   -0.6576 N   0  0  0  0  0  0  0  0  0  0  0  0
   -2.3095   -5.3509   -0.8486 C   0  0  0  0  0  0  0  0  0  0  0  0
   -2.3596   -6.7541   -1.0791 C   0  0  0  0  0  0  0  0  0  0  0  0
   -2.5404   -7.6330    0.0089 C   0  0  0  0  0  0  0  0  0  0  0  0
   -2.2289   -7.2541   -2.3910 C   0  0  0  0  0  0  0  0  0  0  0  0
   -2.5963   -9.0066   -0.2246 C   0  0  0  0  0  0  0  0  0  0  0  0
   -2.6342   -7.2429    1.0237 H   0  0  0  0  0  0  0  0  0  0  0  0
   -2.2876   -8.6310   -2.6057 C   0  0  0  0  0  0  0  0  0  0  0  0
   -2.0819   -6.5722   -3.2301 H   0  0  0  0  0  0  0  0  0  0  0  0
   -2.4719   -9.5059   -1.5275 C   0  0  0  0  0  0  0  0  0  0  0  0
   -2.7370   -9.6970    0.6127 H   0  0  0  0  0  0  0  0  0  0  0  0
   -2.1884   -9.0293   -3.6201 H   0  0  0  0  0  0  0  0  0  0  0  0
   -2.2643   -2.2379   -0.3326 Ir  0  0  0  0  0  0  0  0  0  0  0  0
  -10.6608   -2.3278   -1.3891 H   0  0  0  0  0  0  0  0  0  0  0  0
   -2.5179  -10.5858   -1.7037 H   0  0  0  0  0  0  0  0  0  0  0  0
   -1.6393    0.1261    2.8448 F   0  0  0  0  0  0  0  0  0  0  0  0
   -0.5270   -1.9295    2.9385 F   0  0  0  0  0  0  0  0  0  0  0  0
   -1.0859    0.2969   -2.7865 F   0  0  0  0  0  0  0  0  0  0  0  0
    0.8735   -0.9340   -2.4578 F   0  0  0  0  0  0  0  0  0  0  0  0
  1  2  1  0  0  0  0
  1  3  1  0  0  0  0
  1  9  1  1  0  0  0
  1 46  1  0  0  0  0
  2 12  1  0  0  0  0
  2 15  1  1  0  0  0
  2 47  1  0  0  0  0
  3  5  2  0  0  0  0
  3  7  1  0  0  0  0
  3 41  1  0  0  0  0
  4  5  1  0  0  0  0
  4  6  1  0  0  0  0
  4 10  1  6  0  0  0
  4 44  1  0  0  0  0
  5  8  1  0  0  0  0
  5 41  1  0  0  0  0
  6 11  1  0  0  0  0
  6 16  1  1  0  0  0
  6 45  1  0  0  0  0
 11 12  2  0  0  0  0
 11 13  1  0  0  0  0
 11 41  1  0  0  0  0
 12 14  1  0  0  0  0
 12 41  1  0  0  0  0
```

```
 17 18  3  0  0  0  0
 17 41  1  0  0  0  0
 18 19  1  0  0  0  0
 19 20  2  0  0  0  0
 19 21  1  0  0  0  0
 20 22  1  0  0  0  0
 20 23  1  0  0  0  0
 21 24  2  0  0  0  0
 21 25  1  0  0  0  0
 22 26  2  0  0  0  0
 22 27  1  0  0  0  0
 24 26  1  0  0  0  0
 24 28  1  0  0  0  0
 26 42  1  0  0  0  0
 29 30  3  0  0  0  0
 29 41  1  0  0  0  0
 30 31  1  0  0  0  0
 31 32  2  0  0  0  0
 31 33  1  0  0  0  0
 32 34  1  0  0  0  0
 32 35  1  0  0  0  0
 33 36  2  0  0  0  0
 33 37  1  0  0  0  0
 34 38  2  0  0  0  0
 34 39  1  0  0  0  0
 36 38  1  0  0  0  0
 36 40  1  0  0  0  0
 38 43  1  0  0  0  0
M  END
$$$$
mol081
Optimized Catalyst 081
Optimized Catalyst 081
 59 63  0  0  0  0  0  0  0  0999 V2000
   -0.9769    0.2342   -1.5885 C   0  0  2  0  0  0  0  0  0  0  0  0
    0.3989   -0.3738   -1.1808 C   0  0  1  0  0  0  0  0  0  0  0  0
   -2.0618    0.1659   -0.5228 C   0  0  0  0  0  0  0  0  0  0  0  0
   -0.7418   -0.0264    1.6882 C   0  0  2  0  0  0  0  0  0  0  0  0
   -1.9826    0.0605    0.8258 C   0  0  0  0  0  0  0  0  0  0  0  0
   -0.2531   -1.4975    1.8486 C   0  0  2  0  0  0  0  0  0  0  0  0
   -3.0641    0.3973   -0.9537 H   0  0  0  0  0  0  0  0  0  0  0  0
   -2.9189    0.1465    1.4141 H   0  0  0  0  0  0  0  0  0  0  0  0
   -0.7998    1.3310   -1.7911 H   0  0  0  0  0  0  0  0  0  0  0  0
    0.0841    0.5678    1.2114 H   0  0  0  0  0  0  0  0  0  0  0  0
   -0.0090   -2.2488    0.5474 C   0  0  0  0  0  0  0  0  0  0  0  0
    0.2605   -1.8036   -0.7038 C   0  0  0  0  0  0  0  0  0  0  0  0
    0.1088   -3.3426    0.7306 H   0  0  0  0  0  0  0  0  0  0  0  0
    0.5181   -2.5508   -1.4819 H   0  0  0  0  0  0  0  0  0  0  0  0
    0.8288    0.2535   -0.3536 H   0  0  0  0  0  0  0  0  0  0  0  0
    0.7418   -1.4529    2.3809 H   0  0  0  0  0  0  0  0  0  0  0  0
   -4.3413   -2.3080   -0.6123 N   0  0  0  0  0  0  0  0  0  0  0  0
   -5.5002   -2.3274   -0.7581 C   0  0  0  0  0  0  0  0  0  0  0  0
   -6.9129   -2.3212   -0.9309 C   0  0  0  0  0  0  0  0  0  0  0  0
   -7.4564   -2.2112   -2.2272 C   0  0  0  0  0  0  0  0  0  0  0  0
   -7.7561   -2.4221    0.1947 C   0  0  0  0  0  0  0  0  0  0  0  0
   -8.8416   -2.2001   -2.3879 C   0  0  0  0  0  0  0  0  0  0  0  0
   -6.7999   -2.1352   -3.0953 H   0  0  0  0  0  0  0  0  0  0  0  0
   -9.1391   -2.4092    0.0148 C   0  0  0  0  0  0  0  0  0  0  0  0
   -7.3312   -2.5090    1.1959 H   0  0  0  0  0  0  0  0  0  0  0  0
   -9.6819   -2.2965   -1.2714 C   0  0  0  0  0  0  0  0  0  0  0  0
   -9.2734   -2.1148   -3.3893 H   0  0  0  0  0  0  0  0  0  0  0  0
   -9.8022   -2.4864    0.8814 H   0  0  0  0  0  0  0  0  0  0  0  0
   -2.3815   -4.3060   -0.6585 N   0  0  0  0  0  0  0  0  0  0  0  0
   -2.4235   -5.4580   -0.8484 C   0  0  0  0  0  0  0  0  0  0  0  0
   -2.4436   -6.8619   -1.0815 C   0  0  0  0  0  0  0  0  0  0  0  0
```

```
   -2.6080   -7.7488    0.0020 C   0  0  0  0  0  0  0  0  0  0  0  0
   -2.2959   -7.3529   -2.3950 C   0  0  0  0  0  0  0  0  0  0  0  0
   -2.6204   -9.1230   -0.2365 C   0  0  0  0  0  0  0  0  0  0  0  0
   -2.7240   -7.3640    1.0163 H   0  0  0  0  0  0  0  0  0  0  0  0
   -2.3108   -8.7301   -2.6146 C   0  0  0  0  0  0  0  0  0  0  0  0
   -2.1707   -6.6629   -3.2309 H   0  0  0  0  0  0  0  0  0  0  0  0
   -2.4702   -9.6138   -1.5397 C   0  0  0  0  0  0  0  0  0  0  0  0
   -2.7468   -9.8198    0.5972 H   0  0  0  0  0  0  0  0  0  0  0  0
   -2.1967   -9.1214   -3.6297 H   0  0  0  0  0  0  0  0  0  0  0  0
   -2.4112   -2.3810   -0.3713 Ir  0  0  0  0  0  0  0  0  0  0  0  0
  -10.7685   -2.2833   -1.4048 H   0  0  0  0  0  0  0  0  0  0  0  0
   -2.4771  -10.6939   -1.7190 H   0  0  0  0  0  0  0  0  0  0  0  0
   -1.5408   -0.4144   -2.8702 C   0  0  0  0  0  0  0  0  0  0  0  0
   -2.4205    0.1600   -3.2471 H   0  0  0  0  0  0  0  0  0  0  0  0
   -0.7782   -0.4042   -3.6849 H   0  0  0  0  0  0  0  0  0  0  0  0
   -1.8473   -1.4895   -2.6757 H   0  0  0  0  0  0  0  0  0  0  0  0
    1.4354   -0.2919   -2.3136 C   0  0  0  0  0  0  0  0  0  0  0  0
    1.4784    0.7458   -2.7218 H   0  0  0  0  0  0  0  0  0  0  0  0
    2.4468   -0.5461   -1.9125 H   0  0  0  0  0  0  0  0  0  0  0  0
    1.2095   -0.9914   -3.1479 H   0  0  0  0  0  0  0  0  0  0  0  0
   -1.2250   -2.3419    2.6994 C   0  0  0  0  0  0  0  0  0  0  0  0
   -0.7650   -3.3242    2.9628 H   0  0  0  0  0  0  0  0  0  0  0  0
   -1.4545   -1.8268    3.6624 H   0  0  0  0  0  0  0  0  0  0  0  0
   -2.1985   -2.5180    2.1434 H   0  0  0  0  0  0  0  0  0  0  0  0
   -0.9885    0.6611    3.0411 C   0  0  0  0  0  0  0  0  0  0  0  0
   -0.1050    0.5253    3.7089 H   0  0  0  0  0  0  0  0  0  0  0  0
   -1.1336    1.7574    2.8811 H   0  0  0  0  0  0  0  0  0  0  0  0
   -1.8890    0.2666    3.5604 H   0  0  0  0  0  0  0  0  0  0  0  0
  1  2  1  0  0  0  0
  1  3  1  0  0  0  0
  1  9  1  6  0  0  0
  1 44  1  0  0  0  0
  2 12  1  0  0  0  0
  2 15  1  1  0  0  0
  2 48  1  0  0  0  0
  3  5  2  0  0  0  0
  3  7  1  0  0  0  0
  3 41  1  0  0  0  0
  4  5  1  0  0  0  0
  4  6  1  0  0  0  0
  4 10  1  6  0  0  0
  4 56  1  0  0  0  0
  5  8  1  0  0  0  0
  5 41  1  0  0  0  0
  6 11  1  0  0  0  0
  6 16  1  1  0  0  0
  6 52  1  0  0  0  0
 11 12  2  0  0  0  0
 11 13  1  0  0  0  0
 11 41  1  0  0  0  0
 12 14  1  0  0  0  0
 12 41  1  0  0  0  0
 17 18  3  0  0  0  0
 17 41  1  0  0  0  0
 18 19  1  0  0  0  0
 19 20  2  0  0  0  0
 19 21  1  0  0  0  0
 20 22  1  0  0  0  0
 20 23  1  0  0  0  0
 21 24  2  0  0  0  0
 21 25  1  0  0  0  0
 22 26  2  0  0  0  0
 22 27  1  0  0  0  0
 24 26  1  0  0  0  0
 24 28  1  0  0  0  0
```

```
 26 42  1  0  0  0  0
 29 30  3  0  0  0  0
 29 41  1  0  0  0  0
 30 31  1  0  0  0  0
 31 32  2  0  0  0  0
 31 33  1  0  0  0  0
 32 34  1  0  0  0  0
 32 35  1  0  0  0  0
 33 36  2  0  0  0  0
 33 37  1  0  0  0  0
 34 38  2  0  0  0  0
 34 39  1  0  0  0  0
 36 38  1  0  0  0  0
 36 40  1  0  0  0  0
 38 43  1  0  0  0  0
 44 45  1  0  0  0  0
 44 46  1  0  0  0  0
 48 49  1  0  0  0  0
 48 50  1  0  0  0  0
 48 51  1  0  0  0  0
 52 53  1  0  0  0  0
 52 54  1  0  0  0  0
 52 55  1  0  0  0  0
 56 57  1  0  0  0  0
 56 58  1  0  0  0  0
 56 59  1  0  0  0  0
M  END
$$$$
mol082
Optimized Catalyst 082
Optimized Catalyst 082
 47 52  0  0  0  0  0  0  0  0999 V2000
   -0.9705    0.3461   -1.5686 C   0  0  0  0  0  0  0  0  0  0  0  0
    0.2032   -0.5763   -1.1797 C   0  0  0  0  0  0  0  0  0  0  0  0
   -2.2226    0.1261   -0.7530 C   0  0  0  0  0  0  0  0  0  0  0  0
   -1.0987    0.1614    1.5512 C   0  0  0  0  0  0  0  0  0  0  0  0
   -2.2877    0.0178    0.6299 C   0  0  0  0  0  0  0  0  0  0  0  0
   -0.4205   -1.1939    1.8412 C   0  0  0  0  0  0  0  0  0  0  0  0
   -3.1584    0.2549   -1.3306 H   0  0  0  0  0  0  0  0  0  0  0  0
   -3.2707    0.0133    1.1357 H   0  0  0  0  0  0  0  0  0  0  0  0
   -1.2171    0.1663   -2.6487 H   0  0  0  0  0  0  0  0  0  0  0  0
   -0.6572    1.4188   -1.4570 H   0  0  0  0  0  0  0  0  0  0  0  0
    0.8393   -0.7501   -2.0875 H   0  0  0  0  0  0  0  0  0  0  0  0
   -0.9258   -1.6549    2.7324 H   0  0  0  0  0  0  0  0  0  0  0  0
   -1.4397    0.6061    2.5232 H   0  0  0  0  0  0  0  0  0  0  0  0
   -0.3641    0.8779    1.1015 H   0  0  0  0  0  0  0  0  0  0  0  0
   -0.4957   -2.1840    0.6997 C   0  0  0  0  0  0  0  0  0  0  0  0
   -0.2641   -1.9111   -0.6462 C   0  0  0  0  0  0  0  0  0  0  0  0
   -0.5938   -3.2457    1.0262 H   0  0  0  0  0  0  0  0  0  0  0  0
   -0.2301   -2.7497   -1.3768 H   0  0  0  0  0  0  0  0  0  0  0  0
    0.8521   -0.0792   -0.4137 H   0  0  0  0  0  0  0  0  0  0  0  0
    0.6593   -1.0266    2.0976 H   0  0  0  0  0  0  0  0  0  0  0  0
   -3.9582   -2.0307   -0.4758 N   0  0  0  0  0  0  0  0  0  0  0  0
   -5.0978   -2.2018   -0.6399 C   0  0  0  0  0  0  0  0  0  0  0  0
   -6.5006   -2.3614   -0.8400 C   0  0  0  0  0  0  0  0  0  0  0  0
   -7.0035   -2.5215   -2.1481 C   0  0  0  0  0  0  0  0  0  0  0  0
   -7.3703   -2.3530    0.2704 C   0  0  0  0  0  0  0  0  0  0  0  0
   -8.3735   -2.6712   -2.3509 C   0  0  0  0  0  0  0  0  0  0  0  0
   -6.3209   -2.5279   -3.0003 H   0  0  0  0  0  0  0  0  0  0  0  0
   -8.7419   -2.5040    0.0786 C   0  0  0  0  0  0  0  0  0  0  0  0
   -6.9701   -2.2296    1.2789 H   0  0  0  0  0  0  0  0  0  0  0  0
   -9.2156   -2.6602   -1.2308 C   0  0  0  0  0  0  0  0  0  0  0  0
   -8.7812   -2.7931   -3.3584 H   0  0  0  0  0  0  0  0  0  0  0  0
   -9.4312   -2.4998    0.9281 H   0  0  0  0  0  0  0  0  0  0  0  0
   -2.3278   -4.4264   -0.6595 N   0  0  0  0  0  0  0  0  0  0  0  0
```

```
   -2.4326   -5.5614   -0.8737 C   0  0  0  0  0  0  0  0  0  0  0  0
   -2.5553   -6.9599   -1.1367 C   0  0  0  0  0  0  0  0  0  0  0  0
   -2.7958   -7.8521   -0.0719 C   0  0  0  0  0  0  0  0  0  0  0  0
   -2.4322   -7.4337   -2.4588 C   0  0  0  0  0  0  0  0  0  0  0  0
   -2.9158   -9.2168   -0.3226 C   0  0  0  0  0  0  0  0  0  0  0  0
   -2.8889   -7.4735    0.9485 H   0  0  0  0  0  0  0  0  0  0  0  0
   -2.5515   -8.7959   -2.7224 C   0  0  0  0  0  0  0  0  0  0  0  0
   -2.2439   -6.7332   -3.2754 H   0  0  0  0  0  0  0  0  0  0  0  0
   -2.7922   -9.6602   -1.6460 C   0  0  0  0  0  0  0  0  0  0  0  0
   -3.1005   -9.9234    0.4914 H   0  0  0  0  0  0  0  0  0  0  0  0
   -2.4590   -9.1807   -3.7417 H   0  0  0  0  0  0  0  0  0  0  0  0
   -2.3005   -1.9879   -0.2466 Rh  0  0  0  0  0  0  0  0  0  0  0  0
  -10.5223   -2.8049   -1.4190 F   0  0  0  0  0  0  0  0  0  0  0  0
   -2.9087  -10.9598   -1.8916 F   0  0  0  0  0  0  0  0  0  0  0  0
  1  2  1  0  0  0  0
  1  3  1  0  0  0  0
  1  9  1  0  0  0  0
  1 10  1  0  0  0  0
  2 11  1  0  0  0  0
  2 16  1  0  0  0  0
  2 19  1  0  0  0  0
  3  5  2  0  0  0  0
  3  7  1  0  0  0  0
  3 45  1  0  0  0  0
  4  5  1  0  0  0  0
  4  6  1  0  0  0  0
  4 13  1  0  0  0  0
  4 14  1  0  0  0  0
  5  8  1  0  0  0  0
  5 45  1  0  0  0  0
  6 12  1  0  0  0  0
  6 15  1  0  0  0  0
  6 20  1  0  0  0  0
 15 16  2  0  0  0  0
 15 17  1  0  0  0  0
 15 45  1  0  0  0  0
 16 18  1  0  0  0  0
 16 45  1  0  0  0  0
 21 22  3  0  0  0  0
 21 45  1  0  0  0  0
 22 23  1  0  0  0  0
 23 24  2  0  0  0  0
 23 25  1  0  0  0  0
 24 26  1  0  0  0  0
 24 27  1  0  0  0  0
 25 28  2  0  0  0  0
 25 29  1  0  0  0  0
 26 30  2  0  0  0  0
 26 31  1  0  0  0  0
 28 30  1  0  0  0  0
 28 32  1  0  0  0  0
 30 46  1  0  0  0  0
 33 34  3  0  0  0  0
 33 45  1  0  0  0  0
 34 35  1  0  0  0  0
 35 36  2  0  0  0  0
 35 37  1  0  0  0  0
 36 38  1  0  0  0  0
 36 39  1  0  0  0  0
 37 40  2  0  0  0  0
 37 41  1  0  0  0  0
 38 42  2  0  0  0  0
 38 43  1  0  0  0  0
 40 42  1  0  0  0  0
 40 44  1  0  0  0  0
```

```
 42 47  1  0  0  0  0
M  END
$$$$
mol083
Optimized Catalyst 083
Optimized Catalyst 083
 53 58  0  0  0  0  0  0  0  0999 V2000
   -0.9704    0.3462   -1.5684 C   0  0  0  0  0  0  0  0  0  0  0  0
    0.2022   -0.5781   -1.1792 C   0  0  0  0  0  0  0  0  0  0  0  0
   -2.2227    0.1263   -0.7530 C   0  0  0  0  0  0  0  0  0  0  0  0
   -1.0988    0.1613    1.5512 C   0  0  0  0  0  0  0  0  0  0  0  0
   -2.2877    0.0179    0.6295 C   0  0  0  0  0  0  0  0  0  0  0  0
   -0.4216   -1.1947    1.8415 C   0  0  0  0  0  0  0  0  0  0  0  0
   -3.1585    0.2509   -1.3316 H   0  0  0  0  0  0  0  0  0  0  0  0
   -3.2704    0.0091    1.1354 H   0  0  0  0  0  0  0  0  0  0  0  0
   -1.2176    0.1657   -2.6483 H   0  0  0  0  0  0  0  0  0  0  0  0
   -0.6566    1.4183   -1.4568 H   0  0  0  0  0  0  0  0  0  0  0  0
    0.8377   -0.7527   -2.0868 H   0  0  0  0  0  0  0  0  0  0  0  0
   -0.9282   -1.6549    2.7325 H   0  0  0  0  0  0  0  0  0  0  0  0
   -1.4399    0.6061    2.5228 H   0  0  0  0  0  0  0  0  0  0  0  0
   -0.3638    0.8766    1.1012 H   0  0  0  0  0  0  0  0  0  0  0  0
   -0.4982   -2.1857    0.7007 C   0  0  0  0  0  0  0  0  0  0  0  0
   -0.2670   -1.9127   -0.6461 C   0  0  0  0  0  0  0  0  0  0  0  0
   -0.5963   -3.2474    1.0279 H   0  0  0  0  0  0  0  0  0  0  0  0
   -0.2343   -2.7512   -1.3770 H   0  0  0  0  0  0  0  0  0  0  0  0
    0.8505   -0.0819   -0.4125 H   0  0  0  0  0  0  0  0  0  0  0  0
    0.6581   -1.0286    2.0975 H   0  0  0  0  0  0  0  0  0  0  0  0
   -3.9592   -2.0298   -0.4813 N   0  0  0  0  0  0  0  0  0  0  0  0
   -5.0993   -2.2033   -0.6386 C   0  0  0  0  0  0  0  0  0  0  0  0
   -6.5019   -2.3607   -0.8318 C   0  0  0  0  0  0  0  0  0  0  0  0
   -6.9835   -3.1702   -1.8784 C   0  0  0  0  0  0  0  0  0  0  0  0
   -7.4058   -1.7013    0.0265 C   0  0  0  0  0  0  0  0  0  0  0  0
   -8.3566   -3.3154   -2.0625 C   0  0  0  0  0  0  0  0  0  0  0  0
   -6.2829   -3.6797   -2.5414 H   0  0  0  0  0  0  0  0  0  0  0  0
   -8.7741   -1.8554   -0.1660 C   0  0  0  0  0  0  0  0  0  0  0  0
   -7.0312   -1.0759    0.8379 H   0  0  0  0  0  0  0  0  0  0  0  0
   -9.2670   -2.6611   -1.2126 C   0  0  0  0  0  0  0  0  0  0  0  0
   -8.7265   -3.9445   -2.8743 H   0  0  0  0  0  0  0  0  0  0  0  0
   -9.4754   -1.3488    0.5006 H   0  0  0  0  0  0  0  0  0  0  0  0
   -2.3408   -4.4252   -0.6232 N   0  0  0  0  0  0  0  0  0  0  0  0
   -2.4434   -5.5583   -0.8502 C   0  0  0  0  0  0  0  0  0  0  0  0
   -2.5552   -6.9527   -1.1347 C   0  0  0  0  0  0  0  0  0  0  0  0
   -3.5018   -7.7415   -0.4542 C   0  0  0  0  0  0  0  0  0  0  0  0
   -1.7118   -7.5394   -2.0994 C   0  0  0  0  0  0  0  0  0  0  0  0
   -3.6034   -9.1008   -0.7397 C   0  0  0  0  0  0  0  0  0  0  0  0
   -4.1527   -7.2876    0.2949 H   0  0  0  0  0  0  0  0  0  0  0  0
   -1.8204   -8.8977   -2.3773 C   0  0  0  0  0  0  0  0  0  0  0  0
   -0.9767   -6.9280   -2.6253 H   0  0  0  0  0  0  0  0  0  0  0  0
   -2.7674   -9.6946   -1.7033 C   0  0  0  0  0  0  0  0  0  0  0  0
   -4.3377   -9.7091   -0.2086 H   0  0  0  0  0  0  0  0  0  0  0  0
   -1.1659   -9.3525   -3.1236 H   0  0  0  0  0  0  0  0  0  0  0  0
   -2.2986   -1.9889   -0.2477 Rh  0  0  0  0  0  0  0  0  0  0  0  0
   -2.8666  -11.1498   -2.0113 C   0  0  0  0  0  0  0  0  0  0  0  0
   -1.9559  -11.6775   -1.6290 H   0  0  0  0  0  0  0  0  0  0  0  0
   -3.7639  -11.6172   -1.5407 H   0  0  0  0  0  0  0  0  0  0  0  0
   -2.9294  -11.3130   -3.1156 H   0  0  0  0  0  0  0  0  0  0  0  0
  -10.7381   -2.8120   -1.3996 C   0  0  0  0  0  0  0  0  0  0  0  0
  -11.1882   -3.2969   -0.4971 H   0  0  0  0  0  0  0  0  0  0  0  0
  -11.2149   -1.8086   -1.5341 H   0  0  0  0  0  0  0  0  0  0  0  0
  -10.9866   -3.4362   -2.2903 H   0  0  0  0  0  0  0  0  0  0  0  0
  1  2  1  0  0  0  0
  1  3  1  0  0  0  0
  1  9  1  0  0  0  0
  1 10  1  0  0  0  0
  2 11  1  0  0  0  0
```

```
  2 16  1  0  0  0  0
  2 19  1  0  0  0  0
  3  5  2  0  0  0  0
  3  7  1  0  0  0  0
  3 45  1  0  0  0  0
  4  5  1  0  0  0  0
  4  6  1  0  0  0  0
  4 13  1  0  0  0  0
  4 14  1  0  0  0  0
  5  8  1  0  0  0  0
  5 45  1  0  0  0  0
  6 12  1  0  0  0  0
  6 15  1  0  0  0  0
  6 20  1  0  0  0  0
 15 16  2  0  0  0  0
 15 17  1  0  0  0  0
 15 45  1  0  0  0  0
 16 18  1  0  0  0  0
 16 45  1  0  0  0  0
 21 22  3  0  0  0  0
 21 45  1  0  0  0  0
 22 23  1  0  0  0  0
 23 24  2  0  0  0  0
 23 25  1  0  0  0  0
 24 26  1  0  0  0  0
 24 27  1  0  0  0  0
 25 28  2  0  0  0  0
 25 29  1  0  0  0  0
 26 30  2  0  0  0  0
 26 31  1  0  0  0  0
 28 30  1  0  0  0  0
 28 32  1  0  0  0  0
 30 50  1  0  0  0  0
 33 34  3  0  0  0  0
 33 45  1  0  0  0  0
 34 35  1  0  0  0  0
 35 36  2  0  0  0  0
 35 37  1  0  0  0  0
 36 38  1  0  0  0  0
 36 39  1  0  0  0  0
 37 40  2  0  0  0  0
 37 41  1  0  0  0  0
 38 42  2  0  0  0  0
 38 43  1  0  0  0  0
 40 42  1  0  0  0  0
 40 44  1  0  0  0  0
 42 46  1  0  0  0  0
 46 47  1  0  0  0  0
 46 48  1  0  0  0  0
 46 49  1  0  0  0  0
 50 51  1  0  0  0  0
 50 52  1  0  0  0  0
 50 53  1  0  0  0  0
M  END
$$$$
mol084
Optimized Catalyst 084
Optimized Catalyst 084
 47 52  0  0  0  0  0  0  0  0999 V2000
   -0.9093    0.3819   -1.4917 C   0  0  2  0  0  0  0  0  0  0  0  0
    0.2169   -0.6469   -1.1889 C   0  0  1  0  0  0  0  0  0  0  0  0
   -2.2099    0.0765   -0.7778 C   0  0  0  0  0  0  0  0  0  0  0  0
   -1.1685    0.1718    1.5604 C   0  0  2  0  0  0  0  0  0  0  0  0
   -2.3322   -0.0329    0.6086 C   0  0  0  0  0  0  0  0  0  0  0  0
   -0.3372   -1.1216    1.7981 C   0  0  2  0  0  0  0  0  0  0  0  0
```

```
   -3.1140    0.2069   -1.4070 H   0  0  0  0  0  0  0  0  0  0  0  0
   -3.3324   -0.0101    1.0856 H   0  0  0  0  0  0  0  0  0  0  0  0
   -0.5768    1.4338   -1.2849 H   0  0  0  0  0  0  0  0  0  0  0  0
   -0.5184    1.0216    1.2285 H   0  0  0  0  0  0  0  0  0  0  0  0
   -0.5456   -2.1745    0.7257 C   0  0  0  0  0  0  0  0  0  0  0  0
   -0.3079   -1.9596   -0.6346 C   0  0  0  0  0  0  0  0  0  0  0  0
   -0.6768   -3.2150    1.1092 H   0  0  0  0  0  0  0  0  0  0  0  0
   -0.2569   -2.8253   -1.3355 H   0  0  0  0  0  0  0  0  0  0  0  0
    1.0038   -0.2101   -0.5223 H   0  0  0  0  0  0  0  0  0  0  0  0
    0.7542   -0.8945    1.9274 H   0  0  0  0  0  0  0  0  0  0  0  0
   -3.9665   -2.0134   -0.4853 N   0  0  0  0  0  0  0  0  0  0  0  0
   -5.1071   -2.1858   -0.6450 C   0  0  0  0  0  0  0  0  0  0  0  0
   -6.5117   -2.3515   -0.8424 C   0  0  0  0  0  0  0  0  0  0  0  0
   -7.0147   -2.5148   -2.1468 C   0  0  0  0  0  0  0  0  0  0  0  0
   -7.3785   -2.3454    0.2667 C   0  0  0  0  0  0  0  0  0  0  0  0
   -8.3892   -2.6705   -2.3336 C   0  0  0  0  0  0  0  0  0  0  0  0
   -6.3380   -2.5183   -3.0016 H   0  0  0  0  0  0  0  0  0  0  0  0
   -8.7502   -2.5036    0.0615 C   0  0  0  0  0  0  0  0  0  0  0  0
   -6.9832   -2.2189    1.2750 H   0  0  0  0  0  0  0  0  0  0  0  0
   -9.2556   -2.6649   -1.2340 C   0  0  0  0  0  0  0  0  0  0  0  0
   -8.7897   -2.7961   -3.3427 H   0  0  0  0  0  0  0  0  0  0  0  0
   -9.4319   -2.5003    0.9160 H   0  0  0  0  0  0  0  0  0  0  0  0
   -2.3427   -4.4209   -0.6661 N   0  0  0  0  0  0  0  0  0  0  0  0
   -2.4404   -5.5569   -0.8808 C   0  0  0  0  0  0  0  0  0  0  0  0
   -2.5568   -6.9585   -1.1427 C   0  0  0  0  0  0  0  0  0  0  0  0
   -2.7960   -7.8484   -0.0792 C   0  0  0  0  0  0  0  0  0  0  0  0
   -2.4304   -7.4359   -2.4603 C   0  0  0  0  0  0  0  0  0  0  0  0
   -2.9109   -9.2145   -0.3415 C   0  0  0  0  0  0  0  0  0  0  0  0
   -2.8897   -7.4730    0.9407 H   0  0  0  0  0  0  0  0  0  0  0  0
   -2.5481   -8.8047   -2.7070 C   0  0  0  0  0  0  0  0  0  0  0  0
   -2.2410   -6.7423   -3.2806 H   0  0  0  0  0  0  0  0  0  0  0  0
   -2.7886   -9.6924   -1.6516 C   0  0  0  0  0  0  0  0  0  0  0  0
   -3.0959   -9.9126    0.4785 H   0  0  0  0  0  0  0  0  0  0  0  0
   -2.4510   -9.1842   -3.7270 H   0  0  0  0  0  0  0  0  0  0  0  0
   -2.3062   -1.9734   -0.2493 Rh  0  0  0  0  0  0  0  0  0  0  0  0
   -2.8803  -10.7639   -1.8511 H   0  0  0  0  0  0  0  0  0  0  0  0
  -10.3316   -2.7860   -1.3878 H   0  0  0  0  0  0  0  0  0  0  0  0
    0.8425   -0.9021   -2.3788 F   0  0  0  0  0  0  0  0  0  0  0  0
   -1.1395    0.3216   -2.8409 F   0  0  0  0  0  0  0  0  0  0  0  0
   -0.7543   -1.6412    2.9948 F   0  0  0  0  0  0  0  0  0  0  0  0
   -1.6707    0.5444    2.7777 F   0  0  0  0  0  0  0  0  0  0  0  0
  1  2  1  0  0  0  0
  1  3  1  0  0  0  0
  1  9  1  1  0  0  0
  1 45  1  0  0  0  0
  2 12  1  0  0  0  0
  2 15  1  1  0  0  0
  2 44  1  0  0  0  0
  3  5  2  0  0  0  0
  3  7  1  0  0  0  0
  3 41  1  0  0  0  0
  4  5  1  0  0  0  0
  4  6  1  0  0  0  0
  4 10  1  6  0  0  0
  4 47  1  0  0  0  0
  5  8  1  0  0  0  0
  5 41  1  0  0  0  0
  6 11  1  0  0  0  0
  6 16  1  1  0  0  0
  6 46  1  0  0  0  0
 11 12  2  0  0  0  0
 11 13  1  0  0  0  0
 11 41  1  0  0  0  0
 12 14  1  0  0  0  0
 12 41  1  0  0  0  0
```

```
 17 18  3  0  0  0  0
 17 41  1  0  0  0  0
 18 19  1  0  0  0  0
 19 20  2  0  0  0  0
 19 21  1  0  0  0  0
 20 22  1  0  0  0  0
 20 23  1  0  0  0  0
 21 24  2  0  0  0  0
 21 25  1  0  0  0  0
 22 26  2  0  0  0  0
 22 27  1  0  0  0  0
 24 26  1  0  0  0  0
 24 28  1  0  0  0  0
 26 43  1  0  0  0  0
 29 30  3  0  0  0  0
 29 41  1  0  0  0  0
 30 31  1  0  0  0  0
 31 32  2  0  0  0  0
 31 33  1  0  0  0  0
 32 34  1  0  0  0  0
 32 35  1  0  0  0  0
 33 36  2  0  0  0  0
 33 37  1  0  0  0  0
 34 38  2  0  0  0  0
 34 39  1  0  0  0  0
 36 38  1  0  0  0  0
 36 40  1  0  0  0  0
 38 42  1  0  0  0  0
M  END
$$$$
mol085
Optimized Catalyst 085
Optimized Catalyst 085
 59 64  0  0  0  0  0  0  0  0999 V2000
   -1.1058    0.3703   -1.6653 C   0  0  2  0  0  0  0  0  0  0  0  0
    0.1818   -0.3689   -1.1907 C   0  0  1  0  0  0  0  0  0  0  0  0
   -2.2115    0.3463   -0.6212 C   0  0  0  0  0  0  0  0  0  0  0  0
   -0.9530    0.2174    1.6496 C   0  0  2  0  0  0  0  0  0  0  0  0
   -2.1675    0.2266    0.7424 C   0  0  0  0  0  0  0  0  0  0  0  0
   -0.5244   -1.2546    1.9349 C   0  0  2  0  0  0  0  0  0  0  0  0
   -3.2085    0.5356   -1.0691 H   0  0  0  0  0  0  0  0  0  0  0  0
   -3.1270    0.2525    1.2913 H   0  0  0  0  0  0  0  0  0  0  0  0
   -0.8433    1.4394   -1.8808 H   0  0  0  0  0  0  0  0  0  0  0  0
   -0.1002    0.7442    1.1472 H   0  0  0  0  0  0  0  0  0  0  0  0
   -0.4096   -2.1001    0.6702 C   0  0  0  0  0  0  0  0  0  0  0  0
   -0.1863   -1.7400   -0.6504 C   0  0  0  0  0  0  0  0  0  0  0  0
   -0.4150   -3.1957    0.8921 H   0  0  0  0  0  0  0  0  0  0  0  0
   -0.0970   -2.5607   -1.3985 H   0  0  0  0  0  0  0  0  0  0  0  0
    0.6615    0.2379   -0.3791 H   0  0  0  0  0  0  0  0  0  0  0  0
    0.4761   -1.2372    2.4416 H   0  0  0  0  0  0  0  0  0  0  0  0
   -3.8878   -1.9359   -0.4502 N   0  0  0  0  0  0  0  0  0  0  0  0
   -5.0276   -2.0864   -0.6314 C   0  0  0  0  0  0  0  0  0  0  0  0
   -6.4313   -2.2485   -0.8414 C   0  0  0  0  0  0  0  0  0  0  0  0
   -7.0827   -1.4944   -1.8349 C   0  0  0  0  0  0  0  0  0  0  0  0
   -7.1469   -3.1697   -0.0540 C   0  0  0  0  0  0  0  0  0  0  0  0
   -8.4528   -1.6711   -2.0373 C   0  0  0  0  0  0  0  0  0  0  0  0
   -6.5236   -0.7822   -2.4413 H   0  0  0  0  0  0  0  0  0  0  0  0
   -8.5167   -3.3328   -0.2684 C   0  0  0  0  0  0  0  0  0  0  0  0
   -6.6355   -3.7488    0.7150 H   0  0  0  0  0  0  0  0  0  0  0  0
   -9.1686   -2.5866   -1.2571 C   0  0  0  0  0  0  0  0  0  0  0  0
   -8.9673   -1.0935   -2.8090 H   0  0  0  0  0  0  0  0  0  0  0  0
   -9.0806   -4.0469    0.3366 H   0  0  0  0  0  0  0  0  0  0  0  0
   -2.2701   -4.3438   -0.6745 N   0  0  0  0  0  0  0  0  0  0  0  0
   -2.4024   -5.4789   -0.8705 C   0  0  0  0  0  0  0  0  0  0  0  0
   -2.5636   -6.8815   -1.1076 C   0  0  0  0  0  0  0  0  0  0  0  0
```

```
   -1.7861   -7.8051   -0.3855 C   0  0  0  0  0  0  0  0  0  0  0  0
   -3.5010   -7.3239   -2.0586 C   0  0  0  0  0  0  0  0  0  0  0  0
   -1.9563   -9.1716   -0.6151 C   0  0  0  0  0  0  0  0  0  0  0  0
   -1.0572   -7.4557    0.3465 H   0  0  0  0  0  0  0  0  0  0  0  0
   -3.6607   -8.6933   -2.2777 C   0  0  0  0  0  0  0  0  0  0  0  0
   -4.0960   -6.6025   -2.6196 H   0  0  0  0  0  0  0  0  0  0  0  0
   -2.8919   -9.6151   -1.5572 C   0  0  0  0  0  0  0  0  0  0  0  0
   -1.3574   -9.8962   -0.0579 H   0  0  0  0  0  0  0  0  0  0  0  0
   -4.3869   -9.0461   -3.0136 H   0  0  0  0  0  0  0  0  0  0  0  0
   -2.2449   -1.8986   -0.2374 Rh  0  0  0  0  0  0  0  0  0  0  0  0
   -3.0219  -10.6864   -1.7318 H   0  0  0  0  0  0  0  0  0  0  0  0
  -10.2411   -2.7191   -1.4205 H   0  0  0  0  0  0  0  0  0  0  0  0
   -1.5473   -1.9875    2.8318 C   0  0  0  0  0  0  0  0  0  0  0  0
   -1.1515   -2.9829    3.1390 H   0  0  0  0  0  0  0  0  0  0  0  0
   -1.7562   -1.4057    3.7561 H   0  0  0  0  0  0  0  0  0  0  0  0
   -2.5105   -2.1375    2.2590 H   0  0  0  0  0  0  0  0  0  0  0  0
   -1.2305    1.0262    2.9266 C   0  0  0  0  0  0  0  0  0  0  0  0
   -0.3752    0.9299    3.6349 H   0  0  0  0  0  0  0  0  0  0  0  0
   -1.3471    2.1046    2.6633 H   0  0  0  0  0  0  0  0  0  0  0  0
   -2.1594    0.6950    3.4409 H   0  0  0  0  0  0  0  0  0  0  0  0
    1.2496   -0.4939   -2.2887 C   0  0  0  0  0  0  0  0  0  0  0  0
    1.4432    0.5042   -2.7467 H   0  0  0  0  0  0  0  0  0  0  0  0
    2.2024   -0.8646   -1.8411 H   0  0  0  0  0  0  0  0  0  0  0  0
    0.9447   -1.2016   -3.0910 H   0  0  0  0  0  0  0  0  0  0  0  0
   -1.6913   -0.2850   -2.9357 C   0  0  0  0  0  0  0  0  0  0  0  0
   -2.5672    0.2934   -3.3072 H   0  0  0  0  0  0  0  0  0  0  0  0
   -0.9360   -0.3123   -3.7522 H   0  0  0  0  0  0  0  0  0  0  0  0
   -2.0173   -1.3410   -2.6957 H   0  0  0  0  0  0  0  0  0  0  0  0
  1  2  1  0  0  0  0
  1  3  1  0  0  0  0
  1  9  1  6  0  0  0
  1 56  1  0  0  0  0
  2 12  1  0  0  0  0
  2 15  1  1  0  0  0
  2 52  1  0  0  0  0
  3  5  2  0  0  0  0
  3  7  1  0  0  0  0
  3 41  1  0  0  0  0
  4  5  1  0  0  0  0
  4  6  1  0  0  0  0
  4 10  1  6  0  0  0
  4 48  1  0  0  0  0
  5  8  1  0  0  0  0
  5 41  1  0  0  0  0
  6 11  1  0  0  0  0
  6 16  1  1  0  0  0
  6 44  1  0  0  0  0
 11 12  2  0  0  0  0
 11 13  1  0  0  0  0
 11 41  1  0  0  0  0
 12 14  1  0  0  0  0
 12 41  1  0  0  0  0
 17 18  3  0  0  0  0
 17 41  1  0  0  0  0
 18 19  1  0  0  0  0
 19 20  2  0  0  0  0
 19 21  1  0  0  0  0
 20 22  1  0  0  0  0
 20 23  1  0  0  0  0
 21 24  2  0  0  0  0
 21 25  1  0  0  0  0
 22 26  2  0  0  0  0
 22 27  1  0  0  0  0
 24 26  1  0  0  0  0
 24 28  1  0  0  0  0
```

```
 26 43  1  0  0  0  0
 29 30  3  0  0  0  0
 29 41  1  0  0  0  0
 30 31  1  0  0  0  0
 31 32  2  0  0  0  0
 31 33  1  0  0  0  0
 32 34  1  0  0  0  0
 32 35  1  0  0  0  0
 33 36  2  0  0  0  0
 33 37  1  0  0  0  0
 34 38  2  0  0  0  0
 34 39  1  0  0  0  0
 36 38  1  0  0  0  0
 36 40  1  0  0  0  0
 38 42  1  0  0  0  0
 44 45  1  0  0  0  0
 44 46  1  0  0  0  0
 44 47  1  0  0  0  0
 48 49  1  0  0  0  0
 48 50  1  0  0  0  0
 48 51  1  0  0  0  0
 52 53  1  0  0  0  0
 52 54  1  0  0  0  0
 52 55  1  0  0  0  0
 56 57  1  0  0  0  0
 56 58  1  0  0  0  0
 56 59  1  0  0  0  0
M  END
$$$$
mol086
Optimized Catalyst 086
Optimized Catalyst 086
 83 90  0  0  0  0  0  0  0  0999 V2000
   -0.3593   -0.2074    1.3157 C   0  0  0  0  0  0  0  0  0  0  0  0
   -1.4702    0.6105    2.0062 C   0  0  0  0  0  0  0  0  0  0  0  0
   -0.7977   -0.9470    0.0888 C   0  0  0  0  0  0  0  0  0  0  0  0
   -0.5573    0.9171   -1.6548 C   0  0  0  0  0  0  0  0  0  0  0  0
   -0.8514   -0.4681   -1.1613 C   0  0  0  0  0  0  0  0  0  0  0  0
   -0.8676    2.0829   -0.6950 C   0  0  0  0  0  0  0  0  0  0  0  0
   -1.0577   -2.0056    0.2449 H   0  0  0  0  0  0  0  0  0  0  0  0
   -1.1160   -1.1788   -1.9796 H   0  0  0  0  0  0  0  0  0  0  0  0
    0.0453   -0.9388    2.0600 H   0  0  0  0  0  0  0  0  0  0  0  0
    0.4867    0.4730    1.0383 H   0  0  0  0  0  0  0  0  0  0  0  0
   -2.0287   -0.0705    2.6935 H   0  0  0  0  0  0  0  0  0  0  0  0
   -0.8485    3.0345   -1.2874 H   0  0  0  0  0  0  0  0  0  0  0  0
   -1.1449    1.0872   -2.5939 H   0  0  0  0  0  0  0  0  0  0  0  0
    0.5351    0.9578   -1.9125 H   0  0  0  0  0  0  0  0  0  0  0  0
   -2.2069    1.9437   -0.0370 C   0  0  0  0  0  0  0  0  0  0  0  0
   -2.4548    1.3058    1.1124 C   0  0  0  0  0  0  0  0  0  0  0  0
   -3.0253    2.4235   -0.5825 H   0  0  0  0  0  0  0  0  0  0  0  0
   -3.4752    1.2997    1.5074 H   0  0  0  0  0  0  0  0  0  0  0  0
   -0.9775    1.4011    2.6354 H   0  0  0  0  0  0  0  0  0  0  0  0
   -0.0698    2.1678    0.0820 H   0  0  0  0  0  0  0  0  0  0  0  0
   -2.4783   -3.2323   -1.6659 Rh  0  0  2  0  0  0  0  0  0  0  0  0
   -4.4388   -2.4879   -0.9848 P   0  0  0  0  0  0  0  0  0  0  0  0
   -2.3031   -5.1434   -0.5628 P   0  0  0  0  0  0  0  0  0  0  0  0
   -4.2259   -0.6535   -1.2797 C   0  0  1  0  0  0  0  0  0  0  0  0
   -3.3764   -0.2571   -0.6397 H   0  0  0  0  0  0  0  0  0  0  0  0
   -3.9364   -0.4530   -2.7565 C   0  0  0  0  0  0  0  0  0  0  0  0
   -5.4820    0.1622   -1.0608 C   0  0  0  0  0  0  0  0  0  0  0  0
   -3.0999   -0.9384   -3.2507 H   0  0  0  0  0  0  0  0  0  0  0  0
   -4.8470    0.4052   -3.2720 C   0  0  0  0  0  0  0  0  0  0  0  0
   -5.9575    0.2442   -0.0992 H   0  0  0  0  0  0  0  0  0  0  0  0
   -5.8040    0.7920   -2.2150 C   0  0  0  0  0  0  0  0  0  0  0  0
   -4.9121    0.7754   -4.2829 H   0  0  0  0  0  0  0  0  0  0  0  0
```

```
  -6.6166    1.4799   -2.3891 H   0  0  0  0  0  0  0  0  0  0  0  0
  -5.7091   -3.2781   -2.1235 C   0  0  1  0  0  0  0  0  0  0  0  0
  -6.0201   -4.2811   -1.7379 H   0  0  0  0  0  0  0  0  0  0  0  0
  -6.9347   -2.4225   -2.3846 C   0  0  0  0  0  0  0  0  0  0  0  0
  -5.0802   -3.3960   -3.5029 C   0  0  0  0  0  0  0  0  0  0  0  0
  -7.6145   -2.1190   -1.6087 H   0  0  0  0  0  0  0  0  0  0  0  0
  -7.0312   -2.1777   -3.7120 C   0  0  0  0  0  0  0  0  0  0  0  0
  -4.1385   -3.9142   -3.6716 H   0  0  0  0  0  0  0  0  0  0  0  0
  -5.8745   -2.7780   -4.4080 C   0  0  0  0  0  0  0  0  0  0  0  0
  -7.8051   -1.6315   -4.2298 H   0  0  0  0  0  0  0  0  0  0  0  0
  -5.7297   -2.6982   -5.4749 H   0  0  0  0  0  0  0  0  0  0  0  0
  -5.0102   -2.8088    0.7787 C   0  0  1  0  0  0  0  0  0  0  0  0
  -4.9641   -3.8908    1.0404 H   0  0  0  0  0  0  0  0  0  0  0  0
  -4.1920   -1.9591    1.7316 C   0  0  0  0  0  0  0  0  0  0  0  0
  -6.4248   -2.3072    1.0124 C   0  0  0  0  0  0  0  0  0  0  0  0
  -3.1236   -2.0571    1.8238 H   0  0  0  0  0  0  0  0  0  0  0  0
  -5.0160   -1.1304    2.4137 C   0  0  0  0  0  0  0  0  0  0  0  0
  -7.2802   -2.7367    0.5208 H   0  0  0  0  0  0  0  0  0  0  0  0
  -6.4073   -1.3518    1.9702 C   0  0  0  0  0  0  0  0  0  0  0  0
  -4.7545   -0.4120    3.1767 H   0  0  0  0  0  0  0  0  0  0  0  0
  -7.2470   -0.8157    2.3884 H   0  0  0  0  0  0  0  0  0  0  0  0
  -2.3494   -5.2595    1.3137 C   0  0  1  0  0  0  0  0  0  0  0  0
  -3.3469   -4.9792    1.7290 H   0  0  0  0  0  0  0  0  0  0  0  0
  -1.9829   -6.6706    1.7455 C   0  0  0  0  0  0  0  0  0  0  0  0
  -1.2399   -4.4273    1.9268 C   0  0  0  0  0  0  0  0  0  0  0  0
  -2.5892   -7.5283    1.5027 H   0  0  0  0  0  0  0  0  0  0  0  0
  -0.8662   -6.6308    2.5078 C   0  0  0  0  0  0  0  0  0  0  0  0
  -1.2001   -3.3553    1.8436 H   0  0  0  0  0  0  0  0  0  0  0  0
  -0.4015   -5.2336    2.6184 C   0  0  0  0  0  0  0  0  0  0  0  0
  -0.3646   -7.4550    2.9936 H   0  0  0  0  0  0  0  0  0  0  0  0
   0.4734   -4.9511    3.1859 H   0  0  0  0  0  0  0  0  0  0  0  0
  -3.5186   -6.3962   -1.2802 C   0  0  1  0  0  0  0  0  0  0  0  0
  -3.7943   -6.0864   -2.3256 H   0  0  0  0  0  0  0  0  0  0  0  0
  -2.9627   -7.8092   -1.2867 C   0  0  0  0  0  0  0  0  0  0  0  0
  -4.7401   -6.5270   -0.3908 C   0  0  0  0  0  0  0  0  0  0  0  0
  -2.0960   -8.0858   -1.8664 H   0  0  0  0  0  0  0  0  0  0  0  0
  -3.7580   -8.6123   -0.5440 C   0  0  0  0  0  0  0  0  0  0  0  0
  -5.4211   -5.7244   -0.1906 H   0  0  0  0  0  0  0  0  0  0  0  0
  -4.8662   -7.8135    0.0138 C   0  0  0  0  0  0  0  0  0  0  0  0
  -3.6552   -9.6733   -0.3721 H   0  0  0  0  0  0  0  0  0  0  0  0
  -5.6409   -8.2416    0.6330 H   0  0  0  0  0  0  0  0  0  0  0  0
  -0.5324   -5.4114   -1.1440 C   0  0  1  0  0  0  0  0  0  0  0  0
   0.1358   -4.6547   -0.6395 H   0  0  0  0  0  0  0  0  0  0  0  0
   0.0031   -6.8144   -0.9483 C   0  0  0  0  0  0  0  0  0  0  0  0
  -0.5095   -5.2396   -2.6549 C   0  0  0  0  0  0  0  0  0  0  0  0
   0.1262   -7.2532    0.0279 H   0  0  0  0  0  0  0  0  0  0  0  0
   0.3419   -7.3284   -2.1542 C   0  0  0  0  0  0  0  0  0  0  0  0
  -0.8242   -4.3115   -3.1453 H   0  0  0  0  0  0  0  0  0  0  0  0
   0.0147   -6.3543   -3.2167 C   0  0  0  0  0  0  0  0  0  0  0  0
   0.7939   -8.2866   -2.3632 H   0  0  0  0  0  0  0  0  0  0  0  0
   0.1966   -6.5491   -4.2634 H   0  0  0  0  0  0  0  0  0  0  0  0
  1  2  1  0  0  0  0
  1  9  1  0  0  0  0
  1  3  1  0  0  0  0
  1 10  1  0  0  0  0
  2 19  1  0  0  0  0
  2 11  1  0  0  0  0
  2 16  1  0  0  0  0
  3  7  1  0  0  0  0
  3  5  2  0  0  0  0
  3 21  1  0  0  0  0
  4  5  1  0  0  0  0
  4  6  1  0  0  0  0
  4 13  1  0  0  0  0
  4 14  1  0  0  0  0
```

```
  5  8  1  0  0  0  0
  5 21  1  0  0  0  0
  6 15  1  0  0  0  0
  6 20  1  0  0  0  0
  6 12  1  0  0  0  0
 15 16  2  0  0  0  0
 15 17  1  0  0  0  0
 16 18  1  0  0  0  0
 21 22  1  1  0  0  0
 21 23  1  0  0  0  0
 22 44  1  0  0  0  0
 22 24  1  0  0  0  0
 22 34  1  0  0  0  0
 23 54  1  0  0  0  0
 23 64  1  0  0  0  0
 23 74  1  0  0  0  0
 24 27  1  0  0  0  0
 24 25  1  1  0  0  0
 24 26  1  0  0  0  0
 26 28  1  0  0  0  0
 26 29  2  0  0  0  0
 27 30  1  0  0  0  0
 27 31  2  0  0  0  0
 29 31  1  0  0  0  0
 29 32  1  0  0  0  0
 31 33  1  0  0  0  0
 34 35  1  1  0  0  0
 34 36  1  0  0  0  0
 34 37  1  0  0  0  0
 36 38  1  0  0  0  0
 36 39  2  0  0  0  0
 37 40  1  0  0  0  0
 37 41  2  0  0  0  0
 39 41  1  0  0  0  0
 39 42  1  0  0  0  0
 41 43  1  0  0  0  0
 44 47  1  0  0  0  0
 44 45  1  1  0  0  0
 44 46  1  0  0  0  0
 46 48  1  0  0  0  0
 46 49  2  0  0  0  0
 47 51  2  0  0  0  0
 47 50  1  0  0  0  0
 49 52  1  0  0  0  0
 49 51  1  0  0  0  0
 51 53  1  0  0  0  0
 54 55  1  1  0  0  0
 54 56  1  0  0  0  0
 54 57  1  0  0  0  0
 56 59  2  0  0  0  0
 56 58  1  0  0  0  0
 57 61  2  0  0  0  0
 57 60  1  0  0  0  0
 59 61  1  0  0  0  0
 59 62  1  0  0  0  0
 61 63  1  0  0  0  0
 64 65  1  6  0  0  0
 64 67  1  0  0  0  0
 64 66  1  0  0  0  0
 66 69  2  0  0  0  0
 66 68  1  0  0  0  0
 67 70  1  0  0  0  0
 67 71  2  0  0  0  0
 69 72  1  0  0  0  0
 69 71  1  0  0  0  0
```

```
 71 73  1  0  0  0  0
 74 76  1  0  0  0  0
 74 75  1  1  0  0  0
 74 77  1  0  0  0  0
 76 78  1  0  0  0  0
 76 79  2  0  0  0  0
 77 80  1  0  0  0  0
 77 81  2  0  0  0  0
 79 81  1  0  0  0  0
 79 82  1  0  0  0  0
 81 83  1  0  0  0  0
M  END
$$$$
mol087
Optimized Catalyst 087
Optimized Catalyst 087
113122  0  0  0  0  0  0  0  0999 V2000
   -5.2172   -2.3368   -0.8530 P   0  0  0  0  0  0  0  0  0  0  0  0
   -2.2682   -4.7973   -0.4146 P   0  0  0  0  0  0  0  0  0  0  0  0
   -0.4859   -5.1740   -0.1082 C   0  0  0  0  0  0  0  0  0  0  0  0
    0.3123   -5.5418   -1.2001 C   0  0  0  0  0  0  0  0  0  0  0  0
    0.0284   -5.1704    1.1928 C   0  0  0  0  0  0  0  0  0  0  0  0
    1.6382   -5.9244   -0.9780 C   0  0  0  0  0  0  0  0  0  0  0  0
   -0.0676   -5.5472   -2.2264 H   0  0  0  0  0  0  0  0  0  0  0  0
    1.3603   -5.5442    1.4013 C   0  0  0  0  0  0  0  0  0  0  0  0
   -0.5764   -4.8966    2.0614 H   0  0  0  0  0  0  0  0  0  0  0  0
    2.1630   -5.9252    0.3202 C   0  0  0  0  0  0  0  0  0  0  0  0
    2.2671   -6.2279   -1.8191 H   0  0  0  0  0  0  0  0  0  0  0  0
    1.7737   -5.5426    2.4128 H   0  0  0  0  0  0  0  0  0  0  0  0
    3.2017   -6.2234    0.4893 H   0  0  0  0  0  0  0  0  0  0  0  0
   -2.8310   -6.0350   -1.6706 C   0  0  0  0  0  0  0  0  0  0  0  0
   -2.5859   -7.3903   -1.4270 C   0  0  0  0  0  0  0  0  0  0  0  0
   -3.4506   -5.6132   -2.8501 C   0  0  0  0  0  0  0  0  0  0  0  0
   -3.0211   -8.3394   -2.3594 C   0  0  0  0  0  0  0  0  0  0  0  0
   -2.0474   -7.7365   -0.5388 H   0  0  0  0  0  0  0  0  0  0  0  0
   -3.8823   -6.5690   -3.7755 C   0  0  0  0  0  0  0  0  0  0  0  0
   -3.5926   -4.5520   -3.0925 H   0  0  0  0  0  0  0  0  0  0  0  0
   -3.6759   -7.9313   -3.5258 C   0  0  0  0  0  0  0  0  0  0  0  0
   -2.8397   -9.4023   -2.1785 H   0  0  0  0  0  0  0  0  0  0  0  0
   -4.3721   -6.2533   -4.6999 H   0  0  0  0  0  0  0  0  0  0  0  0
   -4.0168   -8.6767   -4.2507 H   0  0  0  0  0  0  0  0  0  0  0  0
   -3.1812   -5.0450    1.1776 C   0  0  0  0  0  0  0  0  0  0  0  0
   -3.9267   -6.2015    1.4035 C   0  0  0  0  0  0  0  0  0  0  0  0
   -3.0630   -4.0566    2.1660 C   0  0  0  0  0  0  0  0  0  0  0  0
   -4.5455   -6.3844    2.6493 C   0  0  0  0  0  0  0  0  0  0  0  0
   -4.0641   -6.9713    0.6392 H   0  0  0  0  0  0  0  0  0  0  0  0
   -3.6865   -4.2456    3.4003 C   0  0  0  0  0  0  0  0  0  0  0  0
   -2.4860   -3.1378    2.0081 H   0  0  0  0  0  0  0  0  0  0  0  0
   -4.4226   -5.4144    3.6453 C   0  0  0  0  0  0  0  0  0  0  0  0
   -5.1256   -7.2909    2.8367 H   0  0  0  0  0  0  0  0  0  0  0  0
   -3.5951   -3.4890    4.1827 H   0  0  0  0  0  0  0  0  0  0  0  0
   -4.9002   -5.5628    4.6178 H   0  0  0  0  0  0  0  0  0  0  0  0
   -5.6064   -0.8228    0.1349 C   0  0  0  0  0  0  0  0  0  0  0  0
   -6.3843    0.1883   -0.4343 C   0  0  0  0  0  0  0  0  0  0  0  0
   -5.1357   -0.7184    1.4522 C   0  0  0  0  0  0  0  0  0  0  0  0
   -6.7085    1.3155    0.3330 C   0  0  0  0  0  0  0  0  0  0  0  0
   -6.7509    0.1366   -1.4651 H   0  0  0  0  0  0  0  0  0  0  0  0
   -5.4692    0.4065    2.2087 C   0  0  0  0  0  0  0  0  0  0  0  0
   -4.5111   -1.4889    1.9124 H   0  0  0  0  0  0  0  0  0  0  0  0
   -6.2576    1.4233    1.6505 C   0  0  0  0  0  0  0  0  0  0  0  0
   -7.3178    2.1117   -0.1035 H   0  0  0  0  0  0  0  0  0  0  0  0
   -5.1161    0.4986    3.2385 H   0  0  0  0  0  0  0  0  0  0  0  0
   -6.5168    2.3023    2.2487 H   0  0  0  0  0  0  0  0  0  0  0  0
   -6.0016   -2.1608   -2.5229 C   0  0  0  0  0  0  0  0  0  0  0  0
   -7.3936   -2.2607   -2.6125 C   0  0  0  0  0  0  0  0  0  0  0  0
```

```
   -5.2165   -1.9361   -3.6576 C   0  0  0  0  0  0  0  0  0  0  0  0
   -8.0070   -2.1164   -3.8627 C   0  0  0  0  0  0  0  0  0  0  0  0
   -8.0220   -2.4508   -1.7365 H   0  0  0  0  0  0  0  0  0  0  0  0
   -5.8382   -1.7923   -4.9014 C   0  0  0  0  0  0  0  0  0  0  0  0
   -4.1175   -1.8711   -3.6100 H   0  0  0  0  0  0  0  0  0  0  0  0
   -7.2329   -1.8800   -5.0029 C   0  0  0  0  0  0  0  0  0  0  0  0
   -9.0947   -2.1890   -3.9466 H   0  0  0  0  0  0  0  0  0  0  0  0
   -5.2381   -1.6093   -5.7964 H   0  0  0  0  0  0  0  0  0  0  0  0
   -7.7159   -1.7641   -5.9780 H   0  0  0  0  0  0  0  0  0  0  0  0
   -6.0583   -3.7906   -0.0836 C   0  0  0  0  0  0  0  0  0  0  0  0
   -6.6402   -3.6939    1.1830 C   0  0  0  0  0  0  0  0  0  0  0  0
   -6.1917   -4.9563   -0.8501 C   0  0  0  0  0  0  0  0  0  0  0  0
   -7.3543   -4.7855    1.6929 C   0  0  0  0  0  0  0  0  0  0  0  0
   -6.5719   -2.7901    1.7946 H   0  0  0  0  0  0  0  0  0  0  0  0
   -6.9011   -6.0412   -0.3300 C   0  0  0  0  0  0  0  0  0  0  0  0
   -5.7748   -5.0454   -1.8570 H   0  0  0  0  0  0  0  0  0  0  0  0
   -7.4851   -5.9560    0.9406 C   0  0  0  0  0  0  0  0  0  0  0  0
   -7.8168   -4.7163    2.6802 H   0  0  0  0  0  0  0  0  0  0  0  0
   -7.0108   -6.9536   -0.9201 H   0  0  0  0  0  0  0  0  0  0  0  0
   -8.0487   -6.8033    1.3401 H   0  0  0  0  0  0  0  0  0  0  0  0
   -2.4004    0.8268   -0.5310 C   0  0  0  0  0  0  0  0  0  0  0  0
   -1.2659    1.4436    0.2332 C   0  0  0  0  0  0  0  0  0  0  0  0
   -1.3302    2.2991   -3.2050 C   0  0  0  0  0  0  0  0  0  0  0  0
   -0.9514    2.8906   -0.2124 C   0  0  0  0  0  0  0  0  0  0  0  0
   -0.1697    3.1618   -2.6846 C   0  0  0  0  0  0  0  0  0  0  0  0
    0.2187    2.9964   -1.2050 C   0  0  0  0  0  0  0  0  0  0  0  0
   -0.3482    0.8142    0.1180 H   0  0  0  0  0  0  0  0  0  0  0  0
   -1.8651    3.3508   -0.6554 H   0  0  0  0  0  0  0  0  0  0  0  0
   -2.2937    2.7376   -2.8479 H   0  0  0  0  0  0  0  0  0  0  0  0
   -0.4497    4.2321   -2.8581 H   0  0  0  0  0  0  0  0  0  0  0  0
   -3.3192    0.7025    0.0614 H   0  0  0  0  0  0  0  0  0  0  0  0
   -1.5287    1.4645    1.3208 H   0  0  0  0  0  0  0  0  0  0  0  0
   -1.3245    2.3594   -4.3216 H   0  0  0  0  0  0  0  0  0  0  0  0
   -0.6841    3.4744    0.7038 H   0  0  0  0  0  0  0  0  0  0  0  0
    0.7406    2.9432   -3.2975 H   0  0  0  0  0  0  0  0  0  0  0  0
    0.8281    3.8950   -0.9255 H   0  0  0  0  0  0  0  0  0  0  0  0
   -2.3927    0.4965   -1.8388 C   0  0  0  0  0  0  0  0  0  0  0  0
   -3.3199    0.1542   -2.3296 H   0  0  0  0  0  0  0  0  0  0  0  0
   -1.2683    0.8111   -2.7915 C   0  0  0  0  0  0  0  0  0  0  0  0
   -0.2801    0.6020   -2.3118 H   0  0  0  0  0  0  0  0  0  0  0  0
   -1.3509    0.1763   -3.7063 H   0  0  0  0  0  0  0  0  0  0  0  0
    0.4289   -1.9869   -1.8494 C   0  0  0  0  0  0  0  0  0  0  0  0
    1.4367   -1.8597   -2.9529 C   0  0  0  0  0  0  0  0  0  0  0  0
    2.8716   -2.3401    0.2799 C   0  0  0  0  0  0  0  0  0  0  0  0
    2.6708   -2.7695   -2.7673 C   0  0  0  0  0  0  0  0  0  0  0  0
    4.1069   -2.3625   -0.6342 C   0  0  0  0  0  0  0  0  0  0  0  0
    3.8787   -2.0657   -2.1262 C   0  0  0  0  0  0  0  0  0  0  0  0
    1.7631   -0.7893   -3.0195 H   0  0  0  0  0  0  0  0  0  0  0  0
    2.3850   -3.6537   -2.1536 H   0  0  0  0  0  0  0  0  0  0  0  0
    2.3154   -3.3008    0.1570 H   0  0  0  0  0  0  0  0  0  0  0  0
    4.5718   -3.3757   -0.5423 H   0  0  0  0  0  0  0  0  0  0  0  0
   -0.5624   -2.3812   -2.1961 H   0  0  0  0  0  0  0  0  0  0  0  0
    0.9533   -2.1185   -3.9277 H   0  0  0  0  0  0  0  0  0  0  0  0
    3.2316   -2.2865    1.3368 H   0  0  0  0  0  0  0  0  0  0  0  0
    2.9786   -3.1335   -3.7786 H   0  0  0  0  0  0  0  0  0  0  0  0
    4.8435   -1.6104   -0.2536 H   0  0  0  0  0  0  0  0  0  0  0  0
    4.8019   -2.3917   -2.6715 H   0  0  0  0  0  0  0  0  0  0  0  0
    0.6142   -1.6742   -0.5585 C   0  0  0  0  0  0  0  0  0  0  0  0
   -0.2009   -1.8079    0.1725 H   0  0  0  0  0  0  0  0  0  0  0  0
    1.8967   -1.1701    0.0341 C   0  0  0  0  0  0  0  0  0  0  0  0
    2.3650   -0.4198   -0.6499 H   0  0  0  0  0  0  0  0  0  0  0  0
    1.6869   -0.6554    1.0029 H   0  0  0  0  0  0  0  0  0  0  0  0
    3.7897   -0.9625   -2.2678 H   0  0  0  0  0  0  0  0  0  0  0  0
    0.8823    2.1057   -1.0992 H   0  0  0  0  0  0  0  0  0  0  0  0
   -2.6269   -2.3419   -1.2204 Ir  0  0  0  0  0  0  0  0  0  0  0  0
```

```
  1 36  1  0  0  0  0
  1 47  1  0  0  0  0
  1 58  1  0  0  0  0
  1113  1  0  0  0  0
  2  3  1  0  0  0  0
  2 14  1  0  0  0  0
  2 25  1  0  0  0  0
  2113  1  0  0  0  0
  3  4  2  0  0  0  0
  3  5  1  0  0  0  0
  4  6  1  0  0  0  0
  4  7  1  0  0  0  0
  5  8  2  0  0  0  0
  5  9  1  0  0  0  0
  6 10  2  0  0  0  0
  6 11  1  0  0  0  0
  8 10  1  0  0  0  0
  8 12  1  0  0  0  0
 10 13  1  0  0  0  0
 14 15  2  0  0  0  0
 14 16  1  0  0  0  0
 15 17  1  0  0  0  0
 15 18  1  0  0  0  0
 16 19  2  0  0  0  0
 16 20  1  0  0  0  0
 17 21  2  0  0  0  0
 17 22  1  0  0  0  0
 19 21  1  0  0  0  0
 19 23  1  0  0  0  0
 21 24  1  0  0  0  0
 25 26  2  0  0  0  0
 25 27  1  0  0  0  0
 26 28  1  0  0  0  0
 26 29  1  0  0  0  0
 27 30  2  0  0  0  0
 27 31  1  0  0  0  0
 28 32  2  0  0  0  0
 28 33  1  0  0  0  0
 30 32  1  0  0  0  0
 30 34  1  0  0  0  0
 32 35  1  0  0  0  0
 36 37  2  0  0  0  0
 36 38  1  0  0  0  0
 37 39  1  0  0  0  0
 37 40  1  0  0  0  0
 38 41  2  0  0  0  0
 38 42  1  0  0  0  0
 39 43  2  0  0  0  0
 39 44  1  0  0  0  0
 41 43  1  0  0  0  0
 41 45  1  0  0  0  0
 43 46  1  0  0  0  0
 47 48  2  0  0  0  0
 47 49  1  0  0  0  0
 48 50  1  0  0  0  0
 48 51  1  0  0  0  0
 49 52  2  0  0  0  0
 49 53  1  0  0  0  0
 50 54  2  0  0  0  0
 50 55  1  0  0  0  0
 52 54  1  0  0  0  0
 52 56  1  0  0  0  0
 54 57  1  0  0  0  0
 58 59  2  0  0  0  0
 58 60  1  0  0  0  0
```

```
 59 61  1  0  0  0  0
 59 62  1  0  0  0  0
 60 63  2  0  0  0  0
 60 64  1  0  0  0  0
 61 65  2  0  0  0  0
 61 66  1  0  0  0  0
 63 65  1  0  0  0  0
 63 67  1  0  0  0  0
 65 68  1  0  0  0  0
 69 70  1  0  0  0  0
 69 79  1  0  0  0  0
 69 85  2  0  0  0  0
 69113  1  0  0  0  0
 70 72  1  0  0  0  0
 70 75  1  0  0  0  0
 70 80  1  0  0  0  0
 71 73  1  0  0  0  0
 71 77  1  0  0  0  0
 71 81  1  0  0  0  0
 71 87  1  0  0  0  0
 72 74  1  0  0  0  0
 72 76  1  0  0  0  0
 72 82  1  0  0  0  0
 73 74  1  0  0  0  0
 73 78  1  0  0  0  0
 73 83  1  0  0  0  0
 74 84  1  0  0  0  0
 74112  1  0  0  0  0
 85 86  1  0  0  0  0
 85 87  1  0  0  0  0
 85113  1  0  0  0  0
 87 88  1  0  0  0  0
 87 89  1  0  0  0  0
 90 91  1  0  0  0  0
 90100  1  0  0  0  0
 90106  2  0  0  0  0
 90113  1  0  0  0  0
 91 93  1  0  0  0  0
 91 96  1  0  0  0  0
 91101  1  0  0  0  0
 92 94  1  0  0  0  0
 92 98  1  0  0  0  0
 92102  1  0  0  0  0
 92108  1  0  0  0  0
 93 95  1  0  0  0  0
 93 97  1  0  0  0  0
 93103  1  0  0  0  0
 94 95  1  0  0  0  0
 94 99  1  0  0  0  0
 94104  1  0  0  0  0
 95105  1  0  0  0  0
 95111  1  0  0  0  0
106107  1  0  0  0  0
106108  1  0  0  0  0
106113  1  0  0  0  0
108109  1  0  0  0  0
108110  1  0  0  0  0
M  END
$$$$
mol088
Optimized Catalyst 088
Optimized Catalyst 088
 92 99  0  0  0  0  0  0  0  0999 V2000
   -4.8769   -1.9973   -0.7178 P   0  0  0  0  0  0  0  0  0  0  0  0
   -5.9619   -0.7999    0.1965 C   0  0  0  0  0  0  0  0  0  0  0  0
```

-7.1500	-0.2929	-0.3242	C	0	0	0	0	0	0	0	0	0	0	0	0
-5.5276	-0.4487	1.4838	C	0	0	0	0	0	0	0	0	0	0	0	0
-7.9385	0.5520	0.4715	C	0	0	0	0	0	0	0	0	0	0	0	0
-7.4866	-0.5172	-1.3391	H	0	0	0	0	0	0	0	0	0	0	0	0
-6.3229	0.3839	2.2728	C	0	0	0	0	0	0	0	0	0	0	0	0
-4.5616	-0.7999	1.8746	H	0	0	0	0	0	0	0	0	0	0	0	0
-7.5347	0.8776	1.7679	C	0	0	0	0	0	0	0	0	0	0	0	0
-8.8723	0.9555	0.0728	H	0	0	0	0	0	0	0	0	0	0	0	0
-6.0030	0.6563	3.2805	H	0	0	0	0	0	0	0	0	0	0	0	0
-8.1597	1.5244	2.3898	H	0	0	0	0	0	0	0	0	0	0	0	0
-5.4494	-2.1747	-2.4707	C	0	0	0	0	0	0	0	0	0	0	0	0
-6.7580	-2.5666	-2.7655	C	0	0	0	0	0	0	0	0	0	0	0	0
-4.5075	-1.9737	-3.4832	C	0	0	0	0	0	0	0	0	0	0	0	0
-7.1395	-2.7081	-4.1042	C	0	0	0	0	0	0	0	0	0	0	0	0
-7.4892	-2.7857	-1.9821	H	0	0	0	0	0	0	0	0	0	0	0	0
-4.8958	-2.1187	-4.8192	C	0	0	0	0	0	0	0	0	0	0	0	0
-3.4599	-1.7171	-3.2438	H	0	0	0	0	0	0	0	0	0	0	0	0
-6.2133	-2.4771	-5.1279	C	0	0	0	0	0	0	0	0	0	0	0	0
-8.1614	-3.0050	-4.3508	H	0	0	0	0	0	0	0	0	0	0	0	0
-4.1731	-1.9564	-5.6208	H	0	0	0	0	0	0	0	0	0	0	0	0
-6.5180	-2.5854	-6.1725	H	0	0	0	0	0	0	0	0	0	0	0	0
-5.2975	-3.6630	0.0143	C	0	0	0	0	0	0	0	0	0	0	0	0
-5.7687	-3.7794	1.3229	C	0	0	0	0	0	0	0	0	0	0	0	0
-5.2028	-4.7895	-0.8132	C	0	0	0	0	0	0	0	0	0	0	0	0
-6.1368	-5.0391	1.8129	C	0	0	0	0	0	0	0	0	0	0	0	0
-5.8738	-2.9160	1.9851	H	0	0	0	0	0	0	0	0	0	0	0	0
-5.5738	-6.0428	-0.3183	C	0	0	0	0	0	0	0	0	0	0	0	0
-4.8464	-4.7199	-1.8458	H	0	0	0	0	0	0	0	0	0	0	0	0
-6.0417	-6.1685	0.9953	C	0	0	0	0	0	0	0	0	0	0	0	0
-6.5103	-5.1355	2.8348	H	0	0	0	0	0	0	0	0	0	0	0	0
-5.5031	-6.9249	-0.9581	H	0	0	0	0	0	0	0	0	0	0	0	0
-6.3361	-7.1487	1.3785	H	0	0	0	0	0	0	0	0	0	0	0	0
-3.1258	0.9292	-0.4001	C	0	0	0	0	0	0	0	0	0	0	0	0
-1.9332	1.1974	0.4872	C	0	0	0	0	0	0	0	0	0	0	0	0
-1.4619	2.2051	-2.8981	C	0	0	0	0	0	0	0	0	0	0	0	0
-1.1981	2.5153	0.1525	C	0	0	0	0	0	0	0	0	0	0	0	0
-0.1672	2.6988	-2.2336	C	0	0	0	0	0	0	0	0	0	0	0	0
0.0344	2.3387	-0.7513	C	0	0	0	0	0	0	0	0	0	0	0	0
-1.2194	0.3193	0.4140	H	0	0	0	0	0	0	0	0	0	0	0	0
-1.9156	3.2269	-0.3200	H	0	0	0	0	0	0	0	0	0	0	0	0
-2.3058	2.8667	-2.5823	H	0	0	0	0	0	0	0	0	0	0	0	0
-0.1451	3.8138	-2.3349	H	0	0	0	0	0	0	0	0	0	0	0	0
-4.1024	1.0919	0.0882	H	0	0	0	0	0	0	0	0	0	0	0	0
-2.2877	1.2490	1.5492	H	0	0	0	0	0	0	0	0	0	0	0	0
-1.3423	2.3175	-4.0046	H	0	0	0	0	0	0	0	0	0	0	0	0
-0.8589	2.9665	1.1187	H	0	0	0	0	0	0	0	0	0	0	0	0
0.7048	2.2861	-2.8013	H	0	0	0	0	0	0	0	0	0	0	0	0
0.8484	3.0037	-0.3614	H	0	0	0	0	0	0	0	0	0	0	0	0
-3.0836	0.6805	-1.7533	C	0	0	0	0	0	0	0	0	0	0	0	0
-4.0228	0.6331	-2.3297	H	0	0	0	0	0	0	0	0	0	0	0	0
-1.8292	0.7360	-2.5914	C	0	0	0	0	0	0	0	0	0	0	0	0
-0.9850	0.2266	-2.0442	H	0	0	0	0	0	0	0	0	0	0	0	0
-1.9932	0.1869	-3.5507	H	0	0	0	0	0	0	0	0	0	0	0	0
0.0877	-2.2070	-2.1215	C	0	0	0	0	0	0	0	0	0	0	0	0
1.0146	-2.4913	-3.2662	C	0	0	0	0	0	0	0	0	0	0	0	0
2.3798	-3.2386	-0.0472	C	0	0	0	0	0	0	0	0	0	0	0	0
1.9257	-3.7217	-3.0687	C	0	0	0	0	0	0	0	0	0	0	0	0
3.4967	-3.7054	-0.9954	C	0	0	0	0	0	0	0	0	0	0	0	0
3.3147	-3.3974	-2.4915	C	0	0	0	0	0	0	0	0	0	0	0	0
1.6460	-1.5818	-3.4390	H	0	0	0	0	0	0	0	0	0	0	0	0
1.4154	-4.4567	-2.4088	H	0	0	0	0	0	0	0	0	0	0	0	0
1.5343	-3.9656	-0.0958	H	0	0	0	0	0	0	0	0	0	0	0	0
3.6025	-4.8120	-0.8712	H	0	0	0	0	0	0	0	0	0	0	0	0
-0.9865	-2.2459	-2.3797	H	0	0	0	0	0	0	0	0	0	0	0	0
0.4057	-2.6566	-4.1900	H	0	0	0	0	0	0	0	0	0	0	0	0

```
    2.7879   -3.2624    0.9936 H   0  0  0  0  0  0  0  0  0  0  0  0
    2.0711   -4.1974   -4.0706 H   0  0  0  0  0  0  0  0  0  0  0  0
    4.4568   -3.2298   -0.6713 H   0  0  0  0  0  0  0  0  0  0  0  0
    4.0746   -4.0051   -3.0466 H   0  0  0  0  0  0  0  0  0  0  0  0
    0.4390   -1.8850   -0.8690 C   0  0  0  0  0  0  0  0  0  0  0  0
   -0.3488   -1.6622   -0.1247 H   0  0  0  0  0  0  0  0  0  0  0  0
    1.8368   -1.8218   -0.3292 C   0  0  0  0  0  0  0  0  0  0  0  0
    2.5002   -1.2961   -1.0588 H   0  0  0  0  0  0  0  0  0  0  0  0
    1.8449   -1.2297    0.6179 H   0  0  0  0  0  0  0  0  0  0  0  0
    3.5566   -2.3238   -2.6753 H   0  0  0  0  0  0  0  0  0  0  0  0
    0.4068    1.2880   -0.6786 H   0  0  0  0  0  0  0  0  0  0  0  0
   -2.8363   -1.2469   -0.6897 Rh  0  0  0  0  0  0  0  0  0  0  0  0
   -2.1895   -3.9654   -0.1269 C   0  0  0  0  0  0  0  0  0  0  0  0
   -1.7935   -5.3111    0.1359 C   0  0  0  0  0  0  0  0  0  0  0  0
   -2.3929   -6.0247    1.1907 C   0  0  0  0  0  0  0  0  0  0  0  0
   -0.8110   -5.9107   -0.6738 C   0  0  0  0  0  0  0  0  0  0  0  0
   -2.0046   -7.3441    1.4267 C   0  0  0  0  0  0  0  0  0  0  0  0
   -3.1544   -5.5501    1.8113 H   0  0  0  0  0  0  0  0  0  0  0  0
   -0.4301   -7.2291   -0.4204 C   0  0  0  0  0  0  0  0  0  0  0  0
   -0.3577   -5.3460   -1.4908 H   0  0  0  0  0  0  0  0  0  0  0  0
   -1.0258   -7.9447    0.6250 C   0  0  0  0  0  0  0  0  0  0  0  0
   -2.4624   -7.9087    2.2420 H   0  0  0  0  0  0  0  0  0  0  0  0
    0.3362   -7.7021   -1.0398 H   0  0  0  0  0  0  0  0  0  0  0  0
   -0.7252   -8.9785    0.8164 H   0  0  0  0  0  0  0  0  0  0  0  0
   -2.5916   -2.8985   -0.3634 N   0  0  0  0  0  0  0  0  0  0  0  0
  1  2  1  0  0  0  0
  1 13  1  0  0  0  0
  1 24  1  0  0  0  0
  1 79  1  0  0  0  0
  2  3  2  0  0  0  0
  2  4  1  0  0  0  0
  3  5  1  0  0  0  0
  3  6  1  0  0  0  0
  4  7  2  0  0  0  0
  4  8  1  0  0  0  0
  5  9  2  0  0  0  0
  5 10  1  0  0  0  0
  7  9  1  0  0  0  0
  7 11  1  0  0  0  0
  9 12  1  0  0  0  0
 13 14  2  0  0  0  0
 13 15  1  0  0  0  0
 14 16  1  0  0  0  0
 14 17  1  0  0  0  0
 15 18  2  0  0  0  0
 15 19  1  0  0  0  0
 16 20  2  0  0  0  0
 16 21  1  0  0  0  0
 18 20  1  0  0  0  0
 18 22  1  0  0  0  0
 20 23  1  0  0  0  0
 24 25  2  0  0  0  0
 24 26  1  0  0  0  0
 25 27  1  0  0  0  0
 25 28  1  0  0  0  0
 26 29  2  0  0  0  0
 26 30  1  0  0  0  0
 27 31  2  0  0  0  0
 27 32  1  0  0  0  0
 29 31  1  0  0  0  0
 29 33  1  0  0  0  0
 31 34  1  0  0  0  0
 35 36  1  0  0  0  0
 35 45  1  0  0  0  0
 35 51  2  0  0  0  0
```

```
 35 79  1  0  0  0  0
 36 38  1  0  0  0  0
 36 41  1  0  0  0  0
 36 46  1  0  0  0  0
 37 39  1  0  0  0  0
 37 43  1  0  0  0  0
 37 47  1  0  0  0  0
 37 53  1  0  0  0  0
 38 40  1  0  0  0  0
 38 42  1  0  0  0  0
 38 48  1  0  0  0  0
 39 40  1  0  0  0  0
 39 44  1  0  0  0  0
 39 49  1  0  0  0  0
 40 50  1  0  0  0  0
 40 78  1  0  0  0  0
 51 52  1  0  0  0  0
 51 53  1  0  0  0  0
 51 79  1  0  0  0  0
 53 54  1  0  0  0  0
 53 55  1  0  0  0  0
 56 57  1  0  0  0  0
 56 66  1  0  0  0  0
 56 72  2  0  0  0  0
 56 79  1  0  0  0  0
 57 59  1  0  0  0  0
 57 62  1  0  0  0  0
 57 67  1  0  0  0  0
 58 60  1  0  0  0  0
 58 64  1  0  0  0  0
 58 68  1  0  0  0  0
 58 74  1  0  0  0  0
 59 61  1  0  0  0  0
 59 63  1  0  0  0  0
 59 69  1  0  0  0  0
 60 61  1  0  0  0  0
 60 65  1  0  0  0  0
 60 70  1  0  0  0  0
 61 71  1  0  0  0  0
 61 77  1  0  0  0  0
 72 73  1  0  0  0  0
 72 74  1  0  0  0  0
 72 79  1  0  0  0  0
 74 75  1  0  0  0  0
 74 76  1  0  0  0  0
 79 92  1  0  0  0  0
 80 81  1  0  0  0  0
 80 92  3  0  0  0  0
 81 82  2  0  0  0  0
 81 83  1  0  0  0  0
 82 84  1  0  0  0  0
 82 85  1  0  0  0  0
 83 86  2  0  0  0  0
 83 87  1  0  0  0  0
 84 88  2  0  0  0  0
 84 89  1  0  0  0  0
 86 88  1  0  0  0  0
 86 90  1  0  0  0  0
 88 91  1  0  0  0  0
M  END
$$$$
mol089
Optimized Catalyst 089
Optimized Catalyst 089
 92 99  0  0  0  0  0  0  0  0999 V2000
```

```
   -5.1064   -2.3124   -0.8799 P   0  0  0  0  0  0  0  0  0  0  0  0
   -5.8279   -0.8813    0.0497 C   0  0  0  0  0  0  0  0  0  0  0  0
   -6.8797   -0.1466   -0.4990 C   0  0  0  0  0  0  0  0  0  0  0  0
   -5.3204   -0.5744    1.3207 C   0  0  0  0  0  0  0  0  0  0  0  0
   -7.4478    0.8984    0.2441 C   0  0  0  0  0  0  0  0  0  0  0  0
   -7.2811   -0.3504   -1.4969 H   0  0  0  0  0  0  0  0  0  0  0  0
   -5.8955    0.4650    2.0543 C   0  0  0  0  0  0  0  0  0  0  0  0
   -4.4848   -1.1235    1.7659 H   0  0  0  0  0  0  0  0  0  0  0  0
   -6.9632    1.1997    1.5181 C   0  0  0  0  0  0  0  0  0  0  0  0
   -8.2774    1.4738   -0.1761 H   0  0  0  0  0  0  0  0  0  0  0  0
   -5.5193    0.7061    3.0516 H   0  0  0  0  0  0  0  0  0  0  0  0
   -7.4148    2.0089    2.1002 H   0  0  0  0  0  0  0  0  0  0  0  0
   -5.9130   -2.4001   -2.5459 C   0  0  0  0  0  0  0  0  0  0  0  0
   -7.2726   -2.7143   -2.6335 C   0  0  0  0  0  0  0  0  0  0  0  0
   -5.1451   -2.1785   -3.6932 C   0  0  0  0  0  0  0  0  0  0  0  0
   -7.8753   -2.7814   -3.8951 C   0  0  0  0  0  0  0  0  0  0  0  0
   -7.8816   -2.9203   -1.7475 H   0  0  0  0  0  0  0  0  0  0  0  0
   -5.7557   -2.2475   -4.9492 C   0  0  0  0  0  0  0  0  0  0  0  0
   -4.0647   -1.9628   -3.6425 H   0  0  0  0  0  0  0  0  0  0  0  0
   -7.1206   -2.5463   -5.0491 C   0  0  0  0  0  0  0  0  0  0  0  0
   -8.9387   -3.0211   -3.9786 H   0  0  0  0  0  0  0  0  0  0  0  0
   -5.1703   -2.0714   -5.8551 H   0  0  0  0  0  0  0  0  0  0  0  0
   -7.5954   -2.6015   -6.0335 H   0  0  0  0  0  0  0  0  0  0  0  0
   -5.6065   -3.8800   -0.0235 C   0  0  0  0  0  0  0  0  0  0  0  0
   -5.9981   -3.8848    1.3161 C   0  0  0  0  0  0  0  0  0  0  0  0
   -5.6257   -5.0578   -0.7831 C   0  0  0  0  0  0  0  0  0  0  0  0
   -6.3884   -5.0918    1.9113 C   0  0  0  0  0  0  0  0  0  0  0  0
   -6.0300   -2.9753    1.9215 H   0  0  0  0  0  0  0  0  0  0  0  0
   -6.0157   -6.2564   -0.1804 C   0  0  0  0  0  0  0  0  0  0  0  0
   -5.3569   -5.0702   -1.8438 H   0  0  0  0  0  0  0  0  0  0  0  0
   -6.3948   -6.2750    1.1682 C   0  0  0  0  0  0  0  0  0  0  0  0
   -6.7014   -5.1026    2.9585 H   0  0  0  0  0  0  0  0  0  0  0  0
   -6.0311   -7.1801   -0.7630 H   0  0  0  0  0  0  0  0  0  0  0  0
   -6.7049   -7.2139    1.6352 H   0  0  0  0  0  0  0  0  0  0  0  0
   -2.5563    0.8283   -0.4464 C   0  0  0  0  0  0  0  0  0  0  0  0
   -1.4358    1.3623    0.3883 C   0  0  0  0  0  0  0  0  0  0  0  0
   -1.1146    1.9629   -3.0795 C   0  0  0  0  0  0  0  0  0  0  0  0
   -0.9582    2.7528   -0.1051 C   0  0  0  0  0  0  0  0  0  0  0  0
    0.0552    2.7788   -2.5078 C   0  0  0  0  0  0  0  0  0  0  0  0
    0.2971    2.7034   -0.9905 C   0  0  0  0  0  0  0  0  0  0  0  0
   -0.5665    0.6551    0.3861 H   0  0  0  0  0  0  0  0  0  0  0  0
   -1.7854    3.2645   -0.6505 H   0  0  0  0  0  0  0  0  0  0  0  0
   -2.0705    2.4961   -2.8549 H   0  0  0  0  0  0  0  0  0  0  0  0
   -0.1259    3.8502   -2.7827 H   0  0  0  0  0  0  0  0  0  0  0  0
   -3.5522    0.9006    0.0311 H   0  0  0  0  0  0  0  0  0  0  0  0
   -1.7792    1.4686    1.4503 H   0  0  0  0  0  0  0  0  0  0  0  0
   -0.9986    1.9381   -4.1926 H   0  0  0  0  0  0  0  0  0  0  0  0
   -0.7207    3.3623    0.8041 H   0  0  0  0  0  0  0  0  0  0  0  0
    0.9988    2.4532   -3.0147 H   0  0  0  0  0  0  0  0  0  0  0  0
    0.9413    3.5826   -0.7206 H   0  0  0  0  0  0  0  0  0  0  0  0
   -2.4563    0.3402   -1.7177 C   0  0  0  0  0  0  0  0  0  0  0  0
   -3.3835    0.2014   -2.3212 H   0  0  0  0  0  0  0  0  0  0  0  0
   -1.2068    0.5089   -2.5629 C   0  0  0  0  0  0  0  0  0  0  0  0
   -0.2879    0.2760   -1.9633 H   0  0  0  0  0  0  0  0  0  0  0  0
   -1.2449   -0.1887   -3.4408 H   0  0  0  0  0  0  0  0  0  0  0  0
    0.4892   -2.3773   -1.9330 C   0  0  0  0  0  0  0  0  0  0  0  0
    1.5358   -2.0559   -2.9599 C   0  0  0  0  0  0  0  0  0  0  0  0
    2.8151   -2.3662    0.3463 C   0  0  0  0  0  0  0  0  0  0  0  0
    2.8839   -2.7672   -2.7047 C   0  0  0  0  0  0  0  0  0  0  0  0
    4.0955   -2.1613   -0.4785 C   0  0  0  0  0  0  0  0  0  0  0  0
    3.9260   -1.8950   -1.9842 C   0  0  0  0  0  0  0  0  0  0  0  0
    1.6972   -0.9484   -2.9886 H   0  0  0  0  0  0  0  0  0  0  0  0
    2.7065   -3.7005   -2.1218 H   0  0  0  0  0  0  0  0  0  0  0  0
    2.4377   -3.4032    0.1739 H   0  0  0  0  0  0  0  0  0  0  0  0
    4.7225   -3.0806   -0.3522 H   0  0  0  0  0  0  0  0  0  0  0  0
```

```
   -0.3691   -2.9588   -2.3334 H   0  0  0  0  0  0  0  0  0  0  0  0
    1.1640   -2.3664   -3.9687 H   0  0  0  0  0  0  0  0  0  0  0  0
    3.0906   -2.2810    1.4270 H   0  0  0  0  0  0  0  0  0  0  0  0
    3.3050   -3.0603   -3.6990 H   0  0  0  0  0  0  0  0  0  0  0  0
    4.6630   -1.2990   -0.0463 H   0  0  0  0  0  0  0  0  0  0  0  0
    4.9227   -2.0808   -2.4629 H   0  0  0  0  0  0  0  0  0  0  0  0
    0.5350   -2.0536   -0.6297 C   0  0  0  0  0  0  0  0  0  0  0  0
   -0.2806   -2.3653    0.0581 H   0  0  0  0  0  0  0  0  0  0  0  0
    1.6803   -1.3604    0.0499 C   0  0  0  0  0  0  0  0  0  0  0  0
    2.0705   -0.5366   -0.5964 H   0  0  0  0  0  0  0  0  0  0  0  0
    1.3352   -0.9092    1.0106 H   0  0  0  0  0  0  0  0  0  0  0  0
    3.6869   -0.8166   -2.1391 H   0  0  0  0  0  0  0  0  0  0  0  0
    0.8891    1.7871   -0.7575 H   0  0  0  0  0  0  0  0  0  0  0  0
   -2.4343   -5.0540   -0.5773 C   0  0  0  0  0  0  0  0  0  0  0  0
   -2.4465   -6.4348   -0.2358 C   0  0  0  0  0  0  0  0  0  0  0  0
   -2.7502   -6.8105    1.0897 C   0  0  0  0  0  0  0  0  0  0  0  0
   -2.1776   -7.4102   -1.2164 C   0  0  0  0  0  0  0  0  0  0  0  0
   -2.7846   -8.1635    1.4236 C   0  0  0  0  0  0  0  0  0  0  0  0
   -2.9585   -6.0482    1.8424 H   0  0  0  0  0  0  0  0  0  0  0  0
   -2.2200   -8.7594   -0.8641 C   0  0  0  0  0  0  0  0  0  0  0  0
   -1.9368   -7.1132   -2.2389 H   0  0  0  0  0  0  0  0  0  0  0  0
   -2.5221   -9.1362    0.4504 C   0  0  0  0  0  0  0  0  0  0  0  0
   -3.0109   -8.4662    2.4496 H   0  0  0  0  0  0  0  0  0  0  0  0
   -2.0121   -9.5258   -1.6166 H   0  0  0  0  0  0  0  0  0  0  0  0
   -2.5504  -10.1970    0.7197 H   0  0  0  0  0  0  0  0  0  0  0  0
   -2.4639   -3.9171   -0.8458 N   0  0  0  0  0  0  0  0  0  0  0  0
   -2.5346   -2.0168   -1.1957 Ir  0  0  0  0  0  0  0  0  0  0  0  0
  1  2  1  0  0  0  0
  1 13  1  0  0  0  0
  1 24  1  0  0  0  0
  1 92  1  0  0  0  0
  2  3  2  0  0  0  0
  2  4  1  0  0  0  0
  3  5  1  0  0  0  0
  3  6  1  0  0  0  0
  4  7  2  0  0  0  0
  4  8  1  0  0  0  0
  5  9  2  0  0  0  0
  5 10  1  0  0  0  0
  7  9  1  0  0  0  0
  7 11  1  0  0  0  0
  9 12  1  0  0  0  0
 13 14  2  0  0  0  0
 13 15  1  0  0  0  0
 14 16  1  0  0  0  0
 14 17  1  0  0  0  0
 15 18  2  0  0  0  0
 15 19  1  0  0  0  0
 16 20  2  0  0  0  0
 16 21  1  0  0  0  0
 18 20  1  0  0  0  0
 18 22  1  0  0  0  0
 20 23  1  0  0  0  0
 24 25  2  0  0  0  0
 24 26  1  0  0  0  0
 25 27  1  0  0  0  0
 25 28  1  0  0  0  0
 26 29  2  0  0  0  0
 26 30  1  0  0  0  0
 27 31  2  0  0  0  0
 27 32  1  0  0  0  0
 29 31  1  0  0  0  0
 29 33  1  0  0  0  0
 31 34  1  0  0  0  0
 35 36  1  0  0  0  0
```

```
 35 45  1  0  0  0  0
 35 51  2  0  0  0  0
 35 92  1  0  0  0  0
 36 38  1  0  0  0  0
 36 41  1  0  0  0  0
 36 46  1  0  0  0  0
 37 39  1  0  0  0  0
 37 43  1  0  0  0  0
 37 47  1  0  0  0  0
 37 53  1  0  0  0  0
 38 40  1  0  0  0  0
 38 42  1  0  0  0  0
 38 48  1  0  0  0  0
 39 40  1  0  0  0  0
 39 44  1  0  0  0  0
 39 49  1  0  0  0  0
 40 50  1  0  0  0  0
 40 78  1  0  0  0  0
 51 52  1  0  0  0  0
 51 53  1  0  0  0  0
 51 92  1  0  0  0  0
 53 54  1  0  0  0  0
 53 55  1  0  0  0  0
 56 57  1  0  0  0  0
 56 66  1  0  0  0  0
 56 72  2  0  0  0  0
 56 92  1  0  0  0  0
 57 59  1  0  0  0  0
 57 62  1  0  0  0  0
 57 67  1  0  0  0  0
 58 60  1  0  0  0  0
 58 64  1  0  0  0  0
 58 68  1  0  0  0  0
 58 74  1  0  0  0  0
 59 61  1  0  0  0  0
 59 63  1  0  0  0  0
 59 69  1  0  0  0  0
 60 61  1  0  0  0  0
 60 65  1  0  0  0  0
 60 70  1  0  0  0  0
 61 71  1  0  0  0  0
 61 77  1  0  0  0  0
 72 73  1  0  0  0  0
 72 74  1  0  0  0  0
 72 92  1  0  0  0  0
 74 75  1  0  0  0  0
 74 76  1  0  0  0  0
 79 80  1  0  0  0  0
 79 91  3  0  0  0  0
 80 81  2  0  0  0  0
 80 82  1  0  0  0  0
 81 83  1  0  0  0  0
 81 84  1  0  0  0  0
 82 85  2  0  0  0  0
 82 86  1  0  0  0  0
 83 87  2  0  0  0  0
 83 88  1  0  0  0  0
 85 87  1  0  0  0  0
 85 89  1  0  0  0  0
 87 90  1  0  0  0  0
 91 92  1  0  0  0  0
M  END
$$$$
mol090
Optimized Catalyst 090
```

```
Optimized Catalyst 090
 71 76  0  0  0  0  0  0  0  0999 V2000
   -1.6224   -0.2491   -0.6387 C   0  0  0  0  0  0  0  0  0  0  0  0
   -1.2342    0.9527    0.1767 C   0  0  0  0  0  0  0  0  0  0  0  0
   -1.8502    1.7068   -3.2476 C   0  0  0  0  0  0  0  0  0  0  0  0
   -1.9769    2.2440   -0.2353 C   0  0  0  0  0  0  0  0  0  0  0  0
   -1.5587    3.0968   -2.6612 C   0  0  0  0  0  0  0  0  0  0  0  0
   -1.1699    3.1576   -1.1738 C   0  0  0  0  0  0  0  0  0  0  0  0
   -0.1298    1.1125    0.0941 H   0  0  0  0  0  0  0  0  0  0  0  0
   -2.9475    1.9750   -0.7128 H   0  0  0  0  0  0  0  0  0  0  0  0
   -2.8631    1.3757   -2.9138 H   0  0  0  0  0  0  0  0  0  0  0  0
   -2.4749    3.7214   -2.8140 H   0  0  0  0  0  0  0  0  0  0  0  0
   -2.1308   -1.0527   -0.0530 H   0  0  0  0  0  0  0  0  0  0  0  0
   -1.4636    0.7478    1.2526 H   0  0  0  0  0  0  0  0  0  0  0  0
   -1.8775    1.8067   -4.3610 H   0  0  0  0  0  0  0  0  0  0  0  0
   -2.2032    2.8180    0.6973 H   0  0  0  0  0  0  0  0  0  0  0  0
   -0.7261    3.5627   -3.2458 H   0  0  0  0  0  0  0  0  0  0  0  0
   -1.3161    4.2172   -0.8395 H   0  0  0  0  0  0  0  0  0  0  0  0
   -1.4335   -0.4042   -1.9616 C   0  0  0  0  0  0  0  0  0  0  0  0
   -1.8228   -1.3124   -2.4846 H   0  0  0  0  0  0  0  0  0  0  0  0
   -0.8186    0.6195   -2.8756 C   0  0  0  0  0  0  0  0  0  0  0  0
    0.0629    1.0935   -2.3798 H   0  0  0  0  0  0  0  0  0  0  0  0
   -0.4607    0.1230   -3.8094 H   0  0  0  0  0  0  0  0  0  0  0  0
    0.8055   -3.7095   -1.4401 C   0  0  0  0  0  0  0  0  0  0  0  0
    1.2238   -2.9443   -2.6721 C   0  0  0  0  0  0  0  0  0  0  0  0
    2.7728   -1.7058    0.2887 C   0  0  0  0  0  0  0  0  0  0  0  0
    2.7151   -2.5361   -2.6673 C   0  0  0  0  0  0  0  0  0  0  0  0
    3.4439   -0.8602   -0.8047 C   0  0  0  0  0  0  0  0  0  0  0  0
    2.9680   -1.0769   -2.2523 C   0  0  0  0  0  0  0  0  0  0  0  0
    0.5637   -2.0296   -2.7678 H   0  0  0  0  0  0  0  0  0  0  0  0
    3.2836   -3.2235   -1.9971 H   0  0  0  0  0  0  0  0  0  0  0  0
    3.1872   -2.7433    0.2495 H   0  0  0  0  0  0  0  0  0  0  0  0
    4.5431   -1.0728   -0.7590 H   0  0  0  0  0  0  0  0  0  0  0  0
    0.7094   -4.8023   -1.5802 H   0  0  0  0  0  0  0  0  0  0  0  0
    1.0327   -3.5858   -3.5721 H   0  0  0  0  0  0  0  0  0  0  0  0
    3.0527   -1.2682    1.2799 H   0  0  0  0  0  0  0  0  0  0  0  0
    3.1071   -2.6752   -3.7066 H   0  0  0  0  0  0  0  0  0  0  0  0
    3.2958    0.2220   -0.5601 H   0  0  0  0  0  0  0  0  0  0  0  0
    3.7649   -0.6599   -2.9230 H   0  0  0  0  0  0  0  0  0  0  0  0
    0.7835   -3.1750   -0.1362 C   0  0  0  0  0  0  0  0  0  0  0  0
    0.6689   -3.8521    0.7303 H   0  0  0  0  0  0  0  0  0  0  0  0
    1.2332   -1.7748    0.1996 C   0  0  0  0  0  0  0  0  0  0  0  0
    0.8589   -1.0641   -0.5909 H   0  0  0  0  0  0  0  0  0  0  0  0
    0.7800   -1.4696    1.1771 H   0  0  0  0  0  0  0  0  0  0  0  0
    2.0469   -0.4730   -2.4282 H   0  0  0  0  0  0  0  0  0  0  0  0
   -0.0821    2.9322   -1.0729 H   0  0  0  0  0  0  0  0  0  0  0  0
   -2.0686   -5.4279   -0.0715 C   0  0  0  0  0  0  0  0  0  0  0  0
   -2.6440   -6.6578    0.3711 C   0  0  0  0  0  0  0  0  0  0  0  0
   -2.3381   -7.1527    1.6524 C   0  0  0  0  0  0  0  0  0  0  0  0
   -3.5116   -7.3638   -0.4831 C   0  0  0  0  0  0  0  0  0  0  0  0
   -2.9047   -8.3578    2.0720 C   0  0  0  0  0  0  0  0  0  0  0  0
   -1.6672   -6.6005    2.3101 H   0  0  0  0  0  0  0  0  0  0  0  0
   -4.0696   -8.5670   -0.0475 C   0  0  0  0  0  0  0  0  0  0  0  0
   -3.7428   -6.9745   -1.4746 H   0  0  0  0  0  0  0  0  0  0  0  0
   -3.7679   -9.0637    1.2260 C   0  0  0  0  0  0  0  0  0  0  0  0
   -2.6728   -8.7509    3.0648 H   0  0  0  0  0  0  0  0  0  0  0  0
   -4.7428   -9.1235   -0.7036 H   0  0  0  0  0  0  0  0  0  0  0  0
   -4.2078  -10.0068    1.5609 H   0  0  0  0  0  0  0  0  0  0  0  0
   -1.5781   -4.4349   -0.4310 N   0  0  0  0  0  0  0  0  0  0  0  0
   -1.0791   -2.9524   -1.0102 Rh  0  0  0  0  0  0  0  0  0  0  0  0
   -4.8394   -2.6226   -1.2396 C   0  0  0  0  0  0  0  0  0  0  0  0
   -6.2617   -2.4769   -1.1683 C   0  0  0  0  0  0  0  0  0  0  0  0
   -7.0935   -3.3821   -1.8513 C   0  0  0  0  0  0  0  0  0  0  0  0
   -6.8147   -1.4329   -0.4045 C   0  0  0  0  0  0  0  0  0  0  0  0
   -8.4795   -3.2384   -1.7642 C   0  0  0  0  0  0  0  0  0  0  0  0
```

```
   -6.6586   -4.1875   -2.4438 H   0  0  0  0  0  0  0  0  0  0  0  0
   -8.2023   -1.3015   -0.3265 C   0  0  0  0  0  0  0  0  0  0  0  0
   -6.1636   -0.7335    0.1214 H   0  0  0  0  0  0  0  0  0  0  0  0
   -9.0331   -2.2008   -1.0050 C   0  0  0  0  0  0  0  0  0  0  0  0
   -9.1334   -3.9375   -2.2901 H   0  0  0  0  0  0  0  0  0  0  0  0
   -8.6402   -0.4951    0.2662 H   0  0  0  0  0  0  0  0  0  0  0  0
  -10.1191   -2.0916   -0.9423 H   0  0  0  0  0  0  0  0  0  0  0  0
   -3.6872   -2.7424   -1.2854 N   0  0  0  0  0  0  0  0  0  0  0  0
  1  2  1  0  0  0  0
  1 11  1  0  0  0  0
  1 17  2  0  0  0  0
  1 58  1  0  0  0  0
  2  4  1  0  0  0  0
  2  7  1  0  0  0  0
  2 12  1  0  0  0  0
  3  5  1  0  0  0  0
  3  9  1  0  0  0  0
  3 13  1  0  0  0  0
  3 19  1  0  0  0  0
  4  6  1  0  0  0  0
  4  8  1  0  0  0  0
  4 14  1  0  0  0  0
  5  6  1  0  0  0  0
  5 10  1  0  0  0  0
  5 15  1  0  0  0  0
  6 16  1  0  0  0  0
  6 44  1  0  0  0  0
 17 18  1  0  0  0  0
 17 19  1  0  0  0  0
 17 58  1  0  0  0  0
 19 20  1  0  0  0  0
 19 21  1  0  0  0  0
 22 23  1  0  0  0  0
 22 32  1  0  0  0  0
 22 38  2  0  0  0  0
 22 58  1  0  0  0  0
 23 25  1  0  0  0  0
 23 28  1  0  0  0  0
 23 33  1  0  0  0  0
 24 26  1  0  0  0  0
 24 30  1  0  0  0  0
 24 34  1  0  0  0  0
 24 40  1  0  0  0  0
 25 27  1  0  0  0  0
 25 29  1  0  0  0  0
 25 35  1  0  0  0  0
 26 27  1  0  0  0  0
 26 31  1  0  0  0  0
 26 36  1  0  0  0  0
 27 37  1  0  0  0  0
 27 43  1  0  0  0  0
 38 39  1  0  0  0  0
 38 40  1  0  0  0  0
 38 58  1  0  0  0  0
 40 41  1  0  0  0  0
 40 42  1  0  0  0  0
 45 46  1  0  0  0  0
 45 57  3  0  0  0  0
 46 47  2  0  0  0  0
 46 48  1  0  0  0  0
 47 49  1  0  0  0  0
 47 50  1  0  0  0  0
 48 51  2  0  0  0  0
 48 52  1  0  0  0  0
 49 53  2  0  0  0  0
```

```
 49 54  1  0  0  0  0
 51 53  1  0  0  0  0
 51 55  1  0  0  0  0
 53 56  1  0  0  0  0
 57 58  1  0  0  0  0
 58 71  1  0  0  0  0
 59 60  1  0  0  0  0
 59 71  3  0  0  0  0
 60 61  2  0  0  0  0
 60 62  1  0  0  0  0
 61 63  1  0  0  0  0
 61 64  1  0  0  0  0
 62 65  2  0  0  0  0
 62 66  1  0  0  0  0
 63 67  2  0  0  0  0
 63 68  1  0  0  0  0
 65 67  1  0  0  0  0
 65 69  1  0  0  0  0
 67 70  1  0  0  0  0
M  END
$$$$
mol091
Optimized Catalyst 091
Optimized Catalyst 091
 71 76  0  0  0  0  0  0  0  0999 V2000
   -2.6374    0.2954   -0.7769 C   0  0  0  0  0  0  0  0  0  0  0  0
   -1.6996    1.1789   -0.0007 C   0  0  0  0  0  0  0  0  0  0  0  0
   -1.5881    1.7101   -3.5127 C   0  0  0  0  0  0  0  0  0  0  0  0
   -1.6447    2.6164   -0.5750 C   0  0  0  0  0  0  0  0  0  0  0  0
   -0.6771    2.8294   -2.9857 C   0  0  0  0  0  0  0  0  0  0  0  0
   -0.4225    2.8800   -1.4696 C   0  0  0  0  0  0  0  0  0  0  0  0
   -0.6682    0.7467    0.0057 H   0  0  0  0  0  0  0  0  0  0  0  0
   -2.5818    2.8317   -1.1408 H   0  0  0  0  0  0  0  0  0  0  0  0
   -2.6517    1.9834   -3.3048 H   0  0  0  0  0  0  0  0  0  0  0  0
   -1.1395    3.8024   -3.2962 H   0  0  0  0  0  0  0  0  0  0  0  0
   -3.6123    0.1263   -0.2714 H   0  0  0  0  0  0  0  0  0  0  0  0
   -2.0483    1.2429    1.0627 H   0  0  0  0  0  0  0  0  0  0  0  0
   -1.4641    1.6659   -4.6246 H   0  0  0  0  0  0  0  0  0  0  0  0
   -1.6041    3.3235    0.2922 H   0  0  0  0  0  0  0  0  0  0  0  0
    0.3183    2.7502   -3.4920 H   0  0  0  0  0  0  0  0  0  0  0  0
   -0.0435    3.9109   -1.2375 H   0  0  0  0  0  0  0  0  0  0  0  0
   -2.4494   -0.1233   -2.0582 C   0  0  0  0  0  0  0  0  0  0  0  0
   -3.2778   -0.6192   -2.6182 H   0  0  0  0  0  0  0  0  0  0  0  0
   -1.2976    0.3050   -2.9322 C   0  0  0  0  0  0  0  0  0  0  0  0
   -0.3433    0.3408   -2.3518 H   0  0  0  0  0  0  0  0  0  0  0  0
   -1.1732   -0.4165   -3.7776 H   0  0  0  0  0  0  0  0  0  0  0  0
    0.3059   -2.8412   -1.7147 C   0  0  0  0  0  0  0  0  0  0  0  0
    1.2229   -2.4571   -2.8392 C   0  0  0  0  0  0  0  0  0  0  0  0
    2.6087   -1.9269    0.3737 C   0  0  0  0  0  0  0  0  0  0  0  0
    2.7013   -2.8198   -2.5606 C   0  0  0  0  0  0  0  0  0  0  0  0
    3.7905   -1.5779   -0.5432 C   0  0  0  0  0  0  0  0  0  0  0  0
    3.5477   -1.6374   -2.0609 C   0  0  0  0  0  0  0  0  0  0  0  0
    1.1473   -1.3586   -3.0371 H   0  0  0  0  0  0  0  0  0  0  0  0
    2.7523   -3.6578   -1.8273 H   0  0  0  0  0  0  0  0  0  0  0  0
    2.4712   -3.0354    0.3813 H   0  0  0  0  0  0  0  0  0  0  0  0
    4.6231   -2.2843   -0.2909 H   0  0  0  0  0  0  0  0  0  0  0  0
   -0.3544   -3.7057   -1.9506 H   0  0  0  0  0  0  0  0  0  0  0  0
    0.9035   -2.9895   -3.7716 H   0  0  0  0  0  0  0  0  0  0  0  0
    2.8828   -1.6141    1.4129 H   0  0  0  0  0  0  0  0  0  0  0  0
    3.1441   -3.1826   -3.5230 H   0  0  0  0  0  0  0  0  0  0  0  0
    4.1385   -0.5422   -0.2988 H   0  0  0  0  0  0  0  0  0  0  0  0
    4.5555   -1.7039   -2.5504 H   0  0  0  0  0  0  0  0  0  0  0  0
    0.2902   -2.2864   -0.4823 C   0  0  0  0  0  0  0  0  0  0  0  0
   -0.3426   -2.7161    0.3437 H   0  0  0  0  0  0  0  0  0  0  0  0
    1.2704   -1.2485   -0.0027 C   0  0  0  0  0  0  0  0  0  0  0  0
```

```
    1.4548   -0.4833   -0.7957 H   0  0  0  0  0  0  0  0  0  0  0  0
    0.8681   -0.7288    0.9009 H   0  0  0  0  0  0  0  0  0  0  0  0
    3.0896   -0.6766   -2.3953 H   0  0  0  0  0  0  0  0  0  0  0  0
    0.3976    2.1694   -1.2116 H   0  0  0  0  0  0  0  0  0  0  0  0
   -2.3872   -5.3811    0.0089 C   0  0  0  0  0  0  0  0  0  0  0  0
   -2.3927   -6.7639    0.3449 C   0  0  0  0  0  0  0  0  0  0  0  0
   -1.9821   -7.1782    1.6283 C   0  0  0  0  0  0  0  0  0  0  0  0
   -2.8031   -7.7105   -0.6172 C   0  0  0  0  0  0  0  0  0  0  0  0
   -1.9834   -8.5382    1.9396 C   0  0  0  0  0  0  0  0  0  0  0  0
   -1.6668   -6.4432    2.3709 H   0  0  0  0  0  0  0  0  0  0  0  0
   -2.7988   -9.0654   -0.2879 C   0  0  0  0  0  0  0  0  0  0  0  0
   -3.1205   -7.3858   -1.6093 H   0  0  0  0  0  0  0  0  0  0  0  0
   -2.3909   -9.4799    0.9865 C   0  0  0  0  0  0  0  0  0  0  0  0
   -1.6640   -8.8705    2.9317 H   0  0  0  0  0  0  0  0  0  0  0  0
   -3.1133   -9.8077   -1.0273 H   0  0  0  0  0  0  0  0  0  0  0  0
   -2.3914  -10.5455    1.2386 H   0  0  0  0  0  0  0  0  0  0  0  0
   -2.3638   -4.2491   -0.2800 N   0  0  0  0  0  0  0  0  0  0  0  0
   -5.5160   -2.4102   -0.7221 C   0  0  0  0  0  0  0  0  0  0  0  0
   -6.9372   -2.4352   -0.6652 C   0  0  0  0  0  0  0  0  0  0  0  0
   -7.6666   -3.1079   -1.6670 C   0  0  0  0  0  0  0  0  0  0  0  0
   -7.6033   -1.7810    0.3920 C   0  0  0  0  0  0  0  0  0  0  0  0
   -9.0598   -3.1187   -1.6049 C   0  0  0  0  0  0  0  0  0  0  0  0
   -7.1468   -3.6139   -2.4819 H   0  0  0  0  0  0  0  0  0  0  0  0
   -8.9968   -1.8014    0.4367 C   0  0  0  0  0  0  0  0  0  0  0  0
   -7.0344   -1.2647    1.1672 H   0  0  0  0  0  0  0  0  0  0  0  0
   -9.7244   -2.4665   -0.5586 C   0  0  0  0  0  0  0  0  0  0  0  0
   -9.6358   -3.6375   -2.3768 H   0  0  0  0  0  0  0  0  0  0  0  0
   -9.5238   -1.2962    1.2517 H   0  0  0  0  0  0  0  0  0  0  0  0
  -10.8187   -2.4764   -0.5180 H   0  0  0  0  0  0  0  0  0  0  0  0
   -4.3498   -2.3650   -0.7747 N   0  0  0  0  0  0  0  0  0  0  0  0
   -2.3981   -2.3617   -0.7976 Ir  0  0  0  0  0  0  0  0  0  0  0  0
  1  2  1  0  0  0  0
  1 11  1  0  0  0  0
  1 17  2  0  0  0  0
  1 71  1  0  0  0  0
  2  4  1  0  0  0  0
  2  7  1  0  0  0  0
  2 12  1  0  0  0  0
  3  5  1  0  0  0  0
  3  9  1  0  0  0  0
  3 13  1  0  0  0  0
  3 19  1  0  0  0  0
  4  6  1  0  0  0  0
  4  8  1  0  0  0  0
  4 14  1  0  0  0  0
  5  6  1  0  0  0  0
  5 10  1  0  0  0  0
  5 15  1  0  0  0  0
  6 16  1  0  0  0  0
  6 44  1  0  0  0  0
 17 18  1  0  0  0  0
 17 19  1  0  0  0  0
 17 71  1  0  0  0  0
 19 20  1  0  0  0  0
 19 21  1  0  0  0  0
 22 23  1  0  0  0  0
 22 32  1  0  0  0  0
 22 38  2  0  0  0  0
 22 71  1  0  0  0  0
 23 25  1  0  0  0  0
 23 28  1  0  0  0  0
 23 33  1  0  0  0  0
 24 26  1  0  0  0  0
 24 30  1  0  0  0  0
 24 34  1  0  0  0  0
```

```
 24 40  1  0  0  0  0
 25 27  1  0  0  0  0
 25 29  1  0  0  0  0
 25 35  1  0  0  0  0
 26 27  1  0  0  0  0
 26 31  1  0  0  0  0
 26 36  1  0  0  0  0
 27 37  1  0  0  0  0
 27 43  1  0  0  0  0
 38 39  1  0  0  0  0
 38 40  1  0  0  0  0
 38 71  1  0  0  0  0
 40 41  1  0  0  0  0
 40 42  1  0  0  0  0
 45 46  1  0  0  0  0
 45 57  3  0  0  0  0
 46 47  2  0  0  0  0
 46 48  1  0  0  0  0
 47 49  1  0  0  0  0
 47 50  1  0  0  0  0
 48 51  2  0  0  0  0
 48 52  1  0  0  0  0
 49 53  2  0  0  0  0
 49 54  1  0  0  0  0
 51 53  1  0  0  0  0
 51 55  1  0  0  0  0
 53 56  1  0  0  0  0
 57 71  1  0  0  0  0
 58 59  1  0  0  0  0
 58 70  3  0  0  0  0
 59 60  2  0  0  0  0
 59 61  1  0  0  0  0
 60 62  1  0  0  0  0
 60 63  1  0  0  0  0
 61 64  2  0  0  0  0
 61 65  1  0  0  0  0
 62 66  2  0  0  0  0
 62 67  1  0  0  0  0
 64 66  1  0  0  0  0
 64 68  1  0  0  0  0
 66 69  1  0  0  0  0
 70 71  1  0  0  0  0
M  END
$$$$
mol092
Optimized Catalyst 092
Optimized Catalyst 092
113122  0  0  0  0  0  0  0  0999 V2000
   -4.5818   -2.4261   -1.2092 P   0  0  0  0  0  0  0  0  0  0  0  0
   -2.3106   -4.7093   -0.4412 P   0  0  0  0  0  0  0  0  0  0  0  0
   -0.5739   -5.3186   -0.3095 C   0  0  0  0  0  0  0  0  0  0  0  0
    0.0858   -5.6781   -1.4914 C   0  0  0  0  0  0  0  0  0  0  0  0
    0.0113   -5.5409    0.9413 C   0  0  0  0  0  0  0  0  0  0  0  0
    1.3410   -6.2899   -1.4147 C   0  0  0  0  0  0  0  0  0  0  0  0
   -0.3516   -5.4857   -2.4774 H   0  0  0  0  0  0  0  0  0  0  0  0
    1.2746   -6.1388    1.0054 C   0  0  0  0  0  0  0  0  0  0  0  0
   -0.4838   -5.2710    1.8775 H   0  0  0  0  0  0  0  0  0  0  0  0
    1.9366   -6.5162   -0.1685 C   0  0  0  0  0  0  0  0  0  0  0  0
    1.8575   -6.5903   -2.3294 H   0  0  0  0  0  0  0  0  0  0  0  0
    1.7448   -6.3126    1.9754 H   0  0  0  0  0  0  0  0  0  0  0  0
    2.9223   -6.9854   -0.1123 H   0  0  0  0  0  0  0  0  0  0  0  0
   -3.1560   -6.0500   -1.3897 C   0  0  0  0  0  0  0  0  0  0  0  0
   -3.3851   -7.3031   -0.8180 C   0  0  0  0  0  0  0  0  0  0  0  0
   -3.4504   -5.7997   -2.7347 C   0  0  0  0  0  0  0  0  0  0  0  0
   -3.9904   -8.3007   -1.5920 C   0  0  0  0  0  0  0  0  0  0  0  0
```

```
   -3.0868   -7.5413    0.2061 H   0  0  0  0  0  0  0  0  0  0  0  0
   -4.0435   -6.8061   -3.5033 C   0  0  0  0  0  0  0  0  0  0  0  0
   -3.2128   -4.8286   -3.2070 H   0  0  0  0  0  0  0  0  0  0  0  0
   -4.3234   -8.0516   -2.9276 C   0  0  0  0  0  0  0  0  0  0  0  0
   -4.1886   -9.2820   -1.1547 H   0  0  0  0  0  0  0  0  0  0  0  0
   -4.2798   -6.6244   -4.5540 H   0  0  0  0  0  0  0  0  0  0  0  0
   -4.7899   -8.8366   -3.5291 H   0  0  0  0  0  0  0  0  0  0  0  0
   -2.9594   -4.5184    1.2732 C   0  0  0  0  0  0  0  0  0  0  0  0
   -3.9639   -5.3309    1.7956 C   0  0  0  0  0  0  0  0  0  0  0  0
   -2.3557   -3.5220    2.0552 C   0  0  0  0  0  0  0  0  0  0  0  0
   -4.3519   -5.1641    3.1331 C   0  0  0  0  0  0  0  0  0  0  0  0
   -4.4831   -6.0817    1.1935 H   0  0  0  0  0  0  0  0  0  0  0  0
   -2.7504   -3.3608    3.3841 C   0  0  0  0  0  0  0  0  0  0  0  0
   -1.5810   -2.8599    1.6450 H   0  0  0  0  0  0  0  0  0  0  0  0
   -3.7444   -4.1885    3.9258 C   0  0  0  0  0  0  0  0  0  0  0  0
   -5.1328   -5.8023    3.5523 H   0  0  0  0  0  0  0  0  0  0  0  0
   -2.2810   -2.5947    4.0056 H   0  0  0  0  0  0  0  0  0  0  0  0
   -4.0424   -4.0676    4.9709 H   0  0  0  0  0  0  0  0  0  0  0  0
   -4.8362   -0.8712   -0.2450 C   0  0  0  0  0  0  0  0  0  0  0  0
   -5.5616    0.1740   -0.8233 C   0  0  0  0  0  0  0  0  0  0  0  0
   -4.3730   -0.7829    1.0734 C   0  0  0  0  0  0  0  0  0  0  0  0
   -5.8673    1.3026   -0.0515 C   0  0  0  0  0  0  0  0  0  0  0  0
   -5.8958    0.1481   -1.8654 H   0  0  0  0  0  0  0  0  0  0  0  0
   -4.6872    0.3437    1.8372 C   0  0  0  0  0  0  0  0  0  0  0  0
   -3.7606   -1.5688    1.5217 H   0  0  0  0  0  0  0  0  0  0  0  0
   -5.4436    1.3823    1.2779 C   0  0  0  0  0  0  0  0  0  0  0  0
   -6.4336    2.1250   -0.4942 H   0  0  0  0  0  0  0  0  0  0  0  0
   -4.3335    0.4226    2.8663 H   0  0  0  0  0  0  0  0  0  0  0  0
   -5.6907    2.2611    1.8791 H   0  0  0  0  0  0  0  0  0  0  0  0
   -5.2726   -2.1819   -2.9067 C   0  0  0  0  0  0  0  0  0  0  0  0
   -6.6622   -2.1225   -3.0693 C   0  0  0  0  0  0  0  0  0  0  0  0
   -4.4058   -2.0567   -3.9945 C   0  0  0  0  0  0  0  0  0  0  0  0
   -7.1868   -1.9219   -4.3489 C   0  0  0  0  0  0  0  0  0  0  0  0
   -7.3504   -2.2331   -2.2260 H   0  0  0  0  0  0  0  0  0  0  0  0
   -4.9424   -1.8534   -5.2723 C   0  0  0  0  0  0  0  0  0  0  0  0
   -3.3057   -2.1193   -3.8739 H   0  0  0  0  0  0  0  0  0  0  0  0
   -6.3285   -1.7838   -5.4472 C   0  0  0  0  0  0  0  0  0  0  0  0
   -8.2683   -1.8719   -4.4932 H   0  0  0  0  0  0  0  0  0  0  0  0
   -4.2775   -1.7462   -6.1322 H   0  0  0  0  0  0  0  0  0  0  0  0
   -6.7446   -1.6219   -6.4454 H   0  0  0  0  0  0  0  0  0  0  0  0
   -5.6957   -3.6912   -0.4399 C   0  0  0  0  0  0  0  0  0  0  0  0
   -6.2143   -3.5111    0.8446 C   0  0  0  0  0  0  0  0  0  0  0  0
   -6.0899   -4.7861   -1.2191 C   0  0  0  0  0  0  0  0  0  0  0  0
   -7.1173   -4.4494    1.3608 C   0  0  0  0  0  0  0  0  0  0  0  0
   -5.9448   -2.6574    1.4721 H   0  0  0  0  0  0  0  0  0  0  0  0
   -6.9778   -5.7269   -0.6920 C   0  0  0  0  0  0  0  0  0  0  0  0
   -5.7311   -4.9256   -2.2441 H   0  0  0  0  0  0  0  0  0  0  0  0
   -7.4948   -5.5578    0.5982 C   0  0  0  0  0  0  0  0  0  0  0  0
   -7.5302   -4.3084    2.3616 H   0  0  0  0  0  0  0  0  0  0  0  0
   -7.2770   -6.5883   -1.2924 H   0  0  0  0  0  0  0  0  0  0  0  0
   -8.1999   -6.2876    1.0038 H   0  0  0  0  0  0  0  0  0  0  0  0
   -1.6610    0.2656   -0.4740 C   0  0  0  0  0  0  0  0  0  0  0  0
   -1.2475    1.3723    0.4504 C   0  0  0  0  0  0  0  0  0  0  0  0
   -2.9808    2.4702   -2.4665 C   0  0  0  0  0  0  0  0  0  0  0  0
   -2.2253    2.5644    0.4999 C   0  0  0  0  0  0  0  0  0  0  0  0
   -2.7242    3.7955   -1.7301 C   0  0  0  0  0  0  0  0  0  0  0  0
   -1.8670    3.7158   -0.4548 C   0  0  0  0  0  0  0  0  0  0  0  0
   -0.2317    1.7253    0.1360 H   0  0  0  0  0  0  0  0  0  0  0  0
   -3.2508    2.1968    0.2668 H   0  0  0  0  0  0  0  0  0  0  0  0
   -3.7595    1.8927   -1.9132 H   0  0  0  0  0  0  0  0  0  0  0  0
   -3.7181    4.2320   -1.4632 H   0  0  0  0  0  0  0  0  0  0  0  0
   -1.7993   -0.7103    0.0192 H   0  0  0  0  0  0  0  0  0  0  0  0
   -1.1527    0.9586    1.4849 H   0  0  0  0  0  0  0  0  0  0  0  0
   -3.3886    2.7162   -3.4768 H   0  0  0  0  0  0  0  0  0  0  0  0
   -2.2273    2.9634    1.5428 H   0  0  0  0  0  0  0  0  0  0  0  0
```

```
   -2.2149    4.4995   -2.4345 H   0  0  0  0  0  0  0  0  0  0  0  0
   -1.9958    4.6829    0.0932 H   0  0  0  0  0  0  0  0  0  0  0  0
   -1.8345    0.3387   -1.8000 C   0  0  0  0  0  0  0  0  0  0  0  0
   -2.0776   -0.5856   -2.3679 H   0  0  0  0  0  0  0  0  0  0  0  0
   -1.7351    1.5768   -2.6413 C   0  0  0  0  0  0  0  0  0  0  0  0
   -0.8141    2.1437   -2.3576 H   0  0  0  0  0  0  0  0  0  0  0  0
   -1.6339    1.2930   -3.7161 H   0  0  0  0  0  0  0  0  0  0  0  0
    0.6803   -2.4605   -2.1959 C   0  0  0  0  0  0  0  0  0  0  0  0
    1.8485   -2.4089   -3.1365 C   0  0  0  0  0  0  0  0  0  0  0  0
    2.7783   -2.8434    0.2977 C   0  0  0  0  0  0  0  0  0  0  0  0
    3.0352   -3.3088   -2.7289 C   0  0  0  0  0  0  0  0  0  0  0  0
    4.1402   -2.8725   -0.4154 C   0  0  0  0  0  0  0  0  0  0  0  0
    4.1322   -2.5864   -1.9275 C   0  0  0  0  0  0  0  0  0  0  0  0
    2.1888   -1.3441   -3.2125 H   0  0  0  0  0  0  0  0  0  0  0  0
    2.6503   -4.1700   -2.1375 H   0  0  0  0  0  0  0  0  0  0  0  0
    2.2387   -3.7975    0.0841 H   0  0  0  0  0  0  0  0  0  0  0  0
    4.5861   -3.8839   -0.2476 H   0  0  0  0  0  0  0  0  0  0  0  0
   -0.2766   -2.7770   -2.6710 H   0  0  0  0  0  0  0  0  0  0  0  0
    1.5080   -2.7247   -4.1538 H   0  0  0  0  0  0  0  0  0  0  0  0
    2.9707   -2.7959    1.3967 H   0  0  0  0  0  0  0  0  0  0  0  0
    3.4962   -3.7074   -3.6655 H   0  0  0  0  0  0  0  0  0  0  0  0
    4.8092   -2.1152    0.0647 H   0  0  0  0  0  0  0  0  0  0  0  0
    5.1281   -2.9026   -2.3293 H   0  0  0  0  0  0  0  0  0  0  0  0
    0.6860   -2.1359   -0.8962 C   0  0  0  0  0  0  0  0  0  0  0  0
   -0.2532   -2.2094   -0.3238 H   0  0  0  0  0  0  0  0  0  0  0  0
    1.8652   -1.6655   -0.0969 C   0  0  0  0  0  0  0  0  0  0  0  0
    2.4419   -0.9155   -0.6923 H   0  0  0  0  0  0  0  0  0  0  0  0
    1.5038   -1.1510    0.8258 H   0  0  0  0  0  0  0  0  0  0  0  0
    4.0482   -1.4853   -2.0879 H   0  0  0  0  0  0  0  0  0  0  0  0
   -0.7917    3.6426   -0.7439 H   0  0  0  0  0  0  0  0  0  0  0  0
   -2.4209   -2.8331   -1.6106 Rh  0  0  0  0  0  0  0  0  0  0  0  0
  1 36  1  0  0  0  0
  1 47  1  0  0  0  0
  1 58  1  0  0  0  0
  1113  1  0  0  0  0
  2  3  1  0  0  0  0
  2 14  1  0  0  0  0
  2 25  1  0  0  0  0
  2113  1  0  0  0  0
  3  4  2  0  0  0  0
  3  5  1  0  0  0  0
  4  6  1  0  0  0  0
  4  7  1  0  0  0  0
  5  8  2  0  0  0  0
  5  9  1  0  0  0  0
  6 10  2  0  0  0  0
  6 11  1  0  0  0  0
  8 10  1  0  0  0  0
  8 12  1  0  0  0  0
 10 13  1  0  0  0  0
 14 15  2  0  0  0  0
 14 16  1  0  0  0  0
 15 17  1  0  0  0  0
 15 18  1  0  0  0  0
 16 19  2  0  0  0  0
 16 20  1  0  0  0  0
 17 21  2  0  0  0  0
 17 22  1  0  0  0  0
 19 21  1  0  0  0  0
 19 23  1  0  0  0  0
 21 24  1  0  0  0  0
 25 26  2  0  0  0  0
 25 27  1  0  0  0  0
 26 28  1  0  0  0  0
 26 29  1  0  0  0  0
```

```
 27 30  2  0  0  0  0
 27 31  1  0  0  0  0
 28 32  2  0  0  0  0
 28 33  1  0  0  0  0
 30 32  1  0  0  0  0
 30 34  1  0  0  0  0
 32 35  1  0  0  0  0
 36 37  2  0  0  0  0
 36 38  1  0  0  0  0
 37 39  1  0  0  0  0
 37 40  1  0  0  0  0
 38 41  2  0  0  0  0
 38 42  1  0  0  0  0
 39 43  2  0  0  0  0
 39 44  1  0  0  0  0
 41 43  1  0  0  0  0
 41 45  1  0  0  0  0
 43 46  1  0  0  0  0
 47 48  2  0  0  0  0
 47 49  1  0  0  0  0
 48 50  1  0  0  0  0
 48 51  1  0  0  0  0
 49 52  2  0  0  0  0
 49 53  1  0  0  0  0
 50 54  2  0  0  0  0
 50 55  1  0  0  0  0
 52 54  1  0  0  0  0
 52 56  1  0  0  0  0
 54 57  1  0  0  0  0
 58 59  2  0  0  0  0
 58 60  1  0  0  0  0
 59 61  1  0  0  0  0
 59 62  1  0  0  0  0
 60 63  2  0  0  0  0
 60 64  1  0  0  0  0
 61 65  2  0  0  0  0
 61 66  1  0  0  0  0
 63 65  1  0  0  0  0
 63 67  1  0  0  0  0
 65 68  1  0  0  0  0
 69 70  1  0  0  0  0
 69 79  1  0  0  0  0
 69 85  2  0  0  0  0
 69113  1  0  0  0  0
 70 72  1  0  0  0  0
 70 75  1  0  0  0  0
 70 80  1  0  0  0  0
 71 73  1  0  0  0  0
 71 77  1  0  0  0  0
 71 81  1  0  0  0  0
 71 87  1  0  0  0  0
 72 74  1  0  0  0  0
 72 76  1  0  0  0  0
 72 82  1  0  0  0  0
 73 74  1  0  0  0  0
 73 78  1  0  0  0  0
 73 83  1  0  0  0  0
 74 84  1  0  0  0  0
 74112  1  0  0  0  0
 85 86  1  0  0  0  0
 85 87  1  0  0  0  0
 85113  1  0  0  0  0
 87 88  1  0  0  0  0
 87 89  1  0  0  0  0
 90 91  1  0  0  0  0
```

```
 90100  1  0  0  0  0
 90106  2  0  0  0  0
 90113  1  0  0  0  0
 91 93  1  0  0  0  0
 91 96  1  0  0  0  0
 91101  1  0  0  0  0
 92 94  1  0  0  0  0
 92 98  1  0  0  0  0
 92102  1  0  0  0  0
 92108  1  0  0  0  0
 93 95  1  0  0  0  0
 93 97  1  0  0  0  0
 93103  1  0  0  0  0
 94 95  1  0  0  0  0
 94 99  1  0  0  0  0
 94104  1  0  0  0  0
 95105  1  0  0  0  0
 95111  1  0  0  0  0
106107  1  0  0  0  0
106108  1  0  0  0  0
106113  1  0  0  0  0
108109  1  0  0  0  0
108110  1  0  0  0  0
M  END
$$$$
mol093
Optimized Catalyst 093
Optimized Catalyst 093
 71 76  0  0  0  0  0  0  0  0999 V2000
   -0.9611    0.3516   -1.5637 C   0  0  0  0  0  0  0  0  0  0  0  0
    0.2126   -0.5703   -1.1722 C   0  0  0  0  0  0  0  0  0  0  0  0
   -2.2118    0.1348   -0.7449 C   0  0  0  0  0  0  0  0  0  0  0  0
   -1.0808    0.1714    1.5547 C   0  0  0  0  0  0  0  0  0  0  0  0
   -2.2731    0.0293    0.6375 C   0  0  0  0  0  0  0  0  0  0  0  0
   -0.4098   -1.1872    1.8484 C   0  0  0  0  0  0  0  0  0  0  0  0
   -3.1489    0.2560   -1.3217 H   0  0  0  0  0  0  0  0  0  0  0  0
   -3.2543    0.0210    1.1462 H   0  0  0  0  0  0  0  0  0  0  0  0
   -1.2097    0.1659   -2.6424 H   0  0  0  0  0  0  0  0  0  0  0  0
   -0.6481    1.4244   -1.4570 H   0  0  0  0  0  0  0  0  0  0  0  0
    0.8504   -0.7445   -2.0782 H   0  0  0  0  0  0  0  0  0  0  0  0
   -0.9240   -1.6454    2.7361 H   0  0  0  0  0  0  0  0  0  0  0  0
   -1.4167    0.6221    2.5252 H   0  0  0  0  0  0  0  0  0  0  0  0
   -0.3436    0.8813    1.0998 H   0  0  0  0  0  0  0  0  0  0  0  0
   -0.4836   -2.1788    0.7076 C   0  0  0  0  0  0  0  0  0  0  0  0
   -0.2548   -1.9055   -0.6393 C   0  0  0  0  0  0  0  0  0  0  0  0
   -0.5810   -3.2406    1.0349 H   0  0  0  0  0  0  0  0  0  0  0  0
   -0.2241   -2.7429   -1.3715 H   0  0  0  0  0  0  0  0  0  0  0  0
    0.8588   -0.0730   -0.4044 H   0  0  0  0  0  0  0  0  0  0  0  0
    0.6692   -1.0253    2.1094 H   0  0  0  0  0  0  0  0  0  0  0  0
   -3.9479   -2.0279   -0.4596 N   0  0  0  0  0  0  0  0  0  0  0  0
   -5.0876   -2.2027   -0.6175 C   0  0  0  0  0  0  0  0  0  0  0  0
   -6.4896   -2.3663   -0.8180 C   0  0  0  0  0  0  0  0  0  0  0  0
   -6.9612   -3.1729   -1.8681 C   0  0  0  0  0  0  0  0  0  0  0  0
   -7.4058   -1.7152    0.0309 C   0  0  0  0  0  0  0  0  0  0  0  0
   -8.3346   -3.3189   -2.0660 C   0  0  0  0  0  0  0  0  0  0  0  0
   -6.2550   -3.6811   -2.5259 H   0  0  0  0  0  0  0  0  0  0  0  0
   -8.7715   -1.8755   -0.1807 C   0  0  0  0  0  0  0  0  0  0  0  0
   -7.0457   -1.0917    0.8494 H   0  0  0  0  0  0  0  0  0  0  0  0
   -9.2644   -2.6765   -1.2319 C   0  0  0  0  0  0  0  0  0  0  0  0
   -8.6691   -3.9493   -2.8918 H   0  0  0  0  0  0  0  0  0  0  0  0
   -9.4674   -1.3648    0.4881 H   0  0  0  0  0  0  0  0  0  0  0  0
   -2.3030   -4.3975   -0.6547 N   0  0  0  0  0  0  0  0  0  0  0  0
   -2.4065   -5.5322   -0.8737 C   0  0  0  0  0  0  0  0  0  0  0  0
   -2.5292   -6.9290   -1.1451 C   0  0  0  0  0  0  0  0  0  0  0  0
   -3.4801   -7.7019   -0.4570 C   0  0  0  0  0  0  0  0  0  0  0  0
```

```
   -1.6969   -7.5378   -2.1036 C   0  0  0  0  0  0  0  0  0  0  0  0
   -3.6007   -9.0636   -0.7346 C   0  0  0  0  0  0  0  0  0  0  0  0
   -4.1210   -7.2373    0.2939 H   0  0  0  0  0  0  0  0  0  0  0  0
   -1.8286   -8.8979   -2.3658 C   0  0  0  0  0  0  0  0  0  0  0  0
   -0.9524   -6.9454   -2.6369 H   0  0  0  0  0  0  0  0  0  0  0  0
   -2.7862   -9.6860   -1.6950 C   0  0  0  0  0  0  0  0  0  0  0  0
   -4.3460   -9.6379   -0.1820 H   0  0  0  0  0  0  0  0  0  0  0  0
   -1.1705   -9.3541   -3.1076 H   0  0  0  0  0  0  0  0  0  0  0  0
   -2.2863   -1.9837   -0.2344 Rh  0  0  0  0  0  0  0  0  0  0  0  0
   -2.8828  -11.1848   -2.0151 C   0  0  0  0  0  0  0  0  0  0  0  0
  -10.7827   -2.8217   -1.4142 C   0  0  0  0  0  0  0  0  0  0  0  0
  -11.1496   -3.6846   -2.6387 C   0  0  0  0  0  0  0  0  0  0  0  0
  -12.2593   -3.7414   -2.7358 H   0  0  0  0  0  0  0  0  0  0  0  0
  -10.7507   -3.2328   -3.5730 H   0  0  0  0  0  0  0  0  0  0  0  0
  -10.7681   -4.7219   -2.5212 H   0  0  0  0  0  0  0  0  0  0  0  0
  -11.3518   -3.4953   -0.1467 C   0  0  0  0  0  0  0  0  0  0  0  0
  -11.2414   -2.8405    0.7436 H   0  0  0  0  0  0  0  0  0  0  0  0
  -12.4367   -3.7041   -0.2881 H   0  0  0  0  0  0  0  0  0  0  0  0
  -10.8261   -4.4566    0.0436 H   0  0  0  0  0  0  0  0  0  0  0  0
  -11.3908   -1.4158   -1.6082 C   0  0  0  0  0  0  0  0  0  0  0  0
  -10.8813   -0.8925   -2.4470 H   0  0  0  0  0  0  0  0  0  0  0  0
  -12.4744   -1.5060   -1.8509 H   0  0  0  0  0  0  0  0  0  0  0  0
  -11.2990   -0.8011   -0.6881 H   0  0  0  0  0  0  0  0  0  0  0  0
   -2.9800  -11.3690   -3.5456 C   0  0  0  0  0  0  0  0  0  0  0  0
   -2.0145  -11.1431   -4.0454 H   0  0  0  0  0  0  0  0  0  0  0  0
   -3.2407  -12.4259   -3.7824 H   0  0  0  0  0  0  0  0  0  0  0  0
   -3.7721  -10.7077   -3.9599 H   0  0  0  0  0  0  0  0  0  0  0  0
   -4.1187  -11.8452   -1.3709 C   0  0  0  0  0  0  0  0  0  0  0  0
   -4.0512  -11.8239   -0.2617 H   0  0  0  0  0  0  0  0  0  0  0  0
   -5.0508  -11.3416   -1.7074 H   0  0  0  0  0  0  0  0  0  0  0  0
   -4.1734  -12.9150   -1.6813 H   0  0  0  0  0  0  0  0  0  0  0  0
   -1.6078  -11.8563   -1.4620 C   0  0  0  0  0  0  0  0  0  0  0  0
   -1.5276  -11.6821   -0.3662 H   0  0  0  0  0  0  0  0  0  0  0  0
   -1.6535  -12.9532   -1.6470 H   0  0  0  0  0  0  0  0  0  0  0  0
   -0.6967  -11.4565   -1.9562 H   0  0  0  0  0  0  0  0  0  0  0  0
  1  2  1  0  0  0  0
  1  3  1  0  0  0  0
  1  9  1  0  0  0  0
  1 10  1  0  0  0  0
  2 11  1  0  0  0  0
  2 16  1  0  0  0  0
  2 19  1  0  0  0  0
  3  5  2  0  0  0  0
  3  7  1  0  0  0  0
  3 45  1  0  0  0  0
  4  5  1  0  0  0  0
  4  6  1  0  0  0  0
  4 13  1  0  0  0  0
  4 14  1  0  0  0  0
  5  8  1  0  0  0  0
  5 45  1  0  0  0  0
  6 12  1  0  0  0  0
  6 15  1  0  0  0  0
  6 20  1  0  0  0  0
 15 16  2  0  0  0  0
 15 17  1  0  0  0  0
 15 45  1  0  0  0  0
 16 18  1  0  0  0  0
 16 45  1  0  0  0  0
 21 22  3  0  0  0  0
 21 45  1  0  0  0  0
 22 23  1  0  0  0  0
 23 24  2  0  0  0  0
 23 25  1  0  0  0  0
 24 26  1  0  0  0  0
```

```
 24 27  1  0  0  0  0
 25 28  2  0  0  0  0
 25 29  1  0  0  0  0
 26 30  2  0  0  0  0
 26 31  1  0  0  0  0
 28 30  1  0  0  0  0
 28 32  1  0  0  0  0
 30 47  1  0  0  0  0
 33 34  3  0  0  0  0
 33 45  1  0  0  0  0
 34 35  1  0  0  0  0
 35 36  2  0  0  0  0
 35 37  1  0  0  0  0
 36 38  1  0  0  0  0
 36 39  1  0  0  0  0
 37 40  2  0  0  0  0
 37 41  1  0  0  0  0
 38 42  2  0  0  0  0
 38 43  1  0  0  0  0
 40 42  1  0  0  0  0
 40 44  1  0  0  0  0
 42 46  1  0  0  0  0
 46 60  1  0  0  0  0
 46 64  1  0  0  0  0
 46 68  1  0  0  0  0
 47 48  1  0  0  0  0
 47 52  1  0  0  0  0
 47 56  1  0  0  0  0
 48 49  1  0  0  0  0
 48 50  1  0  0  0  0
 48 51  1  0  0  0  0
 52 53  1  0  0  0  0
 52 54  1  0  0  0  0
 52 55  1  0  0  0  0
 56 57  1  0  0  0  0
 56 58  1  0  0  0  0
 56 59  1  0  0  0  0
 60 61  1  0  0  0  0
 60 62  1  0  0  0  0
 60 63  1  0  0  0  0
 64 65  1  0  0  0  0
 64 66  1  0  0  0  0
 64 67  1  0  0  0  0
 68 69  1  0  0  0  0
 68 70  1  0  0  0  0
 68 71  1  0  0  0  0
M  END
$$$$
mol094
Optimized Catalyst 094
Optimized Catalyst 094
 71 76  0  0  0  0  0  0  0  0999 V2000
   -0.7644    0.2531   -1.5202 C   0  0  0  0  0  0  0  0  0  0  0  0
    0.4630   -0.6011   -1.1376 C   0  0  0  0  0  0  0  0  0  0  0  0
   -2.0234   -0.0003   -0.7218 C   0  0  0  0  0  0  0  0  0  0  0  0
   -0.9222    0.0318    1.5827 C   0  0  0  0  0  0  0  0  0  0  0  0
   -2.0982   -0.1258    0.6460 C   0  0  0  0  0  0  0  0  0  0  0  0
   -0.1194   -1.2615    1.8385 C   0  0  0  0  0  0  0  0  0  0  0  0
   -2.9567    0.1657   -1.3077 H   0  0  0  0  0  0  0  0  0  0  0  0
   -3.0889   -0.1119    1.1494 H   0  0  0  0  0  0  0  0  0  0  0  0
   -0.9851    0.0953   -2.6121 H   0  0  0  0  0  0  0  0  0  0  0  0
   -0.4999    1.3425   -1.3918 H   0  0  0  0  0  0  0  0  0  0  0  0
    1.1134   -0.7053   -2.0495 H   0  0  0  0  0  0  0  0  0  0  0  0
   -0.5242   -1.7567    2.7638 H   0  0  0  0  0  0  0  0  0  0  0  0
   -1.2995    0.4086    2.5736 H   0  0  0  0  0  0  0  0  0  0  0  0
```

```
   -0.2457    0.8314    1.1749 H   0  0  0  0  0  0  0  0  0  0  0  0
   -0.1036   -2.2670    0.7091 C   0  0  0  0  0  0  0  0  0  0  0  0
    0.1340   -1.9810   -0.6150 C   0  0  0  0  0  0  0  0  0  0  0  0
   -0.0573   -3.3235    1.0599 H   0  0  0  0  0  0  0  0  0  0  0  0
    0.3293   -2.8045   -1.3352 H   0  0  0  0  0  0  0  0  0  0  0  0
    1.0789   -0.0625   -0.3670 H   0  0  0  0  0  0  0  0  0  0  0  0
    0.9524   -0.9831    2.0561 H   0  0  0  0  0  0  0  0  0  0  0  0
   -4.1757   -2.3050   -0.5978 N   0  0  0  0  0  0  0  0  0  0  0  0
   -5.3299   -2.4055   -0.7522 C   0  0  0  0  0  0  0  0  0  0  0  0
   -6.7322   -2.5102   -0.9355 C   0  0  0  0  0  0  0  0  0  0  0  0
   -7.2510   -3.2814   -1.9949 C   0  0  0  0  0  0  0  0  0  0  0  0
   -7.6144   -1.8482   -0.0544 C   0  0  0  0  0  0  0  0  0  0  0  0
   -8.6291   -3.3891   -2.1616 C   0  0  0  0  0  0  0  0  0  0  0  0
   -6.5742   -3.7918   -2.6828 H   0  0  0  0  0  0  0  0  0  0  0  0
   -8.9859   -1.9690   -0.2382 C   0  0  0  0  0  0  0  0  0  0  0  0
   -7.2187   -1.2466    0.7660 H   0  0  0  0  0  0  0  0  0  0  0  0
   -9.5239   -2.7392   -1.2925 C   0  0  0  0  0  0  0  0  0  0  0  0
   -9.0010   -3.9948   -2.9917 H   0  0  0  0  0  0  0  0  0  0  0  0
   -9.6547   -1.4516    0.4548 H   0  0  0  0  0  0  0  0  0  0  0  0
   -2.2025   -4.2668   -0.6056 N   0  0  0  0  0  0  0  0  0  0  0  0
   -2.3156   -5.4100   -0.8221 C   0  0  0  0  0  0  0  0  0  0  0  0
   -2.4395   -6.7982   -1.0866 C   0  0  0  0  0  0  0  0  0  0  0  0
   -3.3951   -7.5677   -0.3923 C   0  0  0  0  0  0  0  0  0  0  0  0
   -1.6140   -7.4152   -2.0513 C   0  0  0  0  0  0  0  0  0  0  0  0
   -3.5298   -8.9243   -0.6749 C   0  0  0  0  0  0  0  0  0  0  0  0
   -4.0270   -7.1006    0.3652 H   0  0  0  0  0  0  0  0  0  0  0  0
   -1.7621   -8.7711   -2.3158 C   0  0  0  0  0  0  0  0  0  0  0  0
   -0.8651   -6.8286   -2.5865 H   0  0  0  0  0  0  0  0  0  0  0  0
   -2.7286   -9.5524   -1.6457 C   0  0  0  0  0  0  0  0  0  0  0  0
   -4.2772   -9.4950   -0.1183 H   0  0  0  0  0  0  0  0  0  0  0  0
   -1.1097   -9.2330   -3.0619 H   0  0  0  0  0  0  0  0  0  0  0  0
   -2.8543  -11.0470   -1.9782 C   0  0  0  0  0  0  0  0  0  0  0  0
  -11.0488   -2.8390   -1.4470 C   0  0  0  0  0  0  0  0  0  0  0  0
  -11.4657   -3.6878   -2.6642 C   0  0  0  0  0  0  0  0  0  0  0  0
  -12.5793   -3.7130   -2.7328 H   0  0  0  0  0  0  0  0  0  0  0  0
  -11.0808   -3.2450   -3.6086 H   0  0  0  0  0  0  0  0  0  0  0  0
  -11.1123   -4.7365   -2.5606 H   0  0  0  0  0  0  0  0  0  0  0  0
  -11.6151   -3.4953   -0.1702 C   0  0  0  0  0  0  0  0  0  0  0  0
  -11.4515   -2.8570    0.7236 H   0  0  0  0  0  0  0  0  0  0  0  0
  -12.7133   -3.6471   -0.2851 H   0  0  0  0  0  0  0  0  0  0  0  0
  -11.1371   -4.4854   -0.0020 H   0  0  0  0  0  0  0  0  0  0  0  0
  -11.6073   -1.4120   -1.6295 C   0  0  0  0  0  0  0  0  0  0  0  0
  -11.1039   -0.9097   -2.4850 H   0  0  0  0  0  0  0  0  0  0  0  0
  -12.7002   -1.4621   -1.8424 H   0  0  0  0  0  0  0  0  0  0  0  0
  -11.4683   -0.7984   -0.7147 H   0  0  0  0  0  0  0  0  0  0  0  0
   -2.9666  -11.2108   -3.5092 C   0  0  0  0  0  0  0  0  0  0  0  0
   -2.0082  -10.9753   -4.0183 H   0  0  0  0  0  0  0  0  0  0  0  0
   -3.2255  -12.2666   -3.7570 H   0  0  0  0  0  0  0  0  0  0  0  0
   -3.7669  -10.5498   -3.9080 H   0  0  0  0  0  0  0  0  0  0  0  0
   -4.0965  -11.6910   -1.3312 C   0  0  0  0  0  0  0  0  0  0  0  0
   -4.0202  -11.6881   -0.2222 H   0  0  0  0  0  0  0  0  0  0  0  0
   -5.0239  -11.1692   -1.6523 H   0  0  0  0  0  0  0  0  0  0  0  0
   -4.1732  -12.7561   -1.6554 H   0  0  0  0  0  0  0  0  0  0  0  0
   -1.5861  -11.7412   -1.4395 C   0  0  0  0  0  0  0  0  0  0  0  0
   -1.4946  -11.5780   -0.3428 H   0  0  0  0  0  0  0  0  0  0  0  0
   -1.6494  -12.8372   -1.6305 H   0  0  0  0  0  0  0  0  0  0  0  0
   -0.6719  -11.3545   -1.9384 H   0  0  0  0  0  0  0  0  0  0  0  0
   -2.2274   -2.3171   -0.3268 Ir  0  0  0  0  0  0  0  0  0  0  0  0
  1  2  1  0  0  0  0
  1  3  1  0  0  0  0
  1  9  1  0  0  0  0
  1 10  1  0  0  0  0
  2 11  1  0  0  0  0
  2 16  1  0  0  0  0
  2 19  1  0  0  0  0
```

```
  3  5  2  0  0  0  0
  3  7  1  0  0  0  0
  3 71  1  0  0  0  0
  4  5  1  0  0  0  0
  4  6  1  0  0  0  0
  4 13  1  0  0  0  0
  4 14  1  0  0  0  0
  5  8  1  0  0  0  0
  5 71  1  0  0  0  0
  6 12  1  0  0  0  0
  6 15  1  0  0  0  0
  6 20  1  0  0  0  0
 15 16  2  0  0  0  0
 15 17  1  0  0  0  0
 15 71  1  0  0  0  0
 16 18  1  0  0  0  0
 16 71  1  0  0  0  0
 21 22  3  0  0  0  0
 21 71  1  0  0  0  0
 22 23  1  0  0  0  0
 23 24  2  0  0  0  0
 23 25  1  0  0  0  0
 24 26  1  0  0  0  0
 24 27  1  0  0  0  0
 25 28  2  0  0  0  0
 25 29  1  0  0  0  0
 26 30  2  0  0  0  0
 26 31  1  0  0  0  0
 28 30  1  0  0  0  0
 28 32  1  0  0  0  0
 30 46  1  0  0  0  0
 33 34  3  0  0  0  0
 33 71  1  0  0  0  0
 34 35  1  0  0  0  0
 35 36  2  0  0  0  0
 35 37  1  0  0  0  0
 36 38  1  0  0  0  0
 36 39  1  0  0  0  0
 37 40  2  0  0  0  0
 37 41  1  0  0  0  0
 38 42  2  0  0  0  0
 38 43  1  0  0  0  0
 40 42  1  0  0  0  0
 40 44  1  0  0  0  0
 42 45  1  0  0  0  0
 45 59  1  0  0  0  0
 45 63  1  0  0  0  0
 45 67  1  0  0  0  0
 46 47  1  0  0  0  0
 46 51  1  0  0  0  0
 46 55  1  0  0  0  0
 47 48  1  0  0  0  0
 47 49  1  0  0  0  0
 47 50  1  0  0  0  0
 51 52  1  0  0  0  0
 51 53  1  0  0  0  0
 51 54  1  0  0  0  0
 55 56  1  0  0  0  0
 55 57  1  0  0  0  0
 55 58  1  0  0  0  0
 59 60  1  0  0  0  0
 59 61  1  0  0  0  0
 59 62  1  0  0  0  0
 63 64  1  0  0  0  0
 63 65  1  0  0  0  0
```

```
 63 66  1  0  0  0  0
 67 68  1  0  0  0  0
 67 69  1  0  0  0  0
 67 70  1  0  0  0  0
M  END
$$$$
mol095
Optimized Catalyst 095
Optimized Catalyst 095
 53 58  0  0  0  0  0  0  0  0999 V2000
   -0.9672    0.3479   -1.5687 C   0  0  0  0  0  0  0  0  0  0  0  0
    0.2072   -0.5734   -1.1790 C   0  0  0  0  0  0  0  0  0  0  0  0
   -2.2191    0.1312   -0.7522 C   0  0  0  0  0  0  0  0  0  0  0  0
   -1.0961    0.1655    1.5527 C   0  0  0  0  0  0  0  0  0  0  0  0
   -2.2845    0.0242    0.6307 C   0  0  0  0  0  0  0  0  0  0  0  0
   -0.4191   -1.1901    1.8444 C   0  0  0  0  0  0  0  0  0  0  0  0
   -3.1543    0.2624   -1.3302 H   0  0  0  0  0  0  0  0  0  0  0  0
   -3.2674    0.0220    1.1365 H   0  0  0  0  0  0  0  0  0  0  0  0
   -1.2145    0.1663   -2.6486 H   0  0  0  0  0  0  0  0  0  0  0  0
   -0.6534    1.4209   -1.4597 H   0  0  0  0  0  0  0  0  0  0  0  0
    0.8427   -0.7480   -2.0872 H   0  0  0  0  0  0  0  0  0  0  0  0
   -0.9272   -1.6522    2.7339 H   0  0  0  0  0  0  0  0  0  0  0  0
   -1.4371    0.6105    2.5248 H   0  0  0  0  0  0  0  0  0  0  0  0
   -0.3605    0.8812    1.1034 H   0  0  0  0  0  0  0  0  0  0  0  0
   -0.4897   -2.1794    0.7027 C   0  0  0  0  0  0  0  0  0  0  0  0
   -0.2580   -1.9074   -0.6430 C   0  0  0  0  0  0  0  0  0  0  0  0
   -0.5860   -3.2412    1.0296 H   0  0  0  0  0  0  0  0  0  0  0  0
   -0.2234   -2.7465   -1.3726 H   0  0  0  0  0  0  0  0  0  0  0  0
    0.8568   -0.0749   -0.4143 H   0  0  0  0  0  0  0  0  0  0  0  0
    0.6599   -1.0228    2.1052 H   0  0  0  0  0  0  0  0  0  0  0  0
   -3.9427   -2.0298   -0.4743 N   0  0  0  0  0  0  0  0  0  0  0  0
   -5.0837   -2.1972   -0.6349 C   0  0  0  0  0  0  0  0  0  0  0  0
   -6.4923   -2.3564   -0.8276 C   0  0  0  0  0  0  0  0  0  0  0  0
   -6.9699   -3.1631   -1.8752 C   0  0  0  0  0  0  0  0  0  0  0  0
   -7.3877   -1.6953    0.0322 C   0  0  0  0  0  0  0  0  0  0  0  0
   -8.3472   -3.3127   -2.0542 C   0  0  0  0  0  0  0  0  0  0  0  0
   -6.2735   -3.6669   -2.5476 H   0  0  0  0  0  0  0  0  0  0  0  0
   -8.7629   -1.8512   -0.1540 C   0  0  0  0  0  0  0  0  0  0  0  0
   -7.0141   -1.0637    0.8400 H   0  0  0  0  0  0  0  0  0  0  0  0
   -9.2417   -2.6673   -1.1886 C   0  0  0  0  0  0  0  0  0  0  0  0
   -8.7175   -3.9335   -2.8777 H   0  0  0  0  0  0  0  0  0  0  0  0
   -9.4570   -1.3265    0.5121 H   0  0  0  0  0  0  0  0  0  0  0  0
   -2.3360   -4.4128   -0.6331 N   0  0  0  0  0  0  0  0  0  0  0  0
   -2.4352   -5.5455   -0.8563 C   0  0  0  0  0  0  0  0  0  0  0  0
   -2.5504   -6.9472   -1.1365 C   0  0  0  0  0  0  0  0  0  0  0  0
   -3.4992   -7.7209   -0.4478 C   0  0  0  0  0  0  0  0  0  0  0  0
   -1.7088   -7.5323   -2.0984 C   0  0  0  0  0  0  0  0  0  0  0  0
   -3.6106   -9.0855   -0.7285 C   0  0  0  0  0  0  0  0  0  0  0  0
   -4.1453   -7.2631    0.3042 H   0  0  0  0  0  0  0  0  0  0  0  0
   -1.8247   -8.8959   -2.3725 C   0  0  0  0  0  0  0  0  0  0  0  0
   -0.9688   -6.9278   -2.6272 H   0  0  0  0  0  0  0  0  0  0  0  0
   -2.7796   -9.6682   -1.6935 C   0  0  0  0  0  0  0  0  0  0  0  0
   -4.3493   -9.6842   -0.1845 H   0  0  0  0  0  0  0  0  0  0  0  0
   -1.1656   -9.3564   -3.1174 H   0  0  0  0  0  0  0  0  0  0  0  0
   -2.8737  -11.1435   -2.0127 C   0  0  0  0  0  0  0  0  0  0  0  0
  -10.7307   -2.8253   -1.3963 C   0  0  0  0  0  0  0  0  0  0  0  0
   -2.2949   -1.9828   -0.2429 Rh  0  0  0  0  0  0  0  0  0  0  0  0
  -11.1085   -4.0074   -1.9061 F   0  0  0  0  0  0  0  0  0  0  0  0
  -11.2405   -1.9183   -2.2433 F   0  0  0  0  0  0  0  0  0  0  0  0
  -11.4775   -2.7043   -0.2888 F   0  0  0  0  0  0  0  0  0  0  0  0
   -2.8994  -11.4146   -3.3269 F   0  0  0  0  0  0  0  0  0  0  0  0
   -1.8338  -11.8497   -1.5439 F   0  0  0  0  0  0  0  0  0  0  0  0
   -3.9503  -11.7806   -1.5322 F   0  0  0  0  0  0  0  0  0  0  0  0
  1  2  1  0  0  0  0
  1  3  1  0  0  0  0
```

```
  1  9  1  0  0  0  0
  1 10  1  0  0  0  0
  2 11  1  0  0  0  0
  2 16  1  0  0  0  0
  2 19  1  0  0  0  0
  3  5  2  0  0  0  0
  3  7  1  0  0  0  0
  3 47  1  0  0  0  0
  4  5  1  0  0  0  0
  4  6  1  0  0  0  0
  4 13  1  0  0  0  0
  4 14  1  0  0  0  0
  5  8  1  0  0  0  0
  5 47  1  0  0  0  0
  6 12  1  0  0  0  0
  6 15  1  0  0  0  0
  6 20  1  0  0  0  0
 15 16  2  0  0  0  0
 15 17  1  0  0  0  0
 15 47  1  0  0  0  0
 16 18  1  0  0  0  0
 16 47  1  0  0  0  0
 21 22  3  0  0  0  0
 21 47  1  0  0  0  0
 22 23  1  0  0  0  0
 23 24  2  0  0  0  0
 23 25  1  0  0  0  0
 24 26  1  0  0  0  0
 24 27  1  0  0  0  0
 25 28  2  0  0  0  0
 25 29  1  0  0  0  0
 26 30  2  0  0  0  0
 26 31  1  0  0  0  0
 28 30  1  0  0  0  0
 28 32  1  0  0  0  0
 30 46  1  0  0  0  0
 33 34  3  0  0  0  0
 33 47  1  0  0  0  0
 34 35  1  0  0  0  0
 35 36  2  0  0  0  0
 35 37  1  0  0  0  0
 36 38  1  0  0  0  0
 36 39  1  0  0  0  0
 37 40  2  0  0  0  0
 37 41  1  0  0  0  0
 38 42  2  0  0  0  0
 38 43  1  0  0  0  0
 40 42  1  0  0  0  0
 40 44  1  0  0  0  0
 42 45  1  0  0  0  0
 45 51  1  0  0  0  0
 45 52  1  0  0  0  0
 45 53  1  0  0  0  0
 46 48  1  0  0  0  0
 46 49  1  0  0  0  0
 46 50  1  0  0  0  0
M  END
$$$$
mol096
Optimized Catalyst 096
Optimized Catalyst 096
 53 58  0  0  0  0  0  0  0  0999 V2000
   -0.8215    0.2528   -1.5376 C   0  0  0  0  0  0  0  0  0  0  0  0
    0.4091   -0.5885   -1.1389 C   0  0  0  0  0  0  0  0  0  0  0  0
   -2.0817    0.0090   -0.7379 C   0  0  0  0  0  0  0  0  0  0  0  0
```

```
   -0.9833    0.0638    1.5663 C   0  0  0  0  0  0  0  0  0  0  0  0
   -2.1589   -0.1021    0.6306 C   0  0  0  0  0  0  0  0  0  0  0  0
   -0.1869   -1.2291    1.8404 C   0  0  0  0  0  0  0  0  0  0  0  0
   -3.0128    0.1716   -1.3286 H   0  0  0  0  0  0  0  0  0  0  0  0
   -3.1490   -0.0808    1.1352 H   0  0  0  0  0  0  0  0  0  0  0  0
   -1.0383    0.0772   -2.6277 H   0  0  0  0  0  0  0  0  0  0  0  0
   -0.5602    1.3461   -1.4279 H   0  0  0  0  0  0  0  0  0  0  0  0
    1.0668   -0.6972   -2.0460 H   0  0  0  0  0  0  0  0  0  0  0  0
   -0.6010   -1.7162    2.7659 H   0  0  0  0  0  0  0  0  0  0  0  0
   -1.3587    0.4574    2.5520 H   0  0  0  0  0  0  0  0  0  0  0  0
   -0.3014    0.8547    1.1495 H   0  0  0  0  0  0  0  0  0  0  0  0
   -0.1588   -2.2419    0.7183 C   0  0  0  0  0  0  0  0  0  0  0  0
    0.0861   -1.9653   -0.6054 C   0  0  0  0  0  0  0  0  0  0  0  0
   -0.1092   -3.2953    1.0780 H   0  0  0  0  0  0  0  0  0  0  0  0
    0.2889   -2.7928   -1.3187 H   0  0  0  0  0  0  0  0  0  0  0  0
    1.0186   -0.0379   -0.3710 H   0  0  0  0  0  0  0  0  0  0  0  0
    0.8839   -0.9512    2.0687 H   0  0  0  0  0  0  0  0  0  0  0  0
   -4.2338   -2.2970   -0.5881 N   0  0  0  0  0  0  0  0  0  0  0  0
   -5.3890   -2.3827   -0.7387 C   0  0  0  0  0  0  0  0  0  0  0  0
   -6.8023   -2.4767   -0.9154 C   0  0  0  0  0  0  0  0  0  0  0  0
   -7.3211   -3.2496   -1.9719 C   0  0  0  0  0  0  0  0  0  0  0  0
   -7.6642   -1.8019   -0.0306 C   0  0  0  0  0  0  0  0  0  0  0  0
   -8.7039   -3.3535   -2.1313 C   0  0  0  0  0  0  0  0  0  0  0  0
   -6.6518   -3.7630   -2.6655 H   0  0  0  0  0  0  0  0  0  0  0  0
   -9.0462   -1.9119   -0.1996 C   0  0  0  0  0  0  0  0  0  0  0  0
   -7.2616   -1.1955    0.7841 H   0  0  0  0  0  0  0  0  0  0  0  0
   -9.5645   -2.6948   -1.2405 C   0  0  0  0  0  0  0  0  0  0  0  0
   -9.1052   -3.9507   -2.9596 H   0  0  0  0  0  0  0  0  0  0  0  0
   -9.7137   -1.3776    0.4883 H   0  0  0  0  0  0  0  0  0  0  0  0
   -2.2536   -4.2457   -0.6105 N   0  0  0  0  0  0  0  0  0  0  0  0
   -2.3466   -5.3897   -0.8266 C   0  0  0  0  0  0  0  0  0  0  0  0
   -2.4589   -6.7875   -1.0936 C   0  0  0  0  0  0  0  0  0  0  0  0
   -3.4122   -7.5505   -0.3929 C   0  0  0  0  0  0  0  0  0  0  0  0
   -1.6289   -7.3881   -2.0593 C   0  0  0  0  0  0  0  0  0  0  0  0
   -3.5455   -8.9121   -0.6733 C   0  0  0  0  0  0  0  0  0  0  0  0
   -4.0441   -7.0860    0.3673 H   0  0  0  0  0  0  0  0  0  0  0  0
   -1.7690   -8.7502   -2.3306 C   0  0  0  0  0  0  0  0  0  0  0  0
   -0.8810   -6.7988   -2.5947 H   0  0  0  0  0  0  0  0  0  0  0  0
   -2.7340   -9.5070   -1.6485 C   0  0  0  0  0  0  0  0  0  0  0  0
   -4.2880   -9.5006   -0.1204 H   0  0  0  0  0  0  0  0  0  0  0  0
   -1.1193   -9.2230   -3.0785 H   0  0  0  0  0  0  0  0  0  0  0  0
   -2.8682  -10.9799   -1.9783 C   0  0  0  0  0  0  0  0  0  0  0  0
  -11.0636   -2.8095   -1.4295 C   0  0  0  0  0  0  0  0  0  0  0  0
  -11.4767   -3.9906   -1.9114 F   0  0  0  0  0  0  0  0  0  0  0  0
  -11.5503   -1.9034   -2.2895 F   0  0  0  0  0  0  0  0  0  0  0  0
  -11.7908   -2.6444   -0.3159 F   0  0  0  0  0  0  0  0  0  0  0  0
   -2.9330  -11.2335   -3.2939 F   0  0  0  0  0  0  0  0  0  0  0  0
   -1.8344  -11.7128   -1.5407 F   0  0  0  0  0  0  0  0  0  0  0  0
   -3.9486  -11.5921   -1.4759 F   0  0  0  0  0  0  0  0  0  0  0  0
   -2.2912   -2.3078   -0.3252 Ir  0  0  0  0  0  0  0  0  0  0  0  0
  1  2  1  0  0  0  0
  1  3  1  0  0  0  0
  1  9  1  0  0  0  0
  1 10  1  0  0  0  0
  2 11  1  0  0  0  0
  2 16  1  0  0  0  0
  2 19  1  0  0  0  0
  3  5  2  0  0  0  0
  3  7  1  0  0  0  0
  3 53  1  0  0  0  0
  4  5  1  0  0  0  0
  4  6  1  0  0  0  0
  4 13  1  0  0  0  0
  4 14  1  0  0  0  0
  5  8  1  0  0  0  0
```

```
  5 53  1  0  0  0  0
  6 12  1  0  0  0  0
  6 15  1  0  0  0  0
  6 20  1  0  0  0  0
 15 16  2  0  0  0  0
 15 17  1  0  0  0  0
 15 53  1  0  0  0  0
 16 18  1  0  0  0  0
 16 53  1  0  0  0  0
 21 22  3  0  0  0  0
 21 53  1  0  0  0  0
 22 23  1  0  0  0  0
 23 24  2  0  0  0  0
 23 25  1  0  0  0  0
 24 26  1  0  0  0  0
 24 27  1  0  0  0  0
 25 28  2  0  0  0  0
 25 29  1  0  0  0  0
 26 30  2  0  0  0  0
 26 31  1  0  0  0  0
 28 30  1  0  0  0  0
 28 32  1  0  0  0  0
 30 46  1  0  0  0  0
 33 34  3  0  0  0  0
 33 53  1  0  0  0  0
 34 35  1  0  0  0  0
 35 36  2  0  0  0  0
 35 37  1  0  0  0  0
 36 38  1  0  0  0  0
 36 39  1  0  0  0  0
 37 40  2  0  0  0  0
 37 41  1  0  0  0  0
 38 42  2  0  0  0  0
 38 43  1  0  0  0  0
 40 42  1  0  0  0  0
 40 44  1  0  0  0  0
 42 45  1  0  0  0  0
 45 50  1  0  0  0  0
 45 51  1  0  0  0  0
 45 52  1  0  0  0  0
 46 47  1  0  0  0  0
 46 48  1  0  0  0  0
 46 49  1  0  0  0  0
M  END
$$$$
mol097
Optimized Catalyst 097
Optimized Catalyst 097
 33 36  0  0  0  0  0  0  0  0999 V2000
   -0.9663    0.3428   -1.5700 C   0  0  0  0  0  0  0  0  0  0  0  0
    0.2087   -0.5772   -1.1805 C   0  0  0  0  0  0  0  0  0  0  0  0
   -2.2174    0.1204   -0.7536 C   0  0  0  0  0  0  0  0  0  0  0  0
   -1.0943    0.1615    1.5521 C   0  0  0  0  0  0  0  0  0  0  0  0
   -2.2822    0.0132    0.6307 C   0  0  0  0  0  0  0  0  0  0  0  0
   -0.4157   -1.1928    1.8444 C   0  0  0  0  0  0  0  0  0  0  0  0
   -3.1530    0.2518   -1.3309 H   0  0  0  0  0  0  0  0  0  0  0  0
   -3.2650    0.0100    1.1370 H   0  0  0  0  0  0  0  0  0  0  0  0
   -1.2139    0.1608   -2.6501 H   0  0  0  0  0  0  0  0  0  0  0  0
   -0.6556    1.4164   -1.4600 H   0  0  0  0  0  0  0  0  0  0  0  0
    0.8432   -0.7530   -2.0891 H   0  0  0  0  0  0  0  0  0  0  0  0
   -0.9236   -1.6540    2.7346 H   0  0  0  0  0  0  0  0  0  0  0  0
   -1.4372    0.6070    2.5231 H   0  0  0  0  0  0  0  0  0  0  0  0
   -0.3605    0.8779    1.1015 H   0  0  0  0  0  0  0  0  0  0  0  0
   -0.4889   -2.1829    0.7029 C   0  0  0  0  0  0  0  0  0  0  0  0
   -0.2577   -1.9113   -0.6440 C   0  0  0  0  0  0  0  0  0  0  0  0
```

```
   -0.5780   -3.2445    1.0308 H   0  0  0  0  0  0  0  0  0  0  0  0
   -0.2160   -2.7513   -1.3718 H   0  0  0  0  0  0  0  0  0  0  0  0
    0.8585   -0.0784   -0.4166 H   0  0  0  0  0  0  0  0  0  0  0  0
    0.6635   -1.0254    2.1029 H   0  0  0  0  0  0  0  0  0  0  0  0
   -3.9614   -2.0191   -0.4707 N   0  0  0  0  0  0  0  0  0  0  0  0
   -5.0984   -2.1950   -0.6359 C   0  0  0  0  0  0  0  0  0  0  0  0
   -2.3323   -4.4002   -0.6287 N   0  0  0  0  0  0  0  0  0  0  0  0
   -2.4338   -5.5329   -0.8521 C   0  0  0  0  0  0  0  0  0  0  0  0
   -2.2967   -1.9863   -0.2435 Rh  0  0  0  0  0  0  0  0  0  0  0  0
   -6.5134   -2.3653   -0.8361 C   0  0  0  0  0  0  0  0  0  0  0  0
   -6.7947   -2.0839   -1.8890 H   0  0  0  0  0  0  0  0  0  0  0  0
   -6.8074   -3.4365   -0.6600 H   0  0  0  0  0  0  0  0  0  0  0  0
   -7.0910   -1.7124   -0.1240 H   0  0  0  0  0  0  0  0  0  0  0  0
   -2.5536   -6.9397   -1.1306 C   0  0  0  0  0  0  0  0  0  0  0  0
   -1.7984   -7.5257   -0.5363 H   0  0  0  0  0  0  0  0  0  0  0  0
   -3.5805   -7.3114   -0.8616 H   0  0  0  0  0  0  0  0  0  0  0  0
   -2.3767   -7.1411   -2.2236 H   0  0  0  0  0  0  0  0  0  0  0  0
  1  2  1  0  0  0  0
  1  3  1  0  0  0  0
  1  9  1  0  0  0  0
  1 10  1  0  0  0  0
  2 11  1  0  0  0  0
  2 16  1  0  0  0  0
  2 19  1  0  0  0  0
  3  5  2  0  0  0  0
  3  7  1  0  0  0  0
  3 25  1  0  0  0  0
  4  5  1  0  0  0  0
  4  6  1  0  0  0  0
  4 13  1  0  0  0  0
  4 14  1  0  0  0  0
  5  8  1  0  0  0  0
  5 25  1  0  0  0  0
  6 12  1  0  0  0  0
  6 15  1  0  0  0  0
  6 20  1  0  0  0  0
 15 16  2  0  0  0  0
 15 17  1  0  0  0  0
 15 25  1  0  0  0  0
 16 18  1  0  0  0  0
 16 25  1  0  0  0  0
 21 22  3  0  0  0  0
 21 25  1  0  0  0  0
 22 26  1  0  0  0  0
 23 24  3  0  0  0  0
 23 25  1  0  0  0  0
 24 30  1  0  0  0  0
 26 27  1  0  0  0  0
 26 28  1  0  0  0  0
 26 29  1  0  0  0  0
 30 31  1  0  0  0  0
 30 32  1  0  0  0  0
 30 33  1  0  0  0  0
M  END
$$$$
mol099
Optimized Catalyst 099
Optimized Catalyst 099
 51 54  0  0  0  0  0  0  0  0999 V2000
   -0.9284    0.3719   -1.6287 C   0  0  0  0  0  0  0  0  0  0  0  0
    0.2453   -0.5507   -1.2396 C   0  0  0  0  0  0  0  0  0  0  0  0
   -2.1766    0.1597   -0.8044 C   0  0  0  0  0  0  0  0  0  0  0  0
   -1.0372    0.1975    1.4921 C   0  0  0  0  0  0  0  0  0  0  0  0
   -2.2327    0.0568    0.5786 C   0  0  0  0  0  0  0  0  0  0  0  0
   -0.3641   -1.1608    1.7826 C   0  0  0  0  0  0  0  0  0  0  0  0
```

```
   -3.1157    0.2820   -1.3776 H   0  0  0  0  0  0  0  0  0  0  0  0
   -3.2120    0.0523    1.0911 H   0  0  0  0  0  0  0  0  0  0  0  0
   -1.1813    0.1836   -2.7059 H   0  0  0  0  0  0  0  0  0  0  0  0
   -0.6124    1.4443   -1.5265 H   0  0  0  0  0  0  0  0  0  0  0  0
    0.8811   -0.7266   -2.1469 H   0  0  0  0  0  0  0  0  0  0  0  0
   -0.8692   -1.6172    2.6762 H   0  0  0  0  0  0  0  0  0  0  0  0
   -1.3696    0.6469    2.4647 H   0  0  0  0  0  0  0  0  0  0  0  0
   -0.3018    0.9082    1.0353 H   0  0  0  0  0  0  0  0  0  0  0  0
   -0.4483   -2.1540    0.6441 C   0  0  0  0  0  0  0  0  0  0  0  0
   -0.2241   -1.8841   -0.7041 C   0  0  0  0  0  0  0  0  0  0  0  0
   -0.5474   -3.2145    0.9737 H   0  0  0  0  0  0  0  0  0  0  0  0
   -0.1986   -2.7224   -1.4351 H   0  0  0  0  0  0  0  0  0  0  0  0
    0.8946   -0.0536   -0.4741 H   0  0  0  0  0  0  0  0  0  0  0  0
    0.7172   -0.9983    2.0344 H   0  0  0  0  0  0  0  0  0  0  0  0
   -3.9232   -2.0008   -0.5031 N   0  0  0  0  0  0  0  0  0  0  0  0
   -5.0587   -2.1853   -0.6499 C   0  0  0  0  0  0  0  0  0  0  0  0
   -2.3019   -4.3754   -0.7078 N   0  0  0  0  0  0  0  0  0  0  0  0
   -2.4167   -5.5101   -0.9010 C   0  0  0  0  0  0  0  0  0  0  0  0
   -6.5253   -2.3868   -0.8308 C   0  0  0  0  0  0  0  0  0  0  0  0
   -2.5593   -6.9744   -1.1339 C   0  0  0  0  0  0  0  0  0  0  0  0
   -6.7970   -3.9016   -0.8062 C   0  0  0  0  0  0  0  0  0  0  0  0
   -7.8962   -4.0811   -0.8838 H   0  0  0  0  0  0  0  0  0  0  0  0
   -6.2982   -4.4054   -1.6633 H   0  0  0  0  0  0  0  0  0  0  0  0
   -6.4366   -4.3547    0.1444 H   0  0  0  0  0  0  0  0  0  0  0  0
   -7.2430   -1.6775    0.3303 C   0  0  0  0  0  0  0  0  0  0  0  0
   -6.9879   -0.5948    0.3554 H   0  0  0  0  0  0  0  0  0  0  0  0
   -8.3475   -1.7707    0.1966 H   0  0  0  0  0  0  0  0  0  0  0  0
   -6.9723   -2.1381    1.3070 H   0  0  0  0  0  0  0  0  0  0  0  0
   -6.9212   -1.7740   -2.1853 C   0  0  0  0  0  0  0  0  0  0  0  0
   -6.3424   -2.2361   -3.0162 H   0  0  0  0  0  0  0  0  0  0  0  0
   -8.0067   -1.9571   -2.3738 H   0  0  0  0  0  0  0  0  0  0  0  0
   -6.7505   -0.6740   -2.1892 H   0  0  0  0  0  0  0  0  0  0  0  0
   -2.4328   -7.2358   -2.6442 C   0  0  0  0  0  0  0  0  0  0  0  0
   -2.5562   -8.3271   -2.8449 H   0  0  0  0  0  0  0  0  0  0  0  0
   -3.2175   -6.6871   -3.2110 H   0  0  0  0  0  0  0  0  0  0  0  0
   -1.4305   -6.9259   -3.0172 H   0  0  0  0  0  0  0  0  0  0  0  0
   -3.9454   -7.3952   -0.6135 C   0  0  0  0  0  0  0  0  0  0  0  0
   -4.0691   -8.4976   -0.7401 H   0  0  0  0  0  0  0  0  0  0  0  0
   -4.0517   -7.1557    0.4684 H   0  0  0  0  0  0  0  0  0  0  0  0
   -4.7540   -6.8850   -1.1820 H   0  0  0  0  0  0  0  0  0  0  0  0
   -1.4366   -7.6815   -0.3548 C   0  0  0  0  0  0  0  0  0  0  0  0
   -1.5393   -7.5031    0.7392 H   0  0  0  0  0  0  0  0  0  0  0  0
   -1.4923   -8.7815   -0.5358 H   0  0  0  0  0  0  0  0  0  0  0  0
   -0.4362   -7.3251   -0.6888 H   0  0  0  0  0  0  0  0  0  0  0  0
   -2.2560   -1.9561   -0.2908 Rh  0  0  0  0  0  0  0  0  0  0  0  0
  1  2  1  0  0  0  0
  1  3  1  0  0  0  0
  1  9  1  0  0  0  0
  1 10  1  0  0  0  0
  2 11  1  0  0  0  0
  2 16  1  0  0  0  0
  2 19  1  0  0  0  0
  3  5  2  0  0  0  0
  3  7  1  0  0  0  0
  3 51  1  0  0  0  0
  4  5  1  0  0  0  0
  4  6  1  0  0  0  0
  4 13  1  0  0  0  0
  4 14  1  0  0  0  0
  5  8  1  0  0  0  0
  5 51  1  0  0  0  0
  6 12  1  0  0  0  0
  6 15  1  0  0  0  0
  6 20  1  0  0  0  0
 15 16  2  0  0  0  0
```

```
 15 17  1  0  0  0  0
 15 51  1  0  0  0  0
 16 18  1  0  0  0  0
 16 51  1  0  0  0  0
 21 22  3  0  0  0  0
 21 51  1  0  0  0  0
 22 25  1  0  0  0  0
 23 24  3  0  0  0  0
 23 51  1  0  0  0  0
 24 26  1  0  0  0  0
 25 27  1  0  0  0  0
 25 31  1  0  0  0  0
 25 35  1  0  0  0  0
 26 39  1  0  0  0  0
 26 43  1  0  0  0  0
 26 47  1  0  0  0  0
 27 28  1  0  0  0  0
 27 29  1  0  0  0  0
 27 30  1  0  0  0  0
 31 32  1  0  0  0  0
 31 33  1  0  0  0  0
 31 34  1  0  0  0  0
 35 36  1  0  0  0  0
 35 37  1  0  0  0  0
 35 38  1  0  0  0  0
 39 40  1  0  0  0  0
 39 41  1  0  0  0  0
 39 42  1  0  0  0  0
 43 44  1  0  0  0  0
 43 45  1  0  0  0  0
 43 46  1  0  0  0  0
 47 48  1  0  0  0  0
 47 49  1  0  0  0  0
 47 50  1  0  0  0  0
M  END
$$$$
mol100
Optimized Catalyst 100
Optimized Catalyst 100
 51 54  0  0  0  0  0  0  0  0999 V2000
   -0.8080    0.2827   -1.5348 C   0  0  0  0  0  0  0  0  0  0  0  0
    0.4185   -0.5650   -1.1371 C   0  0  0  0  0  0  0  0  0  0  0  0
   -2.0695    0.0412   -0.7368 C   0  0  0  0  0  0  0  0  0  0  0  0
   -0.9717    0.0864    1.5665 C   0  0  0  0  0  0  0  0  0  0  0  0
   -2.1479   -0.0698    0.6305 C   0  0  0  0  0  0  0  0  0  0  0  0
   -0.1844   -1.2130    1.8377 C   0  0  0  0  0  0  0  0  0  0  0  0
   -3.0001    0.1989   -1.3283 H   0  0  0  0  0  0  0  0  0  0  0  0
   -3.1387   -0.0505    1.1332 H   0  0  0  0  0  0  0  0  0  0  0  0
   -1.0248    0.1103   -2.6252 H   0  0  0  0  0  0  0  0  0  0  0  0
   -0.5418    1.3740   -1.4219 H   0  0  0  0  0  0  0  0  0  0  0  0
    1.0779   -0.6710   -2.0429 H   0  0  0  0  0  0  0  0  0  0  0  0
   -0.6069   -1.7011    2.7588 H   0  0  0  0  0  0  0  0  0  0  0  0
   -1.3447    0.4804    2.5527 H   0  0  0  0  0  0  0  0  0  0  0  0
   -0.2845    0.8738    1.1523 H   0  0  0  0  0  0  0  0  0  0  0  0
   -0.1578   -2.2230    0.7124 C   0  0  0  0  0  0  0  0  0  0  0  0
    0.0886   -1.9427   -0.6103 C   0  0  0  0  0  0  0  0  0  0  0  0
   -0.1132   -3.2778    1.0690 H   0  0  0  0  0  0  0  0  0  0  0  0
    0.2832   -2.7680   -1.3287 H   0  0  0  0  0  0  0  0  0  0  0  0
    1.0278   -0.0213   -0.3643 H   0  0  0  0  0  0  0  0  0  0  0  0
    0.8868   -0.9423    2.0709 H   0  0  0  0  0  0  0  0  0  0  0  0
   -4.2402   -2.2848   -0.5802 N   0  0  0  0  0  0  0  0  0  0  0  0
   -5.3927   -2.3642   -0.7215 C   0  0  0  0  0  0  0  0  0  0  0  0
   -2.2601   -4.2374   -0.6128 N   0  0  0  0  0  0  0  0  0  0  0  0
   -2.3644   -5.3769   -0.8253 C   0  0  0  0  0  0  0  0  0  0  0  0
   -6.8755   -2.4458   -0.8986 C   0  0  0  0  0  0  0  0  0  0  0  0
```

```
   -2.4985   -6.8394   -1.1056 C   0  0  0  0  0  0  0  0  0  0  0  0
   -7.2871   -3.9055   -0.6487 C   0  0  0  0  0  0  0  0  0  0  0  0
   -8.3957   -3.9987   -0.7742 H   0  0  0  0  0  0  0  0  0  0  0  0
   -6.8023   -4.5930   -1.3765 H   0  0  0  0  0  0  0  0  0  0  0  0
   -7.0351   -4.2243    0.3881 H   0  0  0  0  0  0  0  0  0  0  0  0
   -7.5114   -1.4980    0.1293 C   0  0  0  0  0  0  0  0  0  0  0  0
   -7.2004   -0.4432   -0.0465 H   0  0  0  0  0  0  0  0  0  0  0  0
   -8.6256   -1.5447    0.0355 H   0  0  0  0  0  0  0  0  0  0  0  0
   -7.2452   -1.7911    1.1704 H   0  0  0  0  0  0  0  0  0  0  0  0
   -7.1899   -2.0109   -2.3379 C   0  0  0  0  0  0  0  0  0  0  0  0
   -6.6846   -2.6683   -3.0810 H   0  0  0  0  0  0  0  0  0  0  0  0
   -8.2931   -2.0812   -2.5118 H   0  0  0  0  0  0  0  0  0  0  0  0
   -6.8853   -0.9545   -2.5166 H   0  0  0  0  0  0  0  0  0  0  0  0
   -2.2517   -7.0408   -2.6084 C   0  0  0  0  0  0  0  0  0  0  0  0
   -2.3566   -8.1267   -2.8565 H   0  0  0  0  0  0  0  0  0  0  0  0
   -2.9913   -6.4782   -3.2211 H   0  0  0  0  0  0  0  0  0  0  0  0
   -1.2227   -6.7255   -2.8958 H   0  0  0  0  0  0  0  0  0  0  0  0
   -3.9290   -7.2433   -0.7125 C   0  0  0  0  0  0  0  0  0  0  0  0
   -4.0680   -8.3357   -0.9111 H   0  0  0  0  0  0  0  0  0  0  0  0
   -4.1139   -7.0699    0.3718 H   0  0  0  0  0  0  0  0  0  0  0  0
   -4.6848   -6.6892   -1.3114 H   0  0  0  0  0  0  0  0  0  0  0  0
   -1.4528   -7.5776   -0.2583 C   0  0  0  0  0  0  0  0  0  0  0  0
   -1.6165   -7.4055    0.8297 H   0  0  0  0  0  0  0  0  0  0  0  0
   -1.5364   -8.6776   -0.4462 H   0  0  0  0  0  0  0  0  0  0  0  0
   -0.4185   -7.2655   -0.5282 H   0  0  0  0  0  0  0  0  0  0  0  0
   -2.2883   -2.2901   -0.3298 Ir  0  0  0  0  0  0  0  0  0  0  0  0
  1  2  1  0  0  0  0
  1  3  1  0  0  0  0
  1  9  1  0  0  0  0
  1 10  1  0  0  0  0
  2 11  1  0  0  0  0
  2 16  1  0  0  0  0
  2 19  1  0  0  0  0
  3  5  2  0  0  0  0
  3  7  1  0  0  0  0
  3 51  1  0  0  0  0
  4  5  1  0  0  0  0
  4  6  1  0  0  0  0
  4 13  1  0  0  0  0
  4 14  1  0  0  0  0
  5  8  1  0  0  0  0
  5 51  1  0  0  0  0
  6 12  1  0  0  0  0
  6 15  1  0  0  0  0
  6 20  1  0  0  0  0
 15 16  2  0  0  0  0
 15 17  1  0  0  0  0
 15 51  1  0  0  0  0
 16 18  1  0  0  0  0
 16 51  1  0  0  0  0
 21 22  3  0  0  0  0
 21 51  1  0  0  0  0
 22 25  1  0  0  0  0
 23 24  3  0  0  0  0
 23 51  1  0  0  0  0
 24 26  1  0  0  0  0
 25 27  1  0  0  0  0
 25 31  1  0  0  0  0
 25 35  1  0  0  0  0
 26 39  1  0  0  0  0
 26 43  1  0  0  0  0
 26 47  1  0  0  0  0
 27 28  1  0  0  0  0
 27 29  1  0  0  0  0
 27 30  1  0  0  0  0
```

```
 31 32  1  0  0  0  0
 31 33  1  0  0  0  0
 31 34  1  0  0  0  0
 35 36  1  0  0  0  0
 35 37  1  0  0  0  0
 35 38  1  0  0  0  0
 39 40  1  0  0  0  0
 39 41  1  0  0  0  0
 39 42  1  0  0  0  0
 43 44  1  0  0  0  0
 43 45  1  0  0  0  0
 43 46  1  0  0  0  0
 47 48  1  0  0  0  0
 47 49  1  0  0  0  0
 47 50  1  0  0  0  0
M  END
$$$$
mol101
Optimized Catalyst 101
Optimized Catalyst 101
 51 54  0  0  0  0  0  0  0  0999 V2000
   -0.5964    0.0197   -1.5095 C   0  0  0  0  0  0  0  0  0  0  0  0
    0.5404   -0.9156   -1.0484 C   0  0  0  0  0  0  0  0  0  0  0  0
   -1.8618   -0.0905   -0.6931 C   0  0  0  0  0  0  0  0  0  0  0  0
   -0.7537    0.0159    1.6166 C   0  0  0  0  0  0  0  0  0  0  0  0
   -1.9414   -0.1141    0.6923 C   0  0  0  0  0  0  0  0  0  0  0  0
   -0.1446   -1.3526    1.9897 C   0  0  0  0  0  0  0  0  0  0  0  0
   -2.7860    0.0591   -1.2855 H   0  0  0  0  0  0  0  0  0  0  0  0
   -2.9267   -0.0327    1.1880 H   0  0  0  0  0  0  0  0  0  0  0  0
   -0.8415   -0.2208   -2.5788 H   0  0  0  0  0  0  0  0  0  0  0  0
   -0.2416    1.0860   -1.4685 H   0  0  0  0  0  0  0  0  0  0  0  0
    1.1873   -1.1588   -1.9337 H   0  0  0  0  0  0  0  0  0  0  0  0
   -0.6892   -1.7448    2.8919 H   0  0  0  0  0  0  0  0  0  0  0  0
   -1.0787    0.5296    2.5611 H   0  0  0  0  0  0  0  0  0  0  0  0
    0.0187    0.6727    1.1384 H   0  0  0  0  0  0  0  0  0  0  0  0
   -0.2348   -2.3967    0.8995 C   0  0  0  0  0  0  0  0  0  0  0  0
    0.0213   -2.2019   -0.4522 C   0  0  0  0  0  0  0  0  0  0  0  0
   -0.3950   -3.4364    1.2748 H   0  0  0  0  0  0  0  0  0  0  0  0
    0.0094   -3.0746   -1.1446 H   0  0  0  0  0  0  0  0  0  0  0  0
    1.1941   -0.3973   -0.2998 H   0  0  0  0  0  0  0  0  0  0  0  0
    0.9363   -1.2177    2.2649 H   0  0  0  0  0  0  0  0  0  0  0  0
   -3.6294   -2.1831   -0.3085 N   0  0  0  0  0  0  0  0  0  0  0  0
   -4.7800   -2.2524   -0.4905 C   0  0  0  0  0  0  0  0  0  0  0  0
   -2.2556   -4.7778   -0.6925 N   0  0  0  0  0  0  0  0  0  0  0  0
   -2.4334   -5.8806   -0.9868 C   0  0  0  0  0  0  0  0  0  0  0  0
   -6.2154   -2.3108   -0.7216 C   0  0  0  0  0  0  0  0  0  0  0  0
   -2.6584   -7.2837   -1.3543 C   0  0  0  0  0  0  0  0  0  0  0  0
   -6.8759   -3.1301    0.4497 C   0  0  0  0  0  0  0  0  0  0  0  0
   -6.7749   -0.8385   -0.7504 C   0  0  0  0  0  0  0  0  0  0  0  0
   -6.4669   -3.0217   -2.1039 C   0  0  0  0  0  0  0  0  0  0  0  0
   -3.0168   -7.3484   -2.8854 C   0  0  0  0  0  0  0  0  0  0  0  0
   -3.8491   -7.8357   -0.4852 C   0  0  0  0  0  0  0  0  0  0  0  0
   -1.3400   -8.0913   -1.0621 C   0  0  0  0  0  0  0  0  0  0  0  0
   -8.2064   -3.1504    0.4315 F   0  0  0  0  0  0  0  0  0  0  0  0
   -6.5422   -2.6661    1.6589 F   0  0  0  0  0  0  0  0  0  0  0  0
   -6.4980   -4.4124    0.4674 F   0  0  0  0  0  0  0  0  0  0  0  0
   -7.7459   -3.2575   -2.3850 F   0  0  0  0  0  0  0  0  0  0  0  0
   -5.9924   -2.3291   -3.1439 F   0  0  0  0  0  0  0  0  0  0  0  0
   -5.8589   -4.2109   -2.1906 F   0  0  0  0  0  0  0  0  0  0  0  0
   -8.0555   -0.7338   -1.0956 F   0  0  0  0  0  0  0  0  0  0  0  0
   -6.1102   -0.0502   -1.6042 F   0  0  0  0  0  0  0  0  0  0  0  0
   -6.6677   -0.2140    0.4268 F   0  0  0  0  0  0  0  0  0  0  0  0
   -4.9960   -7.1867   -0.7071 F   0  0  0  0  0  0  0  0  0  0  0  0
   -3.6342   -7.7025    0.8275 F   0  0  0  0  0  0  0  0  0  0  0  0
   -4.1219   -9.1234   -0.6752 F   0  0  0  0  0  0  0  0  0  0  0  0
```

```
   -1.3926   -9.3777   -1.3958 F   0  0  0  0  0  0  0  0  0  0  0  0
   -0.9917   -8.0672    0.2281 F   0  0  0  0  0  0  0  0  0  0  0  0
   -0.2784   -7.5950   -1.7059 F   0  0  0  0  0  0  0  0  0  0  0  0
   -3.2911   -8.5694   -3.3364 F   0  0  0  0  0  0  0  0  0  0  0  0
   -2.0365   -6.8859   -3.6670 F   0  0  0  0  0  0  0  0  0  0  0  0
   -4.0798   -6.6022   -3.2006 F   0  0  0  0  0  0  0  0  0  0  0  0
   -2.0215   -2.1769   -0.0613 Rh  0  0  0  0  0  0  0  0  0  0  0  0
  1  2  1  0  0  0  0
  1  3  1  0  0  0  0
  1  9  1  0  0  0  0
  1 10  1  0  0  0  0
  2 11  1  0  0  0  0
  2 16  1  0  0  0  0
  2 19  1  0  0  0  0
  3  5  2  0  0  0  0
  3  7  1  0  0  0  0
  3 51  1  0  0  0  0
  4  5  1  0  0  0  0
  4  6  1  0  0  0  0
  4 13  1  0  0  0  0
  4 14  1  0  0  0  0
  5  8  1  0  0  0  0
  5 51  1  0  0  0  0
  6 12  1  0  0  0  0
  6 15  1  0  0  0  0
  6 20  1  0  0  0  0
 15 16  2  0  0  0  0
 15 17  1  0  0  0  0
 15 51  1  0  0  0  0
 16 18  1  0  0  0  0
 16 51  1  0  0  0  0
 21 22  3  0  0  0  0
 21 51  1  0  0  0  0
 22 25  1  0  0  0  0
 23 24  3  0  0  0  0
 23 51  1  0  0  0  0
 24 26  1  0  0  0  0
 25 27  1  0  0  0  0
 25 28  1  0  0  0  0
 25 29  1  0  0  0  0
 26 30  1  0  0  0  0
 26 31  1  0  0  0  0
 26 32  1  0  0  0  0
 27 33  1  0  0  0  0
 27 34  1  0  0  0  0
 27 35  1  0  0  0  0
 28 39  1  0  0  0  0
 28 40  1  0  0  0  0
 28 41  1  0  0  0  0
 29 36  1  0  0  0  0
 29 37  1  0  0  0  0
 29 38  1  0  0  0  0
 30 48  1  0  0  0  0
 30 49  1  0  0  0  0
 30 50  1  0  0  0  0
 31 42  1  0  0  0  0
 31 43  1  0  0  0  0
 31 44  1  0  0  0  0
 32 45  1  0  0  0  0
 32 46  1  0  0  0  0
 32 47  1  0  0  0  0
M  END
$$$$
mol102
Optimized Catalyst 102
```

```
Optimized Catalyst 102
 51 54  0  0  0  0  0  0  0  0999 V2000
   -0.4221   -0.0706   -1.4616 C   0  0  0  0  0  0  0  0  0  0  0  0
    0.7385   -1.0039   -1.0622 C   0  0  0  0  0  0  0  0  0  0  0  0
   -1.7066   -0.2292   -0.6811 C   0  0  0  0  0  0  0  0  0  0  0  0
   -0.6477   -0.3243    1.6420 C   0  0  0  0  0  0  0  0  0  0  0  0
   -1.8151   -0.3712    0.6826 C   0  0  0  0  0  0  0  0  0  0  0  0
    0.0449   -1.6798    1.8933 C   0  0  0  0  0  0  0  0  0  0  0  0
   -2.6117    0.0308   -1.2777 H   0  0  0  0  0  0  0  0  0  0  0  0
   -2.8088   -0.2673    1.1722 H   0  0  0  0  0  0  0  0  0  0  0  0
   -0.6369   -0.2124   -2.5577 H   0  0  0  0  0  0  0  0  0  0  0  0
   -0.0821    1.0003   -1.3342 H   0  0  0  0  0  0  0  0  0  0  0  0
    1.4032   -1.1383   -1.9622 H   0  0  0  0  0  0  0  0  0  0  0  0
   -0.4218   -2.1618    2.7970 H   0  0  0  0  0  0  0  0  0  0  0  0
   -1.0113    0.0700    2.6331 H   0  0  0  0  0  0  0  0  0  0  0  0
    0.0997    0.4251    1.2607 H   0  0  0  0  0  0  0  0  0  0  0  0
    0.0273   -2.6603    0.7430 C   0  0  0  0  0  0  0  0  0  0  0  0
    0.3117   -2.3662   -0.5681 C   0  0  0  0  0  0  0  0  0  0  0  0
    0.0108   -3.7240    1.0751 H   0  0  0  0  0  0  0  0  0  0  0  0
    0.4711   -3.1870   -1.3002 H   0  0  0  0  0  0  0  0  0  0  0  0
    1.3741   -0.5158   -0.2727 H   0  0  0  0  0  0  0  0  0  0  0  0
    1.1285   -1.4866    2.1521 H   0  0  0  0  0  0  0  0  0  0  0  0
   -4.0022   -2.3773   -0.5400 N   0  0  0  0  0  0  0  0  0  0  0  0
   -5.1621   -2.3686   -0.6716 C   0  0  0  0  0  0  0  0  0  0  0  0
   -2.1909   -4.4282   -0.6640 N   0  0  0  0  0  0  0  0  0  0  0  0
   -2.3402   -5.5593   -0.9107 C   0  0  0  0  0  0  0  0  0  0  0  0
   -6.6138   -2.3361   -0.8284 C   0  0  0  0  0  0  0  0  0  0  0  0
   -2.5152   -6.9758   -1.2214 C   0  0  0  0  0  0  0  0  0  0  0  0
   -7.2624   -3.1331    0.3698 C   0  0  0  0  0  0  0  0  0  0  0  0
   -7.0827   -0.8301   -0.8088 C   0  0  0  0  0  0  0  0  0  0  0  0
   -6.9837   -3.0143   -2.2045 C   0  0  0  0  0  0  0  0  0  0  0  0
   -2.8846   -7.1098   -2.7505 C   0  0  0  0  0  0  0  0  0  0  0  0
   -3.6819   -7.5405   -0.3209 C   0  0  0  0  0  0  0  0  0  0  0  0
   -1.1645   -7.7295   -0.9113 C   0  0  0  0  0  0  0  0  0  0  0  0
   -8.5878   -3.0663    0.4244 F   0  0  0  0  0  0  0  0  0  0  0  0
   -6.8246   -2.7079    1.5594 F   0  0  0  0  0  0  0  0  0  0  0  0
   -6.9590   -4.4339    0.3436 F   0  0  0  0  0  0  0  0  0  0  0  0
   -8.2844   -3.2059   -2.3937 F   0  0  0  0  0  0  0  0  0  0  0  0
   -6.5564   -2.3143   -3.2581 F   0  0  0  0  0  0  0  0  0  0  0  0
   -6.4133   -4.2167   -2.3432 F   0  0  0  0  0  0  0  0  0  0  0  0
   -8.3646   -0.6461   -1.1025 F   0  0  0  0  0  0  0  0  0  0  0  0
   -6.3957   -0.0739   -1.6727 F   0  0  0  0  0  0  0  0  0  0  0  0
   -6.8869   -0.2450    0.3761 F   0  0  0  0  0  0  0  0  0  0  0  0
   -4.8670   -6.9999   -0.6187 F   0  0  0  0  0  0  0  0  0  0  0  0
   -3.5024   -7.2653    0.9751 F   0  0  0  0  0  0  0  0  0  0  0  0
   -3.8490   -8.8560   -0.3941 F   0  0  0  0  0  0  0  0  0  0  0  0
   -1.1324   -8.9937   -1.3172 F   0  0  0  0  0  0  0  0  0  0  0  0
   -0.8726   -7.7522    0.3917 F   0  0  0  0  0  0  0  0  0  0  0  0
   -0.1105   -7.1344   -1.4816 F   0  0  0  0  0  0  0  0  0  0  0  0
   -3.2400   -8.3332   -3.1267 F   0  0  0  0  0  0  0  0  0  0  0  0
   -1.8826   -6.7521   -3.5573 F   0  0  0  0  0  0  0  0  0  0  0  0
   -3.9003   -6.3114   -3.0970 F   0  0  0  0  0  0  0  0  0  0  0  0
   -2.0918   -2.5314   -0.3055 Ir  0  0  0  0  0  0  0  0  0  0  0  0
  1  2  1  0  0  0  0
  1  3  1  0  0  0  0
  1  9  1  0  0  0  0
  1 10  1  0  0  0  0
  2 11  1  0  0  0  0
  2 16  1  0  0  0  0
  2 19  1  0  0  0  0
  3  5  2  0  0  0  0
  3  7  1  0  0  0  0
  3 51  1  0  0  0  0
  4  5  1  0  0  0  0
  4  6  1  0  0  0  0
```

```
  4 13  1  0  0  0  0
  4 14  1  0  0  0  0
  5  8  1  0  0  0  0
  5 51  1  0  0  0  0
  6 12  1  0  0  0  0
  6 15  1  0  0  0  0
  6 20  1  0  0  0  0
 15 16  2  0  0  0  0
 15 17  1  0  0  0  0
 15 51  1  0  0  0  0
 16 18  1  0  0  0  0
 16 51  1  0  0  0  0
 21 22  3  0  0  0  0
 21 51  1  0  0  0  0
 22 25  1  0  0  0  0
 23 24  3  0  0  0  0
 23 51  1  0  0  0  0
 24 26  1  0  0  0  0
 25 27  1  0  0  0  0
 25 28  1  0  0  0  0
 25 29  1  0  0  0  0
 26 30  1  0  0  0  0
 26 31  1  0  0  0  0
 26 32  1  0  0  0  0
 27 33  1  0  0  0  0
 27 34  1  0  0  0  0
 27 35  1  0  0  0  0
 28 39  1  0  0  0  0
 28 40  1  0  0  0  0
 28 41  1  0  0  0  0
 29 36  1  0  0  0  0
 29 37  1  0  0  0  0
 29 38  1  0  0  0  0
 30 48  1  0  0  0  0
 30 49  1  0  0  0  0
 30 50  1  0  0  0  0
 31 42  1  0  0  0  0
 31 43  1  0  0  0  0
 31 44  1  0  0  0  0
 32 45  1  0  0  0  0
 32 46  1  0  0  0  0
 32 47  1  0  0  0  0
M  END
$$$$
mol103
Optimized Catalyst 103
Optimized Catalyst 103
 71 76  0  0  0  0  0  0  0  0999 V2000
   -1.6231   -0.2495   -0.6386 C   0  0  0  0  0  0  0  0  0  0  0  0
   -1.2344    0.9521    0.1768 C   0  0  0  0  0  0  0  0  0  0  0  0
   -1.8500    1.7062   -3.2477 C   0  0  0  0  0  0  0  0  0  0  0  0
   -1.9765    2.2441   -0.2343 C   0  0  0  0  0  0  0  0  0  0  0  0
   -1.5592    3.0965   -2.6609 C   0  0  0  0  0  0  0  0  0  0  0  0
   -1.1700    3.1576   -1.1734 C   0  0  0  0  0  0  0  0  0  0  0  0
   -0.1300    1.1125    0.0935 H   0  0  0  0  0  0  0  0  0  0  0  0
   -2.9472    1.9764   -0.7117 H   0  0  0  0  0  0  0  0  0  0  0  0
   -2.8632    1.3756   -2.9140 H   0  0  0  0  0  0  0  0  0  0  0  0
   -2.4761    3.7207   -2.8130 H   0  0  0  0  0  0  0  0  0  0  0  0
   -2.1321   -1.0528   -0.0536 H   0  0  0  0  0  0  0  0  0  0  0  0
   -1.4632    0.7471    1.2528 H   0  0  0  0  0  0  0  0  0  0  0  0
   -1.8769    1.8063   -4.3613 H   0  0  0  0  0  0  0  0  0  0  0  0
   -2.2018    2.8181    0.6988 H   0  0  0  0  0  0  0  0  0  0  0  0
   -0.7272    3.5633   -3.2461 H   0  0  0  0  0  0  0  0  0  0  0  0
   -1.3171    4.2173   -0.8391 H   0  0  0  0  0  0  0  0  0  0  0  0
   -1.4336   -0.4048   -1.9616 C   0  0  0  0  0  0  0  0  0  0  0  0
```

```
   -1.8222   -1.3134   -2.4837 H   0  0  0  0  0  0  0  0  0  0  0  0
   -0.8183    0.6188   -2.8755 C   0  0  0  0  0  0  0  0  0  0  0  0
    0.0635    1.0933   -2.3802 H   0  0  0  0  0  0  0  0  0  0  0  0
   -0.4608    0.1219   -3.8094 H   0  0  0  0  0  0  0  0  0  0  0  0
    0.8052   -3.7102   -1.4399 C   0  0  0  0  0  0  0  0  0  0  0  0
    1.2231   -2.9441   -2.6716 C   0  0  0  0  0  0  0  0  0  0  0  0
    2.7731   -1.7064    0.2886 C   0  0  0  0  0  0  0  0  0  0  0  0
    2.7148   -2.5362   -2.6676 C   0  0  0  0  0  0  0  0  0  0  0  0
    3.4442   -0.8609   -0.8048 C   0  0  0  0  0  0  0  0  0  0  0  0
    2.9679   -1.0770   -2.2524 C   0  0  0  0  0  0  0  0  0  0  0  0
    0.5642   -2.0284   -2.7667 H   0  0  0  0  0  0  0  0  0  0  0  0
    3.2843   -3.2241   -1.9988 H   0  0  0  0  0  0  0  0  0  0  0  0
    3.1885   -2.7436    0.2511 H   0  0  0  0  0  0  0  0  0  0  0  0
    4.5435   -1.0739   -0.7592 H   0  0  0  0  0  0  0  0  0  0  0  0
    0.7104   -4.8031   -1.5799 H   0  0  0  0  0  0  0  0  0  0  0  0
    1.0318   -3.5853   -3.5717 H   0  0  0  0  0  0  0  0  0  0  0  0
    3.0527   -1.2682    1.2797 H   0  0  0  0  0  0  0  0  0  0  0  0
    3.1061   -2.6754   -3.7073 H   0  0  0  0  0  0  0  0  0  0  0  0
    3.2970    0.2214   -0.5601 H   0  0  0  0  0  0  0  0  0  0  0  0
    3.7651   -0.6604   -2.9230 H   0  0  0  0  0  0  0  0  0  0  0  0
    0.7825   -3.1753   -0.1360 C   0  0  0  0  0  0  0  0  0  0  0  0
    0.6690   -3.8518    0.7310 H   0  0  0  0  0  0  0  0  0  0  0  0
    1.2332   -1.7752    0.1989 C   0  0  0  0  0  0  0  0  0  0  0  0
    0.8597   -1.0638   -0.5912 H   0  0  0  0  0  0  0  0  0  0  0  0
    0.7805   -1.4691    1.1762 H   0  0  0  0  0  0  0  0  0  0  0  0
    2.0471   -0.4723   -2.4283 H   0  0  0  0  0  0  0  0  0  0  0  0
   -0.0819    2.9328   -1.0730 H   0  0  0  0  0  0  0  0  0  0  0  0
   -2.0715   -5.4337   -0.0692 C   0  0  0  0  0  0  0  0  0  0  0  0
   -2.6457   -6.6613    0.3729 C   0  0  0  0  0  0  0  0  0  0  0  0
   -2.3391   -7.1573    1.6571 C   0  0  0  0  0  0  0  0  0  0  0  0
   -3.5150   -7.3688   -0.4836 C   0  0  0  0  0  0  0  0  0  0  0  0
   -2.8973   -8.3586    2.0884 C   0  0  0  0  0  0  0  0  0  0  0  0
   -1.6663   -6.6018    2.3134 H   0  0  0  0  0  0  0  0  0  0  0  0
   -4.0790   -8.5707   -0.0617 C   0  0  0  0  0  0  0  0  0  0  0  0
   -3.7438   -6.9763   -1.4762 H   0  0  0  0  0  0  0  0  0  0  0  0
   -3.7570   -9.0414    1.2181 C   0  0  0  0  0  0  0  0  0  0  0  0
   -2.6696   -8.7582    3.0807 H   0  0  0  0  0  0  0  0  0  0  0  0
   -4.7531   -9.1333   -0.7131 H   0  0  0  0  0  0  0  0  0  0  0  0
   -1.5806   -4.4403   -0.4296 N   0  0  0  0  0  0  0  0  0  0  0  0
   -1.0813   -2.9578   -1.0084 Rh  0  0  0  0  0  0  0  0  0  0  0  0
   -4.8502   -2.6225   -1.2381 C   0  0  0  0  0  0  0  0  0  0  0  0
   -6.2695   -2.4764   -1.1676 C   0  0  0  0  0  0  0  0  0  0  0  0
   -7.1013   -3.3840   -1.8535 C   0  0  0  0  0  0  0  0  0  0  0  0
   -6.8218   -1.4291   -0.4023 C   0  0  0  0  0  0  0  0  0  0  0  0
   -8.4858   -3.2520   -1.7753 C   0  0  0  0  0  0  0  0  0  0  0  0
   -6.6613   -4.1891   -2.4461 H   0  0  0  0  0  0  0  0  0  0  0  0
   -8.2046   -1.2865   -0.3163 C   0  0  0  0  0  0  0  0  0  0  0  0
   -6.1658   -0.7307    0.1228 H   0  0  0  0  0  0  0  0  0  0  0  0
   -9.0082   -2.2032   -1.0070 C   0  0  0  0  0  0  0  0  0  0  0  0
   -9.1461   -3.9475   -2.2999 H   0  0  0  0  0  0  0  0  0  0  0  0
   -8.6498   -0.4813    0.2742 H   0  0  0  0  0  0  0  0  0  0  0  0
   -3.6979   -2.7438   -1.2836 N   0  0  0  0  0  0  0  0  0  0  0  0
   -4.2920  -10.1872    1.6248 F   0  0  0  0  0  0  0  0  0  0  0  0
  -10.3275   -2.0720   -0.9294 F   0  0  0  0  0  0  0  0  0  0  0  0
  1  2  1  0  0  0  0
  1 11  1  0  0  0  0
  1 17  2  0  0  0  0
  1 57  1  0  0  0  0
  2  4  1  0  0  0  0
  2  7  1  0  0  0  0
  2 12  1  0  0  0  0
  3  5  1  0  0  0  0
  3  9  1  0  0  0  0
  3 13  1  0  0  0  0
  3 19  1  0  0  0  0
```

```
  4  6  1  0  0  0  0
  4  8  1  0  0  0  0
  4 14  1  0  0  0  0
  5  6  1  0  0  0  0
  5 10  1  0  0  0  0
  5 15  1  0  0  0  0
  6 16  1  0  0  0  0
  6 44  1  0  0  0  0
 17 18  1  0  0  0  0
 17 19  1  0  0  0  0
 17 57  1  0  0  0  0
 19 20  1  0  0  0  0
 19 21  1  0  0  0  0
 22 23  1  0  0  0  0
 22 32  1  0  0  0  0
 22 38  2  0  0  0  0
 22 57  1  0  0  0  0
 23 25  1  0  0  0  0
 23 28  1  0  0  0  0
 23 33  1  0  0  0  0
 24 26  1  0  0  0  0
 24 30  1  0  0  0  0
 24 34  1  0  0  0  0
 24 40  1  0  0  0  0
 25 27  1  0  0  0  0
 25 29  1  0  0  0  0
 25 35  1  0  0  0  0
 26 27  1  0  0  0  0
 26 31  1  0  0  0  0
 26 36  1  0  0  0  0
 27 37  1  0  0  0  0
 27 43  1  0  0  0  0
 38 39  1  0  0  0  0
 38 40  1  0  0  0  0
 38 57  1  0  0  0  0
 40 41  1  0  0  0  0
 40 42  1  0  0  0  0
 45 46  1  0  0  0  0
 45 56  3  0  0  0  0
 46 47  2  0  0  0  0
 46 48  1  0  0  0  0
 47 49  1  0  0  0  0
 47 50  1  0  0  0  0
 48 51  2  0  0  0  0
 48 52  1  0  0  0  0
 49 53  2  0  0  0  0
 49 54  1  0  0  0  0
 51 53  1  0  0  0  0
 51 55  1  0  0  0  0
 53 70  1  0  0  0  0
 56 57  1  0  0  0  0
 57 69  1  0  0  0  0
 58 59  1  0  0  0  0
 58 69  3  0  0  0  0
 59 60  2  0  0  0  0
 59 61  1  0  0  0  0
 60 62  1  0  0  0  0
 60 63  1  0  0  0  0
 61 64  2  0  0  0  0
 61 65  1  0  0  0  0
 62 66  2  0  0  0  0
 62 67  1  0  0  0  0
 64 66  1  0  0  0  0
 64 68  1  0  0  0  0
 66 71  1  0  0  0  0
```

```
M  END
$$$$
mol104
Optimized Catalyst 104
Optimized Catalyst 104
 71 76  0  0  0  0  0  0  0  0999 V2000
   -1.6150    0.2716   -0.1075 C   0  0  0  0  0  0  0  0  0  0  0  0
   -1.1942    1.6145    0.4095 C   0  0  0  0  0  0  0  0  0  0  0  0
   -2.0292    1.5990   -3.0496 C   0  0  0  0  0  0  0  0  0  0  0  0
   -1.9181    2.8150   -0.2361 C   0  0  0  0  0  0  0  0  0  0  0  0
   -1.6825    3.0767   -2.8112 C   0  0  0  0  0  0  0  0  0  0  0  0
   -1.1597    3.4481   -1.4141 C   0  0  0  0  0  0  0  0  0  0  0  0
   -0.0864    1.7186    0.2628 H   0  0  0  0  0  0  0  0  0  0  0  0
   -2.9376    2.5059   -0.5617 H   0  0  0  0  0  0  0  0  0  0  0  0
   -3.0050    1.3713   -2.5577 H   0  0  0  0  0  0  0  0  0  0  0  0
   -2.6091    3.6733   -3.0128 H   0  0  0  0  0  0  0  0  0  0  0  0
   -2.0572   -0.3917    0.6626 H   0  0  0  0  0  0  0  0  0  0  0  0
   -1.3885    1.6498    1.5138 H   0  0  0  0  0  0  0  0  0  0  0  0
   -2.1625    1.4565   -4.1517 H   0  0  0  0  0  0  0  0  0  0  0  0
   -2.0387    3.5976    0.5568 H   0  0  0  0  0  0  0  0  0  0  0  0
   -0.9083    3.3893   -3.5571 H   0  0  0  0  0  0  0  0  0  0  0  0
   -1.2399    4.5634   -1.3202 H   0  0  0  0  0  0  0  0  0  0  0  0
   -1.4623   -0.1712   -1.3656 C   0  0  0  0  0  0  0  0  0  0  0  0
   -1.8082   -1.2804   -1.6480 H   0  0  0  0  0  0  0  0  0  0  0  0
   -0.9631    0.5983   -2.5504 C   0  0  0  0  0  0  0  0  0  0  0  0
   -0.0330    1.1558   -2.2600 H   0  0  0  0  0  0  0  0  0  0  0  0
   -0.6948   -0.0904   -3.3879 H   0  0  0  0  0  0  0  0  0  0  0  0
    0.5259   -3.4517   -1.5972 C   0  0  0  0  0  0  0  0  0  0  0  0
    1.1785   -2.8599   -2.8179 C   0  0  0  0  0  0  0  0  0  0  0  0
    2.7544   -1.9667    0.2462 C   0  0  0  0  0  0  0  0  0  0  0  0
    2.7234   -2.8411   -2.7052 C   0  0  0  0  0  0  0  0  0  0  0  0
    3.7089   -1.3660   -0.7969 C   0  0  0  0  0  0  0  0  0  0  0  0
    3.3023   -1.4831   -2.2758 C   0  0  0  0  0  0  0  0  0  0  0  0
    0.8122   -1.8187   -2.9992 H   0  0  0  0  0  0  0  0  0  0  0  0
    3.0585   -3.6357   -1.9978 H   0  0  0  0  0  0  0  0  0  0  0  0
    2.8893   -3.0761    0.2623 H   0  0  0  0  0  0  0  0  0  0  0  0
    4.7016   -1.8702   -0.6638 H   0  0  0  0  0  0  0  0  0  0  0  0
    0.1678   -4.4951   -1.7409 H   0  0  0  0  0  0  0  0  0  0  0  0
    0.9062   -3.4776   -3.7140 H   0  0  0  0  0  0  0  0  0  0  0  0
    3.0573   -1.5774    1.2513 H   0  0  0  0  0  0  0  0  0  0  0  0
    3.1351   -3.0932   -3.7158 H   0  0  0  0  0  0  0  0  0  0  0  0
    3.8483   -0.2786   -0.5703 H   0  0  0  0  0  0  0  0  0  0  0  0
    4.2285   -1.2974   -2.8827 H   0  0  0  0  0  0  0  0  0  0  0  0
    0.5210   -2.8975   -0.3510 C   0  0  0  0  0  0  0  0  0  0  0  0
    0.1823   -3.4957    0.5279 H   0  0  0  0  0  0  0  0  0  0  0  0
    1.2589   -1.6355    0.0253 C   0  0  0  0  0  0  0  0  0  0  0  0
    1.1702   -0.8565   -0.7723 H   0  0  0  0  0  0  0  0  0  0  0  0
    0.8377   -1.2149    0.9727 H   0  0  0  0  0  0  0  0  0  0  0  0
    2.5846   -0.6667   -2.5256 H   0  0  0  0  0  0  0  0  0  0  0  0
   -0.0749    3.1966   -1.3467 H   0  0  0  0  0  0  0  0  0  0  0  0
   -2.3924   -5.6985   -0.0422 C   0  0  0  0  0  0  0  0  0  0  0  0
   -2.6308   -7.0284    0.3911 C   0  0  0  0  0  0  0  0  0  0  0  0
   -2.2838   -7.4095    1.7078 C   0  0  0  0  0  0  0  0  0  0  0  0
   -3.2171   -7.9580   -0.4984 C   0  0  0  0  0  0  0  0  0  0  0  0
   -2.5256   -8.7097    2.1376 C   0  0  0  0  0  0  0  0  0  0  0  0
   -1.8281   -6.6837    2.3868 H   0  0  0  0  0  0  0  0  0  0  0  0
   -3.4638   -9.2593   -0.0754 C   0  0  0  0  0  0  0  0  0  0  0  0
   -3.4762   -7.6541   -1.5159 H   0  0  0  0  0  0  0  0  0  0  0  0
   -3.1136   -9.6105    1.2372 C   0  0  0  0  0  0  0  0  0  0  0  0
   -2.2646   -9.0233    3.1544 H   0  0  0  0  0  0  0  0  0  0  0  0
   -3.9194   -9.9927   -0.7490 H   0  0  0  0  0  0  0  0  0  0  0  0
   -2.1830   -4.6062   -0.4005 N   0  0  0  0  0  0  0  0  0  0  0  0
   -5.0265   -2.3662   -1.1218 C   0  0  0  0  0  0  0  0  0  0  0  0
   -6.4386   -2.2360   -1.1861 C   0  0  0  0  0  0  0  0  0  0  0  0
   -7.1762   -3.0770   -2.0508 C   0  0  0  0  0  0  0  0  0  0  0  0
```

```
   -7.0926   -1.2726   -0.3850 C   0  0  0  0  0  0  0  0  0  0  0  0
   -8.5606   -2.9630   -2.1117 C   0  0  0  0  0  0  0  0  0  0  0  0
   -6.6592   -3.8146   -2.6701 H   0  0  0  0  0  0  0  0  0  0  0  0
   -8.4770   -1.1511   -0.4415 C   0  0  0  0  0  0  0  0  0  0  0  0
   -6.5120   -0.6256    0.2779 H   0  0  0  0  0  0  0  0  0  0  0  0
   -9.1856   -2.0012   -1.3035 C   0  0  0  0  0  0  0  0  0  0  0  0
   -9.1487   -3.6062   -2.7748 H   0  0  0  0  0  0  0  0  0  0  0  0
   -9.0016   -0.4104    0.1716 H   0  0  0  0  0  0  0  0  0  0  0  0
   -3.8641   -2.4694   -1.0720 N   0  0  0  0  0  0  0  0  0  0  0  0
   -3.3511  -10.8489    1.6451 F   0  0  0  0  0  0  0  0  0  0  0  0
  -10.5054   -1.8919   -1.3568 F   0  0  0  0  0  0  0  0  0  0  0  0
   -1.9259   -2.7528   -0.9771 Ir  0  0  0  0  0  0  0  0  0  0  0  0
  1  2  1  0  0  0  0
  1 11  1  0  0  0  0
  1 17  2  0  0  0  0
  1 71  1  0  0  0  0
  2  4  1  0  0  0  0
  2  7  1  0  0  0  0
  2 12  1  0  0  0  0
  3  5  1  0  0  0  0
  3  9  1  0  0  0  0
  3 13  1  0  0  0  0
  3 19  1  0  0  0  0
  4  6  1  0  0  0  0
  4  8  1  0  0  0  0
  4 14  1  0  0  0  0
  5  6  1  0  0  0  0
  5 10  1  0  0  0  0
  5 15  1  0  0  0  0
  6 16  1  0  0  0  0
  6 44  1  0  0  0  0
 17 19  1  0  0  0  0
 17 71  1  0  0  0  0
 17 18  1  0  0  0  0
 19 20  1  0  0  0  0
 19 21  1  0  0  0  0
 22 23  1  0  0  0  0
 22 32  1  0  0  0  0
 22 38  2  0  0  0  0
 22 71  1  0  0  0  0
 23 25  1  0  0  0  0
 23 28  1  0  0  0  0
 23 33  1  0  0  0  0
 24 26  1  0  0  0  0
 24 30  1  0  0  0  0
 24 34  1  0  0  0  0
 24 40  1  0  0  0  0
 25 27  1  0  0  0  0
 25 29  1  0  0  0  0
 25 35  1  0  0  0  0
 26 27  1  0  0  0  0
 26 31  1  0  0  0  0
 26 36  1  0  0  0  0
 27 37  1  0  0  0  0
 27 43  1  0  0  0  0
 38 39  1  0  0  0  0
 38 40  1  0  0  0  0
 38 71  1  0  0  0  0
 40 41  1  0  0  0  0
 40 42  1  0  0  0  0
 45 46  1  0  0  0  0
 45 56  3  0  0  0  0
 46 47  2  0  0  0  0
 46 48  1  0  0  0  0
 47 49  1  0  0  0  0
```

```
 47 50  1  0  0  0  0
 48 51  2  0  0  0  0
 48 52  1  0  0  0  0
 49 53  2  0  0  0  0
 49 54  1  0  0  0  0
 51 53  1  0  0  0  0
 51 55  1  0  0  0  0
 53 69  1  0  0  0  0
 56 71  1  0  0  0  0
 57 58  1  0  0  0  0
 57 68  3  0  0  0  0
 58 59  2  0  0  0  0
 58 60  1  0  0  0  0
 59 61  1  0  0  0  0
 59 62  1  0  0  0  0
 60 63  2  0  0  0  0
 60 64  1  0  0  0  0
 61 65  2  0  0  0  0
 61 66  1  0  0  0  0
 63 65  1  0  0  0  0
 63 67  1  0  0  0  0
 65 70  1  0  0  0  0
 68 71  1  0  0  0  0
M  END
$$$$
mol105
Optimized Catalyst 105
Optimized Catalyst 105
 77 82  0  0  0  0  0  0  0  0999 V2000
   -1.6241   -0.2484   -0.6389 C   0  0  0  0  0  0  0  0  0  0  0  0
   -1.2340    0.9525    0.1770 C   0  0  0  0  0  0  0  0  0  0  0  0
   -1.8494    1.7061   -3.2476 C   0  0  0  0  0  0  0  0  0  0  0  0
   -1.9760    2.2447   -0.2332 C   0  0  0  0  0  0  0  0  0  0  0  0
   -1.5600    3.0966   -2.6605 C   0  0  0  0  0  0  0  0  0  0  0  0
   -1.1702    3.1582   -1.1731 C   0  0  0  0  0  0  0  0  0  0  0  0
   -0.1297    1.1120    0.0932 H   0  0  0  0  0  0  0  0  0  0  0  0
   -2.9472    1.9769   -0.7099 H   0  0  0  0  0  0  0  0  0  0  0  0
   -2.8626    1.3745   -2.9138 H   0  0  0  0  0  0  0  0  0  0  0  0
   -2.4777    3.7195   -2.8118 H   0  0  0  0  0  0  0  0  0  0  0  0
   -2.1357   -1.0509   -0.0544 H   0  0  0  0  0  0  0  0  0  0  0  0
   -1.4628    0.7466    1.2528 H   0  0  0  0  0  0  0  0  0  0  0  0
   -1.8766    1.8061   -4.3611 H   0  0  0  0  0  0  0  0  0  0  0  0
   -2.2004    2.8185    0.7001 H   0  0  0  0  0  0  0  0  0  0  0  0
   -0.7288    3.5642   -3.2459 H   0  0  0  0  0  0  0  0  0  0  0  0
   -1.3176    4.2177   -0.8390 H   0  0  0  0  0  0  0  0  0  0  0  0
   -1.4338   -0.4040   -1.9616 C   0  0  0  0  0  0  0  0  0  0  0  0
   -1.8240   -1.3124   -2.4840 H   0  0  0  0  0  0  0  0  0  0  0  0
   -0.8175    0.6189   -2.8756 C   0  0  0  0  0  0  0  0  0  0  0  0
    0.0646    1.0928   -2.3807 H   0  0  0  0  0  0  0  0  0  0  0  0
   -0.4609    0.1212   -3.8094 H   0  0  0  0  0  0  0  0  0  0  0  0
    0.8054   -3.7084   -1.4403 C   0  0  0  0  0  0  0  0  0  0  0  0
    1.2239   -2.9431   -2.6721 C   0  0  0  0  0  0  0  0  0  0  0  0
    2.7742   -1.7063    0.2887 C   0  0  0  0  0  0  0  0  0  0  0  0
    2.7155   -2.5362   -2.6687 C   0  0  0  0  0  0  0  0  0  0  0  0
    3.4459   -0.8616   -0.8051 C   0  0  0  0  0  0  0  0  0  0  0  0
    2.9691   -1.0773   -2.2526 C   0  0  0  0  0  0  0  0  0  0  0  0
    0.5651   -2.0271   -2.7665 H   0  0  0  0  0  0  0  0  0  0  0  0
    3.2848   -3.2246   -2.0002 H   0  0  0  0  0  0  0  0  0  0  0  0
    3.1889   -2.7437    0.2519 H   0  0  0  0  0  0  0  0  0  0  0  0
    4.5449   -1.0753   -0.7597 H   0  0  0  0  0  0  0  0  0  0  0  0
    0.7073   -4.8011   -1.5802 H   0  0  0  0  0  0  0  0  0  0  0  0
    1.0306   -3.5841   -3.5718 H   0  0  0  0  0  0  0  0  0  0  0  0
    3.0536   -1.2676    1.2796 H   0  0  0  0  0  0  0  0  0  0  0  0
    3.1060   -2.6749   -3.7086 H   0  0  0  0  0  0  0  0  0  0  0  0
    3.2987    0.2206   -0.5604 H   0  0  0  0  0  0  0  0  0  0  0  0
```

```
    3.7656   -0.6601   -2.9234 H   0  0  0  0  0  0  0  0  0  0  0  0
    0.7825   -3.1734   -0.1369 C   0  0  0  0  0  0  0  0  0  0  0  0
    0.6649   -3.8495    0.7300 H   0  0  0  0  0  0  0  0  0  0  0  0
    1.2346   -1.7740    0.1986 C   0  0  0  0  0  0  0  0  0  0  0  0
    0.8611   -1.0627   -0.5916 H   0  0  0  0  0  0  0  0  0  0  0  0
    0.7811   -1.4681    1.1756 H   0  0  0  0  0  0  0  0  0  0  0  0
    2.0478   -0.4728   -2.4274 H   0  0  0  0  0  0  0  0  0  0  0  0
   -0.0821    2.9333   -1.0733 H   0  0  0  0  0  0  0  0  0  0  0  0
   -2.0707   -5.4297   -0.0703 C   0  0  0  0  0  0  0  0  0  0  0  0
   -2.6429   -6.6571    0.3713 C   0  0  0  0  0  0  0  0  0  0  0  0
   -2.3395   -7.1575    1.6527 C   0  0  0  0  0  0  0  0  0  0  0  0
   -3.5113   -7.3682   -0.4803 C   0  0  0  0  0  0  0  0  0  0  0  0
   -2.9052   -8.3575    2.0740 C   0  0  0  0  0  0  0  0  0  0  0  0
   -1.6669   -6.6058    2.3108 H   0  0  0  0  0  0  0  0  0  0  0  0
   -4.0709   -8.5669   -0.0472 C   0  0  0  0  0  0  0  0  0  0  0  0
   -3.7418   -6.9792   -1.4732 H   0  0  0  0  0  0  0  0  0  0  0  0
   -3.7807   -9.0726    1.2344 C   0  0  0  0  0  0  0  0  0  0  0  0
   -2.6653   -8.7486    3.0646 H   0  0  0  0  0  0  0  0  0  0  0  0
   -4.7380   -9.1215   -0.7094 H   0  0  0  0  0  0  0  0  0  0  0  0
   -1.5777   -4.4383   -0.4304 N   0  0  0  0  0  0  0  0  0  0  0  0
   -4.8412   -2.6237   -1.2379 C   0  0  0  0  0  0  0  0  0  0  0  0
   -6.2597   -2.4772   -1.1690 C   0  0  0  0  0  0  0  0  0  0  0  0
   -7.0973   -3.3794   -1.8522 C   0  0  0  0  0  0  0  0  0  0  0  0
   -6.8192   -1.4330   -0.4073 C   0  0  0  0  0  0  0  0  0  0  0  0
   -8.4795   -3.2396   -1.7652 C   0  0  0  0  0  0  0  0  0  0  0  0
   -6.6623   -4.1846   -2.4465 H   0  0  0  0  0  0  0  0  0  0  0  0
   -8.2024   -1.3014   -0.3265 C   0  0  0  0  0  0  0  0  0  0  0  0
   -6.1683   -0.7317    0.1180 H   0  0  0  0  0  0  0  0  0  0  0  0
   -9.0482   -2.2050   -0.9979 C   0  0  0  0  0  0  0  0  0  0  0  0
   -9.1277   -3.9363   -2.2993 H   0  0  0  0  0  0  0  0  0  0  0  0
   -8.6347   -0.4893    0.2606 H   0  0  0  0  0  0  0  0  0  0  0  0
   -3.6884   -2.7441   -1.2826 N   0  0  0  0  0  0  0  0  0  0  0  0
   -4.3809  -10.3588    1.6910 C   0  0  0  0  0  0  0  0  0  0  0  0
   -5.3812  -10.5302    1.2255 H   0  0  0  0  0  0  0  0  0  0  0  0
   -3.7071  -11.2049    1.3999 H   0  0  0  0  0  0  0  0  0  0  0  0
   -4.5096  -10.3780    2.8001 H   0  0  0  0  0  0  0  0  0  0  0  0
  -10.5293   -2.0566   -0.9113 C   0  0  0  0  0  0  0  0  0  0  0  0
  -10.8806   -1.3816   -1.7332 H   0  0  0  0  0  0  0  0  0  0  0  0
  -11.0457   -3.0405   -1.0198 H   0  0  0  0  0  0  0  0  0  0  0  0
  -10.8381   -1.6113    0.0652 H   0  0  0  0  0  0  0  0  0  0  0  0
   -1.0815   -2.9506   -1.0118 Rh  0  0  0  0  0  0  0  0  0  0  0  0
  1  2  1  0  0  0  0
  1 11  1  0  0  0  0
  1 17  2  0  0  0  0
  1 77  1  0  0  0  0
  2  4  1  0  0  0  0
  2  7  1  0  0  0  0
  2 12  1  0  0  0  0
  3  5  1  0  0  0  0
  3  9  1  0  0  0  0
  3 13  1  0  0  0  0
  3 19  1  0  0  0  0
  4  6  1  0  0  0  0
  4  8  1  0  0  0  0
  4 14  1  0  0  0  0
  5  6  1  0  0  0  0
  5 10  1  0  0  0  0
  5 15  1  0  0  0  0
  6 16  1  0  0  0  0
  6 44  1  0  0  0  0
 17 18  1  0  0  0  0
 17 19  1  0  0  0  0
 17 77  1  0  0  0  0
 19 20  1  0  0  0  0
 19 21  1  0  0  0  0
```

```
 22 23  1  0  0  0  0
 22 32  1  0  0  0  0
 22 38  2  0  0  0  0
 22 77  1  0  0  0  0
 23 25  1  0  0  0  0
 23 28  1  0  0  0  0
 23 33  1  0  0  0  0
 24 26  1  0  0  0  0
 24 30  1  0  0  0  0
 24 34  1  0  0  0  0
 24 40  1  0  0  0  0
 25 27  1  0  0  0  0
 25 29  1  0  0  0  0
 25 35  1  0  0  0  0
 26 27  1  0  0  0  0
 26 31  1  0  0  0  0
 26 36  1  0  0  0  0
 27 37  1  0  0  0  0
 27 43  1  0  0  0  0
 38 39  1  0  0  0  0
 38 40  1  0  0  0  0
 38 77  1  0  0  0  0
 40 41  1  0  0  0  0
 40 42  1  0  0  0  0
 45 46  1  0  0  0  0
 45 56  3  0  0  0  0
 46 47  2  0  0  0  0
 46 48  1  0  0  0  0
 47 49  1  0  0  0  0
 47 50  1  0  0  0  0
 48 51  2  0  0  0  0
 48 52  1  0  0  0  0
 49 53  2  0  0  0  0
 49 54  1  0  0  0  0
 51 53  1  0  0  0  0
 51 55  1  0  0  0  0
 53 69  1  0  0  0  0
 56 77  1  0  0  0  0
 57 58  1  0  0  0  0
 57 68  3  0  0  0  0
 58 59  2  0  0  0  0
 58 60  1  0  0  0  0
 59 61  1  0  0  0  0
 59 62  1  0  0  0  0
 60 63  2  0  0  0  0
 60 64  1  0  0  0  0
 61 65  2  0  0  0  0
 61 66  1  0  0  0  0
 63 65  1  0  0  0  0
 63 67  1  0  0  0  0
 65 73  1  0  0  0  0
 68 77  1  0  0  0  0
 69 70  1  0  0  0  0
 69 71  1  0  0  0  0
 69 72  1  0  0  0  0
 73 74  1  0  0  0  0
 73 75  1  0  0  0  0
 73 76  1  0  0  0  0
M  END
$$$$
mol106
Optimized Catalyst 106
Optimized Catalyst 106
 77 82  0  0  0  0  0  0  0  0999 V2000
   -1.5558    0.2976   -0.1174 C   0  0  0  0  0  0  0  0  0  0  0  0
```

```
   -1.1492    1.6473    0.3935 C   0  0  0  0  0  0  0  0  0  0  0  0
   -2.0207    1.6199   -3.0569 C   0  0  0  0  0  0  0  0  0  0  0  0
   -1.9069    2.8327   -0.2412 C   0  0  0  0  0  0  0  0  0  0  0  0
   -1.6969    3.1028   -2.8196 C   0  0  0  0  0  0  0  0  0  0  0  0
   -1.1738    3.4842   -1.4252 C   0  0  0  0  0  0  0  0  0  0  0  0
   -0.0466    1.7730    0.2285 H   0  0  0  0  0  0  0  0  0  0  0  0
   -2.9223    2.5015   -0.5579 H   0  0  0  0  0  0  0  0  0  0  0  0
   -2.9905    1.3754   -2.5606 H   0  0  0  0  0  0  0  0  0  0  0  0
   -2.6335    3.6840   -3.0186 H   0  0  0  0  0  0  0  0  0  0  0  0
   -1.9787   -0.3716    0.6587 H   0  0  0  0  0  0  0  0  0  0  0  0
   -1.3257    1.6778    1.5006 H   0  0  0  0  0  0  0  0  0  0  0  0
   -2.1566    1.4758   -4.1584 H   0  0  0  0  0  0  0  0  0  0  0  0
   -2.0368    3.6106    0.5545 H   0  0  0  0  0  0  0  0  0  0  0  0
   -0.9302    3.4265   -3.5685 H   0  0  0  0  0  0  0  0  0  0  0  0
   -1.2781    4.5971   -1.3292 H   0  0  0  0  0  0  0  0  0  0  0  0
   -1.4149   -0.1419   -1.3779 C   0  0  0  0  0  0  0  0  0  0  0  0
   -1.7495   -1.2548   -1.6589 H   0  0  0  0  0  0  0  0  0  0  0  0
   -0.9356    0.6365   -2.5649 C   0  0  0  0  0  0  0  0  0  0  0  0
   -0.0132    1.2070   -2.2758 H   0  0  0  0  0  0  0  0  0  0  0  0
   -0.6591   -0.0457   -3.4050 H   0  0  0  0  0  0  0  0  0  0  0  0
    0.5346   -3.4429   -1.5999 C   0  0  0  0  0  0  0  0  0  0  0  0
    1.1899   -2.8555   -2.8216 C   0  0  0  0  0  0  0  0  0  0  0  0
    2.7908   -1.9849    0.2352 C   0  0  0  0  0  0  0  0  0  0  0  0
    2.7356   -2.8675   -2.7164 C   0  0  0  0  0  0  0  0  0  0  0  0
    3.7495   -1.3999   -0.8127 C   0  0  0  0  0  0  0  0  0  0  0  0
    3.3416   -1.5201   -2.2911 C   0  0  0  0  0  0  0  0  0  0  0  0
    0.8423   -1.8061   -2.9932 H   0  0  0  0  0  0  0  0  0  0  0  0
    3.0575   -3.6685   -2.0101 H   0  0  0  0  0  0  0  0  0  0  0  0
    2.9121   -3.0958    0.2560 H   0  0  0  0  0  0  0  0  0  0  0  0
    4.7375   -1.9119   -0.6770 H   0  0  0  0  0  0  0  0  0  0  0  0
    0.1610   -4.4809   -1.7435 H   0  0  0  0  0  0  0  0  0  0  0  0
    0.8997   -3.4604   -3.7205 H   0  0  0  0  0  0  0  0  0  0  0  0
    3.1017   -1.5947    1.2373 H   0  0  0  0  0  0  0  0  0  0  0  0
    3.1383   -3.1267   -3.7288 H   0  0  0  0  0  0  0  0  0  0  0  0
    3.8974   -0.3122   -0.5928 H   0  0  0  0  0  0  0  0  0  0  0  0
    4.2714   -1.3546   -2.8982 H   0  0  0  0  0  0  0  0  0  0  0  0
    0.5452   -2.8904   -0.3531 C   0  0  0  0  0  0  0  0  0  0  0  0
    0.2039   -3.4851    0.5268 H   0  0  0  0  0  0  0  0  0  0  0  0
    1.2993   -1.6361    0.0170 C   0  0  0  0  0  0  0  0  0  0  0  0
    1.2176   -0.8588   -0.7833 H   0  0  0  0  0  0  0  0  0  0  0  0
    0.8852   -1.2072    0.9637 H   0  0  0  0  0  0  0  0  0  0  0  0
    2.6386   -0.6925   -2.5459 H   0  0  0  0  0  0  0  0  0  0  0  0
   -0.0832    3.2567   -1.3663 H   0  0  0  0  0  0  0  0  0  0  0  0
   -2.4860   -5.6346   -0.0097 C   0  0  0  0  0  0  0  0  0  0  0  0
   -2.7990   -6.9458    0.4263 C   0  0  0  0  0  0  0  0  0  0  0  0
   -2.5058   -7.3434    1.7493 C   0  0  0  0  0  0  0  0  0  0  0  0
   -3.4023   -7.8583   -0.4689 C   0  0  0  0  0  0  0  0  0  0  0  0
   -2.8121   -8.6337    2.1646 C   0  0  0  0  0  0  0  0  0  0  0  0
   -2.0401   -6.6385    2.4413 H   0  0  0  0  0  0  0  0  0  0  0  0
   -3.7042   -9.1437   -0.0406 C   0  0  0  0  0  0  0  0  0  0  0  0
   -3.6272   -7.5510   -1.4922 H   0  0  0  0  0  0  0  0  0  0  0  0
   -3.4150   -9.5500    1.2797 C   0  0  0  0  0  0  0  0  0  0  0  0
   -2.5790   -8.9412    3.1875 H   0  0  0  0  0  0  0  0  0  0  0  0
   -4.1661   -9.8522   -0.7332 H   0  0  0  0  0  0  0  0  0  0  0  0
   -2.2076   -4.5603   -0.3766 N   0  0  0  0  0  0  0  0  0  0  0  0
   -5.0049   -2.3072   -1.1064 C   0  0  0  0  0  0  0  0  0  0  0  0
   -6.4175   -2.1934   -1.1481 C   0  0  0  0  0  0  0  0  0  0  0  0
   -7.1658   -3.0462   -1.9904 C   0  0  0  0  0  0  0  0  0  0  0  0
   -7.0778   -1.2422   -0.3401 C   0  0  0  0  0  0  0  0  0  0  0  0
   -8.5518   -2.9598   -2.0001 C   0  0  0  0  0  0  0  0  0  0  0  0
   -6.6545   -3.7691   -2.6290 H   0  0  0  0  0  0  0  0  0  0  0  0
   -8.4654   -1.1655   -0.3607 C   0  0  0  0  0  0  0  0  0  0  0  0
   -6.4986   -0.5724    0.2988 H   0  0  0  0  0  0  0  0  0  0  0  0
   -9.2217   -2.0287   -1.1788 C   0  0  0  0  0  0  0  0  0  0  0  0
   -9.1319   -3.6179   -2.6518 H   0  0  0  0  0  0  0  0  0  0  0  0
```

```
   -8.9753   -0.4275    0.2639 H   0  0  0  0  0  0  0  0  0  0  0  0
   -3.8412   -2.4035   -1.0699 N   0  0  0  0  0  0  0  0  0  0  0  0
   -3.7314  -10.9363    1.7171 C   0  0  0  0  0  0  0  0  0  0  0  0
   -4.7476  -11.2447    1.3659 H   0  0  0  0  0  0  0  0  0  0  0  0
   -2.9800  -11.6449    1.2802 H   0  0  0  0  0  0  0  0  0  0  0  0
   -3.7040  -11.0437    2.8285 H   0  0  0  0  0  0  0  0  0  0  0  0
  -10.7078   -1.9557   -1.1922 C   0  0  0  0  0  0  0  0  0  0  0  0
  -11.0430   -1.3389   -2.0665 H   0  0  0  0  0  0  0  0  0  0  0  0
  -11.1617   -2.9729   -1.2899 H   0  0  0  0  0  0  0  0  0  0  0  0
  -11.1112   -1.4874   -0.2618 H   0  0  0  0  0  0  0  0  0  0  0  0
   -1.9041   -2.7161   -0.9726 Ir  0  0  0  0  0  0  0  0  0  0  0  0
  1  2  1  0  0  0  0
  1 11  1  0  0  0  0
  1 17  2  0  0  0  0
  1 77  1  0  0  0  0
  2  4  1  0  0  0  0
  2  7  1  0  0  0  0
  2 12  1  0  0  0  0
  3  5  1  0  0  0  0
  3  9  1  0  0  0  0
  3 13  1  0  0  0  0
  3 19  1  0  0  0  0
  4  6  1  0  0  0  0
  4  8  1  0  0  0  0
  4 14  1  0  0  0  0
  5  6  1  0  0  0  0
  5 10  1  0  0  0  0
  5 15  1  0  0  0  0
  6 16  1  0  0  0  0
  6 44  1  0  0  0  0
 17 19  1  0  0  0  0
 17 18  1  0  0  0  0
 17 77  1  0  0  0  0
 19 20  1  0  0  0  0
 19 21  1  0  0  0  0
 22 23  1  0  0  0  0
 22 32  1  0  0  0  0
 22 38  2  0  0  0  0
 22 77  1  0  0  0  0
 23 25  1  0  0  0  0
 23 28  1  0  0  0  0
 23 33  1  0  0  0  0
 24 26  1  0  0  0  0
 24 30  1  0  0  0  0
 24 34  1  0  0  0  0
 24 40  1  0  0  0  0
 25 27  1  0  0  0  0
 25 29  1  0  0  0  0
 25 35  1  0  0  0  0
 26 27  1  0  0  0  0
 26 31  1  0  0  0  0
 26 36  1  0  0  0  0
 27 37  1  0  0  0  0
 27 43  1  0  0  0  0
 38 39  1  0  0  0  0
 38 40  1  0  0  0  0
 38 77  1  0  0  0  0
 40 41  1  0  0  0  0
 40 42  1  0  0  0  0
 45 46  1  0  0  0  0
 45 56  3  0  0  0  0
 46 47  2  0  0  0  0
 46 48  1  0  0  0  0
 47 49  1  0  0  0  0
 47 50  1  0  0  0  0
```

```
 48 51  2  0  0  0  0
 48 52  1  0  0  0  0
 49 53  2  0  0  0  0
 49 54  1  0  0  0  0
 51 53  1  0  0  0  0
 51 55  1  0  0  0  0
 53 69  1  0  0  0  0
 56 77  1  0  0  0  0
 57 58  1  0  0  0  0
 57 68  3  0  0  0  0
 58 59  2  0  0  0  0
 58 60  1  0  0  0  0
 59 61  1  0  0  0  0
 59 62  1  0  0  0  0
 60 63  2  0  0  0  0
 60 64  1  0  0  0  0
 61 65  2  0  0  0  0
 61 66  1  0  0  0  0
 63 65  1  0  0  0  0
 63 67  1  0  0  0  0
 65 73  1  0  0  0  0
 68 77  1  0  0  0  0
 69 70  1  0  0  0  0
 69 71  1  0  0  0  0
 69 72  1  0  0  0  0
 73 74  1  0  0  0  0
 73 75  1  0  0  0  0
 73 76  1  0  0  0  0
M  END
$$$$
mol107
Optimized Catalyst 107
Optimized Catalyst 107
 77 82  0  0  0  0  0  0  0  0999 V2000
   -1.5558    0.2976   -0.1174 C   0  0  0  0  0  0  0  0  0  0  0  0
   -1.1492    1.6473    0.3935 C   0  0  0  0  0  0  0  0  0  0  0  0
   -2.0207    1.6199   -3.0569 C   0  0  0  0  0  0  0  0  0  0  0  0
   -1.9069    2.8327   -0.2412 C   0  0  0  0  0  0  0  0  0  0  0  0
   -1.6969    3.1028   -2.8196 C   0  0  0  0  0  0  0  0  0  0  0  0
   -1.1738    3.4842   -1.4252 C   0  0  0  0  0  0  0  0  0  0  0  0
   -0.0466    1.7730    0.2285 H   0  0  0  0  0  0  0  0  0  0  0  0
   -2.9223    2.5015   -0.5579 H   0  0  0  0  0  0  0  0  0  0  0  0
   -2.9905    1.3754   -2.5606 H   0  0  0  0  0  0  0  0  0  0  0  0
   -2.6335    3.6840   -3.0186 H   0  0  0  0  0  0  0  0  0  0  0  0
   -1.9787   -0.3716    0.6587 H   0  0  0  0  0  0  0  0  0  0  0  0
   -1.3257    1.6778    1.5006 H   0  0  0  0  0  0  0  0  0  0  0  0
   -2.1566    1.4758   -4.1584 H   0  0  0  0  0  0  0  0  0  0  0  0
   -2.0368    3.6106    0.5545 H   0  0  0  0  0  0  0  0  0  0  0  0
   -0.9302    3.4265   -3.5685 H   0  0  0  0  0  0  0  0  0  0  0  0
   -1.2781    4.5971   -1.3292 H   0  0  0  0  0  0  0  0  0  0  0  0
   -1.4149   -0.1419   -1.3779 C   0  0  0  0  0  0  0  0  0  0  0  0
   -1.7495   -1.2548   -1.6589 H   0  0  0  0  0  0  0  0  0  0  0  0
   -0.9356    0.6365   -2.5649 C   0  0  0  0  0  0  0  0  0  0  0  0
   -0.0132    1.2070   -2.2758 H   0  0  0  0  0  0  0  0  0  0  0  0
   -0.6591   -0.0457   -3.4050 H   0  0  0  0  0  0  0  0  0  0  0  0
    0.5346   -3.4429   -1.5999 C   0  0  0  0  0  0  0  0  0  0  0  0
    1.1899   -2.8555   -2.8216 C   0  0  0  0  0  0  0  0  0  0  0  0
    2.7908   -1.9849    0.2352 C   0  0  0  0  0  0  0  0  0  0  0  0
    2.7356   -2.8675   -2.7164 C   0  0  0  0  0  0  0  0  0  0  0  0
    3.7495   -1.3999   -0.8127 C   0  0  0  0  0  0  0  0  0  0  0  0
    3.3416   -1.5201   -2.2911 C   0  0  0  0  0  0  0  0  0  0  0  0
    0.8423   -1.8061   -2.9932 H   0  0  0  0  0  0  0  0  0  0  0  0
    3.0575   -3.6685   -2.0101 H   0  0  0  0  0  0  0  0  0  0  0  0
    2.9121   -3.0958    0.2560 H   0  0  0  0  0  0  0  0  0  0  0  0
    4.7375   -1.9119   -0.6770 H   0  0  0  0  0  0  0  0  0  0  0  0
```

```
    0.1610   -4.4809   -1.7435 H   0  0  0  0  0  0  0  0  0  0  0  0
    0.8997   -3.4604   -3.7205 H   0  0  0  0  0  0  0  0  0  0  0  0
    3.1017   -1.5947    1.2373 H   0  0  0  0  0  0  0  0  0  0  0  0
    3.1383   -3.1267   -3.7288 H   0  0  0  0  0  0  0  0  0  0  0  0
    3.8974   -0.3122   -0.5928 H   0  0  0  0  0  0  0  0  0  0  0  0
    4.2714   -1.3546   -2.8982 H   0  0  0  0  0  0  0  0  0  0  0  0
    0.5452   -2.8904   -0.3531 C   0  0  0  0  0  0  0  0  0  0  0  0
    0.2039   -3.4851    0.5268 H   0  0  0  0  0  0  0  0  0  0  0  0
    1.2993   -1.6361    0.0170 C   0  0  0  0  0  0  0  0  0  0  0  0
    1.2176   -0.8588   -0.7833 H   0  0  0  0  0  0  0  0  0  0  0  0
    0.8852   -1.2072    0.9637 H   0  0  0  0  0  0  0  0  0  0  0  0
    2.6386   -0.6925   -2.5459 H   0  0  0  0  0  0  0  0  0  0  0  0
   -0.0832    3.2567   -1.3663 H   0  0  0  0  0  0  0  0  0  0  0  0
   -2.4860   -5.6346   -0.0097 C   0  0  0  0  0  0  0  0  0  0  0  0
   -2.7990   -6.9458    0.4263 C   0  0  0  0  0  0  0  0  0  0  0  0
   -2.5058   -7.3434    1.7493 C   0  0  0  0  0  0  0  0  0  0  0  0
   -3.4023   -7.8583   -0.4689 C   0  0  0  0  0  0  0  0  0  0  0  0
   -2.8121   -8.6337    2.1646 C   0  0  0  0  0  0  0  0  0  0  0  0
   -2.0401   -6.6385    2.4413 H   0  0  0  0  0  0  0  0  0  0  0  0
   -3.7042   -9.1437   -0.0406 C   0  0  0  0  0  0  0  0  0  0  0  0
   -3.6272   -7.5510   -1.4922 H   0  0  0  0  0  0  0  0  0  0  0  0
   -3.4150   -9.5500    1.2797 C   0  0  0  0  0  0  0  0  0  0  0  0
   -2.5790   -8.9412    3.1875 H   0  0  0  0  0  0  0  0  0  0  0  0
   -4.1661   -9.8522   -0.7332 H   0  0  0  0  0  0  0  0  0  0  0  0
   -2.2076   -4.5603   -0.3766 N   0  0  0  0  0  0  0  0  0  0  0  0
   -5.0049   -2.3072   -1.1064 C   0  0  0  0  0  0  0  0  0  0  0  0
   -6.4175   -2.1934   -1.1481 C   0  0  0  0  0  0  0  0  0  0  0  0
   -7.1658   -3.0462   -1.9904 C   0  0  0  0  0  0  0  0  0  0  0  0
   -7.0778   -1.2422   -0.3401 C   0  0  0  0  0  0  0  0  0  0  0  0
   -8.5518   -2.9598   -2.0001 C   0  0  0  0  0  0  0  0  0  0  0  0
   -6.6545   -3.7691   -2.6290 H   0  0  0  0  0  0  0  0  0  0  0  0
   -8.4654   -1.1655   -0.3607 C   0  0  0  0  0  0  0  0  0  0  0  0
   -6.4986   -0.5724    0.2988 H   0  0  0  0  0  0  0  0  0  0  0  0
   -9.2217   -2.0287   -1.1788 C   0  0  0  0  0  0  0  0  0  0  0  0
   -9.1319   -3.6179   -2.6518 H   0  0  0  0  0  0  0  0  0  0  0  0
   -8.9753   -0.4275    0.2639 H   0  0  0  0  0  0  0  0  0  0  0  0
   -3.8412   -2.4035   -1.0699 N   0  0  0  0  0  0  0  0  0  0  0  0
   -3.7314  -10.9363    1.7171 C   0  0  0  0  0  0  0  0  0  0  0  0
   -4.7476  -11.2447    1.3659 H   0  0  0  0  0  0  0  0  0  0  0  0
   -2.9800  -11.6449    1.2802 H   0  0  0  0  0  0  0  0  0  0  0  0
   -3.7040  -11.0437    2.8285 H   0  0  0  0  0  0  0  0  0  0  0  0
  -10.7078   -1.9557   -1.1922 C   0  0  0  0  0  0  0  0  0  0  0  0
  -11.0430   -1.3389   -2.0665 H   0  0  0  0  0  0  0  0  0  0  0  0
  -11.1617   -2.9729   -1.2899 H   0  0  0  0  0  0  0  0  0  0  0  0
  -11.1112   -1.4874   -0.2618 H   0  0  0  0  0  0  0  0  0  0  0  0
   -1.9041   -2.7161   -0.9726 Ir  0  0  0  0  0  0  0  0  0  0  0  0
  1  2  1  0  0  0  0
  1 11  1  0  0  0  0
  1 17  2  0  0  0  0
  1 77  1  0  0  0  0
  2  4  1  0  0  0  0
  2  7  1  0  0  0  0
  2 12  1  0  0  0  0
  3  5  1  0  0  0  0
  3  9  1  0  0  0  0
  3 13  1  0  0  0  0
  3 19  1  0  0  0  0
  4  6  1  0  0  0  0
  4  8  1  0  0  0  0
  4 14  1  0  0  0  0
  5  6  1  0  0  0  0
  5 10  1  0  0  0  0
  5 15  1  0  0  0  0
  6 16  1  0  0  0  0
  6 44  1  0  0  0  0
```

```
 17 19  1  0  0  0  0
 17 18  1  0  0  0  0
 17 77  1  0  0  0  0
 19 20  1  0  0  0  0
 19 21  1  0  0  0  0
 22 23  1  0  0  0  0
 22 32  1  0  0  0  0
 22 38  2  0  0  0  0
 22 77  1  0  0  0  0
 23 25  1  0  0  0  0
 23 28  1  0  0  0  0
 23 33  1  0  0  0  0
 24 26  1  0  0  0  0
 24 30  1  0  0  0  0
 24 34  1  0  0  0  0
 24 40  1  0  0  0  0
 25 27  1  0  0  0  0
 25 29  1  0  0  0  0
 25 35  1  0  0  0  0
 26 27  1  0  0  0  0
 26 31  1  0  0  0  0
 26 36  1  0  0  0  0
 27 37  1  0  0  0  0
 27 43  1  0  0  0  0
 38 39  1  0  0  0  0
 38 40  1  0  0  0  0
 38 77  1  0  0  0  0
 40 41  1  0  0  0  0
 40 42  1  0  0  0  0
 45 46  1  0  0  0  0
 45 56  3  0  0  0  0
 46 47  2  0  0  0  0
 46 48  1  0  0  0  0
 47 49  1  0  0  0  0
 47 50  1  0  0  0  0
 48 51  2  0  0  0  0
 48 52  1  0  0  0  0
 49 53  2  0  0  0  0
 49 54  1  0  0  0  0
 51 53  1  0  0  0  0
 51 55  1  0  0  0  0
 53 69  1  0  0  0  0
 56 77  1  0  0  0  0
 57 58  1  0  0  0  0
 57 68  3  0  0  0  0
 58 59  2  0  0  0  0
 58 60  1  0  0  0  0
 59 61  1  0  0  0  0
 59 62  1  0  0  0  0
 60 63  2  0  0  0  0
 60 64  1  0  0  0  0
 61 65  2  0  0  0  0
 61 66  1  0  0  0  0
 63 65  1  0  0  0  0
 63 67  1  0  0  0  0
 65 73  1  0  0  0  0
 68 77  1  0  0  0  0
 69 70  1  0  0  0  0
 69 71  1  0  0  0  0
 69 72  1  0  0  0  0
 73 74  1  0  0  0  0
 73 75  1  0  0  0  0
 73 76  1  0  0  0  0
M  END
$$$$
```

```
mol108
Optimized Catalyst 108
Optimized Catalyst 108
 77 82  0  0  0  0  0  0  0  0999 V2000
   -1.5213    0.3202   -0.0993 C   0  0  0  0  0  0  0  0  0  0  0  0
   -1.1081    1.6825    0.3704 C   0  0  0  0  0  0  0  0  0  0  0  0
   -2.0993    1.5725   -3.0537 C   0  0  0  0  0  0  0  0  0  0  0  0
   -1.9091    2.8434   -0.2564 C   0  0  0  0  0  0  0  0  0  0  0  0
   -1.8023    3.0658   -2.8475 C   0  0  0  0  0  0  0  0  0  0  0  0
   -1.2351    3.4836   -1.4809 C   0  0  0  0  0  0  0  0  0  0  0  0
   -0.0151    1.8188    0.1555 H   0  0  0  0  0  0  0  0  0  0  0  0
   -2.9310    2.4911   -0.5256 H   0  0  0  0  0  0  0  0  0  0  0  0
   -3.0518    1.3171   -2.5307 H   0  0  0  0  0  0  0  0  0  0  0  0
   -2.7597    3.6223   -3.0181 H   0  0  0  0  0  0  0  0  0  0  0  0
   -1.9155   -0.3364    0.7010 H   0  0  0  0  0  0  0  0  0  0  0  0
   -1.2370    1.7339    1.4834 H   0  0  0  0  0  0  0  0  0  0  0  0
   -2.2585    1.4085   -4.1499 H   0  0  0  0  0  0  0  0  0  0  0  0
   -2.0196    3.6349    0.5290 H   0  0  0  0  0  0  0  0  0  0  0  0
   -1.0736    3.3968   -3.6310 H   0  0  0  0  0  0  0  0  0  0  0  0
   -1.3603    4.5965   -1.4036 H   0  0  0  0  0  0  0  0  0  0  0  0
   -1.4156   -0.1393   -1.3560 C   0  0  0  0  0  0  0  0  0  0  0  0
   -1.7502   -1.2608   -1.6067 H   0  0  0  0  0  0  0  0  0  0  0  0
   -0.9825    0.6195   -2.5726 C   0  0  0  0  0  0  0  0  0  0  0  0
   -0.0627    1.2144   -2.3248 H   0  0  0  0  0  0  0  0  0  0  0  0
   -0.7157   -0.0758   -3.4052 H   0  0  0  0  0  0  0  0  0  0  0  0
    0.5063   -3.4354   -1.5579 C   0  0  0  0  0  0  0  0  0  0  0  0
    1.1562   -2.8530   -2.7851 C   0  0  0  0  0  0  0  0  0  0  0  0
    2.8092   -2.0287    0.2497 C   0  0  0  0  0  0  0  0  0  0  0  0
    2.7027   -2.9007   -2.7016 C   0  0  0  0  0  0  0  0  0  0  0  0
    3.7689   -1.4613   -0.8068 C   0  0  0  0  0  0  0  0  0  0  0  0
    3.3462   -1.5694   -2.2818 C   0  0  0  0  0  0  0  0  0  0  0  0
    0.8311   -1.7944   -2.9436 H   0  0  0  0  0  0  0  0  0  0  0  0
    3.0173   -3.7102   -2.0015 H   0  0  0  0  0  0  0  0  0  0  0  0
    2.9073   -3.1420    0.2653 H   0  0  0  0  0  0  0  0  0  0  0  0
    4.7472   -1.9949   -0.6815 H   0  0  0  0  0  0  0  0  0  0  0  0
    0.1284   -4.4720   -1.6998 H   0  0  0  0  0  0  0  0  0  0  0  0
    0.8413   -3.4449   -3.6843 H   0  0  0  0  0  0  0  0  0  0  0  0
    3.1407   -1.6487    1.2495 H   0  0  0  0  0  0  0  0  0  0  0  0
    3.0847   -3.1677   -3.7202 H   0  0  0  0  0  0  0  0  0  0  0  0
    3.9442   -0.3779   -0.5861 H   0  0  0  0  0  0  0  0  0  0  0  0
    4.2760   -1.4299   -2.8962 H   0  0  0  0  0  0  0  0  0  0  0  0
    0.5404   -2.8860   -0.3100 C   0  0  0  0  0  0  0  0  0  0  0  0
    0.1984   -3.4748    0.5731 H   0  0  0  0  0  0  0  0  0  0  0  0
    1.3221   -1.6469    0.0521 C   0  0  0  0  0  0  0  0  0  0  0  0
    1.2467   -0.8664   -0.7453 H   0  0  0  0  0  0  0  0  0  0  0  0
    0.9307   -1.2114    1.0049 H   0  0  0  0  0  0  0  0  0  0  0  0
    2.6651   -0.7226   -2.5325 H   0  0  0  0  0  0  0  0  0  0  0  0
   -0.1381    3.2817   -1.4596 H   0  0  0  0  0  0  0  0  0  0  0  0
   -2.4641   -5.6409   -0.0053 C   0  0  0  0  0  0  0  0  0  0  0  0
   -2.7502   -6.9712    0.4242 C   0  0  0  0  0  0  0  0  0  0  0  0
   -2.5696   -7.3177    1.7762 C   0  0  0  0  0  0  0  0  0  0  0  0
   -3.2145   -7.9216   -0.5056 C   0  0  0  0  0  0  0  0  0  0  0  0
   -2.8573   -8.6181    2.1975 C   0  0  0  0  0  0  0  0  0  0  0  0
   -2.2085   -6.5788    2.4955 H   0  0  0  0  0  0  0  0  0  0  0  0
   -3.4996   -9.2172   -0.0738 C   0  0  0  0  0  0  0  0  0  0  0  0
   -3.3518   -7.6518   -1.5552 H   0  0  0  0  0  0  0  0  0  0  0  0
   -3.3199   -9.5646    1.2746 C   0  0  0  0  0  0  0  0  0  0  0  0
   -2.7169   -8.8792    3.2537 H   0  0  0  0  0  0  0  0  0  0  0  0
   -3.8658   -9.9598   -0.7940 H   0  0  0  0  0  0  0  0  0  0  0  0
   -2.2191   -4.5531   -0.3527 N   0  0  0  0  0  0  0  0  0  0  0  0
   -5.0079   -2.2739   -1.0533 C   0  0  0  0  0  0  0  0  0  0  0  0
   -6.4284   -2.1411   -1.1108 C   0  0  0  0  0  0  0  0  0  0  0  0
   -7.1527   -2.8833   -2.0642 C   0  0  0  0  0  0  0  0  0  0  0  0
   -7.0913   -1.2844   -0.2133 C   0  0  0  0  0  0  0  0  0  0  0  0
   -8.5422   -2.7684   -2.1082 C   0  0  0  0  0  0  0  0  0  0  0  0
```

```
   -6.6357   -3.5434   -2.7644 H   0  0  0  0  0  0  0  0  0  0  0  0
   -8.4838   -1.1768   -0.2661 C   0  0  0  0  0  0  0  0  0  0  0  0
   -6.5274   -0.7065    0.5228 H   0  0  0  0  0  0  0  0  0  0  0  0
   -9.2059   -1.9181   -1.2094 C   0  0  0  0  0  0  0  0  0  0  0  0
   -9.1109   -3.3469   -2.8471 H   0  0  0  0  0  0  0  0  0  0  0  0
   -8.9939   -0.5095    0.4393 H   0  0  0  0  0  0  0  0  0  0  0  0
   -3.8470   -2.3878   -1.0098 N   0  0  0  0  0  0  0  0  0  0  0  0
   -3.6524  -10.9778    1.7074 C   0  0  0  0  0  0  0  0  0  0  0  0
  -10.7165   -1.8326   -1.2849 C   0  0  0  0  0  0  0  0  0  0  0  0
   -3.0527  -11.9228    0.9686 F   0  0  0  0  0  0  0  0  0  0  0  0
   -4.9614  -11.2571    1.6186 F   0  0  0  0  0  0  0  0  0  0  0  0
   -3.3301  -11.2948    2.9682 F   0  0  0  0  0  0  0  0  0  0  0  0
  -11.3230   -3.0014   -1.0288 F   0  0  0  0  0  0  0  0  0  0  0  0
  -11.1707   -1.4728   -2.4945 F   0  0  0  0  0  0  0  0  0  0  0  0
  -11.2985   -0.9674   -0.4445 F   0  0  0  0  0  0  0  0  0  0  0  0
   -1.9186   -2.7116   -0.9316 Ir  0  0  0  0  0  0  0  0  0  0  0  0
  1  2  1  0  0  0  0
  1 11  1  0  0  0  0
  1 17  2  0  0  0  0
  1 77  1  0  0  0  0
  2  4  1  0  0  0  0
  2  7  1  0  0  0  0
  2 12  1  0  0  0  0
  3  5  1  0  0  0  0
  3  9  1  0  0  0  0
  3 13  1  0  0  0  0
  3 19  1  0  0  0  0
  4  6  1  0  0  0  0
  4  8  1  0  0  0  0
  4 14  1  0  0  0  0
  5  6  1  0  0  0  0
  5 10  1  0  0  0  0
  5 15  1  0  0  0  0
  6 16  1  0  0  0  0
  6 44  1  0  0  0  0
 17 19  1  0  0  0  0
 17 18  1  0  0  0  0
 17 77  1  0  0  0  0
 19 20  1  0  0  0  0
 19 21  1  0  0  0  0
 22 23  1  0  0  0  0
 22 32  1  0  0  0  0
 22 38  2  0  0  0  0
 22 77  1  0  0  0  0
 23 25  1  0  0  0  0
 23 28  1  0  0  0  0
 23 33  1  0  0  0  0
 24 26  1  0  0  0  0
 24 30  1  0  0  0  0
 24 34  1  0  0  0  0
 24 40  1  0  0  0  0
 25 27  1  0  0  0  0
 25 29  1  0  0  0  0
 25 35  1  0  0  0  0
 26 27  1  0  0  0  0
 26 31  1  0  0  0  0
 26 36  1  0  0  0  0
 27 37  1  0  0  0  0
 27 43  1  0  0  0  0
 38 39  1  0  0  0  0
 38 40  1  0  0  0  0
 38 77  1  0  0  0  0
 40 41  1  0  0  0  0
 40 42  1  0  0  0  0
 45 46  1  0  0  0  0
```

```
 45 56  3  0  0  0  0
 46 47  2  0  0  0  0
 46 48  1  0  0  0  0
 47 49  1  0  0  0  0
 47 50  1  0  0  0  0
 48 51  2  0  0  0  0
 48 52  1  0  0  0  0
 49 53  2  0  0  0  0
 49 54  1  0  0  0  0
 51 53  1  0  0  0  0
 51 55  1  0  0  0  0
 53 69  1  0  0  0  0
 56 77  1  0  0  0  0
 57 58  1  0  0  0  0
 57 68  3  0  0  0  0
 58 59  2  0  0  0  0
 58 60  1  0  0  0  0
 59 61  1  0  0  0  0
 59 62  1  0  0  0  0
 60 63  2  0  0  0  0
 60 64  1  0  0  0  0
 61 65  2  0  0  0  0
 61 66  1  0  0  0  0
 63 65  1  0  0  0  0
 63 67  1  0  0  0  0
 65 70  1  0  0  0  0
 68 77  1  0  0  0  0
 69 71  1  0  0  0  0
 69 72  1  0  0  0  0
 69 73  1  0  0  0  0
 70 74  1  0  0  0  0
 70 75  1  0  0  0  0
 70 76  1  0  0  0  0
M  END
$$$$
mol109
Optimized Catalyst 109
Optimized Catalyst 109
 95 99  0  0  0  0  0  0  0  0999 V2000
   -1.5767   -0.2045   -0.3135 C   0  0  0  0  0  0  0  0  0  0  0  0
   -1.3094    0.9959    0.5471 C   0  0  0  0  0  0  0  0  0  0  0  0
   -2.3577    1.8107   -2.7659 C   0  0  0  0  0  0  0  0  0  0  0  0
   -2.2624    2.1819    0.2813 C   0  0  0  0  0  0  0  0  0  0  0  0
   -2.2383    3.1979   -2.1146 C   0  0  0  0  0  0  0  0  0  0  0  0
   -1.6932    3.2430   -0.6766 C   0  0  0  0  0  0  0  0  0  0  0  0
   -0.2483    1.3182    0.3889 H   0  0  0  0  0  0  0  0  0  0  0  0
   -3.2278    1.7942   -0.1176 H   0  0  0  0  0  0  0  0  0  0  0  0
   -3.2687    1.3013   -2.3693 H   0  0  0  0  0  0  0  0  0  0  0  0
   -3.2573    3.6604   -2.1232 H   0  0  0  0  0  0  0  0  0  0  0  0
   -1.8527   -1.1194    0.2627 H   0  0  0  0  0  0  0  0  0  0  0  0
   -1.4129    0.7010    1.6218 H   0  0  0  0  0  0  0  0  0  0  0  0
   -2.5040    1.9624   -3.8640 H   0  0  0  0  0  0  0  0  0  0  0  0
   -2.4708    2.6768    1.2623 H   0  0  0  0  0  0  0  0  0  0  0  0
   -1.5686    3.8323   -2.7482 H   0  0  0  0  0  0  0  0  0  0  0  0
   -1.9380    4.2554   -0.2634 H   0  0  0  0  0  0  0  0  0  0  0  0
   -1.4841   -0.2576   -1.6513 C   0  0  0  0  0  0  0  0  0  0  0  0
   -1.7012   -1.2056   -2.1905 H   0  0  0  0  0  0  0  0  0  0  0  0
   -1.1374    0.8897   -2.5549 C   0  0  0  0  0  0  0  0  0  0  0  0
   -0.2907    1.4736   -2.1169 H   0  0  0  0  0  0  0  0  0  0  0  0
   -0.7998    0.4974   -3.5445 H   0  0  0  0  0  0  0  0  0  0  0  0
    0.3697   -4.0851   -1.3107 C   0  0  0  0  0  0  0  0  0  0  0  0
    0.6345   -3.5599   -2.6989 C   0  0  0  0  0  0  0  0  0  0  0  0
    2.6221   -2.0260   -0.1809 C   0  0  0  0  0  0  0  0  0  0  0  0
    2.0995   -3.1685   -2.9858 C   0  0  0  0  0  0  0  0  0  0  0  0
    3.1505   -1.3468   -1.4542 C   0  0  0  0  0  0  0  0  0  0  0  0
```

```
    2.4216   -1.6806   -2.7677 C   0  0  0  0  0  0  0  0  0  0  0  0
   -0.0606   -2.6654   -2.8648 H   0  0  0  0  0  0  0  0  0  0  0  0
    2.7722   -3.7982   -2.3556 H   0  0  0  0  0  0  0  0  0  0  0  0
    2.9596   -3.0912   -0.1672 H   0  0  0  0  0  0  0  0  0  0  0  0
    4.2281   -1.6328   -1.5621 H   0  0  0  0  0  0  0  0  0  0  0  0
    0.0942   -5.1650   -1.2562 H   0  0  0  0  0  0  0  0  0  0  0  0
    0.3311   -4.3494   -3.4368 H   0  0  0  0  0  0  0  0  0  0  0  0
    3.0860   -1.5137    0.6992 H   0  0  0  0  0  0  0  0  0  0  0  0
    2.3092   -3.4150   -4.0579 H   0  0  0  0  0  0  0  0  0  0  0  0
    3.1041   -0.2373   -1.3146 H   0  0  0  0  0  0  0  0  0  0  0  0
    3.0866   -1.3427   -3.6054 H   0  0  0  0  0  0  0  0  0  0  0  0
    0.4894   -3.3612   -0.1215 C   0  0  0  0  0  0  0  0  0  0  0  0
    0.2857   -3.8737    0.8474 H   0  0  0  0  0  0  0  0  0  0  0  0
    1.0874   -1.9831   -0.0236 C   0  0  0  0  0  0  0  0  0  0  0  0
    0.6403   -1.3307   -0.8308 H   0  0  0  0  0  0  0  0  0  0  0  0
    0.8222   -1.5328    0.9662 H   0  0  0  0  0  0  0  0  0  0  0  0
    1.4840   -1.0782   -2.8325 H   0  0  0  0  0  0  0  0  0  0  0  0
   -0.5806    3.1639   -0.7064 H   0  0  0  0  0  0  0  0  0  0  0  0
   -2.5794   -6.3766    0.5033 C   0  0  0  0  0  0  0  0  0  0  0  0
   -3.1219   -7.6274    0.9298 C   0  0  0  0  0  0  0  0  0  0  0  0
   -2.5377   -8.3227    2.0051 C   0  0  0  0  0  0  0  0  0  0  0  0
   -4.2347   -8.1744    0.2686 C   0  0  0  0  0  0  0  0  0  0  0  0
   -3.0641   -9.5491    2.3992 C   0  0  0  0  0  0  0  0  0  0  0  0
   -1.6759   -7.9012    2.5245 H   0  0  0  0  0  0  0  0  0  0  0  0
   -4.7498   -9.4050    0.6763 C   0  0  0  0  0  0  0  0  0  0  0  0
   -4.6925   -7.6394   -0.5638 H   0  0  0  0  0  0  0  0  0  0  0  0
   -4.1750  -10.1173    1.7419 C   0  0  0  0  0  0  0  0  0  0  0  0
   -2.5976  -10.0733    3.2359 H   0  0  0  0  0  0  0  0  0  0  0  0
   -5.6143   -9.8028    0.1423 H   0  0  0  0  0  0  0  0  0  0  0  0
   -2.1302   -5.3681    0.1476 N   0  0  0  0  0  0  0  0  0  0  0  0
   -4.1609   -2.7545   -1.0679 C   0  0  0  0  0  0  0  0  0  0  0  0
   -5.5694   -2.5532   -1.1418 C   0  0  0  0  0  0  0  0  0  0  0  0
   -6.4256   -3.6389   -1.3926 C   0  0  0  0  0  0  0  0  0  0  0  0
   -6.1050   -1.2642   -0.9537 C   0  0  0  0  0  0  0  0  0  0  0  0
   -7.8043   -3.4338   -1.4475 C   0  0  0  0  0  0  0  0  0  0  0  0
   -6.0135   -4.6364   -1.5437 H   0  0  0  0  0  0  0  0  0  0  0  0
   -7.4834   -1.0809   -1.0075 C   0  0  0  0  0  0  0  0  0  0  0  0
   -5.4409   -0.4195   -0.7649 H   0  0  0  0  0  0  0  0  0  0  0  0
   -8.3597   -2.1583   -1.2538 C   0  0  0  0  0  0  0  0  0  0  0  0
   -8.4436   -4.2960   -1.6437 H   0  0  0  0  0  0  0  0  0  0  0  0
   -7.8836   -0.0769   -0.8522 H   0  0  0  0  0  0  0  0  0  0  0  0
   -3.0062   -2.8924   -1.0182 N   0  0  0  0  0  0  0  0  0  0  0  0
   -4.7093  -11.4779    2.2132 C   0  0  0  0  0  0  0  0  0  0  0  0
   -9.8737   -1.9026   -1.2877 C   0  0  0  0  0  0  0  0  0  0  0  0
   -1.3888   -3.1982   -0.8636 Rh  0  0  0  0  0  0  0  0  0  0  0  0
   -3.5653  -12.5098    2.1089 C   0  0  0  0  0  0  0  0  0  0  0  0
   -2.7505  -12.2850    2.8291 H   0  0  0  0  0  0  0  0  0  0  0  0
   -3.9540  -13.5281    2.3382 H   0  0  0  0  0  0  0  0  0  0  0  0
   -3.1475  -12.5126    1.0780 H   0  0  0  0  0  0  0  0  0  0  0  0
   -5.8953  -11.9767    1.3627 C   0  0  0  0  0  0  0  0  0  0  0  0
   -6.7559  -11.2768    1.4309 H   0  0  0  0  0  0  0  0  0  0  0  0
   -5.5922  -12.1073    0.3013 H   0  0  0  0  0  0  0  0  0  0  0  0
   -6.2338  -12.9680    1.7457 H   0  0  0  0  0  0  0  0  0  0  0  0
   -5.1821  -11.3254    3.6748 C   0  0  0  0  0  0  0  0  0  0  0  0
   -4.3393  -11.0625    4.3484 H   0  0  0  0  0  0  0  0  0  0  0  0
   -5.9583  -10.5316    3.7419 H   0  0  0  0  0  0  0  0  0  0  0  0
   -5.6208  -12.2857    4.0292 H   0  0  0  0  0  0  0  0  0  0  0  0
  -10.6819   -3.1737   -1.6196 C   0  0  0  0  0  0  0  0  0  0  0  0
  -10.3990   -3.5721   -2.6179 H   0  0  0  0  0  0  0  0  0  0  0  0
  -10.5337   -3.9529   -0.8412 H   0  0  0  0  0  0  0  0  0  0  0  0
  -11.7687   -2.9252   -1.6475 H   0  0  0  0  0  0  0  0  0  0  0  0
  -10.1677   -0.8422   -2.3702 C   0  0  0  0  0  0  0  0  0  0  0  0
   -9.7482   -1.1691   -3.3471 H   0  0  0  0  0  0  0  0  0  0  0  0
  -11.2686   -0.7130   -2.4811 H   0  0  0  0  0  0  0  0  0  0  0  0
   -9.7339    0.1440   -2.1022 H   0  0  0  0  0  0  0  0  0  0  0  0
```

```
  -10.2992   -1.3991    0.1084 C   0  0  0  0  0  0  0  0  0  0  0  0
   -9.9977   -2.1350    0.8859 H   0  0  0  0  0  0  0  0  0  0  0  0
   -9.8360   -0.4176    0.3428 H   0  0  0  0  0  0  0  0  0  0  0  0
  -11.4055   -1.2764    0.1429 H   0  0  0  0  0  0  0  0  0  0  0  0
  1  2  1  0  0  0  0
  1 11  1  0  0  0  0
  1 17  2  0  0  0  0
  1 71  1  0  0  0  0
  2  4  1  0  0  0  0
  2  7  1  0  0  0  0
  2 12  1  0  0  0  0
  3  5  1  0  0  0  0
  3  9  1  0  0  0  0
  3 13  1  0  0  0  0
  3 19  1  0  0  0  0
  4  6  1  0  0  0  0
  4  8  1  0  0  0  0
  4 14  1  0  0  0  0
  5  6  1  0  0  0  0
  5 10  1  0  0  0  0
  5 15  1  0  0  0  0
  6 16  1  0  0  0  0
  6 44  1  0  0  0  0
 17 18  1  0  0  0  0
 17 19  1  0  0  0  0
 17 71  1  0  0  0  0
 19 20  1  0  0  0  0
 19 21  1  0  0  0  0
 22 23  1  0  0  0  0
 22 32  1  0  0  0  0
 22 38  2  0  0  0  0
 22 71  1  0  0  0  0
 23 25  1  0  0  0  0
 23 33  1  0  0  0  0
 24 26  1  0  0  0  0
 24 30  1  0  0  0  0
 24 34  1  0  0  0  0
 24 40  1  0  0  0  0
 25 27  1  0  0  0  0
 25 29  1  0  0  0  0
 25 35  1  0  0  0  0
 26 27  1  0  0  0  0
 26 31  1  0  0  0  0
 26 36  1  0  0  0  0
 27 37  1  0  0  0  0
 27 43  1  0  0  0  0
 38 39  1  0  0  0  0
 38 40  1  0  0  0  0
 38 71  1  0  0  0  0
 40 41  1  0  0  0  0
 40 42  1  0  0  0  0
 45 46  1  0  0  0  0
 45 56  3  0  0  0  0
 46 47  2  0  0  0  0
 46 48  1  0  0  0  0
 47 49  1  0  0  0  0
 47 50  1  0  0  0  0
 48 51  2  0  0  0  0
 48 52  1  0  0  0  0
 49 53  2  0  0  0  0
 49 54  1  0  0  0  0
 51 53  1  0  0  0  0
 51 55  1  0  0  0  0
 53 69  1  0  0  0  0
 56 71  1  0  0  0  0
```

```
 57 58  1  0  0  0  0
 57 68  3  0  0  0  0
 58 59  2  0  0  0  0
 58 60  1  0  0  0  0
 59 61  1  0  0  0  0
 59 62  1  0  0  0  0
 60 63  2  0  0  0  0
 60 64  1  0  0  0  0
 61 65  2  0  0  0  0
 61 66  1  0  0  0  0
 63 65  1  0  0  0  0
 63 67  1  0  0  0  0
 65 70  1  0  0  0  0
 68 71  1  0  0  0  0
 69 72  1  0  0  0  0
 69 76  1  0  0  0  0
 69 80  1  0  0  0  0
 70 84  1  0  0  0  0
 70 88  1  0  0  0  0
 70 92  1  0  0  0  0
 72 73  1  0  0  0  0
 72 74  1  0  0  0  0
 72 75  1  0  0  0  0
 76 77  1  0  0  0  0
 76 78  1  0  0  0  0
 76 79  1  0  0  0  0
 80 81  1  0  0  0  0
 80 82  1  0  0  0  0
 80 83  1  0  0  0  0
 84 85  1  0  0  0  0
 84 86  1  0  0  0  0
 84 87  1  0  0  0  0
 88 89  1  0  0  0  0
 88 90  1  0  0  0  0
 88 91  1  0  0  0  0
 92 93  1  0  0  0  0
 92 94  1  0  0  0  0
 92 95  1  0  0  0  0
M  END
$$$$
mol110
Optimized Catalyst 110
Optimized Catalyst 110
 95100  0  0  0  0  0  0  0  0999 V2000
   -1.4817    0.3152   -0.1346 C   0  0  0  0  0  0  0  0  0  0  0  0
   -1.0873    1.6747    0.3586 C   0  0  0  0  0  0  0  0  0  0  0  0
   -2.0249    1.5976   -3.0841 C   0  0  0  0  0  0  0  0  0  0  0  0
   -1.8900    2.8355   -0.2662 C   0  0  0  0  0  0  0  0  0  0  0  0
   -1.7534    3.0921   -2.8519 C   0  0  0  0  0  0  0  0  0  0  0  0
   -1.2046    3.4956   -1.4736 C   0  0  0  0  0  0  0  0  0  0  0  0
    0.0075    1.8214    0.1634 H   0  0  0  0  0  0  0  0  0  0  0  0
   -2.9035    2.4748   -0.5556 H   0  0  0  0  0  0  0  0  0  0  0  0
   -2.9842    1.3212   -2.5843 H   0  0  0  0  0  0  0  0  0  0  0  0
   -2.7173    3.6367   -3.0226 H   0  0  0  0  0  0  0  0  0  0  0  0
   -1.8839   -0.3539    0.6525 H   0  0  0  0  0  0  0  0  0  0  0  0
   -1.2344    1.7102    1.4698 H   0  0  0  0  0  0  0  0  0  0  0  0
   -2.1593    1.4461   -4.1852 H   0  0  0  0  0  0  0  0  0  0  0  0
   -2.0208    3.6159    0.5267 H   0  0  0  0  0  0  0  0  0  0  0  0
   -1.0220    3.4458   -3.6224 H   0  0  0  0  0  0  0  0  0  0  0  0
   -1.3359    4.6060   -1.3820 H   0  0  0  0  0  0  0  0  0  0  0  0
   -1.3550   -0.1282   -1.3952 C   0  0  0  0  0  0  0  0  0  0  0  0
   -1.6824   -1.2448   -1.6679 H   0  0  0  0  0  0  0  0  0  0  0  0
   -0.9064    0.6519   -2.5930 C   0  0  0  0  0  0  0  0  0  0  0  0
    0.0019    1.2514   -2.3187 H   0  0  0  0  0  0  0  0  0  0  0  0
   -0.6159   -0.0309   -3.4280 H   0  0  0  0  0  0  0  0  0  0  0  0
```

```
    0.5748   -3.4134   -1.5899 C   0  0  0  0  0  0  0  0  0  0  0  0
    1.2285   -2.8319   -2.8163 C   0  0  0  0  0  0  0  0  0  0  0  0
    2.8849   -2.0174    0.2227 C   0  0  0  0  0  0  0  0  0  0  0  0
    2.7745   -2.8721   -2.7312 C   0  0  0  0  0  0  0  0  0  0  0  0
    3.8441   -1.4438   -0.8312 C   0  0  0  0  0  0  0  0  0  0  0  0
    3.4116   -1.5396   -2.3042 C   0  0  0  0  0  0  0  0  0  0  0  0
    0.8972   -1.7758   -2.9783 H   0  0  0  0  0  0  0  0  0  0  0  0
    3.0912   -3.6833   -2.0344 H   0  0  0  0  0  0  0  0  0  0  0  0
    2.9823   -3.1309    0.2305 H   0  0  0  0  0  0  0  0  0  0  0  0
    4.8209   -1.9816   -0.7156 H   0  0  0  0  0  0  0  0  0  0  0  0
    0.1833   -4.4452   -1.7348 H   0  0  0  0  0  0  0  0  0  0  0  0
    0.9177   -3.4279   -3.7142 H   0  0  0  0  0  0  0  0  0  0  0  0
    3.2171   -1.6459    1.2248 H   0  0  0  0  0  0  0  0  0  0  0  0
    3.1592   -3.1321   -3.7502 H   0  0  0  0  0  0  0  0  0  0  0  0
    4.0228   -0.3627   -0.6020 H   0  0  0  0  0  0  0  0  0  0  0  0
    4.3340   -1.3858   -2.9249 H   0  0  0  0  0  0  0  0  0  0  0  0
    0.6152   -2.8706   -0.3398 C   0  0  0  0  0  0  0  0  0  0  0  0
    0.2619   -3.4585    0.5396 H   0  0  0  0  0  0  0  0  0  0  0  0
    1.3980   -1.6343    0.0280 C   0  0  0  0  0  0  0  0  0  0  0  0
    1.3200   -0.8502   -0.7655 H   0  0  0  0  0  0  0  0  0  0  0  0
    1.0068   -1.2027    0.9830 H   0  0  0  0  0  0  0  0  0  0  0  0
    2.7204   -0.6965   -2.5387 H   0  0  0  0  0  0  0  0  0  0  0  0
   -0.1074    3.2962   -1.4420 H   0  0  0  0  0  0  0  0  0  0  0  0
   -2.5052   -5.5966   -0.0079 C   0  0  0  0  0  0  0  0  0  0  0  0
   -2.8917   -6.8868    0.4354 C   0  0  0  0  0  0  0  0  0  0  0  0
   -2.8077   -7.2207    1.8023 C   0  0  0  0  0  0  0  0  0  0  0  0
   -3.3725   -7.8404   -0.4881 C   0  0  0  0  0  0  0  0  0  0  0  0
   -3.2137   -8.4807    2.2335 C   0  0  0  0  0  0  0  0  0  0  0  0
   -2.4263   -6.4930    2.5211 H   0  0  0  0  0  0  0  0  0  0  0  0
   -3.7730   -9.0920   -0.0373 C   0  0  0  0  0  0  0  0  0  0  0  0
   -3.4284   -7.5937   -1.5496 H   0  0  0  0  0  0  0  0  0  0  0  0
   -3.7121   -9.4372    1.3306 C   0  0  0  0  0  0  0  0  0  0  0  0
   -3.1330   -8.7100    3.2993 H   0  0  0  0  0  0  0  0  0  0  0  0
   -4.1398   -9.8189   -0.7667 H   0  0  0  0  0  0  0  0  0  0  0  0
   -2.1769   -4.5352   -0.3710 N   0  0  0  0  0  0  0  0  0  0  0  0
   -4.9492   -2.2529   -1.0398 C   0  0  0  0  0  0  0  0  0  0  0  0
   -6.3617   -2.1219   -1.0577 C   0  0  0  0  0  0  0  0  0  0  0  0
   -7.1488   -3.1090   -1.6903 C   0  0  0  0  0  0  0  0  0  0  0  0
   -6.9864   -1.0209   -0.4401 C   0  0  0  0  0  0  0  0  0  0  0  0
   -8.5322   -2.9896   -1.6870 C   0  0  0  0  0  0  0  0  0  0  0  0
   -6.6703   -3.9600   -2.1773 H   0  0  0  0  0  0  0  0  0  0  0  0
   -8.3757   -0.9185   -0.4502 C   0  0  0  0  0  0  0  0  0  0  0  0
   -6.3840   -0.2518    0.0465 H   0  0  0  0  0  0  0  0  0  0  0  0
   -9.1762   -1.8964   -1.0664 C   0  0  0  0  0  0  0  0  0  0  0  0
   -9.1266   -3.7641   -2.1783 H   0  0  0  0  0  0  0  0  0  0  0  0
   -8.8313   -0.0531    0.0378 H   0  0  0  0  0  0  0  0  0  0  0  0
   -3.7862   -2.3626   -1.0269 N   0  0  0  0  0  0  0  0  0  0  0  0
   -4.1668  -10.8349    1.7771 C   0  0  0  0  0  0  0  0  0  0  0  0
  -10.7105   -1.8253   -1.0797 C   0  0  0  0  0  0  0  0  0  0  0  0
   -3.1620  -11.8536    1.2001 C   0  0  0  0  0  0  0  0  0  0  0  0
   -2.1353  -11.6318    1.5668 H   0  0  0  0  0  0  0  0  0  0  0  0
   -3.4423  -12.8806    1.5297 H   0  0  0  0  0  0  0  0  0  0  0  0
   -3.1583  -11.8350    0.0896 H   0  0  0  0  0  0  0  0  0  0  0  0
   -5.5880  -11.0942    1.2328 C   0  0  0  0  0  0  0  0  0  0  0  0
   -6.2622  -10.2522    1.5025 H   0  0  0  0  0  0  0  0  0  0  0  0
   -5.5873  -11.2244    0.1302 H   0  0  0  0  0  0  0  0  0  0  0  0
   -5.9960  -12.0302    1.6809 H   0  0  0  0  0  0  0  0  0  0  0  0
   -4.2019  -10.9788    3.3115 C   0  0  0  0  0  0  0  0  0  0  0  0
   -3.1858  -10.8695    3.7489 H   0  0  0  0  0  0  0  0  0  0  0  0
   -4.8939  -10.2343    3.7618 H   0  0  0  0  0  0  0  0  0  0  0  0
   -4.5719  -11.9964    3.5813 H   0  0  0  0  0  0  0  0  0  0  0  0
  -11.2453   -3.0534   -0.3124 C   0  0  0  0  0  0  0  0  0  0  0  0
  -10.9987   -4.0001   -0.8374 H   0  0  0  0  0  0  0  0  0  0  0  0
  -10.8127   -3.0835    0.7119 H   0  0  0  0  0  0  0  0  0  0  0  0
  -12.3545   -2.9871   -0.2267 H   0  0  0  0  0  0  0  0  0  0  0  0
```

```
  -11.1836   -1.8410   -2.5484 C   0  0  0  0  0  0  0  0  0  0  0  0
  -10.9520   -2.8073   -3.0436 H   0  0  0  0  0  0  0  0  0  0  0  0
  -12.2878   -1.6943   -2.5875 H   0  0  0  0  0  0  0  0  0  0  0  0
  -10.6972   -1.0170   -3.1159 H   0  0  0  0  0  0  0  0  0  0  0  0
  -11.2477   -0.5480   -0.4050 C   0  0  0  0  0  0  0  0  0  0  0  0
  -10.9615   -0.5140    0.6687 H   0  0  0  0  0  0  0  0  0  0  0  0
  -10.8805    0.3618   -0.9279 H   0  0  0  0  0  0  0  0  0  0  0  0
  -12.3624   -0.5411   -0.4572 H   0  0  0  0  0  0  0  0  0  0  0  0
   -1.8508   -2.6951   -0.9695 Ir  0  0  0  0  0  0  0  0  0  0  0  0
  1  2  1  0  0  0  0
  1 11  1  0  0  0  0
  1 17  2  0  0  0  0
  1 95  1  0  0  0  0
  2  4  1  0  0  0  0
  2  7  1  0  0  0  0
  2 12  1  0  0  0  0
  3  5  1  0  0  0  0
  3  9  1  0  0  0  0
  3 13  1  0  0  0  0
  3 19  1  0  0  0  0
  4  6  1  0  0  0  0
  4  8  1  0  0  0  0
  4 14  1  0  0  0  0
  5  6  1  0  0  0  0
  5 10  1  0  0  0  0
  5 15  1  0  0  0  0
  6 16  1  0  0  0  0
  6 44  1  0  0  0  0
 17 19  1  0  0  0  0
 17 18  1  0  0  0  0
 17 95  1  0  0  0  0
 19 20  1  0  0  0  0
 19 21  1  0  0  0  0
 22 23  1  0  0  0  0
 22 32  1  0  0  0  0
 22 38  2  0  0  0  0
 22 95  1  0  0  0  0
 23 25  1  0  0  0  0
 23 28  1  0  0  0  0
 23 33  1  0  0  0  0
 24 26  1  0  0  0  0
 24 30  1  0  0  0  0
 24 34  1  0  0  0  0
 24 40  1  0  0  0  0
 25 27  1  0  0  0  0
 25 29  1  0  0  0  0
 25 35  1  0  0  0  0
 26 27  1  0  0  0  0
 26 31  1  0  0  0  0
 26 36  1  0  0  0  0
 27 37  1  0  0  0  0
 27 43  1  0  0  0  0
 38 39  1  0  0  0  0
 38 40  1  0  0  0  0
 38 95  1  0  0  0  0
 40 41  1  0  0  0  0
 40 42  1  0  0  0  0
 45 46  1  0  0  0  0
 45 56  3  0  0  0  0
 46 47  2  0  0  0  0
 46 48  1  0  0  0  0
 47 49  1  0  0  0  0
 47 50  1  0  0  0  0
 48 51  2  0  0  0  0
 48 52  1  0  0  0  0
```

```
 49 53  2  0  0  0  0
 49 54  1  0  0  0  0
 51 53  1  0  0  0  0
 51 55  1  0  0  0  0
 53 69  1  0  0  0  0
 56 95  1  0  0  0  0
 57 58  1  0  0  0  0
 57 68  3  0  0  0  0
 58 59  2  0  0  0  0
 58 60  1  0  0  0  0
 59 61  1  0  0  0  0
 59 62  1  0  0  0  0
 60 63  2  0  0  0  0
 60 64  1  0  0  0  0
 61 65  2  0  0  0  0
 61 66  1  0  0  0  0
 63 65  1  0  0  0  0
 63 67  1  0  0  0  0
 65 70  1  0  0  0  0
 68 95  1  0  0  0  0
 69 71  1  0  0  0  0
 69 75  1  0  0  0  0
 69 79  1  0  0  0  0
 70 83  1  0  0  0  0
 70 87  1  0  0  0  0
 70 91  1  0  0  0  0
 71 72  1  0  0  0  0
 71 73  1  0  0  0  0
 71 74  1  0  0  0  0
 75 76  1  0  0  0  0
 75 77  1  0  0  0  0
 75 78  1  0  0  0  0
 79 80  1  0  0  0  0
 79 81  1  0  0  0  0
 79 82  1  0  0  0  0
 83 84  1  0  0  0  0
 83 85  1  0  0  0  0
 83 86  1  0  0  0  0
 87 88  1  0  0  0  0
 87 89  1  0  0  0  0
 87 90  1  0  0  0  0
 91 92  1  0  0  0  0
 91 93  1  0  0  0  0
 91 94  1  0  0  0  0
M  END
$$$$
mol111
Optimized Catalyst 111
Optimized Catalyst 111
 57 60  0  0  0  0  0  0  0  0999 V2000
   -1.6603   -0.2662   -0.5682 C   0  0  0  0  0  0  0  0  0  0  0  0
   -1.2188    0.9360    0.2196 C   0  0  0  0  0  0  0  0  0  0  0  0
   -1.8873    1.6488   -3.2036 C   0  0  0  0  0  0  0  0  0  0  0  0
   -1.9460    2.2381   -0.1870 C   0  0  0  0  0  0  0  0  0  0  0  0
   -1.5755    3.0434   -2.6380 C   0  0  0  0  0  0  0  0  0  0  0  0
   -1.1496    3.1227   -1.1616 C   0  0  0  0  0  0  0  0  0  0  0  0
   -0.1148    1.0737    0.1005 H   0  0  0  0  0  0  0  0  0  0  0  0
   -2.9367    1.9865   -0.6311 H   0  0  0  0  0  0  0  0  0  0  0  0
   -2.9019    1.3363   -2.8566 H   0  0  0  0  0  0  0  0  0  0  0  0
   -2.4934    3.6701   -2.7749 H   0  0  0  0  0  0  0  0  0  0  0  0
   -2.1979   -1.0371    0.0369 H   0  0  0  0  0  0  0  0  0  0  0  0
   -1.4187    0.7492    1.3049 H   0  0  0  0  0  0  0  0  0  0  0  0
   -1.9222    1.7354   -4.3182 H   0  0  0  0  0  0  0  0  0  0  0  0
   -2.1293    2.8284    0.7457 H   0  0  0  0  0  0  0  0  0  0  0  0
   -0.7563    3.4999   -3.2487 H   0  0  0  0  0  0  0  0  0  0  0  0
```

```
   -1.2750    4.1902   -0.8426 H   0  0  0  0  0  0  0  0  0  0  0  0
   -1.4955   -0.4466   -1.8928 C   0  0  0  0  0  0  0  0  0  0  0  0
   -1.9274   -1.3441   -2.4027 H   0  0  0  0  0  0  0  0  0  0  0  0
   -0.8678    0.5507   -2.8281 C   0  0  0  0  0  0  0  0  0  0  0  0
    0.0277    1.0176   -2.3509 H   0  0  0  0  0  0  0  0  0  0  0  0
   -0.5310    0.0340   -3.7591 H   0  0  0  0  0  0  0  0  0  0  0  0
    0.7633   -3.6325   -1.4320 C   0  0  0  0  0  0  0  0  0  0  0  0
    1.1574   -2.8598   -2.6666 C   0  0  0  0  0  0  0  0  0  0  0  0
    2.8269   -1.7049    0.2595 C   0  0  0  0  0  0  0  0  0  0  0  0
    2.6651   -2.5173   -2.7087 C   0  0  0  0  0  0  0  0  0  0  0  0
    3.5134   -0.8930   -0.8500 C   0  0  0  0  0  0  0  0  0  0  0  0
    2.9965   -1.0757   -2.2880 C   0  0  0  0  0  0  0  0  0  0  0  0
    0.5379   -1.9171   -2.7164 H   0  0  0  0  0  0  0  0  0  0  0  0
    3.2264   -3.2354   -2.0651 H   0  0  0  0  0  0  0  0  0  0  0  0
    3.2047   -2.7564    0.2217 H   0  0  0  0  0  0  0  0  0  0  0  0
    4.6007   -1.1647   -0.8312 H   0  0  0  0  0  0  0  0  0  0  0  0
    0.6633   -4.7238   -1.5797 H   0  0  0  0  0  0  0  0  0  0  0  0
    0.9037   -3.4742   -3.5697 H   0  0  0  0  0  0  0  0  0  0  0  0
    3.1391   -1.2711    1.2428 H   0  0  0  0  0  0  0  0  0  0  0  0
    3.0145   -2.6612   -3.7622 H   0  0  0  0  0  0  0  0  0  0  0  0
    3.4301    0.1946   -0.5988 H   0  0  0  0  0  0  0  0  0  0  0  0
    3.8000   -0.6933   -2.9722 H   0  0  0  0  0  0  0  0  0  0  0  0
    0.7904   -3.1074   -0.1251 C   0  0  0  0  0  0  0  0  0  0  0  0
    0.7027   -3.7869    0.7431 H   0  0  0  0  0  0  0  0  0  0  0  0
    1.2836   -1.7196    0.1996 C   0  0  0  0  0  0  0  0  0  0  0  0
    0.9205   -0.9986   -0.5830 H   0  0  0  0  0  0  0  0  0  0  0  0
    0.8603   -1.4004    1.1857 H   0  0  0  0  0  0  0  0  0  0  0  0
    2.1036   -0.4259   -2.4418 H   0  0  0  0  0  0  0  0  0  0  0  0
   -0.0623    2.8854   -1.0830 H   0  0  0  0  0  0  0  0  0  0  0  0
   -1.9913   -5.4437    0.0192 C   0  0  0  0  0  0  0  0  0  0  0  0
   -1.5497   -4.4281   -0.3347 N   0  0  0  0  0  0  0  0  0  0  0  0
   -4.8911   -2.5991   -1.3174 C   0  0  0  0  0  0  0  0  0  0  0  0
   -3.7514   -2.7820   -1.2110 N   0  0  0  0  0  0  0  0  0  0  0  0
   -2.5039   -6.7110    0.4687 C   0  0  0  0  0  0  0  0  0  0  0  0
   -2.1008   -6.9555    1.4899 H   0  0  0  0  0  0  0  0  0  0  0  0
   -3.6268   -6.6786    0.5233 H   0  0  0  0  0  0  0  0  0  0  0  0
   -2.2022   -7.5269   -0.2451 H   0  0  0  0  0  0  0  0  0  0  0  0
   -6.3066   -2.3673   -1.4437 C   0  0  0  0  0  0  0  0  0  0  0  0
   -6.6134   -1.4726   -0.8340 H   0  0  0  0  0  0  0  0  0  0  0  0
   -6.5822   -2.1754   -2.5177 H   0  0  0  0  0  0  0  0  0  0  0  0
   -6.8874   -3.2597   -1.0823 H   0  0  0  0  0  0  0  0  0  0  0  0
   -1.1228   -2.9146   -0.9103 Rh  0  0  0  0  0  0  0  0  0  0  0  0
  1  2  1  0  0  0  0
  1 11  1  0  0  0  0
  1 17  2  0  0  0  0
  1 57  1  0  0  0  0
  2  4  1  0  0  0  0
  2  7  1  0  0  0  0
  2 12  1  0  0  0  0
  3  5  1  0  0  0  0
  3  9  1  0  0  0  0
  3 13  1  0  0  0  0
  3 19  1  0  0  0  0
  4  6  1  0  0  0  0
  4  8  1  0  0  0  0
  4 14  1  0  0  0  0
  5  6  1  0  0  0  0
  5 10  1  0  0  0  0
  5 15  1  0  0  0  0
  6 16  1  0  0  0  0
  6 44  1  0  0  0  0
 17 18  1  0  0  0  0
 17 19  1  0  0  0  0
 17 57  1  0  0  0  0
 19 20  1  0  0  0  0
```

```
 19 21  1  0  0  0  0
 22 23  1  0  0  0  0
 22 32  1  0  0  0  0
 22 38  2  0  0  0  0
 22 57  1  0  0  0  0
 23 25  1  0  0  0  0
 23 28  1  0  0  0  0
 23 33  1  0  0  0  0
 24 26  1  0  0  0  0
 24 30  1  0  0  0  0
 24 34  1  0  0  0  0
 24 40  1  0  0  0  0
 25 27  1  0  0  0  0
 25 29  1  0  0  0  0
 25 35  1  0  0  0  0
 26 27  1  0  0  0  0
 26 31  1  0  0  0  0
 26 36  1  0  0  0  0
 27 37  1  0  0  0  0
 27 43  1  0  0  0  0
 38 39  1  0  0  0  0
 38 40  1  0  0  0  0
 38 57  1  0  0  0  0
 40 41  1  0  0  0  0
 40 42  1  0  0  0  0
 45 46  3  0  0  0  0
 45 49  1  0  0  0  0
 46 57  1  0  0  0  0
 47 48  3  0  0  0  0
 47 53  1  0  0  0  0
 48 57  1  0  0  0  0
 49 50  1  0  0  0  0
 49 51  1  0  0  0  0
 49 52  1  0  0  0  0
 53 54  1  0  0  0  0
 53 55  1  0  0  0  0
 53 56  1  0  0  0  0
M  END
$$$$
mol112
Optimized Catalyst 112
Optimized Catalyst 112
 57 60  0  0  0  0  0  0  0  0999 V2000
   -1.5998    0.1846   -0.1163 C   0  0  0  0  0  0  0  0  0  0  0  0
   -1.2426    1.5491    0.3925 C   0  0  0  0  0  0  0  0  0  0  0  0
   -2.0972    1.4849   -3.0586 C   0  0  0  0  0  0  0  0  0  0  0  0
   -2.0426    2.7046   -0.2457 C   0  0  0  0  0  0  0  0  0  0  0  0
   -1.8328    2.9801   -2.8235 C   0  0  0  0  0  0  0  0  0  0  0  0
   -1.3317    3.3840   -1.4275 C   0  0  0  0  0  0  0  0  0  0  0  0
   -0.1450    1.7142    0.2268 H   0  0  0  0  0  0  0  0  0  0  0  0
   -3.0441    2.3356   -0.5644 H   0  0  0  0  0  0  0  0  0  0  0  0
   -3.0579    1.2052   -2.5641 H   0  0  0  0  0  0  0  0  0  0  0  0
   -2.7906    3.5244   -3.0272 H   0  0  0  0  0  0  0  0  0  0  0  0
   -2.0077   -0.4952    0.6571 H   0  0  0  0  0  0  0  0  0  0  0  0
   -1.4198    1.5772    1.4998 H   0  0  0  0  0  0  0  0  0  0  0  0
   -2.2241    1.3342   -4.1605 H   0  0  0  0  0  0  0  0  0  0  0  0
   -2.2050    3.4776    0.5494 H   0  0  0  0  0  0  0  0  0  0  0  0
   -1.0764    3.3332   -3.5699 H   0  0  0  0  0  0  0  0  0  0  0  0
   -1.4824    4.4922   -1.3330 H   0  0  0  0  0  0  0  0  0  0  0  0
   -1.4301   -0.2503   -1.3747 C   0  0  0  0  0  0  0  0  0  0  0  0
   -1.7151   -1.3785   -1.6545 H   0  0  0  0  0  0  0  0  0  0  0  0
   -0.9763    0.5451   -2.5604 C   0  0  0  0  0  0  0  0  0  0  0  0
   -0.0794    1.1545   -2.2683 H   0  0  0  0  0  0  0  0  0  0  0  0
   -0.6674   -0.1250   -3.3989 H   0  0  0  0  0  0  0  0  0  0  0  0
    0.6815   -3.4149   -1.6241 C   0  0  0  0  0  0  0  0  0  0  0  0
```

```
    1.3310   -2.7653   -2.8169 C   0  0  0  0  0  0  0  0  0  0  0  0
    2.8374   -1.9041    0.2807 C   0  0  0  0  0  0  0  0  0  0  0  0
    2.8743   -2.7167   -2.6854 C   0  0  0  0  0  0  0  0  0  0  0  0
    3.7859   -1.2471   -0.7330 C   0  0  0  0  0  0  0  0  0  0  0  0
    3.4185   -1.3554   -2.2228 C   0  0  0  0  0  0  0  0  0  0  0  0
    0.9451   -1.7256   -2.9632 H   0  0  0  0  0  0  0  0  0  0  0  0
    3.2189   -3.5178   -1.9900 H   0  0  0  0  0  0  0  0  0  0  0  0
    3.0099   -3.0083    0.2741 H   0  0  0  0  0  0  0  0  0  0  0  0
    4.7957   -1.7114   -0.5851 H   0  0  0  0  0  0  0  0  0  0  0  0
    0.3659   -4.4664   -1.8011 H   0  0  0  0  0  0  0  0  0  0  0  0
    1.0814   -3.3541   -3.7384 H   0  0  0  0  0  0  0  0  0  0  0  0
    3.1142   -1.5280    1.2985 H   0  0  0  0  0  0  0  0  0  0  0  0
    3.3030   -2.9383   -3.6962 H   0  0  0  0  0  0  0  0  0  0  0  0
    3.8749   -0.1586   -0.4869 H   0  0  0  0  0  0  0  0  0  0  0  0
    4.3567   -1.1459   -2.8035 H   0  0  0  0  0  0  0  0  0  0  0  0
    0.6435   -2.8938   -0.3644 C   0  0  0  0  0  0  0  0  0  0  0  0
    0.3149   -3.5226    0.4962 H   0  0  0  0  0  0  0  0  0  0  0  0
    1.3349   -1.6163    0.0461 C   0  0  0  0  0  0  0  0  0  0  0  0
    1.2325   -0.8257   -0.7384 H   0  0  0  0  0  0  0  0  0  0  0  0
    0.8889   -1.2267    0.9950 H   0  0  0  0  0  0  0  0  0  0  0  0
    2.6940   -0.5483   -2.4822 H   0  0  0  0  0  0  0  0  0  0  0  0
   -0.2329    3.2023   -1.3640 H   0  0  0  0  0  0  0  0  0  0  0  0
   -2.1901   -5.8396   -0.1611 C   0  0  0  0  0  0  0  0  0  0  0  0
   -2.0144   -4.7320   -0.4839 N   0  0  0  0  0  0  0  0  0  0  0  0
   -4.9129   -2.5324   -1.2676 C   0  0  0  0  0  0  0  0  0  0  0  0
   -3.7554   -2.6323   -1.1655 N   0  0  0  0  0  0  0  0  0  0  0  0
   -2.3881   -7.2029    0.2337 C   0  0  0  0  0  0  0  0  0  0  0  0
   -1.8425   -7.4264    1.1969 H   0  0  0  0  0  0  0  0  0  0  0  0
   -3.4838   -7.4198    0.3951 H   0  0  0  0  0  0  0  0  0  0  0  0
   -2.0051   -7.9100   -0.5592 H   0  0  0  0  0  0  0  0  0  0  0  0
   -6.3336   -2.3973   -1.3991 C   0  0  0  0  0  0  0  0  0  0  0  0
   -6.7108   -1.5180   -0.7999 H   0  0  0  0  0  0  0  0  0  0  0  0
   -6.6200   -2.2352   -2.4795 H   0  0  0  0  0  0  0  0  0  0  0  0
   -6.8611   -3.3248   -1.0317 H   0  0  0  0  0  0  0  0  0  0  0  0
   -1.8119   -2.8593   -1.0244 Ir  0  0  0  0  0  0  0  0  0  0  0  0
  1  2  1  0  0  0  0
  1 11  1  0  0  0  0
  1 17  2  0  0  0  0
  1 57  1  0  0  0  0
  2  4  1  0  0  0  0
  2  7  1  0  0  0  0
  2 12  1  0  0  0  0
  3  5  1  0  0  0  0
  3  9  1  0  0  0  0
  3 13  1  0  0  0  0
  3 19  1  0  0  0  0
  4  6  1  0  0  0  0
  4  8  1  0  0  0  0
  4 14  1  0  0  0  0
  5  6  1  0  0  0  0
  5 10  1  0  0  0  0
  5 15  1  0  0  0  0
  6 16  1  0  0  0  0
  6 44  1  0  0  0  0
 17 19  1  0  0  0  0
 17 18  1  0  0  0  0
 17 57  1  0  0  0  0
 19 20  1  0  0  0  0
 19 21  1  0  0  0  0
 22 23  1  0  0  0  0
 22 32  1  0  0  0  0
 22 38  2  0  0  0  0
 22 57  1  0  0  0  0
 23 25  1  0  0  0  0
 23 28  1  0  0  0  0
```

```
 23 33  1  0  0  0  0
 24 26  1  0  0  0  0
 24 30  1  0  0  0  0
 24 34  1  0  0  0  0
 24 40  1  0  0  0  0
 25 27  1  0  0  0  0
 25 29  1  0  0  0  0
 25 35  1  0  0  0  0
 26 27  1  0  0  0  0
 26 31  1  0  0  0  0
 26 36  1  0  0  0  0
 27 37  1  0  0  0  0
 27 43  1  0  0  0  0
 38 39  1  0  0  0  0
 38 40  1  0  0  0  0
 38 57  1  0  0  0  0
 40 41  1  0  0  0  0
 40 42  1  0  0  0  0
 45 46  3  0  0  0  0
 45 49  1  0  0  0  0
 46 57  1  0  0  0  0
 47 48  3  0  0  0  0
 47 53  1  0  0  0  0
 48 57  1  0  0  0  0
 49 50  1  0  0  0  0
 49 51  1  0  0  0  0
 49 52  1  0  0  0  0
 53 54  1  0  0  0  0
 53 55  1  0  0  0  0
 53 56  1  0  0  0  0
M  END
$$$$
mol113
Optimized Catalyst 113
Optimized Catalyst 113
 57 60  0  0  0  0  0  0  0  0999 V2000
   -1.6227   -0.2701   -0.6488 C   0  0  0  0  0  0  0  0  0  0  0  0
   -1.2155    0.9224    0.1706 C   0  0  0  0  0  0  0  0  0  0  0  0
   -1.8572    1.6992   -3.2460 C   0  0  0  0  0  0  0  0  0  0  0  0
   -1.9652    2.2184   -0.2146 C   0  0  0  0  0  0  0  0  0  0  0  0
   -1.5830    3.0859   -2.6431 C   0  0  0  0  0  0  0  0  0  0  0  0
   -1.1782    3.1417   -1.1600 C   0  0  0  0  0  0  0  0  0  0  0  0
   -0.1131    1.0854    0.0672 H   0  0  0  0  0  0  0  0  0  0  0  0
   -2.9471    1.9582   -0.6731 H   0  0  0  0  0  0  0  0  0  0  0  0
   -2.8719    1.3601   -2.9255 H   0  0  0  0  0  0  0  0  0  0  0  0
   -2.5129    3.6957   -2.7782 H   0  0  0  0  0  0  0  0  0  0  0  0
   -2.1470   -1.0647   -0.0662 H   0  0  0  0  0  0  0  0  0  0  0  0
   -1.4237    0.7087    1.2496 H   0  0  0  0  0  0  0  0  0  0  0  0
   -1.8746    1.8115   -4.3592 H   0  0  0  0  0  0  0  0  0  0  0  0
   -2.1697    2.7833    0.7298 H   0  0  0  0  0  0  0  0  0  0  0  0
   -0.7666    3.5755   -3.2322 H   0  0  0  0  0  0  0  0  0  0  0  0
   -1.3346    4.1992   -0.8197 H   0  0  0  0  0  0  0  0  0  0  0  0
   -1.4412   -0.4173   -1.9746 C   0  0  0  0  0  0  0  0  0  0  0  0
   -1.8449   -1.3111   -2.5060 H   0  0  0  0  0  0  0  0  0  0  0  0
   -0.8223    0.6127   -2.8788 C   0  0  0  0  0  0  0  0  0  0  0  0
    0.0557    1.0880   -2.3773 H   0  0  0  0  0  0  0  0  0  0  0  0
   -0.4594    0.1234   -3.8147 H   0  0  0  0  0  0  0  0  0  0  0  0
    0.8541   -3.7081   -1.4349 C   0  0  0  0  0  0  0  0  0  0  0  0
    1.2707   -2.9476   -2.6710 C   0  0  0  0  0  0  0  0  0  0  0  0
    2.8402   -1.7092    0.2837 C   0  0  0  0  0  0  0  0  0  0  0  0
    2.7620   -2.5376   -2.6734 C   0  0  0  0  0  0  0  0  0  0  0  0
    3.5078   -0.8686   -0.8153 C   0  0  0  0  0  0  0  0  0  0  0  0
    3.0128   -1.0783   -2.2572 C   0  0  0  0  0  0  0  0  0  0  0  0
    0.6134   -2.0308   -2.7730 H   0  0  0  0  0  0  0  0  0  0  0  0
    3.3361   -3.2261   -2.0080 H   0  0  0  0  0  0  0  0  0  0  0  0
```

```
    3.2511   -2.7487    0.2469 H   0  0  0  0  0  0  0  0  0  0  0  0
    4.6053   -1.0954   -0.7829 H   0  0  0  0  0  0  0  0  0  0  0  0
    0.7626   -4.8021   -1.5704 H   0  0  0  0  0  0  0  0  0  0  0  0
    1.0799   -3.5963   -3.5667 H   0  0  0  0  0  0  0  0  0  0  0  0
    3.1283   -1.2718    1.2733 H   0  0  0  0  0  0  0  0  0  0  0  0
    3.1493   -2.6766   -3.7149 H   0  0  0  0  0  0  0  0  0  0  0  0
    3.3770    0.2151   -0.5667 H   0  0  0  0  0  0  0  0  0  0  0  0
    3.7998   -0.6558   -2.9372 H   0  0  0  0  0  0  0  0  0  0  0  0
    0.8430   -3.1726   -0.1293 C   0  0  0  0  0  0  0  0  0  0  0  0
    0.7422   -3.8516    0.7377 H   0  0  0  0  0  0  0  0  0  0  0  0
    1.2991   -1.7734    0.2039 C   0  0  0  0  0  0  0  0  0  0  0  0
    0.9213   -1.0586   -0.5816 H   0  0  0  0  0  0  0  0  0  0  0  0
    0.8566   -1.4676    1.1866 H   0  0  0  0  0  0  0  0  0  0  0  0
    2.0880   -0.4758   -2.4184 H   0  0  0  0  0  0  0  0  0  0  0  0
   -0.0865    2.9312   -1.0709 H   0  0  0  0  0  0  0  0  0  0  0  0
   -2.0058   -5.3210   -0.0743 C   0  0  0  0  0  0  0  0  0  0  0  0
   -1.5449   -4.3184   -0.4529 N   0  0  0  0  0  0  0  0  0  0  0  0
   -4.9944   -2.6139   -1.2249 C   0  0  0  0  0  0  0  0  0  0  0  0
   -3.8463   -2.7137   -1.2156 N   0  0  0  0  0  0  0  0  0  0  0  0
   -2.5875   -6.5751    0.3958 C   0  0  0  0  0  0  0  0  0  0  0  0
   -6.4706   -2.4862   -1.2335 C   0  0  0  0  0  0  0  0  0  0  0  0
   -2.3128   -7.6109   -0.4040 F   0  0  0  0  0  0  0  0  0  0  0  0
   -3.9217   -6.5308    0.4841 F   0  0  0  0  0  0  0  0  0  0  0  0
   -2.1563   -6.9325    1.6099 F   0  0  0  0  0  0  0  0  0  0  0  0
   -7.0863   -3.5769   -0.7697 F   0  0  0  0  0  0  0  0  0  0  0  0
   -6.9633   -2.2754   -2.4571 F   0  0  0  0  0  0  0  0  0  0  0  0
   -6.9042   -1.4698   -0.4820 F   0  0  0  0  0  0  0  0  0  0  0  0
   -0.9988   -2.9045   -1.0078 Rh  0  0  0  0  0  0  0  0  0  0  0  0
  1  2  1  0  0  0  0
  1 11  1  0  0  0  0
  1 17  2  0  0  0  0
  1 57  1  0  0  0  0
  2  4  1  0  0  0  0
  2  7  1  0  0  0  0
  2 12  1  0  0  0  0
  3  5  1  0  0  0  0
  3  9  1  0  0  0  0
  3 13  1  0  0  0  0
  3 19  1  0  0  0  0
  4  6  1  0  0  0  0
  4  8  1  0  0  0  0
  4 14  1  0  0  0  0
  5  6  1  0  0  0  0
  5 10  1  0  0  0  0
  5 15  1  0  0  0  0
  6 16  1  0  0  0  0
  6 44  1  0  0  0  0
 17 18  1  0  0  0  0
 17 19  1  0  0  0  0
 17 57  1  0  0  0  0
 19 20  1  0  0  0  0
 19 21  1  0  0  0  0
 22 23  1  0  0  0  0
 22 32  1  0  0  0  0
 22 38  2  0  0  0  0
 22 57  1  0  0  0  0
 23 25  1  0  0  0  0
 23 28  1  0  0  0  0
 23 33  1  0  0  0  0
 24 26  1  0  0  0  0
 24 30  1  0  0  0  0
 24 34  1  0  0  0  0
 24 40  1  0  0  0  0
 25 27  1  0  0  0  0
 25 29  1  0  0  0  0
```

```
 25 35  1  0  0  0  0
 26 27  1  0  0  0  0
 26 31  1  0  0  0  0
 26 36  1  0  0  0  0
 27 37  1  0  0  0  0
 27 43  1  0  0  0  0
 38 39  1  0  0  0  0
 38 40  1  0  0  0  0
 38 57  1  0  0  0  0
 40 41  1  0  0  0  0
 40 42  1  0  0  0  0
 45 46  3  0  0  0  0
 45 49  1  0  0  0  0
 46 57  1  0  0  0  0
 47 48  3  0  0  0  0
 47 50  1  0  0  0  0
 48 57  1  0  0  0  0
 49 51  1  0  0  0  0
 49 52  1  0  0  0  0
 49 53  1  0  0  0  0
 50 54  1  0  0  0  0
 50 55  1  0  0  0  0
 50 56  1  0  0  0  0
M  END
$$$$
mol114
Optimized Catalyst 114
Optimized Catalyst 114
 57 60  0  0  0  0  0  0  0  0999 V2000
   -1.6316    0.1632   -0.1041 C   0  0  0  0  0  0  0  0  0  0  0  0
   -1.2814    1.5193    0.4281 C   0  0  0  0  0  0  0  0  0  0  0  0
   -2.0403    1.4825   -3.0486 C   0  0  0  0  0  0  0  0  0  0  0  0
   -2.0397    2.6927   -0.2280 C   0  0  0  0  0  0  0  0  0  0  0  0
   -1.7605    2.9720   -2.7987 C   0  0  0  0  0  0  0  0  0  0  0  0
   -1.2847    3.3590   -1.3893 C   0  0  0  0  0  0  0  0  0  0  0  0
   -0.1759    1.6730    0.3070 H   0  0  0  0  0  0  0  0  0  0  0  0
   -3.0422    2.3488   -0.5708 H   0  0  0  0  0  0  0  0  0  0  0  0
   -3.0152    1.2132   -2.5764 H   0  0  0  0  0  0  0  0  0  0  0  0
   -2.7065    3.5307   -3.0201 H   0  0  0  0  0  0  0  0  0  0  0  0
   -2.0638   -0.5212    0.6537 H   0  0  0  0  0  0  0  0  0  0  0  0
   -1.5023    1.5408    1.5282 H   0  0  0  0  0  0  0  0  0  0  0  0
   -2.1456    1.3385   -4.1538 H   0  0  0  0  0  0  0  0  0  0  0  0
   -2.2029    3.4675    0.5661 H   0  0  0  0  0  0  0  0  0  0  0  0
   -0.9837    3.3198   -3.5264 H   0  0  0  0  0  0  0  0  0  0  0  0
   -1.4141    4.4702   -1.2950 H   0  0  0  0  0  0  0  0  0  0  0  0
   -1.4291   -0.2673   -1.3600 C   0  0  0  0  0  0  0  0  0  0  0  0
   -1.7098   -1.3890   -1.6494 H   0  0  0  0  0  0  0  0  0  0  0  0
   -0.9403    0.5290   -2.5307 C   0  0  0  0  0  0  0  0  0  0  0  0
   -0.0444    1.1291   -2.2157 H   0  0  0  0  0  0  0  0  0  0  0  0
   -0.6192   -0.1397   -3.3653 H   0  0  0  0  0  0  0  0  0  0  0  0
    0.7177   -3.4390   -1.6629 C   0  0  0  0  0  0  0  0  0  0  0  0
    1.3528   -2.7916   -2.8616 C   0  0  0  0  0  0  0  0  0  0  0  0
    2.8094   -1.8608    0.2383 C   0  0  0  0  0  0  0  0  0  0  0  0
    2.8939   -2.6959   -2.7216 C   0  0  0  0  0  0  0  0  0  0  0  0
    3.7527   -1.1920   -0.7724 C   0  0  0  0  0  0  0  0  0  0  0  0
    3.3957   -1.3176   -2.2630 C   0  0  0  0  0  0  0  0  0  0  0  0
    0.9386   -1.7667   -3.0299 H   0  0  0  0  0  0  0  0  0  0  0  0
    3.2583   -3.4846   -2.0219 H   0  0  0  0  0  0  0  0  0  0  0  0
    3.0030   -2.9616    0.2384 H   0  0  0  0  0  0  0  0  0  0  0  0
    4.7709   -1.6358   -0.6143 H   0  0  0  0  0  0  0  0  0  0  0  0
    0.4224   -4.4972   -1.8277 H   0  0  0  0  0  0  0  0  0  0  0  0
    1.1289   -3.4034   -3.7753 H   0  0  0  0  0  0  0  0  0  0  0  0
    3.0726   -1.4752    1.2564 H   0  0  0  0  0  0  0  0  0  0  0  0
    3.3335   -2.9097   -3.7300 H   0  0  0  0  0  0  0  0  0  0  0  0
    3.8212   -0.1003   -0.5334 H   0  0  0  0  0  0  0  0  0  0  0  0
```

```
    4.3312   -1.0852   -2.8403 H   0  0  0  0  0  0  0  0  0  0  0  0
    0.6347   -2.8971   -0.4154 C   0  0  0  0  0  0  0  0  0  0  0  0
    0.3307   -3.5287    0.4536 H   0  0  0  0  0  0  0  0  0  0  0  0
    1.3032   -1.6032   -0.0093 C   0  0  0  0  0  0  0  0  0  0  0  0
    1.1949   -0.8216   -0.8018 H   0  0  0  0  0  0  0  0  0  0  0  0
    0.8450   -1.2142    0.9343 H   0  0  0  0  0  0  0  0  0  0  0  0
    2.6503   -0.5326   -2.5307 H   0  0  0  0  0  0  0  0  0  0  0  0
   -0.1917    3.1549   -1.2983 H   0  0  0  0  0  0  0  0  0  0  0  0
   -2.2181   -5.7811   -0.0944 C   0  0  0  0  0  0  0  0  0  0  0  0
   -2.0269   -4.6821   -0.4283 N   0  0  0  0  0  0  0  0  0  0  0  0
   -4.8666   -2.5598   -1.2748 C   0  0  0  0  0  0  0  0  0  0  0  0
   -3.7129   -2.6602   -1.1593 N   0  0  0  0  0  0  0  0  0  0  0  0
   -2.4640   -7.1795    0.3303 C   0  0  0  0  0  0  0  0  0  0  0  0
   -6.3354   -2.4283   -1.4254 C   0  0  0  0  0  0  0  0  0  0  0  0
   -1.9992   -8.0662   -0.5513 F   0  0  0  0  0  0  0  0  0  0  0  0
   -3.7649   -7.4363    0.4785 F   0  0  0  0  0  0  0  0  0  0  0  0
   -1.8844   -7.4651    1.4974 F   0  0  0  0  0  0  0  0  0  0  0  0
   -6.9836   -3.5174   -1.0087 F   0  0  0  0  0  0  0  0  0  0  0  0
   -6.6988   -2.2311   -2.6936 F   0  0  0  0  0  0  0  0  0  0  0  0
   -6.8248   -1.4029   -0.7261 F   0  0  0  0  0  0  0  0  0  0  0  0
   -1.8136   -2.8623   -0.9785 Ir  0  0  0  0  0  0  0  0  0  0  0  0
  1  2  1  0  0  0  0
  1 11  1  0  0  0  0
  1 17  2  0  0  0  0
  1 57  1  0  0  0  0
  2  4  1  0  0  0  0
  2  7  1  0  0  0  0
  2 12  1  0  0  0  0
  3  5  1  0  0  0  0
  3  9  1  0  0  0  0
  3 13  1  0  0  0  0
  3 19  1  0  0  0  0
  4  6  1  0  0  0  0
  4  8  1  0  0  0  0
  4 14  1  0  0  0  0
  5  6  1  0  0  0  0
  5 10  1  0  0  0  0
  5 15  1  0  0  0  0
  6 16  1  0  0  0  0
  6 44  1  0  0  0  0
 17 19  1  0  0  0  0
 17 18  1  0  0  0  0
 17 57  1  0  0  0  0
 19 20  1  0  0  0  0
 19 21  1  0  0  0  0
 22 23  1  0  0  0  0
 22 32  1  0  0  0  0
 22 38  2  0  0  0  0
 22 57  1  0  0  0  0
 23 25  1  0  0  0  0
 23 28  1  0  0  0  0
 23 33  1  0  0  0  0
 24 26  1  0  0  0  0
 24 30  1  0  0  0  0
 24 34  1  0  0  0  0
 24 40  1  0  0  0  0
 25 27  1  0  0  0  0
 25 29  1  0  0  0  0
 25 35  1  0  0  0  0
 26 27  1  0  0  0  0
 26 31  1  0  0  0  0
 26 36  1  0  0  0  0
 27 37  1  0  0  0  0
 27 43  1  0  0  0  0
 38 39  1  0  0  0  0
```

```
 38 40  1  0  0  0  0
 38 57  1  0  0  0  0
 40 41  1  0  0  0  0
 40 42  1  0  0  0  0
 45 46  3  0  0  0  0
 45 49  1  0  0  0  0
 46 57  1  0  0  0  0
 47 48  3  0  0  0  0
 47 50  1  0  0  0  0
 48 57  1  0  0  0  0
 49 51  1  0  0  0  0
 49 52  1  0  0  0  0
 49 53  1  0  0  0  0
 50 54  1  0  0  0  0
 50 55  1  0  0  0  0
 50 56  1  0  0  0  0
M  END
$$$$
mol115
Optimized Catalyst 115
Optimized Catalyst 115
 75 78  0  0  0  0  0  0  0  0999 V2000
   -1.6254   -0.2390   -0.6433 C   0  0  0  0  0  0  0  0  0  0  0  0
   -1.2314    0.9598    0.1737 C   0  0  0  0  0  0  0  0  0  0  0  0
   -1.8474    1.7164   -3.2496 C   0  0  0  0  0  0  0  0  0  0  0  0
   -1.9717    2.2554   -0.2283 C   0  0  0  0  0  0  0  0  0  0  0  0
   -1.5666    3.1068   -2.6580 C   0  0  0  0  0  0  0  0  0  0  0  0
   -1.1707    3.1685   -1.1725 C   0  0  0  0  0  0  0  0  0  0  0  0
   -0.1271    1.1178    0.0854 H   0  0  0  0  0  0  0  0  0  0  0  0
   -2.9482    1.9932   -0.6968 H   0  0  0  0  0  0  0  0  0  0  0  0
   -2.8609    1.3812   -2.9214 H   0  0  0  0  0  0  0  0  0  0  0  0
   -2.4902    3.7226   -2.8033 H   0  0  0  0  0  0  0  0  0  0  0  0
   -2.1410   -1.0400   -0.0607 H   0  0  0  0  0  0  0  0  0  0  0  0
   -1.4559    0.7512    1.2500 H   0  0  0  0  0  0  0  0  0  0  0  0
   -1.8688    1.8202   -4.3630 H   0  0  0  0  0  0  0  0  0  0  0  0
   -2.1847    2.8283    0.7085 H   0  0  0  0  0  0  0  0  0  0  0  0
   -0.7419    3.5838   -3.2454 H   0  0  0  0  0  0  0  0  0  0  0  0
   -1.3180    4.2284   -0.8384 H   0  0  0  0  0  0  0  0  0  0  0  0
   -1.4321   -0.3950   -1.9656 C   0  0  0  0  0  0  0  0  0  0  0  0
   -1.8196   -1.3039   -2.4887 H   0  0  0  0  0  0  0  0  0  0  0  0
   -0.8151    0.6296   -2.8774 C   0  0  0  0  0  0  0  0  0  0  0  0
    0.0661    1.1034   -2.3808 H   0  0  0  0  0  0  0  0  0  0  0  0
   -0.4569    0.1331   -3.8113 H   0  0  0  0  0  0  0  0  0  0  0  0
    0.8233   -3.6945   -1.4448 C   0  0  0  0  0  0  0  0  0  0  0  0
    1.2420   -2.9314   -2.6778 C   0  0  0  0  0  0  0  0  0  0  0  0
    2.7943   -1.6970    0.2842 C   0  0  0  0  0  0  0  0  0  0  0  0
    2.7341   -2.5259   -2.6767 C   0  0  0  0  0  0  0  0  0  0  0  0
    3.4726   -0.8588   -0.8107 C   0  0  0  0  0  0  0  0  0  0  0  0
    2.9900   -1.0685   -2.2568 C   0  0  0  0  0  0  0  0  0  0  0  0
    0.5840   -2.0150   -2.7730 H   0  0  0  0  0  0  0  0  0  0  0  0
    3.3050   -3.2169   -2.0124 H   0  0  0  0  0  0  0  0  0  0  0  0
    3.2054   -2.7361    0.2513 H   0  0  0  0  0  0  0  0  0  0  0  0
    4.5694   -1.0841   -0.7678 H   0  0  0  0  0  0  0  0  0  0  0  0
    0.7238   -4.7868   -1.5840 H   0  0  0  0  0  0  0  0  0  0  0  0
    1.0480   -3.5739   -3.5764 H   0  0  0  0  0  0  0  0  0  0  0  0
    3.0742   -1.2560    1.2740 H   0  0  0  0  0  0  0  0  0  0  0  0
    3.1215   -2.6611   -3.7183 H   0  0  0  0  0  0  0  0  0  0  0  0
    3.3382    0.2250   -0.5653 H   0  0  0  0  0  0  0  0  0  0  0  0
    3.7843   -0.6496   -2.9292 H   0  0  0  0  0  0  0  0  0  0  0  0
    0.7996   -3.1583   -0.1420 C   0  0  0  0  0  0  0  0  0  0  0  0
    0.6774   -3.8327    0.7257 H   0  0  0  0  0  0  0  0  0  0  0  0
    1.2543   -1.7599    0.1935 C   0  0  0  0  0  0  0  0  0  0  0  0
    0.8814   -1.0473   -0.5957 H   0  0  0  0  0  0  0  0  0  0  0  0
    0.8026   -1.4537    1.1713 H   0  0  0  0  0  0  0  0  0  0  0  0
    2.0686   -0.4625   -2.4256 H   0  0  0  0  0  0  0  0  0  0  0  0
```

```
   -0.0819    2.9454   -1.0767 H   0  0  0  0  0  0  0  0  0  0  0  0
   -2.0471   -5.4236   -0.0755 C   0  0  0  0  0  0  0  0  0  0  0  0
   -1.5588   -4.4345   -0.4345 N   0  0  0  0  0  0  0  0  0  0  0  0
   -4.8162   -2.6365   -1.2352 C   0  0  0  0  0  0  0  0  0  0  0  0
   -3.6667   -2.7603   -1.2699 N   0  0  0  0  0  0  0  0  0  0  0  0
   -2.6560   -6.7019    0.3902 C   0  0  0  0  0  0  0  0  0  0  0  0
   -6.2945   -2.4769   -1.1745 C   0  0  0  0  0  0  0  0  0  0  0  0
   -4.1835   -6.5103    0.4037 C   0  0  0  0  0  0  0  0  0  0  0  0
   -4.5578   -6.2662   -0.6152 H   0  0  0  0  0  0  0  0  0  0  0  0
   -4.6714   -7.4554    0.7419 H   0  0  0  0  0  0  0  0  0  0  0  0
   -4.4721   -5.6958    1.1041 H   0  0  0  0  0  0  0  0  0  0  0  0
   -2.2458   -7.8062   -0.5991 C   0  0  0  0  0  0  0  0  0  0  0  0
   -2.7056   -8.7742   -0.2866 H   0  0  0  0  0  0  0  0  0  0  0  0
   -2.5984   -7.5690   -1.6281 H   0  0  0  0  0  0  0  0  0  0  0  0
   -1.1408   -7.9362   -0.6156 H   0  0  0  0  0  0  0  0  0  0  0  0
   -2.1219   -6.9954    1.8023 C   0  0  0  0  0  0  0  0  0  0  0  0
   -2.3868   -6.1753    2.5066 H   0  0  0  0  0  0  0  0  0  0  0  0
   -2.5773   -7.9418    2.1812 H   0  0  0  0  0  0  0  0  0  0  0  0
   -1.0165   -7.1225    1.7919 H   0  0  0  0  0  0  0  0  0  0  0  0
   -6.8788   -3.7774   -0.5938 C   0  0  0  0  0  0  0  0  0  0  0  0
   -6.6395   -4.6439   -1.2494 H   0  0  0  0  0  0  0  0  0  0  0  0
   -6.4811   -3.9675    0.4274 H   0  0  0  0  0  0  0  0  0  0  0  0
   -7.9892   -3.6873   -0.5258 H   0  0  0  0  0  0  0  0  0  0  0  0
   -6.8082   -2.2276   -2.6021 C   0  0  0  0  0  0  0  0  0  0  0  0
   -6.5604   -3.0842   -3.2684 H   0  0  0  0  0  0  0  0  0  0  0  0
   -7.9172   -2.1065   -2.5840 H   0  0  0  0  0  0  0  0  0  0  0  0
   -6.3625   -1.2997   -3.0267 H   0  0  0  0  0  0  0  0  0  0  0  0
   -6.5951   -1.2772   -0.2587 C   0  0  0  0  0  0  0  0  0  0  0  0
   -6.1453   -0.3449   -0.6694 H   0  0  0  0  0  0  0  0  0  0  0  0
   -7.6987   -1.1305   -0.1858 H   0  0  0  0  0  0  0  0  0  0  0  0
   -6.1963   -1.4503    0.7657 H   0  0  0  0  0  0  0  0  0  0  0  0
   -1.0671   -2.9406   -1.0169 Rh  0  0  0  0  0  0  0  0  0  0  0  0
  1  2  1  0  0  0  0
  1 11  1  0  0  0  0
  1 17  2  0  0  0  0
  1 75  1  0  0  0  0
  2  4  1  0  0  0  0
  2  7  1  0  0  0  0
  2 12  1  0  0  0  0
  3  5  1  0  0  0  0
  3  9  1  0  0  0  0
  3 13  1  0  0  0  0
  3 19  1  0  0  0  0
  4  6  1  0  0  0  0
  4  8  1  0  0  0  0
  4 14  1  0  0  0  0
  5  6  1  0  0  0  0
  5 10  1  0  0  0  0
  5 15  1  0  0  0  0
  6 16  1  0  0  0  0
  6 44  1  0  0  0  0
 17 18  1  0  0  0  0
 17 19  1  0  0  0  0
 17 75  1  0  0  0  0
 19 20  1  0  0  0  0
 19 21  1  0  0  0  0
 22 23  1  0  0  0  0
 22 32  1  0  0  0  0
 22 38  2  0  0  0  0
 22 75  1  0  0  0  0
 23 25  1  0  0  0  0
 23 28  1  0  0  0  0
 23 33  1  0  0  0  0
 24 26  1  0  0  0  0
 24 30  1  0  0  0  0
```

```
 24 34  1  0  0  0  0
 24 40  1  0  0  0  0
 25 27  1  0  0  0  0
 25 29  1  0  0  0  0
 25 35  1  0  0  0  0
 26 27  1  0  0  0  0
 26 31  1  0  0  0  0
 26 36  1  0  0  0  0
 27 37  1  0  0  0  0
 27 43  1  0  0  0  0
 38 39  1  0  0  0  0
 38 40  1  0  0  0  0
 38 75  1  0  0  0  0
 40 41  1  0  0  0  0
 40 42  1  0  0  0  0
 45 46  3  0  0  0  0
 45 49  1  0  0  0  0
 46 75  1  0  0  0  0
 47 48  3  0  0  0  0
 47 50  1  0  0  0  0
 48 75  1  0  0  0  0
 49 51  1  0  0  0  0
 49 55  1  0  0  0  0
 49 59  1  0  0  0  0
 50 63  1  0  0  0  0
 50 67  1  0  0  0  0
 50 71  1  0  0  0  0
 51 52  1  0  0  0  0
 51 53  1  0  0  0  0
 51 54  1  0  0  0  0
 55 56  1  0  0  0  0
 55 57  1  0  0  0  0
 55 58  1  0  0  0  0
 59 60  1  0  0  0  0
 59 61  1  0  0  0  0
 59 62  1  0  0  0  0
 63 64  1  0  0  0  0
 63 65  1  0  0  0  0
 63 66  1  0  0  0  0
 67 68  1  0  0  0  0
 67 69  1  0  0  0  0
 67 70  1  0  0  0  0
 71 72  1  0  0  0  0
 71 73  1  0  0  0  0
 71 74  1  0  0  0  0
M  END
$$$$
mol116
Optimized Catalyst 116
Optimized Catalyst 116
 75 78  0  0  0  0  0  0  0  0999 V2000
   -3.1536    0.4544   -0.7202 C   0  0  0  0  0  0  0  0  0  0  0  0
   -2.1768    1.0748    0.2462 C   0  0  0  0  0  0  0  0  0  0  0  0
   -1.5181    1.8827   -3.1556 C   0  0  0  0  0  0  0  0  0  0  0  0
   -1.6667    2.4773   -0.1487 C   0  0  0  0  0  0  0  0  0  0  0  0
   -0.4270    2.6813   -2.4259 C   0  0  0  0  0  0  0  0  0  0  0  0
   -0.3269    2.4807   -0.9036 C   0  0  0  0  0  0  0  0  0  0  0  0
   -1.3023    0.3489    0.3627 H   0  0  0  0  0  0  0  0  0  0  0  0
   -2.4477    2.9896   -0.7601 H   0  0  0  0  0  0  0  0  0  0  0  0
   -2.5106    2.3599   -2.9654 H   0  0  0  0  0  0  0  0  0  0  0  0
   -0.6141    3.7665   -2.6303 H   0  0  0  0  0  0  0  0  0  0  0  0
   -4.1949    0.3417   -0.3358 H   0  0  0  0  0  0  0  0  0  0  0  0
   -2.6725    1.1532    1.2499 H   0  0  0  0  0  0  0  0  0  0  0  0
   -1.3174    1.9511   -4.2544 H   0  0  0  0  0  0  0  0  0  0  0  0
   -1.5356    3.0658    0.7951 H   0  0  0  0  0  0  0  0  0  0  0  0
```

```
    0.5662    2.4169   -2.8686 H   0  0  0  0  0  0  0  0  0  0  0  0
    0.2961    3.3232   -0.5030 H   0  0  0  0  0  0  0  0  0  0  0  0
   -2.8796    0.0872   -2.0402 C   0  0  0  0  0  0  0  0  0  0  0  0
   -3.7040   -0.3199   -2.6714 H   0  0  0  0  0  0  0  0  0  0  0  0
   -1.5933    0.3922   -2.7607 C   0  0  0  0  0  0  0  0  0  0  0  0
   -0.7229    0.1264   -2.0902 H   0  0  0  0  0  0  0  0  0  0  0  0
   -1.5225   -0.2431   -3.6794 H   0  0  0  0  0  0  0  0  0  0  0  0
    0.1880   -2.2870   -1.9736 C   0  0  0  0  0  0  0  0  0  0  0  0
    1.1483   -2.5996   -3.0830 C   0  0  0  0  0  0  0  0  0  0  0  0
    2.6571   -2.5947    0.1573 C   0  0  0  0  0  0  0  0  0  0  0  0
    2.2300   -3.6356   -2.7050 C   0  0  0  0  0  0  0  0  0  0  0  0
    3.8292   -3.0052   -0.7484 C   0  0  0  0  0  0  0  0  0  0  0  0
    3.5666   -3.0147   -2.2640 C   0  0  0  0  0  0  0  0  0  0  0  0
    1.6361   -1.6458   -3.4109 H   0  0  0  0  0  0  0  0  0  0  0  0
    1.8402   -4.2997   -1.9008 H   0  0  0  0  0  0  0  0  0  0  0  0
    1.9542   -3.4561    0.2517 H   0  0  0  0  0  0  0  0  0  0  0  0
    4.1502   -4.0310   -0.4375 H   0  0  0  0  0  0  0  0  0  0  0  0
   -0.8735   -2.5297   -2.2130 H   0  0  0  0  0  0  0  0  0  0  0  0
    0.5761   -3.0001   -3.9574 H   0  0  0  0  0  0  0  0  0  0  0  0
    3.0722   -2.3804    1.1733 H   0  0  0  0  0  0  0  0  0  0  0  0
    2.4239   -4.2673   -3.6076 H   0  0  0  0  0  0  0  0  0  0  0  0
    4.6842   -2.3074   -0.5614 H   0  0  0  0  0  0  0  0  0  0  0  0
    4.3968   -3.6001   -2.7372 H   0  0  0  0  0  0  0  0  0  0  0  0
    0.4957   -1.7410   -0.7873 C   0  0  0  0  0  0  0  0  0  0  0  0
   -0.3012   -1.5613   -0.0306 H   0  0  0  0  0  0  0  0  0  0  0  0
    1.8703   -1.3574   -0.3233 C   0  0  0  0  0  0  0  0  0  0  0  0
    2.4226   -0.8554   -1.1559 H   0  0  0  0  0  0  0  0  0  0  0  0
    1.7892   -0.6246    0.5158 H   0  0  0  0  0  0  0  0  0  0  0  0
    3.6415   -1.9712   -2.6516 H   0  0  0  0  0  0  0  0  0  0  0  0
    0.2281    1.5355   -0.6922 H   0  0  0  0  0  0  0  0  0  0  0  0
   -2.1346   -4.1132    0.0956 C   0  0  0  0  0  0  0  0  0  0  0  0
   -2.2717   -3.0110   -0.2384 N   0  0  0  0  0  0  0  0  0  0  0  0
   -6.0383   -2.5468   -0.9235 C   0  0  0  0  0  0  0  0  0  0  0  0
   -4.9455   -2.1682   -0.9377 N   0  0  0  0  0  0  0  0  0  0  0  0
   -1.9059   -5.5261    0.5130 C   0  0  0  0  0  0  0  0  0  0  0  0
   -7.4497   -3.0227   -0.9054 C   0  0  0  0  0  0  0  0  0  0  0  0
   -7.7507   -3.5497    0.5087 C   0  0  0  0  0  0  0  0  0  0  0  0
   -8.8090   -3.9016    0.5572 H   0  0  0  0  0  0  0  0  0  0  0  0
   -7.0870   -4.4056    0.7623 H   0  0  0  0  0  0  0  0  0  0  0  0
   -7.6170   -2.7466    1.2680 H   0  0  0  0  0  0  0  0  0  0  0  0
   -7.5852   -4.1422   -1.9519 C   0  0  0  0  0  0  0  0  0  0  0  0
   -8.6360   -4.5186   -1.9606 H   0  0  0  0  0  0  0  0  0  0  0  0
   -7.3435   -3.7637   -2.9707 H   0  0  0  0  0  0  0  0  0  0  0  0
   -6.9115   -4.9942   -1.7114 H   0  0  0  0  0  0  0  0  0  0  0  0
   -8.3506   -1.8254   -1.2539 C   0  0  0  0  0  0  0  0  0  0  0  0
   -8.1192   -1.4379   -2.2717 H   0  0  0  0  0  0  0  0  0  0  0  0
   -9.4196   -2.1457   -1.2381 H   0  0  0  0  0  0  0  0  0  0  0  0
   -8.2231   -1.0034   -0.5141 H   0  0  0  0  0  0  0  0  0  0  0  0
   -2.5459   -6.4341   -0.5521 C   0  0  0  0  0  0  0  0  0  0  0  0
   -2.4046   -7.5030   -0.2633 H   0  0  0  0  0  0  0  0  0  0  0  0
   -3.6371   -6.2362   -0.6389 H   0  0  0  0  0  0  0  0  0  0  0  0
   -2.0670   -6.2767   -1.5451 H   0  0  0  0  0  0  0  0  0  0  0  0
   -2.5652   -5.7281    1.8870 C   0  0  0  0  0  0  0  0  0  0  0  0
   -2.4148   -6.7828    2.2199 H   0  0  0  0  0  0  0  0  0  0  0  0
   -2.1101   -5.0557    2.6489 H   0  0  0  0  0  0  0  0  0  0  0  0
   -3.6588   -5.5296    1.8359 H   0  0  0  0  0  0  0  0  0  0  0  0
   -0.3842   -5.7439    0.5837 C   0  0  0  0  0  0  0  0  0  0  0  0
    0.0738   -5.0935    1.3625 H   0  0  0  0  0  0  0  0  0  0  0  0
   -0.1714   -6.8072    0.8461 H   0  0  0  0  0  0  0  0  0  0  0  0
    0.0925   -5.5234   -0.3984 H   0  0  0  0  0  0  0  0  0  0  0  0
   -2.5517   -1.4746   -0.7934 Rh  0  0  0  0  0  0  0  0  0  0  0  0
  1  2  1  0  0  0  0
  1 11  1  0  0  0  0
  1 17  2  0  0  0  0
  1 75  1  0  0  0  0
```

```
  2  4  1  0  0  0  0
  2 12  1  0  0  0  0
  2  7  1  0  0  0  0
  3  5  1  0  0  0  0
  3  9  1  0  0  0  0
  3 13  1  0  0  0  0
  3 19  1  0  0  0  0
  4  6  1  0  0  0  0
  4  8  1  0  0  0  0
  4 14  1  0  0  0  0
  5  6  1  0  0  0  0
  5 10  1  0  0  0  0
  5 15  1  0  0  0  0
  6 16  1  0  0  0  0
  6 44  1  0  0  0  0
 17 18  1  0  0  0  0
 17 19  1  0  0  0  0
 17 75  1  0  0  0  0
 19 20  1  0  0  0  0
 19 21  1  0  0  0  0
 22 23  1  0  0  0  0
 22 32  1  0  0  0  0
 22 38  2  0  0  0  0
 22 75  1  0  0  0  0
 23 25  1  0  0  0  0
 23 28  1  0  0  0  0
 23 33  1  0  0  0  0
 24 26  1  0  0  0  0
 24 30  1  0  0  0  0
 24 34  1  0  0  0  0
 24 40  1  0  0  0  0
 25 27  1  0  0  0  0
 25 29  1  0  0  0  0
 25 35  1  0  0  0  0
 26 27  1  0  0  0  0
 26 31  1  0  0  0  0
 26 36  1  0  0  0  0
 27 37  1  0  0  0  0
 27 43  1  0  0  0  0
 38 39  1  0  0  0  0
 38 40  1  0  0  0  0
 38 75  1  0  0  0  0
 40 41  1  0  0  0  0
 40 42  1  0  0  0  0
 45 46  3  0  0  0  0
 45 49  1  0  0  0  0
 46 75  1  0  0  0  0
 47 48  3  0  0  0  0
 47 50  1  0  0  0  0
 48 75  1  0  0  0  0
 49 63  1  0  0  0  0
 49 67  1  0  0  0  0
 49 71  1  0  0  0  0
 50 51  1  0  0  0  0
 50 55  1  0  0  0  0
 50 59  1  0  0  0  0
 51 52  1  0  0  0  0
 51 53  1  0  0  0  0
 51 54  1  0  0  0  0
 55 56  1  0  0  0  0
 55 57  1  0  0  0  0
 55 58  1  0  0  0  0
 59 60  1  0  0  0  0
 59 61  1  0  0  0  0
 59 62  1  0  0  0  0
```

```
 63 64  1  0  0  0  0
 63 65  1  0  0  0  0
 63 66  1  0  0  0  0
 67 68  1  0  0  0  0
 67 69  1  0  0  0  0
 67 70  1  0  0  0  0
 71 72  1  0  0  0  0
 71 73  1  0  0  0  0
 71 74  1  0  0  0  0
M  END
$$$$
mol117
Optimized Catalyst 117
Optimized Catalyst 117
 51 56  0  0  0  0  0  0  0  0999 V2000
   -0.9791    0.3540   -1.5705 C   0  0  0  0  0  0  0  0  0  0  0  0
    0.1922   -0.5694   -1.1772 C   0  0  0  0  0  0  0  0  0  0  0  0
   -2.2315    0.1434   -0.7533 C   0  0  0  0  0  0  0  0  0  0  0  0
   -1.1085    0.1814    1.5524 C   0  0  0  0  0  0  0  0  0  0  0  0
   -2.2974    0.0424    0.6305 C   0  0  0  0  0  0  0  0  0  0  0  0
   -0.4378   -1.1766    1.8484 C   0  0  0  0  0  0  0  0  0  0  0  0
   -3.1664    0.2771   -1.3313 H   0  0  0  0  0  0  0  0  0  0  0  0
   -3.2807    0.0515    1.1357 H   0  0  0  0  0  0  0  0  0  0  0  0
   -1.2268    0.1707   -2.6501 H   0  0  0  0  0  0  0  0  0  0  0  0
   -0.6626    1.4271   -1.4656 H   0  0  0  0  0  0  0  0  0  0  0  0
    0.8273   -0.7486   -2.0851 H   0  0  0  0  0  0  0  0  0  0  0  0
   -0.9495   -1.6336    2.7387 H   0  0  0  0  0  0  0  0  0  0  0  0
   -1.4477    0.6317    2.5230 H   0  0  0  0  0  0  0  0  0  0  0  0
   -0.3695    0.8928    1.1014 H   0  0  0  0  0  0  0  0  0  0  0  0
   -0.5113   -2.1682    0.7087 C   0  0  0  0  0  0  0  0  0  0  0  0
   -0.2785   -1.9001   -0.6375 C   0  0  0  0  0  0  0  0  0  0  0  0
   -0.6163   -3.2292    1.0368 H   0  0  0  0  0  0  0  0  0  0  0  0
   -0.2500   -2.7407   -1.3659 H   0  0  0  0  0  0  0  0  0  0  0  0
    0.8434   -0.0700   -0.4143 H   0  0  0  0  0  0  0  0  0  0  0  0
    0.6414   -1.0128    2.1110 H   0  0  0  0  0  0  0  0  0  0  0  0
   -3.9579   -2.0129   -0.4559 N   0  0  0  0  0  0  0  0  0  0  0  0
   -5.1021   -2.1661   -0.6116 C   0  0  0  0  0  0  0  0  0  0  0  0
   -6.5110   -2.3237   -0.8084 C   0  0  0  0  0  0  0  0  0  0  0  0
   -6.9722   -3.1585   -1.8434 C   0  0  0  0  0  0  0  0  0  0  0  0
   -7.4127   -1.6433    0.0307 C   0  0  0  0  0  0  0  0  0  0  0  0
   -8.3449   -3.3159   -2.0370 C   0  0  0  0  0  0  0  0  0  0  0  0
   -6.2648   -3.6776   -2.4926 H   0  0  0  0  0  0  0  0  0  0  0  0
   -8.7859   -1.8031   -0.1642 C   0  0  0  0  0  0  0  0  0  0  0  0
   -7.0472   -0.9945    0.8291 H   0  0  0  0  0  0  0  0  0  0  0  0
   -9.2368   -2.6383   -1.1947 C   0  0  0  0  0  0  0  0  0  0  0  0
   -8.7349   -3.9614   -2.8374 H   0  0  0  0  0  0  0  0  0  0  0  0
   -9.5144   -1.2836    0.4757 H   0  0  0  0  0  0  0  0  0  0  0  0
   -2.3600   -4.4702   -0.6778 N   0  0  0  0  0  0  0  0  0  0  0  0
   -2.4439   -5.6053   -0.8952 C   0  0  0  0  0  0  0  0  0  0  0  0
   -2.5451   -7.0124   -1.1680 C   0  0  0  0  0  0  0  0  0  0  0  0
   -3.5206   -7.7786   -0.5055 C   0  0  0  0  0  0  0  0  0  0  0  0
   -1.6678   -7.6020   -2.0957 C   0  0  0  0  0  0  0  0  0  0  0  0
   -3.6232   -9.1441   -0.7775 C   0  0  0  0  0  0  0  0  0  0  0  0
   -4.1944   -7.3139    0.2180 H   0  0  0  0  0  0  0  0  0  0  0  0
   -1.7705   -8.9677   -2.3669 C   0  0  0  0  0  0  0  0  0  0  0  0
   -0.9098   -7.0005   -2.6030 H   0  0  0  0  0  0  0  0  0  0  0  0
   -2.7492   -9.7219   -1.7074 C   0  0  0  0  0  0  0  0  0  0  0  0
   -4.3771   -9.7677   -0.2753 H   0  0  0  0  0  0  0  0  0  0  0  0
   -1.0970   -9.4556   -3.0866 H   0  0  0  0  0  0  0  0  0  0  0  0
   -2.3146   -1.9676   -0.2337 Rh  0  0  0  0  0  0  0  0  0  0  0  0
  -10.7074   -2.8143   -1.4041 N   0  0  0  0  0  0  0  0  0  0  0  0
   -2.8666  -11.1849   -2.0056 N   0  0  0  0  0  0  0  0  0  0  0  0
  -11.0445   -3.5822   -2.2836 O   0  0  0  0  0  0  0  0  0  0  0  0
  -11.4450   -2.1779   -0.6782 O   0  0  0  0  0  0  0  0  0  0  0  0
   -3.7713  -11.7823   -1.4575 O   0  0  0  0  0  0  0  0  0  0  0  0
```

```
   -2.0500  -11.6538   -2.7732 O   0  0  0  0  0  0  0  0  0  0  0  0
  1  2  1  0  0  0  0
  1  3  1  0  0  0  0
  1  9  1  0  0  0  0
  1 10  1  0  0  0  0
  2 11  1  0  0  0  0
  2 16  1  0  0  0  0
  2 19  1  0  0  0  0
  3  5  2  0  0  0  0
  3  7  1  0  0  0  0
  3 45  1  0  0  0  0
  4  5  1  0  0  0  0
  4  6  1  0  0  0  0
  4 13  1  0  0  0  0
  4 14  1  0  0  0  0
  5  8  1  0  0  0  0
  5 45  1  0  0  0  0
  6 12  1  0  0  0  0
  6 15  1  0  0  0  0
  6 20  1  0  0  0  0
 15 16  2  0  0  0  0
 15 17  1  0  0  0  0
 15 45  1  0  0  0  0
 16 18  1  0  0  0  0
 16 45  1  0  0  0  0
 21 22  3  0  0  0  0
 21 45  1  0  0  0  0
 22 23  1  0  0  0  0
 23 24  2  0  0  0  0
 23 25  1  0  0  0  0
 24 26  1  0  0  0  0
 24 27  1  0  0  0  0
 25 28  2  0  0  0  0
 25 29  1  0  0  0  0
 26 30  2  0  0  0  0
 26 31  1  0  0  0  0
 28 30  1  0  0  0  0
 28 32  1  0  0  0  0
 30 46  1  0  0  0  0
 33 34  3  0  0  0  0
 33 45  1  0  0  0  0
 34 35  1  0  0  0  0
 35 36  2  0  0  0  0
 35 37  1  0  0  0  0
 36 38  1  0  0  0  0
 36 39  1  0  0  0  0
 37 40  2  0  0  0  0
 37 41  1  0  0  0  0
 38 42  2  0  0  0  0
 38 43  1  0  0  0  0
 40 42  1  0  0  0  0
 40 44  1  0  0  0  0
 42 47  1  0  0  0  0
 46 48  2  0  0  0  0
 46 49  2  0  0  0  0
 47 50  2  0  0  0  0
 47 51  2  0  0  0  0
M  END
$$$$
mol118
Optimized Catalyst 118
Optimized Catalyst 118
 51 56  0  0  0  0  0  0  0  0999 V2000
   -0.8366    0.2562   -1.5377 C   0  0  0  0  0  0  0  0  0  0  0  0
    0.3851   -0.6029   -1.1520 C   0  0  0  0  0  0  0  0  0  0  0  0
```

```
   -2.0992    0.0103   -0.7439 C   0  0  0  0  0  0  0  0  0  0  0  0
   -1.0071    0.0334    1.5638 C   0  0  0  0  0  0  0  0  0  0  0  0
   -2.1812   -0.1122    0.6228 C   0  0  0  0  0  0  0  0  0  0  0  0
   -0.2187   -1.2671    1.8254 C   0  0  0  0  0  0  0  0  0  0  0  0
   -3.0275    0.1790   -1.3365 H   0  0  0  0  0  0  0  0  0  0  0  0
   -3.1733   -0.0827    1.1232 H   0  0  0  0  0  0  0  0  0  0  0  0
   -1.0533    0.1037   -2.6311 H   0  0  0  0  0  0  0  0  0  0  0  0
   -0.5654    1.3454   -1.4088 H   0  0  0  0  0  0  0  0  0  0  0  0
    1.0371   -0.7111   -2.0636 H   0  0  0  0  0  0  0  0  0  0  0  0
   -0.6360   -1.7624    2.7454 H   0  0  0  0  0  0  0  0  0  0  0  0
   -1.3834    0.4177    2.5529 H   0  0  0  0  0  0  0  0  0  0  0  0
   -0.3182    0.8243    1.1587 H   0  0  0  0  0  0  0  0  0  0  0  0
   -0.1933   -2.2687    0.6932 C   0  0  0  0  0  0  0  0  0  0  0  0
    0.0521   -1.9808   -0.6283 C   0  0  0  0  0  0  0  0  0  0  0  0
   -0.1395   -3.3254    1.0433 H   0  0  0  0  0  0  0  0  0  0  0  0
    0.2519   -2.8034   -1.3477 H   0  0  0  0  0  0  0  0  0  0  0  0
    1.0043   -0.0660   -0.3819 H   0  0  0  0  0  0  0  0  0  0  0  0
    0.8532   -0.9970    2.0581 H   0  0  0  0  0  0  0  0  0  0  0  0
   -4.2623   -2.3015   -0.5876 N   0  0  0  0  0  0  0  0  0  0  0  0
   -5.4180   -2.3829   -0.7362 C   0  0  0  0  0  0  0  0  0  0  0  0
   -6.8333   -2.4692   -0.9156 C   0  0  0  0  0  0  0  0  0  0  0  0
   -7.3500   -3.2654   -1.9566 C   0  0  0  0  0  0  0  0  0  0  0  0
   -7.6883   -1.7620   -0.0487 C   0  0  0  0  0  0  0  0  0  0  0  0
   -8.7321   -3.3589   -2.1250 C   0  0  0  0  0  0  0  0  0  0  0  0
   -6.6812   -3.8053   -2.6307 H   0  0  0  0  0  0  0  0  0  0  0  0
   -9.0705   -1.8562   -0.2226 C   0  0  0  0  0  0  0  0  0  0  0  0
   -7.2814   -1.1438    0.7553 H   0  0  0  0  0  0  0  0  0  0  0  0
   -9.5755   -2.6564   -1.2547 C   0  0  0  0  0  0  0  0  0  0  0  0
   -9.1653   -3.9750   -2.9282 H   0  0  0  0  0  0  0  0  0  0  0  0
   -9.7630   -1.3114    0.4381 H   0  0  0  0  0  0  0  0  0  0  0  0
   -2.2882   -4.2551   -0.6084 N   0  0  0  0  0  0  0  0  0  0  0  0
   -2.3695   -5.4013   -0.8183 C   0  0  0  0  0  0  0  0  0  0  0  0
   -2.4653   -6.8018   -1.0861 C   0  0  0  0  0  0  0  0  0  0  0  0
   -3.4364   -7.5683   -0.4122 C   0  0  0  0  0  0  0  0  0  0  0  0
   -1.6010   -7.3896   -2.0297 C   0  0  0  0  0  0  0  0  0  0  0  0
   -3.5513   -8.9296   -0.6964 C   0  0  0  0  0  0  0  0  0  0  0  0
   -4.0945   -7.1083    0.3286 H   0  0  0  0  0  0  0  0  0  0  0  0
   -1.7178   -8.7525   -2.3087 C   0  0  0  0  0  0  0  0  0  0  0  0
   -0.8439   -6.7914   -2.5427 H   0  0  0  0  0  0  0  0  0  0  0  0
   -2.6941   -9.5043   -1.6436 C   0  0  0  0  0  0  0  0  0  0  0  0
   -4.3038   -9.5535   -0.1896 H   0  0  0  0  0  0  0  0  0  0  0  0
   -1.0552   -9.2402   -3.0408 H   0  0  0  0  0  0  0  0  0  0  0  0
  -11.0636   -2.7678   -1.4331 N   0  0  0  0  0  0  0  0  0  0  0  0
   -2.8261  -10.9688   -1.9542 N   0  0  0  0  0  0  0  0  0  0  0  0
  -11.4486   -3.5336   -2.2920 O   0  0  0  0  0  0  0  0  0  0  0  0
  -11.7496   -2.0854   -0.7011 O   0  0  0  0  0  0  0  0  0  0  0  0
   -3.7394  -11.5558   -1.4121 O   0  0  0  0  0  0  0  0  0  0  0  0
   -2.0107  -11.4354   -2.7219 O   0  0  0  0  0  0  0  0  0  0  0  0
   -2.3228   -2.3187   -0.3312 Ir  0  0  0  0  0  0  0  0  0  0  0  0
  1  2  1  0  0  0  0
  1  3  1  0  0  0  0
  1  9  1  0  0  0  0
  1 10  1  0  0  0  0
  2 11  1  0  0  0  0
  2 16  1  0  0  0  0
  2 19  1  0  0  0  0
  3  5  2  0  0  0  0
  3  7  1  0  0  0  0
  3 51  1  0  0  0  0
  4  5  1  0  0  0  0
  4  6  1  0  0  0  0
  4 13  1  0  0  0  0
  4 14  1  0  0  0  0
  5  8  1  0  0  0  0
  5 51  1  0  0  0  0
```

```
  6 12  1  0  0  0  0
  6 15  1  0  0  0  0
  6 20  1  0  0  0  0
 15 16  2  0  0  0  0
 15 17  1  0  0  0  0
 15 51  1  0  0  0  0
 16 18  1  0  0  0  0
 16 51  1  0  0  0  0
 21 22  3  0  0  0  0
 21 51  1  0  0  0  0
 22 23  1  0  0  0  0
 23 24  2  0  0  0  0
 23 25  1  0  0  0  0
 24 26  1  0  0  0  0
 24 27  1  0  0  0  0
 25 28  2  0  0  0  0
 25 29  1  0  0  0  0
 26 30  2  0  0  0  0
 26 31  1  0  0  0  0
 28 30  1  0  0  0  0
 28 32  1  0  0  0  0
 30 45  1  0  0  0  0
 33 34  3  0  0  0  0
 33 51  1  0  0  0  0
 34 35  1  0  0  0  0
 35 36  2  0  0  0  0
 35 37  1  0  0  0  0
 36 38  1  0  0  0  0
 36 39  1  0  0  0  0
 37 40  2  0  0  0  0
 37 41  1  0  0  0  0
 38 42  2  0  0  0  0
 38 43  1  0  0  0  0
 40 42  1  0  0  0  0
 40 44  1  0  0  0  0
 42 46  1  0  0  0  0
 45 47  2  0  0  0  0
 45 48  2  0  0  0  0
 46 49  2  0  0  0  0
 46 50  2  0  0  0  0
M  END
$$$$
mol119
Optimized Catalyst 119
Optimized Catalyst 119
 59 64  0  0  0  0  0  0  0  0999 V2000
   -0.9933    0.3248   -1.5718 C   0  0  0  0  0  0  0  0  0  0  0  0
    0.1827   -0.5960   -1.1850 C   0  0  0  0  0  0  0  0  0  0  0  0
   -2.2438    0.1029   -0.7542 C   0  0  0  0  0  0  0  0  0  0  0  0
   -1.1164    0.1514    1.5473 C   0  0  0  0  0  0  0  0  0  0  0  0
   -2.3064    0.0018    0.6282 C   0  0  0  0  0  0  0  0  0  0  0  0
   -0.4420   -1.2041    1.8458 C   0  0  0  0  0  0  0  0  0  0  0  0
   -3.1806    0.2171   -1.3327 H   0  0  0  0  0  0  0  0  0  0  0  0
   -3.2882   -0.0116    1.1361 H   0  0  0  0  0  0  0  0  0  0  0  0
   -1.2411    0.1451   -2.6517 H   0  0  0  0  0  0  0  0  0  0  0  0
   -0.6818    1.3977   -1.4595 H   0  0  0  0  0  0  0  0  0  0  0  0
    0.8126   -0.7750   -2.0960 H   0  0  0  0  0  0  0  0  0  0  0  0
   -0.9587   -1.6637    2.7316 H   0  0  0  0  0  0  0  0  0  0  0  0
   -1.4560    0.6033    2.5163 H   0  0  0  0  0  0  0  0  0  0  0  0
   -0.3802    0.8622    1.0922 H   0  0  0  0  0  0  0  0  0  0  0  0
   -0.5065   -2.1972    0.7063 C   0  0  0  0  0  0  0  0  0  0  0  0
   -0.2780   -1.9291   -0.6416 C   0  0  0  0  0  0  0  0  0  0  0  0
   -0.5937   -3.2587    1.0361 H   0  0  0  0  0  0  0  0  0  0  0  0
   -0.2388   -2.7722   -1.3669 H   0  0  0  0  0  0  0  0  0  0  0  0
    0.8358   -0.0953   -0.4252 H   0  0  0  0  0  0  0  0  0  0  0  0
```

```
    0.6351   -1.0377    2.1126 H   0  0  0  0  0  0  0  0  0  0  0  0
   -3.9750   -2.0715   -0.4695 N   0  0  0  0  0  0  0  0  0  0  0  0
   -5.1084   -2.2630   -0.6332 C   0  0  0  0  0  0  0  0  0  0  0  0
   -2.3310   -4.4278   -0.6524 N   0  0  0  0  0  0  0  0  0  0  0  0
   -2.4281   -5.5594   -0.8783 C   0  0  0  0  0  0  0  0  0  0  0  0
   -2.3110   -2.0146   -0.2386 Rh  0  0  0  0  0  0  0  0  0  0  0  0
   -2.5467   -6.9934   -1.1604 C   0  0  0  0  0  0  0  0  0  0  0  0
   -2.2533   -7.2654   -2.6541 C   0  0  0  0  0  0  0  0  0  0  0  0
   -1.5895   -7.7912   -0.2447 C   0  0  0  0  0  0  0  0  0  0  0  0
   -3.6061   -7.3066   -0.9366 H   0  0  0  0  0  0  0  0  0  0  0  0
   -2.3471   -8.7705   -2.9496 C   0  0  0  0  0  0  0  0  0  0  0  0
   -1.2252   -6.9080   -2.9020 H   0  0  0  0  0  0  0  0  0  0  0  0
   -2.9894   -6.7165   -3.2866 H   0  0  0  0  0  0  0  0  0  0  0  0
   -1.6861   -9.2935   -0.5529 C   0  0  0  0  0  0  0  0  0  0  0  0
   -0.5407   -7.4498   -0.4158 H   0  0  0  0  0  0  0  0  0  0  0  0
   -1.8588   -7.6139    0.8230 H   0  0  0  0  0  0  0  0  0  0  0  0
   -1.4166   -9.5846   -2.0373 C   0  0  0  0  0  0  0  0  0  0  0  0
   -2.0631   -8.9478   -4.0156 H   0  0  0  0  0  0  0  0  0  0  0  0
   -3.3991   -9.1145   -2.8057 H   0  0  0  0  0  0  0  0  0  0  0  0
   -0.9363   -9.8391    0.0704 H   0  0  0  0  0  0  0  0  0  0  0  0
   -2.7032   -9.6653   -0.2821 H   0  0  0  0  0  0  0  0  0  0  0  0
   -1.5757  -10.6735   -2.2308 H   0  0  0  0  0  0  0  0  0  0  0  0
   -0.3544   -9.3382   -2.2766 H   0  0  0  0  0  0  0  0  0  0  0  0
   -6.5473   -2.4707   -0.8374 C   0  0  0  0  0  0  0  0  0  0  0  0
   -6.8007   -3.0203   -2.2605 C   0  0  0  0  0  0  0  0  0  0  0  0
   -7.0974   -3.4271    0.2463 C   0  0  0  0  0  0  0  0  0  0  0  0
   -7.0585   -1.4713   -0.7353 H   0  0  0  0  0  0  0  0  0  0  0  0
   -8.3009   -3.2785   -2.4714 C   0  0  0  0  0  0  0  0  0  0  0  0
   -6.2411   -3.9760   -2.3954 H   0  0  0  0  0  0  0  0  0  0  0  0
   -6.4433   -2.2818   -3.0162 H   0  0  0  0  0  0  0  0  0  0  0  0
   -8.5955   -3.6837    0.0191 C   0  0  0  0  0  0  0  0  0  0  0  0
   -6.5479   -4.3970    0.2008 H   0  0  0  0  0  0  0  0  0  0  0  0
   -6.9491   -2.9742    1.2549 H   0  0  0  0  0  0  0  0  0  0  0  0
   -8.8712   -4.2152   -1.3956 C   0  0  0  0  0  0  0  0  0  0  0  0
   -8.4500   -3.7409   -3.4776 H   0  0  0  0  0  0  0  0  0  0  0  0
   -8.8537   -2.3087   -2.4478 H   0  0  0  0  0  0  0  0  0  0  0  0
   -8.9515   -4.4323    0.7684 H   0  0  0  0  0  0  0  0  0  0  0  0
   -9.1647   -2.7359    0.1756 H   0  0  0  0  0  0  0  0  0  0  0  0
   -9.9752   -4.3090   -1.5415 H   0  0  0  0  0  0  0  0  0  0  0  0
   -8.4170   -5.2284   -1.5066 H   0  0  0  0  0  0  0  0  0  0  0  0
  1 10  1  0  0  0  0
  1  2  1  0  0  0  0
  1  3  1  0  0  0  0
  1  9  1  0  0  0  0
  2 16  1  0  0  0  0
  2 19  1  0  0  0  0
  2 11  1  0  0  0  0
  3  5  2  0  0  0  0
  3  7  1  0  0  0  0
  3 25  1  0  0  0  0
  4  6  1  0  0  0  0
  4 13  1  0  0  0  0
  4  5  1  0  0  0  0
  4 14  1  0  0  0  0
  5  8  1  0  0  0  0
  5 25  1  0  0  0  0
  6 20  1  0  0  0  0
  6 12  1  0  0  0  0
  6 15  1  0  0  0  0
 15 17  1  0  0  0  0
 15 16  2  0  0  0  0
 15 25  1  0  0  0  0
 16 18  1  0  0  0  0
 16 25  1  0  0  0  0
 21 25  1  0  0  0  0
```

```
 21 22  1  0  0  0  0
 22 43  1  0  0  0  0
 23 24  3  0  0  0  0
 23 25  1  0  0  0  0
 24 26  1  0  0  0  0
 26 29  1  0  0  0  0
 26 28  1  0  0  0  0
 26 27  1  0  0  0  0
 27 30  1  0  0  0  0
 27 31  1  0  0  0  0
 27 32  1  0  0  0  0
 28 35  1  0  0  0  0
 28 33  1  0  0  0  0
 28 34  1  0  0  0  0
 30 38  1  0  0  0  0
 30 36  1  0  0  0  0
 30 37  1  0  0  0  0
 33 40  1  0  0  0  0
 33 39  1  0  0  0  0
 33 36  1  0  0  0  0
 36 41  1  0  0  0  0
 36 42  1  0  0  0  0
 43 46  1  0  0  0  0
 43 45  1  0  0  0  0
 43 44  1  0  0  0  0
 44 47  1  0  0  0  0
 44 48  1  0  0  0  0
 44 49  1  0  0  0  0
 45 52  1  0  0  0  0
 45 50  1  0  0  0  0
 45 51  1  0  0  0  0
 47 55  1  0  0  0  0
 47 53  1  0  0  0  0
 47 54  1  0  0  0  0
 50 57  1  0  0  0  0
 50 56  1  0  0  0  0
 50 53  1  0  0  0  0
 53 58  1  0  0  0  0
 53 59  1  0  0  0  0
M  END
$$$$
mol120
Optimized Catalyst 120
Optimized Catalyst 120
 59 64  0  0  0  0  0  0  0  0999 V2000
   -0.8004    0.2743   -1.5252 C   0  0  0  0  0  0  0  0  0  0  0  0
    0.4217   -0.5901   -1.1495 C   0  0  0  0  0  0  0  0  0  0  0  0
   -2.0615    0.0227   -0.7296 C   0  0  0  0  0  0  0  0  0  0  0  0
   -0.9564    0.0409    1.5718 C   0  0  0  0  0  0  0  0  0  0  0  0
   -2.1361   -0.1044    0.6376 C   0  0  0  0  0  0  0  0  0  0  0  0
   -0.1672   -1.2609    1.8264 C   0  0  0  0  0  0  0  0  0  0  0  0
   -2.9932    0.1927   -1.3164 H   0  0  0  0  0  0  0  0  0  0  0  0
   -3.1255   -0.0828    1.1432 H   0  0  0  0  0  0  0  0  0  0  0  0
   -1.0196    0.1273   -2.6185 H   0  0  0  0  0  0  0  0  0  0  0  0
   -0.5291    1.3616   -1.3887 H   0  0  0  0  0  0  0  0  0  0  0  0
    1.0688   -0.6973   -2.0642 H   0  0  0  0  0  0  0  0  0  0  0  0
   -0.5819   -1.7565    2.7468 H   0  0  0  0  0  0  0  0  0  0  0  0
   -1.3264    0.4236    2.5635 H   0  0  0  0  0  0  0  0  0  0  0  0
   -0.2701    0.8316    1.1628 H   0  0  0  0  0  0  0  0  0  0  0  0
   -0.1526   -2.2607    0.6923 C   0  0  0  0  0  0  0  0  0  0  0  0
    0.0851   -1.9693   -0.6305 C   0  0  0  0  0  0  0  0  0  0  0  0
   -0.1059   -3.3189    1.0383 H   0  0  0  0  0  0  0  0  0  0  0  0
    0.2755   -2.7901   -1.3552 H   0  0  0  0  0  0  0  0  0  0  0  0
    1.0458   -0.0591   -0.3801 H   0  0  0  0  0  0  0  0  0  0  0  0
    0.9062   -0.9940    2.0531 H   0  0  0  0  0  0  0  0  0  0  0  0
```

```
   -4.2335   -2.3128   -0.5896 N   0  0  0  0  0  0  0  0  0  0  0  0
   -5.3823   -2.4458   -0.7365 C   0  0  0  0  0  0  0  0  0  0  0  0
   -2.2436   -4.2463   -0.6297 N   0  0  0  0  0  0  0  0  0  0  0  0
   -2.3399   -5.3888   -0.8399 C   0  0  0  0  0  0  0  0  0  0  0  0
   -2.4633   -6.8233   -1.1102 C   0  0  0  0  0  0  0  0  0  0  0  0
   -2.1893   -7.1041   -2.6070 C   0  0  0  0  0  0  0  0  0  0  0  0
   -1.5091   -7.6317   -0.2003 C   0  0  0  0  0  0  0  0  0  0  0  0
   -3.5272   -7.1211   -0.8730 H   0  0  0  0  0  0  0  0  0  0  0  0
   -2.3333   -8.6086   -2.8890 C   0  0  0  0  0  0  0  0  0  0  0  0
   -1.1508   -6.7843   -2.8647 H   0  0  0  0  0  0  0  0  0  0  0  0
   -2.9123   -6.5377   -3.2387 H   0  0  0  0  0  0  0  0  0  0  0  0
   -1.6564   -9.1334   -0.4968 C   0  0  0  0  0  0  0  0  0  0  0  0
   -0.4536   -7.3248   -0.3955 H   0  0  0  0  0  0  0  0  0  0  0  0
   -1.7535   -7.4404    0.8708 H   0  0  0  0  0  0  0  0  0  0  0  0
   -1.4139   -9.4402   -1.9822 C   0  0  0  0  0  0  0  0  0  0  0  0
   -2.0680   -8.8019   -3.9581 H   0  0  0  0  0  0  0  0  0  0  0  0
   -3.3932   -8.9221   -2.7314 H   0  0  0  0  0  0  0  0  0  0  0  0
   -0.9139   -9.6974    0.1208 H   0  0  0  0  0  0  0  0  0  0  0  0
   -2.6794   -9.4761   -0.2088 H   0  0  0  0  0  0  0  0  0  0  0  0
   -1.6016  -10.5265   -2.1676 H   0  0  0  0  0  0  0  0  0  0  0  0
   -0.3484   -9.2237   -2.2362 H   0  0  0  0  0  0  0  0  0  0  0  0
   -6.8279   -2.6035   -0.9184 C   0  0  0  0  0  0  0  0  0  0  0  0
   -7.1217   -3.1428   -2.3388 C   0  0  0  0  0  0  0  0  0  0  0  0
   -7.3927   -3.5429    0.1742 C   0  0  0  0  0  0  0  0  0  0  0  0
   -7.3070   -1.5864   -0.8078 H   0  0  0  0  0  0  0  0  0  0  0  0
   -8.6349   -3.3419   -2.5234 C   0  0  0  0  0  0  0  0  0  0  0  0
   -6.6071   -4.1224   -2.4817 H   0  0  0  0  0  0  0  0  0  0  0  0
   -6.7500   -2.4191   -3.1022 H   0  0  0  0  0  0  0  0  0  0  0  0
   -8.9035   -3.7400   -0.0305 C   0  0  0  0  0  0  0  0  0  0  0  0
   -6.8863   -4.5352    0.1142 H   0  0  0  0  0  0  0  0  0  0  0  0
   -7.2132   -3.1008    1.1828 H   0  0  0  0  0  0  0  0  0  0  0  0
   -9.2194   -4.2605   -1.4404 C   0  0  0  0  0  0  0  0  0  0  0  0
   -8.8195   -3.7945   -3.5291 H   0  0  0  0  0  0  0  0  0  0  0  0
   -9.1507   -2.3518   -2.4886 H   0  0  0  0  0  0  0  0  0  0  0  0
   -9.2779   -4.4744    0.7252 H   0  0  0  0  0  0  0  0  0  0  0  0
   -9.4343   -2.7712    0.1351 H   0  0  0  0  0  0  0  0  0  0  0  0
  -10.3289   -4.3161   -1.5687 H   0  0  0  0  0  0  0  0  0  0  0  0
   -8.8049   -5.2899   -1.5602 H   0  0  0  0  0  0  0  0  0  0  0  0
   -2.2799   -2.2996   -0.3375 Ir  0  0  0  0  0  0  0  0  0  0  0  0
  1 10  1  0  0  0  0
  1  2  1  0  0  0  0
  1  3  1  0  0  0  0
  1  9  1  0  0  0  0
  2 16  1  0  0  0  0
  2 19  1  0  0  0  0
  2 11  1  0  0  0  0
  3  7  1  0  0  0  0
  3 59  1  0  0  0  0
  3  5  2  0  0  0  0
  4  6  1  0  0  0  0
  4 13  1  0  0  0  0
  4  5  1  0  0  0  0
  4 14  1  0  0  0  0
  5  8  1  0  0  0  0
  5 59  1  0  0  0  0
  6 20  1  0  0  0  0
  6 12  1  0  0  0  0
  6 15  1  0  0  0  0
 15 17  1  0  0  0  0
 15 16  2  0  0  0  0
 15 59  1  0  0  0  0
 16 18  1  0  0  0  0
 16 59  1  0  0  0  0
 21 59  1  0  0  0  0
 21 22  3  0  0  0  0
```

```
 22 42  1  0  0  0  0
 23 59  1  0  0  0  0
 23 24  3  0  0  0  0
 24 25  1  0  0  0  0
 25 28  1  0  0  0  0
 25 27  1  0  0  0  0
 25 26  1  0  0  0  0
 26 29  1  0  0  0  0
 26 30  1  0  0  0  0
 26 31  1  0  0  0  0
 27 34  1  0  0  0  0
 27 32  1  0  0  0  0
 27 33  1  0  0  0  0
 29 37  1  0  0  0  0
 29 35  1  0  0  0  0
 29 36  1  0  0  0  0
 32 39  1  0  0  0  0
 32 38  1  0  0  0  0
 32 35  1  0  0  0  0
 35 40  1  0  0  0  0
 35 41  1  0  0  0  0
 42 45  1  0  0  0  0
 42 44  1  0  0  0  0
 42 43  1  0  0  0  0
 43 46  1  0  0  0  0
 43 47  1  0  0  0  0
 43 48  1  0  0  0  0
 44 51  1  0  0  0  0
 44 49  1  0  0  0  0
 44 50  1  0  0  0  0
 46 54  1  0  0  0  0
 46 52  1  0  0  0  0
 46 53  1  0  0  0  0
 49 56  1  0  0  0  0
 49 55  1  0  0  0  0
 49 52  1  0  0  0  0
 52 57  1  0  0  0  0
 52 58  1  0  0  0  0
M  END
$$$$
mol121
Optimized Catalyst 121
Optimized Catalyst 121
 65 70  0  0  0  0  0  0  0  0999 V2000
   -0.9831    0.3397   -1.5688 C   0  0  0  0  0  0  0  0  0  0  0  0
    0.1858   -0.5904   -1.1823 C   0  0  0  0  0  0  0  0  0  0  0  0
   -2.2365    0.1235   -0.7540 C   0  0  0  0  0  0  0  0  0  0  0  0
   -1.1117    0.1574    1.5485 C   0  0  0  0  0  0  0  0  0  0  0  0
   -2.3018    0.0176    0.6281 C   0  0  0  0  0  0  0  0  0  0  0  0
   -0.4371   -1.2002    1.8387 C   0  0  0  0  0  0  0  0  0  0  0  0
   -3.1716    0.2471   -1.3329 H   0  0  0  0  0  0  0  0  0  0  0  0
   -3.2846    0.0102    1.1341 H   0  0  0  0  0  0  0  0  0  0  0  0
   -1.2304    0.1645   -2.6495 H   0  0  0  0  0  0  0  0  0  0  0  0
   -0.6642    1.4102   -1.4538 H   0  0  0  0  0  0  0  0  0  0  0  0
    0.8189   -0.7672   -2.0913 H   0  0  0  0  0  0  0  0  0  0  0  0
   -0.9433   -1.6579    2.7311 H   0  0  0  0  0  0  0  0  0  0  0  0
   -1.4510    0.6031    2.5205 H   0  0  0  0  0  0  0  0  0  0  0  0
   -0.3750    0.8711    1.0982 H   0  0  0  0  0  0  0  0  0  0  0  0
   -0.5164   -2.1932    0.6997 C   0  0  0  0  0  0  0  0  0  0  0  0
   -0.2859   -1.9235   -0.6480 C   0  0  0  0  0  0  0  0  0  0  0  0
   -0.6184   -3.2541    1.0287 H   0  0  0  0  0  0  0  0  0  0  0  0
   -0.2567   -2.7631   -1.3782 H   0  0  0  0  0  0  0  0  0  0  0  0
    0.8380   -0.0971   -0.4167 H   0  0  0  0  0  0  0  0  0  0  0  0
    0.6435   -1.0358    2.0924 H   0  0  0  0  0  0  0  0  0  0  0  0
   -3.9771   -2.0469   -0.4766 N   0  0  0  0  0  0  0  0  0  0  0  0
```

```
   -5.1112   -2.2349   -0.6388 C   0  0  0  0  0  0  0  0  0  0  0  0
   -2.3518   -4.4470   -0.6407 N   0  0  0  0  0  0  0  0  0  0  0  0
   -2.4486   -5.5795   -0.8630 C   0  0  0  0  0  0  0  0  0  0  0  0
   -2.5673   -7.0132   -1.1475 C   0  0  1  0  0  0  0  0  0  0  0  0
   -2.2726   -7.2784   -2.6422 C   0  0  0  0  0  0  0  0  0  0  0  0
   -1.6103   -7.8167   -0.2371 C   0  0  0  0  0  0  0  0  0  0  0  0
   -3.6260   -7.3285   -0.9255 H   0  0  0  0  0  0  0  0  0  0  0  0
   -2.3562   -8.7805   -2.9467 C   0  0  0  0  0  0  0  0  0  0  0  0
   -1.2480   -6.9106   -2.8885 H   0  0  0  0  0  0  0  0  0  0  0  0
   -3.0136   -6.7322   -3.2712 H   0  0  0  0  0  0  0  0  0  0  0  0
   -1.6982   -9.3156   -0.5579 C   0  0  0  0  0  0  0  0  0  0  0  0
   -0.5629   -7.4675   -0.3996 H   0  0  0  0  0  0  0  0  0  0  0  0
   -1.8853   -7.6515    0.8312 H   0  0  0  0  0  0  0  0  0  0  0  0
   -1.3997   -9.5945   -2.0476 C   0  0  1  0  0  0  0  0  0  0  0  0
   -2.0837   -8.9484   -4.0166 H   0  0  0  0  0  0  0  0  0  0  0  0
   -3.4013   -9.1381   -2.7938 H   0  0  0  0  0  0  0  0  0  0  0  0
   -0.9577   -9.8645    0.0726 H   0  0  0  0  0  0  0  0  0  0  0  0
   -2.7165   -9.6949   -0.3073 H   0  0  0  0  0  0  0  0  0  0  0  0
   -0.3498   -9.2706   -2.2640 H   0  0  0  0  0  0  0  0  0  0  0  0
   -6.5495   -2.4436   -0.8399 C   0  0  1  0  0  0  0  0  0  0  0  0
   -6.8042   -2.9969   -2.2609 C   0  0  0  0  0  0  0  0  0  0  0  0
   -7.0924   -3.4004    0.2468 C   0  0  0  0  0  0  0  0  0  0  0  0
   -7.0640   -1.4461   -0.7385 H   0  0  0  0  0  0  0  0  0  0  0  0
   -8.3007   -3.2711   -2.4654 C   0  0  0  0  0  0  0  0  0  0  0  0
   -6.2331   -3.9453   -2.3992 H   0  0  0  0  0  0  0  0  0  0  0  0
   -6.4587   -2.2540   -3.0180 H   0  0  0  0  0  0  0  0  0  0  0  0
   -8.5861   -3.6712    0.0206 C   0  0  0  0  0  0  0  0  0  0  0  0
   -6.5323   -4.3645    0.2075 H   0  0  0  0  0  0  0  0  0  0  0  0
   -6.9490   -2.9406    1.2532 H   0  0  0  0  0  0  0  0  0  0  0  0
   -8.8510   -4.2362   -1.3923 C   0  0  1  0  0  0  0  0  0  0  0  0
   -8.4517   -3.7214   -3.4760 H   0  0  0  0  0  0  0  0  0  0  0  0
   -8.8678   -2.3112   -2.4254 H   0  0  0  0  0  0  0  0  0  0  0  0
   -8.9402   -4.4068    0.7823 H   0  0  0  0  0  0  0  0  0  0  0  0
   -9.1641   -2.7266    0.1558 H   0  0  0  0  0  0  0  0  0  0  0  0
   -8.3145   -5.2142   -1.4881 H   0  0  0  0  0  0  0  0  0  0  0  0
   -1.5167  -11.0927   -2.3519 C   0  0  0  0  0  0  0  0  0  0  0  0
   -1.2725  -11.2873   -3.4207 H   0  0  0  0  0  0  0  0  0  0  0  0
   -0.8045  -11.6699   -1.7197 H   0  0  0  0  0  0  0  0  0  0  0  0
   -2.5473  -11.4602   -2.1508 H   0  0  0  0  0  0  0  0  0  0  0  0
  -10.3493   -4.4819   -1.6034 C   0  0  0  0  0  0  0  0  0  0  0  0
  -10.5294   -4.9131   -2.6140 H   0  0  0  0  0  0  0  0  0  0  0  0
  -10.7318   -5.1996   -0.8428 H   0  0  0  0  0  0  0  0  0  0  0  0
  -10.9238   -3.5331   -1.5155 H   0  0  0  0  0  0  0  0  0  0  0  0
   -2.3154   -1.9959   -0.2448 Rh  0  0  0  0  0  0  0  0  0  0  0  0
  1  2  1  0  0  0  0
  1  3  1  0  0  0  0
  1  9  1  0  0  0  0
  1 10  1  0  0  0  0
  2 11  1  0  0  0  0
  2 16  1  0  0  0  0
  2 19  1  0  0  0  0
  3  5  2  0  0  0  0
  3  7  1  0  0  0  0
  3 65  1  0  0  0  0
  4  5  1  0  0  0  0
  4  6  1  0  0  0  0
  4 13  1  0  0  0  0
  4 14  1  0  0  0  0
  5  8  1  0  0  0  0
  5 65  1  0  0  0  0
  6 12  1  0  0  0  0
  6 15  1  0  0  0  0
  6 20  1  0  0  0  0
 15 16  2  0  0  0  0
 15 17  1  0  0  0  0
```

```
 15 65  1  0  0  0  0
 16 18  1  0  0  0  0
 16 65  1  0  0  0  0
 21 22  3  0  0  0  0
 21 65  1  0  0  0  0
 22 41  1  0  0  0  0
 23 24  3  0  0  0  0
 23 65  1  0  0  0  0
 24 25  1  0  0  0  0
 25 26  1  0  0  0  0
 25 27  1  0  0  0  0
 25 28  1  1  0  0  0
 26 29  1  0  0  0  0
 26 30  1  0  0  0  0
 26 31  1  0  0  0  0
 27 32  1  0  0  0  0
 27 33  1  0  0  0  0
 27 34  1  0  0  0  0
 29 35  1  0  0  0  0
 29 36  1  0  0  0  0
 29 37  1  0  0  0  0
 32 35  1  0  0  0  0
 32 38  1  0  0  0  0
 32 39  1  0  0  0  0
 35 40  1  6  0  0  0
 35 57  1  0  0  0  0
 41 42  1  0  0  0  0
 41 43  1  0  0  0  0
 41 44  1  1  0  0  0
 42 45  1  0  0  0  0
 42 46  1  0  0  0  0
 42 47  1  0  0  0  0
 43 48  1  0  0  0  0
 43 49  1  0  0  0  0
 43 50  1  0  0  0  0
 45 51  1  0  0  0  0
 45 52  1  0  0  0  0
 45 53  1  0  0  0  0
 48 51  1  0  0  0  0
 48 54  1  0  0  0  0
 48 55  1  0  0  0  0
 51 56  1  6  0  0  0
 51 61  1  0  0  0  0
 57 58  1  0  0  0  0
 57 59  1  0  0  0  0
 57 60  1  0  0  0  0
 61 62  1  0  0  0  0
 61 63  1  0  0  0  0
 61 64  1  0  0  0  0
M  END
$$$$
mol122
Optimized Catalyst 122
Optimized Catalyst 122
 65 70  0  0  0  0  0  0  0  0999 V2000
   -0.7845    0.2715   -1.5176 C   0  0  0  0  0  0  0  0  0  0  0  0
    0.4374   -0.5976   -1.1521 C   0  0  0  0  0  0  0  0  0  0  0  0
   -2.0441    0.0204   -0.7195 C   0  0  0  0  0  0  0  0  0  0  0  0
   -0.9341    0.0303    1.5779 C   0  0  0  0  0  0  0  0  0  0  0  0
   -2.1170   -0.1080    0.6474 C   0  0  0  0  0  0  0  0  0  0  0  0
   -0.1500   -1.2761    1.8243 C   0  0  0  0  0  0  0  0  0  0  0  0
   -2.9768    0.1911   -1.3042 H   0  0  0  0  0  0  0  0  0  0  0  0
   -3.1054   -0.0861    1.1552 H   0  0  0  0  0  0  0  0  0  0  0  0
   -1.0100    0.1296   -2.6104 H   0  0  0  0  0  0  0  0  0  0  0  0
   -0.5094    1.3570   -1.3762 H   0  0  0  0  0  0  0  0  0  0  0  0
```

```
    1.0783   -0.7022   -2.0715 H   0  0  0  0  0  0  0  0  0  0  0  0
   -0.5654   -1.7750    2.7431 H   0  0  0  0  0  0  0  0  0  0  0  0
   -1.2992    0.4114    2.5720 H   0  0  0  0  0  0  0  0  0  0  0  0
   -0.2466    0.8204    1.1693 H   0  0  0  0  0  0  0  0  0  0  0  0
   -0.1379   -2.2716    0.6860 C   0  0  0  0  0  0  0  0  0  0  0  0
    0.1012   -1.9776   -0.6361 C   0  0  0  0  0  0  0  0  0  0  0  0
   -0.0909   -3.3307    1.0288 H   0  0  0  0  0  0  0  0  0  0  0  0
    0.2917   -2.7975   -1.3618 H   0  0  0  0  0  0  0  0  0  0  0  0
    1.0681   -0.0719   -0.3840 H   0  0  0  0  0  0  0  0  0  0  0  0
    0.9245   -1.0132    2.0494 H   0  0  0  0  0  0  0  0  0  0  0  0
   -4.2186   -2.3061   -0.5864 N   0  0  0  0  0  0  0  0  0  0  0  0
   -5.3681   -2.4310   -0.7350 C   0  0  0  0  0  0  0  0  0  0  0  0
   -2.2355   -4.2508   -0.6290 N   0  0  0  0  0  0  0  0  0  0  0  0
   -2.3271   -5.3937   -0.8393 C   0  0  0  0  0  0  0  0  0  0  0  0
   -2.4444   -6.8277   -1.1123 C   0  0  1  0  0  0  0  0  0  0  0  0
   -2.1696   -7.1049   -2.6092 C   0  0  0  0  0  0  0  0  0  0  0  0
   -1.4866   -7.6364   -0.2073 C   0  0  0  0  0  0  0  0  0  0  0  0
   -3.5062   -7.1332   -0.8758 H   0  0  0  0  0  0  0  0  0  0  0  0
   -2.3126   -8.6068   -2.8951 C   0  0  0  0  0  0  0  0  0  0  0  0
   -1.1333   -6.7796   -2.8676 H   0  0  0  0  0  0  0  0  0  0  0  0
   -2.8956   -6.5398   -3.2395 H   0  0  0  0  0  0  0  0  0  0  0  0
   -1.6323   -9.1361   -0.5064 C   0  0  0  0  0  0  0  0  0  0  0  0
   -0.4325   -7.3242   -0.3996 H   0  0  0  0  0  0  0  0  0  0  0  0
   -1.7317   -7.4507    0.8651 H   0  0  0  0  0  0  0  0  0  0  0  0
   -1.3797   -9.4500   -1.9978 C   0  0  1  0  0  0  0  0  0  0  0  0
   -2.0549   -8.7952   -3.9663 H   0  0  0  0  0  0  0  0  0  0  0  0
   -3.3695   -8.9246   -2.7322 H   0  0  0  0  0  0  0  0  0  0  0  0
   -0.8931   -9.7000    0.1142 H   0  0  0  0  0  0  0  0  0  0  0  0
   -2.6551   -9.4806   -0.2238 H   0  0  0  0  0  0  0  0  0  0  0  0
   -0.3196   -9.1833   -2.2405 H   0  0  0  0  0  0  0  0  0  0  0  0
   -6.8134   -2.5886   -0.9181 C   0  0  1  0  0  0  0  0  0  0  0  0
   -7.1019   -3.1293   -2.3387 C   0  0  0  0  0  0  0  0  0  0  0  0
   -7.3732   -3.5305    0.1746 C   0  0  0  0  0  0  0  0  0  0  0  0
   -7.2962   -1.5735   -0.8083 H   0  0  0  0  0  0  0  0  0  0  0  0
   -8.6090   -3.3564   -2.5243 C   0  0  0  0  0  0  0  0  0  0  0  0
   -6.5656   -4.0961   -2.4876 H   0  0  0  0  0  0  0  0  0  0  0  0
   -6.7466   -2.3941   -3.0996 H   0  0  0  0  0  0  0  0  0  0  0  0
   -8.8774   -3.7544   -0.0359 C   0  0  0  0  0  0  0  0  0  0  0  0
   -6.8476   -4.5133    0.1269 H   0  0  0  0  0  0  0  0  0  0  0  0
   -7.2087   -3.0770    1.1811 H   0  0  0  0  0  0  0  0  0  0  0  0
   -9.1813   -4.3034   -1.4470 C   0  0  1  0  0  0  0  0  0  0  0  0
   -8.7835   -3.8016   -3.5344 H   0  0  0  0  0  0  0  0  0  0  0  0
   -9.1460   -2.3793   -2.4798 H   0  0  0  0  0  0  0  0  0  0  0  0
   -9.2425   -4.4841    0.7278 H   0  0  0  0  0  0  0  0  0  0  0  0
   -9.4265   -2.7944    0.1130 H   0  0  0  0  0  0  0  0  0  0  0  0
   -8.6871   -5.3022   -1.5536 H   0  0  0  0  0  0  0  0  0  0  0  0
   -1.5780  -10.9445   -2.2740 C   0  0  0  0  0  0  0  0  0  0  0  0
   -1.3630  -11.1700   -3.3436 H   0  0  0  0  0  0  0  0  0  0  0  0
   -0.8847  -11.5482   -1.6449 H   0  0  0  0  0  0  0  0  0  0  0  0
   -2.6217  -11.2576   -2.0497 H   0  0  0  0  0  0  0  0  0  0  0  0
  -10.6907   -4.4872   -1.6385 C   0  0  0  0  0  0  0  0  0  0  0  0
  -10.9031   -4.9045   -2.6491 H   0  0  0  0  0  0  0  0  0  0  0  0
  -11.0925   -5.1934   -0.8766 H   0  0  0  0  0  0  0  0  0  0  0  0
  -11.2279   -3.5177   -1.5378 H   0  0  0  0  0  0  0  0  0  0  0  0
   -2.2655   -2.3042   -0.3356 Ir  0  0  0  0  0  0  0  0  0  0  0  0
  1  2  1  0  0  0  0
  1  3  1  0  0  0  0
  1  9  1  0  0  0  0
  1 10  1  0  0  0  0
  2 11  1  0  0  0  0
  2 16  1  0  0  0  0
  2 19  1  0  0  0  0
  3  5  2  0  0  0  0
  3  7  1  0  0  0  0
  3 65  1  0  0  0  0
```

```
  4  5  1  0  0  0  0
  4  6  1  0  0  0  0
  4 13  1  0  0  0  0
  4 14  1  0  0  0  0
  5  8  1  0  0  0  0
  5 65  1  0  0  0  0
  6 12  1  0  0  0  0
  6 15  1  0  0  0  0
  6 20  1  0  0  0  0
 15 16  2  0  0  0  0
 15 17  1  0  0  0  0
 15 65  1  0  0  0  0
 16 18  1  0  0  0  0
 16 65  1  0  0  0  0
 21 22  3  0  0  0  0
 21 65  1  0  0  0  0
 22 41  1  0  0  0  0
 23 24  3  0  0  0  0
 23 65  1  0  0  0  0
 24 25  1  0  0  0  0
 25 26  1  0  0  0  0
 25 27  1  0  0  0  0
 25 28  1  1  0  0  0
 26 29  1  0  0  0  0
 26 30  1  0  0  0  0
 26 31  1  0  0  0  0
 27 32  1  0  0  0  0
 27 33  1  0  0  0  0
 27 34  1  0  0  0  0
 29 35  1  0  0  0  0
 29 36  1  0  0  0  0
 29 37  1  0  0  0  0
 32 35  1  0  0  0  0
 32 38  1  0  0  0  0
 32 39  1  0  0  0  0
 35 40  1  6  0  0  0
 35 57  1  0  0  0  0
 41 42  1  0  0  0  0
 41 43  1  0  0  0  0
 41 44  1  1  0  0  0
 42 45  1  0  0  0  0
 42 46  1  0  0  0  0
 42 47  1  0  0  0  0
 43 48  1  0  0  0  0
 43 49  1  0  0  0  0
 43 50  1  0  0  0  0
 45 51  1  0  0  0  0
 45 52  1  0  0  0  0
 45 53  1  0  0  0  0
 48 51  1  0  0  0  0
 48 54  1  0  0  0  0
 48 55  1  0  0  0  0
 51 56  1  6  0  0  0
 51 61  1  0  0  0  0
 57 58  1  0  0  0  0
 57 59  1  0  0  0  0
 57 60  1  0  0  0  0
 61 62  1  0  0  0  0
 61 63  1  0  0  0  0
 61 64  1  0  0  0  0
M  END
$$$$
mol123
Optimized Catalyst 123
Optimized Catalyst 123
```

```
 75 80  0  0  0  0  0  0  0  0999 V2000
   -1.6323   -0.2746   -0.6332 C   0  0  0  0  0  0  0  0  0  0  0  0
   -1.2274    0.9217    0.1821 C   0  0  0  0  0  0  0  0  0  0  0  0
   -1.8669    1.6853   -3.2375 C   0  0  0  0  0  0  0  0  0  0  0  0
   -1.9724    2.2174   -0.2123 C   0  0  0  0  0  0  0  0  0  0  0  0
   -1.5869    3.0743   -2.6423 C   0  0  0  0  0  0  0  0  0  0  0  0
   -1.1787    3.1329   -1.1601 C   0  0  0  0  0  0  0  0  0  0  0  0
   -0.1241    1.0803    0.0810 H   0  0  0  0  0  0  0  0  0  0  0  0
   -2.9517    1.9560   -0.6754 H   0  0  0  0  0  0  0  0  0  0  0  0
   -2.8802    1.3484   -2.9105 H   0  0  0  0  0  0  0  0  0  0  0  0
   -2.5145    3.6869   -2.7777 H   0  0  0  0  0  0  0  0  0  0  0  0
   -2.1511   -1.0717   -0.0477 H   0  0  0  0  0  0  0  0  0  0  0  0
   -1.4393    0.7132    1.2609 H   0  0  0  0  0  0  0  0  0  0  0  0
   -1.8889    1.7919   -4.3509 H   0  0  0  0  0  0  0  0  0  0  0  0
   -2.1802    2.7882    0.7272 H   0  0  0  0  0  0  0  0  0  0  0  0
   -0.7695    3.5573   -3.2353 H   0  0  0  0  0  0  0  0  0  0  0  0
   -1.3245    4.1923   -0.8226 H   0  0  0  0  0  0  0  0  0  0  0  0
   -1.4505   -0.4260   -1.9582 C   0  0  0  0  0  0  0  0  0  0  0  0
   -1.8530   -1.3253   -2.4848 H   0  0  0  0  0  0  0  0  0  0  0  0
   -0.8326    0.5990   -2.8688 C   0  0  0  0  0  0  0  0  0  0  0  0
    0.0489    1.0735   -2.3731 H   0  0  0  0  0  0  0  0  0  0  0  0
   -0.4746    0.1046   -3.8040 H   0  0  0  0  0  0  0  0  0  0  0  0
    0.8257   -3.7154   -1.4314 C   0  0  0  0  0  0  0  0  0  0  0  0
    1.2384   -2.9543   -2.6679 C   0  0  0  0  0  0  0  0  0  0  0  0
    2.8166   -1.7215    0.2888 C   0  0  0  0  0  0  0  0  0  0  0  0
    2.7312   -2.5510   -2.6747 C   0  0  0  0  0  0  0  0  0  0  0  0
    3.4880   -0.8880   -0.8136 C   0  0  0  0  0  0  0  0  0  0  0  0
    2.9920   -1.0945   -2.2556 C   0  0  0  0  0  0  0  0  0  0  0  0
    0.5822   -2.0362   -2.7631 H   0  0  0  0  0  0  0  0  0  0  0  0
    3.3039   -3.2436   -2.0131 H   0  0  0  0  0  0  0  0  0  0  0  0
    3.2266   -2.7612    0.2577 H   0  0  0  0  0  0  0  0  0  0  0  0
    4.5841   -1.1205   -0.7816 H   0  0  0  0  0  0  0  0  0  0  0  0
    0.7252   -4.8081   -1.5690 H   0  0  0  0  0  0  0  0  0  0  0  0
    1.0409   -3.5985   -3.5650 H   0  0  0  0  0  0  0  0  0  0  0  0
    3.1036   -1.2772    1.2754 H   0  0  0  0  0  0  0  0  0  0  0  0
    3.1135   -2.6871   -3.7183 H   0  0  0  0  0  0  0  0  0  0  0  0
    3.3637    0.1969   -0.5673 H   0  0  0  0  0  0  0  0  0  0  0  0
    3.7828   -0.6763   -2.9336 H   0  0  0  0  0  0  0  0  0  0  0  0
    0.8166   -3.1806   -0.1266 C   0  0  0  0  0  0  0  0  0  0  0  0
    0.7076   -3.8585    0.7400 H   0  0  0  0  0  0  0  0  0  0  0  0
    1.2758   -1.7833    0.2084 C   0  0  0  0  0  0  0  0  0  0  0  0
    0.9006   -1.0676   -0.5772 H   0  0  0  0  0  0  0  0  0  0  0  0
    0.8312   -1.4784    1.1904 H   0  0  0  0  0  0  0  0  0  0  0  0
    2.0714   -0.4860   -2.4182 H   0  0  0  0  0  0  0  0  0  0  0  0
   -0.0889    2.9118   -1.0729 H   0  0  0  0  0  0  0  0  0  0  0  0
   -2.0380   -5.3904   -0.0597 C   0  0  0  0  0  0  0  0  0  0  0  0
   -2.6078   -6.6256    0.3819 C   0  0  0  0  0  0  0  0  0  0  0  0
   -2.2807   -7.1315    1.6531 C   0  0  0  0  0  0  0  0  0  0  0  0
   -3.4891   -7.3188   -0.4690 C   0  0  0  0  0  0  0  0  0  0  0  0
   -2.8427   -8.3366    2.0776 C   0  0  0  0  0  0  0  0  0  0  0  0
   -1.5950   -6.5881    2.3056 H   0  0  0  0  0  0  0  0  0  0  0  0
   -4.0484   -8.5237   -0.0439 C   0  0  0  0  0  0  0  0  0  0  0  0
   -3.7318   -6.9195   -1.4549 H   0  0  0  0  0  0  0  0  0  0  0  0
   -3.7219   -9.0188    1.2262 C   0  0  0  0  0  0  0  0  0  0  0  0
   -2.6061   -8.7539    3.0670 H   0  0  0  0  0  0  0  0  0  0  0  0
   -4.7385   -9.0873   -0.6879 H   0  0  0  0  0  0  0  0  0  0  0  0
   -1.5646   -4.3884   -0.4221 N   0  0  0  0  0  0  0  0  0  0  0  0
   -1.0422   -2.9313   -0.9937 Rh  0  0  0  0  0  0  0  0  0  0  0  0
   -4.9165   -2.6214   -1.2340 C   0  0  0  0  0  0  0  0  0  0  0  0
   -6.3429   -2.4632   -1.1779 C   0  0  0  0  0  0  0  0  0  0  0  0
   -7.1708   -3.3755   -1.8547 C   0  0  0  0  0  0  0  0  0  0  0  0
   -6.8891   -1.4040   -0.4317 C   0  0  0  0  0  0  0  0  0  0  0  0
   -8.5574   -3.2330   -1.7774 C   0  0  0  0  0  0  0  0  0  0  0  0
   -6.7365   -4.1900   -2.4382 H   0  0  0  0  0  0  0  0  0  0  0  0
   -8.2755   -1.2621   -0.3535 C   0  0  0  0  0  0  0  0  0  0  0  0
```

```
   -6.2362   -0.6964    0.0843 H   0  0  0  0  0  0  0  0  0  0  0  0
   -9.0935   -2.1801   -1.0246 C   0  0  0  0  0  0  0  0  0  0  0  0
   -9.2296   -3.9324   -2.2952 H   0  0  0  0  0  0  0  0  0  0  0  0
   -8.7305   -0.4457    0.2257 H   0  0  0  0  0  0  0  0  0  0  0  0
   -3.7659   -2.7529   -1.2639 N   0  0  0  0  0  0  0  0  0  0  0  0
   -4.3314  -10.3042    1.6826 N   0  0  0  0  0  0  0  0  0  0  0  0
  -10.5803   -2.0334   -0.9338 N   0  0  0  0  0  0  0  0  0  0  0  0
   -4.0124  -10.7027    2.7853 O   0  0  0  0  0  0  0  0  0  0  0  0
   -5.1015  -10.8474    0.9146 O   0  0  0  0  0  0  0  0  0  0  0  0
  -11.2484   -2.8553   -1.5291 O   0  0  0  0  0  0  0  0  0  0  0  0
  -10.9993   -1.1053   -0.2705 O   0  0  0  0  0  0  0  0  0  0  0  0
  1  2  1  0  0  0  0
  1 11  1  0  0  0  0
  1 17  2  0  0  0  0
  1 57  1  0  0  0  0
  2  4  1  0  0  0  0
  2  7  1  0  0  0  0
  2 12  1  0  0  0  0
  3  5  1  0  0  0  0
  3  9  1  0  0  0  0
  3 13  1  0  0  0  0
  3 19  1  0  0  0  0
  4  6  1  0  0  0  0
  4  8  1  0  0  0  0
  4 14  1  0  0  0  0
  5  6  1  0  0  0  0
  5 10  1  0  0  0  0
  5 15  1  0  0  0  0
  6 16  1  0  0  0  0
  6 44  1  0  0  0  0
 17 18  1  0  0  0  0
 17 19  1  0  0  0  0
 17 57  1  0  0  0  0
 19 20  1  0  0  0  0
 19 21  1  0  0  0  0
 22 23  1  0  0  0  0
 22 32  1  0  0  0  0
 22 38  2  0  0  0  0
 22 57  1  0  0  0  0
 23 25  1  0  0  0  0
 23 28  1  0  0  0  0
 23 33  1  0  0  0  0
 24 26  1  0  0  0  0
 24 30  1  0  0  0  0
 24 34  1  0  0  0  0
 24 40  1  0  0  0  0
 25 27  1  0  0  0  0
 25 29  1  0  0  0  0
 25 35  1  0  0  0  0
 26 27  1  0  0  0  0
 26 31  1  0  0  0  0
 26 36  1  0  0  0  0
 27 37  1  0  0  0  0
 27 43  1  0  0  0  0
 38 39  1  0  0  0  0
 38 40  1  0  0  0  0
 38 57  1  0  0  0  0
 40 41  1  0  0  0  0
 40 42  1  0  0  0  0
 45 46  1  0  0  0  0
 45 56  3  0  0  0  0
 46 47  2  0  0  0  0
 46 48  1  0  0  0  0
 47 49  1  0  0  0  0
 47 50  1  0  0  0  0
```

```
 48 51  2  0  0  0  0
 48 52  1  0  0  0  0
 49 53  2  0  0  0  0
 49 54  1  0  0  0  0
 51 53  1  0  0  0  0
 51 55  1  0  0  0  0
 53 70  1  0  0  0  0
 56 57  1  0  0  0  0
 57 69  1  0  0  0  0
 58 59  1  0  0  0  0
 58 69  3  0  0  0  0
 59 60  2  0  0  0  0
 59 61  1  0  0  0  0
 60 62  1  0  0  0  0
 60 63  1  0  0  0  0
 61 64  2  0  0  0  0
 61 65  1  0  0  0  0
 62 66  2  0  0  0  0
 62 67  1  0  0  0  0
 64 66  1  0  0  0  0
 64 68  1  0  0  0  0
 66 71  1  0  0  0  0
 70 72  2  0  0  0  0
 70 73  2  0  0  0  0
 71 74  2  0  0  0  0
 71 75  2  0  0  0  0
M  END
$$$$
mol124
Optimized Catalyst 124
Optimized Catalyst 124
 75 80  0  0  0  0  0  0  0  0999 V2000
   -2.6393    0.3019   -0.7508 C   0  0  0  0  0  0  0  0  0  0  0  0
   -1.6936    1.1806    0.0198 C   0  0  0  0  0  0  0  0  0  0  0  0
   -1.5941    1.6809   -3.4921 C   0  0  0  0  0  0  0  0  0  0  0  0
   -1.6314    2.6172   -0.5575 C   0  0  0  0  0  0  0  0  0  0  0  0
   -0.6842    2.8077   -2.9813 C   0  0  0  0  0  0  0  0  0  0  0  0
   -0.4195    2.8750   -1.4674 C   0  0  0  0  0  0  0  0  0  0  0  0
   -0.6640    0.7448    0.0247 H   0  0  0  0  0  0  0  0  0  0  0  0
   -2.5734    2.8426   -1.1103 H   0  0  0  0  0  0  0  0  0  0  0  0
   -2.6576    1.9524   -3.2810 H   0  0  0  0  0  0  0  0  0  0  0  0
   -1.1513    3.7759   -3.3005 H   0  0  0  0  0  0  0  0  0  0  0  0
   -3.6175    0.1477   -0.2485 H   0  0  0  0  0  0  0  0  0  0  0  0
   -2.0383    1.2497    1.0844 H   0  0  0  0  0  0  0  0  0  0  0  0
   -1.4773    1.6253   -4.6043 H   0  0  0  0  0  0  0  0  0  0  0  0
   -1.5708    3.3223    0.3108 H   0  0  0  0  0  0  0  0  0  0  0  0
    0.3083    2.7264   -3.4933 H   0  0  0  0  0  0  0  0  0  0  0  0
   -0.0441    3.9112   -1.2512 H   0  0  0  0  0  0  0  0  0  0  0  0
   -2.4484   -0.1396   -2.0248 C   0  0  0  0  0  0  0  0  0  0  0  0
   -3.2817   -0.6233   -2.5901 H   0  0  0  0  0  0  0  0  0  0  0  0
   -1.2934    0.2831   -2.8987 C   0  0  0  0  0  0  0  0  0  0  0  0
   -0.3423    0.3326   -2.3138 H   0  0  0  0  0  0  0  0  0  0  0  0
   -1.1597   -0.4453   -3.7368 H   0  0  0  0  0  0  0  0  0  0  0  0
    0.2933   -2.8325   -1.7306 C   0  0  0  0  0  0  0  0  0  0  0  0
    1.2127   -2.4541   -2.8550 C   0  0  0  0  0  0  0  0  0  0  0  0
    2.5957   -1.9332    0.3680 C   0  0  0  0  0  0  0  0  0  0  0  0
    2.6894   -2.8179   -2.5689 C   0  0  0  0  0  0  0  0  0  0  0  0
    3.7812   -1.5898   -0.5462 C   0  0  0  0  0  0  0  0  0  0  0  0
    3.5348   -1.6374   -2.0635 C   0  0  0  0  0  0  0  0  0  0  0  0
    1.1394   -1.3569   -3.0607 H   0  0  0  0  0  0  0  0  0  0  0  0
    2.7353   -3.6576   -1.8372 H   0  0  0  0  0  0  0  0  0  0  0  0
    2.4526   -3.0408    0.3737 H   0  0  0  0  0  0  0  0  0  0  0  0
    4.6070   -2.3064   -0.2996 H   0  0  0  0  0  0  0  0  0  0  0  0
   -0.3691   -3.6954   -1.9667 H   0  0  0  0  0  0  0  0  0  0  0  0
    0.8962   -2.9920   -3.7855 H   0  0  0  0  0  0  0  0  0  0  0  0
```

```
    2.8688   -1.6234    1.4085 H   0  0  0  0  0  0  0  0  0  0  0  0
    3.1372   -3.1805   -3.5293 H   0  0  0  0  0  0  0  0  0  0  0  0
    4.1407   -0.5597   -0.2951 H   0  0  0  0  0  0  0  0  0  0  0  0
    4.5418   -1.6993   -2.5553 H   0  0  0  0  0  0  0  0  0  0  0  0
    0.2795   -2.2795   -0.4971 C   0  0  0  0  0  0  0  0  0  0  0  0
   -0.3530   -2.7096    0.3283 H   0  0  0  0  0  0  0  0  0  0  0  0
    1.2627   -1.2466   -0.0127 C   0  0  0  0  0  0  0  0  0  0  0  0
    1.4552   -0.4809   -0.8033 H   0  0  0  0  0  0  0  0  0  0  0  0
    0.8598   -0.7265    0.8902 H   0  0  0  0  0  0  0  0  0  0  0  0
    3.0753   -0.6750   -2.3906 H   0  0  0  0  0  0  0  0  0  0  0  0
    0.4073    2.1734   -1.2071 H   0  0  0  0  0  0  0  0  0  0  0  0
   -2.3803   -5.3727   -0.0049 C   0  0  0  0  0  0  0  0  0  0  0  0
   -2.3831   -6.7590    0.3409 C   0  0  0  0  0  0  0  0  0  0  0  0
   -2.0061   -7.1553    1.6381 C   0  0  0  0  0  0  0  0  0  0  0  0
   -2.7623   -7.7078   -0.6293 C   0  0  0  0  0  0  0  0  0  0  0  0
   -2.0093   -8.5123    1.9664 C   0  0  0  0  0  0  0  0  0  0  0  0
   -1.7128   -6.4135    2.3855 H   0  0  0  0  0  0  0  0  0  0  0  0
   -2.7657   -9.0627   -0.2961 C   0  0  0  0  0  0  0  0  0  0  0  0
   -3.0495   -7.3923   -1.6355 H   0  0  0  0  0  0  0  0  0  0  0  0
   -2.3884   -9.4483    0.9967 C   0  0  0  0  0  0  0  0  0  0  0  0
   -1.7199   -8.8523    2.9729 H   0  0  0  0  0  0  0  0  0  0  0  0
   -3.0581   -9.8270   -1.0328 H   0  0  0  0  0  0  0  0  0  0  0  0
   -2.3665   -4.2423   -0.2991 N   0  0  0  0  0  0  0  0  0  0  0  0
   -5.5142   -2.4092   -0.7406 C   0  0  0  0  0  0  0  0  0  0  0  0
   -6.9413   -2.4359   -0.6830 C   0  0  0  0  0  0  0  0  0  0  0  0
   -7.6637   -3.1199   -1.6801 C   0  0  0  0  0  0  0  0  0  0  0  0
   -7.6018   -1.7739    0.3703 C   0  0  0  0  0  0  0  0  0  0  0  0
   -9.0578   -3.1401   -1.6212 C   0  0  0  0  0  0  0  0  0  0  0  0
   -7.1440   -3.6295   -2.4951 H   0  0  0  0  0  0  0  0  0  0  0  0
   -8.9964   -1.7963    0.4245 C   0  0  0  0  0  0  0  0  0  0  0  0
   -7.0342   -1.2454    1.1403 H   0  0  0  0  0  0  0  0  0  0  0  0
   -9.7066   -2.4783   -0.5710 C   0  0  0  0  0  0  0  0  0  0  0  0
   -9.6503   -3.6663   -2.3855 H   0  0  0  0  0  0  0  0  0  0  0  0
   -9.5410   -1.2862    1.2343 H   0  0  0  0  0  0  0  0  0  0  0  0
   -4.3477   -2.3694   -0.7897 N   0  0  0  0  0  0  0  0  0  0  0  0
   -2.4050   -2.3654   -0.8178 Ir  0  0  0  0  0  0  0  0  0  0  0  0
  -11.2076   -2.4993   -0.5129 N   0  0  0  0  0  0  0  0  0  0  0  0
   -2.3899  -10.9080    1.3513 N   0  0  0  0  0  0  0  0  0  0  0  0
   -2.6659  -11.6814    0.4577 O   0  0  0  0  0  0  0  0  0  0  0  0
  -11.7244   -1.8506    0.3728 O   0  0  0  0  0  0  0  0  0  0  0  0
  -11.7720   -3.1629   -1.3577 O   0  0  0  0  0  0  0  0  0  0  0  0
   -2.1154  -11.1866    2.5000 O   0  0  0  0  0  0  0  0  0  0  0  0
  1  2  1  0  0  0  0
  1 11  1  0  0  0  0
  1 17  2  0  0  0  0
  1 69  1  0  0  0  0
  2  4  1  0  0  0  0
  2  7  1  0  0  0  0
  2 12  1  0  0  0  0
  3  5  1  0  0  0  0
  3  9  1  0  0  0  0
  3 13  1  0  0  0  0
  3 19  1  0  0  0  0
  4  6  1  0  0  0  0
  4  8  1  0  0  0  0
  4 14  1  0  0  0  0
  5  6  1  0  0  0  0
  5 10  1  0  0  0  0
  5 15  1  0  0  0  0
  6 16  1  0  0  0  0
  6 44  1  0  0  0  0
 17 18  1  0  0  0  0
 17 19  1  0  0  0  0
 17 69  1  0  0  0  0
 19 20  1  0  0  0  0
```

```
 19 21  1  0  0  0  0
 22 23  1  0  0  0  0
 22 32  1  0  0  0  0
 22 38  2  0  0  0  0
 22 69  1  0  0  0  0
 23 25  1  0  0  0  0
 23 28  1  0  0  0  0
 23 33  1  0  0  0  0
 24 26  1  0  0  0  0
 24 30  1  0  0  0  0
 24 34  1  0  0  0  0
 24 40  1  0  0  0  0
 25 27  1  0  0  0  0
 25 29  1  0  0  0  0
 25 35  1  0  0  0  0
 26 27  1  0  0  0  0
 26 31  1  0  0  0  0
 26 36  1  0  0  0  0
 27 37  1  0  0  0  0
 27 43  1  0  0  0  0
 38 39  1  0  0  0  0
 38 40  1  0  0  0  0
 38 69  1  0  0  0  0
 40 41  1  0  0  0  0
 40 42  1  0  0  0  0
 45 46  1  0  0  0  0
 45 56  3  0  0  0  0
 46 47  2  0  0  0  0
 46 48  1  0  0  0  0
 47 49  1  0  0  0  0
 47 50  1  0  0  0  0
 48 51  2  0  0  0  0
 48 52  1  0  0  0  0
 49 53  2  0  0  0  0
 49 54  1  0  0  0  0
 51 53  1  0  0  0  0
 51 55  1  0  0  0  0
 53 71  1  0  0  0  0
 56 69  1  0  0  0  0
 57 58  1  0  0  0  0
 57 68  3  0  0  0  0
 58 59  2  0  0  0  0
 58 60  1  0  0  0  0
 59 61  1  0  0  0  0
 59 62  1  0  0  0  0
 60 63  2  0  0  0  0
 60 64  1  0  0  0  0
 61 65  2  0  0  0  0
 61 66  1  0  0  0  0
 63 65  1  0  0  0  0
 63 67  1  0  0  0  0
 65 70  1  0  0  0  0
 68 69  1  0  0  0  0
 70 73  2  0  0  0  0
 70 74  2  0  0  0  0
 71 72  2  0  0  0  0
 71 75  2  0  0  0  0
M  END
$$$$
mol125
Optimized Catalyst 125
Optimized Catalyst 125
 83 88  0  0  0  0  0  0  0  0999 V2000
   -1.6324   -0.2506   -0.6336 C   0  0  0  0  0  0  0  0  0  0  0  0
   -1.2286    0.9428    0.1863 C   0  0  0  0  0  0  0  0  0  0  0  0
```

```
   -1.8742    1.7090   -3.2380 C   0  0  0  0  0  0  0  0  0  0  0  0
   -1.9699    2.2397   -0.2099 C   0  0  0  0  0  0  0  0  0  0  0  0
   -1.5900    3.0967   -2.6417 C   0  0  0  0  0  0  0  0  0  0  0  0
   -1.1746    3.1503   -1.1611 C   0  0  0  0  0  0  0  0  0  0  0  0
   -0.1245    1.0987    0.0939 H   0  0  0  0  0  0  0  0  0  0  0  0
   -2.9501    1.9777   -0.6711 H   0  0  0  0  0  0  0  0  0  0  0  0
   -2.8860    1.3720   -2.9059 H   0  0  0  0  0  0  0  0  0  0  0  0
   -2.5168    3.7108   -2.7715 H   0  0  0  0  0  0  0  0  0  0  0  0
   -2.1508   -1.0500   -0.0502 H   0  0  0  0  0  0  0  0  0  0  0  0
   -1.4485    0.7310    1.2630 H   0  0  0  0  0  0  0  0  0  0  0  0
   -1.9022    1.8169   -4.3509 H   0  0  0  0  0  0  0  0  0  0  0  0
   -2.1756    2.8129    0.7281 H   0  0  0  0  0  0  0  0  0  0  0  0
   -0.7739    3.5789   -3.2366 H   0  0  0  0  0  0  0  0  0  0  0  0
   -1.3100    4.2099   -0.8215 H   0  0  0  0  0  0  0  0  0  0  0  0
   -1.4529   -0.4013   -1.9587 C   0  0  0  0  0  0  0  0  0  0  0  0
   -1.8549   -1.3037   -2.4822 H   0  0  0  0  0  0  0  0  0  0  0  0
   -0.8394    0.6214   -2.8748 C   0  0  0  0  0  0  0  0  0  0  0  0
    0.0469    1.0939   -2.3864 H   0  0  0  0  0  0  0  0  0  0  0  0
   -0.4896    0.1235   -3.8112 H   0  0  0  0  0  0  0  0  0  0  0  0
    0.8047   -3.7052   -1.4408 C   0  0  0  0  0  0  0  0  0  0  0  0
    1.2219   -2.9408   -2.6739 C   0  0  0  0  0  0  0  0  0  0  0  0
    2.7995   -1.7274    0.2831 C   0  0  0  0  0  0  0  0  0  0  0  0
    2.7186   -2.5529   -2.6830 C   0  0  0  0  0  0  0  0  0  0  0  0
    3.4896   -0.9036   -0.8154 C   0  0  0  0  0  0  0  0  0  0  0  0
    2.9946   -1.1003   -2.2591 C   0  0  0  0  0  0  0  0  0  0  0  0
    0.5743   -2.0152   -2.7581 H   0  0  0  0  0  0  0  0  0  0  0  0
    3.2865   -3.2530   -2.0255 H   0  0  0  0  0  0  0  0  0  0  0  0
    3.1982   -2.7715    0.2560 H   0  0  0  0  0  0  0  0  0  0  0  0
    4.5813   -1.1538   -0.7795 H   0  0  0  0  0  0  0  0  0  0  0  0
    0.6979   -4.7966   -1.5806 H   0  0  0  0  0  0  0  0  0  0  0  0
    1.0114   -3.5753   -3.5742 H   0  0  0  0  0  0  0  0  0  0  0  0
    3.0841   -1.2852    1.2710 H   0  0  0  0  0  0  0  0  0  0  0  0
    3.0965   -2.6888   -3.7278 H   0  0  0  0  0  0  0  0  0  0  0  0
    3.3811    0.1826   -0.5681 H   0  0  0  0  0  0  0  0  0  0  0  0
    3.7896   -0.6880   -2.9349 H   0  0  0  0  0  0  0  0  0  0  0  0
    0.7902   -3.1683   -0.1381 C   0  0  0  0  0  0  0  0  0  0  0  0
    0.6667   -3.8408    0.7307 H   0  0  0  0  0  0  0  0  0  0  0  0
    1.2588   -1.7729    0.1923 C   0  0  0  0  0  0  0  0  0  0  0  0
    0.8935   -1.0593   -0.5997 H   0  0  0  0  0  0  0  0  0  0  0  0
    0.8100   -1.4577    1.1686 H   0  0  0  0  0  0  0  0  0  0  0  0
    2.0797   -0.4823   -2.4196 H   0  0  0  0  0  0  0  0  0  0  0  0
   -0.0865    2.9187   -1.0806 H   0  0  0  0  0  0  0  0  0  0  0  0
   -2.1186   -5.3885   -0.0687 C   0  0  0  0  0  0  0  0  0  0  0  0
   -1.5918   -4.4159   -0.4234 N   0  0  0  0  0  0  0  0  0  0  0  0
   -1.0742   -2.9332   -1.0097 Rh  0  0  0  0  0  0  0  0  0  0  0  0
   -4.8484   -2.6613   -1.2078 C   0  0  0  0  0  0  0  0  0  0  0  0
   -3.6951   -2.7563   -1.2438 N   0  0  0  0  0  0  0  0  0  0  0  0
   -6.3066   -2.5328   -1.1536 C   0  0  0  0  0  0  0  0  0  0  0  0
   -6.9583   -3.9246   -0.9860 C   0  0  0  0  0  0  0  0  0  0  0  0
   -6.7015   -1.5849    0.0027 C   0  0  0  0  0  0  0  0  0  0  0  0
   -6.6548   -2.0841   -2.1273 H   0  0  0  0  0  0  0  0  0  0  0  0
   -8.4865   -3.7857   -0.8987 C   0  0  0  0  0  0  0  0  0  0  0  0
   -6.5788   -4.4043   -0.0530 H   0  0  0  0  0  0  0  0  0  0  0  0
   -6.6941   -4.5685   -1.8572 H   0  0  0  0  0  0  0  0  0  0  0  0
   -8.2297   -1.4543    0.0878 C   0  0  0  0  0  0  0  0  0  0  0  0
   -6.3152   -1.9944    0.9661 H   0  0  0  0  0  0  0  0  0  0  0  0
   -6.2512   -0.5791   -0.1685 H   0  0  0  0  0  0  0  0  0  0  0  0
   -8.9060   -2.8269    0.2267 C   0  0  0  0  0  0  0  0  0  0  0  0
   -8.9325   -4.7918   -0.7077 H   0  0  0  0  0  0  0  0  0  0  0  0
   -8.8821   -3.4072   -1.8719 H   0  0  0  0  0  0  0  0  0  0  0  0
   -8.4905   -0.8257    0.9743 H   0  0  0  0  0  0  0  0  0  0  0  0
   -8.6129   -0.9428   -0.8275 H   0  0  0  0  0  0  0  0  0  0  0  0
  -10.0149   -2.6920    0.1975 H   0  0  0  0  0  0  0  0  0  0  0  0
   -8.6343   -3.2736    1.2126 H   0  0  0  0  0  0  0  0  0  0  0  0
   -2.7555   -6.6306    0.3829 C   0  0  0  0  0  0  0  0  0  0  0  0
```

```
   -3.5766   -6.3721    1.6673 C   0  0  0  0  0  0  0  0  0  0  0  0
   -3.6457   -7.2036   -0.7447 C   0  0  0  0  0  0  0  0  0  0  0  0
   -1.9374   -7.3705    0.6149 H   0  0  0  0  0  0  0  0  0  0  0  0
   -4.2538   -7.6724    2.1290 C   0  0  0  0  0  0  0  0  0  0  0  0
   -4.3574   -5.6025    1.4611 H   0  0  0  0  0  0  0  0  0  0  0  0
   -2.9045   -5.9967    2.4742 H   0  0  0  0  0  0  0  0  0  0  0  0
   -4.3211   -8.5011   -0.2735 C   0  0  0  0  0  0  0  0  0  0  0  0
   -4.4287   -6.4563   -1.0158 H   0  0  0  0  0  0  0  0  0  0  0  0
   -3.0224   -7.4148   -1.6451 H   0  0  0  0  0  0  0  0  0  0  0  0
   -5.1232   -8.2833    1.0189 C   0  0  0  0  0  0  0  0  0  0  0  0
   -4.8933   -7.4530    3.0185 H   0  0  0  0  0  0  0  0  0  0  0  0
   -3.4729   -8.4103    2.4338 H   0  0  0  0  0  0  0  0  0  0  0  0
   -5.0074   -8.8658   -1.0762 H   0  0  0  0  0  0  0  0  0  0  0  0
   -3.5432   -9.2829   -0.0978 H   0  0  0  0  0  0  0  0  0  0  0  0
   -5.5252   -9.2657    1.3690 H   0  0  0  0  0  0  0  0  0  0  0  0
   -5.9856   -7.6072    0.8097 H   0  0  0  0  0  0  0  0  0  0  0  0
  1  2  1  0  0  0  0
  1 11  1  0  0  0  0
  1 17  2  0  0  0  0
  1 47  1  0  0  0  0
  2  4  1  0  0  0  0
  2  7  1  0  0  0  0
  2 12  1  0  0  0  0
  3  5  1  0  0  0  0
  3  9  1  0  0  0  0
  3 13  1  0  0  0  0
  3 19  1  0  0  0  0
  4  6  1  0  0  0  0
  4  8  1  0  0  0  0
  4 14  1  0  0  0  0
  5  6  1  0  0  0  0
  5 10  1  0  0  0  0
  5 15  1  0  0  0  0
  6 16  1  0  0  0  0
  6 44  1  0  0  0  0
 17 18  1  0  0  0  0
 17 19  1  0  0  0  0
 17 47  1  0  0  0  0
 19 20  1  0  0  0  0
 19 21  1  0  0  0  0
 22 23  1  0  0  0  0
 22 32  1  0  0  0  0
 22 38  2  0  0  0  0
 22 47  1  0  0  0  0
 23 25  1  0  0  0  0
 23 28  1  0  0  0  0
 23 33  1  0  0  0  0
 24 26  1  0  0  0  0
 24 30  1  0  0  0  0
 24 34  1  0  0  0  0
 24 40  1  0  0  0  0
 25 27  1  0  0  0  0
 25 29  1  0  0  0  0
 25 35  1  0  0  0  0
 26 27  1  0  0  0  0
 26 31  1  0  0  0  0
 26 36  1  0  0  0  0
 27 37  1  0  0  0  0
 27 43  1  0  0  0  0
 38 39  1  0  0  0  0
 38 40  1  0  0  0  0
 38 47  1  0  0  0  0
 40 41  1  0  0  0  0
 40 42  1  0  0  0  0
 45 46  3  0  0  0  0
```

```
 45 67  1  0  0  0  0
 46 47  1  0  0  0  0
 47 49  1  0  0  0  0
 48 49  3  0  0  0  0
 48 50  1  0  0  0  0
 50 51  1  0  0  0  0
 50 52  1  0  0  0  0
 50 53  1  0  0  0  0
 51 54  1  0  0  0  0
 51 55  1  0  0  0  0
 51 56  1  0  0  0  0
 52 57  1  0  0  0  0
 52 58  1  0  0  0  0
 52 59  1  0  0  0  0
 54 60  1  0  0  0  0
 54 61  1  0  0  0  0
 54 62  1  0  0  0  0
 57 60  1  0  0  0  0
 57 63  1  0  0  0  0
 57 64  1  0  0  0  0
 60 65  1  0  0  0  0
 60 66  1  0  0  0  0
 67 68  1  0  0  0  0
 67 69  1  0  0  0  0
 67 70  1  0  0  0  0
 68 71  1  0  0  0  0
 68 72  1  0  0  0  0
 68 73  1  0  0  0  0
 69 74  1  0  0  0  0
 69 75  1  0  0  0  0
 69 76  1  0  0  0  0
 71 77  1  0  0  0  0
 71 78  1  0  0  0  0
 71 79  1  0  0  0  0
 74 77  1  0  0  0  0
 74 80  1  0  0  0  0
 74 81  1  0  0  0  0
 77 82  1  0  0  0  0
 77 83  1  0  0  0  0
M  END
$$$$
mol126
Optimized Catalyst 126
Optimized Catalyst 126
 83 88  0  0  0  0  0  0  0  0999 V2000
   -2.6485    0.2867   -0.7574 C   0  0  0  0  0  0  0  0  0  0  0  0
   -1.7077    1.1703    0.0150 C   0  0  0  0  0  0  0  0  0  0  0  0
   -1.6034    1.6831   -3.4963 C   0  0  0  0  0  0  0  0  0  0  0  0
   -1.6479    2.6064   -0.5627 C   0  0  0  0  0  0  0  0  0  0  0  0
   -0.6912    2.8062   -2.9803 C   0  0  0  0  0  0  0  0  0  0  0  0
   -0.4308    2.8666   -1.4653 C   0  0  0  0  0  0  0  0  0  0  0  0
   -0.6775    0.7358    0.0231 H   0  0  0  0  0  0  0  0  0  0  0  0
   -2.5872    2.8260   -1.1224 H   0  0  0  0  0  0  0  0  0  0  0  0
   -2.6663    1.9554   -3.2838 H   0  0  0  0  0  0  0  0  0  0  0  0
   -1.1545    3.7769   -3.2966 H   0  0  0  0  0  0  0  0  0  0  0  0
   -3.6239    0.1211   -0.2520 H   0  0  0  0  0  0  0  0  0  0  0  0
   -2.0554    1.2384    1.0786 H   0  0  0  0  0  0  0  0  0  0  0  0
   -1.4862    1.6324   -4.6085 H   0  0  0  0  0  0  0  0  0  0  0  0
   -1.5968    3.3136    0.3043 H   0  0  0  0  0  0  0  0  0  0  0  0
    0.3021    2.7234   -3.4898 H   0  0  0  0  0  0  0  0  0  0  0  0
   -0.0532    3.9002   -1.2423 H   0  0  0  0  0  0  0  0  0  0  0  0
   -2.4584   -0.1442   -2.0346 C   0  0  0  0  0  0  0  0  0  0  0  0
   -3.2870   -0.6393   -2.5964 H   0  0  0  0  0  0  0  0  0  0  0  0
   -1.3057    0.2824   -2.9091 C   0  0  0  0  0  0  0  0  0  0  0  0
   -0.3532    0.3261   -2.3260 H   0  0  0  0  0  0  0  0  0  0  0  0
```

```
   -1.1751   -0.4427   -3.7505 H   0  0  0  0  0  0  0  0  0  0  0  0
    0.2854   -2.8453   -1.7211 C   0  0  0  0  0  0  0  0  0  0  0  0
    1.2038   -2.4669   -2.8464 C   0  0  0  0  0  0  0  0  0  0  0  0
    2.5911   -1.9430    0.3726 C   0  0  0  0  0  0  0  0  0  0  0  0
    2.6818   -2.8264   -2.5627 C   0  0  0  0  0  0  0  0  0  0  0  0
    3.7738   -1.5939   -0.5431 C   0  0  0  0  0  0  0  0  0  0  0  0
    3.5256   -1.6432   -2.0603 C   0  0  0  0  0  0  0  0  0  0  0  0
    1.1265   -1.3702   -3.0529 H   0  0  0  0  0  0  0  0  0  0  0  0
    2.7306   -3.6642   -1.8291 H   0  0  0  0  0  0  0  0  0  0  0  0
    2.4504   -3.0509    0.3762 H   0  0  0  0  0  0  0  0  0  0  0  0
    4.6034   -2.3060   -0.2976 H   0  0  0  0  0  0  0  0  0  0  0  0
   -0.3812   -3.7053   -1.9578 H   0  0  0  0  0  0  0  0  0  0  0  0
    0.8868   -3.0065   -3.7757 H   0  0  0  0  0  0  0  0  0  0  0  0
    2.8651   -1.6348    1.4132 H   0  0  0  0  0  0  0  0  0  0  0  0
    3.1287   -3.1895   -3.5229 H   0  0  0  0  0  0  0  0  0  0  0  0
    4.1271   -0.5615   -0.2926 H   0  0  0  0  0  0  0  0  0  0  0  0
    4.5316   -1.7028   -2.5538 H   0  0  0  0  0  0  0  0  0  0  0  0
    0.2733   -2.2930   -0.4876 C   0  0  0  0  0  0  0  0  0  0  0  0
   -0.3633   -2.7204    0.3370 H   0  0  0  0  0  0  0  0  0  0  0  0
    1.2556   -1.2594   -0.0040 C   0  0  0  0  0  0  0  0  0  0  0  0
    1.4432   -0.4918   -0.7940 H   0  0  0  0  0  0  0  0  0  0  0  0
    0.8532   -0.7415    0.9006 H   0  0  0  0  0  0  0  0  0  0  0  0
    3.0626   -0.6823   -2.3870 H   0  0  0  0  0  0  0  0  0  0  0  0
    0.3925    2.1605   -1.2052 H   0  0  0  0  0  0  0  0  0  0  0  0
   -2.3870   -5.3935    0.0147 C   0  0  0  0  0  0  0  0  0  0  0  0
   -2.3752   -4.2666   -0.2830 N   0  0  0  0  0  0  0  0  0  0  0  0
   -5.5347   -2.4231   -0.7395 C   0  0  0  0  0  0  0  0  0  0  0  0
   -4.3707   -2.3837   -0.7841 N   0  0  0  0  0  0  0  0  0  0  0  0
   -2.4149   -2.3775   -0.8037 Ir  0  0  0  0  0  0  0  0  0  0  0  0
   -2.3894   -6.8123    0.3816 C   0  0  0  0  0  0  0  0  0  0  0  0
   -1.6128   -7.6286   -0.6793 C   0  0  0  0  0  0  0  0  0  0  0  0
   -1.7881   -6.9954    1.7953 C   0  0  0  0  0  0  0  0  0  0  0  0
   -3.4636   -7.1618    0.3950 H   0  0  0  0  0  0  0  0  0  0  0  0
   -1.5954   -9.1151   -0.2877 C   0  0  0  0  0  0  0  0  0  0  0  0
   -0.5621   -7.2571   -0.7431 H   0  0  0  0  0  0  0  0  0  0  0  0
   -2.1013   -7.5173   -1.6761 H   0  0  0  0  0  0  0  0  0  0  0  0
   -1.7689   -8.4868    2.1678 C   0  0  0  0  0  0  0  0  0  0  0  0
   -0.7433   -6.6026    1.8123 H   0  0  0  0  0  0  0  0  0  0  0  0
   -2.4005   -6.4373    2.5424 H   0  0  0  0  0  0  0  0  0  0  0  0
   -1.0180   -9.3219    1.1202 C   0  0  0  0  0  0  0  0  0  0  0  0
   -0.9707   -9.6757   -1.0268 H   0  0  0  0  0  0  0  0  0  0  0  0
   -2.6334   -9.5250   -0.3270 H   0  0  0  0  0  0  0  0  0  0  0  0
   -1.2660   -8.6037    3.1600 H   0  0  0  0  0  0  0  0  0  0  0  0
   -2.8160   -8.8631    2.2618 H   0  0  0  0  0  0  0  0  0  0  0  0
   -1.0960  -10.4037    1.3915 H   0  0  0  0  0  0  0  0  0  0  0  0
    0.0624   -9.0402    1.1251 H   0  0  0  0  0  0  0  0  0  0  0  0
   -6.9988   -2.4533   -0.6827 C   0  0  0  0  0  0  0  0  0  0  0  0
   -7.5034   -1.4234    0.3564 C   0  0  0  0  0  0  0  0  0  0  0  0
   -7.5879   -2.1834   -2.0876 C   0  0  0  0  0  0  0  0  0  0  0  0
   -7.3084   -3.4877   -0.3505 H   0  0  0  0  0  0  0  0  0  0  0  0
   -9.0400   -1.4345    0.4030 C   0  0  0  0  0  0  0  0  0  0  0  0
   -7.1561   -0.4018    0.0712 H   0  0  0  0  0  0  0  0  0  0  0  0
   -7.1035   -1.6774    1.3665 H   0  0  0  0  0  0  0  0  0  0  0  0
   -9.1238   -2.1888   -2.0216 C   0  0  0  0  0  0  0  0  0  0  0  0
   -7.2431   -1.1872   -2.4545 H   0  0  0  0  0  0  0  0  0  0  0  0
   -7.2478   -2.9725   -2.7992 H   0  0  0  0  0  0  0  0  0  0  0  0
   -9.6490   -1.1852   -0.9846 C   0  0  0  0  0  0  0  0  0  0  0  0
   -9.3851   -0.6348    1.1047 H   0  0  0  0  0  0  0  0  0  0  0  0
   -9.3960   -2.4180    0.7940 H   0  0  0  0  0  0  0  0  0  0  0  0
   -9.5288   -1.9214   -3.0292 H   0  0  0  0  0  0  0  0  0  0  0  0
   -9.4837   -3.2135   -1.7621 H   0  0  0  0  0  0  0  0  0  0  0  0
  -10.7611   -1.2782   -0.9171 H   0  0  0  0  0  0  0  0  0  0  0  0
   -9.4073   -0.1467   -1.3160 H   0  0  0  0  0  0  0  0  0  0  0  0
  1  2  1  0  0  0  0
  1 11  1  0  0  0  0
```

```
  1 17  2  0  0  0  0
  1 49  1  0  0  0  0
  2  4  1  0  0  0  0
  2  7  1  0  0  0  0
  2 12  1  0  0  0  0
  3  5  1  0  0  0  0
  3  9  1  0  0  0  0
  3 13  1  0  0  0  0
  3 19  1  0  0  0  0
  4  6  1  0  0  0  0
  4  8  1  0  0  0  0
  4 14  1  0  0  0  0
  5  6  1  0  0  0  0
  5 10  1  0  0  0  0
  5 15  1  0  0  0  0
  6 16  1  0  0  0  0
  6 44  1  0  0  0  0
 17 18  1  0  0  0  0
 17 19  1  0  0  0  0
 17 49  1  0  0  0  0
 19 20  1  0  0  0  0
 19 21  1  0  0  0  0
 22 23  1  0  0  0  0
 22 32  1  0  0  0  0
 22 38  2  0  0  0  0
 22 49  1  0  0  0  0
 23 25  1  0  0  0  0
 23 28  1  0  0  0  0
 23 33  1  0  0  0  0
 24 26  1  0  0  0  0
 24 30  1  0  0  0  0
 24 34  1  0  0  0  0
 24 40  1  0  0  0  0
 25 27  1  0  0  0  0
 25 29  1  0  0  0  0
 25 35  1  0  0  0  0
 26 27  1  0  0  0  0
 26 31  1  0  0  0  0
 26 36  1  0  0  0  0
 27 37  1  0  0  0  0
 27 43  1  0  0  0  0
 38 39  1  0  0  0  0
 38 40  1  0  0  0  0
 38 49  1  0  0  0  0
 40 41  1  0  0  0  0
 40 42  1  0  0  0  0
 45 46  3  0  0  0  0
 45 50  1  0  0  0  0
 46 49  1  0  0  0  0
 47 48  3  0  0  0  0
 47 67  1  0  0  0  0
 48 49  1  0  0  0  0
 50 51  1  0  0  0  0
 50 52  1  0  0  0  0
 50 53  1  0  0  0  0
 51 54  1  0  0  0  0
 51 55  1  0  0  0  0
 51 56  1  0  0  0  0
 52 57  1  0  0  0  0
 52 58  1  0  0  0  0
 52 59  1  0  0  0  0
 54 60  1  0  0  0  0
 54 61  1  0  0  0  0
 54 62  1  0  0  0  0
 57 60  1  0  0  0  0
```

```
 57 63  1  0  0  0  0
 57 64  1  0  0  0  0
 60 65  1  0  0  0  0
 60 66  1  0  0  0  0
 67 68  1  0  0  0  0
 67 69  1  0  0  0  0
 67 70  1  0  0  0  0
 68 71  1  0  0  0  0
 68 72  1  0  0  0  0
 68 73  1  0  0  0  0
 69 74  1  0  0  0  0
 69 75  1  0  0  0  0
 69 76  1  0  0  0  0
 71 77  1  0  0  0  0
 71 78  1  0  0  0  0
 71 79  1  0  0  0  0
 74 77  1  0  0  0  0
 74 80  1  0  0  0  0
 74 81  1  0  0  0  0
 77 82  1  0  0  0  0
 77 83  1  0  0  0  0
M  END
$$$$
mol127
Optimized Catalyst 127
Optimized Catalyst 127
 89 94  0  0  0  0  0  0  0  0999 V2000
   -1.6325   -0.2534   -0.6385 C   0  0  0  0  0  0  0  0  0  0  0  0
   -1.2362    0.9449    0.1782 C   0  0  0  0  0  0  0  0  0  0  0  0
   -1.8530    1.7001   -3.2463 C   0  0  0  0  0  0  0  0  0  0  0  0
   -1.9760    2.2400   -0.2266 C   0  0  0  0  0  0  0  0  0  0  0  0
   -1.5663    3.0900   -2.6566 C   0  0  0  0  0  0  0  0  0  0  0  0
   -1.1729    3.1518   -1.1702 C   0  0  0  0  0  0  0  0  0  0  0  0
   -0.1318    1.1021    0.0908 H   0  0  0  0  0  0  0  0  0  0  0  0
   -2.9513    1.9766   -0.6970 H   0  0  0  0  0  0  0  0  0  0  0  0
   -2.8664    1.3678   -2.9146 H   0  0  0  0  0  0  0  0  0  0  0  0
   -2.4865    3.7100   -2.8048 H   0  0  0  0  0  0  0  0  0  0  0  0
   -2.1483   -1.0536   -0.0545 H   0  0  0  0  0  0  0  0  0  0  0  0
   -1.4615    0.7373    1.2545 H   0  0  0  0  0  0  0  0  0  0  0  0
   -1.8776    1.8018   -4.3597 H   0  0  0  0  0  0  0  0  0  0  0  0
   -2.1917    2.8137    0.7090 H   0  0  0  0  0  0  0  0  0  0  0  0
   -0.7379    3.5614   -3.2432 H   0  0  0  0  0  0  0  0  0  0  0  0
   -1.3198    4.2119   -0.8369 H   0  0  0  0  0  0  0  0  0  0  0  0
   -1.4428   -0.4092   -1.9612 C   0  0  0  0  0  0  0  0  0  0  0  0
   -1.8373   -1.3151   -2.4845 H   0  0  0  0  0  0  0  0  0  0  0  0
   -0.8222    0.6121   -2.8740 C   0  0  0  0  0  0  0  0  0  0  0  0
    0.0600    1.0847   -2.3778 H   0  0  0  0  0  0  0  0  0  0  0  0
   -0.4646    0.1139   -3.8073 H   0  0  0  0  0  0  0  0  0  0  0  0
    0.7992   -3.7162   -1.4370 C   0  0  0  0  0  0  0  0  0  0  0  0
    1.2156   -2.9492   -2.6686 C   0  0  0  0  0  0  0  0  0  0  0  0
    2.7676   -1.7155    0.2900 C   0  0  0  0  0  0  0  0  0  0  0  0
    2.7079   -2.5450   -2.6708 C   0  0  0  0  0  0  0  0  0  0  0  0
    3.4435   -0.8750   -0.8044 C   0  0  0  0  0  0  0  0  0  0  0  0
    2.9662   -1.0876   -2.2522 C   0  0  0  0  0  0  0  0  0  0  0  0
    0.5589   -2.0303   -2.7572 H   0  0  0  0  0  0  0  0  0  0  0  0
    3.2797   -3.2361   -2.0072 H   0  0  0  0  0  0  0  0  0  0  0  0
    3.1803   -2.7539    0.2564 H   0  0  0  0  0  0  0  0  0  0  0  0
    4.5413   -1.0947   -0.7588 H   0  0  0  0  0  0  0  0  0  0  0  0
    0.6999   -4.8082   -1.5769 H   0  0  0  0  0  0  0  0  0  0  0  0
    1.0162   -3.5870   -3.5692 H   0  0  0  0  0  0  0  0  0  0  0  0
    3.0463   -1.2748    1.2803 H   0  0  0  0  0  0  0  0  0  0  0  0
    3.0922   -2.6809   -3.7134 H   0  0  0  0  0  0  0  0  0  0  0  0
    3.3025    0.2082   -0.5600 H   0  0  0  0  0  0  0  0  0  0  0  0
    3.7647   -0.6726   -2.9222 H   0  0  0  0  0  0  0  0  0  0  0  0
    0.7749   -3.1798   -0.1340 C   0  0  0  0  0  0  0  0  0  0  0  0
```

```
    0.6517   -3.8534    0.7339 H   0  0  0  0  0  0  0  0  0  0  0  0
    1.2280   -1.7802    0.1988 C   0  0  0  0  0  0  0  0  0  0  0  0
    0.8565   -1.0700   -0.5939 H   0  0  0  0  0  0  0  0  0  0  0  0
    0.7739   -1.4710    1.1746 H   0  0  0  0  0  0  0  0  0  0  0  0
    2.0475   -0.4793   -2.4269 H   0  0  0  0  0  0  0  0  0  0  0  0
   -0.0845    2.9274   -1.0727 H   0  0  0  0  0  0  0  0  0  0  0  0
   -2.1013   -5.4184   -0.0739 C   0  0  0  0  0  0  0  0  0  0  0  0
   -1.5969   -4.4354   -0.4316 N   0  0  0  0  0  0  0  0  0  0  0  0
   -1.0853   -2.9510   -1.0157 Rh  0  0  0  0  0  0  0  0  0  0  0  0
   -4.8574   -2.6401   -1.2119 C   0  0  0  0  0  0  0  0  0  0  0  0
   -3.7060   -2.7579   -1.2387 N   0  0  0  0  0  0  0  0  0  0  0  0
   -6.3142   -2.4889   -1.1660 C   0  0  1  0  0  0  0  0  0  0  0  0
   -6.9831   -3.8751   -1.0207 C   0  0  0  0  0  0  0  0  0  0  0  0
   -6.7026   -1.5486   -0.0014 C   0  0  0  0  0  0  0  0  0  0  0  0
   -6.6508   -2.0234   -2.1358 H   0  0  0  0  0  0  0  0  0  0  0  0
   -8.5074   -3.7241   -0.9142 C   0  0  0  0  0  0  0  0  0  0  0  0
   -6.5965   -4.3816   -0.1047 H   0  0  0  0  0  0  0  0  0  0  0  0
   -6.7361   -4.5009   -1.9106 H   0  0  0  0  0  0  0  0  0  0  0  0
   -8.2285   -1.4178    0.0975 C   0  0  0  0  0  0  0  0  0  0  0  0
   -6.3047   -1.9596    0.9569 H   0  0  0  0  0  0  0  0  0  0  0  0
   -6.2558   -0.5408   -0.1739 H   0  0  0  0  0  0  0  0  0  0  0  0
   -8.9042   -2.7975    0.2564 C   0  0  1  0  0  0  0  0  0  0  0  0
   -8.9617   -4.7310   -0.7559 H   0  0  0  0  0  0  0  0  0  0  0  0
   -8.9104   -3.3068   -1.8672 H   0  0  0  0  0  0  0  0  0  0  0  0
   -8.4812   -0.7814    0.9793 H   0  0  0  0  0  0  0  0  0  0  0  0
   -8.6230   -0.9161   -0.8174 H   0  0  0  0  0  0  0  0  0  0  0  0
   -8.5441   -3.2595    1.2100 H   0  0  0  0  0  0  0  0  0  0  0  0
   -2.7158   -6.6699    0.3825 C   0  0  1  0  0  0  0  0  0  0  0  0
   -3.5340   -6.4177    1.6697 C   0  0  0  0  0  0  0  0  0  0  0  0
   -3.6005   -7.2572   -0.7416 C   0  0  0  0  0  0  0  0  0  0  0  0
   -1.8859   -7.3969    0.6127 H   0  0  0  0  0  0  0  0  0  0  0  0
   -4.2169   -7.7140    2.1284 C   0  0  0  0  0  0  0  0  0  0  0  0
   -4.3091   -5.6401    1.4717 H   0  0  0  0  0  0  0  0  0  0  0  0
   -2.8562   -6.0523    2.4767 H   0  0  0  0  0  0  0  0  0  0  0  0
   -4.2822   -8.5467   -0.2630 C   0  0  0  0  0  0  0  0  0  0  0  0
   -4.3780   -6.5111   -1.0312 H   0  0  0  0  0  0  0  0  0  0  0  0
   -2.9696   -7.4824   -1.6335 H   0  0  0  0  0  0  0  0  0  0  0  0
   -5.1123   -8.3046    1.0169 C   0  0  1  0  0  0  0  0  0  0  0  0
   -4.8418   -7.4964    3.0273 H   0  0  0  0  0  0  0  0  0  0  0  0
   -3.4435   -8.4646    2.4173 H   0  0  0  0  0  0  0  0  0  0  0  0
   -4.9534   -8.9239   -1.0712 H   0  0  0  0  0  0  0  0  0  0  0  0
   -3.5110   -9.3274   -0.0610 H   0  0  0  0  0  0  0  0  0  0  0  0
   -5.9174   -7.5639    0.7809 H   0  0  0  0  0  0  0  0  0  0  0  0
  -10.4281   -2.6475    0.3367 C   0  0  0  0  0  0  0  0  0  0  0  0
  -10.9063   -3.6431    0.4757 H   0  0  0  0  0  0  0  0  0  0  0  0
  -10.7057   -2.0012    1.1999 H   0  0  0  0  0  0  0  0  0  0  0  0
  -10.8310   -2.1880   -0.5927 H   0  0  0  0  0  0  0  0  0  0  0  0
   -5.7739   -9.6052    1.4877 C   0  0  0  0  0  0  0  0  0  0  0  0
   -6.3913   -9.4167    2.3949 H   0  0  0  0  0  0  0  0  0  0  0  0
   -6.4367  -10.0105    0.6900 H   0  0  0  0  0  0  0  0  0  0  0  0
   -5.0079  -10.3735    1.7347 H   0  0  0  0  0  0  0  0  0  0  0  0
  1  2  1  0  0  0  0
  1 11  1  0  0  0  0
  1 17  2  0  0  0  0
  1 47  1  0  0  0  0
  2  4  1  0  0  0  0
  2  7  1  0  0  0  0
  2 12  1  0  0  0  0
  3  5  1  0  0  0  0
  3  9  1  0  0  0  0
  3 13  1  0  0  0  0
  3 19  1  0  0  0  0
  4  6  1  0  0  0  0
  4  8  1  0  0  0  0
  4 14  1  0  0  0  0
```

```
  5  6  1  0  0  0  0
  5 10  1  0  0  0  0
  5 15  1  0  0  0  0
  6 16  1  0  0  0  0
  6 44  1  0  0  0  0
 17 18  1  0  0  0  0
 17 19  1  0  0  0  0
 17 47  1  0  0  0  0
 19 20  1  0  0  0  0
 19 21  1  0  0  0  0
 22 23  1  0  0  0  0
 22 32  1  0  0  0  0
 22 38  2  0  0  0  0
 22 47  1  0  0  0  0
 23 25  1  0  0  0  0
 23 28  1  0  0  0  0
 23 33  1  0  0  0  0
 24 26  1  0  0  0  0
 24 30  1  0  0  0  0
 24 34  1  0  0  0  0
 24 40  1  0  0  0  0
 25 27  1  0  0  0  0
 25 29  1  0  0  0  0
 25 35  1  0  0  0  0
 26 27  1  0  0  0  0
 26 31  1  0  0  0  0
 26 36  1  0  0  0  0
 27 37  1  0  0  0  0
 27 43  1  0  0  0  0
 38 39  1  0  0  0  0
 38 40  1  0  0  0  0
 38 47  1  0  0  0  0
 40 41  1  0  0  0  0
 40 42  1  0  0  0  0
 45 46  3  0  0  0  0
 45 66  1  0  0  0  0
 46 47  1  0  0  0  0
 47 49  1  0  0  0  0
 48 49  3  0  0  0  0
 48 50  1  0  0  0  0
 50 51  1  0  0  0  0
 50 52  1  0  0  0  0
 50 53  1  6  0  0  0
 51 54  1  0  0  0  0
 51 55  1  0  0  0  0
 51 56  1  0  0  0  0
 52 57  1  0  0  0  0
 52 58  1  0  0  0  0
 52 59  1  0  0  0  0
 54 60  1  0  0  0  0
 54 61  1  0  0  0  0
 54 62  1  0  0  0  0
 57 60  1  0  0  0  0
 57 63  1  0  0  0  0
 57 64  1  0  0  0  0
 60 65  1  1  0  0  0
 60 82  1  0  0  0  0
 66 67  1  0  0  0  0
 66 68  1  0  0  0  0
 66 69  1  1  0  0  0
 67 70  1  0  0  0  0
 67 71  1  0  0  0  0
 67 72  1  0  0  0  0
 68 73  1  0  0  0  0
 68 74  1  0  0  0  0
```

```
 68 75  1  0  0  0  0
 70 76  1  0  0  0  0
 70 77  1  0  0  0  0
 70 78  1  0  0  0  0
 73 76  1  0  0  0  0
 73 79  1  0  0  0  0
 73 80  1  0  0  0  0
 76 81  1  6  0  0  0
 76 86  1  0  0  0  0
 82 83  1  0  0  0  0
 82 84  1  0  0  0  0
 82 85  1  0  0  0  0
 86 87  1  0  0  0  0
 86 88  1  0  0  0  0
 86 89  1  0  0  0  0
M  END
$$$$
mol128
Optimized Catalyst 128
Optimized Catalyst 128
 89 94  0  0  0  0  0  0  0  0999 V2000
   -2.6395    0.2866   -0.7676 C   0  0  0  0  0  0  0  0  0  0  0  0
   -1.7035    1.1733    0.0069 C   0  0  0  0  0  0  0  0  0  0  0  0
   -1.5962    1.6954   -3.5032 C   0  0  0  0  0  0  0  0  0  0  0  0
   -1.6444    2.6103   -0.5683 C   0  0  0  0  0  0  0  0  0  0  0  0
   -0.6841    2.8171   -2.9839 C   0  0  0  0  0  0  0  0  0  0  0  0
   -0.4261    2.8737   -1.4685 C   0  0  0  0  0  0  0  0  0  0  0  0
   -0.6724    0.7409    0.0176 H   0  0  0  0  0  0  0  0  0  0  0  0
   -2.5830    2.8301   -1.1289 H   0  0  0  0  0  0  0  0  0  0  0  0
   -2.6591    1.9667   -3.2906 H   0  0  0  0  0  0  0  0  0  0  0  0
   -1.1459    3.7888   -3.2993 H   0  0  0  0  0  0  0  0  0  0  0  0
   -3.6123    0.1108   -0.2604 H   0  0  0  0  0  0  0  0  0  0  0  0
   -2.0546    1.2398    1.0695 H   0  0  0  0  0  0  0  0  0  0  0  0
   -1.4782    1.6483   -4.6155 H   0  0  0  0  0  0  0  0  0  0  0  0
   -1.5951    3.3162    0.2997 H   0  0  0  0  0  0  0  0  0  0  0  0
    0.3100    2.7346   -3.4920 H   0  0  0  0  0  0  0  0  0  0  0  0
   -0.0490    3.9066   -1.2419 H   0  0  0  0  0  0  0  0  0  0  0  0
   -2.4488   -0.1394   -2.0460 C   0  0  0  0  0  0  0  0  0  0  0  0
   -3.2740   -0.6414   -2.6063 H   0  0  0  0  0  0  0  0  0  0  0  0
   -1.2983    0.2930   -2.9202 C   0  0  0  0  0  0  0  0  0  0  0  0
   -0.3455    0.3351   -2.3376 H   0  0  0  0  0  0  0  0  0  0  0  0
   -1.1675   -0.4289   -3.7643 H   0  0  0  0  0  0  0  0  0  0  0  0
    0.2913   -2.8391   -1.7181 C   0  0  0  0  0  0  0  0  0  0  0  0
    1.2115   -2.4616   -2.8424 C   0  0  0  0  0  0  0  0  0  0  0  0
    2.5997   -1.9345    0.3736 C   0  0  0  0  0  0  0  0  0  0  0  0
    2.6896   -2.8219   -2.5600 C   0  0  0  0  0  0  0  0  0  0  0  0
    3.7817   -1.5839   -0.5423 C   0  0  0  0  0  0  0  0  0  0  0  0
    3.5353   -1.6392   -2.0596 C   0  0  0  0  0  0  0  0  0  0  0  0
    1.1353   -1.3646   -3.0481 H   0  0  0  0  0  0  0  0  0  0  0  0
    2.7391   -3.6596   -1.8266 H   0  0  0  0  0  0  0  0  0  0  0  0
    2.4613   -3.0426    0.3777 H   0  0  0  0  0  0  0  0  0  0  0  0
    4.6137   -2.2921   -0.2936 H   0  0  0  0  0  0  0  0  0  0  0  0
   -0.3773   -3.6975   -1.9555 H   0  0  0  0  0  0  0  0  0  0  0  0
    0.8939   -3.0001   -3.7721 H   0  0  0  0  0  0  0  0  0  0  0  0
    2.8736   -1.6253    1.4140 H   0  0  0  0  0  0  0  0  0  0  0  0
    3.1352   -3.1851   -3.5209 H   0  0  0  0  0  0  0  0  0  0  0  0
    4.1315   -0.5494   -0.2952 H   0  0  0  0  0  0  0  0  0  0  0  0
    4.5421   -1.7031   -2.5511 H   0  0  0  0  0  0  0  0  0  0  0  0
    0.2800   -2.2872   -0.4848 C   0  0  0  0  0  0  0  0  0  0  0  0
   -0.3607   -2.7112    0.3380 H   0  0  0  0  0  0  0  0  0  0  0  0
    1.2629   -1.2534   -0.0031 C   0  0  0  0  0  0  0  0  0  0  0  0
    1.4496   -0.4879   -0.7954 H   0  0  0  0  0  0  0  0  0  0  0  0
    0.8609   -0.7335    0.9003 H   0  0  0  0  0  0  0  0  0  0  0  0
    3.0751   -0.6784   -2.3909 H   0  0  0  0  0  0  0  0  0  0  0  0
    0.3969    2.1669   -1.2096 H   0  0  0  0  0  0  0  0  0  0  0  0
```

```
   -2.3972   -5.4002    0.0141 C   0  0  0  0  0  0  0  0  0  0  0  0
   -2.3803   -4.2733   -0.2840 N   0  0  0  0  0  0  0  0  0  0  0  0
   -5.5390   -2.4301   -0.7298 C   0  0  0  0  0  0  0  0  0  0  0  0
   -4.3750   -2.3880   -0.7797 N   0  0  0  0  0  0  0  0  0  0  0  0
   -2.4184   -2.3854   -0.8093 Ir  0  0  0  0  0  0  0  0  0  0  0  0
   -2.4031   -6.8191    0.3805 C   0  0  1  0  0  0  0  0  0  0  0  0
   -1.6252   -7.6390   -0.6765 C   0  0  0  0  0  0  0  0  0  0  0  0
   -1.8020   -7.0069    1.7934 C   0  0  0  0  0  0  0  0  0  0  0  0
   -3.4779   -7.1673    0.3928 H   0  0  0  0  0  0  0  0  0  0  0  0
   -1.6114   -9.1239   -0.2842 C   0  0  0  0  0  0  0  0  0  0  0  0
   -0.5740   -7.2684   -0.7396 H   0  0  0  0  0  0  0  0  0  0  0  0
   -2.1120   -7.5272   -1.6740 H   0  0  0  0  0  0  0  0  0  0  0  0
   -1.7867   -8.4974    2.1641 C   0  0  0  0  0  0  0  0  0  0  0  0
   -0.7568   -6.6148    1.8123 H   0  0  0  0  0  0  0  0  0  0  0  0
   -2.4138   -6.4485    2.5410 H   0  0  0  0  0  0  0  0  0  0  0  0
   -1.0104   -9.3337    1.1231 C   0  0  1  0  0  0  0  0  0  0  0  0
   -1.0006   -9.6877   -1.0311 H   0  0  0  0  0  0  0  0  0  0  0  0
   -2.6496   -9.5315   -0.3081 H   0  0  0  0  0  0  0  0  0  0  0  0
   -1.3009   -8.6146    3.1637 H   0  0  0  0  0  0  0  0  0  0  0  0
   -2.8321   -8.8792    2.2405 H   0  0  0  0  0  0  0  0  0  0  0  0
    0.0536   -8.9849    1.1101 H   0  0  0  0  0  0  0  0  0  0  0  0
   -7.0030   -2.4622   -0.6717 C   0  0  1  0  0  0  0  0  0  0  0  0
   -7.5145   -1.4317    0.3629 C   0  0  0  0  0  0  0  0  0  0  0  0
   -7.5940   -2.1918   -2.0755 C   0  0  0  0  0  0  0  0  0  0  0  0
   -7.3120   -3.4967   -0.3387 H   0  0  0  0  0  0  0  0  0  0  0  0
   -9.0499   -1.4485    0.4067 C   0  0  0  0  0  0  0  0  0  0  0  0
   -7.1691   -0.4094    0.0773 H   0  0  0  0  0  0  0  0  0  0  0  0
   -7.1157   -1.6822    1.3744 H   0  0  0  0  0  0  0  0  0  0  0  0
   -9.1285   -2.2026   -2.0107 C   0  0  0  0  0  0  0  0  0  0  0  0
   -7.2511   -1.1945   -2.4416 H   0  0  0  0  0  0  0  0  0  0  0  0
   -7.2507   -2.9791   -2.7876 H   0  0  0  0  0  0  0  0  0  0  0  0
   -9.6611   -1.1772   -0.9856 C   0  0  1  0  0  0  0  0  0  0  0  0
   -9.3983   -0.6612    1.1191 H   0  0  0  0  0  0  0  0  0  0  0  0
   -9.4069   -2.4368    0.7816 H   0  0  0  0  0  0  0  0  0  0  0  0
   -9.5329   -1.9531   -3.0221 H   0  0  0  0  0  0  0  0  0  0  0  0
   -9.4885   -3.2222   -1.7359 H   0  0  0  0  0  0  0  0  0  0  0  0
   -9.3520   -0.1528   -1.3153 H   0  0  0  0  0  0  0  0  0  0  0  0
   -1.0263  -10.8187    1.5012 C   0  0  0  0  0  0  0  0  0  0  0  0
   -0.4417  -11.4107    0.7606 H   0  0  0  0  0  0  0  0  0  0  0  0
   -0.5670  -10.9652    2.5054 H   0  0  0  0  0  0  0  0  0  0  0  0
   -2.0658  -11.2137    1.5273 H   0  0  0  0  0  0  0  0  0  0  0  0
  -11.1916   -1.2220   -0.9224 C   0  0  0  0  0  0  0  0  0  0  0  0
  -11.5696   -0.4612   -0.2019 H   0  0  0  0  0  0  0  0  0  0  0  0
  -11.6246   -0.9990   -1.9242 H   0  0  0  0  0  0  0  0  0  0  0  0
  -11.5495   -2.2240   -0.5982 H   0  0  0  0  0  0  0  0  0  0  0  0
  1  2  1  0  0  0  0
  1 11  1  0  0  0  0
  1 17  2  0  0  0  0
  1 49  1  0  0  0  0
  2  4  1  0  0  0  0
  2  7  1  0  0  0  0
  2 12  1  0  0  0  0
  3  5  1  0  0  0  0
  3  9  1  0  0  0  0
  3 13  1  0  0  0  0
  3 19  1  0  0  0  0
  4  6  1  0  0  0  0
  4  8  1  0  0  0  0
  4 14  1  0  0  0  0
  5  6  1  0  0  0  0
  5 10  1  0  0  0  0
  5 15  1  0  0  0  0
  6 16  1  0  0  0  0
  6 44  1  0  0  0  0
 17 18  1  0  0  0  0
```

```
 17 19  1  0  0  0  0
 17 49  1  0  0  0  0
 19 20  1  0  0  0  0
 19 21  1  0  0  0  0
 22 23  1  0  0  0  0
 22 32  1  0  0  0  0
 22 38  2  0  0  0  0
 22 49  1  0  0  0  0
 23 25  1  0  0  0  0
 23 28  1  0  0  0  0
 23 33  1  0  0  0  0
 24 26  1  0  0  0  0
 24 30  1  0  0  0  0
 24 34  1  0  0  0  0
 24 40  1  0  0  0  0
 25 27  1  0  0  0  0
 25 29  1  0  0  0  0
 25 35  1  0  0  0  0
 26 27  1  0  0  0  0
 26 31  1  0  0  0  0
 26 36  1  0  0  0  0
 27 37  1  0  0  0  0
 27 43  1  0  0  0  0
 38 39  1  0  0  0  0
 38 40  1  0  0  0  0
 38 49  1  0  0  0  0
 40 41  1  0  0  0  0
 40 42  1  0  0  0  0
 45 46  3  0  0  0  0
 45 50  1  0  0  0  0
 46 49  1  0  0  0  0
 47 48  3  0  0  0  0
 47 66  1  0  0  0  0
 48 49  1  0  0  0  0
 50 51  1  0  0  0  0
 50 52  1  0  0  0  0
 50 53  1  1  0  0  0
 51 54  1  0  0  0  0
 51 55  1  0  0  0  0
 51 56  1  0  0  0  0
 52 57  1  0  0  0  0
 52 58  1  0  0  0  0
 52 59  1  0  0  0  0
 54 60  1  0  0  0  0
 54 61  1  0  0  0  0
 54 62  1  0  0  0  0
 57 60  1  0  0  0  0
 57 63  1  0  0  0  0
 57 64  1  0  0  0  0
 60 65  1  6  0  0  0
 60 82  1  0  0  0  0
 66 67  1  0  0  0  0
 66 68  1  0  0  0  0
 66 69  1  1  0  0  0
 67 70  1  0  0  0  0
 67 71  1  0  0  0  0
 67 72  1  0  0  0  0
 68 73  1  0  0  0  0
 68 74  1  0  0  0  0
 68 75  1  0  0  0  0
 70 76  1  0  0  0  0
 70 77  1  0  0  0  0
 70 78  1  0  0  0  0
 73 76  1  0  0  0  0
 73 79  1  0  0  0  0
```

```
 73 80  1  0  0  0  0
 76 81  1  6  0  0  0
 76 86  1  0  0  0  0
 82 83  1  0  0  0  0
 82 84  1  0  0  0  0
 82 85  1  0  0  0  0
 86 87  1  0  0  0  0
 86 88  1  0  0  0  0
 86 89  1  0  0  0  0
M  END
$$$$
mol129
Optimized Catalyst 129
Optimized Catalyst 129
 59 66  0  0  0  0  0  0  0  0999 V2000
   -1.0179    0.2986   -1.5730 C   0  0  0  0  0  0  0  0  0  0  0  0
    0.1657   -0.6095   -1.1798 C   0  0  0  0  0  0  0  0  0  0  0  0
   -2.2640    0.0764   -0.7485 C   0  0  0  0  0  0  0  0  0  0  0  0
   -1.1236    0.1401    1.5460 C   0  0  0  0  0  0  0  0  0  0  0  0
   -2.3192   -0.0181    0.6356 C   0  0  0  0  0  0  0  0  0  0  0  0
   -0.4447   -1.2122    1.8508 C   0  0  0  0  0  0  0  0  0  0  0  0
   -3.2038    0.1862   -1.3235 H   0  0  0  0  0  0  0  0  0  0  0  0
   -3.2975   -0.0268    1.1491 H   0  0  0  0  0  0  0  0  0  0  0  0
   -1.2689    0.1035   -2.6494 H   0  0  0  0  0  0  0  0  0  0  0  0
   -0.7143    1.3750   -1.4755 H   0  0  0  0  0  0  0  0  0  0  0  0
    0.8001   -0.7851   -2.0881 H   0  0  0  0  0  0  0  0  0  0  0  0
   -0.9592   -1.6690    2.7393 H   0  0  0  0  0  0  0  0  0  0  0  0
   -1.4566    0.6001    2.5129 H   0  0  0  0  0  0  0  0  0  0  0  0
   -0.3922    0.8479    1.0786 H   0  0  0  0  0  0  0  0  0  0  0  0
   -0.5092   -2.2103    0.7153 C   0  0  0  0  0  0  0  0  0  0  0  0
   -0.2884   -1.9439   -0.6340 C   0  0  0  0  0  0  0  0  0  0  0  0
   -0.5960   -3.2714    1.0475 H   0  0  0  0  0  0  0  0  0  0  0  0
   -0.2539   -2.7869   -1.3597 H   0  0  0  0  0  0  0  0  0  0  0  0
    0.8126   -0.1013   -0.4196 H   0  0  0  0  0  0  0  0  0  0  0  0
    0.6318   -1.0409    2.1155 H   0  0  0  0  0  0  0  0  0  0  0  0
   -3.9793   -2.0871   -0.4282 N   0  0  0  0  0  0  0  0  0  0  0  0
   -5.1212   -2.2567   -0.5776 C   0  0  0  0  0  0  0  0  0  0  0  0
   -2.3381   -4.4437   -0.6781 N   0  0  0  0  0  0  0  0  0  0  0  0
   -2.4565   -5.5730   -0.9161 C   0  0  0  0  0  0  0  0  0  0  0  0
   -2.3181   -2.0328   -0.2156 Rh  0  0  0  0  0  0  0  0  0  0  0  0
   -2.5921   -6.9682   -1.1976 C   0  0  0  0  0  0  0  0  0  0  0  0
   -2.7448   -7.4150   -2.5569 C   0  0  0  0  0  0  0  0  0  0  0  0
   -2.5678   -7.8750   -0.1499 C   0  0  0  0  0  0  0  0  0  0  0  0
   -2.7834   -6.5129   -3.6665 C   0  0  0  0  0  0  0  0  0  0  0  0
   -2.8614   -8.8107   -2.7962 C   0  0  0  0  0  0  0  0  0  0  0  0
   -2.6892   -9.2636   -0.4061 C   0  0  0  0  0  0  0  0  0  0  0  0
   -2.4544   -7.5338    0.8814 H   0  0  0  0  0  0  0  0  0  0  0  0
   -2.9269   -6.9904   -4.9456 C   0  0  0  0  0  0  0  0  0  0  0  0
   -2.6942   -5.4436   -3.4841 H   0  0  0  0  0  0  0  0  0  0  0  0
   -3.0096   -9.2775   -4.1428 C   0  0  0  0  0  0  0  0  0  0  0  0
   -2.8289   -9.7217   -1.6955 C   0  0  0  0  0  0  0  0  0  0  0  0
   -2.6678   -9.9597    0.4354 H   0  0  0  0  0  0  0  0  0  0  0  0
   -3.0406   -8.3909   -5.1885 C   0  0  0  0  0  0  0  0  0  0  0  0
   -2.9552   -6.3083   -5.7978 H   0  0  0  0  0  0  0  0  0  0  0  0
   -3.0960  -10.3517   -4.3179 H   0  0  0  0  0  0  0  0  0  0  0  0
   -2.9170  -10.7930   -1.8958 H   0  0  0  0  0  0  0  0  0  0  0  0
   -3.1507   -8.7394   -6.2173 H   0  0  0  0  0  0  0  0  0  0  0  0
   -6.5224   -2.4345   -0.7772 C   0  0  0  0  0  0  0  0  0  0  0  0
   -7.4654   -1.6407   -0.0326 C   0  0  0  0  0  0  0  0  0  0  0  0
   -6.9541   -3.3773   -1.6978 C   0  0  0  0  0  0  0  0  0  0  0  0
   -7.0654   -0.6568    0.9255 C   0  0  0  0  0  0  0  0  0  0  0  0
   -8.8507   -1.8537   -0.2661 C   0  0  0  0  0  0  0  0  0  0  0  0
   -8.3402   -3.5700   -1.9215 C   0  0  0  0  0  0  0  0  0  0  0  0
   -6.2373   -3.9793   -2.2592 H   0  0  0  0  0  0  0  0  0  0  0  0
   -8.0074    0.0699    1.6111 C   0  0  0  0  0  0  0  0  0  0  0  0
```

```
   -6.0053   -0.4945    1.1060 H   0  0  0  0  0  0  0  0  0  0  0  0
   -9.8092   -1.0820    0.4678 C   0  0  0  0  0  0  0  0  0  0  0  0
   -9.2658   -2.8295   -1.2242 C   0  0  0  0  0  0  0  0  0  0  0  0
   -8.6583   -4.3147   -2.6542 H   0  0  0  0  0  0  0  0  0  0  0  0
   -9.3980   -0.1455    1.3811 C   0  0  0  0  0  0  0  0  0  0  0  0
   -7.7102    0.8228    2.3442 H   0  0  0  0  0  0  0  0  0  0  0  0
  -10.8714   -1.2567    0.2854 H   0  0  0  0  0  0  0  0  0  0  0  0
  -10.3358   -2.9771   -1.3934 H   0  0  0  0  0  0  0  0  0  0  0  0
  -10.1233    0.4459    1.9443 H   0  0  0  0  0  0  0  0  0  0  0  0
  1  2  1  0  0  0  0
  1  3  1  0  0  0  0
  1  9  1  0  0  0  0
  1 10  1  0  0  0  0
  2 11  1  0  0  0  0
  2 16  1  0  0  0  0
  2 19  1  0  0  0  0
  3  5  2  0  0  0  0
  3  7  1  0  0  0  0
  3 25  1  0  0  0  0
  4  5  1  0  0  0  0
  4  6  1  0  0  0  0
  4 13  1  0  0  0  0
  4 14  1  0  0  0  0
  5  8  1  0  0  0  0
  5 25  1  0  0  0  0
  6 12  1  0  0  0  0
  6 15  1  0  0  0  0
  6 20  1  0  0  0  0
 15 16  2  0  0  0  0
 15 17  1  0  0  0  0
 15 25  1  0  0  0  0
 16 18  1  0  0  0  0
 16 25  1  0  0  0  0
 21 22  3  0  0  0  0
 21 25  1  0  0  0  0
 22 43  1  0  0  0  0
 23 24  3  0  0  0  0
 23 25  1  0  0  0  0
 24 26  1  0  0  0  0
 26 27  1  0  0  0  0
 26 28  2  0  0  0  0
 27 29  1  0  0  0  0
 27 30  2  0  0  0  0
 28 31  1  0  0  0  0
 28 32  1  0  0  0  0
 29 33  2  0  0  0  0
 29 34  1  0  0  0  0
 30 35  1  0  0  0  0
 30 36  1  0  0  0  0
 31 36  2  0  0  0  0
 31 37  1  0  0  0  0
 33 38  1  0  0  0  0
 33 39  1  0  0  0  0
 35 38  2  0  0  0  0
 35 40  1  0  0  0  0
 36 41  1  0  0  0  0
 38 42  1  0  0  0  0
 43 44  1  0  0  0  0
 43 45  2  0  0  0  0
 44 46  1  0  0  0  0
 44 47  2  0  0  0  0
 45 48  1  0  0  0  0
 45 49  1  0  0  0  0
 46 50  2  0  0  0  0
 46 51  1  0  0  0  0
```

```
 47 52  1  0  0  0  0
 47 53  1  0  0  0  0
 48 53  2  0  0  0  0
 48 54  1  0  0  0  0
 50 55  1  0  0  0  0
 50 56  1  0  0  0  0
 52 55  2  0  0  0  0
 52 57  1  0  0  0  0
 53 58  1  0  0  0  0
 55 59  1  0  0  0  0
M  END
$$$$
mol130
Optimized Catalyst 130
Optimized Catalyst 130
 59 66  0  0  0  0  0  0  0  0999 V2000
   -0.8660    0.2259   -1.5492 C   0  0  0  0  0  0  0  0  0  0  0  0
    0.3636   -0.6197   -1.1551 C   0  0  0  0  0  0  0  0  0  0  0  0
   -2.1276   -0.0284   -0.7551 C   0  0  0  0  0  0  0  0  0  0  0  0
   -1.0364    0.0170    1.5535 C   0  0  0  0  0  0  0  0  0  0  0  0
   -2.2081   -0.1491    0.6126 C   0  0  0  0  0  0  0  0  0  0  0  0
   -0.2332   -1.2742    1.8192 C   0  0  0  0  0  0  0  0  0  0  0  0
   -3.0585    0.1265   -1.3481 H   0  0  0  0  0  0  0  0  0  0  0  0
   -3.2003   -0.1392    1.1131 H   0  0  0  0  0  0  0  0  0  0  0  0
   -1.0799    0.0584   -2.6409 H   0  0  0  0  0  0  0  0  0  0  0  0
   -0.6065    1.3174   -1.4282 H   0  0  0  0  0  0  0  0  0  0  0  0
    1.0208   -0.7242   -2.0624 H   0  0  0  0  0  0  0  0  0  0  0  0
   -0.6448   -1.7666    2.7427 H   0  0  0  0  0  0  0  0  0  0  0  0
   -1.4189    0.3989    2.5404 H   0  0  0  0  0  0  0  0  0  0  0  0
   -0.3575    0.8144    1.1457 H   0  0  0  0  0  0  0  0  0  0  0  0
   -0.2055   -2.2836    0.6932 C   0  0  0  0  0  0  0  0  0  0  0  0
    0.0383   -1.9999   -0.6309 C   0  0  0  0  0  0  0  0  0  0  0  0
   -0.1511   -3.3383    1.0477 H   0  0  0  0  0  0  0  0  0  0  0  0
    0.2445   -2.8246   -1.3474 H   0  0  0  0  0  0  0  0  0  0  0  0
    0.9719   -0.0749   -0.3827 H   0  0  0  0  0  0  0  0  0  0  0  0
    0.8366   -0.9937    2.0439 H   0  0  0  0  0  0  0  0  0  0  0  0
   -4.2700   -2.3470   -0.6014 N   0  0  0  0  0  0  0  0  0  0  0  0
   -5.4287   -2.4408   -0.7271 C   0  0  0  0  0  0  0  0  0  0  0  0
   -2.2786   -4.2930   -0.6598 N   0  0  0  0  0  0  0  0  0  0  0  0
   -2.3954   -5.4337   -0.8881 C   0  0  0  0  0  0  0  0  0  0  0  0
   -2.5217   -6.8226   -1.1555 C   0  0  0  0  0  0  0  0  0  0  0  0
   -2.6899   -7.2815   -2.5109 C   0  0  0  0  0  0  0  0  0  0  0  0
   -2.4884   -7.7249   -0.0958 C   0  0  0  0  0  0  0  0  0  0  0  0
   -2.7205   -6.3934   -3.6305 C   0  0  0  0  0  0  0  0  0  0  0  0
   -2.8296   -8.6794   -2.7313 C   0  0  0  0  0  0  0  0  0  0  0  0
   -2.6247   -9.1096   -0.3378 C   0  0  0  0  0  0  0  0  0  0  0  0
   -2.3587   -7.3738    0.9312 H   0  0  0  0  0  0  0  0  0  0  0  0
   -2.8811   -6.8854   -4.9032 C   0  0  0  0  0  0  0  0  0  0  0  0
   -2.6075   -5.3238   -3.4683 H   0  0  0  0  0  0  0  0  0  0  0  0
   -3.0015   -9.1598   -4.0710 C   0  0  0  0  0  0  0  0  0  0  0  0
   -2.7922   -9.5784   -1.6237 C   0  0  0  0  0  0  0  0  0  0  0  0
   -2.5945   -9.8028    0.5077 H   0  0  0  0  0  0  0  0  0  0  0  0
   -3.0252   -8.2860   -5.1276 C   0  0  0  0  0  0  0  0  0  0  0  0
   -2.8987   -6.2129   -5.7650 H   0  0  0  0  0  0  0  0  0  0  0  0
   -3.1110  -10.2348   -4.2324 H   0  0  0  0  0  0  0  0  0  0  0  0
   -2.8973  -10.6523   -1.8116 H   0  0  0  0  0  0  0  0  0  0  0  0
   -3.1504   -8.6452   -6.1520 H   0  0  0  0  0  0  0  0  0  0  0  0
   -6.8357   -2.5435   -0.8851 C   0  0  0  0  0  0  0  0  0  0  0  0
   -7.7119   -1.7125   -0.1002 C   0  0  0  0  0  0  0  0  0  0  0  0
   -7.3495   -3.4589   -1.8001 C   0  0  0  0  0  0  0  0  0  0  0  0
   -7.2281   -0.7549    0.8440 C   0  0  0  0  0  0  0  0  0  0  0  0
   -9.1149   -1.8604   -0.2816 C   0  0  0  0  0  0  0  0  0  0  0  0
   -8.7456   -3.5860   -1.9679 C   0  0  0  0  0  0  0  0  0  0  0  0
   -6.6825   -4.0872   -2.3953 H   0  0  0  0  0  0  0  0  0  0  0  0
   -8.1089    0.0102    1.5693 C   0  0  0  0  0  0  0  0  0  0  0  0
```

```
   -6.1568   -0.6374    0.9844 H   0  0  0  0  0  0  0  0  0  0  0  0
  -10.0073   -1.0486    0.4935 C   0  0  0  0  0  0  0  0  0  0  0  0
   -9.6098   -2.8076   -1.2265 C   0  0  0  0  0  0  0  0  0  0  0  0
   -9.1307   -4.3090   -2.6921 H   0  0  0  0  0  0  0  0  0  0  0  0
   -9.5161   -0.1391    1.3942 C   0  0  0  0  0  0  0  0  0  0  0  0
   -7.7466    0.7461    2.2919 H   0  0  0  0  0  0  0  0  0  0  0  0
  -11.0840   -1.1693    0.3512 H   0  0  0  0  0  0  0  0  0  0  0  0
  -10.6928   -2.9074   -1.3557 H   0  0  0  0  0  0  0  0  0  0  0  0
  -10.1896    0.4831    1.9888 H   0  0  0  0  0  0  0  0  0  0  0  0
   -2.3177   -2.3486   -0.3488 Ir  0  0  0  0  0  0  0  0  0  0  0  0
  1  2  1  0  0  0  0
  1  3  1  0  0  0  0
  1  9  1  0  0  0  0
  1 10  1  0  0  0  0
  2 11  1  0  0  0  0
  2 16  1  0  0  0  0
  2 19  1  0  0  0  0
  3  5  2  0  0  0  0
  3  7  1  0  0  0  0
  3 59  1  0  0  0  0
  4  5  1  0  0  0  0
  4  6  1  0  0  0  0
  4 13  1  0  0  0  0
  4 14  1  0  0  0  0
  5  8  1  0  0  0  0
  5 59  1  0  0  0  0
  6 12  1  0  0  0  0
  6 15  1  0  0  0  0
  6 20  1  0  0  0  0
 15 16  2  0  0  0  0
 15 17  1  0  0  0  0
 15 59  1  0  0  0  0
 16 18  1  0  0  0  0
 16 59  1  0  0  0  0
 21 22  3  0  0  0  0
 21 59  1  0  0  0  0
 22 42  1  0  0  0  0
 23 24  3  0  0  0  0
 23 59  1  0  0  0  0
 24 25  1  0  0  0  0
 25 26  1  0  0  0  0
 25 27  2  0  0  0  0
 26 28  1  0  0  0  0
 26 29  2  0  0  0  0
 27 30  1  0  0  0  0
 27 31  1  0  0  0  0
 28 32  2  0  0  0  0
 28 33  1  0  0  0  0
 29 34  1  0  0  0  0
 29 35  1  0  0  0  0
 30 35  2  0  0  0  0
 30 36  1  0  0  0  0
 32 37  1  0  0  0  0
 32 38  1  0  0  0  0
 34 37  2  0  0  0  0
 34 39  1  0  0  0  0
 35 40  1  0  0  0  0
 37 41  1  0  0  0  0
 42 43  1  0  0  0  0
 42 44  2  0  0  0  0
 43 45  1  0  0  0  0
 43 46  2  0  0  0  0
 44 47  1  0  0  0  0
 44 48  1  0  0  0  0
 45 49  2  0  0  0  0
```

```
 45 50  1  0  0  0  0
 46 51  1  0  0  0  0
 46 52  1  0  0  0  0
 47 52  2  0  0  0  0
 47 53  1  0  0  0  0
 49 54  1  0  0  0  0
 49 55  1  0  0  0  0
 51 54  2  0  0  0  0
 51 56  1  0  0  0  0
 52 57  1  0  0  0  0
 54 58  1  0  0  0  0
M  END
$$$$
mol131
Optimized Catalyst 131
Optimized Catalyst 131
 65 72  0  0  0  0  0  0  0  0999 V2000
   -1.0296    0.2852   -1.5764 C   0  0  0  0  0  0  0  0  0  0  0  0
    0.1546   -0.6229   -1.1845 C   0  0  0  0  0  0  0  0  0  0  0  0
   -2.2725    0.0663   -0.7462 C   0  0  0  0  0  0  0  0  0  0  0  0
   -1.1278    0.1257    1.5479 C   0  0  0  0  0  0  0  0  0  0  0  0
   -2.3237   -0.0292    0.6374 C   0  0  0  0  0  0  0  0  0  0  0  0
   -0.4529   -1.2294    1.8488 C   0  0  0  0  0  0  0  0  0  0  0  0
   -3.2147    0.1741   -1.3180 H   0  0  0  0  0  0  0  0  0  0  0  0
   -3.3007   -0.0420    1.1532 H   0  0  0  0  0  0  0  0  0  0  0  0
   -1.2849    0.0859   -2.6511 H   0  0  0  0  0  0  0  0  0  0  0  0
   -0.7251    1.3614   -1.4835 H   0  0  0  0  0  0  0  0  0  0  0  0
    0.7878   -0.7994   -2.0934 H   0  0  0  0  0  0  0  0  0  0  0  0
   -0.9709   -1.6865    2.7351 H   0  0  0  0  0  0  0  0  0  0  0  0
   -1.4612    0.5825    2.5164 H   0  0  0  0  0  0  0  0  0  0  0  0
   -0.3950    0.8341    1.0844 H   0  0  0  0  0  0  0  0  0  0  0  0
   -0.5201   -2.2251    0.7111 C   0  0  0  0  0  0  0  0  0  0  0  0
   -0.2994   -1.9573   -0.6382 C   0  0  0  0  0  0  0  0  0  0  0  0
   -0.6112   -3.2863    1.0420 H   0  0  0  0  0  0  0  0  0  0  0  0
   -0.2664   -2.7997   -1.3646 H   0  0  0  0  0  0  0  0  0  0  0  0
    0.8016   -0.1136   -0.4255 H   0  0  0  0  0  0  0  0  0  0  0  0
    0.6237   -1.0631    2.1153 H   0  0  0  0  0  0  0  0  0  0  0  0
   -3.9946   -2.0960   -0.4295 N   0  0  0  0  0  0  0  0  0  0  0  0
   -5.1374   -2.2667   -0.5732 C   0  0  0  0  0  0  0  0  0  0  0  0
   -2.3455   -4.4415   -0.6940 N   0  0  0  0  0  0  0  0  0  0  0  0
   -2.4646   -5.5709   -0.9342 C   0  0  0  0  0  0  0  0  0  0  0  0
   -2.6003   -6.9621   -1.2165 C   0  0  0  0  0  0  0  0  0  0  0  0
   -2.7519   -7.4169   -2.5740 C   0  0  0  0  0  0  0  0  0  0  0  0
   -2.5778   -7.8729   -0.1706 C   0  0  0  0  0  0  0  0  0  0  0  0
   -2.7867   -6.5010   -3.6723 C   0  0  0  0  0  0  0  0  0  0  0  0
   -2.8690   -8.8117   -2.8217 C   0  0  0  0  0  0  0  0  0  0  0  0
   -2.6991   -9.2546   -0.4246 C   0  0  0  0  0  0  0  0  0  0  0  0
   -2.4640   -7.5307    0.8611 H   0  0  0  0  0  0  0  0  0  0  0  0
   -2.9289   -6.9588   -4.9586 C   0  0  0  0  0  0  0  0  0  0  0  0
   -2.6947   -5.4341   -3.4765 H   0  0  0  0  0  0  0  0  0  0  0  0
   -3.0147   -9.2513   -4.1775 C   0  0  0  0  0  0  0  0  0  0  0  0
   -2.8370   -9.7361   -1.7163 C   0  0  0  0  0  0  0  0  0  0  0  0
   -2.6792   -9.9421    0.4234 H   0  0  0  0  0  0  0  0  0  0  0  0
   -3.0443   -8.3541   -5.2153 C   0  0  0  0  0  0  0  0  0  0  0  0
   -2.9541   -6.2653   -5.8012 H   0  0  0  0  0  0  0  0  0  0  0  0
   -3.1024  -10.3193   -4.3835 H   0  0  0  0  0  0  0  0  0  0  0  0
   -3.1539   -8.6943   -6.2465 H   0  0  0  0  0  0  0  0  0  0  0  0
   -6.5362   -2.4417   -0.7663 C   0  0  0  0  0  0  0  0  0  0  0  0
   -7.4821   -1.6466   -0.0253 C   0  0  0  0  0  0  0  0  0  0  0  0
   -6.9769   -3.3874   -1.6816 C   0  0  0  0  0  0  0  0  0  0  0  0
   -7.0575   -0.6640    0.9231 C   0  0  0  0  0  0  0  0  0  0  0  0
   -8.8714   -1.8509   -0.2473 C   0  0  0  0  0  0  0  0  0  0  0  0
   -8.3570   -3.5778   -1.8980 C   0  0  0  0  0  0  0  0  0  0  0  0
   -6.2615   -3.9925   -2.2435 H   0  0  0  0  0  0  0  0  0  0  0  0
   -7.9786    0.0800    1.6184 C   0  0  0  0  0  0  0  0  0  0  0  0
```

```
   -5.9931   -0.5148    1.0897 H   0  0  0  0  0  0  0  0  0  0  0  0
   -9.8026   -1.0577    0.4989 C   0  0  0  0  0  0  0  0  0  0  0  0
   -9.3017   -2.8382   -1.2051 C   0  0  0  0  0  0  0  0  0  0  0  0
   -8.6687   -4.3270   -2.6284 H   0  0  0  0  0  0  0  0  0  0  0  0
   -9.3712   -0.1203    1.4036 C   0  0  0  0  0  0  0  0  0  0  0  0
   -7.6611    0.8320    2.3435 H   0  0  0  0  0  0  0  0  0  0  0  0
  -10.8726   -1.2051    0.3423 H   0  0  0  0  0  0  0  0  0  0  0  0
  -10.0848    0.4816    1.9693 H   0  0  0  0  0  0  0  0  0  0  0  0
   -2.3288   -2.0453   -0.2193 Rh  0  0  0  0  0  0  0  0  0  0  0  0
  -10.7544   -3.0707   -1.4553 C   0  0  0  0  0  0  0  0  0  0  0  0
  -11.2589   -3.4049   -0.5145 H   0  0  0  0  0  0  0  0  0  0  0  0
  -11.2403   -2.1312   -1.8190 H   0  0  0  0  0  0  0  0  0  0  0  0
  -10.9206   -3.8595   -2.2283 H   0  0  0  0  0  0  0  0  0  0  0  0
   -2.9486  -11.2052   -1.9534 C   0  0  0  0  0  0  0  0  0  0  0  0
   -3.9231  -11.4449   -2.4467 H   0  0  0  0  0  0  0  0  0  0  0  0
   -2.1104  -11.5546   -2.6068 H   0  0  0  0  0  0  0  0  0  0  0  0
   -2.8986  -11.7843   -0.9996 H   0  0  0  0  0  0  0  0  0  0  0  0
  1  2  1  0  0  0  0
  1  3  1  0  0  0  0
  1  9  1  0  0  0  0
  1 10  1  0  0  0  0
  2 11  1  0  0  0  0
  2 16  1  0  0  0  0
  2 19  1  0  0  0  0
  3  5  2  0  0  0  0
  3  7  1  0  0  0  0
  3 57  1  0  0  0  0
  4  5  1  0  0  0  0
  4  6  1  0  0  0  0
  4 13  1  0  0  0  0
  4 14  1  0  0  0  0
  5  8  1  0  0  0  0
  5 57  1  0  0  0  0
  6 12  1  0  0  0  0
  6 15  1  0  0  0  0
  6 20  1  0  0  0  0
 15 16  2  0  0  0  0
 15 17  1  0  0  0  0
 15 57  1  0  0  0  0
 16 18  1  0  0  0  0
 16 57  1  0  0  0  0
 21 22  3  0  0  0  0
 21 57  1  0  0  0  0
 22 41  1  0  0  0  0
 23 24  3  0  0  0  0
 23 57  1  0  0  0  0
 24 25  1  0  0  0  0
 25 26  1  0  0  0  0
 25 27  2  0  0  0  0
 26 28  1  0  0  0  0
 26 29  2  0  0  0  0
 27 30  1  0  0  0  0
 27 31  1  0  0  0  0
 28 32  2  0  0  0  0
 28 33  1  0  0  0  0
 29 34  1  0  0  0  0
 29 35  1  0  0  0  0
 30 35  2  0  0  0  0
 30 36  1  0  0  0  0
 32 37  1  0  0  0  0
 32 38  1  0  0  0  0
 34 37  2  0  0  0  0
 34 39  1  0  0  0  0
 35 62  1  0  0  0  0
 37 40  1  0  0  0  0
```

```
 41 42  1  0  0  0  0
 41 43  2  0  0  0  0
 42 44  1  0  0  0  0
 42 45  2  0  0  0  0
 43 46  1  0  0  0  0
 43 47  1  0  0  0  0
 44 48  2  0  0  0  0
 44 49  1  0  0  0  0
 45 50  1  0  0  0  0
 45 51  1  0  0  0  0
 46 51  2  0  0  0  0
 46 52  1  0  0  0  0
 48 53  1  0  0  0  0
 48 54  1  0  0  0  0
 50 53  2  0  0  0  0
 50 55  1  0  0  0  0
 51 58  1  0  0  0  0
 53 56  1  0  0  0  0
 58 59  1  0  0  0  0
 58 60  1  0  0  0  0
 58 61  1  0  0  0  0
 62 63  1  0  0  0  0
 62 64  1  0  0  0  0
 62 65  1  0  0  0  0
M  END
$$$$
mol132
Optimized Catalyst 132
Optimized Catalyst 132
 65 72  0  0  0  0  0  0  0  0999 V2000
   -0.8522    0.2091   -1.5465 C   0  0  0  0  0  0  0  0  0  0  0  0
    0.3794   -0.6346   -1.1548 C   0  0  0  0  0  0  0  0  0  0  0  0
   -2.1121   -0.0450   -0.7498 C   0  0  0  0  0  0  0  0  0  0  0  0
   -1.0211   -0.0064    1.5593 C   0  0  0  0  0  0  0  0  0  0  0  0
   -2.1924   -0.1703    0.6177 C   0  0  0  0  0  0  0  0  0  0  0  0
   -0.2119   -1.2957    1.8173 C   0  0  0  0  0  0  0  0  0  0  0  0
   -3.0446    0.1122   -1.3396 H   0  0  0  0  0  0  0  0  0  0  0  0
   -3.1855   -0.1590    1.1164 H   0  0  0  0  0  0  0  0  0  0  0  0
   -1.0705    0.0372   -2.6365 H   0  0  0  0  0  0  0  0  0  0  0  0
   -0.5937    1.3011   -1.4297 H   0  0  0  0  0  0  0  0  0  0  0  0
    1.0369   -0.7354   -2.0623 H   0  0  0  0  0  0  0  0  0  0  0  0
   -0.6172   -1.7923    2.7412 H   0  0  0  0  0  0  0  0  0  0  0  0
   -1.4041    0.3675    2.5489 H   0  0  0  0  0  0  0  0  0  0  0  0
   -0.3470    0.7969    1.1550 H   0  0  0  0  0  0  0  0  0  0  0  0
   -0.1875   -2.3030    0.6893 C   0  0  0  0  0  0  0  0  0  0  0  0
    0.0560   -2.0163   -0.6336 C   0  0  0  0  0  0  0  0  0  0  0  0
   -0.1358   -3.3594    1.0400 H   0  0  0  0  0  0  0  0  0  0  0  0
    0.2583   -2.8398   -1.3519 H   0  0  0  0  0  0  0  0  0  0  0  0
    0.9852   -0.0912   -0.3798 H   0  0  0  0  0  0  0  0  0  0  0  0
    0.8579   -1.0120    2.0372 H   0  0  0  0  0  0  0  0  0  0  0  0
   -4.2626   -2.3579   -0.6031 N   0  0  0  0  0  0  0  0  0  0  0  0
   -5.4220   -2.4531   -0.7267 C   0  0  0  0  0  0  0  0  0  0  0  0
   -2.2709   -4.3099   -0.6615 N   0  0  0  0  0  0  0  0  0  0  0  0
   -2.3926   -5.4487   -0.8997 C   0  0  0  0  0  0  0  0  0  0  0  0
   -2.5188   -6.8304   -1.1754 C   0  0  0  0  0  0  0  0  0  0  0  0
   -2.6878   -7.2905   -2.5315 C   0  0  0  0  0  0  0  0  0  0  0  0
   -2.4841   -7.7443   -0.1222 C   0  0  0  0  0  0  0  0  0  0  0  0
   -2.7185   -6.3818   -3.6338 C   0  0  0  0  0  0  0  0  0  0  0  0
   -2.8280   -8.6861   -2.7686 C   0  0  0  0  0  0  0  0  0  0  0  0
   -2.6217   -9.1184   -0.3705 C   0  0  0  0  0  0  0  0  0  0  0  0
   -2.3513   -7.3980    0.9070 H   0  0  0  0  0  0  0  0  0  0  0  0
   -2.8798   -6.8460   -4.9171 C   0  0  0  0  0  0  0  0  0  0  0  0
   -2.6059   -5.3155   -3.4508 H   0  0  0  0  0  0  0  0  0  0  0  0
   -2.9971   -9.1310   -4.1204 C   0  0  0  0  0  0  0  0  0  0  0  0
   -2.7933   -9.6056   -1.6615 C   0  0  0  0  0  0  0  0  0  0  0  0
```

```
   -2.5900   -9.8080    0.4774 H   0  0  0  0  0  0  0  0  0  0  0  0
   -3.0227   -8.2399   -5.1637 C   0  0  0  0  0  0  0  0  0  0  0  0
   -2.8989   -6.1559   -5.7644 H   0  0  0  0  0  0  0  0  0  0  0  0
   -3.1057  -10.1994   -4.3188 H   0  0  0  0  0  0  0  0  0  0  0  0
   -3.1491   -8.5845   -6.1923 H   0  0  0  0  0  0  0  0  0  0  0  0
   -6.8235   -2.5588   -0.8850 C   0  0  0  0  0  0  0  0  0  0  0  0
   -7.7129   -1.7334   -0.1062 C   0  0  0  0  0  0  0  0  0  0  0  0
   -7.3375   -3.4775   -1.8005 C   0  0  0  0  0  0  0  0  0  0  0  0
   -7.2178   -0.7759    0.8315 C   0  0  0  0  0  0  0  0  0  0  0  0
   -9.1165   -1.8828   -0.2826 C   0  0  0  0  0  0  0  0  0  0  0  0
   -8.7239   -3.6137   -1.9656 C   0  0  0  0  0  0  0  0  0  0  0  0
   -6.6645   -4.1006   -2.3963 H   0  0  0  0  0  0  0  0  0  0  0  0
   -8.0864   -0.0001    1.5610 C   0  0  0  0  0  0  0  0  0  0  0  0
   -6.1448   -0.6657    0.9657 H   0  0  0  0  0  0  0  0  0  0  0  0
   -9.9900   -1.0568    0.4977 C   0  0  0  0  0  0  0  0  0  0  0  0
   -9.6182   -2.8447   -1.2282 C   0  0  0  0  0  0  0  0  0  0  0  0
   -9.0953   -4.3419   -2.6907 H   0  0  0  0  0  0  0  0  0  0  0  0
   -9.4918   -0.1428    1.3919 C   0  0  0  0  0  0  0  0  0  0  0  0
   -7.7136    0.7350    2.2786 H   0  0  0  0  0  0  0  0  0  0  0  0
  -11.0702   -1.1591    0.3752 H   0  0  0  0  0  0  0  0  0  0  0  0
  -10.1616    0.4847    1.9840 H   0  0  0  0  0  0  0  0  0  0  0  0
  -11.0839   -3.0256   -1.4250 C   0  0  0  0  0  0  0  0  0  0  0  0
  -11.5695   -3.3411   -0.4666 H   0  0  0  0  0  0  0  0  0  0  0  0
  -11.5514   -2.0692   -1.7724 H   0  0  0  0  0  0  0  0  0  0  0  0
  -11.3093   -3.8081   -2.1905 H   0  0  0  0  0  0  0  0  0  0  0  0
   -2.9362  -11.0721   -1.8828 C   0  0  0  0  0  0  0  0  0  0  0  0
   -3.9181  -11.3019   -2.3685 H   0  0  0  0  0  0  0  0  0  0  0  0
   -2.1088  -11.4477   -2.5372 H   0  0  0  0  0  0  0  0  0  0  0  0
   -2.8930  -11.6450   -0.9236 H   0  0  0  0  0  0  0  0  0  0  0  0
   -2.3076   -2.3646   -0.3449 Ir  0  0  0  0  0  0  0  0  0  0  0  0
  1  2  1  0  0  0  0
  1  3  1  0  0  0  0
  1  9  1  0  0  0  0
  1 10  1  0  0  0  0
  2 11  1  0  0  0  0
  2 16  1  0  0  0  0
  2 19  1  0  0  0  0
  3  5  2  0  0  0  0
  3  7  1  0  0  0  0
  3 65  1  0  0  0  0
  4  5  1  0  0  0  0
  4  6  1  0  0  0  0
  4 13  1  0  0  0  0
  4 14  1  0  0  0  0
  5  8  1  0  0  0  0
  5 65  1  0  0  0  0
  6 12  1  0  0  0  0
  6 15  1  0  0  0  0
  6 20  1  0  0  0  0
 15 16  2  0  0  0  0
 15 17  1  0  0  0  0
 15 65  1  0  0  0  0
 16 18  1  0  0  0  0
 16 65  1  0  0  0  0
 21 22  3  0  0  0  0
 21 65  1  0  0  0  0
 22 41  1  0  0  0  0
 23 24  3  0  0  0  0
 23 65  1  0  0  0  0
 24 25  1  0  0  0  0
 25 26  2  0  0  0  0
 25 27  1  0  0  0  0
 26 28  1  0  0  0  0
 26 29  1  0  0  0  0
 27 30  2  0  0  0  0
```

```
 27 31  1  0  0  0  0
 28 32  2  0  0  0  0
 28 33  1  0  0  0  0
 29 34  1  0  0  0  0
 29 35  2  0  0  0  0
 30 35  1  0  0  0  0
 30 36  1  0  0  0  0
 32 37  1  0  0  0  0
 32 38  1  0  0  0  0
 34 37  2  0  0  0  0
 34 39  1  0  0  0  0
 35 61  1  0  0  0  0
 37 40  1  0  0  0  0
 41 42  2  0  0  0  0
 41 43  1  0  0  0  0
 42 44  1  0  0  0  0
 42 45  1  0  0  0  0
 43 46  2  0  0  0  0
 43 47  1  0  0  0  0
 44 48  2  0  0  0  0
 44 49  1  0  0  0  0
 45 50  1  0  0  0  0
 45 51  2  0  0  0  0
 46 51  1  0  0  0  0
 46 52  1  0  0  0  0
 48 53  1  0  0  0  0
 48 54  1  0  0  0  0
 50 53  2  0  0  0  0
 50 55  1  0  0  0  0
 51 57  1  0  0  0  0
 53 56  1  0  0  0  0
 57 58  1  0  0  0  0
 57 59  1  0  0  0  0
 57 60  1  0  0  0  0
 61 62  1  0  0  0  0
 61 63  1  0  0  0  0
 61 64  1  0  0  0  0
M  END
$$$$
mol133
Optimized Catalyst 133
Optimized Catalyst 133
 83 90  0  0  0  0  0  0  0  0999 V2000
   -1.6586   -0.2659   -0.6293 C   0  0  0  0  0  0  0  0  0  0  0  0
   -1.2442    0.9282    0.1845 C   0  0  0  0  0  0  0  0  0  0  0  0
   -1.8969    1.6870   -3.2368 C   0  0  0  0  0  0  0  0  0  0  0  0
   -1.9788    2.2290   -0.2116 C   0  0  0  0  0  0  0  0  0  0  0  0
   -1.6022    3.0754   -2.6468 C   0  0  0  0  0  0  0  0  0  0  0  0
   -1.1811    3.1321   -1.1679 C   0  0  0  0  0  0  0  0  0  0  0  0
   -0.1398    1.0770    0.0859 H   0  0  0  0  0  0  0  0  0  0  0  0
   -2.9633    1.9727   -0.6677 H   0  0  0  0  0  0  0  0  0  0  0  0
   -2.9094    1.3576   -2.8984 H   0  0  0  0  0  0  0  0  0  0  0  0
   -2.5267    3.6939   -2.7744 H   0  0  0  0  0  0  0  0  0  0  0  0
   -2.1828   -1.0591   -0.0416 H   0  0  0  0  0  0  0  0  0  0  0  0
   -1.4612    0.7214    1.2628 H   0  0  0  0  0  0  0  0  0  0  0  0
   -1.9285    1.7913   -4.3497 H   0  0  0  0  0  0  0  0  0  0  0  0
   -2.1772    2.8057    0.7260 H   0  0  0  0  0  0  0  0  0  0  0  0
   -0.7861    3.5511   -3.2468 H   0  0  0  0  0  0  0  0  0  0  0  0
   -1.3095    4.1937   -0.8316 H   0  0  0  0  0  0  0  0  0  0  0  0
   -1.4826   -0.4232   -1.9541 C   0  0  0  0  0  0  0  0  0  0  0  0
   -1.8890   -1.3257   -2.4726 H   0  0  0  0  0  0  0  0  0  0  0  0
   -0.8671    0.5943   -2.8746 C   0  0  0  0  0  0  0  0  0  0  0  0
    0.0232    1.0625   -2.3893 H   0  0  0  0  0  0  0  0  0  0  0  0
   -0.5233    0.0933   -3.8114 H   0  0  0  0  0  0  0  0  0  0  0  0
    0.7761   -3.7164   -1.4204 C   0  0  0  0  0  0  0  0  0  0  0  0
```

```
    1.1870   -2.9510   -2.6550 C   0  0  0  0  0  0  0  0  0  0  0  0
    2.7894   -1.7452    0.2916 C   0  0  0  0  0  0  0  0  0  0  0  0
    2.6852   -2.5693   -2.6730 C   0  0  0  0  0  0  0  0  0  0  0  0
    3.4754   -0.9233   -0.8109 C   0  0  0  0  0  0  0  0  0  0  0  0
    2.9707   -1.1177   -2.2517 C   0  0  0  0  0  0  0  0  0  0  0  0
    0.5434   -2.0224   -2.7326 H   0  0  0  0  0  0  0  0  0  0  0  0
    3.2530   -3.2712   -2.0171 H   0  0  0  0  0  0  0  0  0  0  0  0
    3.1840   -2.7908    0.2614 H   0  0  0  0  0  0  0  0  0  0  0  0
    4.5665   -1.1773   -0.7820 H   0  0  0  0  0  0  0  0  0  0  0  0
    0.6671   -4.8079   -1.5597 H   0  0  0  0  0  0  0  0  0  0  0  0
    0.9676   -3.5826   -3.5551 H   0  0  0  0  0  0  0  0  0  0  0  0
    3.0820   -1.3041    1.2776 H   0  0  0  0  0  0  0  0  0  0  0  0
    3.0573   -2.7082   -3.7194 H   0  0  0  0  0  0  0  0  0  0  0  0
    3.3725    0.1632   -0.5625 H   0  0  0  0  0  0  0  0  0  0  0  0
    3.7638   -0.7095   -2.9322 H   0  0  0  0  0  0  0  0  0  0  0  0
    0.7722   -3.1794   -0.1172 C   0  0  0  0  0  0  0  0  0  0  0  0
    0.6583   -3.8531    0.7516 H   0  0  0  0  0  0  0  0  0  0  0  0
    1.2479   -1.7856    0.2104 C   0  0  0  0  0  0  0  0  0  0  0  0
    0.8806   -1.0705   -0.5789 H   0  0  0  0  0  0  0  0  0  0  0  0
    0.8059   -1.4688    1.1890 H   0  0  0  0  0  0  0  0  0  0  0  0
    2.0581   -0.4948   -2.4068 H   0  0  0  0  0  0  0  0  0  0  0  0
   -0.0939    2.8948   -1.0899 H   0  0  0  0  0  0  0  0  0  0  0  0
   -2.1061   -5.3942    0.0000 C   0  0  0  0  0  0  0  0  0  0  0  0
   -1.6078   -4.4116   -0.3775 N   0  0  0  0  0  0  0  0  0  0  0  0
   -1.0959   -2.9385   -0.9760 Rh  0  0  0  0  0  0  0  0  0  0  0  0
   -4.8880   -2.6228   -1.2810 C   0  0  0  0  0  0  0  0  0  0  0  0
   -3.7355   -2.7555   -1.2926 N   0  0  0  0  0  0  0  0  0  0  0  0
   -6.3091   -2.4667   -1.2427 C   0  0  0  0  0  0  0  0  0  0  0  0
   -7.0102   -1.9485   -2.3865 C   0  0  0  0  0  0  0  0  0  0  0  0
   -6.9921   -2.8197   -0.0898 C   0  0  0  0  0  0  0  0  0  0  0  0
   -6.3434   -1.5672   -3.5936 C   0  0  0  0  0  0  0  0  0  0  0  0
   -8.4225   -1.8133   -2.3035 C   0  0  0  0  0  0  0  0  0  0  0  0
   -8.4006   -2.6743   -0.0220 C   0  0  0  0  0  0  0  0  0  0  0  0
   -6.4565   -3.2115    0.7773 H   0  0  0  0  0  0  0  0  0  0  0  0
   -7.0597   -1.0815   -4.6587 C   0  0  0  0  0  0  0  0  0  0  0  0
   -5.2609   -1.6676   -3.6556 H   0  0  0  0  0  0  0  0  0  0  0  0
   -9.1416   -1.3045   -3.4336 C   0  0  0  0  0  0  0  0  0  0  0  0
   -9.0993   -2.1846   -1.1008 C   0  0  0  0  0  0  0  0  0  0  0  0
   -8.9173   -2.9562    0.8978 H   0  0  0  0  0  0  0  0  0  0  0  0
   -8.4777   -0.9490   -4.5795 C   0  0  0  0  0  0  0  0  0  0  0  0
   -6.5581   -0.7892   -5.5835 H   0  0  0  0  0  0  0  0  0  0  0  0
  -10.2264   -1.2070   -3.3629 H   0  0  0  0  0  0  0  0  0  0  0  0
  -10.1858   -2.0711   -1.0529 H   0  0  0  0  0  0  0  0  0  0  0  0
   -9.0189   -0.5614   -5.4448 H   0  0  0  0  0  0  0  0  0  0  0  0
   -2.6884   -6.6185    0.4449 C   0  0  0  0  0  0  0  0  0  0  0  0
   -2.3609   -7.1451    1.7443 C   0  0  0  0  0  0  0  0  0  0  0  0
   -3.5604   -7.2904   -0.3975 C   0  0  0  0  0  0  0  0  0  0  0  0
   -1.4777   -6.4768    2.6495 C   0  0  0  0  0  0  0  0  0  0  0  0
   -2.9461   -8.3800    2.1335 C   0  0  0  0  0  0  0  0  0  0  0  0
   -4.1383   -8.5193    0.0108 C   0  0  0  0  0  0  0  0  0  0  0  0
   -3.8095   -6.8862   -1.3808 H   0  0  0  0  0  0  0  0  0  0  0  0
   -1.1950   -7.0249    3.8760 C   0  0  0  0  0  0  0  0  0  0  0  0
   -1.0384   -5.5266    2.3539 H   0  0  0  0  0  0  0  0  0  0  0  0
   -2.6301   -8.9269    3.4195 C   0  0  0  0  0  0  0  0  0  0  0  0
   -3.8372   -9.0536    1.2415 C   0  0  0  0  0  0  0  0  0  0  0  0
   -4.8215   -9.0322   -0.6701 H   0  0  0  0  0  0  0  0  0  0  0  0
   -1.7769   -8.2668    4.2662 C   0  0  0  0  0  0  0  0  0  0  0  0
   -0.5229   -6.5209    4.5730 H   0  0  0  0  0  0  0  0  0  0  0  0
   -3.0823   -9.8774    3.7096 H   0  0  0  0  0  0  0  0  0  0  0  0
   -4.2762  -10.0041    1.5565 H   0  0  0  0  0  0  0  0  0  0  0  0
   -1.5317   -8.6783    5.2472 H   0  0  0  0  0  0  0  0  0  0  0  0
  1  2  1  0  0  0  0
  1 11  1  0  0  0  0
  1 17  2  0  0  0  0
  1 47  1  0  0  0  0
```

```
  2  4  1  0  0  0  0
  2  7  1  0  0  0  0
  2 12  1  0  0  0  0
  3  5  1  0  0  0  0
  3  9  1  0  0  0  0
  3 13  1  0  0  0  0
  3 19  1  0  0  0  0
  4  6  1  0  0  0  0
  4  8  1  0  0  0  0
  4 14  1  0  0  0  0
  5  6  1  0  0  0  0
  5 10  1  0  0  0  0
  5 15  1  0  0  0  0
  6 16  1  0  0  0  0
  6 44  1  0  0  0  0
 17 18  1  0  0  0  0
 17 19  1  0  0  0  0
 17 47  1  0  0  0  0
 19 20  1  0  0  0  0
 19 21  1  0  0  0  0
 22 23  1  0  0  0  0
 22 32  1  0  0  0  0
 22 38  2  0  0  0  0
 22 47  1  0  0  0  0
 23 25  1  0  0  0  0
 23 28  1  0  0  0  0
 23 33  1  0  0  0  0
 24 26  1  0  0  0  0
 24 30  1  0  0  0  0
 24 34  1  0  0  0  0
 24 40  1  0  0  0  0
 25 27  1  0  0  0  0
 25 29  1  0  0  0  0
 25 35  1  0  0  0  0
 26 27  1  0  0  0  0
 26 31  1  0  0  0  0
 26 36  1  0  0  0  0
 27 37  1  0  0  0  0
 27 43  1  0  0  0  0
 38 39  1  0  0  0  0
 38 40  1  0  0  0  0
 38 47  1  0  0  0  0
 40 41  1  0  0  0  0
 40 42  1  0  0  0  0
 45 46  3  0  0  0  0
 45 67  1  0  0  0  0
 46 47  1  0  0  0  0
 47 49  1  0  0  0  0
 48 49  3  0  0  0  0
 48 50  1  0  0  0  0
 50 51  1  0  0  0  0
 50 52  2  0  0  0  0
 51 53  1  0  0  0  0
 51 54  2  0  0  0  0
 52 55  1  0  0  0  0
 52 56  1  0  0  0  0
 53 57  2  0  0  0  0
 53 58  1  0  0  0  0
 54 59  1  0  0  0  0
 54 60  1  0  0  0  0
 55 60  2  0  0  0  0
 55 61  1  0  0  0  0
 57 62  1  0  0  0  0
 57 63  1  0  0  0  0
 59 62  2  0  0  0  0
```

```
 59 64  1  0  0  0  0
 60 65  1  0  0  0  0
 62 66  1  0  0  0  0
 67 68  1  0  0  0  0
 67 69  2  0  0  0  0
 68 70  1  0  0  0  0
 68 71  2  0  0  0  0
 69 72  1  0  0  0  0
 69 73  1  0  0  0  0
 70 74  2  0  0  0  0
 70 75  1  0  0  0  0
 71 76  1  0  0  0  0
 71 77  1  0  0  0  0
 72 77  2  0  0  0  0
 72 78  1  0  0  0  0
 74 79  1  0  0  0  0
 74 80  1  0  0  0  0
 76 79  2  0  0  0  0
 76 81  1  0  0  0  0
 77 82  1  0  0  0  0
 79 83  1  0  0  0  0
M  END
$$$$
mol134
Optimized Catalyst 134
Optimized Catalyst 134
 83 90  0  0  0  0  0  0  0  0999 V2000
   -1.6103    0.2844   -0.1298 C   0  0  0  0  0  0  0  0  0  0  0  0
   -1.2252    1.6428    0.3739 C   0  0  0  0  0  0  0  0  0  0  0  0
   -2.0223    1.5645   -3.0959 C   0  0  0  0  0  0  0  0  0  0  0  0
   -1.9838    2.8126   -0.2885 C   0  0  0  0  0  0  0  0  0  0  0  0
   -1.7245    3.0543   -2.8670 C   0  0  0  0  0  0  0  0  0  0  0  0
   -1.2359    3.4583   -1.4663 C   0  0  0  0  0  0  0  0  0  0  0  0
   -0.1210    1.7772    0.2276 H   0  0  0  0  0  0  0  0  0  0  0  0
   -2.9903    2.4668   -0.6184 H   0  0  0  0  0  0  0  0  0  0  0  0
   -3.0004    1.3132   -2.6197 H   0  0  0  0  0  0  0  0  0  0  0  0
   -2.6651    3.6201   -3.0904 H   0  0  0  0  0  0  0  0  0  0  0  0
   -2.0491   -0.3784    0.6426 H   0  0  0  0  0  0  0  0  0  0  0  0
   -1.4224    1.6848    1.4772 H   0  0  0  0  0  0  0  0  0  0  0  0
   -2.1298    1.4066   -4.1985 H   0  0  0  0  0  0  0  0  0  0  0  0
   -2.1363    3.5983    0.4956 H   0  0  0  0  0  0  0  0  0  0  0  0
   -0.9468    3.3818   -3.6029 H   0  0  0  0  0  0  0  0  0  0  0  0
   -1.3571    4.5709   -1.3865 H   0  0  0  0  0  0  0  0  0  0  0  0
   -1.4358   -0.1685   -1.3814 C   0  0  0  0  0  0  0  0  0  0  0  0
   -1.7573   -1.2847   -1.6577 H   0  0  0  0  0  0  0  0  0  0  0  0
   -0.9368    0.6016   -2.5658 C   0  0  0  0  0  0  0  0  0  0  0  0
   -0.0295    1.1886   -2.2630 H   0  0  0  0  0  0  0  0  0  0  0  0
   -0.6302   -0.0873   -3.3894 H   0  0  0  0  0  0  0  0  0  0  0  0
    0.5349   -3.4676   -1.5157 C   0  0  0  0  0  0  0  0  0  0  0  0
    1.1745   -2.9008   -2.7564 C   0  0  0  0  0  0  0  0  0  0  0  0
    2.7739   -1.9277    0.2691 C   0  0  0  0  0  0  0  0  0  0  0  0
    2.7197   -2.8683   -2.6560 C   0  0  0  0  0  0  0  0  0  0  0  0
    3.7100   -1.3357   -0.7954 C   0  0  0  0  0  0  0  0  0  0  0  0
    3.2909   -1.4949   -2.2669 C   0  0  0  0  0  0  0  0  0  0  0  0
    0.7977   -1.8673   -2.9602 H   0  0  0  0  0  0  0  0  0  0  0  0
    3.0647   -3.6427   -1.9310 H   0  0  0  0  0  0  0  0  0  0  0  0
    2.9178   -3.0358    0.3009 H   0  0  0  0  0  0  0  0  0  0  0  0
    4.7118   -1.8201   -0.6596 H   0  0  0  0  0  0  0  0  0  0  0  0
    0.1915   -4.5198   -1.6294 H   0  0  0  0  0  0  0  0  0  0  0  0
    0.9002   -3.5423   -3.6352 H   0  0  0  0  0  0  0  0  0  0  0  0
    3.0865   -1.5202    1.2637 H   0  0  0  0  0  0  0  0  0  0  0  0
    3.1271   -3.1436   -3.6620 H   0  0  0  0  0  0  0  0  0  0  0  0
    3.8327   -0.2409   -0.5964 H   0  0  0  0  0  0  0  0  0  0  0  0
    4.2087   -1.3152   -2.8876 H   0  0  0  0  0  0  0  0  0  0  0  0
    0.5410   -2.8857   -0.2815 C   0  0  0  0  0  0  0  0  0  0  0  0
```

```
    0.2080   -3.4654    0.6108 H   0  0  0  0  0  0  0  0  0  0  0  0
    1.2736   -1.6111    0.0599 C   0  0  0  0  0  0  0  0  0  0  0  0
    1.1693   -0.8486   -0.7519 H   0  0  0  0  0  0  0  0  0  0  0  0
    0.8609   -1.1734    1.0031 H   0  0  0  0  0  0  0  0  0  0  0  0
    2.5619   -0.6928   -2.5301 H   0  0  0  0  0  0  0  0  0  0  0  0
   -0.1438    3.2457   -1.3821 H   0  0  0  0  0  0  0  0  0  0  0  0
   -2.4564   -5.6780   -0.0151 C   0  0  0  0  0  0  0  0  0  0  0  0
   -2.1962   -4.5962   -0.3734 N   0  0  0  0  0  0  0  0  0  0  0  0
   -1.8977   -2.7486   -0.9648 Ir  0  0  0  0  0  0  0  0  0  0  0  0
   -4.9985   -2.3445   -1.1240 C   0  0  0  0  0  0  0  0  0  0  0  0
   -3.8355   -2.4426   -1.0682 N   0  0  0  0  0  0  0  0  0  0  0  0
   -6.4128   -2.2287   -1.1732 C   0  0  0  0  0  0  0  0  0  0  0  0
   -7.0637   -1.7849   -2.3786 C   0  0  0  0  0  0  0  0  0  0  0  0
   -7.1533   -2.5632   -0.0423 C   0  0  0  0  0  0  0  0  0  0  0  0
   -6.3438   -1.4228   -3.5591 C   0  0  0  0  0  0  0  0  0  0  0  0
   -8.4842   -1.7101   -2.3819 C   0  0  0  0  0  0  0  0  0  0  0  0
   -8.5626   -2.4821   -0.0677 C   0  0  0  0  0  0  0  0  0  0  0  0
   -6.6547   -2.8922    0.8729 H   0  0  0  0  0  0  0  0  0  0  0  0
   -7.0170   -1.0119   -4.6836 C   0  0  0  0  0  0  0  0  0  0  0  0
   -5.2573   -1.4753   -3.5598 H   0  0  0  0  0  0  0  0  0  0  0  0
   -9.1560   -1.2760   -3.5717 C   0  0  0  0  0  0  0  0  0  0  0  0
   -9.2149   -2.0669   -1.2097 C   0  0  0  0  0  0  0  0  0  0  0  0
   -9.1271   -2.7535    0.8285 H   0  0  0  0  0  0  0  0  0  0  0  0
   -8.4411   -0.9373   -4.6913 C   0  0  0  0  0  0  0  0  0  0  0  0
   -6.4739   -0.7353   -5.5912 H   0  0  0  0  0  0  0  0  0  0  0  0
  -10.2473   -1.2210   -3.5667 H   0  0  0  0  0  0  0  0  0  0  0  0
  -10.3082   -2.0058   -1.2298 H   0  0  0  0  0  0  0  0  0  0  0  0
   -8.9464   -0.6069   -5.6020 H   0  0  0  0  0  0  0  0  0  0  0  0
   -2.7552   -7.0005    0.4056 C   0  0  0  0  0  0  0  0  0  0  0  0
   -2.3573   -7.4495    1.7152 C   0  0  0  0  0  0  0  0  0  0  0  0
   -3.4322   -7.8504   -0.4654 C   0  0  0  0  0  0  0  0  0  0  0  0
   -1.6592   -6.6133    2.6407 C   0  0  0  0  0  0  0  0  0  0  0  0
   -2.6779   -8.7833    2.0909 C   0  0  0  0  0  0  0  0  0  0  0  0
   -3.7395   -9.1712   -0.0723 C   0  0  0  0  0  0  0  0  0  0  0  0
   -3.7317   -7.5077   -1.4592 H   0  0  0  0  0  0  0  0  0  0  0  0
   -1.3028   -7.0936    3.8775 C   0  0  0  0  0  0  0  0  0  0  0  0
   -1.4135   -5.5925    2.3578 H   0  0  0  0  0  0  0  0  0  0  0  0
   -2.2939   -9.2527    3.3901 C   0  0  0  0  0  0  0  0  0  0  0  0
   -3.3711   -9.6296    1.1752 C   0  0  0  0  0  0  0  0  0  0  0  0
   -4.2702   -9.8243   -0.7706 H   0  0  0  0  0  0  0  0  0  0  0  0
   -1.6244   -8.4296    4.2584 C   0  0  0  0  0  0  0  0  0  0  0  0
   -0.7676   -6.4608    4.5904 H   0  0  0  0  0  0  0  0  0  0  0  0
   -2.5460  -10.2777    3.6718 H   0  0  0  0  0  0  0  0  0  0  0  0
   -3.6067  -10.6554    1.4781 H   0  0  0  0  0  0  0  0  0  0  0  0
   -1.3281   -8.7808    5.2499 H   0  0  0  0  0  0  0  0  0  0  0  0
  1  2  1  0  0  0  0
  1 11  1  0  0  0  0
  1 17  2  0  0  0  0
  1 47  1  0  0  0  0
  2  4  1  0  0  0  0
  2  7  1  0  0  0  0
  2 12  1  0  0  0  0
  3  5  1  0  0  0  0
  3  9  1  0  0  0  0
  3 13  1  0  0  0  0
  3 19  1  0  0  0  0
  4  6  1  0  0  0  0
  4  8  1  0  0  0  0
  4 14  1  0  0  0  0
  5  6  1  0  0  0  0
  5 10  1  0  0  0  0
  5 15  1  0  0  0  0
  6 16  1  0  0  0  0
  6 44  1  0  0  0  0
 17 19  1  0  0  0  0
```

```
 17 18  1  0  0  0  0
 17 47  1  0  0  0  0
 19 20  1  0  0  0  0
 19 21  1  0  0  0  0
 22 23  1  0  0  0  0
 22 32  1  0  0  0  0
 22 38  2  0  0  0  0
 22 47  1  0  0  0  0
 23 25  1  0  0  0  0
 23 28  1  0  0  0  0
 23 33  1  0  0  0  0
 24 26  1  0  0  0  0
 24 30  1  0  0  0  0
 24 34  1  0  0  0  0
 24 40  1  0  0  0  0
 25 27  1  0  0  0  0
 25 29  1  0  0  0  0
 25 35  1  0  0  0  0
 26 27  1  0  0  0  0
 26 31  1  0  0  0  0
 26 36  1  0  0  0  0
 27 37  1  0  0  0  0
 27 43  1  0  0  0  0
 38 39  1  0  0  0  0
 38 40  1  0  0  0  0
 38 47  1  0  0  0  0
 40 41  1  0  0  0  0
 40 42  1  0  0  0  0
 45 46  3  0  0  0  0
 45 67  1  0  0  0  0
 46 47  1  0  0  0  0
 47 49  1  0  0  0  0
 48 49  3  0  0  0  0
 48 50  1  0  0  0  0
 50 51  1  0  0  0  0
 50 52  2  0  0  0  0
 51 53  1  0  0  0  0
 51 54  2  0  0  0  0
 52 55  1  0  0  0  0
 52 56  1  0  0  0  0
 53 57  2  0  0  0  0
 53 58  1  0  0  0  0
 54 59  1  0  0  0  0
 54 60  1  0  0  0  0
 55 60  2  0  0  0  0
 55 61  1  0  0  0  0
 57 62  1  0  0  0  0
 57 63  1  0  0  0  0
 59 62  2  0  0  0  0
 59 64  1  0  0  0  0
 60 65  1  0  0  0  0
 62 66  1  0  0  0  0
 67 68  1  0  0  0  0
 67 69  2  0  0  0  0
 68 70  1  0  0  0  0
 68 71  2  0  0  0  0
 69 72  1  0  0  0  0
 69 73  1  0  0  0  0
 70 74  2  0  0  0  0
 70 75  1  0  0  0  0
 71 76  1  0  0  0  0
 71 77  1  0  0  0  0
 72 77  2  0  0  0  0
 72 78  1  0  0  0  0
 74 79  1  0  0  0  0
```

```
 74 80  1  0  0  0  0
 76 79  2  0  0  0  0
 76 81  1  0  0  0  0
 77 82  1  0  0  0  0
 79 83  1  0  0  0  0
M  END
$$$$
mol135
Optimized Catalyst 135
Optimized Catalyst 135
 89 96  0  0  0  0  0  0  0  0999 V2000
   -1.6686   -0.2654   -0.6269 C   0  0  0  0  0  0  0  0  0  0  0  0
   -1.2545    0.9285    0.1872 C   0  0  0  0  0  0  0  0  0  0  0  0
   -1.9028    1.6858   -3.2356 C   0  0  0  0  0  0  0  0  0  0  0  0
   -1.9850    2.2306   -0.2114 C   0  0  0  0  0  0  0  0  0  0  0  0
   -1.6043    3.0738   -2.6468 C   0  0  0  0  0  0  0  0  0  0  0  0
   -1.1834    3.1302   -1.1679 C   0  0  0  0  0  0  0  0  0  0  0  0
   -0.1494    1.0746    0.0919 H   0  0  0  0  0  0  0  0  0  0  0  0
   -2.9695    1.9762   -0.6686 H   0  0  0  0  0  0  0  0  0  0  0  0
   -2.9156    1.3583   -2.8956 H   0  0  0  0  0  0  0  0  0  0  0  0
   -2.5269    3.6948   -2.7750 H   0  0  0  0  0  0  0  0  0  0  0  0
   -2.1925   -1.0590   -0.0395 H   0  0  0  0  0  0  0  0  0  0  0  0
   -1.4755    0.7223    1.2647 H   0  0  0  0  0  0  0  0  0  0  0  0
   -1.9358    1.7892   -4.3484 H   0  0  0  0  0  0  0  0  0  0  0  0
   -2.1832    2.8091    0.7251 H   0  0  0  0  0  0  0  0  0  0  0  0
   -0.7865    3.5462   -3.2470 H   0  0  0  0  0  0  0  0  0  0  0  0
   -1.3076    4.1924   -0.8324 H   0  0  0  0  0  0  0  0  0  0  0  0
   -1.4918   -0.4240   -1.9516 C   0  0  0  0  0  0  0  0  0  0  0  0
   -1.8984   -1.3272   -2.4689 H   0  0  0  0  0  0  0  0  0  0  0  0
   -0.8749    0.5914   -2.8734 C   0  0  0  0  0  0  0  0  0  0  0  0
    0.0169    1.0577   -2.3895 H   0  0  0  0  0  0  0  0  0  0  0  0
   -0.5335    0.0886   -3.8102 H   0  0  0  0  0  0  0  0  0  0  0  0
    0.7590   -3.7217   -1.4184 C   0  0  0  0  0  0  0  0  0  0  0  0
    1.1698   -2.9561   -2.6528 C   0  0  0  0  0  0  0  0  0  0  0  0
    2.7732   -1.7535    0.2931 C   0  0  0  0  0  0  0  0  0  0  0  0
    2.6687   -2.5781   -2.6725 C   0  0  0  0  0  0  0  0  0  0  0  0
    3.4622   -0.9344   -0.8098 C   0  0  0  0  0  0  0  0  0  0  0  0
    2.9582   -1.1274   -2.2510 C   0  0  0  0  0  0  0  0  0  0  0  0
    0.5278   -2.0261   -2.7280 H   0  0  0  0  0  0  0  0  0  0  0  0
    3.2354   -3.2815   -2.0175 H   0  0  0  0  0  0  0  0  0  0  0  0
    3.1653   -2.8002    0.2649 H   0  0  0  0  0  0  0  0  0  0  0  0
    4.5524   -1.1914   -0.7801 H   0  0  0  0  0  0  0  0  0  0  0  0
    0.6481   -4.8130   -1.5571 H   0  0  0  0  0  0  0  0  0  0  0  0
    0.9467   -3.5859   -3.5531 H   0  0  0  0  0  0  0  0  0  0  0  0
    3.0653   -1.3114    1.2787 H   0  0  0  0  0  0  0  0  0  0  0  0
    3.0388   -2.7174   -3.7195 H   0  0  0  0  0  0  0  0  0  0  0  0
    3.3613    0.1524   -0.5619 H   0  0  0  0  0  0  0  0  0  0  0  0
    3.7533   -0.7219   -2.9306 H   0  0  0  0  0  0  0  0  0  0  0  0
    0.7544   -3.1835   -0.1157 C   0  0  0  0  0  0  0  0  0  0  0  0
    0.6365   -3.8554    0.7539 H   0  0  0  0  0  0  0  0  0  0  0  0
    1.2320   -1.7902    0.2101 C   0  0  0  0  0  0  0  0  0  0  0  0
    0.8671   -1.0758   -0.5809 H   0  0  0  0  0  0  0  0  0  0  0  0
    0.7887   -1.4706    1.1872 H   0  0  0  0  0  0  0  0  0  0  0  0
    2.0475   -0.5019   -2.4074 H   0  0  0  0  0  0  0  0  0  0  0  0
   -0.0973    2.8883   -1.0892 H   0  0  0  0  0  0  0  0  0  0  0  0
   -2.1201   -5.4020    0.0101 C   0  0  0  0  0  0  0  0  0  0  0  0
   -1.6206   -4.4217   -0.3714 N   0  0  0  0  0  0  0  0  0  0  0  0
   -1.1137   -2.9441   -0.9756 Rh  0  0  0  0  0  0  0  0  0  0  0  0
   -4.8837   -2.6268   -1.2873 C   0  0  0  0  0  0  0  0  0  0  0  0
   -3.7310   -2.7624   -1.2958 N   0  0  0  0  0  0  0  0  0  0  0  0
   -6.3008   -2.4681   -1.2515 C   0  0  0  0  0  0  0  0  0  0  0  0
   -7.0080   -1.9481   -2.3920 C   0  0  0  0  0  0  0  0  0  0  0  0
   -6.9881   -2.8197   -0.0997 C   0  0  0  0  0  0  0  0  0  0  0  0
   -6.3243   -1.5724   -3.5912 C   0  0  0  0  0  0  0  0  0  0  0  0
   -8.4208   -1.8074   -2.3174 C   0  0  0  0  0  0  0  0  0  0  0  0
```

```
   -8.3893   -2.6724   -0.0329 C   0  0  0  0  0  0  0  0  0  0  0  0
   -6.4538   -3.2134    0.7686 H   0  0  0  0  0  0  0  0  0  0  0  0
   -7.0215   -1.0839   -4.6679 C   0  0  0  0  0  0  0  0  0  0  0  0
   -5.2415   -1.6788   -3.6400 H   0  0  0  0  0  0  0  0  0  0  0  0
   -9.1135   -1.2949   -3.4620 C   0  0  0  0  0  0  0  0  0  0  0  0
   -9.1100   -2.1791   -1.1079 C   0  0  0  0  0  0  0  0  0  0  0  0
   -8.8972   -2.9564    0.8901 H   0  0  0  0  0  0  0  0  0  0  0  0
   -8.4368   -0.9443   -4.6028 C   0  0  0  0  0  0  0  0  0  0  0  0
   -6.5056   -0.7969   -5.5861 H   0  0  0  0  0  0  0  0  0  0  0  0
  -10.1980   -1.1839   -3.4216 H   0  0  0  0  0  0  0  0  0  0  0  0
   -8.9692   -0.5557   -5.4723 H   0  0  0  0  0  0  0  0  0  0  0  0
   -2.6999   -6.6211    0.4600 C   0  0  0  0  0  0  0  0  0  0  0  0
   -2.3737   -7.1503    1.7593 C   0  0  0  0  0  0  0  0  0  0  0  0
   -3.5773   -7.2968   -0.3753 C   0  0  0  0  0  0  0  0  0  0  0  0
   -1.4819   -6.4682    2.6452 C   0  0  0  0  0  0  0  0  0  0  0  0
   -2.9569   -8.3821    2.1632 C   0  0  0  0  0  0  0  0  0  0  0  0
   -4.1566   -8.5146    0.0387 C   0  0  0  0  0  0  0  0  0  0  0  0
   -3.8264   -6.8961   -1.3606 H   0  0  0  0  0  0  0  0  0  0  0  0
   -1.1796   -6.9966    3.8758 C   0  0  0  0  0  0  0  0  0  0  0  0
   -1.0490   -5.5206    2.3319 H   0  0  0  0  0  0  0  0  0  0  0  0
   -2.6168   -8.9028    3.4540 C   0  0  0  0  0  0  0  0  0  0  0  0
   -3.8632   -9.0660    1.2748 C   0  0  0  0  0  0  0  0  0  0  0  0
   -4.8440   -9.0185   -0.6436 H   0  0  0  0  0  0  0  0  0  0  0  0
   -1.7537   -8.2332    4.2847 C   0  0  0  0  0  0  0  0  0  0  0  0
   -0.4996   -6.4807    4.5564 H   0  0  0  0  0  0  0  0  0  0  0  0
   -3.0538   -9.8489    3.7781 H   0  0  0  0  0  0  0  0  0  0  0  0
   -1.4964   -8.6358    5.2662 H   0  0  0  0  0  0  0  0  0  0  0  0
   -4.4863  -10.3616    1.6762 C   0  0  0  0  0  0  0  0  0  0  0  0
   -3.6949  -11.1268    1.8743 H   0  0  0  0  0  0  0  0  0  0  0  0
   -5.1023  -10.2257    2.5995 H   0  0  0  0  0  0  0  0  0  0  0  0
   -5.1545  -10.7638    0.8765 H   0  0  0  0  0  0  0  0  0  0  0  0
  -10.5923   -2.0362   -1.0041 C   0  0  0  0  0  0  0  0  0  0  0  0
  -10.8882   -0.9673   -1.1468 H   0  0  0  0  0  0  0  0  0  0  0  0
  -11.0961   -2.6628   -1.7811 H   0  0  0  0  0  0  0  0  0  0  0  0
  -10.9705   -2.3630   -0.0056 H   0  0  0  0  0  0  0  0  0  0  0  0
  1  2  1  0  0  0  0
  1 11  1  0  0  0  0
  1 17  2  0  0  0  0
  1 47  1  0  0  0  0
  2  4  1  0  0  0  0
  2  7  1  0  0  0  0
  2 12  1  0  0  0  0
  3  5  1  0  0  0  0
  3  9  1  0  0  0  0
  3 13  1  0  0  0  0
  3 19  1  0  0  0  0
  4  6  1  0  0  0  0
  4  8  1  0  0  0  0
  4 14  1  0  0  0  0
  5  6  1  0  0  0  0
  5 10  1  0  0  0  0
  5 15  1  0  0  0  0
  6 16  1  0  0  0  0
  6 44  1  0  0  0  0
 17 18  1  0  0  0  0
 17 19  1  0  0  0  0
 17 47  1  0  0  0  0
 19 20  1  0  0  0  0
 19 21  1  0  0  0  0
 22 23  1  0  0  0  0
 22 32  1  0  0  0  0
 22 38  2  0  0  0  0
 22 47  1  0  0  0  0
 23 25  1  0  0  0  0
 23 28  1  0  0  0  0
```

```
 23 33  1  0  0  0  0
 24 26  1  0  0  0  0
 24 30  1  0  0  0  0
 24 34  1  0  0  0  0
 24 40  1  0  0  0  0
 25 27  1  0  0  0  0
 25 29  1  0  0  0  0
 25 35  1  0  0  0  0
 26 27  1  0  0  0  0
 26 31  1  0  0  0  0
 26 36  1  0  0  0  0
 27 37  1  0  0  0  0
 27 43  1  0  0  0  0
 38 39  1  0  0  0  0
 38 40  1  0  0  0  0
 38 47  1  0  0  0  0
 40 41  1  0  0  0  0
 40 42  1  0  0  0  0
 45 46  3  0  0  0  0
 45 66  1  0  0  0  0
 46 47  1  0  0  0  0
 47 49  1  0  0  0  0
 48 49  3  0  0  0  0
 48 50  1  0  0  0  0
 50 51  1  0  0  0  0
 50 52  2  0  0  0  0
 51 53  1  0  0  0  0
 51 54  2  0  0  0  0
 52 55  1  0  0  0  0
 52 56  1  0  0  0  0
 53 57  2  0  0  0  0
 53 58  1  0  0  0  0
 54 59  1  0  0  0  0
 54 60  1  0  0  0  0
 55 60  2  0  0  0  0
 55 61  1  0  0  0  0
 57 62  1  0  0  0  0
 57 63  1  0  0  0  0
 59 62  2  0  0  0  0
 59 64  1  0  0  0  0
 60 86  1  0  0  0  0
 62 65  1  0  0  0  0
 66 67  1  0  0  0  0
 66 68  2  0  0  0  0
 67 69  1  0  0  0  0
 67 70  2  0  0  0  0
 68 71  1  0  0  0  0
 68 72  1  0  0  0  0
 69 73  2  0  0  0  0
 69 74  1  0  0  0  0
 70 75  1  0  0  0  0
 70 76  1  0  0  0  0
 71 76  2  0  0  0  0
 71 77  1  0  0  0  0
 73 78  1  0  0  0  0
 73 79  1  0  0  0  0
 75 78  2  0  0  0  0
 75 80  1  0  0  0  0
 76 82  1  0  0  0  0
 78 81  1  0  0  0  0
 82 83  1  0  0  0  0
 82 84  1  0  0  0  0
 82 85  1  0  0  0  0
 86 87  1  0  0  0  0
 86 88  1  0  0  0  0
```

```
 86 89  1  0  0  0  0
M  END
$$$$
mol136
Optimized Catalyst 136
Optimized Catalyst 136
 89 96  0  0  0  0  0  0  0  0999 V2000
   -1.5914    0.2892   -0.1413 C   0  0  0  0  0  0  0  0  0  0  0  0
   -1.2193    1.6504    0.3647 C   0  0  0  0  0  0  0  0  0  0  0  0
   -2.0060    1.5672   -3.1069 C   0  0  0  0  0  0  0  0  0  0  0  0
   -1.9909    2.8114   -0.2980 C   0  0  0  0  0  0  0  0  0  0  0  0
   -1.7229    3.0596   -2.8759 C   0  0  0  0  0  0  0  0  0  0  0  0
   -1.2465    3.4681   -1.4720 C   0  0  0  0  0  0  0  0  0  0  0  0
   -0.1169    1.7968    0.2176 H   0  0  0  0  0  0  0  0  0  0  0  0
   -2.9905    2.4522   -0.6339 H   0  0  0  0  0  0  0  0  0  0  0  0
   -2.9829    1.3053   -2.6329 H   0  0  0  0  0  0  0  0  0  0  0  0
   -2.6665    3.6176   -3.1058 H   0  0  0  0  0  0  0  0  0  0  0  0
   -2.0278   -0.3781    0.6290 H   0  0  0  0  0  0  0  0  0  0  0  0
   -1.4165    1.6894    1.4680 H   0  0  0  0  0  0  0  0  0  0  0  0
   -2.1090    1.4093   -4.2099 H   0  0  0  0  0  0  0  0  0  0  0  0
   -2.1590    3.5933    0.4866 H   0  0  0  0  0  0  0  0  0  0  0  0
   -0.9434    3.3933   -3.6071 H   0  0  0  0  0  0  0  0  0  0  0  0
   -1.3822    4.5787   -1.3911 H   0  0  0  0  0  0  0  0  0  0  0  0
   -1.4089   -0.1603   -1.3932 C   0  0  0  0  0  0  0  0  0  0  0  0
   -1.7213   -1.2784   -1.6746 H   0  0  0  0  0  0  0  0  0  0  0  0
   -0.9121    0.6149   -2.5751 C   0  0  0  0  0  0  0  0  0  0  0  0
   -0.0107    1.2098   -2.2699 H   0  0  0  0  0  0  0  0  0  0  0  0
   -0.5972   -0.0713   -3.3983 H   0  0  0  0  0  0  0  0  0  0  0  0
    0.5503   -3.4703   -1.5086 C   0  0  0  0  0  0  0  0  0  0  0  0
    1.1893   -2.9095   -2.7524 C   0  0  0  0  0  0  0  0  0  0  0  0
    2.7976   -1.9248    0.2714 C   0  0  0  0  0  0  0  0  0  0  0  0
    2.7344   -2.8785   -2.6542 C   0  0  0  0  0  0  0  0  0  0  0  0
    3.7302   -1.3402   -0.8002 C   0  0  0  0  0  0  0  0  0  0  0  0
    3.3070   -1.5044   -2.2700 C   0  0  0  0  0  0  0  0  0  0  0  0
    0.8129   -1.8765   -2.9605 H   0  0  0  0  0  0  0  0  0  0  0  0
    3.0793   -3.6498   -1.9257 H   0  0  0  0  0  0  0  0  0  0  0  0
    2.9439   -3.0320    0.3135 H   0  0  0  0  0  0  0  0  0  0  0  0
    4.7317   -1.8248   -0.6658 H   0  0  0  0  0  0  0  0  0  0  0  0
    0.2027   -4.5219   -1.6185 H   0  0  0  0  0  0  0  0  0  0  0  0
    0.9122   -3.5555   -3.6270 H   0  0  0  0  0  0  0  0  0  0  0  0
    3.1116   -1.5067    1.2612 H   0  0  0  0  0  0  0  0  0  0  0  0
    3.1393   -3.1571   -3.6603 H   0  0  0  0  0  0  0  0  0  0  0  0
    3.8549   -0.2449   -0.6062 H   0  0  0  0  0  0  0  0  0  0  0  0
    4.2230   -1.3275   -2.8940 H   0  0  0  0  0  0  0  0  0  0  0  0
    0.5633   -2.8873   -0.2753 C   0  0  0  0  0  0  0  0  0  0  0  0
    0.2269   -3.4650    0.6165 H   0  0  0  0  0  0  0  0  0  0  0  0
    1.2962   -1.6130    0.0649 C   0  0  0  0  0  0  0  0  0  0  0  0
    1.1889   -0.8497   -0.7457 H   0  0  0  0  0  0  0  0  0  0  0  0
    0.8835   -1.1786    1.0097 H   0  0  0  0  0  0  0  0  0  0  0  0
    2.5770   -0.7031   -2.5326 H   0  0  0  0  0  0  0  0  0  0  0  0
   -0.1524    3.2685   -1.3832 H   0  0  0  0  0  0  0  0  0  0  0  0
   -2.5028   -5.6398   -0.0022 C   0  0  0  0  0  0  0  0  0  0  0  0
   -2.1965   -4.5752   -0.3776 N   0  0  0  0  0  0  0  0  0  0  0  0
   -1.8760   -2.7334   -0.9786 Ir  0  0  0  0  0  0  0  0  0  0  0  0
   -4.9793   -2.3179   -1.1337 C   0  0  0  0  0  0  0  0  0  0  0  0
   -3.8150   -2.4122   -1.0821 N   0  0  0  0  0  0  0  0  0  0  0  0
   -6.3889   -2.2054   -1.1696 C   0  0  0  0  0  0  0  0  0  0  0  0
   -7.0647   -1.7692   -2.3653 C   0  0  0  0  0  0  0  0  0  0  0  0
   -7.1195   -2.5348   -0.0278 C   0  0  0  0  0  0  0  0  0  0  0  0
   -6.3448   -1.4119   -3.5465 C   0  0  0  0  0  0  0  0  0  0  0  0
   -8.4858   -1.6971   -2.3595 C   0  0  0  0  0  0  0  0  0  0  0  0
   -8.5201   -2.4599   -0.0357 C   0  0  0  0  0  0  0  0  0  0  0  0
   -6.6058   -2.8569    0.8823 H   0  0  0  0  0  0  0  0  0  0  0  0
   -7.0142   -1.0034   -4.6746 C   0  0  0  0  0  0  0  0  0  0  0  0
   -5.2582   -1.4656   -3.5467 H   0  0  0  0  0  0  0  0  0  0  0  0
```

```
   -9.1472   -1.2654   -3.5556 C   0  0  0  0  0  0  0  0  0  0  0  0
   -9.2141   -2.0548   -1.1710 C   0  0  0  0  0  0  0  0  0  0  0  0
   -9.0630   -2.7281    0.8740 H   0  0  0  0  0  0  0  0  0  0  0  0
   -8.4354   -0.9295   -4.6794 C   0  0  0  0  0  0  0  0  0  0  0  0
   -6.4697   -0.7305   -5.5820 H   0  0  0  0  0  0  0  0  0  0  0  0
  -10.2373   -1.2044   -3.5681 H   0  0  0  0  0  0  0  0  0  0  0  0
   -8.9447   -0.6030   -5.5889 H   0  0  0  0  0  0  0  0  0  0  0  0
   -2.8601   -6.9355    0.4360 C   0  0  0  0  0  0  0  0  0  0  0  0
   -2.4582   -7.4045    1.7384 C   0  0  0  0  0  0  0  0  0  0  0  0
   -3.6089   -7.7556   -0.4085 C   0  0  0  0  0  0  0  0  0  0  0  0
   -1.6835   -6.5916    2.6218 C   0  0  0  0  0  0  0  0  0  0  0  0
   -2.8467   -8.7115    2.1443 C   0  0  0  0  0  0  0  0  0  0  0  0
   -3.9867   -9.0415    0.0048 C   0  0  0  0  0  0  0  0  0  0  0  0
   -3.9085   -7.4028   -1.3995 H   0  0  0  0  0  0  0  0  0  0  0  0
   -1.3121   -7.0631    3.8580 C   0  0  0  0  0  0  0  0  0  0  0  0
   -1.3874   -5.5932    2.3085 H   0  0  0  0  0  0  0  0  0  0  0  0
   -2.4395   -9.1672    3.4408 C   0  0  0  0  0  0  0  0  0  0  0  0
   -3.6255   -9.5325    1.2551 C   0  0  0  0  0  0  0  0  0  0  0  0
   -4.5740   -9.6590   -0.6797 H   0  0  0  0  0  0  0  0  0  0  0  0
   -1.6958   -8.3690    4.2732 C   0  0  0  0  0  0  0  0  0  0  0  0
   -0.7192   -6.4462    4.5381 H   0  0  0  0  0  0  0  0  0  0  0  0
   -2.7278  -10.1685    3.7671 H   0  0  0  0  0  0  0  0  0  0  0  0
   -1.3885   -8.7207    5.2607 H   0  0  0  0  0  0  0  0  0  0  0  0
   -4.0504  -10.9023    1.6579 C   0  0  0  0  0  0  0  0  0  0  0  0
   -3.1547  -11.5441    1.8567 H   0  0  0  0  0  0  0  0  0  0  0  0
   -4.6799  -10.8592    2.5823 H   0  0  0  0  0  0  0  0  0  0  0  0
   -4.6539  -11.4037    0.8617 H   0  0  0  0  0  0  0  0  0  0  0  0
  -10.7029   -1.9991   -1.1427 C   0  0  0  0  0  0  0  0  0  0  0  0
  -11.0577   -0.9547   -1.3348 H   0  0  0  0  0  0  0  0  0  0  0  0
  -11.1321   -2.6791   -1.9215 H   0  0  0  0  0  0  0  0  0  0  0  0
  -11.1145   -2.3179   -0.1536 H   0  0  0  0  0  0  0  0  0  0  0  0
  1  2  1  0  0  0  0
  1 11  1  0  0  0  0
  1 17  2  0  0  0  0
  1 47  1  0  0  0  0
  2  4  1  0  0  0  0
  2  7  1  0  0  0  0
  2 12  1  0  0  0  0
  3  5  1  0  0  0  0
  3  9  1  0  0  0  0
  3 13  1  0  0  0  0
  3 19  1  0  0  0  0
  4  6  1  0  0  0  0
  4  8  1  0  0  0  0
  4 14  1  0  0  0  0
  5  6  1  0  0  0  0
  5 10  1  0  0  0  0
  5 15  1  0  0  0  0
  6 16  1  0  0  0  0
  6 44  1  0  0  0  0
 17 19  1  0  0  0  0
 17 18  1  0  0  0  0
 17 47  1  0  0  0  0
 19 20  1  0  0  0  0
 19 21  1  0  0  0  0
 22 23  1  0  0  0  0
 22 32  1  0  0  0  0
 22 38  2  0  0  0  0
 22 47  1  0  0  0  0
 23 25  1  0  0  0  0
 23 28  1  0  0  0  0
 23 33  1  0  0  0  0
 24 26  1  0  0  0  0
 24 30  1  0  0  0  0
 24 34  1  0  0  0  0
```

```
 24 40  1  0  0  0  0
 25 27  1  0  0  0  0
 25 29  1  0  0  0  0
 25 35  1  0  0  0  0
 26 27  1  0  0  0  0
 26 31  1  0  0  0  0
 26 36  1  0  0  0  0
 27 37  1  0  0  0  0
 27 43  1  0  0  0  0
 38 39  1  0  0  0  0
 38 40  1  0  0  0  0
 38 47  1  0  0  0  0
 40 41  1  0  0  0  0
 40 42  1  0  0  0  0
 45 46  3  0  0  0  0
 45 66  1  0  0  0  0
 46 47  1  0  0  0  0
 47 49  1  0  0  0  0
 48 49  3  0  0  0  0
 48 50  1  0  0  0  0
 50 51  2  0  0  0  0
 50 52  1  0  0  0  0
 51 53  1  0  0  0  0
 51 54  1  0  0  0  0
 52 55  2  0  0  0  0
 52 56  1  0  0  0  0
 53 57  2  0  0  0  0
 53 58  1  0  0  0  0
 54 59  1  0  0  0  0
 54 60  2  0  0  0  0
 55 60  1  0  0  0  0
 55 61  1  0  0  0  0
 57 62  1  0  0  0  0
 57 63  1  0  0  0  0
 59 62  2  0  0  0  0
 59 64  1  0  0  0  0
 60 86  1  0  0  0  0
 62 65  1  0  0  0  0
 66 67  2  0  0  0  0
 66 68  1  0  0  0  0
 67 69  1  0  0  0  0
 67 70  1  0  0  0  0
 68 71  2  0  0  0  0
 68 72  1  0  0  0  0
 69 73  2  0  0  0  0
 69 74  1  0  0  0  0
 70 75  1  0  0  0  0
 70 76  2  0  0  0  0
 71 76  1  0  0  0  0
 71 77  1  0  0  0  0
 73 78  1  0  0  0  0
 73 79  1  0  0  0  0
 75 78  2  0  0  0  0
 75 80  1  0  0  0  0
 76 82  1  0  0  0  0
 78 81  1  0  0  0  0
 82 83  1  0  0  0  0
 82 84  1  0  0  0  0
 82 85  1  0  0  0  0
 86 87  1  0  0  0  0
 86 88  1  0  0  0  0
 86 89  1  0  0  0  0
M  END
$$$$
mol137
```

```
Optimized Catalyst 137
Optimized Catalyst 137
 53 58  0  0  0  0  0  0  0  0999 V2000
   -0.9815    1.0385   -0.8237 C   0  0  0  0  0  0  0  0  0  0  0  0
    0.2568    0.1266   -0.6963 C   0  0  0  0  0  0  0  0  0  0  0  0
   -2.1479    0.6148    0.0379 C   0  0  0  0  0  0  0  0  0  0  0  0
   -0.8242    0.3550    2.2156 C   0  0  0  0  0  0  0  0  0  0  0  0
   -2.0854    0.2857    1.3864 C   0  0  0  0  0  0  0  0  0  0  0  0
   -0.0643   -0.9881    2.2290 C   0  0  0  0  0  0  0  0  0  0  0  0
   -3.1356    0.7783   -0.4343 H   0  0  0  0  0  0  0  0  0  0  0  0
   -3.0184    0.1433    1.9631 H   0  0  0  0  0  0  0  0  0  0  0  0
   -1.3142    1.0338   -1.8954 H   0  0  0  0  0  0  0  0  0  0  0  0
   -0.7015    2.0912   -0.5514 H   0  0  0  0  0  0  0  0  0  0  0  0
    0.8076    0.1323   -1.6736 H   0  0  0  0  0  0  0  0  0  0  0  0
   -0.4645   -1.6092    3.0752 H   0  0  0  0  0  0  0  0  0  0  0  0
   -1.0933    0.6270    3.2700 H   0  0  0  0  0  0  0  0  0  0  0  0
   -0.1649    1.1711    1.8224 H   0  0  0  0  0  0  0  0  0  0  0  0
   -0.2033   -1.7908    0.9534 C   0  0  0  0  0  0  0  0  0  0  0  0
   -0.1067   -1.2986   -0.3473 C   0  0  0  0  0  0  0  0  0  0  0  0
   -0.2300   -2.8940    1.1124 H   0  0  0  0  0  0  0  0  0  0  0  0
   -0.1063   -2.0089   -1.2042 H   0  0  0  0  0  0  0  0  0  0  0  0
    0.9580    0.5279    0.0797 H   0  0  0  0  0  0  0  0  0  0  0  0
    1.0258   -0.8006    2.4169 H   0  0  0  0  0  0  0  0  0  0  0  0
   -3.7578   -1.6556    0.1080 N   0  0  0  0  0  0  0  0  0  0  0  0
   -4.8917   -1.8837    0.0001 C   0  0  0  0  0  0  0  0  0  0  0  0
   -2.0547   -3.9170   -0.6477 N   0  0  0  0  0  0  0  0  0  0  0  0
   -2.0440   -5.0337   -0.9561 C   0  0  0  0  0  0  0  0  0  0  0  0
   -2.0848   -1.5515    0.2027 Rh  0  0  0  0  0  0  0  0  0  0  0  0
   -6.3171   -2.1311   -0.1269 C   0  0  0  0  0  0  0  0  0  0  0  0
   -6.6178   -2.0027   -1.2089 H   0  0  0  0  0  0  0  0  0  0  0  0
   -6.5212   -3.2023    0.1622 H   0  0  0  0  0  0  0  0  0  0  0  0
   -2.0111   -6.4416   -1.3127 C   0  0  0  0  0  0  0  0  0  0  0  0
   -2.4564   -7.0351   -0.4628 H   0  0  0  0  0  0  0  0  0  0  0  0
   -0.9357   -6.7707   -1.4329 H   0  0  0  0  0  0  0  0  0  0  0  0
   -2.7397   -6.7441   -2.6036 C   0  0  0  0  0  0  0  0  0  0  0  0
   -3.7386   -7.7262   -2.6285 C   0  0  0  0  0  0  0  0  0  0  0  0
   -2.3799   -6.0770   -3.7839 C   0  0  0  0  0  0  0  0  0  0  0  0
   -4.3727   -8.0410   -3.8350 C   0  0  0  0  0  0  0  0  0  0  0  0
   -4.0227   -8.2579   -1.7215 H   0  0  0  0  0  0  0  0  0  0  0  0
   -3.0189   -6.3959   -4.9848 C   0  0  0  0  0  0  0  0  0  0  0  0
   -1.5996   -5.3184   -3.7721 H   0  0  0  0  0  0  0  0  0  0  0  0
   -4.0150   -7.3782   -5.0125 C   0  0  0  0  0  0  0  0  0  0  0  0
   -5.1457   -8.8116   -3.8558 H   0  0  0  0  0  0  0  0  0  0  0  0
   -2.7343   -5.8825   -5.9047 H   0  0  0  0  0  0  0  0  0  0  0  0
   -4.5074   -7.6302   -5.9528 H   0  0  0  0  0  0  0  0  0  0  0  0
   -7.1383   -1.1879    0.7257 C   0  0  0  0  0  0  0  0  0  0  0  0
   -7.8526   -1.6782    1.8263 C   0  0  0  0  0  0  0  0  0  0  0  0
   -7.2079    0.1727    0.3907 C   0  0  0  0  0  0  0  0  0  0  0  0
   -8.6373   -0.8065    2.5890 C   0  0  0  0  0  0  0  0  0  0  0  0
   -7.8125   -2.7338    2.0922 H   0  0  0  0  0  0  0  0  0  0  0  0
   -7.9910    1.0378    1.1588 C   0  0  0  0  0  0  0  0  0  0  0  0
   -6.6650    0.5558   -0.4711 H   0  0  0  0  0  0  0  0  0  0  0  0
   -8.7058    0.5499    2.2586 C   0  0  0  0  0  0  0  0  0  0  0  0
   -9.2014   -1.1906    3.4411 H   0  0  0  0  0  0  0  0  0  0  0  0
   -8.0519    2.0953    0.8966 H   0  0  0  0  0  0  0  0  0  0  0  0
   -9.3202    1.2276    2.8540 H   0  0  0  0  0  0  0  0  0  0  0  0
  1  2  1  0  0  0  0
  1  3  1  0  0  0  0
  1  9  1  0  0  0  0
  1 10  1  0  0  0  0
  2 11  1  0  0  0  0
  2 16  1  0  0  0  0
  2 19  1  0  0  0  0
  3  5  2  0  0  0  0
  3  7  1  0  0  0  0
```

```
  3 25  1  0  0  0  0
  4  5  1  0  0  0  0
  4  6  1  0  0  0  0
  4 13  1  0  0  0  0
  4 14  1  0  0  0  0
  5  8  1  0  0  0  0
  5 25  1  0  0  0  0
  6 12  1  0  0  0  0
  6 15  1  0  0  0  0
  6 20  1  0  0  0  0
 15 16  2  0  0  0  0
 15 17  1  0  0  0  0
 15 25  1  0  0  0  0
 16 18  1  0  0  0  0
 16 25  1  0  0  0  0
 21 22  3  0  0  0  0
 21 25  1  0  0  0  0
 22 26  1  0  0  0  0
 23 24  3  0  0  0  0
 23 25  1  0  0  0  0
 24 29  1  0  0  0  0
 26 27  1  0  0  0  0
 26 28  1  0  0  0  0
 26 43  1  0  0  0  0
 29 30  1  0  0  0  0
 29 31  1  0  0  0  0
 29 32  1  0  0  0  0
 32 33  2  0  0  0  0
 32 34  1  0  0  0  0
 33 35  1  0  0  0  0
 33 36  1  0  0  0  0
 34 37  2  0  0  0  0
 34 38  1  0  0  0  0
 35 39  2  0  0  0  0
 35 40  1  0  0  0  0
 37 39  1  0  0  0  0
 37 41  1  0  0  0  0
 39 42  1  0  0  0  0
 43 44  2  0  0  0  0
 43 45  1  0  0  0  0
 44 46  1  0  0  0  0
 44 47  1  0  0  0  0
 45 48  2  0  0  0  0
 45 49  1  0  0  0  0
 46 50  2  0  0  0  0
 46 51  1  0  0  0  0
 48 50  1  0  0  0  0
 48 52  1  0  0  0  0
 50 53  1  0  0  0  0
M  END
$$$$
mol138
Optimized Catalyst 138
Optimized Catalyst 138
 53 58  0  0  0  0  0  0  0  0999 V2000
   -0.9124    0.9316   -0.8691 C   0  0  0  0  0  0  0  0  0  0  0  0
    0.3624    0.0727   -0.7342 C   0  0  0  0  0  0  0  0  0  0  0  0
   -2.1006    0.4919   -0.0430 C   0  0  0  0  0  0  0  0  0  0  0  0
   -0.8312    0.1747    2.1485 C   0  0  0  0  0  0  0  0  0  0  0  0
   -2.0711    0.1377    1.2854 C   0  0  0  0  0  0  0  0  0  0  0  0
    0.0163   -1.1145    2.1203 C   0  0  0  0  0  0  0  0  0  0  0  0
   -3.0781    0.7197   -0.5261 H   0  0  0  0  0  0  0  0  0  0  0  0
   -3.0220    0.0294    1.8516 H   0  0  0  0  0  0  0  0  0  0  0  0
   -1.2101    0.9605   -1.9536 H   0  0  0  0  0  0  0  0  0  0  0  0
   -0.6668    1.9912   -0.5675 H   0  0  0  0  0  0  0  0  0  0  0  0
```

```
    0.9384    0.1509   -1.6981 H   0  0  0  0  0  0  0  0  0  0  0  0
   -0.3129   -1.7775    2.9673 H   0  0  0  0  0  0  0  0  0  0  0  0
   -1.1386    0.3657    3.2142 H   0  0  0  0  0  0  0  0  0  0  0  0
   -0.2041    1.0536    1.8357 H   0  0  0  0  0  0  0  0  0  0  0  0
   -0.0183   -1.9070    0.8327 C   0  0  0  0  0  0  0  0  0  0  0  0
    0.1131   -1.3881   -0.4355 C   0  0  0  0  0  0  0  0  0  0  0  0
    0.1027   -3.0027    0.9910 H   0  0  0  0  0  0  0  0  0  0  0  0
    0.2880   -2.0670   -1.2979 H   0  0  0  0  0  0  0  0  0  0  0  0
    1.0274    0.4951    0.0675 H   0  0  0  0  0  0  0  0  0  0  0  0
    1.0938   -0.8411    2.3163 H   0  0  0  0  0  0  0  0  0  0  0  0
   -4.1651   -1.8733   -0.1719 N   0  0  0  0  0  0  0  0  0  0  0  0
   -5.3273   -1.9129   -0.2597 C   0  0  0  0  0  0  0  0  0  0  0  0
   -2.0973   -3.7568   -0.6030 N   0  0  0  0  0  0  0  0  0  0  0  0
   -2.0495   -4.8847   -0.8956 C   0  0  0  0  0  0  0  0  0  0  0  0
   -2.1999   -1.8700   -0.0469 Ir  0  0  0  0  0  0  0  0  0  0  0  0
   -6.7725   -1.9371   -0.3635 C   0  0  0  0  0  0  0  0  0  0  0  0
   -7.0827   -1.5841   -1.3939 H   0  0  0  0  0  0  0  0  0  0  0  0
   -7.1373   -3.0017   -0.2521 H   0  0  0  0  0  0  0  0  0  0  0  0
   -1.9483   -6.2907   -1.2283 C   0  0  0  0  0  0  0  0  0  0  0  0
   -2.3735   -6.8937   -0.3707 H   0  0  0  0  0  0  0  0  0  0  0  0
   -0.8553   -6.5733   -1.3354 H   0  0  0  0  0  0  0  0  0  0  0  0
   -2.6496   -6.6520   -2.5209 C   0  0  0  0  0  0  0  0  0  0  0  0
   -3.5698   -7.7079   -2.5361 C   0  0  0  0  0  0  0  0  0  0  0  0
   -2.3315   -5.9745   -3.7071 C   0  0  0  0  0  0  0  0  0  0  0  0
   -4.1639   -8.0907   -3.7433 C   0  0  0  0  0  0  0  0  0  0  0  0
   -3.8212   -8.2463   -1.6229 H   0  0  0  0  0  0  0  0  0  0  0  0
   -2.9338   -6.3597   -4.9076 C   0  0  0  0  0  0  0  0  0  0  0  0
   -1.6088   -5.1616   -3.7044 H   0  0  0  0  0  0  0  0  0  0  0  0
   -3.8471   -7.4198   -4.9280 C   0  0  0  0  0  0  0  0  0  0  0  0
   -4.8717   -8.9227   -3.7592 H   0  0  0  0  0  0  0  0  0  0  0  0
   -2.6828   -5.8395   -5.8345 H   0  0  0  0  0  0  0  0  0  0  0  0
   -4.3068   -7.7268   -5.8698 H   0  0  0  0  0  0  0  0  0  0  0  0
   -7.4186   -1.0512    0.6815 C   0  0  0  0  0  0  0  0  0  0  0  0
   -8.0047   -1.6221    1.8184 C   0  0  0  0  0  0  0  0  0  0  0  0
   -7.4565    0.3374    0.4873 C   0  0  0  0  0  0  0  0  0  0  0  0
   -8.6405   -0.8007    2.7553 C   0  0  0  0  0  0  0  0  0  0  0  0
   -7.9837   -2.6994    1.9775 H   0  0  0  0  0  0  0  0  0  0  0  0
   -8.0911    1.1509    1.4294 C   0  0  0  0  0  0  0  0  0  0  0  0
   -7.0124    0.7865   -0.3990 H   0  0  0  0  0  0  0  0  0  0  0  0
   -8.6863    0.5831    2.5616 C   0  0  0  0  0  0  0  0  0  0  0  0
   -9.1080   -1.2440    3.6375 H   0  0  0  0  0  0  0  0  0  0  0  0
   -8.1307    2.2315    1.2770 H   0  0  0  0  0  0  0  0  0  0  0  0
   -9.1924    1.2206    3.2901 H   0  0  0  0  0  0  0  0  0  0  0  0
  1  2  1  0  0  0  0
  1  3  1  0  0  0  0
  1  9  1  0  0  0  0
  1 10  1  0  0  0  0
  2 11  1  0  0  0  0
  2 16  1  0  0  0  0
  2 19  1  0  0  0  0
  3  5  2  0  0  0  0
  3  7  1  0  0  0  0
  3 25  1  0  0  0  0
  4  5  1  0  0  0  0
  4  6  1  0  0  0  0
  4 13  1  0  0  0  0
  4 14  1  0  0  0  0
  5  8  1  0  0  0  0
  5 25  1  0  0  0  0
  6 12  1  0  0  0  0
  6 15  1  0  0  0  0
  6 20  1  0  0  0  0
 15 16  2  0  0  0  0
 15 17  1  0  0  0  0
 15 25  1  0  0  0  0
```

```
 16 18  1  0  0  0  0
 16 25  1  0  0  0  0
 21 22  3  0  0  0  0
 21 25  1  0  0  0  0
 22 26  1  0  0  0  0
 23 24  3  0  0  0  0
 23 25  1  0  0  0  0
 24 29  1  0  0  0  0
 26 27  1  0  0  0  0
 26 28  1  0  0  0  0
 26 43  1  0  0  0  0
 29 30  1  0  0  0  0
 29 31  1  0  0  0  0
 29 32  1  0  0  0  0
 32 33  2  0  0  0  0
 32 34  1  0  0  0  0
 33 35  1  0  0  0  0
 33 36  1  0  0  0  0
 34 37  2  0  0  0  0
 34 38  1  0  0  0  0
 35 39  2  0  0  0  0
 35 40  1  0  0  0  0
 37 39  1  0  0  0  0
 37 41  1  0  0  0  0
 39 42  1  0  0  0  0
 43 44  2  0  0  0  0
 43 45  1  0  0  0  0
 44 46  1  0  0  0  0
 44 47  1  0  0  0  0
 45 48  2  0  0  0  0
 45 49  1  0  0  0  0
 46 50  2  0  0  0  0
 46 51  1  0  0  0  0
 48 50  1  0  0  0  0
 48 52  1  0  0  0  0
 50 53  1  0  0  0  0
M  END
$$$$
mol139
Optimized Catalyst 139
Optimized Catalyst 139
 53 58  0  0  0  0  0  0  0  0999 V2000
   -1.3266    0.4949   -1.4388 C   0  0  0  0  0  0  0  0  0  0  0  0
   -0.0782   -0.4089   -1.3728 C   0  0  0  0  0  0  0  0  0  0  0  0
   -2.4035    0.1326   -0.4434 C   0  0  0  0  0  0  0  0  0  0  0  0
   -0.8802   -0.0147    1.6138 C   0  0  0  0  0  0  0  0  0  0  0  0
   -2.2133   -0.1201    0.9105 C   0  0  0  0  0  0  0  0  0  0  0  0
   -0.1168   -1.3558    1.6236 C   0  0  0  0  0  0  0  0  0  0  0  0
   -3.4317    0.2839   -0.8259 H   0  0  0  0  0  0  0  0  0  0  0  0
   -3.0881   -0.2160    1.5802 H   0  0  0  0  0  0  0  0  0  0  0  0
   -1.7608    0.4199   -2.4711 H   0  0  0  0  0  0  0  0  0  0  0  0
   -1.0267    1.5629   -1.2640 H   0  0  0  0  0  0  0  0  0  0  0  0
    0.3823   -0.4600   -2.3949 H   0  0  0  0  0  0  0  0  0  0  0  0
   -0.4244   -1.9288    2.5395 H   0  0  0  0  0  0  0  0  0  0  0  0
   -1.0499    0.3087    2.6746 H   0  0  0  0  0  0  0  0  0  0  0  0
   -0.2646    0.7811    1.1208 H   0  0  0  0  0  0  0  0  0  0  0  0
   -0.3810   -2.2286    0.4162 C   0  0  0  0  0  0  0  0  0  0  0  0
   -0.4059   -1.8107   -0.9123 C   0  0  0  0  0  0  0  0  0  0  0  0
   -0.3931   -3.3212    0.6384 H   0  0  0  0  0  0  0  0  0  0  0  0
   -0.4888   -2.5683   -1.7240 H   0  0  0  0  0  0  0  0  0  0  0  0
    0.6889    0.0356   -0.6881 H   0  0  0  0  0  0  0  0  0  0  0  0
    0.9864   -1.1593    1.6874 H   0  0  0  0  0  0  0  0  0  0  0  0
   -4.0060   -2.1028   -0.1150 N   0  0  0  0  0  0  0  0  0  0  0  0
   -5.1497   -2.3075   -0.1365 C   0  0  0  0  0  0  0  0  0  0  0  0
   -2.3968   -4.4298   -0.8628 N   0  0  0  0  0  0  0  0  0  0  0  0
```

```
   -2.5509   -5.5493   -1.1176 C   0  0  0  0  0  0  0  0  0  0  0  0
   -6.5877   -2.5209   -0.1595 C   0  0  0  0  0  0  0  0  0  0  0  0
   -6.8847   -2.8583   -1.1949 H   0  0  0  0  0  0  0  0  0  0  0  0
   -6.8491   -3.3536    0.5565 H   0  0  0  0  0  0  0  0  0  0  0  0
   -2.7522   -6.9583   -1.4153 C   0  0  0  0  0  0  0  0  0  0  0  0
   -3.8569   -7.1920   -1.3845 H   0  0  0  0  0  0  0  0  0  0  0  0
   -2.2565   -7.5722   -0.6081 H   0  0  0  0  0  0  0  0  0  0  0  0
   -2.2117   -7.3406   -2.7733 C   0  0  0  0  0  0  0  0  0  0  0  0
   -2.8515   -6.8733   -3.9334 C   0  0  0  0  0  0  0  0  0  0  0  0
   -1.0972   -8.1873   -2.8733 C   0  0  0  0  0  0  0  0  0  0  0  0
   -2.3876   -7.2474   -5.1946 C   0  0  0  0  0  0  0  0  0  0  0  0
   -3.7191   -6.2179   -3.8561 H   0  0  0  0  0  0  0  0  0  0  0  0
   -0.6171   -8.5752   -4.1259 C   0  0  0  0  0  0  0  0  0  0  0  0
   -0.5979   -8.5566   -1.9761 H   0  0  0  0  0  0  0  0  0  0  0  0
   -1.2768   -8.0963   -5.2621 C   0  0  0  0  0  0  0  0  0  0  0  0
   -2.8811   -6.8953   -6.1023 H   0  0  0  0  0  0  0  0  0  0  0  0
    0.2463   -9.2383   -4.2135 H   0  0  0  0  0  0  0  0  0  0  0  0
   -7.3472   -1.2699    0.2184 C   0  0  0  0  0  0  0  0  0  0  0  0
   -7.5408   -0.9539    1.5729 C   0  0  0  0  0  0  0  0  0  0  0  0
   -7.8786   -0.4428   -0.7835 C   0  0  0  0  0  0  0  0  0  0  0  0
   -8.2620    0.1842    1.9354 C   0  0  0  0  0  0  0  0  0  0  0  0
   -7.1340   -1.5990    2.3527 H   0  0  0  0  0  0  0  0  0  0  0  0
   -8.6023    0.7013   -0.4431 C   0  0  0  0  0  0  0  0  0  0  0  0
   -7.7372   -0.6891   -1.8368 H   0  0  0  0  0  0  0  0  0  0  0  0
   -8.7791    0.9894    0.9144 C   0  0  0  0  0  0  0  0  0  0  0  0
   -8.4258    0.4340    2.9857 H   0  0  0  0  0  0  0  0  0  0  0  0
   -9.0249    1.3483   -1.2149 H   0  0  0  0  0  0  0  0  0  0  0  0
   -0.8297   -8.4624   -6.4612 F   0  0  0  0  0  0  0  0  0  0  0  0
   -9.4680    2.0773    1.2499 F   0  0  0  0  0  0  0  0  0  0  0  0
   -2.3314   -2.0181   -0.1606 Rh  0  0  0  0  0  0  0  0  0  0  0  0
  1  2  1  0  0  0  0
  1  3  1  0  0  0  0
  1  9  1  0  0  0  0
  1 10  1  0  0  0  0
  2 11  1  0  0  0  0
  2 16  1  0  0  0  0
  2 19  1  0  0  0  0
  3  5  2  0  0  0  0
  3  7  1  0  0  0  0
  3 53  1  0  0  0  0
  4  5  1  0  0  0  0
  4  6  1  0  0  0  0
  4 13  1  0  0  0  0
  4 14  1  0  0  0  0
  5  8  1  0  0  0  0
  5 53  1  0  0  0  0
  6 12  1  0  0  0  0
  6 15  1  0  0  0  0
  6 20  1  0  0  0  0
 15 16  2  0  0  0  0
 15 17  1  0  0  0  0
 15 53  1  0  0  0  0
 16 18  1  0  0  0  0
 16 53  1  0  0  0  0
 21 22  3  0  0  0  0
 21 53  1  0  0  0  0
 22 25  1  0  0  0  0
 23 24  3  0  0  0  0
 23 53  1  0  0  0  0
 24 28  1  0  0  0  0
 25 26  1  0  0  0  0
 25 27  1  0  0  0  0
 25 41  1  0  0  0  0
 28 29  1  0  0  0  0
 28 30  1  0  0  0  0
```

```
 28 31  1  0  0  0  0
 31 32  2  0  0  0  0
 31 33  1  0  0  0  0
 32 34  1  0  0  0  0
 32 35  1  0  0  0  0
 33 36  2  0  0  0  0
 33 37  1  0  0  0  0
 34 38  2  0  0  0  0
 34 39  1  0  0  0  0
 36 38  1  0  0  0  0
 36 40  1  0  0  0  0
 38 51  1  0  0  0  0
 41 42  2  0  0  0  0
 41 43  1  0  0  0  0
 42 44  1  0  0  0  0
 42 45  1  0  0  0  0
 43 46  2  0  0  0  0
 43 47  1  0  0  0  0
 44 48  2  0  0  0  0
 44 49  1  0  0  0  0
 46 48  1  0  0  0  0
 46 50  1  0  0  0  0
 48 52  1  0  0  0  0
M  END
$$$$
mol140
Optimized Catalyst 140
Optimized Catalyst 140
 53 58  0  0  0  0  0  0  0  0999 V2000
   -0.9309    0.2784   -1.5332 C   0  0  0  0  0  0  0  0  0  0  0  0
    0.2993   -0.5891   -1.1933 C   0  0  0  0  0  0  0  0  0  0  0  0
   -2.1851   -0.0035   -0.7366 C   0  0  0  0  0  0  0  0  0  0  0  0
   -1.0662   -0.0302    1.5564 C   0  0  0  0  0  0  0  0  0  0  0  0
   -2.2518   -0.1619    0.6283 C   0  0  0  0  0  0  0  0  0  0  0  0
   -0.2682   -1.3333    1.7738 C   0  0  0  0  0  0  0  0  0  0  0  0
   -3.1209    0.1759   -1.3140 H   0  0  0  0  0  0  0  0  0  0  0  0
   -3.2385   -0.1574    1.1410 H   0  0  0  0  0  0  0  0  0  0  0  0
   -1.1588    0.1626   -2.6287 H   0  0  0  0  0  0  0  0  0  0  0  0
   -0.6637    1.3628   -1.3668 H   0  0  0  0  0  0  0  0  0  0  0  0
    0.9354   -0.6703   -2.1187 H   0  0  0  0  0  0  0  0  0  0  0  0
   -0.6778   -1.8561    2.6818 H   0  0  0  0  0  0  0  0  0  0  0  0
   -1.4307    0.3276    2.5596 H   0  0  0  0  0  0  0  0  0  0  0  0
   -0.3882    0.7747    1.1608 H   0  0  0  0  0  0  0  0  0  0  0  0
   -0.2496   -2.3045    0.6146 C   0  0  0  0  0  0  0  0  0  0  0  0
   -0.0229   -1.9820   -0.7030 C   0  0  0  0  0  0  0  0  0  0  0  0
   -0.1857   -3.3692    0.9358 H   0  0  0  0  0  0  0  0  0  0  0  0
    0.1731   -2.7858   -1.4454 H   0  0  0  0  0  0  0  0  0  0  0  0
    0.9312   -0.0743   -0.4188 H   0  0  0  0  0  0  0  0  0  0  0  0
    0.8038   -1.0643    2.0049 H   0  0  0  0  0  0  0  0  0  0  0  0
   -4.3443   -2.2899   -0.6104 N   0  0  0  0  0  0  0  0  0  0  0  0
   -5.5035   -2.3243   -0.7336 C   0  0  0  0  0  0  0  0  0  0  0  0
   -2.3362   -4.2732   -0.7135 N   0  0  0  0  0  0  0  0  0  0  0  0
   -2.3590   -5.4241   -0.9005 C   0  0  0  0  0  0  0  0  0  0  0  0
   -6.9456   -2.3599   -0.8902 C   0  0  0  0  0  0  0  0  0  0  0  0
   -7.2009   -2.2738   -1.9894 H   0  0  0  0  0  0  0  0  0  0  0  0
   -7.3342   -3.3621   -0.5360 H   0  0  0  0  0  0  0  0  0  0  0  0
   -2.3697   -6.8576   -1.1270 C   0  0  0  0  0  0  0  0  0  0  0  0
   -3.3886   -7.2716   -0.8608 H   0  0  0  0  0  0  0  0  0  0  0  0
   -1.6220   -7.3499   -0.4347 H   0  0  0  0  0  0  0  0  0  0  0  0
   -2.0443   -7.1910   -2.5648 C   0  0  0  0  0  0  0  0  0  0  0  0
   -0.7302   -7.5282   -2.9261 C   0  0  0  0  0  0  0  0  0  0  0  0
   -3.0666   -7.1889   -3.5279 C   0  0  0  0  0  0  0  0  0  0  0  0
   -0.4271   -7.8578   -4.2478 C   0  0  0  0  0  0  0  0  0  0  0  0
    0.0647   -7.5444   -2.1789 H   0  0  0  0  0  0  0  0  0  0  0  0
   -2.7841   -7.5129   -4.8548 C   0  0  0  0  0  0  0  0  0  0  0  0
```

```
   -4.0907   -6.9425   -3.2467 H   0  0  0  0  0  0  0  0  0  0  0  0
   -1.4642   -7.8412   -5.1874 C   0  0  0  0  0  0  0  0  0  0  0  0
    0.5906   -8.1297   -4.5390 H   0  0  0  0  0  0  0  0  0  0  0  0
   -3.5719   -7.5194   -5.6116 H   0  0  0  0  0  0  0  0  0  0  0  0
   -7.6259   -1.2538   -0.1167 C   0  0  0  0  0  0  0  0  0  0  0  0
   -7.9601   -1.4505    1.2333 C   0  0  0  0  0  0  0  0  0  0  0  0
   -7.9597   -0.0523   -0.7619 C   0  0  0  0  0  0  0  0  0  0  0  0
   -8.6301   -0.4551    1.9445 C   0  0  0  0  0  0  0  0  0  0  0  0
   -7.7078   -2.3848    1.7363 H   0  0  0  0  0  0  0  0  0  0  0  0
   -8.6305    0.9556   -0.0683 C   0  0  0  0  0  0  0  0  0  0  0  0
   -7.7080    0.1031   -1.8120 H   0  0  0  0  0  0  0  0  0  0  0  0
   -8.9552    0.7302    1.2743 C   0  0  0  0  0  0  0  0  0  0  0  0
   -8.9039   -0.6009    2.9924 H   0  0  0  0  0  0  0  0  0  0  0  0
   -8.9046    1.8907   -0.5631 H   0  0  0  0  0  0  0  0  0  0  0  0
   -1.1845   -8.1478   -6.4504 F   0  0  0  0  0  0  0  0  0  0  0  0
   -9.5988    1.6825    1.9424 F   0  0  0  0  0  0  0  0  0  0  0  0
   -2.3877   -2.3307   -0.3966 Ir  0  0  0  0  0  0  0  0  0  0  0  0
  1  2  1  0  0  0  0
  1  3  1  0  0  0  0
  1  9  1  0  0  0  0
  1 10  1  0  0  0  0
  2 11  1  0  0  0  0
  2 16  1  0  0  0  0
  2 19  1  0  0  0  0
  3  5  2  0  0  0  0
  3  7  1  0  0  0  0
  3 53  1  0  0  0  0
  4  5  1  0  0  0  0
  4  6  1  0  0  0  0
  4 13  1  0  0  0  0
  4 14  1  0  0  0  0
  5  8  1  0  0  0  0
  5 53  1  0  0  0  0
  6 12  1  0  0  0  0
  6 15  1  0  0  0  0
  6 20  1  0  0  0  0
 15 16  2  0  0  0  0
 15 17  1  0  0  0  0
 15 53  1  0  0  0  0
 16 18  1  0  0  0  0
 16 53  1  0  0  0  0
 21 22  3  0  0  0  0
 21 53  1  0  0  0  0
 22 25  1  0  0  0  0
 23 24  3  0  0  0  0
 23 53  1  0  0  0  0
 24 28  1  0  0  0  0
 25 26  1  0  0  0  0
 25 27  1  0  0  0  0
 25 41  1  0  0  0  0
 28 29  1  0  0  0  0
 28 30  1  0  0  0  0
 28 31  1  0  0  0  0
 31 32  2  0  0  0  0
 31 33  1  0  0  0  0
 32 34  1  0  0  0  0
 32 35  1  0  0  0  0
 33 36  2  0  0  0  0
 33 37  1  0  0  0  0
 34 38  2  0  0  0  0
 34 39  1  0  0  0  0
 36 38  1  0  0  0  0
 36 40  1  0  0  0  0
 38 51  1  0  0  0  0
 41 42  2  0  0  0  0
```

```
 41 43  1  0  0  0  0
 42 44  1  0  0  0  0
 42 45  1  0  0  0  0
 43 46  2  0  0  0  0
 43 47  1  0  0  0  0
 44 48  2  0  0  0  0
 44 49  1  0  0  0  0
 46 48  1  0  0  0  0
 46 50  1  0  0  0  0
 48 52  1  0  0  0  0
M  END
$$$$
mol141
Optimized Catalyst 141
Optimized Catalyst 141
 59 64  0  0  0  0  0  0  0  0999 V2000
   -1.1898    0.5150   -1.4382 C   0  0  0  0  0  0  0  0  0  0  0  0
    0.0527   -0.3871   -1.2907 C   0  0  0  0  0  0  0  0  0  0  0  0
   -2.3157    0.1684   -0.4927 C   0  0  0  0  0  0  0  0  0  0  0  0
   -0.8984    0.0357    1.6412 C   0  0  0  0  0  0  0  0  0  0  0  0
   -2.1945   -0.0681    0.8713 C   0  0  0  0  0  0  0  0  0  0  0  0
   -0.1587   -1.3170    1.7133 C   0  0  0  0  0  0  0  0  0  0  0  0
   -3.3234    0.3103   -0.9298 H   0  0  0  0  0  0  0  0  0  0  0  0
   -3.1029   -0.1549    1.4963 H   0  0  0  0  0  0  0  0  0  0  0  0
   -1.5705    0.4196   -2.4900 H   0  0  0  0  0  0  0  0  0  0  0  0
   -0.8993    1.5861   -1.2679 H   0  0  0  0  0  0  0  0  0  0  0  0
    0.5722   -0.4486   -2.2833 H   0  0  0  0  0  0  0  0  0  0  0  0
   -0.5500   -1.8804    2.6035 H   0  0  0  0  0  0  0  0  0  0  0  0
   -1.1194    0.3783    2.6862 H   0  0  0  0  0  0  0  0  0  0  0  0
   -0.2459    0.8144    1.1698 H   0  0  0  0  0  0  0  0  0  0  0  0
   -0.3447   -2.1937    0.4940 C   0  0  0  0  0  0  0  0  0  0  0  0
   -0.3019   -1.7844   -0.8366 C   0  0  0  0  0  0  0  0  0  0  0  0
   -0.3633   -3.2854    0.7201 H   0  0  0  0  0  0  0  0  0  0  0  0
   -0.3438   -2.5482   -1.6455 H   0  0  0  0  0  0  0  0  0  0  0  0
    0.7786    0.0632   -0.5664 H   0  0  0  0  0  0  0  0  0  0  0  0
    0.9387   -1.1391    1.8629 H   0  0  0  0  0  0  0  0  0  0  0  0
   -3.9456   -2.0620   -0.1876 N   0  0  0  0  0  0  0  0  0  0  0  0
   -5.0856   -2.2837   -0.2216 C   0  0  0  0  0  0  0  0  0  0  0  0
   -2.3029   -4.3442   -0.8532 N   0  0  0  0  0  0  0  0  0  0  0  0
   -2.4875   -5.4576   -1.1152 C   0  0  0  0  0  0  0  0  0  0  0  0
   -6.5177   -2.5286   -0.2634 C   0  0  0  0  0  0  0  0  0  0  0  0
   -6.8029   -2.7862   -1.3241 H   0  0  0  0  0  0  0  0  0  0  0  0
   -6.7558   -3.4225    0.3853 H   0  0  0  0  0  0  0  0  0  0  0  0
   -2.7275   -6.8573   -1.4217 C   0  0  0  0  0  0  0  0  0  0  0  0
   -3.8362   -7.0663   -1.3653 H   0  0  0  0  0  0  0  0  0  0  0  0
   -2.2185   -7.4872   -0.6362 H   0  0  0  0  0  0  0  0  0  0  0  0
   -2.2410   -7.2448   -2.7991 C   0  0  0  0  0  0  0  0  0  0  0  0
   -1.3728   -8.3330   -2.9549 C   0  0  0  0  0  0  0  0  0  0  0  0
   -2.6903   -6.5458   -3.9300 C   0  0  0  0  0  0  0  0  0  0  0  0
   -0.9658   -8.7257   -4.2315 C   0  0  0  0  0  0  0  0  0  0  0  0
   -1.0131   -8.8866   -2.0878 H   0  0  0  0  0  0  0  0  0  0  0  0
   -2.2765   -6.9348   -5.2028 C   0  0  0  0  0  0  0  0  0  0  0  0
   -3.3671   -5.7006   -3.8185 H   0  0  0  0  0  0  0  0  0  0  0  0
   -1.4162   -8.0358   -5.3685 C   0  0  0  0  0  0  0  0  0  0  0  0
   -0.2953   -9.5787   -4.3420 H   0  0  0  0  0  0  0  0  0  0  0  0
   -2.6257   -6.3854   -6.0770 H   0  0  0  0  0  0  0  0  0  0  0  0
   -7.3230   -1.3464    0.2252 C   0  0  0  0  0  0  0  0  0  0  0  0
   -7.2699   -0.9720    1.5770 C   0  0  0  0  0  0  0  0  0  0  0  0
   -8.1616   -0.6504   -0.6552 C   0  0  0  0  0  0  0  0  0  0  0  0
   -8.0556    0.0805    2.0426 C   0  0  0  0  0  0  0  0  0  0  0  0
   -6.6231   -1.5087    2.2699 H   0  0  0  0  0  0  0  0  0  0  0  0
   -8.9528    0.3998   -0.1849 C   0  0  0  0  0  0  0  0  0  0  0  0
   -8.2128   -0.9246   -1.7089 H   0  0  0  0  0  0  0  0  0  0  0  0
   -8.9135    0.7734    1.1681 C   0  0  0  0  0  0  0  0  0  0  0  0
   -8.0131    0.3637    3.0947 H   0  0  0  0  0  0  0  0  0  0  0  0
```

```
   -9.6110    0.9279   -0.8762 H   0  0  0  0  0  0  0  0  0  0  0  0
   -0.9879   -8.4531   -6.7371 C   0  0  0  0  0  0  0  0  0  0  0  0
   -0.5379   -9.4739   -6.7321 H   0  0  0  0  0  0  0  0  0  0  0  0
   -1.8600   -8.4634   -7.4347 H   0  0  0  0  0  0  0  0  0  0  0  0
   -0.2261   -7.7337   -7.1263 H   0  0  0  0  0  0  0  0  0  0  0  0
   -9.7586    1.8933    1.6795 C   0  0  0  0  0  0  0  0  0  0  0  0
  -10.5527    2.1767    0.9490 H   0  0  0  0  0  0  0  0  0  0  0  0
  -10.2578    1.6027    2.6359 H   0  0  0  0  0  0  0  0  0  0  0  0
   -9.1177    2.7892    1.8688 H   0  0  0  0  0  0  0  0  0  0  0  0
   -2.2657   -1.9805   -0.1788 Rh  0  0  0  0  0  0  0  0  0  0  0  0
  1  2  1  0  0  0  0
  1  3  1  0  0  0  0
  1  9  1  0  0  0  0
  1 10  1  0  0  0  0
  2 11  1  0  0  0  0
  2 16  1  0  0  0  0
  2 19  1  0  0  0  0
  3  5  2  0  0  0  0
  3  7  1  0  0  0  0
  3 59  1  0  0  0  0
  4  5  1  0  0  0  0
  4  6  1  0  0  0  0
  4 13  1  0  0  0  0
  4 14  1  0  0  0  0
  5  8  1  0  0  0  0
  5 59  1  0  0  0  0
  6 12  1  0  0  0  0
  6 15  1  0  0  0  0
  6 20  1  0  0  0  0
 15 16  2  0  0  0  0
 15 17  1  0  0  0  0
 15 59  1  0  0  0  0
 16 18  1  0  0  0  0
 16 59  1  0  0  0  0
 21 22  3  0  0  0  0
 21 59  1  0  0  0  0
 22 25  1  0  0  0  0
 23 24  3  0  0  0  0
 23 59  1  0  0  0  0
 24 28  1  0  0  0  0
 25 26  1  0  0  0  0
 25 27  1  0  0  0  0
 25 41  1  0  0  0  0
 28 29  1  0  0  0  0
 28 30  1  0  0  0  0
 28 31  1  0  0  0  0
 31 32  2  0  0  0  0
 31 33  1  0  0  0  0
 32 34  1  0  0  0  0
 32 35  1  0  0  0  0
 33 36  2  0  0  0  0
 33 37  1  0  0  0  0
 34 38  2  0  0  0  0
 34 39  1  0  0  0  0
 36 38  1  0  0  0  0
 36 40  1  0  0  0  0
 38 51  1  0  0  0  0
 41 42  2  0  0  0  0
 41 43  1  0  0  0  0
 42 44  1  0  0  0  0
 42 45  1  0  0  0  0
 43 46  2  0  0  0  0
 43 47  1  0  0  0  0
 44 48  2  0  0  0  0
 44 49  1  0  0  0  0
```

```
 46 48  1  0  0  0  0
 46 50  1  0  0  0  0
 48 55  1  0  0  0  0
 51 52  1  0  0  0  0
 51 53  1  0  0  0  0
 51 54  1  0  0  0  0
 55 56  1  0  0  0  0
 55 57  1  0  0  0  0
 55 58  1  0  0  0  0
M  END
$$$$
mol142
Optimized Catalyst 142
Optimized Catalyst 142
 59 64  0  0  0  0  0  0  0  0999 V2000
   -0.9704    0.3462   -1.5684 C   0  0  0  0  0  0  0  0  0  0  0  0
    0.2022   -0.5781   -1.1792 C   0  0  0  0  0  0  0  0  0  0  0  0
   -2.2227    0.1263   -0.7530 C   0  0  0  0  0  0  0  0  0  0  0  0
   -1.0988    0.1613    1.5512 C   0  0  0  0  0  0  0  0  0  0  0  0
   -2.2877    0.0179    0.6295 C   0  0  0  0  0  0  0  0  0  0  0  0
   -0.4216   -1.1947    1.8415 C   0  0  0  0  0  0  0  0  0  0  0  0
   -3.1585    0.2509   -1.3316 H   0  0  0  0  0  0  0  0  0  0  0  0
   -3.2704    0.0091    1.1354 H   0  0  0  0  0  0  0  0  0  0  0  0
   -1.2176    0.1657   -2.6483 H   0  0  0  0  0  0  0  0  0  0  0  0
   -0.6566    1.4183   -1.4568 H   0  0  0  0  0  0  0  0  0  0  0  0
    0.8377   -0.7527   -2.0868 H   0  0  0  0  0  0  0  0  0  0  0  0
   -0.9282   -1.6549    2.7325 H   0  0  0  0  0  0  0  0  0  0  0  0
   -1.4399    0.6061    2.5228 H   0  0  0  0  0  0  0  0  0  0  0  0
   -0.3638    0.8766    1.1012 H   0  0  0  0  0  0  0  0  0  0  0  0
   -0.4982   -2.1857    0.7007 C   0  0  0  0  0  0  0  0  0  0  0  0
   -0.2670   -1.9127   -0.6461 C   0  0  0  0  0  0  0  0  0  0  0  0
   -0.5963   -3.2474    1.0279 H   0  0  0  0  0  0  0  0  0  0  0  0
   -0.2343   -2.7512   -1.3770 H   0  0  0  0  0  0  0  0  0  0  0  0
    0.8505   -0.0819   -0.4125 H   0  0  0  0  0  0  0  0  0  0  0  0
    0.6581   -1.0286    2.0975 H   0  0  0  0  0  0  0  0  0  0  0  0
   -3.9592   -2.0298   -0.4813 N   0  0  0  0  0  0  0  0  0  0  0  0
   -5.0993   -2.2033   -0.6386 C   0  0  0  0  0  0  0  0  0  0  0  0
   -2.3373   -4.4391   -0.6245 N   0  0  0  0  0  0  0  0  0  0  0  0
   -2.4407   -5.5587   -0.8494 C   0  0  0  0  0  0  0  0  0  0  0  0
   -6.6078   -2.4329   -0.8467 C   0  0  0  0  0  0  0  0  0  0  0  0
   -6.8348   -2.3868   -1.8914 H   0  0  0  0  0  0  0  0  0  0  0  0
   -6.8781   -3.3953   -0.4651 H   0  0  0  0  0  0  0  0  0  0  0  0
   -2.5796   -7.0624   -1.1515 C   0  0  0  0  0  0  0  0  0  0  0  0
   -3.5935   -7.3657   -0.9937 H   0  0  0  0  0  0  0  0  0  0  0  0
   -1.9359   -7.6177   -0.5018 H   0  0  0  0  0  0  0  0  0  0  0  0
   -2.1857   -7.3303   -2.6160 C   0  0  0  0  0  0  0  0  0  0  0  0
   -1.8361   -8.6192   -3.0197 C   0  0  0  0  0  0  0  0  0  0  0  0
   -2.1786   -6.2844   -3.5389 C   0  0  0  0  0  0  0  0  0  0  0  0
   -1.4788   -8.8619   -4.3458 C   0  0  0  0  0  0  0  0  0  0  0  0
   -1.8411   -9.4433   -2.2917 H   0  0  0  0  0  0  0  0  0  0  0  0
   -1.8221   -6.5272   -4.8656 C   0  0  0  0  0  0  0  0  0  0  0  0
   -2.4543   -5.2685   -3.2209 H   0  0  0  0  0  0  0  0  0  0  0  0
   -1.4721   -7.8157   -5.2692 C   0  0  0  0  0  0  0  0  0  0  0  0
   -1.2026   -9.8777   -4.6640 H   0  0  0  0  0  0  0  0  0  0  0  0
   -1.8170   -5.7025   -5.5931 H   0  0  0  0  0  0  0  0  0  0  0  0
   -7.4006   -1.3436   -0.1006 C   0  0  0  0  0  0  0  0  0  0  0  0
   -6.8044   -0.6414    0.9472 C   0  0  0  0  0  0  0  0  0  0  0  0
   -8.7145   -1.0591   -0.4723 C   0  0  0  0  0  0  0  0  0  0  0  0
   -7.5224    0.3447    1.6235 C   0  0  0  0  0  0  0  0  0  0  0  0
   -5.7688   -0.8665    1.2406 H   0  0  0  0  0  0  0  0  0  0  0  0
   -9.4324   -0.0720    0.2034 C   0  0  0  0  0  0  0  0  0  0  0  0
   -9.1844   -1.6124   -1.2983 H   0  0  0  0  0  0  0  0  0  0  0  0
   -8.8367    0.6298    1.2513 C   0  0  0  0  0  0  0  0  0  0  0  0
   -7.0528    0.8979    2.4499 H   0  0  0  0  0  0  0  0  0  0  0  0
  -10.4682    0.1524   -0.0902 H   0  0  0  0  0  0  0  0  0  0  0  0
```

```
   -1.0776   -8.0841   -6.7334 C   0  0  0  0  0  0  0  0  0  0  0  0
   -0.6240   -9.0502   -6.8092 H   0  0  0  0  0  0  0  0  0  0  0  0
   -1.9514   -8.0510   -7.3501 H   0  0  0  0  0  0  0  0  0  0  0  0
   -0.3833   -7.3376   -7.0583 H   0  0  0  0  0  0  0  0  0  0  0  0
   -9.6291    1.7188    1.9982 C   0  0  0  0  0  0  0  0  0  0  0  0
  -10.5159    1.9551    1.4481 H   0  0  0  0  0  0  0  0  0  0  0  0
   -9.8971    1.3613    2.9704 H   0  0  0  0  0  0  0  0  0  0  0  0
   -9.0249    2.5965    2.0949 H   0  0  0  0  0  0  0  0  0  0  0  0
   -2.2986   -1.9889   -0.2477 Ir  0  0  0  0  0  0  0  0  0  0  0  0
  1  2  1  0  0  0  0
  1  3  1  0  0  0  0
  1  9  1  0  0  0  0
  1 10  1  0  0  0  0
  2 11  1  0  0  0  0
  2 16  1  0  0  0  0
  2 19  1  0  0  0  0
  3  5  2  0  0  0  0
  3  7  1  0  0  0  0
  3 59  1  0  0  0  0
  4  5  1  0  0  0  0
  4  6  1  0  0  0  0
  4 13  1  0  0  0  0
  4 14  1  0  0  0  0
  5  8  1  0  0  0  0
  5 59  1  0  0  0  0
  6 12  1  0  0  0  0
  6 15  1  0  0  0  0
  6 20  1  0  0  0  0
 15 16  2  0  0  0  0
 15 17  1  0  0  0  0
 15 59  1  0  0  0  0
 16 18  1  0  0  0  0
 16 59  1  0  0  0  0
 21 22  3  0  0  0  0
 21 59  1  0  0  0  0
 22 25  1  0  0  0  0
 23 24  3  0  0  0  0
 23 59  1  0  0  0  0
 24 28  1  0  0  0  0
 25 26  1  0  0  0  0
 25 27  1  0  0  0  0
 25 41  1  0  0  0  0
 28 29  1  0  0  0  0
 28 30  1  0  0  0  0
 28 31  1  0  0  0  0
 31 32  2  0  0  0  0
 31 33  1  0  0  0  0
 32 34  1  0  0  0  0
 32 35  1  0  0  0  0
 33 36  2  0  0  0  0
 33 37  1  0  0  0  0
 34 38  2  0  0  0  0
 34 39  1  0  0  0  0
 36 38  1  0  0  0  0
 36 40  1  0  0  0  0
 38 51  1  0  0  0  0
 41 42  2  0  0  0  0
 41 43  1  0  0  0  0
 42 44  1  0  0  0  0
 42 45  1  0  0  0  0
 43 46  2  0  0  0  0
 43 47  1  0  0  0  0
 44 48  2  0  0  0  0
 44 49  1  0  0  0  0
 46 48  1  0  0  0  0
```

```
 46 50  1  0  0  0  0
 48 55  1  0  0  0  0
 51 52  1  0  0  0  0
 51 53  1  0  0  0  0
 51 54  1  0  0  0  0
 55 56  1  0  0  0  0
 55 57  1  0  0  0  0
 55 58  1  0  0  0  0
M  END
$$$$
mol143
Optimized Catalyst 143
Optimized Catalyst 143
 77 82  0  0  0  0  0  0  0  0999 V2000
   -1.6585   -0.2720   -0.6212 C   0  0  0  0  0  0  0  0  0  0  0  0
   -1.2375    0.9233    0.1875 C   0  0  0  0  0  0  0  0  0  0  0  0
   -1.9032    1.6741   -3.2353 C   0  0  0  0  0  0  0  0  0  0  0  0
   -1.9801    2.2201   -0.2066 C   0  0  0  0  0  0  0  0  0  0  0  0
   -1.6176    3.0643   -2.6452 C   0  0  0  0  0  0  0  0  0  0  0  0
   -1.1915    3.1258   -1.1680 C   0  0  0  0  0  0  0  0  0  0  0  0
   -0.1345    1.0749    0.0772 H   0  0  0  0  0  0  0  0  0  0  0  0
   -2.9644    1.9581   -0.6590 H   0  0  0  0  0  0  0  0  0  0  0  0
   -2.9161    1.3395   -2.9032 H   0  0  0  0  0  0  0  0  0  0  0  0
   -2.5468    3.6760   -2.7705 H   0  0  0  0  0  0  0  0  0  0  0  0
   -2.1863   -1.0607   -0.0309 H   0  0  0  0  0  0  0  0  0  0  0  0
   -1.4415    0.7186    1.2686 H   0  0  0  0  0  0  0  0  0  0  0  0
   -1.9302    1.7777   -4.3487 H   0  0  0  0  0  0  0  0  0  0  0  0
   -2.1765    2.7970    0.7312 H   0  0  0  0  0  0  0  0  0  0  0  0
   -0.8070    3.5462   -3.2479 H   0  0  0  0  0  0  0  0  0  0  0  0
   -1.3248    4.1871   -0.8329 H   0  0  0  0  0  0  0  0  0  0  0  0
   -1.4854   -0.4306   -1.9467 C   0  0  0  0  0  0  0  0  0  0  0  0
   -1.8983   -1.3304   -2.4669 H   0  0  0  0  0  0  0  0  0  0  0  0
   -0.8702    0.5856   -2.8692 C   0  0  0  0  0  0  0  0  0  0  0  0
    0.0191    1.0579   -2.3867 H   0  0  0  0  0  0  0  0  0  0  0  0
   -0.5263    0.0817   -3.8046 H   0  0  0  0  0  0  0  0  0  0  0  0
    0.7864   -3.6828   -1.4153 C   0  0  0  0  0  0  0  0  0  0  0  0
    1.1948   -2.9248   -2.6549 C   0  0  0  0  0  0  0  0  0  0  0  0
    2.7987   -1.7109    0.2875 C   0  0  0  0  0  0  0  0  0  0  0  0
    2.6954   -2.5528   -2.6778 C   0  0  0  0  0  0  0  0  0  0  0  0
    3.4965   -0.9058   -0.8199 C   0  0  0  0  0  0  0  0  0  0  0  0
    2.9918   -1.1023   -2.2604 C   0  0  0  0  0  0  0  0  0  0  0  0
    0.5567   -1.9932   -2.7342 H   0  0  0  0  0  0  0  0  0  0  0  0
    3.2605   -3.2571   -2.0223 H   0  0  0  0  0  0  0  0  0  0  0  0
    3.1855   -2.7598    0.2681 H   0  0  0  0  0  0  0  0  0  0  0  0
    4.5840   -1.1751   -0.7879 H   0  0  0  0  0  0  0  0  0  0  0  0
    0.6893   -4.7763   -1.5460 H   0  0  0  0  0  0  0  0  0  0  0  0
    0.9679   -3.5588   -3.5515 H   0  0  0  0  0  0  0  0  0  0  0  0
    3.0904   -1.2641    1.2713 H   0  0  0  0  0  0  0  0  0  0  0  0
    3.0627   -2.6961   -3.7255 H   0  0  0  0  0  0  0  0  0  0  0  0
    3.4089    0.1838   -0.5787 H   0  0  0  0  0  0  0  0  0  0  0  0
    3.7896   -0.7038   -2.9414 H   0  0  0  0  0  0  0  0  0  0  0  0
    0.7788   -3.1351   -0.1162 C   0  0  0  0  0  0  0  0  0  0  0  0
    0.6654   -3.8017    0.7595 H   0  0  0  0  0  0  0  0  0  0  0  0
    1.2571   -1.7401    0.2000 C   0  0  0  0  0  0  0  0  0  0  0  0
    0.8990   -1.0303   -0.5970 H   0  0  0  0  0  0  0  0  0  0  0  0
    0.8135   -1.4124    1.1744 H   0  0  0  0  0  0  0  0  0  0  0  0
    2.0848   -0.4725   -2.4198 H   0  0  0  0  0  0  0  0  0  0  0  0
   -0.1026    2.8953   -1.0940 H   0  0  0  0  0  0  0  0  0  0  0  0
   -2.0555   -5.4069    0.0033 C   0  0  0  0  0  0  0  0  0  0  0  0
   -1.5766   -4.4122   -0.3602 N   0  0  0  0  0  0  0  0  0  0  0  0
   -1.0970   -2.9302   -0.9755 Rh  0  0  0  0  0  0  0  0  0  0  0  0
   -4.8940   -2.6042   -1.2917 C   0  0  0  0  0  0  0  0  0  0  0  0
   -3.7461   -2.7599   -1.2848 N   0  0  0  0  0  0  0  0  0  0  0  0
   -6.3349   -2.4194   -1.2765 C   0  0  0  0  0  0  0  0  0  0  0  0
   -6.6332   -1.9254   -0.3066 H   0  0  0  0  0  0  0  0  0  0  0  0
```

```
   -6.8394   -3.4277   -1.3097 H   0  0  0  0  0  0  0  0  0  0  0  0
   -2.6286   -6.6616    0.4597 C   0  0  0  0  0  0  0  0  0  0  0  0
   -3.7533   -6.5692    0.4597 H   0  0  0  0  0  0  0  0  0  0  0  0
   -2.3542   -7.4721   -0.2762 H   0  0  0  0  0  0  0  0  0  0  0  0
   -2.1477   -7.0307    1.8467 C   0  0  0  0  0  0  0  0  0  0  0  0
   -2.7810   -6.4862    2.9733 C   0  0  0  0  0  0  0  0  0  0  0  0
   -1.0868   -7.9326    2.0057 C   0  0  0  0  0  0  0  0  0  0  0  0
   -2.3547   -6.8488    4.2539 C   0  0  0  0  0  0  0  0  0  0  0  0
   -3.6065   -5.7863    2.8589 H   0  0  0  0  0  0  0  0  0  0  0  0
   -0.6660   -8.2915    3.2901 C   0  0  0  0  0  0  0  0  0  0  0  0
   -0.5893   -8.3638    1.1385 H   0  0  0  0  0  0  0  0  0  0  0  0
   -1.2980   -7.7512    4.4142 C   0  0  0  0  0  0  0  0  0  0  0  0
   -2.8503   -6.4297    5.1311 H   0  0  0  0  0  0  0  0  0  0  0  0
    0.1559   -8.9987    3.4148 H   0  0  0  0  0  0  0  0  0  0  0  0
   -0.9695   -8.0354    5.4153 H   0  0  0  0  0  0  0  0  0  0  0  0
   -6.8053   -1.5762   -2.4412 C   0  0  0  0  0  0  0  0  0  0  0  0
   -7.1785   -2.1913   -3.6440 C   0  0  0  0  0  0  0  0  0  0  0  0
   -6.8859   -0.1830   -2.3082 C   0  0  0  0  0  0  0  0  0  0  0  0
   -7.6425   -1.4116   -4.7074 C   0  0  0  0  0  0  0  0  0  0  0  0
   -7.1146   -3.2723   -3.7569 H   0  0  0  0  0  0  0  0  0  0  0  0
   -7.3515    0.5898   -3.3759 C   0  0  0  0  0  0  0  0  0  0  0  0
   -6.5943    0.3026   -1.3786 H   0  0  0  0  0  0  0  0  0  0  0  0
   -7.7326   -0.0224   -4.5742 C   0  0  0  0  0  0  0  0  0  0  0  0
   -7.9398   -1.8899   -5.6420 H   0  0  0  0  0  0  0  0  0  0  0  0
   -7.4235    1.6733   -3.2711 H   0  0  0  0  0  0  0  0  0  0  0  0
   -8.1043    0.5824   -5.4027 H   0  0  0  0  0  0  0  0  0  0  0  0
  1  2  1  0  0  0  0
  1 11  1  0  0  0  0
  1 17  2  0  0  0  0
  1 47  1  0  0  0  0
  2  4  1  0  0  0  0
  2  7  1  0  0  0  0
  2 12  1  0  0  0  0
  3  5  1  0  0  0  0
  3  9  1  0  0  0  0
  3 13  1  0  0  0  0
  3 19  1  0  0  0  0
  4  6  1  0  0  0  0
  4  8  1  0  0  0  0
  4 14  1  0  0  0  0
  5  6  1  0  0  0  0
  5 10  1  0  0  0  0
  5 15  1  0  0  0  0
  6 16  1  0  0  0  0
  6 44  1  0  0  0  0
 17 18  1  0  0  0  0
 17 19  1  0  0  0  0
 17 47  1  0  0  0  0
 19 20  1  0  0  0  0
 19 21  1  0  0  0  0
 22 23  1  0  0  0  0
 22 32  1  0  0  0  0
 22 38  2  0  0  0  0
 22 47  1  0  0  0  0
 23 25  1  0  0  0  0
 23 28  1  0  0  0  0
 23 33  1  0  0  0  0
 24 26  1  0  0  0  0
 24 30  1  0  0  0  0
 24 34  1  0  0  0  0
 24 40  1  0  0  0  0
 25 27  1  0  0  0  0
 25 29  1  0  0  0  0
 25 35  1  0  0  0  0
 26 27  1  0  0  0  0
```

```
 26 31  1  0  0  0  0
 26 36  1  0  0  0  0
 27 37  1  0  0  0  0
 27 43  1  0  0  0  0
 38 39  1  0  0  0  0
 38 40  1  0  0  0  0
 38 47  1  0  0  0  0
 40 41  1  0  0  0  0
 40 42  1  0  0  0  0
 45 46  3  0  0  0  0
 45 53  1  0  0  0  0
 46 47  1  0  0  0  0
 47 49  1  0  0  0  0
 48 49  3  0  0  0  0
 48 50  1  0  0  0  0
 50 51  1  0  0  0  0
 50 52  1  0  0  0  0
 50 67  1  0  0  0  0
 53 54  1  0  0  0  0
 53 55  1  0  0  0  0
 53 56  1  0  0  0  0
 56 57  2  0  0  0  0
 56 58  1  0  0  0  0
 57 59  1  0  0  0  0
 57 60  1  0  0  0  0
 58 61  2  0  0  0  0
 58 62  1  0  0  0  0
 59 63  2  0  0  0  0
 59 64  1  0  0  0  0
 61 63  1  0  0  0  0
 61 65  1  0  0  0  0
 63 66  1  0  0  0  0
 67 68  2  0  0  0  0
 67 69  1  0  0  0  0
 68 70  1  0  0  0  0
 68 71  1  0  0  0  0
 69 72  2  0  0  0  0
 69 73  1  0  0  0  0
 70 74  2  0  0  0  0
 70 75  1  0  0  0  0
 72 74  1  0  0  0  0
 72 76  1  0  0  0  0
 74 77  1  0  0  0  0
M  END
$$$$
mol144
Optimized Catalyst 144
Optimized Catalyst 144
 77 82  0  0  0  0  0  0  0  0999 V2000
   -2.6450    0.2884   -0.7681 C   0  0  0  0  0  0  0  0  0  0  0  0
   -1.7062    1.1717    0.0070 C   0  0  0  0  0  0  0  0  0  0  0  0
   -1.5977    1.6921   -3.5021 C   0  0  0  0  0  0  0  0  0  0  0  0
   -1.6462    2.6090   -0.5676 C   0  0  0  0  0  0  0  0  0  0  0  0
   -0.6855    2.8137   -2.9832 C   0  0  0  0  0  0  0  0  0  0  0  0
   -0.4277    2.8717   -1.4677 C   0  0  0  0  0  0  0  0  0  0  0  0
   -0.6757    0.7379    0.0163 H   0  0  0  0  0  0  0  0  0  0  0  0
   -2.5848    2.8295   -1.1281 H   0  0  0  0  0  0  0  0  0  0  0  0
   -2.6608    1.9636   -3.2891 H   0  0  0  0  0  0  0  0  0  0  0  0
   -1.1470    3.7853   -3.2992 H   0  0  0  0  0  0  0  0  0  0  0  0
   -3.6219    0.1215   -0.2651 H   0  0  0  0  0  0  0  0  0  0  0  0
   -2.0566    1.2377    1.0699 H   0  0  0  0  0  0  0  0  0  0  0  0
   -1.4802    1.6445   -4.6143 H   0  0  0  0  0  0  0  0  0  0  0  0
   -1.5965    3.3143    0.3008 H   0  0  0  0  0  0  0  0  0  0  0  0
    0.3087    2.7305   -3.4910 H   0  0  0  0  0  0  0  0  0  0  0  0
   -0.0507    3.9049   -1.2420 H   0  0  0  0  0  0  0  0  0  0  0  0
```

```
  -2.4530   -0.1403   -2.0459 C   0  0  0  0  0  0  0  0  0  0  0  0
  -3.2819   -0.6340   -2.6087 H   0  0  0  0  0  0  0  0  0  0  0  0
  -1.3002    0.2897   -2.9186 C   0  0  0  0  0  0  0  0  0  0  0  0
  -0.3481    0.3319   -2.3348 H   0  0  0  0  0  0  0  0  0  0  0  0
  -1.1688   -0.4326   -3.7623 H   0  0  0  0  0  0  0  0  0  0  0  0
   0.2911   -2.8438   -1.7165 C   0  0  0  0  0  0  0  0  0  0  0  0
   1.2090   -2.4624   -2.8413 C   0  0  0  0  0  0  0  0  0  0  0  0
   2.5958   -1.9357    0.3737 C   0  0  0  0  0  0  0  0  0  0  0  0
   2.6872   -2.8235   -2.5593 C   0  0  0  0  0  0  0  0  0  0  0  0
   3.7782   -1.5847   -0.5415 C   0  0  0  0  0  0  0  0  0  0  0  0
   3.5331   -1.6408   -2.0590 C   0  0  0  0  0  0  0  0  0  0  0  0
   1.1327   -1.3652   -3.0448 H   0  0  0  0  0  0  0  0  0  0  0  0
   2.7360   -3.6612   -1.8254 H   0  0  0  0  0  0  0  0  0  0  0  0
   2.4569   -3.0441    0.3769 H   0  0  0  0  0  0  0  0  0  0  0  0
   4.6103   -2.2924   -0.2920 H   0  0  0  0  0  0  0  0  0  0  0  0
  -0.3697   -3.7089   -1.9511 H   0  0  0  0  0  0  0  0  0  0  0  0
   0.8912   -2.9996   -3.7717 H   0  0  0  0  0  0  0  0  0  0  0  0
   2.8690   -1.6276    1.4145 H   0  0  0  0  0  0  0  0  0  0  0  0
   3.1324   -3.1872   -3.5200 H   0  0  0  0  0  0  0  0  0  0  0  0
   4.1269   -0.5499   -0.2944 H   0  0  0  0  0  0  0  0  0  0  0  0
   4.5402   -1.7054   -2.5498 H   0  0  0  0  0  0  0  0  0  0  0  0
   0.2780   -2.2906   -0.4832 C   0  0  0  0  0  0  0  0  0  0  0  0
  -0.3541   -2.7214    0.3424 H   0  0  0  0  0  0  0  0  0  0  0  0
   1.2593   -1.2541   -0.0034 C   0  0  0  0  0  0  0  0  0  0  0  0
   1.4460   -0.4896   -0.7966 H   0  0  0  0  0  0  0  0  0  0  0  0
   0.8573   -0.7332    0.8994 H   0  0  0  0  0  0  0  0  0  0  0  0
   3.0735   -0.6801   -2.3913 H   0  0  0  0  0  0  0  0  0  0  0  0
   0.3953    2.1652   -1.2079 H   0  0  0  0  0  0  0  0  0  0  0  0
  -2.3788   -5.3933    0.0091 C   0  0  0  0  0  0  0  0  0  0  0  0
  -2.3702   -4.2658   -0.2894 N   0  0  0  0  0  0  0  0  0  0  0  0
  -5.5334   -2.4115   -0.7317 C   0  0  0  0  0  0  0  0  0  0  0  0
  -4.3686   -2.3770   -0.7819 N   0  0  0  0  0  0  0  0  0  0  0  0
  -2.4124   -2.3774   -0.8130 Ir  0  0  0  0  0  0  0  0  0  0  0  0
  -2.3749   -6.7958    0.3754 C   0  0  0  0  0  0  0  0  0  0  0  0
  -2.9229   -6.9296    1.3559 H   0  0  0  0  0  0  0  0  0  0  0  0
  -2.9371   -7.3893   -0.4067 H   0  0  0  0  0  0  0  0  0  0  0  0
  -6.9816   -2.4328   -0.6721 C   0  0  0  0  0  0  0  0  0  0  0  0
  -7.3758   -3.2175   -1.3852 H   0  0  0  0  0  0  0  0  0  0  0  0
  -7.3099   -2.7302    0.3689 H   0  0  0  0  0  0  0  0  0  0  0  0
  -7.5649   -1.0805   -1.0268 C   0  0  0  0  0  0  0  0  0  0  0  0
  -7.8137   -0.1399   -0.0177 C   0  0  0  0  0  0  0  0  0  0  0  0
  -7.8762   -0.7844   -2.3612 C   0  0  0  0  0  0  0  0  0  0  0  0
  -8.3852    1.0931   -0.3462 C   0  0  0  0  0  0  0  0  0  0  0  0
  -7.5789   -0.3626    1.0222 H   0  0  0  0  0  0  0  0  0  0  0  0
  -8.4472    0.4507   -2.6815 C   0  0  0  0  0  0  0  0  0  0  0  0
  -7.6895   -1.5107   -3.1510 H   0  0  0  0  0  0  0  0  0  0  0  0
  -8.7044    1.3884   -1.6758 C   0  0  0  0  0  0  0  0  0  0  0  0
  -8.5886    1.8247    0.4392 H   0  0  0  0  0  0  0  0  0  0  0  0
  -8.6987    0.6806   -3.7195 H   0  0  0  0  0  0  0  0  0  0  0  0
  -9.1596    2.3488   -1.9279 H   0  0  0  0  0  0  0  0  0  0  0  0
  -0.9618   -7.3268    0.5018 C   0  0  0  0  0  0  0  0  0  0  0  0
  -0.3252   -7.8891   -0.6134 C   0  0  0  0  0  0  0  0  0  0  0  0
  -0.3051   -7.2749    1.7391 C   0  0  0  0  0  0  0  0  0  0  0  0
   0.9664   -8.4083   -0.4844 C   0  0  0  0  0  0  0  0  0  0  0  0
  -0.8294   -7.9374   -1.5775 H   0  0  0  0  0  0  0  0  0  0  0  0
   0.9865   -7.7963    1.8599 C   0  0  0  0  0  0  0  0  0  0  0  0
  -0.7937   -6.8428    2.6115 H   0  0  0  0  0  0  0  0  0  0  0  0
   1.6218   -8.3647    0.7508 C   0  0  0  0  0  0  0  0  0  0  0  0
   1.4617   -8.8548   -1.3496 H   0  0  0  0  0  0  0  0  0  0  0  0
   1.4975   -7.7646    2.8250 H   0  0  0  0  0  0  0  0  0  0  0  0
   2.6273   -8.7793    0.8505 H   0  0  0  0  0  0  0  0  0  0  0  0
  1  2  1  0  0  0  0
  1 11  1  0  0  0  0
  1 17  2  0  0  0  0
  1 49  1  0  0  0  0
```

```
  2  4  1  0  0  0  0
  2  7  1  0  0  0  0
  2 12  1  0  0  0  0
  3  5  1  0  0  0  0
  3  9  1  0  0  0  0
  3 13  1  0  0  0  0
  3 19  1  0  0  0  0
  4  6  1  0  0  0  0
  4  8  1  0  0  0  0
  4 14  1  0  0  0  0
  5  6  1  0  0  0  0
  5 10  1  0  0  0  0
  5 15  1  0  0  0  0
  6 16  1  0  0  0  0
  6 44  1  0  0  0  0
 17 18  1  0  0  0  0
 17 19  1  0  0  0  0
 17 49  1  0  0  0  0
 19 20  1  0  0  0  0
 19 21  1  0  0  0  0
 22 23  1  0  0  0  0
 22 32  1  0  0  0  0
 22 38  2  0  0  0  0
 22 49  1  0  0  0  0
 23 25  1  0  0  0  0
 23 28  1  0  0  0  0
 23 33  1  0  0  0  0
 24 26  1  0  0  0  0
 24 30  1  0  0  0  0
 24 34  1  0  0  0  0
 24 40  1  0  0  0  0
 25 27  1  0  0  0  0
 25 29  1  0  0  0  0
 25 35  1  0  0  0  0
 26 27  1  0  0  0  0
 26 31  1  0  0  0  0
 26 36  1  0  0  0  0
 27 37  1  0  0  0  0
 27 43  1  0  0  0  0
 38 39  1  0  0  0  0
 38 40  1  0  0  0  0
 38 49  1  0  0  0  0
 40 41  1  0  0  0  0
 40 42  1  0  0  0  0
 45 46  3  0  0  0  0
 45 50  1  0  0  0  0
 46 49  1  0  0  0  0
 47 48  3  0  0  0  0
 47 53  1  0  0  0  0
 48 49  1  0  0  0  0
 50 51  1  0  0  0  0
 50 52  1  0  0  0  0
 50 67  1  0  0  0  0
 53 54  1  0  0  0  0
 53 55  1  0  0  0  0
 53 56  1  0  0  0  0
 56 57  2  0  0  0  0
 56 58  1  0  0  0  0
 57 59  1  0  0  0  0
 57 60  1  0  0  0  0
 58 61  2  0  0  0  0
 58 62  1  0  0  0  0
 59 63  2  0  0  0  0
 59 64  1  0  0  0  0
 61 63  1  0  0  0  0
```

```
 61 65  1  0  0  0  0
 63 66  1  0  0  0  0
 67 68  2  0  0  0  0
 67 69  1  0  0  0  0
 68 70  1  0  0  0  0
 68 71  1  0  0  0  0
 69 72  2  0  0  0  0
 69 73  1  0  0  0  0
 70 74  2  0  0  0  0
 70 75  1  0  0  0  0
 72 74  1  0  0  0  0
 72 76  1  0  0  0  0
 74 77  1  0  0  0  0
M  END
$$$$
mol145
Optimized Catalyst 145
Optimized Catalyst 145
 77 82  0  0  0  0  0  0  0  0999 V2000
   -1.6641   -0.2811   -0.6216 C   0  0  0  0  0  0  0  0  0  0  0  0
   -1.2421    0.9137    0.1871 C   0  0  0  0  0  0  0  0  0  0  0  0
   -1.9121    1.6660   -3.2328 C   0  0  0  0  0  0  0  0  0  0  0  0
   -1.9849    2.2114   -0.2041 C   0  0  0  0  0  0  0  0  0  0  0  0
   -1.6248    3.0557   -2.6424 C   0  0  0  0  0  0  0  0  0  0  0  0
   -1.1970    3.1172   -1.1659 C   0  0  0  0  0  0  0  0  0  0  0  0
   -0.1394    1.0663    0.0750 H   0  0  0  0  0  0  0  0  0  0  0  0
   -2.9703    1.9513   -0.6550 H   0  0  0  0  0  0  0  0  0  0  0  0
   -2.9243    1.3318   -2.8987 H   0  0  0  0  0  0  0  0  0  0  0  0
   -2.5539    3.6680   -2.7665 H   0  0  0  0  0  0  0  0  0  0  0  0
   -2.1921   -1.0693   -0.0300 H   0  0  0  0  0  0  0  0  0  0  0  0
   -1.4444    0.7075    1.2682 H   0  0  0  0  0  0  0  0  0  0  0  0
   -1.9416    1.7707   -4.3461 H   0  0  0  0  0  0  0  0  0  0  0  0
   -2.1788    2.7874    0.7348 H   0  0  0  0  0  0  0  0  0  0  0  0
   -0.8147    3.5377   -3.2461 H   0  0  0  0  0  0  0  0  0  0  0  0
   -1.3299    4.1787   -0.8308 H   0  0  0  0  0  0  0  0  0  0  0  0
   -1.4934   -0.4399   -1.9477 C   0  0  0  0  0  0  0  0  0  0  0  0
   -1.9086   -1.3388   -2.4674 H   0  0  0  0  0  0  0  0  0  0  0  0
   -0.8787    0.5768   -2.8698 C   0  0  0  0  0  0  0  0  0  0  0  0
    0.0111    1.0485   -2.3873 H   0  0  0  0  0  0  0  0  0  0  0  0
   -0.5353    0.0746   -3.8064 H   0  0  0  0  0  0  0  0  0  0  0  0
    0.7928   -3.6832   -1.4068 C   0  0  0  0  0  0  0  0  0  0  0  0
    1.1997   -2.9284   -2.6491 C   0  0  0  0  0  0  0  0  0  0  0  0
    2.8094   -1.7100    0.2878 C   0  0  0  0  0  0  0  0  0  0  0  0
    2.7001   -2.5557   -2.6772 C   0  0  0  0  0  0  0  0  0  0  0  0
    3.5048   -0.9072   -0.8226 C   0  0  0  0  0  0  0  0  0  0  0  0
    2.9979   -1.1051   -2.2620 C   0  0  0  0  0  0  0  0  0  0  0  0
    0.5605   -1.9975   -2.7303 H   0  0  0  0  0  0  0  0  0  0  0  0
    3.2681   -3.2592   -2.0234 H   0  0  0  0  0  0  0  0  0  0  0  0
    3.1957   -2.7592    0.2710 H   0  0  0  0  0  0  0  0  0  0  0  0
    4.5928   -1.1751   -0.7919 H   0  0  0  0  0  0  0  0  0  0  0  0
    0.6937   -4.7766   -1.5352 H   0  0  0  0  0  0  0  0  0  0  0  0
    0.9727   -3.5656   -3.5434 H   0  0  0  0  0  0  0  0  0  0  0  0
    3.1031   -1.2595    1.2695 H   0  0  0  0  0  0  0  0  0  0  0  0
    3.0636   -2.6987   -3.7264 H   0  0  0  0  0  0  0  0  0  0  0  0
    3.4164    0.1827   -0.5831 H   0  0  0  0  0  0  0  0  0  0  0  0
    3.7957   -0.7081   -2.9440 H   0  0  0  0  0  0  0  0  0  0  0  0
    0.7881   -3.1339   -0.1084 C   0  0  0  0  0  0  0  0  0  0  0  0
    0.6761   -3.7986    0.7689 H   0  0  0  0  0  0  0  0  0  0  0  0
    1.2675   -1.7387    0.2044 C   0  0  0  0  0  0  0  0  0  0  0  0
    0.9069   -1.0303   -0.5928 H   0  0  0  0  0  0  0  0  0  0  0  0
    0.8271   -1.4097    1.1798 H   0  0  0  0  0  0  0  0  0  0  0  0
    2.0916   -0.4745   -2.4216 H   0  0  0  0  0  0  0  0  0  0  0  0
   -0.1078    2.8872   -1.0925 H   0  0  0  0  0  0  0  0  0  0  0  0
   -2.0507   -5.3960    0.0113 C   0  0  0  0  0  0  0  0  0  0  0  0
   -1.5733   -4.4011   -0.3535 N   0  0  0  0  0  0  0  0  0  0  0  0
```

```
   -1.0879   -2.9233   -0.9666 Rh  0  0  0  0  0  0  0  0  0  0  0  0
   -4.9207   -2.6021   -1.2873 C   0  0  0  0  0  0  0  0  0  0  0  0
   -3.7736   -2.7620   -1.2718 N   0  0  0  0  0  0  0  0  0  0  0  0
   -6.3619   -2.4107   -1.2834 C   0  0  0  0  0  0  0  0  0  0  0  0
   -6.6644   -1.9166   -0.3144 H   0  0  0  0  0  0  0  0  0  0  0  0
   -6.8695   -3.4177   -1.3181 H   0  0  0  0  0  0  0  0  0  0  0  0
   -2.6217   -6.6525    0.4685 C   0  0  0  0  0  0  0  0  0  0  0  0
   -3.7466   -6.5619    0.4712 H   0  0  0  0  0  0  0  0  0  0  0  0
   -2.3458   -7.4614   -0.2690 H   0  0  0  0  0  0  0  0  0  0  0  0
   -2.1387   -7.0228    1.8517 C   0  0  0  0  0  0  0  0  0  0  0  0
   -2.7744   -6.4805    2.9805 C   0  0  0  0  0  0  0  0  0  0  0  0
   -1.0768   -7.9276    2.0059 C   0  0  0  0  0  0  0  0  0  0  0  0
   -2.3578   -6.8355    4.2641 C   0  0  0  0  0  0  0  0  0  0  0  0
   -3.6019   -5.7805    2.8620 H   0  0  0  0  0  0  0  0  0  0  0  0
   -0.6444   -8.2973    3.2808 C   0  0  0  0  0  0  0  0  0  0  0  0
   -0.5832   -8.3550    1.1321 H   0  0  0  0  0  0  0  0  0  0  0  0
   -1.2984   -7.7415    4.3855 C   0  0  0  0  0  0  0  0  0  0  0  0
   -2.8502   -6.4245    5.1474 H   0  0  0  0  0  0  0  0  0  0  0  0
    0.1753   -9.0068    3.4107 H   0  0  0  0  0  0  0  0  0  0  0  0
   -6.8187   -1.5669   -2.4502 C   0  0  0  0  0  0  0  0  0  0  0  0
   -7.1870   -2.1834   -3.6565 C   0  0  0  0  0  0  0  0  0  0  0  0
   -6.8944   -0.1714   -2.3165 C   0  0  0  0  0  0  0  0  0  0  0  0
   -7.6411   -1.4161   -4.7303 C   0  0  0  0  0  0  0  0  0  0  0  0
   -7.1243   -3.2670   -3.7641 H   0  0  0  0  0  0  0  0  0  0  0  0
   -7.3463    0.6146   -3.3777 C   0  0  0  0  0  0  0  0  0  0  0  0
   -6.6048    0.3096   -1.3816 H   0  0  0  0  0  0  0  0  0  0  0  0
   -7.7143   -0.0289   -4.5641 C   0  0  0  0  0  0  0  0  0  0  0  0
   -7.9354   -1.8866   -5.6703 H   0  0  0  0  0  0  0  0  0  0  0  0
   -7.4167    1.6994   -3.2810 H   0  0  0  0  0  0  0  0  0  0  0  0
   -0.8944   -8.0880    5.6054 F   0  0  0  0  0  0  0  0  0  0  0  0
   -8.1506    0.7117   -5.5808 F   0  0  0  0  0  0  0  0  0  0  0  0
  1  2  1  0  0  0  0
  1 11  1  0  0  0  0
  1 17  2  0  0  0  0
  1 47  1  0  0  0  0
  2  4  1  0  0  0  0
  2  7  1  0  0  0  0
  2 12  1  0  0  0  0
  3  5  1  0  0  0  0
  3  9  1  0  0  0  0
  3 13  1  0  0  0  0
  3 19  1  0  0  0  0
  4  6  1  0  0  0  0
  4  8  1  0  0  0  0
  4 14  1  0  0  0  0
  5  6  1  0  0  0  0
  5 10  1  0  0  0  0
  5 15  1  0  0  0  0
  6 16  1  0  0  0  0
  6 44  1  0  0  0  0
 17 18  1  0  0  0  0
 17 19  1  0  0  0  0
 17 47  1  0  0  0  0
 19 20  1  0  0  0  0
 19 21  1  0  0  0  0
 22 23  1  0  0  0  0
 22 32  1  0  0  0  0
 22 38  2  0  0  0  0
 22 47  1  0  0  0  0
 23 25  1  0  0  0  0
 23 28  1  0  0  0  0
 23 33  1  0  0  0  0
 24 26  1  0  0  0  0
 24 30  1  0  0  0  0
 24 34  1  0  0  0  0
```

```
 24 40  1  0  0  0  0
 25 27  1  0  0  0  0
 25 29  1  0  0  0  0
 25 35  1  0  0  0  0
 26 27  1  0  0  0  0
 26 31  1  0  0  0  0
 26 36  1  0  0  0  0
 27 37  1  0  0  0  0
 27 43  1  0  0  0  0
 38 39  1  0  0  0  0
 38 40  1  0  0  0  0
 38 47  1  0  0  0  0
 40 41  1  0  0  0  0
 40 42  1  0  0  0  0
 45 46  3  0  0  0  0
 45 53  1  0  0  0  0
 46 47  1  0  0  0  0
 47 49  1  0  0  0  0
 48 49  3  0  0  0  0
 48 50  1  0  0  0  0
 50 51  1  0  0  0  0
 50 52  1  0  0  0  0
 50 66  1  0  0  0  0
 53 54  1  0  0  0  0
 53 55  1  0  0  0  0
 53 56  1  0  0  0  0
 56 57  2  0  0  0  0
 56 58  1  0  0  0  0
 57 59  1  0  0  0  0
 57 60  1  0  0  0  0
 58 61  2  0  0  0  0
 58 62  1  0  0  0  0
 59 63  2  0  0  0  0
 59 64  1  0  0  0  0
 61 63  1  0  0  0  0
 61 65  1  0  0  0  0
 63 76  1  0  0  0  0
 66 67  2  0  0  0  0
 66 68  1  0  0  0  0
 67 69  1  0  0  0  0
 67 70  1  0  0  0  0
 68 71  2  0  0  0  0
 68 72  1  0  0  0  0
 69 73  2  0  0  0  0
 69 74  1  0  0  0  0
 71 73  1  0  0  0  0
 71 75  1  0  0  0  0
 73 77  1  0  0  0  0
M  END
$$$$
mol146
Optimized Catalyst 146
Optimized Catalyst 146
 77 82  0  0  0  0  0  0  0  0999 V2000
   -2.6450    0.2888   -0.7664 C   0  0  0  0  0  0  0  0  0  0  0  0
   -1.7057    1.1712    0.0085 C   0  0  0  0  0  0  0  0  0  0  0  0
   -1.5983    1.6899   -3.5010 C   0  0  0  0  0  0  0  0  0  0  0  0
   -1.6462    2.6085   -0.5666 C   0  0  0  0  0  0  0  0  0  0  0  0
   -0.6864    2.8121   -2.9829 C   0  0  0  0  0  0  0  0  0  0  0  0
   -0.4284    2.8709   -1.4675 C   0  0  0  0  0  0  0  0  0  0  0  0
   -0.6750    0.7380    0.0175 H   0  0  0  0  0  0  0  0  0  0  0  0
   -2.5852    2.8290   -1.1265 H   0  0  0  0  0  0  0  0  0  0  0  0
   -2.6614    1.9614   -3.2879 H   0  0  0  0  0  0  0  0  0  0  0  0
   -1.1485    3.7834   -3.2995 H   0  0  0  0  0  0  0  0  0  0  0  0
   -3.6224    0.1246   -0.2640 H   0  0  0  0  0  0  0  0  0  0  0  0
```

```
   -2.0554    1.2376    1.0717 H   0  0  0  0  0  0  0  0  0  0  0  0
   -1.4813    1.6419   -4.6133 H   0  0  0  0  0  0  0  0  0  0  0  0
   -1.5961    3.3141    0.3017 H   0  0  0  0  0  0  0  0  0  0  0  0
    0.3077    2.7293   -3.4909 H   0  0  0  0  0  0  0  0  0  0  0  0
   -0.0521    3.9047   -1.2426 H   0  0  0  0  0  0  0  0  0  0  0  0
   -2.4530   -0.1417   -2.0440 C   0  0  0  0  0  0  0  0  0  0  0  0
   -3.2826   -0.6326   -2.6078 H   0  0  0  0  0  0  0  0  0  0  0  0
   -1.3000    0.2879   -2.9168 C   0  0  0  0  0  0  0  0  0  0  0  0
   -0.3477    0.3312   -2.3334 H   0  0  0  0  0  0  0  0  0  0  0  0
   -1.1685   -0.4346   -3.7603 H   0  0  0  0  0  0  0  0  0  0  0  0
    0.2900   -2.8436   -1.7174 C   0  0  0  0  0  0  0  0  0  0  0  0
    1.2078   -2.4625   -2.8423 C   0  0  0  0  0  0  0  0  0  0  0  0
    2.5943   -1.9367    0.3738 C   0  0  0  0  0  0  0  0  0  0  0  0
    2.6859   -2.8241   -2.5596 C   0  0  0  0  0  0  0  0  0  0  0  0
    3.7768   -1.5860   -0.5413 C   0  0  0  0  0  0  0  0  0  0  0  0
    3.5317   -1.6417   -2.0588 C   0  0  0  0  0  0  0  0  0  0  0  0
    1.1324   -1.3653   -3.0461 H   0  0  0  0  0  0  0  0  0  0  0  0
    2.7348   -3.6621   -1.8263 H   0  0  0  0  0  0  0  0  0  0  0  0
    2.4553   -3.0450    0.3767 H   0  0  0  0  0  0  0  0  0  0  0  0
    4.6086   -2.2943   -0.2917 H   0  0  0  0  0  0  0  0  0  0  0  0
   -0.3699   -3.7092   -1.9524 H   0  0  0  0  0  0  0  0  0  0  0  0
    0.8902   -2.9996   -3.7727 H   0  0  0  0  0  0  0  0  0  0  0  0
    2.8675   -1.6288    1.4146 H   0  0  0  0  0  0  0  0  0  0  0  0
    3.1314   -3.1875   -3.5205 H   0  0  0  0  0  0  0  0  0  0  0  0
    4.1264   -0.5515   -0.2940 H   0  0  0  0  0  0  0  0  0  0  0  0
    4.5390   -1.7066   -2.5493 H   0  0  0  0  0  0  0  0  0  0  0  0
    0.2767   -2.2906   -0.4839 C   0  0  0  0  0  0  0  0  0  0  0  0
   -0.3544   -2.7214    0.3423 H   0  0  0  0  0  0  0  0  0  0  0  0
    1.2582   -1.2543   -0.0037 C   0  0  0  0  0  0  0  0  0  0  0  0
    1.4456   -0.4896   -0.7965 H   0  0  0  0  0  0  0  0  0  0  0  0
    0.8562   -0.7337    0.8993 H   0  0  0  0  0  0  0  0  0  0  0  0
    3.0727   -0.6808   -2.3910 H   0  0  0  0  0  0  0  0  0  0  0  0
    0.3954    2.1654   -1.2075 H   0  0  0  0  0  0  0  0  0  0  0  0
   -2.3714   -5.3911    0.0087 C   0  0  0  0  0  0  0  0  0  0  0  0
   -2.3666   -4.2638   -0.2904 N   0  0  0  0  0  0  0  0  0  0  0  0
   -5.5315   -2.4044   -0.7350 C   0  0  0  0  0  0  0  0  0  0  0  0
   -4.3665   -2.3735   -0.7845 N   0  0  0  0  0  0  0  0  0  0  0  0
   -2.4110   -2.3760   -0.8152 Ir  0  0  0  0  0  0  0  0  0  0  0  0
   -2.3661   -6.7951    0.3760 C   0  0  0  0  0  0  0  0  0  0  0  0
   -2.9158   -6.9274    1.3561 H   0  0  0  0  0  0  0  0  0  0  0  0
   -2.9312   -7.3873   -0.4053 H   0  0  0  0  0  0  0  0  0  0  0  0
   -6.9812   -2.4247   -0.6754 C   0  0  0  0  0  0  0  0  0  0  0  0
   -7.3742   -3.2116   -1.3873 H   0  0  0  0  0  0  0  0  0  0  0  0
   -7.3078   -2.7244    0.3657 H   0  0  0  0  0  0  0  0  0  0  0  0
   -7.5641   -1.0757   -1.0291 C   0  0  0  0  0  0  0  0  0  0  0  0
   -7.8171   -0.1362   -0.0164 C   0  0  0  0  0  0  0  0  0  0  0  0
   -7.8798   -0.7817   -2.3656 C   0  0  0  0  0  0  0  0  0  0  0  0
   -8.3838    1.0996   -0.3299 C   0  0  0  0  0  0  0  0  0  0  0  0
   -7.5832   -0.3654    1.0245 H   0  0  0  0  0  0  0  0  0  0  0  0
   -8.4470    0.4482   -2.6998 C   0  0  0  0  0  0  0  0  0  0  0  0
   -7.6942   -1.5137   -3.1533 H   0  0  0  0  0  0  0  0  0  0  0  0
   -8.6895    1.3649   -1.6699 C   0  0  0  0  0  0  0  0  0  0  0  0
   -8.5927    1.8355    0.4507 H   0  0  0  0  0  0  0  0  0  0  0  0
   -8.7039    0.6848   -3.7354 H   0  0  0  0  0  0  0  0  0  0  0  0
   -0.9562   -7.3254    0.5019 C   0  0  0  0  0  0  0  0  0  0  0  0
   -0.3215   -7.8925   -0.6151 C   0  0  0  0  0  0  0  0  0  0  0  0
   -0.3008   -7.2767    1.7429 C   0  0  0  0  0  0  0  0  0  0  0  0
    0.9686   -8.4114   -0.5020 C   0  0  0  0  0  0  0  0  0  0  0  0
   -0.8321   -7.9420   -1.5780 H   0  0  0  0  0  0  0  0  0  0  0  0
    0.9895   -7.7902    1.8769 C   0  0  0  0  0  0  0  0  0  0  0  0
   -0.7955   -6.8461    2.6150 H   0  0  0  0  0  0  0  0  0  0  0  0
    1.5976   -8.3499    0.7471 C   0  0  0  0  0  0  0  0  0  0  0  0
    1.4697   -8.8621   -1.3622 H   0  0  0  0  0  0  0  0  0  0  0  0
    1.5064   -7.7644    2.8395 H   0  0  0  0  0  0  0  0  0  0  0  0
    2.8266   -8.8434    0.8652 F   0  0  0  0  0  0  0  0  0  0  0  0
```

```
   -9.2324    2.5387   -1.9782 F   0  0  0  0  0  0  0  0  0  0  0  0
  1  2  1  0  0  0  0
  1 11  1  0  0  0  0
  1 17  2  0  0  0  0
  1 49  1  0  0  0  0
  2  4  1  0  0  0  0
  2  7  1  0  0  0  0
  2 12  1  0  0  0  0
  3  5  1  0  0  0  0
  3  9  1  0  0  0  0
  3 13  1  0  0  0  0
  3 19  1  0  0  0  0
  4  6  1  0  0  0  0
  4  8  1  0  0  0  0
  4 14  1  0  0  0  0
  5  6  1  0  0  0  0
  5 10  1  0  0  0  0
  5 15  1  0  0  0  0
  6 16  1  0  0  0  0
  6 44  1  0  0  0  0
 17 18  1  0  0  0  0
 17 19  1  0  0  0  0
 17 49  1  0  0  0  0
 19 20  1  0  0  0  0
 19 21  1  0  0  0  0
 22 23  1  0  0  0  0
 22 32  1  0  0  0  0
 22 38  2  0  0  0  0
 22 49  1  0  0  0  0
 23 25  1  0  0  0  0
 23 28  1  0  0  0  0
 23 33  1  0  0  0  0
 24 26  1  0  0  0  0
 24 30  1  0  0  0  0
 24 34  1  0  0  0  0
 24 40  1  0  0  0  0
 25 27  1  0  0  0  0
 25 29  1  0  0  0  0
 25 35  1  0  0  0  0
 26 27  1  0  0  0  0
 26 31  1  0  0  0  0
 26 36  1  0  0  0  0
 27 37  1  0  0  0  0
 27 43  1  0  0  0  0
 38 39  1  0  0  0  0
 38 40  1  0  0  0  0
 38 49  1  0  0  0  0
 40 41  1  0  0  0  0
 40 42  1  0  0  0  0
 45 46  3  0  0  0  0
 45 50  1  0  0  0  0
 46 49  1  0  0  0  0
 47 48  3  0  0  0  0
 47 53  1  0  0  0  0
 48 49  1  0  0  0  0
 50 51  1  0  0  0  0
 50 52  1  0  0  0  0
 50 66  1  0  0  0  0
 53 54  1  0  0  0  0
 53 55  1  0  0  0  0
 53 56  1  0  0  0  0
 56 57  2  0  0  0  0
 56 58  1  0  0  0  0
 57 59  1  0  0  0  0
 57 60  1  0  0  0  0
```

```
 58 61  2  0  0  0  0
 58 62  1  0  0  0  0
 59 63  2  0  0  0  0
 59 64  1  0  0  0  0
 61 63  1  0  0  0  0
 61 65  1  0  0  0  0
 63 77  1  0  0  0  0
 66 67  2  0  0  0  0
 66 68  1  0  0  0  0
 67 69  1  0  0  0  0
 67 70  1  0  0  0  0
 68 71  2  0  0  0  0
 68 72  1  0  0  0  0
 69 73  2  0  0  0  0
 69 74  1  0  0  0  0
 71 73  1  0  0  0  0
 71 75  1  0  0  0  0
 73 76  1  0  0  0  0
M  END
$$$$
mol147
Optimized Catalyst 147
Optimized Catalyst 147
 83 88  0  0  0  0  0  0  0  0999 V2000
   -1.6498   -0.2659   -0.6331 C   0  0  0  0  0  0  0  0  0  0  0  0
   -1.2393    0.9309    0.1788 C   0  0  0  0  0  0  0  0  0  0  0  0
   -1.8689    1.6844   -3.2434 C   0  0  0  0  0  0  0  0  0  0  0  0
   -1.9791    2.2277   -0.2209 C   0  0  0  0  0  0  0  0  0  0  0  0
   -1.5826    3.0745   -2.6535 C   0  0  0  0  0  0  0  0  0  0  0  0
   -1.1801    3.1376   -1.1697 C   0  0  0  0  0  0  0  0  0  0  0  0
   -0.1355    1.0841    0.0789 H   0  0  0  0  0  0  0  0  0  0  0  0
   -2.9577    1.9662   -0.6860 H   0  0  0  0  0  0  0  0  0  0  0  0
   -2.8841    1.3526   -2.9151 H   0  0  0  0  0  0  0  0  0  0  0  0
   -2.5056    3.6917   -2.7953 H   0  0  0  0  0  0  0  0  0  0  0  0
   -2.1747   -1.0583   -0.0462 H   0  0  0  0  0  0  0  0  0  0  0  0
   -1.4536    0.7253    1.2576 H   0  0  0  0  0  0  0  0  0  0  0  0
   -1.8905    1.7861   -4.3569 H   0  0  0  0  0  0  0  0  0  0  0  0
   -2.1878    2.8015    0.7161 H   0  0  0  0  0  0  0  0  0  0  0  0
   -0.7593    3.5489   -3.2449 H   0  0  0  0  0  0  0  0  0  0  0  0
   -1.3237    4.1982   -0.8364 H   0  0  0  0  0  0  0  0  0  0  0  0
   -1.4649   -0.4243   -1.9566 C   0  0  0  0  0  0  0  0  0  0  0  0
   -1.8711   -1.3248   -2.4808 H   0  0  0  0  0  0  0  0  0  0  0  0
   -0.8402    0.5946   -2.8698 C   0  0  0  0  0  0  0  0  0  0  0  0
    0.0426    1.0656   -2.3739 H   0  0  0  0  0  0  0  0  0  0  0  0
   -0.4842    0.0945   -3.8025 H   0  0  0  0  0  0  0  0  0  0  0  0
    0.7836   -3.7023   -1.4272 C   0  0  0  0  0  0  0  0  0  0  0  0
    1.2013   -2.9427   -2.6628 C   0  0  0  0  0  0  0  0  0  0  0  0
    2.7700   -1.7150    0.2914 C   0  0  0  0  0  0  0  0  0  0  0  0
    2.6965   -2.5497   -2.6692 C   0  0  0  0  0  0  0  0  0  0  0  0
    3.4534   -0.8859   -0.8071 C   0  0  0  0  0  0  0  0  0  0  0  0
    2.9678   -1.0941   -2.2526 C   0  0  0  0  0  0  0  0  0  0  0  0
    0.5511   -2.0205   -2.7546 H   0  0  0  0  0  0  0  0  0  0  0  0
    3.2639   -3.2446   -2.0059 H   0  0  0  0  0  0  0  0  0  0  0  0
    3.1722   -2.7576    0.2612 H   0  0  0  0  0  0  0  0  0  0  0  0
    4.5483   -1.1209   -0.7654 H   0  0  0  0  0  0  0  0  0  0  0  0
    0.6835   -4.7952   -1.5623 H   0  0  0  0  0  0  0  0  0  0  0  0
    0.9964   -3.5835   -3.5601 H   0  0  0  0  0  0  0  0  0  0  0  0
    3.0545   -1.2735    1.2796 H   0  0  0  0  0  0  0  0  0  0  0  0
    3.0771   -2.6898   -3.7126 H   0  0  0  0  0  0  0  0  0  0  0  0
    3.3287    0.1995   -0.5638 H   0  0  0  0  0  0  0  0  0  0  0  0
    3.7669   -0.6857   -2.9259 H   0  0  0  0  0  0  0  0  0  0  0  0
    0.7643   -3.1614   -0.1259 C   0  0  0  0  0  0  0  0  0  0  0  0
    0.6424   -3.8333    0.7448 H   0  0  0  0  0  0  0  0  0  0  0  0
    1.2294   -1.7650    0.2029 C   0  0  0  0  0  0  0  0  0  0  0  0
    0.8631   -1.0534   -0.5895 H   0  0  0  0  0  0  0  0  0  0  0  0
```

```
    0.7811   -1.4506    1.1797 H   0  0  0  0  0  0  0  0  0  0  0  0
    2.0533   -0.4784   -2.4236 H   0  0  0  0  0  0  0  0  0  0  0  0
   -0.0914    2.9122   -1.0783 H   0  0  0  0  0  0  0  0  0  0  0  0
   -2.0833   -5.4204   -0.0433 C   0  0  0  0  0  0  0  0  0  0  0  0
   -1.5916   -4.4305   -0.4015 N   0  0  0  0  0  0  0  0  0  0  0  0
   -1.1025   -2.9450   -1.0028 Rh  0  0  0  0  0  0  0  0  0  0  0  0
   -4.8780   -2.6098   -1.2446 C   0  0  0  0  0  0  0  0  0  0  0  0
   -3.7283   -2.7512   -1.2473 N   0  0  0  0  0  0  0  0  0  0  0  0
   -6.3212   -2.4382   -1.2299 C   0  0  0  0  0  0  0  0  0  0  0  0
   -6.6262   -1.9457   -0.2622 H   0  0  0  0  0  0  0  0  0  0  0  0
   -6.8162   -3.4504   -1.2644 H   0  0  0  0  0  0  0  0  0  0  0  0
   -2.6580   -6.6700    0.4283 C   0  0  0  0  0  0  0  0  0  0  0  0
   -3.7821   -6.5771    0.4361 H   0  0  0  0  0  0  0  0  0  0  0  0
   -2.3860   -7.4915   -0.2952 H   0  0  0  0  0  0  0  0  0  0  0  0
   -2.1651   -7.0110    1.8156 C   0  0  0  0  0  0  0  0  0  0  0  0
   -2.7926   -6.4545    2.9399 C   0  0  0  0  0  0  0  0  0  0  0  0
   -1.0937   -7.8985    1.9866 C   0  0  0  0  0  0  0  0  0  0  0  0
   -2.3539   -6.7847    4.2216 C   0  0  0  0  0  0  0  0  0  0  0  0
   -3.6288   -5.7670    2.8194 H   0  0  0  0  0  0  0  0  0  0  0  0
   -0.6566   -8.2287    3.2698 C   0  0  0  0  0  0  0  0  0  0  0  0
   -0.6005   -8.3426    1.1226 H   0  0  0  0  0  0  0  0  0  0  0  0
   -1.2822   -7.6772    4.4010 C   0  0  0  0  0  0  0  0  0  0  0  0
   -2.8522   -6.3542    5.0905 H   0  0  0  0  0  0  0  0  0  0  0  0
    0.1712   -8.9276    3.3937 H   0  0  0  0  0  0  0  0  0  0  0  0
   -6.7866   -1.6008   -2.3980 C   0  0  0  0  0  0  0  0  0  0  0  0
   -7.1471   -2.2172   -3.6046 C   0  0  0  0  0  0  0  0  0  0  0  0
   -6.8716   -0.2068   -2.2750 C   0  0  0  0  0  0  0  0  0  0  0  0
   -7.5955   -1.4456   -4.6764 C   0  0  0  0  0  0  0  0  0  0  0  0
   -7.0834   -3.2995   -3.7124 H   0  0  0  0  0  0  0  0  0  0  0  0
   -7.3208    0.5627   -3.3483 C   0  0  0  0  0  0  0  0  0  0  0  0
   -6.5930    0.2832   -1.3426 H   0  0  0  0  0  0  0  0  0  0  0  0
   -7.6897   -0.0480   -4.5592 C   0  0  0  0  0  0  0  0  0  0  0  0
   -7.8805   -1.9326   -5.6091 H   0  0  0  0  0  0  0  0  0  0  0  0
   -7.3928    1.6451   -3.2423 H   0  0  0  0  0  0  0  0  0  0  0  0
   -0.8326   -8.0497    5.7751 C   0  0  0  0  0  0  0  0  0  0  0  0
   -0.9533   -7.1972    6.4843 H   0  0  0  0  0  0  0  0  0  0  0  0
   -1.4518   -8.9070    6.1422 H   0  0  0  0  0  0  0  0  0  0  0  0
    0.2386   -8.3603    5.7836 H   0  0  0  0  0  0  0  0  0  0  0  0
   -8.1985    0.7727   -5.6985 C   0  0  0  0  0  0  0  0  0  0  0  0
   -7.9376    0.3093   -6.6791 H   0  0  0  0  0  0  0  0  0  0  0  0
   -9.3130    0.8434   -5.6251 H   0  0  0  0  0  0  0  0  0  0  0  0
   -7.7765    1.8046   -5.6771 H   0  0  0  0  0  0  0  0  0  0  0  0
  1  2  1  0  0  0  0
  1 11  1  0  0  0  0
  1 17  2  0  0  0  0
  1 47  1  0  0  0  0
  2  4  1  0  0  0  0
  2  7  1  0  0  0  0
  2 12  1  0  0  0  0
  3  5  1  0  0  0  0
  3  9  1  0  0  0  0
  3 13  1  0  0  0  0
  3 19  1  0  0  0  0
  4  6  1  0  0  0  0
  4  8  1  0  0  0  0
  4 14  1  0  0  0  0
  5  6  1  0  0  0  0
  5 10  1  0  0  0  0
  5 15  1  0  0  0  0
  6 16  1  0  0  0  0
  6 44  1  0  0  0  0
 17 18  1  0  0  0  0
 17 19  1  0  0  0  0
 17 47  1  0  0  0  0
 19 20  1  0  0  0  0
```

```
 19 21  1  0  0  0  0
 22 23  1  0  0  0  0
 22 32  1  0  0  0  0
 22 38  2  0  0  0  0
 22 47  1  0  0  0  0
 23 25  1  0  0  0  0
 23 28  1  0  0  0  0
 23 33  1  0  0  0  0
 24 26  1  0  0  0  0
 24 30  1  0  0  0  0
 24 34  1  0  0  0  0
 24 40  1  0  0  0  0
 25 27  1  0  0  0  0
 25 29  1  0  0  0  0
 25 35  1  0  0  0  0
 26 27  1  0  0  0  0
 26 31  1  0  0  0  0
 26 36  1  0  0  0  0
 27 37  1  0  0  0  0
 27 43  1  0  0  0  0
 38 39  1  0  0  0  0
 38 40  1  0  0  0  0
 38 47  1  0  0  0  0
 40 41  1  0  0  0  0
 40 42  1  0  0  0  0
 45 46  3  0  0  0  0
 45 53  1  0  0  0  0
 46 47  1  0  0  0  0
 47 49  1  0  0  0  0
 48 49  3  0  0  0  0
 48 50  1  0  0  0  0
 50 51  1  0  0  0  0
 50 52  1  0  0  0  0
 50 66  1  0  0  0  0
 53 54  1  0  0  0  0
 53 55  1  0  0  0  0
 53 56  1  0  0  0  0
 56 57  2  0  0  0  0
 56 58  1  0  0  0  0
 57 59  1  0  0  0  0
 57 60  1  0  0  0  0
 58 61  2  0  0  0  0
 58 62  1  0  0  0  0
 59 63  2  0  0  0  0
 59 64  1  0  0  0  0
 61 63  1  0  0  0  0
 61 65  1  0  0  0  0
 63 76  1  0  0  0  0
 66 67  2  0  0  0  0
 66 68  1  0  0  0  0
 67 69  1  0  0  0  0
 67 70  1  0  0  0  0
 68 71  2  0  0  0  0
 68 72  1  0  0  0  0
 69 73  2  0  0  0  0
 69 74  1  0  0  0  0
 71 73  1  0  0  0  0
 71 75  1  0  0  0  0
 73 80  1  0  0  0  0
 76 77  1  0  0  0  0
 76 78  1  0  0  0  0
 76 79  1  0  0  0  0
 80 81  1  0  0  0  0
 80 82  1  0  0  0  0
 80 83  1  0  0  0  0
```

```
M  END
$$$$
mol148
Optimized Catalyst 148
Optimized Catalyst 148
 83 88  0  0  0  0  0  0  0  0999 V2000
   -2.6339    0.3169   -0.7234 C   0  0  0  0  0  0  0  0  0  0  0  0
   -1.6425    1.2059   -0.0250 C   0  0  0  0  0  0  0  0  0  0  0  0
   -1.6265    1.5375   -3.5614 C   0  0  0  0  0  0  0  0  0  0  0  0
   -1.5402    2.6047   -0.6829 C   0  0  0  0  0  0  0  0  0  0  0  0
   -0.6464    2.6396   -3.1324 C   0  0  0  0  0  0  0  0  0  0  0  0
   -0.3396    2.7646   -1.6300 C   0  0  0  0  0  0  0  0  0  0  0  0
   -0.6299    0.7321   -0.0133 H   0  0  0  0  0  0  0  0  0  0  0  0
   -2.4858    2.8332   -1.2284 H   0  0  0  0  0  0  0  0  0  0  0  0
   -2.6679    1.8669   -3.3250 H   0  0  0  0  0  0  0  0  0  0  0  0
   -1.0703    3.6144   -3.4880 H   0  0  0  0  0  0  0  0  0  0  0  0
   -3.5952    0.2013   -0.1772 H   0  0  0  0  0  0  0  0  0  0  0  0
   -1.9631    1.3477    1.0404 H   0  0  0  0  0  0  0  0  0  0  0  0
   -1.5536    1.4300   -4.6733 H   0  0  0  0  0  0  0  0  0  0  0  0
   -1.4354    3.3547    0.1422 H   0  0  0  0  0  0  0  0  0  0  0  0
    0.3263    2.4799   -3.6630 H   0  0  0  0  0  0  0  0  0  0  0  0
    0.0892    3.7899   -1.4705 H   0  0  0  0  0  0  0  0  0  0  0  0
   -2.5006   -0.1864   -1.9819 C   0  0  0  0  0  0  0  0  0  0  0  0
   -3.3681   -0.6705   -2.4922 H   0  0  0  0  0  0  0  0  0  0  0  0
   -1.3664    0.1549   -2.9171 C   0  0  0  0  0  0  0  0  0  0  0  0
   -0.3922    0.1856   -2.3699 H   0  0  0  0  0  0  0  0  0  0  0  0
   -1.2970   -0.6137   -3.7271 H   0  0  0  0  0  0  0  0  0  0  0  0
    0.2318   -2.9786   -1.6808 C   0  0  0  0  0  0  0  0  0  0  0  0
    1.0980   -2.6671   -2.8663 C   0  0  0  0  0  0  0  0  0  0  0  0
    2.6223   -1.9496    0.2457 C   0  0  0  0  0  0  0  0  0  0  0  0
    2.5886   -3.0082   -2.6287 C   0  0  0  0  0  0  0  0  0  0  0  0
    3.7621   -1.6494   -0.7390 C   0  0  0  0  0  0  0  0  0  0  0  0
    3.4509   -1.7964   -2.2383 C   0  0  0  0  0  0  0  0  0  0  0  0
    1.0080   -1.5851   -3.1348 H   0  0  0  0  0  0  0  0  0  0  0  0
    2.6728   -3.8003   -1.8490 H   0  0  0  0  0  0  0  0  0  0  0  0
    2.4858   -3.0561    0.3153 H   0  0  0  0  0  0  0  0  0  0  0  0
    4.6082   -2.3385   -0.4849 H   0  0  0  0  0  0  0  0  0  0  0  0
   -0.4330   -3.8577   -1.8330 H   0  0  0  0  0  0  0  0  0  0  0  0
    0.7417   -3.2630   -3.7459 H   0  0  0  0  0  0  0  0  0  0  0  0
    2.9408   -1.5851    1.2549 H   0  0  0  0  0  0  0  0  0  0  0  0
    2.9925   -3.4287   -3.5845 H   0  0  0  0  0  0  0  0  0  0  0  0
    4.1158   -0.6010   -0.5689 H   0  0  0  0  0  0  0  0  0  0  0  0
    4.4356   -1.8887   -2.7683 H   0  0  0  0  0  0  0  0  0  0  0  0
    0.2670   -2.3487   -0.4848 C   0  0  0  0  0  0  0  0  0  0  0  0
   -0.3254   -2.7293    0.3921 H   0  0  0  0  0  0  0  0  0  0  0  0
    1.2693   -1.2889   -0.1089 C   0  0  0  0  0  0  0  0  0  0  0  0
    1.4192   -0.5703   -0.9510 H   0  0  0  0  0  0  0  0  0  0  0  0
    0.9083   -0.7187    0.7814 H   0  0  0  0  0  0  0  0  0  0  0  0
    2.9726   -0.8582   -2.6064 H   0  0  0  0  0  0  0  0  0  0  0  0
    0.4591    2.0356   -1.3568 H   0  0  0  0  0  0  0  0  0  0  0  0
   -2.3294   -5.3381    0.2768 C   0  0  0  0  0  0  0  0  0  0  0  0
   -2.3779   -4.2283   -0.0783 N   0  0  0  0  0  0  0  0  0  0  0  0
   -5.5510   -2.3542   -0.5893 C   0  0  0  0  0  0  0  0  0  0  0  0
   -4.3856   -2.3420   -0.6272 N   0  0  0  0  0  0  0  0  0  0  0  0
   -2.4299   -2.3672   -0.6932 Ir  0  0  0  0  0  0  0  0  0  0  0  0
   -2.2365   -6.7207    0.7068 C   0  0  0  0  0  0  0  0  0  0  0  0
   -2.1032   -6.7556    1.8293 H   0  0  0  0  0  0  0  0  0  0  0  0
   -3.2013   -7.2580    0.4658 H   0  0  0  0  0  0  0  0  0  0  0  0
   -7.0014   -2.3462   -0.5515 C   0  0  0  0  0  0  0  0  0  0  0  0
   -7.4003   -2.5102   -1.5970 H   0  0  0  0  0  0  0  0  0  0  0  0
   -7.3665   -3.2060    0.0857 H   0  0  0  0  0  0  0  0  0  0  0  0
   -7.5269   -1.0425    0.0052 C   0  0  0  0  0  0  0  0  0  0  0  0
   -7.6659   -0.8779    1.3911 C   0  0  0  0  0  0  0  0  0  0  0  0
   -7.9091   -0.0082   -0.8614 C   0  0  0  0  0  0  0  0  0  0  0  0
   -8.1800    0.3119    1.9051 C   0  0  0  0  0  0  0  0  0  0  0  0
```

```
   -7.3844   -1.6787    2.0743 H   0  0  0  0  0  0  0  0  0  0  0  0
   -8.4272    1.1791   -0.3448 C   0  0  0  0  0  0  0  0  0  0  0  0
   -7.8162   -0.1261   -1.9407 H   0  0  0  0  0  0  0  0  0  0  0  0
   -8.5697    1.3531    1.0436 C   0  0  0  0  0  0  0  0  0  0  0  0
   -8.2918    0.4296    2.9841 H   0  0  0  0  0  0  0  0  0  0  0  0
   -8.7367    1.9746   -1.0249 H   0  0  0  0  0  0  0  0  0  0  0  0
   -1.0777   -7.4143    0.0264 C   0  0  0  0  0  0  0  0  0  0  0  0
   -1.2653   -8.0443   -1.2128 C   0  0  0  0  0  0  0  0  0  0  0  0
    0.1812   -7.4500    0.6430 C   0  0  0  0  0  0  0  0  0  0  0  0
   -0.2009   -8.6988   -1.8311 C   0  0  0  0  0  0  0  0  0  0  0  0
   -2.2415   -8.0352   -1.6967 H   0  0  0  0  0  0  0  0  0  0  0  0
    1.2439   -8.1070    0.0227 C   0  0  0  0  0  0  0  0  0  0  0  0
    0.3375   -6.9755    1.6117 H   0  0  0  0  0  0  0  0  0  0  0  0
    1.0671   -8.7355   -1.2226 C   0  0  0  0  0  0  0  0  0  0  0  0
   -0.3558   -9.1962   -2.7901 H   0  0  0  0  0  0  0  0  0  0  0  0
    2.2171   -8.1433    0.5152 H   0  0  0  0  0  0  0  0  0  0  0  0
    2.1992   -9.4478   -1.8842 C   0  0  0  0  0  0  0  0  0  0  0  0
    3.1862   -9.0458   -1.5539 H   0  0  0  0  0  0  0  0  0  0  0  0
    2.1559  -10.5352   -1.6182 H   0  0  0  0  0  0  0  0  0  0  0  0
    2.1397   -9.3565   -2.9952 H   0  0  0  0  0  0  0  0  0  0  0  0
   -9.1478    2.6146    1.5921 C   0  0  0  0  0  0  0  0  0  0  0  0
  -10.2651    2.5341    1.5947 H   0  0  0  0  0  0  0  0  0  0  0  0
   -8.8083    2.8007    2.6387 H   0  0  0  0  0  0  0  0  0  0  0  0
   -8.8605    3.4963    0.9710 H   0  0  0  0  0  0  0  0  0  0  0  0
  1  2  1  0  0  0  0
  1 11  1  0  0  0  0
  1 17  2  0  0  0  0
  1 49  1  0  0  0  0
  2  4  1  0  0  0  0
  2  7  1  0  0  0  0
  2 12  1  0  0  0  0
  3  5  1  0  0  0  0
  3  9  1  0  0  0  0
  3 13  1  0  0  0  0
  3 19  1  0  0  0  0
  4  6  1  0  0  0  0
  4  8  1  0  0  0  0
  4 14  1  0  0  0  0
  5  6  1  0  0  0  0
  5 10  1  0  0  0  0
  5 15  1  0  0  0  0
  6 16  1  0  0  0  0
  6 44  1  0  0  0  0
 17 18  1  0  0  0  0
 17 19  1  0  0  0  0
 17 49  1  0  0  0  0
 19 20  1  0  0  0  0
 19 21  1  0  0  0  0
 22 23  1  0  0  0  0
 22 32  1  0  0  0  0
 22 38  2  0  0  0  0
 22 49  1  0  0  0  0
 23 25  1  0  0  0  0
 23 28  1  0  0  0  0
 23 33  1  0  0  0  0
 24 26  1  0  0  0  0
 24 30  1  0  0  0  0
 24 34  1  0  0  0  0
 24 40  1  0  0  0  0
 25 27  1  0  0  0  0
 25 29  1  0  0  0  0
 25 35  1  0  0  0  0
 26 27  1  0  0  0  0
 26 31  1  0  0  0  0
 26 36  1  0  0  0  0
```

```
 27 37  1  0  0  0  0
 27 43  1  0  0  0  0
 38 39  1  0  0  0  0
 38 40  1  0  0  0  0
 38 49  1  0  0  0  0
 40 41  1  0  0  0  0
 40 42  1  0  0  0  0
 45 46  3  0  0  0  0
 45 50  1  0  0  0  0
 46 49  1  0  0  0  0
 47 48  3  0  0  0  0
 47 53  1  0  0  0  0
 48 49  1  0  0  0  0
 50 51  1  0  0  0  0
 50 52  1  0  0  0  0
 50 66  1  0  0  0  0
 53 54  1  0  0  0  0
 53 55  1  0  0  0  0
 53 56  1  0  0  0  0
 56 57  2  0  0  0  0
 56 58  1  0  0  0  0
 57 59  1  0  0  0  0
 57 60  1  0  0  0  0
 58 61  2  0  0  0  0
 58 62  1  0  0  0  0
 59 63  2  0  0  0  0
 59 64  1  0  0  0  0
 61 63  1  0  0  0  0
 61 65  1  0  0  0  0
 63 80  1  0  0  0  0
 66 67  2  0  0  0  0
 66 68  1  0  0  0  0
 67 69  1  0  0  0  0
 67 70  1  0  0  0  0
 68 71  2  0  0  0  0
 68 72  1  0  0  0  0
 69 73  2  0  0  0  0
 69 74  1  0  0  0  0
 71 73  1  0  0  0  0
 71 75  1  0  0  0  0
 73 76  1  0  0  0  0
 76 77  1  0  0  0  0
 76 78  1  0  0  0  0
 76 79  1  0  0  0  0
 80 81  1  0  0  0  0
 80 82  1  0  0  0  0
 80 83  1  0  0  0  0
M  END
$$$$
mol149
Optimized Catalyst 149
Optimized Catalyst 149
 59 64  0  0  0  0  0  0  0  0999 V2000
   -0.9700    0.3566   -1.5579 C   0  0  0  0  0  0  0  0  0  0  0  0
    0.2030   -0.5719   -1.1798 C   0  0  0  0  0  0  0  0  0  0  0  0
   -2.2229    0.1260   -0.7464 C   0  0  0  0  0  0  0  0  0  0  0  0
   -1.0926    0.1595    1.5517 C   0  0  0  0  0  0  0  0  0  0  0  0
   -2.2854    0.0163    0.6353 C   0  0  0  0  0  0  0  0  0  0  0  0
   -0.4263   -1.2009    1.8465 C   0  0  0  0  0  0  0  0  0  0  0  0
   -3.1588    0.2414   -1.3263 H   0  0  0  0  0  0  0  0  0  0  0  0
   -3.2660    0.0010    1.1446 H   0  0  0  0  0  0  0  0  0  0  0  0
   -1.2154    0.1932   -2.6406 H   0  0  0  0  0  0  0  0  0  0  0  0
   -0.6572    1.4272   -1.4299 H   0  0  0  0  0  0  0  0  0  0  0  0
    0.8309   -0.7446   -2.0932 H   0  0  0  0  0  0  0  0  0  0  0  0
   -0.9532   -1.6632    2.7249 H   0  0  0  0  0  0  0  0  0  0  0  0
```

```
   -1.4266    0.6135    2.5213 H   0  0  0  0  0  0  0  0  0  0  0  0
   -0.3534    0.8659    1.0948 H   0  0  0  0  0  0  0  0  0  0  0  0
   -0.4885   -2.1864    0.7000 C   0  0  0  0  0  0  0  0  0  0  0  0
   -0.2609   -1.9081   -0.6465 C   0  0  0  0  0  0  0  0  0  0  0  0
   -0.5779   -3.2503    1.0227 H   0  0  0  0  0  0  0  0  0  0  0  0
   -0.2241   -2.7455   -1.3785 H   0  0  0  0  0  0  0  0  0  0  0  0
    0.8585   -0.0800   -0.4164 H   0  0  0  0  0  0  0  0  0  0  0  0
    0.6491   -1.0414    2.1228 H   0  0  0  0  0  0  0  0  0  0  0  0
   -3.9511   -2.0409   -0.4643 N   0  0  0  0  0  0  0  0  0  0  0  0
   -5.0940   -2.2083   -0.6073 C   0  0  0  0  0  0  0  0  0  0  0  0
   -6.5004   -2.3516   -0.7842 C   0  0  0  0  0  0  0  0  0  0  0  0
   -7.0060   -3.2781   -1.7161 C   0  0  0  0  0  0  0  0  0  0  0  0
   -7.3823   -1.5534   -0.0280 C   0  0  0  0  0  0  0  0  0  0  0  0
   -8.3829   -3.3914   -1.8939 C   0  0  0  0  0  0  0  0  0  0  0  0
   -6.3227   -3.8999   -2.2959 H   0  0  0  0  0  0  0  0  0  0  0  0
   -8.7557   -1.6762   -0.2164 C   0  0  0  0  0  0  0  0  0  0  0  0
   -6.9876   -0.8446    0.7004 H   0  0  0  0  0  0  0  0  0  0  0  0
   -9.2714   -2.5910   -1.1535 C   0  0  0  0  0  0  0  0  0  0  0  0
   -8.7757   -4.1100   -2.6153 H   0  0  0  0  0  0  0  0  0  0  0  0
   -9.4396   -1.0579    0.3688 H   0  0  0  0  0  0  0  0  0  0  0  0
   -2.3127   -4.4057   -0.6638 N   0  0  0  0  0  0  0  0  0  0  0  0
   -2.4081   -5.5385   -0.8954 C   0  0  0  0  0  0  0  0  0  0  0  0
   -2.5171   -6.9321   -1.1856 C   0  0  0  0  0  0  0  0  0  0  0  0
   -3.3494   -7.7545   -0.4028 C   0  0  0  0  0  0  0  0  0  0  0  0
   -1.7882   -7.4812   -2.2583 C   0  0  0  0  0  0  0  0  0  0  0  0
   -3.4519   -9.1114   -0.6981 C   0  0  0  0  0  0  0  0  0  0  0  0
   -3.9094   -7.3294    0.4317 H   0  0  0  0  0  0  0  0  0  0  0  0
   -1.8983   -8.8388   -2.5448 C   0  0  0  0  0  0  0  0  0  0  0  0
   -1.1397   -6.8429   -2.8601 H   0  0  0  0  0  0  0  0  0  0  0  0
   -2.7308   -9.6676   -1.7703 C   0  0  0  0  0  0  0  0  0  0  0  0
   -4.0926   -9.7501   -0.0874 H   0  0  0  0  0  0  0  0  0  0  0  0
   -1.3294   -9.2652   -3.3735 H   0  0  0  0  0  0  0  0  0  0  0  0
   -2.2903   -1.9953   -0.2433 Rh  0  0  0  0  0  0  0  0  0  0  0  0
   -2.8319  -11.1366   -2.0749 C   0  0  0  0  0  0  0  0  0  0  0  0
   -3.8382  -11.5245   -1.7748 H   0  0  0  0  0  0  0  0  0  0  0  0
   -2.7178  -11.3089   -3.1753 H   0  0  0  0  0  0  0  0  0  0  0  0
  -10.7564   -2.7167   -1.3524 C   0  0  0  0  0  0  0  0  0  0  0  0
  -11.2392   -1.7109   -1.2549 H   0  0  0  0  0  0  0  0  0  0  0  0
  -10.9773   -3.0982   -2.3815 H   0  0  0  0  0  0  0  0  0  0  0  0
  -11.3620   -3.6734   -0.3207 C   0  0  0  0  0  0  0  0  0  0  0  0
  -11.1721   -3.3052    0.7126 H   0  0  0  0  0  0  0  0  0  0  0  0
  -12.4634   -3.7444   -0.4779 H   0  0  0  0  0  0  0  0  0  0  0  0
  -10.9185   -4.6889   -0.4280 H   0  0  0  0  0  0  0  0  0  0  0  0
   -1.7450  -11.9123   -1.3235 C   0  0  0  0  0  0  0  0  0  0  0  0
   -1.8286  -12.9988   -1.5575 H   0  0  0  0  0  0  0  0  0  0  0  0
   -0.7344  -11.5571   -1.6275 H   0  0  0  0  0  0  0  0  0  0  0  0
   -1.8595  -11.7725   -0.2248 H   0  0  0  0  0  0  0  0  0  0  0  0
  1  2  1  0  0  0  0
  1  3  1  0  0  0  0
  1  9  1  0  0  0  0
  1 10  1  0  0  0  0
  2 11  1  0  0  0  0
  2 16  1  0  0  0  0
  2 19  1  0  0  0  0
  3  5  2  0  0  0  0
  3  7  1  0  0  0  0
  3 45  1  0  0  0  0
  4  5  1  0  0  0  0
  4  6  1  0  0  0  0
  4 13  1  0  0  0  0
  4 14  1  0  0  0  0
  5  8  1  0  0  0  0
  5 45  1  0  0  0  0
  6 12  1  0  0  0  0
  6 15  1  0  0  0  0
```

```
  6 20  1  0  0  0  0
 15 16  2  0  0  0  0
 15 17  1  0  0  0  0
 15 45  1  0  0  0  0
 16 18  1  0  0  0  0
 16 45  1  0  0  0  0
 21 22  3  0  0  0  0
 21 45  1  0  0  0  0
 22 23  1  0  0  0  0
 23 24  2  0  0  0  0
 23 25  1  0  0  0  0
 24 26  1  0  0  0  0
 24 27  1  0  0  0  0
 25 28  2  0  0  0  0
 25 29  1  0  0  0  0
 26 30  2  0  0  0  0
 26 31  1  0  0  0  0
 28 30  1  0  0  0  0
 28 32  1  0  0  0  0
 30 49  1  0  0  0  0
 33 34  3  0  0  0  0
 33 45  1  0  0  0  0
 34 35  1  0  0  0  0
 35 36  2  0  0  0  0
 35 37  1  0  0  0  0
 36 38  1  0  0  0  0
 36 39  1  0  0  0  0
 37 40  2  0  0  0  0
 37 41  1  0  0  0  0
 38 42  2  0  0  0  0
 38 43  1  0  0  0  0
 40 42  1  0  0  0  0
 40 44  1  0  0  0  0
 42 46  1  0  0  0  0
 46 47  1  0  0  0  0
 46 48  1  0  0  0  0
 46 56  1  0  0  0  0
 49 50  1  0  0  0  0
 49 51  1  0  0  0  0
 49 52  1  0  0  0  0
 52 53  1  0  0  0  0
 52 54  1  0  0  0  0
 52 55  1  0  0  0  0
 56 57  1  0  0  0  0
 56 58  1  0  0  0  0
 56 59  1  0  0  0  0
M  END
$$$$
mol150
Optimized Catalyst 150
Optimized Catalyst 150
 59 64  0  0  0  0  0  0  0  0999 V2000
   -0.8090    0.2719   -1.5126 C   0  0  0  0  0  0  0  0  0  0  0  0
    0.4111   -0.6041   -1.1569 C   0  0  0  0  0  0  0  0  0  0  0  0
   -2.0635    0.0224   -0.7056 C   0  0  0  0  0  0  0  0  0  0  0  0
   -0.9354   -0.0036    1.5837 C   0  0  0  0  0  0  0  0  0  0  0  0
   -2.1257   -0.1247    0.6598 C   0  0  0  0  0  0  0  0  0  0  0  0
   -0.1596   -1.3181    1.8127 C   0  0  0  0  0  0  0  0  0  0  0  0
   -3.0003    0.2066   -1.2805 H   0  0  0  0  0  0  0  0  0  0  0  0
   -3.1103   -0.1097    1.1748 H   0  0  0  0  0  0  0  0  0  0  0  0
   -1.0432    0.1332   -2.6042 H   0  0  0  0  0  0  0  0  0  0  0  0
   -0.5288    1.3559   -1.3718 H   0  0  0  0  0  0  0  0  0  0  0  0
    1.0515   -0.7004   -2.0770 H   0  0  0  0  0  0  0  0  0  0  0  0
   -0.5750   -1.8244    2.7271 H   0  0  0  0  0  0  0  0  0  0  0  0
   -1.2904    0.3710    2.5836 H   0  0  0  0  0  0  0  0  0  0  0  0
```

```
   -0.2450    0.7845    1.1764 H   0  0  0  0  0  0  0  0  0  0  0  0
   -0.1611   -2.2995    0.6626 C   0  0  0  0  0  0  0  0  0  0  0  0
    0.0696   -1.9894   -0.6573 C   0  0  0  0  0  0  0  0  0  0  0  0
   -0.1209   -3.3630    0.9921 H   0  0  0  0  0  0  0  0  0  0  0  0
    0.2508   -2.8010   -1.3944 H   0  0  0  0  0  0  0  0  0  0  0  0
    1.0424   -0.0889   -0.3828 H   0  0  0  0  0  0  0  0  0  0  0  0
    0.9178   -1.0662    2.0360 H   0  0  0  0  0  0  0  0  0  0  0  0
   -4.2455   -2.2772   -0.6039 N   0  0  0  0  0  0  0  0  0  0  0  0
   -5.4044   -2.3601   -0.7325 C   0  0  0  0  0  0  0  0  0  0  0  0
   -6.8122   -2.4367   -0.8852 C   0  0  0  0  0  0  0  0  0  0  0  0
   -7.3725   -3.3078   -1.8449 C   0  0  0  0  0  0  0  0  0  0  0  0
   -7.6533   -1.6413   -0.0755 C   0  0  0  0  0  0  0  0  0  0  0  0
   -8.7534   -3.3736   -1.9905 C   0  0  0  0  0  0  0  0  0  0  0  0
   -6.7229   -3.9245   -2.4688 H   0  0  0  0  0  0  0  0  0  0  0  0
   -9.0317   -1.7153   -0.2346 C   0  0  0  0  0  0  0  0  0  0  0  0
   -7.2208   -0.9728    0.6713 H   0  0  0  0  0  0  0  0  0  0  0  0
   -9.5994   -2.5766   -1.1944 C   0  0  0  0  0  0  0  0  0  0  0  0
   -9.1886   -4.0526   -2.7285 H   0  0  0  0  0  0  0  0  0  0  0  0
   -9.6849   -1.1027    0.3930 H   0  0  0  0  0  0  0  0  0  0  0  0
   -2.2726   -4.2493   -0.6747 N   0  0  0  0  0  0  0  0  0  0  0  0
   -2.3598   -5.3933   -0.8982 C   0  0  0  0  0  0  0  0  0  0  0  0
   -2.4449   -6.7832   -1.1656 C   0  0  0  0  0  0  0  0  0  0  0  0
   -3.2385   -7.6113   -0.3414 C   0  0  0  0  0  0  0  0  0  0  0  0
   -1.7378   -7.3392   -2.2545 C   0  0  0  0  0  0  0  0  0  0  0  0
   -3.3276   -8.9708   -0.6148 C   0  0  0  0  0  0  0  0  0  0  0  0
   -3.7783   -7.1831    0.5054 H   0  0  0  0  0  0  0  0  0  0  0  0
   -1.8357   -8.7009   -2.5147 C   0  0  0  0  0  0  0  0  0  0  0  0
   -1.1196   -6.7005   -2.8883 H   0  0  0  0  0  0  0  0  0  0  0  0
   -2.6361   -9.5323   -1.7065 C   0  0  0  0  0  0  0  0  0  0  0  0
   -3.9358   -9.6140    0.0268 H   0  0  0  0  0  0  0  0  0  0  0  0
   -1.2818   -9.1332   -3.3526 H   0  0  0  0  0  0  0  0  0  0  0  0
   -2.7445  -11.0016   -1.9993 C   0  0  0  0  0  0  0  0  0  0  0  0
   -3.7576  -11.3793   -1.7051 H   0  0  0  0  0  0  0  0  0  0  0  0
   -2.6226  -11.1859   -3.0976 H   0  0  0  0  0  0  0  0  0  0  0  0
  -11.0907   -2.6542   -1.3554 C   0  0  0  0  0  0  0  0  0  0  0  0
  -11.5525   -1.6521   -1.1584 H   0  0  0  0  0  0  0  0  0  0  0  0
  -11.3519   -2.9425   -2.4060 H   0  0  0  0  0  0  0  0  0  0  0  0
  -11.6811   -3.6821   -0.3846 C   0  0  0  0  0  0  0  0  0  0  0  0
  -11.4522   -3.4023    0.6687 H   0  0  0  0  0  0  0  0  0  0  0  0
  -12.7890   -3.7186   -0.5093 H   0  0  0  0  0  0  0  0  0  0  0  0
  -11.2653   -4.6949   -0.5874 H   0  0  0  0  0  0  0  0  0  0  0  0
   -1.6717  -11.7826   -1.2339 C   0  0  0  0  0  0  0  0  0  0  0  0
   -1.7570  -12.8684   -1.4754 H   0  0  0  0  0  0  0  0  0  0  0  0
   -0.6543  -11.4325   -1.5214 H   0  0  0  0  0  0  0  0  0  0  0  0
   -1.8024  -11.6514   -0.1359 H   0  0  0  0  0  0  0  0  0  0  0  0
   -2.2930   -2.3076   -0.3605 Ir  0  0  0  0  0  0  0  0  0  0  0  0
  1  2  1  0  0  0  0
  1  3  1  0  0  0  0
  1  9  1  0  0  0  0
  1 10  1  0  0  0  0
  2 11  1  0  0  0  0
  2 16  1  0  0  0  0
  2 19  1  0  0  0  0
  3  5  2  0  0  0  0
  3  7  1  0  0  0  0
  3 59  1  0  0  0  0
  4  5  1  0  0  0  0
  4  6  1  0  0  0  0
  4 13  1  0  0  0  0
  4 14  1  0  0  0  0
  5  8  1  0  0  0  0
  5 59  1  0  0  0  0
  6 12  1  0  0  0  0
  6 15  1  0  0  0  0
  6 20  1  0  0  0  0
```

```
 15 16  2  0  0  0  0
 15 17  1  0  0  0  0
 15 59  1  0  0  0  0
 16 18  1  0  0  0  0
 16 59  1  0  0  0  0
 21 22  3  0  0  0  0
 21 59  1  0  0  0  0
 22 23  1  0  0  0  0
 23 24  2  0  0  0  0
 23 25  1  0  0  0  0
 24 26  1  0  0  0  0
 24 27  1  0  0  0  0
 25 28  2  0  0  0  0
 25 29  1  0  0  0  0
 26 30  2  0  0  0  0
 26 31  1  0  0  0  0
 28 30  1  0  0  0  0
 28 32  1  0  0  0  0
 30 48  1  0  0  0  0
 33 34  3  0  0  0  0
 33 59  1  0  0  0  0
 34 35  1  0  0  0  0
 35 36  2  0  0  0  0
 35 37  1  0  0  0  0
 36 38  1  0  0  0  0
 36 39  1  0  0  0  0
 37 40  2  0  0  0  0
 37 41  1  0  0  0  0
 38 42  2  0  0  0  0
 38 43  1  0  0  0  0
 40 42  1  0  0  0  0
 40 44  1  0  0  0  0
 42 45  1  0  0  0  0
 45 46  1  0  0  0  0
 45 47  1  0  0  0  0
 45 55  1  0  0  0  0
 48 49  1  0  0  0  0
 48 50  1  0  0  0  0
 48 51  1  0  0  0  0
 51 52  1  0  0  0  0
 51 53  1  0  0  0  0
 51 54  1  0  0  0  0
 55 56  1  0  0  0  0
 55 57  1  0  0  0  0
 55 58  1  0  0  0  0
M  END
$$$$
mol151
Optimized Catalyst 151
Optimized Catalyst 151
 59 64  0  0  0  0  0  0  0  0999 V2000
   -0.9989    0.3288   -1.5723 C   0  0  0  0  0  0  0  0  0  0  0  0
    0.1688   -0.6053   -1.1919 C   0  0  0  0  0  0  0  0  0  0  0  0
   -2.2530    0.1077   -0.7600 C   0  0  0  0  0  0  0  0  0  0  0  0
   -1.1319    0.1386    1.5454 C   0  0  0  0  0  0  0  0  0  0  0  0
   -2.3202   -0.0030    0.6228 C   0  0  0  0  0  0  0  0  0  0  0  0
   -0.4540   -1.2176    1.8317 C   0  0  0  0  0  0  0  0  0  0  0  0
   -3.1872    0.2360   -1.3396 H   0  0  0  0  0  0  0  0  0  0  0  0
   -3.3035   -0.0081    1.1280 H   0  0  0  0  0  0  0  0  0  0  0  0
   -1.2460    0.1614   -2.6544 H   0  0  0  0  0  0  0  0  0  0  0  0
   -0.6786    1.3981   -1.4501 H   0  0  0  0  0  0  0  0  0  0  0  0
    0.7961   -0.7836   -2.1048 H   0  0  0  0  0  0  0  0  0  0  0  0
   -0.9549   -1.6774    2.7261 H   0  0  0  0  0  0  0  0  0  0  0  0
   -1.4742    0.5799    2.5185 H   0  0  0  0  0  0  0  0  0  0  0  0
   -0.3968    0.8561    1.0985 H   0  0  0  0  0  0  0  0  0  0  0  0
```

```
   -0.5373   -2.2077    0.6906 C   0  0  0  0  0  0  0  0  0  0  0  0
   -0.3053   -1.9374   -0.6567 C   0  0  0  0  0  0  0  0  0  0  0  0
   -0.6422   -3.2684    1.0188 H   0  0  0  0  0  0  0  0  0  0  0  0
   -0.2743   -2.7772   -1.3865 H   0  0  0  0  0  0  0  0  0  0  0  0
    0.8271   -0.1149   -0.4298 H   0  0  0  0  0  0  0  0  0  0  0  0
    0.6275   -1.0522    2.0818 H   0  0  0  0  0  0  0  0  0  0  0  0
   -3.9983   -2.0455   -0.4788 N   0  0  0  0  0  0  0  0  0  0  0  0
   -5.1364   -2.2246   -0.6303 C   0  0  0  0  0  0  0  0  0  0  0  0
   -2.3678   -4.4696   -0.6342 N   0  0  0  0  0  0  0  0  0  0  0  0
   -2.4498   -5.6054   -0.8498 C   0  0  0  0  0  0  0  0  0  0  0  0
   -2.3357   -2.0067   -0.2589 Rh  0  0  0  0  0  0  0  0  0  0  0  0
   -2.5517   -7.0276   -1.1244 C   0  0  0  0  0  0  0  0  0  0  0  0
   -3.6186   -7.3628   -0.9834 H   0  0  0  0  0  0  0  0  0  0  0  0
   -2.2684   -7.2237   -2.1982 H   0  0  0  0  0  0  0  0  0  0  0  0
   -6.5646   -2.4077   -0.8142 C   0  0  0  0  0  0  0  0  0  0  0  0
   -6.8024   -2.3769   -1.9159 H   0  0  0  0  0  0  0  0  0  0  0  0
   -6.8599   -3.4222   -0.4208 H   0  0  0  0  0  0  0  0  0  0  0  0
   -7.3798   -1.3201   -0.0837 C   0  0  0  0  0  0  0  0  0  0  0  0
   -7.1313   -1.3286    1.0066 H   0  0  0  0  0  0  0  0  0  0  0  0
   -7.1180   -0.3121   -0.4899 H   0  0  0  0  0  0  0  0  0  0  0  0
   -1.6347   -7.8545   -0.1993 C   0  0  0  0  0  0  0  0  0  0  0  0
   -0.5706   -7.5428   -0.3456 H   0  0  0  0  0  0  0  0  0  0  0  0
   -1.9059   -7.6651    0.8691 H   0  0  0  0  0  0  0  0  0  0  0  0
   -8.8538   -1.5792   -0.2722 C   0  0  0  0  0  0  0  0  0  0  0  0
   -9.5508   -2.3765    0.6471 C   0  0  0  0  0  0  0  0  0  0  0  0
   -9.5304   -1.0273   -1.3696 C   0  0  0  0  0  0  0  0  0  0  0  0
  -10.9167   -2.6142    0.4706 C   0  0  0  0  0  0  0  0  0  0  0  0
   -9.0368   -2.8080    1.5036 H   0  0  0  0  0  0  0  0  0  0  0  0
  -10.8965   -1.2687   -1.5407 C   0  0  0  0  0  0  0  0  0  0  0  0
   -9.0006   -0.4054   -2.0883 H   0  0  0  0  0  0  0  0  0  0  0  0
  -11.5919   -2.0603   -0.6216 C   0  0  0  0  0  0  0  0  0  0  0  0
  -11.4582   -3.2319    1.1879 H   0  0  0  0  0  0  0  0  0  0  0  0
  -11.4221   -0.8366   -2.3931 H   0  0  0  0  0  0  0  0  0  0  0  0
  -12.6591   -2.2418   -0.7537 H   0  0  0  0  0  0  0  0  0  0  0  0
   -1.7823   -9.3234   -0.5095 C   0  0  0  0  0  0  0  0  0  0  0  0
   -0.9563   -9.9271   -1.4686 C   0  0  0  0  0  0  0  0  0  0  0  0
   -2.7458  -10.0910    0.1611 C   0  0  0  0  0  0  0  0  0  0  0  0
   -1.0903  -11.2902   -1.7481 C   0  0  0  0  0  0  0  0  0  0  0  0
   -0.2043   -9.3426   -1.9950 H   0  0  0  0  0  0  0  0  0  0  0  0
   -2.8751  -11.4535   -0.1224 C   0  0  0  0  0  0  0  0  0  0  0  0
   -3.3915   -9.6345    0.9085 H   0  0  0  0  0  0  0  0  0  0  0  0
   -2.0489  -12.0553   -1.0767 C   0  0  0  0  0  0  0  0  0  0  0  0
   -0.4430  -11.7592   -2.4901 H   0  0  0  0  0  0  0  0  0  0  0  0
   -3.6207  -12.0501    0.4044 H   0  0  0  0  0  0  0  0  0  0  0  0
   -2.1499  -13.1190   -1.2945 H   0  0  0  0  0  0  0  0  0  0  0  0
  1  2  1  0  0  0  0
  1  3  1  0  0  0  0
  1  9  1  0  0  0  0
  1 10  1  0  0  0  0
  2 11  1  0  0  0  0
  2 16  1  0  0  0  0
  2 19  1  0  0  0  0
  3  5  2  0  0  0  0
  3  7  1  0  0  0  0
  3 25  1  0  0  0  0
  4  5  1  0  0  0  0
  4  6  1  0  0  0  0
  4 13  1  0  0  0  0
  4 14  1  0  0  0  0
  5  8  1  0  0  0  0
  5 25  1  0  0  0  0
  6 12  1  0  0  0  0
  6 15  1  0  0  0  0
  6 20  1  0  0  0  0
 15 16  2  0  0  0  0
```

```
 15 17  1  0  0  0  0
 15 25  1  0  0  0  0
 16 18  1  0  0  0  0
 16 25  1  0  0  0  0
 21 22  3  0  0  0  0
 21 25  1  0  0  0  0
 22 29  1  0  0  0  0
 23 24  3  0  0  0  0
 23 25  1  0  0  0  0
 24 26  1  0  0  0  0
 26 27  1  0  0  0  0
 26 28  1  0  0  0  0
 26 35  1  0  0  0  0
 29 30  1  0  0  0  0
 29 31  1  0  0  0  0
 29 32  1  0  0  0  0
 32 33  1  0  0  0  0
 32 34  1  0  0  0  0
 32 38  1  0  0  0  0
 35 36  1  0  0  0  0
 35 37  1  0  0  0  0
 35 49  1  0  0  0  0
 38 39  2  0  0  0  0
 38 40  1  0  0  0  0
 39 41  1  0  0  0  0
 39 42  1  0  0  0  0
 40 43  2  0  0  0  0
 40 44  1  0  0  0  0
 41 45  2  0  0  0  0
 41 46  1  0  0  0  0
 43 45  1  0  0  0  0
 43 47  1  0  0  0  0
 45 48  1  0  0  0  0
 49 50  2  0  0  0  0
 49 51  1  0  0  0  0
 50 52  1  0  0  0  0
 50 53  1  0  0  0  0
 51 54  2  0  0  0  0
 51 55  1  0  0  0  0
 52 56  2  0  0  0  0
 52 57  1  0  0  0  0
 54 56  1  0  0  0  0
 54 58  1  0  0  0  0
 56 59  1  0  0  0  0
M  END
$$$$
mol152
Optimized Catalyst 152
Optimized Catalyst 152
 59 63  0  0  0  0  0  0  0  0999 V2000
   -0.8500    0.1894   -1.5569 C   0  0  0  0  0  0  0  0  0  0  0  0
    0.3886   -0.6271   -1.1555 C   0  0  0  0  0  0  0  0  0  0  0  0
   -2.1207   -0.0774   -0.7648 C   0  0  0  0  0  0  0  0  0  0  0  0
   -1.0474   -0.0041    1.5469 C   0  0  0  0  0  0  0  0  0  0  0  0
   -2.2143   -0.1893    0.6033 C   0  0  0  0  0  0  0  0  0  0  0  0
   -0.2299   -1.2739    1.8161 C   0  0  0  0  0  0  0  0  0  0  0  0
   -3.0553    0.0874   -1.3601 H   0  0  0  0  0  0  0  0  0  0  0  0
   -3.2110   -0.1624    1.0983 H   0  0  0  0  0  0  0  0  0  0  0  0
   -1.0706    0.0177   -2.6486 H   0  0  0  0  0  0  0  0  0  0  0  0
   -0.6159    1.2926   -1.4458 H   0  0  0  0  0  0  0  0  0  0  0  0
    1.0410   -0.7265   -2.0604 H   0  0  0  0  0  0  0  0  0  0  0  0
   -0.6336   -1.7717    2.7424 H   0  0  0  0  0  0  0  0  0  0  0  0
   -1.4364    0.3791    2.5334 H   0  0  0  0  0  0  0  0  0  0  0  0
   -0.3762    0.8062    1.1381 H   0  0  0  0  0  0  0  0  0  0  0  0
   -0.1858   -2.2834    0.6936 C   0  0  0  0  0  0  0  0  0  0  0  0
```

```
    0.0679   -2.0032   -0.6283 C   0  0  0  0  0  0  0  0  0  0  0  0
   -0.1258   -3.3399    1.0514 H   0  0  0  0  0  0  0  0  0  0  0  0
    0.2703   -2.8294   -1.3436 H   0  0  0  0  0  0  0  0  0  0  0  0
    0.9761   -0.0722   -0.3837 H   0  0  0  0  0  0  0  0  0  0  0  0
    0.8393   -0.9793    2.0419 H   0  0  0  0  0  0  0  0  0  0  0  0
   -4.2789   -2.3834   -0.5848 N   0  0  0  0  0  0  0  0  0  0  0  0
   -5.4397   -2.4581   -0.7278 C   0  0  0  0  0  0  0  0  0  0  0  0
   -2.2738   -4.3215   -0.6090 N   0  0  0  0  0  0  0  0  0  0  0  0
   -2.3524   -5.4639   -0.8117 C   0  0  0  0  0  0  0  0  0  0  0  0
   -2.3263   -2.3968   -0.3410 Ir  0  0  0  0  0  0  0  0  0  0  0  0
   -2.4474   -6.8779   -1.0793 C   0  0  0  0  0  0  0  0  0  0  0  0
   -3.5149   -7.2184   -0.9583 H   0  0  0  0  0  0  0  0  0  0  0  0
   -2.1390   -7.0862   -2.1455 H   0  0  0  0  0  0  0  0  0  0  0  0
   -6.8815   -2.5464   -0.9079 C   0  0  0  0  0  0  0  0  0  0  0  0
   -7.1232   -2.4466   -2.0297 H   0  0  0  0  0  0  0  0  0  0  0  0
   -7.2403   -3.5329   -0.5260 H   0  0  0  0  0  0  0  0  0  0  0  0
   -7.5761   -1.3357   -0.1521 C   0  0  0  0  0  0  0  0  0  0  0  0
   -7.3103   -1.3892    0.9553 H   0  0  0  0  0  0  0  0  0  0  0  0
   -7.3099   -0.3587   -0.5768 H   0  0  0  0  0  0  0  0  0  0  0  0
   -1.5552   -7.7279   -0.1392 C   0  0  0  0  0  0  0  0  0  0  0  0
   -0.4784   -7.4480   -0.2636 H   0  0  0  0  0  0  0  0  0  0  0  0
   -1.8348   -7.5493    0.9286 H   0  0  0  0  0  0  0  0  0  0  0  0
   -9.0729   -1.5629   -0.2967 C   0  0  2  0  0  0  0  0  0  0  0  0
   -9.7555   -2.3441    0.7317 C   0  0  0  0  0  0  0  0  0  0  0  0
   -9.7836   -0.9986   -1.4612 C   0  0  0  0  0  0  0  0  0  0  0  0
  -11.1415   -2.5572    0.5937 C   0  0  0  0  0  0  0  0  0  0  0  0
   -9.2601   -2.8019    1.5330 H   0  0  0  0  0  0  0  0  0  0  0  0
  -11.1694   -1.2155   -1.5929 C   0  0  0  0  0  0  0  0  0  0  0  0
   -9.2506   -0.4033   -2.1540 H   0  0  0  0  0  0  0  0  0  0  0  0
  -11.8499   -1.9962   -0.5687 C   0  0  0  0  0  0  0  0  0  0  0  0
  -11.7048   -3.1551    1.2768 H   0  0  0  0  0  0  0  0  0  0  0  0
  -11.6948   -0.7643   -2.3981 H   0  0  0  0  0  0  0  0  0  0  0  0
  -12.9279   -2.1456   -0.6908 H   0  0  0  0  0  0  0  0  0  0  0  0
   -1.7379   -9.1867   -0.4729 C   0  0  0  0  0  0  0  0  0  0  0  0
   -0.9357   -9.7903   -1.4458 C   0  0  0  0  0  0  0  0  0  0  0  0
   -2.7174   -9.9380    0.1815 C   0  0  0  0  0  0  0  0  0  0  0  0
   -1.1132  -11.1390   -1.7588 C   0  0  0  0  0  0  0  0  0  0  0  0
   -0.1595   -9.2247   -1.9564 H   0  0  0  0  0  0  0  0  0  0  0  0
   -2.8897  -11.2867   -0.1358 C   0  0  0  0  0  0  0  0  0  0  0  0
   -3.3416   -9.4899    0.9513 H   0  0  0  0  0  0  0  0  0  0  0  0
   -2.0900  -11.8887   -1.1059 C   0  0  0  0  0  0  0  0  0  0  0  0
   -0.4780  -11.6168   -2.5133 H   0  0  0  0  0  0  0  0  0  0  0  0
   -3.6490  -11.8813    0.3863 H   0  0  0  0  0  0  0  0  0  0  0  0
   -2.2202  -12.9520   -1.3448 H   0  0  0  0  0  0  0  0  0  0  0  0
  1  2  1  0  0  0  0
  1  3  1  0  0  0  0
  1  9  1  0  0  0  0
  1 10  1  0  0  0  0
  2 11  1  0  0  0  0
  2 16  1  0  0  0  0
  2 19  1  0  0  0  0
  3  5  2  0  0  0  0
  3  7  1  0  0  0  0
  3 25  1  0  0  0  0
  4  5  1  0  0  0  0
  4  6  1  0  0  0  0
  4 13  1  0  0  0  0
  4 14  1  0  0  0  0
  5  8  1  0  0  0  0
  5 25  1  0  0  0  0
  6 12  1  0  0  0  0
  6 15  1  0  0  0  0
  6 20  1  0  0  0  0
 15 16  2  0  0  0  0
 15 17  1  0  0  0  0
```

```
 15 25  1  0  0  0  0
 16 18  1  0  0  0  0
 16 25  1  0  0  0  0
 21 22  3  0  0  0  0
 21 25  1  0  0  0  0
 22 29  1  0  0  0  0
 23 24  3  0  0  0  0
 23 25  1  0  0  0  0
 24 26  1  0  0  0  0
 26 27  1  0  0  0  0
 26 28  1  0  0  0  0
 26 35  1  0  0  0  0
 29 31  1  0  0  0  0
 29 32  1  0  0  0  0
 32 34  1  0  0  0  0
 32 33  1  0  0  0  0
 35 36  1  0  0  0  0
 35 37  1  0  0  0  0
 35 49  1  0  0  0  0
 38 32  1  6  0  0  0
 38 39  1  0  0  0  0
 38 40  1  0  0  0  0
 39 41  2  0  0  0  0
 39 42  1  0  0  0  0
 40 43  2  0  0  0  0
 40 44  1  0  0  0  0
 41 45  1  0  0  0  0
 41 46  1  0  0  0  0
 43 45  1  0  0  0  0
 43 47  1  0  0  0  0
 45 48  1  0  0  0  0
 49 50  2  0  0  0  0
 49 51  1  0  0  0  0
 50 52  1  0  0  0  0
 50 53  1  0  0  0  0
 51 54  2  0  0  0  0
 51 55  1  0  0  0  0
 52 56  2  0  0  0  0
 52 57  1  0  0  0  0
 54 56  1  0  0  0  0
 54 58  1  0  0  0  0
 56 59  1  0  0  0  0
M  END
$$$$
mol153
Optimized Catalyst 153
Optimized Catalyst 153
 83 88  0  0  0  0  0  0  0  0999 V2000
   -1.6233   -0.2486   -0.6388 C   0  0  0  0  0  0  0  0  0  0  0  0
   -1.2341    0.9527    0.1769 C   0  0  0  0  0  0  0  0  0  0  0  0
   -1.8498    1.7065   -3.2478 C   0  0  0  0  0  0  0  0  0  0  0  0
   -1.9764    2.2445   -0.2341 C   0  0  0  0  0  0  0  0  0  0  0  0
   -1.5593    3.0968   -2.6610 C   0  0  0  0  0  0  0  0  0  0  0  0
   -1.1700    3.1580   -1.1734 C   0  0  0  0  0  0  0  0  0  0  0  0
   -0.1298    1.1125    0.0936 H   0  0  0  0  0  0  0  0  0  0  0  0
   -2.9470    1.9759   -0.7117 H   0  0  0  0  0  0  0  0  0  0  0  0
   -2.8629    1.3754   -2.9137 H   0  0  0  0  0  0  0  0  0  0  0  0
   -2.4762    3.7207   -2.8129 H   0  0  0  0  0  0  0  0  0  0  0  0
   -2.1331   -1.0520   -0.0538 H   0  0  0  0  0  0  0  0  0  0  0  0
   -1.4635    0.7471    1.2527 H   0  0  0  0  0  0  0  0  0  0  0  0
   -1.8772    1.8063   -4.3613 H   0  0  0  0  0  0  0  0  0  0  0  0
   -2.2021    2.8182    0.6988 H   0  0  0  0  0  0  0  0  0  0  0  0
   -0.7272    3.5633   -3.2461 H   0  0  0  0  0  0  0  0  0  0  0  0
   -1.3171    4.2175   -0.8391 H   0  0  0  0  0  0  0  0  0  0  0  0
   -1.4338   -0.4038   -1.9617 C   0  0  0  0  0  0  0  0  0  0  0  0
```

```
   -1.8233   -1.3124   -2.4842 H   0  0  0  0  0  0  0  0  0  0  0  0
   -0.8179    0.6194   -2.8757 C   0  0  0  0  0  0  0  0  0  0  0  0
    0.0639    1.0933   -2.3802 H   0  0  0  0  0  0  0  0  0  0  0  0
   -0.4609    0.1219   -3.8094 H   0  0  0  0  0  0  0  0  0  0  0  0
    0.8060   -3.7092   -1.4402 C   0  0  0  0  0  0  0  0  0  0  0  0
    1.2240   -2.9438   -2.6721 C   0  0  0  0  0  0  0  0  0  0  0  0
    2.7736   -1.7061    0.2887 C   0  0  0  0  0  0  0  0  0  0  0  0
    2.7154   -2.5361   -2.6680 C   0  0  0  0  0  0  0  0  0  0  0  0
    3.4447   -0.8608   -0.8049 C   0  0  0  0  0  0  0  0  0  0  0  0
    2.9684   -1.0769   -2.2525 C   0  0  0  0  0  0  0  0  0  0  0  0
    0.5645   -2.0283   -2.7670 H   0  0  0  0  0  0  0  0  0  0  0  0
    3.2843   -3.2240   -1.9987 H   0  0  0  0  0  0  0  0  0  0  0  0
    3.1885   -2.7434    0.2510 H   0  0  0  0  0  0  0  0  0  0  0  0
    4.5438   -1.0739   -0.7592 H   0  0  0  0  0  0  0  0  0  0  0  0
    0.7082   -4.8019   -1.5800 H   0  0  0  0  0  0  0  0  0  0  0  0
    1.0316   -3.5853   -3.5716 H   0  0  0  0  0  0  0  0  0  0  0  0
    3.0531   -1.2680    1.2798 H   0  0  0  0  0  0  0  0  0  0  0  0
    3.1066   -2.6753   -3.7074 H   0  0  0  0  0  0  0  0  0  0  0  0
    3.2969    0.2214   -0.5602 H   0  0  0  0  0  0  0  0  0  0  0  0
    3.7651   -0.6600   -2.9231 H   0  0  0  0  0  0  0  0  0  0  0  0
    0.7833   -3.1744   -0.1366 C   0  0  0  0  0  0  0  0  0  0  0  0
    0.6668   -3.8510    0.7301 H   0  0  0  0  0  0  0  0  0  0  0  0
    1.2341   -1.7746    0.1990 C   0  0  0  0  0  0  0  0  0  0  0  0
    0.8596   -1.0635   -0.5911 H   0  0  0  0  0  0  0  0  0  0  0  0
    0.7804   -1.4692    1.1760 H   0  0  0  0  0  0  0  0  0  0  0  0
    2.0470   -0.4727   -2.4278 H   0  0  0  0  0  0  0  0  0  0  0  0
   -0.0821    2.9326   -1.0732 H   0  0  0  0  0  0  0  0  0  0  0  0
   -2.0704   -5.4284   -0.0709 C   0  0  0  0  0  0  0  0  0  0  0  0
   -2.6435   -6.6559    0.3711 C   0  0  0  0  0  0  0  0  0  0  0  0
   -2.3406   -7.1554    1.6523 C   0  0  0  0  0  0  0  0  0  0  0  0
   -3.5122   -7.3661   -0.4801 C   0  0  0  0  0  0  0  0  0  0  0  0
   -2.9060   -8.3564    2.0732 C   0  0  0  0  0  0  0  0  0  0  0  0
   -1.6687   -6.6035    2.3097 H   0  0  0  0  0  0  0  0  0  0  0  0
   -4.0711   -8.5656   -0.0467 C   0  0  0  0  0  0  0  0  0  0  0  0
   -3.7425   -6.9768   -1.4720 H   0  0  0  0  0  0  0  0  0  0  0  0
   -3.7768   -9.0726    1.2320 C   0  0  0  0  0  0  0  0  0  0  0  0
   -2.6672   -8.7476    3.0642 H   0  0  0  0  0  0  0  0  0  0  0  0
   -4.7392   -9.1203   -0.7081 H   0  0  0  0  0  0  0  0  0  0  0  0
   -1.5776   -4.4369   -0.4309 N   0  0  0  0  0  0  0  0  0  0  0  0
   -1.0797   -2.9502   -1.0118 Rh  0  0  0  0  0  0  0  0  0  0  0  0
   -4.8399   -2.6234   -1.2384 C   0  0  0  0  0  0  0  0  0  0  0  0
   -6.2589   -2.4779   -1.1679 C   0  0  0  0  0  0  0  0  0  0  0  0
   -7.0962   -3.3803   -1.8499 C   0  0  0  0  0  0  0  0  0  0  0  0
   -6.8180   -1.4346   -0.4055 C   0  0  0  0  0  0  0  0  0  0  0  0
   -8.4789   -3.2396   -1.7632 C   0  0  0  0  0  0  0  0  0  0  0  0
   -6.6613   -4.1852   -2.4434 H   0  0  0  0  0  0  0  0  0  0  0  0
   -8.2017   -1.3026   -0.3253 C   0  0  0  0  0  0  0  0  0  0  0  0
   -6.1670   -0.7343    0.1196 H   0  0  0  0  0  0  0  0  0  0  0  0
   -9.0461   -2.2023   -1.0009 C   0  0  0  0  0  0  0  0  0  0  0  0
   -9.1279   -3.9371   -2.2955 H   0  0  0  0  0  0  0  0  0  0  0  0
   -8.6346   -0.4917    0.2633 H   0  0  0  0  0  0  0  0  0  0  0  0
   -3.6870   -2.7429   -1.2846 N   0  0  0  0  0  0  0  0  0  0  0  0
  -10.5399   -2.0458   -0.9229 C   0  0  0  0  0  0  0  0  0  0  0  0
  -11.0363   -3.0449   -1.0155 H   0  0  0  0  0  0  0  0  0  0  0  0
  -10.8295   -1.6156    0.0693 H   0  0  0  0  0  0  0  0  0  0  0  0
   -4.3733  -10.3755    1.6882 C   0  0  0  0  0  0  0  0  0  0  0  0
   -5.3822  -10.5188    1.2247 H   0  0  0  0  0  0  0  0  0  0  0  0
   -4.5108  -10.3667    2.7993 H   0  0  0  0  0  0  0  0  0  0  0  0
  -11.0393   -1.1250   -2.0411 C   0  0  0  0  0  0  0  0  0  0  0  0
  -12.1473   -1.0238   -1.9790 H   0  0  0  0  0  0  0  0  0  0  0  0
  -10.5819   -0.1142   -1.9451 H   0  0  0  0  0  0  0  0  0  0  0  0
  -10.7738   -1.5452   -3.0376 H   0  0  0  0  0  0  0  0  0  0  0  0
   -3.4641  -11.5454    1.2983 C   0  0  0  0  0  0  0  0  0  0  0  0
   -2.4652  -11.4376    1.7786 H   0  0  0  0  0  0  0  0  0  0  0  0
   -3.9205  -12.5051    1.6339 H   0  0  0  0  0  0  0  0  0  0  0  0
```

```
   -3.3290  -11.5811    0.1935 H   0  0  0  0  0  0  0  0  0  0  0  0
  1  2  1  0  0  0  0
  1 11  1  0  0  0  0
  1 17  2  0  0  0  0
  1 57  1  0  0  0  0
  2  4  1  0  0  0  0
  2  7  1  0  0  0  0
  2 12  1  0  0  0  0
  3  5  1  0  0  0  0
  3  9  1  0  0  0  0
  3 13  1  0  0  0  0
  3 19  1  0  0  0  0
  4  6  1  0  0  0  0
  4  8  1  0  0  0  0
  4 14  1  0  0  0  0
  5  6  1  0  0  0  0
  5 10  1  0  0  0  0
  5 15  1  0  0  0  0
  6 16  1  0  0  0  0
  6 44  1  0  0  0  0
 17 18  1  0  0  0  0
 17 19  1  0  0  0  0
 17 57  1  0  0  0  0
 19 20  1  0  0  0  0
 19 21  1  0  0  0  0
 22 23  1  0  0  0  0
 22 32  1  0  0  0  0
 22 38  2  0  0  0  0
 22 57  1  0  0  0  0
 23 25  1  0  0  0  0
 23 28  1  0  0  0  0
 23 33  1  0  0  0  0
 24 26  1  0  0  0  0
 24 30  1  0  0  0  0
 24 34  1  0  0  0  0
 24 40  1  0  0  0  0
 25 27  1  0  0  0  0
 25 29  1  0  0  0  0
 25 35  1  0  0  0  0
 26 27  1  0  0  0  0
 26 31  1  0  0  0  0
 26 36  1  0  0  0  0
 27 37  1  0  0  0  0
 27 43  1  0  0  0  0
 38 39  1  0  0  0  0
 38 40  1  0  0  0  0
 38 57  1  0  0  0  0
 40 41  1  0  0  0  0
 40 42  1  0  0  0  0
 45 46  1  0  0  0  0
 45 56  3  0  0  0  0
 46 47  2  0  0  0  0
 46 48  1  0  0  0  0
 47 49  1  0  0  0  0
 47 50  1  0  0  0  0
 48 51  2  0  0  0  0
 48 52  1  0  0  0  0
 49 53  2  0  0  0  0
 49 54  1  0  0  0  0
 51 53  1  0  0  0  0
 51 55  1  0  0  0  0
 53 73  1  0  0  0  0
 56 57  1  0  0  0  0
 57 69  1  0  0  0  0
 58 59  1  0  0  0  0
```

```
 58 69  3  0  0  0  0
 59 60  2  0  0  0  0
 59 61  1  0  0  0  0
 60 62  1  0  0  0  0
 60 63  1  0  0  0  0
 61 64  2  0  0  0  0
 61 65  1  0  0  0  0
 62 66  2  0  0  0  0
 62 67  1  0  0  0  0
 64 66  1  0  0  0  0
 64 68  1  0  0  0  0
 66 70  1  0  0  0  0
 70 71  1  0  0  0  0
 70 72  1  0  0  0  0
 70 76  1  0  0  0  0
 73 74  1  0  0  0  0
 73 75  1  0  0  0  0
 73 80  1  0  0  0  0
 76 77  1  0  0  0  0
 76 78  1  0  0  0  0
 76 79  1  0  0  0  0
 80 81  1  0  0  0  0
 80 82  1  0  0  0  0
 80 83  1  0  0  0  0
M  END
$$$$
mol154
Optimized Catalyst 154
Optimized Catalyst 154
 83 88  0  0  0  0  0  0  0  0999 V2000
   -2.6309    0.3201   -0.7334 C   0  0  0  0  0  0  0  0  0  0  0  0
   -1.6830    1.2003    0.0333 C   0  0  0  0  0  0  0  0  0  0  0  0
   -1.5876    1.7079   -3.4863 C   0  0  0  0  0  0  0  0  0  0  0  0
   -1.6149    2.6331   -0.5512 C   0  0  0  0  0  0  0  0  0  0  0  0
   -0.6661    2.8247   -2.9724 C   0  0  0  0  0  0  0  0  0  0  0  0
   -0.3987    2.8813   -1.4585 C   0  0  0  0  0  0  0  0  0  0  0  0
   -0.6563    0.7578    0.0428 H   0  0  0  0  0  0  0  0  0  0  0  0
   -2.5543    2.8554   -1.1099 H   0  0  0  0  0  0  0  0  0  0  0  0
   -2.6488    1.9891   -3.2770 H   0  0  0  0  0  0  0  0  0  0  0  0
   -1.1235    3.7989   -3.2861 H   0  0  0  0  0  0  0  0  0  0  0  0
   -3.6003    0.1489   -0.2189 H   0  0  0  0  0  0  0  0  0  0  0  0
   -2.0290    1.2757    1.0970 H   0  0  0  0  0  0  0  0  0  0  0  0
   -1.4685    1.6532   -4.5981 H   0  0  0  0  0  0  0  0  0  0  0  0
   -1.5573    3.3441    0.3120 H   0  0  0  0  0  0  0  0  0  0  0  0
    0.3247    2.7347   -3.4856 H   0  0  0  0  0  0  0  0  0  0  0  0
   -0.0094    3.9105   -1.2360 H   0  0  0  0  0  0  0  0  0  0  0  0
   -2.4531   -0.1099   -2.0124 C   0  0  0  0  0  0  0  0  0  0  0  0
   -3.2911   -0.5971   -2.5678 H   0  0  0  0  0  0  0  0  0  0  0  0
   -1.3028    0.3067   -2.8951 C   0  0  0  0  0  0  0  0  0  0  0  0
   -0.3470    0.3418   -2.3168 H   0  0  0  0  0  0  0  0  0  0  0  0
   -1.1845   -0.4226   -3.7350 H   0  0  0  0  0  0  0  0  0  0  0  0
    0.2981   -2.8239   -1.7399 C   0  0  0  0  0  0  0  0  0  0  0  0
    1.2164   -2.4471   -2.8661 C   0  0  0  0  0  0  0  0  0  0  0  0
    2.6139   -1.9418    0.3525 C   0  0  0  0  0  0  0  0  0  0  0  0
    2.6934   -2.8138   -2.5869 C   0  0  0  0  0  0  0  0  0  0  0  0
    3.7977   -1.5985   -0.5641 C   0  0  0  0  0  0  0  0  0  0  0  0
    3.5437   -1.6369   -2.0807 C   0  0  0  0  0  0  0  0  0  0  0  0
    1.1424   -1.3498   -3.0703 H   0  0  0  0  0  0  0  0  0  0  0  0
    2.7393   -3.6551   -1.8570 H   0  0  0  0  0  0  0  0  0  0  0  0
    2.4662   -3.0490    0.3529 H   0  0  0  0  0  0  0  0  0  0  0  0
    4.6210   -2.3202   -0.3255 H   0  0  0  0  0  0  0  0  0  0  0  0
   -0.3745   -3.6796   -1.9773 H   0  0  0  0  0  0  0  0  0  0  0  0
    0.8948   -2.9840   -3.7953 H   0  0  0  0  0  0  0  0  0  0  0  0
    2.8907   -1.6386    1.3936 H   0  0  0  0  0  0  0  0  0  0  0  0
    3.1369   -3.1748   -3.5494 H   0  0  0  0  0  0  0  0  0  0  0  0
```

```
    4.1616   -0.5711   -0.3088 H   0  0  0  0  0  0  0  0  0  0  0  0
    4.5475   -1.6969   -2.5784 H   0  0  0  0  0  0  0  0  0  0  0  0
    0.2925   -2.2755   -0.5049 C   0  0  0  0  0  0  0  0  0  0  0  0
   -0.3484   -2.6993    0.3181 H   0  0  0  0  0  0  0  0  0  0  0  0
    1.2824   -1.2492   -0.0216 C   0  0  0  0  0  0  0  0  0  0  0  0
    1.4733   -0.4832   -0.8122 H   0  0  0  0  0  0  0  0  0  0  0  0
    0.8845   -0.7285    0.8830 H   0  0  0  0  0  0  0  0  0  0  0  0
    3.0826   -0.6723   -2.3993 H   0  0  0  0  0  0  0  0  0  0  0  0
    0.4179    2.1659   -1.2028 H   0  0  0  0  0  0  0  0  0  0  0  0
   -2.3905   -5.3813   -0.0051 C   0  0  0  0  0  0  0  0  0  0  0  0
   -2.3923   -6.7541    0.3483 C   0  0  0  0  0  0  0  0  0  0  0  0
   -2.0560   -7.1473    1.6626 C   0  0  0  0  0  0  0  0  0  0  0  0
   -2.7223   -7.7287   -0.6193 C   0  0  0  0  0  0  0  0  0  0  0  0
   -2.0546   -8.4962    1.9970 C   0  0  0  0  0  0  0  0  0  0  0  0
   -1.7996   -6.3938    2.4099 H   0  0  0  0  0  0  0  0  0  0  0  0
   -2.7159   -9.0738   -0.2701 C   0  0  0  0  0  0  0  0  0  0  0  0
   -2.9807   -7.4257   -1.6355 H   0  0  0  0  0  0  0  0  0  0  0  0
   -2.3867   -9.4753    1.0397 C   0  0  0  0  0  0  0  0  0  0  0  0
   -1.7890   -8.8029    3.0125 H   0  0  0  0  0  0  0  0  0  0  0  0
   -2.9636   -9.8289   -1.0208 H   0  0  0  0  0  0  0  0  0  0  0  0
   -2.3658   -4.2516   -0.3051 N   0  0  0  0  0  0  0  0  0  0  0  0
   -5.5243   -2.4110   -0.7351 C   0  0  0  0  0  0  0  0  0  0  0  0
   -6.9398   -2.4341   -0.6715 C   0  0  0  0  0  0  0  0  0  0  0  0
   -7.6868   -3.0148   -1.7195 C   0  0  0  0  0  0  0  0  0  0  0  0
   -7.6011   -1.8673    0.4410 C   0  0  0  0  0  0  0  0  0  0  0  0
   -9.0749   -3.0273   -1.6481 C   0  0  0  0  0  0  0  0  0  0  0  0
   -7.1760   -3.4505   -2.5802 H   0  0  0  0  0  0  0  0  0  0  0  0
   -8.9890   -1.8882    0.4986 C   0  0  0  0  0  0  0  0  0  0  0  0
   -7.0225   -1.4167    1.2496 H   0  0  0  0  0  0  0  0  0  0  0  0
   -9.7430   -2.4699   -0.5402 C   0  0  0  0  0  0  0  0  0  0  0  0
   -9.6544   -3.4695   -2.4627 H   0  0  0  0  0  0  0  0  0  0  0  0
   -9.5034   -1.4456    1.3562 H   0  0  0  0  0  0  0  0  0  0  0  0
   -4.3577   -2.3629   -0.7911 N   0  0  0  0  0  0  0  0  0  0  0  0
   -2.4033   -2.3620   -0.8210 Ir  0  0  0  0  0  0  0  0  0  0  0  0
   -2.3797  -10.9315    1.4083 C   0  0  0  0  0  0  0  0  0  0  0  0
   -2.6846  -11.0586    2.4791 H   0  0  0  0  0  0  0  0  0  0  0  0
   -3.1182  -11.4930    0.7803 H   0  0  0  0  0  0  0  0  0  0  0  0
  -11.2435   -2.4824   -0.4715 C   0  0  0  0  0  0  0  0  0  0  0  0
  -11.5782   -2.5960    0.5919 H   0  0  0  0  0  0  0  0  0  0  0  0
  -11.6512   -3.3532   -1.0464 H   0  0  0  0  0  0  0  0  0  0  0  0
  -11.8163   -1.1840   -1.0492 C   0  0  0  0  0  0  0  0  0  0  0  0
  -12.9287   -1.1973   -0.9670 H   0  0  0  0  0  0  0  0  0  0  0  0
  -11.4302   -0.3019   -0.4898 H   0  0  0  0  0  0  0  0  0  0  0  0
  -11.5400   -1.0810   -2.1233 H   0  0  0  0  0  0  0  0  0  0  0  0
   -0.9847  -11.5311    1.2032 C   0  0  0  0  0  0  0  0  0  0  0  0
   -0.2333  -10.9979    1.8290 H   0  0  0  0  0  0  0  0  0  0  0  0
   -0.9933  -12.6069    1.4981 H   0  0  0  0  0  0  0  0  0  0  0  0
   -0.6827  -11.4580    0.1336 H   0  0  0  0  0  0  0  0  0  0  0  0
  1  2  1  0  0  0  0
  1 11  1  0  0  0  0
  1 17  2  0  0  0  0
  1 69  1  0  0  0  0
  2  4  1  0  0  0  0
  2  7  1  0  0  0  0
  2 12  1  0  0  0  0
  3  5  1  0  0  0  0
  3  9  1  0  0  0  0
  3 13  1  0  0  0  0
  3 19  1  0  0  0  0
  4  6  1  0  0  0  0
  4  8  1  0  0  0  0
  4 14  1  0  0  0  0
  5  6  1  0  0  0  0
  5 10  1  0  0  0  0
  5 15  1  0  0  0  0
```

6	16	1	0	0	0	0
6	44	1	0	0	0	0
17	18	1	0	0	0	0
17	19	1	0	0	0	0
17	69	1	0	0	0	0
19	20	1	0	0	0	0
19	21	1	0	0	0	0
22	23	1	0	0	0	0
22	32	1	0	0	0	0
22	38	2	0	0	0	0
22	69	1	0	0	0	0
23	25	1	0	0	0	0
23	28	1	0	0	0	0
23	33	1	0	0	0	0
24	26	1	0	0	0	0
24	30	1	0	0	0	0
24	34	1	0	0	0	0
24	40	1	0	0	0	0
25	27	1	0	0	0	0
25	29	1	0	0	0	0
25	35	1	0	0	0	0
26	27	1	0	0	0	0
26	31	1	0	0	0	0
26	36	1	0	0	0	0
27	37	1	0	0	0	0
27	43	1	0	0	0	0
38	39	1	0	0	0	0
38	40	1	0	0	0	0
38	69	1	0	0	0	0
40	41	1	0	0	0	0
40	42	1	0	0	0	0
45	46	1	0	0	0	0
45	56	3	0	0	0	0
46	47	2	0	0	0	0
46	48	1	0	0	0	0
47	49	1	0	0	0	0
47	50	1	0	0	0	0
48	51	2	0	0	0	0
48	52	1	0	0	0	0
49	53	2	0	0	0	0
49	54	1	0	0	0	0
51	53	1	0	0	0	0
51	55	1	0	0	0	0
53	70	1	0	0	0	0
56	69	1	0	0	0	0
57	58	1	0	0	0	0
57	68	3	0	0	0	0
58	59	2	0	0	0	0
58	60	1	0	0	0	0
59	61	1	0	0	0	0
59	62	1	0	0	0	0
60	63	2	0	0	0	0
60	64	1	0	0	0	0
61	65	2	0	0	0	0
61	66	1	0	0	0	0
63	65	1	0	0	0	0
63	67	1	0	0	0	0
65	73	1	0	0	0	0
68	69	1	0	0	0	0
70	71	1	0	0	0	0
70	72	1	0	0	0	0
70	80	1	0	0	0	0
73	74	1	0	0	0	0
73	75	1	0	0	0	0
73	76	1	0	0	0	0

```
 76 77  1  0  0  0  0
 76 78  1  0  0  0  0
 76 79  1  0  0  0  0
 80 81  1  0  0  0  0
 80 82  1  0  0  0  0
 80 83  1  0  0  0  0
M  END
$$$$
mol155
Optimized Catalyst 155
Optimized Catalyst 155
 83 88  0  0  0  0  0  0  0  0999 V2000
   -1.6617   -0.2395   -0.5951 C   0  0  0  0  0  0  0  0  0  0  0  0
   -1.1837    0.9347    0.2129 C   0  0  0  0  0  0  0  0  0  0  0  0
   -1.8939    1.7380   -3.1797 C   0  0  0  0  0  0  0  0  0  0  0  0
   -1.8912    2.2600   -0.1501 C   0  0  0  0  0  0  0  0  0  0  0  0
   -1.5455    3.1135   -2.5892 C   0  0  0  0  0  0  0  0  0  0  0  0
   -1.0947    3.1513   -1.1186 C   0  0  0  0  0  0  0  0  0  0  0  0
   -0.0792    1.0514    0.0774 H   0  0  0  0  0  0  0  0  0  0  0  0
   -2.8943    2.0372   -0.5816 H   0  0  0  0  0  0  0  0  0  0  0  0
   -2.9072    1.4365   -2.8194 H   0  0  0  0  0  0  0  0  0  0  0  0
   -2.4528    3.7608   -2.6969 H   0  0  0  0  0  0  0  0  0  0  0  0
   -2.2044   -1.0138    0.0004 H   0  0  0  0  0  0  0  0  0  0  0  0
   -1.3689    0.7258    1.2965 H   0  0  0  0  0  0  0  0  0  0  0  0
   -1.9490    1.8506   -4.2910 H   0  0  0  0  0  0  0  0  0  0  0  0
   -2.0465    2.8319    0.7985 H   0  0  0  0  0  0  0  0  0  0  0  0
   -0.7274    3.5679   -3.2031 H   0  0  0  0  0  0  0  0  0  0  0  0
   -1.1947    4.2133   -0.7737 H   0  0  0  0  0  0  0  0  0  0  0  0
   -1.5180   -0.3947   -1.9241 C   0  0  0  0  0  0  0  0  0  0  0  0
   -1.9638   -1.2798   -2.4406 H   0  0  0  0  0  0  0  0  0  0  0  0
   -0.8893    0.6119   -2.8487 C   0  0  0  0  0  0  0  0  0  0  0  0
    0.0239    1.0500   -2.3781 H   0  0  0  0  0  0  0  0  0  0  0  0
   -0.5793    0.1105   -3.7970 H   0  0  0  0  0  0  0  0  0  0  0  0
    0.6087   -3.7366   -1.5062 C   0  0  0  0  0  0  0  0  0  0  0  0
    1.0023   -2.9747   -2.7474 C   0  0  0  0  0  0  0  0  0  0  0  0
    2.7602   -1.8837    0.1582 C   0  0  0  0  0  0  0  0  0  0  0  0
    2.5157   -2.6639   -2.8160 C   0  0  0  0  0  0  0  0  0  0  0  0
    3.4490   -1.0859   -0.9598 C   0  0  0  0  0  0  0  0  0  0  0  0
    2.8842   -1.2344   -2.3836 C   0  0  0  0  0  0  0  0  0  0  0  0
    0.4022   -2.0177   -2.7896 H   0  0  0  0  0  0  0  0  0  0  0  0
    3.0736   -3.4019   -2.1924 H   0  0  0  0  0  0  0  0  0  0  0  0
    3.1020   -2.9471    0.1052 H   0  0  0  0  0  0  0  0  0  0  0  0
    4.5253   -1.3975   -0.9733 H   0  0  0  0  0  0  0  0  0  0  0  0
    0.4740   -4.8263   -1.6493 H   0  0  0  0  0  0  0  0  0  0  0  0
    0.7199   -3.5844   -3.6449 H   0  0  0  0  0  0  0  0  0  0  0  0
    3.1054   -1.4685    1.1385 H   0  0  0  0  0  0  0  0  0  0  0  0
    2.8410   -2.8025   -3.8779 H   0  0  0  0  0  0  0  0  0  0  0  0
    3.4126    0.0012   -0.6954 H   0  0  0  0  0  0  0  0  0  0  0  0
    3.6704   -0.8524   -3.0868 H   0  0  0  0  0  0  0  0  0  0  0  0
    0.6748   -3.2167   -0.1977 C   0  0  0  0  0  0  0  0  0  0  0  0
    0.5827   -3.9008    0.6673 H   0  0  0  0  0  0  0  0  0  0  0  0
    1.2164   -1.8480    0.1291 C   0  0  0  0  0  0  0  0  0  0  0  0
    0.8564   -1.1069   -0.6372 H   0  0  0  0  0  0  0  0  0  0  0  0
    0.8239   -1.5262    1.1270 H   0  0  0  0  0  0  0  0  0  0  0  0
    1.9970   -0.5675   -2.4959 H   0  0  0  0  0  0  0  0  0  0  0  0
   -0.0113    2.8914   -1.0636 H   0  0  0  0  0  0  0  0  0  0  0  0
   -2.1095   -5.4350    0.0419 C   0  0  0  0  0  0  0  0  0  0  0  0
   -1.7004   -4.4298   -0.3721 N   0  0  0  0  0  0  0  0  0  0  0  0
   -1.2347   -2.9327   -0.9616 Rh  0  0  0  0  0  0  0  0  0  0  0  0
   -4.9976   -2.5563   -1.2855 C   0  0  0  0  0  0  0  0  0  0  0  0
   -3.8473   -2.6898   -1.2468 N   0  0  0  0  0  0  0  0  0  0  0  0
   -2.5668   -6.7049    0.5813 C   0  0  0  0  0  0  0  0  0  0  0  0
   -3.6913   -6.6959    0.6313 H   0  0  0  0  0  0  0  0  0  0  0  0
   -2.1595   -6.8095    1.6280 H   0  0  0  0  0  0  0  0  0  0  0  0
   -6.4406   -2.4006   -1.3133 C   0  0  0  0  0  0  0  0  0  0  0  0
```

```
   -6.7820   -1.9270   -0.3486 H   0  0  0  0  0  0  0  0  0  0  0  0
   -6.9244   -3.4153   -1.3898 H   0  0  0  0  0  0  0  0  0  0  0  0
   -6.9099   -1.5279   -2.4954 C   0  0  0  0  0  0  0  0  0  0  0  0
   -6.6068   -2.0024   -3.4616 H   0  0  0  0  0  0  0  0  0  0  0  0
   -6.4260   -0.5213   -2.4393 H   0  0  0  0  0  0  0  0  0  0  0  0
   -2.1024   -7.9001   -0.2754 C   0  0  0  0  0  0  0  0  0  0  0  0
   -2.5558   -8.8353    0.1490 H   0  0  0  0  0  0  0  0  0  0  0  0
   -2.4889   -7.7836   -1.3187 H   0  0  0  0  0  0  0  0  0  0  0  0
   -0.6016   -8.0447   -0.2889 C   0  0  0  0  0  0  0  0  0  0  0  0
    0.1386   -7.6299   -1.4059 C   0  0  0  0  0  0  0  0  0  0  0  0
    0.0627   -8.6063    0.8127 C   0  0  0  0  0  0  0  0  0  0  0  0
    1.5302   -7.7704   -1.4163 C   0  0  0  0  0  0  0  0  0  0  0  0
   -0.3666   -7.2251   -2.2807 H   0  0  0  0  0  0  0  0  0  0  0  0
    1.4531   -8.7443    0.7969 C   0  0  0  0  0  0  0  0  0  0  0  0
   -0.5015   -8.9540    1.6771 H   0  0  0  0  0  0  0  0  0  0  0  0
    2.1901   -8.3242   -0.3156 C   0  0  0  0  0  0  0  0  0  0  0  0
    2.1010   -7.4596   -2.2916 H   0  0  0  0  0  0  0  0  0  0  0  0
    1.9656   -9.1907    1.6510 H   0  0  0  0  0  0  0  0  0  0  0  0
    3.2742   -8.4430   -0.3278 H   0  0  0  0  0  0  0  0  0  0  0  0
   -8.4102   -1.3796   -2.4462 C   0  0  0  0  0  0  0  0  0  0  0  0
   -9.2313   -2.3138   -3.0940 C   0  0  0  0  0  0  0  0  0  0  0  0
   -8.9889   -0.3122   -1.7441 C   0  0  0  0  0  0  0  0  0  0  0  0
  -10.6214   -2.1767   -3.0420 C   0  0  0  0  0  0  0  0  0  0  0  0
   -8.7933   -3.1450   -3.6430 H   0  0  0  0  0  0  0  0  0  0  0  0
  -10.3795   -0.1800   -1.6958 C   0  0  0  0  0  0  0  0  0  0  0  0
   -8.3611    0.4187   -1.2383 H   0  0  0  0  0  0  0  0  0  0  0  0
  -11.1976   -1.1112   -2.3432 C   0  0  0  0  0  0  0  0  0  0  0  0
  -11.2581   -2.9017   -3.5504 H   0  0  0  0  0  0  0  0  0  0  0  0
  -10.8272    0.6536   -1.1533 H   0  0  0  0  0  0  0  0  0  0  0  0
  -12.2821   -1.0054   -2.3053 H   0  0  0  0  0  0  0  0  0  0  0  0
  1  2  1  0  0  0  0
  1 11  1  0  0  0  0
  1 17  2  0  0  0  0
  1 47  1  0  0  0  0
  2  4  1  0  0  0  0
  2  7  1  0  0  0  0
  2 12  1  0  0  0  0
  3  5  1  0  0  0  0
  3  9  1  0  0  0  0
  3 13  1  0  0  0  0
  3 19  1  0  0  0  0
  4  6  1  0  0  0  0
  4  8  1  0  0  0  0
  4 14  1  0  0  0  0
  5  6  1  0  0  0  0
  5 10  1  0  0  0  0
  5 15  1  0  0  0  0
  6 16  1  0  0  0  0
  6 44  1  0  0  0  0
 17 18  1  0  0  0  0
 17 19  1  0  0  0  0
 17 47  1  0  0  0  0
 19 20  1  0  0  0  0
 19 21  1  0  0  0  0
 22 23  1  0  0  0  0
 22 32  1  0  0  0  0
 22 38  2  0  0  0  0
 22 47  1  0  0  0  0
 23 25  1  0  0  0  0
 23 28  1  0  0  0  0
 23 33  1  0  0  0  0
 24 26  1  0  0  0  0
 24 30  1  0  0  0  0
 24 34  1  0  0  0  0
 24 40  1  0  0  0  0
```

```
 25 27  1  0  0  0  0
 25 29  1  0  0  0  0
 25 35  1  0  0  0  0
 26 27  1  0  0  0  0
 26 31  1  0  0  0  0
 26 36  1  0  0  0  0
 27 37  1  0  0  0  0
 27 43  1  0  0  0  0
 38 39  1  0  0  0  0
 38 40  1  0  0  0  0
 38 47  1  0  0  0  0
 40 41  1  0  0  0  0
 40 42  1  0  0  0  0
 45 46  3  0  0  0  0
 45 50  1  0  0  0  0
 46 47  1  0  0  0  0
 47 49  1  0  0  0  0
 48 49  3  0  0  0  0
 48 53  1  0  0  0  0
 50 51  1  0  0  0  0
 50 52  1  0  0  0  0
 50 59  1  0  0  0  0
 53 54  1  0  0  0  0
 53 55  1  0  0  0  0
 53 56  1  0  0  0  0
 56 57  1  0  0  0  0
 56 58  1  0  0  0  0
 56 73  1  0  0  0  0
 59 60  1  0  0  0  0
 59 61  1  0  0  0  0
 59 62  1  0  0  0  0
 62 63  2  0  0  0  0
 62 64  1  0  0  0  0
 63 65  1  0  0  0  0
 63 66  1  0  0  0  0
 64 67  2  0  0  0  0
 64 68  1  0  0  0  0
 65 69  2  0  0  0  0
 65 70  1  0  0  0  0
 67 69  1  0  0  0  0
 67 71  1  0  0  0  0
 69 72  1  0  0  0  0
 73 74  2  0  0  0  0
 73 75  1  0  0  0  0
 74 76  1  0  0  0  0
 74 77  1  0  0  0  0
 75 78  2  0  0  0  0
 75 79  1  0  0  0  0
 76 80  2  0  0  0  0
 76 81  1  0  0  0  0
 78 80  1  0  0  0  0
 78 82  1  0  0  0  0
 80 83  1  0  0  0  0
M  END
$$$$
mol156
Optimized Catalyst 156
Optimized Catalyst 156
 83 88  0  0  0  0  0  0  0  0999 V2000
   -2.6443    0.2764   -0.7550 C   0  0  0  0  0  0  0  0  0  0  0  0
   -1.7069    1.1636    0.0168 C   0  0  0  0  0  0  0  0  0  0  0  0
   -1.6062    1.6758   -3.4937 C   0  0  0  0  0  0  0  0  0  0  0  0
   -1.6502    2.5994   -0.5620 C   0  0  0  0  0  0  0  0  0  0  0  0
   -0.6941    2.7999   -2.9802 C   0  0  0  0  0  0  0  0  0  0  0  0
   -0.4341    2.8622   -1.4652 C   0  0  0  0  0  0  0  0  0  0  0  0
```

-0.6755	0.7323	0.0270	H	0	0	0	0	0	0	0	0	0	0	0	0
-2.5902	2.8168	-1.1211	H	0	0	0	0	0	0	0	0	0	0	0	0
-2.6687	1.9462	-3.2774	H	0	0	0	0	0	0	0	0	0	0	0	0
-1.1569	3.7702	-3.2986	H	0	0	0	0	0	0	0	0	0	0	0	0
-3.6174	0.1049	-0.2476	H	0	0	0	0	0	0	0	0	0	0	0	0
-2.0561	1.2321	1.0801	H	0	0	0	0	0	0	0	0	0	0	0	0
-1.4931	1.6260	-4.6063	H	0	0	0	0	0	0	0	0	0	0	0	0
-1.6001	3.3072	0.3046	H	0	0	0	0	0	0	0	0	0	0	0	0
0.2993	2.7160	-3.4895	H	0	0	0	0	0	0	0	0	0	0	0	0
-0.0593	3.8970	-1.2428	H	0	0	0	0	0	0	0	0	0	0	0	0
-2.4537	-0.1554	-2.0319	C	0	0	0	0	0	0	0	0	0	0	0	0
-3.2808	-0.6534	-2.5934	H	0	0	0	0	0	0	0	0	0	0	0	0
-1.3036	0.2757	-2.9077	C	0	0	0	0	0	0	0	0	0	0	0	0
-0.3506	0.3222	-2.3257	H	0	0	0	0	0	0	0	0	0	0	0	0
-1.1716	-0.4481	-3.7499	H	0	0	0	0	0	0	0	0	0	0	0	0
0.2754	-2.8390	-1.7235	C	0	0	0	0	0	0	0	0	0	0	0	0
1.1979	-2.4661	-2.8472	C	0	0	0	0	0	0	0	0	0	0	0	0
2.5831	-1.9451	0.3735	C	0	0	0	0	0	0	0	0	0	0	0	0
2.6747	-2.8279	-2.5599	C	0	0	0	0	0	0	0	0	0	0	0	0
3.7663	-1.5936	-0.5404	C	0	0	0	0	0	0	0	0	0	0	0	0
3.5196	-1.6454	-2.0577	C	0	0	0	0	0	0	0	0	0	0	0	0
1.1239	-1.3699	-3.0575	H	0	0	0	0	0	0	0	0	0	0	0	0
2.7205	-3.6650	-1.8256	H	0	0	0	0	0	0	0	0	0	0	0	0
2.4417	-3.0529	0.3730	H	0	0	0	0	0	0	0	0	0	0	0	0
4.5976	-2.3032	-0.2928	H	0	0	0	0	0	0	0	0	0	0	0	0
-0.3967	-3.6946	-1.9627	H	0	0	0	0	0	0	0	0	0	0	0	0
0.8817	-3.0078	-3.7757	H	0	0	0	0	0	0	0	0	0	0	0	0
2.8565	-1.6409	1.4154	H	0	0	0	0	0	0	0	0	0	0	0	0
3.1229	-3.1928	-3.5189	H	0	0	0	0	0	0	0	0	0	0	0	0
4.1169	-0.5601	-0.2905	H	0	0	0	0	0	0	0	0	0	0	0	0
4.5262	-1.7074	-2.5499	H	0	0	0	0	0	0	0	0	0	0	0	0
0.2643	-2.2884	-0.4895	C	0	0	0	0	0	0	0	0	0	0	0	0
-0.3793	-2.7111	0.3324	H	0	0	0	0	0	0	0	0	0	0	0	0
1.2489	-1.2588	-0.0026	C	0	0	0	0	0	0	0	0	0	0	0	0
1.4386	-0.4903	-0.7914	H	0	0	0	0	0	0	0	0	0	0	0	0
0.8472	-0.7417	0.9026	H	0	0	0	0	0	0	0	0	0	0	0	0
3.0585	-0.6843	-2.3865	H	0	0	0	0	0	0	0	0	0	0	0	0
0.3912	2.1585	-1.2050	H	0	0	0	0	0	0	0	0	0	0	0	0
-2.3907	-5.4065	0.0159	C	0	0	0	0	0	0	0	0	0	0	0	0
-2.3842	-4.2798	-0.2866	N	0	0	0	0	0	0	0	0	0	0	0	0
-5.5464	-2.4280	-0.7360	C	0	0	0	0	0	0	0	0	0	0	0	0
-4.3814	-2.3938	-0.7849	N	0	0	0	0	0	0	0	0	0	0	0	0
-2.4274	-2.3941	-0.8122	Ir	0	0	0	0	0	0	0	0	0	0	0	0
-2.3841	-6.8056	0.3859	C	0	0	0	0	0	0	0	0	0	0	0	0
-2.9207	-6.9386	1.3720	H	0	0	0	0	0	0	0	0	0	0	0	0
-2.9434	-7.4044	-0.3933	H	0	0	0	0	0	0	0	0	0	0	0	0
-6.9923	-2.4487	-0.6781	C	0	0	0	0	0	0	0	0	0	0	0	0
-7.3887	-3.2244	-1.3987	H	0	0	0	0	0	0	0	0	0	0	0	0
-7.3240	-2.7438	0.3617	H	0	0	0	0	0	0	0	0	0	0	0	0
-7.6006	-1.0730	-1.0309	C	0	0	0	0	0	0	0	0	0	0	0	0
-7.2885	-0.7701	-2.0614	H	0	0	0	0	0	0	0	0	0	0	0	0
-7.2349	-0.2978	-0.3125	H	0	0	0	0	0	0	0	0	0	0	0	0
-0.9479	-7.3607	0.5136	C	0	0	0	0	0	0	0	0	0	0	0	0
-0.4057	-7.2449	-0.4577	H	0	0	0	0	0	0	0	0	0	0	0	0
-0.3869	-6.7919	1.2966	H	0	0	0	0	0	0	0	0	0	0	0	0
-9.1059	-1.1578	-0.9625	C	0	0	0	0	0	0	0	0	0	0	0	0
-9.8406	-1.5175	-2.1015	C	0	0	0	0	0	0	0	0	0	0	0	0
-9.7721	-0.8728	0.2380	C	0	0	0	0	0	0	0	0	0	0	0	0
-11.2353	-1.5849	-2.0387	C	0	0	0	0	0	0	0	0	0	0	0	0
-9.3347	-1.7379	-3.0396	H	0	0	0	0	0	0	0	0	0	0	0	0
-11.1672	-0.9418	0.2943	C	0	0	0	0	0	0	0	0	0	0	0	0
-9.2129	-0.5900	1.1279	H	0	0	0	0	0	0	0	0	0	0	0	0
-11.9001	-1.2986	-0.8421	C	0	0	0	0	0	0	0	0	0	0	0	0
-11.8066	-1.8579	-2.9276	H	0	0	0	0	0	0	0	0	0	0	0	0
-11.6854	-0.7127	1.2271	H	0	0	0	0	0	0	0	0	0	0	0	0

```
 -12.9890   -1.3515   -0.7957 H   0  0  0  0  0  0  0  0  0  0  0  0
  -1.0065   -8.8208    0.8910 C   0  0  0  0  0  0  0  0  0  0  0  0
  -1.0194   -9.1969    2.2419 C   0  0  0  0  0  0  0  0  0  0  0  0
  -1.0440   -9.8036   -0.1085 C   0  0  0  0  0  0  0  0  0  0  0  0
  -1.0632  -10.5503    2.5883 C   0  0  0  0  0  0  0  0  0  0  0  0
  -0.9878   -8.4431    3.0264 H   0  0  0  0  0  0  0  0  0  0  0  0
  -1.0879  -11.1555    0.2444 C   0  0  0  0  0  0  0  0  0  0  0  0
  -1.0318   -9.5243   -1.1603 H   0  0  0  0  0  0  0  0  0  0  0  0
  -1.0986  -11.5308    1.5916 C   0  0  0  0  0  0  0  0  0  0  0  0
  -1.0662  -10.8422    3.6399 H   0  0  0  0  0  0  0  0  0  0  0  0
  -1.1101  -11.9198   -0.5342 H   0  0  0  0  0  0  0  0  0  0  0  0
  -1.1323  -12.5866    1.8645 H   0  0  0  0  0  0  0  0  0  0  0  0
  1  2  1  0  0  0  0
  1 11  1  0  0  0  0
  1 17  2  0  0  0  0
  1 49  1  0  0  0  0
  2  4  1  0  0  0  0
  2  7  1  0  0  0  0
  2 12  1  0  0  0  0
  3  5  1  0  0  0  0
  3  9  1  0  0  0  0
  3 13  1  0  0  0  0
  3 19  1  0  0  0  0
  4  6  1  0  0  0  0
  4  8  1  0  0  0  0
  4 14  1  0  0  0  0
  5  6  1  0  0  0  0
  5 10  1  0  0  0  0
  5 15  1  0  0  0  0
  6 16  1  0  0  0  0
  6 44  1  0  0  0  0
 17 18  1  0  0  0  0
 17 19  1  0  0  0  0
 17 49  1  0  0  0  0
 19 20  1  0  0  0  0
 19 21  1  0  0  0  0
 22 23  1  0  0  0  0
 22 32  1  0  0  0  0
 22 38  2  0  0  0  0
 22 49  1  0  0  0  0
 23 25  1  0  0  0  0
 23 28  1  0  0  0  0
 23 33  1  0  0  0  0
 24 26  1  0  0  0  0
 24 30  1  0  0  0  0
 24 34  1  0  0  0  0
 24 40  1  0  0  0  0
 25 27  1  0  0  0  0
 25 29  1  0  0  0  0
 25 35  1  0  0  0  0
 26 27  1  0  0  0  0
 26 31  1  0  0  0  0
 26 36  1  0  0  0  0
 27 37  1  0  0  0  0
 27 43  1  0  0  0  0
 38 39  1  0  0  0  0
 38 40  1  0  0  0  0
 38 49  1  0  0  0  0
 40 41  1  0  0  0  0
 40 42  1  0  0  0  0
 45 46  3  0  0  0  0
 45 50  1  0  0  0  0
 46 49  1  0  0  0  0
 47 48  3  0  0  0  0
 47 53  1  0  0  0  0
```

```
 48 49  1  0  0  0  0
 50 51  1  0  0  0  0
 50 52  1  0  0  0  0
 50 59  1  0  0  0  0
 53 54  1  0  0  0  0
 53 55  1  0  0  0  0
 53 56  1  0  0  0  0
 56 57  1  0  0  0  0
 56 58  1  0  0  0  0
 56 62  1  0  0  0  0
 59 60  1  0  0  0  0
 59 61  1  0  0  0  0
 59 73  1  0  0  0  0
 62 63  2  0  0  0  0
 62 64  1  0  0  0  0
 63 65  1  0  0  0  0
 63 66  1  0  0  0  0
 64 67  2  0  0  0  0
 64 68  1  0  0  0  0
 65 69  2  0  0  0  0
 65 70  1  0  0  0  0
 67 69  1  0  0  0  0
 67 71  1  0  0  0  0
 69 72  1  0  0  0  0
 73 74  2  0  0  0  0
 73 75  1  0  0  0  0
 74 76  1  0  0  0  0
 74 77  1  0  0  0  0
 75 78  2  0  0  0  0
 75 79  1  0  0  0  0
 76 80  2  0  0  0  0
 76 81  1  0  0  0  0
 78 80  1  0  0  0  0
 78 82  1  0  0  0  0
 80 83  1  0  0  0  0
M  END
$$$$
mol157
Optimized Catalyst 157
Optimized Catalyst 157
 65 70  0  0  0  0  0  0  0  0999 V2000
   -1.0204    0.3009   -1.5779 C   0  0  0  0  0  0  0  0  0  0  0  0
    0.1555   -0.6200   -1.1907 C   0  0  0  0  0  0  0  0  0  0  0  0
   -2.2734    0.0734   -0.7654 C   0  0  0  0  0  0  0  0  0  0  0  0
   -1.1487    0.1176    1.5364 C   0  0  0  0  0  0  0  0  0  0  0  0
   -2.3386   -0.0330    0.6173 C   0  0  0  0  0  0  0  0  0  0  0  0
   -0.4685   -1.2361    1.8311 C   0  0  0  0  0  0  0  0  0  0  0  0
   -3.2089    0.1925   -1.3451 H   0  0  0  0  0  0  0  0  0  0  0  0
   -3.3211   -0.0454    1.1240 H   0  0  0  0  0  0  0  0  0  0  0  0
   -1.2642    0.1242   -2.6591 H   0  0  0  0  0  0  0  0  0  0  0  0
   -0.7110    1.3739   -1.4607 H   0  0  0  0  0  0  0  0  0  0  0  0
    0.7894   -0.7930   -2.0999 H   0  0  0  0  0  0  0  0  0  0  0  0
   -0.9780   -1.6965    2.7202 H   0  0  0  0  0  0  0  0  0  0  0  0
   -1.4891    0.5663    2.5063 H   0  0  0  0  0  0  0  0  0  0  0  0
   -0.4158    0.8322    1.0814 H   0  0  0  0  0  0  0  0  0  0  0  0
   -0.5372   -2.2285    0.6908 C   0  0  0  0  0  0  0  0  0  0  0  0
   -0.3073   -1.9560   -0.6558 C   0  0  0  0  0  0  0  0  0  0  0  0
   -0.6311   -3.2905    1.0179 H   0  0  0  0  0  0  0  0  0  0  0  0
   -0.2710   -2.7953   -1.3858 H   0  0  0  0  0  0  0  0  0  0  0  0
    0.8050   -0.1221   -0.4260 H   0  0  0  0  0  0  0  0  0  0  0  0
    0.6097   -1.0664    2.0909 H   0  0  0  0  0  0  0  0  0  0  0  0
   -4.0039   -2.0903   -0.4799 N   0  0  0  0  0  0  0  0  0  0  0  0
   -5.1420   -2.2714   -0.6414 C   0  0  0  0  0  0  0  0  0  0  0  0
   -2.3678   -4.4663   -0.6606 N   0  0  0  0  0  0  0  0  0  0  0  0
   -2.4813   -5.5995   -0.8825 C   0  0  0  0  0  0  0  0  0  0  0  0
```

```
   -2.3415   -2.0416   -0.2541 Rh  0  0  0  0  0  0  0  0  0  0  0  0
   -2.6071   -6.9955   -1.1556 C   0  0  0  0  0  0  0  0  0  0  0  0
   -2.6925   -7.4468   -2.4867 C   0  0  0  0  0  0  0  0  0  0  0  0
   -2.6388   -7.9201   -0.0940 C   0  0  0  0  0  0  0  0  0  0  0  0
   -2.8001   -8.8099   -2.7481 C   0  0  0  0  0  0  0  0  0  0  0  0
   -2.6712   -6.7295   -3.3082 H   0  0  0  0  0  0  0  0  0  0  0  0
   -2.7468   -9.2812   -0.3666 C   0  0  0  0  0  0  0  0  0  0  0  0
   -2.5764   -7.5691    0.9370 H   0  0  0  0  0  0  0  0  0  0  0  0
   -2.8224   -9.7418   -1.6939 C   0  0  0  0  0  0  0  0  0  0  0  0
   -2.8683   -9.1596   -3.7802 H   0  0  0  0  0  0  0  0  0  0  0  0
   -2.7740   -9.9977    0.4569 H   0  0  0  0  0  0  0  0  0  0  0  0
   -6.5429   -2.4503   -0.8363 C   0  0  0  0  0  0  0  0  0  0  0  0
   -7.0625   -2.5526   -2.1417 C   0  0  0  0  0  0  0  0  0  0  0  0
   -7.4027   -2.5270    0.2768 C   0  0  0  0  0  0  0  0  0  0  0  0
   -8.4290   -2.7472   -2.3243 C   0  0  0  0  0  0  0  0  0  0  0  0
   -6.3972   -2.4810   -3.0029 H   0  0  0  0  0  0  0  0  0  0  0  0
   -8.7672   -2.7223    0.0797 C   0  0  0  0  0  0  0  0  0  0  0  0
   -7.0005   -2.4364    1.2865 H   0  0  0  0  0  0  0  0  0  0  0  0
   -9.2945   -2.8457   -1.2190 C   0  0  0  0  0  0  0  0  0  0  0  0
   -8.8340   -2.8226   -3.3357 H   0  0  0  0  0  0  0  0  0  0  0  0
   -9.4353   -2.7785    0.9418 H   0  0  0  0  0  0  0  0  0  0  0  0
  -10.7645   -3.0730   -1.4234 C   0  0  0  0  0  0  0  0  0  0  0  0
  -10.9358   -3.6856   -2.3438 H   0  0  0  0  0  0  0  0  0  0  0  0
  -11.1917   -3.6408   -0.5592 H   0  0  0  0  0  0  0  0  0  0  0  0
  -11.5084   -1.7296   -1.5633 C   0  0  0  0  0  0  0  0  0  0  0  0
  -11.0948   -1.1731   -2.4390 H   0  0  0  0  0  0  0  0  0  0  0  0
  -11.3362   -1.1199   -0.6433 H   0  0  0  0  0  0  0  0  0  0  0  0
  -13.0102   -1.9358   -1.7590 C   0  0  0  0  0  0  0  0  0  0  0  0
  -13.4516   -2.4683   -0.8867 H   0  0  0  0  0  0  0  0  0  0  0  0
  -13.5142   -0.9468   -1.8608 H   0  0  0  0  0  0  0  0  0  0  0  0
  -13.2094   -2.5294   -2.6793 H   0  0  0  0  0  0  0  0  0  0  0  0
   -2.9385  -11.2107   -1.9821 C   0  0  0  0  0  0  0  0  0  0  0  0
   -2.4349  -11.4554   -2.9508 H   0  0  0  0  0  0  0  0  0  0  0  0
   -2.4306  -11.8043   -1.1813 H   0  0  0  0  0  0  0  0  0  0  0  0
   -4.4210  -11.6278   -2.0601 C   0  0  0  0  0  0  0  0  0  0  0  0
   -4.9202  -11.0476   -2.8738 H   0  0  0  0  0  0  0  0  0  0  0  0
   -4.9200  -11.3807   -1.0916 H   0  0  0  0  0  0  0  0  0  0  0  0
   -4.5721  -13.1228   -2.3396 C   0  0  0  0  0  0  0  0  0  0  0  0
   -4.1020  -13.7238   -1.5289 H   0  0  0  0  0  0  0  0  0  0  0  0
   -5.6536  -13.3866   -2.3942 H   0  0  0  0  0  0  0  0  0  0  0  0
   -4.0930  -13.3921   -3.3077 H   0  0  0  0  0  0  0  0  0  0  0  0
  1  2  1  0  0  0  0
  1  3  1  0  0  0  0
  1  9  1  0  0  0  0
  1 10  1  0  0  0  0
  2 11  1  0  0  0  0
  2 16  1  0  0  0  0
  2 19  1  0  0  0  0
  3  5  2  0  0  0  0
  3  7  1  0  0  0  0
  3 25  1  0  0  0  0
  4  5  1  0  0  0  0
  4  6  1  0  0  0  0
  4 13  1  0  0  0  0
  4 14  1  0  0  0  0
  5  8  1  0  0  0  0
  5 25  1  0  0  0  0
  6 12  1  0  0  0  0
  6 15  1  0  0  0  0
  6 20  1  0  0  0  0
 15 16  2  0  0  0  0
 15 17  1  0  0  0  0
 15 25  1  0  0  0  0
 16 18  1  0  0  0  0
 16 25  1  0  0  0  0
```

```
 21 22  3  0  0  0  0
 21 25  1  0  0  0  0
 22 36  1  0  0  0  0
 23 24  3  0  0  0  0
 23 25  1  0  0  0  0
 24 26  1  0  0  0  0
 26 27  2  0  0  0  0
 26 28  1  0  0  0  0
 27 29  1  0  0  0  0
 27 30  1  0  0  0  0
 28 31  2  0  0  0  0
 28 32  1  0  0  0  0
 29 33  2  0  0  0  0
 29 34  1  0  0  0  0
 31 33  1  0  0  0  0
 31 35  1  0  0  0  0
 33 56  1  0  0  0  0
 36 37  2  0  0  0  0
 36 38  1  0  0  0  0
 37 39  1  0  0  0  0
 37 40  1  0  0  0  0
 38 41  2  0  0  0  0
 38 42  1  0  0  0  0
 39 43  2  0  0  0  0
 39 44  1  0  0  0  0
 41 43  1  0  0  0  0
 41 45  1  0  0  0  0
 43 46  1  0  0  0  0
 46 47  1  0  0  0  0
 46 48  1  0  0  0  0
 46 49  1  0  0  0  0
 49 50  1  0  0  0  0
 49 51  1  0  0  0  0
 49 52  1  0  0  0  0
 52 53  1  0  0  0  0
 52 54  1  0  0  0  0
 52 55  1  0  0  0  0
 56 57  1  0  0  0  0
 56 58  1  0  0  0  0
 56 59  1  0  0  0  0
 59 60  1  0  0  0  0
 59 61  1  0  0  0  0
 59 62  1  0  0  0  0
 62 63  1  0  0  0  0
 62 64  1  0  0  0  0
 62 65  1  0  0  0  0
M  END
$$$$
mol158
Optimized Catalyst 158
Optimized Catalyst 158
 65 70  0  0  0  0  0  0  0  0999 V2000
   -0.8395    0.2021   -1.5446 C   0  0  0  0  0  0  0  0  0  0  0  0
    0.3914   -0.6439   -1.1569 C   0  0  0  0  0  0  0  0  0  0  0  0
   -2.0986   -0.0513   -0.7469 C   0  0  0  0  0  0  0  0  0  0  0  0
   -0.9980   -0.0181    1.5563 C   0  0  0  0  0  0  0  0  0  0  0  0
   -2.1744   -0.1740    0.6201 C   0  0  0  0  0  0  0  0  0  0  0  0
   -0.1976   -1.3126    1.8145 C   0  0  0  0  0  0  0  0  0  0  0  0
   -3.0317    0.1086   -1.3348 H   0  0  0  0  0  0  0  0  0  0  0  0
   -3.1655   -0.1587    1.1234 H   0  0  0  0  0  0  0  0  0  0  0  0
   -1.0574    0.0365   -2.6356 H   0  0  0  0  0  0  0  0  0  0  0  0
   -0.5811    1.2940   -1.4212 H   0  0  0  0  0  0  0  0  0  0  0  0
    1.0463   -0.7444   -2.0664 H   0  0  0  0  0  0  0  0  0  0  0  0
   -0.6065   -1.8086    2.7373 H   0  0  0  0  0  0  0  0  0  0  0  0
   -1.3745    0.3619    2.5463 H   0  0  0  0  0  0  0  0  0  0  0  0
```

```
   -0.3192    0.7789    1.1466 H   0  0  0  0  0  0  0  0  0  0  0  0
   -0.1740   -2.3158    0.6834 C   0  0  0  0  0  0  0  0  0  0  0  0
    0.0676   -2.0254   -0.6369 C   0  0  0  0  0  0  0  0  0  0  0  0
   -0.1301   -3.3736    1.0305 H   0  0  0  0  0  0  0  0  0  0  0  0
    0.2557   -2.8459   -1.3618 H   0  0  0  0  0  0  0  0  0  0  0  0
    1.0019   -0.1013   -0.3848 H   0  0  0  0  0  0  0  0  0  0  0  0
    0.8730   -1.0344    2.0385 H   0  0  0  0  0  0  0  0  0  0  0  0
   -4.2718   -2.3607   -0.5961 N   0  0  0  0  0  0  0  0  0  0  0  0
   -5.4253   -2.4761   -0.7448 C   0  0  0  0  0  0  0  0  0  0  0  0
   -2.2969   -4.3278   -0.6238 N   0  0  0  0  0  0  0  0  0  0  0  0
   -2.4097   -5.4720   -0.8354 C   0  0  0  0  0  0  0  0  0  0  0  0
   -2.3215   -2.3791   -0.3406 Ir  0  0  0  0  0  0  0  0  0  0  0  0
   -2.5237   -6.8601   -1.1031 C   0  0  0  0  0  0  0  0  0  0  0  0
   -2.6042   -7.3105   -2.4399 C   0  0  0  0  0  0  0  0  0  0  0  0
   -2.5623   -7.7908   -0.0419 C   0  0  0  0  0  0  0  0  0  0  0  0
   -2.7217   -8.6697   -2.7026 C   0  0  0  0  0  0  0  0  0  0  0  0
   -2.5747   -6.5919   -3.2610 H   0  0  0  0  0  0  0  0  0  0  0  0
   -2.6797   -9.1476   -0.3204 C   0  0  0  0  0  0  0  0  0  0  0  0
   -2.5014   -7.4445    0.9916 H   0  0  0  0  0  0  0  0  0  0  0  0
   -2.7592   -9.6065   -1.6502 C   0  0  0  0  0  0  0  0  0  0  0  0
   -2.7874   -9.0181   -3.7369 H   0  0  0  0  0  0  0  0  0  0  0  0
   -2.7132   -9.8683    0.5012 H   0  0  0  0  0  0  0  0  0  0  0  0
   -6.8263   -2.6038   -0.9215 C   0  0  0  0  0  0  0  0  0  0  0  0
   -7.3693   -2.6856   -2.2227 C   0  0  0  0  0  0  0  0  0  0  0  0
   -7.6767   -2.6635    0.2045 C   0  0  0  0  0  0  0  0  0  0  0  0
   -8.7402   -2.8415   -2.3867 C   0  0  0  0  0  0  0  0  0  0  0  0
   -6.7136   -2.6264   -3.0934 H   0  0  0  0  0  0  0  0  0  0  0  0
   -9.0458   -2.8186    0.0247 C   0  0  0  0  0  0  0  0  0  0  0  0
   -7.2580   -2.5887    1.2100 H   0  0  0  0  0  0  0  0  0  0  0  0
   -9.5954   -2.9168   -1.2690 C   0  0  0  0  0  0  0  0  0  0  0  0
   -9.1621   -2.9031   -3.3934 H   0  0  0  0  0  0  0  0  0  0  0  0
   -9.7051   -2.8621    0.8959 H   0  0  0  0  0  0  0  0  0  0  0  0
  -11.0734   -3.0847   -1.4552 C   0  0  0  0  0  0  0  0  0  0  0  0
  -11.2836   -3.6910   -2.3726 H   0  0  0  0  0  0  0  0  0  0  0  0
  -11.5178   -3.6309   -0.5850 H   0  0  0  0  0  0  0  0  0  0  0  0
  -11.7594   -1.7089   -1.5906 C   0  0  0  0  0  0  0  0  0  0  0  0
  -11.3237   -1.1668   -2.4651 H   0  0  0  0  0  0  0  0  0  0  0  0
  -11.5633   -1.1103   -0.6676 H   0  0  0  0  0  0  0  0  0  0  0  0
  -13.2676   -1.8561   -1.7875 C   0  0  0  0  0  0  0  0  0  0  0  0
  -13.7302   -2.3780   -0.9196 H   0  0  0  0  0  0  0  0  0  0  0  0
  -13.7344   -0.8478   -1.8818 H   0  0  0  0  0  0  0  0  0  0  0  0
  -13.4904   -2.4347   -2.7123 H   0  0  0  0  0  0  0  0  0  0  0  0
   -2.8950  -11.0707   -1.9432 C   0  0  0  0  0  0  0  0  0  0  0  0
   -2.3959  -11.3202   -2.9140 H   0  0  0  0  0  0  0  0  0  0  0  0
   -2.3942  -11.6757   -1.1453 H   0  0  0  0  0  0  0  0  0  0  0  0
   -4.3822  -11.4749   -2.0228 C   0  0  0  0  0  0  0  0  0  0  0  0
   -4.8779  -10.8897   -2.8351 H   0  0  0  0  0  0  0  0  0  0  0  0
   -4.8805  -11.2303   -1.0532 H   0  0  0  0  0  0  0  0  0  0  0  0
   -4.5401  -12.9679   -2.3086 C   0  0  0  0  0  0  0  0  0  0  0  0
   -4.0726  -13.5766   -1.5018 H   0  0  0  0  0  0  0  0  0  0  0  0
   -5.6230  -13.2278   -2.3629 H   0  0  0  0  0  0  0  0  0  0  0  0
   -4.0648  -13.2373   -3.2788 H   0  0  0  0  0  0  0  0  0  0  0  0
  1  2  1  0  0  0  0
  1  3  1  0  0  0  0
  1  9  1  0  0  0  0
  1 10  1  0  0  0  0
  2 11  1  0  0  0  0
  2 16  1  0  0  0  0
  2 19  1  0  0  0  0
  3  5  2  0  0  0  0
  3  7  1  0  0  0  0
  3 25  1  0  0  0  0
  4  5  1  0  0  0  0
  4  6  1  0  0  0  0
  4 13  1  0  0  0  0
```

```
  4 14  1  0  0  0  0
  5  8  1  0  0  0  0
  5 25  1  0  0  0  0
  6 12  1  0  0  0  0
  6 15  1  0  0  0  0
  6 20  1  0  0  0  0
 15 16  2  0  0  0  0
 15 17  1  0  0  0  0
 15 25  1  0  0  0  0
 16 18  1  0  0  0  0
 16 25  1  0  0  0  0
 21 22  3  0  0  0  0
 21 25  1  0  0  0  0
 22 36  1  0  0  0  0
 23 24  3  0  0  0  0
 23 25  1  0  0  0  0
 24 26  1  0  0  0  0
 26 27  2  0  0  0  0
 26 28  1  0  0  0  0
 27 29  1  0  0  0  0
 27 30  1  0  0  0  0
 28 31  2  0  0  0  0
 28 32  1  0  0  0  0
 29 33  2  0  0  0  0
 29 34  1  0  0  0  0
 31 33  1  0  0  0  0
 31 35  1  0  0  0  0
 33 56  1  0  0  0  0
 36 37  2  0  0  0  0
 36 38  1  0  0  0  0
 37 39  1  0  0  0  0
 37 40  1  0  0  0  0
 38 41  2  0  0  0  0
 38 42  1  0  0  0  0
 39 43  2  0  0  0  0
 39 44  1  0  0  0  0
 41 43  1  0  0  0  0
 41 45  1  0  0  0  0
 43 46  1  0  0  0  0
 46 47  1  0  0  0  0
 46 48  1  0  0  0  0
 46 49  1  0  0  0  0
 49 50  1  0  0  0  0
 49 51  1  0  0  0  0
 49 52  1  0  0  0  0
 52 53  1  0  0  0  0
 52 54  1  0  0  0  0
 52 55  1  0  0  0  0
 56 57  1  0  0  0  0
 56 58  1  0  0  0  0
 56 59  1  0  0  0  0
 59 60  1  0  0  0  0
 59 61  1  0  0  0  0
 59 62  1  0  0  0  0
 62 63  1  0  0  0  0
 62 64  1  0  0  0  0
 62 65  1  0  0  0  0
M  END
$$$$
mol159
Optimized Catalyst 159
Optimized Catalyst 159
 65 70  0  0  0  0  0  0  0  0999 V2000
   -0.9881    0.3395   -1.5702 C   0  0  0  0  0  0  0  0  0  0  0  0
    0.1835   -0.5900   -1.1896 C   0  0  0  0  0  0  0  0  0  0  0  0
```

```
   -2.2433    0.1109   -0.7613 C   0  0  0  0  0  0  0  0  0  0  0  0
   -1.1261    0.1300    1.5457 C   0  0  0  0  0  0  0  0  0  0  0  0
   -2.3128   -0.0102    0.6209 C   0  0  0  0  0  0  0  0  0  0  0  0
   -0.4478   -1.2268    1.8280 C   0  0  0  0  0  0  0  0  0  0  0  0
   -3.1776    0.2433   -1.3407 H   0  0  0  0  0  0  0  0  0  0  0  0
   -3.2978   -0.0221    1.1244 H   0  0  0  0  0  0  0  0  0  0  0  0
   -1.2322    0.1729   -2.6530 H   0  0  0  0  0  0  0  0  0  0  0  0
   -0.6738    1.4099   -1.4440 H   0  0  0  0  0  0  0  0  0  0  0  0
    0.8171   -0.7589   -2.0998 H   0  0  0  0  0  0  0  0  0  0  0  0
   -0.9557   -1.6944    2.7144 H   0  0  0  0  0  0  0  0  0  0  0  0
   -1.4704    0.5685    2.5192 H   0  0  0  0  0  0  0  0  0  0  0  0
   -0.3914    0.8490    1.1010 H   0  0  0  0  0  0  0  0  0  0  0  0
   -0.5204   -2.2087    0.6792 C   0  0  0  0  0  0  0  0  0  0  0  0
   -0.2853   -1.9280   -0.6650 C   0  0  0  0  0  0  0  0  0  0  0  0
   -0.6205   -3.2727    0.9991 H   0  0  0  0  0  0  0  0  0  0  0  0
   -0.2443   -2.7635   -1.3984 H   0  0  0  0  0  0  0  0  0  0  0  0
    0.8340   -0.1007   -0.4202 H   0  0  0  0  0  0  0  0  0  0  0  0
    0.6314   -1.0616    2.0869 H   0  0  0  0  0  0  0  0  0  0  0  0
   -3.9888   -2.0374   -0.4855 N   0  0  0  0  0  0  0  0  0  0  0  0
   -5.1278   -2.2135   -0.6316 C   0  0  0  0  0  0  0  0  0  0  0  0
   -2.3455   -4.4416   -0.6342 N   0  0  0  0  0  0  0  0  0  0  0  0
   -2.4269   -5.5788   -0.8399 C   0  0  0  0  0  0  0  0  0  0  0  0
   -2.5305   -7.0044   -1.1049 C   0  0  0  0  0  0  0  0  0  0  0  0
   -3.6000   -7.3319   -0.9631 H   0  0  0  0  0  0  0  0  0  0  0  0
   -2.2574   -7.2014   -2.1816 H   0  0  0  0  0  0  0  0  0  0  0  0
   -6.5604   -2.3860   -0.8088 C   0  0  0  0  0  0  0  0  0  0  0  0
   -6.7951   -2.3687   -1.9122 H   0  0  0  0  0  0  0  0  0  0  0  0
   -6.8580   -3.3985   -0.4107 H   0  0  0  0  0  0  0  0  0  0  0  0
   -7.3418   -1.3048   -0.0970 C   0  0  0  0  0  0  0  0  0  0  0  0
   -7.7294   -1.4821    1.2389 C   0  0  0  0  0  0  0  0  0  0  0  0
   -7.6980   -0.1300   -0.7741 C   0  0  0  0  0  0  0  0  0  0  0  0
   -8.4667   -0.4928    1.8902 C   0  0  0  0  0  0  0  0  0  0  0  0
   -7.4642   -2.3931    1.7740 H   0  0  0  0  0  0  0  0  0  0  0  0
   -8.4356    0.8574   -0.1196 C   0  0  0  0  0  0  0  0  0  0  0  0
   -7.4102    0.0171   -1.8142 H   0  0  0  0  0  0  0  0  0  0  0  0
   -8.8272    0.6870    1.2185 C   0  0  0  0  0  0  0  0  0  0  0  0
   -8.7748   -0.6414    2.9259 H   0  0  0  0  0  0  0  0  0  0  0  0
   -8.7197    1.7641   -0.6552 H   0  0  0  0  0  0  0  0  0  0  0  0
   -1.6260   -7.8018   -0.1931 C   0  0  0  0  0  0  0  0  0  0  0  0
   -2.1017   -8.2612    1.0433 C   0  0  0  0  0  0  0  0  0  0  0  0
   -0.3137   -8.0970   -0.5877 C   0  0  0  0  0  0  0  0  0  0  0  0
   -1.2697   -9.0093    1.8770 C   0  0  0  0  0  0  0  0  0  0  0  0
   -3.1208   -8.0422    1.3589 H   0  0  0  0  0  0  0  0  0  0  0  0
    0.5153   -8.8455    0.2490 C   0  0  0  0  0  0  0  0  0  0  0  0
    0.0657   -7.7501   -1.5480 H   0  0  0  0  0  0  0  0  0  0  0  0
    0.0479   -9.3056    1.4909 C   0  0  0  0  0  0  0  0  0  0  0  0
   -1.6491   -9.3749    2.8319 H   0  0  0  0  0  0  0  0  0  0  0  0
    1.5309   -9.0833   -0.0694 H   0  0  0  0  0  0  0  0  0  0  0  0
   -9.6378    1.7452    1.9174 C   0  0  0  0  0  0  0  0  0  0  0  0
   -9.3855    2.7541    1.5048 H   0  0  0  0  0  0  0  0  0  0  0  0
   -9.3937    1.7531    3.0094 H   0  0  0  0  0  0  0  0  0  0  0  0
  -11.1367    1.4853    1.7360 C   0  0  0  0  0  0  0  0  0  0  0  0
  -11.4114    0.4934    2.1610 H   0  0  0  0  0  0  0  0  0  0  0  0
  -11.7218    2.2757    2.2590 H   0  0  0  0  0  0  0  0  0  0  0  0
  -11.4026    1.4968    0.6546 H   0  0  0  0  0  0  0  0  0  0  0  0
    0.9428  -10.1149    2.3910 C   0  0  0  0  0  0  0  0  0  0  0  0
    0.6869   -9.9130    3.4614 H   0  0  0  0  0  0  0  0  0  0  0  0
    2.0098   -9.8168    2.2343 H   0  0  0  0  0  0  0  0  0  0  0  0
    0.7907  -11.6128    2.1087 C   0  0  0  0  0  0  0  0  0  0  0  0
    1.4652  -12.1925    2.7794 H   0  0  0  0  0  0  0  0  0  0  0  0
   -0.2597  -11.9354    2.2884 H   0  0  0  0  0  0  0  0  0  0  0  0
    1.0569  -11.8352    1.0506 H   0  0  0  0  0  0  0  0  0  0  0  0
   -2.3209   -2.0035   -0.2770 Rh  0  0  0  0  0  0  0  0  0  0  0  0
  1  2  1  0  0  0  0
  1  3  1  0  0  0  0
```

```
  1  9  1  0  0  0  0
  1 10  1  0  0  0  0
  2 11  1  0  0  0  0
  2 16  1  0  0  0  0
  2 19  1  0  0  0  0
  3  5  2  0  0  0  0
  3  7  1  0  0  0  0
  3 65  1  0  0  0  0
  4  5  1  0  0  0  0
  4  6  1  0  0  0  0
  4 13  1  0  0  0  0
  4 14  1  0  0  0  0
  5  8  1  0  0  0  0
  5 65  1  0  0  0  0
  6 12  1  0  0  0  0
  6 15  1  0  0  0  0
  6 20  1  0  0  0  0
 15 16  2  0  0  0  0
 15 17  1  0  0  0  0
 15 65  1  0  0  0  0
 16 18  1  0  0  0  0
 16 65  1  0  0  0  0
 21 22  3  0  0  0  0
 21 65  1  0  0  0  0
 22 28  1  0  0  0  0
 23 24  3  0  0  0  0
 23 65  1  0  0  0  0
 24 25  1  0  0  0  0
 25 26  1  0  0  0  0
 25 27  1  0  0  0  0
 25 41  1  0  0  0  0
 28 29  1  0  0  0  0
 28 30  1  0  0  0  0
 28 31  1  0  0  0  0
 31 32  2  0  0  0  0
 31 33  1  0  0  0  0
 32 34  1  0  0  0  0
 32 35  1  0  0  0  0
 33 36  2  0  0  0  0
 33 37  1  0  0  0  0
 34 38  2  0  0  0  0
 34 39  1  0  0  0  0
 36 38  1  0  0  0  0
 36 40  1  0  0  0  0
 38 51  1  0  0  0  0
 41 42  2  0  0  0  0
 41 43  1  0  0  0  0
 42 44  1  0  0  0  0
 42 45  1  0  0  0  0
 43 46  2  0  0  0  0
 43 47  1  0  0  0  0
 44 48  2  0  0  0  0
 44 49  1  0  0  0  0
 46 48  1  0  0  0  0
 46 50  1  0  0  0  0
 48 58  1  0  0  0  0
 51 52  1  0  0  0  0
 51 53  1  0  0  0  0
 51 54  1  0  0  0  0
 54 55  1  0  0  0  0
 54 56  1  0  0  0  0
 54 57  1  0  0  0  0
 58 59  1  0  0  0  0
 58 60  1  0  0  0  0
 58 61  1  0  0  0  0
```

```
 61 62  1  0  0  0  0
 61 63  1  0  0  0  0
 61 64  1  0  0  0  0
M  END
$$$$
mol160
Optimized Catalyst 160
Optimized Catalyst 160
 65 70  0  0  0  0  0  0  0  0999 V2000
   -0.8998    0.2227   -1.5547 C   0  0  0  0  0  0  0  0  0  0  0  0
    0.3389   -0.6064   -1.1551 C   0  0  0  0  0  0  0  0  0  0  0  0
   -2.1619   -0.0425   -0.7649 C   0  0  0  0  0  0  0  0  0  0  0  0
   -1.0818    0.0226    1.5479 C   0  0  0  0  0  0  0  0  0  0  0  0
   -2.2484   -0.1554    0.6035 C   0  0  0  0  0  0  0  0  0  0  0  0
   -0.2643   -1.2583    1.8193 C   0  0  0  0  0  0  0  0  0  0  0  0
   -3.0915    0.1105   -1.3601 H   0  0  0  0  0  0  0  0  0  0  0  0
   -3.2443   -0.1413    1.0985 H   0  0  0  0  0  0  0  0  0  0  0  0
   -1.1082    0.0487   -2.6463 H   0  0  0  0  0  0  0  0  0  0  0  0
   -0.6549    1.3181   -1.4358 H   0  0  0  0  0  0  0  0  0  0  0  0
    0.9995   -0.7064   -2.0608 H   0  0  0  0  0  0  0  0  0  0  0  0
   -0.6737   -1.7548    2.7418 H   0  0  0  0  0  0  0  0  0  0  0  0
   -1.4708    0.4022    2.5334 H   0  0  0  0  0  0  0  0  0  0  0  0
   -0.4119    0.8277    1.1397 H   0  0  0  0  0  0  0  0  0  0  0  0
   -0.2175   -2.2695    0.6961 C   0  0  0  0  0  0  0  0  0  0  0  0
    0.0280   -1.9878   -0.6265 C   0  0  0  0  0  0  0  0  0  0  0  0
   -0.1550   -3.3234    1.0532 H   0  0  0  0  0  0  0  0  0  0  0  0
    0.2416   -2.8121   -1.3401 H   0  0  0  0  0  0  0  0  0  0  0  0
    0.9396   -0.0532   -0.3828 H   0  0  0  0  0  0  0  0  0  0  0  0
    0.8008   -0.9633    2.0489 H   0  0  0  0  0  0  0  0  0  0  0  0
   -4.2995   -2.3499   -0.5916 N   0  0  0  0  0  0  0  0  0  0  0  0
   -5.4556   -2.4098   -0.7327 C   0  0  0  0  0  0  0  0  0  0  0  0
   -2.2957   -4.3094   -0.6088 N   0  0  0  0  0  0  0  0  0  0  0  0
   -2.3769   -5.4550   -0.8116 C   0  0  0  0  0  0  0  0  0  0  0  0
   -2.4677   -6.8786   -1.0737 C   0  0  0  0  0  0  0  0  0  0  0  0
   -3.5393   -7.2140   -0.9413 H   0  0  0  0  0  0  0  0  0  0  0  0
   -2.1851   -7.0761   -2.1513 H   0  0  0  0  0  0  0  0  0  0  0  0
   -6.8944   -2.4629   -0.9062 C   0  0  0  0  0  0  0  0  0  0  0  0
   -7.1389   -2.3947   -2.0082 H   0  0  0  0  0  0  0  0  0  0  0  0
   -7.2807   -3.4593   -0.5371 H   0  0  0  0  0  0  0  0  0  0  0  0
   -7.5813   -1.3442   -0.1557 C   0  0  0  0  0  0  0  0  0  0  0  0
   -7.9304   -1.5153    1.1919 C   0  0  0  0  0  0  0  0  0  0  0  0
   -7.9008   -0.1490   -0.8151 C   0  0  0  0  0  0  0  0  0  0  0  0
   -8.5974   -0.4972    1.8727 C   0  0  0  0  0  0  0  0  0  0  0  0
   -7.6944   -2.4428    1.7123 H   0  0  0  0  0  0  0  0  0  0  0  0
   -8.5691    0.8662   -0.1302 C   0  0  0  0  0  0  0  0  0  0  0  0
   -7.6428   -0.0066   -1.8640 H   0  0  0  0  0  0  0  0  0  0  0  0
   -8.9253    0.7033    1.2192 C   0  0  0  0  0  0  0  0  0  0  0  0
   -8.8790   -0.6393    2.9175 H   0  0  0  0  0  0  0  0  0  0  0  0
   -8.8289    1.7892   -0.6517 H   0  0  0  0  0  0  0  0  0  0  0  0
   -1.5693   -7.6765   -0.1561 C   0  0  0  0  0  0  0  0  0  0  0  0
   -2.0574   -8.1406    1.0739 C   0  0  0  0  0  0  0  0  0  0  0  0
   -0.2605   -7.9871   -0.5520 C   0  0  0  0  0  0  0  0  0  0  0  0
   -1.2409   -8.9128    1.9002 C   0  0  0  0  0  0  0  0  0  0  0  0
   -3.0756   -7.9139    1.3879 H   0  0  0  0  0  0  0  0  0  0  0  0
    0.5524   -8.7598    0.2774 C   0  0  0  0  0  0  0  0  0  0  0  0
    0.1264   -7.6415   -1.5101 H   0  0  0  0  0  0  0  0  0  0  0  0
    0.0709   -9.2322    1.5103 C   0  0  0  0  0  0  0  0  0  0  0  0
   -1.6294   -9.2833    2.8507 H   0  0  0  0  0  0  0  0  0  0  0  0
    1.5657   -9.0115   -0.0412 H   0  0  0  0  0  0  0  0  0  0  0  0
   -9.6641    1.7914    1.9496 C   0  0  0  0  0  0  0  0  0  0  0  0
   -9.3802    2.7918    1.5362 H   0  0  0  0  0  0  0  0  0  0  0  0
   -9.3860    1.7819    3.0335 H   0  0  0  0  0  0  0  0  0  0  0  0
  -11.1780    1.5997    1.8136 C   0  0  0  0  0  0  0  0  0  0  0  0
  -11.4851    0.6205    2.2456 H   0  0  0  0  0  0  0  0  0  0  0  0
  -11.7112    2.4139    2.3562 H   0  0  0  0  0  0  0  0  0  0  0  0
```

```
 -11.4774    1.6285    0.7414 H   0  0  0  0  0  0  0  0  0  0  0  0
   0.9398  -10.0905    2.3887 C   0  0  0  0  0  0  0  0  0  0  0  0
   0.6889   -9.9158    3.4653 H   0  0  0  0  0  0  0  0  0  0  0  0
   2.0166   -9.8224    2.2437 H   0  0  0  0  0  0  0  0  0  0  0  0
   0.7370  -11.5718    2.0540 C   0  0  0  0  0  0  0  0  0  0  0  0
   1.3907  -12.1981    2.7032 H   0  0  0  0  0  0  0  0  0  0  0  0
  -0.3238  -11.8653    2.2230 H   0  0  0  0  0  0  0  0  0  0  0  0
   0.9966  -11.7666    0.9887 H   0  0  0  0  0  0  0  0  0  0  0  0
  -2.3455   -2.3568   -0.3500 Ir  0  0  0  0  0  0  0  0  0  0  0  0
  1  2  1  0  0  0  0
  1  3  1  0  0  0  0
  1  9  1  0  0  0  0
  1 10  1  0  0  0  0
  2 11  1  0  0  0  0
  2 16  1  0  0  0  0
  2 19  1  0  0  0  0
  3  5  2  0  0  0  0
  3  7  1  0  0  0  0
  3 65  1  0  0  0  0
  4  5  1  0  0  0  0
  4  6  1  0  0  0  0
  4 13  1  0  0  0  0
  4 14  1  0  0  0  0
  5  8  1  0  0  0  0
  5 65  1  0  0  0  0
  6 12  1  0  0  0  0
  6 15  1  0  0  0  0
  6 20  1  0  0  0  0
 15 16  2  0  0  0  0
 15 17  1  0  0  0  0
 15 65  1  0  0  0  0
 16 18  1  0  0  0  0
 16 65  1  0  0  0  0
 21 22  3  0  0  0  0
 21 65  1  0  0  0  0
 22 28  1  0  0  0  0
 23 24  3  0  0  0  0
 23 65  1  0  0  0  0
 24 25  1  0  0  0  0
 25 26  1  0  0  0  0
 25 27  1  0  0  0  0
 25 41  1  0  0  0  0
 28 29  1  0  0  0  0
 28 30  1  0  0  0  0
 28 31  1  0  0  0  0
 31 32  2  0  0  0  0
 31 33  1  0  0  0  0
 32 34  1  0  0  0  0
 32 35  1  0  0  0  0
 33 36  2  0  0  0  0
 33 37  1  0  0  0  0
 34 38  2  0  0  0  0
 34 39  1  0  0  0  0
 36 38  1  0  0  0  0
 36 40  1  0  0  0  0
 38 51  1  0  0  0  0
 41 42  2  0  0  0  0
 41 43  1  0  0  0  0
 42 44  1  0  0  0  0
 42 45  1  0  0  0  0
 43 46  2  0  0  0  0
 43 47  1  0  0  0  0
 44 48  2  0  0  0  0
 44 49  1  0  0  0  0
 46 48  1  0  0  0  0
```

```
 46 50  1  0  0  0  0
 48 58  1  0  0  0  0
 51 52  1  0  0  0  0
 51 53  1  0  0  0  0
 51 54  1  0  0  0  0
 54 55  1  0  0  0  0
 54 56  1  0  0  0  0
 54 57  1  0  0  0  0
 58 59  1  0  0  0  0
 58 60  1  0  0  0  0
 58 61  1  0  0  0  0
 61 62  1  0  0  0  0
 61 63  1  0  0  0  0
 61 64  1  0  0  0  0
M  END
$$$$
mol161
Optimized Catalyst 161
Optimized Catalyst 161
 65 70  0  0  0  0  0  0  0  0999 V2000
   -0.9950    0.3311   -1.5691 C   0  0  0  0  0  0  0  0  0  0  0  0
    0.1708   -0.6034   -1.1849 C   0  0  0  0  0  0  0  0  0  0  0  0
   -2.2492    0.1130   -0.7558 C   0  0  0  0  0  0  0  0  0  0  0  0
   -1.1243    0.1480    1.5477 C   0  0  0  0  0  0  0  0  0  0  0  0
   -2.3148    0.0084    0.6277 C   0  0  0  0  0  0  0  0  0  0  0  0
   -0.4479   -1.2094    1.8349 C   0  0  0  0  0  0  0  0  0  0  0  0
   -3.1839    0.2420   -1.3344 H   0  0  0  0  0  0  0  0  0  0  0  0
   -3.2977    0.0107    1.1337 H   0  0  0  0  0  0  0  0  0  0  0  0
   -1.2406    0.1619   -2.6510 H   0  0  0  0  0  0  0  0  0  0  0  0
   -0.6751    1.4008   -1.4481 H   0  0  0  0  0  0  0  0  0  0  0  0
    0.8027   -0.7832   -2.0943 H   0  0  0  0  0  0  0  0  0  0  0  0
   -0.9470   -1.6655    2.7321 H   0  0  0  0  0  0  0  0  0  0  0  0
   -1.4631    0.5921    2.5206 H   0  0  0  0  0  0  0  0  0  0  0  0
   -0.3887    0.8635    1.0981 H   0  0  0  0  0  0  0  0  0  0  0  0
   -0.5360   -2.2032    0.6971 C   0  0  0  0  0  0  0  0  0  0  0  0
   -0.3078   -1.9342   -0.6510 C   0  0  0  0  0  0  0  0  0  0  0  0
   -0.6397   -3.2637    1.0263 H   0  0  0  0  0  0  0  0  0  0  0  0
   -0.2844   -2.7731   -1.3820 H   0  0  0  0  0  0  0  0  0  0  0  0
    0.8262   -0.1131   -0.4201 H   0  0  0  0  0  0  0  0  0  0  0  0
    0.6347   -1.0450    2.0799 H   0  0  0  0  0  0  0  0  0  0  0  0
   -4.0027   -2.0374   -0.4628 N   0  0  0  0  0  0  0  0  0  0  0  0
   -5.1396   -2.2186   -0.6201 C   0  0  0  0  0  0  0  0  0  0  0  0
   -2.3781   -4.4607   -0.6680 N   0  0  0  0  0  0  0  0  0  0  0  0
   -2.4610   -5.5936   -0.8978 C   0  0  0  0  0  0  0  0  0  0  0  0
   -2.3391   -2.0001   -0.2425 Rh  0  0  0  0  0  0  0  0  0  0  0  0
   -6.5662   -2.4035   -0.8131 C   0  0  0  0  0  0  0  0  0  0  0  0
   -6.7974   -2.3683   -1.9156 H   0  0  0  0  0  0  0  0  0  0  0  0
   -6.8632   -3.4192   -0.4254 H   0  0  0  0  0  0  0  0  0  0  0  0
   -2.5618   -7.0135   -1.1830 C   0  0  0  0  0  0  0  0  0  0  0  0
   -3.6337   -7.3439   -1.0770 H   0  0  0  0  0  0  0  0  0  0  0  0
   -1.9486   -7.5925   -0.4352 H   0  0  0  0  0  0  0  0  0  0  0  0
   -7.3917   -1.3220   -0.0845 C   0  0  0  0  0  0  0  0  0  0  0  0
   -7.1479   -1.3312    1.0066 H   0  0  0  0  0  0  0  0  0  0  0  0
   -7.1356   -0.3120   -0.4891 H   0  0  0  0  0  0  0  0  0  0  0  0
   -2.0681   -7.3452   -2.6072 C   0  0  0  0  0  0  0  0  0  0  0  0
   -0.9956   -7.0462   -2.7126 H   0  0  0  0  0  0  0  0  0  0  0  0
   -2.6645   -6.7681   -3.3566 H   0  0  0  0  0  0  0  0  0  0  0  0
   -2.2112   -8.8213   -2.8746 C   0  0  0  0  0  0  0  0  0  0  0  0
   -3.3932   -9.3273   -3.4350 C   0  0  0  0  0  0  0  0  0  0  0  0
   -1.1604   -9.7006   -2.5752 C   0  0  0  0  0  0  0  0  0  0  0  0
   -3.5192  -10.6912   -3.7004 C   0  0  0  0  0  0  0  0  0  0  0  0
   -4.2184   -8.6582   -3.6729 H   0  0  0  0  0  0  0  0  0  0  0  0
   -1.2861  -11.0647   -2.8405 C   0  0  0  0  0  0  0  0  0  0  0  0
   -0.2364   -9.3238   -2.1391 H   0  0  0  0  0  0  0  0  0  0  0  0
   -2.4660  -11.5744   -3.4083 C   0  0  0  0  0  0  0  0  0  0  0  0
```

```
   -4.4390  -11.0715   -4.1443 H   0  0  0  0  0  0  0  0  0  0  0  0
   -0.4591  -11.7368   -2.6115 H   0  0  0  0  0  0  0  0  0  0  0  0
   -8.8617   -1.5904   -0.2790 C   0  0  0  0  0  0  0  0  0  0  0  0
   -9.5617   -2.3889    0.6372 C   0  0  0  0  0  0  0  0  0  0  0  0
   -9.5425   -1.0419   -1.3755 C   0  0  0  0  0  0  0  0  0  0  0  0
  -10.9264   -2.6236    0.4675 C   0  0  0  0  0  0  0  0  0  0  0  0
   -9.0461   -2.8267    1.4905 H   0  0  0  0  0  0  0  0  0  0  0  0
  -10.9074   -1.2766   -1.5455 C   0  0  0  0  0  0  0  0  0  0  0  0
   -9.0123   -0.4247   -2.0990 H   0  0  0  0  0  0  0  0  0  0  0  0
  -11.6151   -2.0662   -0.6233 C   0  0  0  0  0  0  0  0  0  0  0  0
  -11.4621   -3.2376    1.1914 H   0  0  0  0  0  0  0  0  0  0  0  0
  -11.4279   -0.8373   -2.3965 H   0  0  0  0  0  0  0  0  0  0  0  0
  -13.0836   -2.2895   -0.7851 C   0  0  0  0  0  0  0  0  0  0  0  0
  -13.3892   -3.2812   -0.3765 H   0  0  0  0  0  0  0  0  0  0  0  0
  -13.6367   -1.4911   -0.2300 H   0  0  0  0  0  0  0  0  0  0  0  0
  -13.3848   -2.2476   -1.8579 H   0  0  0  0  0  0  0  0  0  0  0  0
   -2.5903  -13.0305   -3.7206 C   0  0  0  0  0  0  0  0  0  0  0  0
   -3.6477  -13.3734   -3.6329 H   0  0  0  0  0  0  0  0  0  0  0  0
   -2.2442  -13.2089   -4.7691 H   0  0  0  0  0  0  0  0  0  0  0  0
   -1.9670  -13.6477   -3.0321 H   0  0  0  0  0  0  0  0  0  0  0  0
  1  2  1  0  0  0  0
  1  3  1  0  0  0  0
  1  9  1  0  0  0  0
  1 10  1  0  0  0  0
  2 11  1  0  0  0  0
  2 16  1  0  0  0  0
  2 19  1  0  0  0  0
  3  5  2  0  0  0  0
  3  7  1  0  0  0  0
  3 25  1  0  0  0  0
  4  5  1  0  0  0  0
  4  6  1  0  0  0  0
  4 13  1  0  0  0  0
  4 14  1  0  0  0  0
  5  8  1  0  0  0  0
  5 25  1  0  0  0  0
  6 12  1  0  0  0  0
  6 15  1  0  0  0  0
  6 20  1  0  0  0  0
 15 16  2  0  0  0  0
 15 17  1  0  0  0  0
 15 25  1  0  0  0  0
 16 18  1  0  0  0  0
 16 25  1  0  0  0  0
 21 22  3  0  0  0  0
 21 25  1  0  0  0  0
 22 26  1  0  0  0  0
 23 24  3  0  0  0  0
 23 25  1  0  0  0  0
 24 29  1  0  0  0  0
 26 27  1  0  0  0  0
 26 28  1  0  0  0  0
 26 32  1  0  0  0  0
 29 30  1  0  0  0  0
 29 31  1  0  0  0  0
 29 35  1  0  0  0  0
 32 33  1  0  0  0  0
 32 34  1  0  0  0  0
 32 48  1  0  0  0  0
 35 36  1  0  0  0  0
 35 37  1  0  0  0  0
 35 38  1  0  0  0  0
 38 39  2  0  0  0  0
 38 40  1  0  0  0  0
 39 41  1  0  0  0  0
```

```
 39 42  1  0  0  0  0
 40 43  2  0  0  0  0
 40 44  1  0  0  0  0
 41 45  2  0  0  0  0
 41 46  1  0  0  0  0
 43 45  1  0  0  0  0
 43 47  1  0  0  0  0
 45 62  1  0  0  0  0
 48 49  2  0  0  0  0
 48 50  1  0  0  0  0
 49 51  1  0  0  0  0
 49 52  1  0  0  0  0
 50 53  2  0  0  0  0
 50 54  1  0  0  0  0
 51 55  2  0  0  0  0
 51 56  1  0  0  0  0
 53 55  1  0  0  0  0
 53 57  1  0  0  0  0
 55 58  1  0  0  0  0
 58 59  1  0  0  0  0
 58 60  1  0  0  0  0
 58 61  1  0  0  0  0
 62 63  1  0  0  0  0
 62 64  1  0  0  0  0
 62 65  1  0  0  0  0
M  END
$$$$
mol162
Optimized Catalyst 162
Optimized Catalyst 162
 65 70  0  0  0  0  0  0  0  0999 V2000
   -0.8478    0.2180   -1.5434 C   0  0  0  0  0  0  0  0  0  0  0  0
    0.3805   -0.6403   -1.1741 C   0  0  0  0  0  0  0  0  0  0  0  0
   -2.1094   -0.0490   -0.7528 C   0  0  0  0  0  0  0  0  0  0  0  0
   -1.0087   -0.0459    1.5510 C   0  0  0  0  0  0  0  0  0  0  0  0
   -2.1864   -0.1933    0.6142 C   0  0  0  0  0  0  0  0  0  0  0  0
   -0.2090   -1.3429    1.7950 C   0  0  0  0  0  0  0  0  0  0  0  0
   -3.0403    0.1294   -1.3389 H   0  0  0  0  0  0  0  0  0  0  0  0
   -3.1765   -0.1826    1.1200 H   0  0  0  0  0  0  0  0  0  0  0  0
   -1.0642    0.0797   -2.6387 H   0  0  0  0  0  0  0  0  0  0  0  0
   -0.5835    1.3056   -1.3965 H   0  0  0  0  0  0  0  0  0  0  0  0
    1.0292   -0.7324   -2.0895 H   0  0  0  0  0  0  0  0  0  0  0  0
   -0.6190   -1.8498    2.7116 H   0  0  0  0  0  0  0  0  0  0  0  0
   -1.3819    0.3263    2.5457 H   0  0  0  0  0  0  0  0  0  0  0  0
   -0.3291    0.7539    1.1483 H   0  0  0  0  0  0  0  0  0  0  0  0
   -0.1840   -2.3313    0.6513 C   0  0  0  0  0  0  0  0  0  0  0  0
    0.0545   -2.0265   -0.6676 C   0  0  0  0  0  0  0  0  0  0  0  0
   -0.1314   -3.3914    0.9883 H   0  0  0  0  0  0  0  0  0  0  0  0
    0.2523   -2.8392   -1.3987 H   0  0  0  0  0  0  0  0  0  0  0  0
    0.9994   -0.1114   -0.3989 H   0  0  0  0  0  0  0  0  0  0  0  0
    0.8620   -1.0686    2.0244 H   0  0  0  0  0  0  0  0  0  0  0  0
   -4.2791   -2.3352   -0.6236 N   0  0  0  0  0  0  0  0  0  0  0  0
   -5.4354   -2.4076   -0.7613 C   0  0  0  0  0  0  0  0  0  0  0  0
   -2.2797   -4.3015   -0.6919 N   0  0  0  0  0  0  0  0  0  0  0  0
   -2.3271   -5.4484   -0.8999 C   0  0  0  0  0  0  0  0  0  0  0  0
   -2.3237   -2.3599   -0.3830 Ir  0  0  0  0  0  0  0  0  0  0  0  0
   -6.8694   -2.4926   -0.9336 C   0  0  0  0  0  0  0  0  0  0  0  0
   -7.1236   -2.4068   -2.0314 H   0  0  0  0  0  0  0  0  0  0  0  0
   -7.2303   -3.5027   -0.5765 H   0  0  0  0  0  0  0  0  0  0  0  0
   -2.3672   -6.8705   -1.1600 C   0  0  0  0  0  0  0  0  0  0  0  0
   -3.4119   -7.2607   -0.9754 H   0  0  0  0  0  0  0  0  0  0  0  0
   -1.6735   -7.4079   -0.4480 H   0  0  0  0  0  0  0  0  0  0  0  0
   -7.6265   -1.3915   -0.1557 C   0  0  0  0  0  0  0  0  0  0  0  0
   -7.3515   -1.4312    0.9271 H   0  0  0  0  0  0  0  0  0  0  0  0
   -7.3473   -0.3843   -0.5525 H   0  0  0  0  0  0  0  0  0  0  0  0
```

```
   -1.9565   -7.2002   -2.6138 C   0  0  0  0  0  0  0  0  0  0  0  0
   -0.8882   -6.9152   -2.7812 H   0  0  0  0  0  0  0  0  0  0  0  0
   -2.5896   -6.6206   -3.3298 H   0  0  0  0  0  0  0  0  0  0  0  0
   -2.1328   -8.6764   -2.8586 C   0  0  0  0  0  0  0  0  0  0  0  0
   -3.3321   -9.1652   -3.3965 C   0  0  0  0  0  0  0  0  0  0  0  0
   -1.0988   -9.5726   -2.5514 C   0  0  0  0  0  0  0  0  0  0  0  0
   -3.4936  -10.5313   -3.6277 C   0  0  0  0  0  0  0  0  0  0  0  0
   -4.1425   -8.4824   -3.6452 H   0  0  0  0  0  0  0  0  0  0  0  0
   -1.2610  -10.9387   -2.7813 C   0  0  0  0  0  0  0  0  0  0  0  0
   -0.1595   -9.2080   -2.1388 H   0  0  0  0  0  0  0  0  0  0  0  0
   -2.4595  -11.4334   -3.3239 C   0  0  0  0  0  0  0  0  0  0  0  0
   -4.4263  -10.9007   -4.0544 H   0  0  0  0  0  0  0  0  0  0  0  0
   -0.4484  -11.6263   -2.5449 H   0  0  0  0  0  0  0  0  0  0  0  0
   -9.1104   -1.6069   -0.3114 C   0  0  0  0  0  0  0  0  0  0  0  0
   -9.8138   -2.3833    0.6212 C   0  0  0  0  0  0  0  0  0  0  0  0
   -9.7988   -1.0354   -1.3914 C   0  0  0  0  0  0  0  0  0  0  0  0
  -11.1854   -2.5890    0.4731 C   0  0  0  0  0  0  0  0  0  0  0  0
   -9.2966   -2.8260    1.4710 H   0  0  0  0  0  0  0  0  0  0  0  0
  -11.1705   -1.2421   -1.5396 C   0  0  0  0  0  0  0  0  0  0  0  0
   -9.2693   -0.4217   -2.1186 H   0  0  0  0  0  0  0  0  0  0  0  0
  -11.8793   -2.0221   -0.6098 C   0  0  0  0  0  0  0  0  0  0  0  0
  -11.7236   -3.1901    1.2062 H   0  0  0  0  0  0  0  0  0  0  0  0
  -11.6968   -0.7904   -2.3808 H   0  0  0  0  0  0  0  0  0  0  0  0
  -13.3506   -2.2313   -0.7590 C   0  0  0  0  0  0  0  0  0  0  0  0
  -13.6609   -3.2227   -0.3523 H   0  0  0  0  0  0  0  0  0  0  0  0
  -13.8948   -1.4316   -0.1963 H   0  0  0  0  0  0  0  0  0  0  0  0
  -13.6601   -2.1828   -1.8296 H   0  0  0  0  0  0  0  0  0  0  0  0
   -2.6234  -12.8923   -3.5974 C   0  0  0  0  0  0  0  0  0  0  0  0
   -3.6889  -13.2061   -3.4930 H   0  0  0  0  0  0  0  0  0  0  0  0
   -2.2914  -13.1066   -4.6444 H   0  0  0  0  0  0  0  0  0  0  0  0
   -2.0087  -13.5080   -2.8991 H   0  0  0  0  0  0  0  0  0  0  0  0
  1  2  1  0  0  0  0
  1  3  1  0  0  0  0
  1  9  1  0  0  0  0
  1 10  1  0  0  0  0
  2 11  1  0  0  0  0
  2 16  1  0  0  0  0
  2 19  1  0  0  0  0
  3  5  2  0  0  0  0
  3  7  1  0  0  0  0
  3 25  1  0  0  0  0
  4  5  1  0  0  0  0
  4  6  1  0  0  0  0
  4 13  1  0  0  0  0
  4 14  1  0  0  0  0
  5  8  1  0  0  0  0
  5 25  1  0  0  0  0
  6 12  1  0  0  0  0
  6 15  1  0  0  0  0
  6 20  1  0  0  0  0
 15 16  2  0  0  0  0
 15 17  1  0  0  0  0
 15 25  1  0  0  0  0
 16 18  1  0  0  0  0
 16 25  1  0  0  0  0
 21 22  3  0  0  0  0
 21 25  1  0  0  0  0
 22 26  1  0  0  0  0
 23 24  3  0  0  0  0
 23 25  1  0  0  0  0
 24 29  1  0  0  0  0
 26 27  1  0  0  0  0
 26 28  1  0  0  0  0
 26 32  1  0  0  0  0
 29 30  1  0  0  0  0
```

```
 29 31  1  0  0  0  0
 29 35  1  0  0  0  0
 32 33  1  0  0  0  0
 32 34  1  0  0  0  0
 32 48  1  0  0  0  0
 35 36  1  0  0  0  0
 35 37  1  0  0  0  0
 35 38  1  0  0  0  0
 38 39  2  0  0  0  0
 38 40  1  0  0  0  0
 39 41  1  0  0  0  0
 39 42  1  0  0  0  0
 40 43  2  0  0  0  0
 40 44  1  0  0  0  0
 41 45  2  0  0  0  0
 41 46  1  0  0  0  0
 43 45  1  0  0  0  0
 43 47  1  0  0  0  0
 45 62  1  0  0  0  0
 48 49  2  0  0  0  0
 48 50  1  0  0  0  0
 49 51  1  0  0  0  0
 49 52  1  0  0  0  0
 50 53  2  0  0  0  0
 50 54  1  0  0  0  0
 51 55  2  0  0  0  0
 51 56  1  0  0  0  0
 53 55  1  0  0  0  0
 53 57  1  0  0  0  0
 55 58  1  0  0  0  0
 58 59  1  0  0  0  0
 58 60  1  0  0  0  0
 58 61  1  0  0  0  0
 62 63  1  0  0  0  0
 62 64  1  0  0  0  0
 62 65  1  0  0  0  0
M  END
$$$$
mol163
Optimized Catalyst 163
Optimized Catalyst 163
 65 70  0  0  0  0  0  0  0  0999 V2000
   -0.9844    0.3382   -1.5688 C   0  0  0  0  0  0  0  0  0  0  0  0
    0.1833   -0.5923   -1.1811 C   0  0  0  0  0  0  0  0  0  0  0  0
   -2.2390    0.1186   -0.7564 C   0  0  0  0  0  0  0  0  0  0  0  0
   -1.1141    0.1540    1.5474 C   0  0  0  0  0  0  0  0  0  0  0  0
   -2.3049    0.0131    0.6277 C   0  0  0  0  0  0  0  0  0  0  0  0
   -0.4365   -1.2023    1.8363 C   0  0  0  0  0  0  0  0  0  0  0  0
   -3.1733    0.2493   -1.3357 H   0  0  0  0  0  0  0  0  0  0  0  0
   -3.2877    0.0162    1.1342 H   0  0  0  0  0  0  0  0  0  0  0  0
   -1.2288    0.1663   -2.6507 H   0  0  0  0  0  0  0  0  0  0  0  0
   -0.6663    1.4086   -1.4495 H   0  0  0  0  0  0  0  0  0  0  0  0
    0.8197   -0.7680   -2.0879 H   0  0  0  0  0  0  0  0  0  0  0  0
   -0.9369   -1.6601    2.7320 H   0  0  0  0  0  0  0  0  0  0  0  0
   -1.4528    0.5986    2.5201 H   0  0  0  0  0  0  0  0  0  0  0  0
   -0.3797    0.8695    1.0959 H   0  0  0  0  0  0  0  0  0  0  0  0
   -0.5223   -2.1952    0.6977 C   0  0  0  0  0  0  0  0  0  0  0  0
   -0.2936   -1.9247   -0.6499 C   0  0  0  0  0  0  0  0  0  0  0  0
   -0.6275   -3.2556    1.0262 H   0  0  0  0  0  0  0  0  0  0  0  0
   -0.2681   -2.7625   -1.3817 H   0  0  0  0  0  0  0  0  0  0  0  0
    0.8336   -0.1013   -0.4123 H   0  0  0  0  0  0  0  0  0  0  0  0
    0.6452   -1.0366    2.0845 H   0  0  0  0  0  0  0  0  0  0  0  0
   -3.9887   -2.0260   -0.4588 N   0  0  0  0  0  0  0  0  0  0  0  0
   -5.1253   -2.2073   -0.6172 C   0  0  0  0  0  0  0  0  0  0  0  0
   -2.3617   -4.4580   -0.6580 N   0  0  0  0  0  0  0  0  0  0  0  0
```

```
   -2.4519   -5.5905   -0.8861 C   0  0  0  0  0  0  0  0  0  0  0  0
   -2.3255   -1.9908   -0.2401 Rh  0  0  0  0  0  0  0  0  0  0  0  0
   -6.5508   -2.3995   -0.8153 C   0  0  0  0  0  0  0  0  0  0  0  0
   -6.7696   -2.3629   -1.9201 H   0  0  0  0  0  0  0  0  0  0  0  0
   -6.8332   -3.4208   -0.4327 H   0  0  0  0  0  0  0  0  0  0  0  0
   -2.5642   -7.0107   -1.1700 C   0  0  0  0  0  0  0  0  0  0  0  0
   -3.6400   -7.3251   -1.0600 H   0  0  0  0  0  0  0  0  0  0  0  0
   -1.9557   -7.5879   -0.4174 H   0  0  0  0  0  0  0  0  0  0  0  0
   -7.3794   -1.3241   -0.0858 C   0  0  0  0  0  0  0  0  0  0  0  0
   -7.1394   -1.3379    1.0057 H   0  0  0  0  0  0  0  0  0  0  0  0
   -7.1266   -0.3134   -0.4899 H   0  0  0  0  0  0  0  0  0  0  0  0
   -2.0717   -7.3384   -2.5937 C   0  0  0  0  0  0  0  0  0  0  0  0
   -0.9991   -7.0408   -2.6970 H   0  0  0  0  0  0  0  0  0  0  0  0
   -2.6709   -6.7614   -3.3404 H   0  0  0  0  0  0  0  0  0  0  0  0
   -8.8854   -1.5751   -0.2702 C   0  0  0  0  0  0  0  0  0  0  0  0
   -9.1457   -1.5662   -1.3574 H   0  0  0  0  0  0  0  0  0  0  0  0
   -9.1600   -2.5793    0.1373 H   0  0  0  0  0  0  0  0  0  0  0  0
   -2.2054   -8.8396   -2.8982 C   0  0  0  0  0  0  0  0  0  0  0  0
   -1.6074   -9.4352   -2.1648 H   0  0  0  0  0  0  0  0  0  0  0  0
   -3.2727   -9.1578   -2.8037 H   0  0  0  0  0  0  0  0  0  0  0  0
   -1.7157   -9.1225   -4.2968 C   0  0  0  0  0  0  0  0  0  0  0  0
   -2.6045   -9.0641   -5.3805 C   0  0  0  0  0  0  0  0  0  0  0  0
   -0.3671   -9.4358   -4.5217 C   0  0  0  0  0  0  0  0  0  0  0  0
   -2.1475   -9.3240   -6.6754 C   0  0  0  0  0  0  0  0  0  0  0  0
   -3.6524   -8.8216   -5.2189 H   0  0  0  0  0  0  0  0  0  0  0  0
    0.0846   -9.6949   -5.8185 C   0  0  0  0  0  0  0  0  0  0  0  0
    0.3320   -9.4830   -3.6894 H   0  0  0  0  0  0  0  0  0  0  0  0
   -0.8036   -9.6399   -6.8971 C   0  0  0  0  0  0  0  0  0  0  0  0
   -2.8418   -9.2836   -7.5149 H   0  0  0  0  0  0  0  0  0  0  0  0
    1.1322   -9.9439   -5.9894 H   0  0  0  0  0  0  0  0  0  0  0  0
   -0.4503   -9.8463   -7.9067 H   0  0  0  0  0  0  0  0  0  0  0  0
   -9.6744   -0.5079    0.4468 C   0  0  0  0  0  0  0  0  0  0  0  0
  -10.0401   -0.6843    1.7895 C   0  0  0  0  0  0  0  0  0  0  0  0
  -10.0383    0.6692   -0.2234 C   0  0  0  0  0  0  0  0  0  0  0  0
  -10.7676    0.3077    2.4525 C   0  0  0  0  0  0  0  0  0  0  0  0
   -9.7652   -1.5940    2.3188 H   0  0  0  0  0  0  0  0  0  0  0  0
  -10.7660    1.6579    0.4445 C   0  0  0  0  0  0  0  0  0  0  0  0
   -9.7620    0.8162   -1.2652 H   0  0  0  0  0  0  0  0  0  0  0  0
  -11.1310    1.4800    1.7825 C   0  0  0  0  0  0  0  0  0  0  0  0
  -11.0555    0.1655    3.4944 H   0  0  0  0  0  0  0  0  0  0  0  0
  -11.0528    2.5693   -0.0807 H   0  0  0  0  0  0  0  0  0  0  0  0
  -11.7000    2.2512    2.3006 H   0  0  0  0  0  0  0  0  0  0  0  0
  1  2  1  0  0  0  0
  1  3  1  0  0  0  0
  1  9  1  0  0  0  0
  1 10  1  0  0  0  0
  2 11  1  0  0  0  0
  2 16  1  0  0  0  0
  2 19  1  0  0  0  0
  3  5  2  0  0  0  0
  3  7  1  0  0  0  0
  3 25  1  0  0  0  0
  4  5  1  0  0  0  0
  4  6  1  0  0  0  0
  4 13  1  0  0  0  0
  4 14  1  0  0  0  0
  5  8  1  0  0  0  0
  5 25  1  0  0  0  0
  6 12  1  0  0  0  0
  6 15  1  0  0  0  0
  6 20  1  0  0  0  0
 15 16  2  0  0  0  0
 15 17  1  0  0  0  0
 15 25  1  0  0  0  0
 16 18  1  0  0  0  0
```

```
 16 25  1  0  0  0  0
 21 22  3  0  0  0  0
 21 25  1  0  0  0  0
 22 26  1  0  0  0  0
 23 24  3  0  0  0  0
 23 25  1  0  0  0  0
 24 29  1  0  0  0  0
 26 27  1  0  0  0  0
 26 28  1  0  0  0  0
 26 32  1  0  0  0  0
 29 30  1  0  0  0  0
 29 31  1  0  0  0  0
 29 35  1  0  0  0  0
 32 33  1  0  0  0  0
 32 34  1  0  0  0  0
 32 38  1  0  0  0  0
 35 36  1  0  0  0  0
 35 37  1  0  0  0  0
 35 41  1  0  0  0  0
 38 39  1  0  0  0  0
 38 40  1  0  0  0  0
 38 55  1  0  0  0  0
 41 42  1  0  0  0  0
 41 43  1  0  0  0  0
 41 44  1  0  0  0  0
 44 45  2  0  0  0  0
 44 46  1  0  0  0  0
 45 47  1  0  0  0  0
 45 48  1  0  0  0  0
 46 49  2  0  0  0  0
 46 50  1  0  0  0  0
 47 51  2  0  0  0  0
 47 52  1  0  0  0  0
 49 51  1  0  0  0  0
 49 53  1  0  0  0  0
 51 54  1  0  0  0  0
 55 56  2  0  0  0  0
 55 57  1  0  0  0  0
 56 58  1  0  0  0  0
 56 59  1  0  0  0  0
 57 60  2  0  0  0  0
 57 61  1  0  0  0  0
 58 62  2  0  0  0  0
 58 63  1  0  0  0  0
 60 62  1  0  0  0  0
 60 64  1  0  0  0  0
 62 65  1  0  0  0  0
M  END
$$$$
mol164
Optimized Catalyst 164
Optimized Catalyst 164
 65 70  0  0  0  0  0  0  0  0999 V2000
   -0.8739    0.2559   -1.5542 C   0  0  0  0  0  0  0  0  0  0  0  0
    0.3570   -0.5866   -1.1597 C   0  0  0  0  0  0  0  0  0  0  0  0
   -2.1387   -0.0060   -0.7682 C   0  0  0  0  0  0  0  0  0  0  0  0
   -1.0596    0.0260    1.5447 C   0  0  0  0  0  0  0  0  0  0  0  0
   -2.2264   -0.1327    0.5976 C   0  0  0  0  0  0  0  0  0  0  0  0
   -0.2585   -1.2667    1.8067 C   0  0  0  0  0  0  0  0  0  0  0  0
   -3.0658    0.1533   -1.3647 H   0  0  0  0  0  0  0  0  0  0  0  0
   -3.2210   -0.1256    1.0938 H   0  0  0  0  0  0  0  0  0  0  0  0
   -1.0806    0.0963   -2.6485 H   0  0  0  0  0  0  0  0  0  0  0  0
   -0.6191    1.3480   -1.4238 H   0  0  0  0  0  0  0  0  0  0  0  0
    1.0202   -0.6809   -2.0643 H   0  0  0  0  0  0  0  0  0  0  0  0
   -0.6775   -1.7678    2.7226 H   0  0  0  0  0  0  0  0  0  0  0  0
```

```
   -1.4466    0.4023    2.5325 H   0  0  0  0  0  0  0  0  0  0  0  0
   -0.3786    0.8265    1.1455 H   0  0  0  0  0  0  0  0  0  0  0  0
   -0.2159   -2.2659    0.6723 C   0  0  0  0  0  0  0  0  0  0  0  0
    0.0358   -1.9714   -0.6466 C   0  0  0  0  0  0  0  0  0  0  0  0
   -0.1603   -3.3230    1.0196 H   0  0  0  0  0  0  0  0  0  0  0  0
    0.2459   -2.7887   -1.3699 H   0  0  0  0  0  0  0  0  0  0  0  0
    0.9597   -0.0446   -0.3807 H   0  0  0  0  0  0  0  0  0  0  0  0
    0.8090   -0.9866    2.0457 H   0  0  0  0  0  0  0  0  0  0  0  0
   -4.2886   -2.3350   -0.6302 N   0  0  0  0  0  0  0  0  0  0  0  0
   -5.4438   -2.4179   -0.7700 C   0  0  0  0  0  0  0  0  0  0  0  0
   -2.2858   -4.2831   -0.6834 N   0  0  0  0  0  0  0  0  0  0  0  0
   -2.3605   -5.4284   -0.8924 C   0  0  0  0  0  0  0  0  0  0  0  0
   -2.3382   -2.3389   -0.3813 Ir  0  0  0  0  0  0  0  0  0  0  0  0
   -6.8780   -2.5133   -0.9404 C   0  0  0  0  0  0  0  0  0  0  0  0
   -7.1280   -2.4292   -2.0389 H   0  0  0  0  0  0  0  0  0  0  0  0
   -7.2237   -3.5266   -0.5802 H   0  0  0  0  0  0  0  0  0  0  0  0
   -2.4427   -6.8515   -1.1465 C   0  0  0  0  0  0  0  0  0  0  0  0
   -3.5066   -7.1936   -0.9830 H   0  0  0  0  0  0  0  0  0  0  0  0
   -1.7866   -7.4012   -0.4091 H   0  0  0  0  0  0  0  0  0  0  0  0
   -7.6255   -1.4112   -0.1600 C   0  0  0  0  0  0  0  0  0  0  0  0
   -7.3515   -1.4590    0.9227 H   0  0  0  0  0  0  0  0  0  0  0  0
   -7.3408   -0.4059   -0.5577 H   0  0  0  0  0  0  0  0  0  0  0  0
   -2.0039   -7.2028   -2.5846 C   0  0  0  0  0  0  0  0  0  0  0  0
   -0.9288   -6.9304   -2.7275 H   0  0  0  0  0  0  0  0  0  0  0  0
   -2.6188   -6.6294   -3.3210 H   0  0  0  0  0  0  0  0  0  0  0  0
   -9.1480   -1.5847   -0.2938 C   0  0  0  0  0  0  0  0  0  0  0  0
   -9.4481   -1.5560   -1.3705 H   0  0  0  0  0  0  0  0  0  0  0  0
   -9.4634   -2.5740    0.1210 H   0  0  0  0  0  0  0  0  0  0  0  0
   -2.1760   -8.7063   -2.8593 C   0  0  0  0  0  0  0  0  0  0  0  0
   -1.5862   -9.3051   -2.1213 H   0  0  0  0  0  0  0  0  0  0  0  0
   -3.2500   -8.9987   -2.7537 H   0  0  0  0  0  0  0  0  0  0  0  0
   -1.7034   -9.0304   -4.2559 C   0  0  0  0  0  0  0  0  0  0  0  0
   -2.6030   -8.9939   -5.3312 C   0  0  0  0  0  0  0  0  0  0  0  0
   -0.3620   -9.3711   -4.4830 C   0  0  0  0  0  0  0  0  0  0  0  0
   -2.1638   -9.3056   -6.6212 C   0  0  0  0  0  0  0  0  0  0  0  0
   -3.6464   -8.7334   -5.1677 H   0  0  0  0  0  0  0  0  0  0  0  0
    0.0716   -9.6816   -5.7749 C   0  0  0  0  0  0  0  0  0  0  0  0
    0.3450   -9.4051   -3.6566 H   0  0  0  0  0  0  0  0  0  0  0  0
   -0.8275   -9.6504   -6.8455 C   0  0  0  0  0  0  0  0  0  0  0  0
   -2.8671   -9.2857   -7.4545 H   0  0  0  0  0  0  0  0  0  0  0  0
    1.1134   -9.9548   -5.9474 H   0  0  0  0  0  0  0  0  0  0  0  0
   -0.4886   -9.9001   -7.8512 H   0  0  0  0  0  0  0  0  0  0  0  0
   -9.8518   -0.4790    0.4544 C   0  0  0  0  0  0  0  0  0  0  0  0
  -10.1762   -0.6411    1.8091 C   0  0  0  0  0  0  0  0  0  0  0  0
  -10.1776    0.7173   -0.2012 C   0  0  0  0  0  0  0  0  0  0  0  0
  -10.8248    0.3863    2.5001 C   0  0  0  0  0  0  0  0  0  0  0  0
   -9.9339   -1.5671    2.3264 H   0  0  0  0  0  0  0  0  0  0  0  0
  -10.8264    1.7411    0.4950 C   0  0  0  0  0  0  0  0  0  0  0  0
   -9.9359    0.8521   -1.2535 H   0  0  0  0  0  0  0  0  0  0  0  0
  -11.1498    1.5786    1.8455 C   0  0  0  0  0  0  0  0  0  0  0  0
  -11.0820    0.2557    3.5518 H   0  0  0  0  0  0  0  0  0  0  0  0
  -11.0849    2.6679   -0.0186 H   0  0  0  0  0  0  0  0  0  0  0  0
  -11.6587    2.3772    2.3857 H   0  0  0  0  0  0  0  0  0  0  0  0
  1  2  1  0  0  0  0
  1  3  1  0  0  0  0
  1  9  1  0  0  0  0
  1 10  1  0  0  0  0
  2 11  1  0  0  0  0
  2 16  1  0  0  0  0
  2 19  1  0  0  0  0
  3  5  2  0  0  0  0
  3  7  1  0  0  0  0
  3 25  1  0  0  0  0
  4  5  1  0  0  0  0
  4  6  1  0  0  0  0
```

```
  4 13  1  0  0  0  0
  4 14  1  0  0  0  0
  5  8  1  0  0  0  0
  5 25  1  0  0  0  0
  6 12  1  0  0  0  0
  6 15  1  0  0  0  0
  6 20  1  0  0  0  0
 15 16  2  0  0  0  0
 15 17  1  0  0  0  0
 15 25  1  0  0  0  0
 16 18  1  0  0  0  0
 16 25  1  0  0  0  0
 21 22  3  0  0  0  0
 21 25  1  0  0  0  0
 22 26  1  0  0  0  0
 23 24  3  0  0  0  0
 23 25  1  0  0  0  0
 24 29  1  0  0  0  0
 26 27  1  0  0  0  0
 26 28  1  0  0  0  0
 26 32  1  0  0  0  0
 29 30  1  0  0  0  0
 29 31  1  0  0  0  0
 29 35  1  0  0  0  0
 32 33  1  0  0  0  0
 32 34  1  0  0  0  0
 32 38  1  0  0  0  0
 35 36  1  0  0  0  0
 35 37  1  0  0  0  0
 35 41  1  0  0  0  0
 38 39  1  0  0  0  0
 38 40  1  0  0  0  0
 38 55  1  0  0  0  0
 41 42  1  0  0  0  0
 41 43  1  0  0  0  0
 41 44  1  0  0  0  0
 44 45  2  0  0  0  0
 44 46  1  0  0  0  0
 45 47  1  0  0  0  0
 45 48  1  0  0  0  0
 46 49  2  0  0  0  0
 46 50  1  0  0  0  0
 47 51  2  0  0  0  0
 47 52  1  0  0  0  0
 49 51  1  0  0  0  0
 49 53  1  0  0  0  0
 51 54  1  0  0  0  0
 55 56  2  0  0  0  0
 55 57  1  0  0  0  0
 56 58  1  0  0  0  0
 56 59  1  0  0  0  0
 57 60  2  0  0  0  0
 57 61  1  0  0  0  0
 58 62  2  0  0  0  0
 58 63  1  0  0  0  0
 60 62  1  0  0  0  0
 60 64  1  0  0  0  0
 62 65  1  0  0  0  0
M  END
$$$$
mol165
Optimized Catalyst 165
Optimized Catalyst 165
 89 94  0  0  0  0  0  0  0  0999 V2000
   -2.2350    1.3394   -0.8963 C   0  0  0  0  0  0  0  0  0  0  0  0
```

```
   -2.4455    1.7579    0.5353 C   0  0  0  0  0  0  0  0  0  0  0  0
    0.6892    2.8610   -0.8189 C   0  0  0  0  0  0  0  0  0  0  0  0
   -1.7970    3.0875    0.9643 C   0  0  0  0  0  0  0  0  0  0  0  0
    0.7610    3.3684    0.6291 C   0  0  0  0  0  0  0  0  0  0  0  0
   -0.3863    2.9393    1.5588 C   0  0  0  0  0  0  0  0  0  0  0  0
   -2.0558    0.8857    1.2022 H   0  0  0  0  0  0  0  0  0  0  0  0
   -1.7735    3.7784    0.0875 H   0  0  0  0  0  0  0  0  0  0  0  0
   -0.0734    3.4541   -1.3817 H   0  0  0  0  0  0  0  0  0  0  0  0
    0.7900    4.4878    0.5918 H   0  0  0  0  0  0  0  0  0  0  0  0
   -3.1719    1.1809   -1.4762 H   0  0  0  0  0  0  0  0  0  0  0  0
   -3.5503    1.8227    0.7252 H   0  0  0  0  0  0  0  0  0  0  0  0
    1.6837    3.0537   -1.2970 H   0  0  0  0  0  0  0  0  0  0  0  0
   -2.4583    3.5511    1.7404 H   0  0  0  0  0  0  0  0  0  0  0  0
    1.7207    3.0183    1.0864 H   0  0  0  0  0  0  0  0  0  0  0  0
   -0.3226    3.5756    2.4799 H   0  0  0  0  0  0  0  0  0  0  0  0
   -1.0171    1.1268   -1.5494 C   0  0  0  0  0  0  0  0  0  0  0  0
   -1.0363    0.8503   -2.6245 H   0  0  0  0  0  0  0  0  0  0  0  0
    0.3566    1.3635   -0.9703 C   0  0  0  0  0  0  0  0  0  0  0  0
    0.4152    0.8288    0.0444 H   0  0  0  0  0  0  0  0  0  0  0  0
    1.1179    0.8848   -1.6398 H   0  0  0  0  0  0  0  0  0  0  0  0
   -1.8416   -2.3695   -2.6313 C   0  0  0  0  0  0  0  0  0  0  0  0
   -2.7167   -3.4937   -3.1037 C   0  0  0  0  0  0  0  0  0  0  0  0
    0.5042   -4.5861   -1.8721 C   0  0  0  0  0  0  0  0  0  0  0  0
   -2.5976   -4.7622   -2.2311 C   0  0  0  0  0  0  0  0  0  0  0  0
   -0.2631   -5.9017   -2.0933 C   0  0  0  0  0  0  0  0  0  0  0  0
   -1.6317   -5.8206   -2.7895 C   0  0  0  0  0  0  0  0  0  0  0  0
   -2.4535   -3.7313   -4.1662 H   0  0  0  0  0  0  0  0  0  0  0  0
   -2.2707   -4.4666   -1.2076 H   0  0  0  0  0  0  0  0  0  0  0  0
    0.1595   -4.1291   -0.9152 H   0  0  0  0  0  0  0  0  0  0  0  0
   -0.4055   -6.3759   -1.0888 H   0  0  0  0  0  0  0  0  0  0  0  0
   -2.4030   -1.4496   -2.3318 H   0  0  0  0  0  0  0  0  0  0  0  0
   -3.7822   -3.1578   -3.0922 H   0  0  0  0  0  0  0  0  0  0  0  0
    1.5856   -4.8464   -1.7531 H   0  0  0  0  0  0  0  0  0  0  0  0
   -3.6134   -5.2205   -2.1520 H   0  0  0  0  0  0  0  0  0  0  0  0
    0.3782   -6.5825   -2.7086 H   0  0  0  0  0  0  0  0  0  0  0  0
   -2.1145   -6.8256   -2.6816 H   0  0  0  0  0  0  0  0  0  0  0  0
   -0.5004   -2.3858   -2.5670 C   0  0  0  0  0  0  0  0  0  0  0  0
    0.0404   -1.5012   -2.1708 H   0  0  0  0  0  0  0  0  0  0  0  0
    0.3793   -3.5291   -2.9870 C   0  0  0  0  0  0  0  0  0  0  0  0
   -0.0297   -3.9998   -3.9149 H   0  0  0  0  0  0  0  0  0  0  0  0
    1.3958   -3.1353   -3.2357 H   0  0  0  0  0  0  0  0  0  0  0  0
   -1.4744   -5.6499   -3.8811 H   0  0  0  0  0  0  0  0  0  0  0  0
   -0.2235    1.8809    1.8795 H   0  0  0  0  0  0  0  0  0  0  0  0
   -1.2723   -2.9807    0.8408 C   0  0  0  0  0  0  0  0  0  0  0  0
   -1.3545   -1.9371    0.3336 N   0  0  0  0  0  0  0  0  0  0  0  0
   -5.0944   -1.6528   -0.6894 C   0  0  0  0  0  0  0  0  0  0  0  0
   -4.0977   -1.1262   -0.4151 N   0  0  0  0  0  0  0  0  0  0  0  0
   -1.4931   -0.5029   -0.4722 Rh  0  0  0  0  0  0  0  0  0  0  0  0
   -6.3115   -2.3037   -1.0550 C   0  0  0  0  0  0  0  0  0  0  0  0
   -6.6343   -2.4476   -2.4187 C   0  0  0  0  0  0  0  0  0  0  0  0
   -7.1802   -2.8028   -0.0678 C   0  0  0  0  0  0  0  0  0  0  0  0
   -7.8027   -3.1072   -2.7855 C   0  0  0  0  0  0  0  0  0  0  0  0
   -5.9726   -2.0377   -3.1826 H   0  0  0  0  0  0  0  0  0  0  0  0
   -8.3517   -3.4558   -0.4458 C   0  0  0  0  0  0  0  0  0  0  0  0
   -6.9377   -2.6750    0.9883 H   0  0  0  0  0  0  0  0  0  0  0  0
   -8.6715   -3.6228   -1.8049 C   0  0  0  0  0  0  0  0  0  0  0  0
   -8.0523   -3.2202   -3.8424 H   0  0  0  0  0  0  0  0  0  0  0  0
   -9.0307   -3.8350    0.3204 H   0  0  0  0  0  0  0  0  0  0  0  0
   -1.1238   -4.2890    1.3868 C   0  0  0  0  0  0  0  0  0  0  0  0
    0.1502   -4.7291    1.7982 C   0  0  0  0  0  0  0  0  0  0  0  0
   -2.2277   -5.1612    1.4365 C   0  0  0  0  0  0  0  0  0  0  0  0
    0.3192   -6.0458    2.2149 C   0  0  0  0  0  0  0  0  0  0  0  0
    0.9990   -4.0443    1.7796 H   0  0  0  0  0  0  0  0  0  0  0  0
   -2.0438   -6.4766    1.8559 C   0  0  0  0  0  0  0  0  0  0  0  0
   -3.2171   -4.8099    1.1411 H   0  0  0  0  0  0  0  0  0  0  0  0
```

```
   -0.7703   -6.9363    2.2365 C   0  0  0  0  0  0  0  0  0  0  0  0
    1.3080   -6.3941    2.5209 H   0  0  0  0  0  0  0  0  0  0  0  0
   -2.8953   -7.1592    1.8807 H   0  0  0  0  0  0  0  0  0  0  0  0
   -0.5649   -8.3690    2.6343 C   0  0  0  0  0  0  0  0  0  0  0  0
    0.2188   -8.4405    3.4296 H   0  0  0  0  0  0  0  0  0  0  0  0
   -1.5082   -8.7979    3.0546 H   0  0  0  0  0  0  0  0  0  0  0  0
   -9.9325   -4.3273   -2.2137 C   0  0  0  0  0  0  0  0  0  0  0  0
   -9.7503   -4.9307   -3.1384 H   0  0  0  0  0  0  0  0  0  0  0  0
  -10.2646   -5.0331   -1.4125 H   0  0  0  0  0  0  0  0  0  0  0  0
   -0.1287   -9.2013    1.4111 C   0  0  0  0  0  0  0  0  0  0  0  0
   -0.9147   -9.1247    0.6211 H   0  0  0  0  0  0  0  0  0  0  0  0
    0.8232   -8.7819    1.0042 H   0  0  0  0  0  0  0  0  0  0  0  0
  -11.0557   -3.3057   -2.4848 C   0  0  0  0  0  0  0  0  0  0  0  0
  -10.7121   -2.5797   -3.2613 H   0  0  0  0  0  0  0  0  0  0  0  0
  -11.2677   -2.7421   -1.5437 H   0  0  0  0  0  0  0  0  0  0  0  0
    0.0815  -10.6701    1.7771 C   0  0  0  0  0  0  0  0  0  0  0  0
    0.3991  -11.2414    0.8746 H   0  0  0  0  0  0  0  0  0  0  0  0
    0.8709  -10.7727    2.5556 H   0  0  0  0  0  0  0  0  0  0  0  0
   -0.8614  -11.1168    2.1650 H   0  0  0  0  0  0  0  0  0  0  0  0
  -12.3346   -3.9899   -2.9662 C   0  0  0  0  0  0  0  0  0  0  0  0
  -13.1261   -3.2244   -3.1406 H   0  0  0  0  0  0  0  0  0  0  0  0
  -12.1546   -4.5352   -3.9199 H   0  0  0  0  0  0  0  0  0  0  0  0
  -12.7041   -4.7134   -2.2054 H   0  0  0  0  0  0  0  0  0  0  0  0
  1  2  1  0  0  0  0
  1 11  1  0  0  0  0
  1 17  2  0  0  0  0
  1 49  1  0  0  0  0
  2  4  1  0  0  0  0
  2 12  1  0  0  0  0
  2  7  1  0  0  0  0
  3  5  1  0  0  0  0
  3  9  1  0  0  0  0
  3 13  1  0  0  0  0
  3 19  1  0  0  0  0
  4  6  1  0  0  0  0
  4  8  1  0  0  0  0
  4 14  1  0  0  0  0
  5  6  1  0  0  0  0
  5 10  1  0  0  0  0
  5 15  1  0  0  0  0
  6 16  1  0  0  0  0
  6 44  1  0  0  0  0
 17 18  1  0  0  0  0
 17 19  1  0  0  0  0
 17 49  1  0  0  0  0
 19 21  1  0  0  0  0
 19 20  1  0  0  0  0
 22 23  1  0  0  0  0
 22 32  1  0  0  0  0
 22 38  2  0  0  0  0
 22 49  1  0  0  0  0
 23 25  1  0  0  0  0
 23 28  1  0  0  0  0
 23 33  1  0  0  0  0
 24 26  1  0  0  0  0
 24 30  1  0  0  0  0
 24 34  1  0  0  0  0
 24 40  1  0  0  0  0
 25 27  1  0  0  0  0
 25 29  1  0  0  0  0
 25 35  1  0  0  0  0
 26 27  1  0  0  0  0
 26 31  1  0  0  0  0
 26 36  1  0  0  0  0
 27 37  1  0  0  0  0
```

```
 27 43  1  0  0  0  0
 38 39  1  0  0  0  0
 38 40  1  0  0  0  0
 38 49  1  0  0  0  0
 40 41  1  0  0  0  0
 40 42  1  0  0  0  0
 45 46  3  0  0  0  0
 45 60  1  0  0  0  0
 46 49  1  0  0  0  0
 47 48  3  0  0  0  0
 47 50  1  0  0  0  0
 48 49  1  0  0  0  0
 50 51  2  0  0  0  0
 50 52  1  0  0  0  0
 51 53  1  0  0  0  0
 51 54  1  0  0  0  0
 52 55  2  0  0  0  0
 52 56  1  0  0  0  0
 53 57  2  0  0  0  0
 53 58  1  0  0  0  0
 55 57  1  0  0  0  0
 55 59  1  0  0  0  0
 57 73  1  0  0  0  0
 60 61  2  0  0  0  0
 60 62  1  0  0  0  0
 61 63  1  0  0  0  0
 61 64  1  0  0  0  0
 62 65  2  0  0  0  0
 62 66  1  0  0  0  0
 63 67  2  0  0  0  0
 63 68  1  0  0  0  0
 65 67  1  0  0  0  0
 65 69  1  0  0  0  0
 67 70  1  0  0  0  0
 70 71  1  0  0  0  0
 70 72  1  0  0  0  0
 70 76  1  0  0  0  0
 73 74  1  0  0  0  0
 73 75  1  0  0  0  0
 73 79  1  0  0  0  0
 76 77  1  0  0  0  0
 76 78  1  0  0  0  0
 76 82  1  0  0  0  0
 79 80  1  0  0  0  0
 79 81  1  0  0  0  0
 79 86  1  0  0  0  0
 82 83  1  0  0  0  0
 82 84  1  0  0  0  0
 82 85  1  0  0  0  0
 86 87  1  0  0  0  0
 86 88  1  0  0  0  0
 86 89  1  0  0  0  0
M  END
$$$$
mol166
Optimized Catalyst 166
Optimized Catalyst 166
 89 94  0  0  0  0  0  0  0  0999 V2000
   -2.6682    0.2787   -0.7471 C   0  0  0  0  0  0  0  0  0  0  0  0
   -1.7230    1.1607    0.0214 C   0  0  0  0  0  0  0  0  0  0  0  0
   -1.6179    1.6679   -3.4913 C   0  0  0  0  0  0  0  0  0  0  0  0
   -1.6622    2.5961   -0.5574 C   0  0  0  0  0  0  0  0  0  0  0  0
   -0.7060    2.7915   -2.9758 C   0  0  0  0  0  0  0  0  0  0  0  0
   -0.4453    2.8539   -1.4610 C   0  0  0  0  0  0  0  0  0  0  0  0
   -0.6940    0.7230    0.0253 H   0  0  0  0  0  0  0  0  0  0  0  0
```

```
   -2.6015    2.8160   -1.1170 H   0  0  0  0  0  0  0  0  0  0  0  0
   -2.6813    1.9430   -3.2846 H   0  0  0  0  0  0  0  0  0  0  0  0
   -1.1689    3.7618   -3.2936 H   0  0  0  0  0  0  0  0  0  0  0  0
   -3.6417    0.1119   -0.2388 H   0  0  0  0  0  0  0  0  0  0  0  0
   -2.0663    1.2295    1.0861 H   0  0  0  0  0  0  0  0  0  0  0  0
   -1.4954    1.6129   -4.6026 H   0  0  0  0  0  0  0  0  0  0  0  0
   -1.6101    3.3040    0.3086 H   0  0  0  0  0  0  0  0  0  0  0  0
    0.2876    2.7078   -3.4848 H   0  0  0  0  0  0  0  0  0  0  0  0
   -0.0667    3.8873   -1.2395 H   0  0  0  0  0  0  0  0  0  0  0  0
   -2.4812   -0.1533   -2.0241 C   0  0  0  0  0  0  0  0  0  0  0  0
   -3.3117   -0.6483   -2.5827 H   0  0  0  0  0  0  0  0  0  0  0  0
   -1.3268    0.2684   -2.8987 C   0  0  0  0  0  0  0  0  0  0  0  0
   -0.3750    0.3096   -2.3144 H   0  0  0  0  0  0  0  0  0  0  0  0
   -1.1980   -0.4602   -3.7373 H   0  0  0  0  0  0  0  0  0  0  0  0
    0.2734   -2.8624   -1.7194 C   0  0  0  0  0  0  0  0  0  0  0  0
    1.1909   -2.4794   -2.8440 C   0  0  0  0  0  0  0  0  0  0  0  0
    2.5769   -1.9565    0.3749 C   0  0  0  0  0  0  0  0  0  0  0  0
    2.6700   -2.8364   -2.5627 C   0  0  0  0  0  0  0  0  0  0  0  0
    3.7600   -1.6071   -0.5401 C   0  0  0  0  0  0  0  0  0  0  0  0
    3.5112   -1.6526   -2.0573 C   0  0  0  0  0  0  0  0  0  0  0  0
    1.1109   -1.3824   -3.0474 H   0  0  0  0  0  0  0  0  0  0  0  0
    2.7210   -3.6765   -1.8319 H   0  0  0  0  0  0  0  0  0  0  0  0
    2.4377   -3.0648    0.3787 H   0  0  0  0  0  0  0  0  0  0  0  0
    4.5881   -2.3217   -0.2967 H   0  0  0  0  0  0  0  0  0  0  0  0
   -0.3919   -3.7231   -1.9562 H   0  0  0  0  0  0  0  0  0  0  0  0
    0.8740   -3.0172   -3.7741 H   0  0  0  0  0  0  0  0  0  0  0  0
    2.8496   -1.6476    1.4154 H   0  0  0  0  0  0  0  0  0  0  0  0
    3.1168   -3.1954   -3.5243 H   0  0  0  0  0  0  0  0  0  0  0  0
    4.1154   -0.5761   -0.2875 H   0  0  0  0  0  0  0  0  0  0  0  0
    4.5169   -1.7080   -2.5516 H   0  0  0  0  0  0  0  0  0  0  0  0
    0.2599   -2.3113   -0.4857 C   0  0  0  0  0  0  0  0  0  0  0  0
   -0.3769   -2.7411    0.3378 H   0  0  0  0  0  0  0  0  0  0  0  0
    1.2403   -1.2757   -0.0024 C   0  0  0  0  0  0  0  0  0  0  0  0
    1.4257   -0.5086   -0.7933 H   0  0  0  0  0  0  0  0  0  0  0  0
    0.8363   -0.7581    0.9013 H   0  0  0  0  0  0  0  0  0  0  0  0
    3.0450   -0.6920   -2.3807 H   0  0  0  0  0  0  0  0  0  0  0  0
    0.3774    2.1472   -1.2003 H   0  0  0  0  0  0  0  0  0  0  0  0
   -2.4142   -5.4131    0.0284 C   0  0  0  0  0  0  0  0  0  0  0  0
   -2.3897   -4.2829   -0.2696 N   0  0  0  0  0  0  0  0  0  0  0  0
   -5.5533   -2.4527   -0.7248 C   0  0  0  0  0  0  0  0  0  0  0  0
   -4.3865   -2.3990   -0.7724 N   0  0  0  0  0  0  0  0  0  0  0  0
   -2.4315   -2.3950   -0.7940 Ir  0  0  0  0  0  0  0  0  0  0  0  0
   -6.9690   -2.4928   -0.6664 C   0  0  0  0  0  0  0  0  0  0  0  0
   -7.7169   -2.8361   -1.8140 C   0  0  0  0  0  0  0  0  0  0  0  0
   -7.6304   -2.1864    0.5439 C   0  0  0  0  0  0  0  0  0  0  0  0
   -9.1042   -2.8821   -1.7419 C   0  0  0  0  0  0  0  0  0  0  0  0
   -7.2061   -3.0638   -2.7517 H   0  0  0  0  0  0  0  0  0  0  0  0
   -9.0178   -2.2361    0.6008 C   0  0  0  0  0  0  0  0  0  0  0  0
   -7.0521   -1.9125    1.4284 H   0  0  0  0  0  0  0  0  0  0  0  0
   -9.7721   -2.5905   -0.5358 C   0  0  0  0  0  0  0  0  0  0  0  0
   -9.6853   -3.1443   -2.6304 H   0  0  0  0  0  0  0  0  0  0  0  0
   -9.5317   -1.9955    1.5356 H   0  0  0  0  0  0  0  0  0  0  0  0
   -2.4274   -6.7857    0.3832 C   0  0  0  0  0  0  0  0  0  0  0  0
   -2.4141   -7.1654    1.7437 C   0  0  0  0  0  0  0  0  0  0  0  0
   -2.4545   -7.7736   -0.6264 C   0  0  0  0  0  0  0  0  0  0  0  0
   -2.4406   -8.5133    2.0815 C   0  0  0  0  0  0  0  0  0  0  0  0
   -2.3831   -6.4022    2.5239 H   0  0  0  0  0  0  0  0  0  0  0  0
   -2.4804   -9.1173   -0.2732 C   0  0  0  0  0  0  0  0  0  0  0  0
   -2.4552   -7.4806   -1.6781 H   0  0  0  0  0  0  0  0  0  0  0  0
   -2.4808   -9.5055    1.0817 C   0  0  0  0  0  0  0  0  0  0  0  0
   -2.4272   -8.8090    3.1342 H   0  0  0  0  0  0  0  0  0  0  0  0
   -2.4979   -9.8832   -1.0535 H   0  0  0  0  0  0  0  0  0  0  0  0
   -2.5155  -10.9573    1.4546 C   0  0  0  0  0  0  0  0  0  0  0  0
   -3.0537  -11.0962    2.4265 H   0  0  0  0  0  0  0  0  0  0  0  0
   -3.0689  -11.5454    0.6793 H   0  0  0  0  0  0  0  0  0  0  0  0
```

```
  -11.2683   -2.6488   -0.4634 C   0  0  0  0  0  0  0  0  0  0  0  0
  -11.6617   -3.4257   -1.1670 H   0  0  0  0  0  0  0  0  0  0  0  0
  -11.5971   -2.9344    0.5680 H   0  0  0  0  0  0  0  0  0  0  0  0
   -1.0841  -11.5183    1.5860 C   0  0  0  0  0  0  0  0  0  0  0  0
   -0.5493  -11.3867    0.6141 H   0  0  0  0  0  0  0  0  0  0  0  0
   -0.5375  -10.9447    2.3736 H   0  0  0  0  0  0  0  0  0  0  0  0
  -11.8830   -1.2812   -0.8272 C   0  0  0  0  0  0  0  0  0  0  0  0
  -11.5669   -1.0013   -1.8615 H   0  0  0  0  0  0  0  0  0  0  0  0
  -11.5009   -0.5077   -0.1172 H   0  0  0  0  0  0  0  0  0  0  0  0
   -1.0974  -13.0004    1.9584 C   0  0  0  0  0  0  0  0  0  0  0  0
   -0.0506  -13.3733    2.0507 H   0  0  0  0  0  0  0  0  0  0  0  0
   -1.6142  -13.1584    2.9318 H   0  0  0  0  0  0  0  0  0  0  0  0
   -1.6165  -13.5992    1.1765 H   0  0  0  0  0  0  0  0  0  0  0  0
  -13.4090   -1.3204   -0.7584 C   0  0  0  0  0  0  0  0  0  0  0  0
  -13.8228   -0.3207   -1.0282 H   0  0  0  0  0  0  0  0  0  0  0  0
  -13.8167   -2.0736   -1.4698 H   0  0  0  0  0  0  0  0  0  0  0  0
  -13.7512   -1.5771    0.2696 H   0  0  0  0  0  0  0  0  0  0  0  0
  1  2  1  0  0  0  0
  1 11  1  0  0  0  0
  1 17  2  0  0  0  0
  1 49  1  0  0  0  0
  2  4  1  0  0  0  0
  2  7  1  0  0  0  0
  2 12  1  0  0  0  0
  3  5  1  0  0  0  0
  3  9  1  0  0  0  0
  3 13  1  0  0  0  0
  3 19  1  0  0  0  0
  4  6  1  0  0  0  0
  4  8  1  0  0  0  0
  4 14  1  0  0  0  0
  5  6  1  0  0  0  0
  5 10  1  0  0  0  0
  5 15  1  0  0  0  0
  6 16  1  0  0  0  0
  6 44  1  0  0  0  0
 17 18  1  0  0  0  0
 17 19  1  0  0  0  0
 17 49  1  0  0  0  0
 19 20  1  0  0  0  0
 19 21  1  0  0  0  0
 22 23  1  0  0  0  0
 22 32  1  0  0  0  0
 22 38  2  0  0  0  0
 22 49  1  0  0  0  0
 23 25  1  0  0  0  0
 23 28  1  0  0  0  0
 23 33  1  0  0  0  0
 24 26  1  0  0  0  0
 24 30  1  0  0  0  0
 24 34  1  0  0  0  0
 24 40  1  0  0  0  0
 25 27  1  0  0  0  0
 25 29  1  0  0  0  0
 25 35  1  0  0  0  0
 26 27  1  0  0  0  0
 26 31  1  0  0  0  0
 26 36  1  0  0  0  0
 27 37  1  0  0  0  0
 27 43  1  0  0  0  0
 38 39  1  0  0  0  0
 38 40  1  0  0  0  0
 38 49  1  0  0  0  0
 40 41  1  0  0  0  0
 40 42  1  0  0  0  0
```

```
 45 46  3  0  0  0  0
 45 60  1  0  0  0  0
 46 49  1  0  0  0  0
 47 48  3  0  0  0  0
 47 50  1  0  0  0  0
 48 49  1  0  0  0  0
 50 51  2  0  0  0  0
 50 52  1  0  0  0  0
 51 53  1  0  0  0  0
 51 54  1  0  0  0  0
 52 55  2  0  0  0  0
 52 56  1  0  0  0  0
 53 57  2  0  0  0  0
 53 58  1  0  0  0  0
 55 57  1  0  0  0  0
 55 59  1  0  0  0  0
 57 73  1  0  0  0  0
 60 61  2  0  0  0  0
 60 62  1  0  0  0  0
 61 63  1  0  0  0  0
 61 64  1  0  0  0  0
 62 65  2  0  0  0  0
 62 66  1  0  0  0  0
 63 67  2  0  0  0  0
 63 68  1  0  0  0  0
 65 67  1  0  0  0  0
 65 69  1  0  0  0  0
 67 70  1  0  0  0  0
 70 71  1  0  0  0  0
 70 72  1  0  0  0  0
 70 76  1  0  0  0  0
 73 74  1  0  0  0  0
 73 75  1  0  0  0  0
 73 79  1  0  0  0  0
 76 77  1  0  0  0  0
 76 78  1  0  0  0  0
 76 82  1  0  0  0  0
 79 80  1  0  0  0  0
 79 81  1  0  0  0  0
 79 86  1  0  0  0  0
 82 83  1  0  0  0  0
 82 84  1  0  0  0  0
 82 85  1  0  0  0  0
 86 87  1  0  0  0  0
 86 88  1  0  0  0  0
 86 89  1  0  0  0  0
M  END
$$$$
mol167
Optimized Catalyst 167
Optimized Catalyst 167
 89 93  0  0  0  0  0  0  0  0999 V2000
   -2.7895    0.6225   -0.6929 C   0  0  0  0  0  0  0  0  0  0  0  0
   -1.8869    1.1555    0.3911 C   0  0  0  0  0  0  0  0  0  0  0  0
   -0.8264    1.9641   -2.9136 C   0  0  0  0  0  0  0  0  0  0  0  0
   -1.2022    2.5042    0.0881 C   0  0  0  0  0  0  0  0  0  0  0  0
    0.2511    2.6511   -2.0600 C   0  0  0  0  0  0  0  0  0  0  0  0
    0.1941    2.3877   -0.5453 C   0  0  0  0  0  0  0  0  0  0  0  0
   -1.1032    0.3457    0.6105 H   0  0  0  0  0  0  0  0  0  0  0  0
   -1.8691    3.1075   -0.5735 H   0  0  0  0  0  0  0  0  0  0  0  0
   -1.7937    2.5108   -2.7953 H   0  0  0  0  0  0  0  0  0  0  0  0
    0.1668    3.7540   -2.2348 H   0  0  0  0  0  0  0  0  0  0  0  0
   -3.8709    0.5715   -0.4215 H   0  0  0  0  0  0  0  0  0  0  0  0
   -2.4957    1.2776    1.3265 H   0  0  0  0  0  0  0  0  0  0  0  0
   -0.5197    2.0499   -3.9864 H   0  0  0  0  0  0  0  0  0  0  0  0
```

```
   -1.0991    3.0540    1.0586 H   0  0  0  0  0  0  0  0  0  0  0  0
    1.2584    2.3246   -2.4226 H   0  0  0  0  0  0  0  0  0  0  0  0
    0.8641    3.1416   -0.0551 H   0  0  0  0  0  0  0  0  0  0  0  0
   -2.4060    0.2362   -1.9800 C   0  0  0  0  0  0  0  0  0  0  0  0
   -3.1891   -0.1264   -2.6857 H   0  0  0  0  0  0  0  0  0  0  0  0
   -1.0467    0.4728   -2.5822 C   0  0  0  0  0  0  0  0  0  0  0  0
   -0.2551    0.1215   -1.8562 H   0  0  0  0  0  0  0  0  0  0  0  0
   -0.9440   -0.1362   -3.5159 H   0  0  0  0  0  0  0  0  0  0  0  0
    0.2894   -2.5094   -1.9492 C   0  0  0  0  0  0  0  0  0  0  0  0
    1.1335   -3.1563   -3.0075 C   0  0  0  0  0  0  0  0  0  0  0  0
    2.6491   -3.1721    0.2160 C   0  0  0  0  0  0  0  0  0  0  0  0
    1.9250   -4.3826   -2.5024 C   0  0  0  0  0  0  0  0  0  0  0  0
    3.6790   -3.9392   -0.6274 C   0  0  0  0  0  0  0  0  0  0  0  0
    3.3860   -4.0771   -2.1318 C   0  0  0  0  0  0  0  0  0  0  0  0
    1.8393   -2.3900   -3.4183 H   0  0  0  0  0  0  0  0  0  0  0  0
    1.3985   -4.8207   -1.6215 H   0  0  0  0  0  0  0  0  0  0  0  0
    1.7611   -3.8301    0.3859 H   0  0  0  0  0  0  0  0  0  0  0  0
    3.7656   -4.9646   -0.1897 H   0  0  0  0  0  0  0  0  0  0  0  0
   -0.8056   -2.5245   -2.1688 H   0  0  0  0  0  0  0  0  0  0  0  0
    0.4739   -3.4877   -3.8475 H   0  0  0  0  0  0  0  0  0  0  0  0
    3.1128   -2.9634    1.2108 H   0  0  0  0  0  0  0  0  0  0  0  0
    1.9245   -5.1489   -3.3156 H   0  0  0  0  0  0  0  0  0  0  0  0
    4.6718   -3.4344   -0.5230 H   0  0  0  0  0  0  0  0  0  0  0  0
    4.0246   -4.9134   -2.5156 H   0  0  0  0  0  0  0  0  0  0  0  0
    0.7392   -1.9324   -0.8243 C   0  0  0  0  0  0  0  0  0  0  0  0
    0.0224   -1.4926   -0.0942 H   0  0  0  0  0  0  0  0  0  0  0  0
    2.1745   -1.8392   -0.3977 C   0  0  0  0  0  0  0  0  0  0  0  0
    2.8132   -1.5761   -1.2768 H   0  0  0  0  0  0  0  0  0  0  0  0
    2.2880   -1.0234    0.3564 H   0  0  0  0  0  0  0  0  0  0  0  0
    3.7172   -3.1467   -2.6512 H   0  0  0  0  0  0  0  0  0  0  0  0
    0.6242    1.3803   -0.3300 H   0  0  0  0  0  0  0  0  0  0  0  0
   -2.0074   -3.9141    0.3937 C   0  0  0  0  0  0  0  0  0  0  0  0
   -2.0722   -2.8409   -0.0467 N   0  0  0  0  0  0  0  0  0  0  0  0
   -5.8133   -2.1257   -1.1718 C   0  0  0  0  0  0  0  0  0  0  0  0
   -4.6888   -1.8797   -1.0418 N   0  0  0  0  0  0  0  0  0  0  0  0
   -2.2611   -1.3278   -0.6948 Rh  0  0  0  0  0  0  0  0  0  0  0  0
   -1.9119   -5.2504    0.9574 C   0  0  0  0  0  0  0  0  0  0  0  0
   -2.1404   -5.1926    2.0612 H   0  0  0  0  0  0  0  0  0  0  0  0
   -2.6975   -5.9043    0.4793 H   0  0  0  0  0  0  0  0  0  0  0  0
   -7.2242   -2.4370   -1.3339 C   0  0  0  0  0  0  0  0  0  0  0  0
   -7.3529   -3.1189   -2.2229 H   0  0  0  0  0  0  0  0  0  0  0  0
   -7.5897   -2.9954   -0.4244 H   0  0  0  0  0  0  0  0  0  0  0  0
   -0.5455   -5.8638    0.7480 C   0  0  0  0  0  0  0  0  0  0  0  0
   -0.2731   -6.5946   -0.4174 C   0  0  0  0  0  0  0  0  0  0  0  0
    0.4426   -5.7388    1.7346 C   0  0  0  0  0  0  0  0  0  0  0  0
    0.9707   -7.2045   -0.5866 C   0  0  0  0  0  0  0  0  0  0  0  0
   -1.0296   -6.6951   -1.1946 H   0  0  0  0  0  0  0  0  0  0  0  0
    1.6854   -6.3507    1.5627 C   0  0  0  0  0  0  0  0  0  0  0  0
    0.2471   -5.1703    2.6430 H   0  0  0  0  0  0  0  0  0  0  0  0
    1.9619   -7.0919    0.4025 C   0  0  0  0  0  0  0  0  0  0  0  0
    1.1715   -7.7793   -1.4913 H   0  0  0  0  0  0  0  0  0  0  0  0
    2.4440   -6.2571    2.3404 H   0  0  0  0  0  0  0  0  0  0  0  0
   -8.0480   -1.1833   -1.5221 C   0  0  0  0  0  0  0  0  0  0  0  0
   -8.5160   -0.4761   -0.4052 C   0  0  0  0  0  0  0  0  0  0  0  0
   -8.3634   -0.7340   -2.8118 C   0  0  0  0  0  0  0  0  0  0  0  0
   -9.2890    0.6722   -0.5788 C   0  0  0  0  0  0  0  0  0  0  0  0
   -8.2870   -0.8220    0.6018 H   0  0  0  0  0  0  0  0  0  0  0  0
   -9.1383    0.4143   -2.9820 C   0  0  0  0  0  0  0  0  0  0  0  0
   -8.0146   -1.2791   -3.6878 H   0  0  0  0  0  0  0  0  0  0  0  0
   -9.6058    1.1307   -1.8684 C   0  0  0  0  0  0  0  0  0  0  0  0
   -9.6600    1.2111    0.2939 H   0  0  0  0  0  0  0  0  0  0  0  0
   -9.3911    0.7501   -3.9885 H   0  0  0  0  0  0  0  0  0  0  0  0
  -10.4517    2.3619   -2.0530 C   0  0  0  0  0  0  0  0  0  0  0  0
  -10.2625    3.0827   -1.2186 H   0  0  0  0  0  0  0  0  0  0  0  0
  -10.1797    2.8718   -3.0110 H   0  0  0  0  0  0  0  0  0  0  0  0
```

```
    3.2964   -7.7650    0.2240 C   0  0  0  0  0  0  0  0  0  0  0  0
    3.5971   -7.7252   -0.8532 H   0  0  0  0  0  0  0  0  0  0  0  0
    4.0797   -7.2307    0.8181 H   0  0  0  0  0  0  0  0  0  0  0  0
    3.2362   -9.2257    0.6825 C   0  0  0  0  0  0  0  0  0  0  0  0
    2.4908   -9.7928    0.0807 H   0  0  0  0  0  0  0  0  0  0  0  0
    4.2360   -9.7009    0.5576 H   0  0  0  0  0  0  0  0  0  0  0  0
    2.9439   -9.2800    1.7559 H   0  0  0  0  0  0  0  0  0  0  0  0
  -11.9392    1.9958   -2.0793 C   0  0  0  0  0  0  0  0  0  0  0  0
  -12.2337    1.5054   -1.1240 H   0  0  0  0  0  0  0  0  0  0  0  0
  -12.5519    2.9156   -2.2162 H   0  0  0  0  0  0  0  0  0  0  0  0
  -12.1490    1.2954   -2.9192 H   0  0  0  0  0  0  0  0  0  0  0  0
  1  2  1  0  0  0  0
  1 11  1  0  0  0  0
  1 17  2  0  0  0  0
  1 49  1  0  0  0  0
  2  4  1  0  0  0  0
  2 12  1  0  0  0  0
  3  5  1  0  0  0  0
  3  9  1  0  0  0  0
  3 13  1  0  0  0  0
  3 19  1  0  0  0  0
  4  6  1  0  0  0  0
  4  8  1  0  0  0  0
  4 14  1  0  0  0  0
  5  6  1  0  0  0  0
  5 10  1  0  0  0  0
  5 15  1  0  0  0  0
  6 16  1  0  0  0  0
  6 44  1  0  0  0  0
 17 18  1  0  0  0  0
 17 19  1  0  0  0  0
 17 49  1  0  0  0  0
 19 20  1  0  0  0  0
 19 21  1  0  0  0  0
 22 23  1  0  0  0  0
 22 32  1  0  0  0  0
 22 38  2  0  0  0  0
 22 49  1  0  0  0  0
 23 25  1  0  0  0  0
 23 28  1  0  0  0  0
 23 33  1  0  0  0  0
 24 26  1  0  0  0  0
 24 30  1  0  0  0  0
 24 34  1  0  0  0  0
 24 40  1  0  0  0  0
 25 27  1  0  0  0  0
 25 29  1  0  0  0  0
 25 35  1  0  0  0  0
 26 27  1  0  0  0  0
 26 31  1  0  0  0  0
 26 36  1  0  0  0  0
 27 37  1  0  0  0  0
 27 43  1  0  0  0  0
 38 39  1  0  0  0  0
 38 40  1  0  0  0  0
 38 49  1  0  0  0  0
 40 41  1  0  0  0  0
 40 42  1  0  0  0  0
 45 46  3  0  0  0  0
 45 50  1  0  0  0  0
 46 49  1  0  0  0  0
 47 48  3  0  0  0  0
 47 53  1  0  0  0  0
 48 49  1  0  0  0  0
 50 51  1  0  0  0  0
```

```
 50 52  1  0  0  0  0
 50 56  1  0  0  0  0
 53 54  1  0  0  0  0
 53 55  1  0  0  0  0
 53 66  1  0  0  0  0
 56 57  2  0  0  0  0
 56 58  1  0  0  0  0
 57 59  1  0  0  0  0
 57 60  1  0  0  0  0
 58 61  2  0  0  0  0
 58 62  1  0  0  0  0
 59 63  2  0  0  0  0
 59 64  1  0  0  0  0
 61 63  1  0  0  0  0
 61 65  1  0  0  0  0
 63 79  1  0  0  0  0
 66 67  2  0  0  0  0
 66 68  1  0  0  0  0
 67 69  1  0  0  0  0
 67 70  1  0  0  0  0
 68 71  2  0  0  0  0
 68 72  1  0  0  0  0
 69 73  2  0  0  0  0
 69 74  1  0  0  0  0
 71 73  1  0  0  0  0
 71 75  1  0  0  0  0
 73 76  1  0  0  0  0
 76 77  1  0  0  0  0
 76 78  1  0  0  0  0
 76 86  1  0  0  0  0
 79 80  1  0  0  0  0
 79 81  1  0  0  0  0
 79 82  1  0  0  0  0
 82 83  1  0  0  0  0
 82 84  1  0  0  0  0
 82 85  1  0  0  0  0
 86 87  1  0  0  0  0
 86 88  1  0  0  0  0
 86 89  1  0  0  0  0
M  END
$$$$
mol168
Optimized Catalyst 168
Optimized Catalyst 168
 89 94  0  0  0  0  0  0  0  0999 V2000
   -2.6646    0.2985   -0.7591 C   0  0  0  0  0  0  0  0  0  0  0  0
   -1.7172    1.1728    0.0150 C   0  0  0  0  0  0  0  0  0  0  0  0
   -1.6041    1.6817   -3.4962 C   0  0  0  0  0  0  0  0  0  0  0  0
   -1.6497    2.6093   -0.5610 C   0  0  0  0  0  0  0  0  0  0  0  0
   -0.6912    2.8036   -2.9789 C   0  0  0  0  0  0  0  0  0  0  0  0
   -0.4317    2.8648   -1.4638 C   0  0  0  0  0  0  0  0  0  0  0  0
   -0.6898    0.7314    0.0217 H   0  0  0  0  0  0  0  0  0  0  0  0
   -2.5881    2.8340   -1.1204 H   0  0  0  0  0  0  0  0  0  0  0  0
   -2.6673    1.9573   -3.2886 H   0  0  0  0  0  0  0  0  0  0  0  0
   -1.1527    3.7747   -3.2964 H   0  0  0  0  0  0  0  0  0  0  0  0
   -3.6452    0.1460   -0.2598 H   0  0  0  0  0  0  0  0  0  0  0  0
   -2.0638    1.2409    1.0790 H   0  0  0  0  0  0  0  0  0  0  0  0
   -1.4825    1.6287   -4.6077 H   0  0  0  0  0  0  0  0  0  0  0  0
   -1.5948    3.3151    0.3067 H   0  0  0  0  0  0  0  0  0  0  0  0
    0.3026    2.7191   -3.4873 H   0  0  0  0  0  0  0  0  0  0  0  0
   -0.0513    3.8976   -1.2418 H   0  0  0  0  0  0  0  0  0  0  0  0
   -2.4723   -0.1393   -2.0346 C   0  0  0  0  0  0  0  0  0  0  0  0
   -3.3069   -0.6207   -2.5999 H   0  0  0  0  0  0  0  0  0  0  0  0
   -1.3141    0.2808   -2.9059 C   0  0  0  0  0  0  0  0  0  0  0  0
   -0.3633    0.3210   -2.3198 H   0  0  0  0  0  0  0  0  0  0  0  0
```

```
   -1.1848   -0.4463   -3.7459 H   0  0  0  0  0  0  0  0  0  0  0  0
    0.2898   -2.8605   -1.7150 C   0  0  0  0  0  0  0  0  0  0  0  0
    1.2017   -2.4718   -2.8423 C   0  0  0  0  0  0  0  0  0  0  0  0
    2.5916   -1.9437    0.3738 C   0  0  0  0  0  0  0  0  0  0  0  0
    2.6812   -2.8298   -2.5630 C   0  0  0  0  0  0  0  0  0  0  0  0
    3.7737   -1.5964   -0.5433 C   0  0  0  0  0  0  0  0  0  0  0  0
    3.5250   -1.6467   -2.0604 C   0  0  0  0  0  0  0  0  0  0  0  0
    1.1211   -1.3746   -3.0431 H   0  0  0  0  0  0  0  0  0  0  0  0
    2.7321   -3.6689   -1.8307 H   0  0  0  0  0  0  0  0  0  0  0  0
    2.4532   -3.0524    0.3808 H   0  0  0  0  0  0  0  0  0  0  0  0
    4.6024   -2.3094   -0.2977 H   0  0  0  0  0  0  0  0  0  0  0  0
   -0.3595   -3.7348   -1.9458 H   0  0  0  0  0  0  0  0  0  0  0  0
    0.8833   -3.0079   -3.7731 H   0  0  0  0  0  0  0  0  0  0  0  0
    2.8651   -1.6320    1.4133 H   0  0  0  0  0  0  0  0  0  0  0  0
    3.1261   -3.1906   -3.5249 H   0  0  0  0  0  0  0  0  0  0  0  0
    4.1287   -0.5643   -0.2940 H   0  0  0  0  0  0  0  0  0  0  0  0
    4.5310   -1.7067   -2.5539 H   0  0  0  0  0  0  0  0  0  0  0  0
    0.2763   -2.3064   -0.4816 C   0  0  0  0  0  0  0  0  0  0  0  0
   -0.3458   -2.7441    0.3469 H   0  0  0  0  0  0  0  0  0  0  0  0
    1.2537   -1.2650   -0.0041 C   0  0  0  0  0  0  0  0  0  0  0  0
    1.4378   -0.5001   -0.7971 H   0  0  0  0  0  0  0  0  0  0  0  0
    0.8497   -0.7457    0.8989 H   0  0  0  0  0  0  0  0  0  0  0  0
    3.0621   -0.6858   -2.3873 H   0  0  0  0  0  0  0  0  0  0  0  0
    0.3894    2.1565   -1.2029 H   0  0  0  0  0  0  0  0  0  0  0  0
   -2.3674   -5.3800    0.0143 C   0  0  0  0  0  0  0  0  0  0  0  0
   -2.3623   -4.2548   -0.2919 N   0  0  0  0  0  0  0  0  0  0  0  0
   -5.5284   -2.4039   -0.7433 C   0  0  0  0  0  0  0  0  0  0  0  0
   -4.3639   -2.3695   -0.7932 N   0  0  0  0  0  0  0  0  0  0  0  0
   -2.4068   -2.3677   -0.8258 Ir  0  0  0  0  0  0  0  0  0  0  0  0
   -2.3604   -6.7815    0.3893 C   0  0  0  0  0  0  0  0  0  0  0  0
   -2.8933   -6.9068    1.3787 H   0  0  0  0  0  0  0  0  0  0  0  0
   -2.9352   -7.3760   -0.3820 H   0  0  0  0  0  0  0  0  0  0  0  0
   -6.9779   -2.4252   -0.6841 C   0  0  0  0  0  0  0  0  0  0  0  0
   -7.3701   -3.2103   -1.3970 H   0  0  0  0  0  0  0  0  0  0  0  0
   -7.3045   -2.7203    0.3576 H   0  0  0  0  0  0  0  0  0  0  0  0
   -0.9481   -7.3117    0.4967 C   0  0  0  0  0  0  0  0  0  0  0  0
   -0.3230   -7.8781   -0.6233 C   0  0  0  0  0  0  0  0  0  0  0  0
   -0.2719   -7.2623    1.7243 C   0  0  0  0  0  0  0  0  0  0  0  0
    0.9716   -8.3862   -0.5156 C   0  0  0  0  0  0  0  0  0  0  0  0
   -0.8427   -7.9363   -1.5791 H   0  0  0  0  0  0  0  0  0  0  0  0
    1.0225   -7.7717    1.8272 C   0  0  0  0  0  0  0  0  0  0  0  0
   -0.7517   -6.8375    2.6055 H   0  0  0  0  0  0  0  0  0  0  0  0
    1.6592   -8.3358    0.7089 C   0  0  0  0  0  0  0  0  0  0  0  0
    1.4490   -8.8389   -1.3861 H   0  0  0  0  0  0  0  0  0  0  0  0
    1.5396   -7.7436    2.7878 H   0  0  0  0  0  0  0  0  0  0  0  0
   -7.5558   -1.0738   -1.0400 C   0  0  0  0  0  0  0  0  0  0  0  0
   -7.8028   -0.1286   -0.0338 C   0  0  0  0  0  0  0  0  0  0  0  0
   -7.8700   -0.7737   -2.3732 C   0  0  0  0  0  0  0  0  0  0  0  0
   -8.3562    1.1091   -0.3619 C   0  0  0  0  0  0  0  0  0  0  0  0
   -7.5773   -0.3547    1.0078 H   0  0  0  0  0  0  0  0  0  0  0  0
   -8.4230    0.4653   -2.6964 C   0  0  0  0  0  0  0  0  0  0  0  0
   -7.6964   -1.5046   -3.1625 H   0  0  0  0  0  0  0  0  0  0  0  0
   -8.6695    1.4205   -1.6958 C   0  0  0  0  0  0  0  0  0  0  0  0
   -8.5604    1.8361    0.4260 H   0  0  0  0  0  0  0  0  0  0  0  0
   -8.6790    0.6892   -3.7334 H   0  0  0  0  0  0  0  0  0  0  0  0
   -9.2806    2.7501   -2.0450 C   0  0  0  0  0  0  0  0  0  0  0  0
   -8.9170    3.5371   -1.3374 H   0  0  0  0  0  0  0  0  0  0  0  0
   -8.9737    3.0538   -3.0775 H   0  0  0  0  0  0  0  0  0  0  0  0
    3.0501   -8.8971    0.8266 C   0  0  0  0  0  0  0  0  0  0  0  0
    3.5922   -8.7848   -0.1459 H   0  0  0  0  0  0  0  0  0  0  0  0
    3.6260   -8.3340    1.6036 H   0  0  0  0  0  0  0  0  0  0  0  0
    2.9994  -10.3796    1.2099 C   0  0  0  0  0  0  0  0  0  0  0  0
    2.4546  -10.9621    0.4328 H   0  0  0  0  0  0  0  0  0  0  0  0
    4.0349  -10.7808    1.2998 H   0  0  0  0  0  0  0  0  0  0  0  0
    2.4778  -10.5098    2.1851 H   0  0  0  0  0  0  0  0  0  0  0  0
```

```
  -10.8090    2.6719   -1.9737 C   0  0  0  0  0  0  0  0  0  0  0  0
  -11.1353    2.3977   -0.9448 H   0  0  0  0  0  0  0  0  0  0  0  0
  -11.2495    3.6602   -2.2390 H   0  0  0  0  0  0  0  0  0  0  0  0
  -11.1910    1.9056   -2.6857 H   0  0  0  0  0  0  0  0  0  0  0  0
  1  2  1  0  0  0  0
  1 11  1  0  0  0  0
  1 17  2  0  0  0  0
  1 49  1  0  0  0  0
  2  4  1  0  0  0  0
  2  7  1  0  0  0  0
  2 12  1  0  0  0  0
  3  5  1  0  0  0  0
  3  9  1  0  0  0  0
  3 13  1  0  0  0  0
  3 19  1  0  0  0  0
  4  6  1  0  0  0  0
  4  8  1  0  0  0  0
  4 14  1  0  0  0  0
  5  6  1  0  0  0  0
  5 10  1  0  0  0  0
  5 15  1  0  0  0  0
  6 16  1  0  0  0  0
  6 44  1  0  0  0  0
 17 18  1  0  0  0  0
 17 19  1  0  0  0  0
 17 49  1  0  0  0  0
 19 20  1  0  0  0  0
 19 21  1  0  0  0  0
 22 23  1  0  0  0  0
 22 32  1  0  0  0  0
 22 38  2  0  0  0  0
 22 49  1  0  0  0  0
 23 25  1  0  0  0  0
 23 28  1  0  0  0  0
 23 33  1  0  0  0  0
 24 26  1  0  0  0  0
 24 30  1  0  0  0  0
 24 34  1  0  0  0  0
 24 40  1  0  0  0  0
 25 27  1  0  0  0  0
 25 29  1  0  0  0  0
 25 35  1  0  0  0  0
 26 27  1  0  0  0  0
 26 31  1  0  0  0  0
 26 36  1  0  0  0  0
 27 37  1  0  0  0  0
 27 43  1  0  0  0  0
 38 39  1  0  0  0  0
 38 40  1  0  0  0  0
 38 49  1  0  0  0  0
 40 41  1  0  0  0  0
 40 42  1  0  0  0  0
 45 46  3  0  0  0  0
 45 50  1  0  0  0  0
 46 49  1  0  0  0  0
 47 48  3  0  0  0  0
 47 53  1  0  0  0  0
 48 49  1  0  0  0  0
 50 51  1  0  0  0  0
 50 52  1  0  0  0  0
 50 56  1  0  0  0  0
 53 54  1  0  0  0  0
 53 55  1  0  0  0  0
 53 66  1  0  0  0  0
 56 57  2  0  0  0  0
```

```
 56 58  1  0  0  0  0
 57 59  1  0  0  0  0
 57 60  1  0  0  0  0
 58 61  2  0  0  0  0
 58 62  1  0  0  0  0
 59 63  2  0  0  0  0
 59 64  1  0  0  0  0
 61 63  1  0  0  0  0
 61 65  1  0  0  0  0
 63 79  1  0  0  0  0
 66 67  2  0  0  0  0
 66 68  1  0  0  0  0
 67 69  1  0  0  0  0
 67 70  1  0  0  0  0
 68 71  2  0  0  0  0
 68 72  1  0  0  0  0
 69 73  2  0  0  0  0
 69 74  1  0  0  0  0
 71 73  1  0  0  0  0
 71 75  1  0  0  0  0
 73 76  1  0  0  0  0
 76 77  1  0  0  0  0
 76 78  1  0  0  0  0
 76 86  1  0  0  0  0
 79 80  1  0  0  0  0
 79 81  1  0  0  0  0
 79 82  1  0  0  0  0
 82 83  1  0  0  0  0
 82 84  1  0  0  0  0
 82 85  1  0  0  0  0
 86 87  1  0  0  0  0
 86 88  1  0  0  0  0
 86 89  1  0  0  0  0
M  END
$$$$
mol169
Optimized Catalyst 169
Optimized Catalyst 169
 89 94  0  0  0  0  0  0  0  0999 V2000
   -2.8669    0.6418   -0.6231 C   0  0  0  0  0  0  0  0  0  0  0  0
   -1.8456    1.0843    0.3937 C   0  0  0  0  0  0  0  0  0  0  0  0
   -0.9525    1.8772   -2.9674 C   0  0  0  0  0  0  0  0  0  0  0  0
   -1.1007    2.3935    0.0578 C   0  0  0  0  0  0  0  0  0  0  0  0
    0.2254    2.4700   -2.1782 C   0  0  0  0  0  0  0  0  0  0  0  0
    0.2441    2.2016   -0.6630 C   0  0  0  0  0  0  0  0  0  0  0  0
   -1.1027    0.2232    0.5282 H   0  0  0  0  0  0  0  0  0  0  0  0
   -1.7701    3.0441   -0.5546 H   0  0  0  0  0  0  0  0  0  0  0  0
   -1.8638    2.4989   -2.7895 H   0  0  0  0  0  0  0  0  0  0  0  0
    0.2162    3.5779   -2.3431 H   0  0  0  0  0  0  0  0  0  0  0  0
   -3.9253    0.6627   -0.2713 H   0  0  0  0  0  0  0  0  0  0  0  0
   -2.3656    1.2224    1.3788 H   0  0  0  0  0  0  0  0  0  0  0  0
   -0.7051    1.9457   -4.0568 H   0  0  0  0  0  0  0  0  0  0  0  0
   -0.9023    2.9244    1.0241 H   0  0  0  0  0  0  0  0  0  0  0  0
    1.1796    2.0691   -2.6043 H   0  0  0  0  0  0  0  0  0  0  0  0
    0.9836    2.9157   -0.2138 H   0  0  0  0  0  0  0  0  0  0  0  0
   -2.6087    0.2747   -1.9466 C   0  0  0  0  0  0  0  0  0  0  0  0
   -3.4644    0.0021   -2.6068 H   0  0  0  0  0  0  0  0  0  0  0  0
   -1.2730    0.4063   -2.6276 C   0  0  0  0  0  0  0  0  0  0  0  0
   -0.4725   -0.0115   -1.9459 H   0  0  0  0  0  0  0  0  0  0  0  0
   -1.2749   -0.2036   -3.5663 H   0  0  0  0  0  0  0  0  0  0  0  0
    0.1343   -2.4924   -1.9244 C   0  0  0  0  0  0  0  0  0  0  0  0
    1.0350   -2.9087   -3.0494 C   0  0  0  0  0  0  0  0  0  0  0  0
    2.5146   -3.2548    0.1799 C   0  0  0  0  0  0  0  0  0  0  0  0
    1.9438   -4.1131   -2.7199 C   0  0  0  0  0  0  0  0  0  0  0  0
    3.6097   -3.8152   -0.7416 C   0  0  0  0  0  0  0  0  0  0  0  0
```

```
    3.3581   -3.7237   -2.2562 C   0  0  0  0  0  0  0  0  0  0  0  0
    1.6657   -2.0301   -3.3422 H   0  0  0  0  0  0  0  0  0  0  0  0
    1.4550   -4.7444   -1.9439 H   0  0  0  0  0  0  0  0  0  0  0  0
    1.6830   -3.9957    0.2414 H   0  0  0  0  0  0  0  0  0  0  0  0
    3.7560   -4.8915   -0.4712 H   0  0  0  0  0  0  0  0  0  0  0  0
   -0.9518   -2.5650   -2.1651 H   0  0  0  0  0  0  0  0  0  0  0  0
    0.4075   -3.1797   -3.9353 H   0  0  0  0  0  0  0  0  0  0  0  0
    2.9540   -3.1496    1.2027 H   0  0  0  0  0  0  0  0  0  0  0  0
    2.0417   -4.7279   -3.6493 H   0  0  0  0  0  0  0  0  0  0  0  0
    4.5663   -3.2750   -0.5274 H   0  0  0  0  0  0  0  0  0  0  0  0
    4.0904   -4.4116   -2.7525 H   0  0  0  0  0  0  0  0  0  0  0  0
    0.5209   -2.0334   -0.7245 C   0  0  0  0  0  0  0  0  0  0  0  0
   -0.2415   -1.7448    0.0316 H   0  0  0  0  0  0  0  0  0  0  0  0
    1.9372   -1.8899   -0.2502 C   0  0  0  0  0  0  0  0  0  0  0  0
    2.5660   -1.4512   -1.0640 H   0  0  0  0  0  0  0  0  0  0  0  0
    1.9714   -1.1867    0.6169 H   0  0  0  0  0  0  0  0  0  0  0  0
    3.5960   -2.6903   -2.6029 H   0  0  0  0  0  0  0  0  0  0  0  0
    0.6306    1.1710   -0.4762 H   0  0  0  0  0  0  0  0  0  0  0  0
   -2.3767   -4.0220    0.1220 C   0  0  0  0  0  0  0  0  0  0  0  0
   -2.4014   -2.9050   -0.1984 N   0  0  0  0  0  0  0  0  0  0  0  0
   -6.0811   -1.9887   -1.0097 C   0  0  0  0  0  0  0  0  0  0  0  0
   -4.9505   -1.7362   -0.9845 N   0  0  0  0  0  0  0  0  0  0  0  0
   -2.2706   -5.4150    0.5156 C   0  0  0  0  0  0  0  0  0  0  0  0
   -2.6732   -5.5442    1.5593 H   0  0  0  0  0  0  0  0  0  0  0  0
   -2.8907   -6.0468   -0.1820 H   0  0  0  0  0  0  0  0  0  0  0  0
   -7.4969   -2.3055   -1.0354 C   0  0  0  0  0  0  0  0  0  0  0  0
   -7.7052   -3.0408   -1.8633 H   0  0  0  0  0  0  0  0  0  0  0  0
   -7.7896   -2.7925   -0.0615 H   0  0  0  0  0  0  0  0  0  0  0  0
   -8.3647   -1.0463   -1.2467 C   0  0  0  0  0  0  0  0  0  0  0  0
   -8.1508   -0.6027   -2.2506 H   0  0  0  0  0  0  0  0  0  0  0  0
   -8.1177   -0.2813   -0.4697 H   0  0  0  0  0  0  0  0  0  0  0  0
   -0.8003   -5.8859    0.4654 C   0  0  0  0  0  0  0  0  0  0  0  0
   -0.3818   -5.6985   -0.5551 H   0  0  0  0  0  0  0  0  0  0  0  0
   -0.1943   -5.3000    1.2004 H   0  0  0  0  0  0  0  0  0  0  0  0
   -9.8215   -1.4197   -1.1527 C   0  0  0  0  0  0  0  0  0  0  0  0
  -10.5098   -1.8729   -2.2879 C   0  0  0  0  0  0  0  0  0  0  0  0
  -10.4981   -1.3272    0.0718 C   0  0  0  0  0  0  0  0  0  0  0  0
  -11.8556   -2.2279   -2.1989 C   0  0  0  0  0  0  0  0  0  0  0  0
   -9.9992   -1.9461   -3.2469 H   0  0  0  0  0  0  0  0  0  0  0  0
  -11.8452   -1.6813    0.1603 C   0  0  0  0  0  0  0  0  0  0  0  0
   -9.9789   -0.9746    0.9617 H   0  0  0  0  0  0  0  0  0  0  0  0
  -12.5383   -2.1358   -0.9735 C   0  0  0  0  0  0  0  0  0  0  0  0
  -12.3830   -2.5745   -3.0873 H   0  0  0  0  0  0  0  0  0  0  0  0
  -12.3620   -1.5991    1.1160 H   0  0  0  0  0  0  0  0  0  0  0  0
   -0.7123   -7.3538    0.7899 C   0  0  0  0  0  0  0  0  0  0  0  0
   -0.5472   -7.7755    2.1180 C   0  0  0  0  0  0  0  0  0  0  0  0
   -0.7826   -8.3103   -0.2322 C   0  0  0  0  0  0  0  0  0  0  0  0
   -0.4449   -9.1336    2.4164 C   0  0  0  0  0  0  0  0  0  0  0  0
   -0.4925   -7.0442    2.9230 H   0  0  0  0  0  0  0  0  0  0  0  0
   -0.6804   -9.6703    0.0666 C   0  0  0  0  0  0  0  0  0  0  0  0
   -0.9119   -7.9991   -1.2676 H   0  0  0  0  0  0  0  0  0  0  0  0
   -0.5086  -10.0962    1.3932 C   0  0  0  0  0  0  0  0  0  0  0  0
   -0.3090   -9.4518    3.4500 H   0  0  0  0  0  0  0  0  0  0  0  0
   -0.7293  -10.4026   -0.7390 H   0  0  0  0  0  0  0  0  0  0  0  0
  -13.9861   -2.4967   -0.8902 C   0  0  0  0  0  0  0  0  0  0  0  0
  -14.5995   -1.6372   -1.2589 H   0  0  0  0  0  0  0  0  0  0  0  0
  -14.2100   -3.3896   -1.5205 H   0  0  0  0  0  0  0  0  0  0  0  0
  -14.2918   -2.7236    0.1576 H   0  0  0  0  0  0  0  0  0  0  0  0
   -0.3857  -11.5475    1.7272 C   0  0  0  0  0  0  0  0  0  0  0  0
   -0.5612  -12.1929    0.8358 H   0  0  0  0  0  0  0  0  0  0  0  0
    0.6410  -11.7571    2.1163 H   0  0  0  0  0  0  0  0  0  0  0  0
   -1.1292  -11.8252    2.5126 H   0  0  0  0  0  0  0  0  0  0  0  0
   -2.5054   -1.3428   -0.7326 Rh  0  0  0  0  0  0  0  0  0  0  0  0
  1  2  1  0  0  0  0
  1 11  1  0  0  0  0
```

1	17	2	0	0	0	0
1	89	1	0	0	0	0
2	4	1	0	0	0	0
2	12	1	0	0	0	0
2	7	1	0	0	0	0
3	5	1	0	0	0	0
3	9	1	0	0	0	0
3	13	1	0	0	0	0
3	19	1	0	0	0	0
4	6	1	0	0	0	0
4	8	1	0	0	0	0
4	14	1	0	0	0	0
5	6	1	0	0	0	0
5	10	1	0	0	0	0
5	15	1	0	0	0	0
6	16	1	0	0	0	0
6	44	1	0	0	0	0
17	18	1	0	0	0	0
17	19	1	0	0	0	0
17	89	1	0	0	0	0
19	20	1	0	0	0	0
19	21	1	0	0	0	0
22	23	1	0	0	0	0
22	32	1	0	0	0	0
22	38	2	0	0	0	0
22	89	1	0	0	0	0
23	25	1	0	0	0	0
23	28	1	0	0	0	0
23	33	1	0	0	0	0
24	26	1	0	0	0	0
24	30	1	0	0	0	0
24	34	1	0	0	0	0
24	40	1	0	0	0	0
25	27	1	0	0	0	0
25	29	1	0	0	0	0
25	35	1	0	0	0	0
26	27	1	0	0	0	0
26	31	1	0	0	0	0
26	36	1	0	0	0	0
27	37	1	0	0	0	0
27	43	1	0	0	0	0
38	39	1	0	0	0	0
38	40	1	0	0	0	0
38	89	1	0	0	0	0
40	41	1	0	0	0	0
40	42	1	0	0	0	0
45	46	3	0	0	0	0
45	49	1	0	0	0	0
46	89	1	0	0	0	0
47	48	3	0	0	0	0
47	52	1	0	0	0	0
48	89	1	0	0	0	0
49	50	1	0	0	0	0
49	51	1	0	0	0	0
49	58	1	0	0	0	0
52	53	1	0	0	0	0
52	54	1	0	0	0	0
52	55	1	0	0	0	0
55	56	1	0	0	0	0
55	57	1	0	0	0	0
55	61	1	0	0	0	0
58	59	1	0	0	0	0
58	60	1	0	0	0	0
58	71	1	0	0	0	0
61	62	2	0	0	0	0

```
 61 63  1  0  0  0  0
 62 64  1  0  0  0  0
 62 65  1  0  0  0  0
 63 66  2  0  0  0  0
 63 67  1  0  0  0  0
 64 68  2  0  0  0  0
 64 69  1  0  0  0  0
 66 68  1  0  0  0  0
 66 70  1  0  0  0  0
 68 81  1  0  0  0  0
 71 72  2  0  0  0  0
 71 73  1  0  0  0  0
 72 74  1  0  0  0  0
 72 75  1  0  0  0  0
 73 76  2  0  0  0  0
 73 77  1  0  0  0  0
 74 78  2  0  0  0  0
 74 79  1  0  0  0  0
 76 78  1  0  0  0  0
 76 80  1  0  0  0  0
 78 85  1  0  0  0  0
 81 82  1  0  0  0  0
 81 83  1  0  0  0  0
 81 84  1  0  0  0  0
 85 86  1  0  0  0  0
 85 87  1  0  0  0  0
 85 88  1  0  0  0  0
M  END
$$$$
mol170
Optimized Catalyst 170
Optimized Catalyst 170
 89 94  0  0  0  0  0  0  0  0999 V2000
   -2.6525    0.2797   -0.7516 C   0  0  0  0  0  0  0  0  0  0  0  0
   -1.7108    1.1636    0.0188 C   0  0  0  0  0  0  0  0  0  0  0  0
   -1.6092    1.6731   -3.4925 C   0  0  0  0  0  0  0  0  0  0  0  0
   -1.6528    2.5991   -0.5603 C   0  0  0  0  0  0  0  0  0  0  0  0
   -0.6970    2.7971   -2.9784 C   0  0  0  0  0  0  0  0  0  0  0  0
   -0.4363    2.8599   -1.4636 C   0  0  0  0  0  0  0  0  0  0  0  0
   -0.6804    0.7295    0.0257 H   0  0  0  0  0  0  0  0  0  0  0  0
   -2.5924    2.8170   -1.1201 H   0  0  0  0  0  0  0  0  0  0  0  0
   -2.6721    1.9455   -3.2800 H   0  0  0  0  0  0  0  0  0  0  0  0
   -1.1603    3.7673   -3.2965 H   0  0  0  0  0  0  0  0  0  0  0  0
   -3.6266    0.1131   -0.2444 H   0  0  0  0  0  0  0  0  0  0  0  0
   -2.0568    1.2321    1.0830 H   0  0  0  0  0  0  0  0  0  0  0  0
   -1.4926    1.6213   -4.6047 H   0  0  0  0  0  0  0  0  0  0  0  0
   -1.6025    3.3074    0.3057 H   0  0  0  0  0  0  0  0  0  0  0  0
    0.2963    2.7133   -3.4880 H   0  0  0  0  0  0  0  0  0  0  0  0
   -0.0603    3.8944   -1.2418 H   0  0  0  0  0  0  0  0  0  0  0  0
   -2.4633   -0.1536   -2.0283 C   0  0  0  0  0  0  0  0  0  0  0  0
   -3.2931   -0.6466   -2.5902 H   0  0  0  0  0  0  0  0  0  0  0  0
   -1.3111    0.2732   -2.9037 C   0  0  0  0  0  0  0  0  0  0  0  0
   -0.3585    0.3179   -2.3208 H   0  0  0  0  0  0  0  0  0  0  0  0
   -1.1807   -0.4526   -3.7446 H   0  0  0  0  0  0  0  0  0  0  0  0
    0.2760   -2.8458   -1.7233 C   0  0  0  0  0  0  0  0  0  0  0  0
    1.1966   -2.4695   -2.8474 C   0  0  0  0  0  0  0  0  0  0  0  0
    2.5818   -1.9489    0.3732 C   0  0  0  0  0  0  0  0  0  0  0  0
    2.6738   -2.8303   -2.5614 C   0  0  0  0  0  0  0  0  0  0  0  0
    3.7653   -1.5988   -0.5409 C   0  0  0  0  0  0  0  0  0  0  0  0
    3.5180   -1.6478   -2.0582 C   0  0  0  0  0  0  0  0  0  0  0  0
    1.1211   -1.3729   -3.0555 H   0  0  0  0  0  0  0  0  0  0  0  0
    2.7207   -3.6680   -1.8276 H   0  0  0  0  0  0  0  0  0  0  0  0
    2.4403   -3.0567    0.3740 H   0  0  0  0  0  0  0  0  0  0  0  0
    4.5947   -2.3111   -0.2950 H   0  0  0  0  0  0  0  0  0  0  0  0
   -0.3926   -3.7040   -1.9616 H   0  0  0  0  0  0  0  0  0  0  0  0
```

```
    0.8801   -3.0098   -3.7766 H   0  0  0  0  0  0  0  0  0  0  0  0
    2.8553   -1.6437    1.4148 H   0  0  0  0  0  0  0  0  0  0  0  0
    3.1219   -3.1942   -3.5208 H   0  0  0  0  0  0  0  0  0  0  0  0
    4.1185   -0.5666   -0.2897 H   0  0  0  0  0  0  0  0  0  0  0  0
    4.5244   -1.7080   -2.5511 H   0  0  0  0  0  0  0  0  0  0  0  0
    0.2639   -2.2946   -0.4893 C   0  0  0  0  0  0  0  0  0  0  0  0
   -0.3761   -2.7200    0.3338 H   0  0  0  0  0  0  0  0  0  0  0  0
    1.2473   -1.2633   -0.0032 C   0  0  0  0  0  0  0  0  0  0  0  0
    1.4363   -0.4949   -0.7921 H   0  0  0  0  0  0  0  0  0  0  0  0
    0.8448   -0.7464    0.9019 H   0  0  0  0  0  0  0  0  0  0  0  0
    3.0559   -0.6865   -2.3852 H   0  0  0  0  0  0  0  0  0  0  0  0
    0.3881    2.1554   -1.2031 H   0  0  0  0  0  0  0  0  0  0  0  0
   -2.3872   -5.4009    0.0239 C   0  0  0  0  0  0  0  0  0  0  0  0
   -2.3806   -4.2747   -0.2804 N   0  0  0  0  0  0  0  0  0  0  0  0
   -5.5446   -2.4265   -0.7379 C   0  0  0  0  0  0  0  0  0  0  0  0
   -4.3796   -2.3906   -0.7849 N   0  0  0  0  0  0  0  0  0  0  0  0
   -2.3833   -6.7992    0.3958 C   0  0  0  0  0  0  0  0  0  0  0  0
   -2.9090   -6.9285    1.3878 H   0  0  0  0  0  0  0  0  0  0  0  0
   -2.9534   -7.3963   -0.3762 H   0  0  0  0  0  0  0  0  0  0  0  0
   -6.9901   -2.4506   -0.6818 C   0  0  0  0  0  0  0  0  0  0  0  0
   -7.3845   -3.2258   -1.4033 H   0  0  0  0  0  0  0  0  0  0  0  0
   -7.3230   -2.7465    0.3569 H   0  0  0  0  0  0  0  0  0  0  0  0
   -7.6042   -1.0762   -1.0342 C   0  0  0  0  0  0  0  0  0  0  0  0
   -7.2931   -0.7742   -2.0648 H   0  0  0  0  0  0  0  0  0  0  0  0
   -7.2389   -0.3015   -0.3160 H   0  0  0  0  0  0  0  0  0  0  0  0
   -0.9489   -7.3649    0.5094 C   0  0  0  0  0  0  0  0  0  0  0  0
   -0.4184   -7.2533   -0.4683 H   0  0  0  0  0  0  0  0  0  0  0  0
   -0.3775   -6.7974    1.2851 H   0  0  0  0  0  0  0  0  0  0  0  0
   -9.1073   -1.1634   -0.9646 C   0  0  0  0  0  0  0  0  0  0  0  0
   -9.8471   -1.5239   -2.1004 C   0  0  0  0  0  0  0  0  0  0  0  0
   -9.7780   -0.8766    0.2330 C   0  0  0  0  0  0  0  0  0  0  0  0
  -11.2390   -1.5896   -2.0409 C   0  0  0  0  0  0  0  0  0  0  0  0
   -9.3413   -1.7485   -3.0382 H   0  0  0  0  0  0  0  0  0  0  0  0
  -11.1704   -0.9422    0.2922 C   0  0  0  0  0  0  0  0  0  0  0  0
   -9.2183   -0.5945    1.1235 H   0  0  0  0  0  0  0  0  0  0  0  0
  -11.9165   -1.2989   -0.8439 C   0  0  0  0  0  0  0  0  0  0  0  0
  -11.8055   -1.8603   -2.9322 H   0  0  0  0  0  0  0  0  0  0  0  0
  -11.6827   -0.7066    1.2254 H   0  0  0  0  0  0  0  0  0  0  0  0
   -1.0101   -8.8222    0.8896 C   0  0  0  0  0  0  0  0  0  0  0  0
   -1.0115   -9.2011    2.2401 C   0  0  0  0  0  0  0  0  0  0  0  0
   -1.0573   -9.8109   -0.1038 C   0  0  0  0  0  0  0  0  0  0  0  0
   -1.0524  -10.5503    2.5918 C   0  0  0  0  0  0  0  0  0  0  0  0
   -0.9736   -8.4457    3.0235 H   0  0  0  0  0  0  0  0  0  0  0  0
   -1.0981  -11.1603    0.2483 C   0  0  0  0  0  0  0  0  0  0  0  0
   -1.0555   -9.5339   -1.1568 H   0  0  0  0  0  0  0  0  0  0  0  0
   -1.0954  -11.5453    1.5998 C   0  0  0  0  0  0  0  0  0  0  0  0
   -1.0424  -10.8346    3.6443 H   0  0  0  0  0  0  0  0  0  0  0  0
   -1.1240  -11.9213   -0.5321 H   0  0  0  0  0  0  0  0  0  0  0  0
  -13.4080   -1.3480   -0.7871 C   0  0  0  0  0  0  0  0  0  0  0  0
  -13.8202   -0.3461   -1.0671 H   0  0  0  0  0  0  0  0  0  0  0  0
  -13.8141   -2.1074   -1.4960 H   0  0  0  0  0  0  0  0  0  0  0  0
  -13.7657   -1.6027    0.2382 H   0  0  0  0  0  0  0  0  0  0  0  0
   -1.1160  -12.9902    1.9772 C   0  0  0  0  0  0  0  0  0  0  0  0
   -1.6389  -13.6027    1.2056 H   0  0  0  0  0  0  0  0  0  0  0  0
   -0.0645  -13.3611    2.0707 H   0  0  0  0  0  0  0  0  0  0  0  0
   -1.6331  -13.1450    2.9534 H   0  0  0  0  0  0  0  0  0  0  0  0
   -2.4247   -2.3886   -0.8094 Ir  0  0  0  0  0  0  0  0  0  0  0  0
  1  2  1  0  0  0  0
  1 11  1  0  0  0  0
  1 17  2  0  0  0  0
  1 89  1  0  0  0  0
  2  4  1  0  0  0  0
  2  7  1  0  0  0  0
  2 12  1  0  0  0  0
  3  5  1  0  0  0  0
```

```
  3  9  1  0  0  0  0
  3 13  1  0  0  0  0
  3 19  1  0  0  0  0
  4  6  1  0  0  0  0
  4  8  1  0  0  0  0
  4 14  1  0  0  0  0
  5  6  1  0  0  0  0
  5 10  1  0  0  0  0
  5 15  1  0  0  0  0
  6 16  1  0  0  0  0
  6 44  1  0  0  0  0
 17 18  1  0  0  0  0
 17 19  1  0  0  0  0
 17 89  1  0  0  0  0
 19 20  1  0  0  0  0
 19 21  1  0  0  0  0
 22 23  1  0  0  0  0
 22 32  1  0  0  0  0
 22 38  2  0  0  0  0
 22 89  1  0  0  0  0
 23 25  1  0  0  0  0
 23 28  1  0  0  0  0
 23 33  1  0  0  0  0
 24 26  1  0  0  0  0
 24 30  1  0  0  0  0
 24 34  1  0  0  0  0
 24 40  1  0  0  0  0
 25 27  1  0  0  0  0
 25 29  1  0  0  0  0
 25 35  1  0  0  0  0
 26 27  1  0  0  0  0
 26 31  1  0  0  0  0
 26 36  1  0  0  0  0
 27 37  1  0  0  0  0
 27 43  1  0  0  0  0
 38 39  1  0  0  0  0
 38 40  1  0  0  0  0
 38 89  1  0  0  0  0
 40 41  1  0  0  0  0
 40 42  1  0  0  0  0
 45 46  3  0  0  0  0
 45 49  1  0  0  0  0
 46 89  1  0  0  0  0
 47 48  3  0  0  0  0
 47 52  1  0  0  0  0
 48 89  1  0  0  0  0
 49 50  1  0  0  0  0
 49 51  1  0  0  0  0
 49 58  1  0  0  0  0
 52 53  1  0  0  0  0
 52 54  1  0  0  0  0
 52 55  1  0  0  0  0
 55 56  1  0  0  0  0
 55 57  1  0  0  0  0
 55 61  1  0  0  0  0
 58 59  1  0  0  0  0
 58 60  1  0  0  0  0
 58 71  1  0  0  0  0
 61 62  2  0  0  0  0
 61 63  1  0  0  0  0
 62 64  1  0  0  0  0
 62 65  1  0  0  0  0
 63 66  2  0  0  0  0
 63 67  1  0  0  0  0
 64 68  2  0  0  0  0
```

```
 64 69  1  0  0  0  0
 66 68  1  0  0  0  0
 66 70  1  0  0  0  0
 68 81  1  0  0  0  0
 71 72  2  0  0  0  0
 71 73  1  0  0  0  0
 72 74  1  0  0  0  0
 72 75  1  0  0  0  0
 73 76  2  0  0  0  0
 73 77  1  0  0  0  0
 74 78  2  0  0  0  0
 74 79  1  0  0  0  0
 76 78  1  0  0  0  0
 76 80  1  0  0  0  0
 78 85  1  0  0  0  0
 81 82  1  0  0  0  0
 81 83  1  0  0  0  0
 81 84  1  0  0  0  0
 85 86  1  0  0  0  0
 85 87  1  0  0  0  0
 85 88  1  0  0  0  0
M  END
$$$$
mol171
Optimized Catalyst 171
Optimized Catalyst 171
 89 93  0  0  0  0  0  0  0  0999 V2000
   -2.6652    0.7321   -0.5881 C   0  0  0  0  0  0  0  0  0  0  0  0
   -1.6707    1.1014    0.4838 C   0  0  0  0  0  0  0  0  0  0  0  0
   -0.4843    1.6713   -2.8187 C   0  0  0  0  0  0  0  0  0  0  0  0
   -0.7487    2.2967    0.1661 C   0  0  0  0  0  0  0  0  0  0  0  0
    0.7114    2.1418   -1.9763 C   0  0  0  0  0  0  0  0  0  0  0  0
    0.6050    1.9184   -0.4577 C   0  0  0  0  0  0  0  0  0  0  0  0
   -1.0459    0.1574    0.7029 H   0  0  0  0  0  0  0  0  0  0  0  0
   -1.2924    3.0031   -0.5067 H   0  0  0  0  0  0  0  0  0  0  0  0
   -1.3278    2.3942   -2.6971 H   0  0  0  0  0  0  0  0  0  0  0  0
    0.8476    3.2370   -2.1675 H   0  0  0  0  0  0  0  0  0  0  0  0
   -3.7371    0.8590   -0.3056 H   0  0  0  0  0  0  0  0  0  0  0  0
   -2.2374    1.3389    1.4233 H   0  0  0  0  0  0  0  0  0  0  0  0
   -0.1766    1.6914   -3.8944 H   0  0  0  0  0  0  0  0  0  0  0  0
   -0.5489    2.8320    1.1295 H   0  0  0  0  0  0  0  0  0  0  0  0
    1.6317    1.6148   -2.3339 H   0  0  0  0  0  0  0  0  0  0  0  0
    1.3994    2.5475    0.0231 H   0  0  0  0  0  0  0  0  0  0  0  0
   -2.3626    0.2756   -1.8745 C   0  0  0  0  0  0  0  0  0  0  0  0
   -3.1988    0.0450   -2.5733 H   0  0  0  0  0  0  0  0  0  0  0  0
   -0.9830    0.2519   -2.4754 C   0  0  0  0  0  0  0  0  0  0  0  0
   -0.2749   -0.2360   -1.7371 H   0  0  0  0  0  0  0  0  0  0  0  0
   -0.9921   -0.3766   -3.4019 H   0  0  0  0  0  0  0  0  0  0  0  0
    0.0692   -2.8043   -1.7719 C   0  0  0  0  0  0  0  0  0  0  0  0
    0.8358   -3.3635   -2.9337 C   0  0  0  0  0  0  0  0  0  0  0  0
    2.5458   -3.6871    0.1812 C   0  0  0  0  0  0  0  0  0  0  0  0
    1.6840   -4.6132   -2.6119 C   0  0  0  0  0  0  0  0  0  0  0  0
    3.5277   -4.3518   -0.7958 C   0  0  0  0  0  0  0  0  0  0  0  0
    3.1571   -4.3096   -2.2878 C   0  0  0  0  0  0  0  0  0  0  0  0
    1.4985   -2.5541   -3.3363 H   0  0  0  0  0  0  0  0  0  0  0  0
    1.2244   -5.1720   -1.7650 H   0  0  0  0  0  0  0  0  0  0  0  0
    1.6762   -4.3693    0.3275 H   0  0  0  0  0  0  0  0  0  0  0  0
    3.6247   -5.4251   -0.4862 H   0  0  0  0  0  0  0  0  0  0  0  0
   -1.0326   -2.7624   -1.9370 H   0  0  0  0  0  0  0  0  0  0  0  0
    0.1134   -3.6381   -3.7431 H   0  0  0  0  0  0  0  0  0  0  0  0
    3.0657   -3.5843    1.1657 H   0  0  0  0  0  0  0  0  0  0  0  0
    1.6595   -5.2772   -3.5117 H   0  0  0  0  0  0  0  0  0  0  0  0
    4.5287   -3.8670   -0.6815 H   0  0  0  0  0  0  0  0  0  0  0  0
    3.7917   -5.0756   -2.8037 H   0  0  0  0  0  0  0  0  0  0  0  0
    0.5823   -2.3326   -0.6256 C   0  0  0  0  0  0  0  0  0  0  0  0
```

```
   -0.0970   -1.9273    0.1520 H   0  0  0  0  0  0  0  0  0  0  0  0
    2.0360   -2.2982   -0.2557 C   0  0  0  0  0  0  0  0  0  0  0  0
    2.6344   -1.9364   -1.1289 H   0  0  0  0  0  0  0  0  0  0  0  0
    2.1898   -1.5729    0.5796 H   0  0  0  0  0  0  0  0  0  0  0  0
    3.4377   -3.3145   -2.7068 H   0  0  0  0  0  0  0  0  0  0  0  0
    0.8452    0.8536   -0.2229 H   0  0  0  0  0  0  0  0  0  0  0  0
   -2.4317   -3.8686    0.5142 C   0  0  0  0  0  0  0  0  0  0  0  0
   -2.4265   -2.7823    0.1007 N   0  0  0  0  0  0  0  0  0  0  0  0
   -5.9524   -1.9114   -1.2223 C   0  0  0  0  0  0  0  0  0  0  0  0
   -4.8438   -1.6134   -1.0660 N   0  0  0  0  0  0  0  0  0  0  0  0
   -2.2753   -5.2295    0.9993 C   0  0  0  0  0  0  0  0  0  0  0  0
   -2.5587   -5.2861    2.0858 H   0  0  0  0  0  0  0  0  0  0  0  0
   -2.9513   -5.9148    0.4178 H   0  0  0  0  0  0  0  0  0  0  0  0
   -7.3398   -2.2949   -1.4148 C   0  0  0  0  0  0  0  0  0  0  0  0
   -7.4282   -2.8913   -2.3664 H   0  0  0  0  0  0  0  0  0  0  0  0
   -7.6586   -2.9530   -0.5578 H   0  0  0  0  0  0  0  0  0  0  0  0
   -8.2669   -1.0658   -1.4914 C   0  0  0  0  0  0  0  0  0  0  0  0
   -8.0085   -0.4561   -2.3920 H   0  0  0  0  0  0  0  0  0  0  0  0
   -8.1284   -0.4307   -0.5824 H   0  0  0  0  0  0  0  0  0  0  0  0
   -0.7996   -5.6358    0.8054 C   0  0  0  0  0  0  0  0  0  0  0  0
   -0.5009   -5.4225   -0.2531 H   0  0  0  0  0  0  0  0  0  0  0  0
   -0.1621   -5.0200    1.4883 H   0  0  0  0  0  0  0  0  0  0  0  0
   -2.4375   -1.2705   -0.5651 Rh  0  0  0  0  0  0  0  0  0  0  0  0
   -9.7407   -1.4939   -1.5829 C   0  0  0  0  0  0  0  0  0  0  0  0
  -10.0324   -2.0661   -0.6677 H   0  0  0  0  0  0  0  0  0  0  0  0
   -9.8905   -2.1649   -2.4644 H   0  0  0  0  0  0  0  0  0  0  0  0
   -0.5403   -7.1212    1.0924 C   0  0  0  0  0  0  0  0  0  0  0  0
   -1.1857   -7.7626    0.4439 H   0  0  0  0  0  0  0  0  0  0  0  0
   -0.7819   -7.3608    2.1569 H   0  0  0  0  0  0  0  0  0  0  0  0
  -10.6254   -0.2796   -1.7165 C   0  0  0  0  0  0  0  0  0  0  0  0
  -11.0814    0.3897   -0.5711 C   0  0  0  0  0  0  0  0  0  0  0  0
  -10.9972    0.1857   -2.9864 C   0  0  0  0  0  0  0  0  0  0  0  0
  -11.9098    1.5082   -0.6970 C   0  0  0  0  0  0  0  0  0  0  0  0
  -10.7984    0.0386    0.4188 H   0  0  0  0  0  0  0  0  0  0  0  0
  -11.8272    1.3039   -3.1065 C   0  0  0  0  0  0  0  0  0  0  0  0
  -10.6476   -0.3233   -3.8821 H   0  0  0  0  0  0  0  0  0  0  0  0
  -12.2854    1.9665   -1.9636 C   0  0  0  0  0  0  0  0  0  0  0  0
  -12.2672    2.0227    0.1953 H   0  0  0  0  0  0  0  0  0  0  0  0
  -12.1213    1.6581   -4.0949 H   0  0  0  0  0  0  0  0  0  0  0  0
  -12.9360    2.8353   -2.0595 H   0  0  0  0  0  0  0  0  0  0  0  0
    0.9152   -7.4145    0.8175 C   0  0  0  0  0  0  0  0  0  0  0  0
    1.3198   -7.8507   -0.4527 C   0  0  0  0  0  0  0  0  0  0  0  0
    1.8743   -7.2207    1.8228 C   0  0  0  0  0  0  0  0  0  0  0  0
    2.6703   -8.1041   -0.7083 C   0  0  0  0  0  0  0  0  0  0  0  0
    0.5855   -8.0012   -1.2415 H   0  0  0  0  0  0  0  0  0  0  0  0
    3.2234   -7.4749    1.5618 C   0  0  0  0  0  0  0  0  0  0  0  0
    1.5718   -6.8793    2.8109 H   0  0  0  0  0  0  0  0  0  0  0  0
    3.6240   -7.9192    0.2977 C   0  0  0  0  0  0  0  0  0  0  0  0
    2.9806   -8.4509   -1.6940 H   0  0  0  0  0  0  0  0  0  0  0  0
    3.9653   -7.3305    2.3477 H   0  0  0  0  0  0  0  0  0  0  0  0
    4.6751   -8.1276    0.0996 H   0  0  0  0  0  0  0  0  0  0  0  0
  1  2  1  0  0  0  0
  1 11  1  0  0  0  0
  1 17  2  0  0  0  0
  1 61  1  0  0  0  0
  2  4  1  0  0  0  0
  2 12  1  0  0  0  0
  3  5  1  0  0  0  0
  3  9  1  0  0  0  0
  3 13  1  0  0  0  0
  3 19  1  0  0  0  0
  4  6  1  0  0  0  0
  4  8  1  0  0  0  0
  4 14  1  0  0  0  0
  5  6  1  0  0  0  0
```

```
  5 10  1  0  0  0  0
  5 15  1  0  0  0  0
  6 16  1  0  0  0  0
  6 44  1  0  0  0  0
 17 18  1  0  0  0  0
 17 19  1  0  0  0  0
 17 61  1  0  0  0  0
 19 20  1  0  0  0  0
 19 21  1  0  0  0  0
 22 23  1  0  0  0  0
 22 32  1  0  0  0  0
 22 38  2  0  0  0  0
 22 61  1  0  0  0  0
 23 25  1  0  0  0  0
 23 28  1  0  0  0  0
 23 33  1  0  0  0  0
 24 26  1  0  0  0  0
 24 30  1  0  0  0  0
 24 34  1  0  0  0  0
 24 40  1  0  0  0  0
 25 27  1  0  0  0  0
 25 29  1  0  0  0  0
 25 35  1  0  0  0  0
 26 27  1  0  0  0  0
 26 31  1  0  0  0  0
 26 36  1  0  0  0  0
 27 37  1  0  0  0  0
 27 43  1  0  0  0  0
 38 39  1  0  0  0  0
 38 40  1  0  0  0  0
 38 61  1  0  0  0  0
 40 41  1  0  0  0  0
 40 42  1  0  0  0  0
 45 46  3  0  0  0  0
 45 49  1  0  0  0  0
 46 61  1  0  0  0  0
 47 48  3  0  0  0  0
 47 52  1  0  0  0  0
 48 61  1  0  0  0  0
 49 50  1  0  0  0  0
 49 51  1  0  0  0  0
 49 58  1  0  0  0  0
 52 53  1  0  0  0  0
 52 54  1  0  0  0  0
 52 55  1  0  0  0  0
 55 56  1  0  0  0  0
 55 57  1  0  0  0  0
 55 62  1  0  0  0  0
 58 59  1  0  0  0  0
 58 60  1  0  0  0  0
 58 65  1  0  0  0  0
 62 63  1  0  0  0  0
 62 64  1  0  0  0  0
 62 68  1  0  0  0  0
 65 66  1  0  0  0  0
 65 67  1  0  0  0  0
 65 79  1  0  0  0  0
 68 69  2  0  0  0  0
 68 70  1  0  0  0  0
 69 71  1  0  0  0  0
 69 72  1  0  0  0  0
 70 73  2  0  0  0  0
 70 74  1  0  0  0  0
 71 75  2  0  0  0  0
 71 76  1  0  0  0  0
```

```
 73 75  1  0  0  0  0
 73 77  1  0  0  0  0
 75 78  1  0  0  0  0
 79 80  2  0  0  0  0
 79 81  1  0  0  0  0
 80 82  1  0  0  0  0
 80 83  1  0  0  0  0
 81 84  2  0  0  0  0
 81 85  1  0  0  0  0
 82 86  2  0  0  0  0
 82 87  1  0  0  0  0
 84 86  1  0  0  0  0
 84 88  1  0  0  0  0
 86 89  1  0  0  0  0
M  END
$$$$
mol172
Optimized Catalyst 172
Optimized Catalyst 172
 89 94  0  0  0  0  0  0  0  0999 V2000
   -2.6388    0.2840   -0.7656 C   0  0  0  0  0  0  0  0  0  0  0  0
   -1.7030    1.1710    0.0087 C   0  0  0  0  0  0  0  0  0  0  0  0
   -1.5982    1.6899   -3.5010 C   0  0  0  0  0  0  0  0  0  0  0  0
   -1.6455    2.6080   -0.5671 C   0  0  0  0  0  0  0  0  0  0  0  0
   -0.6863    2.8124   -2.9835 C   0  0  0  0  0  0  0  0  0  0  0  0
   -0.4279    2.8714   -1.4681 C   0  0  0  0  0  0  0  0  0  0  0  0
   -0.6712    0.7401    0.0195 H   0  0  0  0  0  0  0  0  0  0  0  0
   -2.5848    2.8273   -1.1269 H   0  0  0  0  0  0  0  0  0  0  0  0
   -2.6611    1.9601   -3.2859 H   0  0  0  0  0  0  0  0  0  0  0  0
   -1.1485    3.7835   -3.3000 H   0  0  0  0  0  0  0  0  0  0  0  0
   -3.6129    0.1121   -0.2595 H   0  0  0  0  0  0  0  0  0  0  0  0
   -2.0542    1.2377    1.0714 H   0  0  0  0  0  0  0  0  0  0  0  0
   -1.4832    1.6427   -4.6136 H   0  0  0  0  0  0  0  0  0  0  0  0
   -1.5961    3.3141    0.3009 H   0  0  0  0  0  0  0  0  0  0  0  0
    0.3077    2.7292   -3.4918 H   0  0  0  0  0  0  0  0  0  0  0  0
   -0.0519    3.9052   -1.2432 H   0  0  0  0  0  0  0  0  0  0  0  0
   -2.4476   -0.1441   -2.0436 C   0  0  0  0  0  0  0  0  0  0  0  0
   -3.2745   -0.6416   -2.6058 H   0  0  0  0  0  0  0  0  0  0  0  0
   -1.2971    0.2881   -2.9180 C   0  0  0  0  0  0  0  0  0  0  0  0
   -0.3442    0.3324   -2.3356 H   0  0  0  0  0  0  0  0  0  0  0  0
   -1.1653   -0.4340   -3.7619 H   0  0  0  0  0  0  0  0  0  0  0  0
    0.2873   -2.8370   -1.7192 C   0  0  0  0  0  0  0  0  0  0  0  0
    1.2083   -2.4616   -2.8433 C   0  0  0  0  0  0  0  0  0  0  0  0
    2.5947   -1.9366    0.3738 C   0  0  0  0  0  0  0  0  0  0  0  0
    2.6859   -2.8235   -2.5593 C   0  0  0  0  0  0  0  0  0  0  0  0
    3.7770   -1.5853   -0.5413 C   0  0  0  0  0  0  0  0  0  0  0  0
    3.5315   -1.6409   -2.0588 C   0  0  0  0  0  0  0  0  0  0  0  0
    1.1340   -1.3648   -3.0505 H   0  0  0  0  0  0  0  0  0  0  0  0
    2.7342   -3.6611   -1.8255 H   0  0  0  0  0  0  0  0  0  0  0  0
    2.4547   -3.0447    0.3761 H   0  0  0  0  0  0  0  0  0  0  0  0
    4.6094   -2.2930   -0.2921 H   0  0  0  0  0  0  0  0  0  0  0  0
   -0.3820   -3.6949   -1.9570 H   0  0  0  0  0  0  0  0  0  0  0  0
    0.8909   -3.0012   -3.7726 H   0  0  0  0  0  0  0  0  0  0  0  0
    2.8685   -1.6298    1.4150 H   0  0  0  0  0  0  0  0  0  0  0  0
    3.1319   -3.1877   -3.5196 H   0  0  0  0  0  0  0  0  0  0  0  0
    4.1255   -0.5505   -0.2938 H   0  0  0  0  0  0  0  0  0  0  0  0
    4.5386   -1.7049   -2.5498 H   0  0  0  0  0  0  0  0  0  0  0  0
    0.2756   -2.2849   -0.4856 C   0  0  0  0  0  0  0  0  0  0  0  0
   -0.3629   -2.7101    0.3384 H   0  0  0  0  0  0  0  0  0  0  0  0
    1.2595   -1.2524   -0.0029 C   0  0  0  0  0  0  0  0  0  0  0  0
    1.4481   -0.4866   -0.7946 H   0  0  0  0  0  0  0  0  0  0  0  0
    0.8581   -0.7324    0.9008 H   0  0  0  0  0  0  0  0  0  0  0  0
    3.0717   -0.6801   -2.3906 H   0  0  0  0  0  0  0  0  0  0  0  0
    0.3960    2.1660   -1.2082 H   0  0  0  0  0  0  0  0  0  0  0  0
   -2.3788   -5.3970    0.0154 C   0  0  0  0  0  0  0  0  0  0  0  0
```

```
   -2.3725   -4.2699   -0.2853 N   0  0  0  0  0  0  0  0  0  0  0  0
   -5.5381   -2.4124   -0.7336 C   0  0  0  0  0  0  0  0  0  0  0  0
   -4.3729   -2.3799   -0.7826 N   0  0  0  0  0  0  0  0  0  0  0  0
   -2.3829   -6.7966    0.3859 C   0  0  0  0  0  0  0  0  0  0  0  0
   -2.9167   -6.9147    1.3745 H   0  0  0  0  0  0  0  0  0  0  0  0
   -2.9544   -7.3824   -0.3929 H   0  0  0  0  0  0  0  0  0  0  0  0
   -6.9843   -2.4430   -0.6727 C   0  0  0  0  0  0  0  0  0  0  0  0
   -7.3674   -3.2273   -1.3898 H   0  0  0  0  0  0  0  0  0  0  0  0
   -7.3026   -2.7367    0.3707 H   0  0  0  0  0  0  0  0  0  0  0  0
   -7.6046   -1.0754   -1.0308 C   0  0  0  0  0  0  0  0  0  0  0  0
   -7.3001   -0.7797   -2.0648 H   0  0  0  0  0  0  0  0  0  0  0  0
   -7.2456   -0.2962   -0.3144 H   0  0  0  0  0  0  0  0  0  0  0  0
   -0.9528   -7.3658    0.5059 C   0  0  0  0  0  0  0  0  0  0  0  0
   -0.4188   -7.2606   -0.4704 H   0  0  0  0  0  0  0  0  0  0  0  0
   -0.3850   -6.8029    1.2872 H   0  0  0  0  0  0  0  0  0  0  0  0
   -2.4176   -2.3827   -0.8093 Ir  0  0  0  0  0  0  0  0  0  0  0  0
   -9.1402   -1.1400   -0.9652 C   0  0  0  0  0  0  0  0  0  0  0  0
   -9.4705   -1.4289    0.0634 H   0  0  0  0  0  0  0  0  0  0  0  0
   -9.5242   -1.9125   -1.6768 H   0  0  0  0  0  0  0  0  0  0  0  0
   -0.9907   -8.8539    0.8939 C   0  0  0  0  0  0  0  0  0  0  0  0
   -1.5512   -9.4393    0.1234 H   0  0  0  0  0  0  0  0  0  0  0  0
   -1.5175   -8.9841    1.8718 H   0  0  0  0  0  0  0  0  0  0  0  0
   -9.7252    0.2049   -1.3214 C   0  0  0  0  0  0  0  0  0  0  0  0
   -9.9664    1.1564   -0.3197 C   0  0  0  0  0  0  0  0  0  0  0  0
  -10.0280    0.5098   -2.6565 C   0  0  0  0  0  0  0  0  0  0  0  0
  -10.5087    2.4008   -0.6529 C   0  0  0  0  0  0  0  0  0  0  0  0
   -9.7424    0.9293    0.7204 H   0  0  0  0  0  0  0  0  0  0  0  0
  -10.5700    1.7558   -2.9839 C   0  0  0  0  0  0  0  0  0  0  0  0
   -9.8523   -0.2224   -3.4416 H   0  0  0  0  0  0  0  0  0  0  0  0
  -10.8118    2.7030   -1.9841 C   0  0  0  0  0  0  0  0  0  0  0  0
  -10.6989    3.1372    0.1287 H   0  0  0  0  0  0  0  0  0  0  0  0
  -10.8079    1.9886   -4.0224 H   0  0  0  0  0  0  0  0  0  0  0  0
  -11.2387    3.6725   -2.2412 H   0  0  0  0  0  0  0  0  0  0  0  0
    0.4160   -9.3890    1.0060 C   0  0  0  0  0  0  0  0  0  0  0  0
    1.0490   -9.9462   -0.1145 C   0  0  0  0  0  0  0  0  0  0  0  0
    1.0975   -9.3276    2.2303 C   0  0  0  0  0  0  0  0  0  0  0  0
    2.3523  -10.4399   -0.0080 C   0  0  0  0  0  0  0  0  0  0  0  0
    0.5284  -10.0059   -1.0677 H   0  0  0  0  0  0  0  0  0  0  0  0
    2.4007   -9.8227    2.3309 C   0  0  0  0  0  0  0  0  0  0  0  0
    0.6143   -8.9042    3.1082 H   0  0  0  0  0  0  0  0  0  0  0  0
    3.0303  -10.3796    1.2135 C   0  0  0  0  0  0  0  0  0  0  0  0
    2.8406  -10.8761   -0.8799 H   0  0  0  0  0  0  0  0  0  0  0  0
    2.9268   -9.7770    3.2851 H   0  0  0  0  0  0  0  0  0  0  0  0
    4.0452  -10.7690    1.2953 H   0  0  0  0  0  0  0  0  0  0  0  0
  1  2  1  0  0  0  0
  1 11  1  0  0  0  0
  1 17  2  0  0  0  0
  1 61  1  0  0  0  0
  2  4  1  0  0  0  0
  2  7  1  0  0  0  0
  2 12  1  0  0  0  0
  3  5  1  0  0  0  0
  3  9  1  0  0  0  0
  3 13  1  0  0  0  0
  3 19  1  0  0  0  0
  4  6  1  0  0  0  0
  4  8  1  0  0  0  0
  4 14  1  0  0  0  0
  5  6  1  0  0  0  0
  5 10  1  0  0  0  0
  5 15  1  0  0  0  0
  6 16  1  0  0  0  0
  6 44  1  0  0  0  0
 17 18  1  0  0  0  0
 17 19  1  0  0  0  0
```

```
 17 61  1  0  0  0  0
 19 20  1  0  0  0  0
 19 21  1  0  0  0  0
 22 23  1  0  0  0  0
 22 32  1  0  0  0  0
 22 38  2  0  0  0  0
 22 61  1  0  0  0  0
 23 25  1  0  0  0  0
 23 28  1  0  0  0  0
 23 33  1  0  0  0  0
 24 26  1  0  0  0  0
 24 30  1  0  0  0  0
 24 34  1  0  0  0  0
 24 40  1  0  0  0  0
 25 27  1  0  0  0  0
 25 29  1  0  0  0  0
 25 35  1  0  0  0  0
 26 27  1  0  0  0  0
 26 31  1  0  0  0  0
 26 36  1  0  0  0  0
 27 37  1  0  0  0  0
 27 43  1  0  0  0  0
 38 39  1  0  0  0  0
 38 40  1  0  0  0  0
 38 61  1  0  0  0  0
 40 41  1  0  0  0  0
 40 42  1  0  0  0  0
 45 46  3  0  0  0  0
 45 49  1  0  0  0  0
 46 61  1  0  0  0  0
 47 48  3  0  0  0  0
 47 52  1  0  0  0  0
 48 61  1  0  0  0  0
 49 50  1  0  0  0  0
 49 51  1  0  0  0  0
 49 58  1  0  0  0  0
 52 53  1  0  0  0  0
 52 54  1  0  0  0  0
 52 55  1  0  0  0  0
 55 56  1  0  0  0  0
 55 57  1  0  0  0  0
 55 62  1  0  0  0  0
 58 59  1  0  0  0  0
 58 60  1  0  0  0  0
 58 65  1  0  0  0  0
 62 63  1  0  0  0  0
 62 64  1  0  0  0  0
 62 68  1  0  0  0  0
 65 66  1  0  0  0  0
 65 67  1  0  0  0  0
 65 79  1  0  0  0  0
 68 69  2  0  0  0  0
 68 70  1  0  0  0  0
 69 71  1  0  0  0  0
 69 72  1  0  0  0  0
 70 73  2  0  0  0  0
 70 74  1  0  0  0  0
 71 75  2  0  0  0  0
 71 76  1  0  0  0  0
 73 75  1  0  0  0  0
 73 77  1  0  0  0  0
 75 78  1  0  0  0  0
 79 80  2  0  0  0  0
 79 81  1  0  0  0  0
 80 82  1  0  0  0  0
```

```
 80 83  1  0  0  0  0
 81 84  2  0  0  0  0
 81 85  1  0  0  0  0
 82 86  2  0  0  0  0
 82 87  1  0  0  0  0
 84 86  1  0  0  0  0
 84 88  1  0  0  0  0
 86 89  1  0  0  0  0
M  END
$$$$
mol173
Optimized Catalyst 173
Optimized Catalyst 173
 71 76  0  0  0  0  0  0  0  0999 V2000
   -1.0038    0.3244   -1.5694 C   0  0  0  0  0  0  0  0  0  0  0  0
    0.1627   -0.6107   -1.1883 C   0  0  0  0  0  0  0  0  0  0  0  0
   -2.2570    0.1068   -0.7547 C   0  0  0  0  0  0  0  0  0  0  0  0
   -1.1304    0.1424    1.5479 C   0  0  0  0  0  0  0  0  0  0  0  0
   -2.3214    0.0015    0.6287 C   0  0  0  0  0  0  0  0  0  0  0  0
   -0.4540   -1.2146    1.8362 C   0  0  0  0  0  0  0  0  0  0  0  0
   -3.1921    0.2338   -1.3332 H   0  0  0  0  0  0  0  0  0  0  0  0
   -3.3037    0.0005    1.1359 H   0  0  0  0  0  0  0  0  0  0  0  0
   -1.2523    0.1556   -2.6508 H   0  0  0  0  0  0  0  0  0  0  0  0
   -0.6819    1.3935   -1.4491 H   0  0  0  0  0  0  0  0  0  0  0  0
    0.7894   -0.7919   -2.1010 H   0  0  0  0  0  0  0  0  0  0  0  0
   -0.9538   -1.6709    2.7329 H   0  0  0  0  0  0  0  0  0  0  0  0
   -1.4693    0.5867    2.5208 H   0  0  0  0  0  0  0  0  0  0  0  0
   -0.3951    0.8572    1.0971 H   0  0  0  0  0  0  0  0  0  0  0  0
   -0.5419   -2.2082    0.6984 C   0  0  0  0  0  0  0  0  0  0  0  0
   -0.3135   -1.9407   -0.6501 C   0  0  0  0  0  0  0  0  0  0  0  0
   -0.6481   -3.2680    1.0290 H   0  0  0  0  0  0  0  0  0  0  0  0
   -0.2884   -2.7810   -1.3794 H   0  0  0  0  0  0  0  0  0  0  0  0
    0.8221   -0.1201   -0.4271 H   0  0  0  0  0  0  0  0  0  0  0  0
    0.6282   -1.0501    2.0830 H   0  0  0  0  0  0  0  0  0  0  0  0
   -4.0060   -2.0423   -0.4629 N   0  0  0  0  0  0  0  0  0  0  0  0
   -5.1430   -2.2231   -0.6195 C   0  0  0  0  0  0  0  0  0  0  0  0
   -2.3832   -4.4518   -0.6827 N   0  0  0  0  0  0  0  0  0  0  0  0
   -2.4630   -5.5855   -0.9097 C   0  0  0  0  0  0  0  0  0  0  0  0
   -6.5699   -2.4068   -0.8109 C   0  0  0  0  0  0  0  0  0  0  0  0
   -6.8015   -2.3740   -1.9136 H   0  0  0  0  0  0  0  0  0  0  0  0
   -6.8669   -3.4217   -0.4205 H   0  0  0  0  0  0  0  0  0  0  0  0
   -2.5586   -7.0069   -1.1890 C   0  0  0  0  0  0  0  0  0  0  0  0
   -3.6291   -7.3412   -1.0791 H   0  0  0  0  0  0  0  0  0  0  0  0
   -1.9417   -7.5791   -0.4390 H   0  0  0  0  0  0  0  0  0  0  0  0
   -7.3898   -1.3204   -0.0829 C   0  0  0  0  0  0  0  0  0  0  0  0
   -7.1438   -1.3276    1.0078 H   0  0  0  0  0  0  0  0  0  0  0  0
   -7.1299   -0.3123   -0.4898 H   0  0  0  0  0  0  0  0  0  0  0  0
   -2.0656   -7.3418   -2.6125 C   0  0  0  0  0  0  0  0  0  0  0  0
   -0.9932   -7.0430   -2.7193 H   0  0  0  0  0  0  0  0  0  0  0  0
   -2.6608   -6.7651   -3.3632 H   0  0  0  0  0  0  0  0  0  0  0  0
   -2.2097   -8.8183   -2.8778 C   0  0  0  0  0  0  0  0  0  0  0  0
   -3.3924   -9.3236   -3.4370 C   0  0  0  0  0  0  0  0  0  0  0  0
   -1.1587   -9.6971   -2.5785 C   0  0  0  0  0  0  0  0  0  0  0  0
   -3.5170  -10.6878   -3.7038 C   0  0  0  0  0  0  0  0  0  0  0  0
   -4.2177   -8.6547   -3.6739 H   0  0  0  0  0  0  0  0  0  0  0  0
   -1.2847  -11.0611   -2.8464 C   0  0  0  0  0  0  0  0  0  0  0  0
   -0.2350   -9.3206   -2.1423 H   0  0  0  0  0  0  0  0  0  0  0  0
   -2.4637  -11.5698   -3.4142 C   0  0  0  0  0  0  0  0  0  0  0  0
   -4.4354  -11.0700   -4.1490 H   0  0  0  0  0  0  0  0  0  0  0  0
   -0.4578  -11.7346   -2.6214 H   0  0  0  0  0  0  0  0  0  0  0  0
   -8.8614   -1.5831   -0.2736 C   0  0  0  0  0  0  0  0  0  0  0  0
   -9.5610   -2.3805    0.6437 C   0  0  0  0  0  0  0  0  0  0  0  0
   -9.5420   -1.0320   -1.3687 C   0  0  0  0  0  0  0  0  0  0  0  0
  -10.9257   -2.6158    0.4721 C   0  0  0  0  0  0  0  0  0  0  0  0
   -9.0461   -2.8147    1.4989 H   0  0  0  0  0  0  0  0  0  0  0  0
```

```
 -10.9070   -1.2680   -1.5385 C   0  0  0  0  0  0  0  0  0  0  0  0
  -9.0125   -0.4109   -2.0891 H   0  0  0  0  0  0  0  0  0  0  0  0
 -11.6131   -2.0596   -0.6186 C   0  0  0  0  0  0  0  0  0  0  0  0
 -11.4636   -3.2288    1.1953 H   0  0  0  0  0  0  0  0  0  0  0  0
 -11.4300   -0.8273   -2.3872 H   0  0  0  0  0  0  0  0  0  0  0  0
 -13.0913   -2.2902   -0.7862 C   0  0  0  0  0  0  0  0  0  0  0  0
 -13.3665   -3.2993   -0.3898 H   0  0  0  0  0  0  0  0  0  0  0  0
 -13.3606   -2.2666   -1.8717 H   0  0  0  0  0  0  0  0  0  0  0  0
  -2.5892  -13.0372   -3.7270 C   0  0  0  0  0  0  0  0  0  0  0  0
  -3.6539  -13.3604   -3.6119 H   0  0  0  0  0  0  0  0  0  0  0  0
  -1.9736  -13.6341   -3.0086 H   0  0  0  0  0  0  0  0  0  0  0  0
  -2.3431   -2.0057   -0.2429 Rh  0  0  0  0  0  0  0  0  0  0  0  0
 -13.8919   -1.2161   -0.0417 C   0  0  0  0  0  0  0  0  0  0  0  0
 -14.9823   -1.3941   -0.1794 H   0  0  0  0  0  0  0  0  0  0  0  0
 -13.6557   -1.2440    1.0461 H   0  0  0  0  0  0  0  0  0  0  0  0
 -13.6381   -0.2056   -0.4346 H   0  0  0  0  0  0  0  0  0  0  0  0
  -2.1220  -13.3275   -5.1575 C   0  0  0  0  0  0  0  0  0  0  0  0
  -1.0604  -13.0170   -5.2859 H   0  0  0  0  0  0  0  0  0  0  0  0
  -2.2080  -14.4172   -5.3689 H   0  0  0  0  0  0  0  0  0  0  0  0
  -2.7471  -12.7669   -5.8886 H   0  0  0  0  0  0  0  0  0  0  0  0
  1  2  1  0  0  0  0
  1  3  1  0  0  0  0
  1  9  1  0  0  0  0
  1 10  1  0  0  0  0
  2 11  1  0  0  0  0
  2 16  1  0  0  0  0
  2 19  1  0  0  0  0
  3  5  2  0  0  0  0
  3  7  1  0  0  0  0
  3 63  1  0  0  0  0
  4  5  1  0  0  0  0
  4  6  1  0  0  0  0
  4 13  1  0  0  0  0
  4 14  1  0  0  0  0
  5  8  1  0  0  0  0
  5 63  1  0  0  0  0
  6 12  1  0  0  0  0
  6 15  1  0  0  0  0
  6 20  1  0  0  0  0
 15 16  2  0  0  0  0
 15 17  1  0  0  0  0
 15 63  1  0  0  0  0
 16 18  1  0  0  0  0
 16 63  1  0  0  0  0
 21 22  3  0  0  0  0
 21 63  1  0  0  0  0
 22 25  1  0  0  0  0
 23 24  3  0  0  0  0
 23 63  1  0  0  0  0
 24 28  1  0  0  0  0
 25 26  1  0  0  0  0
 25 27  1  0  0  0  0
 25 31  1  0  0  0  0
 28 29  1  0  0  0  0
 28 30  1  0  0  0  0
 28 34  1  0  0  0  0
 31 32  1  0  0  0  0
 31 33  1  0  0  0  0
 31 47  1  0  0  0  0
 34 35  1  0  0  0  0
 34 36  1  0  0  0  0
 34 37  1  0  0  0  0
 37 38  2  0  0  0  0
 37 39  1  0  0  0  0
 38 40  1  0  0  0  0
```

```
 38 41  1  0  0  0  0
 39 42  2  0  0  0  0
 39 43  1  0  0  0  0
 40 44  2  0  0  0  0
 40 45  1  0  0  0  0
 42 44  1  0  0  0  0
 42 46  1  0  0  0  0
 44 60  1  0  0  0  0
 47 48  2  0  0  0  0
 47 49  1  0  0  0  0
 48 50  1  0  0  0  0
 48 51  1  0  0  0  0
 49 52  2  0  0  0  0
 49 53  1  0  0  0  0
 50 54  2  0  0  0  0
 50 55  1  0  0  0  0
 52 54  1  0  0  0  0
 52 56  1  0  0  0  0
 54 57  1  0  0  0  0
 57 58  1  0  0  0  0
 57 59  1  0  0  0  0
 57 64  1  0  0  0  0
 60 61  1  0  0  0  0
 60 62  1  0  0  0  0
 60 68  1  0  0  0  0
 64 65  1  0  0  0  0
 64 66  1  0  0  0  0
 64 67  1  0  0  0  0
 68 69  1  0  0  0  0
 68 70  1  0  0  0  0
 68 71  1  0  0  0  0
M  END
$$$$
mol174
Optimized Catalyst 174
Optimized Catalyst 174
 71 76  0  0  0  0  0  0  0  0999 V2000
   -0.8410    0.2254   -1.5382 C   0  0  0  0  0  0  0  0  0  0  0  0
    0.3822   -0.6443   -1.1789 C   0  0  0  0  0  0  0  0  0  0  0  0
   -2.1023   -0.0422   -0.7481 C   0  0  0  0  0  0  0  0  0  0  0  0
   -0.9977   -0.0494    1.5517 C   0  0  0  0  0  0  0  0  0  0  0  0
   -2.1782   -0.1864    0.6177 C   0  0  0  0  0  0  0  0  0  0  0  0
   -0.2090   -1.3543    1.7907 C   0  0  0  0  0  0  0  0  0  0  0  0
   -3.0334    0.1334   -1.3336 H   0  0  0  0  0  0  0  0  0  0  0  0
   -3.1678   -0.1739    1.1235 H   0  0  0  0  0  0  0  0  0  0  0  0
   -1.0591    0.0994   -2.6345 H   0  0  0  0  0  0  0  0  0  0  0  0
   -0.5715    1.3104   -1.3801 H   0  0  0  0  0  0  0  0  0  0  0  0
    1.0248   -0.7383   -2.0982 H   0  0  0  0  0  0  0  0  0  0  0  0
   -0.6279   -1.8629    2.7025 H   0  0  0  0  0  0  0  0  0  0  0  0
   -1.3661    0.3225    2.5481 H   0  0  0  0  0  0  0  0  0  0  0  0
   -0.3128    0.7462    1.1493 H   0  0  0  0  0  0  0  0  0  0  0  0
   -0.1887   -2.3385    0.6427 C   0  0  0  0  0  0  0  0  0  0  0  0
    0.0492   -2.0307   -0.6768 C   0  0  0  0  0  0  0  0  0  0  0  0
   -0.1356   -3.4000    0.9763 H   0  0  0  0  0  0  0  0  0  0  0  0
    0.2448   -2.8426   -1.4102 H   0  0  0  0  0  0  0  0  0  0  0  0
    1.0100   -0.1238   -0.4050 H   0  0  0  0  0  0  0  0  0  0  0  0
    0.8631   -1.0898    2.0254 H   0  0  0  0  0  0  0  0  0  0  0  0
   -4.2681   -2.3437   -0.6235 N   0  0  0  0  0  0  0  0  0  0  0  0
   -5.4246   -2.4099   -0.7621 C   0  0  0  0  0  0  0  0  0  0  0  0
   -2.2673   -4.3154   -0.6932 N   0  0  0  0  0  0  0  0  0  0  0  0
   -2.3108   -5.4627   -0.9001 C   0  0  0  0  0  0  0  0  0  0  0  0
   -6.8588   -2.4896   -0.9338 C   0  0  0  0  0  0  0  0  0  0  0  0
   -7.1142   -2.4020   -2.0312 H   0  0  0  0  0  0  0  0  0  0  0  0
   -7.2221   -3.4994   -0.5776 H   0  0  0  0  0  0  0  0  0  0  0  0
   -2.3498   -6.8853   -1.1625 C   0  0  0  0  0  0  0  0  0  0  0  0
```

```
   -3.3935   -7.2765   -0.9732 H   0  0  0  0  0  0  0  0  0  0  0  0
   -1.6517   -7.4243   -0.4561 H   0  0  0  0  0  0  0  0  0  0  0  0
   -7.6133   -1.3881   -0.1543 C   0  0  0  0  0  0  0  0  0  0  0  0
   -7.3372   -1.4288    0.9283 H   0  0  0  0  0  0  0  0  0  0  0  0
   -7.3345   -0.3807   -0.5510 H   0  0  0  0  0  0  0  0  0  0  0  0
   -1.9478   -7.2122   -2.6193 C   0  0  0  0  0  0  0  0  0  0  0  0
   -0.8809   -6.9253   -2.7930 H   0  0  0  0  0  0  0  0  0  0  0  0
   -2.5862   -6.6313   -3.3296 H   0  0  0  0  0  0  0  0  0  0  0  0
   -2.1227   -8.6880   -2.8706 C   0  0  0  0  0  0  0  0  0  0  0  0
   -3.3231   -9.1746   -3.4083 C   0  0  0  0  0  0  0  0  0  0  0  0
   -1.0839   -9.5829   -2.5759 C   0  0  0  0  0  0  0  0  0  0  0  0
   -3.4808  -10.5397   -3.6501 C   0  0  0  0  0  0  0  0  0  0  0  0
   -4.1361   -8.4923   -3.6495 H   0  0  0  0  0  0  0  0  0  0  0  0
   -1.2443  -10.9477   -2.8174 C   0  0  0  0  0  0  0  0  0  0  0  0
   -0.1430   -9.2195   -2.1656 H   0  0  0  0  0  0  0  0  0  0  0  0
   -2.4443  -11.4405   -3.3555 C   0  0  0  0  0  0  0  0  0  0  0  0
   -4.4124  -10.9087   -4.0800 H   0  0  0  0  0  0  0  0  0  0  0  0
   -0.4278  -11.6357   -2.5953 H   0  0  0  0  0  0  0  0  0  0  0  0
   -9.0972   -1.6061   -0.3109 C   0  0  0  0  0  0  0  0  0  0  0  0
   -9.7978   -2.3888    0.6181 C   0  0  0  0  0  0  0  0  0  0  0  0
   -9.7861   -1.0334   -1.3898 C   0  0  0  0  0  0  0  0  0  0  0  0
  -11.1694   -2.5970    0.4683 C   0  0  0  0  0  0  0  0  0  0  0  0
   -9.2800   -2.8330    1.4662 H   0  0  0  0  0  0  0  0  0  0  0  0
  -11.1577   -1.2431   -1.5379 C   0  0  0  0  0  0  0  0  0  0  0  0
   -9.2589   -0.4160   -2.1148 H   0  0  0  0  0  0  0  0  0  0  0  0
  -11.8633   -2.0290   -0.6124 C   0  0  0  0  0  0  0  0  0  0  0  0
  -11.7077   -3.1986    1.2012 H   0  0  0  0  0  0  0  0  0  0  0  0
  -11.6869   -0.7860   -2.3746 H   0  0  0  0  0  0  0  0  0  0  0  0
  -13.3455   -2.2419   -0.7640 C   0  0  0  0  0  0  0  0  0  0  0  0
  -13.6297   -3.2466   -0.3620 H   0  0  0  0  0  0  0  0  0  0  0  0
  -13.6265   -2.2178   -1.8468 H   0  0  0  0  0  0  0  0  0  0  0  0
   -2.6099  -12.9108   -3.6316 C   0  0  0  0  0  0  0  0  0  0  0  0
   -3.6828  -13.2036   -3.5095 H   0  0  0  0  0  0  0  0  0  0  0  0
   -2.0108  -13.5069   -2.8984 H   0  0  0  0  0  0  0  0  0  0  0  0
   -2.3144   -2.3703   -0.3849 Ir  0  0  0  0  0  0  0  0  0  0  0  0
  -14.1252   -1.1565   -0.0143 C   0  0  0  0  0  0  0  0  0  0  0  0
  -15.2191   -1.3269   -0.1350 H   0  0  0  0  0  0  0  0  0  0  0  0
  -13.8737   -1.1799    1.0701 H   0  0  0  0  0  0  0  0  0  0  0  0
  -13.8700   -0.1505   -0.4177 H   0  0  0  0  0  0  0  0  0  0  0  0
   -2.1509  -13.2482   -5.0539 C   0  0  0  0  0  0  0  0  0  0  0  0
   -1.0794  -12.9768   -5.1880 H   0  0  0  0  0  0  0  0  0  0  0  0
   -2.2723  -14.3394   -5.2405 H   0  0  0  0  0  0  0  0  0  0  0  0
   -2.7571  -12.6854   -5.7990 H   0  0  0  0  0  0  0  0  0  0  0  0
  1  2  1  0  0  0  0
  1  3  1  0  0  0  0
  1  9  1  0  0  0  0
  1 10  1  0  0  0  0
  2 11  1  0  0  0  0
  2 16  1  0  0  0  0
  2 19  1  0  0  0  0
  3  5  2  0  0  0  0
  3  7  1  0  0  0  0
  3 63  1  0  0  0  0
  4  5  1  0  0  0  0
  4  6  1  0  0  0  0
  4 13  1  0  0  0  0
  4 14  1  0  0  0  0
  5  8  1  0  0  0  0
  5 63  1  0  0  0  0
  6 12  1  0  0  0  0
  6 15  1  0  0  0  0
  6 20  1  0  0  0  0
 15 16  2  0  0  0  0
 15 17  1  0  0  0  0
 15 63  1  0  0  0  0
```

```
 16 18  1  0  0  0  0
 16 63  1  0  0  0  0
 21 22  3  0  0  0  0
 21 63  1  0  0  0  0
 22 25  1  0  0  0  0
 23 24  3  0  0  0  0
 23 63  1  0  0  0  0
 24 28  1  0  0  0  0
 25 26  1  0  0  0  0
 25 27  1  0  0  0  0
 25 31  1  0  0  0  0
 28 29  1  0  0  0  0
 28 30  1  0  0  0  0
 28 34  1  0  0  0  0
 31 32  1  0  0  0  0
 31 33  1  0  0  0  0
 31 47  1  0  0  0  0
 34 35  1  0  0  0  0
 34 36  1  0  0  0  0
 34 37  1  0  0  0  0
 37 38  2  0  0  0  0
 37 39  1  0  0  0  0
 38 40  1  0  0  0  0
 38 41  1  0  0  0  0
 39 42  2  0  0  0  0
 39 43  1  0  0  0  0
 40 44  2  0  0  0  0
 40 45  1  0  0  0  0
 42 44  1  0  0  0  0
 42 46  1  0  0  0  0
 44 60  1  0  0  0  0
 47 48  2  0  0  0  0
 47 49  1  0  0  0  0
 48 50  1  0  0  0  0
 48 51  1  0  0  0  0
 49 52  2  0  0  0  0
 49 53  1  0  0  0  0
 50 54  2  0  0  0  0
 50 55  1  0  0  0  0
 52 54  1  0  0  0  0
 52 56  1  0  0  0  0
 54 57  1  0  0  0  0
 57 58  1  0  0  0  0
 57 59  1  0  0  0  0
 57 64  1  0  0  0  0
 60 61  1  0  0  0  0
 60 62  1  0  0  0  0
 60 68  1  0  0  0  0
 64 65  1  0  0  0  0
 64 66  1  0  0  0  0
 64 67  1  0  0  0  0
 68 69  1  0  0  0  0
 68 70  1  0  0  0  0
 68 71  1  0  0  0  0
M  END
$$$$
mol175
Optimized Catalyst 175
Optimized Catalyst 175
 77 82  0  0  0  0  0  0  0  0999 V2000
   -1.0145    0.3205   -1.5706 C   0  0  0  0  0  0  0  0  0  0  0  0
    0.1559   -0.6082   -1.1864 C   0  0  0  0  0  0  0  0  0  0  0  0
   -2.2650    0.1040   -0.7515 C   0  0  0  0  0  0  0  0  0  0  0  0
   -1.1325    0.1396    1.5482 C   0  0  0  0  0  0  0  0  0  0  0  0
   -2.3257   -0.0014    0.6320 C   0  0  0  0  0  0  0  0  0  0  0  0
```

```
   -0.4601   -1.2188    1.8384 C   0  0  0  0  0  0  0  0  0  0  0  0
   -3.2019    0.2305   -1.3273 H   0  0  0  0  0  0  0  0  0  0  0  0
   -3.3065   -0.0047    1.1419 H   0  0  0  0  0  0  0  0  0  0  0  0
   -1.2651    0.1432   -2.6502 H   0  0  0  0  0  0  0  0  0  0  0  0
   -0.6966    1.3916   -1.4582 H   0  0  0  0  0  0  0  0  0  0  0  0
    0.7862   -0.7865   -2.0972 H   0  0  0  0  0  0  0  0  0  0  0  0
   -0.9668   -1.6760    2.7310 H   0  0  0  0  0  0  0  0  0  0  0  0
   -1.4675    0.5884    2.5202 H   0  0  0  0  0  0  0  0  0  0  0  0
   -0.3964    0.8510    1.0933 H   0  0  0  0  0  0  0  0  0  0  0  0
   -0.5433   -2.2103    0.6984 C   0  0  0  0  0  0  0  0  0  0  0  0
   -0.3162   -1.9401   -0.6496 C   0  0  0  0  0  0  0  0  0  0  0  0
   -0.6457   -3.2711    1.0266 H   0  0  0  0  0  0  0  0  0  0  0  0
   -0.2890   -2.7793   -1.3796 H   0  0  0  0  0  0  0  0  0  0  0  0
    0.8110   -0.1146   -0.4236 H   0  0  0  0  0  0  0  0  0  0  0  0
    0.6209   -1.0570    2.0915 H   0  0  0  0  0  0  0  0  0  0  0  0
   -4.0112   -2.0431   -0.4560 N   0  0  0  0  0  0  0  0  0  0  0  0
   -5.1498   -2.2172   -0.6079 C   0  0  0  0  0  0  0  0  0  0  0  0
   -2.3872   -4.4371   -0.6703 N   0  0  0  0  0  0  0  0  0  0  0  0
   -2.4740   -5.5686   -0.9050 C   0  0  0  0  0  0  0  0  0  0  0  0
   -6.5777   -2.3963   -0.7948 C   0  0  0  0  0  0  0  0  0  0  0  0
   -6.8126   -2.3685   -1.8969 H   0  0  0  0  0  0  0  0  0  0  0  0
   -6.8778   -3.4080   -0.3981 H   0  0  0  0  0  0  0  0  0  0  0  0
   -2.5763   -6.9865   -1.1971 C   0  0  0  0  0  0  0  0  0  0  0  0
   -3.6466   -7.3204   -1.0857 H   0  0  0  0  0  0  0  0  0  0  0  0
   -1.9571   -7.5684   -0.4565 H   0  0  0  0  0  0  0  0  0  0  0  0
   -7.3953   -1.3047   -0.0719 C   0  0  0  0  0  0  0  0  0  0  0  0
   -7.1506   -1.3082    1.0190 H   0  0  0  0  0  0  0  0  0  0  0  0
   -7.1334   -0.2991   -0.4833 H   0  0  0  0  0  0  0  0  0  0  0  0
   -2.0927   -7.3086   -2.6271 C   0  0  0  0  0  0  0  0  0  0  0  0
   -1.0266   -6.9930   -2.7456 H   0  0  0  0  0  0  0  0  0  0  0  0
   -2.7068   -6.7404   -3.3685 H   0  0  0  0  0  0  0  0  0  0  0  0
   -2.2142   -8.7879   -2.8866 C   0  0  0  0  0  0  0  0  0  0  0  0
   -3.3619   -9.3069   -3.5023 C   0  0  0  0  0  0  0  0  0  0  0  0
   -1.1789   -9.6569   -2.5127 C   0  0  0  0  0  0  0  0  0  0  0  0
   -3.4681  -10.6762   -3.7503 C   0  0  0  0  0  0  0  0  0  0  0  0
   -4.1746   -8.6456   -3.7971 H   0  0  0  0  0  0  0  0  0  0  0  0
   -1.2891  -11.0265   -2.7549 C   0  0  0  0  0  0  0  0  0  0  0  0
   -0.2798   -9.2679   -2.0378 H   0  0  0  0  0  0  0  0  0  0  0  0
   -2.4314  -11.5494   -3.3830 C   0  0  0  0  0  0  0  0  0  0  0  0
   -4.3599  -11.0679   -4.2395 H   0  0  0  0  0  0  0  0  0  0  0  0
   -0.4773  -11.6931   -2.4628 H   0  0  0  0  0  0  0  0  0  0  0  0
   -8.8668   -1.5681   -0.2645 C   0  0  0  0  0  0  0  0  0  0  0  0
   -9.5663   -2.3688    0.6501 C   0  0  0  0  0  0  0  0  0  0  0  0
   -9.5459   -1.0194   -1.3617 C   0  0  0  0  0  0  0  0  0  0  0  0
  -10.9282   -2.6142    0.4709 C   0  0  0  0  0  0  0  0  0  0  0  0
   -9.0535   -2.7985    1.5089 H   0  0  0  0  0  0  0  0  0  0  0  0
  -10.9082   -1.2652   -1.5392 C   0  0  0  0  0  0  0  0  0  0  0  0
   -9.0174   -0.3932   -2.0784 H   0  0  0  0  0  0  0  0  0  0  0  0
  -11.6128   -2.0654   -0.6255 C   0  0  0  0  0  0  0  0  0  0  0  0
  -11.4657   -3.2295    1.1929 H   0  0  0  0  0  0  0  0  0  0  0  0
  -11.4298   -0.8249   -2.3895 H   0  0  0  0  0  0  0  0  0  0  0  0
  -13.0854   -2.3108   -0.8040 C   0  0  0  0  0  0  0  0  0  0  0  0
  -13.3572   -3.3202   -0.4091 H   0  0  0  0  0  0  0  0  0  0  0  0
  -13.3505   -2.2909   -1.8894 H   0  0  0  0  0  0  0  0  0  0  0  0
   -2.5349  -13.0204   -3.6768 C   0  0  0  0  0  0  0  0  0  0  0  0
   -3.5878  -13.3606   -3.5135 H   0  0  0  0  0  0  0  0  0  0  0  0
   -1.8790  -13.5983   -2.9804 H   0  0  0  0  0  0  0  0  0  0  0  0
   -2.3476   -2.0087   -0.2398 Rh  0  0  0  0  0  0  0  0  0  0  0  0
  -13.8992   -1.2340   -0.0595 C   0  0  0  0  0  0  0  0  0  0  0  0
  -13.6380   -1.2687    1.0261 H   0  0  0  0  0  0  0  0  0  0  0  0
  -13.6183   -0.2287   -0.4575 H   0  0  0  0  0  0  0  0  0  0  0  0
   -2.1288  -13.3390   -5.1300 C   0  0  0  0  0  0  0  0  0  0  0  0
   -2.3058  -14.4277   -5.3104 H   0  0  0  0  0  0  0  0  0  0  0  0
   -2.7766  -12.7540   -5.8267 H   0  0  0  0  0  0  0  0  0  0  0  0
  -15.4042   -1.4431   -0.2260 C   0  0  0  0  0  0  0  0  0  0  0  0
```

```
  -15.7082   -2.4356    0.1752 H   0  0  0  0  0  0  0  0  0  0  0  0
  -15.9565   -0.6511    0.3297 H   0  0  0  0  0  0  0  0  0  0  0  0
  -15.6903   -1.3878   -1.3001 H   0  0  0  0  0  0  0  0  0  0  0  0
   -0.6617  -13.0164   -5.4204 C   0  0  0  0  0  0  0  0  0  0  0  0
   -0.4725  -11.9282   -5.2776 H   0  0  0  0  0  0  0  0  0  0  0  0
    0.0049  -13.5899   -4.7391 H   0  0  0  0  0  0  0  0  0  0  0  0
   -0.4162  -13.2868   -6.4723 H   0  0  0  0  0  0  0  0  0  0  0  0
  1  2  1  0  0  0  0
  1  3  1  0  0  0  0
  1  9  1  0  0  0  0
  1 10  1  0  0  0  0
  2 11  1  0  0  0  0
  2 16  1  0  0  0  0
  2 19  1  0  0  0  0
  3  5  2  0  0  0  0
  3  7  1  0  0  0  0
  3 63  1  0  0  0  0
  4  5  1  0  0  0  0
  4  6  1  0  0  0  0
  4 13  1  0  0  0  0
  4 14  1  0  0  0  0
  5  8  1  0  0  0  0
  5 63  1  0  0  0  0
  6 12  1  0  0  0  0
  6 15  1  0  0  0  0
  6 20  1  0  0  0  0
 15 16  2  0  0  0  0
 15 17  1  0  0  0  0
 15 63  1  0  0  0  0
 16 18  1  0  0  0  0
 16 63  1  0  0  0  0
 21 22  3  0  0  0  0
 21 63  1  0  0  0  0
 22 25  1  0  0  0  0
 23 24  3  0  0  0  0
 23 63  1  0  0  0  0
 24 28  1  0  0  0  0
 25 26  1  0  0  0  0
 25 27  1  0  0  0  0
 25 31  1  0  0  0  0
 28 29  1  0  0  0  0
 28 30  1  0  0  0  0
 28 34  1  0  0  0  0
 31 32  1  0  0  0  0
 31 33  1  0  0  0  0
 31 47  1  0  0  0  0
 34 35  1  0  0  0  0
 34 36  1  0  0  0  0
 34 37  1  0  0  0  0
 37 38  2  0  0  0  0
 37 39  1  0  0  0  0
 38 40  1  0  0  0  0
 38 41  1  0  0  0  0
 39 42  2  0  0  0  0
 39 43  1  0  0  0  0
 40 44  2  0  0  0  0
 40 45  1  0  0  0  0
 42 44  1  0  0  0  0
 42 46  1  0  0  0  0
 44 60  1  0  0  0  0
 47 48  2  0  0  0  0
 47 49  1  0  0  0  0
 48 50  1  0  0  0  0
 48 51  1  0  0  0  0
 49 52  2  0  0  0  0
```

```
 49 53  1  0  0  0  0
 50 54  2  0  0  0  0
 50 55  1  0  0  0  0
 52 54  1  0  0  0  0
 52 56  1  0  0  0  0
 54 57  1  0  0  0  0
 57 58  1  0  0  0  0
 57 59  1  0  0  0  0
 57 64  1  0  0  0  0
 60 61  1  0  0  0  0
 60 62  1  0  0  0  0
 60 67  1  0  0  0  0
 64 65  1  0  0  0  0
 64 66  1  0  0  0  0
 64 70  1  0  0  0  0
 67 68  1  0  0  0  0
 67 69  1  0  0  0  0
 67 74  1  0  0  0  0
 70 71  1  0  0  0  0
 70 72  1  0  0  0  0
 70 73  1  0  0  0  0
 74 75  1  0  0  0  0
 74 76  1  0  0  0  0
 74 77  1  0  0  0  0
M  END
$$$$
mol176
Optimized Catalyst 176
Optimized Catalyst 176
 77 82  0  0  0  0  0  0  0  0999 V2000
   -0.8238    0.2411   -1.5310 C   0  0  0  0  0  0  0  0  0  0  0  0
    0.3929   -0.6404   -1.1787 C   0  0  0  0  0  0  0  0  0  0  0  0
   -2.0846   -0.0190   -0.7376 C   0  0  0  0  0  0  0  0  0  0  0  0
   -0.9741   -0.0427    1.5594 C   0  0  0  0  0  0  0  0  0  0  0  0
   -2.1577   -0.1662    0.6275 C   0  0  0  0  0  0  0  0  0  0  0  0
   -0.1950   -1.3543    1.7915 C   0  0  0  0  0  0  0  0  0  0  0  0
   -3.0158    0.1650   -1.3205 H   0  0  0  0  0  0  0  0  0  0  0  0
   -3.1457   -0.1466    1.1362 H   0  0  0  0  0  0  0  0  0  0  0  0
   -1.0479    0.1207   -2.6267 H   0  0  0  0  0  0  0  0  0  0  0  0
   -0.5440    1.3230   -1.3702 H   0  0  0  0  0  0  0  0  0  0  0  0
    1.0314   -0.7375   -2.1006 H   0  0  0  0  0  0  0  0  0  0  0  0
   -0.6135   -1.8626    2.7037 H   0  0  0  0  0  0  0  0  0  0  0  0
   -1.3379    0.3282    2.5579 H   0  0  0  0  0  0  0  0  0  0  0  0
   -0.2831    0.7486    1.1591 H   0  0  0  0  0  0  0  0  0  0  0  0
   -0.1870   -2.3351    0.6406 C   0  0  0  0  0  0  0  0  0  0  0  0
    0.0500   -2.0252   -0.6788 C   0  0  0  0  0  0  0  0  0  0  0  0
   -0.1402   -3.3979    0.9710 H   0  0  0  0  0  0  0  0  0  0  0  0
    0.2397   -2.8369   -1.4138 H   0  0  0  0  0  0  0  0  0  0  0  0
    1.0273   -0.1272   -0.4054 H   0  0  0  0  0  0  0  0  0  0  0  0
    0.8803   -1.0990    2.0223 H   0  0  0  0  0  0  0  0  0  0  0  0
   -4.2667   -2.3118   -0.6226 N   0  0  0  0  0  0  0  0  0  0  0  0
   -5.4231   -2.3833   -0.7596 C   0  0  0  0  0  0  0  0  0  0  0  0
   -2.2779   -4.2910   -0.6992 N   0  0  0  0  0  0  0  0  0  0  0  0
   -2.3228   -5.4381   -0.9069 C   0  0  0  0  0  0  0  0  0  0  0  0
   -6.8566   -2.4717   -0.9314 C   0  0  0  0  0  0  0  0  0  0  0  0
   -7.1118   -2.3841   -2.0290 H   0  0  0  0  0  0  0  0  0  0  0  0
   -7.2134   -3.4846   -0.5770 H   0  0  0  0  0  0  0  0  0  0  0  0
   -2.3587   -6.8614   -1.1645 C   0  0  0  0  0  0  0  0  0  0  0  0
   -3.4024   -7.2546   -0.9811 H   0  0  0  0  0  0  0  0  0  0  0  0
   -1.6643   -7.3936   -0.4495 H   0  0  0  0  0  0  0  0  0  0  0  0
   -7.6185   -1.3766   -0.1502 C   0  0  0  0  0  0  0  0  0  0  0  0
   -7.3456   -1.4192    0.9329 H   0  0  0  0  0  0  0  0  0  0  0  0
   -7.3444   -0.3662   -0.5420 H   0  0  0  0  0  0  0  0  0  0  0  0
   -1.9459   -7.1971   -2.6162 C   0  0  0  0  0  0  0  0  0  0  0  0
   -0.8812   -6.9013   -2.7883 H   0  0  0  0  0  0  0  0  0  0  0  0
```

```
   -2.5869   -6.6291   -3.3346 H   0  0  0  0  0  0  0  0  0  0  0  0
   -2.1020   -8.6774   -2.8535 C   0  0  0  0  0  0  0  0  0  0  0  0
   -3.2658   -9.1781   -3.4549 C   0  0  0  0  0  0  0  0  0  0  0  0
   -1.0813   -9.5641   -2.4807 C   0  0  0  0  0  0  0  0  0  0  0  0
   -3.4022  -10.5467   -3.6880 C   0  0  0  0  0  0  0  0  0  0  0  0
   -4.0666   -8.5037   -3.7529 H   0  0  0  0  0  0  0  0  0  0  0  0
   -1.2212  -10.9329   -2.7115 C   0  0  0  0  0  0  0  0  0  0  0  0
   -0.1691   -9.1909   -2.0180 H   0  0  0  0  0  0  0  0  0  0  0  0
   -2.3805  -11.4391   -3.3223 C   0  0  0  0  0  0  0  0  0  0  0  0
   -4.3059  -10.9257   -4.1660 H   0  0  0  0  0  0  0  0  0  0  0  0
   -0.4184  -11.6134   -2.4252 H   0  0  0  0  0  0  0  0  0  0  0  0
   -9.1008   -1.6010   -0.3115 C   0  0  0  0  0  0  0  0  0  0  0  0
   -9.8001   -2.3884    0.6149 C   0  0  0  0  0  0  0  0  0  0  0  0
   -9.7880   -1.0309   -1.3927 C   0  0  0  0  0  0  0  0  0  0  0  0
  -11.1696   -2.6051    0.4589 C   0  0  0  0  0  0  0  0  0  0  0  0
   -9.2827   -2.8300    1.4650 H   0  0  0  0  0  0  0  0  0  0  0  0
  -11.1576   -1.2492   -1.5471 C   0  0  0  0  0  0  0  0  0  0  0  0
   -9.2611   -0.4095   -2.1151 H   0  0  0  0  0  0  0  0  0  0  0  0
  -11.8616   -2.0419   -0.6259 C   0  0  0  0  0  0  0  0  0  0  0  0
  -11.7081   -3.2103    1.1891 H   0  0  0  0  0  0  0  0  0  0  0  0
  -11.6864   -0.7945   -2.3856 H   0  0  0  0  0  0  0  0  0  0  0  0
  -13.3383   -2.2700   -0.7885 C   0  0  0  0  0  0  0  0  0  0  0  0
  -13.6174   -3.2764   -0.3896 H   0  0  0  0  0  0  0  0  0  0  0  0
  -13.6138   -2.2479   -1.8717 H   0  0  0  0  0  0  0  0  0  0  0  0
   -2.5218  -12.9102   -3.5982 C   0  0  0  0  0  0  0  0  0  0  0  0
   -3.5734  -13.2296   -3.3888 H   0  0  0  0  0  0  0  0  0  0  0  0
   -1.8488  -13.4960   -2.9248 H   0  0  0  0  0  0  0  0  0  0  0  0
   -2.3131   -2.3473   -0.3852 Ir  0  0  0  0  0  0  0  0  0  0  0  0
  -14.1374   -1.1858   -0.0387 C   0  0  0  0  0  0  0  0  0  0  0  0
  -13.8662   -1.2177    1.0445 H   0  0  0  0  0  0  0  0  0  0  0  0
  -13.8569   -0.1829   -0.4428 H   0  0  0  0  0  0  0  0  0  0  0  0
   -2.1818  -13.2472   -5.0641 C   0  0  0  0  0  0  0  0  0  0  0  0
   -2.4307  -14.3224   -5.2424 H   0  0  0  0  0  0  0  0  0  0  0  0
   -2.8132  -12.6208   -5.7397 H   0  0  0  0  0  0  0  0  0  0  0  0
  -15.6449   -1.3897   -0.1899 C   0  0  0  0  0  0  0  0  0  0  0  0
  -15.9492   -2.3790    0.2191 H   0  0  0  0  0  0  0  0  0  0  0  0
  -16.1893   -0.5928    0.3669 H   0  0  0  0  0  0  0  0  0  0  0  0
  -15.9411   -1.3389   -1.2615 H   0  0  0  0  0  0  0  0  0  0  0  0
   -0.7074  -13.0134   -5.3973 C   0  0  0  0  0  0  0  0  0  0  0  0
   -0.4446  -11.9415   -5.2516 H   0  0  0  0  0  0  0  0  0  0  0  0
   -0.0569  -13.6355   -4.7437 H   0  0  0  0  0  0  0  0  0  0  0  0
   -0.5134  -13.2886   -6.4588 H   0  0  0  0  0  0  0  0  0  0  0  0
  1  2  1  0  0  0  0
  1  3  1  0  0  0  0
  1  9  1  0  0  0  0
  1 10  1  0  0  0  0
  2 11  1  0  0  0  0
  2 16  1  0  0  0  0
  2 19  1  0  0  0  0
  3  5  2  0  0  0  0
  3  7  1  0  0  0  0
  3 63  1  0  0  0  0
  4  5  1  0  0  0  0
  4  6  1  0  0  0  0
  4 13  1  0  0  0  0
  4 14  1  0  0  0  0
  5  8  1  0  0  0  0
  5 63  1  0  0  0  0
  6 12  1  0  0  0  0
  6 15  1  0  0  0  0
  6 20  1  0  0  0  0
 15 16  2  0  0  0  0
 15 17  1  0  0  0  0
 15 63  1  0  0  0  0
 16 18  1  0  0  0  0
```

```
 16 63  1  0  0  0  0
 21 22  3  0  0  0  0
 21 63  1  0  0  0  0
 22 25  1  0  0  0  0
 23 24  3  0  0  0  0
 23 63  1  0  0  0  0
 24 28  1  0  0  0  0
 25 26  1  0  0  0  0
 25 27  1  0  0  0  0
 25 31  1  0  0  0  0
 28 29  1  0  0  0  0
 28 30  1  0  0  0  0
 28 34  1  0  0  0  0
 31 32  1  0  0  0  0
 31 33  1  0  0  0  0
 31 47  1  0  0  0  0
 34 35  1  0  0  0  0
 34 36  1  0  0  0  0
 34 37  1  0  0  0  0
 37 38  2  0  0  0  0
 37 39  1  0  0  0  0
 38 40  1  0  0  0  0
 38 41  1  0  0  0  0
 39 42  2  0  0  0  0
 39 43  1  0  0  0  0
 40 44  2  0  0  0  0
 40 45  1  0  0  0  0
 42 44  1  0  0  0  0
 42 46  1  0  0  0  0
 44 60  1  0  0  0  0
 47 48  2  0  0  0  0
 47 49  1  0  0  0  0
 48 50  1  0  0  0  0
 48 51  1  0  0  0  0
 49 52  2  0  0  0  0
 49 53  1  0  0  0  0
 50 54  2  0  0  0  0
 50 55  1  0  0  0  0
 52 54  1  0  0  0  0
 52 56  1  0  0  0  0
 54 57  1  0  0  0  0
 57 58  1  0  0  0  0
 57 59  1  0  0  0  0
 57 64  1  0  0  0  0
 60 61  1  0  0  0  0
 60 62  1  0  0  0  0
 60 67  1  0  0  0  0
 64 65  1  0  0  0  0
 64 66  1  0  0  0  0
 64 70  1  0  0  0  0
 67 68  1  0  0  0  0
 67 69  1  0  0  0  0
 67 74  1  0  0  0  0
 70 71  1  0  0  0  0
 70 72  1  0  0  0  0
 70 73  1  0  0  0  0
 74 75  1  0  0  0  0
 74 76  1  0  0  0  0
 74 77  1  0  0  0  0
M  END
$$$$
mol177
Optimized Catalyst 177
Optimized Catalyst 177
 41 41  0  0  1  0  0  0  0  0999 V2000
```

```
   -1.4537    0.0289    0.4735 Rh  0  0  2  0  0  0  0  0  0  0  0  0
   -2.6082    0.2392    1.8356 C   0  0  0  0  0  0  0  0  0  0  0  0
   -3.3082    0.5464    2.6861 O   0  0  0  0  0  0  0  0  0  0  0  0
   -1.4707   -2.0273    0.1058 P   0  0  0  0  0  0  0  0  0  0  0  0
   -1.2587    2.1697    0.7214 O   0  0  0  0  0  0  0  0  0  0  0  0
   -0.5652    2.7001   -0.1699 C   0  0  0  0  0  0  0  0  0  0  0  0
   -0.2222   -0.3384   -1.1212 O   0  0  0  0  0  0  0  0  0  0  0  0
   -0.4423    4.2073   -0.0662 C   0  0  0  0  0  0  0  0  0  0  0  0
    0.6267    4.4969    0.0461 H   0  0  0  0  0  0  0  0  0  0  0  0
   -0.8616    4.6852   -0.9807 H   0  0  0  0  0  0  0  0  0  0  0  0
   -1.0121    4.5652    0.8253 H   0  0  0  0  0  0  0  0  0  0  0  0
    0.1254    2.0736   -1.2559 C   0  0  0  0  0  0  0  0  0  0  0  0
    0.6455    2.7900   -1.9017 H   0  0  0  0  0  0  0  0  0  0  0  0
    0.2611    0.7531   -1.6344 C   0  0  1  0  0  0  0  0  0  0  0  0
    1.1096    0.4200   -2.8477 C   0  0  0  0  0  0  0  0  0  0  0  0
    2.1387    0.8261   -2.7080 H   0  0  0  0  0  0  0  0  0  0  0  0
    1.1738   -0.6823   -2.9932 H   0  0  0  0  0  0  0  0  0  0  0  0
    0.6538    0.8816   -3.7550 H   0  0  0  0  0  0  0  0  0  0  0  0
   -2.8927   -3.0107    0.9374 C   0  0  0  0  0  0  0  0  0  0  0  0
   -2.7511   -4.1137    0.7538 H   0  0  0  0  0  0  0  0  0  0  0  0
   -2.8761   -2.8278    2.0451 H   0  0  0  0  0  0  0  0  0  0  0  0
   -1.7253   -2.4378   -1.7291 C   0  0  0  0  0  0  0  0  0  0  0  0
   -0.9403   -1.8614   -2.3197 H   0  0  0  0  0  0  0  0  0  0  0  0
   -2.7341   -2.0510   -2.0555 H   0  0  0  0  0  0  0  0  0  0  0  0
    0.1545   -2.8269    0.7921 C   0  0  0  0  0  0  0  0  0  0  0  0
   -0.0388   -4.2844    1.2122 C   0  0  0  0  0  0  0  0  0  0  0  0
   -0.3767   -4.9285    0.3676 H   0  0  0  0  0  0  0  0  0  0  0  0
    0.9545   -4.6854    1.5428 H   0  0  0  0  0  0  0  0  0  0  0  0
   -0.7326   -4.3938    2.0740 H   0  0  0  0  0  0  0  0  0  0  0  0
    0.4943   -1.9297    1.9930 C   0  0  0  0  0  0  0  0  0  0  0  0
    1.4915   -2.2205    2.4019 H   0  0  0  0  0  0  0  0  0  0  0  0
    0.5678   -0.8360    1.6822 H   0  0  0  0  0  0  0  0  0  0  0  0
   -0.2513   -2.0220    2.8152 H   0  0  0  0  0  0  0  0  0  0  0  0
    1.2280   -2.7138   -0.2933 C   0  0  0  0  0  0  0  0  0  0  0  0
    2.2139   -3.0182    0.1330 H   0  0  0  0  0  0  0  0  0  0  0  0
    1.0185   -3.3738   -1.1636 H   0  0  0  0  0  0  0  0  0  0  0  0
    1.3287   -1.6497   -0.6446 H   0  0  0  0  0  0  0  0  0  0  0  0
   -1.6084   -3.8531   -1.9269 C   0  0  0  0  0  0  0  0  0  0  0  0
   -4.1385   -2.5834    0.3614 C   0  0  0  0  0  0  0  0  0  0  0  0
   -1.5131   -4.9963   -2.0766 N   0  0  0  0  0  0  0  0  0  0  0  0
   -5.1431   -2.2556   -0.1085 N   0  0  0  0  0  0  0  0  0  0  0  0
  1  5  1  0  0  0  0
  1  2  1  6  0  0  0
  2  3  1  0  0  0  0
  4  1  1  0  0  0  0
  4 25  1  0  0  0  0
  4 19  1  0  0  0  0
  6  8  1  0  0  0  0
  6  5  1  0  0  0  0
  7  1  1  0  0  0  0
  8  9  1  0  0  0  0
  8 11  1  0  0  0  0
 10  8  1  0  0  0  0
 12  6  2  0  0  0  0
 13 12  1  0  0  0  0
 14 15  1  1  0  0  0
 14 12  1  0  0  0  0
 14  7  1  0  0  0  0
 15 16  1  0  0  0  0
 17 15  1  0  0  0  0
 18 15  1  0  0  0  0
 19 21  1  0  0  0  0
 20 19  1  0  0  0  0
 22  4  1  0  0  0  0
 23 22  1  0  0  0  0
```

```
 24 22  1  0  0  0  0
 25 26  1  0  0  0  0
 25 30  1  0  0  0  0
 26 28  1  0  0  0  0
 26 29  1  0  0  0  0
 27 26  1  0  0  0  0
 30 31  1  0  0  0  0
 30 33  1  0  0  0  0
 32 30  1  0  0  0  0
 34 35  1  0  0  0  0
 34 25  1  0  0  0  0
 36 34  1  0  0  0  0
 37 34  1  0  0  0  0
 38 22  1  0  0  0  0
 39 19  1  0  0  0  0
 40 38  3  0  0  0  0
 41 39  3  0  0  0  0
M  END
$$$$
mol178
Optimized Catalyst 178
Optimized Catalyst 178
106114  0  0  0  0  0  0  0  0999 V2000
   -0.0868    0.1807    1.3010 H   0  0  0  0  0  0  0  0  0  0  0  0
   -0.0166   -2.2697   -0.0328 P   0  0  0  0  0  0  0  0  0  0  0  0
    2.1024    1.1635    0.0003 P   0  0  0  0  0  0  0  0  0  0  0  0
   -2.1118    1.1993   -0.0363 P   0  0  0  0  0  0  0  0  0  0  0  0
   -1.3342   -3.2354   -0.9059 C   0  0  0  0  0  0  0  0  0  0  0  0
   -1.9145   -4.3892   -0.3540 C   0  0  0  0  0  0  0  0  0  0  0  0
   -1.7833   -2.7943   -2.1641 C   0  0  0  0  0  0  0  0  0  0  0  0
   -2.9156   -5.0811   -1.0406 C   0  0  0  0  0  0  0  0  0  0  0  0
   -1.5891   -4.7397    0.6170 H   0  0  0  0  0  0  0  0  0  0  0  0
   -2.7740   -3.4945   -2.8550 C   0  0  0  0  0  0  0  0  0  0  0  0
   -1.3625   -1.8974   -2.6013 H   0  0  0  0  0  0  0  0  0  0  0  0
   -3.3472   -4.6381   -2.2927 C   0  0  0  0  0  0  0  0  0  0  0  0
   -3.3554   -5.9672   -0.5965 H   0  0  0  0  0  0  0  0  0  0  0  0
   -3.1035   -3.1399   -3.8251 H   0  0  0  0  0  0  0  0  0  0  0  0
    1.4864   -3.1585   -0.6298 C   0  0  0  0  0  0  0  0  0  0  0  0
    2.7174   -2.8911    0.0023 C   0  0  0  0  0  0  0  0  0  0  0  0
    1.4706   -4.0338   -1.7273 C   0  0  0  0  0  0  0  0  0  0  0  0
    3.8923   -3.4925   -0.4463 C   0  0  0  0  0  0  0  0  0  0  0  0
    2.7532   -2.2073    0.8423 H   0  0  0  0  0  0  0  0  0  0  0  0
    2.6543   -4.6209   -2.1850 C   0  0  0  0  0  0  0  0  0  0  0  0
    0.5347   -4.2575   -2.2229 H   0  0  0  0  0  0  0  0  0  0  0  0
    3.8661   -4.3568   -1.5457 C   0  0  0  0  0  0  0  0  0  0  0  0
    4.8282   -3.2739    0.0549 H   0  0  0  0  0  0  0  0  0  0  0  0
    2.6237   -5.2908   -3.0371 H   0  0  0  0  0  0  0  0  0  0  0  0
   -0.1764   -2.9334    1.6796 C   0  0  0  0  0  0  0  0  0  0  0  0
    0.4783   -4.0995    2.1123 C   0  0  0  0  0  0  0  0  0  0  0  0
   -1.0281   -2.2637    2.5751 C   0  0  0  0  0  0  0  0  0  0  0  0
    0.2898   -4.5780    3.4112 C   0  0  0  0  0  0  0  0  0  0  0  0
    1.1437   -4.6228    1.4371 H   0  0  0  0  0  0  0  0  0  0  0  0
   -1.2229   -2.7509    3.8681 C   0  0  0  0  0  0  0  0  0  0  0  0
   -1.5321   -1.3596    2.2566 H   0  0  0  0  0  0  0  0  0  0  0  0
   -0.5622   -3.9070    4.2912 C   0  0  0  0  0  0  0  0  0  0  0  0
    0.8078   -5.4745    3.7333 H   0  0  0  0  0  0  0  0  0  0  0  0
   -1.8896   -2.2248    4.5417 H   0  0  0  0  0  0  0  0  0  0  0  0
   -3.0354    1.7437   -1.5382 C   0  0  0  0  0  0  0  0  0  0  0  0
   -4.3984    1.4820   -1.7506 C   0  0  0  0  0  0  0  0  0  0  0  0
   -2.3331    2.4806   -2.5093 C   0  0  0  0  0  0  0  0  0  0  0  0
   -5.0369    1.9378   -2.9079 C   0  0  0  0  0  0  0  0  0  0  0  0
   -4.9562    0.9155   -1.0161 H   0  0  0  0  0  0  0  0  0  0  0  0
   -2.9731    2.9416   -3.6581 C   0  0  0  0  0  0  0  0  0  0  0  0
   -1.2787    2.6863   -2.3625 H   0  0  0  0  0  0  0  0  0  0  0  0
   -4.3288    2.6683   -3.8627 C   0  0  0  0  0  0  0  0  0  0  0  0
```

```
  -6.0887    1.7219   -3.0590 H   0  0  0  0  0  0  0  0  0  0  0  0
  -2.4141    3.5055   -4.3961 H   0  0  0  0  0  0  0  0  0  0  0  0
  -2.1422    2.7540    0.9572 C   0  0  0  0  0  0  0  0  0  0  0  0
  -1.1387    2.9766    1.9113 C   0  0  0  0  0  0  0  0  0  0  0  0
  -3.1622    3.7108    0.8092 C   0  0  0  0  0  0  0  0  0  0  0  0
  -1.1579    4.1215    2.7103 C   0  0  0  0  0  0  0  0  0  0  0  0
  -0.3352    2.2564    2.0030 H   0  0  0  0  0  0  0  0  0  0  0  0
  -3.1767    4.8574    1.6045 C   0  0  0  0  0  0  0  0  0  0  0  0
  -3.9387    3.5581    0.0695 H   0  0  0  0  0  0  0  0  0  0  0  0
  -2.1765    5.0637    2.5592 C   0  0  0  0  0  0  0  0  0  0  0  0
  -0.3661    4.2841    3.4321 H   0  0  0  0  0  0  0  0  0  0  0  0
  -3.9671    5.5891    1.4788 H   0  0  0  0  0  0  0  0  0  0  0  0
  -3.3856    0.2052    0.8520 C   0  0  0  0  0  0  0  0  0  0  0  0
  -3.9356    0.5977    2.0825 C   0  0  0  0  0  0  0  0  0  0  0  0
  -3.7734   -1.0321    0.3030 C   0  0  0  0  0  0  0  0  0  0  0  0
  -4.8466   -0.2285    2.7482 C   0  0  0  0  0  0  0  0  0  0  0  0
  -3.6537    1.5479    2.5170 H   0  0  0  0  0  0  0  0  0  0  0  0
  -4.6929   -1.8466    0.9623 C   0  0  0  0  0  0  0  0  0  0  0  0
  -3.3419   -1.3656   -0.6329 H   0  0  0  0  0  0  0  0  0  0  0  0
  -5.2306   -1.4485    2.1902 C   0  0  0  0  0  0  0  0  0  0  0  0
  -5.2588    0.0879    3.7000 H   0  0  0  0  0  0  0  0  0  0  0  0
  -4.9709   -2.7977    0.5234 H   0  0  0  0  0  0  0  0  0  0  0  0
   3.2570    1.1205   -1.4361 C   0  0  0  0  0  0  0  0  0  0  0  0
   3.4047   -0.0988   -2.1216 C   0  0  0  0  0  0  0  0  0  0  0  0
   3.9970    2.2337   -1.8665 C   0  0  0  0  0  0  0  0  0  0  0  0
   4.2775   -0.2031   -3.2035 C   0  0  0  0  0  0  0  0  0  0  0  0
   2.8344   -0.9627   -1.8019 H   0  0  0  0  0  0  0  0  0  0  0  0
   4.8623    2.1279   -2.9592 C   0  0  0  0  0  0  0  0  0  0  0  0
   3.8891    3.1813   -1.3541 H   0  0  0  0  0  0  0  0  0  0  0  0
   5.0070    0.9112   -3.6277 C   0  0  0  0  0  0  0  0  0  0  0  0
   4.3815   -1.1527   -3.7156 H   0  0  0  0  0  0  0  0  0  0  0  0
   5.4217    2.9975   -3.2860 H   0  0  0  0  0  0  0  0  0  0  0  0
   3.1966    0.5147    1.3402 C   0  0  0  0  0  0  0  0  0  0  0  0
   2.6230   -0.1224    2.4531 C   0  0  0  0  0  0  0  0  0  0  0  0
   4.5967    0.6335    1.2776 C   0  0  0  0  0  0  0  0  0  0  0  0
   3.4259   -0.6138    3.4851 C   0  0  0  0  0  0  0  0  0  0  0  0
   1.5475   -0.2537    2.4900 H   0  0  0  0  0  0  0  0  0  0  0  0
   5.3975    0.1382    2.3087 C   0  0  0  0  0  0  0  0  0  0  0  0
   5.0563    1.1029    0.4162 H   0  0  0  0  0  0  0  0  0  0  0  0
   4.8147   -0.4837    3.4161 C   0  0  0  0  0  0  0  0  0  0  0  0
   2.9651   -1.1087    4.3324 H   0  0  0  0  0  0  0  0  0  0  0  0
   6.4757    0.2344    2.2448 H   0  0  0  0  0  0  0  0  0  0  0  0
   2.0943    2.9687    0.3788 C   0  0  0  0  0  0  0  0  0  0  0  0
   2.8560    3.5560    1.4001 C   0  0  0  0  0  0  0  0  0  0  0  0
   1.2726    3.7932   -0.4107 C   0  0  0  0  0  0  0  0  0  0  0  0
   2.7971    4.9355    1.6233 C   0  0  0  0  0  0  0  0  0  0  0  0
   3.4944    2.9370    2.0174 H   0  0  0  0  0  0  0  0  0  0  0  0
   1.2292    5.1692   -0.1999 C   0  0  0  0  0  0  0  0  0  0  0  0
   0.6514    3.3377   -1.1746 H   0  0  0  0  0  0  0  0  0  0  0  0
   1.9910    5.7449    0.8219 C   0  0  0  0  0  0  0  0  0  0  0  0
   3.3870    5.3750    2.4201 H   0  0  0  0  0  0  0  0  0  0  0  0
   0.5886    5.7895   -0.8162 H   0  0  0  0  0  0  0  0  0  0  0  0
   0.0583    0.1757   -2.2605 C   0  0  0  0  0  0  0  0  0  0  0  0
   0.0784    0.2152   -3.4324 O   0  0  0  0  0  0  0  0  0  0  0  0
  -5.9376   -2.0868    2.7078 H   0  0  0  0  0  0  0  0  0  0  0  0
  -0.7084   -4.2806    5.2984 H   0  0  0  0  0  0  0  0  0  0  0  0
   5.4385   -0.8715    4.2134 H   0  0  0  0  0  0  0  0  0  0  0  0
   4.7817   -4.8171   -1.8990 H   0  0  0  0  0  0  0  0  0  0  0  0
   5.6781    0.8322   -4.4755 H   0  0  0  0  0  0  0  0  0  0  0  0
  -4.8254    3.0193   -4.7600 H   0  0  0  0  0  0  0  0  0  0  0  0
  -2.1877    5.9571    3.1733 H   0  0  0  0  0  0  0  0  0  0  0  0
   1.9482    6.8141    0.9953 H   0  0  0  0  0  0  0  0  0  0  0  0
  -4.1238   -5.1761   -2.8243 H   0  0  0  0  0  0  0  0  0  0  0  0
  -0.0084    0.1265   -0.3227 Rh  0  0  0  0  0  0  0  0  0  0  0  0
 1106  1  0  0  0  0
```

```
  2  5  1  0  0  0  0
  2 15  1  0  0  0  0
  2 25  1  0  0  0  0
  2106  1  0  0  0  0
  3 65  1  0  0  0  0
  3 75  1  0  0  0  0
  3 85  1  0  0  0  0
  3106  1  0  0  0  0
  4 35  1  0  0  0  0
  4 45  1  0  0  0  0
  4 55  1  0  0  0  0
  4106  1  0  0  0  0
  5  6  2  0  0  0  0
  5  7  1  0  0  0  0
  6  8  1  0  0  0  0
  6  9  1  0  0  0  0
  7 10  2  0  0  0  0
  7 11  1  0  0  0  0
  8 12  2  0  0  0  0
  8 13  1  0  0  0  0
 10 12  1  0  0  0  0
 10 14  1  0  0  0  0
 12105  1  0  0  0  0
 15 16  2  0  0  0  0
 15 17  1  0  0  0  0
 16 18  1  0  0  0  0
 16 19  1  0  0  0  0
 17 20  2  0  0  0  0
 17 21  1  0  0  0  0
 18 22  2  0  0  0  0
 18 23  1  0  0  0  0
 20 22  1  0  0  0  0
 20 24  1  0  0  0  0
 22100  1  0  0  0  0
 25 26  2  0  0  0  0
 25 27  1  0  0  0  0
 26 28  1  0  0  0  0
 26 29  1  0  0  0  0
 27 30  2  0  0  0  0
 27 31  1  0  0  0  0
 28 32  2  0  0  0  0
 28 33  1  0  0  0  0
 30 32  1  0  0  0  0
 30 34  1  0  0  0  0
 32 98  1  0  0  0  0
 35 36  2  0  0  0  0
 35 37  1  0  0  0  0
 36 38  1  0  0  0  0
 36 39  1  0  0  0  0
 37 40  2  0  0  0  0
 37 41  1  0  0  0  0
 38 42  2  0  0  0  0
 38 43  1  0  0  0  0
 40 42  1  0  0  0  0
 40 44  1  0  0  0  0
 42102  1  0  0  0  0
 45 46  2  0  0  0  0
 45 47  1  0  0  0  0
 46 48  1  0  0  0  0
 46 49  1  0  0  0  0
 47 50  2  0  0  0  0
 47 51  1  0  0  0  0
 48 52  2  0  0  0  0
 48 53  1  0  0  0  0
 50 52  1  0  0  0  0
```

```
 50 54  1  0  0  0  0
 52103  1  0  0  0  0
 55 56  2  0  0  0  0
 55 57  1  0  0  0  0
 56 58  1  0  0  0  0
 56 59  1  0  0  0  0
 57 60  2  0  0  0  0
 57 61  1  0  0  0  0
 58 62  2  0  0  0  0
 58 63  1  0  0  0  0
 60 62  1  0  0  0  0
 60 64  1  0  0  0  0
 62 97  1  0  0  0  0
 65 66  2  0  0  0  0
 65 67  1  0  0  0  0
 66 68  1  0  0  0  0
 66 69  1  0  0  0  0
 67 70  2  0  0  0  0
 67 71  1  0  0  0  0
 68 72  2  0  0  0  0
 68 73  1  0  0  0  0
 70 72  1  0  0  0  0
 70 74  1  0  0  0  0
 72101  1  0  0  0  0
 75 76  2  0  0  0  0
 75 77  1  0  0  0  0
 76 78  1  0  0  0  0
 76 79  1  0  0  0  0
 77 80  2  0  0  0  0
 77 81  1  0  0  0  0
 78 82  2  0  0  0  0
 78 83  1  0  0  0  0
 80 82  1  0  0  0  0
 80 84  1  0  0  0  0
 82 99  1  0  0  0  0
 85 86  2  0  0  0  0
 85 87  1  0  0  0  0
 86 88  1  0  0  0  0
 86 89  1  0  0  0  0
 87 90  2  0  0  0  0
 87 91  1  0  0  0  0
 88 92  2  0  0  0  0
 88 93  1  0  0  0  0
 90 92  1  0  0  0  0
 90 94  1  0  0  0  0
 92104  1  0  0  0  0
 95 96  2  0  0  0  0
 95106  1  0  0  0  0
M  END
$$$$
mol179
Optimized Catalyst 179
Optimized Catalyst 179
 79 86  0  0  0  0  0  0  0  0999 V2000
   -0.8406    2.6776   -0.1037 C   0  0  0  0  0  0  0  0  0  0  0  0
    0.0255    3.1908    1.0185 C   0  0  0  0  0  0  0  0  0  0  0  0
    1.3599    3.8517   -2.2471 C   0  0  0  0  0  0  0  0  0  0  0  0
    0.8008    4.4893    0.7089 C   0  0  0  0  0  0  0  0  0  0  0  0
    2.4193    4.4777   -1.3270 C   0  0  0  0  0  0  0  0  0  0  0  0
    2.2299    4.2686    0.1853 C   0  0  0  0  0  0  0  0  0  0  0  0
    0.7486    2.3598    1.3092 H   0  0  0  0  0  0  0  0  0  0  0  0
    0.2155    5.0958   -0.0226 H   0  0  0  0  0  0  0  0  0  0  0  0
    0.4351    4.4792   -2.2225 H   0  0  0  0  0  0  0  0  0  0  0  0
    2.4349    5.5781   -1.5351 H   0  0  0  0  0  0  0  0  0  0  0  0
   -1.9355    2.6956    0.0996 H   0  0  0  0  0  0  0  0  0  0  0  0
```

```
   -0.6310    3.3842    1.9075 H   0  0  0  0  0  0  0  0  0  0  0  0
    1.7567    3.8778   -3.2931 H   0  0  0  0  0  0  0  0  0  0  0  0
    0.8665    5.0768    1.6597 H   0  0  0  0  0  0  0  0  0  0  0  0
    3.4220    4.0630   -1.6019 H   0  0  0  0  0  0  0  0  0  0  0  0
    2.9080    4.9955    0.7043 H   0  0  0  0  0  0  0  0  0  0  0  0
   -0.4256    2.3010   -1.3801 C   0  0  0  0  0  0  0  0  0  0  0  0
   -1.1998    2.0251   -2.1293 H   0  0  0  0  0  0  0  0  0  0  0  0
    0.9847    2.3956   -1.8992 C   0  0  0  0  0  0  0  0  0  0  0  0
    1.6929    1.9897   -1.1129 H   0  0  0  0  0  0  0  0  0  0  0  0
    1.0874    1.7544   -2.8106 H   0  0  0  0  0  0  0  0  0  0  0  0
    2.8745   -0.4047   -0.6843 C   0  0  0  0  0  0  0  0  0  0  0  0
    4.0928   -1.0053   -1.3175 C   0  0  0  0  0  0  0  0  0  0  0  0
    4.1650   -0.9605    2.2621 C   0  0  0  0  0  0  0  0  0  0  0  0
    4.7109   -2.1729   -0.5184 C   0  0  0  0  0  0  0  0  0  0  0  0
    5.4887   -1.6613    1.9145 C   0  0  0  0  0  0  0  0  0  0  0  0
    5.8536   -1.7530    0.4227 C   0  0  0  0  0  0  0  0  0  0  0  0
    4.8556   -0.1965   -1.4556 H   0  0  0  0  0  0  0  0  0  0  0  0
    3.9105   -2.6843    0.0631 H   0  0  0  0  0  0  0  0  0  0  0  0
    3.3195   -1.6602    2.0577 H   0  0  0  0  0  0  0  0  0  0  0  0
    5.4421   -2.6973    2.3334 H   0  0  0  0  0  0  0  0  0  0  0  0
    1.9616   -0.4697   -1.2991 H   0  0  0  0  0  0  0  0  0  0  0  0
    3.8211   -1.3860   -2.3342 H   0  0  0  0  0  0  0  0  0  0  0  0
    4.1743   -0.7432    3.3586 H   0  0  0  0  0  0  0  0  0  0  0  0
    5.1239   -2.9055   -1.2556 H   0  0  0  0  0  0  0  0  0  0  0  0
    6.3216   -1.1192    2.4299 H   0  0  0  0  0  0  0  0  0  0  0  0
    6.6777   -2.5065    0.3305 H   0  0  0  0  0  0  0  0  0  0  0  0
    2.8020    0.1988    0.5093 C   0  0  0  0  0  0  0  0  0  0  0  0
    1.8309    0.6108    0.8477 H   0  0  0  0  0  0  0  0  0  0  0  0
    3.9161    0.3581    1.4996 C   0  0  0  0  0  0  0  0  0  0  0  0
    4.8482    0.6735    0.9685 H   0  0  0  0  0  0  0  0  0  0  0  0
    3.6579    1.1636    2.2283 H   0  0  0  0  0  0  0  0  0  0  0  0
    6.2690   -0.7718    0.0905 H   0  0  0  0  0  0  0  0  0  0  0  0
    2.5792    3.2445    0.4601 H   0  0  0  0  0  0  0  0  0  0  0  0
   -0.2048   -3.9914   -2.5223 C   0  0  0  0  0  0  0  0  0  0  0  0
   -0.0442   -4.3266   -1.2004 C   0  0  0  0  0  0  0  0  0  0  0  0
   -0.0419   -3.3137   -0.1890 C   0  0  0  0  0  0  0  0  0  0  0  0
   -0.2080   -1.9618   -0.6004 C   0  0  2  0  0  0  0  0  0  0  0  0
   -0.3724   -1.6257   -1.9848 C   0  0  0  0  0  0  0  0  0  0  0  0
   -0.3722   -2.6296   -2.9206 C   0  0  0  0  0  0  0  0  0  0  0  0
    0.2469   -4.6315    1.5277 H   0  0  0  0  0  0  0  0  0  0  0  0
   -0.2056   -4.7642   -3.2975 H   0  0  0  0  0  0  0  0  0  0  0  0
    0.0864   -5.3691   -0.8978 H   0  0  0  0  0  0  0  0  0  0  0  0
    0.1207   -3.5937    1.2045 C   0  0  0  0  0  0  0  0  0  0  0  0
   -0.4970   -0.5593   -2.2563 H   0  0  0  0  0  0  0  0  0  0  0  0
   -0.4964   -2.4054   -3.9836 H   0  0  0  0  0  0  0  0  0  0  0  0
   -0.0380   -1.2186    1.6677 C   0  0  0  0  0  0  0  0  0  0  0  0
    0.1185   -2.5625    2.1139 C   0  0  0  0  0  0  0  0  0  0  0  0
   -0.0220   -0.3652    2.3728 H   0  0  0  0  0  0  0  0  0  0  0  0
    0.2400   -2.7440    3.1860 H   0  0  0  0  0  0  0  0  0  0  0  0
   -0.1971   -0.9256    0.3586 N   0  0  0  0  0  0  0  0  0  0  0  0
   -5.0612    0.1918    2.9986 C   0  0  0  0  0  0  0  0  0  0  0  0
   -5.7268   -0.1886    1.8600 C   0  0  0  0  0  0  0  0  0  0  0  0
   -5.0489   -0.2132    0.6006 C   0  0  0  0  0  0  0  0  0  0  0  0
   -3.6759    0.1572    0.5568 C   0  0  0  0  0  0  0  0  0  0  0  0
   -3.0015    0.5522    1.7582 C   0  0  0  0  0  0  0  0  0  0  0  0
   -3.6846    0.5682    2.9482 C   0  0  0  0  0  0  0  0  0  0  0  0
   -6.7388   -0.8722   -0.6147 H   0  0  0  0  0  0  0  0  0  0  0  0
   -5.5740    0.2171    3.9633 H   0  0  0  0  0  0  0  0  0  0  0  0
   -6.7805   -0.4748    1.8945 H   0  0  0  0  0  0  0  0  0  0  0  0
   -5.6837   -0.5867   -0.6253 C   0  0  0  0  0  0  0  0  0  0  0  0
   -1.9384    0.8465    1.6857 H   0  0  0  0  0  0  0  0  0  0  0  0
   -3.1929    0.8711    3.8751 H   0  0  0  0  0  0  0  0  0  0  0  0
   -3.5807   -0.1952   -1.7554 C   0  0  0  0  0  0  0  0  0  0  0  0
   -4.9587   -0.5766   -1.7917 C   0  0  0  0  0  0  0  0  0  0  0  0
   -2.9938   -0.1836   -2.6836 H   0  0  0  0  0  0  0  0  0  0  0  0
```

```
   -5.4101   -0.8494   -2.7465 H   0  0  0  0  0  0  0  0  0  0  0  0
   -2.9455    0.1612   -0.6350 N   0  0  0  0  0  0  0  0  0  0  0  0
   -0.3904    0.6871   -0.1494 Rh  0  0  0  0  0  0  0  0  0  0  0  0
  1 11  1  0  0  0  0
  1  2  1  0  0  0  0
  1 17  2  0  0  0  0
  1 79  1  0  0  0  0
  2  7  1  0  0  0  0
  2 12  1  0  0  0  0
  2  4  1  0  0  0  0
  3  9  1  0  0  0  0
  3 19  1  0  0  0  0
  3  5  1  0  0  0  0
  3 13  1  0  0  0  0
  4 14  1  0  0  0  0
  4  6  1  0  0  0  0
  4  8  1  0  0  0  0
  5  6  1  0  0  0  0
  5 10  1  0  0  0  0
  5 15  1  0  0  0  0
  6 44  1  0  0  0  0
  6 16  1  0  0  0  0
 17 18  1  0  0  0  0
 17 79  1  0  0  0  0
 17 19  1  0  0  0  0
 19 20  1  0  0  0  0
 19 21  1  0  0  0  0
 22 38  2  0  0  0  0
 22 23  1  0  0  0  0
 22 32  1  0  0  0  0
 22 79  1  0  0  0  0
 23 25  1  0  0  0  0
 23 28  1  0  0  0  0
 23 33  1  0  0  0  0
 24 34  1  0  0  0  0
 24 26  1  0  0  0  0
 24 30  1  0  0  0  0
 24 40  1  0  0  0  0
 25 29  1  0  0  0  0
 25 27  1  0  0  0  0
 25 35  1  0  0  0  0
 26 31  1  0  0  0  0
 26 36  1  0  0  0  0
 26 27  1  0  0  0  0
 27 37  1  0  0  0  0
 27 43  1  0  0  0  0
 38 39  1  0  0  0  0
 38 40  1  0  0  0  0
 38 79  1  0  0  0  0
 40 42  1  0  0  0  0
 40 41  1  0  0  0  0
 45 46  2  0  0  0  0
 45 50  1  0  0  0  0
 45 52  1  0  0  0  0
 46 53  1  0  0  0  0
 46 47  1  0  0  0  0
 47 54  2  0  0  0  0
 48 47  1  6  0  0  0
 48 49  1  0  0  0  0
 48 61  1  0  0  0  0
 49 50  2  0  0  0  0
 49 55  1  0  0  0  0
 50 56  1  0  0  0  0
 51 54  1  0  0  0  0
 54 58  1  0  0  0  0
```

```
 57 58  2  0  0  0  0
 57 59  1  0  0  0  0
 57 61  1  0  0  0  0
 58 60  1  0  0  0  0
 61 79  1  0  0  0  0
 62 69  1  0  0  0  0
 62 63  2  0  0  0  0
 62 67  1  0  0  0  0
 63 70  1  0  0  0  0
 63 64  1  0  0  0  0
 64 65  1  0  0  0  0
 64 71  2  0  0  0  0
 65 66  1  0  0  0  0
 65 78  2  0  0  0  0
 66 67  2  0  0  0  0
 66 72  1  0  0  0  0
 67 73  1  0  0  0  0
 68 71  1  0  0  0  0
 71 75  1  0  0  0  0
 74 78  1  0  0  0  0
 74 75  2  0  0  0  0
 74 76  1  0  0  0  0
 75 77  1  0  0  0  0
 78 79  1  0  0  0  0
M  END
$$$$
mol180
Optimized Catalyst 180
Optimized Catalyst 180
 79 86  0  0  0  0  0  0  0  0999 V2000
   -3.2034    0.0158   -1.4233 C   0  0  0  0  0  0  0  0  0  0  0  0
   -2.5020    1.0271   -0.5605 C   0  0  0  0  0  0  0  0  0  0  0  0
   -1.4524    1.0327   -3.9551 C   0  0  0  0  0  0  0  0  0  0  0  0
   -2.2467    2.3645   -1.2970 C   0  0  0  0  0  0  0  0  0  0  0  0
   -0.6802    2.2291   -3.3785 C   0  0  0  0  0  0  0  0  0  0  0  0
   -0.8274    2.5043   -1.8719 C   0  0  0  0  0  0  0  0  0  0  0  0
   -1.5290    0.6145   -0.1957 H   0  0  0  0  0  0  0  0  0  0  0  0
   -2.9987    2.4910   -2.1108 H   0  0  0  0  0  0  0  0  0  0  0  0
   -2.5281    1.3151   -4.0629 H   0  0  0  0  0  0  0  0  0  0  0  0
   -1.0182    3.1396   -3.9378 H   0  0  0  0  0  0  0  0  0  0  0  0
   -4.2708   -0.1264   -1.1691 H   0  0  0  0  0  0  0  0  0  0  0  0
   -3.1382    1.2434    0.3359 H   0  0  0  0  0  0  0  0  0  0  0  0
   -1.0529    0.8311   -4.9807 H   0  0  0  0  0  0  0  0  0  0  0  0
   -2.4050    3.1901   -0.5577 H   0  0  0  0  0  0  0  0  0  0  0  0
    0.4097    2.0864   -3.5893 H   0  0  0  0  0  0  0  0  0  0  0  0
   -0.4899    3.5609   -1.7008 H   0  0  0  0  0  0  0  0  0  0  0  0
   -2.6880   -0.5919   -2.5191 C   0  0  0  0  0  0  0  0  0  0  0  0
   -3.3376   -1.2236   -3.1632 H   0  0  0  0  0  0  0  0  0  0  0  0
   -1.3470   -0.2684   -3.1250 C   0  0  0  0  0  0  0  0  0  0  0  0
   -0.5744   -0.1302   -2.3293 H   0  0  0  0  0  0  0  0  0  0  0  0
   -1.0214   -1.1001   -3.7968 H   0  0  0  0  0  0  0  0  0  0  0  0
    0.0798   -3.0580   -1.1807 C   0  0  0  0  0  0  0  0  0  0  0  0
    1.1414   -2.8919   -2.2306 C   0  0  0  0  0  0  0  0  0  0  0  0
    2.0348   -1.5861    0.9479 C   0  0  0  0  0  0  0  0  0  0  0  0
    2.5773   -3.0722   -1.6855 C   0  0  0  0  0  0  0  0  0  0  0  0
    3.3243   -1.3712    0.1407 C   0  0  0  0  0  0  0  0  0  0  0  0
    3.2892   -1.7511   -1.3493 C   0  0  0  0  0  0  0  0  0  0  0  0
    1.0538   -1.8819   -2.7029 H   0  0  0  0  0  0  0  0  0  0  0  0
    2.5551   -3.7291   -0.7856 H   0  0  0  0  0  0  0  0  0  0  0  0
    1.9299   -2.6740    1.1785 H   0  0  0  0  0  0  0  0  0  0  0  0
    4.1305   -1.9686    0.6389 H   0  0  0  0  0  0  0  0  0  0  0  0
   -0.5139   -3.9883   -1.2939 H   0  0  0  0  0  0  0  0  0  0  0  0
    0.9786   -3.6556   -3.0328 H   0  0  0  0  0  0  0  0  0  0  0  0
    2.1489   -1.0442    1.9198 H   0  0  0  0  0  0  0  0  0  0  0  0
    3.1731   -3.5951   -2.4756 H   0  0  0  0  0  0  0  0  0  0  0  0
```

```
    3.6103   -0.2911    0.2059 H   0  0  0  0  0  0  0  0  0  0  0  0
    4.3545   -1.8359   -1.6903 H   0  0  0  0  0  0  0  0  0  0  0  0
   -0.1203   -2.2557   -0.1168 C   0  0  0  0  0  0  0  0  0  0  0  0
   -0.8973   -2.5084    0.6414 H   0  0  0  0  0  0  0  0  0  0  0  0
    0.7456   -1.0853    0.2557 C   0  0  0  0  0  0  0  0  0  0  0  0
    1.0209   -0.4948   -0.6519 H   0  0  0  0  0  0  0  0  0  0  0  0
    0.1999   -0.4096    0.9568 H   0  0  0  0  0  0  0  0  0  0  0  0
    2.8286   -0.9162   -1.9281 H   0  0  0  0  0  0  0  0  0  0  0  0
   -0.1250    1.8459   -1.3092 H   0  0  0  0  0  0  0  0  0  0  0  0
   -2.5239   -7.8595   -3.0501 C   0  0  0  0  0  0  0  0  0  0  0  0
   -2.7075   -8.1418   -1.7205 C   0  0  0  0  0  0  0  0  0  0  0  0
   -2.8340   -7.0827   -0.7630 C   0  0  0  0  0  0  0  0  0  0  0  0
   -2.7620   -5.7381   -1.2262 C   0  0  2  0  0  0  0  0  0  0  0  0
   -2.5666   -5.4637   -2.6189 C   0  0  0  0  0  0  0  0  0  0  0  0
   -2.4511   -6.5076   -3.5047 C   0  0  0  0  0  0  0  0  0  0  0  0
   -3.0863   -8.3529    0.9974 H   0  0  0  0  0  0  0  0  0  0  0  0
   -2.4266   -8.6656   -3.7858 H   0  0  0  0  0  0  0  0  0  0  0  0
   -2.7572   -9.1764   -1.3662 H   0  0  0  0  0  0  0  0  0  0  0  0
   -3.0283   -7.3215    0.6298 C   0  0  0  0  0  0  0  0  0  0  0  0
   -2.4998   -4.4138   -2.9634 H   0  0  0  0  0  0  0  0  0  0  0  0
   -2.2986   -6.3209   -4.5737 H   0  0  0  0  0  0  0  0  0  0  0  0
   -3.0658   -4.9302    0.9875 C   0  0  0  0  0  0  0  0  0  0  0  0
   -3.1418   -6.2523    1.4924 C   0  0  0  0  0  0  0  0  0  0  0  0
   -3.1617   -4.0623    1.6716 H   0  0  0  0  0  0  0  0  0  0  0  0
   -3.2898   -6.4029    2.5679 H   0  0  0  0  0  0  0  0  0  0  0  0
   -2.8768   -4.6550   -0.3276 N   0  0  0  0  0  0  0  0  0  0  0  0
   -6.9954   -1.3179    2.3989 C   0  0  0  0  0  0  0  0  0  0  0  0
   -7.7016   -1.8709    1.3612 C   0  0  0  0  0  0  0  0  0  0  0  0
   -7.0209   -2.3741    0.2044 C   0  0  0  0  0  0  0  0  0  0  0  0
   -5.6003   -2.2840    0.1616 C   0  0  2  0  0  0  0  0  0  0  0  0
   -4.8812   -1.6937    1.2513 C   0  0  0  0  0  0  0  0  0  0  0  0
   -5.5712   -1.2250    2.3427 C   0  0  0  0  0  0  0  0  0  0  0  0
   -8.8007   -3.0354   -0.8784 H   0  0  0  0  0  0  0  0  0  0  0  0
   -7.5115   -0.9349    3.2867 H   0  0  0  0  0  0  0  0  0  0  0  0
   -8.7940   -1.9359    1.3952 H   0  0  0  0  0  0  0  0  0  0  0  0
   -7.7069   -2.9585   -0.9012 C   0  0  0  0  0  0  0  0  0  0  0  0
   -3.7788   -1.6105    1.1931 H   0  0  0  0  0  0  0  0  0  0  0  0
   -5.0416   -0.7703    3.1875 H   0  0  0  0  0  0  0  0  0  0  0  0
   -5.5716   -3.3135   -1.9793 C   0  0  0  0  0  0  0  0  0  0  0  0
   -6.9849   -3.4181   -1.9818 C   0  0  0  0  0  0  0  0  0  0  0  0
   -4.9795   -3.6869   -2.8400 H   0  0  0  0  0  0  0  0  0  0  0  0
   -7.4826   -3.8662   -2.8496 H   0  0  0  0  0  0  0  0  0  0  0  0
   -4.8768   -2.7643   -0.9512 N   0  0  0  0  0  0  0  0  0  0  0  0
   -2.8606   -2.7511   -0.9811 Ir  0  0  0  0  0  0  0  0  0  0  0  0
  1 11  1  0  0  0  0
  1  2  1  0  0  0  0
  1 17  2  0  0  0  0
  1 79  1  0  0  0  0
  2  7  1  0  0  0  0
  2 12  1  0  0  0  0
  2  4  1  0  0  0  0
  3  5  1  0  0  0  0
  3 19  1  0  0  0  0
  3  9  1  0  0  0  0
  3 13  1  0  0  0  0
  4 14  1  0  0  0  0
  4  6  1  0  0  0  0
  4  8  1  0  0  0  0
  5  6  1  0  0  0  0
  5 10  1  0  0  0  0
  5 15  1  0  0  0  0
  6 16  1  0  0  0  0
  6 44  1  0  0  0  0
 17 18  1  0  0  0  0
 17 19  1  0  0  0  0
```

```
 17 79  1  0  0  0  0
 19 20  1  0  0  0  0
 19 21  1  0  0  0  0
 22 38  2  0  0  0  0
 22 23  1  0  0  0  0
 22 32  1  0  0  0  0
 22 79  1  0  0  0  0
 23 25  1  0  0  0  0
 23 28  1  0  0  0  0
 23 33  1  0  0  0  0
 24 30  1  0  0  0  0
 24 34  1  0  0  0  0
 24 26  1  0  0  0  0
 24 40  1  0  0  0  0
 25 27  1  0  0  0  0
 25 29  1  0  0  0  0
 25 35  1  0  0  0  0
 26 36  1  0  0  0  0
 26 31  1  0  0  0  0
 26 27  1  0  0  0  0
 27 37  1  0  0  0  0
 27 43  1  0  0  0  0
 38 40  1  0  0  0  0
 38 39  1  0  0  0  0
 38 79  1  0  0  0  0
 40 42  1  0  0  0  0
 40 41  1  0  0  0  0
 45 46  2  0  0  0  0
 45 50  1  0  0  0  0
 45 52  1  0  0  0  0
 46 53  1  0  0  0  0
 46 47  1  0  0  0  0
 47 54  2  0  0  0  0
 48 47  1  1  0  0  0
 48 49  1  0  0  0  0
 48 61  1  0  0  0  0
 49 50  2  0  0  0  0
 49 55  1  0  0  0  0
 50 56  1  0  0  0  0
 51 54  1  0  0  0  0
 54 58  1  0  0  0  0
 57 58  2  0  0  0  0
 57 59  1  0  0  0  0
 57 61  1  0  0  0  0
 58 60  1  0  0  0  0
 61 79  1  0  0  0  0
 62 69  1  0  0  0  0
 62 63  2  0  0  0  0
 62 67  1  0  0  0  0
 63 70  1  0  0  0  0
 63 64  1  0  0  0  0
 64 71  2  0  0  0  0
 65 64  1  6  0  0  0
 65 66  1  0  0  0  0
 65 78  1  0  0  0  0
 66 67  2  0  0  0  0
 66 72  1  0  0  0  0
 67 73  1  0  0  0  0
 68 71  1  0  0  0  0
 71 75  1  0  0  0  0
 74 78  1  0  0  0  0
 74 75  2  0  0  0  0
 74 76  1  0  0  0  0
 75 77  1  0  0  0  0
 78 79  1  0  0  0  0
```

```
M  END
$$$$
mol181
Optimized Catalyst 181
Optimized Catalyst 181
107116  0  0  0  0  0  0  0  0999 V2000
    0.8354    3.8774    0.3454 C   0  0  0  0  0  0  0  0  0  0  0  0
    0.7382    3.1288    1.6909 C   0  0  0  0  0  0  0  0  0  0  0  0
    0.1248    3.1809   -0.7706 C   0  0  0  0  0  0  0  0  0  0  0  0
   -2.1200    4.2760   -0.3823 C   0  0  0  0  0  0  0  0  0  0  0  0
   -1.1698    3.3471   -1.0693 C   0  0  0  0  0  0  0  0  0  0  0  0
   -2.0648    4.2578    1.1578 C   0  0  0  0  0  0  0  0  0  0  0  0
    0.7575    2.5699   -1.4238 H   0  0  0  0  0  0  0  0  0  0  0  0
   -1.6017    2.8130   -1.9192 H   0  0  0  0  0  0  0  0  0  0  0  0
    1.9046    4.0272    0.0888 H   0  0  0  0  0  0  0  0  0  0  0  0
    0.4308    4.9068    0.4632 H   0  0  0  0  0  0  0  0  0  0  0  0
    1.5894    2.4236    1.7802 H   0  0  0  0  0  0  0  0  0  0  0  0
   -3.0289    4.6382    1.5576 H   0  0  0  0  0  0  0  0  0  0  0  0
   -3.1611    4.0677   -0.7081 H   0  0  0  0  0  0  0  0  0  0  0  0
   -1.9161    5.3126   -0.7365 H   0  0  0  0  0  0  0  0  0  0  0  0
   -1.7784    2.9030    1.7191 C   0  0  0  0  0  0  0  0  0  0  0  0
   -0.5531    2.4132    1.9500 C   0  0  0  0  0  0  0  0  0  0  0  0
   -2.6663    2.3283    1.9824 H   0  0  0  0  0  0  0  0  0  0  0  0
   -0.4382    1.4509    2.4681 H   0  0  0  0  0  0  0  0  0  0  0  0
    0.8990    3.8629    2.5159 H   0  0  0  0  0  0  0  0  0  0  0  0
   -1.3064    4.9859    1.5157 H   0  0  0  0  0  0  0  0  0  0  0  0
   -1.5857   -0.8580   -0.0983 P   0  0  0  0  0  4  0  0  0  0  0  0
    1.8677   -0.3860   -0.1710 P   0  0  0  0  0  4  0  0  0  0  0  0
   -3.2210   -0.0459    0.3985 C   0  0  0  0  0  0  0  0  0  0  0  0
   -3.0347    0.3951    1.4363 H   0  0  0  0  0  0  0  0  0  0  0  0
   -3.7067    1.1158   -0.5145 C   0  0  0  0  0  0  0  0  0  0  0  0
   -4.4850   -0.9485    0.5245 C   0  0  0  0  0  0  0  0  0  0  0  0
   -3.1231    2.0378   -0.3400 H   0  0  0  0  0  0  0  0  0  0  0  0
   -5.1866    1.3224   -0.1401 C   0  0  0  0  0  0  0  0  0  0  0  0
   -4.4588   -1.5953    1.4187 H   0  0  0  0  0  0  0  0  0  0  0  0
   -5.6442    0.0612    0.6103 C   0  0  0  0  0  0  0  0  0  0  0  0
   -5.3126    2.2212    0.4881 H   0  0  0  0  0  0  0  0  0  0  0  0
   -5.8819    0.2951    1.6628 H   0  0  0  0  0  0  0  0  0  0  0  0
   -1.6510   -1.5687   -1.8663 C   0  0  0  0  0  0  0  0  0  0  0  0
   -0.7707   -2.2819   -1.9325 H   0  0  0  0  0  0  0  0  0  0  0  0
   -2.9133   -2.3616   -2.3121 C   0  0  0  0  0  0  0  0  0  0  0  0
   -1.4655   -0.4794   -2.9765 C   0  0  0  0  0  0  0  0  0  0  0  0
   -2.5826   -3.2089   -2.9488 H   0  0  0  0  0  0  0  0  0  0  0  0
   -3.7667   -1.3940   -3.1430 C   0  0  0  0  0  0  0  0  0  0  0  0
   -0.6433   -0.7940   -3.6479 H   0  0  0  0  0  0  0  0  0  0  0  0
   -2.7867   -0.3878   -3.7639 C   0  0  0  0  0  0  0  0  0  0  0  0
   -4.3445   -1.9296   -3.9153 H   0  0  0  0  0  0  0  0  0  0  0  0
   -2.6163   -0.6169   -4.8311 H   0  0  0  0  0  0  0  0  0  0  0  0
   -1.3237   -2.3522    1.0647 C   0  0  0  0  0  0  0  0  0  0  0  0
   -0.3753   -2.8624    0.7018 H   0  0  0  0  0  0  0  0  0  0  0  0
   -1.0988   -1.9342    2.5536 C   0  0  0  0  0  0  0  0  0  0  0  0
   -2.4235   -3.4586    1.1176 C   0  0  0  0  0  0  0  0  0  0  0  0
   -0.2578   -2.5308    2.9610 H   0  0  0  0  0  0  0  0  0  0  0  0
   -2.3949   -2.2851    3.2959 C   0  0  0  0  0  0  0  0  0  0  0  0
   -1.9677   -4.4344    0.8580 H   0  0  0  0  0  0  0  0  0  0  0  0
   -2.9723   -3.4991    2.5572 C   0  0  0  0  0  0  0  0  0  0  0  0
   -2.2055   -2.5062    4.3600 H   0  0  0  0  0  0  0  0  0  0  0  0
   -2.6717   -4.4377    3.0575 H   0  0  0  0  0  0  0  0  0  0  0  0
    2.3895   -1.1742    1.4828 C   0  0  0  0  0  0  0  0  0  0  0  0
    1.7580   -2.1168    1.5568 H   0  0  0  0  0  0  0  0  0  0  0  0
    3.8805   -1.5959    1.6656 C   0  0  0  0  0  0  0  0  0  0  0  0
    2.0852   -0.3019    2.7410 C   0  0  0  0  0  0  0  0  0  0  0  0
    3.9122   -2.6528    1.9975 H   0  0  0  0  0  0  0  0  0  0  0  0
    4.4976   -0.6897    2.7486 C   0  0  0  0  0  0  0  0  0  0  0  0
    1.7106   -0.9706    3.5433 H   0  0  0  0  0  0  0  0  0  0  0  0
```

```
    3.4208    0.3214    3.1660 C   0  0  0  0  0  0  0  0  0  0  0  0
    4.8223   -1.2939    3.6153 H   0  0  0  0  0  0  0  0  0  0  0  0
    3.4460    0.5191    4.2516 H   0  0  0  0  0  0  0  0  0  0  0  0
    2.0763   -1.7159   -1.5330 C   0  0  0  0  0  0  0  0  0  0  0  0
    1.4239   -1.3515   -2.3893 H   0  0  0  0  0  0  0  0  0  0  0  0
    3.5096   -1.9409   -2.1114 C   0  0  0  0  0  0  0  0  0  0  0  0
    1.6337   -3.1635   -1.1580 C   0  0  0  0  0  0  0  0  0  0  0  0
    3.4149   -2.0853   -3.2079 H   0  0  0  0  0  0  0  0  0  0  0  0
    4.0797   -3.2159   -1.4692 C   0  0  0  0  0  0  0  0  0  0  0  0
    1.1649   -3.6208   -2.0546 H   0  0  0  0  0  0  0  0  0  0  0  0
    2.9087   -3.9428   -0.7924 C   0  0  0  0  0  0  0  0  0  0  0  0
    4.5599   -3.8550   -2.2320 H   0  0  0  0  0  0  0  0  0  0  0  0
    2.8386   -4.9928   -1.1267 H   0  0  0  0  0  0  0  0  0  0  0  0
    3.0105    1.0633   -0.6286 C   0  0  0  0  0  0  0  0  0  0  0  0
    2.6323    1.9586   -0.0262 H   0  0  0  0  0  0  0  0  0  0  0  0
    4.5309    0.9547   -0.3155 C   0  0  0  0  0  0  0  0  0  0  0  0
    2.9866    1.4824   -2.1320 C   0  0  0  0  0  0  0  0  0  0  0  0
    4.7449    1.0798    0.7611 H   0  0  0  0  0  0  0  0  0  0  0  0
    5.1668    2.0920   -1.1393 C   0  0  0  0  0  0  0  0  0  0  0  0
    2.0363    1.9659   -2.4313 H   0  0  0  0  0  0  0  0  0  0  0  0
    4.1705    2.4597   -2.2522 C   0  0  0  0  0  0  0  0  0  0  0  0
    5.3822    2.9662   -0.5007 H   0  0  0  0  0  0  0  0  0  0  0  0
    3.8299    3.5044   -2.1442 H   0  0  0  0  0  0  0  0  0  0  0  0
   -5.7965    1.4951   -1.0430 H   0  0  0  0  0  0  0  0  0  0  0  0
   -3.6222    0.8959   -1.5947 H   0  0  0  0  0  0  0  0  0  0  0  0
   -4.6410   -1.6109   -0.3460 H   0  0  0  0  0  0  0  0  0  0  0  0
   -6.5673   -0.3579    0.1763 H   0  0  0  0  0  0  0  0  0  0  0  0
   -4.5162   -0.8814   -2.5129 H   0  0  0  0  0  0  0  0  0  0  0  0
   -3.1979    0.6355   -3.7411 H   0  0  0  0  0  0  0  0  0  0  0  0
   -1.1577    0.5225   -2.6124 H   0  0  0  0  0  0  0  0  0  0  0  0
   -3.4986   -2.8294   -1.5046 H   0  0  0  0  0  0  0  0  0  0  0  0
    4.2108   -1.0963   -2.0037 H   0  0  0  0  0  0  0  0  0  0  0  0
    4.8754   -2.9794   -0.7426 H   0  0  0  0  0  0  0  0  0  0  0  0
    3.0502   -3.9872    0.3009 H   0  0  0  0  0  0  0  0  0  0  0  0
    0.8815   -3.2594   -0.3584 H   0  0  0  0  0  0  0  0  0  0  0  0
    4.4902   -1.5759    0.7471 H   0  0  0  0  0  0  0  0  0  0  0  0
    5.4072   -0.1851    2.3836 H   0  0  0  0  0  0  0  0  0  0  0  0
    3.5807    1.2997    2.6807 H   0  0  0  0  0  0  0  0  0  0  0  0
    1.2945    0.4626    2.6180 H   0  0  0  0  0  0  0  0  0  0  0  0
    3.1326    0.6382   -2.8302 H   0  0  0  0  0  0  0  0  0  0  0  0
    4.6365    2.3961   -3.2497 H   0  0  0  0  0  0  0  0  0  0  0  0
    6.1381    1.7770   -1.5568 H   0  0  0  0  0  0  0  0  0  0  0  0
    4.9798   -0.0114   -0.6089 H   0  0  0  0  0  0  0  0  0  0  0  0
   -3.2490   -3.3397    0.3967 H   0  0  0  0  0  0  0  0  0  0  0  0
   -4.0747   -3.4964    2.5714 H   0  0  0  0  0  0  0  0  0  0  0  0
   -3.1031   -1.4380    3.2878 H   0  0  0  0  0  0  0  0  0  0  0  0
   -0.8085   -0.8804    2.7223 H   0  0  0  0  0  0  0  0  0  0  0  0
   -0.0330    0.6104    0.0537 Rh  0  0  0  0  0  0  0  0  0  0  0  0
  1  2  1  0  0  0  0
  1  3  1  0  0  0  0
  1  9  1  0  0  0  0
  1 10  1  0  0  0  0
  2 11  1  0  0  0  0
  2 16  1  0  0  0  0
  2 19  1  0  0  0  0
  3  5  2  0  0  0  0
  3  7  1  0  0  0  0
  3107  1  0  0  0  0
  4  5  1  0  0  0  0
  4  6  1  0  0  0  0
  4 13  1  0  0  0  0
  4 14  1  0  0  0  0
  5  8  1  0  0  0  0
  5107  1  0  0  0  0
  6 12  1  0  0  0  0
```

```
  6 15  1  0  0  0  0
  6 20  1  0  0  0  0
 15 16  2  0  0  0  0
 15 17  1  0  0  0  0
 15107  1  0  0  0  0
 16 18  1  0  0  0  0
 16107  1  0  0  0  0
 21 23  1  0  0  0  0
 21 33  1  0  0  0  0
 21 43  1  0  0  0  0
 21107  1  0  0  0  0
 22 53  1  0  0  0  0
 22 63  1  0  0  0  0
 22 73  1  0  0  0  0
 22107  1  0  0  0  0
 23 25  1  0  0  0  0
 23 26  1  0  0  0  0
 23 24  1  0  0  0  0
 25 27  1  0  0  0  0
 25 28  1  0  0  0  0
 25 84  1  0  0  0  0
 26 29  1  0  0  0  0
 26 30  1  0  0  0  0
 26 85  1  0  0  0  0
 28 30  1  0  0  0  0
 28 31  1  0  0  0  0
 28 83  1  0  0  0  0
 30 32  1  0  0  0  0
 30 86  1  0  0  0  0
 33 35  1  0  0  0  0
 33 36  1  0  0  0  0
 33 34  1  0  0  0  0
 35 37  1  0  0  0  0
 35 38  1  0  0  0  0
 35 90  1  0  0  0  0
 36 39  1  0  0  0  0
 36 40  1  0  0  0  0
 36 89  1  0  0  0  0
 38 40  1  0  0  0  0
 38 41  1  0  0  0  0
 38 87  1  0  0  0  0
 40 42  1  0  0  0  0
 40 88  1  0  0  0  0
 43 45  1  0  0  0  0
 43 46  1  0  0  0  0
 43 44  1  0  0  0  0
 45 47  1  0  0  0  0
 45 48  1  0  0  0  0
 45106  1  0  0  0  0
 46 49  1  0  0  0  0
 46 50  1  0  0  0  0
 46103  1  0  0  0  0
 48 50  1  0  0  0  0
 48 51  1  0  0  0  0
 48105  1  0  0  0  0
 50 52  1  0  0  0  0
 50104  1  0  0  0  0
 53 55  1  0  0  0  0
 53 56  1  0  0  0  0
 53 54  1  0  0  0  0
 55 57  1  0  0  0  0
 55 58  1  0  0  0  0
 55 95  1  0  0  0  0
 56 59  1  0  0  0  0
 56 60  1  0  0  0  0
```

```
 56 98  1  0  0  0  0
 58 60  1  0  0  0  0
 58 61  1  0  0  0  0
 58 96  1  0  0  0  0
 60 62  1  0  0  0  0
 60 97  1  0  0  0  0
 63 65  1  0  0  0  0
 63 66  1  0  0  0  0
 63 64  1  0  0  0  0
 65 67  1  0  0  0  0
 65 68  1  0  0  0  0
 65 91  1  0  0  0  0
 66 69  1  0  0  0  0
 66 70  1  0  0  0  0
 66 94  1  0  0  0  0
 68 70  1  0  0  0  0
 68 71  1  0  0  0  0
 68 92  1  0  0  0  0
 70 72  1  0  0  0  0
 70 93  1  0  0  0  0
 73 75  1  0  0  0  0
 73 76  1  0  0  0  0
 73 74  1  0  0  0  0
 75 77  1  0  0  0  0
 75 78  1  0  0  0  0
 75102  1  0  0  0  0
 76 79  1  0  0  0  0
 76 80  1  0  0  0  0
 76 99  1  0  0  0  0
 78 80  1  0  0  0  0
 78 81  1  0  0  0  0
 78101  1  0  0  0  0
 80 82  1  0  0  0  0
 80100  1  0  0  0  0
M  END
$$$$
mol182
Optimized Catalyst 182
Optimized Catalyst 182
107116  0  0  0  0  0  0  0  0999 V2000
    1.3950   -0.6384    0.0216 C   0  0  0  0  0  0  0  0  0  0  0  0
    0.6036   -0.5734    1.3417 C   0  0  0  0  0  0  0  0  0  0  0  0
    0.6808   -1.3916   -1.0594 C   0  0  0  0  0  0  0  0  0  0  0  0
   -0.6325    0.5696   -2.0216 C   0  0  0  0  0  0  0  0  0  0  0  0
   -0.2201   -0.8747   -1.9148 C   0  0  0  0  0  0  0  0  0  0  0  0
   -0.8646    1.3319   -0.7023 C   0  0  0  0  0  0  0  0  0  0  0  0
    0.9972   -2.4367   -1.1541 H   0  0  0  0  0  0  0  0  0  0  0  0
   -0.6192   -1.5049   -2.7344 H   0  0  0  0  0  0  0  0  0  0  0  0
    2.3784   -1.1373    0.2304 H   0  0  0  0  0  0  0  0  0  0  0  0
    1.6302    0.3959   -0.3382 H   0  0  0  0  0  0  0  0  0  0  0  0
    0.7447   -1.5450    1.8808 H   0  0  0  0  0  0  0  0  0  0  0  0
   -1.5111    2.2213   -0.9234 H   0  0  0  0  0  0  0  0  0  0  0  0
   -1.5596    0.6448   -2.6446 H   0  0  0  0  0  0  0  0  0  0  0  0
    0.1890    1.0978   -2.5851 H   0  0  0  0  0  0  0  0  0  0  0  0
   -1.5061    0.5150    0.3807 C   0  0  0  0  0  0  0  0  0  0  0  0
   -0.8663   -0.2815    1.2533 C   0  0  0  0  0  0  0  0  0  0  0  0
   -2.5816    0.6907    0.4984 H   0  0  0  0  0  0  0  0  0  0  0  0
   -1.4365   -0.7308    2.0794 H   0  0  0  0  0  0  0  0  0  0  0  0
    1.0596    0.2386    1.9760 H   0  0  0  0  0  0  0  0  0  0  0  0
    0.1081    1.7365   -0.3186 H   0  0  0  0  0  0  0  0  0  0  0  0
   -2.5631   -2.3582   -0.9011 Ir  0  0  0  0  0  0  0  0  0  0  0  0
   -5.1633   -2.0880   -0.7245 P   0  0  0  0  0  4  0  0  0  0  0  0
   -2.2952   -4.9330   -0.4178 P   0  0  0  0  0  4  0  0  0  0  0  0
   -5.3567   -0.1825   -0.7017 C   0  0  0  0  0  0  0  0  0  0  0  0
   -4.8204    0.1591    0.2259 H   0  0  0  0  0  0  0  0  0  0  0  0
```

```
   -4.6967    0.4541   -1.9469 C   0  0  0  0  0  0  0  0  0  0  0  0
   -6.8008    0.3644   -0.6204 C   0  0  0  0  0  0  0  0  0  0  0  0
   -4.0086    1.2707   -1.6061 H   0  0  0  0  0  0  0  0  0  0  0  0
   -5.8275    1.0953   -2.7770 C   0  0  0  0  0  0  0  0  0  0  0  0
   -6.9306    0.9088    0.3479 H   0  0  0  0  0  0  0  0  0  0  0  0
   -6.9751    1.3594   -1.7891 C   0  0  0  0  0  0  0  0  0  0  0  0
   -5.4826    2.0451   -3.2479 H   0  0  0  0  0  0  0  0  0  0  0  0
   -6.9095    2.4055   -1.4045 H   0  0  0  0  0  0  0  0  0  0  0  0
   -5.9881   -3.0069   -2.1853 C   0  0  0  0  0  0  0  0  0  0  0  0
   -6.2732   -4.0182   -1.7915 H   0  0  0  0  0  0  0  0  0  0  0  0
   -7.2629   -2.3336   -2.7399 C   0  0  0  0  0  0  0  0  0  0  0  0
   -5.0209   -3.1896   -3.3821 C   0  0  0  0  0  0  0  0  0  0  0  0
   -8.1772   -2.8284   -2.3400 H   0  0  0  0  0  0  0  0  0  0  0  0
   -7.1911   -2.4947   -4.2738 C   0  0  0  0  0  0  0  0  0  0  0  0
   -4.9200   -4.2818   -3.5991 H   0  0  0  0  0  0  0  0  0  0  0  0
   -5.6908   -2.5258   -4.6055 C   0  0  0  0  0  0  0  0  0  0  0  0
   -7.6740   -3.4500   -4.5854 H   0  0  0  0  0  0  0  0  0  0  0  0
   -5.4965   -3.1079   -5.5347 H   0  0  0  0  0  0  0  0  0  0  0  0
   -5.8725   -2.8584    0.8722 C   0  0  0  0  0  0  0  0  0  0  0  0
   -5.6518   -3.9518    0.8311 H   0  0  0  0  0  0  0  0  0  0  0  0
   -5.2419   -2.2421    2.1374 C   0  0  0  0  0  0  0  0  0  0  0  0
   -7.3999   -2.6764    1.0175 C   0  0  0  0  0  0  0  0  0  0  0  0
   -5.1023   -3.0606    2.8900 H   0  0  0  0  0  0  0  0  0  0  0  0
   -6.2655   -1.2352    2.6996 C   0  0  0  0  0  0  0  0  0  0  0  0
   -7.8433   -3.6630    1.3093 H   0  0  0  0  0  0  0  0  0  0  0  0
   -7.6418   -1.6627    2.1574 C   0  0  0  0  0  0  0  0  0  0  0  0
   -6.2589   -1.2552    3.8149 H   0  0  0  0  0  0  0  0  0  0  0  0
   -8.2375   -2.1456    2.9687 H   0  0  0  0  0  0  0  0  0  0  0  0
   -2.5288   -5.1144    1.4718 C   0  0  0  0  0  0  0  0  0  0  0  0
   -3.6287   -5.0609    1.6687 H   0  0  0  0  0  0  0  0  0  0  0  0
   -1.9805   -6.4238    2.0790 C   0  0  0  0  0  0  0  0  0  0  0  0
   -1.8018   -3.9663    2.2054 C   0  0  0  0  0  0  0  0  0  0  0  0
   -2.7731   -6.8707    2.7311 H   0  0  0  0  0  0  0  0  0  0  0  0
   -0.7600   -6.0584    2.9541 C   0  0  0  0  0  0  0  0  0  0  0  0
   -2.4361   -3.6470    3.0726 H   0  0  0  0  0  0  0  0  0  0  0  0
   -0.4888   -4.5563    2.7560 C   0  0  0  0  0  0  0  0  0  0  0  0
   -0.9934   -6.2629    4.0267 H   0  0  0  0  0  0  0  0  0  0  0  0
   -0.2140   -4.0723    3.7223 H   0  0  0  0  0  0  0  0  0  0  0  0
   -3.4885   -6.0647   -1.4078 C   0  0  0  0  0  0  0  0  0  0  0  0
   -3.7512   -5.4934   -2.3332 H   0  0  0  0  0  0  0  0  0  0  0  0
   -2.8918   -7.4253   -1.8305 C   0  0  0  0  0  0  0  0  0  0  0  0
   -4.7653   -6.4387   -0.6284 C   0  0  0  0  0  0  0  0  0  0  0  0
   -3.3349   -7.6856   -2.8286 H   0  0  0  0  0  0  0  0  0  0  0  0
   -3.3567   -8.4710   -0.7988 C   0  0  0  0  0  0  0  0  0  0  0  0
   -5.6071   -6.4981   -1.3669 H   0  0  0  0  0  0  0  0  0  0  0  0
   -4.5403   -7.8473   -0.0348 C   0  0  0  0  0  0  0  0  0  0  0  0
   -3.6762   -9.4046   -1.3204 H   0  0  0  0  0  0  0  0  0  0  0  0
   -5.4579   -8.4682   -0.1630 H   0  0  0  0  0  0  0  0  0  0  0  0
   -0.4753   -5.1955   -0.9469 C   0  0  0  0  0  0  0  0  0  0  0  0
    0.1138   -4.3608   -0.4754 H   0  0  0  0  0  0  0  0  0  0  0  0
    0.1908   -6.5096   -0.4819 C   0  0  0  0  0  0  0  0  0  0  0  0
   -0.3206   -5.1250   -2.4860 C   0  0  0  0  0  0  0  0  0  0  0  0
    0.7131   -6.3610    0.4920 H   0  0  0  0  0  0  0  0  0  0  0  0
    1.2167   -6.8460   -1.5839 C   0  0  0  0  0  0  0  0  0  0  0  0
    0.1241   -4.1430   -2.7804 H   0  0  0  0  0  0  0  0  0  0  0  0
    0.6549   -6.2571   -2.8884 C   0  0  0  0  0  0  0  0  0  0  0  0
    2.2005   -6.3771   -1.3458 H   0  0  0  0  0  0  0  0  0  0  0  0
    1.4783   -5.8600   -3.5260 H   0  0  0  0  0  0  0  0  0  0  0  0
   -6.1581    0.4089   -3.5882 H   0  0  0  0  0  0  0  0  0  0  0  0
   -4.1108   -0.2543   -2.5799 H   0  0  0  0  0  0  0  0  0  0  0  0
   -7.5871   -0.4165   -0.6677 H   0  0  0  0  0  0  0  0  0  0  0  0
   -7.9647    1.2338   -2.2818 H   0  0  0  0  0  0  0  0  0  0  0  0
   -7.7053   -1.6541   -4.7906 H   0  0  0  0  0  0  0  0  0  0  0  0
   -5.3025   -1.4925   -4.7503 H   0  0  0  0  0  0  0  0  0  0  0  0
   -3.9947   -2.7599   -3.2259 H   0  0  0  0  0  0  0  0  0  0  0  0
```

```
  -7.3136   -1.2460   -2.5174 H   0  0  0  0  0  0  0  0  0  0  0  0
  -1.7900   -7.4367   -1.9563 H   0  0  0  0  0  0  0  0  0  0  0  0
  -2.5346   -8.7404   -0.1005 H   0  0  0  0  0  0  0  0  0  0  0  0
  -4.3161   -7.7871    1.0524 H   0  0  0  0  0  0  0  0  0  0  0  0
  -5.0587   -5.7282    0.1652 H   0  0  0  0  0  0  0  0  0  0  0  0
  -1.7050   -7.1881    1.3247 H   0  0  0  0  0  0  0  0  0  0  0  0
   0.1300   -6.6654    2.6784 H   0  0  0  0  0  0  0  0  0  0  0  0
   0.3486   -4.3991    2.0409 H   0  0  0  0  0  0  0  0  0  0  0  0
  -1.6040   -3.0600    1.5725 H   0  0  0  0  0  0  0  0  0  0  0  0
  -1.2723   -5.2588   -3.0471 H   0  0  0  0  0  0  0  0  0  0  0  0
   0.1168   -7.0424   -3.4672 H   0  0  0  0  0  0  0  0  0  0  0  0
   1.3637   -7.9462   -1.6690 H   0  0  0  0  0  0  0  0  0  0  0  0
  -0.5171   -7.3590   -0.3781 H   0  0  0  0  0  0  0  0  0  0  0  0
  -7.9122   -2.3537    0.0876 H   0  0  0  0  0  0  0  0  0  0  0  0
  -8.2137   -0.7805    1.7942 H   0  0  0  0  0  0  0  0  0  0  0  0
  -6.0210   -0.1996    2.3757 H   0  0  0  0  0  0  0  0  0  0  0  0
  -4.2525   -1.7574    1.9764 H   0  0  0  0  0  0  0  0  0  0  0  0
  1  9  1  0  0  0  0
  1  2  1  0  0  0  0
  1  3  1  0  0  0  0
  1 10  1  0  0  0  0
  2 11  1  0  0  0  0
  2 19  1  0  0  0  0
  2 16  1  0  0  0  0
  3  5  2  0  0  0  0
  3  7  1  0  0  0  0
  3 21  1  0  0  0  0
  4  5  1  0  0  0  0
  4  6  1  0  0  0  0
  4 13  1  0  0  0  0
  4 14  1  0  0  0  0
  5  8  1  0  0  0  0
  5 21  1  0  0  0  0
  6 20  1  0  0  0  0
  6 15  1  0  0  0  0
  6 12  1  0  0  0  0
 15 17  1  0  0  0  0
 15 16  2  0  0  0  0
 15 21  1  0  0  0  0
 16 18  1  0  0  0  0
 16 21  1  0  0  0  0
 21 22  1  0  0  0  0
 21 23  1  0  0  0  0
 22 24  1  0  0  0  0
 22 44  1  0  0  0  0
 22 34  1  0  0  0  0
 23 54  1  0  0  0  0
 23 64  1  0  0  0  0
 23 74  1  0  0  0  0
 24 27  1  0  0  0  0
 24 25  1  0  0  0  0
 24 26  1  0  0  0  0
 26 28  1  0  0  0  0
 26 29  1  0  0  0  0
 26 85  1  0  0  0  0
 27 30  1  0  0  0  0
 27 31  1  0  0  0  0
 27 86  1  0  0  0  0
 29 31  1  0  0  0  0
 29 32  1  0  0  0  0
 29 84  1  0  0  0  0
 31 33  1  0  0  0  0
 31 87  1  0  0  0  0
 34 35  1  0  0  0  0
 34 36  1  0  0  0  0
```

```
 34 37  1  0  0  0  0
 36 91  1  0  0  0  0
 36 38  1  0  0  0  0
 36 39  1  0  0  0  0
 37 90  1  0  0  0  0
 37 40  1  0  0  0  0
 37 41  1  0  0  0  0
 39 41  1  0  0  0  0
 39 42  1  0  0  0  0
 39 88  1  0  0  0  0
 41 43  1  0  0  0  0
 41 89  1  0  0  0  0
 44 47  1  0  0  0  0
 44 46  1  0  0  0  0
 44 45  1  0  0  0  0
 46 49  1  0  0  0  0
 46 48  1  0  0  0  0
 46107  1  0  0  0  0
 47 50  1  0  0  0  0
 47 51  1  0  0  0  0
 47104  1  0  0  0  0
 49 52  1  0  0  0  0
 49 51  1  0  0  0  0
 49106  1  0  0  0  0
 51 53  1  0  0  0  0
 51105  1  0  0  0  0
 54 55  1  0  0  0  0
 54 56  1  0  0  0  0
 54 57  1  0  0  0  0
 56 58  1  0  0  0  0
 56 59  1  0  0  0  0
 56 96  1  0  0  0  0
 57 61  1  0  0  0  0
 57 60  1  0  0  0  0
 57 99  1  0  0  0  0
 59 62  1  0  0  0  0
 59 61  1  0  0  0  0
 59 97  1  0  0  0  0
 61 63  1  0  0  0  0
 61 98  1  0  0  0  0
 64 67  1  0  0  0  0
 64 65  1  0  0  0  0
 64 66  1  0  0  0  0
 66 69  1  0  0  0  0
 66 68  1  0  0  0  0
 66 92  1  0  0  0  0
 67 71  1  0  0  0  0
 67 95  1  0  0  0  0
 67 70  1  0  0  0  0
 69 93  1  0  0  0  0
 69 71  1  0  0  0  0
 69 72  1  0  0  0  0
 71 94  1  0  0  0  0
 71 73  1  0  0  0  0
 74 76  1  0  0  0  0
 74 75  1  0  0  0  0
 74 77  1  0  0  0  0
 76103  1  0  0  0  0
 76 78  1  0  0  0  0
 76 79  1  0  0  0  0
 77 80  1  0  0  0  0
 77 81  1  0  0  0  0
 77100  1  0  0  0  0
 79 82  1  0  0  0  0
 79 81  1  0  0  0  0
```

```
 79102  1  0  0  0  0
 81 83  1  0  0  0  0
 81101  1  0  0  0  0
M  END
$$$$
mol185
Optimized Catalyst 185
Optimized Catalyst 185
 75 82  0  0  0  0  0  0  0  0999 V2000
    0.4694   -0.2434   -0.1877 C   0  0  0  0  0  0  0  0  0  0  0  0
   -0.7803    0.6647   -0.1565 C   0  0  0  0  0  0  0  0  0  0  0  0
    0.1284   -1.6855   -0.4478 C   0  0  0  0  0  0  0  0  0  0  0  0
    0.1974   -1.5626   -2.9971 C   0  0  0  0  0  0  0  0  0  0  0  0
    0.0232   -2.2520   -1.6691 C   0  0  0  0  0  0  0  0  0  0  0  0
   -0.5409   -0.2126   -3.1283 C   0  0  0  0  0  0  0  0  0  0  0  0
    0.0072   -2.2935    0.4604 H   0  0  0  0  0  0  0  0  0  0  0  0
   -0.1289   -3.3380   -1.7620 H   0  0  0  0  0  0  0  0  0  0  0  0
    0.9955   -0.1621    0.7970 H   0  0  0  0  0  0  0  0  0  0  0  0
    1.1795    0.1162   -0.9742 H   0  0  0  0  0  0  0  0  0  0  0  0
   -1.1508    0.7150    0.8985 H   0  0  0  0  0  0  0  0  0  0  0  0
   -0.7802   -0.0387   -4.2079 H   0  0  0  0  0  0  0  0  0  0  0  0
   -0.1748   -2.2444   -3.8029 H   0  0  0  0  0  0  0  0  0  0  0  0
    1.2940   -1.3943   -3.1620 H   0  0  0  0  0  0  0  0  0  0  0  0
   -1.8126   -0.1872   -2.3260 C   0  0  0  0  0  0  0  0  0  0  0  0
   -1.9113    0.2078   -1.0405 C   0  0  0  0  0  0  0  0  0  0  0  0
   -2.7019   -0.5030   -2.8846 H   0  0  0  0  0  0  0  0  0  0  0  0
   -2.8969    0.2798   -0.5573 H   0  0  0  0  0  0  0  0  0  0  0  0
   -0.4849    1.6999   -0.4713 H   0  0  0  0  0  0  0  0  0  0  0  0
    0.1261    0.6219   -2.7970 H   0  0  0  0  0  0  0  0  0  0  0  0
   -4.6446   -2.2357   -0.5404 P   0  0  1  0  0  4  0  0  0  0  0  0
   -2.4140   -4.4987   -0.1126 P   0  0  1  0  0  4  0  0  0  0  0  0
   -0.6857   -5.0263    0.2786 C   0  0  0  0  0  0  0  0  0  0  0  0
    0.0196   -5.8122   -0.6375 C   0  0  0  0  0  0  0  0  0  0  0  0
   -0.1363   -4.7001    1.5248 C   0  0  0  0  0  0  0  0  0  0  0  0
    1.2934   -6.2820   -0.2965 C   0  0  0  0  0  0  0  0  0  0  0  0
   -0.3931   -6.0765   -1.6162 H   0  0  0  0  0  0  0  0  0  0  0  0
    1.1352   -5.1766    1.8567 C   0  0  0  0  0  0  0  0  0  0  0  0
   -0.6701   -4.0792    2.2485 H   0  0  0  0  0  0  0  0  0  0  0  0
    1.8487   -5.9683    0.9480 C   0  0  0  0  0  0  0  0  0  0  0  0
    1.8544   -6.8955   -1.0051 H   0  0  0  0  0  0  0  0  0  0  0  0
    1.5745   -4.9313    2.8262 H   0  0  0  0  0  0  0  0  0  0  0  0
    2.8423   -6.3403    1.2126 H   0  0  0  0  0  0  0  0  0  0  0  0
   -2.9902   -5.5447   -1.5202 C   0  0  0  0  0  0  0  0  0  0  0  0
   -3.4225   -6.8571   -1.3245 C   0  0  0  0  0  0  0  0  0  0  0  0
   -2.9514   -4.9706   -2.7968 C   0  0  0  0  0  0  0  0  0  0  0  0
   -3.8355   -7.6068   -2.4334 C   0  0  0  0  0  0  0  0  0  0  0  0
   -3.4462   -7.3226   -0.3366 H   0  0  0  0  0  0  0  0  0  0  0  0
   -3.3583   -5.7295   -3.8975 C   0  0  0  0  0  0  0  0  0  0  0  0
   -2.6053   -3.9304   -2.9493 H   0  0  0  0  0  0  0  0  0  0  0  0
   -3.8018   -7.0459   -3.7138 C   0  0  0  0  0  0  0  0  0  0  0  0
   -4.1775   -8.6354   -2.2978 H   0  0  0  0  0  0  0  0  0  0  0  0
   -3.3266   -5.3013   -4.9015 H   0  0  0  0  0  0  0  0  0  0  0  0
   -4.1184   -7.6379   -4.5771 H   0  0  0  0  0  0  0  0  0  0  0  0
   -4.9219   -1.1799    0.9470 C   0  0  0  0  0  0  0  0  0  0  0  0
   -6.1780   -0.6136    1.1845 C   0  0  0  0  0  0  0  0  0  0  0  0
   -3.8506   -0.9687    1.8207 C   0  0  0  0  0  0  0  0  0  0  0  0
   -6.3644    0.1661    2.3309 C   0  0  0  0  0  0  0  0  0  0  0  0
   -7.0193   -0.7599    0.5027 H   0  0  0  0  0  0  0  0  0  0  0  0
   -4.0484   -0.1897    2.9661 C   0  0  0  0  0  0  0  0  0  0  0  0
   -2.8472   -1.3869    1.6166 H   0  0  0  0  0  0  0  0  0  0  0  0
   -5.3040    0.3735    3.2211 C   0  0  0  0  0  0  0  0  0  0  0  0
   -7.3390    0.6177    2.5300 H   0  0  0  0  0  0  0  0  0  0  0  0
   -3.2231   -0.0150    3.6591 H   0  0  0  0  0  0  0  0  0  0  0  0
   -5.4565    0.9826    4.1168 H   0  0  0  0  0  0  0  0  0  0  0  0
   -5.7884   -3.6824   -0.3948 C   0  0  0  0  0  0  0  0  0  0  0  0
```

```
   -6.4677   -3.9163    0.8058 C   0  0  0  0  0  0  0  0  0  0  0  0
   -6.0047   -4.4970   -1.5099 C   0  0  0  0  0  0  0  0  0  0  0  0
   -7.3582   -4.9913    0.8920 C   0  0  0  0  0  0  0  0  0  0  0  0
   -6.3247   -3.2833    1.6875 H   0  0  0  0  0  0  0  0  0  0  0  0
   -6.8931   -5.5735   -1.4142 C   0  0  0  0  0  0  0  0  0  0  0  0
   -5.4942   -4.3254   -2.4621 H   0  0  0  0  0  0  0  0  0  0  0  0
   -7.5693   -5.8207   -0.2153 C   0  0  0  0  0  0  0  0  0  0  0  0
   -7.8964   -5.1802    1.8236 H   0  0  0  0  0  0  0  0  0  0  0  0
   -7.0614   -6.2178   -2.2797 H   0  0  0  0  0  0  0  0  0  0  0  0
   -8.2674   -6.6594   -0.1438 H   0  0  0  0  0  0  0  0  0  0  0  0
   -5.2276   -1.3087   -2.0603 C   0  0  0  0  0  0  0  0  0  0  0  0
   -4.9885   -0.2194   -1.9667 H   0  0  0  0  0  0  0  0  0  0  0  0
   -4.7276   -1.7188   -2.9731 H   0  0  0  0  0  0  0  0  0  0  0  0
   -6.3354   -1.4192   -2.1741 H   0  0  0  0  0  0  0  0  0  0  0  0
   -3.3872   -4.9640    1.4202 C   0  0  0  0  0  0  0  0  0  0  0  0
   -3.4448   -4.1055    2.1305 H   0  0  0  0  0  0  0  0  0  0  0  0
   -4.4205   -5.2918    1.1457 H   0  0  0  0  0  0  0  0  0  0  0  0
   -2.8817   -5.8185    1.9399 H   0  0  0  0  0  0  0  0  0  0  0  0
   -2.4648   -2.4013   -0.7706 Rh  0  0  0  0  0  0  0  0  0  0  0  0
  1  2  1  0  0  0  0
  1  9  1  0  0  0  0
  1  3  1  0  0  0  0
  1 10  1  0  0  0  0
  2 11  1  0  0  0  0
  2 16  1  0  0  0  0
  2 19  1  0  0  0  0
  3  7  1  0  0  0  0
  3  5  2  0  0  0  0
  3 75  1  0  0  0  0
  4  5  1  0  0  0  0
  4  6  1  0  0  0  0
  4 13  1  0  0  0  0
  4 14  1  0  0  0  0
  5  8  1  0  0  0  0
  5 75  1  0  0  0  0
  6 20  1  0  0  0  0
  6 15  1  0  0  0  0
  6 12  1  0  0  0  0
 15 16  2  0  0  0  0
 15 17  1  0  0  0  0
 15 75  1  0  0  0  0
 16 18  1  0  0  0  0
 16 75  1  0  0  0  0
 21 56  1  0  0  0  0
 21 45  1  0  0  0  0
 21 67  1  6  0  0  0
 21 75  1  0  0  0  0
 22 23  1  0  0  0  0
 22 71  1  1  0  0  0
 22 34  1  0  0  0  0
 22 75  1  0  0  0  0
 23 25  2  0  0  0  0
 23 24  1  0  0  0  0
 24 26  2  0  0  0  0
 24 27  1  0  0  0  0
 25 28  1  0  0  0  0
 25 29  1  0  0  0  0
 26 30  1  0  0  0  0
 26 31  1  0  0  0  0
 28 32  1  0  0  0  0
 28 30  2  0  0  0  0
 30 33  1  0  0  0  0
 34 35  1  0  0  0  0
 34 36  2  0  0  0  0
 35 38  1  0  0  0  0
```

```
 35 37  2  0  0  0  0
 36 39  1  0  0  0  0
 36 40  1  0  0  0  0
 37 42  1  0  0  0  0
 37 41  1  0  0  0  0
 39 41  2  0  0  0  0
 39 43  1  0  0  0  0
 41 44  1  0  0  0  0
 45 46  2  0  0  0  0
 45 47  1  0  0  0  0
 46 48  1  0  0  0  0
 46 49  1  0  0  0  0
 47 50  2  0  0  0  0
 47 51  1  0  0  0  0
 48 53  1  0  0  0  0
 48 52  2  0  0  0  0
 50 52  1  0  0  0  0
 50 54  1  0  0  0  0
 52 55  1  0  0  0  0
 56 57  2  0  0  0  0
 56 58  1  0  0  0  0
 57 59  1  0  0  0  0
 57 60  1  0  0  0  0
 58 61  2  0  0  0  0
 58 62  1  0  0  0  0
 59 64  1  0  0  0  0
 59 63  2  0  0  0  0
 61 63  1  0  0  0  0
 61 65  1  0  0  0  0
 63 66  1  0  0  0  0
 67 68  1  0  0  0  0
 67 69  1  0  0  0  0
 67 70  1  0  0  0  0
 71 74  1  0  0  0  0
 71 72  1  0  0  0  0
 71 73  1  0  0  0  0
M  END
$$$$
mol186
Optimized Catalyst 186
Optimized Catalyst 186
 75 82  0  0  0  0  0  0  0  0999 V2000
    0.7550   -0.2240    0.2780 C   0  0  0  0  0  0  0  0  0  0  0  0
   -0.5386    0.5644    0.5609 C   0  0  0  0  0  0  0  0  0  0  0  0
    0.5353   -1.6213   -0.2288 C   0  0  0  0  0  0  0  0  0  0  0  0
    0.3619   -1.0513   -2.7194 C   0  0  0  0  0  0  0  0  0  0  0  0
    0.3715   -1.9665   -1.5246 C   0  0  0  0  0  0  0  0  0  0  0  0
   -0.3250    0.3190   -2.5627 C   0  0  0  0  0  0  0  0  0  0  0  0
    0.6316   -2.4045    0.5421 H   0  0  0  0  0  0  0  0  0  0  0  0
    0.3816   -3.0364   -1.8052 H   0  0  0  0  0  0  0  0  0  0  0  0
    1.3443   -0.2734    1.2333 H   0  0  0  0  0  0  0  0  0  0  0  0
    1.3903    0.3333   -0.4594 H   0  0  0  0  0  0  0  0  0  0  0  0
   -0.8989    0.2922    1.5881 H   0  0  0  0  0  0  0  0  0  0  0  0
   -0.5575    0.7056   -3.5921 H   0  0  0  0  0  0  0  0  0  0  0  0
   -0.1147   -1.5856   -3.5826 H   0  0  0  0  0  0  0  0  0  0  0  0
    1.4406   -0.8667   -2.9942 H   0  0  0  0  0  0  0  0  0  0  0  0
   -1.5929    0.2958   -1.7581 C   0  0  0  0  0  0  0  0  0  0  0  0
   -1.6751    0.4048   -0.4142 C   0  0  0  0  0  0  0  0  0  0  0  0
   -2.5113    0.2851   -2.3664 H   0  0  0  0  0  0  0  0  0  0  0  0
   -2.6656    0.5523    0.0573 H   0  0  0  0  0  0  0  0  0  0  0  0
   -0.2782    1.6619    0.5777 H   0  0  0  0  0  0  0  0  0  0  0  0
    0.3839    1.0543   -2.0990 H   0  0  0  0  0  0  0  0  0  0  0  0
   -4.8878   -2.2103   -0.5964 P   0  0  1  0  0  4  0  0  0  0  0  0
   -2.2204   -4.9084   -0.1604 P   0  0  1  0  0  4  0  0  0  0  0  0
   -0.4656   -5.4631   -0.0483 C   0  0  0  0  0  0  0  0  0  0  0  0
```

```
    0.1214   -6.1019   -1.1461 C   0  0  0  0  0  0  0  0  0  0  0  0
    0.2419   -5.2858    1.1482 C   0  0  0  0  0  0  0  0  0  0  0  0
    1.4370   -6.5677   -1.0424 C   0  0  0  0  0  0  0  0  0  0  0  0
   -0.4164   -6.2595   -2.0863 H   0  0  0  0  0  0  0  0  0  0  0  0
    1.5549   -5.7547    1.2400 C   0  0  0  0  0  0  0  0  0  0  0  0
   -0.1972   -4.7976    2.0215 H   0  0  0  0  0  0  0  0  0  0  0  0
    2.1529   -6.3955    0.1467 C   0  0  0  0  0  0  0  0  0  0  0  0
    1.9041   -7.0734   -1.8920 H   0  0  0  0  0  0  0  0  0  0  0  0
    2.1175   -5.6277    2.1691 H   0  0  0  0  0  0  0  0  0  0  0  0
    3.1807   -6.7636    0.2254 H   0  0  0  0  0  0  0  0  0  0  0  0
   -3.0044   -5.7849   -1.5882 C   0  0  0  0  0  0  0  0  0  0  0  0
   -3.5249   -7.0690   -1.4201 C   0  0  0  0  0  0  0  0  0  0  0  0
   -3.0355   -5.1485   -2.8363 C   0  0  0  0  0  0  0  0  0  0  0  0
   -4.1041   -7.7196   -2.5189 C   0  0  0  0  0  0  0  0  0  0  0  0
   -3.4956   -7.5937   -0.4618 H   0  0  0  0  0  0  0  0  0  0  0  0
   -3.6093   -5.8069   -3.9258 C   0  0  0  0  0  0  0  0  0  0  0  0
   -2.6097   -4.1471   -2.9924 H   0  0  0  0  0  0  0  0  0  0  0  0
   -4.1478   -7.0918   -3.7656 C   0  0  0  0  0  0  0  0  0  0  0  0
   -4.5138   -8.7265   -2.3996 H   0  0  0  0  0  0  0  0  0  0  0  0
   -3.6326   -5.3288   -4.9085 H   0  0  0  0  0  0  0  0  0  0  0  0
   -4.5969   -7.6041   -4.6225 H   0  0  0  0  0  0  0  0  0  0  0  0
   -5.4234   -0.8326    0.5145 C   0  0  0  0  0  0  0  0  0  0  0  0
   -6.5713   -0.0984    0.2094 C   0  0  0  0  0  0  0  0  0  0  0  0
   -4.6889   -0.5756    1.6801 C   0  0  0  0  0  0  0  0  0  0  0  0
   -6.9874    0.9142    1.0849 C   0  0  0  0  0  0  0  0  0  0  0  0
   -7.1668   -0.2820   -0.6879 H   0  0  0  0  0  0  0  0  0  0  0  0
   -5.1142    0.4323    2.5480 C   0  0  0  0  0  0  0  0  0  0  0  0
   -3.7870   -1.1444    1.9404 H   0  0  0  0  0  0  0  0  0  0  0  0
   -6.2641    1.1777    2.2505 C   0  0  0  0  0  0  0  0  0  0  0  0
   -7.8832    1.4978    0.8535 H   0  0  0  0  0  0  0  0  0  0  0  0
   -4.5532    0.6425    3.4622 H   0  0  0  0  0  0  0  0  0  0  0  0
   -6.5942    1.9667    2.9336 H   0  0  0  0  0  0  0  0  0  0  0  0
   -5.7977   -3.7263   -0.0695 C   0  0  0  0  0  0  0  0  0  0  0  0
   -6.2967   -3.8012    1.2360 C   0  0  0  0  0  0  0  0  0  0  0  0
   -6.0355   -4.7517   -0.9916 C   0  0  0  0  0  0  0  0  0  0  0  0
   -7.0295   -4.9278    1.6240 C   0  0  0  0  0  0  0  0  0  0  0  0
   -6.1451   -2.9988    1.9656 H   0  0  0  0  0  0  0  0  0  0  0  0
   -6.7675   -5.8741   -0.5925 C   0  0  0  0  0  0  0  0  0  0  0  0
   -5.6718   -4.7114   -2.0217 H   0  0  0  0  0  0  0  0  0  0  0  0
   -7.2672   -5.9619    0.7119 C   0  0  0  0  0  0  0  0  0  0  0  0
   -7.4300   -4.9939    2.6393 H   0  0  0  0  0  0  0  0  0  0  0  0
   -6.9596   -6.6792   -1.3057 H   0  0  0  0  0  0  0  0  0  0  0  0
   -7.8522   -6.8351    1.0160 H   0  0  0  0  0  0  0  0  0  0  0  0
   -5.4408   -1.8081   -2.3434 C   0  0  0  0  0  0  0  0  0  0  0  0
   -5.1927   -0.7476   -2.6024 H   0  0  0  0  0  0  0  0  0  0  0  0
   -4.9644   -2.4874   -3.0914 H   0  0  0  0  0  0  0  0  0  0  0  0
   -6.5537   -1.9360   -2.4280 H   0  0  0  0  0  0  0  0  0  0  0  0
   -3.0864   -5.4404    1.4136 C   0  0  0  0  0  0  0  0  0  0  0  0
   -2.8570   -4.7492    2.2607 H   0  0  0  0  0  0  0  0  0  0  0  0
   -4.1960   -5.4765    1.2623 H   0  0  0  0  0  0  0  0  0  0  0  0
   -2.7395   -6.4669    1.7091 H   0  0  0  0  0  0  0  0  0  0  0  0
   -2.2904   -2.3429   -0.5538 Ir  0  0  0  0  0  0  0  0  0  0  0  0
  1  2  1  0  0  0  0
  1  9  1  0  0  0  0
  1  3  1  0  0  0  0
  1 10  1  0  0  0  0
  2 19  1  0  0  0  0
  2 11  1  0  0  0  0
  2 16  1  0  0  0  0
  3  7  1  0  0  0  0
  3  5  2  0  0  0  0
  3 75  1  0  0  0  0
  4  6  1  0  0  0  0
  4  5  1  0  0  0  0
  4 13  1  0  0  0  0
```

```
  4 14  1  0  0  0  0
  5  8  1  0  0  0  0
  5 75  1  0  0  0  0
  6 20  1  0  0  0  0
  6 15  1  0  0  0  0
  6 12  1  0  0  0  0
 15 16  2  0  0  0  0
 15 17  1  0  0  0  0
 15 75  1  0  0  0  0
 16 18  1  0  0  0  0
 16 75  1  0  0  0  0
 21 75  1  0  0  0  0
 21 56  1  0  0  0  0
 21 45  1  0  0  0  0
 21 67  1  6  0  0  0
 22 23  1  0  0  0  0
 22 71  1  1  0  0  0
 22 34  1  0  0  0  0
 22 75  1  0  0  0  0
 23 25  2  0  0  0  0
 23 24  1  0  0  0  0
 24 26  2  0  0  0  0
 24 27  1  0  0  0  0
 25 28  1  0  0  0  0
 25 29  1  0  0  0  0
 26 30  1  0  0  0  0
 26 31  1  0  0  0  0
 28 32  1  0  0  0  0
 28 30  2  0  0  0  0
 30 33  1  0  0  0  0
 34 35  1  0  0  0  0
 34 36  2  0  0  0  0
 35 38  1  0  0  0  0
 35 37  2  0  0  0  0
 36 39  1  0  0  0  0
 36 40  1  0  0  0  0
 37 42  1  0  0  0  0
 37 41  1  0  0  0  0
 39 41  2  0  0  0  0
 39 43  1  0  0  0  0
 41 44  1  0  0  0  0
 45 47  1  0  0  0  0
 45 46  2  0  0  0  0
 46 48  1  0  0  0  0
 46 49  1  0  0  0  0
 47 51  1  0  0  0  0
 47 50  2  0  0  0  0
 48 52  2  0  0  0  0
 48 53  1  0  0  0  0
 50 54  1  0  0  0  0
 50 52  1  0  0  0  0
 52 55  1  0  0  0  0
 56 57  2  0  0  0  0
 56 58  1  0  0  0  0
 57 59  1  0  0  0  0
 57 60  1  0  0  0  0
 58 61  2  0  0  0  0
 58 62  1  0  0  0  0
 59 64  1  0  0  0  0
 59 63  2  0  0  0  0
 61 63  1  0  0  0  0
 61 65  1  0  0  0  0
 63 66  1  0  0  0  0
 67 68  1  0  0  0  0
 67 69  1  0  0  0  0
```

```
 67 70  1  0  0  0  0
 71 74  1  0  0  0  0
 71 72  1  0  0  0  0
 71 73  1  0  0  0  0
M  END
$$$$
mol187
Optimized Catalyst 187
Optimized Catalyst 187
 81 88  0  0  0  0  0  0  0  0999 V2000
    0.3999   -0.1635   -0.2430 C   0  0  0  0  0  0  0  0  0  0  0  0
   -0.8530    0.7389   -0.3256 C   0  0  0  0  0  0  0  0  0  0  0  0
    0.0746   -1.6192   -0.4376 C   0  0  0  0  0  0  0  0  0  0  0  0
    0.2471   -1.6287   -2.9793 C   0  0  0  0  0  0  0  0  0  0  0  0
    0.0226   -2.2524   -1.6272 C   0  0  0  0  0  0  0  0  0  0  0  0
   -0.5427   -0.3213   -3.2160 C   0  0  0  0  0  0  0  0  0  0  0  0
   -0.1025   -2.1730    0.4950 H   0  0  0  0  0  0  0  0  0  0  0  0
   -0.1388   -3.3401   -1.6663 H   0  0  0  0  0  0  0  0  0  0  0  0
    0.8770   -0.0230    0.7591 H   0  0  0  0  0  0  0  0  0  0  0  0
    1.1437    0.1542   -1.0158 H   0  0  0  0  0  0  0  0  0  0  0  0
   -1.2453    0.8969    0.7098 H   0  0  0  0  0  0  0  0  0  0  0  0
   -0.7686   -0.2291   -4.3078 H   0  0  0  0  0  0  0  0  0  0  0  0
   -0.0473   -2.3662   -3.7669 H   0  0  0  0  0  0  0  0  0  0  0  0
    1.3423   -1.4173   -3.0926 H   0  0  0  0  0  0  0  0  0  0  0  0
   -1.8275   -0.2952   -2.4347 C   0  0  0  0  0  0  0  0  0  0  0  0
   -1.9588    0.1783   -1.1805 C   0  0  0  0  0  0  0  0  0  0  0  0
   -2.6912   -0.6995   -2.9754 H   0  0  0  0  0  0  0  0  0  0  0  0
   -2.9468    0.2245   -0.7017 H   0  0  0  0  0  0  0  0  0  0  0  0
   -0.5559    1.7380   -0.7382 H   0  0  0  0  0  0  0  0  0  0  0  0
    0.0871    0.5575   -2.9294 H   0  0  0  0  0  0  0  0  0  0  0  0
   -4.7237   -2.3061   -0.4815 P   0  0  1  0  0  4  0  0  0  0  0  0
   -2.4687   -4.5964   -0.1152 P   0  0  1  0  0  4  0  0  0  0  0  0
   -0.7178   -5.0742    0.2314 C   0  0  0  0  0  0  0  0  0  0  0  0
   -0.0622   -5.9598   -0.6288 C   0  0  0  0  0  0  0  0  0  0  0  0
   -0.1005   -4.6105    1.4002 C   0  0  0  0  0  0  0  0  0  0  0  0
    1.2287   -6.3953   -0.3057 C   0  0  0  0  0  0  0  0  0  0  0  0
   -0.5239   -6.3247   -1.5514 H   0  0  0  0  0  0  0  0  0  0  0  0
    1.1879   -5.0522    1.7135 C   0  0  0  0  0  0  0  0  0  0  0  0
   -0.5971   -3.9072    2.0751 H   0  0  0  0  0  0  0  0  0  0  0  0
    1.8514   -5.9456    0.8628 C   0  0  0  0  0  0  0  0  0  0  0  0
    1.7500   -7.0884   -0.9698 H   0  0  0  0  0  0  0  0  0  0  0  0
    1.6805   -4.6998    2.6223 H   0  0  0  0  0  0  0  0  0  0  0  0
    2.8593   -6.2891    1.1118 H   0  0  0  0  0  0  0  0  0  0  0  0
   -3.0322   -5.5798   -1.5758 C   0  0  0  0  0  0  0  0  0  0  0  0
   -3.5233   -6.8805   -1.4568 C   0  0  0  0  0  0  0  0  0  0  0  0
   -2.9219   -4.9568   -2.8262 C   0  0  0  0  0  0  0  0  0  0  0  0
   -3.9115   -7.5698   -2.6131 C   0  0  0  0  0  0  0  0  0  0  0  0
   -3.6198   -7.3835   -0.4928 H   0  0  0  0  0  0  0  0  0  0  0  0
   -3.3055   -5.6548   -3.9745 C   0  0  0  0  0  0  0  0  0  0  0  0
   -2.5397   -3.9214   -2.9220 H   0  0  0  0  0  0  0  0  0  0  0  0
   -3.7997   -6.9614   -3.8668 C   0  0  0  0  0  0  0  0  0  0  0  0
   -4.2968   -8.5888   -2.5345 H   0  0  0  0  0  0  0  0  0  0  0  0
   -3.2162   -5.1856   -4.9565 H   0  0  0  0  0  0  0  0  0  0  0  0
   -4.0961   -7.5067   -4.7674 H   0  0  0  0  0  0  0  0  0  0  0  0
   -4.9551   -1.2476    1.0138 C   0  0  0  0  0  0  0  0  0  0  0  0
   -6.2146   -0.7440    1.3536 C   0  0  0  0  0  0  0  0  0  0  0  0
   -3.8294   -0.9696    1.7977 C   0  0  0  0  0  0  0  0  0  0  0  0
   -6.3455    0.0455    2.5013 C   0  0  0  0  0  0  0  0  0  0  0  0
   -7.1048   -0.9482    0.7538 H   0  0  0  0  0  0  0  0  0  0  0  0
   -3.9704   -0.1800    2.9442 C   0  0  0  0  0  0  0  0  0  0  0  0
   -2.8236   -1.3479    1.5270 H   0  0  0  0  0  0  0  0  0  0  0  0
   -5.2273    0.3254    3.2956 C   0  0  0  0  0  0  0  0  0  0  0  0
   -7.3229    0.4485    2.7770 H   0  0  0  0  0  0  0  0  0  0  0  0
   -3.0992    0.0474    3.5621 H   0  0  0  0  0  0  0  0  0  0  0  0
   -5.3358    0.9430    4.1917 H   0  0  0  0  0  0  0  0  0  0  0  0
```

```
   -5.8490   -3.7632   -0.3156 C   0  0  0  0  0  0  0  0  0  0  0  0
   -6.5412   -3.9846    0.8798 C   0  0  0  0  0  0  0  0  0  0  0  0
   -6.0348   -4.6049   -1.4171 C   0  0  0  0  0  0  0  0  0  0  0  0
   -7.4236   -5.0661    0.9715 C   0  0  0  0  0  0  0  0  0  0  0  0
   -6.4107   -3.3415    1.7561 H   0  0  0  0  0  0  0  0  0  0  0  0
   -6.9123   -5.6892   -1.3141 C   0  0  0  0  0  0  0  0  0  0  0  0
   -5.5074   -4.4481   -2.3634 H   0  0  0  0  0  0  0  0  0  0  0  0
   -7.6091   -5.9180   -0.1229 C   0  0  0  0  0  0  0  0  0  0  0  0
   -7.9737   -5.2427    1.8987 H   0  0  0  0  0  0  0  0  0  0  0  0
   -7.0548   -6.3546   -2.1680 H   0  0  0  0  0  0  0  0  0  0  0  0
   -8.3017   -6.7607   -0.0476 H   0  0  0  0  0  0  0  0  0  0  0  0
   -5.2573   -1.3822   -2.0468 C   0  0  0  0  0  0  0  0  0  0  0  0
   -4.8440   -0.3388   -1.9964 H   0  0  0  0  0  0  0  0  0  0  0  0
   -4.7968   -1.9130   -2.9235 H   0  0  0  0  0  0  0  0  0  0  0  0
   -3.4641   -5.1070    1.4143 C   0  0  0  0  0  0  0  0  0  0  0  0
   -3.5375   -4.2256    2.1027 H   0  0  0  0  0  0  0  0  0  0  0  0
   -4.4948   -5.4005    1.0811 H   0  0  0  0  0  0  0  0  0  0  0  0
   -2.5379   -2.4773   -0.7322 Rh  0  0  0  0  0  0  0  0  0  0  0  0
   -6.7690   -1.3151   -2.2145 C   0  0  0  0  0  0  0  0  0  0  0  0
   -6.9919   -0.9156   -3.2388 H   0  0  0  0  0  0  0  0  0  0  0  0
   -7.2616   -2.3127   -2.1325 H   0  0  0  0  0  0  0  0  0  0  0  0
   -7.2358   -0.6153   -1.4824 H   0  0  0  0  0  0  0  0  0  0  0  0
   -2.8393   -6.2789    2.1619 C   0  0  0  0  0  0  0  0  0  0  0  0
   -3.5668   -6.6328    2.9369 H   0  0  0  0  0  0  0  0  0  0  0  0
   -2.6041   -7.1448    1.5012 H   0  0  0  0  0  0  0  0  0  0  0  0
   -1.9036   -5.9826    2.6956 H   0  0  0  0  0  0  0  0  0  0  0  0
  1  9  1  0  0  0  0
  1  2  1  0  0  0  0
  1  3  1  0  0  0  0
  1 10  1  0  0  0  0
  2 11  1  0  0  0  0
  2 16  1  0  0  0  0
  2 19  1  0  0  0  0
  3  7  1  0  0  0  0
  3  5  2  0  0  0  0
  3 73  1  0  0  0  0
  4  5  1  0  0  0  0
  4  6  1  0  0  0  0
  4 13  1  0  0  0  0
  4 14  1  0  0  0  0
  5  8  1  0  0  0  0
  5 73  1  0  0  0  0
  6 20  1  0  0  0  0
  6 15  1  0  0  0  0
  6 12  1  0  0  0  0
 15 16  2  0  0  0  0
 15 17  1  0  0  0  0
 15 73  1  0  0  0  0
 16 18  1  0  0  0  0
 16 73  1  0  0  0  0
 21 56  1  0  0  0  0
 21 45  1  0  0  0  0
 21 67  1  6  0  0  0
 21 73  1  0  0  0  0
 22 23  1  0  0  0  0
 22 70  1  1  0  0  0
 22 34  1  0  0  0  0
 22 73  1  0  0  0  0
 23 25  2  0  0  0  0
 23 24  1  0  0  0  0
 24 26  2  0  0  0  0
 24 27  1  0  0  0  0
 25 28  1  0  0  0  0
 25 29  1  0  0  0  0
 26 30  1  0  0  0  0
```

```
 26 31  1  0  0  0  0
 28 32  1  0  0  0  0
 28 30  2  0  0  0  0
 30 33  1  0  0  0  0
 34 35  1  0  0  0  0
 34 36  2  0  0  0  0
 35 38  1  0  0  0  0
 35 37  2  0  0  0  0
 36 39  1  0  0  0  0
 36 40  1  0  0  0  0
 37 42  1  0  0  0  0
 37 41  1  0  0  0  0
 39 41  2  0  0  0  0
 39 43  1  0  0  0  0
 41 44  1  0  0  0  0
 45 46  2  0  0  0  0
 45 47  1  0  0  0  0
 46 48  1  0  0  0  0
 46 49  1  0  0  0  0
 47 50  2  0  0  0  0
 47 51  1  0  0  0  0
 48 53  1  0  0  0  0
 48 52  2  0  0  0  0
 50 52  1  0  0  0  0
 50 54  1  0  0  0  0
 52 55  1  0  0  0  0
 56 57  2  0  0  0  0
 56 58  1  0  0  0  0
 57 59  1  0  0  0  0
 57 60  1  0  0  0  0
 58 61  2  0  0  0  0
 58 62  1  0  0  0  0
 59 64  1  0  0  0  0
 59 63  2  0  0  0  0
 61 63  1  0  0  0  0
 61 65  1  0  0  0  0
 63 66  1  0  0  0  0
 67 68  1  0  0  0  0
 67 69  1  0  0  0  0
 67 74  1  0  0  0  0
 70 71  1  0  0  0  0
 70 78  1  0  0  0  0
 70 72  1  0  0  0  0
 74 76  1  0  0  0  0
 74 77  1  0  0  0  0
 74 75  1  0  0  0  0
 78 81  1  0  0  0  0
 78 79  1  0  0  0  0
 78 80  1  0  0  0  0
M  END
$$$$
mol188
Optimized Catalyst 188
Optimized Catalyst 188
 81 88  0  0  0  0  0  0  0  0999 V2000
    0.7568   -0.2717    0.2463 C   0  0  0  0  0  0  0  0  0  0  0  0
   -0.5206    0.4903    0.6490 C   0  0  0  0  0  0  0  0  0  0  0  0
    0.5082   -1.6472   -0.3014 C   0  0  0  0  0  0  0  0  0  0  0  0
    0.1673   -0.9752   -2.7458 C   0  0  0  0  0  0  0  0  0  0  0  0
    0.2451   -1.9422   -1.5930 C   0  0  0  0  0  0  0  0  0  0  0  0
   -0.5211    0.3794   -2.4855 C   0  0  0  0  0  0  0  0  0  0  0  0
    0.6463   -2.4584    0.4311 H   0  0  0  0  0  0  0  0  0  0  0  0
    0.2341   -3.0012   -1.9160 H   0  0  0  0  0  0  0  0  0  0  0  0
    1.4086   -0.3556    1.1575 H   0  0  0  0  0  0  0  0  0  0  0  0
    1.3370    0.3220   -0.5075 H   0  0  0  0  0  0  0  0  0  0  0  0
```

```
   -0.8085    0.1680    1.6839 H   0  0  0  0  0  0  0  0  0  0  0  0
   -0.8236    0.8088   -3.4784 H   0  0  0  0  0  0  0  0  0  0  0  0
   -0.3494   -1.4733   -3.6081 H   0  0  0  0  0  0  0  0  0  0  0  0
    1.2279   -0.7680   -3.0683 H   0  0  0  0  0  0  0  0  0  0  0  0
   -1.7328    0.3030   -1.5993 C   0  0  0  0  0  0  0  0  0  0  0  0
   -1.7197    0.3623   -0.2502 C   0  0  0  0  0  0  0  0  0  0  0  0
   -2.6910    0.3162   -2.1436 H   0  0  0  0  0  0  0  0  0  0  0  0
   -2.6760    0.4518    0.2993 H   0  0  0  0  0  0  0  0  0  0  0  0
   -0.2680    1.5885    0.6960 H   0  0  0  0  0  0  0  0  0  0  0  0
    0.2098    1.1015   -2.0360 H   0  0  0  0  0  0  0  0  0  0  0  0
   -4.9913   -2.2210   -0.6240 P   0  0  1  0  0  4  0  0  0  0  0  0
   -2.2789   -4.9576   -0.2320 P   0  0  1  0  0  4  0  0  0  0  0  0
   -0.5049   -5.4244   -0.0517 C   0  0  0  0  0  0  0  0  0  0  0  0
    0.1365   -6.1068   -1.0904 C   0  0  0  0  0  0  0  0  0  0  0  0
    0.1681   -5.1239    1.1413 C   0  0  0  0  0  0  0  0  0  0  0  0
    1.4727   -6.4933   -0.9306 C   0  0  0  0  0  0  0  0  0  0  0  0
   -0.3712   -6.3566   -2.0277 H   0  0  0  0  0  0  0  0  0  0  0  0
    1.5000   -5.5167    1.2897 C   0  0  0  0  0  0  0  0  0  0  0  0
   -0.3143   -4.5974    1.9688 H   0  0  0  0  0  0  0  0  0  0  0  0
    2.1527   -6.2017    0.2555 C   0  0  0  0  0  0  0  0  0  0  0  0
    1.9839   -7.0288   -1.7354 H   0  0  0  0  0  0  0  0  0  0  0  0
    2.0359   -5.2941    2.2162 H   0  0  0  0  0  0  0  0  0  0  0  0
    3.1957   -6.5096    0.3788 H   0  0  0  0  0  0  0  0  0  0  0  0
   -2.9730   -5.8749   -1.6818 C   0  0  0  0  0  0  0  0  0  0  0  0
   -3.3263   -7.2203   -1.5791 C   0  0  0  0  0  0  0  0  0  0  0  0
   -3.0994   -5.1880   -2.8979 C   0  0  0  0  0  0  0  0  0  0  0  0
   -3.8426   -7.8807   -2.7035 C   0  0  0  0  0  0  0  0  0  0  0  0
   -3.2051   -7.7886   -0.6546 H   0  0  0  0  0  0  0  0  0  0  0  0
   -3.6079   -5.8547   -4.0139 C   0  0  0  0  0  0  0  0  0  0  0  0
   -2.7936   -4.1347   -3.0102 H   0  0  0  0  0  0  0  0  0  0  0  0
   -3.9854   -7.2019   -3.9150 C   0  0  0  0  0  0  0  0  0  0  0  0
   -4.1243   -8.9351   -2.6314 H   0  0  0  0  0  0  0  0  0  0  0  0
   -3.7041   -5.3337   -4.9701 H   0  0  0  0  0  0  0  0  0  0  0  0
   -4.3846   -7.7213   -4.7921 H   0  0  0  0  0  0  0  0  0  0  0  0
   -5.3043   -0.8021    0.5234 C   0  0  0  0  0  0  0  0  0  0  0  0
   -6.0828    0.2885    0.1325 C   0  0  0  0  0  0  0  0  0  0  0  0
   -4.7510   -0.8628    1.8127 C   0  0  0  0  0  0  0  0  0  0  0  0
   -6.3195    1.3268    1.0451 C   0  0  0  0  0  0  0  0  0  0  0  0
   -6.5217    0.3764   -0.8641 H   0  0  0  0  0  0  0  0  0  0  0  0
   -4.9953    0.1739    2.7150 C   0  0  0  0  0  0  0  0  0  0  0  0
   -4.1292   -1.7015    2.1431 H   0  0  0  0  0  0  0  0  0  0  0  0
   -5.7822    1.2700    2.3325 C   0  0  0  0  0  0  0  0  0  0  0  0
   -6.9302    2.1831    0.7445 H   0  0  0  0  0  0  0  0  0  0  0  0
   -4.5750    0.1344    3.7234 H   0  0  0  0  0  0  0  0  0  0  0  0
   -5.9722    2.0806    3.0433 H   0  0  0  0  0  0  0  0  0  0  0  0
   -5.9030   -3.7015    0.0005 C   0  0  0  0  0  0  0  0  0  0  0  0
   -6.5554   -3.6533    1.2358 C   0  0  0  0  0  0  0  0  0  0  0  0
   -5.9953   -4.8357   -0.8178 C   0  0  0  0  0  0  0  0  0  0  0  0
   -7.2775   -4.7709    1.6734 C   0  0  0  0  0  0  0  0  0  0  0  0
   -6.5379   -2.7644    1.8748 H   0  0  0  0  0  0  0  0  0  0  0  0
   -6.7154   -5.9459   -0.3705 C   0  0  0  0  0  0  0  0  0  0  0  0
   -5.5304   -4.8884   -1.8066 H   0  0  0  0  0  0  0  0  0  0  0  0
   -7.3549   -5.9157    0.8758 C   0  0  0  0  0  0  0  0  0  0  0  0
   -7.7930   -4.7409    2.6375 H   0  0  0  0  0  0  0  0  0  0  0  0
   -6.7907   -6.8357   -1.0001 H   0  0  0  0  0  0  0  0  0  0  0  0
   -7.9238   -6.7849    1.2192 H   0  0  0  0  0  0  0  0  0  0  0  0
   -5.6891   -1.8292   -2.3480 C   0  0  0  0  0  0  0  0  0  0  0  0
   -5.4192   -0.7753   -2.6208 H   0  0  0  0  0  0  0  0  0  0  0  0
   -5.2230   -2.5199   -3.0991 H   0  0  0  0  0  0  0  0  0  0  0  0
   -3.1963   -5.4258    1.3641 C   0  0  0  0  0  0  0  0  0  0  0  0
   -3.0027   -4.6275    2.1276 H   0  0  0  0  0  0  0  0  0  0  0  0
   -4.2975   -5.4581    1.1447 H   0  0  0  0  0  0  0  0  0  0  0  0
   -7.2024   -2.0069   -2.3886 C   0  0  0  0  0  0  0  0  0  0  0  0
   -7.5740   -1.6700   -3.3921 H   0  0  0  0  0  0  0  0  0  0  0  0
   -7.5022   -3.0767   -2.2646 H   0  0  0  0  0  0  0  0  0  0  0  0
```

```
   -7.7367   -1.4038   -1.6177 H   0  0  0  0  0  0  0  0  0  0  0  0
   -2.7643   -6.7715    1.9345 C   0  0  0  0  0  0  0  0  0  0  0  0
   -3.0673   -6.8126    3.0144 H   0  0  0  0  0  0  0  0  0  0  0  0
   -3.2724   -7.6225    1.4243 H   0  0  0  0  0  0  0  0  0  0  0  0
   -1.6607   -6.9365    1.8960 H   0  0  0  0  0  0  0  0  0  0  0  0
   -2.3942   -2.4105   -0.7584 Ir  0  0  0  0  0  0  0  0  0  0  0  0
  1  2  1  0  0  0  0
  1  9  1  0  0  0  0
  1  3  1  0  0  0  0
  1 10  1  0  0  0  0
  2 19  1  0  0  0  0
  2 11  1  0  0  0  0
  2 16  1  0  0  0  0
  3  7  1  0  0  0  0
  3  5  2  0  0  0  0
  3 81  1  0  0  0  0
  4  6  1  0  0  0  0
  4  5  1  0  0  0  0
  4 13  1  0  0  0  0
  4 14  1  0  0  0  0
  5  8  1  0  0  0  0
  5 81  1  0  0  0  0
  6 20  1  0  0  0  0
  6 15  1  0  0  0  0
  6 12  1  0  0  0  0
 15 16  2  0  0  0  0
 15 17  1  0  0  0  0
 15 81  1  0  0  0  0
 16 18  1  0  0  0  0
 16 81  1  0  0  0  0
 21 56  1  0  0  0  0
 21 45  1  0  0  0  0
 21 67  1  6  0  0  0
 21 81  1  0  0  0  0
 22 23  1  0  0  0  0
 22 70  1  1  0  0  0
 22 34  1  0  0  0  0
 22 81  1  0  0  0  0
 23 25  2  0  0  0  0
 23 24  1  0  0  0  0
 24 26  2  0  0  0  0
 24 27  1  0  0  0  0
 25 28  1  0  0  0  0
 25 29  1  0  0  0  0
 26 30  1  0  0  0  0
 26 31  1  0  0  0  0
 28 32  1  0  0  0  0
 28 30  2  0  0  0  0
 30 33  1  0  0  0  0
 34 35  1  0  0  0  0
 34 36  2  0  0  0  0
 35 38  1  0  0  0  0
 35 37  2  0  0  0  0
 36 39  1  0  0  0  0
 36 40  1  0  0  0  0
 37 42  1  0  0  0  0
 37 41  1  0  0  0  0
 39 41  2  0  0  0  0
 39 43  1  0  0  0  0
 41 44  1  0  0  0  0
 45 47  1  0  0  0  0
 45 46  2  0  0  0  0
 46 48  1  0  0  0  0
 46 49  1  0  0  0  0
 47 51  1  0  0  0  0
```

```
 47 50  2  0  0  0  0
 48 52  2  0  0  0  0
 48 53  1  0  0  0  0
 50 54  1  0  0  0  0
 50 52  1  0  0  0  0
 52 55  1  0  0  0  0
 56 57  2  0  0  0  0
 56 58  1  0  0  0  0
 57 59  1  0  0  0  0
 57 60  1  0  0  0  0
 58 61  2  0  0  0  0
 58 62  1  0  0  0  0
 59 64  1  0  0  0  0
 59 63  2  0  0  0  0
 61 63  1  0  0  0  0
 61 65  1  0  0  0  0
 63 66  1  0  0  0  0
 67 68  1  0  0  0  0
 67 69  1  0  0  0  0
 67 73  1  0  0  0  0
 70 77  1  0  0  0  0
 70 71  1  0  0  0  0
 70 72  1  0  0  0  0
 73 75  1  0  0  0  0
 73 76  1  0  0  0  0
 73 74  1  0  0  0  0
 77 78  1  0  0  0  0
 77 79  1  0  0  0  0
 77 80  1  0  0  0  0
M  END
$$$$
mol189
Optimized Catalyst 189
Optimized Catalyst 189
 77 84  0  0  0  0  0  0  0  0999 V2000
   -2.3541   -4.3621   -0.6787 P   0  0  2  0  0  4  0  0  0  0  0  0
   -1.0532    0.3554   -1.3613 C   0  0  0  0  0  0  0  0  0  0  0  0
    0.2180   -0.4451   -1.0132 C   0  0  0  0  0  0  0  0  0  0  0  0
   -2.2528    0.0731   -0.4928 C   0  0  0  0  0  0  0  0  0  0  0  0
   -1.0729    0.0040    1.7599 C   0  0  0  0  0  0  0  0  0  0  0  0
   -2.2686   -0.1094    0.8515 C   0  0  0  0  0  0  0  0  0  0  0  0
   -0.3689   -1.3525    1.9668 C   0  0  0  0  0  0  0  0  0  0  0  0
   -3.2065    0.0711   -1.0507 H   0  0  0  0  0  0  0  0  0  0  0  0
   -3.2217   -0.3155    1.3669 H   0  0  0  0  0  0  0  0  0  0  0  0
   -1.3299    0.1184   -2.4230 H   0  0  0  0  0  0  0  0  0  0  0  0
   -0.8221    1.4518   -1.2942 H   0  0  0  0  0  0  0  0  0  0  0  0
    0.8657   -0.4817   -1.9294 H   0  0  0  0  0  0  0  0  0  0  0  0
   -0.9450   -1.9221    2.7455 H   0  0  0  0  0  0  0  0  0  0  0  0
   -1.4115    0.3803    2.7610 H   0  0  0  0  0  0  0  0  0  0  0  0
   -0.3567    0.7595    1.3515 H   0  0  0  0  0  0  0  0  0  0  0  0
   -0.2461   -2.2428    0.7524 C   0  0  0  0  0  0  0  0  0  0  0  0
   -0.0382   -1.8596   -0.5522 C   0  0  0  0  0  0  0  0  0  0  0  0
   -0.1349   -3.3090    1.0279 H   0  0  0  0  0  0  0  0  0  0  0  0
    0.1613   -2.6244   -1.3255 H   0  0  0  0  0  0  0  0  0  0  0  0
    0.8020    0.0883   -0.2190 H   0  0  0  0  0  0  0  0  0  0  0  0
    0.6688   -1.1663    2.3553 H   0  0  0  0  0  0  0  0  0  0  0  0
   -3.9513   -2.4645   -0.4712 N   0  0  0  0  0  2  0  0  0  0  0  0
   -5.0951   -2.4417   -0.6855 C   0  0  0  0  0  2  0  0  0  0  0  0
   -6.4904   -2.5133   -0.9830 C   0  0  0  0  0  0  0  0  0  0  0  0
   -6.9812   -3.6369   -1.6761 C   0  0  0  0  0  0  0  0  0  0  0  0
   -7.3554   -1.4727   -0.5993 C   0  0  0  0  0  0  0  0  0  0  0  0
   -8.3410   -3.7127   -1.9788 C   0  0  0  0  0  0  0  0  0  0  0  0
   -6.3006   -4.4364   -1.9760 H   0  0  0  0  0  0  0  0  0  0  0  0
   -8.7136   -1.5636   -0.9114 C   0  0  0  0  0  0  0  0  0  0  0  0
   -6.9684   -0.6036   -0.0660 H   0  0  0  0  0  0  0  0  0  0  0  0
```

```
   -9.2060   -2.6795   -1.5981 C   0  0  0  0  0  0  0  0  0  0  0  0
   -0.7433   -5.2152   -1.0409 C   0  0  0  0  0  0  0  0  0  0  0  0
   -0.0376   -5.8411   -0.0070 C   0  0  0  0  0  0  0  0  0  0  0  0
   -0.2781   -5.2852   -2.3576 C   0  0  0  0  0  0  0  0  0  0  0  0
    1.1392   -6.5330   -0.2923 C   0  0  0  0  0  0  0  0  0  0  0  0
   -0.3898   -5.8131    1.0289 H   0  0  0  0  0  0  0  0  0  0  0  0
    0.8992   -5.9796   -2.6413 C   0  0  0  0  0  0  0  0  0  0  0  0
   -0.8183   -4.8146   -3.1847 H   0  0  0  0  0  0  0  0  0  0  0  0
    1.6197   -6.6131   -1.6134 C   0  0  0  0  0  0  0  0  0  0  0  0
    1.6881   -7.0247    0.5120 H   0  0  0  0  0  0  0  0  0  0  0  0
    1.2556   -6.0391   -3.6705 H   0  0  0  0  0  0  0  0  0  0  0  0
   -3.0763   -5.2950    0.7443 C   0  0  0  0  0  0  0  0  0  0  0  0
   -3.1446   -6.6911    0.7403 C   0  0  0  0  0  0  0  0  0  0  0  0
   -3.5159   -4.5627    1.8535 C   0  0  0  0  0  0  0  0  0  0  0  0
   -3.7116   -7.3556    1.8291 C   0  0  0  0  0  0  0  0  0  0  0  0
   -2.7448   -7.2870   -0.0856 H   0  0  0  0  0  0  0  0  0  0  0  0
   -4.0812   -5.2285    2.9403 C   0  0  0  0  0  0  0  0  0  0  0  0
   -3.4092   -3.4622    1.8890 H   0  0  0  0  0  0  0  0  0  0  0  0
   -4.1971   -6.6327    2.9336 C   0  0  0  0  0  0  0  0  0  0  0  0
   -3.7727   -8.4448    1.8232 H   0  0  0  0  0  0  0  0  0  0  0  0
   -4.4338   -4.6628    3.8037 H   0  0  0  0  0  0  0  0  0  0  0  0
   -3.3528   -4.6531   -2.2164 C   0  0  0  0  0  0  0  0  0  0  0  0
   -4.0779   -5.8227   -2.4399 C   0  0  0  0  0  0  0  0  0  0  0  0
   -3.2944   -3.6491   -3.1938 C   0  0  0  0  0  0  0  0  0  0  0  0
   -4.7448   -5.9958   -3.6562 C   0  0  0  0  0  0  0  0  0  0  0  0
   -4.1528   -6.6125   -1.6892 H   0  0  0  0  0  0  0  0  0  0  0  0
   -3.9573   -3.8255   -4.4061 C   0  0  0  0  0  0  0  0  0  0  0  0
   -2.7409   -2.7122   -3.0071 H   0  0  0  0  0  0  0  0  0  0  0  0
   -4.6831   -5.0081   -4.6540 C   0  0  0  0  0  0  0  0  0  0  0  0
   -5.3109   -6.9112   -3.8344 H   0  0  0  0  0  0  0  0  0  0  0  0
   -3.9153   -3.0466   -5.1685 H   0  0  0  0  0  0  0  0  0  0  0  0
  -10.2706   -2.7439   -1.8390 H   0  0  0  0  0  0  0  0  0  0  0  0
   -8.7321   -4.5809   -2.5134 H   0  0  0  0  0  0  0  0  0  0  0  0
   -9.3936   -0.7598   -0.6180 H   0  0  0  0  0  0  0  0  0  0  0  0
   -2.2474   -2.2770   -0.2171 Rh  0  0  0  0  0  0  0  0  0  0  0  0
   -5.3956   -5.2025   -5.9495 C   0  0  0  0  0  0  0  0  0  0  0  0
   -5.3744   -6.2749   -6.2628 H   0  0  0  0  0  0  0  0  0  0  0  0
   -6.4636   -4.8881   -5.8383 H   0  0  0  0  0  0  0  0  0  0  0  0
   -4.9352   -4.5971   -6.7669 H   0  0  0  0  0  0  0  0  0  0  0  0
    2.8623   -7.3808   -1.9149 C   0  0  0  0  0  0  0  0  0  0  0  0
    2.6144   -8.4708   -1.9827 H   0  0  0  0  0  0  0  0  0  0  0  0
    3.3188   -7.0651   -2.8831 H   0  0  0  0  0  0  0  0  0  0  0  0
    3.6256   -7.2433   -1.1111 H   0  0  0  0  0  0  0  0  0  0  0  0
   -4.8194   -7.3329    4.0939 C   0  0  0  0  0  0  0  0  0  0  0  0
   -4.2277   -7.1350    5.0225 H   0  0  0  0  0  0  0  0  0  0  0  0
   -5.8619   -6.9599    4.2533 H   0  0  0  0  0  0  0  0  0  0  0  0
   -4.8664   -8.4374    3.9418 H   0  0  0  0  0  0  0  0  0  0  0  0
  1 65  1  0  0  0  0
  1 42  1  1  0  0  0
  1 32  1  0  0  0  0
  1 52  1  0  0  0  0
  2 11  1  0  0  0  0
  2  3  1  0  0  0  0
  2  4  1  0  0  0  0
  2 10  1  0  0  0  0
  3 17  1  0  0  0  0
  3 20  1  0  0  0  0
  3 12  1  0  0  0  0
  4  6  2  0  0  0  0
  4  8  1  0  0  0  0
  4 65  1  0  0  0  0
  5  7  1  0  0  0  0
  5 14  1  0  0  0  0
  5  6  1  0  0  0  0
  5 15  1  0  0  0  0
```

```
  6  9  1  0  0  0  0
  6 65  1  0  0  0  0
  7 21  1  0  0  0  0
  7 13  1  0  0  0  0
  7 16  1  0  0  0  0
 16 18  1  0  0  0  0
 16 17  2  0  0  0  0
 16 65  1  0  0  0  0
 17 19  1  0  0  0  0
 17 65  1  0  0  0  0
 22 65  1  0  0  0  0
 22 23  1  0  0  0  0
 23 24  1  0  0  0  0
 24 26  2  0  0  0  0
 24 25  1  0  0  0  0
 25 27  2  0  0  0  0
 25 28  1  0  0  0  0
 26 30  1  0  0  0  0
 26 29  1  0  0  0  0
 27 31  1  0  0  0  0
 27 63  1  0  0  0  0
 29 64  1  0  0  0  0
 29 31  2  0  0  0  0
 31 62  1  0  0  0  0
 32 33  2  0  0  0  0
 32 34  1  0  0  0  0
 33 36  1  0  0  0  0
 33 35  1  0  0  0  0
 34 37  2  0  0  0  0
 34 38  1  0  0  0  0
 35 40  1  0  0  0  0
 35 39  2  0  0  0  0
 37 39  1  0  0  0  0
 37 41  1  0  0  0  0
 39 70  1  0  0  0  0
 42 44  1  0  0  0  0
 42 43  2  0  0  0  0
 43 45  1  0  0  0  0
 43 46  1  0  0  0  0
 44 48  1  0  0  0  0
 44 47  2  0  0  0  0
 45 49  2  0  0  0  0
 45 50  1  0  0  0  0
 47 51  1  0  0  0  0
 47 49  1  0  0  0  0
 49 74  1  0  0  0  0
 52 53  1  0  0  0  0
 52 54  2  0  0  0  0
 53 56  1  0  0  0  0
 53 55  2  0  0  0  0
 54 58  1  0  0  0  0
 54 57  1  0  0  0  0
 55 59  1  0  0  0  0
 55 60  1  0  0  0  0
 57 59  2  0  0  0  0
 57 61  1  0  0  0  0
 59 66  1  0  0  0  0
 66 68  1  0  0  0  0
 66 67  1  0  0  0  0
 66 69  1  0  0  0  0
 70 73  1  0  0  0  0
 70 71  1  0  0  0  0
 70 72  1  0  0  0  0
 74 76  1  0  0  0  0
 74 75  1  0  0  0  0
```

```
 74 77  1  0  0  0  0
M  END
$$$$
mol190
Optimized Catalyst 190
Optimized Catalyst 190
 86 93  0  0  0  0  0  0  0  0999 V2000
   -2.3831   -4.3720   -0.7125 P   0  0  2  0  0  4  0  0  0  0  0  0
   -0.9448    0.3349   -1.2428 C   0  0  0  0  0  0  0  0  0  0  0  0
    0.2741   -0.4855   -0.7756 C   0  0  0  0  0  0  0  0  0  0  0  0
   -2.2265    0.0712   -0.4941 C   0  0  0  0  0  0  0  0  0  0  0  0
   -1.2724   -0.0485    1.8626 C   0  0  0  0  0  0  0  0  0  0  0  0
   -2.3756   -0.1215    0.8403 C   0  0  0  0  0  0  0  0  0  0  0  0
   -0.6307   -1.4269    2.1211 C   0  0  0  0  0  0  0  0  0  0  0  0
   -3.1225    0.0897   -1.1408 H   0  0  0  0  0  0  0  0  0  0  0  0
   -3.3784   -0.3078    1.2588 H   0  0  0  0  0  0  0  0  0  0  0  0
   -1.1226    0.0998   -2.3261 H   0  0  0  0  0  0  0  0  0  0  0  0
   -0.7042    1.4274   -1.1530 H   0  0  0  0  0  0  0  0  0  0  0  0
    1.0116   -0.5217   -1.6212 H   0  0  0  0  0  0  0  0  0  0  0  0
   -1.3118   -1.9968    2.8103 H   0  0  0  0  0  0  0  0  0  0  0  0
   -1.6973    0.3285    2.8299 H   0  0  0  0  0  0  0  0  0  0  0  0
   -0.4993    0.6897    1.5353 H   0  0  0  0  0  0  0  0  0  0  0  0
   -0.3842   -2.2987    0.9115 C   0  0  0  0  0  0  0  0  0  0  0  0
   -0.0466   -1.9013   -0.3615 C   0  0  0  0  0  0  0  0  0  0  0  0
   -0.3078   -3.3696    1.1818 H   0  0  0  0  0  0  0  0  0  0  0  0
    0.2186   -2.6595   -1.1227 H   0  0  0  0  0  0  0  0  0  0  0  0
    0.7818    0.0306    0.0798 H   0  0  0  0  0  0  0  0  0  0  0  0
    0.3557   -1.2763    2.6369 H   0  0  0  0  0  0  0  0  0  0  0  0
   -3.9591   -2.4438   -0.6096 N   0  0  0  0  0  2  0  0  0  0  0  0
   -5.0947   -2.3969   -0.8587 C   0  0  0  0  0  2  0  0  0  0  0  0
   -6.4816   -2.4365   -1.1976 C   0  0  0  0  0  0  0  0  0  0  0  0
   -7.0186   -3.6159   -1.7476 C   0  0  0  0  0  0  0  0  0  0  0  0
   -7.2927   -1.3056   -0.9925 C   0  0  0  0  0  0  0  0  0  0  0  0
   -8.3701   -3.6557   -2.0922 C   0  0  0  0  0  0  0  0  0  0  0  0
   -6.3802   -4.4880   -1.9049 H   0  0  0  0  0  0  0  0  0  0  0  0
   -8.6432   -1.3622   -1.3425 C   0  0  0  0  0  0  0  0  0  0  0  0
   -6.8702   -0.3952   -0.5661 H   0  0  0  0  0  0  0  0  0  0  0  0
   -9.1811   -2.5326   -1.8907 C   0  0  0  0  0  0  0  0  0  0  0  0
   -0.7547   -5.1965   -1.1174 C   0  0  0  0  0  0  0  0  0  0  0  0
   -0.0412   -5.8266   -0.0979 C   0  0  0  0  0  0  0  0  0  0  0  0
   -0.3076   -5.2047   -2.4360 C   0  0  0  0  0  0  0  0  0  0  0  0
    1.1565   -6.4959   -0.4096 C   0  0  0  0  0  0  0  0  0  0  0  0
   -0.3903   -5.8372    0.9362 H   0  0  0  0  0  0  0  0  0  0  0  0
    0.8940   -5.8700   -2.7522 C   0  0  0  0  0  0  0  0  0  0  0  0
   -0.8659   -4.7276   -3.2432 H   0  0  0  0  0  0  0  0  0  0  0  0
    1.6129   -6.5145   -1.7366 C   0  0  0  0  0  0  0  0  0  0  0  0
   -3.0381   -5.3064    0.7585 C   0  0  0  0  0  0  0  0  0  0  0  0
   -3.0195   -6.6982    0.7818 C   0  0  0  0  0  0  0  0  0  0  0  0
   -3.4885   -4.5541    1.8434 C   0  0  0  0  0  0  0  0  0  0  0  0
   -3.5012   -7.3697    1.9232 C   0  0  0  0  0  0  0  0  0  0  0  0
   -2.6201   -7.2902   -0.0443 H   0  0  0  0  0  0  0  0  0  0  0  0
   -3.9644   -5.2205    2.9880 C   0  0  0  0  0  0  0  0  0  0  0  0
   -3.4676   -3.4517    1.8252 H   0  0  0  0  0  0  0  0  0  0  0  0
   -3.9723   -6.6246    3.0133 C   0  0  0  0  0  0  0  0  0  0  0  0
   -3.4368   -4.7529   -2.2061 C   0  0  0  0  0  0  0  0  0  0  0  0
   -4.2182   -5.8995   -2.2741 C   0  0  0  0  0  0  0  0  0  0  0  0
   -3.3571   -3.8463   -3.2662 C   0  0  0  0  0  0  0  0  0  0  0  0
   -4.9419   -6.1641   -3.4553 C   0  0  0  0  0  0  0  0  0  0  0  0
   -4.2975   -6.6031   -1.4451 H   0  0  0  0  0  0  0  0  0  0  0  0
   -4.0641   -4.1147   -4.4516 C   0  0  0  0  0  0  0  0  0  0  0  0
   -2.7674   -2.9238   -3.1836 H   0  0  0  0  0  0  0  0  0  0  0  0
   -4.8530   -5.2740   -4.5336 C   0  0  0  0  0  0  0  0  0  0  0  0
  -10.2394   -2.5693   -2.1625 H   0  0  0  0  0  0  0  0  0  0  0  0
   -8.7952   -4.5660   -2.5195 H   0  0  0  0  0  0  0  0  0  0  0  0
   -9.2818   -0.4892   -1.1877 H   0  0  0  0  0  0  0  0  0  0  0  0
```

```
   -2.2791   -2.2827   -0.2496 Rh  0  0  0  0  0  0  0  0  0  0  0  0
    2.5347   -7.0447   -1.9803 H   0  0  0  0  0  0  0  0  0  0  0  0
   -4.3443   -7.1416    3.8998 H   0  0  0  0  0  0  0  0  0  0  0  0
   -5.4052   -5.4844   -5.4511 H   0  0  0  0  0  0  0  0  0  0  0  0
   -3.9972   -3.1678   -5.6046 C   0  0  0  0  0  0  0  0  0  0  0  0
   -4.1696   -3.6981   -6.5727 H   0  0  0  0  0  0  0  0  0  0  0  0
   -4.7889   -2.3862   -5.4880 H   0  0  0  0  0  0  0  0  0  0  0  0
   -3.0033   -2.6654   -5.6610 H   0  0  0  0  0  0  0  0  0  0  0  0
   -5.7927   -7.3869   -3.5620 C   0  0  0  0  0  0  0  0  0  0  0  0
   -5.2607   -8.1524   -4.1825 H   0  0  0  0  0  0  0  0  0  0  0  0
   -6.0085   -7.8335   -2.5639 H   0  0  0  0  0  0  0  0  0  0  0  0
   -6.7654   -7.1497   -4.0566 H   0  0  0  0  0  0  0  0  0  0  0  0
    1.3781   -5.8991   -4.1654 C   0  0  0  0  0  0  0  0  0  0  0  0
    1.9232   -7.2032    0.6601 C   0  0  0  0  0  0  0  0  0  0  0  0
    1.5443   -8.2519    0.7508 H   0  0  0  0  0  0  0  0  0  0  0  0
    3.0122   -7.2455    0.4159 H   0  0  0  0  0  0  0  0  0  0  0  0
    1.8107   -6.6949    1.6457 H   0  0  0  0  0  0  0  0  0  0  0  0
   -3.4802   -8.8626    1.9714 C   0  0  0  0  0  0  0  0  0  0  0  0
   -2.4681   -9.2073    2.3017 H   0  0  0  0  0  0  0  0  0  0  0  0
   -4.2360   -9.2578    2.6919 H   0  0  0  0  0  0  0  0  0  0  0  0
   -3.6957   -9.3021    0.9691 H   0  0  0  0  0  0  0  0  0  0  0  0
   -4.4615   -4.4488    4.1669 C   0  0  0  0  0  0  0  0  0  0  0  0
   -5.5804   -4.4553    4.1712 H   0  0  0  0  0  0  0  0  0  0  0  0
   -4.1049   -4.9124    5.1194 H   0  0  0  0  0  0  0  0  0  0  0  0
   -4.1131   -3.3908    4.1445 H   0  0  0  0  0  0  0  0  0  0  0  0
    2.4659   -6.1435   -4.2200 H   0  0  0  0  0  0  0  0  0  0  0  0
    0.8150   -6.6810   -4.7338 H   0  0  0  0  0  0  0  0  0  0  0  0
    1.2199   -4.9137   -4.6632 H   0  0  0  0  0  0  0  0  0  0  0  0
  1 59  1  0  0  0  0
  1 40  1  1  0  0  0
  1 32  1  0  0  0  0
  1 48  1  0  0  0  0
  2 11  1  0  0  0  0
  2  3  1  0  0  0  0
  2  4  1  0  0  0  0
  2 10  1  0  0  0  0
  3 17  1  0  0  0  0
  3 20  1  0  0  0  0
  3 12  1  0  0  0  0
  4  6  2  0  0  0  0
  4  8  1  0  0  0  0
  4 59  1  0  0  0  0
  5  7  1  0  0  0  0
  5 14  1  0  0  0  0
  5  6  1  0  0  0  0
  5 15  1  0  0  0  0
  6  9  1  0  0  0  0
  6 59  1  0  0  0  0
  7 21  1  0  0  0  0
  7 13  1  0  0  0  0
  7 16  1  0  0  0  0
 16 18  1  0  0  0  0
 16 17  2  0  0  0  0
 16 59  1  0  0  0  0
 17 19  1  0  0  0  0
 17 59  1  0  0  0  0
 22 59  1  0  0  0  0
 22 23  1  0  0  0  0
 23 24  1  0  0  0  0
 24 26  1  0  0  0  0
 24 25  2  0  0  0  0
 25 27  1  0  0  0  0
 25 28  1  0  0  0  0
 26 30  1  0  0  0  0
 26 29  2  0  0  0  0
```

```
 27 31  2  0  0  0  0
 27 57  1  0  0  0  0
 29 58  1  0  0  0  0
 29 31  1  0  0  0  0
 31 56  1  0  0  0  0
 32 33  2  0  0  0  0
 32 34  1  0  0  0  0
 33 36  1  0  0  0  0
 33 35  1  0  0  0  0
 34 37  2  0  0  0  0
 34 38  1  0  0  0  0
 35 72  1  0  0  0  0
 35 39  2  0  0  0  0
 37 39  1  0  0  0  0
 37 71  1  0  0  0  0
 39 60  1  0  0  0  0
 40 41  2  0  0  0  0
 40 42  1  0  0  0  0
 41 43  1  0  0  0  0
 41 44  1  0  0  0  0
 42 45  2  0  0  0  0
 42 46  1  0  0  0  0
 43 76  1  0  0  0  0
 43 47  2  0  0  0  0
 45 47  1  0  0  0  0
 45 80  1  0  0  0  0
 47 61  1  0  0  0  0
 48 49  1  0  0  0  0
 48 50  2  0  0  0  0
 49 52  1  0  0  0  0
 49 51  2  0  0  0  0
 50 54  1  0  0  0  0
 50 53  1  0  0  0  0
 51 55  1  0  0  0  0
 51 67  1  0  0  0  0
 53 55  2  0  0  0  0
 53 63  1  0  0  0  0
 55 62  1  0  0  0  0
 63 65  1  0  0  0  0
 63 64  1  0  0  0  0
 63 66  1  0  0  0  0
 67 69  1  0  0  0  0
 67 68  1  0  0  0  0
 67 70  1  0  0  0  0
 71 84  1  0  0  0  0
 71 85  1  0  0  0  0
 71 86  1  0  0  0  0
 72 73  1  0  0  0  0
 72 75  1  0  0  0  0
 72 74  1  0  0  0  0
 76 77  1  0  0  0  0
 76 78  1  0  0  0  0
 76 79  1  0  0  0  0
 80 81  1  0  0  0  0
 80 82  1  0  0  0  0
 80 83  1  0  0  0  0
M  END
$$$$
mol191
Optimized Catalyst 191
Optimized Catalyst 191
 86 93  0  0  0  0  0  0  0  0999 V2000
   -2.4734   -4.7611   -0.5738 P   0  0  2  0  0  4  0  0  0  0  0  0
   -1.4150    1.0309   -1.4794 C   0  0  0  0  0  0  0  0  0  0  0  0
    0.0578    0.5699   -1.3849 C   0  0  0  0  0  0  0  0  0  0  0  0
```

-2.3974	0.2477	-0.6516	C	0	0	0	0	0	0	0	0	0	0	0	0
-1.0465	0.1578	1.4805	C	0	0	0	0	0	0	0	0	0	0	0	0
-2.2616	-0.1078	0.6377	C	0	0	0	0	0	0	0	0	0	0	0	0
-0.0375	-1.0060	1.3430	C	0	0	0	0	0	0	0	0	0	0	0	0
-3.3221	-0.0097	-1.1838	H	0	0	0	0	0	0	0	0	0	0	0	0
-3.0686	-0.6606	1.1552	H	0	0	0	0	0	0	0	0	0	0	0	0
-1.7272	0.9748	-2.5526	H	0	0	0	0	0	0	0	0	0	0	0	0
-1.4737	2.1045	-1.1575	H	0	0	0	0	0	0	0	0	0	0	0	0
0.5827	0.8759	-2.3258	H	0	0	0	0	0	0	0	0	0	0	0	0
-0.3822	-1.8272	2.0292	H	0	0	0	0	0	0	0	0	0	0	0	0
-1.3448	0.2506	2.5551	H	0	0	0	0	0	0	0	0	0	0	0	0
-0.5741	1.1251	1.1839	H	0	0	0	0	0	0	0	0	0	0	0	0
0.1100	-1.5856	-0.0440	C	0	0	0	0	0	0	0	0	0	0	0	0
0.1735	-0.9155	-1.2090	C	0	0	0	0	0	0	0	0	0	0	0	0
0.2480	-2.6895	-0.0573	H	0	0	0	0	0	0	0	0	0	0	0	0
0.3331	-1.4658	-2.1420	H	0	0	0	0	0	0	0	0	0	0	0	0
0.5722	1.0894	-0.5385	H	0	0	0	0	0	0	0	0	0	0	0	0
0.9709	-0.6536	1.6811	H	0	0	0	0	0	0	0	0	0	0	0	0
-3.8918	-2.7616	-0.4431	N	0	0	0	0	0	2	0	0	0	0	0	0
-5.0075	-2.5462	-0.6980	C	0	0	0	0	0	2	0	0	0	0	0	0
-6.3871	-2.3791	-1.0220	C	0	0	0	0	0	0	0	0	0	0	0	0
-6.8752	-2.8682	-2.2480	C	0	0	0	0	0	0	0	0	0	0	0	0
-7.2425	-1.7283	-0.1134	C	0	0	0	0	0	0	0	0	0	0	0	0
-8.2264	-2.7056	-2.5566	C	0	0	0	0	0	0	0	0	0	0	0	0
-6.2021	-3.3675	-2.9482	H	0	0	0	0	0	0	0	0	0	0	0	0
-8.5910	-1.5735	-0.4382	C	0	0	0	0	0	0	0	0	0	0	0	0
-6.8536	-1.3453	0.8306	H	0	0	0	0	0	0	0	0	0	0	0	0
-9.0821	-2.0605	-1.6556	C	0	0	0	0	0	0	0	0	0	0	0	0
-0.7402	-5.4211	-0.8161	C	0	0	0	0	0	0	0	0	0	0	0	0
0.0780	-5.3548	0.3415	C	0	0	0	0	0	0	0	0	0	0	0	0
-0.2274	-6.0106	-1.9904	C	0	0	0	0	0	0	0	0	0	0	0	0
1.3982	-5.8227	0.2973	C	0	0	0	0	0	0	0	0	0	0	0	0
1.1169	-6.4403	-2.0108	C	0	0	0	0	0	0	0	0	0	0	0	0
1.9243	-6.3500	-0.8833	C	0	0	0	0	0	0	0	0	0	0	0	0
-3.1861	-5.8613	0.7555	C	0	0	0	0	0	0	0	0	0	0	0	0
-3.0778	-7.2627	0.5833	C	0	0	0	0	0	0	0	0	0	0	0	0
-3.7718	-5.3526	1.9354	C	0	0	0	0	0	0	0	0	0	0	0	0
-3.5714	-8.1243	1.5799	C	0	0	0	0	0	0	0	0	0	0	0	0
-4.2773	-6.2420	2.8998	C	0	0	0	0	0	0	0	0	0	0	0	0
-4.1756	-7.6208	2.7279	C	0	0	0	0	0	0	0	0	0	0	0	0
-3.4766	-4.8484	-2.1369	C	0	0	0	0	0	0	0	0	0	0	0	0
-4.6454	-5.6174	-2.2989	C	0	0	0	0	0	0	0	0	0	0	0	0
-2.9936	-4.0709	-3.2224	C	0	0	0	0	0	0	0	0	0	0	0	0
-5.1629	-5.7951	-3.5989	C	0	0	0	0	0	0	0	0	0	0	0	0
-3.5581	-4.2326	-4.4941	C	0	0	0	0	0	0	0	0	0	0	0	0
-4.6079	-5.1342	-4.6901	C	0	0	0	0	0	0	0	0	0	0	0	0
-10.1391	-1.9331	-1.9045	H	0	0	0	0	0	0	0	0	0	0	0	0
-8.6160	-3.0787	-3.5066	H	0	0	0	0	0	0	0	0	0	0	0	0
-9.2639	-1.0676	0.2593	H	0	0	0	0	0	0	0	0	0	0	0	0
-2.2188	-2.7050	-0.0649	Rh	0	0	0	0	0	0	0	0	0	0	0	0
2.9610	-6.6967	-0.9163	H	0	0	0	0	0	0	0	0	0	0	0	0
-4.5649	-8.3010	3.4908	H	0	0	0	0	0	0	0	0	0	0	0	0
-5.0083	-5.2967	-5.6949	H	0	0	0	0	0	0	0	0	0	0	0	0
-6.0183	-6.4555	-3.7525	H	0	0	0	0	0	0	0	0	0	0	0	0
-3.1825	-3.6588	-5.3420	H	0	0	0	0	0	0	0	0	0	0	0	0
1.5363	-6.8595	-2.9279	H	0	0	0	0	0	0	0	0	0	0	0	0
2.0287	-5.7802	1.1869	H	0	0	0	0	0	0	0	0	0	0	0	0
-4.7544	-5.8579	3.8031	H	0	0	0	0	0	0	0	0	0	0	0	0
-3.4805	-9.2057	1.4615	H	0	0	0	0	0	0	0	0	0	0	0	0
-0.9641	-6.2663	-3.2689	C	0	0	0	0	0	0	0	0	0	0	0	0
-2.0492	-6.4657	-3.1409	H	0	0	0	0	0	0	0	0	0	0	0	0
-0.8322	-5.3963	-3.9598	H	0	0	0	0	0	0	0	0	0	0	0	0
-0.5413	-7.1672	-3.7853	H	0	0	0	0	0	0	0	0	0	0	0	0
-0.3863	-4.8308	1.6712	C	0	0	0	0	0	0	0	0	0	0	0	0
-1.0027	-3.8919	1.5931	H	0	0	0	0	0	0	0	0	0	0	0	0

```
   -0.9804   -5.6243    2.1975 H   0  0  0  0  0  0  0  0  0  0  0  0
    0.4860   -4.5785    2.3261 H   0  0  0  0  0  0  0  0  0  0  0  0
   -1.9296   -3.0238   -3.0608 C   0  0  0  0  0  0  0  0  0  0  0  0
   -1.5624   -2.6598   -4.0510 H   0  0  0  0  0  0  0  0  0  0  0  0
   -2.3600   -2.1269   -2.5129 H   0  0  0  0  0  0  0  0  0  0  0  0
   -1.0341   -3.3913   -2.4910 H   0  0  0  0  0  0  0  0  0  0  0  0
   -5.4548   -6.2112   -1.1910 C   0  0  0  0  0  0  0  0  0  0  0  0
   -5.1286   -7.2588   -0.9739 H   0  0  0  0  0  0  0  0  0  0  0  0
   -5.4043   -5.6136   -0.2516 H   0  0  0  0  0  0  0  0  0  0  0  0
   -6.5357   -6.2550   -1.4836 H   0  0  0  0  0  0  0  0  0  0  0  0
   -3.9304   -3.8987    2.2788 C   0  0  0  0  0  0  0  0  0  0  0  0
   -3.0346   -3.2643    2.0076 H   0  0  0  0  0  0  0  0  0  0  0  0
   -4.8521   -3.4971    1.7919 H   0  0  0  0  0  0  0  0  0  0  0  0
   -4.0572   -3.7704    3.3856 H   0  0  0  0  0  0  0  0  0  0  0  0
   -2.4661   -7.9452   -0.6017 C   0  0  0  0  0  0  0  0  0  0  0  0
   -1.3485   -7.8722   -0.5461 H   0  0  0  0  0  0  0  0  0  0  0  0
   -2.7167   -9.0363   -0.6123 H   0  0  0  0  0  0  0  0  0  0  0  0
   -2.8250   -7.5313   -1.5692 H   0  0  0  0  0  0  0  0  0  0  0  0
  1 53  1  0  0  0  0
  1 38  1  1  0  0  0
  1 32  1  0  0  0  0
  1 44  1  0  0  0  0
  2  3  1  0  0  0  0
  2 11  1  0  0  0  0
  2  4  1  0  0  0  0
  2 10  1  0  0  0  0
  3 17  1  0  0  0  0
  3 20  1  0  0  0  0
  3 12  1  0  0  0  0
  4  6  2  0  0  0  0
  4  8  1  0  0  0  0
  4 53  1  0  0  0  0
  5 14  1  0  0  0  0
  5  6  1  0  0  0  0
  5  7  1  0  0  0  0
  5 15  1  0  0  0  0
  6  9  1  0  0  0  0
  6 53  1  0  0  0  0
  7 21  1  0  0  0  0
  7 13  1  0  0  0  0
  7 16  1  0  0  0  0
 16 18  1  0  0  0  0
 16 17  2  0  0  0  0
 16 53  1  0  0  0  0
 17 19  1  0  0  0  0
 17 53  1  0  0  0  0
 22 53  1  0  0  0  0
 22 23  1  0  0  0  0
 23 24  1  0  0  0  0
 24 26  2  0  0  0  0
 24 25  1  0  0  0  0
 25 27  2  0  0  0  0
 25 28  1  0  0  0  0
 26 30  1  0  0  0  0
 26 29  1  0  0  0  0
 27 31  1  0  0  0  0
 27 51  1  0  0  0  0
 29 52  1  0  0  0  0
 29 31  2  0  0  0  0
 31 50  1  0  0  0  0
 32 33  2  0  0  0  0
 32 34  1  0  0  0  0
 33 67  1  0  0  0  0
 33 35  1  0  0  0  0
 34 36  2  0  0  0  0
```

```
 34 63  1  0  0  0  0
 35 60  1  0  0  0  0
 35 37  2  0  0  0  0
 36 37  1  0  0  0  0
 36 59  1  0  0  0  0
 37 54  1  0  0  0  0
 38 40  1  0  0  0  0
 38 39  2  0  0  0  0
 39 41  1  0  0  0  0
 39 83  1  0  0  0  0
 40 79  1  0  0  0  0
 40 42  2  0  0  0  0
 41 43  2  0  0  0  0
 41 62  1  0  0  0  0
 42 61  1  0  0  0  0
 42 43  1  0  0  0  0
 43 55  1  0  0  0  0
 44 45  1  0  0  0  0
 44 46  2  0  0  0  0
 45 75  1  0  0  0  0
 45 47  2  0  0  0  0
 46 71  1  0  0  0  0
 46 48  1  0  0  0  0
 47 49  1  0  0  0  0
 47 57  1  0  0  0  0
 48 49  2  0  0  0  0
 48 58  1  0  0  0  0
 49 56  1  0  0  0  0
 63 64  1  0  0  0  0
 63 65  1  0  0  0  0
 63 66  1  0  0  0  0
 67 69  1  0  0  0  0
 67 70  1  0  0  0  0
 67 68  1  0  0  0  0
 71 73  1  0  0  0  0
 71 74  1  0  0  0  0
 71 72  1  0  0  0  0
 75 76  1  0  0  0  0
 75 77  1  0  0  0  0
 75 78  1  0  0  0  0
 79 82  1  0  0  0  0
 79 80  1  0  0  0  0
 79 81  1  0  0  0  0
 83 84  1  0  0  0  0
 83 85  1  0  0  0  0
 83 86  1  0  0  0  0
M  END
$$$$
mol192
Optimized Catalyst 192
Optimized Catalyst 192
 79 86  0  0  0  0  0  0  0  0999 V2000
   -2.3701   -4.3389   -0.6969 P   0  0  2  0  0  4  0  0  0  0  0  0
   -1.0544    0.3854   -1.3632 C   0  0  0  0  0  0  0  0  0  0  0  0
    0.2198   -0.4189   -1.0377 C   0  0  0  0  0  0  0  0  0  0  0  0
   -2.2422    0.1031   -0.4791 C   0  0  0  0  0  0  0  0  0  0  0  0
   -1.0323   -0.0122    1.7578 C   0  0  0  0  0  0  0  0  0  0  0  0
   -2.2402   -0.0975    0.8628 C   0  0  0  0  0  0  0  0  0  0  0  0
   -0.3453   -1.3812    1.9362 C   0  0  0  0  0  0  0  0  0  0  0  0
   -3.2047    0.1233   -1.0212 H   0  0  0  0  0  0  0  0  0  0  0  0
   -3.1885   -0.2983    1.3887 H   0  0  0  0  0  0  0  0  0  0  0  0
   -1.3469    0.1550   -2.4221 H   0  0  0  0  0  0  0  0  0  0  0  0
   -0.8196    1.4813   -1.2940 H   0  0  0  0  0  0  0  0  0  0  0  0
    0.8606   -0.4412   -1.9595 H   0  0  0  0  0  0  0  0  0  0  0  0
   -0.9335   -1.9629    2.6974 H   0  0  0  0  0  0  0  0  0  0  0  0
```

```
   -1.3538    0.3540    2.7687 H   0  0  0  0  0  0  0  0  0  0  0  0
   -0.3114    0.7408    1.3532 H   0  0  0  0  0  0  0  0  0  0  0  0
   -0.2275   -2.2452    0.7027 C   0  0  0  0  0  0  0  0  0  0  0  0
   -0.0327   -1.8395   -0.5959 C   0  0  0  0  0  0  0  0  0  0  0  0
   -0.1102   -3.3167    0.9556 H   0  0  0  0  0  0  0  0  0  0  0  0
    0.1509   -2.5924   -1.3849 H   0  0  0  0  0  0  0  0  0  0  0  0
    0.8114    0.1032   -0.2415 H   0  0  0  0  0  0  0  0  0  0  0  0
    0.6920   -1.2170    2.3361 H   0  0  0  0  0  0  0  0  0  0  0  0
   -3.9407   -2.4166   -0.4881 N   0  0  0  0  0  2  0  0  0  0  0  0
   -5.0846   -2.4082   -0.7028 C   0  0  0  0  0  2  0  0  0  0  0  0
   -6.4779   -2.5145   -1.0180 C   0  0  0  0  0  0  0  0  0  0  0  0
   -6.9202   -3.6673   -1.6956 C   0  0  0  0  0  0  0  0  0  0  0  0
   -7.3731   -1.4887   -0.6710 C   0  0  0  0  0  0  0  0  0  0  0  0
   -8.2672   -3.7918   -2.0303 C   0  0  0  0  0  0  0  0  0  0  0  0
   -6.2118   -4.4568   -1.9622 H   0  0  0  0  0  0  0  0  0  0  0  0
   -8.7231   -1.6148   -1.0081 C   0  0  0  0  0  0  0  0  0  0  0  0
   -7.0220   -0.5985   -0.1442 H   0  0  0  0  0  0  0  0  0  0  0  0
   -9.1543   -2.7625   -1.6852 C   0  0  0  0  0  0  0  0  0  0  0  0
   -0.7681   -5.2160   -1.0305 C   0  0  0  0  0  0  0  0  0  0  0  0
   -0.0970   -5.8641    0.0131 C   0  0  0  0  0  0  0  0  0  0  0  0
   -0.2775   -5.2853   -2.3381 C   0  0  0  0  0  0  0  0  0  0  0  0
    1.0699   -6.5787   -0.2540 C   0  0  0  0  0  0  0  0  0  0  0  0
    0.8901   -6.0026   -2.6034 C   0  0  0  0  0  0  0  0  0  0  0  0
    1.5758   -6.6592   -1.5659 C   0  0  0  0  0  0  0  0  0  0  0  0
   -3.1317   -5.2556    0.7174 C   0  0  0  0  0  0  0  0  0  0  0  0
   -3.2463   -6.6485    0.7122 C   0  0  0  0  0  0  0  0  0  0  0  0
   -3.5418   -4.5130    1.8321 C   0  0  0  0  0  0  0  0  0  0  0  0
   -3.8303   -7.2969    1.8023 C   0  0  0  0  0  0  0  0  0  0  0  0
   -4.1225   -5.1621    2.9200 C   0  0  0  0  0  0  0  0  0  0  0  0
   -4.2843   -6.5623    2.9122 C   0  0  0  0  0  0  0  0  0  0  0  0
   -3.3439   -4.6065   -2.2550 C   0  0  0  0  0  0  0  0  0  0  0  0
   -4.0531   -5.7782   -2.5185 C   0  0  0  0  0  0  0  0  0  0  0  0
   -3.2751   -3.5830   -3.2111 C   0  0  0  0  0  0  0  0  0  0  0  0
   -4.7030   -5.9267   -3.7463 C   0  0  0  0  0  0  0  0  0  0  0  0
   -3.9208   -3.7351   -4.4362 C   0  0  0  0  0  0  0  0  0  0  0  0
   -4.6377   -4.9146   -4.7205 C   0  0  0  0  0  0  0  0  0  0  0  0
   -8.6398   -4.6805   -2.5578 H   0  0  0  0  0  0  0  0  0  0  0  0
   -9.4471   -0.8282   -0.7504 H   0  0  0  0  0  0  0  0  0  0  0  0
   -5.2569   -6.8436   -3.9566 H   0  0  0  0  0  0  0  0  0  0  0  0
   -3.8681   -2.9398   -5.1812 H   0  0  0  0  0  0  0  0  0  0  0  0
    1.2662   -6.0620   -3.6258 H   0  0  0  0  0  0  0  0  0  0  0  0
    1.5919   -7.0876    0.5578 H   0  0  0  0  0  0  0  0  0  0  0  0
   -4.4504   -4.5875    3.7873 H   0  0  0  0  0  0  0  0  0  0  0  0
   -3.9277   -8.3838    1.7937 H   0  0  0  0  0  0  0  0  0  0  0  0
  -10.5965   -2.8966   -2.0546 N   0  0  0  0  0  0  0  0  0  0  0  0
  -11.3378   -1.9939   -1.7205 O   0  0  0  0  0  0  0  0  0  0  0  0
  -10.9132   -3.9008   -2.6637 O   0  0  0  0  0  0  0  0  0  0  0  0
   -3.3984   -3.4169    1.8716 H   0  0  0  0  0  0  0  0  0  0  0  0
   -2.8686   -7.2570   -0.1147 H   0  0  0  0  0  0  0  0  0  0  0  0
   -0.4690   -5.8353    1.0422 H   0  0  0  0  0  0  0  0  0  0  0  0
   -0.7904   -4.7967   -3.1722 H   0  0  0  0  0  0  0  0  0  0  0  0
   -2.7240   -2.6494   -2.9997 H   0  0  0  0  0  0  0  0  0  0  0  0
   -4.1265   -6.5891   -1.7908 H   0  0  0  0  0  0  0  0  0  0  0  0
   -4.9201   -7.2420    4.0768 C   0  0  0  0  0  0  0  0  0  0  0  0
   -4.3302   -7.0428    5.0067 H   0  0  0  0  0  0  0  0  0  0  0  0
   -5.9580   -6.8526    4.2286 H   0  0  0  0  0  0  0  0  0  0  0  0
   -4.9825   -8.3474    3.9367 H   0  0  0  0  0  0  0  0  0  0  0  0
   -5.3300   -5.0946   -6.0280 C   0  0  0  0  0  0  0  0  0  0  0  0
   -5.0237   -6.0628   -6.4986 H   0  0  0  0  0  0  0  0  0  0  0  0
   -6.4379   -5.1152   -5.8741 H   0  0  0  0  0  0  0  0  0  0  0  0
   -5.0932   -4.2738   -6.7461 H   0  0  0  0  0  0  0  0  0  0  0  0
    2.8096   -7.4479   -1.8458 C   0  0  0  0  0  0  0  0  0  0  0  0
    2.5513   -8.5369   -1.8872 H   0  0  0  0  0  0  0  0  0  0  0  0
    3.2728   -7.1616   -2.8200 H   0  0  0  0  0  0  0  0  0  0  0  0
    3.5716   -7.2979   -1.0427 H   0  0  0  0  0  0  0  0  0  0  0  0
```

```
   -2.2497   -2.2515   -0.2354 Rh  0  0  0  0  0  0  0  0  0  0  0  0
  1 79  1  0  0  0  0
  1 38  1  1  0  0  0
  1 32  1  0  0  0  0
  1 44  1  0  0  0  0
  2 11  1  0  0  0  0
  2  3  1  0  0  0  0
  2  4  1  0  0  0  0
  2 10  1  0  0  0  0
  3 17  1  0  0  0  0
  3 20  1  0  0  0  0
  3 12  1  0  0  0  0
  4  6  2  0  0  0  0
  4  8  1  0  0  0  0
  4 79  1  0  0  0  0
  5  7  1  0  0  0  0
  5 14  1  0  0  0  0
  5  6  1  0  0  0  0
  5 15  1  0  0  0  0
  6  9  1  0  0  0  0
  6 79  1  0  0  0  0
  7 21  1  0  0  0  0
  7 13  1  0  0  0  0
  7 16  1  0  0  0  0
 16 18  1  0  0  0  0
 16 17  2  0  0  0  0
 16 79  1  0  0  0  0
 17 19  1  0  0  0  0
 17 79  1  0  0  0  0
 22 79  1  0  0  0  0
 22 23  1  0  0  0  0
 23 24  1  0  0  0  0
 24 26  2  0  0  0  0
 24 25  1  0  0  0  0
 25 27  2  0  0  0  0
 25 28  1  0  0  0  0
 26 30  1  0  0  0  0
 26 29  1  0  0  0  0
 27 31  1  0  0  0  0
 27 50  1  0  0  0  0
 29 51  1  0  0  0  0
 29 31  2  0  0  0  0
 31 58  1  0  0  0  0
 32 33  2  0  0  0  0
 32 34  1  0  0  0  0
 33 63  1  0  0  0  0
 33 35  1  0  0  0  0
 34 36  2  0  0  0  0
 34 64  1  0  0  0  0
 35 55  1  0  0  0  0
 35 37  2  0  0  0  0
 36 37  1  0  0  0  0
 36 54  1  0  0  0  0
 37 75  1  0  0  0  0
 38 40  1  0  0  0  0
 38 39  2  0  0  0  0
 39 41  1  0  0  0  0
 39 62  1  0  0  0  0
 40 61  1  0  0  0  0
 40 42  2  0  0  0  0
 41 43  2  0  0  0  0
 41 57  1  0  0  0  0
 42 56  1  0  0  0  0
 42 43  1  0  0  0  0
 43 67  1  0  0  0  0
```

```
 44 45  1  0  0  0  0
 44 46  2  0  0  0  0
 45 66  1  0  0  0  0
 45 47  2  0  0  0  0
 46 65  1  0  0  0  0
 46 48  1  0  0  0  0
 47 49  1  0  0  0  0
 47 52  1  0  0  0  0
 48 49  2  0  0  0  0
 48 53  1  0  0  0  0
 49 71  1  0  0  0  0
 58 59  2  0  0  0  0
 58 60  2  0  0  0  0
 67 69  1  0  0  0  0
 67 68  1  0  0  0  0
 67 70  1  0  0  0  0
 71 73  1  0  0  0  0
 71 72  1  0  0  0  0
 71 74  1  0  0  0  0
 75 78  1  0  0  0  0
 75 76  1  0  0  0  0
 75 77  1  0  0  0  0
M  END
$$$$
mol193
Optimized Catalyst 193
Optimized Catalyst 193
 79 86  0  0  0  0  0  0  0  0999 V2000
   -2.4329   -4.6450   -0.7091 P   0  0  2  0  0  4  0  0  0  0  0  0
   -0.3806    0.6048   -1.0185 C   0  0  0  0  0  0  0  0  0  0  0  0
    0.7137   -0.3419   -0.4866 C   0  0  0  0  0  0  0  0  0  0  0  0
   -1.7203    0.5592   -0.3332 C   0  0  0  0  0  0  0  0  0  0  0  0
   -0.8871    0.4059    2.0534 C   0  0  0  0  0  0  0  0  0  0  0  0
   -1.9524    0.4728    0.9948 C   0  0  0  0  0  0  0  0  0  0  0  0
   -0.4046   -1.0261    2.3577 C   0  0  0  0  0  0  0  0  0  0  0  0
   -2.5681    0.7317   -1.0234 H   0  0  0  0  0  0  0  0  0  0  0  0
   -2.9825    0.5471    1.3851 H   0  0  0  0  0  0  0  0  0  0  0  0
   -0.5232    0.3991   -2.1129 H   0  0  0  0  0  0  0  0  0  0  0  0
   -0.0012    1.6637   -0.9266 H   0  0  0  0  0  0  0  0  0  0  0  0
    1.4727   -0.4813   -1.3055 H   0  0  0  0  0  0  0  0  0  0  0  0
   -1.0948   -1.4759    3.1228 H   0  0  0  0  0  0  0  0  0  0  0  0
   -1.2909    0.8462    3.0062 H   0  0  0  0  0  0  0  0  0  0  0  0
   -0.0231    1.0524    1.7487 H   0  0  0  0  0  0  0  0  0  0  0  0
   -0.2777   -1.9807    1.1912 C   0  0  0  0  0  0  0  0  0  0  0  0
    0.2286   -1.6928   -0.0412 C   0  0  0  0  0  0  0  0  0  0  0  0
   -0.3754   -3.0435    1.5091 H   0  0  0  0  0  0  0  0  0  0  0  0
    0.4385   -2.5105   -0.7603 H   0  0  0  0  0  0  0  0  0  0  0  0
    1.2545    0.1433    0.3712 H   0  0  0  0  0  0  0  0  0  0  0  0
    0.6176   -0.9570    2.8313 H   0  0  0  0  0  0  0  0  0  0  0  0
   -4.2797   -2.1432   -0.6058 N   0  0  0  0  0  2  0  0  0  0  0  0
   -5.3889   -2.1522   -0.9698 C   0  0  0  0  0  2  0  0  0  0  0  0
   -6.7425   -2.1743   -1.4281 C   0  0  0  0  0  0  0  0  0  0  0  0
   -7.1606   -3.2203   -2.2737 C   0  0  0  0  0  0  0  0  0  0  0  0
   -7.6310   -1.1562   -1.0347 C   0  0  0  0  0  0  0  0  0  0  0  0
   -8.4775   -3.2438   -2.7317 C   0  0  0  0  0  0  0  0  0  0  0  0
   -6.4615   -4.0079   -2.5706 H   0  0  0  0  0  0  0  0  0  0  0  0
   -8.9499   -1.1853   -1.4930 C   0  0  0  0  0  0  0  0  0  0  0  0
   -7.2987   -0.3482   -0.3775 H   0  0  0  0  0  0  0  0  0  0  0  0
   -9.3568   -2.2274   -2.3349 C   0  0  0  0  0  0  0  0  0  0  0  0
   -0.7675   -5.2918   -1.1646 C   0  0  0  0  0  0  0  0  0  0  0  0
    0.1488   -5.5956   -0.1467 C   0  0  0  0  0  0  0  0  0  0  0  0
   -0.4479   -5.5429   -2.5031 C   0  0  0  0  0  0  0  0  0  0  0  0
    1.3946   -6.1227   -0.4750 C   0  0  0  0  0  0  0  0  0  0  0  0
    0.8014   -6.0741   -2.8268 C   0  0  0  0  0  0  0  0  0  0  0  0
    1.7385   -6.3687   -1.8208 C   0  0  0  0  0  0  0  0  0  0  0  0
```

```
   -3.0380   -5.6529    0.7123 C   0  0  0  0  0  0  0  0  0  0  0  0
   -2.7631   -7.0237    0.7617 C   0  0  0  0  0  0  0  0  0  0  0  0
   -3.8026   -5.0392    1.7121 C   0  0  0  0  0  0  0  0  0  0  0  0
   -3.2791   -7.7897    1.8088 C   0  0  0  0  0  0  0  0  0  0  0  0
   -4.3169   -5.8072    2.7533 C   0  0  0  0  0  0  0  0  0  0  0  0
   -4.0661   -7.1950    2.8109 C   0  0  0  0  0  0  0  0  0  0  0  0
   -3.5672   -4.9630   -2.1362 C   0  0  0  0  0  0  0  0  0  0  0  0
   -4.4310   -6.0619   -2.0985 C   0  0  0  0  0  0  0  0  0  0  0  0
   -3.5067   -4.1450   -3.2723 C   0  0  0  0  0  0  0  0  0  0  0  0
   -5.2343   -6.3454   -3.2047 C   0  0  0  0  0  0  0  0  0  0  0  0
   -4.3076   -4.4346   -4.3755 C   0  0  0  0  0  0  0  0  0  0  0  0
   -5.1764   -5.5447   -4.3594 C   0  0  0  0  0  0  0  0  0  0  0  0
   -8.8324   -4.0444   -3.3963 H   0  0  0  0  0  0  0  0  0  0  0  0
   -9.6675   -0.4030   -1.2017 H   0  0  0  0  0  0  0  0  0  0  0  0
   -5.9022   -7.2099   -3.1788 H   0  0  0  0  0  0  0  0  0  0  0  0
   -4.2520   -3.8037   -5.2653 H   0  0  0  0  0  0  0  0  0  0  0  0
    1.0482   -6.2744   -3.8719 H   0  0  0  0  0  0  0  0  0  0  0  0
    2.1093   -6.3615    0.3155 H   0  0  0  0  0  0  0  0  0  0  0  0
   -4.9172   -5.3373    3.5349 H   0  0  0  0  0  0  0  0  0  0  0  0
   -3.0670   -8.8606    1.8476 H   0  0  0  0  0  0  0  0  0  0  0  0
  -10.7744   -2.2605   -2.8260 N   0  0  0  0  0  0  0  0  0  0  0  0
  -11.5090   -1.3780   -2.4344 O   0  0  0  0  0  0  0  0  0  0  0  0
  -11.0662   -3.1717   -3.5751 O   0  0  0  0  0  0  0  0  0  0  0  0
   -2.4413   -2.1372   -0.0234 Ir  0  0  0  0  0  0  0  0  0  0  0  0
   -4.0055   -3.9557    1.7047 H   0  0  0  0  0  0  0  0  0  0  0  0
   -2.1483   -7.5180    0.0014 H   0  0  0  0  0  0  0  0  0  0  0  0
   -0.0910   -5.4499    0.9106 H   0  0  0  0  0  0  0  0  0  0  0  0
   -1.1542   -5.3479   -3.3163 H   0  0  0  0  0  0  0  0  0  0  0  0
   -2.8433   -3.2741   -3.3254 H   0  0  0  0  0  0  0  0  0  0  0  0
   -4.5041   -6.7147   -1.2215 H   0  0  0  0  0  0  0  0  0  0  0  0
   -4.6263   -8.0023    3.9301 C   0  0  0  0  0  0  0  0  0  0  0  0
   -4.1532   -7.6911    4.8967 H   0  0  0  0  0  0  0  0  0  0  0  0
   -5.7306   -7.8423    4.0139 H   0  0  0  0  0  0  0  0  0  0  0  0
   -4.4451   -9.0954    3.7923 H   0  0  0  0  0  0  0  0  0  0  0  0
   -6.0301   -5.8707   -5.5352 C   0  0  0  0  0  0  0  0  0  0  0  0
   -5.7265   -6.8638   -5.9589 H   0  0  0  0  0  0  0  0  0  0  0  0
   -7.1054   -5.9381   -5.2364 H   0  0  0  0  0  0  0  0  0  0  0  0
   -5.9405   -5.1114   -6.3486 H   0  0  0  0  0  0  0  0  0  0  0  0
    3.0628   -6.9562   -2.1647 C   0  0  0  0  0  0  0  0  0  0  0  0
    3.0030   -8.0729   -2.0832 H   0  0  0  0  0  0  0  0  0  0  0  0
    3.3715   -6.7011   -3.2074 H   0  0  0  0  0  0  0  0  0  0  0  0
    3.8618   -6.5982   -1.4709 H   0  0  0  0  0  0  0  0  0  0  0  0
  1 61  1  0  0  0  0
  1 38  1  1  0  0  0
  1 32  1  0  0  0  0
  1 44  1  0  0  0  0
  2 11  1  0  0  0  0
  2  3  1  0  0  0  0
  2  4  1  0  0  0  0
  2 10  1  0  0  0  0
  3 17  1  0  0  0  0
  3 20  1  0  0  0  0
  3 12  1  0  0  0  0
  4  6  2  0  0  0  0
  4  8  1  0  0  0  0
  4 61  1  0  0  0  0
  5  7  1  0  0  0  0
  5 14  1  0  0  0  0
  5  6  1  0  0  0  0
  5 15  1  0  0  0  0
  6  9  1  0  0  0  0
  6 61  1  0  0  0  0
  7 21  1  0  0  0  0
  7 13  1  0  0  0  0
  7 16  1  0  0  0  0
```

```
 16 18  1  0  0  0  0
 16 17  2  0  0  0  0
 16 61  1  0  0  0  0
 17 19  1  0  0  0  0
 17 61  1  0  0  0  0
 22 61  1  0  0  0  0
 22 23  1  0  0  0  0
 23 24  1  0  0  0  0
 24 26  1  0  0  0  0
 24 25  2  0  0  0  0
 25 27  1  0  0  0  0
 25 28  1  0  0  0  0
 26 30  1  0  0  0  0
 26 29  2  0  0  0  0
 27 31  2  0  0  0  0
 27 50  1  0  0  0  0
 29 51  1  0  0  0  0
 29 31  1  0  0  0  0
 31 58  1  0  0  0  0
 32 33  2  0  0  0  0
 32 34  1  0  0  0  0
 33 64  1  0  0  0  0
 33 35  1  0  0  0  0
 34 36  2  0  0  0  0
 34 65  1  0  0  0  0
 35 55  1  0  0  0  0
 35 37  2  0  0  0  0
 36 37  1  0  0  0  0
 36 54  1  0  0  0  0
 37 76  1  0  0  0  0
 38 39  2  0  0  0  0
 38 40  1  0  0  0  0
 39 41  1  0  0  0  0
 39 63  1  0  0  0  0
 40 42  2  0  0  0  0
 40 62  1  0  0  0  0
 41 57  1  0  0  0  0
 41 43  2  0  0  0  0
 42 43  1  0  0  0  0
 42 56  1  0  0  0  0
 43 68  1  0  0  0  0
 44 45  1  0  0  0  0
 44 46  2  0  0  0  0
 45 67  1  0  0  0  0
 45 47  2  0  0  0  0
 46 48  1  0  0  0  0
 46 66  1  0  0  0  0
 47 52  1  0  0  0  0
 47 49  1  0  0  0  0
 48 49  2  0  0  0  0
 48 53  1  0  0  0  0
 49 72  1  0  0  0  0
 58 59  2  0  0  0  0
 58 60  2  0  0  0  0
 68 70  1  0  0  0  0
 68 69  1  0  0  0  0
 68 71  1  0  0  0  0
 72 74  1  0  0  0  0
 72 73  1  0  0  0  0
 72 75  1  0  0  0  0
 76 77  1  0  0  0  0
 76 79  1  0  0  0  0
 76 78  1  0  0  0  0
M  END
$$$$
```

```
mol194
Optimized Catalyst 194
Optimized Catalyst 194
 88 95  0  0  0  0  0  0  0  0999 V2000
   -2.3909   -4.3662   -0.7236 P   0  0  2  0  0  4  0  0  0  0  0  0
   -0.9456    0.3284   -1.2488 C   0  0  0  0  0  0  0  0  0  0  0  0
    0.2736   -0.4915   -0.7826 C   0  0  0  0  0  0  0  0  0  0  0  0
   -2.2222    0.0855   -0.4847 C   0  0  0  0  0  0  0  0  0  0  0  0
   -1.2526   -0.0461    1.8668 C   0  0  0  0  0  0  0  0  0  0  0  0
   -2.3622   -0.1049    0.8507 C   0  0  0  0  0  0  0  0  0  0  0  0
   -0.6244   -1.4315    2.1190 C   0  0  0  0  0  0  0  0  0  0  0  0
   -3.1243    0.1223   -1.1219 H   0  0  0  0  0  0  0  0  0  0  0  0
   -3.3642   -0.2752    1.2774 H   0  0  0  0  0  0  0  0  0  0  0  0
   -1.1357    0.0805   -2.3275 H   0  0  0  0  0  0  0  0  0  0  0  0
   -0.6969    1.4206   -1.1761 H   0  0  0  0  0  0  0  0  0  0  0  0
    1.0104   -0.5289   -1.6294 H   0  0  0  0  0  0  0  0  0  0  0  0
   -1.3114   -1.9986    2.8051 H   0  0  0  0  0  0  0  0  0  0  0  0
   -1.6685    0.3316    2.8382 H   0  0  0  0  0  0  0  0  0  0  0  0
   -0.4743    0.6873    1.5400 H   0  0  0  0  0  0  0  0  0  0  0  0
   -0.3831   -2.3015    0.9077 C   0  0  0  0  0  0  0  0  0  0  0  0
   -0.0472   -1.9059   -0.3655 C   0  0  0  0  0  0  0  0  0  0  0  0
   -0.3111   -3.3733    1.1767 H   0  0  0  0  0  0  0  0  0  0  0  0
    0.2124   -2.6649   -1.1279 H   0  0  0  0  0  0  0  0  0  0  0  0
    0.7829    0.0262    0.0711 H   0  0  0  0  0  0  0  0  0  0  0  0
    0.3624   -1.2924    2.6380 H   0  0  0  0  0  0  0  0  0  0  0  0
   -3.9477   -2.4152   -0.6143 N   0  0  0  0  0  2  0  0  0  0  0  0
   -5.0824   -2.3682   -0.8672 C   0  0  0  0  0  2  0  0  0  0  0  0
   -6.4681   -2.4168   -1.2250 C   0  0  0  0  0  0  0  0  0  0  0  0
   -6.9893   -3.6182   -1.7408 C   0  0  0  0  0  0  0  0  0  0  0  0
   -7.2784   -1.2777   -1.0772 C   0  0  0  0  0  0  0  0  0  0  0  0
   -8.3303   -3.6784   -2.1172 C   0  0  0  0  0  0  0  0  0  0  0  0
   -6.3459   -4.4960   -1.8520 H   0  0  0  0  0  0  0  0  0  0  0  0
   -8.6216   -1.3400   -1.4540 C   0  0  0  0  0  0  0  0  0  0  0  0
   -6.8648   -0.3506   -0.6745 H   0  0  0  0  0  0  0  0  0  0  0  0
   -9.1320   -2.5378   -1.9713 C   0  0  0  0  0  0  0  0  0  0  0  0
   -0.7668   -5.2024   -1.1213 C   0  0  0  0  0  0  0  0  0  0  0  0
   -0.0565   -5.8254   -0.0933 C   0  0  0  0  0  0  0  0  0  0  0  0
   -0.3201   -5.2294   -2.4389 C   0  0  0  0  0  0  0  0  0  0  0  0
    1.1390   -6.5003   -0.3957 C   0  0  0  0  0  0  0  0  0  0  0  0
    0.8807   -5.9027   -2.7469 C   0  0  0  0  0  0  0  0  0  0  0  0
    1.5987   -6.5324   -1.7230 C   0  0  0  0  0  0  0  0  0  0  0  0
   -3.0604   -5.2979    0.7431 C   0  0  0  0  0  0  0  0  0  0  0  0
   -3.0492   -6.6904    0.7624 C   0  0  0  0  0  0  0  0  0  0  0  0
   -3.5121   -4.5458    1.8277 C   0  0  0  0  0  0  0  0  0  0  0  0
   -3.5376   -7.3622    1.9000 C   0  0  0  0  0  0  0  0  0  0  0  0
   -4.0017   -5.2130    2.9663 C   0  0  0  0  0  0  0  0  0  0  0  0
   -4.0133   -6.6165    2.9884 C   0  0  0  0  0  0  0  0  0  0  0  0
   -3.4474   -4.7339   -2.2196 C   0  0  0  0  0  0  0  0  0  0  0  0
   -4.2266   -5.8792   -2.2967 C   0  0  0  0  0  0  0  0  0  0  0  0
   -3.3692   -3.8183   -3.2748 C   0  0  0  0  0  0  0  0  0  0  0  0
   -4.9481   -6.1397   -3.4833 C   0  0  0  0  0  0  0  0  0  0  0  0
   -4.0776   -4.0773   -4.4590 C   0  0  0  0  0  0  0  0  0  0  0  0
   -4.8624   -5.2417   -4.5519 C   0  0  0  0  0  0  0  0  0  0  0  0
   -8.7620   -4.6023   -2.5256 H   0  0  0  0  0  0  0  0  0  0  0  0
   -9.2787   -0.4645   -1.3517 H   0  0  0  0  0  0  0  0  0  0  0  0
  -10.5673   -2.6036   -2.3820 N   0  0  0  0  0  0  0  0  0  0  0  0
  -11.2285   -1.5941   -2.2396 O   0  0  0  0  0  0  0  0  0  0  0  0
  -10.9606   -3.6642   -2.8293 O   0  0  0  0  0  0  0  0  0  0  0  0
   -3.4777   -3.4440    1.8170 H   0  0  0  0  0  0  0  0  0  0  0  0
   -2.6495   -7.2810   -0.0645 H   0  0  0  0  0  0  0  0  0  0  0  0
   -0.4103   -5.8237    0.9392 H   0  0  0  0  0  0  0  0  0  0  0  0
   -0.8751   -4.7619   -3.2541 H   0  0  0  0  0  0  0  0  0  0  0  0
   -2.7735   -2.9008   -3.1864 H   0  0  0  0  0  0  0  0  0  0  0  0
   -4.3078   -6.5918   -1.4751 H   0  0  0  0  0  0  0  0  0  0  0  0
   -2.2842   -2.2744   -0.2511 Rh  0  0  0  0  0  0  0  0  0  0  0  0
```

```
  -4.3898   -7.1368    3.8716 H   0  0  0  0  0  0  0  0  0  0  0  0
   2.5236   -7.0630   -1.9557 H   0  0  0  0  0  0  0  0  0  0  0  0
  -5.4068   -5.4454   -5.4761 H   0  0  0  0  0  0  0  0  0  0  0  0
  -4.4956   -4.4508    4.1522 C   0  0  0  0  0  0  0  0  0  0  0  0
  -5.5452   -4.7550    4.3915 H   0  0  0  0  0  0  0  0  0  0  0  0
  -3.8543   -4.6780    5.0404 H   0  0  0  0  0  0  0  0  0  0  0  0
  -4.4811   -3.3509    3.9809 H   0  0  0  0  0  0  0  0  0  0  0  0
  -3.5369   -8.8541    1.9634 C   0  0  0  0  0  0  0  0  0  0  0  0
  -2.8245   -9.1957    2.7560 H   0  0  0  0  0  0  0  0  0  0  0  0
  -4.5572   -9.2306    2.2232 H   0  0  0  0  0  0  0  0  0  0  0  0
  -3.2304   -9.3154    0.9966 H   0  0  0  0  0  0  0  0  0  0  0  0
  -5.7819   -7.3738   -3.5854 C   0  0  0  0  0  0  0  0  0  0  0  0
  -5.1238   -8.2419   -3.8457 H   0  0  0  0  0  0  0  0  0  0  0  0
  -6.2961   -7.5972   -2.6213 H   0  0  0  0  0  0  0  0  0  0  0  0
  -6.5594   -7.2797   -4.3810 H   0  0  0  0  0  0  0  0  0  0  0  0
  -4.0176   -3.1363   -5.6166 C   0  0  0  0  0  0  0  0  0  0  0  0
  -3.6790   -3.6810   -6.5338 H   0  0  0  0  0  0  0  0  0  0  0  0
  -5.0340   -2.7146   -5.8167 H   0  0  0  0  0  0  0  0  0  0  0  0
  -3.3151   -2.2909   -5.4371 H   0  0  0  0  0  0  0  0  0  0  0  0
   1.3600   -5.9545   -4.1609 C   0  0  0  0  0  0  0  0  0  0  0  0
   0.7726   -6.7232   -4.7230 H   0  0  0  0  0  0  0  0  0  0  0  0
   1.2295   -4.9675   -4.6639 H   0  0  0  0  0  0  0  0  0  0  0  0
   2.4401   -6.2311   -4.2185 H   0  0  0  0  0  0  0  0  0  0  0  0
   1.9196   -7.1909    0.6745 C   0  0  0  0  0  0  0  0  0  0  0  0
   1.9474   -8.2904    0.4697 H   0  0  0  0  0  0  0  0  0  0  0  0
   2.9708   -6.8102    0.6891 H   0  0  0  0  0  0  0  0  0  0  0  0
   1.4776   -7.0348    1.6849 H   0  0  0  0  0  0  0  0  0  0  0  0
  1 61  1  0  0  0  0
  1 38  1  1  0  0  0
  1 32  1  0  0  0  0
  1 44  1  0  0  0  0
  2 11  1  0  0  0  0
  2  3  1  0  0  0  0
  2  4  1  0  0  0  0
  2 10  1  0  0  0  0
  3 17  1  0  0  0  0
  3 20  1  0  0  0  0
  3 12  1  0  0  0  0
  4  6  2  0  0  0  0
  4  8  1  0  0  0  0
  4 61  1  0  0  0  0
  5  7  1  0  0  0  0
  5 14  1  0  0  0  0
  5  6  1  0  0  0  0
  5 15  1  0  0  0  0
  6  9  1  0  0  0  0
  6 61  1  0  0  0  0
  7 21  1  0  0  0  0
  7 13  1  0  0  0  0
  7 16  1  0  0  0  0
 16 18  1  0  0  0  0
 16 17  2  0  0  0  0
 16 61  1  0  0  0  0
 17 19  1  0  0  0  0
 17 61  1  0  0  0  0
 22 61  1  0  0  0  0
 22 23  1  0  0  0  0
 23 24  1  0  0  0  0
 24 26  1  0  0  0  0
 24 25  2  0  0  0  0
 25 27  1  0  0  0  0
 25 28  1  0  0  0  0
 26 30  1  0  0  0  0
 26 29  2  0  0  0  0
 27 31  2  0  0  0  0
```

```
 27 50  1  0  0  0  0
 29 51  1  0  0  0  0
 29 31  1  0  0  0  0
 31 52  1  0  0  0  0
 32 33  2  0  0  0  0
 32 34  1  0  0  0  0
 33 57  1  0  0  0  0
 33 35  1  0  0  0  0
 34 36  2  0  0  0  0
 34 58  1  0  0  0  0
 35 85  1  0  0  0  0
 35 37  2  0  0  0  0
 36 37  1  0  0  0  0
 36 81  1  0  0  0  0
 37 63  1  0  0  0  0
 38 39  2  0  0  0  0
 38 40  1  0  0  0  0
 39 41  1  0  0  0  0
 39 56  1  0  0  0  0
 40 42  2  0  0  0  0
 40 55  1  0  0  0  0
 41 69  1  0  0  0  0
 41 43  2  0  0  0  0
 42 43  1  0  0  0  0
 42 65  1  0  0  0  0
 43 62  1  0  0  0  0
 44 45  1  0  0  0  0
 44 46  2  0  0  0  0
 45 60  1  0  0  0  0
 45 47  2  0  0  0  0
 46 59  1  0  0  0  0
 46 48  1  0  0  0  0
 47 49  1  0  0  0  0
 47 73  1  0  0  0  0
 48 49  2  0  0  0  0
 48 77  1  0  0  0  0
 49 64  1  0  0  0  0
 52 53  2  0  0  0  0
 52 54  2  0  0  0  0
 65 66  1  0  0  0  0
 65 67  1  0  0  0  0
 65 68  1  0  0  0  0
 69 71  1  0  0  0  0
 69 70  1  0  0  0  0
 69 72  1  0  0  0  0
 73 75  1  0  0  0  0
 73 74  1  0  0  0  0
 73 76  1  0  0  0  0
 77 80  1  0  0  0  0
 77 78  1  0  0  0  0
 77 79  1  0  0  0  0
 81 82  1  0  0  0  0
 81 83  1  0  0  0  0
 81 84  1  0  0  0  0
 85 87  1  0  0  0  0
 85 88  1  0  0  0  0
 85 86  1  0  0  0  0
M  END
$$$$
mol195
Optimized Catalyst 195
Optimized Catalyst 195
 88 95  0  0  0  0  0  0  0  0999 V2000
   -2.4435   -4.6213   -0.7232 P   0  0  2  0  0  4  0  0  0  0  0  0
   -0.3868    0.6013   -0.9946 C   0  0  0  0  0  0  0  0  0  0  0  0
```

0.7131	-0.3506	-0.4843	C	0	0	0	0	0	0	0	0	0	0	0	0
-1.7182	0.5528	-0.2925	C	0	0	0	0	0	0	0	0	0	0	0	0
-0.8643	0.3512	2.0862	C	0	0	0	0	0	0	0	0	0	0	0	0
-1.9373	0.4419	1.0368	C	0	0	0	0	0	0	0	0	0	0	0	0
-0.3835	-1.0866	2.3640	C	0	0	0	0	0	0	0	0	0	0	0	0
-2.5731	0.7492	-0.9687	H	0	0	0	0	0	0	0	0	0	0	0	0
-2.9643	0.5136	1.4373	H	0	0	0	0	0	0	0	0	0	0	0	0
-0.5447	0.4029	-2.0888	H	0	0	0	0	0	0	0	0	0	0	0	0
-0.0046	1.6593	-0.9012	H	0	0	0	0	0	0	0	0	0	0	0	0
1.4650	-0.4774	-1.3121	H	0	0	0	0	0	0	0	0	0	0	0	0
-1.0765	-1.5514	3.1180	H	0	0	0	0	0	0	0	0	0	0	0	0
-1.2615	0.7756	3.0493	H	0	0	0	0	0	0	0	0	0	0	0	0
-0.0005	1.0008	1.7870	H	0	0	0	0	0	0	0	0	0	0	0	0
-0.2519	-2.0202	1.1819	C	0	0	0	0	0	0	0	0	0	0	0	0
0.2378	-1.7107	-0.0526	C	0	0	0	0	0	0	0	0	0	0	0	0
-0.3350	-3.0894	1.4831	H	0	0	0	0	0	0	0	0	0	0	0	0
0.4557	-2.5198	-0.7803	H	0	0	0	0	0	0	0	0	0	0	0	0
1.2609	0.1241	0.3747	H	0	0	0	0	0	0	0	0	0	0	0	0
0.6370	-1.0265	2.8430	H	0	0	0	0	0	0	0	0	0	0	0	0
-4.2380	-2.0976	-0.6317	N	0	0	0	0	0	2	0	0	0	0	0	0
-5.3455	-2.1246	-0.9996	C	0	0	0	0	0	2	0	0	0	0	0	0
-6.6981	-2.1741	-1.4574	C	0	0	0	0	0	0	0	0	0	0	0	0
-7.1760	-3.3628	-2.0419	C	0	0	0	0	0	0	0	0	0	0	0	0
-7.5264	-1.0445	-1.3203	C	0	0	0	0	0	0	0	0	0	0	0	0
-8.4937	-3.4194	-2.4951	C	0	0	0	0	0	0	0	0	0	0	0	0
-6.5224	-4.2350	-2.1436	H	0	0	0	0	0	0	0	0	0	0	0	0
-8.8460	-1.1064	-1.7721	C	0	0	0	0	0	0	0	0	0	0	0	0
-7.1474	-0.1258	-0.8657	H	0	0	0	0	0	0	0	0	0	0	0	0
-9.3131	-2.2917	-2.3533	C	0	0	0	0	0	0	0	0	0	0	0	0
-0.7838	-5.3097	-1.1901	C	0	0	0	0	0	0	0	0	0	0	0	0
0.0977	-5.6552	-0.1614	C	0	0	0	0	0	0	0	0	0	0	0	0
-0.4668	-5.5036	-2.5295	C	0	0	0	0	0	0	0	0	0	0	0	0
1.3472	-6.2075	-0.4910	C	0	0	0	0	0	0	0	0	0	0	0	0
0.7866	-6.0628	-2.8637	C	0	0	0	0	0	0	0	0	0	0	0	0
1.6807	-6.4052	-1.8429	C	0	0	0	0	0	0	0	0	0	0	0	0
-3.0496	-5.6166	0.7242	C	0	0	0	0	0	0	0	0	0	0	0	0
-2.8135	-6.9869	0.7476	C	0	0	0	0	0	0	0	0	0	0	0	0
-3.7419	-4.9595	1.7422	C	0	0	0	0	0	0	0	0	0	0	0	0
-3.3113	-7.7459	1.8280	C	0	0	0	0	0	0	0	0	0	0	0	0
-4.2332	-5.7125	2.8251	C	0	0	0	0	0	0	0	0	0	0	0	0
-4.0169	-7.1010	2.8519	C	0	0	0	0	0	0	0	0	0	0	0	0
-3.6006	-4.9449	-2.1465	C	0	0	0	0	0	0	0	0	0	0	0	0
-4.4517	-6.0405	-2.1009	C	0	0	0	0	0	0	0	0	0	0	0	0
-3.5508	-4.0900	-3.2527	C	0	0	0	0	0	0	0	0	0	0	0	0
-5.2762	-6.3181	-3.2160	C	0	0	0	0	0	0	0	0	0	0	0	0
-4.3695	-4.3607	-4.3615	C	0	0	0	0	0	0	0	0	0	0	0	0
-5.2249	-5.4785	-4.3326	C	0	0	0	0	0	0	0	0	0	0	0	0
-8.8952	-4.3323	-2.9584	H	0	0	0	0	0	0	0	0	0	0	0	0
-9.5187	-0.2401	-1.6767	H	0	0	0	0	0	0	0	0	0	0	0	0
-10.7314	-2.3585	-2.8374	N	0	0	0	0	0	0	0	0	0	0	0	0
-11.4050	-1.3589	-2.6967	O	0	0	0	0	0	0	0	0	0	0	0	0
-11.0861	-3.4109	-3.3308	O	0	0	0	0	0	0	0	0	0	0	0	0
-3.9148	-3.8747	1.7306	H	0	0	0	0	0	0	0	0	0	0	0	0
-2.2449	-7.4963	-0.0347	H	0	0	0	0	0	0	0	0	0	0	0	0
-0.1613	-5.5475	0.8926	H	0	0	0	0	0	0	0	0	0	0	0	0
-1.1567	-5.2604	-3.3405	H	0	0	0	0	0	0	0	0	0	0	0	0
-2.8850	-3.2246	-3.2935	H	0	0	0	0	0	0	0	0	0	0	0	0
-4.5088	-6.7081	-1.2368	H	0	0	0	0	0	0	0	0	0	0	0	0
-4.4030	-7.6860	3.6901	H	0	0	0	0	0	0	0	0	0	0	0	0
2.6490	-6.8432	-2.0955	H	0	0	0	0	0	0	0	0	0	0	0	0
-5.8469	-5.6953	-5.2043	H	0	0	0	0	0	0	0	0	0	0	0	0
-4.9909	-5.0539	3.9300	C	0	0	0	0	0	0	0	0	0	0	0	0
-6.0885	-5.1277	3.7228	H	0	0	0	0	0	0	0	0	0	0	0	0
-4.7891	-5.5582	4.9078	H	0	0	0	0	0	0	0	0	0	0	0	0
-4.7191	-3.9789	4.0380	H	0	0	0	0	0	0	0	0	0	0	0	0

```
   -3.0695   -9.2173    1.8819 C   0  0  0  0  0  0  0  0  0  0  0  0
   -2.1248   -9.4162    2.4494 H   0  0  0  0  0  0  0  0  0  0  0  0
   -3.9054   -9.7459    2.4018 H   0  0  0  0  0  0  0  0  0  0  0  0
   -2.9668   -9.6553    0.8612 H   0  0  0  0  0  0  0  0  0  0  0  0
   -6.1865   -7.4997   -3.1803 C   0  0  0  0  0  0  0  0  0  0  0  0
   -5.5834   -8.4413   -3.1231 H   0  0  0  0  0  0  0  0  0  0  0  0
   -6.8563   -7.4542   -2.2879 H   0  0  0  0  0  0  0  0  0  0  0  0
   -6.8263   -7.5609   -4.0936 H   0  0  0  0  0  0  0  0  0  0  0  0
   -4.3357   -3.4833   -5.5679 C   0  0  0  0  0  0  0  0  0  0  0  0
   -3.7833   -4.0058   -6.3913 H   0  0  0  0  0  0  0  0  0  0  0  0
   -5.3709   -3.2668   -5.9298 H   0  0  0  0  0  0  0  0  0  0  0  0
   -3.8268   -2.5123   -5.3691 H   0  0  0  0  0  0  0  0  0  0  0  0
    1.1323   -6.2925   -4.2976 C   0  0  0  0  0  0  0  0  0  0  0  0
    0.4935   -7.1123   -4.7122 H   0  0  0  0  0  0  0  0  0  0  0  0
    0.9609   -5.3672   -4.8982 H   0  0  0  0  0  0  0  0  0  0  0  0
    2.2009   -6.5917   -4.4233 H   0  0  0  0  0  0  0  0  0  0  0  0
    2.3064   -6.6026    0.5824 C   0  0  0  0  0  0  0  0  0  0  0  0
    2.1845   -7.6940    0.8027 H   0  0  0  0  0  0  0  0  0  0  0  0
    3.3626   -6.4298    0.2604 H   0  0  0  0  0  0  0  0  0  0  0  0
    2.1348   -6.0318    1.5241 H   0  0  0  0  0  0  0  0  0  0  0  0
   -2.3999   -2.1073   -0.0581 Ir  0  0  0  0  0  0  0  0  0  0  0  0
  1 88  1  0  0  0  0
  1 38  1  1  0  0  0
  1 32  1  0  0  0  0
  1 44  1  0  0  0  0
  2 11  1  0  0  0  0
  2  3  1  0  0  0  0
  2  4  1  0  0  0  0
  2 10  1  0  0  0  0
  3 17  1  0  0  0  0
  3 20  1  0  0  0  0
  3 12  1  0  0  0  0
  4  6  2  0  0  0  0
  4  8  1  0  0  0  0
  4 88  1  0  0  0  0
  5  7  1  0  0  0  0
  5 14  1  0  0  0  0
  5  6  1  0  0  0  0
  5 15  1  0  0  0  0
  6  9  1  0  0  0  0
  6 88  1  0  0  0  0
  7 21  1  0  0  0  0
  7 13  1  0  0  0  0
  7 16  1  0  0  0  0
 16 18  1  0  0  0  0
 16 17  2  0  0  0  0
 16 88  1  0  0  0  0
 17 19  1  0  0  0  0
 17 88  1  0  0  0  0
 22 88  1  0  0  0  0
 22 23  1  0  0  0  0
 23 24  1  0  0  0  0
 24 26  1  0  0  0  0
 24 25  2  0  0  0  0
 25 27  1  0  0  0  0
 25 28  1  0  0  0  0
 26 30  1  0  0  0  0
 26 29  2  0  0  0  0
 27 31  2  0  0  0  0
 27 50  1  0  0  0  0
 29 51  1  0  0  0  0
 29 31  1  0  0  0  0
 31 52  1  0  0  0  0
 32 33  2  0  0  0  0
 32 34  1  0  0  0  0
```

```
 33 57  1  0  0  0  0
 33 35  1  0  0  0  0
 34 36  2  0  0  0  0
 34 58  1  0  0  0  0
 35 84  1  0  0  0  0
 35 37  2  0  0  0  0
 36 37  1  0  0  0  0
 36 80  1  0  0  0  0
 37 62  1  0  0  0  0
 38 39  2  0  0  0  0
 38 40  1  0  0  0  0
 39 41  1  0  0  0  0
 39 56  1  0  0  0  0
 40 42  2  0  0  0  0
 40 55  1  0  0  0  0
 41 68  1  0  0  0  0
 41 43  2  0  0  0  0
 42 43  1  0  0  0  0
 42 64  1  0  0  0  0
 43 61  1  0  0  0  0
 44 45  1  0  0  0  0
 44 46  2  0  0  0  0
 45 60  1  0  0  0  0
 45 47  2  0  0  0  0
 46 48  1  0  0  0  0
 46 59  1  0  0  0  0
 47 72  1  0  0  0  0
 47 49  1  0  0  0  0
 48 49  2  0  0  0  0
 48 76  1  0  0  0  0
 49 63  1  0  0  0  0
 52 53  2  0  0  0  0
 52 54  2  0  0  0  0
 64 67  1  0  0  0  0
 64 66  1  0  0  0  0
 64 65  1  0  0  0  0
 68 70  1  0  0  0  0
 68 69  1  0  0  0  0
 68 71  1  0  0  0  0
 72 73  1  0  0  0  0
 72 74  1  0  0  0  0
 72 75  1  0  0  0  0
 76 79  1  0  0  0  0
 76 77  1  0  0  0  0
 76 78  1  0  0  0  0
 80 81  1  0  0  0  0
 80 82  1  0  0  0  0
 80 83  1  0  0  0  0
 84 85  1  0  0  0  0
 84 87  1  0  0  0  0
 84 86  1  0  0  0  0
M  END
$$$$
mol196
Optimized Catalyst 196
Optimized Catalyst 196
 88 95  0  0  0  0  0  0  0999 V2000
   -2.4786   -4.7963   -0.5548 P   0  0  2  0  0  4  0  0  0  0  0  0
   -1.4898    1.0600   -1.5584 C   0  0  0  0  0  0  0  0  0  0  0  0
   -0.0099    0.6345   -1.4254 C   0  0  0  0  0  0  0  0  0  0  0  0
   -2.4755    0.2738   -0.7390 C   0  0  0  0  0  0  0  0  0  0  0  0
   -1.1487    0.1881    1.4096 C   0  0  0  0  0  0  0  0  0  0  0  0
   -2.3499   -0.0846    0.5497 C   0  0  0  0  0  0  0  0  0  0  0  0
   -0.1322   -0.9700    1.2881 C   0  0  0  0  0  0  0  0  0  0  0  0
   -3.3948    0.0172   -1.2795 H   0  0  0  0  0  0  0  0  0  0  0  0
```

```
   -3.1576   -0.6432    1.0594 H   0  0  0  0  0  0  0  0  0  0  0  0
   -1.7774    0.9781   -2.6370 H   0  0  0  0  0  0  0  0  0  0  0  0
   -1.5768    2.1383   -1.2574 H   0  0  0  0  0  0  0  0  0  0  0  0
    0.5331    0.9623   -2.3490 H   0  0  0  0  0  0  0  0  0  0  0  0
   -0.5001   -1.8045    1.9494 H   0  0  0  0  0  0  0  0  0  0  0  0
   -1.4631    0.2778    2.4802 H   0  0  0  0  0  0  0  0  0  0  0  0
   -0.6780    1.1590    1.1222 H   0  0  0  0  0  0  0  0  0  0  0  0
    0.0705   -1.5292   -0.1006 C   0  0  0  0  0  0  0  0  0  0  0  0
    0.1427   -0.8482   -1.2573 C   0  0  0  0  0  0  0  0  0  0  0  0
    0.2408   -2.6280   -0.1165 H   0  0  0  0  0  0  0  0  0  0  0  0
    0.3408   -1.3855   -2.1905 H   0  0  0  0  0  0  0  0  0  0  0  0
    0.4692    1.1605   -0.5624 H   0  0  0  0  0  0  0  0  0  0  0  0
    0.8622   -0.6210    1.6687 H   0  0  0  0  0  0  0  0  0  0  0  0
   -3.8877   -2.7801   -0.4286 N   0  0  0  0  0  2  0  0  0  0  0  0
   -5.0005   -2.5744   -0.7040 C   0  0  0  0  0  2  0  0  0  0  0  0
   -6.3743   -2.4095   -1.0666 C   0  0  0  0  0  0  0  0  0  0  0  0
   -6.8181   -2.8923   -2.3113 C   0  0  0  0  0  0  0  0  0  0  0  0
   -7.2562   -1.7713   -0.1765 C   0  0  0  0  0  0  0  0  0  0  0  0
   -8.1581   -2.7383   -2.6663 C   0  0  0  0  0  0  0  0  0  0  0  0
   -6.1202   -3.3821   -2.9961 H   0  0  0  0  0  0  0  0  0  0  0  0
   -8.5965   -1.6170   -0.5344 C   0  0  0  0  0  0  0  0  0  0  0  0
   -6.8989   -1.3941    0.7841 H   0  0  0  0  0  0  0  0  0  0  0  0
   -9.0328   -2.1044   -1.7734 C   0  0  0  0  0  0  0  0  0  0  0  0
   -0.7369   -5.4266   -0.8021 C   0  0  0  0  0  0  0  0  0  0  0  0
    0.0860   -5.3384    0.3510 C   0  0  0  0  0  0  0  0  0  0  0  0
   -0.2208   -6.0158   -1.9751 C   0  0  0  0  0  0  0  0  0  0  0  0
    1.4118   -5.7882    0.3047 C   0  0  0  0  0  0  0  0  0  0  0  0
    1.1288   -6.4294   -1.9969 C   0  0  0  0  0  0  0  0  0  0  0  0
    1.9396   -6.3191   -0.8740 C   0  0  0  0  0  0  0  0  0  0  0  0
   -3.1894   -5.9107    0.7612 C   0  0  0  0  0  0  0  0  0  0  0  0
   -3.0631   -7.3100    0.5863 C   0  0  0  0  0  0  0  0  0  0  0  0
   -3.7978   -5.4121    1.9346 C   0  0  0  0  0  0  0  0  0  0  0  0
   -3.5633   -8.1800    1.5727 C   0  0  0  0  0  0  0  0  0  0  0  0
   -4.3093   -6.3093    2.8873 C   0  0  0  0  0  0  0  0  0  0  0  0
   -4.1914   -7.6870    2.7119 C   0  0  0  0  0  0  0  0  0  0  0  0
   -3.4754   -4.8738   -2.1217 C   0  0  0  0  0  0  0  0  0  0  0  0
   -4.6340   -5.6548   -2.2994 C   0  0  0  0  0  0  0  0  0  0  0  0
   -2.9891   -4.0839   -3.1973 C   0  0  0  0  0  0  0  0  0  0  0  0
   -5.1453   -5.8188   -3.6037 C   0  0  0  0  0  0  0  0  0  0  0  0
   -3.5465   -4.2354   -4.4731 C   0  0  0  0  0  0  0  0  0  0  0  0
   -4.5905   -5.1406   -4.6842 C   0  0  0  0  0  0  0  0  0  0  0  0
   -8.5326   -3.1025   -3.6330 H   0  0  0  0  0  0  0  0  0  0  0  0
   -9.3079   -1.1188    0.1400 H   0  0  0  0  0  0  0  0  0  0  0  0
   -2.2418   -2.7485   -0.0118 Rh  0  0  0  0  0  0  0  0  0  0  0  0
    2.9811   -6.6517   -0.9083 H   0  0  0  0  0  0  0  0  0  0  0  0
   -4.5856   -8.3741    3.4663 H   0  0  0  0  0  0  0  0  0  0  0  0
   -4.9865   -5.2929   -5.6928 H   0  0  0  0  0  0  0  0  0  0  0  0
   -5.9934   -6.4862   -3.7707 H   0  0  0  0  0  0  0  0  0  0  0  0
   -3.1685   -3.6527   -5.3143 H   0  0  0  0  0  0  0  0  0  0  0  0
    1.5495   -6.8528   -2.9118 H   0  0  0  0  0  0  0  0  0  0  0  0
    2.0467   -5.7313    1.1906 H   0  0  0  0  0  0  0  0  0  0  0  0
   -4.8024   -5.9341    3.7861 H   0  0  0  0  0  0  0  0  0  0  0  0
   -3.4572   -9.2602    1.4535 H   0  0  0  0  0  0  0  0  0  0  0  0
   -0.9568   -6.2839   -3.2512 C   0  0  0  0  0  0  0  0  0  0  0  0
   -2.0452   -6.4642   -3.1269 H   0  0  0  0  0  0  0  0  0  0  0  0
   -0.8086   -5.4283   -3.9571 H   0  0  0  0  0  0  0  0  0  0  0  0
   -0.5448   -7.2008   -3.7483 H   0  0  0  0  0  0  0  0  0  0  0  0
   -0.3823   -4.8106    1.6781 C   0  0  0  0  0  0  0  0  0  0  0  0
   -0.9833   -3.8615    1.5992 H   0  0  0  0  0  0  0  0  0  0  0  0
   -0.9903   -5.5970    2.1993 H   0  0  0  0  0  0  0  0  0  0  0  0
    0.4886   -4.5713    2.3404 H   0  0  0  0  0  0  0  0  0  0  0  0
   -1.9288   -3.0360   -3.0196 C   0  0  0  0  0  0  0  0  0  0  0  0
   -1.5649   -2.6556   -4.0055 H   0  0  0  0  0  0  0  0  0  0  0  0
   -2.3565   -2.1476   -2.4573 H   0  0  0  0  0  0  0  0  0  0  0  0
   -1.0297   -3.4103   -2.4603 H   0  0  0  0  0  0  0  0  0  0  0  0
```

```
   -5.4385   -6.2766   -1.2036 C   0  0  0  0  0  0  0  0  0  0  0  0
   -5.0913   -7.3203   -0.9964 H   0  0  0  0  0  0  0  0  0  0  0  0
   -5.4056   -5.6900   -0.2570 H   0  0  0  0  0  0  0  0  0  0  0  0
   -6.5162   -6.3434   -1.5033 H   0  0  0  0  0  0  0  0  0  0  0  0
   -3.9719   -3.9601    2.2807 C   0  0  0  0  0  0  0  0  0  0  0  0
   -3.0684   -3.3235    2.0426 H   0  0  0  0  0  0  0  0  0  0  0  0
   -4.8807   -3.5571    1.7721 H   0  0  0  0  0  0  0  0  0  0  0  0
   -4.1309   -3.8395    3.3848 H   0  0  0  0  0  0  0  0  0  0  0  0
   -2.4254   -7.9830   -0.5900 C   0  0  0  0  0  0  0  0  0  0  0  0
   -1.3099   -7.8927   -0.5196 H   0  0  0  0  0  0  0  0  0  0  0  0
   -2.6581   -9.0786   -0.6026 H   0  0  0  0  0  0  0  0  0  0  0  0
   -2.7784   -7.5770   -1.5628 H   0  0  0  0  0  0  0  0  0  0  0  0
  -10.4689   -1.9420   -2.1557 N   0  0  0  0  0  0  0  0  0  0  0  0
  -11.2025   -1.4342   -1.3310 O   0  0  0  0  0  0  0  0  0  0  0  0
  -10.7879   -2.3345   -3.2613 O   0  0  0  0  0  0  0  0  0  0  0  0
  1 52  1  0  0  0  0
  1 38  1  1  0  0  0
  1 32  1  0  0  0  0
  1 44  1  0  0  0  0
  2  3  1  0  0  0  0
  2 11  1  0  0  0  0
  2  4  1  0  0  0  0
  2 10  1  0  0  0  0
  3 17  1  0  0  0  0
  3 20  1  0  0  0  0
  3 12  1  0  0  0  0
  4  6  2  0  0  0  0
  4  8  1  0  0  0  0
  4 52  1  0  0  0  0
  5 14  1  0  0  0  0
  5  6  1  0  0  0  0
  5  7  1  0  0  0  0
  5 15  1  0  0  0  0
  6  9  1  0  0  0  0
  6 52  1  0  0  0  0
  7 21  1  0  0  0  0
  7 13  1  0  0  0  0
  7 16  1  0  0  0  0
 16 18  1  0  0  0  0
 16 52  1  0  0  0  0
 16 17  2  0  0  0  0
 17 19  1  0  0  0  0
 17 52  1  0  0  0  0
 22 52  1  0  0  0  0
 22 23  1  0  0  0  0
 23 24  1  0  0  0  0
 24 26  1  0  0  0  0
 24 25  2  0  0  0  0
 25 27  1  0  0  0  0
 25 28  1  0  0  0  0
 26 30  1  0  0  0  0
 26 29  2  0  0  0  0
 27 31  2  0  0  0  0
 27 50  1  0  0  0  0
 29 51  1  0  0  0  0
 29 31  1  0  0  0  0
 31 86  1  0  0  0  0
 32 33  1  0  0  0  0
 32 34  2  0  0  0  0
 33 66  1  0  0  0  0
 33 35  2  0  0  0  0
 34 36  1  0  0  0  0
 34 62  1  0  0  0  0
 35 59  1  0  0  0  0
 35 37  1  0  0  0  0
```

```
 36 37  2  0  0  0  0
 36 58  1  0  0  0  0
 37 53  1  0  0  0  0
 38 40  1  0  0  0  0
 38 39  2  0  0  0  0
 39 41  1  0  0  0  0
 39 82  1  0  0  0  0
 40 78  1  0  0  0  0
 40 42  2  0  0  0  0
 41 43  2  0  0  0  0
 41 61  1  0  0  0  0
 42 60  1  0  0  0  0
 42 43  1  0  0  0  0
 43 54  1  0  0  0  0
 44 45  1  0  0  0  0
 44 46  2  0  0  0  0
 45 74  1  0  0  0  0
 45 47  2  0  0  0  0
 46 70  1  0  0  0  0
 46 48  1  0  0  0  0
 47 49  1  0  0  0  0
 47 56  1  0  0  0  0
 48 49  2  0  0  0  0
 48 57  1  0  0  0  0
 49 55  1  0  0  0  0
 62 63  1  0  0  0  0
 62 64  1  0  0  0  0
 62 65  1  0  0  0  0
 66 68  1  0  0  0  0
 66 69  1  0  0  0  0
 66 67  1  0  0  0  0
 70 73  1  0  0  0  0
 70 72  1  0  0  0  0
 70 71  1  0  0  0  0
 74 75  1  0  0  0  0
 74 76  1  0  0  0  0
 74 77  1  0  0  0  0
 78 81  1  0  0  0  0
 78 79  1  0  0  0  0
 78 80  1  0  0  0  0
 82 83  1  0  0  0  0
 82 84  1  0  0  0  0
 82 85  1  0  0  0  0
 86 87  2  0  0  0  0
 86 88  2  0  0  0  0
M  END
$$$$
mol197
Optimized Catalyst 197
Optimized Catalyst 197
 88 95  0  0  0  0  0  0  0  0999 V2000
   -2.5514   -4.9122   -0.5118 P   0  0  2  0  0  4  0  0  0  0  0  0
   -0.6242    0.5006   -1.6579 C   0  0  0  0  0  0  0  0  0  0  0  0
    0.6175   -0.1911   -1.0618 C   0  0  0  0  0  0  0  0  0  0  0  0
   -1.9070    0.4082   -0.8808 C   0  0  0  0  0  0  0  0  0  0  0  0
   -0.9631    0.6622    1.4546 C   0  0  0  0  0  0  0  0  0  0  0  0
   -2.0677    0.4652    0.4549 C   0  0  0  0  0  0  0  0  0  0  0  0
   -0.2989   -0.6560    1.9014 C   0  0  0  0  0  0  0  0  0  0  0  0
   -2.7978    0.3757   -1.5293 H   0  0  0  0  0  0  0  0  0  0  0  0
   -3.0824    0.4587    0.8901 H   0  0  0  0  0  0  0  0  0  0  0  0
   -0.7937    0.0854   -2.6851 H   0  0  0  0  0  0  0  0  0  0  0  0
   -0.3890    1.5977   -1.7720 H   0  0  0  0  0  0  0  0  0  0  0  0
    1.3645   -0.3309   -1.8898 H   0  0  0  0  0  0  0  0  0  0  0  0
   -0.9072   -1.0916    2.7420 H   0  0  0  0  0  0  0  0  0  0  0  0
   -1.3781    1.1720    2.3659 H   0  0  0  0  0  0  0  0  0  0  0  0
```

```
   -0.1970    1.3581    1.0252 H   0  0  0  0  0  0  0  0  0  0  0  0
   -0.0982   -1.7150    0.8400 C   0  0  0  0  0  0  0  0  0  0  0  0
    0.3334   -1.5188   -0.4288 C   0  0  0  0  0  0  0  0  0  0  0  0
   -0.0738   -2.7446    1.2581 H   0  0  0  0  0  0  0  0  0  0  0  0
    0.5581   -2.3891   -1.0693 H   0  0  0  0  0  0  0  0  0  0  0  0
    1.1019    0.4793   -0.3030 H   0  0  0  0  0  0  0  0  0  0  0  0
    0.7192   -0.4098    2.3180 H   0  0  0  0  0  0  0  0  0  0  0  0
   -4.3641   -2.3165   -0.2328 N   0  0  0  0  0  2  0  0  0  0  0  0
   -5.4561   -2.2025   -0.6301 C   0  0  0  0  0  2  0  0  0  0  0  0
   -6.7879   -2.0735   -1.1307 C   0  0  0  0  0  0  0  0  0  0  0  0
   -7.3311   -3.1220   -1.8981 C   0  0  0  0  0  0  0  0  0  0  0  0
   -7.5286   -0.9075   -0.8622 C   0  0  0  0  0  0  0  0  0  0  0  0
   -8.6243   -2.9995   -2.4049 C   0  0  0  0  0  0  0  0  0  0  0  0
   -6.7447   -4.0228   -2.1002 H   0  0  0  0  0  0  0  0  0  0  0  0
   -8.8235   -0.7886   -1.3718 C   0  0  0  0  0  0  0  0  0  0  0  0
   -7.1014   -0.0995   -0.2624 H   0  0  0  0  0  0  0  0  0  0  0  0
   -9.3544   -1.8336   -2.1377 C   0  0  0  0  0  0  0  0  0  0  0  0
   -0.7804   -5.3348   -0.8955 C   0  0  0  0  0  0  0  0  0  0  0  0
    0.1055   -5.3073    0.2162 C   0  0  0  0  0  0  0  0  0  0  0  0
   -0.2852   -5.6960   -2.1644 C   0  0  0  0  0  0  0  0  0  0  0  0
    1.4836   -5.4212    0.0013 C   0  0  0  0  0  0  0  0  0  0  0  0
    1.1129   -5.7755   -2.3510 C   0  0  0  0  0  0  0  0  0  0  0  0
    1.9912   -5.6204   -1.2871 C   0  0  0  0  0  0  0  0  0  0  0  0
   -3.0998   -6.1866    0.7301 C   0  0  0  0  0  0  0  0  0  0  0  0
   -2.7148   -7.5347    0.5454 C   0  0  0  0  0  0  0  0  0  0  0  0
   -3.8252   -5.8183    1.8861 C   0  0  0  0  0  0  0  0  0  0  0  0
   -2.9960   -8.4739    1.5566 C   0  0  0  0  0  0  0  0  0  0  0  0
   -4.1071   -6.7834    2.8631 C   0  0  0  0  0  0  0  0  0  0  0  0
   -3.6801   -8.1040    2.7091 C   0  0  0  0  0  0  0  0  0  0  0  0
   -3.6342   -4.8762   -2.0212 C   0  0  0  0  0  0  0  0  0  0  0  0
   -4.7455   -5.7310   -2.1843 C   0  0  0  0  0  0  0  0  0  0  0  0
   -3.2855   -3.9750   -3.0635 C   0  0  0  0  0  0  0  0  0  0  0  0
   -5.3980   -5.7723   -3.4345 C   0  0  0  0  0  0  0  0  0  0  0  0
   -3.9789   -4.0210   -4.2795 C   0  0  0  0  0  0  0  0  0  0  0  0
   -5.0101   -4.9424   -4.4794 C   0  0  0  0  0  0  0  0  0  0  0  0
   -9.0752   -3.8004   -3.0086 H   0  0  0  0  0  0  0  0  0  0  0  0
   -9.4269    0.1119   -1.1797 H   0  0  0  0  0  0  0  0  0  0  0  0
    3.0719   -5.6862   -1.4461 H   0  0  0  0  0  0  0  0  0  0  0  0
   -3.8906   -8.8436    3.4880 H   0  0  0  0  0  0  0  0  0  0  0  0
   -5.5160   -4.9979   -5.4486 H   0  0  0  0  0  0  0  0  0  0  0  0
   -6.2163   -6.4789   -3.5953 H   0  0  0  0  0  0  0  0  0  0  0  0
   -3.7133   -3.3454   -5.0953 H   0  0  0  0  0  0  0  0  0  0  0  0
    1.5119   -5.9912   -3.3456 H   0  0  0  0  0  0  0  0  0  0  0  0
    2.1804   -5.3825    0.8413 H   0  0  0  0  0  0  0  0  0  0  0  0
   -4.6674   -6.5153    3.7614 H   0  0  0  0  0  0  0  0  0  0  0  0
   -2.6757   -9.5122    1.4399 H   0  0  0  0  0  0  0  0  0  0  0  0
   -1.0833   -6.0899   -3.3676 C   0  0  0  0  0  0  0  0  0  0  0  0
   -2.1621   -6.2732   -3.1802 H   0  0  0  0  0  0  0  0  0  0  0  0
   -0.9916   -5.3214   -4.1749 H   0  0  0  0  0  0  0  0  0  0  0  0
   -0.6657   -7.0508   -3.7745 H   0  0  0  0  0  0  0  0  0  0  0  0
   -0.3319   -5.2555    1.6528 C   0  0  0  0  0  0  0  0  0  0  0  0
   -1.1718   -4.5579    1.8792 H   0  0  0  0  0  0  0  0  0  0  0  0
   -0.6528   -6.2939    1.9526 H   0  0  0  0  0  0  0  0  0  0  0  0
    0.5212   -4.9804    2.3252 H   0  0  0  0  0  0  0  0  0  0  0  0
   -2.2052   -2.9321   -2.9635 C   0  0  0  0  0  0  0  0  0  0  0  0
   -1.1885   -3.3977   -2.9241 H   0  0  0  0  0  0  0  0  0  0  0  0
   -2.2120   -2.2708   -3.8701 H   0  0  0  0  0  0  0  0  0  0  0  0
   -2.3570   -2.2472   -2.0734 H   0  0  0  0  0  0  0  0  0  0  0  0
   -5.3498   -6.5962   -1.1255 C   0  0  0  0  0  0  0  0  0  0  0  0
   -4.7476   -7.5294   -0.9825 H   0  0  0  0  0  0  0  0  0  0  0  0
   -5.4447   -6.0716   -0.1480 H   0  0  0  0  0  0  0  0  0  0  0  0
   -6.3811   -6.9263   -1.4128 H   0  0  0  0  0  0  0  0  0  0  0  0
   -4.3754   -4.4439    2.1496 C   0  0  0  0  0  0  0  0  0  0  0  0
   -3.5875   -3.6314    2.0873 H   0  0  0  0  0  0  0  0  0  0  0  0
   -5.2351   -4.2435    1.4638 H   0  0  0  0  0  0  0  0  0  0  0  0
```

```
   -4.7841   -4.3812    3.1956 H   0  0  0  0  0  0  0  0  0  0  0  0
   -2.0287   -8.0831   -0.6668 C   0  0  0  0  0  0  0  0  0  0  0  0
   -0.9336   -7.8476   -0.6292 H   0  0  0  0  0  0  0  0  0  0  0  0
   -2.1180   -9.1994   -0.7056 H   0  0  0  0  0  0  0  0  0  0  0  0
   -2.4620   -7.7019   -1.6170 H   0  0  0  0  0  0  0  0  0  0  0  0
  -10.7432   -1.7040   -2.6911 N   0  0  0  0  0  0  0  0  0  0  0  0
  -11.3477   -0.6883   -2.4169 O   0  0  0  0  0  0  0  0  0  0  0  0
  -11.1440   -2.6289   -3.3691 O   0  0  0  0  0  0  0  0  0  0  0  0
   -2.5185   -2.4148    0.3113 Ir  0  0  0  0  0  0  0  0  0  0  0  0
  1 88  1  0  0  0  0
  1 38  1  1  0  0  0
  1 32  1  0  0  0  0
  1 44  1  0  0  0  0
  2  3  1  0  0  0  0
  2  4  1  0  0  0  0
  2 10  1  0  0  0  0
  2 11  1  0  0  0  0
  3 17  1  0  0  0  0
  3 20  1  0  0  0  0
  3 12  1  0  0  0  0
  4  6  2  0  0  0  0
  4  8  1  0  0  0  0
  4 88  1  0  0  0  0
  5  7  1  0  0  0  0
  5 14  1  0  0  0  0
  5  6  1  0  0  0  0
  5 15  1  0  0  0  0
  6  9  1  0  0  0  0
  6 88  1  0  0  0  0
  7 21  1  0  0  0  0
  7 13  1  0  0  0  0
  7 16  1  0  0  0  0
 16 18  1  0  0  0  0
 16 17  2  0  0  0  0
 16 88  1  0  0  0  0
 17 19  1  0  0  0  0
 17 88  1  0  0  0  0
 22 88  1  0  0  0  0
 22 23  1  0  0  0  0
 23 24  1  0  0  0  0
 24 26  1  0  0  0  0
 24 25  2  0  0  0  0
 25 27  1  0  0  0  0
 25 28  1  0  0  0  0
 26 30  1  0  0  0  0
 26 29  2  0  0  0  0
 27 31  2  0  0  0  0
 27 50  1  0  0  0  0
 29 51  1  0  0  0  0
 29 31  1  0  0  0  0
 31 85  1  0  0  0  0
 32 33  2  0  0  0  0
 32 34  1  0  0  0  0
 33 65  1  0  0  0  0
 33 35  1  0  0  0  0
 34 36  2  0  0  0  0
 34 61  1  0  0  0  0
 35 58  1  0  0  0  0
 35 37  2  0  0  0  0
 36 37  1  0  0  0  0
 36 57  1  0  0  0  0
 37 52  1  0  0  0  0
 38 40  1  0  0  0  0
 38 39  2  0  0  0  0
 39 41  1  0  0  0  0
```

```
 39 81  1  0  0  0  0
 40 77  1  0  0  0  0
 40 42  2  0  0  0  0
 41 43  2  0  0  0  0
 41 60  1  0  0  0  0
 42 59  1  0  0  0  0
 42 43  1  0  0  0  0
 43 53  1  0  0  0  0
 44 45  1  0  0  0  0
 44 46  2  0  0  0  0
 45 73  1  0  0  0  0
 45 47  2  0  0  0  0
 46 69  1  0  0  0  0
 46 48  1  0  0  0  0
 47 49  1  0  0  0  0
 47 55  1  0  0  0  0
 48 49  2  0  0  0  0
 48 56  1  0  0  0  0
 49 54  1  0  0  0  0
 61 62  1  0  0  0  0
 61 63  1  0  0  0  0
 61 64  1  0  0  0  0
 65 66  1  0  0  0  0
 65 67  1  0  0  0  0
 65 68  1  0  0  0  0
 69 70  1  0  0  0  0
 69 72  1  0  0  0  0
 69 71  1  0  0  0  0
 73 74  1  0  0  0  0
 73 75  1  0  0  0  0
 73 76  1  0  0  0  0
 77 80  1  0  0  0  0
 77 78  1  0  0  0  0
 77 79  1  0  0  0  0
 81 82  1  0  0  0  0
 81 83  1  0  0  0  0
 81 84  1  0  0  0  0
 85 86  2  0  0  0  0
 85 87  2  0  0  0  0
M  END
$$$$
mol198
Optimized Catalyst 198
Optimized Catalyst 198
 67 74  0  0  1  0  0  0  0  0999 V2000
   -2.2570   -4.3531   -0.5904 P   0  0  2  0  0  4  0  0  0  0  0  0
   -0.6390    0.1802   -1.5129 C   0  0  0  0  0  0  0  0  0  0  0  0
    0.5406   -0.5388   -0.8317 C   0  0  0  0  0  0  0  0  0  0  0  0
   -1.9669    0.0532   -0.8106 C   0  0  0  0  0  0  0  0  0  0  0  0
   -1.1702    0.2553    1.6025 C   0  0  0  0  0  0  0  0  0  0  0  0
   -2.2026    0.0513    0.5258 C   0  0  0  0  0  0  0  0  0  0  0  0
   -0.5703   -1.0778    2.0914 C   0  0  0  0  0  0  0  0  0  0  0  0
   -2.8220    0.0153   -1.5105 H   0  0  0  0  0  0  0  0  0  0  0  0
   -3.2350   -0.0432    0.9002 H   0  0  0  0  0  0  0  0  0  0  0  0
   -0.7532   -0.2471   -2.5456 H   0  0  0  0  0  0  0  0  0  0  0  0
   -0.3942    1.2722   -1.6098 H   0  0  0  0  0  0  0  0  0  0  0  0
    1.3473   -0.6863   -1.5995 H   0  0  0  0  0  0  0  0  0  0  0  0
   -1.3033   -1.5403    2.8070 H   0  0  0  0  0  0  0  0  0  0  0  0
   -1.6539    0.7654    2.4780 H   0  0  0  0  0  0  0  0  0  0  0  0
   -0.3661    0.9391    1.2328 H   0  0  0  0  0  0  0  0  0  0  0  0
   -0.2563   -2.1080    1.0318 C   0  0  0  0  0  0  0  0  0  0  0  0
    0.1804   -1.8832   -0.2492 C   0  0  0  0  0  0  0  0  0  0  0  0
   -0.2439   -3.1336    1.4441 H   0  0  0  0  0  0  0  0  0  0  0  0
    0.4872   -2.7281   -0.8854 H   0  0  0  0  0  0  0  0  0  0  0  0
    0.9748    0.1041   -0.0234 H   0  0  0  0  0  0  0  0  0  0  0  0
```

```
    0.3834   -0.8629    2.6474 H   0  0  0  0  0  0  0  0  0  0  0  0
   -3.7530   -2.3259   -0.6045 N   0  0  0  0  0  2  0  0  0  0  0  0
   -4.9050   -2.2021   -0.7287 C   0  0  0  0  0  2  0  0  0  0  0  0
   -6.3252   -2.1195   -0.8868 C   0  0  0  0  0  0  0  0  0  0  0  0
   -7.0682   -3.2969   -1.1017 C   0  0  0  0  0  0  0  0  0  0  0  0
   -6.9560   -0.8653   -0.8112 C   0  0  0  0  0  0  0  0  0  0  0  0
   -8.4534   -3.2131   -1.2520 C   0  0  0  0  0  0  0  0  0  0  0  0
   -6.5743   -4.2686   -1.1459 H   0  0  0  0  0  0  0  0  0  0  0  0
   -8.3433   -0.7855   -0.9576 C   0  0  0  0  0  0  0  0  0  0  0  0
   -6.3711    0.0412   -0.6395 H   0  0  0  0  0  0  0  0  0  0  0  0
   -9.0749   -1.9577   -1.1774 C   0  0  0  0  0  0  0  0  0  0  0  0
   -9.0598   -4.1129   -1.4262 H   0  0  0  0  0  0  0  0  0  0  0  0
   -8.8627    0.1835   -0.9037 H   0  0  0  0  0  0  0  0  0  0  0  0
  -10.5612   -1.8715   -1.3337 N   0  0  0  0  0  0  0  0  0  0  0  0
  -11.0485   -0.7593   -1.3334 O   0  0  0  0  0  0  0  0  0  0  0  0
  -11.1589   -2.9263   -1.4442 O   0  0  0  0  0  0  0  0  0  0  0  0
   -2.0892   -2.2406   -0.2477 Rh  0  0  0  0  0  0  0  0  0  0  0  0
   -3.1100   -5.1485    0.9133 C   0  0  1  0  0  0  0  0  0  0  0  0
   -2.3341   -5.5752    1.6017 H   0  0  0  0  0  0  0  0  0  0  0  0
   -3.9349   -4.1039    1.6409 C   0  0  0  0  0  0  0  0  0  0  0  0
   -4.1393   -6.1991    0.5556 C   0  0  0  0  0  0  0  0  0  0  0  0
   -3.4884   -3.1967    2.0364 H   0  0  0  0  0  0  0  0  0  0  0  0
   -5.2216   -4.5206    1.7157 C   0  0  0  0  0  0  0  0  0  0  0  0
   -3.8830   -7.1155    0.0522 H   0  0  0  0  0  0  0  0  0  0  0  0
   -5.3489   -5.8244    1.0352 C   0  0  0  0  0  0  0  0  0  0  0  0
   -6.0542   -4.0282    2.1935 H   0  0  0  0  0  0  0  0  0  0  0  0
   -6.2826   -6.3621    0.9757 H   0  0  0  0  0  0  0  0  0  0  0  0
   -0.6173   -5.3121   -0.7158 C   0  0  1  0  0  0  0  0  0  0  0  0
    0.0836   -4.9742    0.0890 H   0  0  0  0  0  0  0  0  0  0  0  0
   -0.8044   -6.8150   -0.6110 C   0  0  0  0  0  0  0  0  0  0  0  0
    0.0204   -5.1528   -2.0831 C   0  0  0  0  0  0  0  0  0  0  0  0
   -1.2169   -7.2949    0.2586 H   0  0  0  0  0  0  0  0  0  0  0  0
   -0.2963   -7.4092   -1.7160 C   0  0  0  0  0  0  0  0  0  0  0  0
    0.2718   -4.1958   -2.5065 H   0  0  0  0  0  0  0  0  0  0  0  0
    0.2156   -6.3737   -2.6342 C   0  0  0  0  0  0  0  0  0  0  0  0
   -0.2437   -8.4640   -1.9373 H   0  0  0  0  0  0  0  0  0  0  0  0
    0.6632   -6.6065   -3.5880 H   0  0  0  0  0  0  0  0  0  0  0  0
   -3.2821   -4.7259   -2.1385 C   0  0  1  0  0  0  0  0  0  0  0  0
   -4.3689   -4.5667   -1.9284 H   0  0  0  0  0  0  0  0  0  0  0  0
   -2.7726   -3.7965   -3.2280 C   0  0  0  0  0  0  0  0  0  0  0  0
   -3.0996   -6.0973   -2.7554 C   0  0  0  0  0  0  0  0  0  0  0  0
   -2.7255   -2.7222   -3.0962 H   0  0  0  0  0  0  0  0  0  0  0  0
   -2.4300   -4.5285   -4.3132 C   0  0  0  0  0  0  0  0  0  0  0  0
   -3.3527   -7.0046   -2.2362 H   0  0  0  0  0  0  0  0  0  0  0  0
   -2.6420   -5.9607   -4.0208 C   0  0  0  0  0  0  0  0  0  0  0  0
   -2.0542   -4.1773   -5.2614 H   0  0  0  0  0  0  0  0  0  0  0  0
   -2.4394   -6.7389   -4.7398 H   0  0  0  0  0  0  0  0  0  0  0  0
  1 37  1  1  0  0  0
  1 38  1  0  0  0  0
  1 48  1  0  0  0  0
  1 58  1  0  0  0  0
  2  3  1  0  0  0  0
  2  4  1  0  0  0  0
  2 10  1  0  0  0  0
  2 11  1  0  0  0  0
  3 17  1  0  0  0  0
  3 20  1  0  0  0  0
  3 12  1  0  0  0  0
  4  6  2  0  0  0  0
  4  8  1  0  0  0  0
  4 37  1  0  0  0  0
  5  7  1  0  0  0  0
  5 14  1  0  0  0  0
  5  6  1  0  0  0  0
  5 15  1  0  0  0  0
```

```
  6  9  1  0  0  0  0
  6 37  1  0  0  0  0
  7 21  1  0  0  0  0
  7 13  1  0  0  0  0
  7 16  1  0  0  0  0
 16 18  1  0  0  0  0
 16 17  2  0  0  0  0
 16 37  1  0  0  0  0
 17 19  1  0  0  0  0
 17 37  1  0  0  0  0
 22 37  1  0  0  0  0
 22 23  1  0  0  0  0
 23 24  1  0  0  0  0
 24 26  1  0  0  0  0
 24 25  2  0  0  0  0
 25 27  1  0  0  0  0
 25 28  1  0  0  0  0
 26 30  1  0  0  0  0
 26 29  2  0  0  0  0
 27 31  2  0  0  0  0
 27 32  1  0  0  0  0
 29 33  1  0  0  0  0
 29 31  1  0  0  0  0
 31 34  1  0  0  0  0
 34 35  2  0  0  0  0
 34 36  2  0  0  0  0
 38 39  1  1  0  0  0
 38 40  1  0  0  0  0
 38 41  1  0  0  0  0
 40 43  2  0  0  0  0
 40 42  1  0  0  0  0
 41 45  2  0  0  0  0
 41 44  1  0  0  0  0
 43 46  1  0  0  0  0
 43 45  1  0  0  0  0
 45 47  1  0  0  0  0
 48 50  1  0  0  0  0
 48 49  1  1  0  0  0
 48 51  1  0  0  0  0
 50 52  1  0  0  0  0
 50 53  2  0  0  0  0
 51 54  1  0  0  0  0
 51 55  2  0  0  0  0
 53 55  1  0  0  0  0
 53 56  1  0  0  0  0
 55 57  1  0  0  0  0
 58 59  1  1  0  0  0
 58 60  1  0  0  0  0
 58 61  1  0  0  0  0
 60 62  1  0  0  0  0
 60 63  2  0  0  0  0
 61 64  1  0  0  0  0
 61 65  2  0  0  0  0
 63 65  1  0  0  0  0
 63 66  1  0  0  0  0
 65 67  1  0  0  0  0
M  END
$$$$
mol199
Optimized Catalyst 199
Optimized Catalyst 199
 67 74  0  0  1  0  0  0  0  0999 V2000
   -2.3155   -4.6872   -0.8154 P   0  0  1  0  0  4  0  0  0  0  0  0
   -0.7279    1.2087   -0.7263 C   0  0  0  0  0  0  0  0  0  0  0  0
    0.5642    0.4480   -0.3740 C   0  0  0  0  0  0  0  0  0  0  0  0
```

```
   -2.0088    0.7192   -0.1089 C   0  0  0  0  0  0  0  0  0  0  0  0
   -1.1205    0.0872    2.1842 C   0  0  0  0  0  0  0  0  0  0  0  0
   -2.1936    0.2366    1.1402 C   0  0  0  0  0  0  0  0  0  0  0  0
   -0.3473   -1.2456    2.0930 C   0  0  0  0  0  0  0  0  0  0  0  0
   -2.8891    0.8995   -0.7545 H   0  0  0  0  0  0  0  0  0  0  0  0
   -3.2174    0.0210    1.5036 H   0  0  0  0  0  0  0  0  0  0  0  0
   -0.8401    1.2109   -1.8439 H   0  0  0  0  0  0  0  0  0  0  0  0
   -0.5988    2.2829   -0.4020 H   0  0  0  0  0  0  0  0  0  0  0  0
    1.3441    0.7223   -1.1385 H   0  0  0  0  0  0  0  0  0  0  0  0
   -0.9210   -2.0291    2.6632 H   0  0  0  0  0  0  0  0  0  0  0  0
   -1.5889    0.1482    3.2049 H   0  0  0  0  0  0  0  0  0  0  0  0
   -0.4150    0.9561    2.1128 H   0  0  0  0  0  0  0  0  0  0  0  0
   -0.0513   -1.7746    0.7028 C   0  0  0  0  0  0  0  0  0  0  0  0
    0.3992   -1.0403   -0.3507 C   0  0  0  0  0  0  0  0  0  0  0  0
    0.0979   -2.8766    0.6878 H   0  0  0  0  0  0  0  0  0  0  0  0
    0.7302   -1.5482   -1.2746 H   0  0  0  0  0  0  0  0  0  0  0  0
    0.9598    0.7927    0.6193 H   0  0  0  0  0  0  0  0  0  0  0  0
    0.6438   -1.1096    2.6141 H   0  0  0  0  0  0  0  0  0  0  0  0
   -4.1854   -2.1666   -0.6720 N   0  0  0  0  0  2  0  0  0  0  0  0
   -5.3286   -2.1384   -0.9111 C   0  0  0  0  0  2  0  0  0  0  0  0
   -6.7263   -2.1012   -1.2063 C   0  0  0  0  0  0  0  0  0  0  0  0
   -7.3392   -3.2156   -1.8125 C   0  0  0  0  0  0  0  0  0  0  0  0
   -7.4677   -0.9473   -0.8882 C   0  0  0  0  0  0  0  0  0  0  0  0
   -8.7030   -3.1714   -2.1041 C   0  0  0  0  0  0  0  0  0  0  0  0
   -6.7611   -4.1073   -2.0584 H   0  0  0  0  0  0  0  0  0  0  0  0
   -8.8326   -0.9077   -1.1814 C   0  0  0  0  0  0  0  0  0  0  0  0
   -6.9868   -0.0863   -0.4166 H   0  0  0  0  0  0  0  0  0  0  0  0
   -9.4331   -2.0186   -1.7856 C   0  0  0  0  0  0  0  0  0  0  0  0
   -9.2095   -4.0253   -2.5801 H   0  0  0  0  0  0  0  0  0  0  0  0
   -9.4373   -0.0179   -0.9453 H   0  0  0  0  0  0  0  0  0  0  0  0
  -10.9010   -1.9745   -2.1028 N   0  0  0  0  0  0  0  0  0  0  0  0
  -11.4832   -0.9448   -1.8327 O   0  0  0  0  0  0  0  0  0  0  0  0
  -11.3761   -2.9740   -2.6010 O   0  0  0  0  0  0  0  0  0  0  0  0
   -3.1029   -5.6061    0.6510 C   0  0  1  0  0  0  0  0  0  0  0  0
   -2.9155   -6.7069    0.5986 H   0  0  0  0  0  0  0  0  0  0  0  0
   -2.5571   -4.9933    1.9257 C   0  0  0  0  0  0  0  0  0  0  0  0
   -4.5832   -5.2943    0.7276 C   0  0  0  0  0  0  0  0  0  0  0  0
   -1.5322   -5.1138    2.2406 H   0  0  0  0  0  0  0  0  0  0  0  0
   -3.5726   -4.4089    2.6086 C   0  0  0  0  0  0  0  0  0  0  0  0
   -5.2927   -5.6601    0.0047 H   0  0  0  0  0  0  0  0  0  0  0  0
   -4.8357   -4.6013    1.8637 C   0  0  0  0  0  0  0  0  0  0  0  0
   -3.5296   -3.9092    3.5659 H   0  0  0  0  0  0  0  0  0  0  0  0
   -5.7890   -4.2563    2.2350 H   0  0  0  0  0  0  0  0  0  0  0  0
   -0.5818   -5.4350   -0.9668 C   0  0  1  0  0  0  0  0  0  0  0  0
   -0.0177   -5.3051   -0.0080 H   0  0  0  0  0  0  0  0  0  0  0  0
   -0.6252   -6.9084   -1.3331 C   0  0  0  0  0  0  0  0  0  0  0  0
    0.1670   -4.8212   -2.1339 C   0  0  0  0  0  0  0  0  0  0  0  0
   -1.0733   -7.6543   -0.6995 H   0  0  0  0  0  0  0  0  0  0  0  0
    0.0303   -7.0982   -2.5008 C   0  0  0  0  0  0  0  0  0  0  0  0
    0.4029   -3.7753   -2.1982 H   0  0  0  0  0  0  0  0  0  0  0  0
    0.5213   -5.7983   -3.0013 C   0  0  0  0  0  0  0  0  0  0  0  0
    0.2002   -8.0281   -3.0225 H   0  0  0  0  0  0  0  0  0  0  0  0
    1.0756   -5.6995   -3.9230 H   0  0  0  0  0  0  0  0  0  0  0  0
   -3.3708   -5.1192   -2.3284 C   0  0  1  0  0  0  0  0  0  0  0  0
   -4.3825   -4.6541   -2.2330 H   0  0  0  0  0  0  0  0  0  0  0  0
   -2.6934   -4.6687   -3.6096 C   0  0  0  0  0  0  0  0  0  0  0  0
   -3.5088   -6.6182   -2.5283 C   0  0  0  0  0  0  0  0  0  0  0  0
   -2.4517   -3.6396   -3.8130 H   0  0  0  0  0  0  0  0  0  0  0  0
   -2.5203   -5.7373   -4.4212 C   0  0  0  0  0  0  0  0  0  0  0  0
   -3.9710   -7.2640   -1.8027 H   0  0  0  0  0  0  0  0  0  0  0  0
   -3.0304   -6.9504   -3.7492 C   0  0  0  0  0  0  0  0  0  0  0  0
   -2.0868   -5.7570   -5.4101 H   0  0  0  0  0  0  0  0  0  0  0  0
   -3.0051   -7.9273   -4.2088 H   0  0  0  0  0  0  0  0  0  0  0  0
   -2.3248   -2.1444   -0.2081 Ir  0  0  0  0  0  0  0  0  0  0  0  0
  1 67  1  1  0  0  0
```

```
  1 37  1  0  0  0  0
  1 47  1  0  0  0  0
  1 57  1  0  0  0  0
  2 11  1  0  0  0  0
  2  3  1  0  0  0  0
  2  4  1  0  0  0  0
  2 10  1  0  0  0  0
  3 17  1  0  0  0  0
  3 20  1  0  0  0  0
  3 12  1  0  0  0  0
  4  6  2  0  0  0  0
  4  8  1  0  0  0  0
  4 67  1  0  0  0  0
  5 14  1  0  0  0  0
  5  6  1  0  0  0  0
  5  7  1  0  0  0  0
  5 15  1  0  0  0  0
  6  9  1  0  0  0  0
  6 67  1  0  0  0  0
  7 21  1  0  0  0  0
  7 13  1  0  0  0  0
  7 16  1  0  0  0  0
 16 18  1  0  0  0  0
 16 17  2  0  0  0  0
 16 67  1  0  0  0  0
 17 19  1  0  0  0  0
 17 67  1  0  0  0  0
 22 67  1  0  0  0  0
 22 23  1  0  0  0  0
 23 24  1  0  0  0  0
 24 26  2  0  0  0  0
 24 25  1  0  0  0  0
 25 27  2  0  0  0  0
 25 28  1  0  0  0  0
 26 30  1  0  0  0  0
 26 29  1  0  0  0  0
 27 31  1  0  0  0  0
 27 32  1  0  0  0  0
 29 33  1  0  0  0  0
 29 31  2  0  0  0  0
 31 34  1  0  0  0  0
 34 35  2  0  0  0  0
 34 36  2  0  0  0  0
 37 38  1  6  0  0  0
 37 40  1  0  0  0  0
 37 39  1  0  0  0  0
 39 41  1  0  0  0  0
 39 42  2  0  0  0  0
 40 44  2  0  0  0  0
 40 43  1  0  0  0  0
 42 45  1  0  0  0  0
 42 44  1  0  0  0  0
 44 46  1  0  0  0  0
 47 48  1  1  0  0  0
 47 49  1  0  0  0  0
 47 50  1  0  0  0  0
 49 51  1  0  0  0  0
 49 52  2  0  0  0  0
 50 53  1  0  0  0  0
 50 54  2  0  0  0  0
 52 54  1  0  0  0  0
 52 55  1  0  0  0  0
 54 56  1  0  0  0  0
 57 58  1  1  0  0  0
 57 59  1  0  0  0  0
```

```
 57 60  1  0  0  0  0
 59 61  1  0  0  0  0
 59 62  2  0  0  0  0
 60 63  1  0  0  0  0
 60 64  2  0  0  0  0
 62 64  1  0  0  0  0
 62 65  1  0  0  0  0
 64 66  1  0  0  0  0
M  END
$$$$
```

References

[1] Todeschini R, Consonni V. Handbook of Molecular Descriptors 2000. https://doi.org/10.1002/9783527613106.

[2] Ponce YM. Total and Local Quadratic Indices of the Molecular Pseudograph's Atom Adjacency Matrix: Applications to the Prediction of Physical Properties of Organic Compounds. Mol 2003, Vol 8, Pages 687-726 2003;8:687–726. https://doi.org/10.3390/80900687.

[3] Marrero-Ponce Y, Martínez-Albelo ER, Casañola-Martín GM, Castillo-Garit JA, Echevería-Díaz Y, Zaldivar VR, et al. Bond-based linear indices of the non-stochastic and stochastic edge-adjacency matrix. 1. Theory and modeling of ChemPhys properties of organic molecules. Mol Divers 2010;14:731–53. https://doi.org/10.1007/S11030-009-9201-5/METRICS.

[4] Cubillán N, Marrero-Ponce Y, Ariza-Rico H, Barigye SJ, García-Jacas CR, Valdes-Martini JR, et al. Novel global and local 3D atom-based linear descriptors of the Minkowski distance matrix: theory, diversity–variability analysis and QSPR applications. J Math Chem 2015;53:2028–64. https://doi.org/10.1007/s10910-015-0533-3.

[5] Garcia-Jacas C, Marrero-Ponce Y, Barigye S, Valdes-Martini J, Rivera-Borroto O, Olivero-Verbel J. N-linear algebraic maps for chemical structure codification: a suitable generalization for atom-pair approaches? Curr Drug Metab 2014;15:441–69. https://doi.org/10.2174/1389200215666140605124506.

[6] Marrero-Ponce Y, García-Jacas C, . Barigye S, Valdés-Martiní J, Rivera-Borroto O, Pino-Urias R, et al. Optimum Search Strategies or Novel 3D Molecular Descriptors: is there a Stalemate? Curr Bioinform 2015;10:533–64. https://doi.org/10.2174/1574893610666151008011457.

[7] Marrero-Ponce Y, Machado-Tugores Y, Pereira DM, Escario JA, Barrio AG, Nogal-Ruiz JJ, et al. A Computer-Based Approach to the Rational Discovery of New Trichomonacidal Drugs by Atom-Type Linear Indices. Curr Drug Discov Technol 2005;2:245–65. https://doi.org/10.2174/157016305775202955.

[8] Martínez-López Y, Marrero-Ponce Y, Barigye SJ, Teran E, Martínez-Santiago O, Zambrano CH, et al. When global and local molecular descriptors are more than the sum of its parts: Simple, But Not Simpler? Mol Divers 2020;24:913–32. https://doi.org/10.1007/S11030-019-10002-3/METRICS.

[9] Marrero-Ponce Y, Santiago OM, López YM, Barigye SJ, Torrens F. Derivatives in discrete mathematics: a novel graph-theoretical invariant for generating new 2/3D molecular descriptors. I. Theory and QSPR application. J Comput Aided Mol Des 2012;26:1229–46. https://doi.org/10.1007/S10822-012-9591-9/TABLES/7.

[10] García-Jacas CR, Marrero-Ponce Y, Barigye SJ, Hernández-Ortega T, Cabrera-Leyva L, Fernández-Castillo A. N-tuple topological/geometric cutoffs for 3D N-linear algebraic molecular codifications: variability, linear independence and QSAR analysis. Http://DxDoiOrg/101080/1062936X20161231714 2016;27:949–75. https://doi.org/10.1080/1062936X.2016.1231714.

[11] Sánchez-Delgado RA, Rondón D, Andriollo A, Herrera V, Martín G, Chaudret B. Kinetics and Mechanism of the Regioselective Homogeneous Hydrogenation of Quinoline Using [Rh(COD)(PPh3)2]PF6 as the Catalyst Precursor. Organometallics 1993;12:4291–6. https://doi.org/10.1021/om00035a013.

[12] Rosales M, Vallejo R, Soto JJ, Bastidas LJ, Molina K, Baricelli PJ. Kinetics and mechanisms of homogeneous catalytic reactions. Part 10. regioselective hydrogenation of quinoline catalyzed by the systems M 2Cl2(COE)4/2 Triphos [M = Rh, Ir; COE = cyclooctene; Triphos = 1,1,1-tris(diphenylphosphinomethyl)ethane]. Catal Letters 2010;134:56–62. https://doi.org/10.1007/s10562-009-0225-3.

[13] Rosales M, Bastidas LJ, González B, Vallejo R, Baricelli PJ, Molina K, et al. Kinetics and mechanisms of homogeneous catalytic reactions. Part 11. Regioselective hydrogenation of quinoline catalyzed by rhodium systems containing 1,2-bis(diphenylphosphino)ethane. Catal Letters 2011;141:1305–10. https://doi.org/10.1007/s10562-011-0641-z.

[14] Rosales M, Molina K, Vallejo R, Ocando-Mavárez E. Kinetics and mechanisms of homogeneous catalytic reactions: Part 13. Regioselective reduction of quinoline catalysed by Rh(acac)(CO)[P(tBu)(CH2CH=CH2)2]. Transit Met Chem 2016;41:467–73. https://doi.org/10.1007/s11243-016-0042-7.

[15] Rosales M, Alvarado Y, Boves M, Rubio R, Soscún H, Sánchez-Delgado RA. Kinetics and mechanisms of homogeneous catalytic reactions. Part 3. Regioselective, catalysed [RuH(CO)(NCMe)2(PPh3)2]BF4 reduction of quinoline. Transit Met Chem 1995;20:246–51. https://doi.org/10.1007/BF00143486.

[16] Rosales M, Castillo J, González A, González L, Molina K, Navarro J, et al. Kinetics and mechanisms of homogeneous catalytic reactions. Part 5. Regioselective reduction of heteroaromatic nitrogen compounds catalysed by [OsH(CO)(NC-Me)2(PPh3)2]BF4. Transit Met Chem 2004;29:221–8. https://doi.org/10.1023/B:TMCH.0000019452.37770.e4.

[17] Toropov AA, Toropova AP. QSPR/QSAR: State-of-Art, Weirdness, the Future. Mol 2020, Vol 25, Page 1292 2020;25:1292. https://doi.org/10.3390/MOLECULES25061292.
